# An automated method to find reaction mechanisms and solve the kinetics in organometallic catalysis†
†Electronic supplementary information (ESI) available: Details of the automated procedure, main computer codes developed in the present work, reaction network, rate coefficients, intermediates and transition states data, and structures of the main intermediates. See DOI: 10.1039/c7sc00549k



**DOI:** 10.1039/c7sc00549k

**Published:** 2017-03-07

**Authors:** J. A. Varela, S. A. Vázquez, E. Martínez-Núñez

**Affiliations:** a Centro Singular de Investigación en Química Biolóxica e Materiais Moleculares (CIQUS) , Departamento de Química Orgánica , Universidad de Santiago de Compostela , 15782 Santiago de Compostela , Spain; b Centro Singular de Investigación en Química Biolóxica e Materiais Moleculares (CIQUS) , Departamento de Química Física , Universidad de Santiago de Compostela , 15782 Santiago de Compostela , Spain . Email: emilio.nunez@usc.es

## Abstract

A novel computational method is proposed in this work for use in discovering reaction mechanisms and solving the kinetics of transition metal-catalyzed reactions. The method does not rely on either chemical intuition or assumed *a priori* mechanisms, and it works in a fully automated fashion. Its core is a procedure, recently developed by one of the authors, that combines accelerated direct dynamics with an efficient geometry-based post-processing algorithm to find transition states (Martinez-Nunez, E., *J. Comput. Chem.* **2015**, *36*, 222–234). In the present work, several auxiliary tools have been added to deal with the specific features of transition metal catalytic reactions. As a test case, we chose the cobalt-catalyzed hydroformylation of ethylene because of its well-established mechanism, and the fact that it has already been used in previous automated computational studies. Besides the generally accepted mechanism of Heck and Breslow, several side reactions, such as hydrogenation of the alkene, emerged from our calculations. Additionally, the calculated rate law for the hydroformylation reaction agrees reasonably well with those obtained in previous experimental and theoretical studies.

## Abstract 2

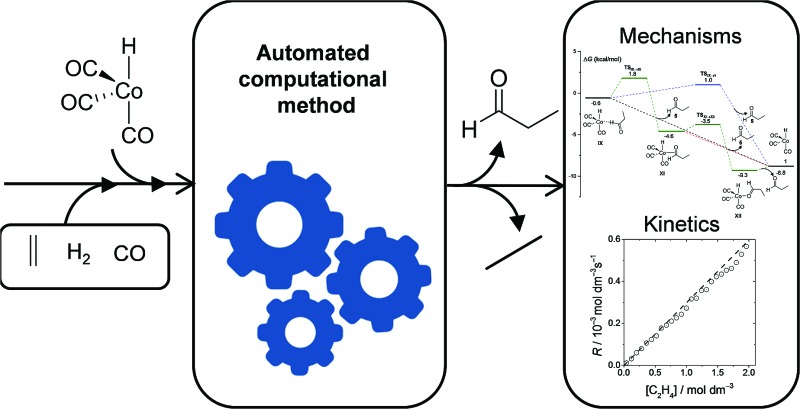
A novel computational method based on a procedure combining accelerated direct dynamics with an efficient geometry-based post-processing algorithm is proposed for use in discovering reaction mechanisms and solving the kinetics of transition metal-catalyzed reactions.

## Introduction

Transition metal catalysis is of fundamental importance in organic synthesis. A great deal of work has been focused on the understanding and elucidation of the underlying mechanisms of organometallic catalysis.^1^ Among the different techniques employed to study these reaction mechanisms, computational methods are becoming increasingly more important, either because of the difficulties of experimentally characterizing fleeting catalytic intermediates, or because they nicely supplement the spectroscopic methods and/or experimental studies of isotope effects.^2^

In recent years, there has been a surge of methods in density functional theory (DFT), which is the electronic structure approach most widely used in computational organometallic catalysis nowadays. However, the inherent complexity of catalytic cycles means computational studies of these systems are still challenging. The strategy commonly adopted in computational organometallic catalysis is to use chemical intuition and/or comparisons with similar catalytic reactions in the design of reaction routes and construction of guess structures for the involved transition states (TSs). Although this procedure led to a thorough understanding of the mechanisms of a number of important organometallic reactions,^3,2b,2d^ the complex reaction networks involved in the catalytic cycles, and number of intermediates interconnected by different TSs, make it very tedious, and, most importantly, prone to overlooking meaningful mechanisms. To overcome these drawbacks, the development of robust automated procedures to find the TSs and discover the reaction mechanisms seems pivotal in computational mechanistic studies.

Three computational automated protocols have been recently tested on the cobalt-catalyzed hydroformylation of ethylene, namely the artificial force induced reaction (AFIR) method,^4^ basin-hopping sampling (BHS),^5^ and graph-based sampling (GBS).^6^ However, some of them just focus on the hydroformylation reaction and neglect side-reactions like hydrogenation of the alkene, which is indeed significant under certain concentrations of the starting materials.^3,7^ Furthermore, AFIR and BHS do not include kinetics simulations.

In recent work, we have shown that the use of accelerated direct dynamics in combination with an efficient geometry-based algorithm to identify bond breaking/formation can be very convenient to find the TSs of a molecular system.^8^ The method, called transition state search using chemical dynamics simulations (TSSCDS), shares the use of accelerated dynamics with other methods proposed in the literature.^9^ However, the novelty of our approach is the direct search for the saddle points‡
‡Here and after, TS and saddle point will be employed interchangeably. from the simulation snapshots.

In this paper, we present an automated method partly based on TSSCDS to study organometallic catalysis. While the discovery of reactions that occur over a barrier is accomplished using the TSSCDS strategy, the barrierless associative/dissociate processes are studied with additional tools developed in the present work. The method is also equipped with computer codes to calculate the rate coefficients and to run Kinetic Monte Carlo (KMC)^10^ simulations, providing a general and robust protocol not only for the discovery of the reaction mechanisms, but also for use in solving the kinetics of the catalytic cycles. Besides KMC, there are recent methods like the rate constant matrix contraction,^11^ which can be very useful in providing the branching ratios in a complex reaction network.

As a case study for testing our novel procedure, we choose the cobalt-catalyzed carbonylation of ethylene with carbon monoxide and hydrogen, known as the hydroformylation or oxo reaction.^12^ This choice is motivated by it having a well-established and accepted mechanism,^13^ and due to the existence of previous mechanistic computational studies.^3–6,14^ Additionally, the rate law for this reaction has been determined experimentally^7^ and in two computational studies,^3,6^ showing fractional orders with respect to some of the reactants, which indicates a complex underlying mechanism.

The method presented in this study intends to fill the gaps left by the other automated procedures. Specifically, it identifies the reaction mechanisms leading to the main products, as well as to the side products, and incorporates algorithms to study the kinetics, which can be very useful in improving the reaction efficiency. The computational procedure will be described in Section 2, and the results obtained for the cobalt-catalyzed hydroformylation of ethylene are presented in Section 3. Finally, the conclusions will be given in the last section.

## Method

The method is an adaptation of TSSCDS, which was recently developed by one of the authors to find the transition states (TSs) of unimolecular reactions.^8^ The basic idea behind TSSCDS is to run accelerated (high-temperature) direct dynamics to break/form new bonds within the first few hundred femtoseconds. Then, an efficient post-processing algorithm (BBFS) identifies the geometries with partly formed/broken bonds, which will serve as guess structures for transition state optimizations. BBFS is based on the connectivity matrix, which can be defined for each molecular species. This matrix is the basis of other successful automated methods like the one developed by Zimmerman.^15^

Once the TSs are optimized, a reaction network can be constructed by computing the intrinsic reaction coordinates (IRCs),^16^ which connect the TSs with the intermediates.^16^ This method employs two levels of theory: semi-empirical and *ab initio*/DFT. The semi-empirical calculations are performed to run the direct dynamics and to obtain the approximate TS structures, while a higher level of theory is used to re-optimize the TSs and run IRC calculations. Two different electronic structure programs are employed: MOPAC2016 ^17^ and Gaussian09 ^18^ for the semi-empirical and *ab initio*/DFT calculations, respectively. Semi-empirical methods are fast, which allows for sampling of a large reactive space, but they might not be well parameterized for some transition metal systems. In those cases, the procedure would entail a re-parameterization of the semi-empirical Hamiltonian.

**Scheme 1:**
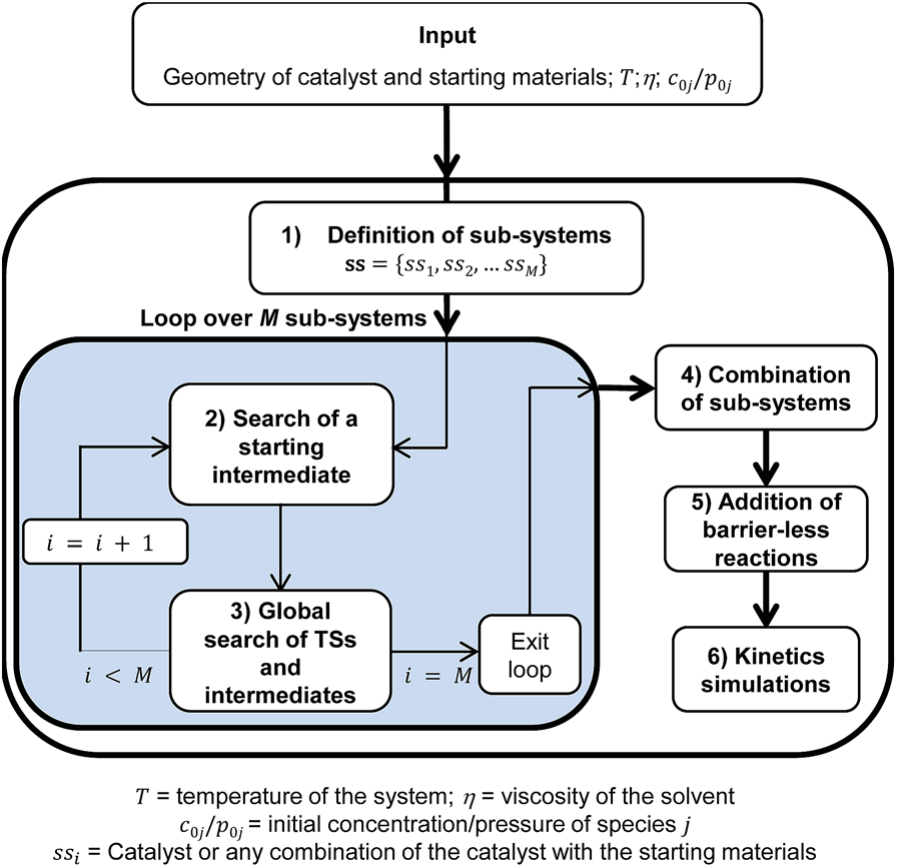
Flow-chart outlining the different steps of the automated method presented in this work to study organometallic catalysis.

Besides the core TSSCDS tool, which spawns the TSs for reactions occurring over a barrier,^8^ several auxiliary algorithms have been developed in the present work to carry out the specific tasks needed to study organometallic catalysis. In particular, the adaptation of TSSCDS entails the division of the whole system into smaller sub-systems, which are sorted by order of increasing complexity. Then, TSSCDS and the new algorithms developed in this work are applied within each of the sub-systems to find the TSs and intermediates, which are finally merged to make up a single reaction network that can be used to solve the kinetics. The specific input data include the geometries and initial concentrations of the catalyst and starting materials, as well as the viscosity of the solvent (see Scheme 1). The geometries are needed to generate the TSs and intermediates, while the initial concentrations and the viscosity of the solvent are employed in the kinetics simulations.

The method consists of six different but inter-dependent steps (see Scheme 1), which are explained in the following.

### 1 Definition of the sub-systems

A sub-system, ss_*i*_, is defined here as either the catalyst or any possible combination of the catalyst with the starting materials. They can be arranged in a list **ss** = {ss_1_, ss_2_, …, ss_*M*_} where 
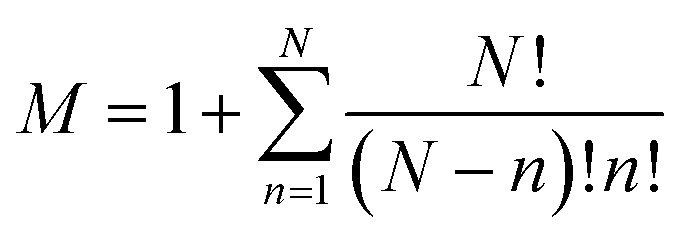
, and *N* is the number of starting materials. The sub-systems are sorted in ascending order of size, *i.e.*, the first sub-system is the smallest (the catalyst) and the last one is the largest, containing the catalyst and the starting materials. This particular order was chosen because the starting intermediates of the larger sub-systems are dependent on the intermediates of the smaller ones (*vide infra*).

For the test case selected in this study, the hydroformylation of ethylene, the active catalyst is HCo(CO)_3_ (**1**), and the three starting materials are ethylene (**2**), carbon monoxide (**3**), and molecular hydrogen (**4**), which give rise to the eight sub-systems collected in Table 1.

**Table 1:** Definition of the sub-systems and species A and B utilized in step 2 of the method, which were investigated in this study

Sub-system		A^*a*^	B^*a*^
ss_1_	HCo(CO)_3_	—	—
ss_2_	HCo(CO)_3_/C_2_H_4_	HCo(CO)_3_	C_2_H_4_
ss_3_	HCo(CO)_3_/CO	HCo(CO)_3_	CO
ss_4_	HCo(CO)_3_/H_2_	HCo(CO)_3_	H_2_
ss_5_	HCo(CO)_3_/C_2_H_4_/CO	HCo(CO)_3_/C_2_H_4_	CO
ss_6_	HCo(CO)_3_/C_2_H_4_/H_2_	HCo(CO)_3_/C_2_H_4_	H_2_
ss_7_	HCo(CO)_3_/CO/H_2_	HCo(CO)_3_/CO	H_2_
ss_8_	HCo(CO)_3_/C_2_H_4_/CO/H_2_	HCo(CO)_3_/C_2_H_4_/CO	H_2_

^*a*^Chemical species A and B employed to find the starting intermediates of each sub-system (step 2 of our method).

### 2 Search for a starting intermediate of each sub-system

TSSCDS needs a starting intermediate I0*i* for each sub-system, *i*, to initiate the dynamics. For the first sub-system, I01 is just the active catalyst, and its geometry is provided as an input. For any other sub-system, ss_*i*_ (*i* > 1), the starting intermediate is constructed from the association of two smaller units, A and B. Species A is a sub-system with one component less than ss_*i*_, and species B is the extra component in ss_*i*_ (see Table 1). While the geometry of the starting material B is given as the input, the structure of complex A is chosen among those obtained in step 3 of a previous cycle of the loop (see Scheme 1). The criteria employed to determine the structure for A are both its energy and the valence of its metal center. In particular, we seek a low-energy unsaturated complex.

Having chosen A and B, we now need to generate an A···B complex that will be the starting intermediate I0*i*. The procedure followed here consists of generating one hundred guess A···B structures by randomly rotating each species about a pivot point;^19^ the metal center of A and the center of mass of B are the pivot points, and the distance between them remains fixed at 2 Å throughout the random rotations. Furthermore, if an A···B structure has an intermolecular distance lower than a given threshold (1 Å in this study), the structure is replaced by a new one. Finally, each A···B structure is subjected to geometry optimization.

Among the successfully optimized A···B complexes, the starting intermediate is selected using the above two criteria: the energy and the valence of the metal center. In this case, the selection is biased towards a low-energy A···B complex with high valence of the metal, *i.e.*, the metal center presents no coordination vacancies, which ensures a stable intermediate. At any rate, the selection method is not crucial because all intermediates should be obtained in the next step, and its only role is to provide a starting geometry for the accelerated dynamics.

All electronic structure calculations of this step, which include the energies of the structures and the valences of their metal centers, are carried out with MOPAC2016.^17^ For the example studied in this work, the latest PM7 Hamiltonian^20^ was chosen.

### 3 Global search for the TSs and intermediates

Once a suitable starting intermediate is obtained, the TSSCDS machinery comes into play. The chemical dynamics simulations are carried out using a development version of MOPAC^17*a*^ with the forces and energies calculated at the PM7 level of theory^20^ and using a step size of 0.5 fs. All atoms of the active catalyst, **1**, except the hydrogen, are kept frozen throughout the simulations, and each atom is given an average kinetic energy of 3/2*RT* with *R* being the gas constant, and the temperature (*T*) is 10^4^ K. We use here the iterative version of the method,^8*b*^ which involves the initialization of the dynamics from multiple intermediates. A full description of this step can be found in the ESI†.

TSSCDS is very effective and spawns thousands of optimized TS structures at the PM7 level. However, the geometries and energies of the TSs obtained with PM7 are only approximate, and they must be re-optimized using a higher level of theory; the high level of theory chosen for the hydroformylation of ethylene was B3LYP/6-31G(d,p). Finally, the intermediates are obtained after running IRC calculations using the obtained TS structures. Even though B3LYP/6-31G(d,p) is not the best possible electronic structure protocol to study this reaction,^3^ our choice was based on the fact that the most recent automated method applied to the same system employs the same level of theory,^6^ which facilitates a direct comparison. We also notice that the computations performed in this work simulate the reactivity in the gas phase. However, according to Harvey’s calculations,^3^ the solvent effects are expected to be unimportant for the system investigated here.

Some screening is called for since the DFT calculations are, by far, the bottleneck of the whole procedure. In particular, we only considered reaction mechanisms with TSs that have relative free energy values (calculated by DFT) of less than 40 kcal mol^–1^, with respect to the starting intermediate.

### 4 Combination of the sub-systems

The reaction mechanisms discovered within each sub-system are finally merged into a single reaction network, and a single list of TSs (**TS** = {TS_1_, TS_2_, …, TS_*N*_TS__}) and intermediates (**I** = {I_1_, I_2_, …, I_*N*_I__}) can be obtained. This step is indispensable to solve the kinetics for the whole system (step 6 of the procedure).

Specifically, the step entails two different tasks, namely: (1) setting a common free energy *G* scale, and (2) identifying common molecules in different sub-systems. The former is done by adding the values of *G* for the starting materials that are lacking in the sub-system, ss_*i*_, to the free energies of each TS and intermediate of the ss_*i*_. Finally, the relative free energy values, Δ*G*, are calculated by subtracting the sum of the free energies of the catalyst and the starting materials.

Additionally, the intermediates of any sub-system can dissociate by releasing several fragments. The fragments can be categorized as: (a) products and side products **P** = {P_1_, P_2_, …, P_*N*_P__} that do not contain the metal, and (b) species containing the metal. On the one hand, a starting material can be an element of **P**, and, on the other hand, the species containing the metal might be one of the intermediates, I_*i*_, of a smaller sub-system. To detect those matchings, the tools of spectral graph theory, which are available within TSSCDS,^8^ are employed here.

### 5 Addition of barrierless reactions

Since the algorithm employed to discover the reaction mechanisms is designed to find the saddle points,^8*a*^ barrierless (dissociative–associative) processes are elusive. The procedure followed here to characterize the barrierless reactions consists of the following two steps:

(a) Analysis of all intermediates, **I**, to check whether their structures contain one or several elements of **P**. A product, P_*j*_, can be contained within an organometallic complex, I_*i*_, either because P_*j*_ is one of its ligands, or because of non-covalent interactions between P_*j*_ and I_*i*_. In practice, the metal center is removed from the intermediate, I_*i*_, and the resulting fragments are compared with the elements of **P**. Then, if a product, P_*j*_, is one of those fragments, we proceed to step b. Otherwise, we advance to the next intermediate.

(b) The original structure of I_*i*_ is recovered, and the distance, *r*, between the metal and center of mass of P_*j*_ is doubled. The resulting stretched structure is first partially optimized (keeping *r* constant), followed by a downhill calculation with Gaussian09; both the partial optimization and downhill calculation are carried out at the DFT level for the example studied in this work. If the downhill calculation leads to the original intermediate, I_*i*_, the process I_*i*_ ⇆ A_*i*_ + P_*j*_ is regarded as barrierless and added to the reaction network, where A_*i*_ is the structure that results after removing P_*j*_ from I_*i*_.

It is worth noting here that besides the barrierless dissociative–associative mechanisms discussed above, the associative ligand substitutions occurring in a single step can also take place and be very competitive.^21^ In the previous study, these mechanisms were not found for the Co-catalyzed alkene hydroformylation, regardless of efforts to locate a TS for the displacement of CO by the solvent (toluene) in the saturated species [H(CO)_4_].^3^ Such a TS was also not found here because toluene is not one of the input species. However, two associative ligand substitution mechanisms, namely the displacement of CO by ethylene or H_2_, have been discovered in this work. They proceed with free energy barriers of 43 and 67 kcal mol^–1^, respectively, which make them non-competitive with the corresponding two-step barrierless mechanisms found in this study (*vide infra*).

### 6 Kinetics simulations

Once a single reaction network is created, the thermal rate coefficient, *k*_l_(*T*), for each process l presenting a barrier is calculated according to transition state theory:
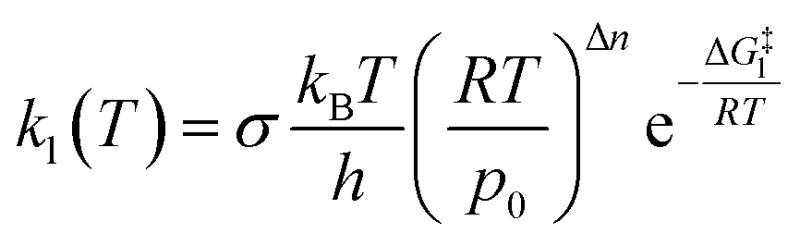
where *σ* is the reaction path degeneracy, *T* is the temperature, *h* is Planck’s constant, Δ*G*‡l is the free energy of activation, *p*_0_ is 1 bar and Δ*n* = 1 (0) for bimolecular (unimolecular) reactions.

Following the approach of Harvey and co-workers,^3^ the barrierless associative reactions are assumed to be diffusion-controlled, and their thermal rate coefficients, *k*_diff_(*T*), are calculated by the following equation:
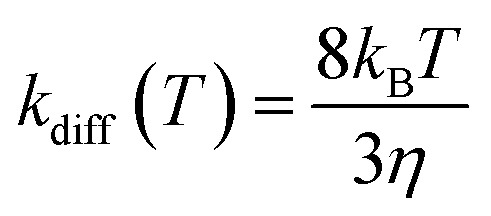
where *η* is the viscosity of the solvent (toluene in the example studied here). The rate for the corresponding reverse dissociative process is calculated using detailed balance. A temperature of 423 K was employed to compare with the available experimental^7^ and theoretical kinetics results.^3,6^

Having computed all rate coefficients, the time evolution of all intermediates can be monitored by running KMC simulations,^10^ provided that the initial concentrations of the catalyst and starting materials are known. This task is accomplished using our own KMC code, as well as the fast open-source program StochKit2.0.^22^ As in previous studies,^8b,23^ the KMC simulations are coarse-grained, *i.e.*, we assume that conformational isomers interconvert very quickly and form a single state, which allows us to extend the simulation time up to minutes.

To determine a rate law for the hydroformylation of ethylene, the concentrations of the catalyst and starting materials are varied, and the rate is determined from the time-dependence of the concentration of propanal. In particular, the concentrations of the catalyst and ethylene range from 0.0004–0.02 mol dm^–3^ and 0.04–2 mol dm^–3^, respectively, and the partial pressures of CO and H_2_ vary from 1–60 bar. Additionally, the solubilities of both gases in toluene are taken from the previous experimental study.^7^

All steps described above are fully automated and described in more detail in the ESI.† The procedure has been implemented in a computer program called TSSCDS1.0, which can be freely obtained from the authors upon request. TSSCDS1.0 is interfaced with MOPAC2016 (employed in steps 2 and 3), Gaussian09 (employed in steps 3–5) and the two KMC codes employed in step 6. Although the input data is very simple, TSSCDS1.0 features a friendly Graphical User Interface (GUI), which runs on both Windows and Linux.

## Results and discussion

The procedure described in the previous section has been employed to study the cobalt-catalyzed reactions of ethylene in the presence of carbon monoxide and molecular hydrogen. A total of 230 chemical species and 448 elementary reactions have been discovered. Of the 448 reactions, 57 are barrierless and the remaining 391 present saddle points (see the ESI†). The obtained products include propanal (**5**), ethane (**6**), formaldehyde, propene, and water. However, our kinetics results predict 100% selectivity towards the formation of propanal under most conditions for low ethylene conversion, which agrees with the experimental study,^7^ with hydrogenation of ethylene to ethane being competitive for very low CO partial pressures. Therefore, in the following discussion, only the hydroformylation and hydrogenation mechanisms will be described. A full description of all reaction steps with their intermediates and TSs is gathered in the ESI.†

### 1 Hydroformylation

**Scheme 2:**
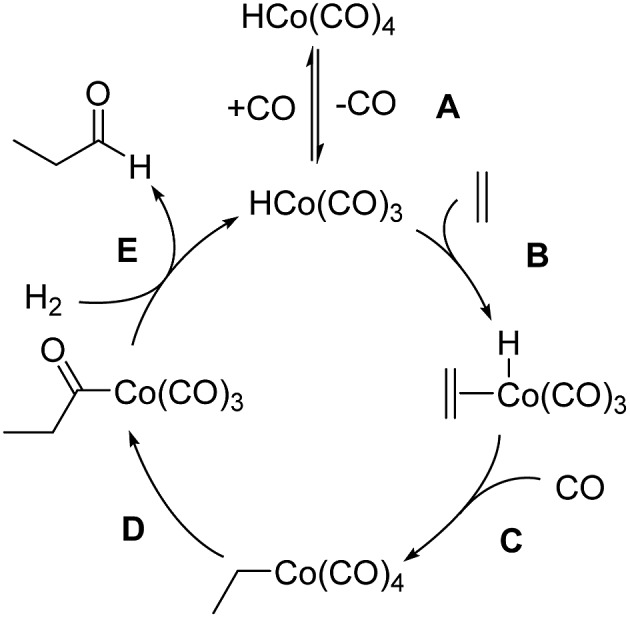
Heck and Breslow mechanism for the Co-catalyzed hydroformylation of ethylene.

The mechanism of hydroformylation was first proposed by Heck and Breslow^13*a*^ in the 1960s and is still valid nowadays. The Co-catalyzed hydroformylation of ethylene consists of the five steps depicted in Scheme 2: (A) CO dissociation from the catalyst resting state, HCo(CO)_4_, (B) alkene coordination, (C) 1,2-insertion of the alkene into the Co–H bond followed by CO coordination to the generated vacant site of the metal, (D) 1,1-insertion of CO into the Co–C bond, and (E) H_2_ oxidative addition to cobalt followed by reductive elimination to afford the final aldehyde.

**Fig. 1:**
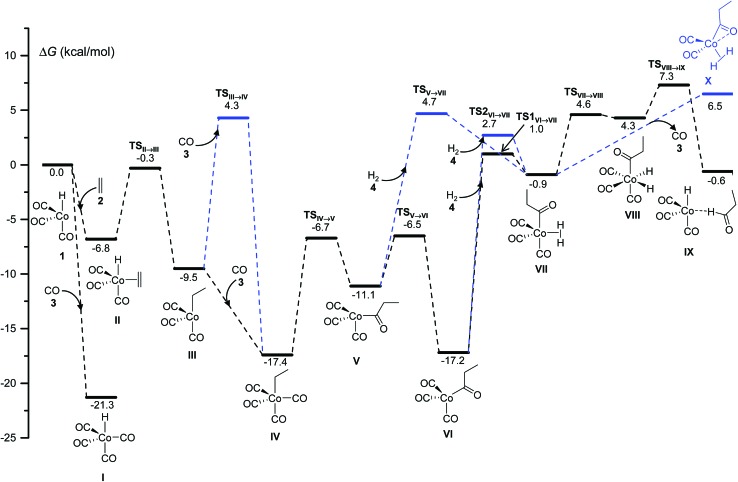
DFT-calculated free energy profile for the Co-catalyzed hydroformylation of ethylene up to the reductive elimination affording the species IX. The blue lines are used for the less competitive pathways observed in the kinetics simulations. The relative free energy values Δ*G* are calculated by subtracting the sum of the free energies of the catalyst and the starting materials.

Our calculations show that the mechanism is initiated with a pre-equilibrium between the 16 electron species **1** and the 21.3 kcal mol^–1^ more stable saturated complex, **I**, formed by coordination of one CO molecule (Fig. 1). The predominant pathway continues with the barrierless coordination of ethylene to the less stable complex, **1**, to afford **II**. A less favorable route from **I** to **II** (not shown in Fig. 1 for clarity) was also found, involving a single-step associative ligand substitution, but its very high free energy barrier of 43 kcal mol^–1^ makes it very unlikely.

Following this, a 1,2-insertion of ethylene into the Co–H bond, occurring over a free energy barrier of 6.5 kcal mol^–1^, affords the unsaturated alkyl-cobalt complex, **III**, which evolves to form the most stable 18 electron cobalt complex, **IV**, by coordination of a CO molecule, either over a free energy barrier of 13.8 kcal mol^–1^ or, most likely, through a barrierless process.

The preferred pathway continues through a 1,1-insertion of CO into the Co-alkyl bond giving rise to the acyl complex, **V** (Δ*G* ^‡^ = 10.7 kcal mol^–1^), followed by a small free-energy barrier process (4.6 kcal mol^–1^) to afford the more stable intermediate, **VI**, which only differs from **V** in the coordination geometry of the ligands around the metal center.

The next intermediate in the catalytic cycle, the 18 electron species **VII** where the hydrogen molecule is coordinated to cobalt, can be reached from two different intermediates (**V** and **VI**) and *via* three different routes, one from **V** and two from **VI**. The direct coordination of H_2_ to intermediate **V** affords **VII** with a Δ*G* ^‡^ of 15.8 kcal mol^–1^, while two different transition states were found for the coordination of H_2_ to **VI**, and the free energies of these transition states only differ by 1.7 kcal mol^–1^. Interestingly, the intermediate **VII** was not optimized in the most recent automated study by Habershon,^6^ where only the dihydride complex **VIII** was obtained, while in our study **VIII** was obtained from **VII** by the oxidative addition of H_2_ to cobalt over a free energy barrier of 5.5 kcal mol^–1^. Two other paths, with higher free-energy barriers of 8.0 and 10.3 kcal mol^–1^, were also found connecting **VII** and **VIII** (not shown in Fig. 1 for clarity).

Finally, reductive elimination in **VIII** affords the van der Waals complex **IX** (Δ*G* ^‡^ = 3 kcal mol^–1^), where the catalytic active species, **1**, weakly interacts with the final product aldehyde **5**. Again, two other less competitive paths (not shown in Fig. 1 for clarity) were found for this reductive elimination with free-energy barriers 2.4 and 3.9 kcal mol^–1^ above TS**_VIII_**_→_ **_IX_**. Besides these two processes, a third one consisting of the barrierless release of CO from **VII** to afford complex **X** was found, even though this path is not very competitive under the usually high CO partial pressures employed in the experiments.

**Fig. 2:**
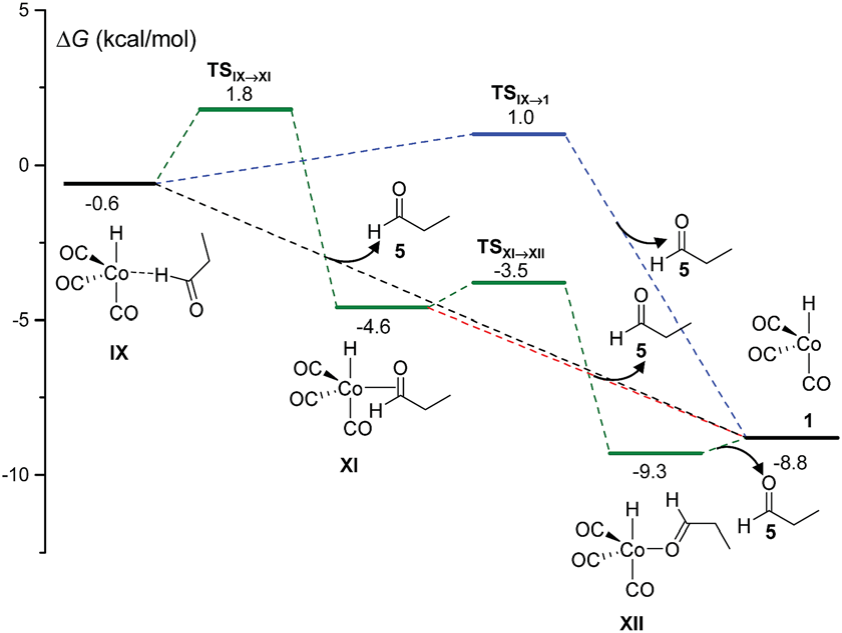
DFT-calculated free energy profile for the release of propanal (**5**) and recovery of the catalytic active species (**1**) in the Co-catalyzed hydroformylation of ethylene.

The final step of the catalytic cycle must be the release of the product, and the regeneration of the free catalytic active species. The kinetics simulations predict that the product aldehyde can be obtained through the competitive pathways shown in Fig. 2, although a total of ten different paths leading to products consistent with the molecular formula C_3_H_6_O were discovered in our work.

The most favorable pathways of Fig. 2 are the direct dissociations of **5** from **IX**, either without a free-energy barrier or with a Δ*G* ^‡^ of 1.6 kcal mol^–1^. Alternatively, the more stable 18 electron complexes, **XI** and **XII**, can be formed by coordination of the propanal carbonyl group to cobalt (either through the two π electrons of the carbonyl bond or a lone electron pair of oxygen) in a more energetically demanding process. Again, the barrierless dissociation of propanal with the concomitant recovery of **1** is possible from both **XI** or **XII**. The species **IX**, **XI** and **XII** went unnoticed in previous theoretical work, except in the study of Harvey and co-workers,^3^ where a structure similar to **XII** was optimized.

**Fig. 3:**
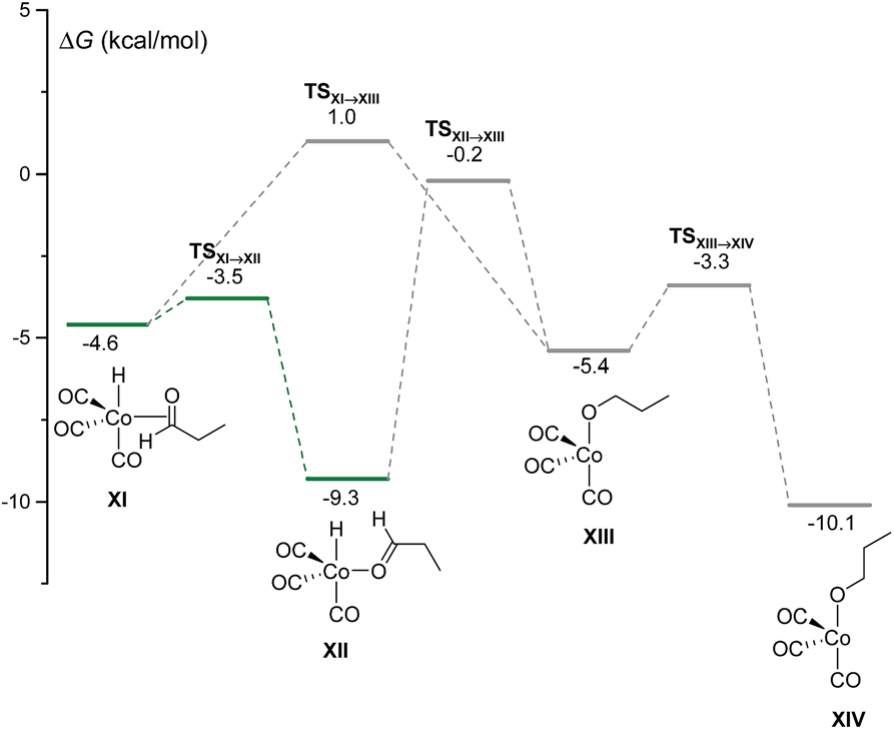
DFT-calculated free energy profile for the insertion of the aldehyde carbonyl into the Co–H bond to afford XIII and XIV.

Using our automated method, two alternative dead-end pathways were found leading to the very stable alcoxycobalt intermediate **XIV** (gray lines in Fig. 3). In particular, the 1,2-insertion of the aldehyde carbonyl into the Co–H bond, either from **XI** or **XII**, afforded the alcoxycobalt species, **XIII**, which evolved to the more stable complex, **XIV**, after surmounting a free energy barrier of 2.1 kcal mol^–1^.

### 2 Hydrogenation

**Fig. 4:**
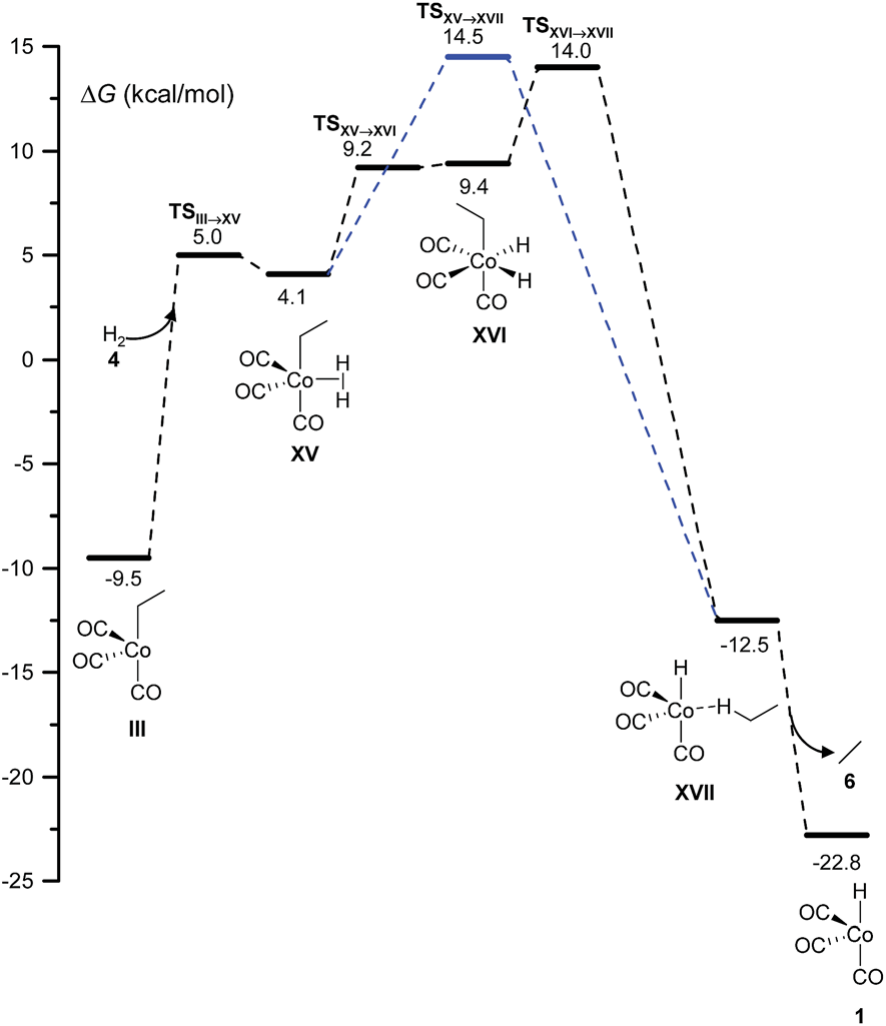
DFT-calculated free energy profile for the hydrogenation reaction.

Two different pathways affording ethane from the hydrogenation of ethylene arise from this study (Fig. 4). Both start with the coordination of molecular hydrogen to the unsaturated intermediate, **III**, to form **XV** over a free-energy barrier of 14.5 kcal mol^–1^. Then, either a single-step σ-metathesis *via* a free-energy barrier of 18.6 kcal mol^–1^, or the oxidative addition of H_2_ to the cobalt center followed by reductive elimination (similar to the transformation of **VII** into **IX** in Fig. 1), afforded the weakly bound van der Waals complex, **XVII**, which easily released the alkane, **6**.

This side reaction has been neglected in all previous computational studies, except in the one by Harvey and co-workers.^3^ However, as shown below, it may be the main channel under certain conditions.

### 3 Kinetics

**Fig. 5:**
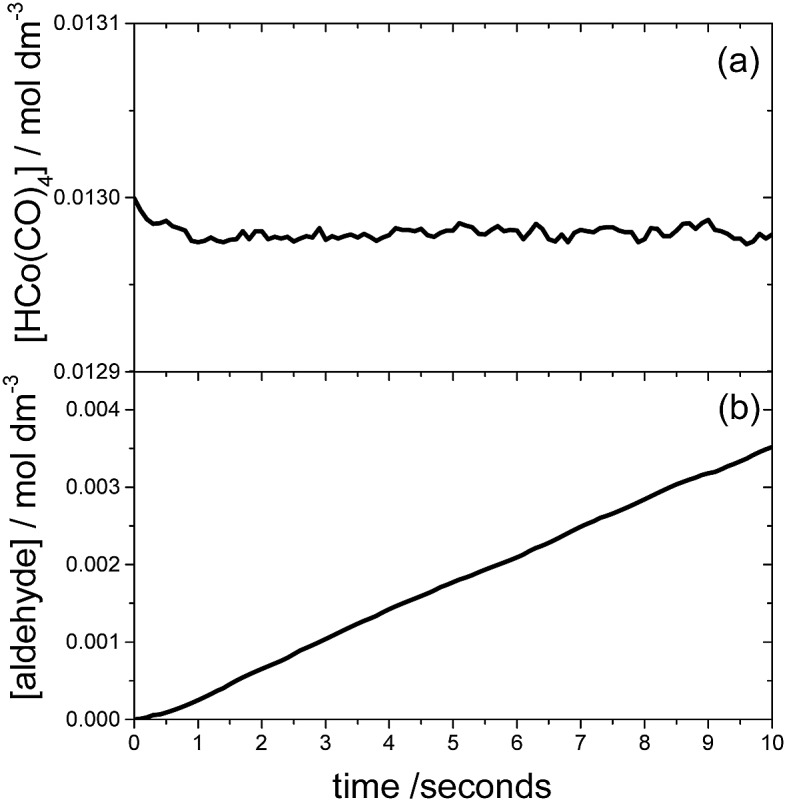
The time evolution of the concentrations of HCo(CO)_4_ (plot a) and the aldehyde (plot b) obtained in our study for the following initial concentrations/pressures: [HCo(CO)_4_]_0_ = 0.013 M, [C_2_H_4_]_0_ = 1.32 M and *p*_CO_ = *p*_H_2__ = 46.7 bar.

The time evolution of each chemical species can be monitored using KMC, assuming that the catalyst and starting materials are mixed and continuously stirred. Fig. 5 shows the results of such a KMC simulation. In particular Fig. 5a displays the time dependence of the concentration of species **I**, using the initial concentrations of 0.013 and 1.32 M for HCo(CO)_4_ and ethylene, respectively, and an initial pressure of 46.7 bar for both H_2_ and CO. The figure clearly shows that **I** is the resting state of the catalyst; the kinetics simulations predict that species **IV** and **VI** are also present, albeit in negligible concentrations of approximately 10^–5^ M.

The time dependence of the product aldehyde, **5**, is shown in Fig. 5b. As seen in the figure, there is an induction period of less than 1 second, which was also observed experimentally.^7^ To avoid the induction period, the hydroformylation rate, *R*, is obtained from the slope of the curve in the range 1–2 seconds, which provides a value of 3.6 × 10^–4^ mol dm^–3^ s^–1^ for the initial conditions of Fig. 5. Our reaction rate is an order of magnitude greater than that obtained by Habershon using the same initial conditions and DFT level of theory.^6^ In our opinion, the difference between our kinetics results and those of Habershon comes from the fact that his reaction rates are calculated from the slope of the [aldehyde] *vs.* time curve between 8 × 10^–3^ and 10^–2^ seconds, which according to our simulations is clearly within the induction period.

The experimentally determined rate law for hydroformylation reads:^7^ 
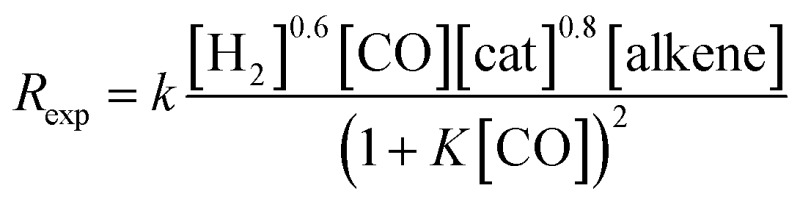


On the other hand, two different rate laws were determined in previous theoretical studies. Harvey and co-workers obtained this expression:^3^ 
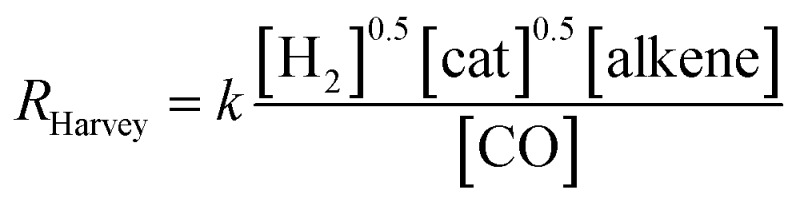
whereas Habershon best fitted his kinetic data to the following equation:^6^ 
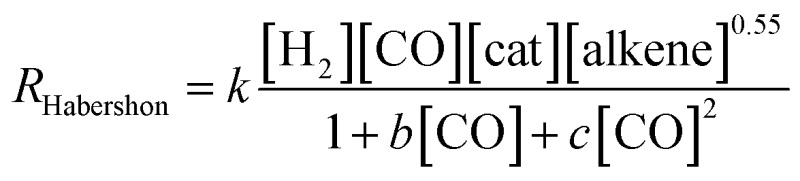


However, as mentioned above, Habershon employed a very short simulation time, which was clearly within the induction period, and this fact makes his results uncertain.

**Fig. 6:**
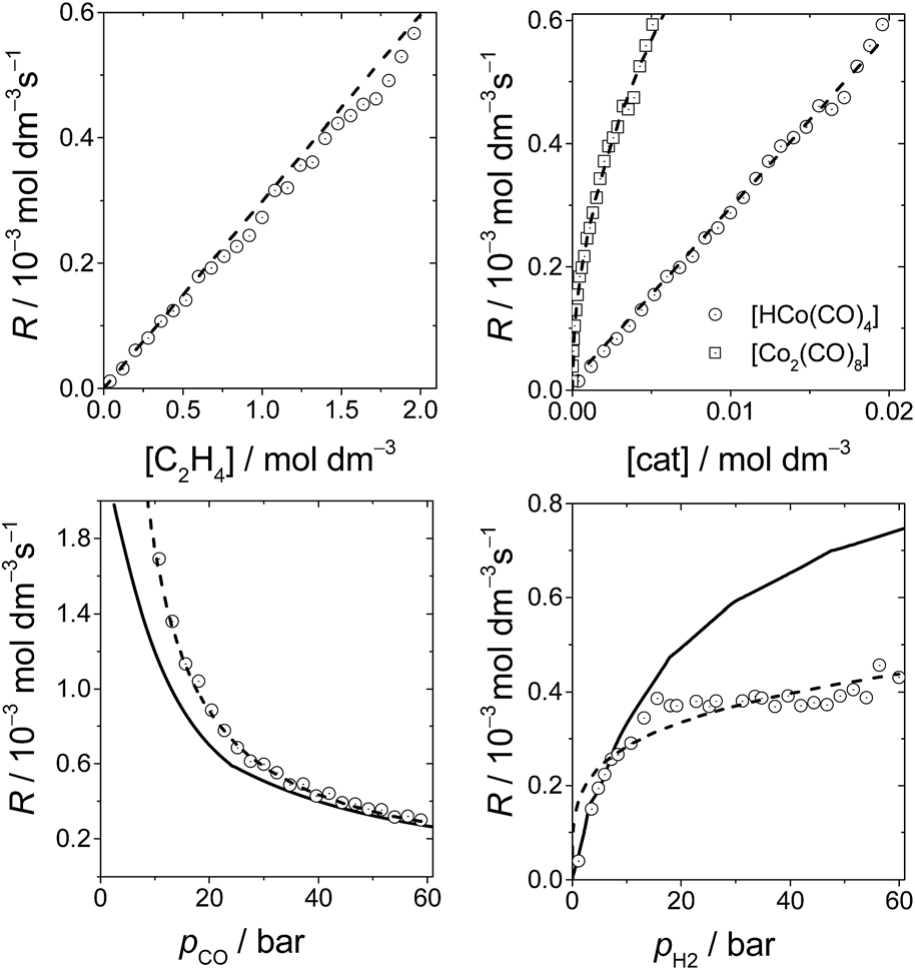
The rates of hydroformylation of ethylene for various initial conditions (symbols). The dashed line is a fit to our computed rates, and the solid line corresponds to the rates obtained by Harvey and co-workers.^3^

Fig. 6 shows the dependence of our reaction rates on the concentrations of the catalyst and starting materials (the symbols and dashed lines in the figure). Our reaction rate is first-order with respect to the concentration of alkene, as was the case in the experimental study^7^ and the computational work by Harvey and co-workers,^3^ but not in Habershon's work.^6^

We also found a first-order dependence with respect to the catalyst species, HCo(CO)_4_. However, the actual catalyst employed in the experiment is Co_2_(CO)_8_, which is in equilibrium with HCo(CO)_4_(Co_2_(CO)_8_ + H_2_ ⇆ 2HCo(CO)_4_). Actually, when we include this equilibrium in our calculations, since the concentration of Co_2_(CO)_8_ displays a quadratic dependence on [HCo(CO)_4_], the reaction rate presents a fractional order of 0.5 with respect to [Co_2_(CO)_8_] (Fig. 6), which is the same dependence found by Harvey and co-workers.

On the other hand, the reaction rate is inversely proportional to the partial pressure of CO, like in all previous studies, and it was best fitted by the following equation:
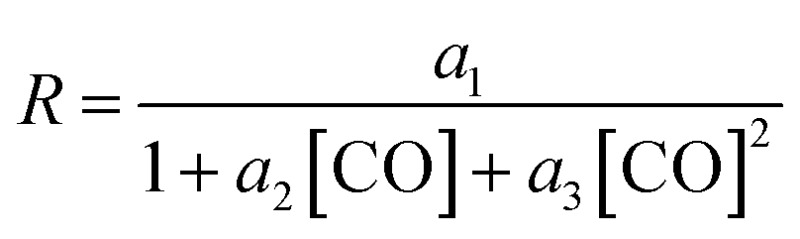


Finally, the order with respect to molecular hydrogen is also fractional (0.4), and similar to that observed in the experimental study and the work of Harvey and co-workers. Habershon's calculations predict a first-order dependence, which is again at odds with the experimental result.

It is interesting to see that the dependence of our reaction rates on the partial pressures of CO and H_2_ compare reasonably well with those obtained by Harvey and co-workers^3^ (solid line in Fig. 6), despite the different alkenes and levels of theory employed in both studies.

In summary, the following rate law fits very well with our kinetics results:
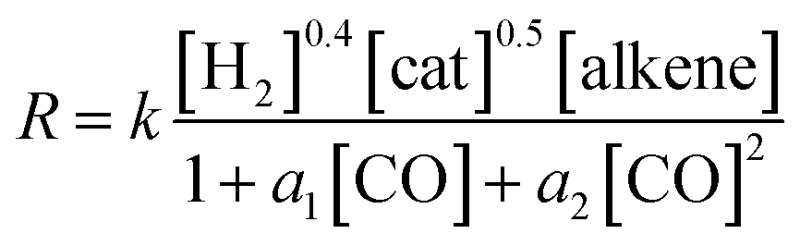


Quite interestingly, even though the alkene, initial conditions, and level of theory employed in our work match those used by Habershon,^6^ the rates observed in the present work agree much better with the other two kinetics studies.^3,7^ As mentioned above, this could be attributed to the very short simulation times employed by Habershon in his kinetics simulations.

**Fig. 7:**
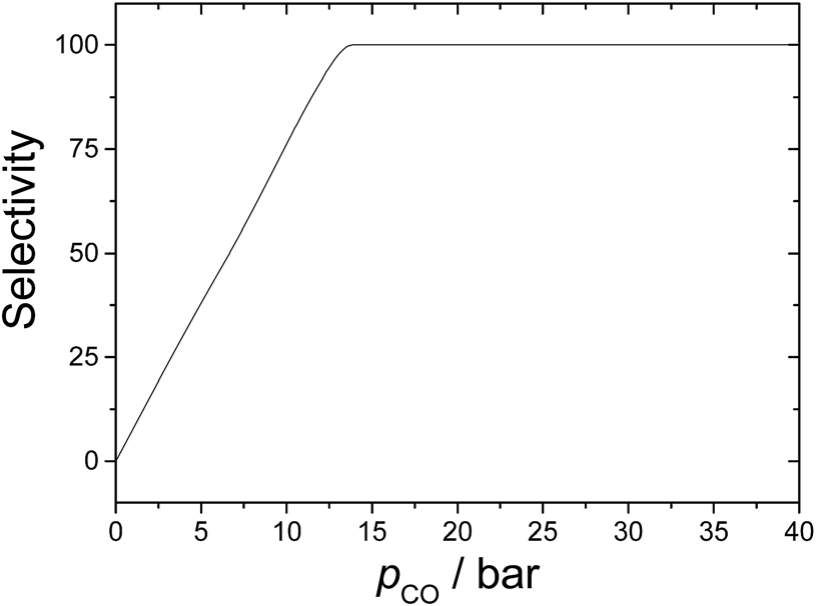
Hydroformylation selectivity as a function of the CO partial pressure.

Finally, it is worth discussing here the relative importance of hydrogenation of the alkene. This side reaction was only studied theoretically by Harvey and co-workers,^3^ and their calculations suggest that the selectivity is about 92% towards hydroformylation for *p*_CO_ = 10 bar. This agrees very well with our results seen in Fig. 7, showing that selectivity increases almost linearly up to CO partial pressures of 13 bar. Our kinetics simulations indicate that hydrogenation dominates over hydroformylation for carbon monoxide partial pressures lower than 7–8 bar. On the other hand, when *p*_CO_ > 13 bar, the selectivity is 100%. This result highlights the importance of studying all possible reaction mechanisms in organometallic catalysis to optimize the reaction conditions.

## Conclusions

We have developed a new automated strategy to predict the reaction mechanisms and kinetics of organometallic-catalyzed reactions that only needs the geometries of the catalyst and starting materials and the experimental conditions (the temperature, concentrations of the chemical species and viscosity of the solvent) as inputs. With these data, the automated method generates the starting geometries of the intermediates from which the accelerated (high-temperature) dynamics simulations are carried out. The trajectories are very short, lasting only a few hundred femtoseconds, and are integrated “on the fly” with the gradients taken from semi-empirical calculations. An efficient post-processing algorithm that monitors the changes in the connectivities of the atoms is responsible for finding the first order saddle points. After optimizing the TSs, the intermediates are easily determined from IRC calculations. Additionally, the barrierless reactions can also be identified and added to the reaction network. Finally, the Kinetic Monte Carlo simulations provide all sort of kinetic information for the system.

The proposed methodology has been successfully tested on the cobalt-catalyzed hydroformylation of ethylene, finding that the main pathway is the one proposed by Heck and Breslow. Moreover, the predicted rate law is in line with that obtained experimentally, and also in a recent computational study using much higher levels of theory. This result shows that DFT methods may be successfully applied to organometallic catalysis, thus providing at least a qualitative description of the very complex potential energy surfaces of these systems. Additionally, our method, unlike other automated methods, is capable of predicting the wasteful side reactions and their yields, a feature that can be employed to optimize the reaction conditions. Specifically, for the test case chosen here, the hydrogenation of the alkene was observed to be predominant for very low CO pressures.

The method presented in this work is currently being employed to study larger catalytic systems. One of the advantages of the method is the use of dynamics simulations which are currently parallelized. The dynamics module is furnished with algorithms to sample the phase space non-uniformly,^24^ which can be used to accelerate finding the TS or to guide the dynamics towards mechanisms of greater interest. Finally, the specific reaction parameters could be employed in the semi-empirical Hamiltonian for systems where standard parameterization does not work well, or to speed up the procedure by skipping the high-energy calculations.
