# Supplementary material for: An automated method to find reaction mechanisms and solve the kinetics in organometallic catalysis
Source: Chem Sci. 2017 Mar 7;8(5):3843–51. doi: 10.1039/c7sc00549k (PMC5577717; doi:10.1039/c7sc00549k)
Supplement: Supplementary file 1 [file SC-008-C7SC00549K-s001.pdf]

Electronic Supplementary Material (ESI) for Chemical Science.
This journal is © The Royal Society of Chemistry 2017

SUPPLEMENTARY INFORMATION FOR:

An automated Method to Find Reaction Mechanisms and Solve the Kinetics in Organometallic Catalysis

Jesús A. Varela,[†] *Saulo A. Vázquez*[‡] *and Emilio Martínez-Núñez*[‡]*

[†]Centro Singular de Investigación en Química Biolóxica e Materiais Moleculares (CIQUS) e Departamento de Química Orgánica, Universidad de Santiago de Compostela, 15782 Santiago de Compostela, Spain

[‡]Centro Singular de Investigación en Química Biolóxica e Materiais Moleculares (CIQUS) e Departamento de Química Física, Universidad de Santiago de Compostela, 15782 Santiago de Compostela, Spain

Index:

1. **Full details of the automated procedure** **S3**
2. **Main computer codes developed in the present work** **S10**
3. **Reaction network** **S39**
4. **Rate coefficients** **S47**
5. **Intermediates** **S59**
6. **Transition states** **S229**
7. **Structures of the main intermediates** **S443**

1. Full details of the automated procedure

The method consists of six steps. Although steps 1, 2, 4-6 are fully described in the main text, they are repeated here to facilitate the reading.

1) Definition of the sub-systems. A sub-system ss_i is defined here as either the catalyst or any possible combination of the catalyst with the N starting materials. They can be arranged in a list $\mathbf{ss} = \{ss_1, ss_2, \ldots, ss_M\}$ where $M = 1 + \sum_{n=1}^{N} \frac{N!}{(N-n)!n!}$. The sub-systems are sorted in ascending order of size, i.e., the first sub-system is the smallest (the catalyst) and the last one is the largest, containing the catalyst and the N starting materials. This particular order was chosen because the starting intermediates of the bigger sub-systems are dependent on the intermediates of the smaller ones.

For the test case selected in this study, hydroformylation of ethylene, the active catalyst is $HCo(CO)_3$ (**1**), and the three starting materials are ethylene (**2**), carbon monoxide (**3**), and molecular hydrogen (**4**), which give rise to the eight sub-systems collected in Table 1 of the main text.

2) Search of a starting intermediate of each sub-system. TSSCDS needs a starting intermediate I_i^0 for each sub-system i to initiate the dynamics. For the first sub-system, I_1^0 is just the active catalyst, whose geometry is provided as input. For any other sub-system ss_i ($i > 1$), the starting intermediate is constructed from the association of two smaller units A and B. Species A is a sub-system with one component less than ss_i, with B being the extra component in ss_i (see Table below).

sub-system		A[a]	B[a]
ss_1	$HCo(CO)_3$	-	-
ss_2	$HCo(CO)_3/C_2H_4$	$HCo(CO)_3$	C_2H_4
ss_3	$HCo(CO)_3/CO$	$HCo(CO)_3$	CO
ss_4	$HCo(CO)_3/H_2$	$HCo(CO)_3$	H_2
ss_5	$HCo(CO)_3/C_2H_4/CO$	$HCo(CO)_3/C_2H_4$	CO
ss_6	$HCo(CO)_3/C_2H_4/H_2$	$HCo(CO)_3/C_2H_4$	H_2
ss_7	$HCo(CO)_3/CO/H_2$	$HCo(CO)_3/CO$	H_2
ss_8	$HCo(CO)_3/C_2H_4/CO/H_2$	$HCo(CO)_3/C_2H_4/CO$	H_2

[a]Chemical species A and B employed to find the starting intermediates of each sub-system (step 2 of our method).

While the geometry of the starting material B is given as input, the structure of complex A is chosen among those obtained in step 3 of a previous cycle of the loop. The criteria employed to select such structure for A are both its energy and the valence of its metal center. In particular, we seek a low-energy unsaturated complex.

Having chosen A and B, we now need to generate a A$\cdots$B complex that will be the starting intermediate I_i^0. The procedure followed here consists of generating one hundred guess A$\cdots$B structures by rotating each species about a pivot point, randomly varying the Euler's angles; the metal center of A and the center of mass of B are the pivot points, and the distance between them remains fixed at 2 Å throughout the random rotations. Furthermore, if a A$\cdots$B structure has an intermolecular distance lower than a given threshold (1 Å in this study), the structure is replaced by a new one. Finally, each A$\cdots$B structure is subjected to geometry optimization.

Among the successfully optimized A ⋯ B complexes, the starting intermediate is selected using the above two criteria: the energy and the valence of the metal center. In this case, the selection is biased towards a low-energy A ⋯ B complex with high valence of the metal, i.e., the metal center presents no coordination vacancies, which ensures a stable intermediate. At any rate, the selection method is not crucial because all intermediates should be obtained in the next step, and its only role is to provide a starting geometry for the accelerated dynamics.

All electronic structure calculations of this step, which include the energies of the structures and the valences of their metal centers, are carried out with MOPAC2016. For the example studied in this work, the latest PM7 Hamiltonian was chosen.

3) Global search of TSs and intermediates. Once a suitable starting intermediate is obtained in the previous step, the TSSCDS machinery comes into play. TSSCDS has been developed very recently by one of the authors.[1] Its main steps are summarized in the following:

i. Optimization and frequency calculation of an initial structure

The first step is the optimization and frequency calculation of the initial structure selected in step 2 using a semi-empirical calculation. Since the starting intermediate has been already optimized in step 2, only a frequency calculation would be needed if normal mode sampling is employed (*vide infra*).

ii. Preparation of the ensemble of molecules for the chemical dynamics simulations

In the current version of our method either microcanonical or canonical ensembles can be initially prepared. The microcanonical normal mode sampling (NMS) of Hase and co-workers[2] is implemented in the package. This type of sampling requires the normal modes of the starting intermediate, which are computed in step *i*.

Additionally a canonical sampling can also be employed, which does not require a frequency calculation. Here the three components of the initial momenta of each atom *i* with mass m_i are taken from a Gaussian distribution[3] with a mean of zero and standard deviation of $(RTm_i)^{1/2}$. Canonical sampling with a temperature of 10000 K was employed for the test case studied in this work. The chemical dynamics simulations of the present work are also constrained, which means that all atoms except the hydrogen of the active catalyst species $HCo(CO)_3$ **1** are fixed during the dynamics, which is achieved by setting an artificially huge mass of 10^{70} a.u. to each of them.

In the original version of TSSCDS, the ensemble of trajectories always started at the same input geometry. An improvement of the method, called iterative TSSCDS (*i*TSSCDS),[4] consists of starting the dynamics from multiple intermediates, which enhances phase space sampling. The latest *i*TSSCDS version was also employed in the present work. More specifically, a first batch of trajectories starting from the starting structure is run. Once some transitions states and intermediates are found (following the steps described below), new batches of trajectories can be run from the newly generated intermediates. In the present work *i*TSSCDS has been improved with respect to the original implementation.[4] Here, the program first checks whether new intermediates are found. If more than one intermediate is already present in the reaction network, it automatically picks one of them to start the trajectories according to its importance in the kinetics. More specifically, it only considers as starting intermediates those visited in a slightly accelerated kinetics simulation using a temperature (or an energy) 1.5 greater than the actual one.

iii. Chemical dynamics simulations

The direct dynamics simulations are carried out using a development version of MOPAC2016.[5] This version employs a step size that can be defined by the user (0.5 fs for the test case selected in this study), and also stops the simulation whenever any of the interatomic distances reaches a distance five times greater than its initial value. Additionally, it incorporates an algorithm developed by one of the authors to sample phase space more efficiently.[6] The maximum simulation time is 500 fs.

iv. Reaction pathway (RP) search algorithm

TSSCDS incorporates an algorithm called bond breakage/formation search (BBFS), which locates saddle points. An additional module of the method also samples torsional TSs. In the following BBFS will be described.

Firstly, a $N(N-1)/2$-dimensional connectivity vector $\mathbf{C}^i = (C_{12}^i, C_{13}^i, \dots, C_{(N-1)N/2}^i)$ is constructed, where the element C_{jk}^i provides information about the connectivity between atoms j and k at step i along the trajectory. Similar vector (or matrix) representations have been employed in the literature to distinguish the different molecular structures.[7]

In the definition of the connectivity vector, two additional vectors are needed: $\mathbf{d}^i$ and $\mathbf{d}^{ref}$, which contain, respectively, the interatomic distances at step i, and some reference distances between each pair of atoms of the system. The vector $\mathbf{d}^i$ can be easily calculated from the Cartesian coordinates $\mathbf{q}^i$. On the other hand, the efficiency of BBFS does not seem to be very sensitive to the actual values of the reference distances. As a rule of thumb the reference distance $d_{jk,ref}$ should be of the order of the sum of the covalent radii of the j and k atoms.[8] The following table shows the elements of $\mathbf{d}^{ref}$ for the study of the cobalt-catalyzed hydroformylation of ethylene.

j	k	d_{jk}^{ref} (Å)
H	Co	2.000
H	C	1.236
H	O	1.236
H	H	0.840
Co	C	2.100
Co	O	2.200
C	C	1.632
C	O	1.632
O	O	1.632

The initial ($i = 0$) connectivity vector $\mathbf{C}^{i=0}$ is defined from $\mathbf{d}^{i=0}$ and $\mathbf{d}^{ref}$ as:

$$C_{jk}^{i=0} = \begin{cases} 1 \text{ if } \delta_{jk}^{i=0} < 1 \\ 0 \text{ otherwise} \end{cases}, \text{ with } \delta_{jk}^{i=0} = \frac{d_{jk}^{i=0}}{d_{jk}^{ref}}$$

where the dimensionless parameter $\delta_{jk}^{i=0}$, called here normalized distance, provides a measure for the initial elongation of the interatomic distance between atoms j and k. The elements $C_{jk}^{i=0}$ can either be one or zero, indicating the existence or the absence of a bond between the jk pair. The way the connectivity vector is updated for any step i along the trajectory is described below. Once $\mathbf{C}$ has been set, a list of neighbors of each atom j are defined as those atoms n for which $C_{jn} = 1$.

In a similar fashion, a list of non-neighbors of j, or outer atoms, can be defined as those atoms o for which $C_{jo} = 1$. Here and after the indices n and o are reserved, respectively, for the neighbors and outer atoms of an atom j.

The following criteria are adopted in the BBFS algorithm to identify RPs and for updating the connectivity vector:

a) A RP takes place when, during a time window Δt of 20 fs, the maximum normalized distance between an atom j and its neighbors becomes greater than the minimum normalized distance between j and the outer atoms:

$$\max\left(\delta_{jn}^{\Delta t}\right) > \min\left(\delta_{jo}^{\Delta t}\right)$$

The step at which the inequality of the above equation is fulfilled for the first time is termed here the transition step (denoted as $i^{\ddagger}$), and therefore Δt ranges from $i^{\ddagger}$ to $i^{\ddagger}$ + 20. When only one (j,n,o) combination of atoms satisfies the above equation within Δt, the process is called single RP. Single RPs are the most common situations.

b) The three elements of the connectivity vector $C_{\alpha\beta}$ (with $\alpha\beta = jn, jo$ and no) will be updated according to the following condition:

$$C_{\alpha\beta}^{i^{\ddagger}+20} = \begin{cases} 1 \text{ if } \delta_{\alpha\beta}^{h} < 1 \\ 0 \text{ otherwise} \end{cases}$$

with h being any step within Δt. The condition of the above equation means that, if the $\alpha\beta$ bond performs an inner turning point (ITP) within Δt at a distance shorter than $d_{\alpha\beta}^{ref}$, atoms α and β are regarded as connected (bonded) at the end of the time window (step $i^{\ddagger}$ + 20).

c) If several (j, n, o) combinations fulfill the inequality given in the previous equation within the same Δt, the corresponding separate processes are merged and regarded as one, which is called a multiple RP. This may occur either because two fast processes occur consecutively, or because of a complex rearrangement with usually more than three atoms involved.

The rationale behind the use of a time window is to avoid TS recrossings can be counted as reactive events. We have chosen a small value for Δt to increase the chances that the multiple RPs actually correspond to a complex process, and not to a sequence of fast mechanisms. On the other hand, Δt is big enough to allow bonds formed by heavy atoms perform at least one ITP, and therefore update **C** properly. Anyway, the value of 20 fs can be changed.

In summary, the BBFS algorithm provides the following data for each trajectory:

1) The number of reactive pathways. This number is typically more than one.
2) For each RP, the corresponding transition step $i^{\ddagger}$.
3) The atoms involved in each RP.

v. Optimization

Finally, a partial optimization of the structures around the transition step $i^{\ddagger}$ is carried out using a semi-empirical calculation (PM7 for the current example) with MOPAC2016. In this optimization, the atoms involved in each RP are frozen. The partially-optimized structures are then subjected to TS optimization using the standard EF algorithm of MOPAC2016.

It is worth mentioning here that BBFS finds transition states for reactions where bonds are broken/formed. Besides those TSs, there are torsional TSs (connecting conformers), which in principle are not captured by BBFS, since no bonds are

broken or formed in the process. As indicated elsewhere,[1, 4] since BBFS entails a TS optimization using the EF algorithm, this may fail, *i.e.*, it may stray from the original guess structure optimizing a torsional TS. The consequence of this is that a number of torsional TSs are actually optimized with BBFS although the method is not designed to find this type of TSs. Nevertheless, to avoid loosing conformational isomers, a new tool (called tors.sh in the package) has been developed to map all possible conformational isomers of a given intermediate. This is done by automatically performing scans about those bonds with bond orders in the range 0.5-1.5, and optimizing the highest energy points in the scans. This is done at low level using MOPAC2016.

vi. Screening

Since the dynamics can visit a particular TS multiple times, there might be duplicates that need to be removed. Also TS structures can be associated to intermolecular interactions (connecting van der Waals intermediates), which sometimes are unimportant. Therefore some screening of the optimized structures is called for.

For the above purposes, the so-called social permutation invariant (SPRINT) topological coordinates,[9] as well as the adjacency and Laplacian matrices are employed.[10] The adjacency matrix defined below is equivalent to the connectivity vector described above, but in matrix form.

In spectral graph theory, a graph (or molecular structure) can be characterized by an adjacency (or contact) matrix A, whose elements a_{ij} are usually defined as:

$$a_{ij} \begin{cases} 1 \text{ if } i \text{ and } j \text{ are connected} \\ 0 \qquad\qquad \text{otherwise} \end{cases}$$

If matrix A represents a connected graph (with only one component), the Perron-Frobenius holds. This theorem states that the eigenvalue of largest modulus λ^{max} is real, positive and non-degenerate. Additionally, its corresponding eigenvector $\mathbf{v}^{max}$ has all components of equal sign. When a molecule dissociates, the corresponding matrix A becomes disconnected (it has at least two fragments or components), and using the above definition of A, the Perron-Frobenius theorem would not hold. This can be avoided by defining the contact matrix elements as:[9]

$$a_{ij} = \frac{1 - (\delta_{ij})^n}{1 - (\delta_{ij})^m}$$

Where ij represent a pair of vertices (atoms), δ_{ij} is the normalized distance. The chosen values for n and m are 6 and 12 as in previous work.[9] The SPRINT coordinates are thus defined as:

$$S_i = \sqrt{N}\lambda^{\mathrm{max}} v_i^{\mathrm{max,sorted}} \quad i = 1, \dots, N$$

Where N is the number of atoms of our system, and the ith component of the eigenvector is taken after sorting from the smallest to the largest value (we adopt the positive sign convention for the components of the eigenvector). The advantage of using these N SPRINT coordinates is that they are invariant with respect to permutation of like atoms, which is very convenient for our purposes.

Besides the above topological coordinates, we also employ the degree d_i of each vertex (or atom) i, which is defined as $d_i = \sum_{j=1}^{N} a_{ij}$, as well as the eigenvalues of the Laplacian matrix. The Laplacian (or Kirchhoff) matrix L is defined as $L = D - A$, where D is the diagonal degree matrix. An important property of this matrix (relevant to our study) is related with the number of zero eigenvalues, which coincides with the number of components of the graph. Since, by

construction, the above adjacency matrix is always connected (only one component), a lower threshold value of 0.005 is set for the eigenvalues. This means that when the two lowest eigenvalues of L are below this threshold, the TS structure is discarded because it corresponds to a van der Waals structure. These tools from spectral graph theory are also employed to compare intermediates.

The above procedure is very effective, spawning thousands of optimized TS structures at the PM7 level. The following table shows the total number of trajectories employed in each sub-system vs the number of transition states optimized at the PM7 level.

sub-system		Number of trajectories	Number of optimized TSs
ss_1	$HCo(CO)_3$	3000	266
ss_2	$HCo(CO)_3/C_2H_4$	14000	442
ss_3	$HCo(CO)_3/CO$	13000	199
ss_4	$HCo(CO)_3/H_2$	9000	105
ss_5	$HCo(CO)_3/C_2H_4/CO$	13000	1257
ss_6	$HCo(CO)_3/C_2H_4/H_2$	14000	236
ss_7	$HCo(CO)_3/CO/H_2$	8000	1110
ss_8	$HCo(CO)_3/C_2H_4/CO/H_2$	19000	1773

The geometries and energies of the TSs obtained with PM7 are only approximate and they must be re-optimized using a higher level of theory; the high level of theory chosen for the hydroformylation of ethylene was B3LYP/6-31G(d,p). Finally, the intermediates are obtained after running IRC calculations from the obtained TS structures. Even though B3LYP/6-31G(d,p) is not the best possible electronic structure protocol to study this reaction,[11] our choice was based on the fact that the most recent automated method applied to the same system employs the same level of theory,[12] which facilitates a direct comparison. We also notice that the computations performed in this work simulate the reactivity in the gas phase. However, according to Harvey's calculations, solvent effects are expected to be unimportant for the system investigated here.

Since the DFT calculations are, by far, the bottleneck of the whole procedure, some screening is called for. In particular, we only considered reaction mechanisms whose TSs have relative free energy values (calculated by DFT), with respect to the starting intermediate, of less than 40 kcal/mol. That cut-off significantly reduces the number of paths considered at the DFT level.

4) Combination of sub-systems. The reaction mechanisms discovered within each sub-system are finally merged into a single reaction network, and a single list of TSs ($\mathbf{TS} = \{TS_1, TS_2, \dots TS_{N_{TS}}\}$) and intermediates ($\mathbf{I} = \{I_1, I_2, \dots I_{N_I}\}$) can be obtained. This step is indispensable to solve the kinetics for the whole system (step 6 of the procedure).

Specifically, the step entails two different tasks, namely: 1) setting a common free energy G scale, and 2) identifying common molecules in different sub-systems. The former is done by adding the values of G for the starting materials that are lacking in sub-system ss_i to the free energies of each TS and intermediate of ss_i. Finally, relative free energy values ΔG are calculated by subtracting the sum of the free energies of the catalyst and the starting materials.

Additionally, intermediates of any sub-system can dissociate releasing several fragments. The fragments can be categorized as: a) products and side products $\mathbf{P} = \{P_1, P_2, \dots P_{N_P}\}$ that do not contain the metal, and b) species containing

the metal. On the one hand, a starting material can be an element of **P**, and, on the other hand, the species containing the metal might be one of the intermediates I_i of a smaller sub-system. To detect those matchings, tools of spectral graph theory, available within TSSCDS, are employed here.

5) Addition of barrier-less reactions. Since the algorithm employed to discover reaction mechanisms is designed to find saddle points, barrierless (dissociative-associative) processes are elusive. The procedure followed here to characterize barrierless reactions consists of the following two steps:

a) Analysis of all intermediates I to check whether their structures contain one or several elements of **P**. A product P_j can be contained within an organometallic complex I_i either because P_j is one of its ligands, or because of non-covalent interactions between P_j and I_i. In practice, the metal center is removed from intermediate I_i and the resulting fragments are compared with the elements of **P**. Then, if a product P_j is one of those fragments, we proceed to step b. Otherwise, we advance to the next intermediate.

b) The original structure of I_i is recovered, and the distance r between the metal and the center of mass of P_j is doubled. The resulting stretched structure is first partially optimized (keeping r constant), followed by a downhill calculation with Gaussian09; both the partial optimization and downhill calculation are carried out at the DFT level for the example studied in this work. If the downhill calculation leads to the original intermediate I_i, the process $I_i \leftrightarrows A_i + P_j$ is regarded barrierless and added to the reaction network, where A_i is the structure that results after removing P_j from I_i.

6) Kinetics simulations. Once a single reaction network is created, the thermal rate coefficient $k_l(T)$ for each process l presenting a barrier is calculated according to transition state theory:

$$k_l(T) = \sigma \frac{k_B T}{h} \left(\frac{RT}{p_0}\right)^{\Delta n} e^{-\frac{\Delta G_l^{\ddagger}}{RT}}$$

where σ is the reaction path degeneracy, T is the temperature, h is Planck's constant, $\Delta G_l^{\ddagger}$ is the free energy of activation, p_0 is 1 bar and Δn = 1 (0) for bimolecular (unimolecular) reactions.

Barrierless associative reactions are assumed to be diffusion-controlled, and their thermal rate coefficients $k_{diff}(T)$ are calculated by the following equation:

$$k_{diff}(T) = \frac{8 k_B T}{3\eta}$$

where η is the viscosity of the solvent (toluene in the example studied here). The rate for the corresponding reverse dissociative process is calculated using detailed balance. A temperature of 423 K was employed to compare with the available experimental and theoretical kinetics results.

Having computed all rate coefficients, the time evolution of all intermediates can be monitored by running KMC simulations, provided the initial concentrations of catalyst and starting materials are known. This task is accomplished using our own KMC code (listed below), as well as the fast open-source program StochKit2.0.[13] As in previous work, the KMC simulations are coarse-grained, i.e., we assume that conformational isomers interconvert very quickly and form a single state, which allows us to extend the simulation time up to minutes.

2. Main computer codes developed in the present work

In the following, the main computer codes developed in this work to study catalysis are collected. There are also a number of specific (awk and bash) scripts employed to study catalysis, which are not included for simplicity. Anyway, TSSCDS1.0 package is freely distributed upon request.

Catalysis.py: This Python code performs step 4

```
#!/usr/bin/python

import sys
import os
import shutil
import math
import subprocess

#Functions
def system_call(command):
	p = subprocess.Popen([command], stdout=subprocess.PIPE, shell=True)
	return p.stdout.read()

def fileread(name):
        with open(name) as f:
                line=f.read().splitlines()
        return line

if not os.path.exists('catalysis_res'):
	os.makedirs('catalysis_res')
	print "catalysis_res does not exist and it'll be created"

if not os.path.exists('catalysis_res/KMC'):
	os.makedirs('catalysis_res/KMC')
	print "catalysis_res/KMC does not exist and it'll be created"

if os.path.isfile('wrkdir'):
	wrkdir=fileread('wrkdir')[0]
else:
	print "wrkdir file is missing"

if os.path.isfile('dynamics.dat'):
        inputfile='dynamics.dat'
else:
        inputfile=wrkdir+'dynamics.dat'

print "Path to inputfile:",inputfile

min_name={}
gap={}
min_form={}
min_form_subs={}
natom={}
energy={}
zpe={}
gcorr={}
TKMC='298'
noHLcalc=0

xyz=fileread(inputfile)
for i in range(len(xyz)):
        col=xyz[i].split()
        if len(col)>0 and col[0]=='species':
                nmolecule=len(col)-1
                min_name=col
        if len(col)>0 and col[0]=='TKMC':
                TKMC=col[1]
        if len(col)>0 and col[0]=='eta':
                eta=col[1]
        if len(col)>0 and col[0]=='HLcalc':
                noHLcalc=col[1]
                if int(noHLcalc)==0:
                        print "The level of theory should be specified"
                        sys.exit(0)
                elif int(noHLcalc)==1:
```

```
                        HLcalc1=col[2]
                        HLcalc=HLcalc1
                        print "\nHL using one level only for energies and frequencies ",HLcalc1
                elif int(noHLcalc)==2:
                        HLcalc1=col[2]
                        HLcalc2=col[3]
                        HLcalc=HLcalc2
                        print "\nHL using two levels ",HLcalc1," and ",HLcalc2
        if len(col)>0 and col[0]=='level1':
                        level1=col[1]
        if len(col)>0 and col[0]=='basis1':
                        basis1=col[1]
        if len(col)>0 and col[0]=='level2':
                        level2=col[1]
        if len(col)>0 and col[0]=='basis2':
                        basis2=col[1]

#Diffusion controlled rate constant calculation
diffbim=open('catalysis_res/diffbim.dat','w')
diffbim.write(str(eta)+' '+str(TKMC))
diffbim.close()
kdiff=float(system_call('diff_bim.exe<catalysis_res/diffbim.dat'))

if int(noHLcalc) == 1:
       print level1,"/",basis1
elif int(noHLcalc)==2:
       print level1,"/",basis1,"//",level2,"/",basis2
print "\nNumber of species=",nmolecule
if nmolecule>4:
       print "right now the maximum number of species is 4"
       sys.exit(0)
for i in range(1,nmolecule+1):
       print "Molecule",i,"=",min_name[i]
print "Temperature of the KMC calcs=",TKMC
print "Diffusion-controlled rate constant=",kdiff," M-1 s-1"

if not min_name[1]=="cat":
       print "The first molecule should be cat"
        sys.exit(0)

#Now we calculate the number of possible combinations of nmolecules
subsystem=['cat']
pairs={}
trios={}
fours={}
i=1
#First the pairs
print "\nCombinations with all molecules"
print "Single"
print 1,subsystem[0]
print "Pairs"
p=0
for i in range(2,nmolecule+1):
       p+=1
       pairs[p]='cat_'+min_name[i]
       print p,pairs[p]
subsystem=subsystem+pairs.values()
print "Trios"
t=0
for i in range(1,p+1):
       for j in range(i+2,nmolecule+1):
                t+=1
               trios[t]=pairs[i]+'_'+min_name[j]
               print t,trios[t]
subsystem=subsystem+trios.values()
print "Fours"
fo=1
fours[fo]=trios[1]+'_'+min_name[nmolecule]
print fo,fours[fo]
subsystem=subsystem+fours.values()
nsubsystem=1+p+t+fo
print "Number of Subsystems=",nsubsystem,"\n"
#We start off optimizing the minima
for i in range(1,nmolecule+1):
       name=min_name[i]
       if os.path.isfile('catalysis_res/'+name+'.log'):
               print name,"alredy optimized"
```

```
        else:
                print name," not optimized"
                fg09=open('catalysis_res/'+name+'.dat','w')
                chk='%chk='+name
                theory='#p '+level1+'/'+basis1+' opt=(calcall,noraman) temperature='+TKMC
##construct g09 input file
                fg09.write(chk+'\n')
                fg09.write(theory+'\n')
                fg09.write('\nmin opt')
                fg09.write('\n\n')
                fg09.write('0 1\n')
                fcoor = name+'.xyz'
                xyz=fileread(fcoor)
                for ii in range(2,len(xyz)):
                        fg09.write(xyz[ii]+'\n')
                fg09.write('\n')
                if int(noHLcalc) ==2:
                        fg09.write("--Link1--\n")
                        fg09.write(chk+'\n')
                         theory='#p '+level2+'/'+basis2+' geom=check'
                         fg09.write(theory+'\n')
# set up cesga script
                cmd='queueHL_sub.sh catalysis_res'+'\t'+ name +' lanza_'+name+'.sh'
                 os.system(cmd)
                sys.exit(0)
cmd='sed "s/\'/ /g" atsymb.f90 >atsdum1'
os.system(cmd)
cmd='sed "s/,/ /g" atsdum1 > atsdum2'
os.system(cmd)
cmd='CATscripts/atsdum2.sh'
os.system(cmd)

cmd='rm -f catalysis_res/geome_* catalysis_res/frequ_* catalysis_res/min0.rxyz'
os.system(cmd)

for i in range(1,nmolecule+1):
        cmd='get_energy'+HLcalc+'.sh catalysis_res/'+min_name[i]+'.log '+noHLcalc
         energy[i]=float(system_call(cmd))
         line=fileread('catalysis_res/'+min_name[i]+'.log')
        with open('catalysis_res/'+min_name[i]+'.log','r') as f:
                for line in f:
                        if 'Zero-point vibrational energy' in line:
                                zpe[i]=float(f.next().split()[0])
                        if 'Thermal correction to Gibbs Free Energy' in line:
                                gcorr[i]=float(line.split()[6])
        cmd='CATscripts/log_to_rxyz.sh
'+min_name[i]+'\t'+str(energy[i])+'\t'+str(zpe[i])+'\t'+str(gcorr[i])+'\t'+str(i)
        os.system(cmd)
        xyz=fileread(min_name[i]+'.xyz')
        natom[i]=int((xyz[0]))
entot=sum(energy.values())
zptot=sum(zpe.values())
gitot=sum(gcorr.values())
natot=sum(natom.values())


fmin0=open('catalysis_res/min0.rxyz','w')
fmin0.write(str(natot)+'\n')
fmin0.write('E= '+str(entot)+' ZPE= '+str(zptot)+' Gcorr= '+str(gitot)+'\n')
fmin0.close()
for i in range(1,nmolecule+1):
        cmd=('cat catalysis_res/geome_'+str(i)+' >> catalysis_res/min0.rxyz')
        os.system(cmd)
for i in range(1,nmolecule+1):
        cmd=('cat catalysis_res/frequ_'+str(i)+' >> catalysis_res/min0.rxyz')
        os.system(cmd)

cmd='rm -f catalysis_res/geome_* catalysis_res/frequ_*'
os.system(cmd)
# e0 is the energy of the separated nmolecule fragments. This is the zero of energy
e0=entot+gitot

enadf=open('catalysis_res/KMC/Energies','w')
rxnet=open('catalysis_res/KMC/RXNet_catalysis','w')
rates=open('catalysis_res/KMC/Rates','w')
correl=open('catalysis_res/KMC/Correl','w')
rxnet.write('Reference system:\n')
```

```
rxnet.write('Klabel    Name   Formula\n')
for i in range(2,nmolecule+1):
       min_form[i]=system_call('CATscripts/FormulaMOL.sh '+min_name[i]+'.xyz')
       rxnet.write(' %2s %10s %10s' % (str(i-1),min_name[i],min_form[i]))
min_form_list=min_form.values()
for i in range(len(min_form_list)):
       min_form_list[i]=min_form_list[i].strip()
rxnet.write('Reference energy: '+str(e0)+' Hartree\n')
enadf.write('Reference energy: '+str(e0)+' Hartree\n')
rxnet.write('Klabel==label employed in the KMC simulations\n')
rxnet.write('Gibbs Free Energy (G) in kcal/mol\n')
rxnet.write('Diffusion-controlled rate constant= '+str(kdiff)+' M-1 s-1\n')
lastmm=nmolecule-1

for i in range(nsubsystem):
       print 'Subsystem ',i+1,' is: ',subsystem[i]

for i in range(nsubsystem):
       gap[i]=lastmm
       rates.write('Subsystem: '+str(i+1)+'\t'+subsystem[i]+'\n')
       correl.write('Subsystem: '+str(i+1)+'\t'+subsystem[i]+'\n')
       tsdirll=wrkdir+'/tsdirLL_'+subsystem[i]
       tsdirhl=wrkdir+'/tsdirHL_'+subsystem[i]
       if not os.path.exists(tsdirhl):
              print tsdirhl,'does not exist\n'
              print 'Complete all calculations before proceeding'
              sys.exit(0)
       min_form_subs[i]=system_call('CATscripts/FormulaMOL.sh '+tsdirhl+'/MINs/min0.rxyz')
       min_form_subs_list=min_form_subs.values()
       for ii in range(len(min_form_subs_list)):
              min_form_subs_list[ii]=min_form_subs_list[ii].strip()
       print '\nSubsystem',i+1,subsystem[i]
       rxnet.write('\nSubsystem '+str(i+1)+' '+subsystem[i]+' '+min_form_subs[i]+'\n')
       rxnet.write('  TSlabel        G_TS          MINlabel Klabel       G_MIN              PRlabel
Klabel        G_PRO  DEG_PATH\n')
       print 'tsdirll= ',tsdirll
       k=0
       edum={}
       for j in range(1,nmolecule+1):
              if not min_name[j] in subsystem[i]:
                     ee=fileread('catalysis_res/'+min_name[j]+'.rxyz')
                     col=ee[1].split()
                     k+=1
                     edum[k]=float(col[1])+float(col[5])
       if k>0:
              enadd=sum(edum.values())
       else:
              enadd=0
       enadf.write('Subsystem '+str(i+1)+' '+subsystem[i]+' Enadd= '+str(enadd)+'\n')
       file=tsdirhl+'/MINs/min0.rxyz'
       if os.path.isfile(file):
              ee=fileread(file)
              col=ee[1].split()
              enall=float(col[1])+float(col[5])+float(enadd)
       else:
              print "wrkdir file is missing"
              print "You should first optimize the global minimum of this subsystem"
              sys.exit(0)
##ediff is the energy that has to be added to every single min and ts of subsystem[i]
       ediff=(enall-e0)*627.51
       rxnetfile=fileread(tsdirhl+'/KMC/RXNet_long.cg_groupedprods')
       ratefile=fileread(tsdirhl+'/KMC/TST/rate'+TKMC+'.out')
       minn={}
       kk=0
       for n in range(2,len(rxnetfile)):
              coll=rxnetfile[n].split()
              kk+=1
              minn[kk]=int(coll[7])
              if coll[9] =='MIN':
                     kk+=1
                     minn[kk]=int(coll[10])
       lastmin=max(minn.values())+gap[i]
       prfile=fileread(tsdirhl+'/PRODs/PRlist_kmc')
       klabel={}
       r1={}
       ts={}
       p1={}
```

```
        p2={}
        kc=0
        for line in range(len(rxnetfile)):
                col=rxnetfile[line].split()
                if line >=2:
                        file=tsdirhl+'/MINs/SORTED/MINlist_sorted'
                        cmd='CATscripts/get_e+g.sh '+file+' '+col[7]
                        emin1=float(system_call(cmd))
                        klabel[int(col[7])]=int(col[7])+gap[i]
                        if klabel[int(col[7])] > lastmm:
                                lastmm=klabel[int(col[7])]
                        if col[9]=='MIN':
                                 cmd='CATscripts/get_e+g.sh '+file+' '+col[10]
                                 emin2=float(system_call(cmd))
                                klabel[int(col[10])]=int(col[10])+gap[i]
                                if klabel[int(col[10])] >lastmm:
                                        lastmm=klabel[int(col[10])]
                                rxnet.write('TS%5s_%s    %8.3f ==>    MIN%5s_%s  %4s      %7.3f    <-->
MIN%5s_%s               %4s      %8.3f\n' %
(col[1],i+1,float(col[4])+ediff,col[7],i+1,klabel[int(col[7])],emin1+ediff,col[10],i+1,klabel[int(col[10])],emin
2+ediff))
                                kc+=1
                                ts[kc]=int(col[1])
                                r1[kc]=klabel[int(col[7])]
                                p1[kc]=klabel[int(col[10])]
                                p2[kc]=0
                                kc+=1
                                ts[kc]=int(col[1])
                                r1[kc]=klabel[int(col[10])]
                                p1[kc]=klabel[int(col[7])]
                                p2[kc]=0
                        if col[9]=='PROD':
                                deg=col[13]
                                code=col[2]
                                for line2 in range(len(prfile)):
                                        col2=prfile[line2].split()
                                        if code in col2[2]:
                                                prcode=col2[1]
                                                prname=fileread(tsdirhl+'/PRODs/CALC/PR'+col2[1]+'_'+col2[2])
                                                cole=prname[1].split()
                                                eprod=float(cole[1])+float(cole[5])+float(enadd)
                                                epr=(eprod-e0)*627.51
                                                del col2[0:3]
                                                matches=list(set(col2)&set(min_form_list))
                                                matches2=list(set(col2)&set(min_form_subs_list))
                                                for ik in range(len(matches)):
                                                        klabel[int(col[10]),ik]=min_form_list.index(matches[ik])+1
                                                if len(matches)==0:
                                                        lastmin+=1
                                                        klabel[int(col[10]),0]=lastmin
                                                        lastmin+=1
                                                        klabel[int(col[10]),1]=lastmin
                                                elif len(matches)==1:
                                                        lastmin+=1
                                                        klabel[int(col[10]),1]=lastmin
                                                pr=' '.join(col2)

                                                if len(matches2)==1:
                                                        indxs=min_form_subs_list.index(matches2[0])
                                                        matchmin=open('catalysis_res/matchmin.dat','w')
                                                        matchmin.write('Where= '+subsystem[indxs]+'\n')
                                                        matchmin.write('From= '+subsystem[i]+'\n')
                                                        matchmin.write(min_form_subs[indxs]+' '+str(prcode)+'\n')
                                                        matchmin.write(HLcalc+' '+noHLcalc+'\n')
                                                        matchmin.close()
                                                         minp=system_call('CATscripts/matchmin.sh
catalysis_res/matchmin.dat')

                                                        os.system('rm -f catalysis_res/matchmin.dat')
                                                        if int(minp)>0:
                                                                klabel[int(col[10]),1]=int(minp)+gap[indxs]
                                                                lastmin-=1
                                                if klabel[int(col[10]),1] > lastmm:
                                                        lastmm=klabel[int(col[10]),1]
                                                deltag=float(col[4])+ediff-epr
                                                tstbim=open('catalysis_res/tstbim.dat','w')
                                                tstbim.write(str(deltag)+' '+str(TKMC)+' '+str(deg))
                                                tstbim.close()
```

```
                                            krev=float(system_call('tst_bim.exe<catalysis_res/tstbim.dat'))
                                            if krev>kdiff:
                                                   krev=kdiff
                                            os.system('rm -f catalysis_res/tstbim.dat')
                                            rates.write('%30s %4s %4s %4s    0\n' %
(krev,klabel[int(col[10]),0],klabel[int(col[10]),1],klabel[int(col[7])]))
                                            correl.write('%30s %4s %4s %4s    0 TS%5s_%s\n' %
(krev,klabel[int(col[10]),0],klabel[int(col[10]),1],klabel[int(col[7])],col[1],i+1))
                                            rxnet.write('TS%5s_%s    %8.3f ==>    MIN%5s_%s  %4s      %7.3f
<-->   %-18s %4s +%4s      %8.3f  %4s\n' %
(col[1],i+1,float(col[4])+ediff,col[7],i+1,klabel[int(col[7])],emin1+ediff,pr,klabel[int(col[10]),0],klabel[int(
col[10]),1],epr,deg))
                                            kc+=1
                                            ts[kc]=col[1]
                                            r1[kc]=klabel[int(col[7])]
                                            p1[kc]=klabel[int(col[10]),0]
                                            p2[kc]=klabel[int(col[10]),1]
       ikc=0
       for line2 in range(len(ratefile)):
              col=ratefile[line2].split()
              ikc+=1
              if klabel.has_key(int(col[2])):
                     rates.write('%30s %4s    0 %4s    0\n' % (col[0],r1[ikc],p1[ikc]))
                     correl.write('%30s %4s    0 %4s    0 TS%5s_%s\n' % (col[0],r1[ikc],p1[ikc],ts[ikc],i+1))
              else:
                     rates.write('%30s %4s    0 %4s %4s\n' % (col[0],r1[ikc],p1[ikc],p2[ikc]))
                     correl.write('%30s %4s    0 %4s %4s TS%5s_%s\n' %
(col[0],r1[ikc],p1[ikc],p2[ikc],ts[ikc],i+1))
rxnet.close()
rates.close()
correl.close()
enadf.close()
```

Barrierless_CAT.sh: This bash script performs (alongside with Barrierless2_CAT.sh) step 5.

```
###########
#This script finds reactions that proceed without a barrier:
# for instance:
# Here, we look for shallow minima (of any type that can dissociate)
#it puts both fragments some distance appart and then it runs downhill calculation
#This script performs g09 calculations and the second part does the analysis
##########
if [ $# -eq 0 ]; then
   ci=1
elif [ $1 -eq 0 ]; then
   ci=0
else
  ci=1
fi

bd=barrierless_diss
if [ -f wrkdir ]; then
   wrkdir=`awk '{print $0}' wrkdir `
   echo "wrkdir is" $wrkdir
else
   echo "wrkdir file is missing"
   exit
fi
if [ -f dynamics.dat ];then
   echo "dynamics.dat is in the current dir"
   inputfile=dynamics.dat
else
   echo "read catalysis file from the wrkdir"
   inputfile=$wrkdir/dynamics.dat
fi
if [ -d $bd ];then
   echo "$bd already exists"
   exit
else
   mkdir $bd
fi
catsum=$wrkdir/catalysis_res/KMC/RXNet_catalysis
awk '{if(NR>2){
  if(NF==3) {
    print $3
    }
   else
```

```
    exit
  }
}' $catsum >prods0.log
vdw=`awk 'BEGIN{vdw=0};{if($1=="vdw") vdw=1};END{print vdw}' $inputfile `
if [ $vdw -eq 1 ]; then
   cp thdist thdist_backup
   nvdw=`awk '{if($1=="vdw") print $2}' $inputfile `
   echo $nvdw "Van der Waals distances to be taken into account"
   for i in $(seq 1 $nvdw)
   do
      at1=`awk '/vdw/{i=1; while(i<='$i'){getline;if(i=='$i')print $1; i++}  }' $inputfile `
      at2=`awk '/vdw/{i=1; while(i<='$i'){getline;if(i=='$i')print $2; i++}  }' $inputfile `
      dis=`awk '/vdw/{i=1; while(i<='$i'){getline;if(i=='$i') print $3; i++}  }' $inputfile `
      echo "Distance $i between atoms ${at1} and ${at2} is $14"
      awk '{if($1=="'${at1}'" && $2=="'${at2}'") {print $1,$2,"'${dis}'"}
      else if($2=="'${at1}'" && $1=="'${at2}'") {print $1,$2,"'${dis}'"}
      else {print $0}
      } ' thdist >thdist_vdw
      cp thdist_vdw thdist
   done
fi

echo "Summary of the calcs" > $bd/barrierless.dat
echo "********************" >> $bd/barrierless.dat
TKMC=`awk '{if($1 == "TKMC") {print $2;nend=1}};END{if(nend==0) print "298"}' $inputfile`
charge=`awk 'BEGIN{ch=0};{if($1=="charge") ch=$2};END{print ch}' $inputfile `
mult=`awk 'BEGIN{mu=1};{if($1=="mult") mu=$2};END{print mu}' $inputfile `
noHLcalc=`awk '/HLcalc/{print $2}' $inputfile`
if [ $noHLcalc -eq 1 ]; then
   HLcalc1=`awk '/HLcalc/{print $3}' $inputfile`
   level1=`awk '{if($1=="level1") print $2}' $inputfile`
   basis1=`awk '{if($1=="basis1") print $2}' $inputfile`
   echo "HL using one level only for energies and frequencies" $HLcalc1
   HLcalc=$HLcalc1
elif [ $noHLcalc -eq 2 ]; then
   HLcalc1=`awk '/HLcalc/{print $3}' $inputfile`
   HLcalc2=`awk '/HLcalc/{print $4}' $inputfile`
   level2=`awk '{if($1=="level2") print $2}' $inputfile`
   basis2=`awk '{if($1=="basis2") print $2}' $inputfile`
   echo "HL using two levels" $HLcalc1 "and" $HLcalc2
   HLcalc=$HLcalc2
else
   echo "check the input file "
fi

sed 's/level1/'$level1'\/'$basis1'/g' hl_input_template | sed 's/charge/'$charge'/g' | sed 's/mult/'$mult'/g'  >
g09inp0
sed 's/tkmc/'$TKMC' calc/g' g09inp0 | sed 's/opt=(ts,noeigentest,calcall,noraman)//g'  >g09inp

sed "s/'/ /g" atsymb.f90 >atsdum1
sed "s/,/ /g" atsdum1 > atsdum2
sed "s/+00/+00 /g" atsdum2 > atsdum3
awk '/character/{++nt}
/end/{exit}
{i0=1;if($1 ~ /character/) i0=3
for(i=i0;i<=(NF-1);i++) if(nt>=1) print $i
} ' atsdum2  > atsdum2.out
awk '/ams=/{amlab=1}
/character/{++nt}
{for(i=1;i<=(NF-1);i++) {if($1 != "real" && amlab==1 && nt==0) {++j;m[j]=$i} }}
/end/{exit}
{i0=1;if($1 ~ /character/) i0=3
for(i=i0;i<=(NF-1);i++) if(nt>=1) {++k;print $i,m[k]}
} ' atsdum3 > atsdum3.out


awk '{if(NF==4) print $0}' cat.xyz >mingeom0
met_label=`awk '{if( NR == FNR) {l[NR]=$1;tne=NR}}
{if(FNR > 1 && NR >FNR ) {
   IGNORECASE = 1
   i=1
   while(i<=tne){
      if( $1 == l[i] && i>=21 && i<=30) print $1
      if( $1 == l[i] && i>=39 && i<=48) print $1
      if( $1 == l[i] && i>=72 && i<=80) print $1
      i++
      }
```

```
  }
}' atsdum2.out mingeom0`
awk '{if($1=="'$met_label'") {print NR-2;exit}
}' cat.xyz >atoms_to_remove
nmet_label=`awk '{print $1}' atoms_to_remove`
rm -f minn minn.log
$wrkdir/CATscripts/subsystems.py >subsystems.out
#Getting other possibilities from tsdirHL
n=0
tc=0
#Search for possible products besides the prods0.log
if [ $ci -eq 1 ]; then
   echo "possible prods"> prods
   set `awk '{print $0}' subs`
   for i
   do
      prodir=$wrkdir"/tsdirHL_"$i"/PRODs/CALC"
      if [ -d $prodir ]; then
         set `ls $prodir/*.log | awk '{print $1}' `
         for j
         do
           pr=`basename $j`
           echo $pr >pr
           sed 's/\./ /g' pr | sed 's/\-/ /g' | sed 's/m//g'  >pr.log
           prod=`awk '{if($1 !~/'$met_label'/) print $1}' pr.log`
           multi=`awk '{print $3}' pr.log`
           if [ $multi -eq 1 ] && [ ! -z $prod ] ; then
              get_energy$HLcalc.sh $j $noHLcalc >en
              get_gcorr.sh $j >>en
              en=`awk '{e[NR]=$1};END{printf "%20.10f",e[1]+e[2]}' en`
              echo $prod $en >> prods
           fi
         done
      fi
   done
   awk '{if(NR>1) {++i;p[i]=$1;tot=i;pe[i]=$0}}
   END{i=1
   while(i<=tot){ok=1
      j=1
      while(j<i){
        if(p[i]==p[j]) {ok=0;break}
        j++
        }
      if(ok==1) print pe[i]
      i++
      }
   }' prods >prods.out
   paste prods0.log prods.out > prod_comp
   awk '{if(NF==3) {++i;ni[i]=$1;tot=i;p[NR]=$2;e[NR]=$3}}
   {if(NF==2) p[NR]=$1;e[NR]=$2}
   END{i=1
   while(i<=NR){ok=1
      j=1
      while(j<=tot){
        if(p[i]==ni[j]) {ok=0;break}
        j++
        }
      if(ok==1) print p[i],e[i]
      i++
      }
   }' prod_comp>prods.log
fi

nprod=`wc -l prods.log | awk '{print $1}'`
nprod0=`wc -l prods0.log | awk '{print $1}'`
nc=0
tc=0
set `awk '{print $0}' subs`
for i
do
   ((nc=nc+1))
   echo "" >> $bd/barrierless.dat
   echo "System: $i" >> $bd/barrierless.dat
   echo "*******" >> $bd/barrierless.dat
   ((n=n+1))
# ${frag[$j]}
   minfile=$wrkdir"/tsdirHL_"$i"/MINs/SORTED/MINlist_sorted"
```

```
   mindir=$wrkdir"/tsdirHL_"$i"/MINs/SORTED"
   confile=$wrkdir"/tsdirHL_"$i"/working/conf_isomer.out"
   minn=`awk '/min0/{print $2}' $minfile`

   res=`awk 'BEGIN{res=0}
   {for(i=1;i<=NF;i++) if($i=='$minn') res=$1}
   END{
   print res
   }' $confile`
   if [ $res -gt 0 ]; then
      minn=$res
   fi
   $wrkdir/CATscripts/get_minn_fromoverall.sh $catsum $minn $nc 20
   set `awk '{print $0}' minn`
   for j
# Loop over the structures (most important minima) of a given subsystem
   do
      min=$mindir/MIN$j"_*.rxyz"
      min_name=`basename $min`
      awk '{if(NF==4) print $0}' $min >mingeom0
      natom=`wc -l mingeom0 | awk '{print $1}'`
      echo $natom > filemin
      echo "" >> filemin
      cat mingeom0 >> filemin
#remove only the metal
      cat atoms_to_remove mingeom0 > pmg
      awk '{if(NF==1) {++i;lab[i]=$1;tot=i}}
      {if(NF==4){ok=1;++k
        i=1
        while(i<=tot){
          if(k==lab[i]) {ok=0;break}
          i++
          }
        if(ok==1) print $0
        }
      }' pmg >mingeom
#mingeom does not contain catalyzer now

      $wrkdir/createthdist.sh
      $wrkdir/createMat.sh
#       ConnMat Look for fragments and choose appropriate ones
# now identify two or more fragments (if there is more than one)
      awk 'END{print NR"\n"}' mingeom> geom
      cat mingeom >> geom
      $wrkdir/CATscripts/FormulaPROD_ef.sh geom > molec
      nmolec=`wc -l molec | awk '{print $1}'`
      for inmp0 in $(seq 1 $nprod0)
      do
          m[$inmp0]=1
      done
      for nm in $(seq 1 $nmolec)
      do
         molec=`awk 'NR=='$nm',NR=='$nm'{print $1}' molec`
######First, the initial prods (prods0)
         for nmp0 in $(seq 1 $nprod0)
         do
            prodi0=`awk 'NR=='$nmp0',NR=='$nmp0'{print $1}' prods0.log`
            if [ "$molec" == "$prodi0" ] && [ ${m[$nmp0]} -eq 1 ] ; then
               m[$nmp0]=0
              ((tc=tc+1))
              natomB=`awk 'NR==1,NR==1{print $1}' frag$nm.xyz`
               ((natomA=natom-natomB))
              echo "$min_name of subsystem $i leading to $prodi0"
              echo "$min_name of subsystem $i leading to $prodi0" >>$bd/barrierless.dat
               if [ $noHLcalc -eq 1 ]; then
                 $wrkdir/CATscripts/balecalc.sh $natom $natomA $tc $bd $wrkdir $nm $nmet_label 1
               elif [ $noHLcalc -eq 2 ]; then
                 $wrkdir/CATscripts/balecalc.sh $natom $natomA $tc $bd $wrkdir $nm $nmet_label 2 $charge $mult
$level2 $basis2
               fi
               $wrkdir/CATscripts/queueHLoptdown_sub.sh $bd  diss$tc lanza$tc $tc $wrkdir
               $wrkdir/queueHL_sub.sh $bd  fragtoop$tc lanzaop$tc $tc
            fi
         done
######Now, other prods (prods)
         for nmp in $(seq 1 $nprod)
         do
```

```
            prodi=`awk 'NR=='$nmp',NR=='$nmp'{print $1}' prods.log`
            if [ "$molec" == "$prodi" ]; then
               ((tc=tc+1))
               natomB=`awk 'NR==1,NR==1{print $1}' frag$nm.xyz`
                ((natomA=natom-natomB))
               echo "$min_name of subsystem $i leading to $prodi"
               echo "$min_name of subsystem $i leading to $prodi" >>$bd/barrierless.dat
                if [ $noHLcalc -eq 1 ]; then
                  $wrkdir/CATscripts/balecalc.sh $natom $natomA $tc $bd $wrkdir $nm $nmet_label 1
                elif [ $noHLcalc -eq 2 ]; then
                  $wrkdir/CATscripts/balecalc.sh $natom $natomA $tc $bd $wrkdir $nm $met_label 2 $charge $mult
$level2 $basis2
                fi
                $wrkdir/CATscripts/queueHLoptdown_sub.sh $bd  diss$tc lanza$tc $tc $wrkdir
                $wrkdir/queueHL_sub.sh $bd  fragtoop$tc lanzaop$tc $tc
            fi
         done

      done
   done
done
```

Barrierless2_CAT.sh: This bash script performs (alongside with Barrierless_CAT.sh) step 5.

```
dir=barrierless_diss
file=$dir/barrierless.dat
if [ -f wrkdir ]; then
   wrkdir=`awk '{print $0}' wrkdir `
   echo "wrkdir is" $wrkdir
else
   echo "wrkdir file is missing"
   exit
fi
catsum=$wrkdir/catalysis_res/KMC/RXNet_catalysis
ratsum=$wrkdir/catalysis_res/KMC/Rates
correlsum=$wrkdir/catalysis_res/KMC/Correl
cp $ratsum ratsum
cp $catsum catsum
cp $correlsum correlsum
lastkmc=`awk 'BEGIN{max=0};{if(NF==5)
{if($2>max)max=$2;if($3>max)max=$3;if($4>max)max=$4;if($5>max)max=$5}};END{print max}' ratsum  `
if [ -f dynamics.dat ];then
   echo "dynamics.dat is in the current dir"
   inputfile=dynamics.dat
else
   echo "read catalysis file from the wrkdir"
   inputfile=$wrkdir/dynamics.dat
fi
eta=`awk '{if($1=="eta") print $2}' dynamics.dat`
tkmc=`awk '{if($1=="TKMC") print $2}' dynamics.dat`
noHLcalc=`awk '/HLcalc/{print $2}' $inputfile`
if [ $noHLcalc -eq 1 ]; then
   HLcalc1=`awk '/HLcalc/{print $3}' $inputfile`
   level1=`awk '{if($1=="level1") print $2}' $inputfile`
   basis1=`awk '{if($1=="basis1") print $2}' $inputfile`
   echo "HL using one level only for energies and frequencies" $HLcalc1
   HLcalc=$HLcalc1
elif [ $noHLcalc -eq 2 ]; then
   HLcalc1=`awk '/HLcalc/{print $3}' $inputfile`
   HLcalc2=`awk '/HLcalc/{print $4}' $inputfile`
   level2=`awk '{if($1=="level2") print $2}' $inputfile`
   basis2=`awk '{if($1=="basis2") print $2}' $inputfile`
   echo "HL using two levels" $HLcalc1 "and" $HLcalc2
   HLcalc=$HLcalc2
else
   echo "check the input file "
fi

awk '/leading/{print $0}' $file > dumigb
if [ ! -f $catsum ]; then
   echo "Please, run catalysis.py first"
   exit
fi
sed 's/MIN//g' dumigb | sed 's/_/ /g'  >dumigb.log
nt=0
set `awk '{print NR}' dumigb`
for i
```

```
do
   ((nt=nt+1))
   subs=`awk 'NR=='$i',NR=='$i'{print $4}' dumigb`
###
   natom=`$wrkdir/CATscripts/get_n_atoms.sh $dir/diss$nt.log`
###
   natoma=`$wrkdir/CATscripts/get_n_atoms.sh $dir/fragtoop$nt.log`
   ((natomb=natom-natoma))
   cn=$nt
   min=`awk 'NR=='$nt',NR=='$nt'{print $1}' dumigb.log`
   if [ ! -f $dir/down$cn.log ];then
      echo "Please, run Barrierless_CAT.sh first"
      exit
   fi
   flb=`$wrkdir/CATscripts/get_dist.sh $dir/down$cn.log $natoma $wrkdir`
#only the barrierless channels ($flb -eq 1)  are analyzed
   if [ $flb -eq 1 ]; then
      echo $natoma > geoma
      echo "" >> geoma
      $wrkdir/CATscripts/get_lastgeomg09.sh  $dir/fragtoop$cn".log" >>geoma
      formul_a=`$wrkdir/CATscripts/FormulaMOL.sh geoma`
      where=`awk '/Subsystem/{if($4=="'$formul_a'") print $3}' $catsum`
      formul_b=`awk 'NR=='$nt',NR=='$nt'{print $NF}' dumigb`
      formul_ab=`echo "$formul_b+$formul_a"`
      subi=`awk '/Subsystem/{if($3=="'$subs'") {print $2;exit}}' $catsum`
      minkmc=`awk '/Subsystem/{if($3=="'$subs'") {subi=$2;ok=1}}
      {for(i=1;i<=NF;i++) if($i=="'$min'_"subi && ok==1 && i>2) {print $(i+1);exit}}' $catsum`
      gmin=`awk '/Subsystem/{if($3=="'$subs'") {subi=$2;ok=1}}
      {for(i=1;i<=NF;i++) if($i=="'$min'_"subi && ok==1 && i>2) {print $(i+2);exit}}' $catsum`

      if [ ! -z $where ]; then
         echo "Where= $where" > matchmin2.dat
         echo $HLcalc $noHLcalc >> matchmin2.dat
         fragmin=`$wrkdir/CATscripts/matchmin2.sh matchmin2.dat $dir/fragtoop$cn".log"`
         if [ $fragmin -gt 0 ]; then
            fragminkmc=`awk '/Subsystem/{if($3=="'$where'") {subi=$2;ok=1}}
            {for(i=1;i<=NF;i++) if($i=="'$fragmin'_"subi && ok==1 && i>2) {print $(i+1);exit}}' $catsum`
###the min might not be in RXNet    because it might be connected by a higher enery barrier
            if [ -z $fragminkmc ]; then
               ((fragminkmc=lastkmc+1))
               lastkmc=$fragminkmc
            fi
         else
            ((fragminkmc=lastkmc+1))
            lastkmc=$fragminkmc
         fi
      else
######emilio
         ((fragminkmc=lastkmc+1))
         lastkmc=$fragminkmc
      fi
      get_energy$HLcalc.sh $dir/fragtoop$cn".log" $noHLcalc >gfragmin
      get_gcorr.sh $dir/fragtoop$cn".log" >>gfragmin
      nif=`$wrkdir/CATscripts/get_NIF.sh $dir/fragtoop$cn".log" `
      if [ $nif -gt 0 ]; then
         echo "Calc. number $cn barrierless dissociation. One fragment has $nif imaginary frequencies...skip it"
      fi
      awk '/Reference energy:/{printf "%20.10f\n",$3}' $catsum >gpr
      awk '/Subsystem/{if($2=='$subi') printf "%20.10f\n",$NF}' $wrkdir/catalysis_res/KMC/Energies >>gpr
      awk '{e[NR]=$1};END{printf "%20.10f\n",e[1]+e[2]}' gfragmin >>gpr
      awk '{if($1=="'$formul_b'") printf "%20.10f\n",$2}' prods.out >>gpr
      gpr=`awk '{e[NR]=$1};END{print (e[3]+e[4]+e[2]-e[1])*627.51}' gpr`
#this is the minimum}
      frb=$formul_b
      frbkmc=`awk '{if(NR>2 && NF==3 && $3=="'$frb'") {print $1;exit} }' $catsum`
      if [ -z $frbkmc ]; then
         ((frbkmc=lastkmc+1))
         lastkmc=$frbkmc
      fi
      echo $gpr >dif
      echo $gmin >>dif
      deltag=`awk 'BEGIN{dif=0};{e[NR]=$1};END{printf "%8.3f\n",e[1]-e[2]}' dif`
#      dif=`awk 'BEGIN{dif=0};{e[NR]=$1};END{diff=e[1]-e[2];if(diff>0) dif=1;print dif}' dif`
#      if [ $dif -eq 0 ]; then
#         echo "Calc. number $cn barrierless dissociation. DeltaG is negative ( $deltag  )...skip it"
#      fi
#      awk '/Reference energy:/{printf "%20.10f\n",$3}' $catsum >gpr
```

```
#      if [ $dif -eq 1 ] && [ $nif -eq 0 ] ; then
      if [ $nif -eq 0 ] ; then
         echo "Calc. number $cn barrierless dissociation from min: (orig: $min of $subs) $minkmc $gmin <-->
$fragminkmc + $frbkmc  $gfragmin "
         echo $eta $deltag $tkmc > diff_and_diss.dat
         $wrkdir/diff_and_diss.exe <diff_and_diss.dat>diff_and_diss.out
         kdiff=`awk 'NR==1,NR==1{print $1}' diff_and_diss.out`
         kdiss=`awk 'NR==2,NR==2{print $1}' diff_and_diss.out`
         awk '{if($1=="Subsystem:" && $2=="'$subi'") {
            print $0
           printf "%30s %4s %4s %4s    0\n",'$kdiff','$fragminkmc','$frbkmc','$minkmc'
           printf "%30s %4s    0 %4s %4s\n",'$kdiss','$minkmc','$fragminkmc','$frbkmc'
            }
         else
           print $0
         }' ratsum > ratsum.log
         cp ratsum.log ratsum
###
         awk '{if($1=="Subsystem:" && $2=="'$subi'") {
            print $0
           printf "%30s %4s %4s %4s    0 Gprod=%10s\n",'$kdiff','$fragminkmc','$frbkmc','$minkmc','$gpr'
           printf "%30s %4s    0 %4s %4s Gprod=%10s\n",'$kdiss','$minkmc','$fragminkmc','$frbkmc','$gpr'
            }
         else
           print $0
         }' correlsum > correlsum.log
         cp correlsum.log correlsum
###
         awk '{if($1=="Subsystem" && $2=="'$subi'") add=1}
         {if($1=="TSlabel" && add==1)  {
            print $0
           printf "TS   null        null ==>    MIN    '$min'_'$subi'    '$minkmc'       '$gmin'    <-->
'$formul_a' + '$formul_b'            '$frbkmc' + '$fragminkmc'         '$gpr'\n"
            add=0
            }
         else
           print $0
         }' catsum > catsum.log
         cp catsum.log catsum
      fi
   else
      echo "Calc. number $cn barrierless dissociation presumably has a barrier...skip it"
   fi
done
$wrkdir/CATscripts/reunite_prods.sh
cp dratsum $ratsum"_with_barrierless"
cp dcatsum $catsum"_with_barrierless"
cp dcorrelsum $correlsum"_with_barrierless"
rm -f ratsum* catsum* dratsum ddratsum dcatsum ddcatsum correlsum*
```

kmc.f90: This fortran90 code performs step 6 (alongside with rate_dependence1.py and rate_dependece2.py).

```
program kmc
implicit none

! rate: rate constant
! p:    population of a given species (p0 is its initial value)
! re (pr): reactant (product) for a given process
! vol is the volume in l
! The vol relates to population of species i p_i and concentration c_i as:
! vol=p_i/c_i/avog
! nespct is the number of species with constant values of population throughout
! the simulation.

character*80 :: title
integer, parameter :: dp = selected_real_kind(15,307)
integer :: m,nran,nr,nesp,inran,i,j,k,l,mu,pd,kk,ia,iz,ijk,nespct,spp
real(dp) :: t,tmax,tprint,tint,a0,r2a0,suma,vol,avog,conv
real(dp) :: rnd(2)
integer,dimension(:),allocatable :: re1,re2,pr1,pr2,n,iespct
real (dp) ,dimension(:),allocatable :: a,rate,c0,p,p0,cont
avog=6.022d23
read(*,"(a80)") title
print "(t3,a80)",title
read(*,*) m,nesp,nran
print*, m,nesp,nran
allocate(re1(m),re2(m),pr1(m),pr2(m),a(m),rate(m),p0(nesp),p(nesp),n(nesp),c0(nesp),cont(m))
```

```
n=(/ (l,l=1,nesp) /)
read(*,*) vol
conv=vol*avog
read(*,*) nespct
print*, "Number of species that keep their concentrations constant=",nespct
allocate(iespct(nespct))
do i=1,nespct
   read(*,*) iespct(i)
   print*, iespct(i)
enddo
print*, ""
print*, "Rates in (time-1),i.e.,s-1"
do i=1,m
   read(*,*) rate(i),re1(i),re2(i),pr1(i),pr2(i)
   if(re1(i)/=0.and.re2(i)/=0) rate(i)=rate(i)/conv
   print*, i,rate(i),re1(i),re2(i),pr1(i),pr2(i)
   if(re1(i)==re2(i)) rate(i)=2*rate(i)
enddo
print*, "Initial concentration (M) and population of each species"
do ijk=1,m
   cont(ijk)=0
enddo
do i=1,nesp
   read(*,*) c0(i)
   p0(i)=c0(i)*conv
   print*, i,c0(i),p0(i)
enddo
read(*,*) tmax,tint
ia=1
iz=nesp
print "(/,t3,a,1p,e10.2,a,/,t3,a,1p,e10.2,a,/)","Total time=",tmax," in input units","Step size =",tint," in 
input units"
big: do inran=1,nran
   print "(/,t3,a,i4,/,t3,a,500(i10))","Calculation number",inran, &
 "Time    ",(n(i),i=ia,iz)
   p=p0
   t=0.d0
   tprint=0.d0
   do while(tprint<tmax)
! keep the initial population for the iespct species
     do j=1,nespct
        p(iespct(j))=p0(iespct(j))
     enddo
     do j=1,m
        if(re1(j)==0) then
! unimolecular reaction
          a(j)=rate(j)*p(re2(j))
        else if(re2(j)==0) then
          a(j)=rate(j)*p(re1(j))
        else
          if(re1(j)/=re2(j)) a(j)=rate(j)*p(re1(j))*p(re2(j))
          if(re1(j)==re2(j)) a(j)=rate(j)*p(re1(j))*(p(re2(j))-1)/2
        endif
     enddo
     a0=sum(a)
     call random_number(rnd)
     t=t-log(rnd(1))/a0
     do while (t>=tprint)
        print*, "Time=",tprint
        do spp=1,nesp
           if(p(spp)>0) print "(t3,i10,f15.0))",spp,p(spp)
        enddo
        print*,
        tprint=tprint+tint
        if(tprint>tmax) cycle big
     enddo
     r2a0=rnd(2)*a0
     suma=0.d0
     s1: do mu=1,m
       suma=suma+a(mu)
       if(suma>=r2a0) exit s1
     enddo s1
     if(re1(mu)==0) then
! unimolecular reaction
        p(re2(mu))=p(re2(mu))-1
     else if(re2(mu)==0) then
        p(re1(mu))=p(re1(mu))-1
```

```
! unimolecular reaction
     else
        p(re1(mu))=p(re1(mu))-1
        p(re2(mu))=p(re2(mu))-1
     endif
     if(pr1(mu)==0) then
! only one product
        p(pr2(mu))=p(pr2(mu))+1
     else if(pr2(mu)==0) then
        p(pr1(mu))=p(pr1(mu))+1
     else
        p(pr1(mu))=p(pr1(mu))+1
        p(pr2(mu))=p(pr2(mu))+1
     endif
     cont(mu)=cont(mu)+1
   enddo
enddo big
print*,"Population of every species"
do i=1,nesp
   print "(i6,f20.0)",i,p(i)
enddo
print*,"counts per process"
do i=1,m
   print "(i6,f30.0)",i,cont(i)
enddo
end program kmc
```

rate_dependence1.py: This Python code performs step 6 (alongside with kmc.f90 and rate_dependece2.py).

```
#!/usr/bin/python

import sys
import os
import shutil
import math
import subprocess

#Functions
def system_call(command):
       p = subprocess.Popen([command], stdout=subprocess.PIPE, shell=True)
       return p.stdout.read()


def fileread(name):
        with open(name) as f:
                line=f.read().splitlines()
        return line

#use os.path.isfile to check whether a file exists
if os.path.isfile('wrkdir'):
        wrkdir=fileread('wrkdir')[0]
else:
        print "wrkdir file is missing"

cmd=wrkdir+'/CATscripts/create_kmc_template.sh'
os.system(cmd)

nsteps=50
j=0
#
dc0={}
dc0_max={}
sol={}
inputfile=wrkdir+'/catalysis_res/KMC/kmc_template.dat'
makekmc=wrkdir+'/CATscripts/makekmci.sh'
makekmci=wrkdir+'/catalysis_res/KMC/makekmcinput'
icon=fileread(inputfile)
for i in range(len(icon)):
       col=icon[i].split()
       if 'C0' in col[0]:
               j+=1
               dc0[j]=float(col[1])
               dc0_max[j]=float(col[2])
               if (len(col))==4:
                       sol=float(col[3])
                       dc0[j]=dc0[j]*sol
                       dc0_max[j]=dc0_max[j]*sol
```

```
c0i=dc0.values()
c0_max=dc0_max.values()
dflag={}
dc0f={}

for ie in range(len(c0i)):
        dflag[ie]='C0_'+str(ie+1)
flag=dflag.values()

n=0
for ie in range(len(c0i)):
       print "Varying concentration of species ",ie+1
       for i in range(nsteps):
              n+=1
              delta=float(c0_max[ie]/nsteps)
              c0x=delta*(i+1)
              for iei in range(len(c0i)):
                     if iei==ie:
                            dc0f[iei]=c0x
                     else:
                            dc0f[iei]=c0i[iei]
              c0f=dc0f.values()
              makekmcinput=open(makekmci,'w')
              for jk in range(len(c0i)):
                     makekmcinput.write('C0_'+str(jk+1)+'\t'+str(c0f[jk])+'\n')
              makekmcinput.close()
              outputfile=wrkdir+'/catalysis_res/KMC/kmc_files/kmc_'+str(ie)+'_'+str(i)+'.dat'
              cmd=makekmc+'\t'+makekmci+'\t'+inputfile+'\t'+outputfile
              os.system(cmd)
              lanza=wrkdir+'/catalysis_res/KMC/kmc_files/lanza_'+str(n)+'.sh'
              with open(lanza,'w') as file:
                     file.write('cd '+wrkdir+'/catalysis_res/KMC/kmc_files\n')

       file.write(wrkdir+'/kmc_bim.exe<kmc_'+str(ie)+'_'+str(i)+'.dat>kmc_'+str(ie)+'_'+str(i)+'.out\n')
              cmd=wrkdir+'/CATscripts/queue_kmc.sh '+lanza
              os.system(cmd)
```

rate_dependence2.py: This Python code performs step 6 (alongside with kmc.f90 and rate_dependece1.py).

```
#!/usr/bin/python

import sys
import os
import shutil
import math
import subprocess
import numpy as np

#Functions
def system_call(command):
       p = subprocess.Popen([command], stdout=subprocess.PIPE, shell=True)
       return p.stdout.read()

def fileread(name):
        with open(name) as f:
                line=f.read().splitlines()
        return line

if os.path.isfile('wrkdir'):
        wrkdir=fileread('wrkdir')[0]
else:
        print "wrkdir file is missing"

nsteps=50
j=0
#
dc0_max={}
sol={}
originpf='dynamics.dat'
imt=fileread(originpf)
for i in range(len(imt)):
       col=imt[i].split()
       if len(col)>1 and col[0]=='Maxtime':
              maxtime=col[1]
       if len(col)>1 and col[0]=='Product':
```

```
                prod=col[1]
code_prod=int(system_call(wrkdir+'/CATscripts/code_prod.sh
'+wrkdir+'/catalysis_res/KMC/RXNet_catalysis_with_barrierless '+ prod))
inputfile=wrkdir+'/catalysis_res/KMC/kmc_template.dat'
dirkmcs=wrkdir+'/catalysis_res/KMC/kmc_files'
icon=fileread(inputfile)
for i in range(len(icon)):
        col=icon[i].split()
        if i==2:
                vol=col[0]
        if 'C0' in col[0]:
                j+=1
                dc0_max[j]=float(col[2])
                if (len(col))==4:
                        sol=float(col[3])
                        dc0_max[j]=dc0_max[j]*sol
c0_max=dc0_max.values()
cmd='cp '+wrkdir+'/CATscripts/param .'
os.system(cmd)
for ie in range(len(c0_max)):
        rate_ie=open(wrkdir+'/catalysis_res/KMC/kmc_files/rate_'+str(ie),'w')
        rate_ie_stat=open(wrkdir+'/catalysis_res/KMC/kmc_files/rate_'+str(ie)+'_stat','w')
        for i in range(nsteps):
                delta=float(c0_max[ie]/nsteps)
                c0_i=delta*(i+1)
                file=dirkmcs+'/kmc_'+str(ie)+'_'+str(i)+'.out'
                rate={}
                if os.path.isfile(file):
                        for j in range(1,4):
                                cmd=wrkdir+'/CATscripts/get_rate.sh '+file+' '+str(j)+' '+str(maxtime)+'
'+str(code_prod)
                                os.system(cmd)
                                if not os.path.isfile('k'):
                                        print "Calc. ",j," of molecule ",ie," step ",i," has not finished yet"
                                else:
                                        cmd='gnuplot '+wrkdir+'/CATscripts/fit.gp 2>error.log'
                                        os.system(cmd)
                                        rate[j]=float(system_call(wrkdir+'/CATscripts/get_rate2.sh '+vol))
                                        cmd='rm error.log fit.log'
                                        os.system(cmd)
                        media=np.mean(rate.values())
                        std=np.std(rate.values())
                        print i,c0_i,media,std
                        rate_ie.write(str(c0_i)+' '+str(media)+' '+str(std)+'\n' )
                        rate_ie_stat.write(str(rate.values())+'\n' )
                else:
                        print "File ",file," does not exist"
        rate_ie.close()
        rate_ie_stat.close()
cmd='rm param'
os.system(cmd)
```

gui.py: This python code creates the GUI.

```
from Tkinter import *
import sys
import os

#funciones de procesamiento
def myfunction(event):
    canvas.configure(scrollregion=canvas.bbox("all"),width=70,height=90)

def myfunction2(event):
    canvas2.configure(scrollregion=canvas2.bbox("all"),width=70,height=90)

def fileread(name):
        with open(name) as f:
                line=f.read().splitlines()
        return line

###########3
# load the listbox with data
def load_item():
# Get the number of atoms of the molecule
        listbox1.delete(0,END)
        if job.get()==1:
                name=molecule.get()
```

```
                xyz=fileread(name+'.xyz')
                natom.set(int(xyz[0]))
        if sampling.get()==1:
                for item in range(1,natom.get()+1):
                        listbox1.insert(END, item)

def load_item2():
# Get the number of atoms of the molecule
        listbox2.delete(0,END)
        if job.get()==1:
                name=molecule.get()
                xyz=fileread(name+'.xyz')
                nmode.set(3*int((xyz[0]))-6)
        if sampling.get()==2:
                for item in range(1,nmode.get()+1):
                        listbox2.insert(END, item)

def delete_item():
        try:
                index=listbox1.curselection()[0]
                listbox1.delete(index)
        except IndexError:
                pass

def delete_item2():
        try:
                index=listbox2.curselection()[0]
                listbox2.delete(index)
        except IndexError:
                pass
#######33k
def proc1():
        if sampling.get()==1:
                name=molecule.get()
                samp="canonical"
        elif sampling.get()==2:
                name=molecule.get()
                samp="microcanonical"
        else:
                name=fr1.get()+'_'+fr2.get()
                samp="association"
        fname=name+'_'+samp+'.dat'
        inpf=open(fname,'w')
        inpf.write('--General section--\n')
        if job.get()==1:
                inpf.write('molecule '+str(name)+'\n')
                inpf.write('charge '+str(charge.get())+'\n')
                inpf.write('mult '+str(mult.get())+'\n')
        else:
                inpf.write('molecule '+str(name)+'\n')
                inpf.write('charge '+str(chcat.get())+'\n')
                inpf.write('mult '+str(mucat.get())+'\n')
        inpf.write('LLcalc '+str(llcalc.get())+'\n')
         sp2corr=spcorr.get()
        if (sp2corr=="CCSD(T)"):
                sp2corr="CCSDT"
        if (sp2corr=="NO"):
                inpf.write('HLcalc 1'+'\t'+str(hlcalc1.get())+'\n')
                inpf.write('level1'+'\t'+str(level1.get())+'\n')
                inpf.write('basis1'+'\t'+str(basis1.get())+'\n')
        else:
                inpf.write('HLcalc 2'+'\t'+str(hlcalc1.get())+'\t'+str(sp2corr)+'\n')
                inpf.write('level1'+'\t'+str(level1.get())+'\n')
                inpf.write('basis1'+'\t'+str(basis1.get())+'\n')
                inpf.write('level2'+'\t'+str(level2.get())+'\n')
                inpf.write('basis2'+'\t'+str(basis2.get())+'\n')

        if job.get()==2:
                inpf.write('\n--Catalysis section--\n')
                inpf.write('species 
'+str(species1.get())+'\t'+str(species2.get())+'\t'+str(species3.get())+'\t'+str(species4.get())+'\n')
                inpf.write('eta '+str(eta.get())+'\n')

        inpf.write('\n--CDS section--\n')
        if sampling.get()<=2:
                if job.get()==1:
                        xyz=fileread(name+'.xyz')
```

```
			natom.set(int(xyz[0]))
		nmode.set(3*natom.get()-6)
	if sampling.get() ==1:
		inpf.write('sampling canonical\n')
		inpf.write('temp'+'\t'+str(tdyn.get())+'\n')
###
		temp_list = list(listbox1.get(0, END))
		temp_list = [str(item) for item in temp_list]
		inpf.write('atoms ')
		if natom.get()==len(temp_list) or len(temp_list)==0:
			inpf.write('all\n')
		else:
			inpf.write(str(len(temp_list))+' ')
			for i in range(len(temp_list)-1):
				inpf.write(temp_list[i]+',')
			inpf.write(temp_list[len(temp_list)-1]+'\n')
###
	if sampling.get() ==2:
		inpf.write('sampling microcanonical\n')
		inpf.write('etraj'+'\t'+str(edyn.get())+'\n')
###
		temp_list = list(listbox2.get(0, END))
		temp_list = [str(item) for item in temp_list]
		inpf.write('modes ')
		if nmode.get()==len(temp_list) or len(temp_list)==0:
			inpf.write('all\n')
		else:
			inpf.write(str(len(temp_list))+' ')
			for i in range(len(temp_list)-1):
				inpf.write(temp_list[i]+',')
			inpf.write(temp_list[len(temp_list)-1]+'\n')
	if sampling.get() ==3:
		inpf.write('sampling association\n')
		inpf.write('A= '+str(fr1.get())+'\n')
		inpf.write('B= '+str(fr2.get())+'\n')
		inpf.write('rotate
'+str(pivot1.get())+'\t'+str(pivot2.get())+'\t'+str(dbp.get())+'\t'+str(mind.get())+'\n')

	if sampling.get()<=2:
		inpf.write('ntraj '+str(ntraj.get())+'\n')
		inpf.write('fs '+str(time.get())+'\n')
		if seed.get() != "random":
			inpf.write('seed '+str(seed.get())+'\n')
		if sampling.get()==1 and thmass.get() != 0:
			inpf.write('thmass '+str(thmass.get())+'\n')
		inpf.write('\n--BBFS section--\n')
		inpf.write('emaxts '+str(emaxts.get())+'\n')
		inpf.write('emints '+str(emints.get())+'\n')
		inpf.write('freqmin '+str(freqmin.get())+'\n')
		if fastmode.get()==1:
			inpf.write('fastmode\n')
		if refdist.get()==1:
			inpf.write('NOcreatethdist\n')
			cmd='createthdist0.sh '+str(name)+'\t'+str(job.get())
			os.system(cmd)
		tsdirll=str(os.getcwd())+'/tsdirLL_'+name
		tsdirhl=str(os.getcwd())+'/tsdirHL_'+name
		inpf.write('tsdirll '+str(tsdirll)+'\n')
		inpf.write('tsdirhl '+str(tsdirhl)+'\n')
	inpf.write('\n--Screening of the structures section--\n')
	inpf.write('avgerr '+str(avgerr.get())+'\n')
	inpf.write('bigerr '+str(bigerr.get())+'\n')
	inpf.write('thdiss '+str(thdiss.get())+'\n')
	if nvdw.get()>0:
		inpf.write('vdw '+str(nvdw.get())+'\n')
		inpf.write(str(atp1.get())+'\t'+str(atpd1.get())+'\n')
		if nvdw.get()==2:
			inpf.write(str(atp2.get())+'\t'+str(atpd2.get())+'\n')
		if nvdw.get()==3:
			inpf.write(str(atp2.get())+'\t'+str(atpd2.get())+'\n')
			inpf.write(str(atp3.get())+'\t'+str(atpd3.get())+'\n')


	if sampling.get()<=2:
		inpf.write('\n--Kinetics section--\n')
		if samplingkmc.get()==1:
			inpf.write('Rate canonical\n')
```

```
                inpf.write('TKMC '+str(tkmc.get())+'\n')
        else:
                inpf.write('Rate microcanonical\n')
                inpf.write('EKMC '+str(ekmc.get())+'\n')
        inpf.write('MaxEn '+str(emaxkmc.get())+'\n')
        inpf.write('nmol '+str(nmol.get())+'\n')
        inpf.write('imin '+str(min0.get())+'\n')
        inpf.write('Maxtime '+str(timekmc.get())+'\n')
        inpf.write('Stepsize '+str(dt.get())+'\n')
        inpf.write('ImpPaths '+str(percentrel.get())+'\n')
        inpf.write('PathInfo All\n')

    inpf.close()

def proc22kmc():
    if samplingkmc.get()==1 and sampling.get()<=2:
        tkmc.set("298")
        ekmc.set("")
        nmol.set("1000")
        timekmc.set("1e12")
        dt.set("1e11")
        emaxkmc.set("40")
        percentrel.set("0.1")
    if samplingkmc.get()==2 and sampling.get()<=2:
        tkmc.set("")
        ekmc.set("150")
        nmol.set("1000")
        timekmc.set("10")
        dt.set("0.1")
        emaxkmc.set("200")
        percentrel.set("0.1")
    if sampling.get()==3:
        tkmc.set("")
        ekmc.set("")
        nmol.set("")
        timekmc.set("")
        dt.set("")
        emaxkmc.set("")
        percentrel.set("")


def proc22():
    if sampling.get()==1:
        tdyn.set("1e4")
        edyn.set("")
        fr1.set("")
        fr2.set("")
        pivot1.set("")
        pivot2.set("")
        dbp.set("")
        mind.set("")
        emaxts.set("200")
        emints.set("-200")
        freqmin.set("200")
        nmol.set("1000")
        ntraj.set("1")
        time.set("500")
        seed.set("random")
        thmass.set("0.0")
        percentrel.set("0.1")
    elif sampling.get()==2:
        tdyn.set("")
        edyn.set("400")
        emaxts.set("400")
        fr1.set("")
        fr2.set("")
        pivot1.set("")
        pivot2.set("")
        dbp.set("")
        mind.set("")
        freqmin.set("200")
        emints.set("-200")
        nmol.set("1000")
        ntraj.set("1")
        time.set("500")
        seed.set("random")
        thmass.set("0.0")
```

```
		percentrel.set("0.1")
	else:
		tdyn.set("")
		edyn.set("")
		fr1.set("name")
		fr2.set("name")
		pivot1.set("com")
		pivot2.set("com")
		dbp.set("2.0")
		mind.set("1.0")
		emaxts.set("")
		emints.set("")
		freqmin.set("")
		tkmc.set("")
		ekmc.set("")
		nmol.set("")
		timekmc.set("")
		dt.set("")
		emaxkmc.set("")
		ntraj.set("")
		time.set("")
		seed.set("")
		thmass.set("")
		percentrel.set("")


def proc2():
	if job.get()==1:
		molecule.set("name")
		min0.set("ask later")
		species1.set("")
		species2.set("")
		species3.set("")
		species4.set("")
		ss1.set("")
		ss2.set("")
		ss3.set("")
		ss4.set("")
		ss5.set("")
		ss6.set("")
		ss7.set("")
		ss8.set("")
		eta.set("")
		charge.set("0")
		charge1.set("")
		charge2.set("")
		charge3.set("")
		charge4.set("")
		mult.set("1")
		mult1.set("")
		mult2.set("")
		mult3.set("")
		mult4.set("")
	else:
		min0.set("min0")
		molecule.set("")
		 charge.set("")
		 charge1.set("0")
		 charge2.set("0")
		 charge3.set("0")
		 charge4.set("0")
		 mult.set("")
		mult1.set("1")
		mult2.set("1")
		mult3.set("1")
		mult4.set("1")
		species1.set("cat")
		eta.set("0.01")
def proc3():
	 if job.get()==1:
		return
	ss1.set("")
	ss2.set("")
	ss3.set("")
	ss4.set("")
	ss5.set("")
	ss6.set("")
```

```
	ss7.set("")
	ss8.set("")
	min_name={}
	nmolecule=0
	if not species1.get()=="":
		nmolecule+=1
		min_name[1]=species1.get()
	if not species2.get()=="":
		nmolecule+=1
		min_name[2]=species2.get()
	if not species3.get()=="":
		nmolecule+=1
		min_name[3]=species3.get()
	if not species4.get()=="":
		nmolecule+=1
		min_name[4]=species4.get()
	if nmolecule==1:
		return
	 subsystem=['cat']
	 pairs={}
	 i=1
	 p=0
	 for i in range(2,nmolecule+1):
		 p+=1
		 pairs[p]='cat_'+min_name[i]
	 subsystem=subsystem+pairs.values()
	ss1.set(subsystem[0])
	ss2.set(subsystem[1])
	 t=0
	if nmolecule>2:
		trios={}
		 for i in range(1,p+1):
			for j in range(i+2,nmolecule+1):
				t+=1
				trios[t]=pairs[i]+'_'+min_name[j]
		subsystem=subsystem+trios.values()
		ss3.set(subsystem[2])
		ss4.set(subsystem[3])
		if nmolecule==4:
			fours={}
			fours[1]=trios[1]+'_'+min_name[nmolecule]
			subsystem=subsystem+fours.values()
			ss5.set(subsystem[4])
			ss6.set(subsystem[5])
			ss7.set(subsystem[6])
			ss8.set(subsystem[7])
	i=0
	for txt in subsystem:
		i+=1

	Radiobutton(f02,text=txt,font=("Helvetica",11),indicatoron=0,variable=cs,value=i,width=25,command=proc4)
.grid(row=i,column=8,columnspan=2)

def proc4():
	if species2.get()=="":
		nos=1
	elif species3.get()=="":
		nos=2
	elif species4.get()=="":
		nos=3
	else:
		nos=4
	for i in range(1,9):
		if cs.get()==i:
			if i==1:
				ss.set(ss1.get())
				chcat.set(charge1.get())
				mucat.set(mult1.get())
				xyz1=fileread(species1.get()+'.xyz')
				natom1=(int(xyz1[0]))
				natom2=0
				natom.set(natom1)
				if sampling.get()==3:
					fr1.set(species1.get())
					fr2.set("")
			if i==2:
				ss.set(ss2.get())
```

```
        chcat.set(charge1.get()+charge2.get())
        mucat.set(mult1.get()+mult2.get()-1)
        xyz1=fileread(species1.get()+'.xyz')
        xyz2=fileread(species2.get()+'.xyz')
        natom1=(int(xyz1[0]))
        natom2=(int(xyz2[0]))
        natom.set(natom1+natom2)
        if sampling.get()==3:
                fr1.set(species1.get())
                fr2.set(species2.get())
if i==3:
        ss.set(ss3.get())
        chcat.set(charge1.get()+charge3.get())
        mucat.set(mult1.get()+mult3.get()-1)
        xyz1=fileread(species1.get()+'.xyz')
        xyz2=fileread(species3.get()+'.xyz')
        natom1=(int(xyz1[0]))
        natom2=(int(xyz2[0]))
        natom.set(natom1+natom2)
        if sampling.get()==3:
                fr1.set(species1.get())
                fr2.set(species3.get())
if i==4:
        ss.set(ss4.get())
        if nos==3:
                chcat.set(charge1.get()+charge2.get()+charge3.get())
                mucat.set(mult1.get()+mult2.get()+mult3.get()-2)
                xyz1=fileread(species1.get()+'.xyz')
                xyz2=fileread(species2.get()+'.xyz')
                xyz3=fileread(species3.get()+'.xyz')
                natom1=(int(xyz1[0]))
                natom2=(int(xyz2[0]))
                natom3=(int(xyz3[0]))
                natom.set(natom1+natom2+natom3)
                if sampling.get()==3:
                        fr1.set(species1.get()+'_'+species2.get())
                        fr2.set(species3.get())
        elif nos==4:
                chcat.set(charge1.get()+charge4.get())
                mucat.set(mult1.get()+mult4.get()-1)
                xyz1=fileread(species1.get()+'.xyz')
                xyz2=fileread(species4.get()+'.xyz')
                natom1=(int(xyz1[0]))
                natom2=(int(xyz2[0]))
                natom.set(natom1+natom2)
                if sampling.get()==3:
                        fr1.set(species1.get())
                        fr2.set(species4.get())
if i==5:
        ss.set(ss5.get())
        chcat.set(charge1.get()+charge2.get()+charge3.get())
        mucat.set(mult1.get()+mult2.get()+mult3.get()-2)
        xyz1=fileread(species1.get()+'.xyz')
        xyz2=fileread(species2.get()+'.xyz')
        xyz3=fileread(species3.get()+'.xyz')
        natom1=(int(xyz1[0]))
        natom2=(int(xyz2[0]))
        natom3=(int(xyz3[0]))
        natom.set(natom1+natom2+natom3)
        if sampling.get()==3:
                fr1.set(species1.get()+'_'+species2.get())
                fr2.set(species3.get())
if i==6:
        ss.set(ss6.get())
        chcat.set(charge1.get()+charge2.get()+charge4.get())
        mucat.set(mult1.get()+mult2.get()+mult4.get()-2)
        xyz1=fileread(species1.get()+'.xyz')
        xyz2=fileread(species2.get()+'.xyz')
        xyz3=fileread(species4.get()+'.xyz')
        natom1=(int(xyz1[0]))
        natom2=(int(xyz2[0]))
        natom3=(int(xyz3[0]))
        natom.set(natom1+natom2+natom3)
        if sampling.get()==3:
                fr1.set(species1.get()+'_'+species2.get())
                fr2.set(species4.get())
if i==7:
```

```
                    ss.set(ss7.get())
                    chcat.set(charge1.get()+charge3.get()+charge4.get())
                    mucat.set(mult1.get()+mult3.get()+mult4.get()-2)
                    xyz1=fileread(species1.get()+'.xyz')
                    xyz2=fileread(species3.get()+'.xyz')
                    xyz3=fileread(species4.get()+'.xyz')
                    natom1=(int(xyz1[0]))
                    natom2=(int(xyz2[0]))
                    natom3=(int(xyz3[0]))
                    natom.set(natom1+natom2+natom3)
                    if sampling.get()==3:
                        fr1.set(species1.get()+'_'+species3.get())
                        fr2.set(species4.get())
                if i==8:
                    ss.set(ss8.get())
                    chcat.set(charge1.get()+charge2.get()+charge3.get()+charge4.get())
                    mucat.set(mult1.get()+mult2.get()+mult3.get()+mult4.get()-3)
                    xyz1=fileread(species1.get()+'.xyz')
                    xyz2=fileread(species2.get()+'.xyz')
                    xyz3=fileread(species3.get()+'.xyz')
                    xyz4=fileread(species4.get()+'.xyz')
                    natom1=(int(xyz1[0]))
                    natom2=(int(xyz2[0]))
                    natom3=(int(xyz3[0]))
                    natom4=(int(xyz4[0]))
                    natom.set(natom1+natom2+natom3+natom4)
                    if sampling.get()==3:
                        fr1.set(species1.get()+'_'+species2.get()+'_'+species3.get())
                        fr2.set(species4.get())
        molecule.set(ss.get())
        charge.set(chcat.get())
        mult.set(mucat.get())
         nmode.set(3*natom.get()-6)


def proc5():
        ventana.destroy()


#Instancia de la clase Tk
ventana = Tk()
ventana.title('TSSCDS Graphical User Interface')

f0=Frame(ventana,width=1100,height=240,relief=RIDGE,bd=3)
f0.grid(row=0,column=0,columnspan=10,rowspan=7)
f0.grid_propagate(False)
f01=Frame(f0,width=270,height=230,relief=RIDGE,bd=3)
f01.grid(row=0,column=0,columnspan=2,rowspan=7)
f01.grid_propagate(False)
f02=Frame(f0,width=830,height=230,relief=RIDGE,bd=3)
f02.grid(row=0,column=2,columnspan=8,rowspan=7)
f02.grid_propagate(False)

f1=Frame(ventana,width=465,height=125,relief=RIDGE,bd=3)
f1.grid(row=10,column=0,columnspan=4,rowspan=3)
f1.grid_propagate(False)
f2=Frame(ventana,width=635,height=125,relief=RIDGE,bd=3)
f2.grid(row=10,column=5,columnspan=6,rowspan=5)
f2.grid_propagate(False)

f3=Frame(ventana,width=1100,height=200,relief=RIDGE,bd=3)
f3.grid(row=15,column=0,columnspan=10,rowspan=8)
f3.grid_propagate(False)
f31=Frame(f3,width=635,height=160,relief=RIDGE,bd=3)
f31.grid(row=16,column=0,columnspan=5,rowspan=4)
f31.grid_propagate(False)
f311=Frame(f31,width=210,height=150,relief=RIDGE,bd=3)
f311.grid(row=16,column=0,columnspan=2,rowspan=5)
f311.grid_propagate(False)
fcanvas=Frame(f311,width=50,height=50,relief=RIDGE,bd=3)
fcanvas.grid(row=18,column=0,columnspan=1,rowspan=3)
fcanvas.grid_propagate(False)
f312=Frame(f31,width=210,height=150,relief=RIDGE,bd=3)
f312.grid(row=16,column=2,columnspan=2,rowspan=5)
f312.grid_propagate(False)
fcanvas2=Frame(f312,width=50,height=50,relief=RIDGE,bd=3)
fcanvas2.grid(row=18,column=2,columnspan=1,rowspan=3)
```

```
fcanvas2.grid_propagate(False)
f313=Frame(f31,width=210,height=150,relief=RIDGE,bd=3)
f313.grid(row=16,column=4,columnspan=2,rowspan=4)
f313.grid_propagate(False)

f32=Frame(f3,width=465,height=160,relief=RIDGE,bd=3)
f32.grid(row=16,column=6,columnspan=4,rowspan=4,sticky=N)
f32.grid_propagate(False)

f4=Frame(ventana,width=1100,height=80,relief=RIDGE,bd=3)
f4.grid(row=33,column=0,columnspan=10,rowspan=3)
f4.grid_propagate(False)

f5=Frame(ventana,width=1100,height=40,relief=RIDGE,bd=3)
f5.grid(row=36,column=0,columnspan=10,rowspan=1)
f5.grid_propagate(False)

#f5=Frame(f0,width=500,height=500,relief=SUNKEN,bd=2)
#f5.grid(row=18,column=0,columnspan=4,rowspan=2)

#Variables que almacenarn los datos
species1 = StringVar()
species2 = StringVar()
species3 = StringVar()
species4 = StringVar()
nvdw=IntVar()
min0=StringVar()
fastmode=IntVar()
refdist=IntVar()
atp1=StringVar()
atpd1=DoubleVar()
atp2=StringVar()
atpd2=DoubleVar()
atp3=StringVar()
atpd3=DoubleVar()
charge = IntVar()
charge1 = IntVar()
charge2 = IntVar()
charge3 = IntVar()
charge4 = IntVar()
natom=IntVar()
nmode=IntVar()
mult = IntVar()
mult1 = IntVar()
mult2 = IntVar()
mult3 = IntVar()
mult4 = IntVar()
llcalc = StringVar()
nhlcalc = IntVar()
spcorr=StringVar()
hlcalc1 = StringVar()
title1 = StringVar()
title2 = StringVar()
title3 = StringVar()
title4 = StringVar()
level1 = StringVar()
basis1 = StringVar()
level2 = StringVar()
basis2 = StringVar()
eta = DoubleVar()
tdyn = DoubleVar()
tkmc = DoubleVar()
ekmc = DoubleVar()
edyn = DoubleVar()
fr1 = StringVar()
fr2 = StringVar()
pivot1 = StringVar()
pivot2 = StringVar()
dbp = DoubleVar()
mind = DoubleVar()
sampling = IntVar()
samplingkmc = IntVar()
ntraj = IntVar()
seed = StringVar()
thmass=DoubleVar()
emaxts = DoubleVar()
emaxkmc = DoubleVar()
```

```
percentrel=DoubleVar()
avgerr=DoubleVar()
bigerr=DoubleVar()
thdiss=DoubleVar()
emints = DoubleVar()
freqmin = DoubleVar()
time = IntVar()
molecule=StringVar()
ss1=StringVar()
ss2=StringVar()
ss3=StringVar()
ss4=StringVar()
ss5=StringVar()
ss6=StringVar()
ss7=StringVar()
ss8=StringVar()
ss=StringVar()
chcat=IntVar()
mucat=IntVar()
job=IntVar()
cs=IntVar()
nmol=IntVar()
timekmc=DoubleVar()
dt=DoubleVar()
#genero.set(1)
timekmc.set("1e12")
dt.set("1e11")
nmol.set("1000")
eta.set("")
tkmc.set("298")
ekmc.set("")
tdyn.set("1e4")
edyn.set("")
percentrel.set("0.1")
charge.set("0")
atp1.set("Co H")
atpd1.set("2.0")
atpd2.set("")
atpd3.set("")
charge1.set("")
charge2.set("")
charge3.set("")
charge4.set("")
min0.set("ask later")
fr1.set("")
fr2.set("")
dbp.set("")
mind.set("")
nvdw.set("1")
pivot1.set("")
pivot2.set("")
llcalc.set("PM7")
hlcalc1.set("DFT")
level1.set("B3LYP")
basis1.set("6-31G*")
mult.set("1")
mult1.set("")
mult2.set("")
mult3.set("")
mult4.set("")
spcorr.set("NO")
seed.set("random")
thmass.set("0.0")
emaxts.set("200")
emaxkmc.set("40")
avgerr.set("1e-4")
bigerr.set("5")
thdiss.set("0.1")
emints.set("-200")
freqmin.set("200")
time.set("500")
job.set("1")
molecule.set("name")
sampling.set("1")
samplingkmc.set("1")
ntraj.set("1")
```

```
#title Label
#Label(f0, text='Job Type',font=("Helvetica",14),height=1,anchor=S).grid(row=0, column=0,columnspan=10)


#Radiobutton
Radiobutton(f01,variable=job,text="Single
system",font=("Helvetica",11),value=1,command=proc2).grid(row=0,column=0,columnspan=2)
Radiobutton(f02,variable=job,text="Catalysis",font=("Helvetica",11),value=2,command=proc2).grid(row=0,column=2,c
olumnspan=7)
#Button
Button(f02, text='Get subsystems',font=("Helvetica",11),command=proc3).grid(row=0, column=8,columnspan=2)

#title
Label(f01, text='Molecule: ',font=("Helvetica",11),width=12).grid(row=1, column=0,sticky=E)
Entry(f01, textvariable=molecule,font=("Helvetica",11),width=12,bg="yellow").grid(row=1, column=1,sticky=W)

Label(f01, text='Charge: ',font=("Helvetica",11),width=12).grid(row=2, column=0,sticky=E)
Entry(f01, textvariable=charge,font=("Helvetica",11),width=12).grid(row=2, column=1,sticky=W)
Label(f01, text='Multiplicity: ',font=("Helvetica",11),width=12).grid(row=3, column=0,sticky=E)
Entry(f01, textvariable=mult,font=("Helvetica",11),width=12).grid(row=3, column=1,sticky=W)

#title

#species
Label(f02, text='Molecule 1: ',font=("Helvetica",11),width=12).grid(row=1, column=2,sticky=E)
Label(f02, text='Molecule 2: ',font=("Helvetica",11),width=12).grid(row=2, column=2,sticky=E)
Label(f02, text='Molecule 3: ',font=("Helvetica",11),width=12).grid(row=3, column=2,sticky=E)
Label(f02, text='Molecule 4: ',font=("Helvetica",11),width=12).grid(row=4, column=2,sticky=E)
Entry(f02, textvariable=species1,font=("Helvetica",11),width=12).grid(row=1, column=3,sticky=W)
Entry(f02, textvariable=species2,font=("Helvetica",11),width=12).grid(row=2, column=3,sticky=W)
Entry(f02, textvariable=species3,font=("Helvetica",11),width=12).grid(row=3, column=3,sticky=W)
Entry(f02, textvariable=species4,font=("Helvetica",11),width=12).grid(row=4, column=3,sticky=W)
Label(f02, text='Charge: ',font=("Helvetica",11),      width=8).grid(row=1, column=4,sticky=E)
Entry(f02, textvariable=charge1,font=("Helvetica",11), width= 2).grid(row=1, column=5,sticky=W)
Label(f02, text='Multiplicity: ',font=("Helvetica",11),width=10).grid(row=1, column=6,sticky=E)
Entry(f02, textvariable=mult1,font=("Helvetica",11),   width= 2).grid(row=1, column=7,sticky=W)
Label(f02, text='Charge: ',font=("Helvetica",11),      width=8).grid(row=2, column=4,sticky=E)
Entry(f02, textvariable=charge2,font=("Helvetica",11), width= 2).grid(row=2, column=5,sticky=W)
Label(f02, text='Multiplicity: ',font=("Helvetica",11),width=10).grid(row=2, column=6,sticky=E)
Entry(f02, textvariable=mult2,font=("Helvetica",11),   width= 2).grid(row=2, column=7,sticky=W)
Label(f02, text='Charge: ',font=("Helvetica",11),      width=8).grid(row=3, column=4,sticky=E)
Entry(f02, textvariable=charge3,font=("Helvetica",11), width= 2).grid(row=3, column=5,sticky=W)
Label(f02, text='Multiplicity: ',font=("Helvetica",11),width=10).grid(row=3, column=6,sticky=E)
Entry(f02, textvariable=mult3,font=("Helvetica",11),   width= 2).grid(row=3, column=7,sticky=W)
Label(f02, text='Charge: ',font=("Helvetica",11),      width=8).grid(row=4, column=4,sticky=E)
Entry(f02, textvariable=charge4,font=("Helvetica",11), width= 2).grid(row=4, column=5,sticky=W)
Label(f02, text='Multiplicity: ',font=("Helvetica",11),width=10).grid(row=4, column=6,sticky=E)
Entry(f02, textvariable=mult4,font=("Helvetica",11),   width= 2).grid(row=4, column=7,sticky=W)
Label(f02, text=u'\u03B7 (Pas): ',font=("Helvetica",11),width=12).grid(row=5, column=2,sticky=E)
Entry(f02, textvariable=eta,font=("Helvetica",11),width=12).grid(row=5, column=3,sticky=W)

#title Label
Label(f1, text='MOPAC calc: ',font=("Helvetica",11),width=12).grid(row=10, column=1,sticky=E)
OptionMenu(f1, llcalc, "AM1","PM3","PM6","PM7").grid(row=10,column=2,sticky=W)
Label(f1, text='G09 calc: ',font=("Helvetica",11),width=12).grid(row=11, column=0,sticky=E)
OptionMenu(f1, hlcalc1, "HF","MP2","DFT").grid(row=11, column=1,sticky=W)
Entry(f1, textvariable=level1,font=("Helvetica",11),width=12).grid(row=12, column=0,sticky=W)
Entry(f1, textvariable=basis1,font=("Helvetica",11),width=12).grid(row=12, column=1,sticky=W)
Label(f1, text='SP correction?',font=("Helvetica",11),width=12).grid(row=11, column=2)
OptionMenu(f1, spcorr, "NO","HF","MP2","DFT","CCSD(T)").grid(row=11, column=3)
Entry(f1, textvariable=level2,font=("Helvetica",11),width=12).grid(row=12, column=2,sticky=W)
Entry(f1, textvariable=basis2, font=("Helvetica",11),width=12).grid(row=12, column=3,sticky=W)

#title Label
Label(f3, text='Sampling method',font=("Helvetica",11),height=0,anchor=S).grid(row=15, column=0,columnspan=10)

#sampling OptionMenu
Radiobutton(f311,variable=sampling,text="Canonical",font=("Helvetica",11),value=1,command=proc22).grid(row=16,co
lumn=0,columnspan=2)
Radiobutton(f312,variable=sampling,text="Microcanonical",font=("Helvetica",11),value=2,command=proc22).grid(row=
16,column=2,columnspan=2)
Radiobutton(f32,variable=sampling,text="Association",font=("Helvetica",11),value=3,command=proc22).grid(row=16,c
olumn=6,columnspan=4)

Label(f311, text='Temp (K):',font=("Helvetica",11),width=8).grid(row=17, column=0,sticky=E)
Entry(f311, textvariable=tdyn,font=("Helvetica",11),width=8).grid(row=17, column=1,sticky=W)
```

```
##############33333333
# create the listbox (note that size is in characters)
canvas=Canvas(fcanvas)
canvas2=Canvas(fcanvas2)
frame=Frame(canvas)
frame2=Frame(canvas2)
listbox1 = Listbox(frame, width=50, height=natom.get(),selectmode='multiple')
listbox1.grid(row=18, column=0,columnspan=1,rowspan=3)

myscrollbar = Scrollbar(fcanvas,command=canvas.yview, orient=VERTICAL)
canvas.configure(yscrollcommand=myscrollbar.set)
myscrollbar.pack(side="right",fill="y")
canvas.pack(side="right")
canvas.create_window((0,0),window=frame,anchor='nw')
frame.bind("<Configure>",myfunction)
Label(f311, text='Excite subset:',font=("Helvetica",11),width=12).grid(row=18, column=1)
button1 = Button(f311, text='Load atoms', command=load_item)
button1.grid(row=19, column=1,sticky=W)
button2 = Button(f311, text='Delete frozen', command=delete_item)
button2.grid(row=20, column=1,sticky=W)
################33
listbox2 = Listbox(frame2, width=50, height=3*natom.get()-6,selectmode='multiple')
listbox2.grid(row=18, column=2,sticky=E)

myscrollbar2 = Scrollbar(fcanvas2,command=canvas2.yview, orient=VERTICAL)
canvas2.configure(yscrollcommand=myscrollbar2.set)
myscrollbar2.pack(side="right",fill="y")
canvas2.pack(side="right")
canvas2.create_window((0,0),window=frame2,anchor='nw')
frame2.bind("<Configure>",myfunction2)

Label(f312, text='Excite subset:',font=("Helvetica",11),width=12).grid(row=18, column=3)
button1 = Button(f312, text='Load modes', command=load_item2)
button1.grid(row=19, column=3,sticky=W)
button2 = Button(f312, text='Delete frozen', command=delete_item2)
button2.grid(row=20, column=3,sticky=W)
################33


###############
Label(f312, text='E (kcal): ',font=("Helvetica",11),width=8).grid(row=17, column=2,sticky=E)
Entry(f312, textvariable=edyn,font=("Helvetica",11),width=8).grid(row=17, column=3,sticky=W)
Label(f32, text='Fragment A: ',font=("Helvetica",11),width=16).grid(row=17, column=6,sticky=E)
Entry(f32, textvariable=fr1,font=("Helvetica",11),width=6).grid(row=17, column=7,sticky=W)
Label(f32, text='Fragment B: ',font=("Helvetica",11),width=16).grid(row=17, column=8,sticky=E)
Entry(f32, textvariable=fr2,font=("Helvetica",11),width=6).grid(row=17, column=9,sticky=W)
Label(f32, text='Pivot of A: ',font=("Helvetica",11),width=16).grid(row=18, column=6,sticky=E)
Entry(f32, textvariable=pivot1,font=("Helvetica",11),width=6).grid(row=18, column=7,sticky=W)
Label(f32, text='Pivot of B: ',font=("Helvetica",11),width=16).grid(row=18, column=8,sticky=E)
Entry(f32, textvariable=pivot2,font=("Helvetica",11),width=6).grid(row=18, column=9,sticky=W)
Label(f32, text=u"R betw pivots (\u212B):",font=("Helvetica",11),width=16).grid(row=19, column=6,sticky=E)
Entry(f32, textvariable=dbp,font=("Helvetica",11),width=6).grid(row=19, column=7,sticky=W)


Label(f32, text=u'R\u2098\u1d62\u2099 betw frags (\u212B):',font=("Helvetica",11),width=15).grid(row=19,
column=8,sticky=E)
Entry(f32, textvariable=mind,font=("Helvetica",11),width=6).grid(row=19, column=9,sticky=W)

#spcorr Entry
Label(f313, text='Simulation details:',font=("Helvetica",11),width=20).grid(row=18,
column=4,sticky=E,columnspan=2)
Label(f313, text='Trajectories:',font=("Helvetica",11),width=12).grid(row=19, column=4,sticky=E)
Entry(f313, textvariable=ntraj,font=("Helvetica",11),width=8).grid(row=19, column=5,sticky=W)
Label(f313, text='Time (fs):',font=("Helvetica",11),width=12).grid(row=20, column=4,sticky=E)
Entry(f313, textvariable=time,font=("Helvetica",11),width=8).grid(row=20, column=5,sticky=W)
Label(f313, text='Seed:',font=("Helvetica",11),width=12).grid(row=21, column=4,sticky=E)
Entry(f313, textvariable=seed,font=("Helvetica",11),width=8).grid(row=21, column=5,sticky=W)
Label(f313, text='Mass_exc.(au):',font=("Helvetica",11),width=12).grid(row=22, column=4,sticky=E)
Entry(f313, textvariable=thmass,font=("Helvetica",11),width=8).grid(row=22, column=5,sticky=W)

#title Label
#EMax and Emints and freqmin
Label(f2, text=u'E\u2098\u2090\u2093_TS (kcal):',font=("Helvetica",11),width=15).grid(row=10, column=5,sticky=E)
Entry(f2, textvariable=emaxts,font=("Helvetica",11),width=7).grid(row=10, column=6,sticky=W)
Label(f2, text=u'E\u2098\u1d62\u2099_TS (kcal):',font=("Helvetica",11),width=15).grid(row=10, column=7,sticky=E)
Entry(f2, textvariable=emints,font=("Helvetica",11),width=7).grid(row=10, column=8,sticky=W)
```

```
Label(f2, text=u'\u03bd\u2098\u1d62\u2099_TS (cm\u207B\u00B9):',font=("Helvetica",11),width=15).grid(row=10,
column=9,sticky=E)
Entry(f2, textvariable=freqmin,font=("Helvetica",11),width=7).grid(row=10, column=10,sticky=W)


Checkbutton(f2,variable=fastmode,text="Fast
calculation",font=("Helvetica",11)).grid(row=12,column=5,columnspan=2)
Checkbutton(f2,variable=refdist,text="Modify reference
distances",font=("Helvetica",11)).grid(row=13,column=5,columnspan=2)


Label(f2, text='RMSE:',font=("Helvetica",11),width=15).grid(row=11, column=5,sticky=E)
Entry(f2, textvariable=avgerr,font=("Helvetica",11),width=7).grid(row=11, column=6,sticky=W)
Label(f2, text='Number add_dist:',font=("Helvetica",11),width=15).grid(row=12, column=7,sticky=E)
Label(f2, text=u'If the above number>=1',font=("Helvetica",11),width=22).grid(row=13,
column=7,columnspan=2,sticky=E)
Label(f2, text=u'Atom pairs and dists. (\u212B)',font=("Helvetica",11),width=22).grid(row=14,
column=7,columnspan=2,sticky=E)
Entry(f2, textvariable=nvdw,font=("Helvetica",11),width=7,bg="yellow").grid(row=12, column=8,sticky=W)

Entry(f2, textvariable=atp1,font=("Helvetica",11),width=7,bg="yellow").grid(row=12, column=9,sticky=E)
Entry(f2, textvariable=atpd1,font=("Helvetica",11),width=7,bg="yellow").grid(row=12, column=10,sticky=W)
Entry(f2, textvariable=atp2,font=("Helvetica",11),width=7).grid(row=13, column=9,sticky=E)
Entry(f2, textvariable=atpd2,font=("Helvetica",11),width=7).grid(row=13, column=10,sticky=W)
Entry(f2, textvariable=atp3,font=("Helvetica",11),width=7).grid(row=14, column=9,sticky=E)
Entry(f2, textvariable=atpd3,font=("Helvetica",11),width=7).grid(row=14, column=10,sticky=W)

Label(f2, text='Largest error:',font=("Helvetica",11),width=15).grid(row=11, column=7,sticky=E)
Entry(f2, textvariable=bigerr,font=("Helvetica",11),width=7).grid(row=11, column=8,sticky=W)
Label(f2, text='MaxEig Laplacian:',font=("Helvetica",11),width=15).grid(row=11, column=9,sticky=E)
Entry(f2, textvariable=thdiss,font=("Helvetica",11),width=7).grid(row=11, column=10,sticky=W)

#title Label
Label(f4, text='Kinetic simulations',font=("Helvetica",11),height=1,anchor=S).grid(row=35,
column=0,columnspan=10)
Radiobutton(f4,variable=samplingkmc,text="Canonical",font=("Helvetica",11),value=1,command=proc22kmc).grid(row=3
6,column=0,columnspan=2)
Radiobutton(f4,variable=samplingkmc,text="Microcanonical",font=("Helvetica",11),value=2,command=proc22kmc).grid(
row=36,column=2,columnspan=2)
Label(f4, text='Temp (K):',font=("Helvetica",11),width=12).grid(row=37, column=0,sticky=E)
Entry(f4, textvariable=tkmc,font=("Helvetica",11),width=8).grid(row=37, column=1,sticky=W)
Label(f4, text='E (kcal):',font=("Helvetica",11),width=12).grid(row=37, column=2,sticky=E)
Entry(f4, textvariable=ekmc,font=("Helvetica",11),width=8).grid(row=37, column=3,sticky=W)

Label(f4, text='Starting Min:',font=("Helvetica",11),width=20).grid(row=36, column=4,sticky=E)
Entry(f4, textvariable=min0,font=("Helvetica",11),width=8).grid(row=36, column=5,sticky=W)

Label(f4, text='Number of molecules:',font=("Helvetica",11),width=20).grid(row=37, column=4,sticky=E)
Entry(f4, textvariable=nmol,font=("Helvetica",11),width=8).grid(row=37, column=5,sticky=W)
Label(f4, text='Time (s/ps):',font=("Helvetica",11),width=12).grid(row=36, column=6,sticky=E)
Entry(f4, textvariable=timekmc,font=("Helvetica",11),width=8).grid(row=36, column=7,sticky=W)
Label(f4, text=u'\u0394t (s/ps):',font=("Helvetica",11),width=12).grid(row=37, column=6,sticky=E)
Entry(f4, textvariable=dt,font=("Helvetica",11),width=8).grid(row=37, column=7,sticky=W)
Label(f4, text='MaxEn (kcal):',font=("Helvetica",11),width=18).grid(row=36, column=8,sticky=E)
Entry(f4, textvariable=emaxkmc,font=("Helvetica",11),width=8).grid(row=36, column=9,sticky=W)
Label(f4, text='Relevant Paths (%):',font=("Helvetica",11),width=18).grid(row=37, column=8,sticky=E)
Entry(f4, textvariable=percentrel,font=("Helvetica",11),width=8).grid(row=37, column=9,sticky=W)

#Label(ventana, text=u'\u0394t (s/ps):',font=("Helvetica",11),width=12).grid(row=35, column=8,sticky=E)
#Entry(ventana, textvariable=emaxkmc,font=("Helvetica",11),width=8).grid(row=35, column=9,sticky=W)

#title Label
Label(ventana, text='',font=("Helvetica",11,"bold"),height=1,anchor=S).grid(row=42, column=0,columnspan=6)

#boton Button
Button(f5, text='Create input', command=proc1,
width=15,height=1,bg='white',font=("Helvetica",11,"bold")).grid(row=36, column=0)
Button(f5, text='Quit', command=proc5,width=15,height=1,bg='white',font=("Helvetica",11,"bold")).grid(row=36,
column=1)

#ejecucin de ventana
ventana.mainloop()
```

The above computer codes are gathered in a package called TSSCDS1.0, which can be freely obtained from the authors upon request. TSSCDS1.0 is interfaced with MOPAC2016 (employed in steps 2 and 3), Gaussian09 (employed in steps 3-5) and two KMC codes employed in step 6. Although the input data is very simple, TSSCDS1.0 features a friendly Graphical User Interface (GUI), which runs on both Windows and Linux. A complete manual of TSSCD1.0 is being written and it will be available very soon.

The GUI that has been coded using Tkinter (see above the code), a toolkit of Python, presents the following appearance for the test case selected in our study.

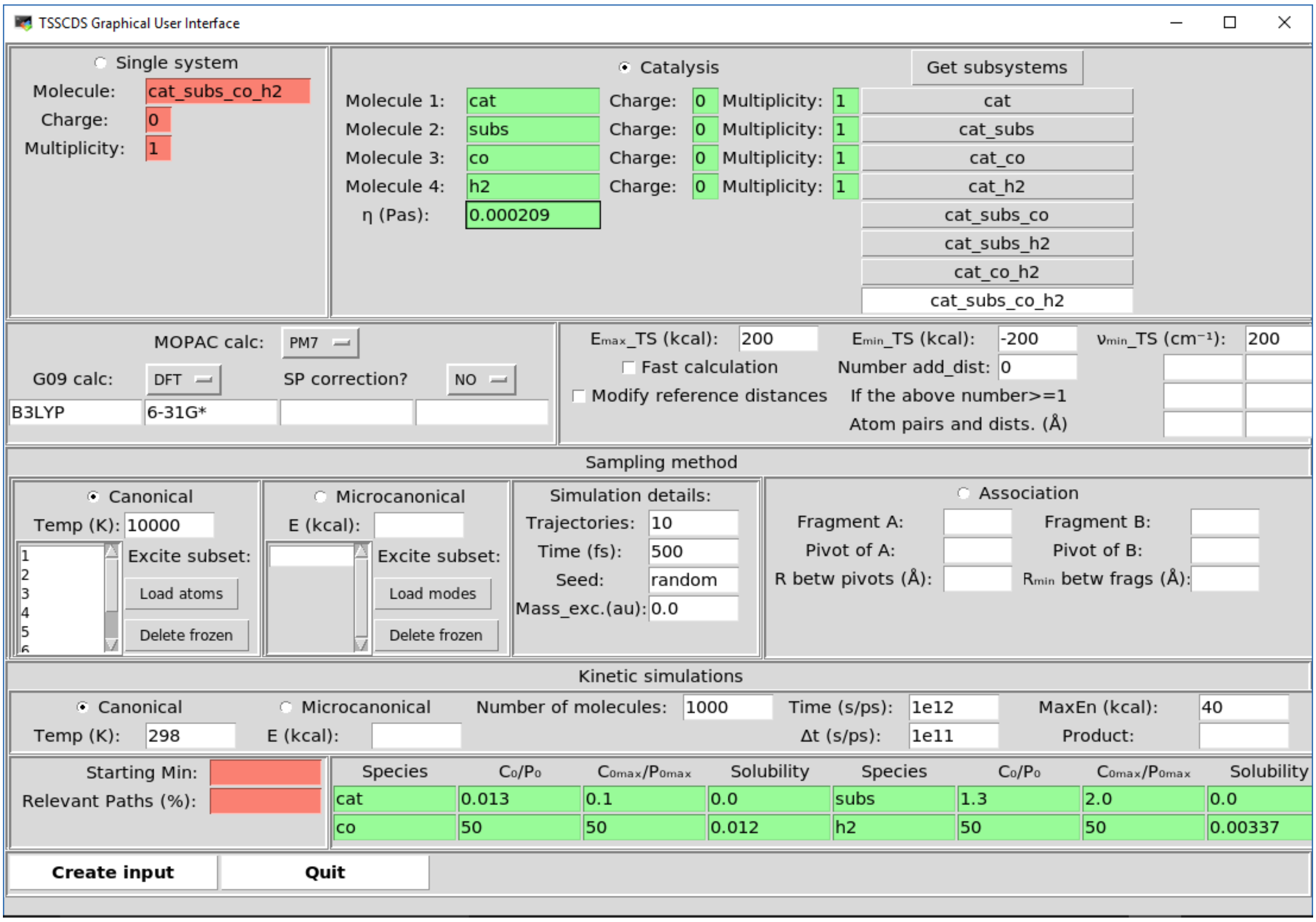

3. **Reaction network**

Below you can find the merged reaction network for the test case of this study. A total of four species are provided as input of our program: the active catalyst **1**, the alkene **2**, carbon monoxide **3** and molecular hydrogen **4**. In the lines below, these four species have different labels from those employed in the manuscript. There are two additional (and different labels):

i) *TAGX_Y*: Within each sub-system the intermediates and TSs are sorted and labelled as TAGX_Y, where TAG is either MIN or TS, and X is the order within sub-system Y. For instance, the active catalyst is MIN1_1, with means that it is the most stable intermediate of sub-system 1.

ii) *Klabel:* The second label is an overall one, which is a consequence of merging all sub-systems to get a single reaction network. For instance, for the active catalyst Klabel is 4, and the Klabels of the alkene, CO and H_2 are 1, 2 and 3, respectively.

Additionally, the species and transition states of the main text are labelled using roman numerals, which correspond to the following TAGX_Y and Klabels:

Species	MINX_Y	Klabel	Transition states	TSX_Y	Transition states	TSX_Y
**I**	MIN1_3	60	$\mathbf{TS_{II\rightarrow III}}$	TS9_2	$\mathbf{TS_{XV\rightarrow XVII}}$	TS20_6
**II**	MIN3_2	15	$\mathbf{TS_{III\rightarrow IV}}$	TS36_5	$\mathbf{TS_{XVI\rightarrow XVII}}$	TS19_6
**III**	MIN1_2	13	$\mathbf{TS_{IV\rightarrow V}}$	TS13_5		
**IV**	MIN1_5	86	$\mathbf{TS_{V\rightarrow VI}}$	TS14_5		
**V**	MIN6_5	91	$\mathbf{TS_{V\rightarrow VII}}$	TS36_8		
**VI**	MIN2_5	87	$\mathbf{TS1_{VI\rightarrow VII}}$	TS23_8		
**VII**	MIN41_8	448	$\mathbf{TS2_{VI\rightarrow VII}}$	TS31_8		
**VIII**	MIN77_8	484	$\mathbf{TS_{VII\rightarrow VIII}}$	TS34_8		
**IX**	MIN43_8	450	$\mathbf{TS_{VIII\rightarrow IX}}$	TS55_8		
**X**	-	705	$\mathbf{TS_{IX\rightarrow XI}}$	TS29_8		
**XI**	MIN21_8	428	$\mathbf{TS_{IX\rightarrow 1}}$	TS24_8		
**XII**	MIN6_8	413	$\mathbf{TS_{XI\rightarrow XII}}$	TS9_8		
**XIII**	MIN16_8	423	$\mathbf{TS_{XI\rightarrow XIII}}$	TS25_8		
**XIV**	MIN1_8	408	$\mathbf{TS_{XII\rightarrow XIII}}$	TS20_8		
**XV**	MIN7_6	168	$\mathbf{TS_{III\rightarrow XIV}}$	TS11_8		
**XVI**	MIN12_6	173	$\mathbf{TS_{III\rightarrow XV}}$	TS5_6		
**XVII**	MIN1_6	162	$\mathbf{TS_{XV\rightarrow XVI}}$	TS12_6		

Reference system:

Klabel	Name	Formula
1	subs	C2H4
2	co	CO
3	h2	H2

Reference energy: -1916.35602455 Hartree
Klabel==label employed in the KMC simulations
Gibbs Free Energy (G) in kcal/mol
Diffusion-controlled rate constant= 44873167286.5 M^{-1} s^{-1}

Subsystem 1 cat C3CoHO3

TSlabel	G_TS		MINlabel	Klabel	G_MIN		PRlabel	Klabel	G_PRO
Barrier-less dissoc		==>	MIN 1_1	4	0.000	<-->	C2CoHO2 + CO	2 + 674	38.283
TS 2_1	27.062	==>	MIN 3_1	6	12.773	<-->	MIN 4_1	7	25.550
TS 3_1	30.697	==>	MIN 1_1	4	0.000	<-->	MIN 4_1	7	25.550
TS 4_1	32.448	==>	MIN 1_1	4	0.000	<-->	MIN 5_1	8	31.435
TS 6_1	33.648	==>	MIN 1_1	4	0.000	<-->	MIN 4_1	7	25.550
TS 7_1	35.613	==>	MIN 1_1	4	0.000	<-->	MIN 5_1	8	31.435
TS 12_1	37.527	==>	MIN 1_1	4	0.000	<-->	MIN 9_1	12	33.306
TS 13_1	37.990	==>	MIN 5_1	8	31.435	<-->	MIN 9_1	12	33.306
TS 14_1	38.011	==>	MIN 1_1	4	0.000	<-->	MIN 3_1	6	12.773

Subsystem 2 cat_subs C5CoH5O3

TSlabel	G_TS		MINlabel	Klabel	G_MIN		PRlabel	Klabel	G_PRO
Barrier-less dissoc		==>	MIN 11_2	23	9.677	<-->	C3CoHO3 + C2H4	1 + 7	25.550
Barrier-less dissoc		==>	MIN 11_2	23	9.677	<-->	C4CoH5O2 + CO	2 + 677	25.623
Barrier-less dissoc		==>	MIN 1_2	13	-9.477	<-->	C4CoH5O2 + CO	2 + 676	21.585
Barrier-less dissoc		==>	MIN 3_2	15	-6.802	<-->	C3CoHO3 + C2H4	1 + 4	0.000
Barrier-less dissoc		==>	MIN 3_2	15	-6.802	<-->	C4CoH5O2 + CO	2 + 675	19.538
TS 9_2	-0.304	==>	MIN 1_2	13	-9.477	<-->	MIN 3_2	15	-6.802
TS 17_2	12.440	==>	MIN 3_2	15	-6.802	<-->	MIN 11_2	23	9.677
TS 20_2	15.441	==>	MIN 1_2	13	-9.477	<-->	MIN 3_2	15	-6.802
TS 23_2	20.105	==>	MIN 22_2	34	20.309	<-->	H2 + C5CoH3O3	3 + 55	9.662
TS 26_2	21.392	==>	MIN 20_2	32	20.195	<-->	H2 + C5CoH3O3	3 + 56	11.074
TS 27_2	21.611	==>	MIN 22_2	34	20.309	<-->	H2 + C5CoH3O3	3 + 57	11.043
TS 28_2	21.630	==>	MIN 1_2	13	-9.477	<-->	MIN 16_2	28	18.988
TS 38_2	25.511	==>	MIN 20_2	32	20.195	<-->	H2 + C5CoH3O3	3 + 58	11.069
TS 39_2	25.815	==>	MIN 20_2	32	20.195	<-->	H2 + C5CoH3O3	3 + 59	11.074
TS 40_2	25.963	==>	MIN 3_2	15	-6.802	<-->	MIN 11_2	23	9.677
TS 42_2	26.263	==>	MIN 22_2	34	20.309	<-->	MIN 41_2	53	26.121
TS 43_2	26.904	==>	MIN 22_2	34	20.309	<-->	MIN 42_2	54	26.792
TS 44_2	28.114	==>	MIN 41_2	53	26.121	<-->	C2H4 + C3CoHO3	1 + 4	0.002
TS 45_2	28.235	==>	MIN 42_2	54	26.792	<-->	C2H4 + C3CoHO3	1 + 4	0.006
TS 48_2	29.187	==>	MIN 3_2	15	-6.802	<-->	MIN 22_2	34	20.309
TS 49_2	29.194	==>	MIN 39_2	51	24.148	<-->	MIN 42_2	54	26.792
TS 51_2	30.188	==>	MIN 1_2	13	-9.477	<-->	MIN 24_2	36	20.420
TS 52_2	30.498	==>	MIN 1_2	13	-9.477	<-->	MIN 16_2	28	18.988
TS 55_2	31.394	==>	MIN 20_2	32	20.195	<-->	MIN 22_2	34	20.309
TS 56_2	32.145	==>	MIN 20_2	32	20.195	<-->	MIN 22_2	34	20.309
TS 57_2	32.296	==>	MIN 22_2	34	20.309	<-->	MIN 35_2	47	23.014

Subsystem 3 cat_co C4CoHO4

TSlabel	G_TS		MINlabel	Klabel	G_MIN		PRlabel	Klabel	G_PRO
Barrier-less dissoc		==>	MIN 1_3	60	-21.339	<-->	C3CoHO3 + CO	2 + 4	0.000
TS 6_3	7.682	==>	MIN 1_3	60	-21.339	<-->	MIN 3_3	62	7.236
TS 9_3	9.686	==>	MIN 4_3	63	8.577	<-->	CO + C3CoHO3	2 + 4	0.000
TS 10_3	10.316	==>	MIN 1_3	60	-21.339	<-->	MIN 2_3	61	0.950
TS 11_3	12.165	==>	MIN 1_3	60	-21.339	<-->	MIN 2_3	61	0.950
TS 12_3	15.663	==>	MIN 1_3	60	-21.339	<-->	MIN 7_3	66	14.269
TS 13_3	15.931	==>	MIN 4_3	63	8.577	<-->	MIN 7_3	66	14.269

Subsystem 4 cat_h2 C3CoH3O3

TSlabel	G_TS		MINlabel	Klabel	G_MIN		PRlabel	Klabel	G_PRO
Barrier-less dissoc		==>	MIN 3_4	69	5.962	<-->	C2CoH3O2 + CO	2 + 679	37.047
Barrier-less dissoc		==>	MIN 1_4	67	2.980	<-->	C2CoH3O2 + CO	2 + 678	28.152
Barrier-less dissoc		==>	MIN 1_4	67	2.980	<-->	C3CoHO3 + H2	3 + 4	0.000
TS 2_4	6.602	==>	MIN 1_4	67	2.980	<-->	MIN 3_4	69	5.962
TS 3_4	11.033	==>	MIN 1_4	67	2.980	<-->	MIN 3_4	69	5.962
TS 5_4	25.930	==>	MIN 7_4	73	24.410	<-->	H2 + C3CoHO3	3 + 6	12.779
TS 9_4	29.492	==>	MIN 7_4	73	24.410	<-->	MIN 11_4	77	29.804
TS 11_4	32.316	==>	MIN 1_4	67	2.980	<-->	MIN 7_4	73	24.410
TS 12_4	32.618	==>	MIN 1_4	67	2.980	<-->	MIN 7_4	73	24.410
TS 13_4	32.689	==>	MIN 1_4	67	2.980	<-->	MIN 14_4	80	33.122

TS 15_4	35.176	==>	MIN 6_4	72	17.134	<-->	MIN 15_4	81	34.344
TS 16_4	36.511	==>	MIN 15_4	81	34.344	<-->	MIN 11_4	77	29.804
TS 17_4	36.740	==>	MIN 1_4	67	2.980	<-->	MIN 15_4	81	34.344
TS 20_4	37.345	==>	MIN 1_4	67	2.980	<-->	MIN 7_4	73	24.410
TS 22_4	40.006	==>	MIN 7_4	73	24.410	<-->	MIN 11_4	77	29.804
TS 24_4	40.086	==>	MIN 7_4	73	24.410	<-->	MIN 19_4	85	40.409

Subsystem 5 cat_subs_co C6CoH5O4

TSlabel	G_TS		MINlabel	Klabel	G_MIN		PRlabel	Klabel	G_PRO
Barrier-less dissoc		==>	MIN 2_5	87	-17.138	<-->	C5CoH5O3 + CO	2 + 680	-5.984
Barrier-less dissoc		==>	MIN 1_5	86	-17.382	<-->	C5CoH5O3 + CO	2 + 13	-5.439
TS 13_5	-6.665	==>	MIN 1_5	86	-17.382	<-->	MIN 6_5	91	-11.124
TS 14_5	-6.546	==>	MIN 2_5	87	-17.138	<-->	MIN 6_5	91	-11.124
TS 16_5	-4.585	==>	MIN 6_5	91	-11.124	<-->	MIN 10_5	95	-6.555
TS 17_5	-4.117	==>	MIN 2_5	87	-17.138	<-->	MIN 10_5	95	-6.555
TS 19_5	-1.826	==>	MIN 2_5	87	-17.138	<-->	MIN 6_5	91	-11.124
TS 21_5	-0.039	==>	MIN 2_5	87	-17.138	<-->	MIN 6_5	91	-11.124
TS 25_5	2.177	==>	MIN 2_5	87	-17.138	<-->	MIN 10_5	95	-6.555
TS 31_5	3.115	==>	MIN 7_5	92	-10.052	<-->	MIN 13_5	98	-0.500
TS 36_5	4.338	==>	MIN 1_5	86	-17.382	<-->	CO + C5CoH5O3	2 + 13	-8.186
TS 40_5	5.782	==>	MIN 7_5	92	-10.052	<-->	MIN 13_5	98	-0.500
TS 42_5	6.907	==>	MIN 7_5	92	-10.052	<-->	MIN 19_5	104	4.348
TS 44_5	7.516	==>	MIN 7_5	92	-10.052	<-->	MIN 19_5	104	4.348
TS 45_5	8.537	==>	MIN 7_5	92	-10.052	<-->	MIN 12_5	97	-1.052
TS 46_5	9.479	==>	MIN 7_5	92	-10.052	<-->	MIN 20_5	105	4.710
TS 48_5	11.267	==>	MIN 2_5	87	-17.138	<-->	MIN 6_5	91	-11.124
TS 49_5	11.284	==>	MIN 2_5	87	-17.138	<-->	MIN 10_5	95	-6.555
TS 58_5	13.291	==>	MIN 6_5	91	-11.124	<-->	MIN 20_5	105	4.710
TS 66_5	14.957	==>	MIN 6_5	91	-11.124	<-->	MIN 20_5	105	4.710
TS 74_5	16.838	==>	MIN 6_5	91	-11.124	<-->	MIN 57_5	142	15.988
TS 80_5	17.445	==>	MIN 57_5	142	15.988	<-->	MIN 58_5	143	16.087
TS 83_5	17.835	==>	MIN 2_5	87	-17.138	<-->	MIN 32_5	117	9.339
TS 84_5	18.196	==>	MIN 58_5	143	16.087	<-->	CO + C5CoH5O3	2 + 13	-5.448
TS 86_5	18.571	==>	MIN 2_5	87	-17.138	<-->	MIN 32_5	117	9.339
TS 102_5	21.171	==>	MIN 10_5	95	-6.555	<-->	MIN 76_5	161	20.571

Subsystem 6 cat_subs_h2 C5CoH7O3

TSlabel	G_TS		MINlabel	Klabel	G_MIN		PRlabel	Klabel	G_PRO
Barrier-less dissoc		==>	MIN 1_6	162	-12.541	<-->	C3CoHO3 + C2H6	681 + 4	-22.825
TS 5_6	4.965	==>	MIN 7_6	168	4.191	<-->	H2 + C5CoH5O3	3 + 13	-5.443
TS 6_6	6.346	==>	MIN 7_6	168	4.191	<-->	H2 + C5CoH5O3	3 + 13	-8.178
TS 12_6	9.204	==>	MIN 7_6	168	4.191	<-->	MIN 12_6	173	9.372
TS 13_6	9.451	==>	MIN 7_6	168	4.191	<-->	MIN 12_6	173	9.372
TS 15_6	12.826	==>	MIN 7_6	168	4.191	<-->	MIN 12_6	173	9.372
TS 17_6	13.638	==>	MIN 7_6	168	4.191	<-->	MIN 12_6	173	9.372
TS 19_6	13.984	==>	MIN 1_6	162	-12.541	<-->	MIN 12_6	173	9.372
TS 20_6	14.458	==>	MIN 1_6	162	-12.541	<-->	MIN 7_6	168	4.191
TS 21_6	15.436	==>	MIN 1_6	162	-12.541	<-->	MIN 12_6	173	9.372
TS 24_6	22.513	==>	MIN 10_6	171	7.763	<-->	MIN 19_6	180	11.902
TS 26_6	25.439	==>	MIN 19_6	180	11.902	<-->	MIN 35_6	196	25.782
TS 27_6	28.394	==>	MIN 12_6	173	9.372	<-->	MIN 35_6	196	25.782
TS 31_6	31.675	==>	MIN 17_6	178	11.261	<-->	MIN 12_6	173	9.372
TS 32_6	31.797	==>	MIN 31_6	192	19.964	<-->	MIN 36_6	197	25.975
TS 35_6	33.607	==>	MIN 1_6	162	-12.541	<-->	MIN 36_6	197	25.975
TS 36_6	33.615	==>	MIN 31_6	192	19.964	<-->	MIN 36_6	197	25.975
TS 37_6	34.326	==>	MIN 36_6	197	25.975	<-->	MIN 56_6	217	31.630
TS 38_6	35.091	==>	MIN 12_6	173	9.372	<-->	MIN 36_6	197	25.975
TS 39_6	35.270	==>	MIN 46_6	207	29.368	<-->	MIN 56_6	217	31.630
TS 42_6	38.328	==>	MIN 19_6	180	11.902	<-->	MIN 45_6	206	29.244
TS 43_6	41.641	==>	MIN 19_6	180	11.902	<-->	MIN 45_6	206	29.244

Subsystem 7 cat_co_h2 C4CoH3O4

TSlabel	G_TS		MINlabel	Klabel	G_MIN		PRlabel	Klabel	G_PRO
Barrier-less dissoc		==>	MIN 68_7	285	29.629	<-->	C3CoH3O3 + CO	2 + 704	52.101
Barrier-less dissoc		==>	MIN 68_7	285	29.629	<-->	C3CoHO3 + CH2O	703 + 7	32.082
Barrier-less dissoc		==>	MIN 25_7	242	16.095	<-->	C3CoH3O3 + CO	2 + 702	40.639
Barrier-less dissoc		==>	MIN 25_7	242	16.095	<-->	C3CoHO3 + CH2O	703 + 4	16.497
Barrier-less dissoc		==>	MIN 12_7	229	10.039	<-->	C3CoH3O3 + CO	2 + 700	31.840
Barrier-less dissoc		==>	MIN 40_7	257	20.129	<-->	C3CoH3O3 + CO	2 + 699	56.461
Barrier-less dissoc		==>	MIN 3_7	220	4.175	<-->	C3CoH3O3 + CO	2 + 698	15.495
Barrier-less dissoc		==>	MIN 56_7	273	23.625	<-->	C3CoH3O3 + CO	2 + 697	40.974
Barrier-less dissoc		==>	MIN 28_7	245	16.656	<-->	C3CoH3O3 + CO	2 + 696	26.884
Barrier-less dissoc		==>	MIN 28_7	245	16.656	<-->	C3CoHO3 + CH2O	703 + 4	6.522
Barrier-less dissoc		==>	MIN 6_7	223	7.746	<-->	C3CoH3O3 + CO	2 + 694	26.057

Barrier-less dissoc			==>	MIN	6_7	223	7.746	<-->	C3CoHO3 + CH2O	703 + 4	16.535
Barrier-less dissoc			==>	MIN	4_7	221	4.483	<-->	C3CoH3O3 + CO	2 + 692	9.246
Barrier-less dissoc			==>	MIN	26_7	243	16.142	<-->	C3CoH3O3 + CO	2 + 691	24.869
Barrier-less dissoc			==>	MIN	26_7	243	16.142	<-->	C3CoHO3 + CH2O	703 + 4	6.523
Barrier-less dissoc			==>	MIN	50_7	267	22.461	<-->	C3CoH3O3 + CO	2 + 689	43.150
Barrier-less dissoc			==>	MIN	72_7	289	32.769	<-->	C3CoH3O3 + CO	2 + 81	34.340
Barrier-less dissoc			==>	MIN	64_7	281	27.200	<-->	C3CoH3O3 + CO	2 + 688	38.735
Barrier-less dissoc			==>	MIN	65_7	282	27.779	<-->	C3CoH3O3 + CO	2 + 687	46.043
Barrier-less dissoc			==>	MIN	65_7	282	27.779	<-->	C3CoHO3 + CH2O	703 + 6	19.300
Barrier-less dissoc			==>	MIN	1_7	218	-1.026	<-->	C3CoH3O3 + CO	2 + 685	6.185
Barrier-less dissoc			==>	MIN	5_7	222	7.091	<-->	C3CoH3O3 + CO	2 + 72	17.136
Barrier-less dissoc			==>	MIN	5_7	222	7.091	<-->	C3CoHO3 + CH2O	703 + 4	6.523
Barrier-less dissoc			==>	MIN	29_7	246	17.046	<-->	C3CoH3O3 + CO	2 + 72	17.109
Barrier-less dissoc			==>	MIN	29_7	246	17.046	<-->	C3CoHO3 + CH2O	703 + 4	6.523
Barrier-less dissoc			==>	MIN	20_7	237	14.383	<-->	C3CoH3O3 + CO	2 + 73	24.448
TS	5_7	11.903	==>	MIN	4_7	221	4.483	<-->	MIN 5_7	222	7.091
TS	10_7	13.824	==>	MIN	3_7	220	4.175	<-->	MIN 12_7	229	10.039
TS	16_7	15.721	==>	MIN	6_7	223	7.746	<-->	MIN 29_7	246	17.046
TS	17_7	15.745	==>	MIN	6_7	223	7.746	<-->	MIN 29_7	246	17.046
TS	19_7	16.006	==>	MIN	6_7	223	7.746	<-->	MIN 5_7	222	7.091
TS	22_7	16.281	==>	MIN	20_7	237	14.383	<-->	H2 + C4CoHO4	3 + 60	-21.350
TS	25_7	16.809	==>	MIN	20_7	237	14.383	<-->	H2 + C4CoHO4	3 + 61	0.950
TS	28_7	17.000	==>	MIN	6_7	223	7.746	<-->	MIN 28_7	245	16.656
TS	29_7	17.229	==>	MIN	3_7	220	4.175	<-->	MIN 12_7	229	10.039
TS	32_7	17.391	==>	MIN	5_7	222	7.091	<-->	MIN 26_7	243	16.142
TS	36_7	18.730	==>	MIN	5_7	222	7.091	<-->	MIN 29_7	246	17.046
TS	39_7	19.091	==>	MIN	20_7	237	14.383	<-->	MIN 37_7	254	18.838
TS	44_7	20.438	==>	MIN	20_7	237	14.383	<-->	MIN 37_7	254	18.838
TS	46_7	21.028	==>	MIN	12_7	229	10.039	<-->	MIN 25_7	242	16.095
TS	48_7	21.505	==>	MIN	12_7	229	10.039	<-->	MIN 25_7	242	16.095
TS	52_7	22.998	==>	MIN	29_7	246	17.046	<-->	MIN 37_7	254	18.838
TS	56_7	24.504	==>	MIN	12_7	229	10.039	<-->	MIN 25_7	242	16.095
TS	60_7	25.313	==>	MIN	29_7	246	17.046	<-->	MIN 37_7	254	18.838
TS	62_7	25.419	==>	MIN	20_7	237	14.383	<-->	MIN 37_7	254	18.838
TS	64_7	26.050	==>	MIN	26_7	243	16.142	<-->	MIN 56_7	273	23.625
TS	66_7	26.754	==>	MIN	2_7	219	1.421	<-->	MIN 44_7	261	21.380
TS	67_7	26.772	==>	MIN	3_7	220	4.175	<-->	MIN 5_7	222	7.091
TS	69_7	27.266	==>	MIN	5_7	222	7.091	<-->	MIN 29_7	246	17.046
TS	71_7	27.512	==>	MIN	12_7	229	10.039	<-->	MIN 25_7	242	16.095
TS	76_7	29.022	==>	MIN	1_7	218	-1.026	<-->	MIN 5_7	222	7.091
TS	78_7	30.300	==>	MIN	6_7	223	7.746	<-->	MIN 65_7	282	27.779
TS	79_7	30.486	==>	MIN	25_7	242	16.095	<-->	MIN 68_7	285	29.629
TS	82_7	30.693	==>	MIN	26_7	243	16.142	<-->	MIN 64_7	281	27.200
TS	86_7	31.317	==>	MIN	44_7	261	21.380	<-->	MIN 60_7	277	24.918
TS	87_7	31.441	==>	MIN	26_7	243	16.142	<-->	MIN 56_7	273	23.625
TS	89_7	32.052	==>	MIN	44_7	261	21.380	<-->	MIN 60_7	277	24.918
TS	90_7	32.435	==>	MIN	5_7	222	7.091	<-->	MIN 65_7	282	27.779
TS	91_7	32.603	==>	MIN	43_7	260	20.987	<-->	MIN 44_7	261	21.380
TS	93_7	32.918	==>	MIN	64_7	281	27.200	<-->	MIN 65_7	282	27.779
TS	95_7	33.076	==>	MIN	26_7	243	16.142	<-->	MIN 40_7	257	20.129
TS	96_7	33.387	==>	MIN	37_7	254	18.838	<-->	MIN 72_7	289	32.769
TS	99_7	33.722	==>	MIN	50_7	267	22.461	<-->	MIN 65_7	282	27.779
TS	100_7	33.848	==>	MIN	26_7	243	16.142	<-->	MIN 29_7	246	17.046
TS	101_7	34.277	==>	MIN	77_7	294	33.836	<-->	H2 + C4CoHO4	3 + 393	22.197
TS	102_7	34.307	==>	MIN	12_7	229	10.039	<-->	MIN 25_7	242	16.095
TS	103_7	34.462	==>	MIN	79_7	296	34.205	<-->	CO + C3CoH3O3	2 + 394	9.238
TS	104_7	34.505	==>	MIN	6_7	223	7.746	<-->	MIN 80_7	297	34.317
TS	105_7	34.555	==>	MIN	20_7	237	14.383	<-->	CO + C3CoH3O3	2 + 67	2.981
TS	106_7	34.603	==>	MIN	20_7	237	14.383	<-->	MIN 25_7	242	16.095
TS	109_7	35.037	==>	MIN	5_7	222	7.091	<-->	CO + C3CoH3O3	2 + 395	24.527
TS	115_7	35.636	==>	MIN	6_7	223	7.746	<-->	MIN 80_7	297	34.317
TS	116_7	35.669	==>	MIN	5_7	222	7.091	<-->	MIN 79_7	296	34.205
TS	118_7	35.860	==>	MIN	3_7	220	4.175	<-->	MIN 26_7	243	16.142
TS	119_7	35.932	==>	MIN	39_7	256	19.594	<-->	MIN 86_7	303	35.070
TS	120_7	36.029	==>	MIN	12_7	229	10.039	<-->	MIN 25_7	242	16.095
TS	121_7	36.103	==>	MIN	5_7	222	7.091	<-->	MIN 65_7	282	27.779
TS	122_7	36.318	==>	MIN	20_7	237	14.383	<-->	CO + C3CoH3O3	2 + 73	24.409
TS	123_7	36.914	==>	MIN	93_7	310	36.492	<-->	CO + C3CoH3O3	2 + 396	24.871
TS	124_7	37.151	==>	MIN	3_7	220	4.175	<-->	MIN 12_7	229	10.039
TS	126_7	37.514	==>	MIN	26_7	243	16.142	<-->	MIN 72_7	289	32.769
TS	128_7	37.888	==>	MIN	5_7	222	7.091	<-->	MIN 26_7	243	16.142
TS	129_7	37.905	==>	MIN	5_7	222	7.091	<-->	MIN 72_7	289	32.769
TS	130_7	38.313	==>	MIN	20_7	237	14.383	<-->	MIN 77_7	294	33.836
TS	131_7	38.350	==>	MIN	29_7	246	17.046	<-->	MIN 82_7	299	34.690
TS	133_7	38.629	==>	MIN	12_7	229	10.039	<-->	MIN 103_7	320	37.953
TS	134_7	38.861	==>	MIN	3_7	220	4.175	<-->	MIN 91_7	308	36.310
TS	136_7	39.033	==>	MIN	20_7	237	14.383	<-->	CO + C3CoH3O3	2 + 73	25.722

TS	137_7	39.043	==>	MIN	5_7	222	7.091	<-->	MIN 72_7	289	32.769
TS	142_7	39.555	==>	MIN	93_7	310	36.492	<-->	CO + C3CoH3O3	2 + 397	24.869
TS	143_7	39.565	==>	MIN	9_7	226	9.190	<-->	MIN 60_7	277	24.918
TS	146_7	39.917	==>	MIN	26_7	243	16.142	<-->	CO + C3CoH3O3	2 + 398	28.920
TS	147_7	40.003	==>	MIN	93_7	310	36.492	<-->	MIN 113_7	330	39.543
TS	148_7	40.138	==>	MIN	26_7	243	16.142	<-->	MIN 72_7	289	32.769
TS	149_7	40.616	==>	MIN	37_7	254	18.838	<-->	MIN 72_7	289	32.769
TS	150_7	40.694	==>	MIN	72_7	289	32.769	<-->	CH2O + C3CoHO3	703 + 6	19.297
TS	152_7	41.012	==>	MIN	12_7	229	10.039	<-->	MIN 72_7	289	32.769
TS	154_7	41.063	==>	MIN	95_7	312	36.598	<-->	MIN 129_7	346	41.551
TS	156_7	41.191	==>	MIN	109_7	326	38.841	<-->	CH2O + C3CoHO3	703 + 6	19.296
TS	157_7	41.224	==>	MIN	29_7	246	17.046	<-->	MIN 72_7	289	32.769
TS	158_7	41.371	==>	MIN	20_7	237	14.383	<-->	MIN 95_7	312	36.598
TS	159_7	41.567	==>	MIN	26_7	243	16.142	<-->	MIN 109_7	326	38.841
TS	160_7	41.793	==>	MIN	37_7	254	18.838	<-->	MIN 109_7	326	38.841
TS	161_7	41.806	==>	MIN	26_7	243	16.142	<-->	CO + C3CoH3O3	2 + 401	24.869
TS	162_7	42.204	==>	MIN	20_7	237	14.383	<-->	MIN 77_7	294	33.836
TS	165_7	42.453	==>	MIN	91_7	308	36.310	<-->	MIN 132_7	349	42.769
TS	168_7	42.853	==>	MIN	26_7	243	16.142	<-->	MIN 109_7	326	38.841
TS	169_7	42.997	==>	MIN	129_7	346	41.551	<-->	CO + C3CoH3O3	2 + 80	33.122
TS	170_7	43.091	==>	MIN	5_7	222	7.091	<-->	MIN 119_7	336	40.260
TS	173_7	43.245	==>	MIN	37_7	254	18.838	<-->	MIN 72_7	289	32.769
TS	174_7	43.355	==>	MIN	2_7	219	1.421	<-->	MIN 16_7	233	10.925
TS	175_7	43.418	==>	MIN	5_7	222	7.091	<-->	MIN 136_7	353	43.148
TS	176_7	43.458	==>	MIN	37_7	254	18.838	<-->	CO + C3CoH3O3	2 + 69	10.693
TS	177_7	43.496	==>	MIN	26_7	243	16.142	<-->	MIN 112_7	329	39.351
TS	178_7	43.712	==>	MIN	37_7	254	18.838	<-->	CO + C3CoH3O3	2 + 80	33.124
TS	179_7	43.823	==>	MIN	95_7	312	36.598	<-->	CO + C3CoH3O3	2 + 67	2.983
TS	180_7	43.883	==>	MIN	5_7	222	7.091	<-->	MIN 6_7	223	7.746
TS	184_7	43.991	==>	MIN	56_7	273	23.625	<-->	CO + C3CoH3O3	2 + 402	28.919
TS	185_7	44.099	==>	MIN	26_7	243	16.142	<-->	MIN 109_7	326	38.841
TS	186_7	44.104	==>	MIN	12_7	229	10.039	<-->	MIN 132_7	349	42.769
TS	189_7	44.332	==>	MIN	9_7	226	9.190	<-->	MIN 43_7	260	20.987
TS	193_7	44.623	==>	MIN	103_7	320	37.953	<-->	MIN 135_7	352	43.114
TS	194_7	44.634	==>	MIN	144_7	361	44.281	<-->	CO + C3CoH3O3	2 + 67	2.980
TS	195_7	44.716	==>	MIN	5_7	222	7.091	<-->	MIN 39_7	256	19.594
TS	196_7	44.784	==>	MIN	109_7	326	38.841	<-->	CH2O + C3CoHO3	703 + 6	19.295
TS	197_7	44.787	==>	MIN	109_7	326	38.841	<-->	CH2O + C3CoHO3	703 + 6	19.294
TS	198_7	44.884	==>	MIN	22_7	239	15.161	<-->	MIN 128_7	345	41.421
TS	199_7	44.970	==>	MIN	29_7	246	17.046	<-->	MIN 82_7	299	34.690
TS	200_7	45.026	==>	MIN	60_7	277	24.918	<-->	MIN 125_7	342	41.278
TS	203_7	45.067	==>	MIN	20_7	237	14.383	<-->	MIN 144_7	361	44.281
TS	205_7	45.107	==>	MIN	60_7	277	24.918	<-->	MIN 77_7	294	33.836
TS	210_7	45.378	==>	MIN	79_7	296	34.205	<-->	CO + C3CoH3O3	2 + 405	9.237
TS	211_7	45.413	==>	MIN	69_7	286	29.756	<-->	MIN 119_7	336	40.260
TS	213_7	45.527	==>	MIN	26_7	243	16.142	<-->	MIN 37_7	254	18.838
TS	214_7	45.669	==>	MIN	144_7	361	44.281	<-->	H2 + C4CoHO4	3 + 61	0.952
TS	215_7	45.906	==>	MIN	58_7	275	24.593	<-->	MIN 153_7	370	45.228
TS	217_7	46.158	==>	MIN	37_7	254	18.838	<-->	MIN 109_7	326	38.841
TS	219_7	46.203	==>	MIN	60_7	277	24.918	<-->	MIN 141_7	358	43.823
TS	220_7	46.242	==>	MIN	136_7	353	43.148	<-->	MIN 79_7	296	34.205
TS	222_7	46.303	==>	MIN	95_7	312	36.598	<-->	H2 + C4CoHO4	3 + 60	-21.336
TS	223_7	46.344	==>	MIN	74_7	291	33.238	<-->	MIN 96_7	313	36.765
TS	224_7	46.380	==>	MIN	22_7	239	15.161	<-->	MIN 60_7	277	24.918
TS	226_7	46.479	==>	MIN	37_7	254	18.838	<-->	MIN 72_7	289	32.769
TS	227_7	46.523	==>	MIN	22_7	239	15.161	<-->	MIN 60_7	277	24.918
TS	229_7	46.661	==>	MIN	37_7	254	18.838	<-->	MIN 82_7	299	34.690
TS	230_7	46.684	==>	MIN	25_7	242	16.095	<-->	MIN 68_7	285	29.629
TS	232_7	46.868	==>	MIN	95_7	312	36.598	<-->	MIN 129_7	346	41.551
TS	233_7	46.932	==>	MIN	5_7	222	7.091	<-->	MIN 29_7	246	17.046
TS	234_7	47.083	==>	MIN	109_7	326	38.841	<-->	CH2O + C3CoHO3	703 + 6	19.294
TS	235_7	47.100	==>	MIN	3_7	220	4.175	<-->	MIN 102_7	319	37.739
TS	236_7	47.534	==>	MIN	12_7	229	10.039	<-->	MIN 37_7	254	18.838
TS	237_7	47.545	==>	MIN	39_7	256	19.594	<-->	MIN 122_7	339	40.684
TS	239_7	47.822	==>	MIN	29_7	246	17.046	<-->	MIN 26_7	243	16.142
TS	240_7	47.895	==>	MIN	26_7	243	16.142	<-->	MIN 113_7	330	39.543
TS	242_7	47.982	==>	MIN	25_7	242	16.095	<-->	MIN 68_7	285	29.629
TS	243_7	48.131	==>	MIN	79_7	296	34.205	<-->	CO + C3CoH3O3	2 + 407	24.869
TS	245_7	48.597	==>	MIN	60_7	277	24.918	<-->	MIN 125_7	342	41.278
TS	246_7	48.632	==>	MIN	29_7	246	17.046	<-->	MIN 119_7	336	40.260
TS	247_7	48.785	==>	MIN	144_7	361	44.281	<-->	MIN 129_7	346	41.551
TS	249_7	49.315	==>	MIN	74_7	291	33.238	<-->	MIN 77_7	294	33.836
TS	250_7	49.442	==>	MIN	144_7	361	44.281	<-->	MIN 129_7	346	41.551
TS	251_7	49.447	==>	MIN	12_7	229	10.039	<-->	MIN 81_7	298	34.532
TS	252_7	49.477	==>	MIN	95_7	312	36.598	<-->	H2 + C4CoHO4	3 + 61	0.950
TS	253_7	49.526	==>	MIN	162_7	379	46.582	<-->	MIN 141_7	358	43.823
TS	254_7	49.913	==>	MIN	37_7	254	18.838	<-->	MIN 109_7	326	38.841

TS 256_7	50.048	==>	MIN 58_7	275	24.593	<-->	MIN 86_7	303	35.070
TS 257_7	50.063	==>	MIN 26_7	243	16.142	<-->	MIN 152_7	369	44.787
TS 258_7	50.176	==>	MIN 60_7	277	24.918	<-->	MIN 96_7	313	36.765
TS 260_7	50.374	==>	MIN 25_7	242	16.095	<-->	MIN 92_7	309	36.489
TS 262_7	50.768	==>	MIN 25_7	242	16.095	<-->	MIN 92_7	309	36.489
TS 265_7	50.916	==>	MIN 25_7	242	16.095	<-->	MIN 92_7	309	36.489
TS 267_7	51.473	==>	MIN 5_7	222	7.091	<-->	MIN 169_7	386	49.877
TS 268_7	51.745	==>	MIN 152_7	369	44.787	<-->	MIN 167_7	384	47.589
TS 271_7	52.291	==>	MIN 12_7	229	10.039	<-->	MIN 128_7	345	41.421
TS 272_7	52.387	==>	MIN 12_7	229	10.039	<-->	MIN 128_7	345	41.421
TS 273_7	52.419	==>	MIN 25_7	242	16.095	<-->	H2 + C4CoHO4	3 + 60	-21.349
TS 274_7	52.594	==>	MIN 72_7	289	32.769	<-->	MIN 147_7	364	44.460
TS 276_7	52.634	==>	MIN 26_7	243	16.142	<-->	MIN 56_7	273	23.625
TS 280_7	52.884	==>	MIN 5_7	222	7.091	<-->	MIN 136_7	353	43.148
TS 283_7	52.963	==>	MIN 3_7	220	4.175	<-->	MIN 145_7	362	44.305
TS 285_7	53.094	==>	MIN 72_7	289	32.769	<-->	H2 + C4CoHO4	3 + 60	-21.347
TS 286_7	53.223	==>	MIN 4_7	221	4.483	<-->	H2 + C4CoHO4	3 + 61	0.950
TS 287_7	53.231	==>	MIN 9_7	226	9.190	<-->	MIN 175_7	392	50.929
TS 289_7	53.309	==>	MIN 87_7	304	35.348	<-->	MIN 96_7	313	36.765
TS 290_7	53.438	==>	MIN 40_7	257	20.129	<-->	MIN 169_7	386	49.877
TS 293_7	53.839	==>	MIN 77_7	294	33.836	<-->	MIN 141_7	358	43.823
TS 299_7	54.174	==>	MIN 25_7	242	16.095	<-->	MIN 92_7	309	36.489
TS 300_7	54.192	==>	MIN 25_7	242	16.095	<-->	MIN 37_7	254	18.838
TS 301_7	54.241	==>	MIN 9_7	226	9.190	<-->	MIN 68_7	285	29.629
TS 302_7	54.274	==>	MIN 9_7	226	9.190	<-->	MIN 89_7	306	35.530
TS 303_7	54.289	==>	MIN 5_7	222	7.091	<-->	MIN 26_7	243	16.142

Subsystem 8 cat_subs_co_h2 C6CoH7O4

TSlabel	G_TS		MINlabel	Klabel	G_MIN		PRlabel	Klabel	G_PRO
Barrier-less dissoc		==>	MIN 62_8	469	2.723	<-->	C5CoH7O3 + CO	2 + 723	22.037
Barrier-less dissoc		==>	MIN 62_8	469	2.723	<-->	C3CoHO3 + C3H6O	722 + 721	1.269
Barrier-less dissoc		==>	MIN 52_8	459	0.800	<-->	C5CoH7O3 + CO	2 + 720	27.263
Barrier-less dissoc		==>	MIN 114_8	521	12.862	<-->	C5CoH7O3 + CO	2 + 719	29.088
Barrier-less dissoc		==>	MIN 114_8	521	12.862	<-->	C3CoHO3 + C3H6O	722 + 6	4.017
Barrier-less dissoc		==>	MIN 14_8	421	-5.595	<-->	C5CoH7O3 + CO	2 + 717	5.740
Barrier-less dissoc		==>	MIN 80_8	487	4.737	<-->	C5CoH7O3 + CO	2 + 716	24.648
Barrier-less dissoc		==>	MIN 80_8	487	4.737	<-->	C3CoHO3 + C3H6O	722 + 4	-8.759
Barrier-less dissoc		==>	MIN 1_8	408	-10.095	<-->	C5CoH7O3 + CO	2 + 714	21.749
Barrier-less dissoc		==>	MIN 6_8	413	-9.324	<-->	C5CoH7O3 + CO	2 + 713	8.804
Barrier-less dissoc		==>	MIN 6_8	413	-9.324	<-->	C3CoHO3 + C3H6O	722 + 4	-8.789
Barrier-less dissoc		==>	MIN 16_8	423	-5.378	<-->	C5CoH7O3 + CO	2 + 711	25.452
Barrier-less dissoc		==>	MIN 21_8	428	-4.559	<-->	C5CoH7O3 + CO	2 + 710	5.279
Barrier-less dissoc		==>	MIN 21_8	428	-4.559	<-->	C3CoHO3 + C3H6O	722 + 4	-8.756
Barrier-less dissoc		==>	MIN 43_8	450	-0.576	<-->	C5CoH7O3 + CO	2 + 708	20.566
Barrier-less dissoc		==>	MIN 43_8	450	-0.576	<-->	C3CoHO3 + C3H6O	722 + 4	-8.755
Barrier-less dissoc		==>	MIN 77_8	484	4.351	<-->	C5CoH7O3 + CO	2 + 706	19.040
Barrier-less dissoc		==>	MIN 41_8	448	-0.929	<-->	C5CoH7O3 + CO	2 + 705	6.459
TS 9_8	-3.462	==>	MIN 6_8	413	-9.324	<-->	MIN 21_8	428	-4.559
TS 10_8	-3.415	==>	MIN 1_8	408	-10.095	<-->	MIN 16_8	423	-5.378
TS 11_8	-3.280	==>	MIN 1_8	408	-10.095	<-->	MIN 16_8	423	-5.378
TS 12_8	-3.058	==>	MIN 6_8	413	-9.324	<-->	MIN 21_8	428	-4.559
TS 13_8	-2.946	==>	MIN 6_8	413	-9.324	<-->	MIN 21_8	428	-4.559
TS 14_8	-2.832	==>	MIN 6_8	413	-9.324	<-->	MIN 21_8	428	-4.559
TS 15_8	-2.395	==>	MIN 6_8	413	-9.324	<-->	MIN 21_8	428	-4.559
TS 19_8	-0.974	==>	MIN 21_8	428	-4.559	<-->	MIN 6_8	413	-9.324
TS 20_8	-0.227	==>	MIN 16_8	423	-5.378	<-->	MIN 6_8	413	-9.324
TS 21_8	0.129	==>	MIN 16_8	423	-5.378	<-->	MIN 6_8	413	-9.324
TS 23_8	0.984	==>	MIN 41_8	448	-0.929	<-->	H2 + C6CoH5O4	3 + 87	-17.140
TS 24_8	1.043	==>	MIN 43_8	450	-0.576	<-->	C3CoHO3 + C3H6O	722 + 4	-8.755
TS 25_8	1.052	==>	MIN 16_8	423	-5.378	<-->	MIN 21_8	428	-4.559
TS 29_8	1.835	==>	MIN 21_8	428	-4.559	<-->	MIN 43_8	450	-0.576
TS 31_8	2.671	==>	MIN 41_8	448	-0.929	<-->	H2 + C6CoH5O4	3 + 87	-16.764
TS 34_8	4.554	==>	MIN 41_8	448	-0.929	<-->	MIN 77_8	484	4.351
TS 35_8	4.559	==>	MIN 14_8	421	-5.595	<-->	MIN 52_8	459	0.800
TS 36_8	4.664	==>	MIN 41_8	448	-0.929	<-->	H2 + C6CoH5O4	3 + 91	-11.123
TS 42_8	5.549	==>	MIN 14_8	421	-5.595	<-->	MIN 52_8	459	0.800
TS 45_8	6.217	==>	MIN 21_8	428	-4.559	<-->	MIN 80_8	487	4.737
TS 46_8	6.431	==>	MIN 14_8	421	-5.595	<-->	MIN 52_8	459	0.800
TS 50_8	6.849	==>	MIN 38_8	445	-1.348	<-->	C3CoHO3 + C3H6O	722 + 4	2.529
TS 51_8	6.950	==>	MIN 38_8	445	-1.348	<-->	C3CoHO3 + C3H6O	722 + 4	3.222
TS 52_8	7.061	==>	MIN 41_8	448	-0.929	<-->	MIN 77_8	484	4.351
TS 55_8	7.286	==>	MIN 43_8	450	-0.576	<-->	MIN 77_8	484	4.351
TS 58_8	8.157	==>	MIN 52_8	459	0.800	<-->	MIN 62_8	469	2.723
TS 59_8	8.219	==>	MIN 52_8	459	0.800	<-->	MIN 62_8	469	2.723
TS 60_8	8.426	==>	MIN 21_8	428	-4.559	<-->	MIN 80_8	487	4.737
TS 63_8	9.222	==>	MIN 52_8	459	0.800	<-->	MIN 62_8	469	2.723

TS	65_8	9.398	==>	MIN 41_8	448	-0.929	<-->	MIN 77_8	484	4.351
TS	66_8	9.739	==>	MIN 43_8	450	-0.576	<-->	MIN 77_8	484	4.351
TS	72_8	11.219	==>	MIN 43_8	450	-0.576	<-->	MIN 77_8	484	4.351
TS	76_8	12.156	==>	MIN 52_8	459	0.800	<-->	MIN 62_8	469	2.723
TS	77_8	12.324	==>	MIN 52_8	459	0.800	<-->	MIN 62_8	469	2.723
TS	80_8	12.497	==>	MIN 52_8	459	0.800	<-->	MIN 62_8	469	2.723
TS	81_8	12.560	==>	MIN 99_8	506	7.183	<-->	MIN 103_8	510	8.616
TS	83_8	13.627	==>	MIN 125_8	532	14.706	<-->	CH2O + C5CoH5O3	703 + 13	1.075
TS	84_8	13.873	==>	MIN 103_8	510	8.616	<-->	MIN 125_8	532	14.706
TS	86_8	14.753	==>	MIN 56_8	463	1.929	<-->	MIN 120_8	527	13.875
TS	88_8	15.392	==>	MIN 120_8	527	13.875	<-->	C3CoHO3 + C3H6O	722 + 4	11.691
TS	89_8	15.481	==>	MIN 43_8	450	-0.576	<-->	MIN 77_8	484	4.351
TS	92_8	15.882	==>	MIN 14_8	421	-5.595	<-->	MIN 21_8	428	-4.559
TS	94_8	16.072	==>	MIN 14_8	421	-5.595	<-->	MIN 21_8	428	-4.559
TS	97_8	17.035	==>	MIN 14_8	421	-5.595	<-->	MIN 21_8	428	-4.559
TS	99_8	17.530	==>	MIN 71_8	478	3.214	<-->	MIN 99_8	506	7.183
TS	100_8	17.834	==>	MIN 21_8	428	-4.559	<-->	MIN 43_8	450	-0.576
TS	104_8	18.275	==>	MIN 16_8	423	-5.378	<-->	MIN 21_8	428	-4.559
TS	105_8	18.550	==>	MIN 80_8	487	4.737	<-->	MIN 114_8	521	12.862
TS	106_8	18.567	==>	MIN 16_8	423	-5.378	<-->	MIN 21_8	428	-4.559
TS	113_8	19.636	==>	MIN 14_8	421	-5.595	<-->	MIN 117_8	524	13.544
TS	116_8	19.984	==>	MIN 148_8	555	20.920	<-->	H2 + C6CoH5O4	3 + 117	9.342
TS	119_8	20.613	==>	MIN 21_8	428	-4.559	<-->	MIN 6_8	413	-9.324
TS	121_8	21.228	==>	MIN 80_8	487	4.737	<-->	MIN 114_8	521	12.862
TS	122_8	21.455	==>	MIN 148_8	555	20.920	<-->	H2 + C6CoH5O4	3 + 117	9.514
TS	123_8	21.790	==>	MIN 21_8	428	-4.559	<-->	MIN 133_8	540	17.877
TS	124_8	21.853	==>	MIN 21_8	428	-4.559	<-->	MIN 133_8	540	17.877
TS	126_8	22.642	==>	MIN 63_8	470	2.837	<-->	C3CoHO4 + C3H6	660 + 661	-13.061
TS	128_8	22.766	==>	MIN 125_8	532	14.706	<-->	MIN 143_8	550	20.331
TS	130_8	23.338	==>	MIN 142_8	549	19.823	<-->	MIN 149_8	556	20.923
TS	131_8	23.356	==>	MIN 54_8	461	1.645	<-->	MIN 142_8	549	19.823
TS	132_8	23.542	==>	MIN 21_8	428	-4.559	<-->	MIN 80_8	487	4.737
TS	135_8	23.902	==>	MIN 21_8	428	-4.559	<-->	MIN 80_8	487	4.737
TS	136_8	24.283	==>	MIN 63_8	470	2.837	<-->	H2O + C6CoH5O3	662 + 663	-1.153
TS	138_8	24.375	==>	MIN 103_8	510	8.616	<-->	MIN 143_8	550	20.331
TS	139_8	24.414	==>	MIN 131_8	538	16.849	<-->	CH2O + C5CoH5O3	664 + 703	0.845
TS	141_8	24.709	==>	MIN 62_8	469	2.723	<-->	MIN 134_8	541	18.172
TS	147_8	25.729	==>	MIN 143_8	550	20.331	<-->	C2H6 + C4CoHO4	681 + 60	-44.188
TS	150_8	25.873	==>	MIN 1_8	408	-10.095	<-->	MIN 160_8	567	21.773
TS	151_8	26.118	==>	MIN 52_8	459	0.800	<-->	MIN 62_8	469	2.723
TS	154_8	26.547	==>	MIN 143_8	550	20.331	<-->	C2H6 + C4CoHO4	681 + 61	-21.893
TS	157_8	26.917	==>	MIN 143_8	550	20.331	<-->	C2H6 + C4CoHO4	681 + 60	-44.175
TS	162_8	27.355	==>	MIN 62_8	469	2.723	<-->	MIN 134_8	541	18.172
TS	164_8	27.627	==>	MIN 41_8	448	-0.929	<-->	MIN 148_8	555	20.920
TS	165_8	28.051	==>	MIN 38_8	445	-1.348	<-->	MIN 145_8	552	20.594
TS	166_8	28.262	==>	MIN 103_8	510	8.616	<-->	MIN 180_8	587	24.654
TS	167_8	28.265	==>	MIN 143_8	550	20.331	<-->	C2H6 + C4CoHO4	681 + 61	-21.895
TS	173_8	28.885	==>	MIN 62_8	469	2.723	<-->	MIN 77_8	484	4.351
TS	176_8	29.183	==>	MIN 71_8	478	3.214	<-->	MIN 180_8	587	24.654
TS	178_8	29.286	==>	MIN 38_8	445	-1.348	<-->	MIN 117_8	524	13.544
TS	181_8	29.582	==>	MIN 38_8	445	-1.348	<-->	MIN 145_8	552	20.594
TS	182_8	30.051	==>	MIN 177_8	584	24.605	<-->	MIN 183_8	590	24.781
TS	183_8	30.076	==>	MIN 62_8	469	2.723	<-->	MIN 77_8	484	4.351
TS	184_8	30.193	==>	MIN 52_8	459	0.800	<-->	MIN 77_8	484	4.351
TS	185_8	30.288	==>	MIN 1_8	408	-10.095	<-->	MIN 99_8	506	7.183
TS	186_8	30.444	==>	MIN 80_8	487	4.737	<-->	MIN 155_8	562	21.488
TS	187_8	30.553	==>	MIN 54_8	461	1.645	<-->	MIN 71_8	478	3.214
TS	188_8	30.661	==>	MIN 38_8	445	-1.348	<-->	MIN 145_8	552	20.594
TS	189_8	30.793	==>	MIN 41_8	448	-0.929	<-->	MIN 143_8	550	20.331
TS	196_8	31.349	==>	MIN 52_8	459	0.800	<-->	MIN 77_8	484	4.351
TS	197_8	31.565	==>	MIN 220_8	627	29.654	<-->	MIN 226_8	633	31.255
TS	199_8	31.807	==>	MIN 218_8	625	29.416	<-->	MIN 226_8	633	31.255
TS	200_8	31.825	==>	MIN 143_8	550	20.331	<-->	MIN 218_8	625	29.416
TS	201_8	31.946	==>	MIN 80_8	487	4.737	<-->	MIN 205_8	612	27.019
TS	202_8	32.030	==>	MIN 43_8	450	-0.576	<-->	MIN 229_8	636	31.453
TS	203_8	32.039	==>	MIN 77_8	484	4.351	<-->	MIN 133_8	540	17.877
TS	204_8	32.102	==>	MIN 14_8	421	-5.595	<-->	MIN 183_8	590	24.781
TS	205_8	32.300	==>	MIN 6_8	413	-9.324	<-->	H2 + C6CoH5O4	3 + 98	0.819
TS	206_8	32.364	==>	MIN 131_8	538	16.849	<-->	MIN 139_8	546	19.573
TS	207_8	32.653	==>	MIN 14_8	421	-5.595	<-->	MIN 183_8	590	24.781
TS	208_8	32.699	==>	MIN 14_8	421	-5.595	<-->	MIN 183_8	590	24.781
TS	209_8	32.702	==>	MIN 77_8	484	4.351	<-->	MIN 180_8	587	24.654
TS	210_8	33.133	==>	MIN 143_8	550	20.331	<-->	C2H6 + C4CoHO4	681 + 60	-44.184
TS	211_8	33.388	==>	MIN 43_8	450	-0.576	<-->	MIN 138_8	545	18.959
TS	212_8	33.491	==>	MIN 43_8	450	-0.576	<-->	MIN 229_8	636	31.453
TS	213_8	33.594	==>	MIN 144_8	551	20.506	<-->	MIN 238_8	645	32.991
TS	221_8	34.667	==>	MIN 154_8	561	21.428	<-->	MIN 159_8	566	21.736

TS	222_8	34.929 ==>	MIN	38_8	445	-1.348	<-->	MIN	145_8		552	20.594
TS	224_8	35.189 ==>	MIN	154_8	561	21.428	<-->	MIN	159_8		566	21.736
TS	226_8	35.264 ==>	MIN	63_8	470	2.837	<-->	MIN	183_8		590	24.781
TS	227_8	35.303 ==>	MIN	38_8	445	-1.348	<-->	MIN	145_8		552	20.594
TS	229_8	35.519 ==>	MIN	143_8	550	20.331	<-->	MIN	180_8		587	24.654
TS	231_8	35.797 ==>	MIN	6_8	413	-9.324	<-->	MIN	143_8		550	20.331
TS	232_8	36.028 ==>	MIN	143_8	550	20.331	<-->	MIN	218_8		625	29.416
TS	233_8	36.045 ==>	MIN	63_8	470	2.837	<-->	MIN	183_8		590	24.781
TS	234_8	36.051 ==>	MIN	148_8	555	20.920	<-->	MIN	238_8		645	32.991
TS	238_8	36.175 ==>	MIN	14_8	421	-5.595	<-->	MIN	159_8		566	21.736
TS	240_8	36.266 ==>	MIN	38_8	445	-1.348	<-->	MIN	159_8		566	21.736
TS	241_8	36.413 ==>	MIN	62_8	469	2.723	<-->	MIN	120_8		527	13.875
TS	242_8	36.428 ==>	MIN	14_8	421	-5.595	<-->	MIN	159_8		566	21.736
TS	244_8	36.584 ==>	MIN	38_8	445	-1.348	<-->	MIN	145_8		552	20.594
TS	247_8	36.948 ==>	MIN	38_8	445	-1.348	<-->	MIN	145_8		552	20.594
TS	248_8	36.956 ==>	MIN	143_8	550	20.331	<-->	MIN	229_8		636	31.453
TS	250_8	37.001 ==>	MIN	143_8	550	20.331	<-->	MIN	229_8		636	31.453
TS	252_8	37.557 ==>	MIN	131_8	538	16.849	<-->	MIN	242_8		649	35.021
TS	253_8	37.798 ==>	MIN	16_8	423	-5.378	<-->	C2H6 + C4CoHO4		681 +	61	-21.894
TS	254_8	37.802 ==>	MIN	177_8	584	24.605	<-->	MIN	187_8		594	25.572
TS	256_8	37.982 ==>	MIN	133_8	540	17.877	<-->	MIN	242_8		649	35.021
TS	257_8	38.056 ==>	MIN	16_8	423	-5.378	<-->	C2H6 + C4CoHO4		681 +	61	-21.893
TS	258_8	38.079 ==>	MIN	6_8	413	-9.324	<-->	MIN	80_8		487	4.737
TS	260_8	38.309 ==>	MIN	38_8	445	-1.348	<-->	MIN	74_8		481	3.961
TS	262_8	38.373 ==>	MIN	63_8	470	2.837	<-->	MIN	183_8		590	24.781
TS	263_8	38.615 ==>	MIN	145_8	552	20.594	<-->	C2H6 + C4CoHO4		681 +	61	-21.878
TS	266_8	39.062 ==>	MIN	155_8	562	21.488	<-->	MIN	247_8		654	37.602

4. Rate coefficients

Rate coefficients employed in the KMC simulations. The units are dm^3 mol^{-1} s^{-1} for a bimolecular process or s^{-1} for a unimolecular process. The first column below is the rate, and columns 2-5 are the Klabels, explained above, for re1, re2, pr1 and pr2, which are the reactants and products of a general process of the type: re1 + re2 $\rightarrow$ pr1 + pr2.

Rate	re1	re2	pr1	pr2
4.48732e+10	674	2	4	0
2.13971e-11	4	0	674	2
729359.60122695612	6	0	7	0
2916979528603.3159	7	0	6	0
6.0634448854017O755E-004	4	0	7	0
19327810398.427834	7	0	4	0
1.51005186335905102E-004	4	0	8	0
660208658739.89844	8	0	4	0
3.62193781753826786E-005	4	0	7	0
1153154513.4771247	7	0	4	0
3.49629190275299317E-006	4	0	8	0
15286111978.597960	8	0	4	0
1.79306711363525619E-007	4	0	12	0
14523742720.915796	12	0	4	0
903815110.52919173	8	0	12	0
16724592245.919584	12	0	8	0
2.01622667053596157E-007	4	0	6	0
1.6050707911676743	6	0	4	0
4.48732e+10	7	1	23	0
8.12466	23	0	7	1
4.48732e+10	677	2	23	0
7.44879	23	0	677	2
4.48732e+10	676	2	13	0
1.15216e-07	13	0	676	2
4.48732e+10	4	1	15	0
405251	15	0	4	1
4.48732e+10	675	2	15	0
3.1688e-05	15	0	675	2
2457814144.45	3	55	34	0
2851648927.96	3	56	32	0
2119215577.14	3	57	34	0
21094413.8259	3	58	32	0
14780571.6412	3	59	32	0
1.82365940832	1	4	53	0
1.58742744839	1	4	54	0
53490120.659163944	13	0	15	0
1289645409.6804581	15	0	13	0
681.75000702221439	15	0	23	0
219487255433.41705	23	0	15	0
0.39146181606643898	13	0	15	0
9.4381341439126274	15	0	13	0
11235079112212.762	34	0	3	55
1060807897352.4700	32	0	3	56
1872458876919.8389	34	0	3	57
4.96372454515137574E-004	13	0	28	0
190104392027.83023	28	0	13	0
7894272626.0901775	32	0	3	58
5498352333.0724239	32	0	3	59
6.90405192804985140E-005	15	0	23	0
22565.032438966438	23	0	15	0
3695317335.5508733	34	0	53	0
7443801776484.0723	53	0	34	0
3447237668.6505251	34	0	54	0
3861737814918.6909	54	0	34	0
1645873007209.8643	53	0	1	4
792579845033.00085	54	0	1	4
1.49016389051967486E-006	15	0	34	0
227942044.68341875	34	0	15	0
10884878034.067144	51	0	54	0
126616131009.05394	54	0	51	0
1.87850141217069087E-008	13	0	36	0
79059374.695796639	36	0	13	0
1.29906749734010157E-008	13	0	28	0
4975264.7339423336	28	0	13	0
7202988.3539759722	32	0	34	0
16498617.012905639	34	0	32	0

2947570.5418888363	32	0	34	0
6751480.7881513443	34	0	32	0
5641258.0714690825	34	0	47	0
140952888.08590439	47	0	34	0
4.48732e+10	4	2	60	0
0.0121751	60	0	4	2
6061601089.96	2	4	63	0
8.90745868843834457E-003	60	0	62	0
5184599874267.4121	62	0	60	0
1177896527999.4878	63	0	2	4
7.75839960911279755E-004	60	0	61	0
127698891.33171685	61	0	60	0
8.59759712117002986E-005	60	0	61	0
14134334.443430414	61	0	60	0
1.33945705438757974E-006	60	0	66	0
3356642201765.3892	66	0	60	0
349324509.43285996	63	0	66	0
1220104492936.7786	66	0	63	0
4.48732e+10	679	2	69	0
1.12106e-07	69	0	679	2
4.48732e+10	678	2	67	0
0.000127329	67	0	678	2
8.45729e+12	4	3	67	0
8.81389e+12	67	0	4	3
98115556.8321	3	6	73	0
59240302904.357208	67	0	69	0
2060442031002.7690	69	0	67	0
304144116.44302696	67	0	69	0
10578462470.274122	69	0	67	0
1444673521966.8354	73	0	3	6
20857009384.803257	73	0	77	0
4263592931570.5278	77	0	73	0
3.06168472054433498E-003	67	0	73	0
362704012.99974614	73	0	67	0
4.27508078591343991E-003	67	0	73	0
506449585.27056789	73	0	67	0
3.92878246356087677E-003	67	0	80	0
14753753050109.609	80	0	67	0
4194.7163750881846	72	0	81	0
3279308361416.5098	81	0	72	0
681847066005.43164	81	0	77	0
1005722399.4484817	77	0	81	0
3.17007527647616893E-005	67	0	81	0
510092906807.78448	81	0	67	0
1.54333637761944324E-005	67	0	73	0
1826147.1832885083	73	0	67	0
38507.291255644908	73	0	77	0
7862329.8629961787	77	0	73	0
70022.278844497414	73	0	85	0
12943881047128.996	85	0	73	0
4.48732e+10	680	2	87	0
2229.23	87	0	680	2
4.48732e+10	13	2	86	0
871.911	86	0	13	2
206941228.843	2	13	86	0
371.434624411	2	13	143	0
51124193.683237471	86	0	91	0
43768511963.935059	91	0	86	0
19773978.908650465	87	0	91	0
38035586077.384209	91	0	87	0
3689127313.8001566	91	0	95	0
845766804428.19751	95	0	91	0
1099025.4680219695	87	0	95	0
485229843542.22772	95	0	87	0
71982.561507118357	87	0	91	0
138459686.18267578	91	0	87	0
8587.5287566186998	87	0	91	0
16518258.197974171	91	0	87	0
614.95158386371475	87	0	95	0
271183937.72708631	95	0	87	0
692834.14013037272	92	0	98	0
29867869026.136368	98	0	92	0
105.50588127388122	86	0	2	13
29011.234748641113	92	0	98	0
1250665505.0166211	98	0	92	0
7607.9652315214380	92	0	104	0
209835518654.04727	104	0	92	0

3686.3171847510926	92	0	104	0
101672425523.28117	104	0	92	0
1094.0388622433973	92	0	97	0
48970102.205515891	97	0	92	0
356.69154371902357	92	0	105	0
7566969464.4503031	105	0	92	0
6.17909553094790447E-003	87	0	91	0
11.885613592091223	91	0	87	0
1.21107629254887592E-002	87	0	95	0
5340.6552079734065	95	0	87	0
2.1391067713512069	91	0	105	0
81138512.120172217	105	0	91	0
0.29471019939516224	91	0	105	0
11178659.899457946	105	0	91	0
3.14387609770645712E-002	91	0	142	0
3206009347081.0532	142	0	91	0
1558974792514.0056	142	0	143	0
1753853404371.5483	143	0	142	0
4.99126285523000291E-006	87	0	117	0
179546649.58920419	117	0	87	0
717703038720.51221	143	0	2	13
2.07927806749297434E-006	87	0	117	0
74796183.132590815	117	0	87	0
4.15807244138323914E-002	95	0	161	0
4316602416684.3145	161	0	95	0
1.48522e+09	4	681	162	0
8.81389e+12	162	0	4	681
2560073944.08	3	13	168	0
19129506.8606	3	13	168	0
1169803681626.6956	168	0	3	13
226223527536.48071	168	0	3	13
7547155058.2276983	168	0	173	0
3583737660369.8828	173	0	168	0
2812726091.6236725	168	0	173	0
1335612206862.9978	173	0	168	0
101452569.87915063	168	0	173	0
48231714421.778191	173	0	168	0
38609599.327036858	168	0	173	0
18333620296.995190	173	0	168	0
2.88929937150345859E-002	162	0	173	0
12161400932.727701	173	0	162	0
1.64388884335733322E-002	162	0	168	0
14554385.184665246	168	0	162	0
5.13488812575399207E-003	162	0	173	0
2161334815.5578094	173	0	162	0
210470.93109986864	171	0	180	0
5799594.5356875435	180	0	171	0
178446.04154664528	180	0	196	0
13255578757308.510	196	0	180	0
435.71853252333841	173	0	196	0
394024673733.43945	196	0	173	0
249.20227275999412	178	0	173	0
8.7774202638581009	173	0	178	0
6775621.9046262037	192	0	197	0
1079644148.0208931	197	0	192	0
2.09609340809194781E-012	162	0	197	0
125324861.70710778	197	0	162	0
779062.12264226074	192	0	197	0
124137661.97331154	197	0	192	0
53274995.161052123	197	0	217	0
178274760337.33313	217	0	197	0
0.15094038326270845	173	0	197	0
21440800.062607162	197	0	173	0
7862318016.1839294	207	0	217	0
57985112935.935898	217	0	207	0
3.90520491025472469E-002	180	0	206	0
89197437.374516547	206	0	180	0
7.58205108901486538E-004	180	0	206	0
1729730.9170854071	206	0	180	0
4.48732e+10	704	2	285	0
0.00316259	285	0	704	2
4.48732e+10	7	703	285	0
6.98298e+07	285	0	7	703
4.48732e+10	702	2	242	0
0.000268795	242	0	702	2
4.48732e+10	4	703	242	0
8.0133e+08	242	0	4	703

4.48732e+10	700	2	229	0
0.00702674	229	0	700	2
4.48732e+10	699	2	257	0
2.17999e-10	257	0	699	2
4.48732e+10	698	2	220	0
1829.71	220	0	698	2
4.48732e+10	697	2	273	0
1.40327	273	0	697	2
4.48732e+10	696	2	245	0
6818.54	245	0	696	2
1.7754e+09	4	703	245	0
8.81389e+12	245	0	4	703
4.48732e+10	694	2	223	0
0.446754	223	0	694	2
4.48732e+10	4	703	223	0
37168.3	223	0	4	703
4.48732e+10	692	2	221	0
4.47139e+06	221	0	692	2
4.48732e+10	691	2	243	0
70313.7	243	0	691	2
3.27643e+09	4	703	243	0
8.81389e+12	243	0	4	703
4.48732e+10	689	2	267	0
0.0263836	267	0	689	2
4.48732e+10	81	2	289	0
1.99424e+08	289	0	81	2
4.48732e+10	688	2	281	0
1416.74	281	0	688	2
4.48732e+10	687	2	282	0
0.472448	282	0	687	2
1.27189e+10	6	703	282	0
8.81389e+12	282	0	6	703
4.48732e+10	685	2	218	0
242962	218	0	685	2
4.48732e+10	72	2	222	0
8340.38	222	0	72	2
1.55645e+14	4	703	222	0
8.81389e+12	222	0	4	703
4.48732e+10	72	2	246	0
1.19801e+09	246	0	72	2
1.11763e+09	4	703	246	0
8.81389e+12	246	0	4	703
4.48732e+10	73	2	237	0
8134.58	237	0	73	2
1.09951334562e-05	3	60	237	0
3907589.02544	3	61	237	0
350522879.432	3	393	294	0
56.6413448528	2	394	296	0
0.014820079087	2	67	237	0
2270492492.23	2	395	222	0
214855879.813	2	73	237	0
366286379.592	2	396	310	0
81011116.0644	2	73	237	0
15772293.6418	2	397	310	0
1271137355.94	2	398	243	0
5379.11262711	703	6	289	0
2973.39028107	703	6	326	0
1083384.84686	2	401	243	0
4831837729.59	2	80	346	0
0.00718489406089	2	69	254	0
2066905643.54	2	80	254	0
4.83404582938e-07	2	67	312	0
9972460.73238	2	402	273	0
1.83360251206e-07	2	67	361	0
41.3107653059	703	6	326	0
41.132826059	703	6	326	0
0.000129446097638	2	405	296	0
4.79345178829e-09	3	61	361	0
6.86807822831e-21	3	60	312	0
2.67805416325	703	6	326	0
584.248954392	2	407	296	0
5.15669894951e-11	3	61	312	0
4.67725961821e-24	3	60	242	0
2.10050964328e-24	3	60	289	0
5.98112496618e-13	3	61	221	0
645118574.41157281	221	0	222	0
2872412275.5345397	222	0	221	0

91083928.426494673	220	0	229	0
48797064125.143585	229	0	220	0
222480029.29772037	223	0	246	0
8527799644316.8281	246	0	223	0
216217088.78551742	223	0	246	0
8287737189988.0771	246	0	223	0
158499884.67557794	223	0	222	0
43625076.350963280	222	0	223	0
230352906605.42059	237	0	3	60
245810996748.60080	237	0	3	61
48575820.334787466	223	0	245	0
11707106385238.527	245	0	223	0
1585066.1791535551	220	0	229	0
848169459.44317913	229	0	220	0
8396419.9157777596	222	0	243	0
664777297600.19202	243	0	222	0
1706949.6737251626	222	0	246	0
237433893170.79831	246	0	222	0
16273135094.702875	237	0	254	0
1085857919075.8438	254	0	237	0
3276907061.1004305	237	0	254	0
218918571900.02322	254	0	237	0
9236446.2318637706	229	0	242	0
4145477820.0521150	242	0	229	0
5236422.5466957549	229	0	242	0
2352995424.3002467	242	0	229	0
1481648374.3113384	246	0	254	0
10411358698.802164	254	0	246	0
147714.86746902455	229	0	242	0
66297003.350953840	242	0	229	0
94311287.087749377	246	0	254	0
662713675.01256990	254	0	246	0
4372263.1633306080	237	0	254	0
292094738.31202334	254	0	237	0
22309632.557239175	243	0	273	0
164068921601.09085	273	0	243	0
1.4335301477055973	219	0	261	0
14735775304.116379	261	0	219	0
18.582015883263942	220	0	222	0
119.35660180447364	222	0	220	0
66.312006503464161	222	0	246	0
9234871.8875480667	246	0	222	0
4122.5255184260013	229	0	242	0
1852463.8901683739	242	0	229	0
1.31239212323490720E-003	218	0	222	0
4.0992495037044065	222	0	218	0
6.5191297906938228	223	0	282	0
219279098949.48843	282	0	223	0
53834.043689059719	242	0	285	0
635127777824.87378	285	0	242	0
89004.557545518081	243	0	281	0
68985216296.345383	281	0	243	0
64659083.381900333	261	0	277	0
1450856680.3397453	277	0	261	0
36552.229150191488	243	0	273	0
268491823.66404843	273	0	243	0
26967977.898755178	261	0	277	0
604403046.28418684	277	0	261	0
0.14148911531318792	222	0	282	0
17291130637.071594	282	0	222	0
8771523.4683486298	260	0	261	0
14000440.195632661	261	0	260	0
4893223782.5588284	281	0	282	0
9744725824.1446190	282	0	281	0
5225.0993482373869	243	0	257	0
900130.07672213751	257	0	243	0
44608.433335918031	254	0	289	0
845028355223.22791	289	0	254	0
13381560.022947453	267	0	282	0
3739545570.9696431	282	0	267	0
2085.4277921923904	243	0	246	0
3668.1900893509533	246	0	243	0
1736442441709.1267	294	0	3	393
1.2709406799630893	229	0	242	0
571.09936752473129	242	0	229	0
2163971658717.9766	296	0	2	394
4.37953523340159009E-002	223	0	297	0

3519487913396.7920	297	0	223	0
83.186396048312787	237	0	2	67
78.568827924703498	237	0	242	0
200.78661542505580	242	0	237	0
6.40097385705919869E-003	222	0	2	395
1.14032875604005747E-002	223	0	297	0
916392507490.93298	297	0	223	0
3.01776853953698550E-003	222	0	296	0
514735037816.67017	296	0	222	0
3.74318142776292799E-004	220	0	243	0
190.36036356677675	243	0	220	0
31854.797011355724	256	0	303	0
3160561718101.1611	303	0	256	0
0.16381447603685084	229	0	242	0
73.610314887991024	242	0	229	0
1.80067026935275439E-003	222	0	282	0
220056679.22051948	282	0	222	0
10.211610336168141	237	0	2	73
2664216770485.5371	310	0	2	396
8.05692793601674254E-005	220	0	229	0
4.31126491886489743E-002	229	0	220	0
26.603558881386231	243	0	289	0
6228923338.4909964	289	0	243	0
2.15331972925326026E-004	222	0	243	0
17.048671753448957	243	0	222	0
2.11020411447383538E-004	222	0	289	0
3911827927.5577950	289	0	222	0
1.9023466261582296	237	0	294	0
14280365713.422998	294	0	237	0
17.307232940721455	246	0	299	0
37702827730.278053	299	0	246	0
7.42862982374451353E-003	229	0	320	0
1971706032646.8179	320	0	229	0
1.05340890237468969E-005	220	0	308	0
423180806371.56995	308	0	220	0
0.80771682289939972	237	0	2	73
5.44890898342141367E-005	222	0	289	0
1010101069.8381342	289	0	222	0
115064386622.78954	310	0	2	397
4.44702689930893936E-004	226	0	277	0
79397.746395128139	277	0	226	0
1.5250632774662234	243	0	2	398
67523550045.172729	310	0	330	0
2549478315625.6128	330	0	310	0
1.1724522411077782	243	0	289	0
274516472.04284781	289	0	243	0
4.1029490922002783	254	0	289	0
77723344.774322197	289	0	254	0
141670090.32318696	289	0	703	6
4.36104958226365047E-004	229	0	289	0
97043106.223681584	289	0	229	0
7242877594.1719770	312	0	346	0
2625252776666.3311	346	0	312	0
53750744386.147087	326	0	703	6
0.56650767317378359	246	0	289	0
75408858.771144331	289	0	246	0
5.00257194145919126E-002	237	0	312	0
5020705581.4889488	312	0	237	0
0.21414991800154420	243	0	326	0
34363854857.641426	326	0	243	0
2.0228356605475453	254	0	326	0
26261868850.628597	326	0	254	0
0.16114782445412823	243	0	2	401
1.85684090504515883E-002	237	0	294	0
139221934.13150382	294	0	237	0
5895314973.0129652	308	0	349	0
12821263938886.293	349	0	308	0
4.63692487283162980E-002	243	0	326	0
7440703914.4709768	326	0	243	0
262939481282.94070	346	0	2	80
4.41236339796156344E-007	222	0	336	0
303643601313.42377	336	0	222	0
0.35950012366812606	254	0	289	0
6810097.9005065942	289	0	254	0
3.78895723250569384E-009	219	0	233	0
1.54090518886960184E-004	233	0	219	0
2.99024938855860466E-007	222	0	353	0

6392283675745.6104	353	0	222	0
0.27902300655076229	254	0	2	69
2.15767551403783682E-002	243	0	329	0
63517254545.004555	329	0	243	0
0.20625092856278643	254	0	2	80
271515193.08770961	312	0	2	67
1.71964062630348847E-007	222	0	223	0
6.24041882330780308E-007	223	0	222	0
87.949291405918544	273	0	2	402
1.05295683754258601E-002	243	0	326	0
1689641363.1320515	326	0	243	0
1.10135317123550850E-005	229	0	349	0
1800366359716.1677	349	0	229	0
1.53079720627976656E-006	226	0	260	0
7.6312283479439245	260	0	226	0
1574603971.1636171	320	0	352	0
1461964421456.8760	352	0	320	0
2895604160920.2651	361	0	2	67
6.38291481714589134E-008	222	0	256	0
0.92130291876143611	256	0	222	0
747909114.19392872	326	0	703	6
745244360.86316323	326	0	703	6
1.28795997431119941E-003	239	0	345	0
72277085591.812210	345	0	239	0
6.57073046866277991E-003	246	0	299	0
14313964.558667021	299	0	246	0
119.54826637519243	277	0	342	0
50993160363.775719	342	0	277	0
6.15795700234466801E-004	237	0	361	0
1727776088878.9873	361	0	237	0
108.56500298026076	277	0	294	0
4402519.1909227744	294	0	277	0
4952853.1878052354	296	0	2	405
143076.06896909609	286	0	336	0
19167509765.743793	336	0	286	0
1.92552884772238656E-003	243	0	254	0
2.37995620879280223E-002	254	0	243	0
845172414134.26025	361	0	3	61
42.756343453037445	275	0	370	0
3934039740805.0605	370	0	275	0
5.61687178465737538E-003	254	0	326	0
72922337.221701130	326	0	254	0
29.469768033151876	277	0	358	0
173092492334.46777	358	0	277	0
222059649774.91418	353	0	296	0
1771816.8431364761	296	0	353	0
14202201.304588897	312	0	3	60
744986.72246081952	291	0	313	0
32998158.073257856	313	0	291	0
2.17222901792638544E-004	239	0	277	0
23.873671489547394	277	0	239	0
3.83381278848740495E-003	254	0	289	0
72625.018362101589	289	0	254	0
1.83238283650023455E-004	239	0	277	0
20.138625126853054	277	0	239	0
6.17477149367093997E-003	254	0	299	0
1916556.4872911349	299	0	254	0
2.29828364648470879E-004	242	0	285	0
2714.7162378797148	285	0	242	0
7251286.1647268720	312	0	346	0
2628300546.4111590	346	0	312	0
4.57079526335881811E-009	222	0	246	0
6.35790106159078716E-004	246	0	222	0
48521553.111840792	326	0	703	6
5.82681229275217520E-010	220	0	319	0
128308067.23011957	319	0	220	0
1.86043510704094672E-007	229	0	254	0
2.18541480054106714E-003	254	0	229	0
3.18150428482831191E-002	256	0	339	0
2509053416.6179261	339	0	256	0
2.20779837736450544E-004	246	0	243	0
1.25367805728754991E-004	243	0	246	0
1.15075526929436789E-004	243	0	330	0
213099980.64420849	330	0	243	0
4.90586129614517817E-005	242	0	285	0
578.78780225661330	285	0	242	0
187221.36571080718	296	0	2	407

1.7075570578416517	277	0	342	0
728356282.53731692	342	0	277	0
8.42219068811959584E-005	246	0	336	0
416178101.47796184	336	0	246	0
20743396958.347775	361	0	346	0
268623346.19224429	346	0	361	0
21727.307523745396	291	0	294	0
29470.637304193064	294	0	291	0
9492968177.8978710	361	0	346	0
122932270.07918814	346	0	361	0
1.91051341642552703E-008	229	0	298	0
115441.18984772044	298	0	229	0
325327.74537105177	312	0	3	61
265445892655.93756	379	0	358	0
3320886406.2583871	358	0	379	0
1.28908935767189302E-004	254	0	326	0
1673586.0608051925	326	0	254	0
0.30959335094940654	275	0	303	0
160848.07750214660	303	0	275	0
8.72484513830258382E-006	243	0	369	0
16538410585.208981	369	0	243	0
0.26091053578336509	277	0	313	0
345509.96338516619	313	0	277	0
2.84935912847124991E-006	242	0	309	0
235896.86039186720	309	0	242	0
1.78305122555595083E-006	242	0	309	0
147617.82108252204	309	0	242	0
1.49517104893392106E-006	242	0	309	0
123784.38107995261	309	0	242	0
2.05880835039956625E-011	222	0	386	0
722102743987.11218	386	0	222	0
2238235797.7857485	369	0	384	0
62766025275.603783	384	0	369	0
6.48081346290314689E-010	229	0	345	0
10653933.797184378	345	0	229	0
5.78129927221495821E-010	229	0	345	0
9492688.1716581937	345	0	229	0
2.50078963104020010E-007	242	0	3	60
100.56349088142694	289	0	364	0
551746100.25068510	364	0	289	0
4.09542267448260865E-007	243	0	273	0
3.01184513003097511E-003	273	0	243	0
3.84184142882324548E-012	222	0	353	0
82127398.284020528	353	0	222	0
5.44459777152376012E-013	220	0	362	0
98596621.810602888	362	0	220	0
55.473530943337551	289	0	3	60
5.75772776625251743E-013	221	0	3	61
3.86121268234899612E-011	226	0	392	0
569774786571.06165	392	0	226	0
4613.5934978367841	304	0	313	0
8300.3112335556179	313	0	304	0
2.70743885346921128E-005	257	0	386	0
63699573306.244003	386	0	257	0
135.45584007079924	294	0	358	0
19619495.643737253	358	0	294	0
3.09922250703611139E-008	242	0	309	0
2565.8291078874245	309	0	242	0
1.51677428180981605E-008	242	0	254	0
3.96509787088053644E-007	254	0	242	0
1.16104104198938507E-011	226	0	285	0
0.33762714390356396	285	0	226	0
1.11633919442118764E-011	226	0	306	0
1819.5870628104008	306	0	226	0
7.22043236583936722E-013	222	0	243	0
5.71669778764634388E-008	243	0	222	0
4.48732e+10	723	2	469	0
0.13546	469	0	723	2
5.42414e+13	721	722	469	0
8.81389e+12	469	0	721	722
4.48732e+10	720	2	459	0
2.74067e-05	459	0	720	2
4.48732e+10	719	2	521	0
5.33835	521	0	719	2
8.22876e+09	6	722	521	0
8.81389e+12	521	0	6	722
4.48732e+10	717	2	421	0

1797.34	421	0	717	2
4.48732e+10	716	2	487	0
0.0665785	487	0	716	2
3.25177e+07	4	722	487	0
8.81389e+12	487	0	4	722
4.48732e+10	714	2	408	0
4.54407e-08	408	0	714	2
4.48732e+10	713	2	413	0
0.555426	413	0	713	2
4.48732e+10	4	722	413	0
6.84052e+08	413	0	4	722
4.48732e+10	711	2	423	0
1.5166e-07	423	0	711	2
4.48732e+10	710	2	428	0
10669.5	428	0	710	2
2.0749e+12	4	722	428	0
8.81389e+12	428	0	4	722
4.48732e+10	708	2	450	0
0.0153909	450	0	708	2
1.81745e+10	4	722	450	0
8.81389e+12	450	0	4	722
4.48732e+10	706	2	484	0
33.2345	484	0	706	2
4.48732e+10	705	2	448	0
196826	448	0	705	2
132154.263472	3	87	448	0
5298940452.07	722	4	450	0
55523.1637634	3	87	448	0
4262965.96436	3	91	448	0
44873167286.5	722	4	445	0
44873167286.5	722	4	445	0
199938764.172	703	13	532	0
44873167286.5	722	4	527	0
1940436744.28	3	117	555	0
413827650.62	3	117	555	0
0.000218114986411	660	661	470	0
44.0409248746	662	663	470	0
405.711186737	664	703	538	0
4.57349858922e-22	681	60	550	0
5.72157493456e-11	681	61	550	0
1.1294063843e-22	681	60	550	0
7.39094886206e-12	681	61	550	0
0.0331362729152	3	98	413	0
6.86375555838e-26	681	60	550	0
8.77455213622e-17	681	61	423	0
6.46731920794e-17	681	61	423	0
3.38511642407e-17	681	61	552	0
825535355.64816153	413	0	428	0
298708553691.00934	428	0	413	0
623133288.19861722	408	0	423	0
284279740217.25397	423	0	408	0
530670845.85860074	408	0	423	0
242385644996.89725	423	0	408	0
510487623.10280752	413	0	428	0
184712887862.29755	428	0	413	0
446800656.02152741	413	0	428	0
161668639429.25528	428	0	413	0
390129659.88051569	413	0	428	0
141163023070.49918	428	0	413	0
231956800.30157509	413	0	428	0
83930360901.959518	428	0	413	0
15476598439.679420	428	0	413	0
42772391.480763547	413	0	428	0
6412009119.6404467	423	0	413	0
17565687.621187385	413	0	423	0
4198031621.1439786	423	0	413	0
11514191.240984218	413	0	423	0
90511351964.396652	448	0	3	87
256829858019.49054	450	0	722	4
1399984546.7651782	423	0	428	0
1389386120.5105479	428	0	423	0
547318037.51526451	428	0	450	0
100094896558.63542	450	0	428	0
24324552604.581619	448	0	3	87
2588699850.4326859	448	0	484	0
990133561660.72095	484	0	448	0
9989268.1875919364	421	0	459	0

16776746524.180830	459	0	421	0
2271138931.0174441	448	0	3	91
3076038.0428911326	421	0	459	0
5166135253.8741112	459	0	421	0
2978658.0853784638	428	0	487	0
252815910981.04056	487	0	428	0
1077095.7448628591	421	0	459	0
1808957568.7119462	459	0	421	0
128127785.21737181	445	0	722	4
113620240.42229486	445	0	722	4
131126986.87343024	448	0	484	0
50094201202.250313	484	0	448	0
152697118.48631138	450	0	484	0
38329037797.377312	484	0	450	0
232053722.82963273	459	0	469	0
2747370000.2552347	469	0	459	0
215552258.86760470	459	0	469	0
2552003054.6326718	469	0	459	0
215085.10592893633	428	0	487	0
18255514877.924828	487	0	428	0
65357192.737639196	459	0	469	0
773788019.59629440	469	0	459	0
8130982.1082042409	448	0	484	0
3109962042.6254606	484	0	448	0
8247902.4189133784	450	0	484	0
2072799515.8271616	484	0	450	0
1417797.1641761351	450	0	484	0
355886225.18500823	484	0	450	0
1991906.7523922301	459	0	469	0
23582922.041056819	469	0	459	0
1631029.7457794643	459	0	469	0
19310365.455193587	469	0	459	0
1327612.1365281623	459	0	469	0
15718091.963342754	469	0	459	0
2936654531.9280272	506	0	510	0
26956404734.361687	510	0	506	0
31819735373682.988	532	0	703	13
5645550048.1535444	510	0	532	0
23745763303104.070	532	0	510	0
2086446.2092193570	463	0	527	0
1550482735685.8811	527	0	463	0
724919604129.70557	527	0	722	4
8900.2065150823364	450	0	484	0
2236731.5734628742	484	0	450	0
14.087598684989661	421	0	428	0
30.201434833109804	428	0	421	0
11.237307099775293	421	0	428	0
24.119570147057974	428	0	421	0
3.5733159968748658	421	0	428	0
7.6697063699496875	428	0	421	0
117716.91580260351	478	0	506	0
7939786.4953200854	506	0	478	0
2.9608164738208451	428	0	450	0
541.48154886624968	450	0	428	0
1.7653998210089146	423	0	428	0
1.7520350593362739	428	0	423	0
107209.07505288473	487	0	521	0
5077068918.6439371	521	0	487	0
1.2472802118634156	423	0	428	0
1.2378378166778106	428	0	423	0
0.16184942283523360	421	0	524	0
3135782188.2764721	524	0	421	0
6818369497184.7764	555	0	3	117
0.10851180982991690	428	0	413	0
2.99892100219634791E-004	413	0	428	0
4430.8258711219914	487	0	521	0
209579829.83765569	521	0	487	0
1167306372100.5137	555	0	3	117
2.67491780642728147E-002	428	0	540	0
20951973588.071445	540	0	428	0
2.48174851628012345E-002	428	0	540	0
19462067574.580070	540	0	428	0
171.64171183894740	470	0	660	661
603243223.30784416	532	0	550	0
34783218832.595360	550	0	532	0
134565935731.00507	549	0	556	0
498098350105.14832	556	0	549	0

26.660424467970074	461	0	549	0
131871541132.47379	549	0	461	0
3.32687256985590447E-003	428	0	487	0
282.37088492789991	487	0	428	0
2.16780844692649691E-003	428	0	487	0
183.77563994492488	487	0	428	0
24.361457489135645	470	0	662	663
21145.974272258682	510	0	550	0
5128435400.1819458	550	0	510	0
271771208.69088584	538	0	664	703
7.6883307643817957	469	0	541	0
1848958083.5635879	541	0	469	0
1022925750.0297433	550	0	681	60
4.58359211102500707E-007	408	0	567	0
68170262244.652695	567	0	408	0
0.12161218436483966	459	0	469	0
1.4380996866898048	469	0	459	0
386523674.05395681	550	0	681	61
248882147.99149248	550	0	681	60
0.33008051085368045	469	0	541	0
79286305.273356229	541	0	469	0
3.09780107416616221E-003	448	0	555	0
755190520.03177023	555	0	448	0
1.42029252156293572E-003	445	0	552	0
176801624.29108074	552	0	445	0
207.38606821873384	510	0	587	0
40204643269.928757	587	0	510	0
50057589.395106636	550	0	681	61
2.67316042674199360E-002	469	0	484	0
0.13246450262960696	484	0	469	0
0.11210582603619733	478	0	587	0
13439625512.866541	587	0	478	0
3.26769948402392402E-004	445	0	524	0
32367.074457282444	524	0	445	0
2.29771535952663929E-004	445	0	552	0
28568534.891678326	552	0	445	0
13542119670.006599	584	0	590	0
2779459506.3159285	590	0	584	0
1.29614779935266732E-002	469	0	484	0
6.42285930493373403E-002	484	0	469	0
9.53647038006385757E-004	459	0	484	0
5.59487203187625862E-002	484	0	459	0
2.39847644338361211E-009	408	0	506	0
2.0306478678292041	506	0	408	0
7.66467904031727376E-002	487	0	562	0
51996947.696644828	562	0	487	0
5.09462174691418732E-003	461	0	478	0
2.19652812772245035E-002	478	0	461	0
6.36454190345817371E-005	445	0	552	0
7913322.9746939670	552	0	445	0
7.16395151269528765E-005	448	0	550	0
2473030.2263751156	550	0	448	0
2.41030493123866408E-004	459	0	484	0
1.41408163719272534E-002	484	0	459	0
302783001714.66095	627	0	633	0
3043974238324.0166	633	0	627	0
256568203971.80209	625	0	633	0
2282429541481.1064	633	0	625	0
724412.06228649383	550	0	625	0
251132009015.79037	625	0	550	0
1.28350322212241463E-002	487	0	612	0
25080832111.332340	612	0	487	0
2.50254460799942847E-005	450	0	636	0
1109088919969.3313	636	0	450	0
6.21481952243238631E-003	484	0	540	0
106167.22776065735	540	0	484	0
5.85889879510191143E-008	421	0	590	0
242497226.28631246	590	0	421	0
2.74275001220144360E-010	413	0	3	98
21201.491208239280	538	0	546	0
2167414.0414350303	546	0	538	0
3.04165045303137768E-008	421	0	590	0
125892565.13685560	590	0	421	0
2.87965650374028839E-008	421	0	590	0
119045986.09420800	590	0	421	0
2.82390715393477385E-003	484	0	587	0
203972336.88012198	587	0	484	0

152802.41596844478	550	0	681	60
4.9738298252749653E-006	450	0	545	0
308725.45551692677	545	0	450	0
4.4001743889419036E-006	450	0	636	0
195008897947.66010	636	0	450	0
3044453.0924386280	551	0	645	0
4306343129075.6484	645	0	551	0
1273423.7111352668	561	0	566	0
611618.93892878399	566	0	561	0
3.9669148308340322E-007	445	0	552	0
49322.447311465665	552	0	445	0
703306.71571057977	561	0	566	0
329060.31114335405	566	0	561	0
5.1608479602894377E-005	470	0	590	0
5634724.9751533736	590	0	470	0
2.5421647203786880E-007	445	0	552	0
31645.512804914171	552	0	445	0
8938.4538165806280	550	0	587	0
7223499.5603611860	587	0	550	0
4.2781365086121463E-012	413	0	550	0
6421.2160345215398	550	0	413	0
4878.1762598934647	550	0	625	0
1691117898.5803330	625	0	550	0
2.0378456558196881E-005	470	0	590	0
2222318.1717442358	590	0	470	0
33519.643942553223	555	0	645	0
231226581540.76685	645	0	555	0
4.6053218751912972E-010	421	0	566	0
101691.26749119957	566	0	421	0
8.0837497643913282E-008	445	0	566	0
91256.358755039444	566	0	445	0
6.8907930185590333E-006	469	0	527	0
9.9666375744024656	527	0	469	0
3.4082590516612610E-010	421	0	566	0
75258.623022389598	566	0	421	0
5.5373169120023754E-008	445	0	552	0
6884.7967069084643	552	0	445	0
3.5910155146119604E-008	445	0	552	0
4464.8721000361311	552	0	445	0
1617.1543726720731	550	0	636	0
3159784980.3659692	636	0	550	0
1532.8496944518354	550	0	636	0
2995060659.3509402	636	0	550	0
43.963904168198439	538	0	649	0
215913610714.48215	649	0	538	0
1.4425539803582154E-010	423	0	681	61
1338673.6431317935	584	0	594	0
4224892.5287594385	594	0	584	0
90.196259117062581	540	0	649	0
130065453644.21802	649	0	540	0
1.0612583873790007E-010	423	0	681	61
2.8322041844786543E-013	413	0	487	0
8.6980177579614022E-006	487	0	413	0
7.1117461929917647E-009	445	0	481	0
7.8743488556576702E-006	481	0	445	0
1.2772402411342064E-006	470	0	590	0
139286.02440766428	590	0	470	0
614.40539059775722	552	0	681	61
1832.2264987206338	562	0	654	0
1553420262970.1199	654	0	562	0

5. Intermediates

In the following, the energies, Cartesian coordinates and vibrational frequencies of the optimized intermediates are listed. The structure of the data of each intermediate is the following:

1st line:	MINX_Y (this is a label explained above)
2nd line:	Number of atoms N
3rd line:	Electronic energy (E), zero-point energy (ZPE), and Gibbs free-energy correction (Gcorr)
	E and Gcorr are given in Hartrees, while ZPE is given in kcal/mol.
4th-$(4 + N - 1)$th line:	The Cartesian coordinates given in Å.
Last line:	Vibrational frequencies in cm^{-1}.

```
MIN01_1
8
E= -1723.23815027 ZPE=    19.66 Gcorr   -0.022129000
H 0.000013 -1.739850 -0.383039
Co 0.000004 -0.236283 -0.299056
C -1.684149 -0.598753 0.049036
C 1.684162 -0.598730 0.049037
O -2.725762 -0.924496 0.408948
O 2.725780 -0.924460 0.408949
C -0.000014 1.509267 -0.050522
O -0.000031 2.630054 0.203634
FREQS=   60   75   98  173  290  336  435  483  486  504  539  584  587  757 1943 2101 2117 2186
MIN02_1
8
E= -1723.22150060 ZPE=    19.43 Gcorr   -0.022889000
H 0.000487 0.001017 1.159572
Co 0.000117 0.000154 -0.269500
C 1.679149 0.557758 -0.048228
C -0.356454 -1.732914 -0.048271
O 2.723060 0.904456 0.291094
O -0.578112 -2.810320 0.291036
C -1.322823 1.174985 -0.048296
O -2.145306 1.905344 0.291083
FREQS=   65   65   88  253  253  311  427  428  461  491  514  514  613  614 2091 2091 2130 2182
MIN03_1
8
E= -1723.22104928 ZPE=    21.83 Gcorr   -0.018875000
H 3.053723 0.025599 0.000390
Co 0.202014 -0.098668 -0.000106
C 1.985387 -0.228915 0.000180
C -1.418700 -0.834071 -0.000069
O 1.594994 -1.411884 0.000005
O -2.458404 -1.323782 0.000162
C -0.225486 1.560877 -0.000019
O -0.456004 2.692053 0.000072
FREQS=   29   88  108  114  361  376  432  468  505  556  627  680  812 1215 1587 2091 2151 3070
MIN04_1
8
E= -1723.19975130 ZPE=    21.28 Gcorr   -0.019811000
H -0.000018 1.328677 1.778696
Co 0.000001 -0.022280 -0.455925
C -1.574429 -0.677762 -0.028437
C -0.000007 1.346382 0.669174
O -2.486585 -1.221368 0.426831
O -0.000001 2.358693 0.003521
C 1.574432 -0.677758 -0.028437
O 2.486588 -1.221363 0.426834
FREQS=   60   91   98  153  187  253  344  445  500  527  535  682  878 1272 1754 2058 2125 2928
MIN05_1
8
E= -1723.18404152 ZPE=    18.37 Gcorr   -0.026143000
H -0.206278 -1.720352 0.278338
Co -0.144652 -0.244832 -0.024371
C 1.492232 -0.699700 -0.002913
C -2.030220 -1.309809 0.135092
O 2.593637 -1.042755 -0.002499
O -2.099484 -0.150674 -0.089101
C 0.113270 1.499562 -0.060096
```

```
O 0.338371 2.617242 0.084996
FREQS=   47   63   93  103  196  243  314  383  444  499  534  543  609  703 1854 1964 2103 2158
MIN06_1
8
E= -1723.18661829 ZPE=    19.07 Gcorr   -0.023113000
H 0.022737 -0.952364 1.261238
Co 0.003826 0.087304 0.255740
C 1.484456 -0.751283 -0.049270
C -1.614859 -0.580068 -0.013707
O 2.362872 -1.451783 -0.308479
O -2.545623 -1.197358 -0.287247
C 0.817890 1.975408 -0.117358
O -0.348619 1.990492 -0.289801
FREQS=   68   90  113  125  275  323  362  401  438  461  505  553  629  773 1869 2092 2105 2157
MIN07_1
8
E= -1723.18474683 ZPE=    18.62 Gcorr   -0.024748000
H 0.417880 -1.694094 0.562437
Co -0.047508 -0.292628 0.255791
C 1.640428 -0.320486 -0.036482
C -1.442231 -1.156325 -0.857576
O 2.769305 -0.406914 -0.246300
O -2.050481 -0.953517 0.125156
C -0.373044 1.447312 -0.005594
O -0.479586 2.581935 -0.137719
FREQS=   75   80   94  120  178  229  360  399  405  472  547  563  590  816 1900 1926 2110 2161
MIN08_1
8
E= -1723.18494869 ZPE=    19.48 Gcorr   -0.022815000
H 0.757912 -1.642701 -0.000374
Co 0.190763 -0.177198 0.000082
C 2.062948 -1.204135 -0.000264
C -1.375407 -0.876807 0.000001
O 2.070290 0.007716 0.000099
O -2.422052 -1.360852 -0.000041
C -0.312132 1.499790 0.000103
O -0.668360 2.592382 -0.000169
FREQS=   32   84  100  130  323  404  414  434  508  555  562  633  653  920 1713 1899 2100 2161
MIN09_1
8
E= -1723.18263822 ZPE=    18.95 Gcorr   -0.024565000
H -0.097320 -1.779669 0.347892
Co 0.018386 -0.307848 -0.006587
C -1.647402 -0.614163 -0.005623
C 3.049115 -0.709445 -0.001822
O -2.769054 -0.885572 -0.006051
O 1.919362 -0.518313 -0.003354
C -0.060601 1.442199 -0.089388
O -0.206029 2.576387 0.060774
FREQS=   47   87  103  131  180  245  325  381  436  509  563  580  597  828 1891 2089 2115 2147
MIN001_2
14
E= -1801.88238749 ZPE=    56.50 Gcorr    0.030780000
H 0.505519 -0.000143 1.847870
Co 0.014435 -0.000018 0.122468
C -1.703329 0.000679 0.588637
C 0.031313 1.556788 -0.718936
O -2.813458 0.001148 0.889136
O 0.050173 2.492155 -1.393426
C 0.030056 -1.556887 -0.718833
O 0.048109 -2.492334 -1.393236
C 1.982965 -0.000780 0.191609
C 1.643379 -0.000478 1.656301
H 2.484470 -0.896148 -0.166985
H 2.485179 0.894105 -0.167202
H 1.975358 0.895357 2.183492
H 1.974825 -0.896367 2.183739
FREQS=   59   83   91  107  118  131  277  322  333  411  421  472  505  513  529  553  568  569  851  900 1006
1042 1141 1171 1390 1466 1523 1581 2078 2094 2153 2477 3094 3133 3159 3201
MIN002_2
14
E= -1801.87658542 ZPE=    56.35 Gcorr    0.027043000
H 1.614614 0.887317 1.986408
Co -0.057170 0.000053 0.044104
C -0.017021 -1.635978 -0.610576
C -0.027123 1.636918 -0.609187
O 0.001738 -2.622151 -1.206260
```

O -0.014456 2.623707 -1.204002
C -1.753112 -0.005527 0.587652
O -2.826826 -0.009132 0.998593
C 2.063490 0.003665 1.515055
C 1.920386 0.006219 -0.009616
H 1.629739 -0.889719 1.981807
H 3.123609 0.012387 1.803519
H 2.383118 0.889422 -0.452823
H 2.389135 -0.872012 -0.456338
FREQS= 29 49 77 92 103 104 159 177 313 402 405 469 498 503 534 548 578 581 790 949 955
985 1216 1248 1410 1501 1503 1509 2078 2097 2161 3013 3061 3079 3094 3144
MIN003_2
14
E= -1801.87608177 ZPE= 55.08 Gcorr 0.028737000
H 0.000002 -0.925219 -1.385991
Co 0.000001 -0.078488 -0.170113
C -1.497725 -0.998973 0.062577
C 1.497727 -0.998970 0.062586
O -2.447698 -1.643011 0.133934
O 2.447701 -1.643006 0.133949
C -0.000007 0.910324 1.331712
O -0.000012 1.530683 2.296890
C 0.699004 1.435327 -1.421051
C -0.698994 1.435328 -1.421056
H 1.242319 2.189998 -0.860465
H 1.244824 1.004433 -2.253405
H -1.244809 1.004434 -2.253413
H -1.242312 2.189998 -0.860474
FREQS= 66 75 87 106 122 128 343 350 387 430 443 478 484 500 511 550 560 720 758 833 850
981 989 994 1239 1264 1490 1575 2017 2108 2125 2167 3154 3162 3231 3253
MIN004_2
14
E= -1801.87384763 ZPE= 56.07 Gcorr 0.028660000
H 3.195985 0.450404 0.593644
Co -0.195119 0.015605 -0.178242
C -1.780496 -0.778535 -0.364717
C 0.658941 -1.369058 0.501906
O -2.786197 -1.284813 -0.596224
O 1.186334 -2.183414 1.122330
C -0.794931 1.572146 0.399085
O -1.126787 2.535203 0.935939
C 1.482089 0.882779 -0.667434
C 2.882581 0.327532 -0.448583
H 1.123849 0.690209 -1.710113
H 1.496322 1.965736 -0.527759
H 3.615460 0.863072 -1.066690
H 2.960689 -0.735752 -0.694453
FREQS= 47 75 85 95 103 143 220 290 315 337 404 438 492 504 514 529 579 584 651 878 1019
1055 1190 1282 1424 1444 1506 1513 2082 2099 2163 2826 3027 3087 3102 3123
MIN005_2
14
E= -1801.86962837 ZPE= 54.55 Gcorr 0.027054000
H 0.000006 -0.077828 -1.612480
Co 0.000011 0.026891 -0.146303
C -1.620513 0.607803 -0.579264
C 1.620572 0.607736 -0.579222
O -2.650598 0.973813 -0.941643
O 2.650677 0.973701 -0.941588
C -0.000042 -1.763707 -0.085899
O -0.000079 -2.913116 -0.112262
C -0.000014 1.294861 1.554276
C -0.000036 -0.029576 1.977467
H -0.913446 1.878582 1.574124
H 0.913390 1.878625 1.574110
H 0.916976 -0.491655 2.327113
H -0.917044 -0.491675 2.327096
FREQS= 56 70 89 98 115 130 283 322 362 410 454 460 475 476 518 518 549 679 685 748 844
982 997 1013 1239 1278 1491 1581 2043 2091 2102 2156 3164 3172 3241 3264
MIN006_2
14
E= -1801.86647258 ZPE= 54.33 Gcorr 0.025990000
H 0.917976 -2.473411 0.785975
Co 0.000001 -0.113370 -0.138193
C 0.000027 1.545690 0.500683
C 1.653773 -0.094389 -0.753739
O 0.000050 2.631441 0.881081
O 2.699777 -0.124915 -1.235664

C -1.653760 -0.094343 -0.753769
O -2.699760 -0.124841 -1.235706
C -0.000017 -1.940370 1.008487
C -0.000081 -0.882988 1.892539
H -0.000001 -1.124941 -1.226823
H -0.917967 -2.473447 0.785884
H 0.918625 -0.561130 2.371686
H -0.918849 -0.561165 2.371593
FREQS= 34 81 91 91 116 118 263 346 347 399 412 440 476 477 509 554 575 643 655 727 844 969 994 1015 1242 1305 1488 1599 2004 2091 2108 2156 3162 3171 3241 3264
MIN007_2
14
E= -1801.86239789 ZPE= 57.64 Gcorr 0.033937000
H -3.125851 -1.659845 0.308177
Co 0.236608 -0.024651 -0.061522
C -2.304941 -0.937363 0.187177
C 1.551991 -1.198489 0.003048
O -1.146611 -1.375125 0.118520
O 2.436331 -1.933846 -0.083742
C 1.245385 1.338477 0.086700
O 1.877297 2.289057 0.274885
C -2.536031 0.523179 0.125175
C -1.266158 1.187168 -0.439469
H -3.478490 0.735751 -0.403636
H -2.715633 0.840084 1.166370
H -1.221999 2.231679 -0.123637
H -1.324074 1.199369 -1.539271
FREQS= 43 63 96 110 148 276 327 373 396 449 468 498 538 588 616 643 736 825 903 942 1005 1142 1167 1237 1334 1396 1437 1472 1688 2068 2126 2997 3007 3027 3059 3116
MIN008_2
14
E= -1801.86039756 ZPE= 56.10 Gcorr 0.033413000
H 2.107424 1.723626 0.637849
Co -0.078716 -0.010607 -0.154968
C 1.745401 0.691334 0.541800
C -1.292351 -1.261742 0.181797
O 1.104999 0.130293 1.502829
O -2.087464 -2.077259 0.316358
C -0.964339 1.543234 -0.130718
O -1.613345 2.484173 -0.230500
C 1.882871 -0.026438 -0.684778
C 1.346721 -1.345924 -0.679739
H -0.576792 0.029572 -1.529797
H 2.244907 0.473380 -1.576894
H 1.522526 -1.963616 0.196501
H 1.283924 -1.877021 -1.623197
FREQS= 74 87 101 115 288 322 371 385 414 422 469 494 498 513 541 705 745 792 839 926 982 1012 1043 1084 1207 1328 1435 1498 1541 2045 2124 2164 3065 3154 3214 3248
MIN009_2
14
E= -1801.86012355 ZPE= 57.84 Gcorr 0.033752000
H -1.688261 0.449341 1.456580
Co 0.284156 0.054884 0.022918
C -1.714405 0.461432 0.361091
C 2.019393 0.094998 -0.039272
O -2.821610 -0.123457 -0.212217
O 3.172706 0.129992 -0.073148
C 0.027382 -1.656855 -0.012017
O -0.169110 -2.797154 -0.015903
C -1.099862 1.506940 -0.338422
C 0.112029 2.047908 0.151857
H 0.651878 2.745845 -0.478843
H -1.388876 1.646090 -1.379379
H 0.230426 2.236365 1.220259
H -3.000489 -0.961078 0.233307
FREQS= 40 62 80 99 227 289 304 352 401 411 438 463 471 526 562 583 620 735 884 953 980 996 1168 1201 1225 1304 1379 1517 1594 2066 2123 3069 3115 3164 3226 3834
MIN010_2
14
E= -1801.85907459 ZPE= 57.87 Gcorr 0.033859000
H -1.708710 0.442628 -1.464729
Co 0.278453 0.061247 -0.050713
C 0.074923 -1.662911 -0.003318
C -1.744458 0.437501 -0.374781
O -0.052610 -2.809849 0.051297
O -2.835086 -0.274077 0.054115
C 2.008819 0.142774 0.025792

O 3.159753 0.211118 0.089431
C -1.144471 1.464977 0.364294
C 0.067332 2.038709 -0.103626
H -2.888609 -0.230726 1.018733
H -1.428272 1.579310 1.412295
H 0.593079 2.721025 0.555669
H 0.164954 2.290248 -1.161626
FREQS= 39 62 91 100 212 285 312 365 397 414 435 465 473 528 561 586 617 737 869 954 966
992 1130 1225 1270 1297 1402 1511 1577 2071 2126 3108 3127 3147 3222 3809
MIN011_2
14
E= -1801.85108915 ZPE= 56.14 Gcorr 0.030005000
H 0.000645 2.615386 -0.531243
Co -0.000016 -0.049547 -0.077938
C 0.000236 1.574768 -0.906505
C 1.481307 0.285665 0.793090
O -0.000076 1.292889 -2.085279
O 2.359393 0.472499 1.519881
C -1.481722 0.285475 0.792667
O -2.359939 0.472146 1.519334
C 0.695885 -1.960461 -0.552732
C -0.695171 -1.960844 -0.552412
H 1.251547 -2.413145 0.262435
H 1.246468 -1.848922 -1.483309
H -1.246254 -1.849646 -1.482732
H -1.250203 -2.413787 0.263041
FREQS= 69 80 102 115 115 125 164 264 350 357 455 470 484 504 543 553 676 679 827 894 965
972 993 1241 1273 1280 1478 1571 1750 2067 2117 2963 3148 3155 3225 3247
MIN012_2
14
E= -1801.84595902 ZPE= 55.29 Gcorr 0.027012000
H 0.519782 0.006258 2.201837
Co 0.140121 0.184190 -0.147101
C -0.281052 -0.393645 1.528625
C -1.506845 -0.094872 -0.791109
O -1.202860 -1.030738 1.944850
O -2.508346 -0.253705 -1.332693
C 1.222886 -1.197271 -0.450865
O 1.900206 -2.082535 -0.738675
C 0.173626 2.203302 0.017092
C 1.483460 1.700382 0.109265
H -0.350678 2.562999 0.898289
H -0.165105 2.657826 -0.912484
H 1.997373 1.659764 1.065704
H 2.150902 1.768477 -0.747522
FREQS= 43 65 73 91 109 127 163 280 376 396 409 452 478 501 507 513 590 667 813 833 934
960 974 1226 1238 1251 1478 1554 1841 2091 2140 2811 3129 3139 3201 3223
MIN013_2
14
E= -1801.84073787 ZPE= 56.01 Gcorr 0.029950000
H 2.562461 -1.274356 -0.056785
Co 0.050370 -0.072886 -0.081508
C 0.700610 1.555020 0.182173
C 1.633617 -0.858472 -0.492699
O 1.081437 2.584288 0.534765
O 1.352175 -0.949614 -1.676879
C -1.675414 0.328881 -0.494837
O -2.747192 0.533675 -0.850932
C -0.140290 -0.705825 1.824891
C -0.509237 -1.756264 0.960174
H 0.824427 -0.732175 2.325555
H -0.901886 -0.145849 2.361396
H -1.556910 -2.011215 0.830803
H 0.164840 -2.595309 0.805897
FREQS= 62 80 88 108 122 153 191 272 376 417 423 434 482 514 525 535 633 743 836 883 949
982 1003 1230 1233 1254 1488 1563 1708 2091 2140 2956 3133 3142 3204 3227
MIN014_2
14
E= -1801.83731619 ZPE= 55.24 Gcorr 0.028851000
H 0.620013 -2.398936 -1.137861
Co 0.050993 0.058394 -0.387560
C -0.893599 -1.154918 0.732354
C 1.590848 0.612595 0.281557
O -0.682902 -1.558176 1.822684
O 2.581377 0.870909 0.803429
C -0.892251 1.547270 -0.075309
O -1.489992 2.525061 0.000679

C 1.075804 -1.406998 -1.213399
C -1.596319 -1.060465 -0.494107
H 2.133725 -1.506211 -0.961975
H 1.004622 -1.079764 -2.265492
H -1.541433 -1.942319 -1.128273
H -2.568506 -0.576649 -0.443194
FREQS= 63 75 92 100 135 164 278 295 364 407 432 448 460 477 528 545 568 594 700 767 870
875 986 1003 1263 1430 1435 1494 1994 2105 2153 2979 3092 3125 3136 3215
MIN015_2
14
E= -1801.83048458 ZPE= 55.20 Gcorr 0.023975000
H 1.121038 1.704701 0.931666
Co 0.423455 0.175106 0.000037
C 2.006292 -0.582914 -0.000096
C -0.370568 -1.344488 0.000123
O 3.041322 -1.090518 -0.000224
O -0.920045 -2.356220 0.000194
C -2.493176 0.541707 -0.000081
O -3.559129 0.050375 -0.000228
C -1.280005 1.050985 0.000072
C 1.418281 2.227966 0.000071
H 2.500466 2.343479 0.000049
H -1.263779 2.140760 0.000231
H 1.120985 1.704789 -0.931553
H 0.905877 3.189764 0.000130
FREQS= 6 66 87 103 108 147 219 264 284 353 379 420 431 442 485 488 524 544 574 621 721
1088 1270 1285 1398 1413 1518 1648 2090 2128 2189 2871 2951 3136 3154 3203
MIN016_2
14
E= -1801.82985952 ZPE= 53.20 Gcorr 0.023614000
H -0.714336 -0.562755 1.515751
Co -0.182115 -0.034637 0.199545
C 1.326922 -0.225868 1.117405
C -1.689445 0.903517 0.374658
O 2.269847 -0.370219 1.760130
O -2.674607 1.470886 0.545883
C -0.585594 -1.605723 -0.537243
O -0.831133 -2.606460 -1.045250
C 1.912214 1.225802 -1.559717
C 0.512557 1.023798 -1.094471
H 1.959619 1.065528 -2.648026
H 2.651253 0.585011 -1.074984
H 2.203274 2.277573 -1.412193
H -0.155493 1.687030 -1.658167
FREQS= 47 74 80 99 105 136 177 287 317 331 433 449 474 494 500 516 545 631 665 687 818
983 1003 1134 1356 1378 1479 1490 1899 2103 2112 2161 3000 3044 3066 3138
MIN017_2
14
E= -1801.83018240 ZPE= 53.80 Gcorr 0.024274000
H 0.512242 0.036604 -1.449291
Co -0.008168 0.001601 -0.077172
C -0.890949 1.455836 -0.635489
C -0.909335 -1.406120 -0.719216
O -1.342555 2.423577 -1.059455
O -1.373788 -2.340844 -1.199642
C -0.447704 -0.047901 1.672880
O -0.688475 -0.080272 2.793827
C 1.758392 -0.016542 0.292873
C 2.972825 0.002713 -0.574341
H 2.089218 -0.047205 1.340310
H 3.600901 0.866807 -0.305939
H 2.764350 0.033723 -1.644346
H 3.592990 -0.880769 -0.355164
FREQS= 38 72 85 91 109 120 167 313 329 355 425 460 463 465 507 531 543 642 684 828 850
988 1010 1140 1370 1385 1485 1486 2065 2109 2135 2166 2996 3039 3039 3147
MIN018_2
14
E= -1801.83877994 ZPE= 56.07 Gcorr 0.033420000
H -0.463385 0.000100 -1.584239
Co -0.079654 -0.000019 -0.172765
C -1.161946 -1.400446 0.040484
C 1.760209 -0.000396 0.267123
O -1.854773 -2.310456 0.085998
O 1.222044 -0.000686 1.456402
C -1.161274 1.400898 0.040441
O -1.853529 2.311356 0.085876
C 1.578162 1.204282 -0.493587

C 1.578076 -1.204534 -0.494415
H 1.925922 1.254255 -1.520298
H 1.546624 2.140993 0.052828
H 1.546573 -2.141769 0.051112
H 1.925626 -1.253618 -1.521239
FREQS= 68 89 97 122 289 346 380 387 433 434 490 492 496 523 540 701 726 837 845 872 970
981 989 1029 1082 1327 1429 1510 1513 2054 2144 2179 3161 3164 3259 3262
MIN019_2
14
E= -1801.82964920 ZPE= 53.85 Gcorr 0.024460000
H 3.003830 -0.001006 0.232446
Co -0.168668 0.000232 -0.233695
C 0.695085 -0.002378 1.346580
C -1.225229 1.434862 -0.060577
O 1.257023 -0.004169 2.347123
O -1.871091 2.385065 -0.074594
C -1.226853 -1.433574 -0.063631
O -1.873863 -2.382966 -0.079675
C 2.796945 0.000551 -0.839390
C 1.350786 0.001316 -1.215022
H 3.291130 0.875109 -1.290670
H 3.290801 -0.872921 -1.293131
H 1.286870 0.002929 -2.308165
H -0.759546 0.001533 -1.581297
FREQS= 35 75 80 97 117 124 173 312 318 349 432 443 464 470 519 535 556 639 665 836 866
989 1022 1124 1364 1382 1484 1495 2043 2109 2129 2164 2999 3042 3085 3137
MIN020_2
14
E= -1801.83242291 ZPE= 53.63 Gcorr 0.028100000
H 0.089979 0.954065 1.980096
Co 0.017978 0.162229 0.485664
C -1.723471 -0.174476 0.370681
C 0.212026 1.685264 -0.420361
O -2.854412 -0.352119 0.268555
O 0.340851 2.623828 -1.072791
C 0.417004 -1.231464 -0.771676
O 0.017914 -1.681780 -1.798669
C 2.024032 -0.229802 0.651356
C 1.286235 -1.402685 0.350535
H 2.552838 0.288001 -0.141666
H 2.504377 -0.160106 1.623962
H -0.146197 0.197479 2.155509
H 1.183809 -2.260084 1.009211
FREQS= 65 82 98 113 224 289 321 353 391 416 457 467 491 501 524 574 616 631 643 783 855
904 929 1023 1059 1219 1361 1471 1535 1949 2105 2149 3148 3189 3229 3350
MIN021_2
14
E= -1801.83166596 ZPE= 54.73 Gcorr 0.027480000
H -2.618941 0.789142 0.638283
Co 0.347134 0.010173 -0.490701
C 2.067916 -0.146053 -0.147622
C -0.001873 1.570661 0.303294
O 3.163486 -0.382128 0.107574
O -0.203590 2.590466 0.791235
C -0.769967 -1.214024 0.429166
O -0.593862 -1.895023 1.378766
C -2.769540 0.187154 -0.262072
C -1.532335 -0.629706 -0.618646
H -3.632505 -0.466410 -0.086042
H -3.026344 0.870753 -1.076381
H 0.670377 -1.263675 -1.219434
H -1.658685 -1.319188 -1.452808
FREQS= 43 72 88 96 169 171 223 266 310 393 424 458 468 524 540 577 616 637 823 878 884
1054 1066 1122 1338 1426 1499 1516 1932 1985 2111 2156 3038 3105 3126 3144
MIN022_2
14
E= -1801.82713311 ZPE= 52.27 Gcorr 0.022992000
H -0.378597 -0.406502 2.002263
Co -0.201637 0.000016 0.379171
C 0.119498 -1.613733 -0.294890
C 0.119641 1.613738 -0.294888
O 0.368467 -2.635418 -0.759928
O 0.368709 2.635401 -0.759920
C -1.964805 0.000096 0.042733
O -3.079506 0.000066 -0.226228
C 1.752394 -0.000096 0.660485
C 2.736837 -0.000095 -0.237997

H 2.022426 -0.000166 1.717853
H -0.378544 0.406576 2.002257
H 3.782796 -0.000158 0.066080
H 2.553384 -0.000033 -1.310123
FREQS= 50 78 93 98 118 136 274 300 369 369 441 445 463 483 503 531 538 568 605 616 622
883 936 1016 1025 1293 1429 1433 1662 2107 2119 2169 3115 3135 3207 3337
MIN023_2
14
E= -1801.83396888 ZPE= 56.84 Gcorr 0.029965000
H -0.559413 -1.516596 1.092626
Co 0.260073 -0.128174 0.157088
C -1.450518 0.528481 -0.289414
C 1.799935 -1.014106 -0.140445
O -1.857553 1.461535 -0.939710
O 2.738627 -1.456292 -0.641127
C 0.876566 1.460340 0.304981
O 1.292784 2.522897 0.473466
C -2.340170 -0.654733 0.140598
C -1.395317 -1.836379 0.418969
H -2.851759 -0.349275 1.062045
H -3.101381 -0.878285 -0.614567
H -0.988223 -2.263811 -0.504819
H -1.855027 -2.658068 0.974180
FREQS= 35 47 89 98 104 201 270 271 336 361 405 429 456 508 547 593 612 761 890 998 1017
1108 1247 1295 1408 1458 1487 1599 1798 2076 2131 2783 3046 3066 3106 3121
MIN024_2
14
E= -1801.83182718 ZPE= 54.86 Gcorr 0.027863000
H 2.616974 -1.222832 -1.176160
Co -0.249818 0.001230 -0.407141
C -1.721776 -0.963294 -0.323047
C 1.053620 -0.439296 0.890625
O -2.602154 -1.693209 -0.209019
O 1.031253 -1.138669 1.843488
C -0.843557 1.590435 0.148886
O -1.228444 2.624857 0.465958
C 2.801660 -0.176166 -0.923881
C 1.671436 0.400574 -0.077552
H 2.899015 0.376196 -1.862735
H 3.758436 -0.109484 -0.391260
H 1.853317 1.407346 0.297773
H 0.243817 -1.301789 -0.968411
FREQS= 50 72 84 94 159 180 227 287 316 406 417 467 478 516 531 575 622 652 823 883 921
1053 1064 1108 1343 1420 1498 1514 1945 1982 2113 2157 3037 3108 3129 3144
MIN025_2
14
E= -1801.82948021 ZPE= 55.30 Gcorr 0.025842000
H 1.868833 -2.194762 -0.023858
Co -0.302039 -0.050328 0.044574
C -1.846500 -0.880179 -0.010891
C -1.053381 1.483595 -0.091475
O -2.870783 -1.407474 0.020051
O -1.526945 2.528232 -0.195547
C 2.517355 0.428017 0.051992
O 3.538499 -0.086249 -0.231642
C 1.340473 0.911741 0.373405
C 0.789963 -2.051274 -0.060211
H 1.348336 1.936934 0.731800
H 0.488450 -1.618392 0.911887
H 0.256041 -2.988809 -0.203485
H 0.579772 -1.403601 -0.939657
FREQS= 34 40 92 105 123 144 219 242 275 355 389 416 441 450 483 520 527 544 568 633 717
1089 1264 1274 1402 1418 1516 1643 2090 2132 2168 2856 2965 3143 3195 3208
MIN026_2
14
E= -1801.83810177 ZPE= 57.01 Gcorr 0.034477000
H -2.251501 0.564815 1.496004
Co -0.025191 0.203431 -0.220370
C -1.830150 0.822043 0.516257
C 1.625052 0.782805 0.028283
O -0.922362 1.747466 0.441562
O 2.686234 1.200152 0.159856
C 0.266446 -1.578783 0.277674
O 0.101775 -2.152217 1.304818
C -2.038080 0.033503 -0.623550
C 0.522340 -1.484203 -1.106822
H -1.972080 0.503583 -1.603534

H -2.650438 -0.859674 -0.554016
H -0.203743 -1.952863 -1.766423
H 1.559104 -1.563881 -1.422994
FREQS= 71 87 96 119 210 305 352 370 397 438 466 484 500 544 586 612 718 767 875 912 991
1002 1009 1036 1243 1319 1438 1470 1545 1978 2139 3078 3130 3136 3217 3237
MIN027_2
14
E= -1801.82525249 ZPE= 52.19 Gcorr 0.021701000
H 0.180987 0.405905 1.832476
Co -0.117276 -0.000026 0.233365
C -1.905424 0.001595 0.387754
C 0.003827 1.626081 -0.480125
O -3.051237 0.002681 0.434008
O 0.142432 2.652912 -0.978462
C 0.000855 -1.626580 -0.479687
O 0.137579 -2.653778 -0.977779
C 1.834290 -0.001525 -0.098959
C 2.796840 -0.001055 0.825125
H 2.140664 -0.002809 -1.144850
H 0.180575 -0.406283 1.832437
H 3.847372 -0.002027 0.536560
H 2.604321 0.000291 1.895740
FREQS= 30 49 92 95 108 179 276 310 360 384 444 458 460 488 492 517 547 549 559 595 598
902 929 1017 1042 1291 1434 1435 1652 2105 2119 2170 3122 3142 3203 3354
MIN028_2
14
E= -1801.82963463 ZPE= 54.86 Gcorr 0.026086000
H -0.882379 2.590614 0.742431
Co -0.000016 0.205192 -0.033356
C -1.746445 0.451171 -0.251787
C 1.746416 0.451274 -0.251781
O -2.844385 0.681631 -0.496576
O 2.844352 0.681739 -0.496568
C 0.000033 -1.672271 0.298812
O 0.000062 -2.199155 1.361556
C 0.000066 -1.552652 -1.090777
C -0.000060 1.969891 0.914960
H 0.921025 -1.797315 -1.610672
H -0.920863 -1.797360 -1.610704
H 0.881859 2.590958 0.741593
H 0.000487 1.674720 1.974099
FREQS= 39 62 72 95 116 131 201 285 298 386 409 443 463 495 515 524 551 595 686 738 841
864 989 1024 1256 1438 1452 1496 2002 2095 2159 3021 3109 3137 3148 3237
MIN029_2
14
E= -1801.83241872 ZPE= 55.97 Gcorr 0.028927000
H -0.622874 -1.379184 0.538972
Co 0.275957 -0.069055 0.115528
C 0.835781 1.465843 -0.453653
C 1.825580 -0.870464 0.358271
O 1.149991 2.517191 -0.803393
O 2.825245 -1.433143 0.439837
C -1.452876 -0.987775 -0.331808
O -1.791203 -1.869371 -1.084886
C -1.641117 0.413231 -0.119064
C -2.142670 0.896906 1.236713
H -1.862441 1.937549 1.417945
H -2.007039 0.955834 -0.987069
H -3.236568 0.826960 1.291346
H -1.742372 0.299455 2.064339
FREQS= 37 49 91 96 140 170 201 295 352 402 435 467 488 509 569 595 620 880 933 959 1062
1096 1116 1344 1425 1452 1500 1514 1813 1890 2096 2149 3027 3084 3122 3174
MIN030_2
14
E= -1801.83013439 ZPE= 54.25 Gcorr 0.026666000
H 0.860944 1.854116 -0.262135
Co 0.294077 0.455761 -0.254131
C 2.032489 0.146147 -0.239359
C -0.229731 -1.070140 0.821953
O 3.176276 0.058924 -0.167691
O -0.250349 -1.178578 2.000306
C -1.156389 1.420655 0.047661
O -2.010774 2.142906 0.310864
C -1.848092 -1.626933 -1.040635
C -0.435013 -1.406912 -0.517279
H -1.914073 -1.379083 -2.103909
H -2.576326 -1.006958 -0.510874

H -2.145763 -2.675394 -0.918440
H 0.334333 -2.061139 -0.924984
FREQS= 52 60 90 113 150 169 234 279 304 418 431 450 465 475 518 548 573 596 692 849 871 1047 1066 1126 1347 1425 1495 1515 1909 2005 2094 2162 3038 3106 3129 3148
MIN031_2
14
E= -1801.83012518 ZPE= 55.03 Gcorr 0.027503000
H -0.922351 -1.771724 -1.742180
Co 0.000002 0.255730 -0.262001
C 0.000005 -1.648581 0.161528
C 1.749452 0.374600 0.023245
O 0.000004 -2.211196 1.202000
O 2.855737 0.485700 0.311202
C -1.749450 0.374589 0.023234
O -2.855747 0.485679 0.311201
C -0.000015 2.203282 0.226817
C 0.000011 -1.509393 -1.230694
H -0.880518 2.568181 0.758908
H 0.880451 2.568169 0.758975
H 0.000026 2.653926 -0.779490
H 0.922377 -1.771723 -1.742170
FREQS= 37 72 99 123 131 145 208 286 294 418 443 455 461 479 509 512 551 590 698 733 850 864 994 1023 1258 1432 1448 1501 2008 2094 2160 2996 3113 3133 3148 3224
MIN032_2
14
E= -1801.82839901 ZPE= 54.48 Gcorr 0.026944000
H 2.113549 -1.011566 -1.455263
Co -0.453864 -0.032614 0.001339
C 2.331858 -0.558601 -0.455158
C -0.202359 1.735320 0.010668
O 3.346618 0.089528 -0.283878
O -0.101228 2.869645 0.152738
C -2.158531 -0.428698 -0.266674
O -3.282405 -0.668163 -0.291561
C 0.310551 -1.816170 0.351734
C 1.308184 -0.826254 0.571992
H 0.354879 -2.403909 -0.568790
H -0.114038 -2.357372 1.191465
H 1.589421 -0.532861 1.580378
H -0.931589 0.224625 1.322288
FREQS= 55 60 77 89 117 180 288 349 373 410 420 437 474 479 524 595 636 648 781 894 986 998 1017 1167 1217 1358 1447 1517 1793 2110 2129 2171 2823 3099 3180 3214
MIN033_2
14
E= -1801.82692500 ZPE= 53.07 Gcorr 0.026048000
H 0.006086 -0.528423 1.886578
Co 0.154341 -0.028443 0.303968
C -0.044334 1.670843 -0.136783
C 1.934862 -0.134361 0.082631
O -0.157518 2.774601 -0.443557
O 3.057485 -0.245367 -0.141339
C -1.704467 -0.387862 -0.047698
O -2.802094 -0.039605 0.262104
C -0.006058 -2.036727 -0.212615
C -0.934786 -1.238851 -0.912023
H 0.785384 -2.521597 -0.775886
H -0.314239 -2.572294 0.679989
H -0.017444 0.283804 1.924256
H -0.881280 -1.048820 -1.980821
FREQS= 49 88 96 110 183 224 271 330 339 386 429 462 480 508 514 545 574 623 643 796 859 912 951 1011 1049 1227 1369 1454 1536 1941 2100 2141 3152 3182 3234 3353
MIN034_2
14
E= -1801.82848990 ZPE= 53.45 Gcorr 0.027665000
H -0.383746 -0.098413 1.879267
Co 0.161497 -0.020333 0.323596
C -1.678222 -0.459944 -0.166005
C -0.118579 1.691066 -0.028982
O -2.794171 -0.054082 -0.066552
O -0.284930 2.801289 -0.279441
C 1.912583 -0.052937 -0.067545
O 3.017587 -0.127370 -0.378240
C 0.063610 -2.060054 0.110032
C -0.816107 -1.412148 -0.788683
H -0.320401 -2.462524 1.043255
H 0.895826 -2.618748 -0.306837
H 0.433808 -0.069947 1.954318

H -0.673508 -1.395963 -1.866142
FREQS= 58 90 97 111 222 279 333 368 372 416 435 469 484 505 533 554 572 614 636 796 859 915 982 1016 1055 1222 1371 1505 1533 1945 2102 2143 3147 3180 3229 3242
MIN035_2
14
E= -1801.82786602 ZPE= 53.78 Gcorr 0.028036000
H -0.075791 0.332124 -1.872323
Co 0.145566 -0.032675 -0.315973
C 1.917780 -0.393075 -0.332853
C -1.710918 -0.519949 -0.148944
O 3.039594 -0.631129 -0.259828
O -2.775326 -0.266660 -0.623035
C 0.012067 1.681734 0.163928
O -0.066395 2.774858 0.513522
C -0.958425 -1.487791 0.590987
C -0.072255 -0.910829 1.530127
H 0.020279 -0.501945 -1.851573
H -0.882665 -2.514979 0.244638
H -0.421590 -0.136009 2.204579
H 0.716998 -1.534042 1.941192
FREQS= 62 78 90 109 223 289 300 349 381 426 452 475 488 497 526 581 612 632 672 800 875 920 1028 1050 1084 1223 1368 1540 1713 1943 2104 2144 3020 3152 3184 3232
MIN036_2
14
E= -1801.82848071 ZPE= 54.72 Gcorr 0.029552000
H -0.253656 0.394973 -1.390993
Co -0.190753 0.022149 0.010076
C 1.626569 -0.360303 -0.491721
C -1.904169 -0.510241 -0.277883
O 2.560984 0.121174 -1.054744
O -2.950393 -0.768639 -0.675374
C -0.152908 1.760965 0.341377
O -0.108849 2.902899 0.465745
C 1.019040 -1.534841 0.015081
C 0.602315 -1.437460 1.448356
H 0.914808 -2.450515 -0.555793
H 1.414561 -1.194821 2.137525
H 0.067249 -2.325442 1.784517
H -0.151657 -0.574418 1.656417
FREQS= 66 76 92 102 213 284 320 348 395 428 436 449 458 504 543 599 627 677 724 783 915 1057 1082 1121 1308 1373 1489 1659 1952 2099 2107 2156 2368 3089 3168 3208
MIN037_2
14
E= -1801.82967793 ZPE= 57.08 Gcorr 0.030971000
H 2.889228 -1.618926 0.388399
Co -0.370356 0.074352 -0.093914
C -1.463935 -1.245320 -0.135939
C 1.249347 -0.710430 0.056546
O -2.162188 -2.163552 -0.184035
O 1.935486 -1.749975 0.524927
C -1.546432 1.372260 0.118955
O -2.280808 2.223738 0.376194
C 1.626304 0.532080 -0.323118
C 2.902252 1.303698 -0.127793
H 0.845549 1.098614 -0.909670
H 2.712187 2.278803 0.332306
H 3.423183 1.482275 -1.075860
H 3.584324 0.756301 0.531906
FREQS= 54 65 80 98 128 177 203 282 362 400 408 461 481 503 555 587 624 672 712 925 1044 1083 1195 1325 1420 1491 1496 1525 1690 2081 2134 2719 3034 3097 3112 3707
MIN038_2
14
E= -1801.82839321 ZPE= 54.74 Gcorr 0.030118000
H -0.755941 -0.459158 1.263211
Co 0.253002 -0.021915 -0.107579
C -1.728919 -0.454215 0.531434
C 1.919830 -0.354304 0.410732
O -2.726829 0.066202 0.971913
O 3.020260 -0.573857 0.652105
C 0.061451 1.749672 -0.211519
O 0.044396 2.882768 -0.389727
C -0.202993 -1.912786 -0.660212
C -1.362276 -1.097145 -0.705414
H -0.038040 -2.570238 0.188941
H 0.184536 -2.299192 -1.597256
H 0.867592 0.202447 -1.403936
H -1.914371 -0.870399 -1.610766

FREQS= 65 83 94 107 189 297 358 382 403 448 455 485 504 523 540 623 710 783 829 893 943 991 1035 1136 1228 1382 1469 1537 1817 2006 2088 2121 2164 3149 3216 3235
MIN039_2
14
E= -1801.81955838 ZPE= 52.07 Gcorr 0.021535000
H -0.000383 -0.312524 -2.072981
Co -0.000296 -0.226981 -0.446340
C 1.645111 0.470499 -0.569889
C -0.005447 -1.857953 0.347639
O 2.708323 0.907950 -0.584556
O -0.008656 -2.839319 0.944504
C -1.641899 0.479304 -0.570689
O -2.702779 0.922361 -0.585722
C 0.001739 0.674469 1.306142
C 0.004399 1.986843 1.539210
H -0.001695 -1.103719 -1.844666
H 0.000480 -0.005039 2.157978
H 0.005274 2.373766 2.557616
H 0.005805 2.729102 0.744956
FREQS= 53 57 67 103 111 137 300 300 304 315 362 423 456 481 491 514 526 538 567 577 624 934 1009 1023 1032 1295 1425 1655 1685 2102 2106 2161 3123 3144 3206 3213
MIN040_2
14
E= -1801.81914499 ZPE= 52.73 Gcorr 0.023804000
H -0.906555 -0.678512 -2.031205
Co -0.489701 -0.147005 -0.388569
C -2.148394 -0.282804 0.287551
C 1.127159 -0.824666 0.017901
O -3.138499 -0.396485 0.863648
O 0.475697 -1.881747 0.015000
C -0.258030 1.539841 -0.129841
O -0.063596 2.660021 0.087423
C 2.536190 -0.692313 0.361171
C 3.130408 0.506750 0.383288
H 3.075301 -1.606495 0.601415
H -0.213052 -0.307819 -2.122364
H 2.575763 1.410172 0.149026
H 4.177645 0.616622 0.645518
FREQS= 38 73 90 101 145 182 265 309 334 373 415 433 451 464 498 516 567 591 607 713 757 978 1016 1023 1175 1229 1309 1443 1648 1696 2071 2128 3168 3180 3266 3634
MIN041_2
14
E= -1801.81748858 ZPE= 51.97 Gcorr 0.022610000
H -0.480666 -0.896287 1.463683
Co -0.240369 0.000000 0.323726
C 0.213111 -1.470826 -0.621066
C 0.213111 1.470826 -0.621066
O 0.522256 -2.454629 -1.115792
O 0.522256 2.454629 -1.115792
C -2.029542 0.000000 0.166371
O -3.173180 0.000000 0.196173
C 1.591827 0.000000 1.068390
C 2.737067 0.000000 0.384851
H 1.641470 0.000000 2.153790
H -0.480666 0.896287 1.463683
H 3.697630 0.000000 0.899255
H 2.788091 0.000000 -0.702610
FREQS= 62 63 91 101 108 131 270 300 392 397 421 457 468 511 533 536 546 555 635 651 765 859 929 1025 1039 1288 1433 1655 2054 2071 2154 2162 2195 3120 3173 3199
MIN042_2
14
E= -1801.81549838 ZPE= 51.81 Gcorr 0.021690000
H 0.298451 0.580344 -1.551336
Co 0.139103 -0.019888 -0.223767
C 1.768736 -0.735219 -0.463373
C 0.585994 1.572242 0.506297
O 2.764731 -1.222772 -0.746066
O 0.846417 2.623804 0.873546
C -0.575622 -1.015474 1.103148
O -1.111169 -1.679429 1.865380
C -2.689003 -0.051227 -1.204801
C -1.698384 0.608612 -0.604997
H -2.585484 -1.063265 -1.586805
H -3.664639 0.414545 -1.341779
H -0.246005 -1.064628 -1.173002
H -1.908256 1.623551 -0.265885

FREQS=   50   65   84   97 104 118 259 323 365 399 424 458 477 494 506 536 546 553 639 653 690
858 930 1026 1034 1308 1431 1662 2082 2098 2154 2164 2195 3115 3136 3211
MIN001_3
10
E= -1836.60912983 ZPE=   25.38 Gcorr   -0.018372000
H 0.002618 0.000068 -1.643689
Co 0.000099 0.000013 -0.163852
C 0.990993 -1.459030 -0.459713
C 0.770273 1.586981 -0.458854
O 1.617737 -2.381107 -0.733944
O 1.257414 2.590074 -0.732224
C -0.003328 -0.000894 1.631621
O -0.005520 -0.001624 2.777428
C -1.758410 -0.127011 -0.463650
O -2.869938 -0.207429 -0.739852
FREQS=   61   62   91   91 100 303 303 317 409 410 437 473 499 499 546 549 549 725 726 2032 2119
2119 2141 2196
MIN002_3
10
E= -1836.57583394 ZPE=   26.94 Gcorr   -0.016147000
H -2.827826 0.025111 0.206486
Co -0.054252 0.000538 0.114466
C -0.168581 -1.542734 -0.739246
C 1.713082 -0.015570 0.527343
O -0.211745 -2.453795 -1.443957
O 2.803883 -0.025525 0.879015
C -0.140293 1.546277 -0.738278
O -0.166309 2.458222 -1.442692
C -1.810589 0.015864 0.636347
O -1.584462 0.013267 1.830876
FREQS=   56   80   80 103 108 148 240 304 374 399 456 502 512 513 528 551 660 891 1262 1730 2082
2108 2167 2988
MIN003_3
10
E= -1836.56221870 ZPE=   25.96 Gcorr   -0.019746000
H 0.740800 -0.000165 2.144169
Co 0.126946 0.000006 -0.227220
C -1.611517 0.000432 -0.764673
C 1.041453 -1.552926 -0.230097
O -2.662642 0.000701 -1.227413
O 1.616131 -2.547026 -0.308633
C -0.212967 -0.000075 1.563233
O -1.286127 -0.000037 2.089267
C 1.042146 1.552534 -0.229867
O 1.617258 2.546390 -0.308322
FREQS=   50   55   57   73 107 173 224 263 298 339 412 477 481 484 491 509 590 838 1225 1832 2085
2094 2159 2844
MIN004_3
10
E= -1836.55461657 ZPE=   23.85 Gcorr   -0.025211000
H 0.013987 0.148638 -1.678978
Co -0.005073 -0.045276 -0.193376
C -1.657148 -0.425709 -0.643843
C 1.541167 -0.740534 -0.643868
O -2.674083 -0.797484 -1.037421
O 2.466123 -1.303353 -1.037510
C -0.059412 -0.596238 1.496585
O -0.105099 -1.059642 2.546998
C 0.328806 3.286675 0.145686
O 0.213371 2.151561 0.124530
FREQS=   43   45   72   87   92 118 123 131 322 336 340 453 485 490 514 563 575 648 746 1961 2091
2111 2166 2171
MIN005_3
10
E= -1836.55163290 ZPE=   24.25 Gcorr   -0.022217000
H -0.003060 0.001839 -1.598398
Co -0.000293 0.000253 -0.153096
C -0.797913 -1.573707 -0.441137
C 1.761281 0.097407 -0.443520
O -1.292072 -2.548146 -0.803738
O 2.851739 0.157808 -0.807475
C 0.005522 -0.003464 3.006548
O 0.003397 -0.002074 1.863512
C -0.965713 1.477895 -0.437856
O -1.564075 2.392730 -0.798827
FREQS=   59   59   82   82   89 212 212 251 306 346 346 438 439 455 511 511 511 720 722 2093 2093
2107 2150 2171

```
MIN006_3
10
E= -1836.55072655 ZPE=    26.69 Gcorr   -0.020173000
H 3.447157 -0.001078 0.061046
Co 0.019954 -0.000014 0.014022
C -0.416053 1.451369 -0.947742
C -0.416836 -1.451018 -0.947950
O -0.607979 2.404232 -1.562802
O -0.609267 -2.403685 -1.563155
C -1.152550 0.000159 1.369935
O -1.819254 0.000252 2.306167
C 1.596509 -0.000414 0.456746
O 2.829957 -0.000689 0.816594
FREQS=   17   48   73   74   88  100  272  280  305  434  473  486  490  510  522  579  588  649 1081 1543 2102
2106 2161 3686
MIN007_3
10
E= -1836.54842726 ZPE=    24.02 Gcorr   -0.022330000
H 0.000000 0.182165 -1.514868
Co 0.000000 -0.009415 -0.084689
C -1.606134 -0.631720 -0.558641
C 1.606134 -0.631720 -0.558641
O -2.607686 -0.978222 -1.004479
O 2.607686 -0.978222 -1.004479
C 0.000000 -1.412853 1.672015
O 0.000001 -0.319140 2.064317
C 0.000000 1.798709 0.050504
O 0.000000 2.942778 -0.034101
FREQS=   53   67   92   92  107  127  229  307  310  321  359  401  443  445  490  507  518  677  752 1983 2102
2114 2124 2178
MIN001_4
10
E= -1724.43620322 ZPE=    30.28 Gcorr   -0.005631000
H 0.417690 0.188147 2.011791
Co -0.000001 -0.127996 0.465723
C -1.540835 -0.673036 -0.203879
C 1.540828 -0.673043 -0.203880
O -2.499053 -1.118060 -0.657272
O 2.499039 -1.118084 -0.657270
C 0.000002 1.571809 -0.092807
O 0.000018 2.647611 -0.489402
H -0.417702 0.188115 2.011796
H 0.000022 -1.566497 0.836840
FREQS=   69   88   95  250  278  308  413  414  431  471  501  502  552  576  583  755  759 1024 1599 2015 2115
2131 2180 3077
MIN002_4
10
E= -1724.43391933 ZPE=    30.48 Gcorr   -0.006336000
H 0.000355 -0.105304 -1.220698
Co 0.000119 -0.055639 0.237363
C 1.577279 -0.833932 -0.044116
C -1.575888 -0.836340 -0.044054
O 2.577009 -1.308263 -0.356516
O -2.574925 -1.312133 -0.356438
C -0.001503 1.714007 -0.021282
O -0.002562 2.827101 -0.306686
H 0.000421 -0.564366 1.779085
H 0.000512 0.255875 1.846629
FREQS=   54   66  102  234  268  273  318  400  458  472  507  519  522  562  588  721  735 1075 1737 2093 2107
2111 2177 3222
MIN003_4
10
E= -1724.43065599 ZPE=    29.68 Gcorr   -0.006425000
H -0.883201 0.511617 1.523155
Co -0.000004 -0.000643 0.469924
C 1.427475 -0.828034 -0.224027
C 0.004205 1.649837 -0.222735
O 2.376296 -1.378062 -0.551389
O 0.007001 2.746338 -0.550758
C -1.431674 -0.820759 -0.224032
O -2.383282 -1.365955 -0.551392
H -0.002621 -1.026191 1.518794
H 0.885779 0.507101 1.523169
FREQS=   80   80   95  266  267  285  442  448  449  504  505  554  559  599  601  748  749  761 2071 2072 2097
2163 2163 2202
MIN004_4
10
```

E= -1724.43672654 ZPE= 36.39 Gcorr 0.004863000
H 2.692946 -1.099831 -0.906772
Co 0.171072 -0.103301 -0.000162
C -1.240782 -1.110476 -0.000002
C -0.698285 1.391726 -0.000143
O -2.159152 -1.809316 0.000071
O -1.259993 2.393976 0.000346
C 2.179934 -0.751945 0.000413
O 1.837575 0.572009 -0.000319
H 2.692719 -1.098888 0.908088
H 1.202761 -1.441343 0.000660
FREQS= 63 82 102 131 328 405 412 471 491 506 559 614 678 1109 1170 1254 1322 1535 1737 2095 2159 2161 3006 3063
MIN005_4
10
E= -1724.42189651 ZPE= 29.03 Gcorr -0.007652000
H 0.000001 -1.580796 0.675859
Co 0.000000 -0.232021 0.087420
C 1.714286 -0.617600 -0.034164
C 0.000000 1.555845 -0.051529
O 2.807304 -0.942620 -0.133404
O 0.000000 2.699595 -0.046733
C -1.714286 -0.617600 -0.034164
O -2.807304 -0.942620 -0.133403
H 0.000000 0.007838 1.569968
H 0.000000 -0.601188 -1.378719
FREQS= 68 93 96 183 297 350 375 461 486 495 505 550 563 620 626 648 657 786 1848 2010 2061 2152 2165 2208
MIN006_4
10
E= -1724.42011992 ZPE= 34.04 Gcorr 0.000842000
H 1.958716 -1.738731 0.957567
Co 0.038330 -0.159782 0.111158
C 0.880735 1.348958 -0.047097
C -1.746419 0.126736 -0.041776
O 1.456162 2.340106 -0.153090
O -2.855331 0.407097 -0.088610
C 1.499718 -1.445715 0.008198
O 0.282313 -1.906368 -0.266997
H 2.192168 -1.300819 -0.826828
H -0.055157 0.447107 1.421634
FREQS= 69 76 115 145 304 348 403 426 431 516 536 612 650 713 805 1082 1203 1269 1561 2086 2115 2173 3040 3129
MIN007_4
10
E= -1724.40541184 ZPE= 32.33 Gcorr -0.002271000
H 0.000001 1.406790 -1.913545
Co 0.000000 0.064859 0.445346
C 1.501564 -0.686647 -0.069680
C -1.501565 -0.686647 -0.069680
O 2.366479 -1.326568 -0.488442
O -2.366480 -1.326568 -0.488442
C 0.000000 1.354372 -0.810747
O 0.000000 2.327472 -0.083104
H -0.410208 -0.219573 2.034870
H 0.410208 -0.219573 2.034870
FREQS= 71 96 111 147 239 282 367 406 473 520 523 543 552 655 687 902 1008 1266 1600 1734 2073 2127 2993 3243
MIN008_4
10
E= -1724.40907995 ZPE= 34.38 Gcorr 0.002127000
H 2.664047 0.448348 0.102403
Co 0.139901 -0.187785 -0.200172
C -0.136335 1.527226 -0.031159
C -1.458197 -0.704392 0.025811
O -0.285236 2.666581 0.025016
O -2.537846 -1.001611 0.358980
C 1.971178 -0.280239 0.537482
O 1.583038 -1.300582 -0.193717
H 2.022300 -0.392119 1.627006
H -0.803196 -1.156699 -1.039806
FREQS= 61 100 119 139 367 386 436 448 520 529 590 609 651 767 968 1079 1206 1315 1570 1867 2043 2137 3025 3113
MIN009_4
10
E= -1724.40179406 ZPE= 31.50 Gcorr -0.003801000
H 2.653403 0.327972 -0.960915

Co 0.191693 -0.088214 0.298137
C -0.244751 1.551391 -0.022421
C 1.797975 -0.150158 -0.463712
O -0.518772 2.643423 -0.277695
O 1.672066 -1.370489 -0.345455
C -1.409228 -0.854871 -0.016125
O -2.384097 -1.350260 -0.371500
H 0.832533 0.000709 1.910046
H 0.320804 -0.606457 1.971910
FREQS= 73 95 113 128 277 305 346 419 439 460 483 516 532 587 692 778 848 1209 1240 1638 2090 2141 3060 3566
MIN010_4
10
E= -1724.40360802 ZPE= 33.52 Gcorr -0.001543000
H 0.000132 3.672361 0.024789
Co -0.000006 -0.158959 -0.400577
C 1.558886 -0.749611 0.077931
C -1.558939 -0.749504 0.077931
O 2.463949 -1.312216 0.529544
O -2.464041 -1.312045 0.529545
C 0.000098 2.674572 0.477710
O 0.000056 1.678600 -0.246262
H 0.000102 2.589680 1.571047
H -0.000049 -1.457589 -1.084302
FREQS= 68 71 74 183 184 277 353 409 433 464 487 552 559 631 766 1079 1252 1521 1693 2038 2053 2120 3034 3143
MIN011_4
10
E= -1724.39565150 ZPE= 31.33 Gcorr -0.003435000
H 2.495962 -1.310539 0.908306
Co 0.019313 -0.215940 0.236010
C 0.532825 1.461268 -0.149862
C 1.625505 -1.041862 0.289792
O 0.814842 2.572947 -0.178673
O 1.446378 -1.439955 -0.851259
C -1.751898 -0.210107 -0.117723
O -2.888994 -0.267672 -0.242377
H -0.126634 0.272045 1.591024
H -0.307193 -1.309493 1.173622
FREQS= 77 90 119 168 254 340 379 438 482 494 521 548 601 626 670 793 902 1246 1701 2022 2108 2134 2172 3031
MIN012_4
10
E= -1724.39824518 ZPE= 35.47 Gcorr 0.002402000
H 0.846406 -1.611891 0.065629
Co -0.084548 -0.232110 0.229759
C -0.100678 1.462510 0.071896
C -1.787670 -0.608044 -0.067000
O -0.045353 2.600042 -0.131614
O -2.862397 -0.894719 -0.369116
C 1.658377 -0.698922 0.336913
O 2.703571 -0.673106 -0.530802
H 2.068158 -0.921237 1.331183
H 2.381481 -0.390893 -1.398910
FREQS= 53 72 96 144 249 337 404 438 488 524 582 616 642 813 1053 1218 1280 1411 1460 1875 2072 2134 3057 3793
MIN013_4
10
E= -1724.39140780 ZPE= 31.13 Gcorr -0.003312000
H 0.000000 2.300473 1.455239
Co 0.000000 -0.119550 -0.043224
C -1.706982 -0.567979 -0.016104
C 1.706982 -0.567979 -0.016104
O -2.772817 -0.986062 0.048843
O 2.772817 -0.986062 0.048843
C 0.000000 1.603084 0.600324
O 0.000000 1.980538 -0.561070
H 0.000000 -0.847958 1.216855
H 0.000000 -1.094716 -1.206682
FREQS= 83 102 129 196 200 363 419 446 479 491 531 542 627 659 684 736 882 1239 1701 1888 2067 2129 2179 3002
MIN014_4
10
E= -1724.38885996 ZPE= 30.95 Gcorr -0.004939000
H 0.469982 2.339460 0.524738
Co 0.124266 -0.118454 0.629511
C 1.598757 -0.474486 -0.319795

C -1.336114 -0.863007 -0.175549
O 2.604037 -0.640542 -0.845250
O -2.279577 -1.388970 -0.551367
C -0.154655 1.580578 0.009269
O -0.922715 1.861593 -0.864382
H -0.694869 0.350133 1.777864
H 1.007808 0.393493 1.705053
FREQS= 71 75 103 153 256 308 316 381 418 497 512 569 627 642 679 794 887 1264 1823 2009 2020
2142 2178 2924
MIN015_4
10
E= -1724.39009851 ZPE= 32.69 Gcorr -0.001753000
H 1.529465 2.054206 0.936190
Co -0.010684 0.019587 -0.127132
C -1.676903 -0.609340 0.035182
C 1.442977 -0.908216 0.009596
O -2.679514 -1.143425 0.200859
O 2.329117 -1.645223 0.060447
C 0.586794 1.698131 0.501743
O -0.067986 2.305525 -0.343419
H 0.004871 0.926618 1.261983
H -0.016032 -0.728133 -1.387826
FREQS= 59 97 104 173 197 326 357 441 474 483 500 543 675 760 809 1073 1303 1466 1715 1931 2058
2097 2155 3071
MIN016_4
10
E= -1724.38620206 ZPE= 30.88 Gcorr -0.005362000
H 2.886295 -0.492397 -0.000065
Co 0.117814 -0.144450 0.000002
C -1.674308 -0.436530 0.000001
C 0.262332 1.575201 -0.000001
O -2.801398 -0.627037 -0.000007
O 0.385634 2.716140 0.000005
C 1.811982 -0.720688 -0.000026
O 1.329106 -1.840431 0.000025
H 0.112961 -0.052401 -1.522018
H 0.112982 -0.052337 1.522018
FREQS= 39 84 104 109 293 318 434 468 487 513 550 580 620 628 714 803 868 1210 1704 1742 1933
2141 2183 3073
MIN017_4
10
E= -1724.37995508 ZPE= 29.25 Gcorr -0.007780000
H 0.404938 0.204401 1.993786
Co -0.000040 -0.082360 0.377956
C -1.628520 -0.544407 -0.139305
C 1.628332 -0.544680 -0.139333
O -2.631234 -0.986050 -0.490484
O 2.630984 -0.986470 -0.490513
C -0.000016 1.588251 -0.990071
O 0.000563 2.016465 0.096934
H -0.405136 0.204619 1.993720
H -0.000008 -1.531844 0.492458
FREQS= 70 110 114 141 266 298 323 374 386 427 464 488 512 529 538 671 776 911 1397 1943 2084
2102 2153 3386
MIN018_4
10
E= -1724.37683375 ZPE= 29.33 Gcorr -0.008437000
H 0.373718 0.815969 1.842981
Co -0.004616 0.090774 0.422386
C -1.603709 -0.467309 -0.139082
C 1.318735 -0.916281 -0.137906
O -2.573108 -0.990656 -0.465698
O 2.094127 -1.704949 -0.459158
C 1.058725 1.878219 -0.342913
O -0.063928 1.941550 -0.644464
H -0.456519 0.894974 1.784227
H -0.091795 -1.097171 1.242325
FREQS= 66 78 105 120 246 277 339 340 385 425 439 500 528 564 633 734 778 976 1598 1970 2102
2108 2159 3049
MIN019_4
10
E= -1724.37986952 ZPE= 32.35 Gcorr -0.002318000
H 2.650804 0.752392 -0.078549
Co 0.069391 -0.189130 0.266749
C -0.186817 1.504178 -0.142862
C 1.992531 -0.121784 0.027418
O -0.476720 2.619136 -0.188227

O 2.144297 -1.176385 -0.562874
C -1.542677 -0.757306 -0.080416
O -2.585458 -1.190744 -0.291748
H 1.411873 -0.132624 1.130619
H -0.171424 -1.279840 1.263671
FREQS= 69 99 106 157 181 329 371 405 443 498 520 549 570 694 855 1050 1342 1524 1735 1879 1953 2101 2151 3044
MIN001_5
16
E= -1915.23011290 ZPE= 62.02 Gcorr 0.032691000
H -2.872485 -0.882265 -0.741685
Co 0.222015 0.000001 0.008697
C 0.805451 -0.000004 1.709839
C 1.848012 0.000012 -0.749456
O 1.121058 -0.000008 2.814681
O 2.885785 0.000018 -1.237582
C -0.284430 1.578158 -0.679313
O -0.641096 2.597893 -1.073181
C -2.862655 -0.000009 -0.094884
C -1.685376 -0.000008 0.875238
H -2.872497 0.882258 -0.741669
H -3.805951 -0.000021 0.467598
H -1.730232 -0.880344 1.521298
H -1.730239 0.880325 1.521301
C -0.284409 -1.578157 -0.679325
O -0.641065 -2.597894 -1.073200
FREQS= 50 53 90 90 102 107 108 125 256 265 317 355 365 415 434 477 479 485 502 503 545 552 564 723 985 985 1051 1239 1278 1433 1485 1511 1512 2098 2103 2119 2178 3030 3069 3098 3114 3136
MIN002_5
16
E= -1915.22948093 ZPE= 62.67 Gcorr 0.032448000
H -4.715927 -0.000270 0.139891
Co 0.556178 0.000011 -0.095322
C 0.596954 -1.542430 0.763351
C 2.232892 -0.000262 -0.776759
O 0.674589 -2.459460 1.460029
O 3.261516 -0.000387 -1.284878
C 0.597345 1.542464 0.763299
O 0.675248 2.459548 1.459872
C -3.814506 -0.000077 -0.478733
C -2.565722 0.000132 0.405816
H -3.839022 -0.883004 -1.122467
H -3.839372 0.882877 -1.122417
H -2.534467 -0.870367 1.074033
H -2.534613 0.870811 1.073807
C -1.270699 0.000152 -0.350517
O -1.137728 0.000274 -1.563509
FREQS= 42 46 56 73 83 85 119 193 194 279 293 301 345 411 434 461 512 521 537 547 564 698 782 953 1026 1085 1111 1277 1366 1428 1453 1508 1513 1744 2071 2098 2159 3048 3064 3077 3139 3143
MIN003_5
16
E= -1915.22840250 ZPE= 62.65 Gcorr 0.032003000
H -4.111432 0.075207 -0.823856
Co 0.511554 -0.010429 0.145239
C 0.227861 1.570019 -0.594859
C 2.316581 0.037456 0.272067
O 0.059752 2.518817 -1.230291
O 3.452044 0.061221 0.434484
C 0.330911 -1.491715 -0.793449
O 0.211874 -2.359868 -1.545351
C -3.031648 0.196904 -0.704210
C -2.605896 -0.235351 0.697773
H -2.536270 -0.404252 -1.471408
H -2.787465 1.246731 -0.887931
H -2.854955 -1.292330 0.870080
H -3.123989 0.331854 1.480737
C -1.130656 -0.127919 0.969287
O -0.603767 -0.141670 2.070067
FREQS= 24 49 51 76 84 87 117 166 219 282 300 343 382 419 450 474 510 522 539 549 564 576 770 944 1053 1082 1144 1277 1340 1435 1451 1512 1516 1738 2070 2097 2158 3033 3063 3081 3137 3140
MIN004_5
16
E= -1915.22787180 ZPE= 62.93 Gcorr 0.032527000
H -4.320641 -0.286151 0.493942
Co 0.520986 0.009013 -0.118324
C 0.051191 -1.497335 0.671512
C 2.244137 -0.388759 -0.502689

O -0.199823 -2.407287 1.336837
O 3.317169 -0.629759 -0.829495
C 0.770096 1.489525 0.813195
O 0.937789 2.353104 1.560284
C -2.559288 0.760442 -0.210562
C -3.321116 -0.535154 0.128763
H -3.081542 1.320493 -0.994106
H -2.478126 1.389087 0.681552
H -3.427818 -1.168924 -0.755717
H -2.808215 -1.108876 0.904453
C -1.170896 0.456896 -0.696976
O -0.809514 0.421108 -1.861981
FREQS= 22 51 54 77 85 93 116 189 211 264 300 315 365 410 461 488 512 523 541 548 574
657 812 934 1022 1081 1166 1281 1329 1426 1487 1513 1520 1742 2069 2096 2158 3058 3064 3114 3142 3149
MIN005_5
16
E= -1915.22644341 ZPE= 62.34 Gcorr 0.033279000
H 2.934357 1.026698 0.442216
Co -0.235983 -0.006332 0.024972
C -1.749355 -0.938885 0.552157
C -0.467228 1.676939 0.603031
O -2.725888 -1.504405 0.753255
O -0.581015 2.733083 1.036500
C -0.419541 0.375209 -1.674431
O -0.541394 0.577201 -2.802168
C 2.920834 0.014081 0.033752
C 0.955648 -1.628869 -0.437865
H 3.079768 0.043053 -1.046828
H 3.755608 -0.541091 0.479311
H 0.496813 -2.492466 0.030472
H 1.326209 -1.791125 -1.445574
C 1.636650 -0.685410 0.398004
O 1.037890 -0.424940 1.514698
FREQS= 20 59 65 81 94 118 167 181 296 327 352 380 385 409 444 447 455 496 518 540 573
604 836 858 981 990 1039 1076 1333 1414 1457 1487 1506 1519 2100 2118 2164 3047 3117 3155 3158 3247
MIN006_5
16
E= -1915.22135883 ZPE= 62.53 Gcorr 0.033911000
H 2.615263 -1.857328 -0.032319
Co -0.359776 -0.014152 -0.040136
C 1.333026 0.662136 0.074153
C -1.078970 1.576504 -0.322704
O 2.097385 1.370193 0.654290
O -1.609923 2.597544 -0.369202
C -0.304225 -0.669830 1.602333
O -0.374593 -0.989193 2.706655
C 2.869843 -1.146207 -0.823067
C 1.679134 -0.242426 -1.156410
H 3.707502 -0.535755 -0.476234
H 3.185017 -1.711782 -1.704380
H 0.827145 -0.855757 -1.511026
H 1.903497 0.448006 -1.974407
C -1.693951 -1.053732 -0.712669
O -2.532070 -1.711535 -1.140215
FREQS= 48 55 74 84 88 97 138 200 250 270 317 351 365 389 439 477 501 514 531 534 569
673 710 820 983 1060 1091 1276 1321 1422 1456 1508 1514 1886 2091 2104 2163 2935 3058 3097 3135 3144
MIN007_5
16
E= -1915.22002067 ZPE= 62.55 Gcorr 0.034280000
H 2.938644 -1.294524 -0.351736
Co -0.273547 0.018232 -0.021700
C 0.293264 -0.871481 1.379060
C -0.862478 1.604565 0.710325
O 0.720787 -1.423163 2.294437
O -1.277474 2.571391 1.165386
C -1.649781 -1.076843 -0.406092
O -2.546976 -1.729891 -0.699457
C 1.746134 0.498824 -0.576401
C 2.987210 -0.225253 -0.123854
H 1.149586 -0.949544 -2.122929
H 1.742152 1.576196 -0.437979
H 3.870406 0.189365 -0.625802
H 3.152399 -0.120950 0.952361
C 0.933950 0.002898 -1.622138
O -0.165990 0.645528 -1.884542
FREQS= 30 56 68 89 94 107 179 205 285 303 356 377 396 420 426 432 449 482 516 536 575
616 881 935 973 1059 1136 1226 1302 1368 1431 1493 1504 1542 2104 2116 2164 3038 3068 3102 3125 3189

MIN008_5
16
E= -1915.21793428 ZPE= 62.75 Gcorr 0.035362000
H -2.956587 -0.981739 -0.079787
Co 0.237135 0.034125 0.001848
C 0.617378 -1.295876 1.079918
C -0.287718 -0.796472 -1.559486
O 0.829124 -2.177780 1.788928
O -0.543250 -1.367635 -2.519819
C 1.940133 0.597350 -0.206698
O 2.995610 1.015401 -0.370793
C -1.597835 0.326119 1.000230
C -2.800506 0.082717 0.116361
H -0.332144 1.989219 1.747985
H -1.683303 -0.072435 2.010348
H -2.699144 0.607177 -0.835551
H -3.702502 0.456460 0.619595
C -0.782852 1.489931 0.882334
O -0.476555 1.862182 -0.321872
FREQS= 40 61 65 86 96 107 220 246 270 302 329 359 383 414 438 442 447 500 529 563 565
702 884 932 986 1064 1094 1192 1307 1409 1449 1490 1505 1516 2105 2119 2166 3030 3077 3101 3146 3159
MIN009_5
16
E= -1915.21897437 ZPE= 63.17 Gcorr 0.036495000
H 0.628134 -1.827191 -1.696468
Co -0.227043 -0.011786 -0.006265
C -1.704278 -1.055445 -0.210991
C -0.419388 0.860719 1.518857
O -2.667292 -1.673645 -0.329135
O -0.550095 1.393399 2.531857
C -0.442736 1.387226 -1.058420
O -0.583809 2.274847 -1.781257
C 1.050163 -0.871278 -1.406973
C 1.038696 -1.367458 0.952909
H 2.987321 0.532025 -0.705697
H 1.444688 -0.292347 -2.238832
H 1.432441 -1.159029 1.942009
H 0.610336 -2.355040 0.826154
C 1.691092 -0.759958 -0.146091
O 2.769441 0.037521 0.096817
FREQS= 28 65 67 88 96 121 218 308 339 367 381 400 432 440 452 457 486 494 535 539 570
645 804 846 898 970 990 1008 1048 1209 1411 1449 1541 1559 2086 2092 2145 3146 3167 3231 3253 3806
MIN010_5
16
E= -1915.21165165 ZPE= 62.25 Gcorr 0.031484000
H 0.407972 -0.261137 -2.304805
Co -0.349004 0.020611 0.031985
C 1.564627 0.126632 0.309584
C -0.403317 1.748864 0.385002
O 2.232851 0.259655 1.300906
O -0.520825 2.826622 0.773483
C -0.338312 -1.467763 0.986151
O -0.409016 -2.339762 1.733927
C 1.393148 -0.721581 -2.144823
C 2.211755 0.036719 -1.092876
H 1.242341 -1.767052 -1.861839
H 1.899006 -0.711978 -3.114537
H 2.369776 1.075139 -1.412022
H 3.202334 -0.408996 -0.952488
C -2.021638 -0.138218 -0.621486
O -3.069996 -0.245315 -1.076720
FREQS= 37 39 67 73 90 96 99 127 224 270 310 315 360 399 456 479 484 504 543 552 560
596 773 864 1021 1055 1108 1271 1316 1425 1465 1502 1515 1811 2085 2104 2166 3016 3043 3089 3106 3129
MIN011_5
16
E= -1915.21117174 ZPE= 63.40 Gcorr 0.037100000
H 2.738685 0.723425 -0.266853
Co -0.247048 0.018048 0.018534
C -0.571467 -1.705321 0.232445
C -1.925051 0.592665 0.211651
O -0.764949 -2.834749 0.354552
O -2.999373 1.000816 0.295255
C 0.238011 0.144162 -1.706997
O 0.487238 0.231805 -2.828046
C 0.522527 1.843695 0.610751
C 0.884746 0.795177 1.497175
H 1.226406 2.218385 -0.127759

H -0.154460 2.609987 0.973409
H 0.404623 0.682663 2.463404
H 1.767027 -1.172054 1.579491
C 1.665080 -0.268851 0.987439
O 2.752702 -0.142730 0.163629
FREQS= 42 64 74 89 98 109 206 248 304 320 356 385 420 433 464 478 498 522 535 556 597
658 808 899 964 983 1011 1038 1207 1236 1324 1436 1522 1550 2085 2089 2143 3143 3199 3216 3229 3809
MIN012_5
16
E= -1915.20608495 ZPE= 62.36 Gcorr 0.034688000
H -0.820416 -1.627074 2.306745
Co 0.435933 -0.021966 0.118079
C 1.221013 -1.390746 -0.682442
C -0.296221 1.386846 -0.707891
O 1.692898 -2.226061 -1.320630
O -0.669202 2.245232 -1.374781
C 1.947236 0.796614 0.568636
O 2.922549 1.319084 0.876780
C -1.271019 -1.044560 0.254361
C -0.995580 -0.738231 1.699822
H -2.796639 -0.872768 -1.313036
H -1.153197 -2.086331 -0.040413
H -0.048789 -0.110086 1.860891
H -1.782322 -0.110010 2.121528
C -2.503644 -0.455671 -0.325280
O -3.168687 0.420976 0.197746
FREQS= 45 57 87 93 94 110 132 239 284 285 320 331 393 409 462 490 499 519 535 570 575
719 873 956 1010 1076 1109 1125 1361 1411 1426 1452 1567 1783 2091 2108 2163 2536 2915 3097 3143 3173
MIN013_5
16
E= -1915.20282778 ZPE= 62.34 Gcorr 0.032309000
H -4.529933 -0.592289 -0.116404
Co 0.656216 -0.031097 0.005887
C 1.930190 -1.311191 -0.097033
C -0.262755 1.193753 -0.932259
O 2.729714 -2.100544 -0.315399
O -0.732840 1.919315 -1.686284
C 1.771949 1.162660 0.652131
O 2.429771 1.940208 1.187170
C -2.562434 -0.224189 0.682351
C -4.042232 0.015636 0.652097
H -2.486954 -1.688173 -0.849086
H -1.979819 0.299316 1.440238
H -4.509664 -0.222014 1.616359
H -4.277298 1.067051 0.440740
C -1.921299 -1.080523 -0.136216
O -0.603482 -1.329121 -0.137639
FREQS= 34 45 65 89 97 101 120 157 215 293 301 332 360 401 435 443 470 506 557 584 611
640 824 936 966 1059 1129 1228 1293 1359 1429 1491 1506 1677 2105 2118 2178 3022 3068 3101 3111 3148
MIN014_5
16
E= -1915.20234444 ZPE= 62.31 Gcorr 0.032044000
H -4.450988 1.193275 -0.310006
Co 0.774605 -0.013935 -0.126687
C 2.410259 0.487528 -0.541344
C 0.359637 1.573590 0.563733
O 3.447220 0.812952 -0.917537
O 0.117229 2.524459 1.160658
C 1.293694 -1.668867 0.404209
O 1.596105 -2.661405 0.888484
C -3.247338 -0.590603 -0.169511
C -4.570312 0.112320 -0.184054
H -2.031788 1.118565 -0.466822
H -3.238905 -1.671337 -0.041930
H -5.211924 -0.249476 -0.998335
H -5.125182 -0.057473 0.747956
C -2.067912 0.032135 -0.329129
O -0.901018 -0.630247 -0.378321
FREQS= 37 50 58 83 96 98 124 161 214 287 309 311 372 378 415 439 473 481 515 585 597
738 833 939 962 1060 1136 1217 1293 1367 1430 1490 1507 1699 2103 2115 2174 3020 3066 3074 3108 3173
MIN015_5
16
E= -1915.20409974 ZPE= 62.22 Gcorr 0.034440000
H -0.801313 1.237826 2.624600
Co 0.461359 0.026908 0.162396
C -0.030960 -1.509073 -0.603951
C 0.728195 1.529187 -0.739643

O -0.257872 -2.426482 -1.260489
O 0.868734 2.435583 -1.434954
C 2.179918 -0.399314 0.322533
O 3.291192 -0.662679 0.444669
C -0.800702 0.359051 1.977964
C -1.382621 0.630392 0.616898
H 0.311660 0.065649 1.950750
H -1.272234 -0.484735 2.487805
H -2.527753 -1.242273 0.633484
H -1.575193 1.676192 0.388210
C -2.511945 -0.222340 0.176634
O -3.362451 0.115254 -0.620745
FREQS= 45 56 81 92 95 117 134 260 284 318 322 331 394 415 433 484 498 511 523 567 570
608 914 992 1011 1090 1104 1171 1315 1396 1434 1468 1574 1804 2093 2106 2162 2501 2846 3087 3153 3166
MIN016_5
16
E= -1915.20118776 ZPE= 62.42 Gcorr 0.032314000
H -2.609587 1.559286 1.239913
Co 0.504539 -0.046327 -0.042732
C 1.535851 1.253280 0.542854
C 1.826895 -1.283258 0.047576
O 2.125403 2.108698 1.036629
O 2.666333 -2.053750 -0.061592
C -0.354437 1.016487 -1.200481
O -0.787671 1.623404 -2.072075
C -2.866453 -0.419049 0.391440
C -2.500449 0.502682 1.516682
H -2.442172 -1.906610 -1.044737
H -3.919182 -0.481176 0.125184
H -1.464129 0.351101 1.838399
H -3.151029 0.337465 2.384003
C -2.033624 -1.231212 -0.288783
O -0.714458 -1.383703 -0.083551
FREQS= 33 44 54 87 96 100 104 172 200 272 308 365 405 421 436 448 470 506 548 591 632
698 796 929 961 1058 1059 1170 1313 1404 1445 1486 1502 1676 2105 2118 2179 3021 3072 3096 3110 3177
MIN017_5
16
E= -1915.20078685 ZPE= 62.36 Gcorr 0.032373000
H 4.585073 -0.220171 0.475171
Co -0.718871 -0.022174 0.210358
C -1.370407 -1.613942 -0.322695
C 0.166338 1.530332 -0.099409
O -1.687138 -2.608376 -0.791801
O 0.589753 2.557880 -0.374305
C -2.266838 0.772998 0.008172
O -3.301929 1.274782 -0.005266
C 2.594589 -0.226496 -0.353440
C 4.074182 0.001281 -0.467599
H 2.588491 -0.937323 1.640249
H 1.989024 -0.073474 -1.245989
H 4.526022 -0.627062 -1.246256
H 4.306156 1.041928 -0.730029
C 1.990409 -0.668898 0.764566
O 0.684952 -0.893893 0.952576
FREQS= 26 42 72 92 100 112 131 170 225 281 300 318 371 391 426 434 474 500 536 589 621
636 807 939 953 1062 1128 1234 1300 1354 1432 1492 1512 1693 2110 2125 2187 3019 3064 3105 3109 3150
MIN018_5
16
E= -1915.19896877 ZPE= 60.96 Gcorr 0.032996000
H 0.165997 -1.827085 -2.017334
Co 0.461226 0.075043 -0.180186
C 2.180189 -0.333492 -0.331792
C 0.147370 1.823102 0.010801
O 3.289325 -0.564071 -0.519716
O -0.015064 2.957498 0.046046
C 0.208919 -0.468608 1.528341
O 0.142339 -0.786012 2.627051
C -1.309849 -0.572932 -1.056798
C -0.337999 -1.592075 -1.086758
H -2.285439 -1.353306 0.742125
H -1.516668 0.012354 -1.947862
H 0.764160 0.557302 -1.544053
H -0.419958 -2.425428 -0.392914
C -2.419231 -0.609947 -0.080246
O -3.413799 0.084300 -0.137908
FREQS= 54 66 70 87 93 123 129 253 313 354 370 387 416 433 448 474 495 507 544 553 607
754 824 844 918 1007 1012 1023 1178 1235 1369 1448 1544 1804 2022 2124 2138 2177 2861 3149 3196 3242

MIN019_5
16
E= -1915.19564184 ZPE= 62.02 Gcorr 0.032849000
H -2.352836 1.854650 0.369743
Co 0.548860 -0.001739 -0.153088
C 0.404896 -1.717747 0.320812
C 2.311320 -0.083409 -0.379249
O 0.431947 -2.740755 0.844774
O 3.423362 -0.102318 -0.665058
C 0.563209 1.589374 0.609944
O 0.559876 2.533101 1.265473
C -2.043029 1.589582 -0.644151
C -1.350078 0.232700 -0.665417
H -2.955820 1.562067 -1.251286
H -1.404655 2.386146 -1.034493
H -0.912009 -0.010197 -1.665412
H -1.939778 -1.889858 -0.592757
C -2.263354 -0.872436 -0.270507
O -3.289172 -0.725558 0.364686
FREQS= 28 50 78 95 100 116 141 206 261 275 314 319 351 393 443 471 494 503 526 581 587 696 831 912 957 1081 1103 1140 1313 1418 1430 1498 1508 1794 2094 2108 2168 2828 2874 3045 3109 3141
MIN020_5
16
E= -1915.19343407 ZPE= 60.37 Gcorr 0.031218000
H -3.054956 -1.086610 -0.256210
Co 0.234017 -0.147872 -0.096734
C 0.867266 -0.136903 1.586362
C -0.040810 -1.919809 -0.365590
O 1.272560 -0.143120 2.659516
O -0.341401 -2.999689 -0.610005
C 1.690266 0.421217 -0.950809
O 2.575826 0.797918 -1.575563
C -2.858142 -0.017274 -0.379171
C -1.665181 0.423288 0.449220
H -2.694418 0.170707 -1.443011
H -3.755637 0.531449 -0.068422
H -0.280783 -0.031057 -1.486320
H -1.790398 0.361117 1.528550
C -0.859432 1.498049 0.035092
O -0.700245 2.649332 -0.213121
FREQS= 56 62 67 87 90 107 177 192 252 296 316 374 402 427 441 464 476 513 526 533 562 620 722 823 875 898 1041 1073 1139 1353 1423 1496 1511 1974 2002 2121 2130 2174 3038 3105 3133 3156
MIN021_5
16
E= -1915.19384939 ZPE= 61.84 Gcorr 0.031681000
H 2.574276 2.346809 -1.076454
Co -0.482361 -0.032958 -0.115779
C -2.136498 -0.580068 -0.463691
C 0.078193 -1.652850 0.430592
O -3.177759 -0.899834 -0.828438
O 0.300251 -2.629789 0.987203
C -0.975066 1.522251 0.540628
O -1.266043 2.461206 1.138728
C 1.338984 0.644792 -0.494455
C 1.653330 2.142482 -0.514185
H 3.171743 0.424208 0.699373
H 1.096622 0.263410 -1.513474
H 0.857018 2.725031 -0.983267
H 1.800533 2.536195 0.497312
C 2.496538 -0.144135 0.017848
O 2.742383 -1.306661 -0.247227
FREQS= 16 52 83 89 102 118 132 207 218 275 283 320 388 402 430 470 485 495 517 575 584 598 858 913 978 1082 1125 1142 1275 1425 1434 1505 1518 1785 2096 2114 2172 2871 2878 3029 3091 3130
MIN022_5
16
E= -1915.19606354 ZPE= 61.91 Gcorr 0.034036000
H 2.148957 0.000263 -1.484831
Co -0.093242 0.000005 -0.046262
C -1.886371 0.000068 0.258548
C -0.085131 1.553967 -0.922526
O -3.017916 0.000164 0.440560
O -0.068893 2.557440 -1.485002
C -0.085229 -1.553707 -0.922981
O -0.069087 -2.557031 -1.485723
C 0.302953 0.694569 1.922434
C 0.303011 -0.695549 1.922089
H 1.245378 1.227509 1.885662

H -0.546055 1.244502 2.317690
H 1.245476 -1.228393 1.885007
H -0.545948 -1.245774 2.317041
C 1.858913 0.000116 -0.408412
O 2.721002 0.000048 0.434364
FREQS= 53 63 73 86 98 102 181 226 233 292 360 371 419 419 431 454 469 504 512 536 536 611 725 838 898 1005 1006 1024 1242 1277 1372 1470 1569 1794 2097 2113 2159 2844 3157 3165 3253 3270
MIN023_5
16
E= -1915.19487928 ZPE= 61.65 Gcorr 0.033145000
H -0.189448 -1.249839 2.394078
Co 0.113725 0.000005 0.060881
C -1.770196 0.000193 0.666387
C -0.320873 1.529098 -0.761720
O -2.761384 0.000104 -0.012360
O -0.604175 2.511521 -1.284308
C -0.321225 -1.529178 -0.761347
O -0.604842 -2.511628 -1.283712
C 0.640980 -0.698877 1.963290
C 0.641069 0.699471 1.963042
H 1.579626 -1.243726 1.942108
H -1.860841 0.000397 1.784813
H 1.579788 1.244189 1.941638
H -0.189255 1.250708 2.393681
C 1.848101 -0.000297 -0.494810
O 2.933203 -0.000537 -0.863264
FREQS= 50 54 71 87 97 106 126 212 286 314 341 366 404 416 422 473 479 501 506 534 538 633 729 840 893 985 991 998 1237 1260 1363 1490 1571 1809 2113 2121 2168 2762 3151 3157 3226 3245
MIN024_5
16
E= -1915.19302634 ZPE= 60.38 Gcorr 0.031350000
H -3.704187 0.292741 -0.461829
Co 0.343158 0.091549 -0.148481
C 1.808576 -0.917938 -0.080163
C 0.753162 1.834692 -0.431141
O 2.750663 -1.571401 -0.128820
O 0.957686 2.920453 -0.740466
C -0.057491 0.090517 1.606599
O -0.271804 0.095779 2.733495
C -2.751215 0.556286 0.013006
C -1.593437 0.083550 -0.848686
H -2.729370 1.642039 0.146257
H -2.737550 0.097817 1.005402
H 0.682132 -0.036886 -1.589296
H -1.601738 0.426616 -1.879922
C -1.060003 -1.204375 -0.678623
O -1.158057 -2.388649 -0.701406
FREQS= 53 66 67 89 94 113 175 195 256 284 310 377 410 425 432 459 483 513 521 546 561 608 720 842 867 885 1041 1070 1140 1351 1426 1499 1513 1975 2001 2120 2129 2173 3039 3104 3130 3173
MIN025_5
16
E= -1915.18869034 ZPE= 59.74 Gcorr 0.029328000
H -2.536121 -1.113241 0.639905
Co 0.379297 -0.030899 -0.291478
C 1.586360 1.208791 0.105149
C -0.781328 -0.252553 -1.618993
O 2.441934 1.961016 0.271285
O -1.427522 -0.427197 -2.555476
C 1.047509 -1.645056 0.164457
O 1.446807 -2.672496 0.486967
C -1.936968 -1.040658 1.550968
C -0.828451 -0.009071 1.408578
H -2.605559 -0.774156 2.377837
H -1.517280 -2.029668 1.753243
H 1.246386 -0.012454 -1.490817
H -0.198747 0.149470 2.281396
C -1.053148 1.141741 0.668568
O -1.565410 2.163071 0.376720
FREQS= 51 54 73 76 98 104 173 190 213 259 285 324 382 407 438 464 474 495 515 529 553 605 660 705 870 882 1044 1072 1131 1352 1427 1498 1510 2012 2039 2095 2108 2161 3044 3114 3137 3165
MIN026_5
16
E= -1915.18290806 ZPE= 59.95 Gcorr 0.025962000
H 4.897797 0.218831 -1.102189
Co -0.602500 0.002726 -0.112623
C -0.840580 1.646728 -0.674958
C -1.161224 -1.438312 -0.944271

O -1.036052 2.667284 -1.179472
O -1.553576 -2.297214 -1.608842
C -1.794320 0.007930 1.221004
O -2.613896 0.021795 2.026101
C 2.981583 0.011088 -0.208583
C 4.322394 -0.008021 -0.210933
H 2.848503 -0.535730 1.901457
H 0.225121 0.023065 -1.357556
H 4.886449 -0.253323 0.686069
H 2.399042 0.251721 -1.092669
C 2.243605 -0.296455 1.009085
O 1.016252 -0.306355 1.119419
FREQS= 11 46 56 78 83 92 121 129 222 254 316 338 346 391 449 480 495 520 551 571 592
616 740 758 942 998 1013 1038 1192 1301 1402 1471 1683 1717 1970 2074 2101 2153 3001 3159 3208 3255
MIN027_5
16
E= -1915.18470079 ZPE= 62.77 Gcorr 0.027980000
H -5.203583 -0.000586 -0.449519
Co 0.736537 0.001228 0.044533
C -0.839727 0.000957 0.528834
C 1.998860 0.039586 1.319537
O -2.073932 0.000631 0.808985
O 2.730755 0.065175 2.206546
C 1.085410 1.431803 -0.968103
O 1.232688 2.364342 -1.628127
C -4.446136 0.025425 0.339867
C -3.074831 -0.034871 -0.290614
H -4.609985 -0.826968 1.003721
H -4.574368 0.948035 0.911219
H -2.900278 -0.960951 -0.842913
H -2.864007 0.820913 -0.935792
C 1.107989 -1.468520 -0.902071
O 1.270029 -2.427632 -1.519129
FREQS= 9 24 27 63 70 83 84 149 255 283 301 304 332 443 477 483 502 525 526 586 621
690 812 832 1002 1125 1165 1295 1370 1431 1497 1510 1516 1585 2091 2098 2152 3064 3087 3138 3139 3161
MIN028_5
16
E= -1915.18786420 ZPE= 62.12 Gcorr 0.031884000
H 0.979126 1.256018 1.955746
Co -0.438537 0.004338 0.080287
C -0.832352 1.721792 0.205191
C -1.684048 -0.385279 -1.134278
O -1.164570 2.797672 0.445210
O -2.465837 -0.638099 -1.936090
C -0.625590 -1.485229 1.009484
O -0.815105 -2.364561 1.726724
C 2.266970 0.752212 0.270979
C 1.137241 0.420429 1.270490
H 2.134723 1.773686 -0.115001
H 3.272462 0.681436 0.702002
H 1.080465 -0.205358 -1.323806
H 1.428747 -0.435953 1.882474
C 2.146881 -0.167277 -0.913584
O 3.006807 -0.835866 -1.425700
FREQS= 28 45 54 79 95 99 108 153 202 314 330 394 407 465 487 494 512 527 568 584 589
761 816 963 972 1047 1137 1183 1265 1306 1403 1468 1501 1860 2088 2108 2166 2556 3022 3079 3091 3131
MIN029_5
16
E= -1915.18188026 ZPE= 59.85 Gcorr 0.026525000
H 5.621704 0.000002 -0.218180
Co -0.814122 0.000001 -0.209514
C -1.570206 1.566555 -0.440378
C -1.570201 -1.566556 -0.440385
O -2.175636 2.525660 -0.655869
O -2.175627 -2.525662 -0.655879
C -0.733412 -0.000004 1.566471
O -0.764840 -0.000007 2.718271
C 3.531977 0.000005 -0.603358
C 4.615436 -0.000001 0.186841
H 2.119616 -0.000007 1.070929
H -1.144326 0.000005 -1.664972
H 4.523824 -0.000008 1.270297
H 3.601924 0.000013 -1.687583
C 2.192987 0.000001 -0.031844
O 1.173485 0.000005 -0.723733
FREQS= 29 42 63 65 86 88 124 133 195 209 321 338 350 383 453 479 502 518 554 562 610
620 724 734 946 1001 1017 1042 1200 1307 1395 1469 1685 1718 1980 2077 2092 2145 2989 3162 3203 3258

MIN030_5
16
E= -1915.18832330 ZPE= 62.73 Gcorr 0.033393000
H 3.761474 -0.120779 0.336210
Co -0.308397 -0.058321 0.009505
C -0.133798 -1.381332 -1.193898
C -1.122995 -0.818508 1.434684
O 0.104291 -2.228329 -1.936147
O -1.584334 -1.266750 2.388746
C -1.518638 0.982218 -0.762847
O -2.261968 1.717725 -1.249424
C 2.833645 -0.495093 -0.115777
C 1.631129 -0.059693 0.702191
H 2.780714 -0.118616 -1.141033
H 2.905306 -1.586449 -0.162858
H 0.640847 2.928966 -0.034456
H 1.657709 -0.371137 1.745452
C 0.929818 1.123887 0.400439
O 1.350225 2.369082 0.323239
FREQS= 29 63 68 78 78 86 127 168 254 294 315 408 413 436 459 469 486 506 524 543 570
660 700 879 912 1050 1067 1143 1318 1347 1417 1495 1506 1574 2085 2093 2143 3030 3096 3124 3142 3730
MIN031_5
16
E= -1915.18460865 ZPE= 62.88 Gcorr 0.029687000
H 3.129121 -1.314830 0.499705
Co -0.675031 0.001928 -0.038661
C -0.638528 -1.531393 0.879128
C -1.186570 1.314935 1.060945
O -0.531304 -2.516709 1.466631
O -1.441661 2.187040 1.768984
C -2.052819 -0.183016 -1.171274
O -2.869699 -0.286121 -1.974612
C 3.796731 -0.583414 0.037286
C 3.092543 0.739124 -0.172977
H 4.652952 -0.430380 0.702296
H 4.163602 -0.986446 -0.909918
H 3.726539 1.484428 -0.653825
H 2.671277 1.148566 0.747516
C 0.803780 0.318588 -0.697505
O 1.966605 0.565497 -1.130446
FREQS= 17 25 36 62 71 82 85 163 257 281 304 328 395 439 477 483 492 526 527 583 602
670 816 827 991 1113 1182 1302 1366 1430 1496 1501 1513 1591 2090 2097 2151 3060 3098 3135 3144 3172
MIN032_5
16
E= -1915.19113093 ZPE= 63.35 Gcorr 0.036292000
H -2.256791 -2.073429 1.184884
Co 0.504092 -0.117020 0.002237
C 1.169800 1.215222 0.897235
C 1.974130 -1.156415 -0.113844
O 1.572712 2.078760 1.544239
O 2.929017 -1.744469 -0.346482
C -0.695939 0.987072 -1.042638
O -0.663570 2.038854 -1.602025
C -2.645564 -0.168627 0.214900
C -2.554194 -1.058655 1.459006
H -3.456813 -0.520524 -0.438337
H -2.892979 0.865040 0.479358
H -1.816956 -0.666522 2.167951
H -3.520290 -1.106082 1.969021
C -1.397776 -0.176968 -0.638262
O -0.684333 -1.271735 -0.855941
FREQS= 38 50 74 84 95 116 190 240 255 278 316 391 427 429 474 486 513 542 591 607 638
753 789 987 1058 1083 1199 1293 1350 1382 1423 1479 1507 1518 1952 2107 2162 3030 3051 3089 3122 3146
MIN033_5
16
E= -1915.19034845 ZPE= 63.31 Gcorr 0.035782000
H 3.531078 0.647103 0.166720
Co -0.621733 -0.101681 -0.093913
C -0.879826 1.592519 -0.378424
C -2.331136 -0.675561 -0.173977
O -1.023899 2.708781 -0.623444
O -3.412989 -1.029082 -0.047574
C 0.879277 0.170257 1.094424
O 1.181674 0.906043 1.982926
C 2.354254 -0.961044 -0.727402
C 3.169863 0.332854 -0.816722
H 1.978973 -1.269541 -1.709895

H 2.995059 -1.781000 -0.373140
H 4.039027 0.187445 -1.463111
H 2.575700 1.151746 -1.235000
C 1.187930 -0.914421 0.231687
O 0.178309 -1.767993 0.159660
FREQS= 34 40 65 84 96 140 157 240 265 301 306 402 413 457 464 477 493 538 588 616 642
727 793 974 1071 1086 1257 1273 1311 1382 1434 1486 1515 1518 1946 2107 2162 3030 3055 3078 3127 3137
MIN034_5
16
E= -1915.18605385 ZPE= 62.11 Gcorr 0.032109000
H 3.838980 0.829767 0.950597
Co -0.523042 0.000505 -0.122049
C -0.264245 -1.594468 0.582911
C -0.319878 1.595543 0.622988
O -0.063843 -2.560947 1.179706
O -0.148433 2.556739 1.233308
C -2.315881 -0.034692 -0.148417
O -3.461426 -0.059268 -0.224412
C 2.548117 0.041977 -0.583097
C 3.201645 -0.043489 0.765330
H 0.713090 -0.691815 -2.517885
H 3.198841 0.115213 -1.455699
H 2.458993 -0.096444 1.565715
H 3.844528 -0.929039 0.841514
C 1.235826 0.047231 -0.777750
O 0.617975 0.149235 -2.046192
FREQS= 36 41 75 84 86 98 156 161 238 262 304 321 390 404 449 464 474 502 525 537 557
579 582 816 911 1034 1063 1126 1182 1308 1428 1494 1504 1754 2081 2103 2162 3032 3082 3124 3139 3780
MIN035_5
16
E= -1915.18623576 ZPE= 62.43 Gcorr 0.032679000
H 4.158145 -1.073938 -0.651340
Co -0.524817 -0.026994 -0.046727
C -0.793953 1.711376 0.225582
C -2.094127 -0.334980 -0.844001
O -1.030161 2.789651 0.544094
O -3.050089 -0.563409 -1.439559
C -0.494009 -1.488612 0.937078
O -0.531601 -2.347075 1.703788
C 3.395284 -0.465551 -1.156530
C 2.020023 -0.706311 -0.586440
H 3.698350 0.584787 -1.081887
H 3.430243 -0.733504 -2.217960
H 1.555666 -1.657600 -0.829248
H 2.860531 1.176746 0.771787
C 1.334644 0.163146 0.198131
O 1.894345 1.265577 0.770098
FREQS= 30 55 75 83 94 98 128 146 233 295 321 331 381 394 424 469 484 504 511 575 582
613 705 761 947 1037 1061 1136 1305 1355 1420 1494 1506 1621 2091 2105 2165 3019 3078 3104 3186 3746
MIN036_5
16
E= -1915.18354904 ZPE= 61.35 Gcorr 0.030881000
H -1.969514 -0.925654 1.739989
Co -0.138779 -0.000456 -0.062119
C -0.224242 -1.681017 -0.659958
C 1.589913 0.006406 -1.025780
O -0.282955 -2.775552 -1.015612
O 2.664819 0.009507 -0.487681
C 0.680329 0.003374 1.555419
O 1.206448 0.005902 2.574448
C -2.323478 -0.010188 -0.170122
C -1.948497 -0.007529 1.162968
H -2.643612 -0.925809 -0.653986
H -2.651552 0.901845 -0.655453
H 1.520634 0.007283 -2.136842
H -1.977537 0.911244 1.738614
C -0.238525 1.679122 -0.660701
O -0.306359 2.772941 -1.016912
FREQS= 43 43 66 84 89 106 124 128 211 265 322 328 413 428 462 465 468 479 484 519 559
607 698 842 898 976 1005 1006 1237 1288 1346 1489 1586 1799 2077 2103 2154 2862 3167 3177 3246 3269
MIN037_5
16
E= -1915.18541446 ZPE= 61.64 Gcorr 0.032843000
H 3.727082 -0.006471 -0.833676
Co -0.208367 0.107551 -0.225647
C -0.239162 -1.470041 0.815037
C -0.127209 1.411954 0.984518

O -0.301750 -1.733806 1.973284
O -0.101316 2.150169 1.865450
C -1.967665 0.230047 -0.674430
O -3.047828 0.325933 -1.045667
C 2.930833 -0.192672 -0.108066
C -0.136091 -1.874060 -0.546412
H 2.904439 -1.251519 0.166978
H 3.126568 0.367659 0.812894
H 0.812680 -2.316781 -0.840119
H -1.009384 -2.341190 -0.993241
C 1.597509 0.231114 -0.645870
O 1.415298 0.836001 -1.689194
FREQS= 53 61 76 84 86 106 123 170 233 293 304 394 406 419 446 463 474 504 535 555 584
610 763 872 947 979 993 1022 1158 1388 1441 1465 1475 1751 1954 2109 2155 3048 3122 3130 3148 3214
MIN038_5
16
E= -1915.18356391 ZPE= 62.10 Gcorr 0.031661000
H 3.067608 0.942958 -2.130073
Co -0.268026 0.128096 -0.137370
C -0.862820 1.622861 0.504493
C -1.804581 -0.323818 -0.995481
O -1.232473 2.599697 0.984122
O -2.767640 -0.549342 -1.573288
C 0.028574 -1.559100 0.936892
O -0.156853 -1.885560 2.056605
C 2.539908 0.628747 -0.067232
C 2.717515 0.133962 -1.476244
H 1.456375 1.113465 1.640843
H 3.458201 0.987118 0.406671
H 3.475893 -0.656936 -1.522338
H 1.791887 -0.263470 -1.901716
C 1.397546 0.689261 0.638828
O 0.393200 -1.756446 -0.221432
FREQS= 27 38 55 85 86 114 144 169 212 231 312 342 398 406 455 474 486 529 553 575 626
644 697 927 988 1061 1069 1210 1251 1358 1422 1486 1493 1645 2111 2125 2164 3030 3082 3094 3120 3144
MIN039_5
16
E= -1915.18367183 ZPE= 60.90 Gcorr 0.031986000
H -0.946013 -2.243151 -0.946322
Co 0.148235 -0.020425 0.000189
C -0.383609 -0.826782 1.502645
C 1.956528 -0.128961 -0.260170
O -0.733425 -1.396834 2.435358
O 3.061975 -0.250170 -0.540249
C 0.110507 1.647276 0.665691
O 0.107822 2.711689 1.093409
C 0.085278 -1.944391 -0.744981
C -0.488954 0.606749 -1.845675
H 0.521155 -2.670604 -0.056048
H 0.651105 -1.976994 -1.679004
H -0.404240 1.669952 -2.049065
H -0.203768 -0.060186 -2.652420
C -1.543231 0.202581 -1.015090
O -2.691333 -0.002982 -0.793115
FREQS= 59 64 78 90 96 125 126 163 253 285 355 390 405 427 432 441 464 494 502 519 534
555 575 763 836 861 872 985 1028 1285 1430 1482 1489 1991 2117 2120 2169 3054 3146 3149 3150 3242
MIN040_5
16
E= -1915.18167710 ZPE= 60.13 Gcorr 0.030044000
H 1.509375 -0.244344 -1.879335
Co -0.526693 -0.086829 -0.164556
C -2.242226 0.330674 -0.476678
C -0.203424 -1.760236 0.269624
O -3.348724 0.485992 -0.735428
O -0.044374 -2.879875 0.481664
C -0.304423 0.702523 1.454860
O -0.207869 1.191984 2.485084
C 2.490940 0.631059 -0.130388
C 3.605647 -0.104630 -0.217729
H -0.772557 -0.802620 -1.440990
H 2.391936 1.419970 0.612658
H 3.712163 -0.884564 -0.967824
H 4.446499 0.049401 0.449825
C 1.357468 0.469610 -1.054266
O 0.439639 1.350965 -1.156803
FREQS= 45 57 62 81 85 115 128 185 279 301 335 365 399 423 453 459 491 507 526 548 567
664 744 815 923 960 975 1027 1181 1306 1359 1439 1542 1697 1994 2112 2136 2175 3015 3157 3177 3251

MIN041_5
16
E= -1915.18056225 ZPE= 59.36 Gcorr 0.029131000
H -2.883927 1.121612 -1.073387
Co 0.297022 -0.012684 -0.337967
C -0.703295 0.265119 1.394398
C -0.689752 -1.313069 -1.021291
O -0.659003 -0.194348 2.490584
O -1.301938 -2.135149 -1.541312
C 1.614951 -1.003768 0.381006
O 2.445686 -1.629222 0.865747
C -2.738450 0.797409 -0.038798
C -1.317743 1.068466 0.424725
H -3.446835 1.346450 0.593488
H -2.991390 -0.263922 0.018823
H 0.449142 0.135804 -1.838598
H -1.043151 2.118579 0.489447
C 1.184930 1.489296 -0.604286
O 1.739343 2.466624 -0.849917
FREQS= 40 61 87 90 101 114 170 185 235 267 283 351 359 411 433 464 471 497 527 540 560 595 607 622 863 891 1036 1067 1139 1351 1423 1497 1510 1855 1974 2109 2122 2168 3040 3107 3136 3166
MIN042_5
16
E= -1915.18131670 ZPE= 60.12 Gcorr 0.029892000
H 0.080957 -0.156945 -1.499124
Co -0.447120 -0.006124 -0.121435
C -1.166671 0.282889 1.520429
C -1.879257 -0.646299 -0.982518
O -1.648970 0.478889 2.539717
O -2.751119 -0.999291 -1.639248
C 0.042973 1.635236 -0.522184
O 0.359980 2.681446 -0.880786
C 3.687116 -0.087830 -0.079450
C 2.553160 -0.735181 -0.371732
H 3.815437 0.431198 0.867549
H 4.522897 -0.056736 -0.770011
H 2.423010 -1.261668 -1.314275
H 1.604303 -0.379345 1.566665
C 1.429245 -0.824770 0.572770
O 0.493388 -1.680472 0.430824
FREQS= 45 54 65 79 83 114 130 186 273 303 333 366 404 422 453 459 491 506 528 544 567 665 745 820 922 959 969 1029 1181 1302 1359 1440 1545 1699 2003 2112 2137 2176 2993 3157 3187 3250
MIN043_5
16
E= -1915.18423160 ZPE= 62.47 Gcorr 0.032907000
H 2.804318 -1.755164 -0.130062
Co -0.309721 -0.043990 0.012636
C -1.174006 -0.685381 1.466390
C -1.452699 1.052219 -0.798086
O -1.658956 -1.055961 2.442072
O -2.159421 1.799938 -1.312755
C -0.244683 -1.420147 -1.141741
O -0.071327 -2.309118 -1.852600
C 2.795558 -0.660585 -0.118624
C 1.623110 -0.130942 0.692192
H 3.754825 -0.334624 0.308197
H 2.752343 -0.320073 -1.157369
H 2.300022 2.436965 0.485614
H 1.645465 -0.404963 1.746660
C 0.984058 1.083481 0.348094
O 1.379386 2.331857 0.187839
FREQS= 27 65 68 76 86 87 125 161 254 296 316 406 411 436 459 470 484 502 524 542 567 585 698 879 905 1048 1061 1128 1310 1362 1418 1494 1509 1547 2091 2099 2149 3016 3088 3119 3134 3699
MIN044_5
16
E= -1915.18077780 ZPE= 61.56 Gcorr 0.029804000
H 2.483164 -0.084504 -1.319979
Co -0.599937 -0.002733 0.063369
C 0.898737 -0.252456 1.040537
C -0.138367 -1.115962 -1.186829
O 0.327141 -0.224596 2.115623
O 0.202659 -1.752884 -2.089773
C -0.362713 1.724720 -0.396721
O -0.128192 2.716952 -0.932009
C 2.962318 0.233481 -0.391014
C 2.370580 -0.488614 0.821416
H 4.029519 0.010519 -0.470844

H 2.850330 1.317741 -0.302233
H 2.885727 -0.217214 1.749448
H 2.477652 -1.576008 0.703830
C -2.415363 -1.067798 0.462756
O -2.704015 0.063407 0.384652
FREQS= 31 52 67 79 87 99 126 131 170 220 258 270 316 359 371 372 455 491 511 547 566
580 782 943 1049 1081 1158 1280 1340 1434 1461 1514 1516 1748 1925 2070 2123 3038 3064 3094 3136 3146
MIN045_5
16
E= -1915.18282733 ZPE= 62.16 Gcorr 0.032003000
H -2.953669 -1.875703 1.504278
Co 0.454452 -0.031916 0.015453
C 2.071808 -0.661220 0.392879
C -0.068761 -1.401051 -0.968179
O 3.088310 -1.100913 0.699306
O -0.376263 -2.199103 -1.736073
C 0.901910 1.695588 0.102084
O 1.220064 2.794298 0.024378
C -2.353210 0.034170 0.684915
C -2.182401 -1.108082 1.646789
H -2.602529 1.734899 -1.016512
H -3.339142 0.511029 0.674525
H -1.205436 -1.592289 1.533124
H -2.252039 -0.780109 2.692156
C -1.398570 0.532221 -0.121718
O -1.649865 1.544987 -1.015791
FREQS= 26 41 71 89 96 104 144 174 234 287 299 347 382 412 423 466 473 485 489 543 571
594 605 791 941 1058 1115 1145 1255 1369 1429 1483 1507 1666 2101 2112 2178 3016 3066 3076 3091 3725
MIN046_5
16
E= -1915.18518347 ZPE= 62.91 Gcorr 0.035072000
H 1.406920 -0.367218 1.771471
Co -0.454215 0.019058 0.077906
C 0.134340 -1.242199 -1.016821
C -1.888076 -0.842163 0.715583
O 0.422295 -1.975358 -1.861064
O -2.786091 -1.395621 1.172521
C -1.298830 1.435691 -0.556157
O -1.833335 2.298301 -1.106830
C 3.482434 -0.234604 0.373394
C 2.534236 0.460654 -0.264756
H 3.424595 -0.482202 1.431337
H 4.377268 -0.567789 -0.139948
H 2.675067 0.720750 -1.312202
H 1.150875 2.010642 0.293333
C 1.254834 0.925747 0.301148
O 0.936563 0.466741 1.612647
FREQS= 48 59 75 81 88 100 127 230 256 299 302 392 408 466 481 509 519 533 556 559 598
637 730 887 911 1011 1025 1091 1131 1235 1319 1399 1470 1701 2069 2088 2148 3134 3149 3173 3245 3765
MIN047_5
16
E= -1915.17054280 ZPE= 59.13 Gcorr 0.020869000
H 4.617356 -0.254681 1.263943
Co -0.892505 0.002787 -0.207018
C -0.465569 -0.147628 1.509301
C -1.586391 1.610242 -0.164292
O -0.297422 -0.246086 2.643716
O -2.182848 2.597975 -0.169694
C -1.629028 -1.571866 -0.433527
O -2.253196 -2.527252 -0.601761
C 4.342257 -0.484472 0.229573
C 3.382026 0.555123 -0.309043
H 5.259086 -0.505407 -0.368437
H 3.903893 -1.484530 0.210719
H 3.677355 1.600138 -0.355936
H -1.525661 0.125211 -1.557785
C 2.174793 0.287770 -0.736286
O 1.090602 0.043987 -1.144433
FREQS= 6 33 49 54 83 89 104 111 148 178 219 324 339 353 456 485 494 500 518 562 570
584 635 674 744 904 1056 1081 1156 1413 1428 1496 1519 1971 2081 2099 2156 2198 3049 3112 3142 3190
MIN048_5
16
E= -1915.17810959 ZPE= 61.58 Gcorr 0.028588000
H 2.562837 -0.782823 -1.219690
Co -0.508644 -0.002336 0.103326
C 1.019839 -0.023170 1.054857
C -0.221529 -1.508284 -0.752733

O 0.477664 -0.022188 2.151284
O 0.059661 -2.361782 -1.483805
C -0.224796 1.535832 -0.699663
O 0.051740 2.413419 -1.403081
C 2.959108 0.044627 -0.625469
C 2.511647 -0.053741 0.830848
H 4.050419 0.011540 -0.686932
H 2.623779 0.979203 -1.082634
H 2.931548 0.759180 1.437399
H 2.865043 -0.985754 1.292339
C -3.660518 -0.011503 0.453222
O -2.539407 -0.007055 0.223520
FREQS= 25 49 53 72 76 86 110 154 204 209 223 238 307 311 374 408 460 483 540 546 563
570 767 952 1064 1082 1148 1280 1338 1438 1452 1514 1520 1725 2042 2098 2131 3038 3064 3077 3135 3144
MIN049_5
16
E= -1915.17173059 ZPE= 59.18 Gcorr 0.022546000
H -5.336329 -0.298643 0.524326
Co 0.734306 -0.012099 -0.136586
C 1.352304 1.479313 -0.816579
C 1.829621 -0.138012 1.261783
O 1.805718 2.371113 -1.391793
O 2.608267 -0.232511 2.102048
C 0.864123 -1.659683 -0.716806
O 1.019450 -2.681128 -1.231097
C -4.447922 -0.426465 -0.102569
C -3.451075 0.682811 0.159483
H -4.021411 -1.408755 0.111250
H -4.767217 -0.416643 -1.149620
H 0.001542 0.061136 -1.441705
H -3.731856 1.720167 -0.003509
C -2.224090 0.485074 0.565841
O -1.122029 0.308923 0.963363
FREQS= 16 32 49 66 77 87 105 118 149 181 222 321 338 358 455 485 492 497 518 560 576
585 636 684 748 904 1055 1081 1156 1413 1428 1496 1519 1956 2078 2103 2159 2195 3050 3112 3143 3190
MIN050_5
16
E= -1915.17983894 ZPE= 62.02 Gcorr 0.030884000
H -2.778020 -1.870021 1.712337
Co 0.443594 -0.021241 0.026800
C 2.087433 -0.605868 0.375538
C -0.060497 -1.409345 -0.947041
O 3.134874 -1.008358 0.620305
O -0.358364 -2.233305 -1.690634
C 0.832150 1.710551 0.117236
O 1.092812 2.826380 0.027164
C -2.327413 -0.002105 0.721746
C -2.041983 -1.057137 1.752920
H -1.113741 1.706465 -1.604818
H -3.333566 0.418310 0.698649
H -1.047622 -1.504146 1.621338
H -2.071007 -0.655753 2.774303
C -1.432345 0.481024 -0.155801
O -1.866465 1.387274 -1.095961
FREQS= 19 47 72 83 96 96 138 186 189 246 289 310 350 389 410 467 472 483 496 535 568
591 597 813 935 1057 1104 1177 1210 1339 1427 1480 1507 1685 2093 2112 2172 3008 3062 3074 3140 3862
MIN051_5
16
E= -1915.17726155 ZPE= 60.39 Gcorr 0.028523000
H -1.785916 2.906120 -1.776735
Co 0.068249 0.133672 -0.068990
C 0.821608 -1.564238 -0.196771
C 1.587140 1.056158 -0.522095
O 1.839936 -2.106177 0.089060
O 2.543412 1.648740 -0.739605
C -0.141296 0.419087 1.660498
O -0.208813 0.513570 2.804156
C -1.681000 2.248296 -0.911637
C -0.362130 -1.668397 -0.996283
H -1.365132 2.828841 -0.044849
H -2.617956 1.732835 -0.704344
H -0.913393 1.500025 -1.188363
H -0.354459 -2.000222 -2.032027
C -1.429810 -1.013593 -0.330532
O -2.621152 -0.986211 -0.229864
FREQS= 56 63 73 79 93 102 105 130 145 222 280 310 323 348 359 401 417 467 490 510 527
575 626 673 740 974 1073 1227 1322 1341 1376 1540 1575 1913 1959 2112 2161 2920 3096 3171 3176 3197

```
MIN052_5
16
E= -1915.17537823 ZPE=    59.89 Gcorr    0.028891000
H -4.464696 0.119296 -1.196158
Co 0.534496 0.007620 -0.152810
C 0.555540 1.758657 0.000650
C 0.617366 -0.472295 1.617226
O 0.547697 2.906596 0.014586
O 0.672448 -0.757837 2.721152
C 2.114894 -0.634950 -0.778110
O 3.090096 -0.978380 -1.267971
C -3.902764 -0.381484 -0.398180
C -2.573395 0.297146 -0.157473
H -4.528124 -0.361889 0.502843
H -3.767135 -1.426140 -0.689515
H 0.442743 0.477762 -1.550322
H -2.560246 1.338360 0.141314
C -1.413945 -0.333432 -0.305246
O -0.802754 -1.389751 -0.587204
FREQS=   48   52   70   81   83  109  145  165  189  260  314  375  392  408  423  446  486  497  507  516  542
667  712  730  840  916 1046 1088 1131 1388 1420 1497 1514 1883 2019 2131 2157 2194 3028 3079 3129 3215
MIN053_5
16
E= -1915.17802846 ZPE=    61.47 Gcorr    0.031693000
H -1.807109 -1.700271 -0.579805
Co 0.508285 -0.008035 -0.015655
C 1.621671 0.573665 -1.260981
C 0.424446 1.534106 0.919595
O 2.324372 0.949272 -2.087686
O 0.408930 2.424799 1.642577
C 1.434839 -1.499972 0.394005
O 2.008494 -2.404789 0.804368
C -2.836111 0.177793 -0.244185
C -3.996176 -0.212227 -0.768518
H -0.902922 -0.247795 -1.059273
H -2.726026 1.160088 0.211775
H -4.118486 -1.197376 -1.212491
H -4.868891 0.433026 -0.766599
C -1.604840 -0.683343 -0.207543
O -0.937202 -0.665639 0.995094
FREQS=   42   45   67   87   90  105  125  145  250  288  340  381  413  435  446  468  492  521  556  568  607
640  910  932  958 1022 1091 1162 1273 1306 1356 1463 1547 1723 2113 2117 2171 2181 2997 3152 3171 3241
MIN054_5
16
E= -1915.17510625 ZPE=    59.90 Gcorr    0.029611000
H 3.934498 0.829167 0.923530
Co -0.474162 0.021424 0.152623
C 0.017566 1.665152 -0.230229
C -0.507910 -0.694217 -1.538969
O 0.309290 2.763345 -0.396928
O -0.520388 -1.126911 -2.595183
C -2.235596 0.003456 0.611631
O -3.312811 0.047073 0.993177
C 3.315048 0.384877 0.134535
C 2.524963 -0.782809 0.649489
H 2.665524 1.173153 -0.253627
H 3.994426 0.086118 -0.673775
H -0.357305 0.665492 1.477057
H 3.071089 -1.628357 1.067928
C 1.203987 -0.917304 0.660002
O 0.222135 -1.640877 0.951349
FREQS=   36   65   72   84   96  117  142  173  229  308  333  374  403  409  433  449  467  497  501  522  541
570  687  735  847  905 1064 1087 1171 1375 1435 1493 1518 1881 2022 2128 2159 2195 3028 3072 3130 3146
MIN055_5
16
E= -1915.17728580 ZPE=    61.32 Gcorr    0.032510000
H -2.464918 -2.070215 -0.704893
Co 0.137789 -0.166949 -0.146982
C 0.477729 1.678169 0.168015
C 0.024441 -0.883149 1.473580
O 0.787992 2.308911 1.120872
O -0.002579 -1.230204 2.568946
C 1.823082 -0.694917 -0.613820
O 2.833764 -1.095346 -0.979937
C -1.813721 -1.347327 -1.203411
C 0.172511 1.535794 -1.211953
H -0.858709 -1.846985 -1.418927
```

H -2.257928 -1.037662 -2.152215
H -0.782994 1.958314 -1.510392
H 0.993540 1.695167 -1.906394
C -1.707673 -0.089594 -0.294365
O -2.645116 0.593534 0.009252
FREQS= 55 63 73 83 90 102 143 186 208 292 337 391 403 430 447 464 479 491 530 560 572
595 776 788 895 941 993 1002 1063 1353 1432 1459 1473 1852 1982 2107 2153 3021 3104 3131 3155 3222
MIN056_5
16
E= -1915.17647355 ZPE= 61.93 Gcorr 0.031966000
H 3.303658 -0.785227 0.712297
Co -0.126718 -0.368707 0.156133
C -1.902333 -0.821211 0.231699
C 0.263527 0.604622 1.594170
O -2.982196 -1.198324 0.281163
O 0.569222 1.135505 2.566914
C -0.418194 1.207159 -0.837143
O -1.450552 1.505988 -1.389204
C 1.518476 -1.037060 -0.424974
C 2.973877 -0.785185 -0.331360
H 0.273806 -2.555569 -1.566258
H 0.460106 2.704062 -1.555273
H 3.556155 -1.528489 -0.885931
H 3.194499 0.208687 -0.734327
C 0.547047 -1.732339 -0.924753
O 0.705872 1.968794 -0.962865
FREQS= 33 59 75 78 92 98 102 115 242 276 348 356 411 423 451 456 494 512 524 569 621
679 704 746 871 961 1047 1083 1092 1312 1410 1486 1489 1787 1792 2112 2160 3043 3116 3118 3289 3689
MIN057_5
16
E= -1915.17500065 ZPE= 61.48 Gcorr 0.030758000
H 3.026651 -0.995384 0.662504
Co -0.434553 -0.165204 -0.048701
C 1.033610 0.861191 -0.108085
C -1.490824 1.146816 -0.524848
O 1.473299 1.899368 0.284595
O -2.217451 2.027267 -0.684127
C -0.361559 -0.454833 1.711152
O -0.473319 -0.479307 2.856247
C 3.170959 -0.489427 -0.296361
C 1.827191 -0.179856 -0.965128
H 3.717051 0.439107 -0.114680
H 3.776578 -1.136355 -0.937188
H 1.272347 -1.111563 -1.143014
H 1.966579 0.291321 -1.943355
C -1.388006 -1.476127 -1.516328
O -1.129342 -2.131479 -0.583183
FREQS= 42 67 72 83 98 110 129 132 173 226 249 255 320 354 376 404 448 482 517 525 571
671 740 830 980 1061 1094 1275 1313 1422 1461 1507 1515 1877 1929 2089 2138 3036 3059 3094 3133 3149
MIN058_5
16
E= -1915.17301068 ZPE= 61.25 Gcorr 0.028926000
H 3.686388 -0.701392 -0.302766
Co -0.323164 0.028256 -0.011564
C 1.321170 0.651362 -0.132216
C -1.123742 1.407048 -0.759600
O 2.101448 1.497982 0.183375
O -1.641954 2.393678 -1.064094
C -0.359219 -0.111456 1.744586
O -0.438381 -0.004076 2.892121
C 2.837662 -1.378115 -0.429434
C 1.652476 -0.610518 -1.020672
H 3.138601 -2.198578 -1.087608
H 2.586390 -1.796225 0.549470
H 0.785283 -1.287202 -1.149898
H 1.882073 -0.223651 -2.017642
C -2.550220 -2.272902 -0.727024
O -1.773871 -1.471130 -0.478048
FREQS= 43 53 67 71 82 93 128 150 175 182 214 254 276 324 367 386 451 493 518 537 572
668 698 822 984 1060 1094 1276 1316 1421 1458 1508 1516 1888 2066 2119 2141 2946 3056 3095 3130 3144
MIN059_5
16
E= -1915.17592815 ZPE= 60.71 Gcorr 0.032619000
H -0.223166 -2.276745 -1.402802
Co -0.256844 0.044542 -0.098135
C -2.007252 -0.383977 -0.388138
C -0.014489 -0.284986 1.658803

O -3.032326 -0.778098 -0.712553
O 0.130209 -0.525015 2.770523
C -0.344172 1.850716 -0.118947
O -0.343606 2.988299 -0.229943
C 1.763029 -1.479681 -0.812175
C 0.297442 -1.866581 -0.538821
H 2.512137 -2.002656 -0.202489
H 2.055138 -1.583295 -1.865771
H 0.180990 -2.552485 0.301265
H -0.114307 0.182941 -1.577072
C 1.684141 -0.018706 -0.461054
O 2.527198 0.830927 -0.408212
FREQS= 41 69 77 85 92 99 132 278 332 381 409 413 431 446 463 489 515 523 537 555 617
697 749 830 858 987 1012 1067 1174 1232 1273 1434 1499 1856 1975 2134 2144 2186 3027 3080 3104 3168
MIN060_5
16
E= -1915.17357850 ZPE= 62.19 Gcorr 0.030703000
H 3.964467 -0.306637 1.099858
Co -0.445212 -0.009133 0.000628
C -1.404341 -0.575449 -1.423574
C -1.423583 -0.407502 1.464304
O -1.931825 -1.002458 -2.353182
O -1.965458 -0.732755 2.426430
C -0.571739 1.759252 -0.084176
O -0.601771 2.908003 -0.141821
C 0.888665 -1.533523 0.102271
C 3.666966 -0.280385 0.048612
H 0.946942 -2.189274 -0.765076
H 1.005051 -2.046499 1.056007
H 3.533922 -1.293918 -0.333294
H 4.410217 0.263121 -0.534574
C 1.297128 -0.178911 -0.014919
O 2.429246 0.467074 -0.068301
FREQS= 28 33 65 76 79 84 86 155 238 275 315 342 409 425 454 473 478 486 533 547 552
750 788 919 961 989 1115 1161 1199 1420 1467 1494 1507 1570 2091 2099 2150 3065 3106 3155 3178 3180
MIN061_5
16
E= -1915.16346298 ZPE= 59.03 Gcorr 0.020867000
H 0.614224 -1.013193 -0.535193
Co -0.957839 0.033889 -0.206685
C -2.240437 -1.163508 -0.271099
C -1.180886 0.588729 1.464734
O -3.146018 -1.859118 -0.415251
O -1.407711 0.992131 2.516650
C -0.517790 1.566687 -0.945595
O -0.357331 2.558894 -1.505216
C 1.466376 -1.448975 0.040833
C 2.772304 -1.026873 -0.577889
H 1.400215 -1.153760 1.088742
H 1.328537 -2.533845 -0.010147
H -0.950624 -0.329739 -1.661561
H 3.012313 -1.306328 -1.598843
C 3.676473 -0.305036 0.053603
O 4.486153 0.327556 0.617564
FREQS= 10 21 41 51 79 94 98 110 148 211 309 324 336 357 460 487 489 509 515 553 573
580 645 663 761 933 1047 1102 1165 1389 1434 1487 1518 1969 2097 2114 2173 2227 2789 3076 3147 3205
MIN062_5
16
E= -1915.16303396 ZPE= 58.99 Gcorr 0.020565000
H 0.843670 -0.598743 -0.237476
Co -0.962840 0.003018 -0.230625
C -1.916927 -1.420065 -0.619245
C -1.626183 0.354102 1.378228
O -2.604455 -2.267692 -0.984315
O -2.139861 0.612109 2.373441
C -0.745821 1.650434 -0.799372
O -0.709013 2.699720 -1.270206
C 1.607971 -0.980919 0.483563
C 2.729549 0.009022 0.657752
H 1.134712 -1.210417 1.440533
H 1.954021 -1.923009 0.051676
H -0.593538 -0.221201 -1.665866
H 2.581637 0.913491 1.239152
C 3.920997 -0.140604 0.114512
O 4.985663 -0.278313 -0.355641
FREQS= 10 25 41 53 67 92 97 102 134 216 319 328 335 386 460 486 490 509 514 547 572
578 651 653 754 906 1056 1101 1166 1387 1424 1486 1527 1967 2097 2114 2173 2228 2776 3091 3141 3205

MIN063_5
16
E= -1915.16594705 ZPE= 59.80 Gcorr 0.024225000
H -1.256894 2.861478 -1.987694
Co -0.367061 0.100634 -0.064906
C 0.374564 -1.467976 0.027908
C -0.110858 0.682344 1.602015
O 0.453148 -2.591464 0.393484
O -0.052733 0.901884 2.732687
C -2.015727 -0.431033 -0.448746
O -3.097360 -0.812152 -0.569801
C -0.699159 2.571553 -1.095794
C 1.255234 -0.684672 -1.084221
H 0.134213 3.252976 -0.924818
H -1.367005 2.583894 -0.235089
H 1.224307 -1.083158 -2.092516
H -0.283622 1.566993 -1.312808
C 2.340290 -0.036748 -0.667561
O 3.271142 0.539218 -0.268398
FREQS= 29 40 60 69 79 88 100 117 145 155 188 298 318 343 358 378 434 456 483 506 529
554 574 625 705 768 1097 1245 1282 1352 1405 1545 1598 1923 2077 2121 2206 2907 3099 3180 3188 3204
MIN064_5
16
E= -1915.16562156 ZPE= 59.32 Gcorr 0.024251000
H -0.851831 -0.242808 -1.705446
Co -0.636729 -0.176923 -0.221212
C -1.971202 0.942664 -0.430526
C -0.255541 -1.867943 -0.555258
O -2.920689 1.564083 -0.629598
O -0.147939 -2.979509 -0.824133
C -0.606202 -0.242794 1.554713
O -0.656764 -0.325705 2.699280
C 1.576737 1.188978 -0.455762
C 1.507924 2.511007 -0.252007
H 3.540440 1.002325 0.488159
H 0.835255 0.663288 -1.083548
H 2.293400 3.034059 0.289364
H 0.676918 3.109895 -0.608685
C 2.731773 0.400233 0.018128
O 2.824962 -0.806707 -0.080904
FREQS= 18 41 44 67 77 93 100 108 156 260 319 328 343 365 457 482 486 505 571 578 580
629 667 758 920 1003 1027 1132 1154 1277 1397 1460 1683 1800 1958 2097 2119 2176 2910 2941 3157 3253
MIN065_5
16
E= -1915.16215851 ZPE= 59.03 Gcorr 0.020906000
H 0.512889 -2.279557 0.421396
Co -0.784684 0.111294 -0.218249
C -2.302130 -0.717904 -0.529376
C -0.878142 0.227344 1.553358
O -3.343050 -1.124219 -0.805329
O -1.007553 0.367553 2.685920
C -0.076765 1.684626 -0.549173
O 0.233622 2.756015 -0.830447
C 2.534245 -1.589859 0.077995
C 1.136396 -1.905464 -0.396158
H 3.149144 -2.370302 0.515036
H 1.136303 -2.669601 -1.181586
H 0.663454 -1.019310 -0.867074
H -0.849685 0.159837 -1.715260
C 3.081778 -0.395525 -0.003886
O 3.567240 0.669985 -0.074688
FREQS= 8 28 41 56 71 89 97 109 123 226 320 325 338 364 460 486 490 506 508 545 570
580 646 654 760 906 1053 1086 1158 1394 1425 1509 1525 1966 2097 2117 2176 2229 2887 3065 3098 3204
MIN066_5
16
E= -1915.16504248 ZPE= 59.07 Gcorr 0.024044000
H 0.086149 1.059182 1.276570
Co 0.593223 0.157652 0.193755
C 1.427781 -0.893825 -0.978611
C 0.601650 -0.915903 1.588697
O 2.038136 -1.554984 -1.689506
O 0.689743 -1.513323 2.568158
C 1.323447 1.695002 -0.232915
O 1.849741 2.709846 -0.373798
C -2.998700 1.435518 0.336540
C -3.040335 0.189537 -0.147948
H -2.077832 2.012706 0.323921

H -3.873226 1.914149 0.764542
H -3.947514 -0.408978 -0.142568
H -0.942219 0.234132 -0.815943
C -1.856799 -0.453176 -0.736087
O -1.828203 -1.567878 -1.206849
FREQS= 20 41 46 73 78 88 101 110 144 299 310 328 348 359 449 478 488 507 565 576 581 625 669 759 924 1003 1023 1095 1189 1298 1312 1451 1694 1808 1971 2095 2122 2176 2502 3163 3197 3257
MIN067_5
16
E= -1915.17655951 ZPE= 63.21 Gcorr 0.035963000
H -0.563380 -2.413016 0.888916
Co -0.219668 0.092504 -0.000005
C -1.255151 0.015478 -1.467198
C -1.255285 0.015720 1.467103
O -1.887294 -0.129223 -2.418278
O -1.887519 -0.128822 2.418146
C 0.238678 1.810795 -0.000108
O 0.699723 2.868264 -0.000163
C 1.410712 -1.940286 0.000117
C -0.127260 -1.958161 0.000183
H 1.880608 -2.393997 0.885276
H 1.880500 -2.394006 -0.885093
H 2.703187 1.014136 0.000055
H -0.563473 -2.413208 -0.888406
C 1.524833 -0.465543 0.000069
O 2.746894 0.044090 0.000092
FREQS= 38 57 58 74 86 105 129 316 325 362 389 441 457 467 476 489 526 542 547 590 616 676 759 860 995 1053 1061 1153 1220 1265 1348 1417 1431 1500 2081 2092 2140 3008 3052 3106 3170 3741
MIN068_5
16
E= -1915.16975269 ZPE= 60.29 Gcorr 0.030043000
H 0.572819 -2.427315 -0.662124
Co -0.186538 -0.010880 -0.057760
C 0.061390 1.690772 0.583069
C -1.975747 0.137614 -0.423809
O 0.215689 2.744320 0.995757
O -3.099592 0.146682 -0.638293
C 0.087941 -0.864154 1.462480
O 0.269311 -1.471424 2.419871
C -0.380085 -1.909160 -0.774110
C 2.883777 -0.238431 -0.524537
H -0.587617 -1.766560 -1.835884
H -1.174995 -2.499016 -0.313661
H 3.666797 -0.129318 -1.268909
H 3.142250 -0.570688 0.469975
C 1.626833 0.029891 -0.859406
O 0.813668 0.406354 -1.728834
FREQS= 59 60 75 85 90 104 123 154 245 266 342 357 384 406 422 450 460 492 501 508 525 541 556 628 699 824 859 1005 1117 1266 1451 1461 1479 1871 2125 2146 2186 3063 3164 3171 3172 3284
MIN069_5
16
E= -1915.17426354 ZPE= 61.11 Gcorr 0.034865000
H -1.420297 2.094175 -0.428179
Co 0.370590 -0.000023 -0.174676
C 1.444177 -1.431394 -0.090295
C 1.443929 1.431528 -0.090369
O 2.105348 -2.363975 -0.151549
O 2.104879 2.364261 -0.151710
C -0.299340 -0.000016 1.543982
O -0.668433 0.000045 2.622941
C -1.138754 1.177788 -0.946662
C -1.138617 -1.177839 -0.946865
H 0.852006 -0.000031 -1.559308
H -0.903816 1.346832 -1.996454
H -1.420140 -2.094349 -0.428584
H -0.903658 -1.346687 -1.996681
C -2.003109 -0.000104 -0.665294
O -3.049261 -0.000218 -0.032372
FREQS= 70 78 86 87 98 114 148 340 360 394 410 432 440 453 471 500 517 520 534 543 674 779 803 808 873 877 954 1066 1077 1105 1230 1464 1477 1775 2066 2144 2164 2197 3112 3115 3195 3196
MIN070_5
16
E= -1915.16920641 ZPE= 60.67 Gcorr 0.030200000
H -0.879131 -2.719452 -0.271583
Co 0.000003 -0.156797 -0.063955
C 1.739163 -0.417943 -0.294194
C -0.000030 -0.171376 1.735196

O 2.853270 -0.631695 -0.475083
O -0.000048 -0.172267 2.882354
C -1.739129 -0.418071 -0.294248
O -2.853220 -0.631892 -0.475158
C 0.000121 -2.201732 -0.658104
C -0.000017 1.173104 -1.640475
H 0.879261 -2.719409 -0.271272
H 0.000301 -2.255795 -1.747101
H 0.917610 1.207099 -2.219565
H -0.917629 1.207030 -2.219592
C -0.000059 1.802115 -0.376122
O -0.000101 2.800538 0.270833
FREQS= 54 62 64 66 92 109 121 145 159 279 364 375 417 427 435 469 470 491 526 549 552
575 601 759 794 835 867 961 1019 1252 1433 1481 1489 1966 2111 2123 2167 3068 3147 3154 3173 3236
MIN071_5
16
E= -1915.17195019 ZPE= 62.39 Gcorr 0.033193000
H 3.793491 0.455432 1.095961
Co -0.581435 0.010684 0.097784
C -2.302338 0.276973 0.502450
C -0.232979 1.695131 -0.359992
O -3.385333 0.417927 0.858922
O -0.048950 2.735824 -0.810628
C -0.787912 -1.535402 -0.724203
O -0.920282 -2.453602 -1.405742
C 1.460348 -1.232192 1.313487
C 3.583787 0.140001 0.066986
H 0.686361 -1.934133 1.601328
H 2.368690 -1.249058 1.910406
H 3.817510 -0.925278 -0.038774
H 4.190060 0.728406 -0.622508
C 1.263704 -0.366389 0.291803
O 2.221437 0.395780 -0.283774
FREQS= 31 59 76 86 93 97 140 183 230 268 318 342 375 394 462 479 486 506 545 578 593
673 695 798 921 1048 1167 1211 1227 1407 1482 1499 1517 1604 2092 2107 2167 3029 3098 3156 3159 3242
MIN072_5
16
E= -1915.17322478 ZPE= 61.10 Gcorr 0.034960000
H -1.310215 1.386286 1.463115
Co 0.268735 -0.000118 -0.098478
C 1.122556 -1.427694 -0.765246
C 1.123462 1.426275 -0.766557
O 1.575867 -2.359111 -1.251763
O 1.577457 2.356928 -1.253894
C 0.878991 0.000762 1.615318
O 1.214045 0.001398 2.708526
C -1.353129 1.178536 0.392414
C -1.353559 -1.177360 0.394181
H -0.322617 -0.000542 -1.451443
H -1.540669 2.082821 -0.185206
H -1.310555 -1.383303 1.465213
H -1.541489 -2.082531 -0.181914
C -2.190350 0.000445 0.017462
O -3.192136 0.000119 -0.674907
FREQS= 71 72 89 95 97 114 160 331 363 411 421 436 438 474 478 502 505 524 545 549 660
746 800 829 868 887 952 1078 1093 1109 1226 1458 1469 1801 2027 2147 2154 2189 3099 3103 3184 3185
MIN073_5
16
E= -1915.16961469 ZPE= 61.22 Gcorr 0.031482000
H 0.081150 -2.462942 1.001749
Co -0.259982 -0.066012 -0.081186
C -1.146372 0.247187 1.486441
C 0.067886 1.679999 -0.347383
O -2.316707 0.330726 1.707052
O 0.264242 2.779837 -0.609787
C -1.740557 -0.553348 -0.974245
O -2.617391 -0.875879 -1.640056
C 1.509241 -0.866466 0.588453
C 0.557289 -1.857452 0.236357
H 2.469088 -0.884011 -1.377787
H 1.709981 -0.632501 1.631709
H -0.373570 0.331085 2.289812
H 0.676028 -2.376430 -0.716613
C 2.602888 -0.508358 -0.332925
O 3.589180 0.135036 -0.029339
FREQS= 50 60 63 72 87 107 128 168 195 289 324 374 393 405 443 457 489 502 510 582 609
772 845 889 981 995 1019 1164 1221 1232 1358 1445 1520 1794 1846 2111 2155 2835 2839 3112 3170 3212

MIN074_5
16
E= -1915.17437612 ZPE= 63.33 Gcorr 0.036422000
H 1.779479 2.752947 1.358220
Co -0.407110 -0.099502 -0.145892
C 1.181812 -0.864680 0.514157
C -1.726725 1.090564 -0.263543
O 1.651308 -1.756969 1.166072
O -2.552833 1.883206 -0.148764
C -1.296854 -1.591390 -0.012456
O -1.877422 -2.583056 0.065416
C 0.959056 1.455235 -0.152569
C 0.891817 2.125520 1.210365
H 2.605736 -0.915174 -1.518571
H 0.989490 2.132134 -1.005490
H 0.012977 2.769726 1.296615
H 0.864894 1.403102 2.031127
C 1.627577 0.237876 -0.340819
O 2.143859 -0.065048 -1.576927
FREQS= 25 56 88 93 107 170 194 219 240 290 336 364 388 410 445 469 486 515 564 612 631
665 771 938 959 1077 1095 1202 1223 1379 1434 1498 1499 1549 1870 2089 2139 3038 3103 3131 3158 3783
MIN075_5
16
E= -1915.16478928 ZPE= 61.07 Gcorr 0.026931000
H 0.727827 2.470360 1.031606
Co -0.250165 -0.086605 -0.006497
C -0.306032 -0.983671 -1.513609
C 1.583140 -0.163811 0.047183
O -0.362653 -1.765037 -2.363428
O 2.378137 -1.049107 0.225943
C -0.400724 -0.733349 1.618197
O -0.518063 -1.358602 2.582642
C 1.024666 2.402695 -0.018967
C 2.056911 1.292563 -0.246393
H 0.125600 2.253350 -0.633752
H 1.429746 3.376845 -0.307749
H 2.945436 1.447845 0.373514
H 2.390195 1.288125 -1.290837
C -3.198635 1.290038 -0.162251
O -2.174959 0.782123 -0.112948
FREQS= 38 44 55 66 69 88 100 126 151 167 187 238 306 319 364 389 457 487 524 538 572
591 772 859 1020 1054 1102 1275 1312 1422 1465 1507 1515 1821 2058 2118 2138 3017 3056 3091 3112 3126
MIN076_5
16
E= -1915.16704032 ZPE= 61.48 Gcorr 0.030101000
H 1.837799 -1.685518 -1.171570
Co -0.362716 -0.168302 0.011216
C 1.092638 0.979068 -0.065974
C -1.063139 0.642142 -1.373092
O 1.161941 2.177735 -0.084517
O -1.633366 1.263376 -2.158561
C -0.742601 0.388557 1.656293
O -1.166681 0.873425 2.610271
C 2.295106 -1.398163 -0.219753
C 2.394096 0.120952 -0.067805
H 1.734412 -1.860708 0.598857
H 3.296588 -1.837402 -0.201047
H 2.998476 0.552530 -0.872724
H 2.892559 0.380968 0.873288
C -0.975624 -2.090185 -0.813201
O -0.983063 -2.172029 0.354252
FREQS= 37 47 59 88 99 102 126 130 160 227 277 312 317 356 375 413 455 471 517 533 562
573 765 860 1036 1060 1102 1283 1319 1430 1458 1519 1521 1822 1923 2084 2137 3048 3056 3101 3119 3126
MIN001_6
16
E= -1803.08382817 ZPE= 67.82 Gcorr 0.041034000
H -2.119199 -2.063390 0.801345
Co 0.318275 0.020128 -0.204249
C 1.332350 -1.331487 -0.679587
C 1.021758 0.336860 1.395633
O 2.053016 -2.126284 -1.097299
O 1.557004 0.576245 2.384102
C 0.033114 1.677797 -0.708990
O -0.047776 2.739385 -1.147239
C -3.537088 -0.562443 0.073155
C -2.166721 -0.989675 0.599257
H -3.787378 -1.083115 -0.855977

H -4.319870 -0.788751 0.804147
H -3.569836 0.512881 -0.126700
H -1.402871 -0.790939 -0.186441
H -0.080016 -0.167270 -1.637297
H -1.912663 -0.463954 1.522319
FREQS= 27 38 48 78 88 96 101 153 181 321 336 344 377 460 487 492 510 571 578 659 751 825 873 1013 1210 1247 1383 1432 1502 1513 1517 1522 1963 2093 2112 2172 2842 3051 3088 3117 3122 3142
MIN002_6
16
E= -1803.08324789 ZPE= 67.79 Gcorr 0.040780000
H -1.339255 0.802231 0.110600
Co 0.360113 -0.026837 -0.210938
C 0.578514 -0.217479 1.540034
C 1.422475 1.344322 -0.475756
O 0.826447 -0.381651 2.650440
O 2.206492 2.147382 -0.733088
C 0.302109 -1.725738 -0.654074
O 0.403594 -2.813793 -1.017189
C -3.479303 0.501220 0.274457
C -2.243902 0.903182 -0.531142
H -3.434750 -0.549761 0.576053
H -3.581015 1.106185 1.180527
H -4.388301 0.636964 -0.320112
H -2.135972 0.297562 -1.432572
H 0.457640 0.029980 -1.705246
H -2.273027 1.952890 -0.836346
FREQS= 24 31 62 67 89 95 102 158 182 326 341 343 370 454 486 488 511 569 578 639 755 822 868 1012 1208 1242 1383 1431 1504 1514 1517 1522 1968 2093 2111 2171 2851 3051 3092 3118 3123 3149
MIN003_6
16
E= -1803.08379552 ZPE= 67.88 Gcorr 0.041604000
H -4.064561 -1.334480 0.525314
Co 0.341880 0.058790 -0.222766
C 0.566178 0.245880 1.529570
C -0.031428 1.726268 -0.621333
O 0.794322 0.417859 2.642793
O -0.145354 2.822098 -0.956094
C 1.628776 -1.093208 -0.544489
O 2.538630 -1.735284 -0.836599
C -3.329417 -0.680892 0.045118
C -2.010757 -1.420308 -0.175409
H -3.195088 0.194934 0.687138
H -3.757182 -0.338085 -0.901854
H -2.134571 -2.302075 -0.811589
H -1.292794 -0.775876 -0.732009
H 0.346104 0.022240 -1.720766
H -1.573552 -1.757814 0.766906
FREQS= 27 37 57 77 94 100 109 141 199 322 337 343 423 464 486 495 510 570 578 656 751 816 851 1010 1201 1236 1385 1433 1504 1514 1516 1540 1966 2093 2112 2172 2844 3051 3084 3115 3122 3139
MIN004_6
16
E= -1803.08328332 ZPE= 67.83 Gcorr 0.041390000
H -4.212782 -1.286946 0.039734
Co 0.294364 0.016026 -0.183121
C 1.396221 -1.272803 -0.643128
C -0.115331 1.623878 -0.752456
O 2.167304 -2.019265 -1.060427
O -0.280413 2.654166 -1.239605
C 1.030676 0.448724 1.373061
O 1.596333 0.770560 2.320514
C -3.425838 -0.554480 0.245569
C -2.086255 -1.253161 0.473399
H -3.730886 0.023447 1.123300
H -3.378184 0.129510 -0.606989
H -1.309131 -0.501060 0.739803
H -2.118730 -1.942088 1.322856
H -1.776659 -1.822533 -0.404744
H -0.084099 -0.229681 -1.612226
FREQS= 29 39 54 79 87 96 111 136 195 327 333 343 414 455 487 489 511 568 578 642 756 819 854 1011 1204 1238 1382 1433 1504 1514 1516 1534 1967 2092 2111 2171 2847 3052 3086 3118 3124 3146
MIN005_6
16
E= -1803.07681178 ZPE= 67.96 Gcorr 0.041509000
H 1.038309 0.568670 0.810673
Co -0.371298 0.010316 -0.139429
C -1.131358 1.556335 0.331193
C 0.521389 -0.123413 -1.672913

O -1.752390 2.506516 0.527089
O 0.967245 -0.212645 -2.731647
C -1.192688 -1.446580 0.496778
O -1.850114 -2.346059 0.788289
C 3.132853 0.167941 0.986109
C 1.688094 -0.155689 1.369929
H 3.315460 -0.030739 -0.073785
H 3.374425 1.217026 1.179564
H 1.456097 -1.200850 1.170004
H 1.478715 0.039903 2.424111
H 3.825295 -0.449287 1.566446
H -1.490913 0.002685 -1.048856
FREQS= 19 40 57 67 89 89 144 199 218 308 330 337 427 439 446 462 514 517 523 693 708
818 903 1015 1192 1262 1356 1430 1502 1512 1516 1609 2086 2088 2116 2166 2756 3056 3106 3127 3131 3167
MIN006_6
16
E= -1803.07703738 ZPE= 68.01 Gcorr 0.042693000
H 3.102608 -0.059725 -0.230315
Co -0.373684 0.100712 -0.055820
C -0.027353 1.772453 0.466559
C -0.041352 -0.220783 -1.784908
O 0.057605 2.896391 0.704289
O 0.027101 -0.324374 -2.929880
C -1.641049 -0.853735 0.750926
O -2.558411 -1.388986 1.197119
C 3.022908 -0.677677 0.668194
C 1.609101 -1.227190 0.842974
H 3.739627 -1.499260 0.574729
H 1.513296 -1.863053 1.728080
H 1.294380 -1.824912 -0.011531
H 0.908668 -0.384387 1.089990
H 3.323209 -0.067053 1.524707
H -1.536222 0.756509 -0.603207
FREQS= 26 47 62 66 89 112 146 188 230 308 335 342 431 444 456 497 515 519 523 690 706
801 877 1014 1174 1245 1365 1432 1501 1511 1516 1627 2087 2088 2115 2166 2751 3056 3093 3125 3132 3161
MIN007_6
16
E= -1803.06143420 ZPE= 67.12 Gcorr 0.045305000
H -2.933568 -0.959753 -0.351490
Co 0.168827 0.019600 -0.266085
C -0.498471 -1.283922 0.735578
C 0.723940 1.555157 0.434092
O -0.941732 -2.061214 1.459944
O 1.028735 2.537034 0.950758
C 1.800585 -0.702560 -0.465568
O 2.844213 -1.166915 -0.572076
C -2.799979 0.029261 -0.800886
C -1.706015 0.843998 -0.113326
H -3.763630 0.551450 -0.727856
H -2.597804 -0.121576 -1.866919
H -1.649007 1.834734 -0.575137
H -1.960528 0.999034 0.938605
H -0.258954 -0.362334 -1.816238
H 0.035079 0.406410 -1.865024
FREQS= 50 74 88 97 102 117 249 258 284 365 370 442 452 472 488 506 522 559 564 602 645
724 941 984 995 1049 1248 1281 1428 1485 1508 1510 1536 2097 2111 2164 3024 3058 3085 3102 3123 3192
MIN008_6
16
E= -1803.06169409 ZPE= 67.13 Gcorr 0.045771000
H -1.891430 0.878532 1.482296
Co 0.271994 0.000001 0.391473
C -0.060584 1.582199 -0.341745
C -0.060615 -1.582196 -0.341737
O -0.330707 2.579211 -0.849830
O -0.330749 -2.579206 -0.849821
C 2.032628 -0.000018 0.049394
O 3.151639 -0.000023 -0.204136
C -2.740862 -0.000006 -0.296857
C -1.734881 0.000033 0.848251
H -2.640865 -0.882116 -0.937307
H -3.766421 0.000002 0.095789
H -2.640875 0.882064 -0.937364
H -1.891432 -0.878421 1.482359
H 0.475812 0.410729 1.985461
H 0.475801 -0.410745 1.985456
FREQS= 51 82 94 98 108 128 255 256 278 367 374 433 456 468 500 517 522 557 563 598 633
729 929 970 993 1060 1241 1272 1428 1484 1508 1511 1513 2095 2110 2164 3028 3050 3092 3094 3122 3230

MIN009_6
16
E= -1803.05597412 ZPE=   66.97 Gcorr   0.044706000
H 2.511201 -0.924416 0.905252
Co -0.298229 0.005411 -0.465673
C 0.466066 1.614716 -0.652323
C 0.410360 -1.629150 -0.653046
O 0.949905 2.658613 -0.687155
O 0.857971 -2.689083 -0.687909
C -1.878142 0.031412 0.392398
O -2.843956 0.046896 1.016705
C 2.107128 -0.035645 1.399105
C 0.582599 -0.010130 1.390660
H 2.540754 0.839937 0.906764
H 2.474919 -0.042588 2.434339
H 0.188183 -0.883850 1.917319
H 0.217628 0.875856 1.918079
H -0.472547 0.008381 -2.088584
H -1.247381 0.021964 -1.813899
FREQS=   52   59   98 101 103 131 258 265 273 315 315 364 423 477 492 495 508 522 546 574 601
731 982 984 1016 1053 1245 1274 1429 1485 1509 1514 1669 2088 2096 2154 3026 3060 3096 3106 3131 3224
MIN010_6
16
E= -1803.05493533 ZPE=   67.25 Gcorr   0.044498000
H -3.428809 -0.997871 1.352166
Co 0.598594 -0.104363 -0.413668
C -1.174869 -0.142855 -0.123933
C 2.063173 -0.882716 0.280253
O -0.992743 -1.364159 -0.158629
O 2.918509 -1.392341 0.858004
C 0.964756 1.522628 0.019527
O 1.178299 2.609460 0.352904
C -3.640023 -0.375256 0.479037
C -2.463656 0.554611 0.172409
H -4.542036 0.208162 0.682800
H -3.844242 -1.041028 -0.363541
H -2.257059 1.242435 1.002434
H -2.670139 1.206669 -0.686524
H 0.381864 -0.029128 -2.139381
H 0.869583 -0.653605 -2.060917
FREQS=   39   45   69   89 117 190 199 279 286 326 348 386 435 448 462 503 517 568 590 696 766
789 961 1035 1081 1120 1227 1276 1371 1428 1453 1509 1514 1680 2077 2130 3048 3063 3076 3138 3142 3568
MIN011_6
16
E= -1803.05699156 ZPE=   69.96 Gcorr   0.047905000
H -1.301772 -0.786672 1.166355
Co 0.202551 -0.025998 0.038236
C 1.292000 -1.234277 -0.621798
C -0.176074 1.469799 -0.799939
O 2.029568 -1.886018 -1.229802
O -0.280298 2.375535 -1.512358
C 1.454565 0.723736 1.101002
O 1.039337 0.577793 2.234329
C -3.233361 -0.516558 0.198855
C -1.832960 -1.123869 0.258226
H -3.190057 0.574754 0.151085
H -3.780144 -0.874243 -0.678346
H -3.808165 -0.792452 1.087776
H -1.852883 -2.214938 0.299751
H 2.414644 1.226052 0.872066
H -1.284369 -0.882032 -0.686481
FREQS=   53   60   73   95   99 118 157 184 201 247 274 324 400 469 505 519 537 542 687 825 896
907 1015 1196 1252 1290 1378 1433 1488 1512 1516 1621 1734 2052 2112 2793 2948 2974 3058 3119 3130 3141
MIN012_6
16
E= -1803.05275169 ZPE=   66.73 Gcorr   0.044879000
H -2.772010 0.882177 -0.481566
Co 0.292913 0.000000 0.322208
C -0.145242 1.468557 -0.620817
C -0.145243 -1.468557 -0.620817
O -0.423586 2.461041 -1.120830
O -0.423588 -2.461041 -1.120831
C 2.079508 -0.000001 0.158470
O 3.223984 -0.000001 0.191123
C -2.765722 0.000000 0.167763
C -1.596825 0.000000 1.149944
H -2.772010 -0.882177 -0.481565

H -3.717961 0.000001 0.715400
H -1.655277 0.876310 1.797417
H -1.655278 -0.876308 1.797418
H 0.545268 0.871815 1.475156
H 0.545268 -0.871815 1.475155
FREQS= 59 62 89 96 107 115 255 257 270 389 392 424 450 489 501 513 535 547 603 639 713
749 860 993 997 1048 1239 1279 1431 1490 1510 1511 2062 2081 2145 2150 2186 3024 3079 3097 3102 3146
MIN013_6
16
E= -1803.05252219 ZPE= 66.74 Gcorr 0.044682000
H 2.463632 -0.604815 -2.013178
Co -0.196913 -0.010110 -0.224187
C -0.678211 1.567996 0.493628
C -1.795664 -0.780313 -0.480413
O -0.956189 2.615302 0.864789
O -2.778670 -1.291250 -0.769790
C 0.494830 -1.013963 1.099094
O 1.003358 -1.684463 1.876484
C 2.735139 -0.198243 -1.034483
C 1.684449 0.778869 -0.516512
H 3.703684 0.309482 -1.143117
H 2.889177 -1.045738 -0.358553
H 2.016516 1.207232 0.435107
H 1.574477 1.608083 -1.217438
H 0.262700 -0.984103 -1.218499
H -0.384782 0.540054 -1.571029
FREQS= 58 66 82 94 98 117 251 265 285 385 387 422 447 482 494 512 535 542 579 639 718
771 870 987 999 1061 1252 1283 1427 1492 1509 1513 2073 2089 2145 2155 2187 3021 3056 3089 3111 3133
MIN014_6
16
E= -1803.05013157 ZPE= 66.21 Gcorr 0.042435000
H -0.477843 -3.123668 1.856323
Co 0.034953 0.197307 -0.354820
C 1.713698 -0.290332 -0.590252
C -1.710519 0.156926 -0.609039
O 2.778834 -0.654767 -0.803964
O -2.830867 0.076608 -0.835575
C 0.226741 1.704037 0.607628
O 0.352277 2.690605 1.173981
C -0.395324 -2.517766 0.943091
C -0.139917 -1.053700 1.285871
H -1.324783 -2.655182 0.380314
H 0.412547 -2.948798 0.342344
H -0.949902 -0.666041 1.911051
H 0.777210 -0.960089 1.875101
H -0.105808 -0.930011 -1.297409
H 0.154806 1.061950 -1.586926
FREQS= 17 56 89 89 102 116 251 272 352 376 419 437 449 481 499 503 552 562 628 651 699
710 765 972 984 1054 1233 1273 1428 1487 1510 1512 1885 2049 2138 2155 2193 3018 3057 3083 3093 3117
MIN015_6
16
E= -1803.05011610 ZPE= 66.11 Gcorr 0.042833000
H -0.878880 -2.840534 0.359092
Co -0.000181 0.201142 -0.354890
C 1.726702 -0.065791 -0.598068
C -1.726744 -0.067938 -0.597960
O 2.828736 -0.290841 -0.816791
O -2.828516 -0.294356 -0.816595
C -0.001074 1.720724 0.606437
O -0.001638 2.716543 1.170525
C 0.001955 -2.549185 0.941064
C 0.000649 -1.063138 1.284396
H 0.883555 -2.839110 0.359539
H 0.002215 -3.160262 1.854593
H -0.875750 -0.818166 1.891953
H 0.876438 -0.816676 1.892236
H 0.000500 -0.930911 -1.302345
H -0.000778 1.076030 -1.585363
FREQS= 30 53 88 89 102 115 249 259 351 365 421 426 448 480 499 503 552 562 626 652 690
708 762 967 982 1052 1228 1270 1426 1487 1509 1509 1888 2048 2138 2155 2192 3018 3056 3082 3092 3117
MIN016_6
16
E= -1803.04975523 ZPE= 66.28 Gcorr 0.043957000
H 1.680174 1.753360 2.639045
Co -0.210575 -0.402964 -0.198023
C 1.404383 -1.073234 -0.442402
C -1.923023 -0.439887 0.220893

O 2.417295 -1.589133 -0.586064
O -3.027534 -0.541736 0.507747
C -0.118820 1.145439 -1.109453
O -0.081709 2.105953 -1.730534
C 1.473971 1.382586 1.625384
C 0.304775 0.400076 1.637062
H 2.400544 0.926865 1.258248
H 1.274904 2.260797 1.000903
H 0.526707 -0.440060 2.299663
H -0.580033 0.894977 2.048275
H -0.328814 -1.714591 0.468678
H -0.600083 -1.091884 -1.486300
FREQS= 43 67 86 92 104 112 246 272 352 382 421 435 454 478 499 504 551 562 623 658 697
709 763 981 990 1048 1238 1277 1428 1489 1510 1512 1876 2048 2139 2154 2193 3018 3061 3080 3093 3122
MIN017_6
16
E= -1803.05579428 ZPE= 70.40 Gcorr 0.050931000
H 2.528388 -1.100076 2.297438
Co -0.390942 0.161327 -0.077107
C 0.840107 1.431163 -0.565958
C 0.004735 -1.280897 -0.980442
O 1.593098 2.182483 -0.992096
O 0.191114 -2.213739 -1.630493
C -2.220554 -0.150245 0.503137
O -1.835605 1.123222 0.452588
C 2.117789 -0.782453 1.328763
C 0.620162 -0.577595 1.441111
H 2.376691 -1.557772 0.600340
H 2.642968 0.134914 1.043524
H 0.119046 -1.500592 1.745530
H 0.376052 0.212174 2.163077
H -2.926733 -0.501621 -0.256658
H -2.323267 -0.618420 1.488976
FREQS= 64 69 80 90 100 129 161 243 262 385 400 425 473 492 502 514 559 611 717 803 905
971 1053 1077 1202 1214 1259 1265 1423 1480 1500 1508 1557 2095 2150 3023 3024 3037 3087 3102 3110 3125
MIN018_6
16
E= -1803.05186064 ZPE= 69.74 Gcorr 0.047742000
H -2.253079 1.697664 -0.568308
Co 0.054871 0.010710 -0.049413
C 0.350217 -1.524882 -0.850823
C 0.812359 1.529936 -0.494264
O 0.700869 -2.420448 -1.493656
O 1.421556 2.404261 -0.944503
C 1.294387 -0.331160 1.244872
O 0.820986 -0.367026 2.361098
C -2.281540 0.608067 -0.608872
C -2.393785 -0.026331 0.782005
H -3.131193 0.314381 -1.232514
H -3.210119 0.442665 1.338797
H -1.495692 0.118376 1.407182
H -2.597027 -1.097267 0.723718
H -1.409689 0.263854 -1.219721
H 2.378180 -0.496921 1.063992
FREQS= 59 63 82 91 99 122 132 177 215 245 301 321 373 422 473 514 536 541 670 830 865
905 993 1197 1219 1319 1424 1450 1475 1490 1527 1582 1754 2051 2113 2800 2900 2975 3085 3096 3139 3159
MIN019_6
16
E= -1803.05141802 ZPE= 67.82 Gcorr 0.047577000
H -3.472513 -0.249452 0.669633
Co 0.424486 0.055968 -0.536951
C 1.678773 -1.079490 -0.054036
C 0.638733 1.694529 0.070827
O 2.566070 -1.692368 0.355226
O 0.921719 2.703660 0.552319
C -0.762595 -0.443045 0.696555
O -1.020217 -0.557599 1.855430
C -3.057561 -0.024524 -0.316074
C -1.732773 -0.770731 -0.497953
H -2.914407 1.057237 -0.387542
H -3.780227 -0.326700 -1.079583
H -1.874042 -1.854785 -0.473562
H -1.305369 -0.545958 -1.493198
H 0.922622 -0.122869 -2.135570
H 0.634783 0.641417 -2.102227
FREQS= 50 73 82 100 135 204 250 279 296 358 377 402 476 492 522 531 534 576 627 673 701
822 933 985 1062 1094 1278 1316 1421 1456 1508 1515 1539 1892 2083 2133 2960 3057 3098 3133 3144 3273

MIN020_6
16
E= -1803.04629454 ZPE=   66.14 Gcorr   0.042610000
H 3.778583 -0.000477 -0.036783
Co -0.238003 0.000019 0.078080
C 0.033364 1.739529 -0.050614
C 0.032678 -1.739589 -0.050658
O 0.259376 2.856962 -0.165469
O 0.258237 -2.857113 -0.165513
C -2.026639 0.000371 -0.070125
O -3.170936 0.000591 -0.080451
C 2.755932 -0.000295 -0.438368
C 1.744230 -0.000381 0.698443
H 2.656410 -0.881353 -1.079902
H 2.656600 0.881007 -1.079596
H 1.877330 -0.871014 1.343878
H 1.877535 0.869975 1.344209
H 0.082701 -0.000023 -1.401813
H -0.513892 0.000031 1.561254
FREQS=   25   35   95   98 106 154 247 250 283 375 403 444 461 506 524 542 550 561 609 633 686
739 753 981 983 1061 1239 1262 1423 1494 1503 1506 1819 1987 2137 2150 2193 3024 3081 3096 3109 3135
MIN021_6
16
E= -1803.05529356 ZPE=   71.79 Gcorr   0.053450000
H -3.485574 0.912141 -1.113139
Co 0.329233 0.112093 0.047418
C -1.408193 -0.686243 0.183524
C 1.729910 1.152178 0.247003
O -1.634029 -1.188355 1.480881
O 2.675332 1.779144 0.461530
C 1.105816 -1.307095 -0.498238
O 1.619666 -2.234770 -0.964460
C -2.440857 1.232661 -1.157041
C -1.705138 0.763488 0.099646
H -2.420749 2.321828 -1.254370
H -1.992680 0.806829 -2.059277
H -0.709481 1.407629 0.133864
H -2.176285 1.116351 1.021982
H -1.244225 -2.071348 1.531181
H -1.837274 -1.298043 -0.613483
FREQS=   34   47   97   98 140 176 209 307 333 360 411 429 457 467 530 580 598 627 777 895 1006
1050 1080 1144 1155 1252 1288 1350 1427 1433 1509 1514 1754 2062 2123 2177 3056 3092 3095 3130 3134 3818
MIN022_6
16
E= -1803.04320395 ZPE=   67.00 Gcorr   0.042576000
H 0.000747 -2.487457 0.812725
Co -0.000006 -0.053835 -0.157833
C 1.753977 -0.281131 -0.238381
C -1.753967 -0.281282 -0.238361
O 2.880365 -0.478217 -0.307618
O -2.880340 -0.478455 -0.307613
C -0.000087 1.741407 -0.188013
O -0.000135 2.885755 -0.192028
C 0.000147 -2.115899 -0.212014
C 0.000015 -0.079463 1.906241
H 0.875023 -2.496505 -0.740787
H -0.883834 -0.611809 2.263476
H -0.000267 0.919703 2.347874
H 0.884171 -0.611299 2.263477
H -0.875276 -2.496671 -0.739760
H -0.000040 -0.096861 -1.664284
FREQS=   18   51   94 100 114 116 130 182 220 404 419 427 442 457 490 517 535 554 567 585 660
699 830 850 873 889 1268 1304 1480 1482 1492 1495 1891 2136 2148 2189 3056 3071 3145 3150 3155 3179
MIN023_6
16
E= -1803.04628412 ZPE=   67.58 Gcorr   0.046209000
H -2.395999 1.003588 -0.761494
Co 0.001207 -0.047372 -0.213222
C -0.004189 1.187776 1.113088
C 1.382571 -1.102357 0.224679
O 0.003987 2.040953 1.876138
O 2.292914 -1.778240 0.386266
C -1.364311 -1.127936 0.217880
O -2.266844 -1.813882 0.380680
C 1.356175 1.082650 -1.253415
C -1.397111 1.079524 -1.199333
H -1.088524 2.126100 -1.174756

H 1.463497 2.067523 -0.793340
H 2.345806 0.623091 -1.301113
H -1.446753 0.754075 -2.237740
H 0.027708 -0.811990 -1.468322
H 0.982414 1.208059 -2.268302
FREQS= 72 83 101 107 118 135 145 150 274 373 415 436 441 457 482 511 522 529 537 551 764
781 848 867 875 888 1274 1305 1475 1481 1492 1498 2056 2139 2148 2184 3059 3062 3142 3144 3175 3178
MIN024_6
16
E= -1803.05184148 ZPE= 71.65 Gcorr 0.053137000
H 2.157311 1.058549 -1.874290
Co -0.323138 0.149051 -0.049806
C 1.365001 -0.740002 0.077027
C -1.175377 -1.274054 -0.463016
O 1.614377 -1.582242 1.174302
O -1.743685 -2.233879 -0.764524
C -1.682294 1.233268 0.212211
O -2.600955 1.866017 0.510194
C 2.557678 1.320393 -0.890324
C 1.708799 0.710939 0.229591
H 3.577430 0.930553 -0.828173
H 2.600493 2.411542 -0.825412
H 2.121838 0.908216 1.222749
H 0.746292 1.392142 0.272229
H 0.934120 -1.428781 1.842643
H 1.786506 -1.199032 -0.817693
FREQS= 40 53 96 98 122 185 205 232 320 342 405 428 458 465 529 576 585 624 764 885 999
1042 1098 1109 1158 1255 1272 1367 1425 1435 1509 1512 1737 2071 2129 2235 3054 3095 3126 3132 3134 3811
MIN025_6
16
E= -1803.04483608 ZPE= 67.38 Gcorr 0.046484000
H -3.043333 -2.079705 -1.114751
Co 0.375660 -0.042797 0.293915
C -1.283756 0.648472 0.207354
C 1.704911 -1.269294 0.085395
O -2.090310 1.422865 0.624493
O 2.547838 -2.035971 -0.061847
C 1.169787 1.392956 -0.334280
O 1.724412 2.349680 -0.659472
C -2.772666 -1.335099 -0.360697
C -1.560290 -0.517794 -0.810826
H -2.570570 -1.857908 0.578293
H -3.624251 -0.668321 -0.201283
H -0.687923 -1.186870 -0.970716
H -1.725845 -0.032335 -1.776552
H 0.029750 -0.582712 1.759403
H 0.475913 0.155333 1.842834
FREQS= 48 66 85 91 143 199 245 270 287 342 369 402 445 490 507 535 552 567 638 676 702
820 986 1040 1089 1094 1275 1325 1422 1454 1508 1514 1729 1891 2097 2144 2749 2907 3056 3098 3132 3141
MIN026_6
16
E= -1803.04181334 ZPE= 66.74 Gcorr 0.043633000
H -3.759776 -1.073550 -0.883747
Co 0.577752 -0.107946 0.000006
C 2.216524 -0.883982 -0.000008
C -1.222607 -0.212834 0.000003
O 3.246066 -1.383284 -0.000023
O -1.008619 -1.417688 0.000034
C 0.912769 1.583285 0.000002
O 1.106687 2.715981 0.000004
C -3.755141 -0.430635 -0.000011
C -2.534571 0.493352 -0.000020
H -3.759789 -1.073516 0.883750
H -4.674336 0.161075 -0.000029
H -2.522086 1.160518 -0.871458
H -2.522099 1.160551 0.871393
H 0.591945 -0.017841 1.523613
H 0.591923 -0.017886 -1.523603
FREQS= 26 52 62 85 103 181 204 268 293 359 395 441 479 490 529 556 611 634 685 700 782
812 961 1035 1089 1118 1278 1374 1429 1452 1508 1514 1733 1735 1925 2130 2174 3052 3065 3082 3141 3145
MIN027_6
16
E= -1803.04451613 ZPE= 67.37 Gcorr 0.046382000
H -1.892783 -0.315948 -2.095003
Co 0.430671 -0.017492 0.410153
C -1.345875 0.154174 0.637571
C 2.008959 -0.826577 -0.001242

O -2.269362 0.759417 1.089801
O 3.003591 -1.331686 -0.276219
C 0.602583 1.481616 -0.490729
O 0.761311 2.502323 -1.002183
C -2.329300 -1.025032 -1.386172
C -1.431035 -1.201640 -0.158433
H -0.444335 -1.602212 -0.472614
H -2.475757 -1.978672 -1.901398
H -3.303537 -0.639201 -1.074207
H -1.835149 -1.933126 0.545487
H 0.765608 0.422272 1.873683
H 0.561518 -0.416513 1.952748
FREQS= 44 71 78 93 139 206 238 259 298 353 368 405 441 485 506 523 551 576 612 677 708
824 985 1037 1086 1102 1278 1323 1422 1458 1509 1515 1732 1891 2097 2144 2745 2914 3056 3104 3133 3141
MIN028_6
16
E= -1803.04562846 ZPE= 68.20 Gcorr 0.049926000
H -1.611210 -1.976014 -0.973173
Co 0.218041 -0.011431 -0.303796
C 1.561909 -1.117581 0.060437
C -1.729000 -0.281052 0.333130
O 2.412449 -1.860307 0.297317
O -2.777191 0.231643 -0.377878
C 1.032716 1.533890 -0.132320
O 1.556576 2.546232 0.042704
C -1.023016 0.441948 1.317403
C -1.168266 -1.513007 -0.096756
H -2.925873 1.147956 -0.104690
H -0.585686 -0.089275 2.155582
H -1.344765 1.455158 1.547470
H -0.832745 -2.212210 0.661462
H -0.372911 0.424166 -1.754908
H 0.205338 -0.166860 -1.917779
FREQS= 40 84 89 106 217 259 350 376 387 424 435 451 462 487 503 531 553 572 648 671 774
845 897 950 991 1000 1015 1050 1212 1408 1451 1542 1557 1609 2081 2123 2981 3143 3159 3230 3242 3802
MIN029_6
16
E= -1803.03992661 ZPE= 66.70 Gcorr 0.045932000
H -3.562004 -0.825966 0.240984
Co 0.388782 -0.037924 -0.238354
C 1.713420 -1.272159 -0.054267
C -1.305922 0.545307 -0.430366
O 2.548747 -2.055137 -0.117305
O -2.126346 1.149236 -1.046233
C 1.105217 1.447780 0.440891
O 1.620678 2.439853 0.701923
C -2.667897 -1.340472 0.603110
C -1.563296 -0.316404 0.864856
H -2.359639 -2.063299 -0.156817
H -2.920427 -1.887146 1.516089
H -1.831165 0.400086 1.645606
H -0.655866 -0.834269 1.252614
H -0.045610 -0.857044 -1.388862
H 0.843839 0.435640 -1.526469
FREQS= 64 68 86 92 145 204 250 291 317 364 374 417 421 459 499 520 570 593 627 664 683
780 824 991 1064 1092 1274 1336 1423 1467 1509 1515 1908 2041 2122 2130 2166 2872 3058 3105 3135 3143
MIN030_6
16
E= -1803.03974292 ZPE= 66.72 Gcorr 0.046025000
H 2.204574 0.568653 -1.773413
Co -0.459947 -0.107898 0.296592
C -2.098403 -0.717751 -0.209575
C 1.273138 -0.236512 0.774889
O -3.162592 -1.120891 -0.349993
O 2.157212 0.015015 1.531484
C -0.447112 1.584734 -0.267251
O -0.508233 2.715654 -0.455456
C 2.481081 -0.440407 -1.454676
C 1.399936 -1.056334 -0.565041
H 3.418246 -0.374559 -0.895698
H 2.645115 -1.047858 -2.349125
H 0.447571 -1.133109 -1.139440
H 1.633838 -2.080008 -0.265271
H -0.554569 -1.115602 1.372495
H -0.919148 0.415128 1.564124
FREQS= 62 75 81 90 149 203 251 292 310 359 386 409 440 464 500 518 556 599 633 657 677
779 828 991 1065 1093 1278 1341 1423 1465 1510 1514 1908 2040 2121 2129 2165 2867 3057 3115 3134 3142

MIN031_6
16
E= -1803.03905281 ZPE=   69.76 Gcorr   0.048060000
H 1.410297 -2.578968 1.256733
Co -0.111829 0.002595 0.082279
C -1.508525 -1.102232 -0.102840
C 1.344588 1.252761 0.286627
O -2.468096 -1.726976 -0.215718
O 2.076389 1.589404 -0.618145
C -1.064420 1.419729 0.063122
O -1.726403 2.369186 0.047916
C 1.407134 -1.641893 -0.703197
C 1.448213 -1.570290 0.839529
H 0.873528 -2.525764 -1.052745
H 0.969284 -0.760789 -1.237564
H 2.427143 -1.669347 -1.092373
H 2.355978 -1.081831 1.195337
H 0.590948 -1.058762 1.367173
H 1.575159 1.604030 1.330023
FREQS=   23   70   89   95 112 132 178 198 254 286 333 353 392 439 465 477 572 580 597 885 898
949 979 1159 1229 1366 1433 1454 1463 1485 1573 1606 1773 2072 2132 2659 2737 2778 3096 3104 3152 3168
MIN032_6
16
E= -1803.03281100 ZPE=   67.28 Gcorr   0.045334000
H 0.167389 -1.546326 1.143130
Co -0.343959 -0.060035 -0.316088
C 1.540288 0.216154 -0.433234
C -2.068315 -0.605799 -0.184006
O 2.223074 0.869766 -1.179909
O -3.170799 -0.919765 -0.113252
C -0.464403 1.439945 0.568863
O -0.595795 2.478310 1.054605
C 1.195355 -1.897480 0.929156
C 2.132442 -0.713342 0.643728
H 1.159338 -2.595256 0.089146
H 1.499258 -2.460246 1.816257
H 2.253101 -0.100365 1.546020
H 3.130261 -1.036382 0.329055
H -0.180587 -0.759484 -1.757343
H -0.405912 0.055645 -1.870474
FREQS=   47   61   80   96 100 135 233 246 283 313 348 407 449 488 499 533 553 570 607 686 779
885 1012 1030 1079 1114 1259 1309 1423 1467 1488 1524 1737 1821 2089 2142 2862 2932 3046 3088 3106 3136
MIN033_6
16
E= -1803.03216581 ZPE=   67.16 Gcorr   0.045043000
H -0.199463 -1.846752 0.687017
Co 0.337880 -0.075816 -0.332229
C 2.070797 -0.602049 -0.230322
C -1.547125 0.204464 -0.459911
O 3.180725 -0.893071 -0.181205
O -2.216190 1.000145 -1.067912
C 0.464289 1.400736 0.598199
O 0.591895 2.420413 1.121188
C -1.141865 -1.650900 1.237514
C -2.170571 -0.970226 0.323658
H -0.910243 -1.045333 2.116079
H -1.479660 -2.630706 1.586488
H -2.538884 -1.672515 -0.434557
H -3.044698 -0.608655 0.876167
H 0.376097 0.085624 -1.873745
H 0.169490 -0.746674 -1.778651
FREQS=   47   62   72   86   99 144 227 261 295 319 344 400 451 476 515 537 565 573 589 696 771
886 1020 1024 1076 1108 1259 1308 1423 1462 1487 1521 1762 1820 2092 2143 2768 2910 3048 3090 3102 3141
MIN034_6
16
E= -1803.03125929 ZPE=   67.12 Gcorr   0.045844000
H 1.869224 -2.146724 0.715727
Co -0.127869 -0.117414 -0.114560
C 1.647061 -0.243049 -0.280702
C -1.896745 -0.622029 -0.277418
O 2.709235 0.273355 -0.398483
O -3.003143 -0.846040 -0.468120
C -0.224407 1.609958 -0.123492
O -0.282708 2.755751 -0.142722
C -0.111723 -0.149816 1.914373
C 1.353565 -1.793501 -0.177428
H -0.196239 -1.196465 2.231237

H 0.816366 0.258736 2.322636
H -0.944235 0.395956 2.365162
H 1.735346 -2.248564 -1.089968
H 0.300290 -2.106333 -0.078510
H -0.121871 -0.060349 -1.630551
FREQS= 45 82 92 99 124 130 201 303 351 362 381 397 435 454 496 506 527 547 580 646 659
688 805 824 955 1054 1268 1361 1436 1469 1489 1493 1871 1926 2130 2166 2996 3032 3115 3121 3138 3196
MIN035_6
16
E= -1803.02878505 ZPE= 67.06 Gcorr 0.047063000
H 2.569343 -0.054627 1.219485
Co -0.341362 -0.062218 -0.184313
C 1.512416 0.025278 -0.620511
C -2.074312 -0.626516 -0.144903
O 2.153974 0.547651 -1.490359
O -3.148865 -0.958873 -0.365031
C -0.372271 1.543121 0.591463
O -0.426503 2.644247 0.912292
C 2.137470 -0.787971 0.527222
C 1.058154 -1.625013 1.232234
H 1.379282 -1.976421 2.217052
H 2.960426 -1.403154 0.148251
H 0.760611 -2.495620 0.647001
H 0.155501 -1.013189 1.469578
H -0.166682 -1.036094 -1.288113
H -0.639286 0.621403 -1.405063
FREQS= 69 75 89 91 120 207 285 323 331 358 381 439 457 484 498 518 578 604 655 683 770
778 897 1014 1037 1106 1255 1308 1428 1460 1486 1563 1845 2018 2124 2153 2179 2842 3050 3092 3106 3156
MIN036_6
16
E= -1803.03019378 ZPE= 68.12 Gcorr 0.048779000
H 2.499855 0.757445 -0.740120
Co 0.142160 -0.231755 -0.100788
C 0.473051 1.134136 1.025118
C -1.330573 -0.995221 0.620745
O 0.644359 2.069155 1.667245
O -2.243004 -1.565625 1.010159
C -0.811925 0.873327 -1.311591
O -1.990766 1.103700 -1.226738
C 2.155645 -1.279640 -0.001626
C 1.899879 -0.129395 -0.941731
H 2.287328 -2.238665 -0.504239
H 2.967876 -1.104425 0.708497
H 1.264929 -1.469526 0.698253
H 1.933444 -0.387256 -1.997647
H -0.204963 1.321099 -2.127143
H -0.187963 -1.098376 -1.267138
FREQS= 73 78 98 108 134 150 184 295 317 337 414 425 441 482 505 537 565 626 707 823 860
895 919 1001 1037 1137 1181 1333 1410 1461 1519 1565 1793 1979 2130 2170 2525 2893 3083 3115 3157 3188
MIN037_6
16
E= -1803.02944381 ZPE= 69.39 Gcorr 0.048509000
H 2.958450 1.134874 -0.591363
Co -0.656647 0.124346 -0.381232
C 0.866686 -0.487834 0.358639
C -0.693560 1.801658 0.061696
O 1.183782 -0.696049 1.625592
O -0.858510 2.875224 0.456816
C -1.793262 -1.160784 -0.118343
O -2.633845 -1.901598 0.164631
C 3.215849 0.081574 -0.451640
C 1.971275 -0.810940 -0.628459
H 3.665673 -0.022685 0.541818
H 3.979900 -0.186320 -1.185806
H 1.584919 -0.705340 -1.648677
H 2.239979 -1.872017 -0.506230
H 2.088591 -1.047368 1.731068
H -1.721399 0.578841 -1.355220
FREQS= 41 60 70 80 89 145 224 257 302 348 365 472 516 520 530 592 615 622 699 748 798
900 1016 1058 1087 1257 1313 1329 1420 1430 1485 1510 1518 1907 2066 2122 3009 3050 3075 3121 3142 3635
MIN038_6
16
E= -1803.02299019 ZPE= 69.45 Gcorr 0.043588000
H -4.383390 -0.202371 -0.243803
Co 0.221742 -0.006765 -0.076288
C 0.952857 -1.616659 -0.207386
C 1.095569 1.122112 -0.854076

O 1.631852 -2.546850 -0.277922
O 2.072605 1.869296 -1.258210
C 0.068310 0.318016 1.655345
O 0.189350 0.615986 2.764958
C -3.482684 0.394785 -0.418129
C -2.292730 -0.504744 -0.751995
H -3.294002 0.984817 0.483607
H -3.697217 1.091296 -1.234529
H -2.467267 -1.105596 -1.648715
H -1.410877 0.131635 -0.998248
H -2.076952 -1.196108 0.065805
H 2.144275 2.690481 -0.737497
FREQS= 41 41 57 74 76 92 107 141 183 210 275 316 343 393 482 522 527 562 578 670 821
869 1013 1100 1202 1244 1380 1432 1503 1507 1515 1518 1564 2068 2120 2832 3051 3089 3115 3123 3140 3690
MIN039_6
16
E= -1803.02634851 ZPE= 69.20 Gcorr 0.047452000
H 0.719203 -1.244045 3.209090
Co -0.048273 0.067088 -0.219534
C -1.639905 0.742671 -0.068157
C 1.476526 0.990640 -0.490353
O -2.628265 1.325431 0.066724
O 2.307971 1.749706 -0.723471
C -0.500897 -1.410574 -1.378749
O 0.350697 -2.192944 -0.965820
C 0.607689 -1.167311 2.118483
C 0.133332 0.233401 1.739601
H -0.101487 -1.945607 1.816577
H 1.576826 -1.414512 1.674741
H 0.862982 0.992931 2.029632
H -1.491961 -1.719046 -1.742229
H -0.807881 0.476809 2.238021
H -0.158003 -0.348405 -1.842818
FREQS= 36 49 84 102 109 127 172 214 219 283 329 413 449 468 506 514 535 555 778 803 955
975 1013 1107 1233 1260 1316 1420 1458 1499 1503 1508 1715 2027 2077 2130 3020 3037 3073 3087 3110 3132
MIN040_6
16
E= -1803.02781766 ZPE= 68.24 Gcorr 0.049100000
H 0.620955 -2.530857 -0.572959
Co 0.104657 0.011326 -0.118961
C 1.909179 -0.264448 -0.038479
C -1.725866 0.257576 -0.708547
O 3.030298 -0.391249 -0.240291
O -2.677266 -0.070227 -0.036083
C 0.104831 1.749743 0.277000
O 0.102280 2.884274 0.453072
C -0.506592 -1.699994 1.123254
C -0.238967 -1.908955 -0.336544
H 0.192634 -2.209489 1.787629
H -0.402312 -0.587629 1.453883
H -1.543478 -1.878041 1.406188
H -1.106109 -2.180413 -0.931575
H 0.410932 0.185172 -1.533513
H -1.896361 0.709526 -1.711380
FREQS= 51 78 87 114 135 209 230 298 361 386 399 425 445 481 492 528 546 599 629 829 857
892 919 1004 1053 1137 1172 1373 1389 1458 1513 1643 1776 2072 2119 2158 2380 2864 3105 3137 3183 3211
MIN041_6
16
E= -1803.02387631 ZPE= 67.22 Gcorr 0.045521000
H 1.174381 -1.299797 -2.326750
Co 0.131620 -0.007138 -0.302095
C 0.857124 -0.921854 1.074035
C -1.622293 0.043919 0.174689
O 1.422212 -1.457245 1.915353
O -2.515755 0.565425 0.759936
C 0.546832 1.621991 0.058901
O 0.872844 2.712673 0.222548
C -1.753132 -1.252322 -0.732691
C 1.633363 -0.774361 -1.481188
H -0.834389 -1.620697 -1.214585
H -2.134909 -2.047950 -0.092269
H -2.459393 -1.005701 -1.524550
H 2.300434 -1.476267 -0.975138
H 2.245620 0.030506 -1.892557
H -0.051264 0.741571 -1.562749
FREQS= 57 70 87 103 106 145 157 251 289 355 385 420 431 469 483 492 520 557 565 643 716
734 779 833 941 1048 1267 1350 1446 1473 1487 1497 1911 2036 2125 2160 3018 3040 3119 3126 3146 3190

MIN042_6
16
E= -1803.02420634 ZPE=   67.74 Gcorr   0.046370000
H -2.832397 -0.526939 0.720937
Co 0.290287 -0.267747 -0.379567
C 0.084606 -0.812149 1.367323
C 2.068195 -0.049212 -0.310702
O -0.369149 -0.118009 2.234681
O 3.215432 -0.042561 -0.321460
C -0.321347 1.453607 -0.123085
O -0.662053 2.541951 -0.045366
C -2.752251 -0.391900 -0.363062
C -1.458751 -0.994066 -0.908072
H -2.832935 0.682417 -0.560950
H -3.631752 -0.870736 -0.813500
H -1.504251 -2.082950 -0.839784
H -1.352994 -0.765039 -1.988675
H 0.701557 -1.643648 -0.748075
H 0.418481 -1.852670 1.561115
FREQS=   67   74   85   92 101 129 173 225 275 318 370 388 411 434 491 518 542 629 656 786 827
884 911 1013 1044 1207 1273 1288 1425 1447 1509 1512 1806 2008 2131 2170 2917 2919 3025 3083 3101 3118
MIN043_6
16
E= -1803.02023236 ZPE=   67.52 Gcorr   0.043801000
H 1.501991 -0.456065 1.726399
Co -0.297326 -0.068953 -0.095259
C -0.425135 1.673119 -0.368352
C 0.444801 -1.652152 -0.367499
O -0.424660 2.782375 -0.664975
O 0.986874 -2.618426 -0.668700
C -2.079958 -0.538042 -0.077577
O -2.126393 -0.549995 1.140022
C 2.677702 0.621035 0.257829
C 1.381601 0.380019 1.028933
H 2.973412 -0.251271 -0.334948
H 2.591914 1.466072 -0.433989
H 3.508738 0.845836 0.940265
H 1.137701 1.257276 1.636709
H -2.958365 -0.768709 -0.708055
H -0.208234 -0.046897 -1.545173
FREQS=   17   62   86   98 105 128 152 209 240 245 365 442 465 473 489 524 539 558 681 719 739
870 954 978 1039 1217 1256 1262 1428 1489 1511 1512 1718 2074 2111 2162 2971 3020 3047 3082 3087 3110
MIN044_6
16
E= -1803.02287423 ZPE=   67.17 Gcorr   0.046556000
H -2.571261 -1.230026 -0.715735
Co 0.136801 0.011587 -0.388544
C -1.156908 -0.530251 0.767692
C 0.597179 1.452020 0.451552
O -1.707694 -0.430451 1.816361
O 0.910455 2.432813 0.961746
C 1.533422 -1.050834 -0.011104
O 2.496538 -1.650546 0.159979
C -0.989759 1.271135 -1.489087
C -1.564893 -1.511747 -0.405402
H -1.406148 0.666391 -2.299919
H -1.806277 1.695205 -0.897763
H -0.426190 2.084654 -1.946186
H -1.534526 -2.519811 0.008396
H -0.931369 -1.508103 -1.307531
H 0.873502 -0.097633 -1.737182
FREQS=   61   72   95 112 140 144 206 260 353 367 410 419 438 457 491 519 529 537 564 604 652
705 820 854 942 1050 1281 1356 1450 1467 1486 1490 1817 1912 2120 2157 3000 3048 3119 3132 3162 3187
MIN045_6
16
E= -1803.01868918 ZPE=   64.48 Gcorr   0.042484000
H -2.357458 -2.586322 -0.642440
Co 0.315106 0.444371 -0.382624
C 2.005147 0.001161 -0.123029
C -1.031294 1.455415 0.159358
O 3.118926 -0.180665 0.100815
O -1.816078 2.191886 0.565605
C -0.280937 -0.998101 0.843589
O -0.322987 -1.124553 2.021169
C -1.986687 -1.593080 -0.924126
C -0.545330 -1.407430 -0.473510
H -2.648797 -0.848349 -0.474018

```
H -2.071960 -1.495572 -2.010600
H 0.163604 -2.144970 -0.846397
H 0.153192 0.381646 -2.072150
H 0.471871 1.106368 -1.968685
H 0.977396 1.748059 -0.049261
FREQS=   66   76   96  108  168  190  240  282  304  322  331  419  432  465  482  493  518  548  552  587  620
682  838  866  887 1046 1069 1127 1344 1356 1427 1501 1513 1942 1991 2099 2152 3036 3103 3126 3150 3552
MIN046_6
16
E= -1803.01949237 ZPE=    66.83 Gcorr    0.043485000
H -0.498546 0.407780 1.969037
Co -0.184874 0.000115 0.358216
C 0.192095 1.491522 -0.509239
C 0.194461 -1.490283 -0.509845
O 0.459498 2.413131 -1.146598
O 0.463180 -2.411240 -1.147569
C 1.665843 0.001762 0.906600
O 2.655841 -0.000888 0.223407
C -2.252480 -0.691140 0.107512
C -2.253696 0.686456 0.103327
H -2.501279 -1.250227 1.002801
H -2.339062 -1.240935 -0.823070
H -2.340940 1.230422 -0.830625
H -2.503597 1.250529 0.995177
H -0.497228 -0.404702 1.969957
H 1.766755 0.006095 2.020847
FREQS=   13   65   72  114  122  156  164  264  277  341  398  427  441  492  503  523  532  603  692  705  822
837  853  882  978  989 1020 1238 1306 1374 1465 1489 1597 1804 2091 2131 2800 3168 3175 3245 3267 3318
MIN047_6
16
E= -1803.02453642 ZPE=    67.96 Gcorr    0.048864000
H 0.077713 -2.434758 1.692855
Co 0.093281 -0.017396 0.004813
C 1.725326 0.739224 -0.289409
C -1.639177 -0.848769 -0.248145
O 2.658991 1.240636 -0.726812
O -2.527751 -0.586149 -1.014024
C -0.783240 1.458758 0.489200
O -1.375621 2.412098 0.724896
C 0.762046 -1.725941 1.220874
C 0.801445 -1.822260 -0.275622
H 1.740239 -1.754550 1.702217
H 0.332546 -0.713469 1.607584
H 1.792776 -1.895797 -0.715176
H 0.119787 -2.549482 -0.711339
H -1.773130 -1.719350 0.460619
H -0.051877 0.198328 -1.420573
FREQS=   63   79   88  112  125  191  273  346  353  369  407  415  446  453  490  529  545  583  636  790  853
880  912 1004 1049 1137 1175 1382 1391 1465 1516 1638 1815 2116 2123 2162 2369 2672 3090 3131 3162 3203
MIN048_6
16
E= -1803.02215568 ZPE=    66.63 Gcorr    0.046533000
H 0.185589 0.514615 -1.832492
Co -0.012282 -0.556863 -0.148001
C -1.738840 -0.879433 0.001117
C 1.698956 -0.967102 -0.055661
O -2.826771 -1.208297 0.156162
O 2.771518 -1.357499 0.063413
C 0.066063 1.108600 0.824316
O -0.010939 1.421415 1.982934
C -0.201504 1.542898 -1.645705
C 0.288881 2.126359 -0.309284
H -1.291256 1.517089 -1.705725
H 0.163043 2.109365 -2.507674
H 1.370679 2.307840 -0.347140
H -0.194052 3.080071 -0.069319
H -0.051463 -1.709601 -1.174869
H -0.002714 -1.216970 1.124493
FREQS=   74   78   94   97  120  166  281  331  341  358  387  458  486  488  529  543  574  594  624  627  751
775  893 1008 1030 1111 1251 1308 1436 1462 1483 1557 1781 1834 2124 2147 2176 2854 3044 3088 3101 3143
MIN049_6
16
E= -1803.02202018 ZPE=    67.17 Gcorr    0.046458000
H 0.895508 2.991146 -0.069369
Co 0.000002 0.142371 -0.186961
C -1.743398 -0.113239 -0.402472
C 1.743487 -0.112649 -0.402388
```

O -2.848322 -0.337617 -0.614254
O 2.848465 -0.336758 -0.614187
C 0.000254 -1.459791 0.884852
O 0.000433 -2.575639 0.443782
C -0.000400 2.377073 0.043370
C -0.000420 1.489601 1.253556
H -0.000325 1.768949 -0.950123
H -0.896369 2.991038 -0.069513
H 0.897157 1.549158 1.866259
H -0.898218 1.548724 1.865970
H 0.000216 -0.500336 -1.562132
H 0.000224 -1.278560 1.982628
FREQS= 43 69 91 115 139 144 172 327 331 375 423 450 462 497 512 521 542 575 608 620 799
882 909 969 1047 1116 1184 1322 1401 1466 1514 1603 1815 1873 2118 2167 2368 2873 3085 3119 3152 3189
MIN050_6
16
E= -1803.02032898 ZPE= 67.59 Gcorr 0.044782000
H 1.259263 -2.217958 -0.605067
Co -0.242193 -0.018245 -0.326502
C 0.442780 -1.111844 1.022976
C -0.450020 1.584519 0.396121
O 0.706088 -0.994671 2.176186
O -0.488103 2.562565 1.002095
C 1.296637 0.421590 -1.133058
O 2.475863 0.470795 -0.940100
C -2.626588 -0.560594 -0.691468
C 0.319064 -1.894111 -0.165330
H -2.009966 -0.320453 -1.566523
H -2.083052 -0.504765 0.268117
H -2.993252 -1.580992 -0.806551
H -0.468985 -2.648219 -0.161084
H 0.844612 0.668341 -2.157814
H -3.451415 0.149793 -0.636398
FREQS= 57 62 89 94 104 141 150 171 275 297 313 334 353 440 452 475 495 495 535 614 731
822 889 977 987 1217 1275 1365 1407 1419 1536 1595 1861 1955 2121 2531 2948 3067 3106 3138 3189 3200
MIN051_6
16
E= -1803.02298175 ZPE= 68.12 Gcorr 0.047586000
H 2.400962 0.135095 2.057530
Co -0.152951 -0.031170 -0.036235
C 0.590161 -1.669534 -0.342930
C -0.645691 1.690639 -0.320307
O 0.427612 -1.526495 -1.544517
O -0.896304 2.804374 -0.415366
C -1.769850 -0.691051 0.408754
O -2.731763 -1.069181 0.906886
C 2.721116 -0.004393 1.020464
C 1.749524 0.648695 0.039169
H 2.833280 -1.083129 0.860774
H 3.723061 0.435132 0.924488
H 1.753830 1.727033 0.223801
H 2.093597 0.509610 -0.995739
H 1.076698 -2.551400 0.107389
H -0.019681 0.153529 1.393181
FREQS= 56 64 89 105 118 190 226 239 268 279 389 427 450 457 488 515 536 575 696 711 743
895 958 1010 1050 1237 1252 1297 1427 1472 1508 1511 1706 2098 2124 2163 3000 3019 3025 3074 3090 3113
MIN052_6
16
E= -1803.02458350 ZPE= 68.07 Gcorr 0.049662000
H -1.717288 -2.152288 0.467706
Co 0.101360 -0.055495 -0.238228
C 1.871527 -0.524055 -0.297771
C -1.659132 0.454185 -0.877638
O 2.983933 -0.684425 -0.523112
O -2.655969 -0.163463 -0.581450
C 0.290061 1.578492 0.318213
O 0.411708 2.652032 0.713682
C -0.673739 -1.881973 0.631004
C -0.488137 -0.728445 1.535358
H -0.382812 -1.642103 -0.499912
H -0.028585 -2.737662 0.834720
H -1.400908 -0.256064 1.886586
H 0.274768 -0.825655 2.302164
H 0.297381 -0.044607 -1.736826
H -1.740134 1.334361 -1.550230
FREQS= 60 87 95 112 147 215 232 310 397 407 431 453 463 513 530 541 574 589 647 738 821
895 909 977 1089 1126 1180 1372 1380 1454 1507 1725 1778 1901 2114 2139 2157 2889 3099 3147 3184 3230

MIN053_6
16
E= -1803.01827043 ZPE=   65.46 Gcorr   0.044110000
H 0.575957 -1.991973 0.932296
Co -0.335058 -0.081037 -0.581755
C 1.516427 -0.659048 -0.400251
C -1.970820 -0.491756 0.047044
O 2.512622 -0.640872 -1.055421
O -2.939251 -0.905134 0.505198
C -0.114651 1.622058 -0.047101
O -0.002971 2.718555 0.272171
C 1.240952 -0.224676 2.089430
C 0.857082 -0.948892 0.812926
H 2.030324 -0.781777 2.609199
H 1.621485 0.781129 1.891981
H 0.389554 -0.133327 2.771049
H -0.426665 -1.477764 -1.089168
H -0.848830 0.379995 -2.061656
H -0.032404 0.245197 -2.134184
FREQS=   55   79   82   99 178 192 258 278 295 355 367 426 440 460 491 508 535 557 589 618 747
863 888 894 1032 1052 1071 1144 1351 1425 1497 1513 1665 1954 1988 2122 2154 3036 3101 3127 3138 3171
MIN054_6
16
E= -1803.01842035 ZPE=   65.88 Gcorr   0.045017000
H -0.073443 -2.537641 -0.988331
Co 0.003345 0.085604 -0.473993
C 0.033219 -1.369719 0.789954
C -0.868352 1.408480 0.327609
O -0.298415 -1.501690 1.922421
O -1.481214 2.195040 0.897388
C 1.662763 0.659325 -0.094508
O 2.721841 1.026891 0.149718
C -1.861306 -0.565821 -0.947506
C 0.560028 -1.859966 -0.423942
H -1.763388 -1.175200 -1.850640
H -2.311061 -1.179476 -0.162929
H -2.558804 0.247170 -1.166433
H 1.622487 -2.086741 -0.432956
H 0.015495 0.876431 -2.067468
H 0.282599 0.148440 -2.199296
FREQS=   70   86   93 116 125 158 273 296 344 394 416 434 445 447 470 489 519 533 551 565 590
710 772 829 866 887 997 1014 1252 1294 1432 1481 1492 1982 2115 2157 3045 3129 3138 3140 3229 3708
MIN055_6
16
E= -1803.02632116 ZPE=   70.89 Gcorr   0.052951000
H -0.064732 0.728000 1.810294
Co -0.071270 0.025417 0.154449
C 0.343928 -1.535162 -0.642365
C -0.297555 1.458957 -0.888694
O 1.502853 -1.975371 -1.129597
O -0.415268 2.201633 -1.764513
C -1.757533 -0.495552 0.409766
O -2.838136 -0.845821 0.601328
C 1.080784 0.830813 1.897587
C 1.784165 0.443501 0.623929
H 1.328560 0.197877 2.750806
H 1.189995 1.884134 2.164568
H 2.286799 1.264581 0.115621
H 2.470080 -0.396579 0.763546
H 2.196618 -1.348932 -0.867915
H -0.401369 -2.274193 -0.966123
FREQS=   54   80   91 100 120 173 216 292 338 362 386 421 463 497 521 530 547 669 684 814 911
991 999 1047 1146 1192 1236 1308 1392 1466 1503 1513 1588 2072 2121 2479 3052 3069 3089 3149 3165 3747
MIN056_6
16
E= -1803.01952040 ZPE=   66.71 Gcorr   0.047118000
H -1.119113 -2.296600 0.276250
Co 0.144854 -0.031870 -0.322075
C 1.952412 -0.240330 -0.218817
C -1.686917 -0.020758 -0.973489
O 3.082692 -0.364821 -0.355878
O -2.614584 -0.550918 -0.407395
C -0.074241 1.611010 0.332808
O -0.216812 2.699813 0.667301
C -0.260559 -1.851246 0.762436
C -0.442479 -0.792206 1.623345
H 0.134478 -1.072029 -1.369295

```
H 0.648717 -2.442385 0.792750
H -1.443922 -0.416465 1.798283
H 0.321873 -0.519332 2.343878
H 0.473043 0.588146 -1.600581
H -1.865790 0.507758 -1.935192
FREQS=   71   82   90  110  170  194  231  297  347  362  403  436  452  454  514  518  580  641  648  687  713
839  865  920 1004 1036 1052 1246 1307 1379 1468 1585 1782 2044 2086 2132 2162 2879 3166 3175 3260 3281
MIN001_7
12
E= -1837.78715399 ZPE=    41.70 Gcorr    0.005720000
H 3.159271 1.368985 -1.269709
Co -0.114560 0.019809 -0.153264
C 2.648696 0.722273 -0.543677
C -1.023444 1.497267 0.385982
O 1.283599 1.061543 -0.539647
O -1.527437 2.393874 0.888059
C -1.575970 -0.934726 -0.396505
O -2.501490 -1.551505 -0.689896
C 0.794475 -1.353761 0.511240
O 1.359553 -2.162730 1.098905
H 2.845565 -0.321929 -0.832047
H 3.111953 0.902324 0.438282
FREQS=   48   77   87   93  101  128  166  278  301  379  410  436  457  481  519  552  591  647 1110 1167 1194
1481 1500 1506 2102 2116 2181 2973 3029 3058
MIN002_7
12
E= -1837.78851173 ZPE=    42.89 Gcorr    0.010978000
H 3.250524 1.401882 -0.000013
Co -0.195951 -0.001622 -0.000008
C 2.410107 -0.715015 0.000018
C -1.394253 -1.251427 -0.000006
O 1.144424 -1.252995 0.000016
O -2.171753 -2.098041 -0.000005
C -1.403025 1.248491 0.000002
O -2.179951 2.100809 0.000023
C 2.354187 0.765865 -0.000006
O 1.232102 1.299111 -0.000020
H 3.019754 -1.018349 -0.877694
H 3.019737 -1.018318 0.877754
FREQS=   36   66  102  113  170  292  350  364  420  477  485  502  539  604  671  672  824  911 1062 1167 1206
1337 1388 1427 1671 2094 2152 2909 2916 3071
MIN003_7
12
E= -1837.78131534 ZPE=    42.36 Gcorr    0.008170000
H 1.489760 0.667495 2.126593
Co 0.042304 -0.007724 0.095575
C 1.910072 -0.301371 0.450218
C -1.661976 0.276324 0.561806
O 1.335287 -0.213851 1.759785
O -2.743560 0.461003 0.904477
C 0.310570 1.482541 -0.806901
O 0.473319 2.365658 -1.534452
C -0.177752 -1.559587 -0.728022
O -0.300312 -2.491381 -1.396953
H 2.643868 0.480599 0.256917
H 2.320806 -1.298399 0.310508
FREQS=   55   79   81  104  106  135  275  300  406  417  471  506  512  532  544  560  568  601  844  991 1066
1204 1319 1516 2069 2091 2153 3105 3198 3821
MIN004_7
12
E= -1837.77723862 ZPE=    40.61 Gcorr    0.004583000
H -0.021908 -0.698149 1.814993
Co 0.000085 -0.002426 0.126149
C -0.046889 -1.804613 1.444112
C -1.628853 0.020527 -0.647040
O -0.046899 -1.879298 0.068238
O -2.589381 -0.034267 -1.271652
C 0.043428 1.715895 0.537587
O 0.071537 2.826725 0.827287
C 1.628106 -0.061651 -0.646298
O 2.584667 -0.165202 -1.270851
H 0.846428 -2.241674 1.913541
H -0.960973 -2.199284 1.911110
FREQS=   52   80   88   99  105  148  185  283  288  402  409  451  455  469  520  552  571  605 1062 1147 1208
1347 1512 1563 2114 2116 2178 2356 2995 3048
MIN005_7
12
```

E= -1837.77223079 ZPE= 39.44 Gcorr 0.003732000
H 0.142979 1.027128 -1.313549
Co 0.017112 0.074868 -0.186675
C -1.094274 -1.086348 -1.487265
C -0.148041 -1.026478 1.259013
O 0.139011 -1.429966 -1.459377
O -0.232113 -1.704849 2.175250
C 1.732960 0.581236 -0.013345
O 2.800775 0.993695 0.032605
C -1.148642 1.321741 0.241629
O -1.874794 2.187230 0.453545
H -1.843165 -1.683631 -0.947680
H -1.476889 -0.474715 -2.314938
FREQS= 66 73 82 107 124 130 328 340 375 411 419 456 486 497 513 525 545 710 748 832 1064
1222 1397 1597 2005 2122 2147 2187 3000 3081
MIN006_7
12
E= -1837.76632909 ZPE= 38.59 Gcorr -0.001126000
H -2.694508 -0.000300 1.233370
Co 0.146280 0.000035 -0.143050
C -1.882232 -0.000360 1.975454
C -1.263963 0.000998 -1.228759
O -0.706944 -0.000237 1.641346
O -2.048274 0.001630 -2.076484
C 1.084788 1.512656 0.025904
O 1.749636 2.452684 -0.016667
C 1.083557 -1.513462 0.024813
O 1.747649 -2.453995 -0.018416
H 0.820552 0.000242 -1.435566
H -2.145047 -0.000531 3.041840
FREQS= 32 59 60 86 96 111 230 302 318 337 359 422 444 456 462 510 515 525 725 729 1167
1267 1527 1755 2069 2075 2086 2153 3007 3114
MIN007_7
12
E= -1837.76529481 ZPE= 38.50 Gcorr -0.000691000
H -0.605977 -0.000778 -1.491782
Co 0.014003 -0.000044 -0.128191
C -2.957604 -0.002596 0.213539
C 0.944928 0.000914 1.397136
O -1.851501 -0.001906 0.730277
O 1.600814 0.001524 2.340277
C 0.535872 -1.558355 -0.745258
O 0.930448 -2.508486 -1.268412
C 0.532569 1.559367 -0.745241
O 0.925152 2.510345 -1.268356
H -3.084870 -0.002310 -0.879950
H -3.861121 -0.003521 0.841540
FREQS= 52 53 82 83 102 104 204 253 302 323 349 390 449 481 500 519 552 571 739 740 1167
1261 1525 1756 1947 2079 2103 2157 2993 3092
MIN008_7
12
E= -1837.76783676 ZPE= 38.89 Gcorr 0.002533000
H -0.926818 -2.280198 1.017596
Co 0.000007 -0.027761 -0.116611
C -0.000017 -1.699562 1.122934
C -1.683316 -0.373620 -0.591723
O 0.000010 -0.595016 1.773441
O -2.736124 -0.569365 -1.005435
C -0.000025 1.769631 0.229970
O -0.000053 2.892114 0.447765
C 1.683344 -0.373588 -0.591700
O 2.736156 -0.569313 -1.005412
H 0.000040 0.105440 -1.586413
H 0.926757 -2.280237 1.017577
FREQS= 59 79 90 107 115 138 276 328 377 403 426 430 466 469 495 510 555 624 704 739 1086
1215 1388 1596 2013 2116 2138 2184 3002 3074
MIN009_7
12
E= -1837.77718651 ZPE= 43.43 Gcorr 0.012033000
H 1.662853 -0.334935 -2.036073
Co -0.167833 -0.065591 -0.081719
C 1.545512 -0.506743 -0.963945
C -0.766120 1.552750 -0.082902
O 0.919509 -1.567112 -0.488351
O -1.152696 2.637923 -0.159036
C -1.700705 -0.879606 0.205420
O -2.698401 -1.428258 0.357829

C 1.879973 0.449880 0.002313
O 2.049766 0.089248 1.309896
H 2.280970 1.426256 -0.238298
H 1.890277 -0.872472 1.352773
FREQS= 54 72 95 110 165 261 331 373 431 448 450 495 543 595 605 632 787 892 930 1042 1195 1251 1344 1442 1541 2091 2151 3128 3247 3679
MIN010_7
12
E= -1837.76301668 ZPE= 38.33 Gcorr -0.002040000
H -3.749150 -0.000161 -1.168778
Co 0.161966 0.000004 -0.215169
C -2.861427 -0.000100 -0.518313
C -0.174024 -0.000007 1.531900
O -1.742494 -0.000091 -1.003504
O -0.303592 -0.000011 2.676070
C 0.937597 1.569571 -0.336692
O 1.564431 2.530045 -0.462948
C 0.937738 -1.569494 -0.336688
O 1.564658 -2.529912 -0.462941
H 0.697546 0.000028 -1.608380
H -3.024783 -0.000048 0.572057
FREQS= 35 49 72 75 101 102 182 248 298 321 347 376 456 481 502 518 554 562 715 730 1165 1264 1530 1762 1974 2082 2097 2151 2982 3080
MIN011_7
12
E= -1837.77556659 ZPE= 42.91 Gcorr 0.010510000
H 1.662638 -0.434384 1.466394
Co -0.290529 -0.111862 0.064891
C 1.744150 -0.376285 0.378147
C -2.046729 -0.224582 0.007487
O 2.811334 0.314270 -0.141711
O -3.191889 -0.303412 -0.041670
C -0.180885 1.602957 -0.042251
O -0.045592 2.747931 -0.124425
C 1.203559 -1.423454 -0.382995
O 0.089474 -1.939644 0.058929
H 2.966040 1.106476 0.387067
H 1.588422 -1.676790 -1.376832
FREQS= 43 79 85 94 219 238 313 362 399 443 445 473 487 513 573 634 648 812 972 1139 1208 1262 1290 1424 1578 2083 2149 3099 3112 3844
MIN012_7
12
E= -1837.77055134 ZPE= 41.44 Gcorr 0.006750000
H -0.808683 0.495561 -1.657310
Co 0.121413 0.027202 -0.179612
C 0.723742 1.539399 0.515550
C -1.480450 0.817687 -0.750351
O 1.082674 2.460409 1.107476
O -2.738050 0.277262 -0.763584
C -0.609732 -1.269829 0.762231
O -1.052413 -2.026192 1.513518
C 1.698353 -0.760558 -0.537909
O 2.684731 -1.261662 -0.847321
H -1.602797 1.899900 -0.749062
H -2.673681 -0.688650 -0.761948
FREQS= 54 81 88 92 100 187 308 321 342 403 442 502 505 515 533 555 575 605 815 1124 1172 1258 1345 1473 2077 2098 2156 2316 3155 3789
MIN013_7
12
E= -1837.77440536 ZPE= 42.98 Gcorr 0.010823000
H 1.619341 -1.625481 1.378895
Co -0.286052 -0.115053 -0.074687
C 1.240779 -1.389291 0.374510
C -0.227455 1.606930 0.020135
O 0.130156 -1.929958 -0.055244
O -0.152497 2.755055 0.113677
C -2.034650 -0.267685 -0.001366
O -3.176143 -0.378786 0.071700
C 1.776589 -0.346979 -0.392164
O 2.819707 0.441030 0.011666
H 2.916645 0.391508 0.972679
H 1.686067 -0.376180 -1.476112
FREQS= 43 79 92 97 203 303 309 364 391 438 447 467 487 516 574 630 648 820 948 1111 1258 1278 1310 1413 1564 2090 2150 3050 3181 3809
MIN014_7
12
E= -1837.76875190 ZPE= 41.14 Gcorr 0.005334000

H 1.599225 1.920829 -0.635365
Co -0.150053 0.024604 -0.172357
C 1.467840 0.839470 -0.721576
C -1.721379 -0.792316 -0.467710
O 2.685217 0.168073 -0.742125
O -2.707292 -1.312811 -0.746838
C -0.758994 1.548732 0.469527
O -1.113227 2.482995 1.046485
C 0.668069 -1.291490 0.681324
O 1.168768 -2.054899 1.382804
H 3.355488 0.723681 -0.324934
H 0.895763 0.597950 -1.678050
FREQS= 52 78 90 91 98 154 216 298 323 364 403 446 496 510 524 553 563 596 741 1110 1144
1246 1336 1462 2079 2099 2159 2615 3097 3832
MIN015_7
12
E= -1837.76380915 ZPE= 38.65 Gcorr 0.000620000
H 0.000003 -1.185405 -1.123406
Co 0.000001 -0.106981 -0.089480
C 0.000034 -2.058086 0.913256
C -0.000006 1.515428 0.677143
O -0.000033 -1.105431 1.728214
O -0.000014 2.567117 1.130396
C 1.635393 -0.006706 -0.761169
O 2.642320 0.053990 -1.314422
C -1.635391 -0.006722 -0.761176
O -2.642315 0.053963 -1.314436
H -0.935098 -2.573393 0.646489
H 0.935226 -2.573310 0.646540
FREQS= 41 63 86 90 114 118 241 326 337 354 395 402 456 464 492 555 557 622 658 752 1111
1241 1474 1646 1983 2099 2128 2178 2989 3068
MIN016_7
12
E= -1837.77418361 ZPE= 43.36 Gcorr 0.011794000
H -1.277986 1.990091 0.555577
Co 0.194730 -0.007761 -0.014641
C -2.540014 0.532582 0.023848
C 1.409463 -1.236634 0.003606
O -1.297865 1.243491 -0.059622
O 2.197507 -2.073142 0.024056
C 1.372987 1.261973 -0.008527
O 2.129910 2.133959 0.000891
C -2.346316 -0.792817 0.003450
O -1.121776 -1.303901 0.006898
H -3.178227 -1.496337 -0.020162
H -3.440426 1.121908 -0.052158
FREQS= 37 67 101 111 210 335 353 395 435 472 481 495 517 542 590 596 679 834 913 987 1138
1219 1274 1375 1691 2088 2153 3161 3285 3798
MIN017_7
12
E= -1837.76469473 ZPE= 38.61 Gcorr 0.002371000
H -0.931665 -0.635762 2.296877
Co 0.000000 -0.088266 -0.156340
C -0.000001 -0.901052 1.777611
C 1.714048 -0.205655 -0.626684
O -0.000005 -1.804415 0.876704
O 2.789134 -0.308375 -1.009863
C -1.714049 -0.205649 -0.626683
O -2.789136 -0.308365 -1.009863
C 0.000003 1.617642 0.344995
O 0.000008 2.729336 0.638417
H 0.931666 -0.635766 2.296874
H -0.000001 -0.642438 -1.551162
FREQS= 67 85 88 115 119 128 282 356 380 406 413 426 460 468 504 526 562 592 627 694 1052
1225 1408 1601 1917 2119 2132 2183 2998 3077
MIN018_7
12
E= -1837.76264416 ZPE= 38.80 Gcorr 0.001128000
H 0.934351 0.733415 2.288809
Co 0.000000 -0.052459 -0.128452
C -0.000006 0.242164 1.985920
C 0.000006 1.731235 -0.249711
O -0.000002 -0.979747 1.637015
O 0.000008 2.861676 -0.460427
C 1.626994 -0.719702 -0.511675
O 2.645542 -1.144243 -0.825789
C -1.626995 -0.719696 -0.511678

```
O -2.645544 -1.144233 -0.825795
H -0.934369 0.733410 2.288803
H 0.000000 -0.002070 -1.586579
FREQS=   50   52   87   90  117  117  278  301  349  383  413  437  445  479  487  510  536  663  697  739 1064
1231 1423 1608 2077 2111 2119 2174 3010 3095
MIN019_7
12
E= -1837.77098578 ZPE=    43.16 Gcorr    0.011379000
H 1.708839 -0.649447 -1.990447
Co -0.164893 -0.079521 -0.075491
C 1.518866 -0.731034 -0.914047
C -0.661224 1.565970 -0.150466
O 0.771219 -1.684199 -0.433171
O -0.979113 2.669756 -0.283929
C -1.755226 -0.774582 0.235839
O -2.787956 -1.239332 0.425239
C 1.870997 0.322361 -0.051241
O 2.064565 0.054885 1.289639
H 2.353347 1.208016 -0.457715
H 1.999733 0.883306 1.783682
FREQS=   52   74   95  112  169  272  317  370  375  431  452  459  494  537  595  628  765  904  952 1028 1221
1247 1304 1467 1557 2083 2151 3082 3176 3819
MIN020_7
12
E= -1837.75562164 ZPE=    37.40 Gcorr   -0.001256000
H -2.307598 0.001246 -0.991328
Co 0.055915 0.000023 0.240224
C -1.908527 0.000544 0.049175
C 1.864643 -0.000518 0.450627
O -2.661390 0.000197 0.987086
O 3.004882 -0.000884 0.556178
C -0.016861 1.590325 -0.555504
O -0.065281 2.593723 -1.115801
C -0.017796 -1.590148 -0.555661
O -0.066804 -2.593449 -1.116083
H -0.211072 -0.406013 1.841239
H -0.211061 0.406239 1.841191
FREQS=   46   50   87   88  103  167  264  311  364  376  436  449  464  474  520  537  541  583  599  645  905
913 1367 1477 1809 2102 2120 2172 2850 3350
MIN021_7
12
E= -1837.75534615 ZPE=    37.40 Gcorr   -0.001070000
H -2.049029 0.000003 1.791446
Co 0.144861 -0.000003 0.392481
C -1.795364 0.000006 0.702151
C 1.951533 -0.000004 0.150584
O -2.675980 0.000014 -0.115212
O 3.079248 0.000000 -0.047858
C -0.132472 1.572424 -0.401238
O -0.314252 2.558939 -0.960643
C -0.132481 -1.572428 -0.401238
O -0.314268 -2.558942 -0.960643
H 0.296246 -0.412461 1.992436
H 0.296246 0.412458 1.992435
FREQS=   51   52   87   93  107  135  267  309  364  364  429  442  462  480  504  539  539  613  656  729  886
938 1375 1525 1809 2115 2127 2177 2805 3181
MIN022_7
12
E= -1837.76571964 ZPE=    43.09 Gcorr    0.010082000
H -1.581766 -1.561365 0.018278
Co 0.363886 -0.056359 -0.243155
C -1.452567 0.192957 -0.768401
C 1.024265 1.495265 0.034494
O -1.096699 -1.196579 -0.766038
O 1.415575 2.565283 0.222985
C 1.756765 -1.019930 0.204652
O 2.646447 -1.672759 0.534830
C -2.371682 0.465183 0.333871
O -2.657541 -0.418824 1.139765
H -2.770028 1.490301 0.428840
H -1.676067 0.574944 -1.761975
FREQS=   40   53   91   99  116  280  291  378  410  425  465  513  565  610  660  784  878  895  923 1047 1082
1232 1355 1407 1725 2081 2144 3009 3176 3409
MIN023_7
12
E= -1837.75182135 ZPE=    36.83 Gcorr   -0.003441000
H 0.361681 -0.378502 -2.050993
```

Co 0.140409 0.010932 -0.398578
C -1.564709 -0.587820 -0.657563
C 1.575691 -1.048696 -0.159950
O -2.640522 -0.946807 -0.819138
O 2.499171 -1.710765 0.017892
C -0.136624 -0.080586 1.505112
O -1.168050 -0.368073 2.051447
C 0.351858 1.781975 -0.191712
O 0.477836 2.913217 -0.026941
H 0.376250 0.419950 -2.056760
H 0.766259 0.173560 2.107963
FREQS= 35 47 57 96 120 167 218 308 309 339 366 416 464 478 496 503 517 548 581 627 829
902 1351 1420 1805 2097 2113 2171 2848 3535
MIN024_7
12
E= -1837.75171857 ZPE= 36.82 Gcorr -0.003496000
H 0.000045 -0.836879 2.129129
Co 0.000017 -0.205972 -0.388146
C -0.000026 0.094018 1.517686
C -1.621324 -0.950745 -0.168285
O -0.000122 1.171450 2.050383
O -2.671885 -1.393039 -0.014861
C -0.000043 1.593354 -0.682869
O -0.000078 2.726947 -0.849360
C 1.621407 -0.950631 -0.168243
O 2.671996 -1.392849 -0.014789
H 0.000045 -0.223778 -2.112141
H 0.000067 -0.994163 -1.897764
FREQS= 34 50 57 91 122 165 221 304 309 335 370 416 460 472 486 504 520 545 577 627 840
907 1345 1431 1805 2095 2114 2171 2864 3521
MIN025_7
12
E= -1837.75574272 ZPE= 39.70 Gcorr 0.001593000
H 0.258926 -0.210281 2.428631
Co 0.140435 -0.031648 -0.171176
C -0.420077 -0.100660 1.572881
C -1.309968 -0.630872 -0.998490
O -1.634269 -0.032086 2.076006
O -2.201499 -1.023855 -1.616103
C 0.457907 1.709379 -0.326981
O 0.680321 2.829602 -0.467150
C 1.622700 -1.003191 0.043019
O 2.592573 -1.620282 0.093192
H -2.280634 0.071081 1.355816
H 0.629581 -0.081280 -1.592830
FREQS= 21 58 69 94 108 121 314 319 330 411 449 461 477 510 516 544 636 668 707 752 1041
1243 1355 1494 1944 2086 2102 2154 3074 3715
MIN026_7
12
E= -1837.75937978 ZPE= 40.73 Gcorr 0.005305000
H -0.056596 0.471552 2.225574
Co 0.014515 0.291800 -0.140821
C 1.380268 1.706700 -0.035262
C -1.758618 -0.056913 -0.361203
O 0.128904 2.126533 -0.016475
O -2.853304 -0.326468 -0.560852
C 0.891860 -1.076848 -0.855305
O 1.464331 -1.898623 -1.416203
C 0.096321 -0.410588 1.553659
O 0.270012 -1.515726 1.970683
H 1.967963 1.852879 -0.948702
H 1.958204 1.737122 0.896760
FREQS= 63 73 84 98 114 133 177 313 385 401 414 461 475 503 506 568 625 788 843 1077 1207
1236 1293 1569 1852 2119 2167 2826 3018 3101
MIN027_7
12
E= -1837.75914702 ZPE= 40.90 Gcorr 0.005890000
H -2.603246 -1.116987 -0.711451
Co -0.189780 -0.206723 -0.148806
C -0.736897 1.431297 -0.509712
C -1.849317 -1.219705 0.075537
O -1.137238 2.454729 -0.849097
O -0.763064 -1.932357 -0.186249
C 0.196340 0.294216 1.590590
O 1.240714 0.662860 2.038841
C 1.563194 -0.315233 -0.713075
O 2.613852 -0.349690 -1.161179

H -2.245849 -1.240029 1.099783
H -0.700869 0.110771 2.230859
FREQS= 54 72 88 100 129 162 179 313 388 394 423 459 492 504 532 593 621 793 868 1070 1201
1257 1279 1558 1844 2113 2173 2837 3010 3105
MIN028_7
12
E= -1837.75285539 ZPE= 38.46 Gcorr -0.000401000
H -0.850511 0.000000 -1.677333
Co -0.083278 0.000000 -0.237785
C 2.851844 0.000000 -0.602148
C -0.018231 1.493505 0.728485
O 1.708920 0.000003 -1.041512
O -0.041820 2.395225 1.447667
C -1.731774 -0.000002 -0.706799
O -2.895719 -0.000003 -0.850429
C -0.018228 -1.493504 0.728486
O -0.041815 -2.395223 1.447667
H 3.058248 -0.000005 0.478192
H 3.702581 0.000002 -1.295961
FREQS= 46 59 81 84 108 111 216 287 311 319 375 380 454 480 495 498 555 573 587 1080 1143
1261 1523 1733 1799 2014 2077 2127 3009 3116
MIN029_7
12
E= -1837.74944850 ZPE= 37.88 Gcorr -0.003186000
H 0.680478 -2.763404 -0.701388
Co -0.225254 0.188477 -0.225517
C 1.258098 -1.821282 -0.753532
C -0.080886 -0.113084 1.522653
O 2.405991 -1.728011 -0.402494
O -0.050088 -0.231453 2.663878
C 0.751234 1.657358 -0.321207
O 1.275982 2.674023 -0.423299
C -1.891985 -0.318939 -0.448907
O -3.010864 -0.519520 -0.627290
H 0.719422 -0.955062 -1.264833
H -0.504990 0.644939 -1.625233
FREQS= 30 55 69 83 95 99 123 219 328 338 350 436 474 485 504 567 574 597 643 758 1218
1254 1482 1827 1969 2105 2124 2179 2545 2969
MIN030_7
12
E= -1837.75036380 ZPE= 40.51 Gcorr -0.001779000
H 2.774097 0.781855 -1.238925
Co -0.049288 -0.089529 -0.084770
C 2.377865 1.451177 -0.462003
C 1.078811 -1.266474 0.389691
O 0.996577 1.294243 -0.262210
O 1.883374 -2.016630 0.737143
C -1.334185 -1.262576 -0.280178
O -2.149280 -2.042695 -0.522768
C -2.154467 2.419725 0.641430
O -1.576689 1.492906 0.311359
H 2.944114 1.277417 0.464974
H 2.572570 2.484304 -0.779100
FREQS= 25 55 59 64 71 102 113 118 160 194 282 321 453 476 499 563 596 696 1141 1174 1194
1485 1503 1508 2082 2138 2173 2985 3042 3061
MIN031_7
12
E= -1837.74981596 ZPE= 38.08 Gcorr -0.002235000
H 1.389015 -2.555452 0.373390
Co -0.154088 -0.035122 -0.127254
C 1.608271 -1.538515 0.743387
C 0.837649 1.170914 -0.952286
O 2.721315 -1.096994 0.868925
O 1.407020 1.926481 -1.603867
C -1.538769 -1.053685 -0.503340
O -2.432639 -1.667804 -0.887488
C -0.928139 1.048760 1.059234
O -1.464126 1.777977 1.764732
H 0.699076 -0.970676 1.148726
H 0.345650 -0.807689 -1.306636
FREQS= 27 60 73 89 95 120 129 252 331 347 358 450 474 483 501 564 576 612 697 764 1195
1265 1474 1822 1980 2105 2125 2178 2502 2992
MIN032_7
12
E= -1837.75029379 ZPE= 38.03 Gcorr -0.001704000
H -1.839876 0.038869 -2.226191
Co 0.234947 0.135633 -0.179357

```
C -1.636583 -0.657047 -1.393990
C 0.089631 -0.900671 1.275557
O -2.496611 -1.256381 -0.798495
O 0.036301 -1.526621 2.233217
C 1.938825 -0.138015 -0.558848
O 3.060646 -0.181297 -0.807293
C -0.583947 1.572778 0.415990
O -1.010980 2.578106 0.776469
H -0.523045 -0.961342 -1.287351
H 0.456945 1.087665 -1.307264
FREQS=   40   58   75   88   96  118  135  249  327  352  367  443  472  484  503  554  575  666  725  768 1173
1322 1456 1810 1987 2106 2130 2181 2348 2996
MIN033_7
12
E= -1837.75771281 ZPE=    41.02 Gcorr    0.005868000
H -1.793512 -0.203409 -2.113106
Co 0.034101 -0.065732 -0.201021
C -0.402860 -0.232908 1.598271
C -0.327123 1.655008 -0.131112
O -1.337500 -0.919033 1.899058
O -0.532343 2.784450 -0.217102
C 1.826624 -0.272342 0.032785
O 2.971124 -0.303501 0.077779
C -1.515947 -0.769548 -1.217930
O -0.417608 -1.494145 -1.251993
H 0.255032 0.246535 2.354874
H -2.335807 -1.091793 -0.568216
FREQS=   57   71   84   99  120  160  186  271  383  402  419  465  476  502  531  594  633  779  874 1064 1214
1291 1307 1560 1824 2103 2156 2896 3038 3134
MIN034_7
12
E= -1837.75564954 ZPE=    40.21 Gcorr    0.004037000
H 2.155749 0.001543 1.402463
Co 0.017439 0.000022 -0.096123
C 1.785829 0.001166 0.368231
C -0.808948 1.473050 -0.690508
O 2.872039 0.001691 -0.389579
O -1.251702 2.434682 -1.139255
C -0.806867 -1.474478 -0.689767
O -1.248216 -2.437002 -1.137981
C -0.515408 0.000142 1.612952
O -0.836878 0.000258 2.715076
H 0.564803 0.000153 -1.476860
H 2.599019 0.001394 -1.321823
FREQS=   46   66   89   91  111  131  310  342  347  427  444  459  464  517  544  552  640  651  750  869 1023
1242 1337 1510 1980 2104 2121 2163 3055 3740
MIN035_7
12
E= -1837.74829038 ZPE=    37.93 Gcorr   -0.002697000
H -2.094044 0.022856 -1.822976
Co 0.150382 -0.071755 0.114084
C -1.823992 -0.787384 -1.121560
C -0.728390 1.089480 1.097220
O -2.632700 -1.502414 -0.590148
O -1.229737 1.810185 1.840393
C 1.526807 -1.124075 0.446781
O 2.403075 -1.783396 0.792009
C 0.902294 1.042021 -1.057984
O 1.434386 1.792420 -1.744659
H -0.699549 -1.039260 -1.081379
H -0.327226 -0.900830 1.256588
FREQS=   27   59   77   86   90  113  126  241  330  351  353  432  474  485  508  559  567  647  687  752 1184
1297 1465 1823 2002 2107 2122 2175 2414 2976
MIN036_7
12
E= -1837.75181518 ZPE=    40.64 Gcorr    0.001844000
H -2.771011 0.580437 -1.173210
Co 0.099777 -0.069873 -0.112063
C -2.745689 -0.334306 -0.563922
C -0.421755 1.476218 0.437304
O -1.446431 -0.836370 -0.375308
O -0.829390 2.488683 0.800711
C 1.759428 0.480624 -0.392599
O 2.795678 0.866808 -0.708142
C 0.693811 -1.623963 1.299781
O 0.847500 -2.201352 0.307674
H -3.238317 -0.128022 0.397533
```

H -3.338283 -1.099430 -1.081475
FREQS= 37 79 85 89 101 126 130 163 185 251 281 355 440 459 501 545 582 675 1130 1173 1192 1481 1501 1506 2011 2101 2150 2987 3045 3069
MIN037_7
12
E= -1837.74908129 ZPE= 37.01 Gcorr -0.000697000
H -1.674367 0.947012 -1.954151
Co 0.176651 0.035427 -0.310325
C -1.619191 0.295215 -1.057658
C 0.010418 1.531892 0.669480
O -2.618737 -0.200341 -0.603853
O -0.088919 2.533680 1.214827
C -0.428786 -1.319255 0.744403
O -0.796653 -2.216140 1.345513
C 1.965160 -0.311008 -0.243746
O 3.082708 -0.507019 -0.385638
H 0.602598 0.899197 -1.424218
H 0.109402 -0.865238 -1.484519
FREQS= 59 78 82 98 115 161 272 301 359 404 414 441 477 493 526 533 603 651 664 696 888 911 1366 1791 2024 2057 2150 2167 2205 2903
MIN038_7
12
E= -1837.75232456 ZPE= 40.26 Gcorr 0.003646000
H -2.757233 -0.000040 -0.049132
Co 0.171650 0.000005 -0.209121
C -1.313766 0.000030 -1.286860
C 1.164595 1.475009 -0.031422
O -2.612620 0.000004 -1.009049
O 1.800657 2.432955 -0.024107
C 1.164634 -1.474983 -0.031494
O 1.800738 -2.432901 -0.024206
C -0.733381 -0.000052 1.324432
O -1.381805 -0.000088 2.276600
H -1.263395 0.000075 -2.380894
H 0.837834 0.000048 -1.525542
FREQS= 41 65 79 81 110 118 304 341 343 433 450 456 467 534 548 565 626 653 740 870 1027 1255 1311 1508 2031 2105 2108 2156 3096 3745
MIN039_7
12
E= -1837.75281552 ZPE= 42.15 Gcorr 0.004242000
H -2.623263 0.080943 -1.525386
Co 0.692233 0.294218 -0.229095
C -2.221816 0.649804 -0.671949
C 2.271800 0.647142 0.399304
O -0.844094 0.807908 -0.777553
O 3.301058 0.936191 0.829463
C 0.968423 -1.379784 -0.207134
O 1.084837 -2.526899 -0.194996
C -2.633302 -0.067478 0.614227
O -3.779133 -0.260517 0.938319
H -2.713128 1.633401 -0.687226
H -1.765856 -0.409815 1.229628
FREQS= 22 39 41 90 100 106 208 293 341 414 445 484 523 586 624 715 724 1017 1100 1194 1251 1337 1381 1461 1845 2091 2146 2860 3002 3046
MIN040_7
12
E= -1837.75643887 ZPE= 42.80 Gcorr 0.008717000
H -1.374831 -1.884227 0.422058
Co 0.387930 -0.069095 -0.252728
C -0.894794 -1.436507 -0.445508
C 1.019349 1.559377 -0.058994
O -1.482754 -0.119352 -0.749717
O 1.395510 2.627434 0.159183
C -2.353550 0.471723 0.157281
O -2.911592 -0.106485 1.040469
C 1.755388 -0.953842 0.249343
O 2.643635 -1.600336 0.605244
H -2.450940 1.534043 -0.103695
H -0.965091 -2.038839 -1.348873
FREQS= 39 48 75 93 107 213 285 370 414 433 468 518 546 590 647 725 748 835 1000 1020 1182 1199 1391 1479 1857 2079 2141 3090 3125 3219
MIN041_7
12
E= -1837.74545744 ZPE= 36.80 Gcorr -0.001934000
H 1.881840 1.686910 -0.586505
Co -0.104963 -0.010364 -0.230207
C 1.705442 0.597285 -0.745278

C 0.592829 -1.072952 1.042688
O 2.583807 -0.089967 -1.187858
O 1.072416 -1.797592 1.786697
C -1.786559 -0.671564 -0.437882
O -2.790201 -1.127810 -0.741692
C -0.502097 1.560044 0.576640
O -0.749066 2.588646 1.011904
H -0.254372 0.594928 -1.571990
H 0.213195 -1.065110 -1.195334
FREQS= 48 70 80 92 103 132 258 326 362 379 422 444 472 493 516 537 579 644 652 702 869 909 1383 1815 2033 2088 2153 2160 2196 2830
MIN042_7
12
E= -1837.74934927 ZPE= 39.44 Gcorr 0.002483000
H 0.656526 -0.212854 1.591170
Co 0.154894 0.002165 0.187786
C -0.441073 0.491856 -1.462197
C 0.472840 -1.733088 -0.032281
O -1.705961 0.546867 -1.849285
O 0.686979 -2.856911 -0.155380
C 1.645421 0.974699 0.160494
O 2.627974 1.572883 0.223134
C -1.342194 0.321223 1.100265
O -2.269335 0.509370 1.754764
H -1.800419 0.849791 -2.768054
H 0.234546 0.798798 -2.276908
FREQS= 51 59 73 97 108 135 308 322 325 418 447 457 485 507 515 547 613 645 691 717 987 1264 1306 1496 1937 2094 2103 2157 3007 3718
MIN043_7
12
E= -1837.75481944 ZPE= 40.50 Gcorr 0.008466000
H -2.364768 1.258303 0.760047
Co 0.066361 -0.018593 -0.171587
C -1.824471 0.311036 0.620681
C 0.656525 1.645476 -0.115475
O -1.051572 -0.162307 1.521175
O 1.100386 2.699985 -0.188743
C 1.550656 -1.018651 0.150229
O 2.491505 -1.651374 0.278316
C -1.853015 -0.371282 -0.658182
O -1.132185 -1.440197 -0.741570
H -2.386166 0.042547 -1.520957
H 0.515947 0.232840 -1.543183
FREQS= 71 82 103 113 289 313 361 372 395 436 460 468 490 501 535 675 814 851 911 949 1025 1278 1346 1460 1523 2028 2141 2190 3052 3099
MIN044_7
12
E= -1837.75205228 ZPE= 41.76 Gcorr 0.006325000
H 1.021376 0.778227 0.421807
Co -0.449733 -0.012210 0.142025
C 1.608320 -0.258841 0.554561
C -1.828314 -1.012066 -0.178177
O 0.771919 -1.318919 0.373584
O -2.726347 -1.691534 -0.401196
C -1.303943 1.492987 -0.003770
O -1.841185 2.508656 -0.094242
C 2.719339 -0.148139 -0.499709
O 3.722426 0.500062 -0.333555
H 2.514813 -0.737568 -1.419186
H 2.019671 -0.140765 1.568558
FREQS= 33 41 71 100 122 164 286 357 366 451 470 494 552 575 608 673 748 1030 1069 1146 1274 1325 1387 1677 1842 2102 2126 2165 2936 3023
MIN045_7
12
E= -1837.74456060 ZPE= 38.25 Gcorr -0.000740000
H -0.431527 1.799963 1.853121
Co 0.233471 -0.141928 -0.093019
C -0.972674 0.893896 1.533041
C -0.350682 1.155378 -1.187401
O -2.162231 0.751663 1.626431
O -0.638757 1.913042 -2.001932
C 1.925976 0.034288 0.443407
O 3.052489 0.115680 0.661661
C -0.595763 -1.707722 -0.322856
O -1.069724 -2.723569 -0.573199
H 0.920746 -0.604929 -1.288043
H -0.288314 -0.072548 1.445613

FREQS=   39   60   72   87   98 158 190 295 312 347 393 425 450 459 506 509 518 690 730 835 1143
1424 1597 1826 2060 2103 2110 2135 2175 3005
MIN046_7
12
E= -1837.74838178 ZPE=   39.96 Gcorr   0.003606000
H -1.344420 -0.000088 -2.338540
Co 0.163629 -0.000007 -0.227691
C -1.345317 -0.000028 -1.239680
C -0.724208 0.000085 1.334043
O -2.601882 0.000014 -0.781974
O -1.295975 0.000154 2.328325
C 1.169648 -1.465794 -0.048525
O 1.809141 -2.422130 -0.050008
C 1.169746 1.465724 -0.048617
O 1.809310 2.422012 -0.050177
H 0.794453 -0.000034 -1.563180
H -3.251978 -0.000016 -1.503265
FREQS=   40   69   84   94 113 147 310 323 344 435 442 454 469 527 548 558 565 671 699 871 974
1247 1289 1495 2026 2100 2126 2165 3035 3728
MIN047_7
12
E= -1837.75061049 ZPE=   43.09 Gcorr   0.005917000
H 2.203467 -0.025228 2.016904
Co -0.640028 -0.104629 0.060558
C 2.228501 -0.072999 0.931528
C -2.037819 -1.098862 -0.089202
O 1.070255 -0.194620 0.267593
O -2.952363 -1.798577 -0.169629
C -1.537122 1.341224 -0.065469
O -2.101642 2.346046 -0.132304
C 3.390459 -0.027967 0.244943
O 3.471156 -0.088881 -1.101252
H 4.353702 0.059329 0.733522
H 2.560206 -0.169231 -1.431553
FREQS=   14   31   48   96 102 206 225 301 382 444 450 517 534 592 603 674 788 843 894 1090 1195
1266 1375 1426 1672 2078 2132 3182 3242 3736
MIN048_7
12
E= -1837.75209845 ZPE=   42.49 Gcorr   0.007753000
H 2.698204 -1.277699 1.002475
Co -0.412268 -0.100542 -0.181124
C 0.462866 -1.765945 -0.223231
C -2.009447 -0.542343 0.204274
O 1.412098 -0.672081 -0.490882
O -3.069044 -0.902238 0.492341
C 2.452580 -0.399350 0.384856
O 3.007000 0.655264 0.400161
C -0.587513 1.653902 -0.133992
O -0.679610 2.797421 -0.039781
H 0.441081 -2.416873 -1.095616
H 0.717494 -2.295292 0.697336
FREQS=   43   46   71 100 111 129 275 389 392 427 461 499 528 569 637 677 727 921 1010 1021 1174
1203 1393 1490 1877 2082 2146 3046 3091 3187
MIN049_7
12
E= -1837.74321666 ZPE=   36.67 Gcorr   -0.000966000
H 0.000000 -1.101249 2.280179
Co 0.000000 -0.324834 -0.277908
C 0.000000 -0.142596 1.706838
C 0.000000 1.451716 -0.650156
O 0.000000 0.906228 2.299512
O 0.000000 2.554044 -0.943462
C 1.736949 -0.662263 -0.314137
O 2.840181 -0.959396 -0.391068
C -1.736949 -0.662263 -0.314137
O -2.840181 -0.959396 -0.391068
H 0.000000 -1.796039 -0.159771
H 0.000000 -0.571589 -1.778649
FREQS=   64   65   92   94 103 177 294 348 400 415 436 457 487 499 539 551 589 643 648 723 767
878 1365 1793 1841 2047 2145 2171 2207 2818
MIN050_7
12
E= -1837.75640227 ZPE=   43.41 Gcorr   0.012397000
H 3.108609 0.738589 -0.912237
Co -0.202236 -0.009130 -0.108163
C 2.412497 0.688642 -0.061576
C -1.422777 1.324876 0.037712

O 1.183892 1.162129 -0.330654
O -2.166213 2.149191 0.321209
C -1.389233 -1.260801 -0.074803
O -2.141988 -2.136413 -0.087731
C 1.137710 -1.196692 0.277535
O 2.362342 -0.761873 0.271745
H 1.137407 -2.263063 0.531569
H 2.880903 1.130545 0.831299
FREQS= 48 74 91 111 183 320 368 384 438 456 484 510 600 609 629 765 820 883 1127 1187 1221
1290 1390 1482 1520 2093 2154 2998 3040 3090
MIN051_7
12
E= -1837.74772484 ZPE= 39.97 Gcorr 0.003726000
H 2.175522 -0.008289 1.321401
Co 0.002442 0.000000 -0.084341
C 1.777855 -0.005440 0.292692
C -0.482813 -0.002708 1.643008
O 2.783993 -0.006478 -0.581548
O -0.771522 -0.004655 2.755215
C -0.824680 1.467336 -0.682045
O -1.263174 2.429841 -1.133968
C -0.834164 -1.459215 -0.688641
O -1.279169 -2.416575 -1.145117
H 0.533379 0.000907 -1.454630
H 3.646944 -0.009528 -0.136296
FREQS= 45 67 87 88 108 155 315 348 348 429 449 458 470 517 539 552 571 664 713 836 961
1268 1302 1508 2059 2103 2125 2160 2982 3729
MIN052_7
12
E= -1837.75172580 ZPE= 42.55 Gcorr 0.008498000
H -1.652613 -0.431349 1.554186
Co 0.444950 -0.121446 0.078081
C -1.359733 -0.538507 0.509483
C 0.537935 1.588134 0.102079
O -0.570174 -1.737684 0.245513
O 0.558771 2.740376 0.147611
C 2.133255 -0.481433 -0.225040
O 3.243172 -0.742084 -0.394963
C -2.439348 -0.358778 -0.476805
O -3.333461 0.454249 -0.347291
H -2.357840 -1.007635 -1.377934
H -0.422323 -2.257312 1.050282
FREQS= 36 57 77 94 140 208 289 389 403 423 465 480 516 571 590 644 653 873 972 1028 1068
1182 1328 1424 1797 2084 2144 2898 3143 3782
MIN053_7
12
E= -1837.74158293 ZPE= 36.36 Gcorr -0.001559000
H 0.491092 -0.437198 2.395538
Co 0.148483 -0.200792 -0.242654
C -0.353593 -0.294903 1.678596
C 0.179592 1.604898 -0.204168
O -1.480462 -0.225519 2.097911
O 0.208845 2.748400 -0.213125
C 1.868785 -0.593200 -0.143000
O 2.966192 -0.921279 -0.140697
C -1.479768 -0.510892 -0.885407
O -2.490360 -0.783642 -1.342739
H 0.059973 -1.660555 -0.007968
H 0.516077 -0.259950 -1.722848
FREQS= 61 84 88 98 107 136 311 345 365 423 438 459 475 493 537 544 583 595 642 674 767
874 1369 1792 1825 2023 2149 2162 2205 2812
MIN054_7
12
E= -1837.75123530 ZPE= 42.49 Gcorr 0.008673000
H 1.732642 -0.461952 -1.562750
Co -0.453074 -0.119328 -0.204422
C 1.371038 -0.545304 -0.539581
C -0.533176 1.590694 -0.139367
O 0.552789 -1.740176 -0.400263
O -0.532220 2.741579 -0.066850
C -2.111059 -0.484301 0.231287
O -3.175209 -0.751623 0.584320
C 2.366870 -0.348259 0.522680
O 3.278770 0.450538 0.478862
H 2.204366 -0.988945 1.430596
H 0.740917 -2.206756 0.432882

```
FREQS=   38   58   81   95  144  218  288  392  400  422  466  482  515  572  604  650  655  890  968 1053 1089
1176 1327 1435 1804 2085 2145 2785 3168 3714
MIN055_7
12
E= -1837.74137445 ZPE=    36.62 Gcorr   -0.001153000
H 0.000000 -0.683629 2.265919
Co 0.000000 0.093766 -0.302449
C 0.000000 -1.165219 1.256413
C -1.732025 -0.169320 -0.555383
O 0.000000 -2.364367 1.174250
O -2.831507 -0.358530 -0.812355
C 0.000000 1.685765 0.550660
O 0.000000 2.732392 1.011884
C 1.732025 -0.169321 -0.555383
O 2.831507 -0.358530 -0.812355
H 0.000000 -1.074359 -1.199004
H 0.000000 0.927160 -1.570042
FREQS=   64   70   89   93  108  152  311  349  380  404  440  452  490  498  535  543  588  646  650  721  760
874 1365 1805 1857 2067 2147 2160 2200 2793
MIN056_7
12
E= -1837.74763459 ZPE=    40.72 Gcorr    0.005484000
H 0.948855 -0.938915 2.217889
Co 0.059097 -0.058639 -0.060601
C -0.009854 -1.020237 1.685685
C 0.350690 1.636912 0.265214
O -0.122119 -1.837843 0.685896
O 0.528086 2.728385 0.588881
C -1.745773 0.036468 -0.469042
O -2.840327 0.072611 -0.794906
C 1.805456 -0.446641 -0.443163
O 1.579650 -0.625844 -1.622418
H -0.894834 -0.782239 2.292296
H 2.784928 -0.633061 0.034266
FREQS=   57   76   88  109  126  153  171  263  373  396  419  429  475  523  530  569  630  703  885 1068 1209
1250 1346 1581 1744 2106 2169 2978 2991 3065
MIN057_7
12
E= -1837.73923787 ZPE=    36.61 Gcorr   -0.001693000
H 2.074034 0.000102 1.541154
Co -0.132163 -0.000005 0.077878
C 1.833384 0.000083 0.457292
C 0.108758 -1.752986 -0.038599
O 2.691702 0.000111 -0.375966
O 0.263367 -2.881318 -0.154122
C -1.942276 -0.000089 -0.043599
O -3.084963 -0.000140 -0.044766
C 0.108590 1.752998 -0.038600
O 0.263092 2.881346 -0.154123
H -0.361769 -0.000014 1.575097
H 0.139814 0.000016 -1.406091
FREQS=   35   77   93   99  103  183  267  306  387  393  434  472  510  519  532  548  591  607  673  692  791
909 1337 1822 1831 1991 2144 2159 2200 2903
MIN058_7
12
E= -1837.74888914 ZPE=    42.56 Gcorr    0.008281000
H -2.550788 -1.211893 -1.130190
Co 0.248931 -0.096516 -0.083155
C -2.104085 -1.127477 -0.133242
C 1.909295 -0.737536 0.037595
O -0.785069 -1.777631 -0.092773
O 2.952689 -1.111249 0.351324
C 0.683607 1.551763 -0.133921
O 0.944917 2.672667 -0.216553
C -1.610568 0.308334 0.148529
O -2.359038 1.209978 0.447695
H -0.701025 -2.540376 -0.678845
H -2.766795 -1.562425 0.622920
FREQS=   26   57   86  102  128  272  296  337  364  404  444  459  481  495  586  612  631  869  958  964 1138
1216 1334 1481 1811 2080 2137 3046 3119 3834
MIN059_7
12
E= -1837.74968843 ZPE=    42.69 Gcorr    0.009324000
H -2.914783 -1.357649 -0.482276
Co 0.244063 -0.082416 -0.211403
C -2.054510 -1.139055 0.159662
C 1.863375 -0.780959 0.062808
```

O -0.832549 -1.723200 -0.418188
O 2.823131 -1.210826 0.536632
C 0.720786 1.556307 -0.133433
O 1.015805 2.670874 -0.105684
C -1.582409 0.330856 0.163404
O -2.317149 1.251804 0.437691
H -0.643259 -2.615659 -0.101973
H -2.229012 -1.513560 1.173872
FREQS= 38 68 85 101 133 295 298 345 382 417 448 459 488 500 585 610 625 878 951 961 1138
1218 1334 1481 1808 2075 2133 3050 3122 3835
MIN060_7
12
E= -1837.74423513 ZPE= 39.77 Gcorr 0.004145000
H -1.728455 -0.049084 1.552742
Co 0.426152 -0.074748 0.063299
C -1.387512 -0.534336 0.632393
C 2.124341 -0.666137 -0.202584
O -0.460673 -1.492759 0.719062
O 3.197219 -0.972222 -0.450756
C -2.381841 -0.593258 -0.475144
O -3.361270 0.121817 -0.527943
C 0.478497 1.674079 0.103055
O 0.495002 2.821255 0.132798
H -2.145927 -1.341941 -1.263746
H 0.405140 0.302404 -1.329684
FREQS= 51 62 77 97 134 211 291 347 374 408 418 428 489 510 567 636 671 718 960 1021 1108
1243 1347 1432 1802 2100 2132 2183 2911 3093
MIN061_7
12
E= -1837.74367552 ZPE= 39.71 Gcorr 0.004084000
H 0.905689 0.410707 1.443245
Co 0.466111 -0.103873 0.169766
C -1.302730 -0.909077 0.446555
C 0.219440 1.624281 0.037828
O -0.330827 -1.702071 -0.010004
O 0.050457 2.756865 -0.038779
C 2.215949 -0.420775 -0.207838
O 3.341551 -0.549199 -0.358660
C -2.339347 -0.427317 -0.507397
O -3.379857 0.092140 -0.160945
H -1.626073 -1.019900 1.485946
H -2.075082 -0.570772 -1.580657
FREQS= 51 65 78 94 135 198 288 345 377 393 411 437 477 517 570 624 674 713 964 1018 1109
1246 1349 1433 1803 2103 2132 2183 2888 3103
MIN062_7
12
E= -1837.73893072 ZPE= 40.27 Gcorr 0.000959000
H -2.398876 -0.841990 1.138113
Co 0.063325 -0.020563 -0.010508
C -2.432731 0.237024 0.901595
C 0.580017 1.632132 -0.243886
O -1.629213 0.600577 -0.198207
O 0.858583 2.729534 -0.416410
C 1.691099 -0.624133 0.340890
O 2.728008 -0.980282 0.685876
C -0.432543 -1.634812 -1.308743
O -0.718617 -2.041012 -0.255431
H -3.476771 0.476063 0.658515
H -2.179288 0.789325 1.821335
FREQS= 38 41 91 95 104 134 135 185 198 239 280 356 446 451 494 509 596 631 1063 1168 1189
1482 1499 1513 1963 2115 2167 2946 2992 3050
MIN063_7
12
E= -1837.74120741 ZPE= 39.76 Gcorr 0.003980000
H 0.394385 0.223777 -1.344115
Co 0.344739 -0.025462 0.079055
C -1.466633 0.071199 0.818188
C 0.849320 1.632370 -0.071215
O -0.796790 -1.062883 1.002417
O 1.175063 2.731248 -0.156758
C 1.803684 -1.083595 -0.145514
O 2.746934 -1.693944 -0.353200
C -2.533795 0.141305 -0.224894
O -2.737368 -0.715589 -1.059056
H -1.583690 0.748221 1.672526
H -3.136823 1.077158 -0.189508

```
FREQS=   52   64   77  103  107  194  275  317  359  398  426  435  511  532  628  654  717  801  914  932 1032
1263 1414 1432 1804 2098 2126 2183 2892 3071
MIN064_7
12
E= -1837.73805108 ZPE=    40.06 Gcorr    0.001598000
H -3.481496 0.262201 -1.696660
Co 0.125030 -0.080789 0.023933
C -0.422763 -1.315615 1.169344
C 0.767458 1.600606 0.115572
O -1.245088 -0.497376 1.604795
O 1.258087 2.597987 0.421558
C 1.566586 -0.851147 -0.487454
O 2.544364 -1.384708 -0.803292
C -2.704517 0.227789 -0.917792
O -1.540261 0.018816 -1.221748
H -0.266666 -2.302458 1.631706
H -3.005049 0.374013 0.130254
FREQS=   28   71   83   94  130  148  159  189  214  292  329  376  420  475  490  531  592  707  826 1174 1235
1268 1518 1610 1754 2067 2119 2997 3029 3098
MIN065_7
12
E= -1837.73978907 ZPE=    40.74 Gcorr    0.004258000
H -3.455049 -1.026103 -0.951885
Co 0.163964 0.012255 -0.174272
C -2.608049 -0.688873 -0.342116
C 0.794753 -1.618813 0.233003
O -1.508071 -0.549239 -0.878577
O 1.169393 -2.567682 0.768152
C -0.609693 1.666888 0.340741
O -1.592646 1.543676 1.057454
C 1.701287 0.766448 -0.216501
O 2.723985 1.286072 -0.359294
H -0.224760 2.694348 0.148558
H -2.758298 -0.455643 0.716018
FREQS=   30   75   90  102  108  199  212  251  270  354  395  406  447  467  501  566  599  641  861 1117 1263
1367 1509 1696 1714 2072 2124 2853 3044 3166
MIN066_7
12
E= -1837.73518760 ZPE=    39.71 Gcorr   -0.000048000
H -3.611521 0.077041 -1.546304
Co 0.091628 0.001005 0.191446
C -2.526711 -0.101525 -1.471555
C -0.205232 -0.540337 1.854369
O -1.920390 0.179041 -0.453005
O -0.551964 0.630077 2.044349
C 0.982003 -1.333657 -0.411331
O 1.614259 -2.237151 -0.767070
C 1.041048 1.318612 -0.577976
O 1.803866 2.136351 -0.860713
H -0.152897 -1.290571 2.656834
H -2.022358 -0.538720 -2.349099
FREQS=   38   60   75   86  110  117  137  164  200  248  287  370  427  477  496  530  594  695  824 1172 1217
1262 1533 1624 1771 2062 2115 2975 3044 3067
MIN067_7
12
E= -1837.73680513 ZPE=    40.50 Gcorr    0.003766000
H -0.932862 -2.532394 -0.114608
Co 0.000021 0.024173 0.167795
C 0.000028 -2.125585 0.302100
C -1.636125 -0.044689 -0.537932
O 0.000695 -1.491950 1.394454
O -2.595746 -0.114536 -1.172460
C -0.000316 1.860111 -0.031364
O -0.000506 2.216756 1.127470
C 1.636147 -0.044154 -0.538009
O 2.595784 -0.113784 -1.172532
H 0.932391 -2.532440 -0.115746
H -0.000325 2.566180 -0.884329
FREQS=   47   49   97   97  119  132  195  219  296  341  426  443  486  496  508  529  623  657  890 1110 1229
1292 1449 1629 1753 2072 2126 2949 2997 3075
MIN068_7
12
E= -1837.73576263 ZPE=    40.36 Gcorr    0.003180000
H 2.408194 -0.714719 -0.086278
Co -0.308219 0.085789 0.227827
C 0.606929 -0.267932 -1.296092
C -1.656800 -1.125843 0.026992
```

O 1.761956 -0.589765 -1.525265
O -2.397820 -2.005232 -0.017431
C -0.632335 1.860589 0.019289
O -0.660510 3.004081 -0.107425
C 1.125586 -0.319908 1.182266
O 2.343656 -0.671937 0.928169
H 0.960749 -0.262015 2.279995
H -0.085576 -0.118189 -2.164137
FREQS= 14 53 75 85 183 232 253 317 328 404 408 434 456 482 517 574 602 803 870 1108 1222
1272 1417 1552 1731 2082 2131 2803 2879 2949
MIN069_7
12
E= -1837.74000248 ZPE= 41.97 Gcorr 0.007622000
H -1.204574 -2.944136 -0.000098
Co 0.270362 -0.128803 -0.000021
C -0.973927 -1.879590 -0.000063
C -1.634800 0.238487 0.000039
O -2.148185 -1.083193 -0.000011
O -2.391942 1.164757 0.000111
C 1.972380 -0.656219 0.000016
O 3.071856 -0.989019 0.000091
C 0.615451 1.556724 -0.000038
O 0.813683 2.688944 -0.000059
H -0.366571 -1.693227 -0.927900
H -0.366538 -1.693299 0.927767
FREQS= 21 68 91 104 201 250 293 300 317 390 422 454 475 524 571 618 654 924 1078 1147 1190
1423 1499 1564 1860 2108 2160 2719 2776 3161
MIN070_7
12
E= -1837.73769083 ZPE= 40.57 Gcorr 0.005416000
H 2.082224 -1.966477 0.268279
Co 0.037113 -0.095702 0.041439
C -0.882836 -1.561816 -1.011722
C 1.129199 1.322734 -0.226682
O -0.274500 -0.684510 -1.747681
O 1.786947 2.194114 -0.578684
C -1.565475 0.752452 0.415545
O -2.533226 1.226705 0.801241
C 1.305146 -1.252761 0.592892
O 0.947328 -1.100530 1.750345
H -1.979273 -1.544681 -0.944409
H -0.433588 -2.554757 -0.864691
FREQS= 61 78 83 111 121 162 202 283 360 393 400 426 460 487 488 563 633 755 881 1055 1203
1221 1344 1578 1700 2121 2161 2990 3002 3061
MIN071_7
12
E= -1837.73284788 ZPE= 39.89 Gcorr 0.001160000
H 3.744412 0.403362 -0.483569
Co -0.077354 0.103253 -0.100954
C 2.797689 0.286019 0.054669
C 0.403312 -1.647208 0.485182
O 1.739848 0.276742 -0.576974
O 0.246108 -2.772092 0.087866
C -1.718537 -0.397504 -0.088888
O -2.820708 -0.729750 -0.165534
C -0.430145 1.845435 0.035436
O -0.648509 2.925793 0.372620
H 1.075463 -1.482661 1.401619
H 2.820839 0.165446 1.145467
FREQS= 19 59 75 96 109 149 194 228 279 374 380 410 449 465 499 526 590 609 841 1092 1256
1319 1514 1690 1808 2079 2131 2488 3032 3145
MIN072_7
12
E= -1837.72871879 ZPE= 37.83 Gcorr 0.001141000
H 1.243004 -1.359725 1.272097
Co -0.038039 -0.017203 -0.411413
C 1.711432 -0.364638 -0.910987
C 0.302643 1.582692 0.323504
O 2.661756 -0.902584 -0.408913
O 0.546373 2.579211 0.834528
C -1.872019 -0.043212 -0.581024
O -2.993795 -0.167655 -0.759689
C 0.185748 -1.011253 1.159282
O -0.697780 -1.232101 1.929952
H -0.097381 -1.427394 -0.908934
H 1.782170 0.055104 -1.966692

FREQS= 68 79 84 97 132 184 278 304 344 366 392 399 443 493 519 539 641 663 737 866 943 1221 1267 1818 1850 1975 2139 2178 2619 2824
MIN073_7
12
E= -1837.72766793 ZPE= 38.11 Gcorr 0.000692000
H 0.175354 1.071094 2.220806
Co 0.114147 -0.271878 0.054368
C 1.775322 -0.593054 0.780075
C 0.612757 1.105366 -0.997574
O 2.306171 -1.363786 0.008769
O 0.918374 2.027010 -1.605146
C -1.487086 -0.829119 -0.681123
O -2.460434 -1.285104 -1.066309
C -0.557822 0.813598 1.418626
O -1.686301 1.219063 1.449997
H 2.256419 -0.262342 1.729626
H -0.195257 -1.226269 1.163122
FREQS= 61 76 80 96 109 152 229 243 334 367 393 400 461 484 509 618 653 697 748 892 908 1276 1312 1755 1819 1963 2143 2182 2830 2867
MIN074_7
12
E= -1837.73202203 ZPE= 40.09 Gcorr 0.005191000
H 1.939908 0.440438 -1.321554
Co -0.470449 -0.144760 -0.476238
C 1.496002 -0.278492 -0.623147
C -1.712488 -0.775929 0.492280
O 0.788629 -1.292891 -1.092088
O -2.443832 -1.152340 1.320085
C -0.743337 1.563929 -0.137077
O -0.900512 2.686075 0.041003
C 2.189739 -0.413871 0.692520
O 2.954408 0.421984 1.127772
H -1.744609 -1.064142 -0.789296
H 1.937804 -1.344225 1.247660
FREQS= 47 62 76 98 141 211 324 381 406 434 449 490 521 557 584 629 657 943 1000 1018 1098 1267 1351 1441 1800 1867 2048 2152 2916 3079
MIN075_7
12
E= -1837.73159957 ZPE= 40.02 Gcorr 0.005102000
H 2.502930 -0.340011 -1.276025
Co -0.465490 -0.199704 -0.258910
C 1.258564 -0.890547 0.451015
C -0.359062 1.554417 -0.107697
O 0.523814 -1.713952 -0.275808
O -0.260047 2.694744 -0.033790
C -2.110101 -0.363291 0.126202
O -3.181285 -0.430729 0.583449
C 2.451374 -0.240403 -0.167767
O 3.297376 0.347013 0.473037
H -1.719999 -0.785653 -1.058108
H 1.301774 -1.019985 1.539080
FREQS= 48 59 86 97 135 203 333 381 390 433 439 482 522 558 581 628 652 945 987 1018 1104 1268 1354 1442 1803 1866 2050 2152 2898 3077
MIN076_7
12
E= -1837.72657873 ZPE= 38.07 Gcorr 0.000551000
H 0.304026 0.599556 -1.537141
Co 0.145570 -0.090489 -0.220132
C -1.437236 -0.517049 -1.055887
C -0.344800 -0.660761 1.415853
O -1.280810 -1.657793 -1.429885
O -0.674055 -0.955265 2.473030
C 1.982594 -0.048765 -0.160981
O 3.121381 -0.023217 -0.267796
C -0.306871 1.707771 0.098180
O -1.396739 2.204029 0.045748
H -2.342972 0.094995 -1.244246
H 0.588215 2.319439 0.373195
FREQS= 64 74 79 95 108 169 198 231 346 366 392 414 461 484 515 586 657 689 746 882 912 1231 1310 1763 1825 1970 2135 2171 2814 2941
MIN077_7
12
E= -1837.72851164 ZPE= 37.91 Gcorr 0.002633000
H 2.492911 0.608814 -1.044355
Co -0.095044 -0.128661 -0.487076
C 1.887867 -0.019064 -0.381338
C -1.117475 -1.356394 0.383423

O 1.517656 -1.226070 -0.682807
O -1.705847 -2.071970 1.056683
C 1.195231 0.497091 0.782320
O 1.284485 1.089001 1.809738
C -1.035967 1.353171 -0.328017
O -1.679420 2.298395 -0.219421
H -0.602099 -0.695373 -2.055495
H -0.237552 -0.003266 -2.200958
FREQS= 60 77 97 104 222 274 317 355 366 405 426 442 473 485 522 560 589 610 651 783 842
945 1275 1352 1405 1935 2121 2167 3086 3572
MIN078_7
12
E= -1837.73186283 ZPE= 40.23 Gcorr 0.006114000
H 3.519030 0.742164 -0.359668
Co -0.319046 -0.021443 0.201309
C 0.998237 -1.202488 -0.073755
C -1.289185 1.472015 -0.185730
O 3.153998 -0.153860 -0.469447
O -1.868932 2.436472 -0.394291
C -1.477254 -1.282731 -0.069557
O -2.222405 -2.147040 -0.184757
C 1.906409 -0.125287 0.003212
O 1.431686 0.951114 0.506881
H 1.218026 -2.229201 -0.324536
H -0.906834 0.203462 1.536756
FREQS= 45 78 88 104 129 320 328 375 434 450 468 541 547 555 603 622 703 708 732 774 1004
1081 1173 1478 1526 1963 2136 2171 3276 3731
MIN079_7
12
E= -1837.72359924 ZPE= 38.19 Gcorr -0.001690000
H -0.000520 0.942748 -1.428066
Co 0.042431 0.068884 -0.218436
C 0.284528 -1.387691 -1.580951
C 0.397389 -0.966668 1.234340
O -0.932028 -1.302995 -1.161533
O 0.737330 -1.608656 2.119842
C 1.374240 1.164756 -0.082175
O 2.274379 1.884937 -0.106814
C -2.865110 1.348484 0.562870
O -1.801216 0.955206 0.437395
H 0.555440 -0.974293 -2.561196
H 0.925426 -2.209532 -1.228594
FREQS= 49 54 80 93 99 124 159 165 167 357 401 415 428 451 526 558 568 691 740 870 1089
1214 1372 1592 1954 2108 2158 2168 2994 3074
MIN080_7
12
E= -1837.72297100 ZPE= 37.76 Gcorr -0.002141000
H 0.488183 -0.232189 2.841917
Co -0.051523 -0.038249 -0.276051
C 0.529002 0.798600 2.463447
C 1.259079 -1.182973 -0.280771
O 0.342060 1.059078 1.281386
O 2.008035 -2.062228 -0.312648
C -1.692438 -0.623017 0.050807
O -2.672452 -1.216594 0.184363
C 0.794569 1.519163 -1.535778
O -0.286183 1.887552 -1.305511
H -0.313647 -0.757548 -1.526635
H 0.743629 1.609350 3.171137
FREQS= 57 68 81 91 111 129 153 186 230 299 326 351 360 443 475 477 522 544 738 765 1145
1261 1521 1732 1962 2036 2075 2127 3018 3126
MIN081_7
12
E= -1837.72921163 ZPE= 40.58 Gcorr 0.004443000
H 2.857114 -0.000259 1.245039
Co 0.355222 0.000024 0.355509
C -1.515756 -0.000030 0.186796
C 0.609872 -1.475356 -0.547379
O -1.901708 0.000068 1.346377
O 0.894990 -2.359528 -1.226327
C 0.609793 1.475446 -0.547373
O 0.894905 2.359626 -1.226312
C 1.776176 -0.000090 1.408020
O -2.339317 -0.000163 -0.869017
H 1.533987 -0.000052 2.482041
H -3.253573 -0.000161 -0.523973

FREQS=   48   58   86   86 113 135 255 326 327 358 463 486 491 507 523 589 638 674 711 762 981
1164 1349 1422 1736 2100 2144 3038 3157 3663
MIN082_7
12
E= -1837.72479827 ZPE=   38.10 Gcorr   0.000281000
H -1.159143 -0.862956 1.194047
Co -0.140222 -0.230763 0.036657
C -1.903148 -0.563573 0.268070
C -0.126995 1.296802 -0.875597
O -3.086839 -0.484547 0.388209
O -0.091023 2.256012 -1.501728
C 0.963890 0.540377 1.378648
O 1.828574 1.365354 1.274800
C 1.171036 -1.366379 -0.450183
O 1.972892 -2.066753 -0.876396
H -1.392989 -1.009531 -0.767549
H 0.720602 0.099197 2.379066
FREQS=   43   67   85   86 109 134 258 315 346 362 428 437 476 498 522 537 589 666 862 931 1301
1317 1425 1819 1896 1983 2069 2127 2169 2794
MIN083_7
12
E= -1837.72749727 ZPE=   37.92 Gcorr   0.003010000
H 0.645984 -1.554527 -1.797864
Co -0.156856 -0.027066 0.337160
C 0.804784 -1.438065 -0.719024
C 1.677261 -0.422102 -0.154384
O 0.030613 -1.974524 0.166632
O 2.792474 -0.005701 -0.120448
C -1.933562 -0.128140 -0.068704
O -3.014830 -0.257542 -0.422592
C 0.064397 1.671125 -0.038122
O 0.194174 2.782168 -0.302047
H 0.344073 -0.061119 1.970109
H -0.451673 -0.105678 2.033469
FREQS=   62   86   96 114 227 276 342 368 374 421 429 433 460 490 516 545 568 606 658 820 846
923 1289 1337 1425 1925 2121 2164 3073 3530
MIN084_7
12
E= -1837.72243610 ZPE=   38.02 Gcorr   -0.001779000
H 0.618613 -0.024826 2.730964
Co -0.027730 -0.120201 0.174555
C 0.925294 0.529883 1.835328
C -1.759346 -0.086246 0.187440
O 1.720817 -0.016143 0.983420
O -2.905541 -0.084056 0.302694
C 0.265697 1.354823 -0.865598
O 0.383343 2.328473 -1.453609
C 0.545254 -2.278805 -0.676462
O 0.780765 -1.516852 -1.507052
H -0.453653 -1.164507 1.141028
H 0.887279 1.625454 1.927144
FREQS=   59   73   73   86 107 125 127 145 163 365 390 418 419 446 524 552 561 671 721 875 1090
1214 1379 1594 1982 2072 2115 2170 2997 3081
MIN085_7
12
E= -1837.71757725 ZPE=   37.31 Gcorr   -0.006572000
H -0.048486 -0.001528 3.632975
Co -0.057338 0.000026 -0.237666
C -0.504382 -0.000698 2.635756
C -0.814727 -1.527424 -0.565711
O 0.215306 -0.000981 1.639368
O -1.499406 -2.404327 -0.890107
C -0.812004 1.529083 -0.564491
O -1.495116 2.407469 -0.888181
C 3.342071 -0.001424 -0.648053
O 2.269005 -0.001825 -0.261916
H 0.011376 0.000560 -1.710124
H -1.598811 0.000370 2.555831
FREQS=   37   51   69   71   80   97 112 132 140 220 297 344 386 464 470 493 553 564 672 755 1108
1259 1520 1704 2012 2045 2108 2173 3028 3136
MIN086_7
12
E= -1837.72966563 ZPE=   40.00 Gcorr   0.005755000
H -2.366980 -1.704338 -1.042400
Co 0.207778 -0.066199 -0.059995
C -1.786654 -1.500965 -0.130408
C 2.020167 -0.562340 0.002610

O -0.385285 -1.735362 -0.312246
O 3.054442 -0.929398 0.313059
C 0.436147 1.684954 -0.167961
O 0.511083 2.827687 -0.205942
C -1.703066 -0.018460 0.158999
O -2.542303 0.805684 0.384704
H 0.072371 0.213480 1.352117
H -2.218439 -2.089793 0.694116
FREQS= 52 77 88 96 163 271 312 348 375 422 440 458 500 539 587 591 690 732 943 998 1018
1160 1313 1481 1868 2092 2142 2185 2994 3045
MIN087_7
12
E= -1837.73312470 ZPE= 41.81 Gcorr 0.009656000
H -2.303390 0.709588 1.326487
Co 0.068998 0.013537 -0.071652
C -1.742916 -0.079780 0.814285
C 1.695929 -0.739663 -0.096953
O -0.807266 -0.751019 1.424985
O 2.709561 -1.256923 0.026945
C 0.601362 1.650927 0.453010
O 0.577071 2.241951 -0.609288
C -1.955387 -0.312550 -0.590643
O -1.141357 -1.059941 -1.238489
H -2.709237 0.285075 -1.122468
H 0.851670 2.133699 1.419178
FREQS= 58 81 91 123 197 219 278 299 396 412 436 465 506 554 652 844 892 907 963 1038 1257
1273 1342 1452 1526 1737 2156 2943 3053 3096
MIN088_7
12
E= -1837.72204489 ZPE= 37.91 Gcorr -0.001245000
H 2.884819 0.208547 -0.527012
Co -0.182278 0.001461 0.198819
C 2.292014 -0.179131 -1.367376
C -0.512389 1.741650 0.004252
O 1.080408 -0.331081 -1.286612
O -0.966543 2.797138 -0.082112
C -1.456571 -1.089206 -0.299794
O -2.402260 -1.687844 -0.576345
C 0.862803 -1.230300 1.535832
O 1.446885 -0.210121 1.563319
H -1.156488 0.185713 1.276036
H 2.810126 -0.436526 -2.300623
FREQS= 54 68 86 94 120 143 166 225 239 327 345 353 367 430 466 477 506 533 753 772 1163
1268 1523 1743 1886 2045 2086 2137 3017 3123
MIN089_7
12
E= -1837.73405394 ZPE= 43.11 Gcorr 0.010876000
H -2.276597 -1.934949 0.732197
Co 0.240841 -0.075273 -0.254937
C -1.812414 -1.405598 -0.119221
C 1.911886 -0.648582 0.171391
O -0.450137 -1.766176 -0.356658
O 2.884096 -1.015709 0.653914
C 0.570681 1.626240 -0.232017
O 0.668288 2.778673 -0.197596
C -1.523726 0.024040 0.146111
O -2.424145 0.786420 0.712743
H -2.464079 -1.554171 -0.998964
H -2.065420 1.679224 0.853268
FREQS= 45 69 87 92 142 297 312 403 419 456 476 505 551 597 636 671 698 967 1015 1043 1153
1323 1360 1469 1495 2076 2150 2950 2980 3725
MIN090_7
12
E= -1837.72603617 ZPE= 40.47 Gcorr 0.004023000
H 0.228299 -2.518120 -1.037387
Co -0.291663 -0.048550 -0.377323
C 0.031199 -1.480018 -1.312452
C -1.914249 -0.345088 0.246227
O 2.501671 0.081608 -0.141837
O -2.870925 -0.680876 0.787237
C 1.397225 -0.476953 0.419687
O 1.447686 -1.137091 1.429914
C -0.149468 1.703149 0.007968
O -0.063824 2.847417 0.051562
H 0.090289 -1.312980 -2.398220
H 3.251210 -0.153061 0.439742

FREQS= 35 66 86 92 126 136 233 306 333 385 418 455 478 514 531 578 651 668 696 790 988
1099 1303 1412 1793 2107 2148 3043 3171 3663
MIN091_7
12
E= -1837.72624773 ZPE= 41.16 Gcorr 0.004312000
H 1.845345 1.655586 -0.857956
Co 0.014228 0.007694 -0.070554
C 1.659871 0.584383 -0.791138
C 0.698849 -1.299931 0.887054
O 1.010178 0.160344 -2.002926
O 1.200384 -1.966951 1.692219
C -0.373786 1.556212 0.662559
O -0.487646 2.511011 1.308285
C -2.865851 -1.068078 -0.664156
O -1.829661 -0.680585 -0.368435
H 2.581217 0.031947 -0.597963
H 1.328736 -0.721334 -2.238176
FREQS= 58 69 73 93 99 135 204 237 251 295 313 430 477 504 527 529 557 628 852 982 1068
1237 1324 1516 2039 2092 2124 3084 3169 3825
MIN092_7
12
E= -1837.72775411 ZPE= 41.38 Gcorr 0.006104000
H -1.188858 -0.000690 -2.246977
Co 0.182489 0.000064 -0.135093
C -1.326668 -0.000479 -1.153307
C -0.002887 1.491610 0.758555
O -2.627114 -0.000761 -0.932261
O -0.234835 2.378867 1.462863
C -0.001997 -1.491403 0.758885
O -0.233372 -2.378727 1.463290
C 2.024815 0.000622 -0.210770
O 2.091052 0.000137 -1.428996
H 2.927506 0.001276 0.429985
H -2.791274 -0.000556 0.025154
FREQS= 63 65 86 107 109 135 204 233 342 393 429 483 493 523 548 597 649 700 876 993 1209
1269 1358 1472 1713 2061 2110 2953 3030 3745
MIN093_7
12
E= -1837.72320983 ZPE= 40.00 Gcorr 0.001564000
H 1.354199 2.182965 1.044416
Co 0.184733 0.206013 -0.137049
C 1.040274 1.912013 0.027257
C 1.345814 -1.046538 -0.484054
O -0.261604 1.941900 -0.255684
O 2.196568 -1.761107 -0.795751
C 0.034706 -0.343668 1.595078
O -0.961302 -0.888672 1.992785
C -2.850433 -0.604534 -1.140544
O -1.770435 -0.468245 -0.797632
H 0.864620 -0.045575 2.278430
H 1.745395 2.205611 -0.758714
FREQS= 47 58 75 95 117 145 160 169 184 194 319 440 488 512 570 616 667 781 890 1101 1195
1263 1295 1559 1810 2105 2169 2853 3008 3093
MIN094_7
12
E= -1837.72084598 ZPE= 37.21 Gcorr -0.000774000
H -0.000080 0.771000 2.087213
Co 0.000004 -0.201719 -0.370749
C -0.000001 -0.192111 1.522013
C 1.761967 -0.415859 -0.561416
O 0.000073 -1.281069 2.019505
O 2.883331 -0.589879 -0.711655
C -1.761943 -0.415935 -0.561414
O -2.883301 -0.589995 -0.711654
C -0.000028 1.664654 -0.249033
O -0.000112 2.472929 0.635108
H 0.000037 -1.257349 -1.495964
H 0.000048 1.992371 -1.332356
FREQS= 54 58 83 99 153 169 236 253 350 351 404 427 454 469 509 534 560 646 651 850 930
1209 1252 1804 1821 1847 2133 2184 2687 2851
MIN095_7
12
E= -1837.71128544 ZPE= 35.64 Gcorr -0.010191000
H 0.326118 0.453595 1.976976
Co -0.015780 0.014348 0.414562
C 3.455232 -0.067168 0.108949
C -0.092790 1.536282 -0.497599

O 2.322837 -0.042919 0.245970
O -0.159549 2.475921 -1.159987
C -0.146240 -1.516490 -0.483030
O -0.229455 -2.461454 -1.134869
C -1.824452 0.055391 0.757596
O -2.752546 -0.062267 0.007176
H 0.293944 -0.382640 1.994566
H -1.994787 0.219328 1.853481
FREQS= 16 46 53 64 86 103 113 133 165 260 344 378 415 435 479 503 514 555 633 858 879
964 1320 1569 1828 2092 2140 2174 2789 3022
MIN096_7
12
E= -1837.72814547 ZPE= 41.57 Gcorr 0.006935000
H -1.218816 -1.213039 1.384163
Co 0.478870 -0.119691 -0.285711
C -1.185820 -0.926251 0.325412
C 1.842561 -0.405449 0.819993
O -0.446497 -1.634825 -0.519749
O 2.705408 -0.868829 0.111564
C 0.543034 1.620337 -0.134081
O 0.464514 2.766161 -0.065051
C -2.399734 -0.220641 -0.174454
O -3.215437 0.301145 0.557610
H -2.489561 -0.191074 -1.284378
H 1.914741 -0.281413 1.918196
FREQS= 46 65 86 109 131 160 195 236 361 406 479 485 532 583 650 690 882 936 1020 1108 1251
1263 1352 1440 1770 1798 2125 2897 2957 3065
MIN097_7
12
E= -1837.72828301 ZPE= 41.66 Gcorr 0.007282000
H 1.693244 0.769790 -1.440210
Co -0.507962 -0.152932 -0.400224
C 1.395586 -0.024427 -0.745327
C -1.052054 -0.902279 1.115174
O 0.779523 -1.107110 -1.210275
O -1.790732 -1.762201 0.690323
C -1.197708 1.409976 -0.043311
O -1.566024 2.490523 0.101514
C 2.226225 -0.140164 0.487447
O 2.879848 0.775403 0.944334
H -0.808413 -0.731895 2.180163
H 2.176919 -1.140301 0.975029
FREQS= 50 62 80 118 132 156 213 250 356 431 472 489 536 577 654 689 877 941 1025 1102 1246
1258 1351 1438 1758 1798 2125 2899 2979 3075
MIN098_7
12
E= -1837.71873039 ZPE= 37.29 Gcorr -0.002159000
H 3.101112 -1.427575 -0.774680
Co -0.487646 -0.160787 0.413642
C 2.547710 -0.531085 -0.430408
C -2.143398 -0.296090 -0.301324
O 3.037835 0.569990 -0.385304
O -3.114924 -0.408154 -0.904318
C -0.229523 1.529186 0.131258
O -0.076954 2.651752 -0.076831
C 1.111313 -0.787188 -0.013123
O 0.595530 -1.910996 0.007086
H -0.245223 -0.279260 2.126959
H -0.937965 -0.661599 2.035900
FREQS= 30 52 76 90 112 169 251 277 311 366 416 432 453 480 503 529 540 579 634 790 976
1155 1253 1372 1649 1816 2101 2146 2960 3567
MIN099_7
12
E= -1837.72609578 ZPE= 41.43 Gcorr 0.006164000
H -1.185346 -0.002836 -2.317385
Co 0.046489 0.000013 0.068606
C -1.261845 -0.001564 -1.222036
C 0.889063 1.481064 -0.335486
O -2.551753 -0.001836 -0.925090
O 1.541240 2.356335 -0.714882
C 0.891135 -1.480049 -0.334473
O 1.544507 -2.354590 -0.713515
C 0.279080 0.000511 1.879152
O -0.933943 0.000474 2.076536
H -2.658444 -0.000911 0.043843
H 1.003588 0.000567 2.713829

FREQS= 67 79 84 85 99 140 198 269 334 342 419 457 510 545 546 648 657 723 875 1031 1229 1258 1320 1483 1660 2067 2114 2977 3070 3695
MIN100_7
12
E= -1837.72203776 ZPE= 39.45 Gcorr 0.002117000
H -2.185478 0.442735 1.352038
Co 0.784386 -0.249422 0.018332
C -2.112875 -0.015368 0.358254
C 2.384530 -0.843847 0.010810
O -1.022693 -0.266268 -0.179087
O 3.436747 -1.315597 -0.020559
C 1.166342 1.461417 -0.174756
O 1.443116 2.566031 0.007320
C -3.378692 -0.342163 -0.355251
O -4.461413 -0.106411 0.141326
H 0.732441 -1.483425 0.878797
H -3.247270 -0.807198 -1.352155
FREQS= 39 49 95 108 128 138 259 270 288 378 406 451 492 555 577 590 681 772 855 1026 1097 1337 1388 1640 1786 1909 2087 2131 2972 3095
MIN101_7
12
E= -1837.72056517 ZPE= 39.74 Gcorr 0.000895000
H 1.295606 2.150103 -0.731952
Co -0.036218 0.019963 -0.214375
C 0.593116 1.501525 -1.265381
C 1.011791 -1.362467 -0.371452
O -0.692456 1.536396 -0.920327
O 1.745040 -2.219374 -0.614547
C 0.605359 0.377994 1.452318
O 1.581870 0.990492 1.779982
C -3.174390 -0.753081 0.413056
O -2.038120 -0.632383 0.411253
H -0.140594 0.016320 2.214567
H 0.836932 1.309696 -2.316608
FREQS= 47 61 76 97 111 129 146 165 184 192 361 436 481 510 555 571 650 759 860 1089 1199 1267 1297 1561 1840 2107 2155 2741 3030 3121
MIN102_7
12
E= -1837.72296252 ZPE= 39.63 Gcorr 0.003304000
H 0.258698 0.543273 2.669752
Co 0.064508 0.005803 0.256935
C 0.869174 0.220206 1.821907
C -0.245372 1.512349 -0.760707
O -1.617997 0.307627 1.101605
O -0.416530 2.542561 -1.222931
C 1.640463 -0.530230 -0.381952
O 2.666622 -0.895626 -0.737881
C -1.047795 -1.235828 -0.574865
O -1.828614 -1.999291 -0.915444
H -1.653037 -0.268811 1.876247
H 1.925958 0.127714 2.087693
FREQS= 65 73 84 100 134 146 282 315 352 362 396 409 435 449 490 511 522 538 590 746 821 925 991 1450 2136 2147 2186 3072 3168 3827
MIN103_7
12
E= -1837.72221545 ZPE= 40.00 Gcorr 0.002898000
H -1.843421 1.728219 -0.029940
Co 0.148486 0.120509 -0.078016
C -1.552457 0.782975 -0.502745
C -0.465532 -1.357996 0.569705
O -2.638966 0.024116 -0.908466
O -0.896742 -2.319714 1.035584
C 1.819187 -0.456609 -0.485218
O 2.798955 -0.903155 -0.884937
C 0.603423 1.397548 1.409624
O 0.702027 2.143189 0.506240
H -0.906764 0.999226 -1.419875
H -3.408865 0.267817 -0.379310
FREQS= 53 83 89 105 128 160 170 195 242 313 368 372 413 436 482 541 544 607 725 1128 1156 1250 1344 1450 1889 2096 2145 2597 3066 3831
MIN104_7
12
E= -1837.72134904 ZPE= 39.99 Gcorr 0.002199000
H 3.292985 0.399045 0.140936
Co -0.123942 -0.036923 -0.135345
C 1.589884 0.508770 -0.761703
C -1.835435 -0.611399 -0.283250

O 2.733630 -0.194278 -0.376560
O -2.924260 -0.881805 -0.529369
C -0.464594 1.587331 0.307638
O -0.666544 2.674319 0.640456
C 0.519629 -1.183088 1.410930
O 0.633743 -1.947904 0.531993
H 1.804113 1.561054 -0.967145
H 1.179877 0.024481 -1.693324
FREQS= 47 80 86 91 124 135 177 203 217 293 353 373 415 445 466 542 545 578 727 1095 1119
1241 1359 1457 1920 2086 2141 2740 3089 3829
MIN105_7
12
E= -1837.72353706 ZPE= 41.12 Gcorr 0.004614000
H 0.750930 2.417357 -0.405282
Co 0.005596 -0.013250 0.137815
C -0.136805 1.867236 -0.099113
C -1.558182 -0.280749 -0.649894
O -0.161154 1.787657 1.331839
O -2.463939 -0.409239 -1.357521
C 1.622640 -0.026948 -0.596232
O 2.571725 0.001163 -1.253177
C 0.174510 -2.220214 0.406282
O 0.129027 -1.660028 1.427697
H -1.045303 2.304417 -0.515280
H -1.074986 1.843611 1.642606
FREQS= 51 56 96 100 122 128 181 225 282 298 347 447 481 499 517 528 549 618 867 995 1072
1233 1326 1519 1960 2056 2113 3093 3181 3823
MIN106_7
12
E= -1837.72448616 ZPE= 41.55 Gcorr 0.005701000
H 2.815231 -0.734972 -1.059696
Co -0.594415 -0.271873 -0.097397
C 1.231857 -0.627168 -0.053360
C -0.674095 1.412503 -0.286440
O 1.895377 -1.056882 -1.165521
O -0.694964 2.566819 -0.333290
C -2.309427 -0.593794 0.193711
O -3.408187 -0.812882 0.458367
C 2.141441 0.010390 0.936480
O 3.277046 0.317450 0.610165
H 1.731400 0.237609 1.936379
H 0.609750 -1.489689 0.452923
FREQS= 28 50 62 96 137 175 298 304 336 377 427 487 554 573 578 637 820 886 994 1083 1220
1303 1369 1418 1758 2079 2139 2286 3001 3589
MIN107_7
12
E= -1837.72707586 ZPE= 42.87 Gcorr 0.008700000
H 2.785555 1.286893 0.012040
Co -0.493600 -0.129206 -0.058955
C 1.252437 -0.600109 0.076150
C -0.501513 1.573003 0.005789
O 0.489361 -1.837120 -0.013757
O -0.440404 2.724697 0.080301
C -2.237167 -0.404632 -0.055599
O -3.364842 -0.622615 0.042846
C 2.583415 -0.604730 -0.012971
O 3.361542 0.507918 -0.021247
H 0.418221 -2.248119 0.863321
H 3.175129 -1.514453 -0.068922
FREQS= 35 60 71 93 164 185 282 372 395 440 457 490 494 518 559 609 657 674 888 914 1126
1174 1307 1377 1723 2076 2135 3198 3737 3780
MIN108_7
12
E= -1837.71606372 ZPE= 37.64 Gcorr -0.002280000
H 3.437206 -0.800021 0.680400
Co -0.458401 -0.203780 -0.181287
C 2.695798 -0.327471 0.003282
C -0.391513 1.476023 0.319287
O 3.004699 0.349720 -0.945168
O -0.356250 2.609293 0.524996
C -2.255878 -0.435895 -0.238179
O -3.383603 -0.635565 -0.180801
C 1.233407 -0.616196 0.351333
O 0.937802 -1.442446 1.203047
H -0.250248 -1.077180 -1.511795
H -0.122207 -0.247520 -1.704781

FREQS=   19   52   75   89 104 140 229 247 322 397 433 471 477 515 540 555 569 616 678 972 1071
1134 1362 1715 1726 1823 2102 2154 2793 2947
MIN109_7
12
E= -1837.71700763 ZPE=   37.57 Gcorr   -0.000895000
H 0.141274 -0.252162 -1.722511
Co 0.109820 0.061818 -0.225580
C 0.278852 1.238481 1.222338
C -1.636504 0.321814 -0.626336
O 0.468408 2.281683 0.628198
O -2.716654 0.487782 -0.956554
C 1.817137 -0.370944 -0.479129
O 2.896863 -0.665862 -0.723331
C -0.251288 -1.433897 0.840666
O -1.300414 -2.022559 0.833552
H 0.268835 1.194952 2.333171
H 0.589938 -1.792948 1.479842
FREQS=   45   64   90   97 101 143 188 241 316 400 410 442 469 509 528 552 618 632 665 856 877
1281 1323 1732 1804 1837 2139 2191 2838 2893
MIN110_7
12
E= -1837.71464740 ZPE=   36.41 Gcorr   -0.002814000
H -2.246691 0.154760 0.000000
Co -0.001417 -0.518651 0.000000
C -1.318237 0.810945 0.000000
C 1.321555 0.805328 0.000000
O -1.389599 1.997113 0.000000
O 1.396966 1.991281 0.000000
C -0.001350 -0.794480 1.764016
O -0.001350 -1.019238 2.886806
C -0.001350 -0.794480 -1.764016
O -0.001350 -1.019238 -2.886806
H 2.247727 0.146180 0.000000
H -0.003842 -2.060586 0.000000
FREQS=   33   69   83   87 162 189 202 231 360 360 404 414 432 457 495 512 540 576 642 830 857
1182 1212 1799 1858 1917 2131 2179 2621 2638
MIN111_7
12
E= -1837.72010768 ZPE=   40.00 Gcorr   0.002998000
H 1.546469 0.000018 2.436280
Co 0.382296 -0.000077 0.339060
C -1.519966 0.000278 -0.020978
C 0.583642 -1.515318 -0.514114
O -2.287861 0.000514 -0.946380
O 0.831870 -2.423830 -1.173135
C 0.584257 1.514906 -0.514260
O 0.832836 2.423227 -1.173424
C 1.812540 0.000021 1.365139
O -1.951890 0.000322 1.294444
H -2.927555 0.000664 1.276302
H 2.896621 0.000200 1.226031
FREQS=   44   58   88   90   97 127 241 316 331 347 467 474 479 483 518 564 603 651 665 767 978
994 1240 1417 1829 2106 2151 3016 3157 3681
MIN112_7
12
E= -1837.72590571 ZPE=   41.85 Gcorr   0.008816000
H 0.930978 1.795023 1.633987
Co 0.334671 0.228209 -0.314818
C 1.213606 1.683691 0.581262
C 1.213145 -1.273775 -0.193208
O 0.328300 2.017862 -0.355323
O 1.910991 -2.184678 -0.091700
C -0.981083 -0.322288 0.800891
O -1.152418 -0.449555 1.983471
C -2.091051 -0.427365 -0.263028
O -1.740580 -0.276639 -1.422204
H -3.141414 -0.614994 0.032005
H 2.276263 1.840831 0.364643
FREQS=   62   94 101 113 133 147 191 336 398 430 465 492 518 582 600 656 754 919 1006 1097 1197
1265 1329 1561 1740 1845 2118 2979 3028 3116
MIN113_7
12
E= -1837.71962475 ZPE=   39.97 Gcorr   0.002842000
H -1.426237 1.954719 1.268917
Co -0.134557 0.219027 -0.146530
C -1.241290 1.761915 0.202982
C 0.398089 -0.276825 1.533327

O -0.039193 2.020266 -0.285336
O 1.463318 -0.785573 1.752286
C -1.196173 -1.166898 -0.249252
O -2.007604 -1.976470 -0.362956
C 1.562197 -1.036511 -1.320331
O 1.874959 0.055119 -1.130933
H -0.297898 0.028821 2.350213
H -2.111605 1.905898 -0.447659
FREQS= 65 84 90 108 122 133 148 179 186 209 326 453 484 491 554 605 647 776 892 1101 1200
1287 1295 1565 1820 2076 2116 2851 3005 3093
MIN114_7
12
E= -1837.71913043 ZPE= 38.02 Gcorr 0.002364000
H 0.817163 -2.541140 0.300582
Co -0.151751 -0.023617 -0.297981
C 0.901796 -1.492284 0.613845
C 0.007969 1.712229 0.192650
O 0.095372 -0.954085 1.456902
O 0.144971 2.807947 0.498459
C 1.686172 -0.511527 -0.141566
O 2.763476 -0.313751 -0.610203
C -1.957468 -0.367968 -0.293395
O -3.069734 -0.619101 -0.201515
H 0.019956 0.309793 -1.839798
H -0.043332 -0.541783 -1.793652
FREQS= 55 75 81 98 225 259 304 341 344 411 413 445 463 476 555 564 619 645 681 849 917
1102 1294 1439 1789 1922 2136 2173 2861 3059
MIN115_7
12
E= -1837.72182703 ZPE= 41.05 Gcorr 0.005595000
H -1.233174 -0.000032 -2.195317
Co 0.184139 0.000006 -0.135189
C -1.353929 0.000025 -1.095236
C 0.018755 -1.485804 0.774115
O -2.627085 -0.000015 -0.738220
O -0.195787 -2.371671 1.483683
C 0.018815 1.485853 0.774087
O -0.195762 2.371704 1.483663
C 2.021497 -0.000089 -0.270372
O 2.058569 0.000015 -1.491059
H 2.942505 -0.000207 0.344912
H -3.231380 -0.000096 -1.499581
FREQS= 60 67 87 107 108 136 222 240 345 394 427 481 491 519 543 599 612 650 876 936 1241
1273 1308 1480 1707 2069 2117 2945 2963 3710
MIN116_7
12
E= -1837.71826048 ZPE= 38.59 Gcorr 0.002211000
H 1.977269 0.420766 -1.749967
Co -0.106330 -0.043578 -0.145813
C 1.752191 0.085144 -0.714125
C 0.004711 1.714949 0.275956
O 2.644150 -0.177279 0.053405
O 0.075751 2.842858 0.460322
C -1.921334 -0.221203 -0.028972
O -3.052908 -0.278872 -0.194671
C 0.213220 -1.850718 -0.100213
O 0.424544 -1.764805 1.095733
H 0.283739 -2.786501 -0.677644
H -0.415128 0.198102 -1.549617
FREQS= 53 83 88 106 140 191 230 239 301 404 414 426 464 500 514 558 613 699 823 881 908
1235 1358 1709 1798 2058 2134 2169 2878 3019
MIN117_7
12
E= -1837.71813328 ZPE= 38.42 Gcorr 0.002085000
H -0.424609 -2.771802 -0.660859
Co -0.103174 -0.058614 -0.005840
C -0.299906 -1.881527 -0.023154
C 0.394725 1.623677 0.438915
O -0.254404 -1.917154 1.197200
O 0.727747 2.705524 0.610552
C 1.790087 -0.469644 -0.133704
O 2.594456 -0.310458 -1.007382
C -1.882095 0.261653 -0.252257
O -2.928583 0.505196 -0.648579
H -0.014587 0.212230 -1.426463
H 2.094299 -0.927663 0.851873

FREQS= 53 84 87 104 141 193 229 302 355 380 405 435 461 475 514 568 591 697 771 887 909
1246 1330 1698 1834 2097 2134 2171 2709 3016
MIN118_7
12
E= -1837.72151181 ZPE= 41.27 Gcorr 0.005532000
H -0.273829 -1.887475 -1.620266
Co 0.153429 -0.062828 0.124966
C -0.757246 -1.130747 -0.985499
C 1.159030 1.196562 -0.652177
O -2.031997 -1.112794 -1.372994
O 1.669624 1.937985 -1.371984
C -1.224928 0.853084 0.853049
O -2.080045 1.383625 1.413191
C 1.398189 -1.357653 0.372293
O 1.612926 -1.093348 1.544440
H 1.850539 -2.225886 -0.148082
H -2.533636 -0.481497 -0.832973
FREQS= 48 67 76 107 113 153 193 265 336 359 403 434 492 500 536 641 652 711 869 997 1235
1250 1303 1489 1707 2078 2123 2937 3041 3753
MIN119_7
12
E= -1837.72170425 ZPE= 40.08 Gcorr 0.006064000
H -0.070806 -2.475191 0.821824
Co 0.239913 -0.025482 -0.141814
C -0.344266 -1.855241 -0.032686
C -1.656894 -0.175495 0.075655
O -1.771428 -1.521611 0.041647
O -2.572653 0.601408 0.110243
C 2.010549 -0.433810 -0.066202
O 3.060642 -0.796540 0.212597
C 0.299059 1.789716 -0.103370
O 0.272362 2.918912 0.068235
H -0.163150 -2.405666 -0.967465
H -0.005769 0.000507 1.272461
FREQS= 56 80 86 90 163 282 329 372 416 424 444 478 508 547 566 610 687 706 750 978 1073
1176 1293 1488 1837 2123 2135 2182 3018 3140
MIN120_7
12
E= -1837.71992738 ZPE= 41.01 Gcorr 0.004508000
H -0.003971 -1.919860 -1.520844
Co 0.069445 0.011687 0.146959
C -0.618931 -1.274604 -0.879538
C 1.779344 -0.607872 0.040171
O -1.881054 -1.568123 -1.191701
O 2.857280 -1.001673 0.087424
C 0.109465 1.623028 -0.640724
O 0.056002 2.505585 -1.380490
C -1.333375 0.175417 1.285580
O -0.604295 0.448935 2.230148
H -2.429406 0.052550 1.420805
H -2.484105 -1.021826 -0.663829
FREQS= 38 69 78 101 106 152 165 263 331 363 394 437 483 494 502 624 648 690 853 978 1221
1269 1310 1488 1689 2085 2134 2898 3061 3760
MIN121_7
12
E= -1837.71734543 ZPE= 38.47 Gcorr 0.002331000
H -1.537093 -2.337558 -0.156006
Co 0.073011 -0.020580 -0.076537
C -0.934378 -1.518610 0.256402
C 0.714839 1.637661 0.294591
O -0.839599 -1.216409 1.430727
O 1.087913 2.698782 0.508180
C 1.684067 -0.896859 -0.206809
O 2.652372 -1.398110 -0.557778
C -1.609615 0.702851 -0.672191
O -2.663961 0.116766 -0.518792
H 0.162515 0.037276 -1.536092
H -1.619983 1.697448 -1.172060
FREQS= 63 77 87 99 172 181 245 266 318 399 414 430 455 496 515 561 619 689 725 868 905
1200 1383 1715 1742 2043 2130 2167 2862 3081
MIN122_7
12
E= -1837.72492864 ZPE= 41.94 Gcorr 0.009963000
H 2.677591 -1.414873 -0.399112
Co -0.133454 0.103580 -0.631235
C 2.108015 -0.528989 -0.083950
C -1.774571 -0.440715 -0.269199

O 1.556725 0.194671 -1.198144
O -2.824523 -0.858212 -0.065874
C 0.828350 -0.904996 0.615880
O 0.505311 -1.580728 1.541965
C -0.319418 1.653639 0.319577
O -0.005852 1.920889 1.443538
H 2.746072 0.095898 0.557566
H -0.727925 2.435731 -0.380834
FREQS= 72 81 93 106 172 204 292 357 400 459 500 520 531 551 615 683 866 948 973 990 1171 1288 1310 1476 1838 1907 2138 2736 3005 3056
MIN123_7
12
E= -1837.71599499 ZPE= 37.93 Gcorr 0.001556000
H -0.141336 -1.465076 2.402152
Co -0.119867 -0.126302 -0.108338
C -0.242185 -1.261381 1.324457
C 0.514113 1.366878 0.543914
O -0.469167 -2.130376 0.492771
O 0.931696 2.349062 0.962114
C 1.617849 -0.640847 -0.725913
O 2.652231 -0.055040 -0.874321
C -1.883439 0.363650 -0.410884
O -2.894630 0.777683 -0.749269
H 1.531093 -1.743036 -0.918504
H 0.047594 0.117842 -1.598332
FREQS= 56 81 101 111 147 214 223 288 346 355 390 404 472 506 522 569 625 643 682 843 915 1217 1304 1675 1829 1900 2135 2171 2780 3027
MIN124_7
12
E= -1837.71150729 ZPE= 36.31 Gcorr -0.002715000
H 0.545080 -0.464532 2.102715
Co 0.235841 -0.122505 -0.331921
C -0.218860 0.080114 1.488086
C -1.089603 -1.315332 -0.452176
O -1.136633 0.647645 2.011491
O -2.050020 -1.936815 -0.565116
C -0.231692 1.560619 -0.623414
O -0.656845 2.609984 -0.817888
C 2.387844 0.078918 0.177634
O 2.134240 -1.056070 0.261591
H 0.709531 -0.759315 -1.888686
H 0.965611 -0.012393 -1.913554
FREQS= 64 70 92 109 129 176 185 225 311 332 354 373 425 463 495 503 512 537 566 621 793 888 1330 1369 1828 1955 2106 2152 2788 3652
MIN125_7
12
E= -1837.71239973 ZPE= 37.11 Gcorr -0.001618000
H -0.138625 -1.371844 -0.846935
Co -0.442182 -0.174014 -0.040570
C 1.288535 -0.419013 0.425524
C -2.164648 -0.704016 -0.206481
O 1.008840 -0.797170 1.555569
O -3.218621 -1.108222 -0.396466
C -0.575203 1.601915 0.207046
O -0.642258 2.744380 0.140545
C 2.726770 -0.401842 -0.089121
O 3.002644 -0.063161 -1.211585
H 3.484926 -0.723063 0.654482
H -0.264955 0.124413 -1.438459
FREQS= 39 56 80 90 114 150 262 288 361 427 432 472 481 515 531 546 600 617 643 782 981 1154 1367 1712 1828 2034 2132 2142 2175 2949
MIN126_7
12
E= -1837.71496673 ZPE= 37.85 Gcorr 0.001031000
H -0.052620 -0.625492 -1.760322
Co -0.114242 -0.230748 -0.276141
C -0.021387 1.447540 -0.744354
C -1.841217 -0.610179 -0.567138
O 0.027178 2.535749 -1.101151
O -1.648593 -1.678604 -0.006870
C -0.256123 0.164102 1.632926
O 0.117227 1.082145 2.310139
C 1.626704 -0.778626 -0.211631
O 2.714366 -1.127148 -0.269766
H -2.789759 -0.270689 -1.002016
H -0.802382 -0.707793 2.100502

FREQS=   68   72   86 100 114 197 211 302 337 376 430 462 492 506 523 558 580 704 732 854 875
1205 1316 1681 1780 1825 2140 2176 2692 3081
MIN127_7
12
E= -1837.71723456 ZPE=   40.34 Gcorr   0.003332000
H -1.243987 -0.311278 1.816224
Co 0.000410 0.088657 -0.332666
C -0.202933 -0.439672 1.406006
C 1.646260 -0.678330 -0.579190
O 0.669910 -0.853845 2.119779
O 2.623580 -1.215516 -0.853311
C -1.565556 -0.576194 -0.829730
O -2.672600 -1.095798 -0.316587
C 0.116390 1.860775 -0.061197
O 0.075757 2.991139 0.153468
H -2.585026 -1.161232 0.651388
H -1.720195 -0.528551 -1.927754
FREQS=   46   61   65   87 115 175 184 309 335 354 372 427 440 478 505 556 621 658 848 940 1188
1292 1350 1448 1805 2090 2139 2711 2941 3674
MIN128_7
12
E= -1837.71805563 ZPE=   39.63 Gcorr   0.004265000
H -1.935454 1.390105 0.136679
Co 0.226910 0.007092 -0.420729
C -1.665887 0.382388 -0.179744
C 1.703241 -0.969785 -0.269115
O -2.723915 -0.242951 -0.854042
O 2.569498 -1.709757 -0.133106
C -1.045737 -0.408655 0.881855
O -1.056813 -1.095457 1.839793
C 0.788771 1.598710 0.166866
O 1.175707 2.642521 0.443673
H -0.238386 -1.233092 -1.141341
H -2.350879 -0.719300 -1.605370
FREQS=   58   76   86   99 184 197 245 283 329 393 409 465 484 535 544 564 612 644 802 859 947
1115 1270 1376 1917 1963 2122 2166 3130 3843
MIN129_7
12
E= -1837.70522188 ZPE=   35.28 Gcorr   -0.008361000
H -1.979924 0.990880 -1.678670
Co -0.010438 0.080179 -0.387450
C -1.796113 0.282426 -0.845231
C 0.048843 1.539128 0.646783
O -2.698205 -0.290826 -0.297137
O 0.067075 2.539808 1.206509
C -0.319714 -1.390092 0.652224
O -0.447850 -2.367060 1.232075
C 3.412827 -0.450906 -0.474574
O 2.314849 -0.287456 -0.213426
H 0.335553 0.950739 -1.534765
H -0.035815 -0.745511 -1.624794
FREQS=   45   51   73   75   95 109 113 140 176 262 335 376 410 435 496 520 577 631 664 732 870
915 1314 1808 2006 2026 2143 2179 2180 2925
MIN130_7
12
E= -1837.71778617 ZPE=   41.44 Gcorr   0.005649000
H -0.278697 -2.740876 0.313640
Co 0.056759 -0.038504 -0.067981
C -0.389963 -1.846173 -0.333866
C 0.467745 1.603173 -0.625883
O -0.741718 -1.942307 -1.491963
O 0.700355 2.616796 -1.118868
C 1.640184 -0.471886 0.567614
O 2.598482 -0.688428 1.176712
C -1.698581 0.183115 0.364286
O -2.222732 0.769889 1.426961
H -1.505622 1.108366 1.989294
H -2.539569 -0.184857 -0.243080
FREQS=   44   69   84   95 108 136 216 269 308 364 417 453 492 542 559 616 625 715 888 1035 1218
1305 1334 1476 1742 2072 2126 2903 3041 3733
MIN131_7
12
E= -1837.71935633 ZPE=   41.50 Gcorr   0.007582000
H -1.607405 -0.240129 1.605439
Co 0.400388 -0.013422 -0.086821
C -1.344373 -0.581437 0.599076
C 2.146550 -0.479625 -0.297139

O -0.389914 -1.510387 0.466435
O 3.236094 -0.817551 -0.195641
C 0.510833 1.548404 0.710385
O 0.459081 2.093148 -0.379849
C -2.425998 -0.504676 -0.411774
O -3.403510 0.209833 -0.296613
H -2.258970 -1.138390 -1.312819
H 0.519822 2.044562 1.693617
FREQS= 57 61 91 117 154 172 214 298 365 405 423 442 481 568 652 705 861 953 1011 1112 1214
1238 1345 1430 1702 1788 2150 2892 3038 3089
MIN132_7
12
E= -1837.71471872 ZPE= 40.22 Gcorr 0.003076000
H -0.855493 2.321182 -0.100418
Co 0.195869 -0.059342 -0.250037
C -1.087607 1.324615 -0.489289
C 1.328199 1.164473 0.342020
O -2.465345 1.055617 -0.412978
O 1.949914 1.936708 0.931742
C -1.051469 -1.136661 0.420419
O -1.808203 -1.727387 1.055634
C 1.703721 -1.601581 0.211748
O 1.549702 -1.501820 -0.940068
H -0.808944 1.290117 -1.569406
H -2.789620 1.380908 0.436798
FREQS= 45 83 102 111 117 135 166 232 280 310 315 338 447 454 479 519 533 562 704 1081 1120
1261 1370 1455 1955 2072 2134 2855 3073 3824
MIN133_7
12
E= -1837.71317141 ZPE= 38.09 Gcorr 0.001543000
H 1.831435 1.501943 -1.197513
Co -0.047113 -0.042458 -0.114349
C 0.443318 -0.767116 1.501938
C 1.678844 0.473750 -0.810426
O 0.770974 -1.749718 0.864074
O 2.575734 -0.327589 -0.793646
C -1.750424 -0.718696 -0.365828
O -2.801726 -1.027486 -0.695207
C -0.414743 1.607717 0.300712
O -0.656893 2.698337 0.571799
H -0.008023 -0.027408 -1.634831
H 0.601967 -0.650454 2.585228
FREQS= 61 82 89 104 142 194 227 245 331 363 404 426 477 516 530 564 631 640 675 837 932
1206 1355 1707 1804 1887 2119 2163 2903 3031
MIN134_7
12
E= -1837.71078475 ZPE= 37.42 Gcorr -0.000759000
H 2.828267 -1.447993 -1.053764
Co -0.264100 -0.057779 0.323462
C 1.827341 -1.143640 -0.684676
C -0.371214 1.513224 -0.442974
O 0.797508 -1.718927 -1.019098
O -0.461717 2.594959 -0.829594
C -2.018536 -0.526222 0.203714
O -3.099731 -0.878871 0.066463
C 1.624259 0.017081 0.302015
O 2.552455 0.574559 0.837687
H -0.257761 0.059069 1.867636
H -0.119020 -0.787452 1.740522
FREQS= 43 61 89 96 124 140 242 297 313 350 421 441 466 515 541 557 601 621 686 900 1024
1062 1329 1723 1765 1812 2104 2159 2736 2953
MIN135_7
12
E= -1837.71337523 ZPE= 40.40 Gcorr 0.002282000
H 3.053078 0.612812 -0.038587
Co -0.171160 -0.026049 0.008779
C 1.245152 1.261912 -0.336991
C -1.475147 -1.209737 -0.264966
O 2.531018 0.895368 -0.800215
O -2.197375 -2.008296 -0.677654
C 1.024944 -1.144843 0.463437
O 1.851625 -1.844142 0.870270
C -2.002729 2.408583 0.600232
O -1.340000 1.511916 0.333521
H 1.306715 2.043787 0.440377
H 0.746060 1.712466 -1.216481

FREQS= 37 70 87 92 103 116 189 255 267 294 327 361 421 433 477 529 577 630 658 1073 1139 1258 1385 1452 2068 2099 2129 2924 2982 3824
MIN136_7
12
E= -1837.71210424 ZPE= 38.36 Gcorr 0.001066000
H -2.443483 0.120727 1.056723
Co 0.048962 -0.194275 0.134967
C -1.490862 -0.193269 1.504378
C -0.607854 1.199763 -0.887881
O -0.573985 0.678333 1.733757
O -0.967297 2.129345 -1.444984
C 1.807640 0.134036 0.088918
O 2.936909 0.295925 0.198635
C -1.063064 -1.846246 -0.755982
O -0.129036 -1.635868 -1.421217
H -1.514649 -1.127872 2.076314
H 0.628278 -1.255018 0.996732
FREQS= 71 74 83 110 135 140 175 249 298 358 364 396 420 438 459 516 537 714 783 899 1052 1216 1382 1590 1952 1980 2143 2180 3013 3103
MIN137_7
12
E= -1837.70430332 ZPE= 35.91 Gcorr -0.006595000
H 1.747973 1.494383 -1.546240
Co -0.160476 0.077727 -0.397813
C 1.609659 0.740173 -0.733476
C -0.397924 1.507215 0.584358
O 2.574895 0.322656 -0.135486
O -0.593838 2.494491 1.147849
C -1.831408 -0.620627 -0.191464
O -2.914654 -0.985625 -0.099040
C 1.397784 -2.435599 1.076888
O 0.739608 -1.666928 0.548539
H -0.456145 0.361840 -1.898052
H -0.075720 -0.418591 -1.927496
FREQS= 41 60 79 79 89 118 131 170 213 256 294 383 409 445 486 530 541 624 656 741 907 1034 1370 1737 1784 2095 2148 2165 2711 2823
MIN138_7
12
E= -1837.71576910 ZPE= 39.81 Gcorr 0.004953000
H -3.439569 -0.302523 -0.457395
Co 0.239885 0.013622 -0.419886
C -1.655818 0.401746 -0.184195
C 0.823610 1.604190 0.165988
O -2.624584 -0.230459 -0.977911
O 1.213253 2.632912 0.491192
C 1.692989 -0.990590 -0.274452
O 2.543013 -1.750101 -0.139317
C -1.046203 -0.419732 0.850535
O -1.103927 -1.127806 1.796558
H -0.247675 -1.256067 -1.040743
H -1.899169 1.420729 0.123621
FREQS= 58 78 84 96 181 230 292 297 344 409 420 468 493 537 555 564 642 661 814 861 938 1123 1269 1375 1943 1984 2122 2165 3099 3746
MIN139_7
12
E= -1837.71219070 ZPE= 37.98 Gcorr 0.001679000
H 0.000000 -3.122281 -0.340414
Co 0.000000 -0.202182 -0.097782
C 0.000000 -2.079682 0.030095
C 0.000000 1.512231 0.876635
O 0.000000 -1.835215 1.233359
O 0.000000 2.635404 0.449623
C 1.734585 0.033896 -0.401106
O 2.817841 0.220455 -0.727539
C -1.734585 0.033896 -0.401106
O -2.817841 0.220455 -0.727539
H 0.000000 0.318328 -1.456364
H 0.000000 1.332023 1.986556
FREQS= 70 84 89 120 133 175 233 256 364 389 393 467 478 498 525 527 565 645 747 806 861 1242 1335 1667 1814 2082 2125 2173 2741 2961
MIN140_7
12
E= -1837.71366535 ZPE= 40.33 Gcorr 0.003188000
H -1.305549 -0.204039 1.785226
Co 0.010576 0.085476 -0.335722
C -1.596922 -0.480043 -0.812294
C 1.584084 -0.819386 -0.563169

O -2.661737 -0.917780 -0.129904
O 2.515338 -1.441130 -0.822066
C 0.279704 1.837494 -0.083607
O 0.333892 2.976675 0.081654
C -0.267811 -0.374514 1.421487
O 0.593914 -0.810587 2.134202
H -3.421494 -1.104037 -0.702525
H -1.804084 -0.438519 -1.903800
FREQS= 48 59 62 85 114 172 191 302 337 348 372 433 445 483 509 516 572 655 839 909 1201
1257 1285 1455 1804 2083 2134 2889 2900 3752
MIN141_7
12
E= -1837.71132397 ZPE= 37.33 Gcorr 0.001362000
H 2.805043 -1.331145 1.199221
Co -0.272764 -0.100339 -0.191079
C 1.834140 -0.974209 0.805993
C -0.296332 1.597328 0.387989
O 0.776916 -1.146021 1.415326
O -0.335237 2.730654 0.554817
C 1.595703 -0.199628 -0.500705
O 2.466900 0.104244 -1.274558
C -2.018033 -0.593986 -0.151212
O -3.094335 -0.960013 -0.280253
H -0.119242 -1.237854 -1.129262
H -0.528004 0.470208 -1.485866
FREQS= 69 78 89 101 178 225 306 335 355 402 432 453 491 517 523 601 640 651 695 788 885
992 1323 1705 1830 2010 2134 2141 2180 2984
MIN142_7
12
E= -1837.70628412 ZPE= 36.24 Gcorr -0.003186000
H -2.078419 0.751351 -1.606879
Co 0.097820 -0.043583 -0.360997
C -0.379164 -1.280518 1.412325
C 0.035113 1.561009 0.332217
O -0.354236 -2.026502 0.523612
O 0.005171 2.629348 0.760484
C 1.910156 -0.277854 -0.167470
O 3.050571 -0.327085 -0.081602
C -1.790888 0.193044 -0.682433
O -2.648697 -0.277307 0.023902
H 0.285227 0.028068 -1.967322
H 0.078274 -0.764407 -1.857877
FREQS= 61 74 89 99 162 173 176 193 228 293 317 391 405 427 473 531 535 579 617 680 904
972 1368 1560 1795 1994 2110 2160 2819 3164
MIN143_7
12
E= -1837.70206001 ZPE= 35.70 Gcorr -0.007257000
H -1.720086 1.591014 0.496796
Co 0.016780 -0.059703 -0.288332
C -1.720973 0.726962 -0.232760
C -0.622606 -1.288032 0.779950
O -2.719794 0.488317 -0.859523
O -1.056281 -2.164257 1.391676
C 1.699303 -0.738700 -0.439186
O 2.710347 -1.259947 -0.589580
C 1.241977 2.778193 1.027007
O 0.809291 1.825390 0.569298
H -0.298142 -0.377209 -1.784420
H 0.030465 0.411615 -1.832447
FREQS= 41 55 79 84 91 104 134 162 203 264 321 375 387 436 489 528 538 597 635 715 875
1050 1352 1723 1818 2096 2146 2157 2679 2841
MIN144_7
12
E= -1837.70572552 ZPE= 36.10 Gcorr -0.003507000
H 2.112506 -1.226473 0.610934
Co -0.063709 -0.122165 -0.226235
C 1.836433 -0.472150 -0.173351
C -1.858493 0.226863 -0.433942
O 2.680352 -0.039941 -0.912667
O -2.934800 0.563965 -0.628876
C 0.345768 1.404326 0.528636
O 0.610465 2.418105 1.003402
C -0.412009 -1.367265 1.499655
O -0.361400 -2.119221 0.609512
H 0.157379 -0.005302 -1.845439
H 0.023120 -0.803688 -1.754087

FREQS= 63 77 84 98 134 171 183 214 247 316 330 380 384 418 473 519 527 537 607 670 871
946 1362 1511 1817 1952 2115 2162 2767 3314
MIN145_7
12
E= -1837.71417684 ZPE= 40.77 Gcorr 0.004982000
H 2.710501 -0.716424 -0.083288
Co 0.142715 0.058579 -0.159103
C 2.060669 -0.319060 -0.874990
C 0.316838 -1.046928 1.191605
O 1.763418 0.921459 -0.899323
O 0.328230 -1.773499 2.086809
C -0.576866 1.557885 0.531590
O -1.088730 2.466290 1.010903
C -1.095931 -0.600763 -1.048775
O -2.271470 -1.144056 -1.035043
H 1.984471 -0.925793 -1.788280
H -2.628118 -1.247779 -1.935996
FREQS= 60 70 77 92 117 132 263 286 341 368 396 461 479 496 513 533 560 666 708 1069 1123
1225 1410 1448 1602 2097 2148 3000 3079 3703
MIN146_7
12
E= -1837.71308912 ZPE= 40.58 Gcorr 0.004108000
H -1.769334 -0.957238 -1.951940
Co -0.103253 0.045679 -0.152552
C -1.942624 -0.342729 -1.057630
C 0.639391 1.590151 0.413930
O -1.653909 0.897665 -1.062913
O 1.167476 2.532443 0.800921
C -0.497184 -0.834414 1.317147
O -0.630024 -1.441002 2.290118
C 1.113127 -0.778995 -0.912965
O 2.215559 -1.433420 -1.026343
H -2.662428 -0.740890 -0.328707
H 2.550514 -1.784759 -0.177602
FREQS= 47 68 75 92 112 130 254 281 322 360 395 456 472 497 524 533 566 658 698 1070 1093
1226 1418 1487 1606 2090 2147 2999 3079 3631
MIN147_7
12
E= -1837.71207354 ZPE= 39.55 Gcorr 0.003126000
H -0.220321 -0.992821 2.007144
Co 0.168029 0.153648 -0.257533
C -0.070500 0.024827 1.572994
C -1.537151 0.659867 -0.613723
O -0.037515 1.039373 2.218013
O -2.634777 0.934194 -0.791249
C 1.988247 0.129527 -0.266302
O 1.910611 1.068906 -1.057450
C -0.122788 -1.647844 -0.488116
O -0.332613 -2.544957 0.284877
H 2.955649 -0.272637 0.080615
H -0.064619 -1.861422 -1.597030
FREQS= 65 66 75 106 122 163 207 227 277 320 395 423 455 518 539 633 661 838 867 930 1243
1253 1290 1663 1801 1826 2151 2691 2856 3004
MIN148_7
12
E= -1837.70852664 ZPE= 38.20 Gcorr -0.000394000
H -1.657081 -1.910202 -0.865583
Co 0.026915 0.051880 -0.235604
C -0.935555 -1.285859 -1.413013
C 0.001925 -0.971421 1.285859
O 0.330253 -1.522997 -1.286289
O 0.093997 -1.663293 2.192293
C 1.569461 0.898924 -0.020062
O 2.594550 1.419040 -0.023651
C -2.329711 2.054818 0.463190
O -1.489084 1.306762 0.262370
H -1.310303 -0.835086 -2.340774
H 0.166227 0.849675 -1.485978
FREQS= 59 63 81 97 112 133 194 205 221 366 380 405 436 442 475 530 548 713 755 881 1031
1210 1355 1576 1960 2122 2142 2167 2992 3074
MIN149_7
12
E= -1837.70544720 ZPE= 36.38 Gcorr -0.003454000
H -2.091543 0.598408 1.665172
Co 0.102090 -0.033656 0.353256
C -0.269400 -2.132815 -0.300345
C -0.028005 1.584190 -0.357225

O -0.335176 -1.521541 -1.280151
O -0.089032 2.665001 -0.745409
C -1.792234 0.126903 0.697426
O -2.636413 -0.291013 -0.054865
C 1.913714 -0.220613 0.143331
O 3.056207 -0.253378 0.077520
H 0.128132 -0.727314 1.808246
H 0.297856 0.099073 1.912778
FREQS= 51 77 83 93 149 151 177 184 233 305 314 381 412 433 480 507 526 615 666 741 907
1049 1369 1721 1801 2018 2115 2160 2817 2911
MIN150_7
12
E= -1837.70407579 ZPE= 35.89 Gcorr -0.004558000
H 1.595101 1.139190 -1.949517
Co -0.190969 0.057367 -0.332726
C 1.534236 0.438755 -1.089061
C -0.172327 1.514132 0.707709
O 2.539389 -0.072483 -0.651897
O -0.217451 2.547975 1.202478
C -1.905899 -0.552863 -0.193172
O -2.999990 -0.854757 -0.344611
C 1.289762 -2.177854 1.437321
O 0.669831 -1.398443 0.879885
H -0.127859 -0.733693 -1.589197
H -0.719941 0.934226 -1.341306
FREQS= 59 70 82 84 106 157 182 190 237 310 341 370 435 451 490 505 622 635 654 700 851
921 1376 1780 1997 2130 2134 2171 2176 2887
MIN151_7
12
E= -1837.70588932 ZPE= 36.46 Gcorr -0.002692000
H -2.096515 -0.996460 0.826571
Co 0.061897 -0.095889 -0.207614
C 0.416850 -2.180281 0.350029
C -0.344396 1.472670 0.516993
O 0.331903 -1.623689 1.367986
O -0.602155 2.524457 0.902588
C 1.863198 0.218692 -0.391040
O 2.952427 0.507428 -0.591860
C -1.831643 -0.443084 -0.114778
O -2.682564 -0.173916 -0.919483
H -0.023923 -0.756642 -1.686001
H -0.171738 0.059886 -1.776052
FREQS= 53 80 89 96 140 177 187 236 276 320 329 380 392 423 485 503 520 606 636 697 899
1039 1346 1683 1822 1969 2117 2162 2761 3086
MIN152_7
12
E= -1837.71849215 ZPE= 41.77 Gcorr 0.010066000
H -1.688569 2.147877 -0.886083
Co 0.087136 0.278834 -0.266376
C -1.246192 1.716675 0.021310
C 1.852318 -0.097334 0.067257
O 0.000941 2.007891 0.314882
O 2.896585 -0.405288 0.411232
C -0.853340 -0.994215 0.675367
O -1.135008 -1.261996 1.799273
C -1.231539 -1.141003 -0.731386
O -0.213180 -1.234517 -1.508130
H -1.940716 1.506536 0.842199
H -2.245560 -0.936382 -1.097317
FREQS= 68 78 108 149 179 238 313 339 405 411 419 459 512 547 617 634 776 847 918 1078 1211
1293 1312 1441 1563 1936 2174 3015 3068 3108
MIN153_7
12
E= -1837.71536513 ZPE= 41.81 Gcorr 0.007642000
H -3.100765 -0.006213 -0.599945
Co 0.248856 -0.193756 -0.167778
C -2.256229 -0.222516 0.062271
C -1.207452 0.902158 0.152861
O -1.408532 -1.266985 -0.528766
O -1.470029 2.049711 0.420088
C 1.405435 1.076746 -0.164067
O 2.174513 1.933905 -0.210009
C 1.158198 -1.426202 1.039071
O 1.456549 -1.975937 0.036811
H -1.625352 -2.152991 -0.210400
H -2.612722 -0.516062 1.054543

FREQS= 48 77 93 118 131 167 220 313 330 390 407 420 460 493 544 593 620 892 965 973 1138
1226 1337 1481 1823 1860 2115 3057 3128 3828
MIN154_7
12
E= -1837.70929154 ZPE= 38.11 Gcorr 0.001846000
H 0.258775 0.999146 -1.324778
Co -0.225497 0.189283 -0.178694
C 0.915372 1.282746 0.543048
C -1.932987 0.942262 -0.203138
O 1.671126 2.039625 0.957265
O -2.084722 0.017671 -0.997727
C -0.373325 -1.070190 1.138358
O -0.382595 -1.842454 1.983550
C 0.957878 -0.969149 -1.218484
O 2.144954 -1.136977 -1.116790
H -2.741214 1.669861 -0.010304
H 0.379133 -1.516589 -2.009267
FREQS= 67 78 89 115 134 162 225 296 375 410 431 436 465 494 525 564 595 642 796 819 880
1213 1341 1656 1807 1984 2136 2169 2762 2990
MIN155_7
12
E= -1837.70922176 ZPE= 37.77 Gcorr 0.002082000
H 0.834974 -1.919794 -1.207122
Co -0.292211 -0.045169 0.361381
C 0.817534 -1.067472 -0.530814
C -0.998222 1.581214 -0.168097
O 3.184243 -0.422536 -0.418164
O -1.291292 2.549856 -0.696572
C -1.639332 -1.125390 -0.031243
O -2.465707 -1.855610 -0.341501
C 2.014029 -0.242453 -0.166313
O 1.453957 0.734533 0.539813
H -0.434350 -0.253807 2.115543
H -0.724580 0.467832 2.044469
FREQS= 63 82 98 103 145 284 310 370 386 406 422 427 460 496 540 557 609 681 707 770 860
951 1124 1219 1222 1811 2143 2182 3190 3797
MIN156_7
12
E= -1837.70712679 ZPE= 38.31 Gcorr 0.000661000
H 0.368451 -1.220706 -1.230047
Co 0.037875 0.038784 0.041824
C -1.405086 -1.011425 0.857603
C -1.271443 1.106450 -0.451883
O -1.991991 -1.919376 0.329749
O -2.088510 1.805746 -0.857829
C 1.235114 1.336551 0.293751
O 1.922675 2.192919 0.633303
C 1.223548 -1.315206 -0.448330
O 2.245062 -1.909171 -0.613746
H 0.843925 -1.153159 0.744952
H -1.625689 -0.732458 1.917188
FREQS= 37 80 83 98 102 195 229 276 328 340 392 458 467 502 524 536 558 672 865 899 1294
1330 1400 1798 1869 1965 2107 2150 2431 2815
MIN157_7
12
E= -1837.70968615 ZPE= 40.35 Gcorr 0.003255000
H -3.396008 -1.477302 0.132757
Co -0.128860 -0.013513 -0.141720
C -1.662445 -0.700774 0.495523
C 1.313353 -0.898003 -0.846336
O -2.607348 -1.166445 -0.338507
O 2.123027 -1.535272 -1.354308
C -0.082633 1.735592 -0.535551
O -0.136703 2.874321 -0.700682
C 0.533625 0.102619 1.559884
O 1.680909 -0.074412 1.866246
H -1.983720 -0.773455 1.549640
H -0.231523 0.393455 2.320935
FREQS= 58 59 67 86 118 184 184 252 276 342 347 405 465 486 499 509 604 708 852 944 1208
1252 1275 1473 1806 2086 2136 2824 2972 3745
MIN158_7
12
E= -1837.70719326 ZPE= 38.07 Gcorr 0.001467000
H 0.391472 0.225982 -1.619080
Co 0.110701 0.183156 -0.161128
C 1.732172 -0.407696 -0.284028
C 0.217270 2.029290 0.063876

O 2.808255 -0.782680 -0.435097
O -0.967847 1.947518 -0.226911
C -0.359721 -0.399681 1.505340
O -0.605006 -0.839179 2.534008
C -0.976943 -1.235648 -0.986208
O -2.145808 -1.061322 -1.220818
H 0.716759 3.000929 0.224182
H -0.490584 -2.204418 -1.257992
FREQS= 71 78 88 112 118 167 203 274 336 396 429 441 465 507 535 583 602 637 799 803 871
1213 1358 1682 1794 1981 2125 2163 2806 2992
MIN159_7
12
E= -1837.70945843 ZPE= 39.84 Gcorr 0.003735000
H 2.066696 -1.785059 -0.804714
Co 0.029130 -0.231158 -0.190059
C 1.520557 -1.498467 0.102294
C -1.784298 -0.094927 -0.214067
O 0.309065 -1.978055 0.289098
O -2.928499 -0.106071 -0.178804
C 0.197693 0.623083 1.417982
O 0.454717 1.785288 1.566189
C 1.519641 1.037827 -1.199215
O 0.440639 1.422567 -1.420644
H 2.151870 -1.289209 0.974290
H 0.066000 -0.079396 2.273345
FREQS= 66 75 105 128 138 160 166 199 231 324 335 408 411 444 473 576 615 777 891 1069 1204
1280 1304 1571 1826 1953 2153 2874 3016 3098
MIN160_7
12
E= -1837.70516578 ZPE= 38.68 Gcorr -0.000464000
H 1.341725 -0.391103 0.776607
Co -0.006906 0.062601 -0.111046
C 1.867313 -0.290132 -0.295742
C 0.131510 1.809843 0.270707
O 3.058028 -0.367213 -0.364707
O 0.142477 2.893332 0.646627
C -1.728804 0.139948 -0.328711
O -2.864287 0.219703 -0.511281
C -0.404452 -1.859631 -0.003647
O -0.461865 -2.427082 1.054815
H -0.546815 -2.421438 -0.958945
H 1.203318 -0.227766 -1.278689
FREQS= 13 52 87 99 103 179 234 283 304 375 402 453 461 490 538 572 585 669 834 898 1350
1410 1627 1805 1880 2042 2102 2155 2233 2819
MIN161_7
12
E= -1837.70206876 ZPE= 36.72 Gcorr -0.003557000
H -0.475665 -0.089337 -1.520161
Co -0.482363 -0.178598 0.001075
C 1.175698 -0.838176 0.000789
C -0.195784 1.524946 0.001607
O 0.701681 -1.964121 0.004354
O -0.014612 2.656386 0.002540
C -2.298810 -0.306132 -0.001886
O -3.438489 -0.387501 -0.004495
C 2.667615 -0.527626 -0.002014
O 3.070835 0.606785 -0.004996
H 3.330259 -1.414943 -0.001040
H -0.478418 -0.084049 1.521987
FREQS= 12 59 74 88 102 174 236 304 360 427 458 464 502 527 558 596 606 615 686 798 976
1156 1367 1721 1750 1826 1939 2150 2186 2974
MIN162_7
12
E= -1837.70780149 ZPE= 37.22 Gcorr 0.002236000
H -1.004100 2.125402 0.866122
Co -0.173865 -0.379116 0.008599
C -0.072573 1.536379 0.851101
C 0.619135 1.436946 -0.411906
O 0.163327 0.635997 1.731452
O 1.273052 1.983373 -1.235142
C 1.407817 -1.187409 -0.042760
O 2.370090 -1.800810 -0.111245
C -1.918908 -0.406151 -0.266274
O -3.028745 -0.541247 -0.518070
H -0.526063 -1.701356 0.606728
H -0.210087 -0.685008 -1.421941

```
FREQS=   68   85   97  124  220  263  316  378  413  421  454  474  487  493  521  595  647  653  671  757  872
932 1321 1461 1943 1964 2057 2144 2191 3016
MIN163_7
12
E= -1837.70057224 ZPE=    35.64 Gcorr   -0.004809000
H 1.895839 0.687580 -1.877248
Co -0.127776 0.003626 -0.318681
C 0.562438 -2.058839 0.777029
C -0.020112 1.617332 0.465153
O 0.473235 -1.176595 1.512698
O 0.020426 2.710569 0.804984
C 1.678123 0.106112 -0.956164
O 2.569543 -0.464500 -0.371384
C -1.912705 -0.388681 -0.182602
O -3.028361 -0.583293 -0.343866
H -0.077067 -0.987533 -1.417844
H -0.494032 0.657053 -1.540487
FREQS=   65   79   84   98  108  129  175  184  227  303  324  368  427  446  478  506  593  614  647  698  843
922 1372 1779 2020 2071 2141 2153 2182 2893
MIN164_7
12
E= -1837.70606705 ZPE=    37.02 Gcorr    0.001311000
H -0.000057 2.938543 1.163173
Co -0.000004 -0.511710 0.064426
C -0.000008 1.877930 0.846061
C 0.000027 1.313676 -0.589723
O -0.000018 0.955318 1.665778
O 0.000031 2.005441 -1.578002
C 1.729183 -0.846234 -0.077462
O 2.815229 -1.175346 -0.234961
C -1.729187 -0.846213 -0.077487
O -2.815229 -1.175336 -0.234987
H -0.000027 -1.853474 0.777876
H -0.000017 -1.144473 -1.231525
FREQS=   80   84   91  107  179  216  284  350  382  438  464  468  486  530  535  571  613  649  659  782  857
963 1316 1693 1820 1861 2117 2137 2186 2979
MIN165_7
12
E= -1837.69957646 ZPE=    35.56 Gcorr   -0.005121000
H 1.838363 1.577799 -0.379173
Co -0.074988 -0.063428 -0.252052
C -0.667363 2.815867 1.091504
C 0.489286 -1.085781 1.103312
O -0.452161 1.783750 0.651900
O 0.897157 -1.878835 1.824184
C -1.821040 -0.549058 -0.455524
O -2.824591 -0.978964 -0.802368
C 1.701824 0.502784 -0.695944
O 2.618121 -0.074494 -1.214614
H -0.157380 0.404020 -1.658351
H 0.219256 -1.183795 -1.089980
FREQS=   59   73   85   87  105  165  177  184  231  324  355  375  399  443  489  505  596  610  648  698  838
900 1372 1826 2005 2131 2151 2164 2188 2690
MIN166_7
12
E= -1837.70674964 ZPE=    39.37 Gcorr    0.002134000
H 0.625899 0.399177 1.384628
Co 0.493409 -0.108526 0.045616
C -1.192435 -0.909024 -0.096016
C 0.177290 1.593485 -0.130842
O -0.214804 -1.756377 -0.305493
O -0.069837 2.710784 -0.235558
C 2.312464 -0.337939 0.044582
O 3.447714 -0.443363 0.107936
C -2.515634 -0.848345 -0.038637
O -3.268069 0.272247 0.242600
H -3.128652 -1.721147 -0.235368
H -2.669420 0.996784 0.468719
FREQS=   40   63   72   75  158  190  244  311  331  370  405  410  437  485  523  589  597  634  710  753 1022
1173 1268 1392 1830 2113 2128 2187 3215 3814
MIN167_7
12
E= -1837.71201191 ZPE=    41.73 Gcorr    0.008051000
H -1.841011 1.956332 0.819370
Co -0.306389 -0.030486 0.053648
C -0.766575 1.743632 0.738786
C -1.923447 -0.532312 -0.338940
```

O -0.196734 1.767850 -0.432403
O -3.004295 -0.858602 -0.554868
C 2.304739 -0.248222 -0.122472
O 2.966843 0.218001 -0.952329
C 1.466859 -0.750186 0.824527
O 0.496155 -1.602720 0.390625
H -0.181261 2.002733 1.635826
H 1.709566 -0.609639 1.876713
FREQS= 49 53 96 116 139 183 240 340 393 448 465 486 529 566 644 669 711 778 1065 1092 1152
1204 1334 1355 1576 2129 2193 2975 3059 3154
MIN168_7
12
E= -1837.69608638 ZPE= 35.35 Gcorr -0.005004000
H 1.864673 -1.635142 0.014624
Co -0.080682 -0.073972 -0.249234
C 1.719609 -0.659259 -0.529254
C -1.869906 0.257413 -0.499496
O 2.630407 -0.180743 -1.145565
O -2.904787 0.537136 -0.898787
C 0.450159 1.332221 0.728651
O 0.821338 2.306754 1.202614
C -0.374686 -1.239361 1.809248
O -0.451828 -1.964559 0.915524
H 0.142756 0.726923 -1.410568
H -0.141115 -0.829311 -1.519918
FREQS= 67 79 92 105 110 146 170 177 257 315 348 378 395 429 482 498 513 601 641 694 818
901 1358 1832 2026 2055 2141 2171 2202 2724
MIN169_7
12
E= -1837.70701713 ZPE= 40.08 Gcorr 0.006702000
H 0.058818 -2.565672 0.248440
Co -0.263991 -0.040106 -0.325921
C 0.269709 -1.591166 0.703540
C 1.646005 -0.248155 -0.165909
O 1.726596 -1.345204 0.594562
O 2.556940 0.306659 -0.721773
C -2.065646 -0.354574 -0.277863
O -3.166372 -0.564238 -0.048543
C -0.092602 1.545793 0.241972
O -0.013876 2.573633 0.785097
H -0.023509 -1.598302 1.755151
H -0.278658 1.368652 -1.088898
FREQS= 61 84 86 105 172 272 321 387 401 448 454 503 550 589 600 639 686 698 887 961 1093
1178 1291 1478 1830 1854 2061 2163 3053 3135
MIN170_7
12
E= -1837.70550663 ZPE= 39.54 Gcorr 0.005371000
H 2.153565 1.486338 -0.605529
Co -0.149908 0.023384 -0.384500
C 1.749619 0.511881 -0.361994
C -1.119435 1.373967 0.053702
O 1.547784 -0.365007 -1.445990
O -1.785012 2.274027 0.335731
C -1.149684 -1.407951 0.018890
O -1.815260 -2.281445 0.354371
C 1.278316 -0.045020 0.883492
O 1.600393 -0.138908 2.032993
H 1.930644 -1.234937 -1.253468
H -0.972822 0.610633 -1.540883
FREQS= 53 83 94 104 213 274 298 327 389 412 458 482 537 567 578 590 615 657 698 721 952
1075 1213 1310 1812 1901 2100 2147 3233 3762
MIN171_7
12
E= -1837.69713291 ZPE= 37.97 Gcorr -0.002680000
H -3.817149 0.000488 -1.376614
Co 0.677967 -0.000011 0.504006
C -3.199773 0.000230 -0.471641
C 1.547696 -1.424870 -0.107913
O -3.558101 -0.000160 0.661145
O 2.031565 -2.328918 -0.630749
C 1.548206 1.424578 -0.107814
O 2.032390 2.328489 -0.630594
C -0.854252 0.000253 -0.052284
O -1.851705 0.000449 -0.896746
H 1.013824 0.406509 2.090978
H 1.013762 -0.406730 2.090944

FREQS= 10 51 57 78 137 183 266 275 289 332 451 477 495 519 537 541 619 636 773 968 987
999 1334 1439 1523 1875 2098 2142 3120 3349
MIN172_7
12
E= -1837.69774798 ZPE= 38.00 Gcorr -0.001634000
H -3.831288 -0.001797 0.024489
Co 0.826766 -0.000089 0.620791
C -2.764908 -0.000755 -0.226256
C 1.138349 -1.421826 -0.395877
O -2.266492 0.001054 -1.304974
O 1.210049 -2.315073 -1.117788
C 1.136706 1.422993 -0.394487
O 1.207395 2.317042 -1.115502
C -0.747789 -0.001202 1.052140
O -2.052871 -0.001948 0.997203
H 2.074598 0.406423 1.665004
H 2.075199 -0.406069 1.664512
FREQS= 17 43 62 81 146 182 268 302 302 353 459 482 492 519 538 552 613 631 759 948 973
995 1326 1434 1515 1871 2101 2144 3120 3357
MIN173_7
12
E= -1837.70559278 ZPE= 39.91 Gcorr 0.006577000
H 2.100562 -1.657965 -0.390423
Co -0.125771 -0.044602 -0.374418
C 1.726284 -0.649555 -0.272137
C 1.264801 0.049748 0.894875
O 1.622871 0.129251 -1.452195
O 1.545144 0.274992 2.035875
C -1.242024 -1.278683 0.036198
O -1.973322 -2.085925 0.461186
C -1.075729 1.436413 -0.108598
O -1.666530 2.377853 0.183445
H 2.090648 0.971328 -1.341927
H -1.060681 -1.026015 -1.286872
FREQS= 64 86 95 103 213 290 320 336 408 437 483 507 519 565 590 610 617 676 719 904 971
1070 1211 1301 1734 1913 2036 2138 3238 3762
MIN174_7
12
E= -1837.69912071 ZPE= 38.04 Gcorr 0.000452000
H -0.797894 -0.643529 1.100032
Co 0.534997 -0.245412 0.271318
C -1.247900 -0.923952 -0.015569
C 0.176633 1.418831 -0.217287
O -0.944997 -1.986985 -0.542187
O 0.071649 2.559037 -0.327392
C 2.251468 -0.200163 -0.033438
O 3.384659 -0.229348 -0.217275
C -2.551408 -0.194685 -0.285394
O -2.944203 0.698459 0.426288
H -3.101786 -0.566812 -1.171205
H 1.145147 -1.093038 1.340239
FREQS= 43 63 82 105 125 154 193 295 360 383 417 455 479 496 551 559 592 696 979 1000 1104
1361 1473 1724 1817 1907 1954 2112 2154 2979
MIN175_7
12
E= -1837.70525193 ZPE= 39.90 Gcorr 0.006614000
H -0.699293 0.276903 1.603851
Co -0.198938 -0.015111 0.234105
C 1.535935 -0.438063 0.071027
C -1.963783 -0.108854 -0.218772
O 2.700154 0.122717 -0.269588
O -3.080031 -0.244101 -0.427849
C 0.063516 1.695214 -0.004844
O 0.247237 2.825615 -0.064591
C 0.925591 -1.636023 -0.360638
O -0.094992 -1.978469 0.377916
H 3.350036 -0.562543 -0.492448
H 1.174100 -2.186096 -1.279980
FREQS= 58 90 99 110 286 291 302 327 385 404 429 458 519 546 573 626 637 646 697 912 1073
1236 1317 1366 1504 1922 2139 2173 3038 3750
MIN001_8
18
E= -1916.42631065 ZPE= 77.51 Gcorr 0.054198000
H -3.243002 -1.627140 0.138012
Co 0.751197 -0.034496 0.090247
C 2.424155 -0.030086 0.642733
C 0.810871 -1.762003 -0.465009

O 3.465636 -0.019873 1.131088
O 0.836755 -2.783392 -0.980970
C 0.849325 1.623737 -0.540961
O 0.922156 2.617369 -1.112041
C -3.286617 0.470656 0.694791
C -3.929316 -0.778163 0.087643
H -3.979475 1.321078 0.637311
H -3.076203 0.305757 1.758846
H -4.848493 -1.048215 0.617327
H -4.186245 -0.616570 -0.965800
C -1.982873 0.880381 0.004894
O -1.030976 -0.154491 0.144157
H -1.626343 1.828762 0.439377
H -2.184404 1.063694 -1.064146
FREQS= 15 43 62 72 91 97 118 144 196 240 288 306 377 408 421 437 465 481 519 585 590
674 799 883 931 1010 1094 1135 1169 1272 1309 1382 1401 1427 1491 1507 1520 1523 2103 2116 2181 2958 2992
3033 3044 3072 3109 3135
MIN002_8
18
E= -1916.42636780 ZPE= 77.52 Gcorr 0.054999000
H 5.350121 0.400409 0.880960
Co -0.816891 0.015614 0.078062
C -2.385086 -0.457951 0.726188
C -1.387060 1.665242 -0.422381
O -3.349950 -0.761551 1.274178
O -1.728962 2.645051 -0.905307
C -0.494795 -1.597286 -0.594336
O -0.328414 -2.566453 -1.187592
C 3.204037 0.591598 0.555859
C 4.547822 -0.111418 0.340957
H 2.958733 0.625197 1.624235
H 3.257665 1.631542 0.213491
H 4.518998 -1.147871 0.695903
H 4.823292 -0.132373 -0.719481
C 2.055260 -0.098536 -0.181600
O 0.862380 0.623481 0.036718
H 1.980100 -1.143351 0.164840
H 2.285650 -0.129261 -1.259724
FREQS= 31 53 61 67 88 97 109 144 195 236 290 306 347 380 412 439 467 482 519 586 594
706 771 902 909 1042 1097 1135 1187 1272 1321 1335 1416 1427 1504 1515 1516 1530 2102 2115 2181 2954 2991
3040 3047 3083 3110 3117
MIN003_8
18
E= -1916.42681077 ZPE= 77.55 Gcorr 0.055548000
H 4.881107 0.101142 -1.033794
Co -0.812865 0.007185 0.193711
C -2.440703 -0.666134 0.239436
C -0.101086 -1.501418 -0.419598
O -3.490807 -1.104087 0.408903
O 0.355281 -2.398051 -0.974043
C -1.373102 1.634596 -0.383017
O -1.643875 2.619385 -0.899814
C 2.852640 0.449946 -0.314166
C 4.274280 -0.093933 -0.144509
H 2.875782 1.529994 -0.501507
H 2.376639 -0.008835 -1.190977
H 4.778985 0.371324 0.709608
H 4.271081 -1.177001 0.022633
C 1.971406 0.194840 0.911270
O 0.692170 0.771728 0.779613
H 2.438813 0.658859 1.793579
H 1.922166 -0.888658 1.116488
FREQS= 34 44 59 85 91 98 113 129 227 261 284 306 376 392 416 443 479 494 521 570 596
664 763 893 902 1038 1104 1126 1191 1275 1318 1329 1408 1426 1500 1512 1514 1526 2101 2115 2180 2957 3011
3036 3041 3078 3109 3118
MIN004_8
18
E= -1916.43018603 ZPE= 78.62 Gcorr 0.058973000
H 2.506602 2.448773 0.762231
Co -0.818177 0.013629 -0.014854
C -1.300380 1.681522 -0.178606
C 1.138825 -1.519759 -0.198037
O -1.597131 2.778203 -0.363765
O 1.977012 -2.384726 -0.310269
C -2.451180 -0.538565 0.213606
O -3.513261 -0.917327 0.418809
C 2.838786 0.317020 0.408892

```
C 3.237276 1.787260 0.282137
H 3.584978 -0.333098 -0.056798
H 2.790188 0.022540 1.463159
H 4.205298 1.967894 0.758517
H 3.321733 2.089533 -0.767518
C 1.497875 -0.018132 -0.264683
O -0.161215 -1.696479 -0.037341
H 0.761554 0.628849 0.312067
H 1.509978 0.314074 -1.309945
FREQS=   24   55   66   97   99  104  146  174  226  261  322  339  401  446  466  493  507  560  636  649  745
777  884  896  945 1063 1105 1118 1248 1271 1306 1375 1412 1433 1501 1513 1517 1578 1841 2109 2169 2601 3040
3061 3074 3098 3123 3124
MIN005_8
18
E= -1916.42637590 ZPE=    77.59 Gcorr    0.055352000
H -3.381042 -0.636081 -1.916401
Co 0.692688 0.023430 -0.024178
C 2.195959 -0.224038 -0.908472
C 1.157285 1.684770 0.540681
O 3.100092 -0.377152 -1.603115
O 1.462016 2.658702 1.059088
C 0.630440 -1.660652 0.539953
O 0.647677 -2.682755 1.063198
C -3.403134 0.178865 0.105625
C -3.475201 0.334514 -1.414756
H -4.223648 -0.459400 0.460255
H -3.528717 1.154941 0.589633
H -4.428065 0.776257 -1.723380
H -2.669772 0.979155 -1.776714
C -2.088270 -0.432935 0.595921
O -1.018219 0.435939 0.286439
H -1.958923 -1.427330 0.134758
H -2.147405 -0.581169 1.686064
FREQS=   27   46   57   80   93  100  104  170  211  246  293  300  379  404  417  435  465  480  519  586  590
679  793  877  933 1013 1091 1135 1172 1274 1309 1381 1406 1427 1489 1508 1521 1524 2103 2115 2181 2954 2997
3035 3041 3076 3108 3131
MIN006_8
18
E= -1916.41810358 ZPE=    74.30 Gcorr    0.047221000
H 3.598645 0.876000 1.378410
Co -0.791535 0.000277 -0.154458
C -1.470119 -1.564555 -0.560810
C -1.159132 -0.003510 1.590590
O -2.008862 -2.520355 -0.921837
O -1.460489 -0.005864 2.700053
C -1.470989 1.566403 -0.554107
O -2.010244 2.523425 -0.911134
C 3.590985 0.002828 -0.611718
C 3.990669 -0.003586 0.861636
H 4.001965 0.874409 -1.144335
H 4.002146 -0.863953 -1.151969
H 3.599009 -0.887857 1.370635
H 5.079047 -0.003799 0.960319
C 2.115630 0.003793 -0.865840
O 1.278192 -0.000005 0.028813
H -0.667520 0.003699 -1.647868
H 1.787107 0.008177 -1.920502
FREQS=   19   37   42   70   88   89  126  131  153  180  238  313  316  335  391  455  481  504  520  556  574
683  688  739  744  876  915 1013 1119 1146 1282 1381 1413 1434 1455 1511 1514 1762 1947 2073 2098 2154 2996
3020 3040 3067 3143 3144
MIN007_8
18
E= -1916.42563584 ZPE=    77.51 Gcorr    0.054825000
H 4.330331 0.780880 -0.270120
Co -0.723026 -0.019201 0.020932
C -1.966879 0.992978 0.750023
C -0.035934 1.334336 -0.897732
O -2.702324 1.640973 1.352512
O 0.313862 2.139176 -1.639374
C -1.836865 -1.448336 -0.163000
O -2.526636 -2.313067 -0.454915
C 2.714508 -0.206585 0.819375
C 4.196899 0.074541 0.557215
H 2.601444 -0.894299 1.666228
H 2.198857 0.722055 1.100410
H 4.681262 0.503095 1.439824
H 4.735159 -0.843835 0.297361
```

C 2.002488 -0.816202 -0.392013
O 0.655916 -1.149945 -0.121660
H 2.517489 -1.743141 -0.685996
H 2.085325 -0.127800 -1.248595
FREQS= 28 35 56 80 89 100 113 127 229 258 280 312 373 392 415 451 472 503 517 567 593 657 761 893 896 1037 1095 1126 1192 1277 1319 1331 1407 1426 1500 1513 1514 1527 2099 2115 2181 2980 3016 3029 3040 3074 3108 3117
MIN008_8
18
E= -1916.41572765 ZPE= 74.18 Gcorr 0.046384000
H -4.802917 -0.478237 0.963410
Co 0.679037 0.006893 0.109128
C 0.623105 -1.676416 0.591658
C 1.816163 -0.145923 -1.261604
O 0.648184 -2.736347 1.052068
O 2.597181 -0.269212 -2.095421
C 1.512564 1.274866 0.989995
O 2.071589 2.010936 1.681788
C -2.814543 -0.000534 0.237183
C -4.339404 0.035589 0.117956
H -2.470488 0.464575 1.171608
H -2.430773 -1.029138 0.279240
H -4.715069 1.063864 0.107937
H -4.681193 -0.456987 -0.797969
C -2.103797 0.693973 -0.886550
O -0.886575 0.728972 -1.030162
H -0.116138 0.074674 1.372862
H -2.724972 1.211009 -1.641539
FREQS= 14 31 48 60 79 82 96 125 199 217 251 315 335 336 387 452 481 497 523 552 558 573 655 738 750 887 918 1047 1130 1144 1277 1337 1432 1438 1444 1516 1517 1751 1970 2072 2100 2154 2985 3036 3054 3065 3122 3138
MIN009_8
18
E= -1916.41660937 ZPE= 74.40 Gcorr 0.047414000
H -4.261657 1.446325 0.203995
Co 0.810709 0.017315 -0.147592
C 1.164424 1.670827 -0.611763
C 1.563781 0.035978 1.469335
O 1.472503 2.701440 -1.032620
O 2.108788 0.057567 2.481292
C 1.584834 -1.426888 -0.771825
O 2.150133 -2.290009 -1.291043
C -3.514149 -0.601381 0.382890
C -4.527290 0.438964 -0.128753
H -3.440673 -0.600707 1.473794
H -3.837664 -1.604505 0.066816
H -4.577464 0.443936 -1.222282
H -5.525801 0.209513 0.251091
C -2.149481 -0.383501 -0.192945
O -1.138640 -0.279077 0.492036
H 0.351369 0.028145 -1.573744
H -2.072246 -0.313574 -1.293631
FREQS= 25 38 47 67 75 89 92 142 150 194 232 320 334 347 389 453 482 502 516 548 556 575 742 751 770 889 935 1018 1134 1171 1276 1333 1416 1430 1475 1514 1519 1761 1953 2072 2099 2154 2989 3025 3057 3114 3133 3141
MIN010_8
18
E= -1916.41990179 ZPE= 74.24 Gcorr 0.050986000
H 4.911980 -0.984902 0.000274
Co -0.899233 -0.014334 0.000021
C -1.147903 0.851401 -1.534211
C -1.241748 -1.772306 0.001101
O -1.465817 1.395314 -2.501754
O -1.626762 -2.858391 0.001755
C -1.147802 0.853363 1.533152
O -1.465659 1.398516 2.500018
C 3.384262 0.546646 -0.000183
C 3.821435 -0.916144 0.000248
H 3.780956 1.094662 0.868597
H 3.780974 1.094157 -0.869272
H 3.443530 -1.438675 0.882484
H 3.443540 -1.439190 -0.881687
C 1.906355 0.765462 -0.000254
O 1.093408 -0.154290 0.000033
H -2.357545 0.032532 0.000067
H 1.546915 1.808726 -0.000571

FREQS=   16   48   48   65   65   79 134 139 160 200 240 303 335 359 385 448 448 459 512 519 528
687 700 724 731 879 922 1015 1121 1145 1281 1382 1410 1434 1453 1511 1514 1755 2065 2070 2079 2150 3007
3024 3039 3069 3145 3145
MIN011_8
18
E= -1916.41402490 ZPE=   74.21 Gcorr   0.046573000
H -4.018689 0.519359 -1.240526
Co 0.780621 0.036655 -0.215079
C 0.508278 1.743827 -0.469595
C 0.761344 0.034511 1.560995
O 0.475603 2.876632 -0.699108
O 0.809657 0.079340 2.710844
C 2.280232 -0.847355 -0.466249
O 3.319733 -1.293980 -0.691317
C -2.726364 -0.211713 0.362090
C -4.106990 0.099003 -0.234554
H -2.087833 0.674738 0.420973
H -2.845525 -0.590767 1.389344
H -4.728974 -0.800069 -0.295196
H -4.632568 0.826882 0.387925
C -1.992946 -1.275881 -0.395835
O -0.793687 -1.277846 -0.639944
H 1.000130 0.185139 -1.686502
H -2.595065 -2.132485 -0.753788
FREQS=   13   44   46   58   73   90 100 111 190 204 246 319 336 353 383 457 481 502 520 527 557
568 700 724 736 880 925 1027 1133 1163 1273 1331 1426 1440 1465 1513 1519 1767 1969 2072 2094 2150 2979
3017 3056 3105 3129 3141
MIN012_8
18
E= -1916.41586641 ZPE=   74.11 Gcorr   0.050152000
H 5.126080 0.006995 -0.937451
Co -0.797365 0.000877 -0.218935
C -1.441801 -1.573679 -0.642292
C -1.200044 -0.005002 1.510093
O -1.958694 -2.538634 -1.009696
O -1.548720 -0.008703 2.608506
C -1.441971 1.578220 -0.631618
O -1.958976 2.545556 -0.992527
C 3.568927 -0.004311 0.566126
C 4.034373 0.006698 -0.887885
H 3.954414 -0.877899 1.114247
H 3.954390 0.860877 1.127427
H 3.665086 -0.870816 -1.424372
H 3.665223 0.892311 -1.410993
C 2.082433 -0.005731 0.748860
O 1.288853 0.001167 -0.183256
H -0.735645 0.006072 -1.711575
H 1.708090 -0.013491 1.790050
FREQS=   19   34   41   67   81   87 124 128 148 166 237 311 319 339 375 458 480 503 519 558 563
683 687 707 728 874 913 1012 1119 1145 1281 1379 1417 1434 1456 1510 1514 1767 1971 2076 2091 2148 2975
3018 3040 3068 3143 3145
MIN013_8
18
E= -1916.41219085 ZPE=   74.11 Gcorr   0.046952000
H 3.963645 1.227964 -0.976801
Co -0.629753 0.038583 -0.132376
C -1.546780 -0.984491 -1.221057
C -1.657068 -0.143181 1.314639
O -2.179944 -1.523675 -2.021986
O -2.386215 -0.212118 2.201075
C -0.496657 1.784209 -0.134799
O -0.481924 2.930311 -0.280226
C 3.376116 -0.532691 0.126974
C 3.047603 0.773256 -0.590507
H 3.883021 -1.246374 -0.542278
H 4.095253 -0.377288 0.945764
H 2.366825 0.607539 -1.427703
H 2.573267 1.489189 0.085351
C 2.223473 -1.284851 0.726634
O 1.037306 -1.011950 0.639047
H 0.018788 0.307617 -1.452508
H 2.508630 -2.184449 1.307731
FREQS=   24   39   44   70   76   83 102 111 186 198 240 254 322 339 377 454 484 496 520 555 572
654 678 705 748 852 908 1016 1119 1142 1287 1384 1430 1441 1460 1510 1514 1791 1970 2072 2099 2154 2955
3013 3036 3068 3139 3153
MIN014_8
18

E= -1916.42254483 ZPE= 77.78 Gcorr 0.057604000
H 4.655587 -0.440031 0.001609
Co -0.550892 0.027962 0.082205
C -1.067632 1.504197 -0.740600
C -0.292061 -1.442537 -0.855546
O -1.400823 2.385024 -1.408828
O -0.189268 -2.309286 -1.614039
C -2.122103 -0.405898 0.830846
O -3.105755 -0.682718 1.358011
C 2.497189 -0.279481 -0.273403
C 3.825849 0.184971 0.345424
H 2.555842 -0.226271 -1.366603
H 2.318681 -1.338096 -0.037175
H 4.047328 1.220823 0.068779
H 3.798662 0.134319 1.439023
C 1.308118 0.546633 0.162361
O 0.932914 0.373271 1.539800
H 1.230933 -0.498211 1.842244
H 1.474352 1.614833 0.018543
FREQS= 48 58 74 83 87 95 128 188 225 289 290 300 390 416 444 470 516 522 538 553 560 594 670 777 871 959 1046 1070 1122 1158 1247 1320 1344 1416 1426 1487 1512 1517 2062 2085 2147 3025 3046 3081 3118 3123 3127 3784
MIN015_8
18
E= -1916.42228931 ZPE= 77.74 Gcorr 0.057511000
H -4.634850 -0.494472 -0.051374
Co 0.542910 0.030814 -0.078664
C 1.072356 1.444204 0.829039
C 0.280474 -1.497262 0.776109
O 1.418357 2.273758 1.556459
O 0.175002 -2.409762 1.475527
C 2.105217 -0.376867 -0.858151
O 3.083877 -0.629324 -1.406696
C -2.481342 -0.320362 0.243256
C -3.814446 0.168440 -0.343137
H -2.542360 -0.358093 1.337557
H -2.277993 -1.342417 -0.094518
H -4.059458 1.176743 0.009212
H -3.773859 0.191428 -1.436519
C -1.312693 0.566330 -0.117544
O -0.950265 0.414369 -1.504520
H -0.775521 1.279391 -1.899940
H -1.507693 1.616214 0.115902
FREQS= 46 59 74 83 87 95 125 190 227 289 295 301 389 410 445 471 515 520 538 551 562 584 664 774 884 955 1036 1078 1102 1136 1247 1311 1349 1417 1429 1481 1511 1519 2064 2085 2146 3043 3052 3084 3097 3117 3125 3819
MIN016_8
18
E= -1916.41901524 ZPE= 76.46 Gcorr 0.054421000
H 4.659421 -0.502896 -0.872648
Co -0.550379 0.002912 -0.002908
C -1.480913 -0.786654 -1.282354
C -0.454306 -1.427133 1.095459
O -2.065992 -1.297728 -2.128243
O -0.457958 -2.235632 1.909522
C -1.615490 1.447335 0.145653
O -2.290933 2.340116 0.397539
C 2.853449 0.098142 0.182714
C 3.702627 -0.017192 -1.084402
H 2.644932 -0.891303 0.604692
H 3.392768 0.660086 0.954370
H 3.195855 -0.609291 -1.855585
H 3.919426 0.968327 -1.511455
C 1.521868 0.820179 -0.037794
O 0.718300 0.876860 1.077021
H 0.986700 0.212477 -0.895338
H 1.670380 1.806998 -0.507870
FREQS= 42 44 66 86 89 93 120 148 226 264 288 315 380 407 438 447 469 490 528 558 569 611 742 874 897 1040 1092 1122 1172 1285 1304 1334 1398 1429 1497 1513 1517 1559 2112 2115 2175 2237 2978 3039 3053 3087 3110 3122
MIN017_8
18
E= -1916.42261230 ZPE= 77.89 Gcorr 0.058050000
H 4.205799 -0.369118 -0.657838
Co -0.519930 0.015873 0.115861
C -0.850224 1.545096 -0.704995
C 0.082190 -1.356911 -0.811919

O -1.019301 2.475452 -1.367815
O 0.446067 -2.152456 -1.568755
C -2.217218 -0.524948 0.323702
O -3.296748 -0.873154 0.511940
C 2.510068 -0.160737 0.685623
C 3.236129 0.135282 -0.630695
H 2.336162 -1.242649 0.775296
H 3.153692 0.129908 1.530205
H 2.651227 -0.205688 -1.488883
H 3.414845 1.209311 -0.747783
C 1.197513 0.581928 0.801677
O 0.433008 0.261307 1.973780
H 0.684779 -0.621258 2.285574
H 1.336650 1.663464 0.801629
FREQS= 43 53 77 85 92 110 123 187 233 292 300 337 403 411 450 478 505 520 535 546 560 583 598 824 887 996 1046 1075 1109 1183 1251 1309 1354 1410 1432 1499 1513 1520 2060 2085 2146 3000 3038 3054 3123 3127 3138 3786
MIN018_8
18
E= -1916.41897415 ZPE= 76.56 Gcorr 0.054865000
H 4.195959 1.213104 -0.534640
Co -0.591913 0.010104 -0.032941
C -0.378631 -1.480027 0.963121
C -1.365387 1.565488 0.445450
O -0.254979 -2.326327 1.727920
O -1.814834 2.518369 0.899994
C -1.947287 -0.586131 -0.999212
O -2.813404 -0.973210 -1.646475
C 2.694633 -0.337113 -0.851298
C 3.925116 0.228502 -0.137090
H 2.894168 -0.451391 -1.924798
H 2.454742 -1.331554 -0.457475
H 3.727761 0.338656 0.932317
H 4.788354 -0.431978 -0.263432
C 1.473238 0.566331 -0.658335
O 1.039751 0.655627 0.645841
H 0.633016 0.096251 -1.335500
H 1.625290 1.556142 -1.121129
FREQS= 44 46 73 80 87 95 129 146 230 232 287 349 401 407 446 457 469 482 526 559 569 611 785 870 895 997 1100 1111 1164 1291 1307 1354 1399 1422 1490 1510 1522 1559 2112 2115 2175 2256 2979 3037 3047 3082 3115 3138
MIN019_8
18
E= -1916.42183059 ZPE= 77.81 Gcorr 0.057968000
H 2.636225 -0.335538 -1.458561
Co -0.512541 0.023327 0.114391
C -0.837739 1.465082 -0.840044
C 0.095852 -1.432300 -0.692806
O -1.002576 2.327954 -1.592056
O 0.456822 -2.295028 -1.370286
C -2.207794 -0.506462 0.355754
O -3.289055 -0.836983 0.565946
C 2.493977 -0.118353 0.707351
C 3.225424 0.059864 -0.626584
H 2.293248 -1.178958 0.895128
H 3.138203 0.228402 1.529272
H 3.428862 1.116909 -0.831204
H 4.184409 -0.465468 -0.612615
C 1.197671 0.656892 0.758935
O 0.447027 0.349282 1.947170
H 0.094015 1.160752 2.337329
H 1.361543 1.733935 0.670277
FREQS= 40 54 78 86 95 111 125 188 231 296 301 340 403 406 452 480 508 518 535 543 560 575 583 816 892 994 1032 1075 1081 1166 1246 1302 1338 1420 1432 1500 1514 1521 2062 2083 2146 3012 3050 3075 3094 3119 3135 3817
MIN020_8
18
E= -1916.41879515 ZPE= 76.58 Gcorr 0.055343000
H 2.628824 1.350702 0.867399
Co -0.503226 0.001414 0.023432
C -1.579630 0.950440 1.057584
C 0.020699 1.369548 -1.024917
O -2.259642 1.563145 1.751146
O 0.295766 2.144132 -1.825848
C -1.702119 -1.284279 -0.379912
O -2.425879 -2.069423 -0.799750
C 2.788849 -0.820452 0.748142

C 3.150038 0.586714 0.278183
H 3.361680 -1.561617 0.177422
H 3.055300 -0.959192 1.803693
H 4.224105 0.768354 0.379422
H 2.878777 0.722240 -0.771592
C 1.310378 -1.186757 0.564269
O 0.850887 -1.066671 -0.728090
H 0.726956 -0.445268 1.267121
H 1.093121 -2.174136 1.004103
FREQS= 44 56 65 86 90 92 129 155 241 253 290 326 390 413 435 455 467 489 525 564 569 622 779 862 897 1002 1094 1129 1149 1301 1312 1349 1391 1429 1491 1507 1523 1540 2110 2114 2174 2262 2982 3041 3044 3080 3112 3141
MIN021_8
18
E= -1916.41559473 ZPE= 74.97 Gcorr 0.052305000
H 3.868471 -1.556161 -0.091121
Co -0.535817 0.002422 -0.121580
C -1.266834 -0.063061 1.541800
C -0.503197 1.740450 -0.367573
O -1.757876 -0.098363 2.574756
O -0.472069 2.862159 -0.624101
C -1.773012 -0.917224 -1.035229
O -2.534397 -1.436905 -1.717754
C 2.575160 0.163656 -0.449400
C 3.917725 -0.465085 -0.032637
H 2.653317 1.256315 -0.419342
H 2.321030 -0.131085 -1.471639
H 4.720848 -0.122242 -0.691720
H 4.187515 -0.191658 0.993213
C 1.477496 -0.299304 0.482675
O 0.842655 -1.386908 0.281284
H -0.023036 0.122171 -1.505824
H 1.548396 0.080821 1.517786
FREQS= 49 56 69 77 80 89 119 169 210 232 325 358 381 409 428 460 490 495 508 527 544 549 742 796 834 883 952 1004 1092 1156 1284 1335 1373 1417 1486 1511 1519 1558 2011 2110 2138 2179 2969 3047 3058 3107 3123 3134
MIN022_8
18
E= -1916.41561188 ZPE= 74.91 Gcorr 0.052642000
H -2.722466 1.070116 0.643662
Co 0.563076 0.069863 -0.176359
C 0.113127 1.744269 0.097014
C 0.285078 -0.601857 1.486266
O -0.116626 2.866788 0.208399
O 0.149699 -1.010502 2.547055
C 2.323866 -0.240409 -0.338843
O 3.452816 -0.340567 -0.512843
C -2.440353 -0.891253 -0.270328
C -3.262085 0.371571 -0.002901
H -2.170710 -1.403953 0.658563
H -3.052138 -1.605287 -0.844039
H -3.498698 0.895731 -0.935180
H -4.208065 0.123858 0.486347
C -1.199601 -0.671692 -1.109441
O -0.232857 -1.502587 -1.108888
H 0.848365 0.698335 -1.489353
H -1.343783 -0.033940 -1.998700
FREQS= 42 57 68 83 84 108 118 168 222 271 329 353 375 407 425 457 481 496 512 514 529 551 744 772 826 895 934 1038 1098 1157 1277 1336 1373 1430 1479 1512 1514 1554 1998 2110 2136 2177 2987 3009 3050 3092 3120 3129
MIN023_8
18
E= -1916.41456101 ZPE= 74.79 Gcorr 0.051651000
H 3.057229 1.648488 -0.599606
Co -0.431896 0.063521 0.085308
C -1.337332 1.582125 0.354049
C -1.628316 -0.831386 -0.945118
O -1.867907 2.557786 0.643399
O -2.416755 -1.383129 -1.565192
C -0.300902 -1.060057 1.424899
O -0.180157 -1.719737 2.361344
C 2.706541 -0.477180 -0.339542
C 3.162217 0.886080 0.175904
H 3.400200 -0.824044 -1.122834
H 2.751525 -1.245045 0.441681
H 2.572843 1.206260 1.039451
H 4.211358 0.843393 0.481848

C 1.344426 -0.512843 -1.002893
O 0.772548 0.525968 -1.457737
H 1.070622 -1.494394 -1.428140
H 0.455768 0.782733 1.025974
FREQS= 37 55 66 79 80 91 121 167 209 256 298 322 368 405 427 462 467 495 509 533 550 637 733 760 810 849 933 1018 1108 1113 1285 1372 1378 1424 1476 1505 1517 1574 2024 2106 2135 2176 2974 3004 3061 3073 3129 3143
MIN024_8
18
E= -1916.41550232 ZPE= 75.01 Gcorr 0.052669000
H -3.814198 -1.455097 -0.473746
Co 0.613224 0.031766 -0.182738
C 0.734509 1.780220 -0.076863
C 0.399836 -0.372343 1.574211
O 0.856139 2.924695 -0.085166
O 0.317805 -0.628056 2.687011
C 2.142521 -0.850505 -0.506607
O 3.144031 -1.326595 -0.798870
C -2.501946 0.157674 0.188774
C -3.849479 -0.370292 -0.339112
H -2.568372 1.239521 0.350901
H -2.268345 -0.315982 1.147376
H -4.654191 -0.137051 0.364353
H -4.104616 0.083551 -1.302589
C -1.404163 -0.145054 -0.808525
O -0.779239 -1.257248 -0.802340
H 0.885133 0.415781 -1.589422
H -1.470021 0.411050 -1.759278
FREQS= 49 59 71 77 88 93 121 163 212 231 327 356 377 413 428 458 489 496 507 529 547 551 745 797 828 888 956 1002 1091 1155 1292 1337 1372 1419 1484 1511 1518 1554 2000 2111 2137 2177 2990 3049 3055 3104 3123 3134
MIN025_8
18
E= -1916.41549229 ZPE= 74.92 Gcorr 0.052682000
H 2.891733 1.204039 -0.396893
Co -0.471133 0.013974 -0.114355
C 0.117300 1.666408 -0.122000
C -1.898254 -0.302593 -1.152071
O 0.478916 2.754436 -0.230805
O -2.753479 -0.437175 -1.904249
C -1.257423 -0.016480 1.524694
O -1.775769 -0.019360 2.544794
C 2.520011 -0.946774 -0.425509
C 3.391947 0.272315 -0.118527
H 2.228405 -0.985522 -1.479235
H 3.098560 -1.863604 -0.230009
H 3.635202 0.328428 0.948455
H 4.335639 0.221906 -0.668670
C 1.283040 -1.075513 0.435542
O 0.303786 -1.814333 0.092055
H 1.435140 -0.868280 1.510545
H 0.128558 0.133024 -1.463749
FREQS= 37 63 70 81 92 105 116 178 230 263 323 354 375 407 424 457 472 494 511 515 530 551 750 766 828 895 924 1040 1098 1156 1279 1337 1373 1431 1475 1514 1518 1557 2014 2106 2138 2178 2966 3008 3051 3099 3121 3136
MIN026_8
18
E= -1916.42029666 ZPE= 77.67 Gcorr 0.058340000
H -4.156625 0.108564 -0.398679
Co 0.430026 -0.013348 -0.013742
C 1.826037 0.090925 1.096543
C 0.842479 -1.594817 -0.677530
O 2.708680 0.155101 1.830668
O 1.134461 -2.552651 -1.251468
C 0.696483 1.537538 -0.809216
O 0.935206 2.446141 -1.482501
C -1.339722 -0.140714 -0.963089
C -3.517896 -0.197562 0.436428
H -1.611542 -1.166507 -1.224239
H -1.487262 0.490500 -1.840019
H -3.497087 -1.290598 0.474082
H -3.972321 0.173845 1.362502
C -2.114315 0.352492 0.245333
O -1.247528 -0.075426 1.361480
H -2.135008 1.450420 0.247518
H -1.355803 0.521688 2.113625

FREQS=   47   60   76   79 100 103 152 220 250 298 311 399 404 415 433 469 505 511 516 538 570
574 625 822 839 926 987 1057 1100 1145 1178 1269 1380 1384 1422 1499 1500 1506 2064 2087 2148 3032 3040
3085 3110 3131 3149 3819
MIN027_8
18
E= -1916.41730108 ZPE=   76.67 Gcorr   0.055422000
H -3.956754 0.033443 -1.664712
Co 0.450039 -0.100741 -0.306311
C -0.016426 -1.394929 0.740667
C 2.142228 -0.691183 -0.642889
O -0.325769 -2.187719 1.514380
O 3.131420 -1.171019 -0.964183
C 0.939767 1.578618 0.607135
O 1.089080 1.921713 1.732160
C -2.655346 -0.312601 0.056847
C -3.949583 0.210942 -0.583454
H -2.703583 -0.146826 1.140659
H -2.602886 -1.399052 -0.084450
H -4.829053 -0.283279 -0.157390
H -4.060683 1.289056 -0.424167
C -1.411950 0.365767 -0.503585
O 0.919176 1.823357 -0.604486
H -1.502809 1.455107 -0.418531
H -1.298665 0.141217 -1.592314
FREQS=   38   59   74   82   87 110 125 178 248 256 270 286 340 358 426 446 495 543 556 564 591
645 680 792 916 965 1044 1102 1177 1210 1251 1330 1366 1422 1434 1500 1511 1519 2082 2112 2162 2845 3034
3038 3062 3078 3110 3113
MIN028_8
18
E= -1916.41333263 ZPE=   74.83 Gcorr   0.051565000
H -2.948097 -1.718884 -0.141599
Co 0.577589 0.096647 -0.164060
C 0.613475 1.747727 0.427512
C 0.126958 -0.746845 1.369531
O 0.707506 2.846946 0.758764
O -0.038726 -1.266769 2.377585
C 2.121838 -0.747070 -0.474872
O 3.161433 -1.170881 -0.713904
C -2.645766 0.387392 -0.569807
C -3.068948 -0.752577 0.354050
H -3.370676 0.477146 -1.395359
H -2.674885 1.358769 -0.062401
H -2.482368 -0.772817 1.275184
H -4.118820 -0.638136 0.637642
C -1.311394 0.222135 -1.268749
O -0.712816 -0.888607 -1.370008
H 1.124323 0.839698 -1.328882
H -1.080538 1.014680 -2.000446
FREQS=   26   59   67   78   89 103 118 153 212 250 294 332 367 401 431 455 468 497 512 535 553
640 741 755 804 852 936 1021 1107 1114 1286 1372 1385 1427 1474 1510 1522 1586 1987 2105 2127 2170 2989
3006 3068 3074 3137 3150
MIN029_8
18
E= -1916.42026271 ZPE=   77.77 Gcorr   0.058612000
H 3.948431 -0.196777 1.366309
Co -0.426435 0.017039 -0.015097
C -0.865989 1.572646 -0.707130
C -0.698231 -1.545354 -0.792886
O -1.179259 2.508396 -1.308425
O -0.938235 -2.465323 -1.448277
C -1.801463 -0.089022 1.127043
O -2.664900 -0.151671 1.882336
C 2.101648 -0.353843 0.237231
C 3.511376 0.177609 0.434789
H 1.477241 -0.486073 -1.853455
H 2.110399 -1.447818 0.257745
H 4.149655 -0.136458 -0.396823
H 3.517699 1.274689 0.456462
C 1.335849 0.143871 -0.973925
O 1.251482 -0.013220 1.401189
H 1.447412 0.901934 1.649515
H 1.611062 1.169557 -1.237451
FREQS=   49   61   76   77   97 102 150 219 256 302 312 404 405 417 460 475 510 512 536 544 570
573 621 810 844 915 982 1059 1080 1158 1182 1280 1374 1395 1422 1500 1502 1505 2064 2088 2151 3034 3070
3084 3110 3127 3141 3796
MIN030_8
18

E= -1916.41030578 ZPE=   74.24 Gcorr   0.048745000
H 3.748085 1.372092 -0.334748
Co -0.511283 0.017935 -0.089700
C -0.311095 -1.526112 -0.920709
C -2.057425 -0.349139 0.736347
O -0.199225 -2.457688 -1.589499
O -3.064937 -0.588988 1.227591
C -0.972911 1.598068 -0.717875
O -1.261261 2.574915 -1.257790
C 2.728931 -0.270921 0.676059
C 3.517069 0.313787 -0.497521
H 2.461580 -1.319900 0.515533
H 3.350466 -0.248197 1.585403
H 4.464649 -0.215463 -0.627057
H 2.954147 0.232401 -1.432057
C 1.486595 0.497662 1.051434
O 0.565290 -0.002986 1.724865
H 0.629022 0.316910 -1.014203
H 1.530782 1.595832 0.921291
FREQS=   15   46   73   76   81   97 115 124 194 238 318 332 336 373 392 429 465 475 503 519 552
570 666 729 758 895 917 1040 1107 1157 1277 1338 1405 1431 1463 1512 1517 1656 1980 2087 2118 2170 2966
3013 3052 3100 3123 3134
MIN031_8
18
E= -1916.41960683 ZPE=   77.94 Gcorr   0.058113000
H -2.557702 -1.443780 0.263563
Co 0.474946 0.024894 -0.083752
C -0.168279 -1.128863 1.067998
C 1.811154 -0.985116 -0.725565
O -0.495142 -1.798131 1.954504
O 2.642530 -1.630908 -1.188599
C 1.440279 1.371499 0.556947
O 2.046462 2.166972 1.133233
C -2.565749 0.747452 0.090436
C -3.101464 -0.648855 -0.249608
H -3.223261 1.494839 -0.378464
H -2.641374 0.915866 1.170671
H -4.152087 -0.720745 0.044975
H -3.077354 -0.859571 -1.328156
C -1.135841 1.073120 -0.311880
O -0.766918 0.674444 -1.640380
H -1.316744 -0.077587 -1.908303
H -0.951088 2.144400 -0.243017
FREQS=   39   57   78   83   86 112 117 206 246 286 302 320 390 422 468 489 515 520 537 558 560
601 633 788 842 960 1055 1077 1123 1175 1235 1311 1378 1405 1441 1488 1513 1525 2057 2083 2144 3012 3022
3082 3107 3134 3157 3782
MIN032_8
18
E= -1916.40997811 ZPE=   74.40 Gcorr   0.048522000
H -4.281532 -1.262655 0.335482
Co 0.594191 -0.005892 -0.120746
C 0.574930 1.627158 -0.791054
C 1.814353 0.273927 1.161385
O 0.622653 2.633142 -1.350398
O 2.623750 0.461462 1.951032
C 1.287755 -1.497653 -0.752508
O 1.763500 -2.403025 -1.284145
C -2.720149 0.122485 -0.343404
C -4.048682 -0.193353 0.373455
H -2.792180 -0.154389 -1.400176
H -2.497415 1.191195 -0.275990
H -4.004429 0.109596 1.423124
H -4.871086 0.344897 -0.105557
C -1.607431 -0.655417 0.312015
O -0.965503 -0.216647 1.286917
H -0.201921 -0.198325 -1.374119
H -1.554450 -1.733586 0.070789
FREQS=   16   45   60   76   84   91 107 126 161 229 318 320 339 387 428 442 464 477 502 507 552
570 665 753 804 883 937 1000 1097 1161 1282 1331 1403 1421 1488 1512 1519 1657 1977 2088 2118 2170 2972
3052 3067 3115 3130 3140
MIN033_8
18
E= -1916.41169489 ZPE=   76.92 Gcorr   0.051596000
H -3.552789 -2.359277 1.026882
Co 0.062157 0.522980 -0.075285
C -1.523474 0.894944 -0.493310
C 0.524501 2.189945 0.277984

O -2.599753 1.133292 -0.846332
O 0.733673 3.267748 0.634157
C 2.721357 -1.049739 -0.060626
O 3.456516 -1.942283 -0.153739
C -1.787927 -2.036201 -0.215640
C -2.593356 -1.833113 1.071077
H -1.667669 -3.117874 -0.378792
H -2.370235 -1.672531 -1.071209
H -2.045602 -2.214669 1.941238
H -2.804224 -0.774314 1.251642
C -0.405736 -1.377204 -0.197429
O 2.016938 -0.112170 0.035342
H 0.159293 -1.730464 -1.079451
H 0.131820 -1.755834 0.694624
FREQS= 19 24 41 69 84 92 124 135 179 210 225 296 336 398 422 433 480 523 594 610 625
647 649 801 899 1003 1034 1089 1205 1237 1316 1373 1379 1426 1446 1504 1514 1520 2064 2125 2444 2916 2971
3005 3037 3059 3103 3117
MIN034_8
18
E= -1916.41554182 ZPE= 76.67 Gcorr 0.055735000
H 2.829370 1.373282 -0.193526
Co -0.334035 0.107662 -0.276648
C 0.511497 0.922173 0.986802
C -1.667403 1.332726 -0.475011
O 1.029951 1.374337 1.909398
O -2.374481 2.205167 -0.702294
C -1.480235 -1.381828 0.345199
O -1.821686 -1.791868 1.403672
C 2.662537 -0.813375 -0.114391
C 3.334356 0.482254 -0.580572
H 3.256836 -1.659993 -0.488054
H 2.712900 -0.887150 0.979677
H 4.375209 0.528758 -0.245361
H 3.334724 0.550476 -1.674743
C 1.223651 -1.002582 -0.586862
O -1.466414 -1.441105 -0.887514
H 0.919976 -2.049038 -0.471519
H 1.144541 -0.771668 -1.674088
FREQS= 41 55 67 87 93 112 138 185 226 249 285 307 346 398 413 443 492 504 552 570 590
645 678 787 903 972 1058 1091 1195 1210 1237 1310 1377 1414 1435 1499 1515 1516 2087 2109 2159 2875 3013
3041 3054 3077 3108 3118
MIN035_8
18
E= -1916.41565049 ZPE= 76.86 Gcorr 0.055877000
H -4.170168 -0.343666 0.497321
Co 0.506973 0.119107 -0.365981
C 0.543552 1.618481 0.498261
C -0.064524 -1.440239 0.682360
O 0.515127 2.577560 1.132483
O -0.270123 -1.669624 1.828490
C 2.291385 -0.230573 -0.271022
O 3.431542 -0.312660 -0.347595
C -2.513564 -0.087716 -0.895980
C -3.207000 0.173879 0.446652
H -2.343710 -1.160659 -1.027287
H -3.193854 0.212297 -1.707310
H -2.597356 -0.172309 1.287131
H -3.396036 1.243775 0.590659
C -1.214569 0.695738 -1.064247
O -0.026487 -1.804313 -0.499789
H -0.771859 0.535317 -2.075479
H -1.427442 1.764252 -0.968413
FREQS= 28 58 74 82 90 105 142 199 232 255 284 334 358 409 427 440 495 513 543 564 598
647 694 823 881 965 1056 1078 1170 1204 1256 1314 1369 1427 1443 1506 1514 1521 2070 2111 2160 2861 3008
3043 3082 3096 3115 3122
MIN036_8
18
E= -1916.41036171 ZPE= 74.45 Gcorr 0.050892000
H 4.686473 -0.188469 0.335668
Co -0.657554 -0.024040 -0.163923
C -2.024322 -0.541907 0.935704
C -0.029353 -1.515394 -0.909054
O -2.896025 -0.871580 1.600184
O 0.263908 -2.473575 -1.473679
C -1.046247 1.631839 -0.688078
O -1.375703 2.648864 -1.112240
C 2.552911 -0.159313 -0.098558

C 3.793246 0.354276 0.658282
H 2.703645 -0.040400 -1.177156
H 2.398861 -1.221201 0.112094
H 3.963092 1.419829 0.470664
H 3.676745 0.213219 1.736548
C 1.339300 0.628078 0.342488
O 0.623643 0.270355 1.334330
H -1.485841 -0.210646 -1.361613
H 1.371193 1.698760 0.076263
FREQS= 42 54 67 76 87 103 119 156 201 228 289 314 393 404 425 446 460 471 480 510 528 570 645 732 794 881 966 1000 1094 1157 1285 1332 1376 1419 1483 1511 1518 1561 2039 2099 2123 2174 2983 3049 3061 3111 3126 3136
MIN037_8
18
E= -1916.41066272 ZPE= 74.46 Gcorr 0.051514000
H 4.312993 -0.104610 -0.278008
Co -0.568658 -0.015257 -0.154716
C -2.188271 -0.650241 0.407936
C 0.315745 -1.414685 -0.811794
O -3.210187 -1.051574 0.731524
O 0.809929 -2.306714 -1.344314
C -0.834296 1.685314 -0.605648
O -1.051530 2.740651 -1.008306
C 2.478658 -0.108732 0.887294
C 3.309572 0.327186 -0.321917
H 2.327441 -1.191735 0.912352
H 3.021219 0.148111 1.811376
H 2.844818 0.007644 -1.258982
H 3.418311 1.416658 -0.356164
C 1.148346 0.596857 1.022913
O 0.180720 0.105822 1.688449
H -0.982046 -0.076284 -1.561906
H 1.201040 1.692468 0.897136
FREQS= 39 49 75 79 92 116 124 170 220 252 311 317 377 397 423 433 461 466 495 503 519 551 645 735 779 894 943 1035 1100 1162 1275 1336 1377 1431 1481 1513 1516 1562 2044 2097 2122 2174 2979 3006 3052 3102 3123 3134
MIN038_8
18
E= -1916.41543970 ZPE= 77.18 Gcorr 0.057267000
H 3.484133 -1.541256 0.645634
Co -0.358788 -0.037135 0.049743
C -1.821576 -0.714653 0.834567
C -0.785700 1.682172 0.154329
O -2.763976 -1.145713 1.333591
O -1.105186 2.785924 0.056774
C -0.500680 -0.894036 -1.483333
O -0.643454 -1.330083 -2.543810
C 1.660753 -0.414194 0.949259
C 2.427265 -1.735475 0.851511
H 0.622002 -0.667342 1.383780
H 2.068653 0.243330 1.721706
H 2.361344 -2.304832 1.783490
H 2.032628 -2.362334 0.046503
C 1.535163 0.370599 -0.321828
O 2.033420 1.671424 -0.173699
H 1.911645 2.140076 -1.009842
H 1.949087 -0.123878 -1.204200
FREQS= 46 57 76 88 93 101 147 212 260 311 318 347 365 399 420 454 472 505 512 534 548 553 616 761 891 1023 1046 1077 1137 1174 1253 1290 1348 1426 1447 1507 1512 1579 2070 2089 2147 2460 3052 3084 3100 3126 3131 3823
MIN039_8
18
E= -1916.40848200 ZPE= 74.12 Gcorr 0.050507000
H 4.102051 -0.737990 0.544167
Co -0.652890 0.241173 -0.086426
C -2.085565 -0.801383 -0.211169
C -0.045277 1.904693 -0.076846
O -3.061630 -1.396214 -0.314322
O 0.260139 3.011721 -0.088724
C -0.217617 -0.175938 1.580098
O -0.013972 -0.390496 2.693542
C 2.395339 -0.471261 -0.772858
C 3.040360 -0.996823 0.511106
H 2.437314 0.620885 -0.835891
H 2.959921 -0.845126 -1.641595
H 2.565643 -0.577693 1.402600
H 2.964477 -2.087875 0.574636

C 0.973929 -0.934322 -1.006248
O 0.212179 -0.365156 -1.836847
H -1.692719 1.059620 -0.786301
H 0.750595 -1.972137 -0.697803
FREQS= 30 50 79 88 89 102 118 155 209 237 335 350 390 397 412 435 439 468 510 514 527
571 601 656 752 895 909 1040 1098 1153 1278 1336 1387 1433 1474 1514 1517 1587 1938 2102 2116 2171 2971
3011 3053 3091 3122 3136
MIN040_8
18
E= -1916.41432124 ZPE= 77.02 Gcorr 0.056356000
H 2.623291 -1.670066 0.667741
Co 0.041202 0.195263 -0.093483
C -1.009853 1.595300 -0.243907
C 0.256444 -1.073010 -1.277234
O -1.673827 2.489102 -0.542455
O 0.338498 -1.785857 -2.180532
C -1.433336 -0.767900 0.583974
O -2.499549 -1.163203 0.192625
C 2.142553 0.325614 -0.047277
C 2.655148 -0.631481 1.006895
H 1.585749 2.371282 -0.447842
H 2.559006 0.196504 -1.043870
H 3.700290 -0.390763 1.241193
H 2.084752 -0.559156 1.937939
C 1.584978 1.558299 0.271539
O -0.993402 -0.992896 1.876247
H -1.697435 -1.496827 2.325286
H 1.482542 1.858830 1.312562
FREQS= 49 54 63 86 98 102 120 164 212 260 317 338 386 390 423 463 482 505 522 548 559
661 683 788 902 944 973 1000 1022 1076 1194 1252 1278 1419 1442 1496 1506 1570 1828 2075 2127 3034 3102
3132 3141 3168 3231 3691
MIN041_8
18
E= -1916.40384631 ZPE= 73.04 Gcorr 0.046341000
H 4.310958 -0.015373 -1.283914
Co -0.675346 0.000756 0.310846
C -2.477052 0.006560 0.107028
C -0.446041 1.612554 -0.397202
O -3.608961 0.010758 -0.073677
O -0.308025 2.642380 -0.894759
C -0.457837 -1.615411 -0.390961
O -0.328957 -2.648917 -0.883370
C 2.225120 -0.018352 -0.696980
C 3.716701 0.002214 -0.365650
H 1.941532 0.835582 -1.324962
H 1.958298 -0.904858 -1.286409
H 3.981231 0.900002 0.198875
H 3.995572 -0.861967 0.242546
C 1.325100 -0.002017 0.535344
O 1.776804 0.009269 1.652753
H -0.759045 -0.400080 1.952346
H -0.757029 0.405094 1.951641
FREQS= 10 43 51 87 88 102 108 196 205 261 262 329 356 366 426 443 469 478 517 533 550
557 573 610 683 785 874 907 1014 1077 1098 1284 1356 1421 1423 1459 1505 1512 1810 2087 2109 2163 3048
3060 3079 3133 3141 3443
MIN042_8
18
E= -1916.41467508 ZPE= 77.22 Gcorr 0.057394000
H -3.595311 0.370447 1.286897
Co 0.410930 0.016435 0.077326
C 1.779121 1.043574 0.612440
C 0.113506 0.774179 -1.501510
O 2.663336 1.699624 0.945710
O 0.010540 1.132280 -2.592918
C 1.239100 -1.535368 0.156082
O 1.785736 -2.549973 0.070011
C -1.470810 -0.032400 1.290752
C -2.658630 0.933543 1.310527
H -0.536410 0.639902 1.417484
H -1.437351 -0.646531 2.193876
H -2.640898 1.546205 2.216321
H -2.646657 1.592977 0.441249
C -1.341652 -0.894688 0.065787
O -2.300943 -0.596189 -0.906684
H -1.297699 -1.965710 0.272347
H -2.173931 -1.200006 -1.649405

FREQS= 48 58 81 88 90 100 162 198 264 285 314 321 347 402 414 464 497 507 527 545 552
589 593 764 875 1007 1019 1076 1137 1188 1261 1311 1363 1424 1448 1504 1507 1581 2070 2088 2146 2419 3063
3089 3106 3136 3157 3833
MIN043_8
18
E= -1916.39871254 ZPE= 73.37 Gcorr 0.041770000
H 3.068384 0.421677 1.445358
Co -0.684220 0.222063 0.138397
C -1.574746 1.404355 -0.805119
C -1.456597 -1.268552 -0.456011
O -2.201785 2.235812 -1.296687
O -2.027464 -2.202448 -0.798191
C -0.522366 -0.199152 1.836481
O -0.511080 -0.355814 2.977096
C 3.020660 0.286281 -0.734063
C 3.050236 1.087258 0.577568
H 3.044643 0.980694 -1.585614
H 3.880651 -0.384623 -0.816083
H 3.945273 1.713846 0.612495
H 2.176690 1.739492 0.666711
C 1.777069 -0.553495 -0.883651
O 1.740610 -1.750432 -1.015007
H -0.216448 1.517224 0.727489
H 0.806952 0.058881 -0.935997
FREQS= 5 31 35 46 71 77 94 103 106 202 237 310 328 344 400 448 479 490 508 524 565
582 624 765 786 887 943 1016 1118 1161 1277 1316 1347 1425 1476 1510 1517 1843 1975 2093 2121 2175 2489
3034 3057 3111 3136 3136
MIN044_8
18
E= -1916.41346722 ZPE= 77.26 Gcorr 0.057167000
H 3.547182 -1.411870 0.594910
Co -0.349985 -0.043073 0.056832
C -1.778101 -0.808222 0.831250
C -0.882822 1.639708 0.160090
O -2.699099 -1.292937 1.320490
O -1.283945 2.718331 0.065553
C -0.450972 -0.864160 -1.502302
O -0.582471 -1.280546 -2.570588
C 1.677788 -0.378375 0.952594
C 2.503532 -1.660507 0.808519
H 0.657348 -0.696353 1.376482
H 2.067121 0.260967 1.750603
H 2.474642 -2.262333 1.721875
H 2.125769 -2.276903 -0.012448
C 1.525040 0.439433 -0.311764
O 1.995677 1.749045 -0.297678
H 1.613295 2.217944 0.456871
H 1.956155 -0.026903 -1.195299
FREQS= 41 57 74 88 89 103 143 208 265 312 317 343 385 409 420 455 470 509 511 533 542
551 604 759 881 1027 1037 1095 1137 1154 1251 1294 1358 1424 1459 1506 1512 1565 2071 2088 2147 2500 3052
3083 3126 3132 3156 3800
MIN045_8
18
E= -1916.40158299 ZPE= 73.58 Gcorr 0.045347000
H -4.733068 -0.992147 0.769234
Co 0.783455 -0.100498 0.203247
C 1.508430 0.799089 -1.153536
C 0.586511 1.226044 1.341368
O 2.033756 1.383325 -1.989063
O 0.545643 2.019781 2.173858
C 1.764126 -1.557554 0.139823
O 2.442443 -2.483206 0.233061
C -2.655072 -0.945374 0.160886
C -4.071711 -0.377218 0.153361
H -2.233765 -0.996833 1.174039
H -2.626157 -1.981416 -0.208290
H -4.085274 0.642982 0.545405
H -4.475975 -0.344815 -0.861562
C -1.685181 -0.157671 -0.681235
O -1.945208 0.820617 -1.335746
H 0.344475 -0.828066 1.436177
H -0.639224 -0.634293 -0.763570
FREQS= 17 39 47 71 72 83 95 108 126 193 263 325 334 346 364 453 481 487 507 564 581
644 669 741 760 856 982 1008 1108 1188 1283 1303 1367 1431 1458 1508 1513 1840 1972 2097 2122 2175 2446
3028 3063 3065 3140 3142
MIN046_8
18

E= -1916.41362402 ZPE=   77.44 Gcorr   0.057797000
H -2.720862 1.597916 -2.138492
Co 0.397924 0.020221 -0.079514
C 0.172395 0.711069 1.533418
C 1.215697 -1.537570 -0.210283
O 0.125489 1.029307 2.642661
O 1.767063 -2.550149 -0.160333
C 1.760414 1.058074 -0.618820
O 2.641168 1.718391 -0.951845
C -1.490444 0.022628 -1.282043
C -2.672833 0.992528 -1.229475
H -1.499038 -0.566899 -2.202084
H -0.556274 0.680983 -1.423852
H -3.609147 0.437150 -1.128281
H -2.594393 1.682222 -0.383245
C -1.347459 -0.894006 -0.078811
O -2.279466 -0.782128 0.946524
H -1.308245 -1.950206 -0.332150
H -2.316648 0.133155 1.255009
FREQS=   42   59   79   87   91 100 166 210 274 295 313 337 388 412 416 460 495 510 525 541 551
578 606 762 855 1011 1018 1085 1142 1170 1254 1317 1360 1428 1461 1505 1518 1566 2070 2088 2146 2485 3055
3097 3126 3140 3166 3810
MIN047_8
18
E= -1916.40978584 ZPE=   75.42 Gcorr   0.054174000
H 3.519152 -1.237562 0.437332
Co -0.345145 -0.037029 -0.118568
C -1.296571 -0.111606 1.393507
C -1.081758 1.403454 -0.842707
O -1.919802 -0.164321 2.357498
O -1.521627 2.310595 -1.397517
C -0.757321 -1.622274 -0.800969
O -0.964475 -2.621194 -1.334810
C 1.531157 -0.470607 0.757725
C 2.542403 -1.236568 -0.064123
H 1.296069 -0.920909 1.718353
H 0.390627 0.024879 -1.409259
H 2.237647 -2.277487 -0.199426
H 2.681423 -0.821308 -1.067473
C 1.408821 0.920443 0.714406
O 2.108190 1.751834 -0.116539
H 1.067791 1.471892 1.583855
H 2.427527 1.247915 -0.878151
FREQS=   57   59   66   82   92 104 161 198 217 285 320 338 345 387 429 445 480 496 507 519 554
565 635 768 815 878 903 982 1026 1073 1140 1251 1345 1418 1441 1488 1515 1573 1993 2098 2114 2157 3031
3095 3124 3172 3213 3806
MIN048_8
18
E= -1916.41183242 ZPE=   76.79 Gcorr   0.056741000
H -2.332537 2.064827 0.479683
Co 0.409112 0.024932 -0.017556
C 1.513965 -0.031589 1.380469
C 0.885333 -1.513489 -0.747305
O 2.214646 -0.070632 2.290312
O 1.258023 -2.439416 -1.326585
C 0.939485 1.580202 -0.687868
O 1.333461 2.517918 -1.230141
C -1.234078 0.067090 -1.122595
C -2.795377 1.086503 0.629625
H -1.439092 -0.790401 -1.761664
H -1.425996 0.996815 -1.653795
H -3.688760 1.013145 0.003685
H -3.095233 0.991857 1.676354
C -1.826595 -0.030528 0.245964
O -2.436951 -1.263341 0.565296
H -0.955698 0.115780 1.036562
H -1.958531 -1.970566 0.112398
FREQS=   52   55   76   84   92 100 158 209 250 279 316 358 395 422 444 468 494 508 515 526 563
567 604 839 862 982 997 1032 1095 1116 1154 1256 1366 1421 1492 1510 1514 1583 2078 2097 2152 2161 3061
3119 3142 3148 3186 3813
MIN049_8
18
E= -1916.41233505 ZPE=   77.43 Gcorr   0.057394000
H 0.639893 1.698503 -2.290861
Co -0.364980 -0.022631 0.085894
C -0.393536 -1.475609 -0.844362
C 1.256701 -0.603285 0.824738

O -0.430008 -2.367698 -1.576258
O 1.227891 -0.585671 2.043238
C -2.140603 0.074751 0.231627
O -3.258267 0.086819 0.504427
C 0.858209 1.713172 -0.134161
C 1.423707 1.771623 -1.531644
H -0.694397 2.372647 1.199060
H 1.590207 1.750462 0.668293
H 1.941030 2.729711 -1.675910
H 2.151349 0.974412 -1.701581
C -0.443523 2.075757 0.183058
O 2.333621 -1.053788 0.139571
H -1.115745 2.440466 -0.588384
H 2.990486 -1.330898 0.806892
FREQS= 39 49 84 89 102 108 147 192 211 229 312 345 371 403 428 436 472 502 519 592 599
673 709 796 907 945 972 1038 1082 1127 1200 1281 1318 1416 1442 1492 1510 1576 1743 2083 2130 3031 3103
3139 3140 3178 3226 3673
MIN050_8
18
E= -1916.39984268 ZPE= 73.60 Gcorr 0.045032000
H 2.582857 1.951422 -0.112860
Co -0.653552 0.148071 -0.196425
C -0.934709 -1.155314 -1.343081
C -1.559570 -0.499896 1.195457
O -1.183869 -1.907934 -2.177657
O -2.200099 -0.902471 2.057263
C -1.141313 1.833427 -0.096702
O -1.508991 2.922464 -0.165373
C 2.852067 -0.206851 -0.340660
C 3.378532 1.203839 -0.032755
H 2.463129 -0.234801 -1.366988
H 3.635073 -0.964992 -0.249464
H 3.800068 1.259332 0.975208
H 4.163521 1.476480 -0.742492
C 1.723304 -0.602367 0.577067
O 1.673516 -1.593237 1.260823
H -0.059711 0.719844 -1.446365
H 0.906629 0.207174 0.690015
FREQS= 18 33 48 52 72 84 94 109 125 201 248 327 331 349 426 470 487 494 505 517 563
582 642 766 776 876 989 1021 1144 1193 1276 1285 1345 1426 1471 1514 1517 1839 1974 2096 2122 2175 2417
3046 3055 3112 3132 3137
MIN051_8
18
E= -1916.39973495 ZPE= 73.68 Gcorr 0.044929000
H -3.917740 0.293381 -1.399098
Co 0.714436 -0.191954 -0.233467
C 1.768948 0.991331 -1.006507
C 1.196089 0.072798 1.458673
O 2.514121 1.658495 -1.573013
O 1.582417 0.198124 2.532860
C 0.519310 -1.934031 -0.187514
O 0.501429 -3.084184 -0.250187
C -2.812586 0.159923 0.480971
C -3.480318 -0.471745 -0.751312
H -3.515601 0.761261 1.064377
H -2.419974 -0.634648 1.130816
H -2.762458 -1.049166 -1.342680
H -4.279401 -1.150065 -0.442042
C -1.649329 1.042687 0.104299
O -1.503335 2.197826 0.414199
H 0.494302 -0.509082 -1.680485
H -0.898648 0.543197 -0.609816
FREQS= 17 34 43 55 65 92 96 107 120 196 239 324 337 345 435 470 486 495 507 519 569
578 643 758 777 879 985 1020 1148 1188 1278 1304 1353 1428 1472 1513 1517 1843 1972 2096 2119 2173 2475
3035 3056 3112 3132 3138
MIN052_8
18
E= -1916.41058640 ZPE= 76.70 Gcorr 0.055837000
H 4.430781 1.072448 -0.447703
Co -0.569135 0.029935 -0.115629
C -2.053687 0.489852 -1.023770
C -1.145250 -1.477621 0.599557
O -2.953275 0.781167 -1.677430
O -1.552068 -2.368169 1.212958
C -0.418944 1.461021 0.912616
O -0.388757 2.306800 1.696231
C 2.416396 0.538099 0.168812

C 3.634151 0.390138 -0.758536
H 2.708979 0.299316 1.198988
H 2.067967 1.573125 0.162845
H 4.023467 -0.629778 -0.735555
H 3.371513 0.631957 -1.794304
C 1.264368 -0.379691 -0.190249
O 1.744409 -1.663367 -0.211078
H 0.750010 -0.067797 -1.229624
H 1.029238 -2.269771 -0.448689
FREQS= 46 58 72 84 89 94 106 221 240 296 315 325 356 402 450 494 505 508 529 543 563 577 663 799 909 1010 1050 1062 1105 1218 1275 1320 1342 1390 1421 1485 1510 1521 2070 2090 2098 2148 3050 3055 3115 3123 3150 3799
MIN053_8
18
E= -1916.41092982 ZPE= 76.81 Gcorr 0.056472000
H -2.670753 0.756178 1.315559
Co 0.578888 0.012878 -0.159537
C 2.271943 0.412239 -0.620341
C 0.132911 1.541931 0.609818
O 3.329756 0.654614 -0.999377
O -0.127169 2.467242 1.247644
C 0.899498 -1.402543 0.843967
O 1.092332 -2.222618 1.634726
C -2.349235 0.476868 -0.823380
C -3.226932 0.419985 0.435340
H -2.946552 0.172729 -1.694381
H -2.007407 1.498638 -1.006323
H -4.099830 1.068451 0.318021
H -3.578193 -0.598165 0.618924
C -1.150524 -0.446299 -0.739387
O -1.616543 -1.734910 -0.779133
H -0.872335 -2.348957 -0.709794
H -0.347868 -0.224302 -1.601482
FREQS= 47 55 78 86 90 103 116 207 247 304 315 333 363 402 448 504 507 525 529 549 559 571 674 808 897 1014 1036 1070 1107 1222 1280 1312 1351 1378 1424 1493 1511 1522 2068 2090 2112 2148 3031 3058 3109 3130 3145 3799
MIN054_8
18
E= -1916.40740762 ZPE= 76.68 Gcorr 0.054005000
H -3.723538 0.680429 -1.026847
Co 0.697779 0.022576 -0.087118
C 0.556114 1.797598 0.208011
C 0.571351 -0.835714 1.416950
O 0.555532 2.890732 0.558110
O 0.590421 -1.342607 2.454123
C 2.000125 -1.247969 -0.926081
O 2.137431 -0.000395 -1.283507
C -2.320143 -0.078107 0.453454
C -3.652497 -0.129536 -0.296161
H -2.198162 0.858135 1.009729
H -2.251587 -0.875307 1.205701
H -3.762528 -1.074097 -0.834027
H -4.484482 -0.031238 0.406601
C -1.111397 -0.241340 -0.445733
O -1.147664 -0.563536 -1.608462
H 2.714551 -1.679721 -0.216153
H 1.518651 -1.950888 -1.617562
FREQS= 35 38 66 74 85 91 127 169 179 207 274 295 364 400 419 427 497 505 511 537 592 694 768 783 934 1015 1071 1082 1105 1215 1277 1333 1354 1426 1461 1508 1514 1568 1801 2085 2142 3018 3048 3064 3088 3108 3137 3146
MIN055_8
18
E= -1916.40143992 ZPE= 73.17 Gcorr 0.048193000
H -4.457581 -0.002200 -1.469970
Co 0.596641 -0.000129 -0.273458
C 2.375320 -0.001076 -0.628046
C 0.597780 1.586799 0.543352
O 3.510339 -0.001687 -0.787296
O 0.617674 2.593096 1.098354
C 0.596389 -1.586127 0.545023
O 0.615448 -2.591869 1.101082
C -2.338229 -0.001121 -1.022933
C -3.803875 -0.000504 -0.592598
H -2.103598 -0.876295 -1.642472
H -2.103671 0.872126 -1.645206
H -4.034285 -0.880244 0.013174
H -4.034509 0.881402 0.009930

C -1.361374 0.000765 0.164977
O -1.769844 0.002647 1.298785
H 0.219625 -0.416633 -1.813941
H 0.219706 0.415430 -1.814193
FREQS= 23 44 70 87 89 99 110 203 210 267 271 333 354 359 427 444 462 471 514 526 544 555 662 679 683 793 887 983 1024 1069 1099 1283 1340 1423 1464 1504 1511 1577 1796 2108 2119 2170 3038 3061 3074 3098 3134 3145
MIN056_8
18
E= -1916.40571374 ZPE= 75.10 Gcorr 0.052764000
H -1.607955 2.434066 -0.851238
Co 0.500762 -0.026105 0.241108
C 1.098962 1.643780 0.027266
C -0.567460 -0.200568 1.655522
O 1.518486 2.712901 -0.057262
O -1.161027 -0.307716 2.637119
C 1.646753 -1.233128 -0.359876
O 2.447908 -2.001912 -0.677035
C -0.917397 0.443193 -1.337754
C -2.005800 1.424674 -0.980615
H -0.479962 -1.630039 -1.699571
H -0.278756 0.712043 -2.173779
H -2.525836 1.136373 -0.065567
H -2.748850 1.459113 -1.787752
C -1.032716 -0.914923 -1.097505
O -2.145164 -1.403302 -0.468785
H 1.481535 -0.076120 1.330259
H -1.976419 -2.308528 -0.176806
FREQS= 31 56 67 88 94 100 185 196 209 260 309 324 365 372 393 451 462 471 514 521 528 554 647 695 717 874 918 978 1035 1075 1152 1269 1290 1410 1444 1498 1503 1615 2046 2076 2088 2145 3040 3113 3153 3177 3196 3828
MIN057_8
18
E= -1916.40851926 ZPE= 76.60 Gcorr 0.055720000
H 3.928413 -1.343394 -0.341444
Co -0.470486 -0.041254 -0.116199
C 0.080196 -1.202670 1.089893
C -1.429899 1.323233 0.473526
O 0.347350 -1.873685 1.990272
O -2.060265 2.118205 1.026292
C -1.784541 -1.020987 -0.861923
O -2.579245 -1.641698 -1.413787
C 2.568410 0.317389 -0.087630
C 2.880319 -1.107684 -0.547742
H 2.732476 0.414107 0.993390
H 3.269244 1.016991 -0.559827
H 2.720415 -1.220063 -1.625554
H 2.269699 -1.855089 -0.036853
C 1.169732 0.834835 -0.382714
O 1.268694 2.193390 -0.523411
H 0.676969 0.303277 -1.346443
H 0.388340 2.563648 -0.681279
FREQS= 46 55 74 82 87 97 106 197 234 313 324 339 379 421 444 501 506 514 537 539 561 569 608 790 916 1016 1063 1084 1107 1205 1277 1313 1349 1395 1434 1484 1513 1518 2062 2067 2088 2147 3041 3054 3075 3120 3144 3787
MIN058_8
18
E= -1916.40761011 ZPE= 76.47 Gcorr 0.055130000
H -3.550738 0.532280 0.726997
Co 0.596582 -0.022861 -0.156132
C 0.852204 1.457358 0.785368
C 0.148003 -1.576534 0.541084
O 0.993590 2.326505 1.528146
O -0.118573 -2.522269 1.149246
C 2.312669 -0.386911 -0.537476
O 3.391552 -0.606335 -0.869628
C -2.344570 -0.486775 -0.805768
C -3.188130 -0.471832 0.478755
H -2.011144 -1.505785 -1.016000
H -2.970397 -0.180931 -1.659802
H -4.064623 -1.116118 0.368104
H -2.602874 -0.830393 1.329667
C -1.139175 0.437763 -0.743299
O -1.503427 1.774380 -0.752584
H -0.395604 0.248832 -1.626142
H -2.463480 1.852694 -0.660677

FREQS=   44   54   78   88   93 105 118 185 199 257 298 322 335 365 402 457 497 518 523 545 557
575 654 806 886 1003 1023 1084 1118 1214 1262 1295 1342 1378 1431 1496 1511 1519 2069 2093 2150 2341 2997
3047 3105 3121 3140 3789
MIN059_8
18
E= -1916.40706615 ZPE=   76.73 Gcorr   0.054707000
H 3.733509 -1.409896 -0.528261
Co -0.588670 0.254632 0.080552
C -0.088638 -0.934131 1.248471
C 0.587315 1.712893 0.334096
O 0.162099 -1.669930 2.096508
O 1.650516 2.083267 0.737803
C -2.293552 -0.360698 -0.412909
O -3.350949 -0.775253 -0.546380
C 1.905916 -0.605126 -1.379042
C 2.661356 -1.398698 -0.310849
H 2.226876 0.441362 -1.352765
H 2.199604 -0.982091 -2.373104
H 2.537482 -0.951946 0.679469
H 2.319988 -2.439143 -0.265152
C 0.390641 -0.702067 -1.340784
O -0.474639 2.123317 -0.238503
H -0.058049 -0.153427 -2.180019
H 0.060238 -1.744156 -1.384389
FREQS=   29   51   66   75   93 106 116 132 216 281 302 311 366 380 413 433 481 498 515 568 598
713 734 806 886 990 1031 1086 1154 1193 1231 1301 1387 1427 1460 1486 1510 1523 1962 2107 2160 2985 3026
3046 3085 3107 3118 3130
MIN060_8
18
E= -1916.40733773 ZPE=   76.56 Gcorr   0.054994000
H 4.429613 -1.095787 -0.439225
Co -0.602407 -0.039311 -0.108754
C -0.409663 -1.526606 0.813254
C -1.045500 1.526185 0.593931
O -0.354231 -2.410099 1.555542
O -1.346497 2.459688 1.198536
C -2.165688 -0.422499 -0.907787
O -3.120075 -0.661203 -1.503108
C 2.403483 -0.559490 0.152456
C 3.629670 -0.426018 -0.767938
H 2.063491 -1.597279 0.156901
H 2.689123 -0.327719 1.189273
H 3.371786 -0.688465 -1.799133
H 4.029582 0.592440 -0.779558
C 1.247401 0.352836 -0.230471
O 1.635915 1.687675 -0.292530
H 2.399399 1.822227 0.286483
H 0.802867 0.081059 -1.266560
FREQS=   41   57   72   88   90   95 110 202 236 251 295 317 325 371 401 453 489 502 522 537 562
573 651 794 904 1008 1020 1078 1119 1201 1235 1308 1345 1379 1427 1490 1511 1518 2071 2092 2150 2433 3023
3050 3111 3125 3131 3797
MIN061_8
18
E= -1916.40552897 ZPE=   75.89 Gcorr   0.053468000
H -1.561801 -0.086809 1.761727
Co 0.360068 -0.024789 0.005650
C 1.874700 0.415078 0.837252
C 0.984111 -1.514631 -0.700082
O 2.853079 0.694597 1.370290
O 1.393744 -2.404295 -1.312724
C 0.214151 1.517003 -0.839296
O 0.179856 2.436059 -1.538520
C -1.425553 -0.551883 -0.894364
C -3.354983 -0.229230 0.689696
H -1.365741 -0.331164 -1.962297
H -1.591769 -1.625637 -0.768316
H -4.108838 0.413659 1.132941
H -3.387685 -1.283123 0.961575
C -2.494765 0.226876 -0.244681
O -0.604468 -0.201536 1.955167
H -2.554425 1.282665 -0.513561
H -0.515240 -1.078155 2.350513
FREQS=   44   59   67   82   97 100 133 165 212 261 276 312 330 384 397 410 452 494 504 511 537
548 571 577 699 779 824 896 959 1026 1079 1136 1237 1327 1447 1496 1639 1664 2062 2087 2150 3068 3127
3139 3145 3235 3481 3861
MIN062_8
18

E= -1916.40308471 ZPE= 75.15 Gcorr 0.051400000
H -4.420456 0.662518 0.000000
Co 0.739500 0.106570 0.000000
C 0.549795 1.878977 0.000000
C 1.233410 -0.652333 1.524635
O 0.517804 3.031033 0.000000
O 1.629201 -1.089475 2.516341
C 1.233410 -0.652333 -1.524635
O 1.629201 -1.089475 -2.516341
C -2.263850 0.526397 0.000000
C -3.652768 -0.116161 0.000000
H -2.134749 1.190589 -0.865501
H -2.134749 1.190589 0.865501
H -3.804136 -0.742814 -0.882676
H -3.804136 -0.742814 0.882676
C -1.066275 -0.383419 0.000000
O -1.450750 -1.646096 0.000000
H 2.182244 0.517898 0.000000
H -0.656493 -2.208006 0.000000
FREQS= 24 52 59 60 84 93 96 221 234 311 315 333 340 403 437 459 487 500 523 544 551 682 690 704 774 808 911 1025 1079 1094 1279 1296 1357 1408 1433 1452 1512 1516 1959 2080 2083 2142 3041 3063 3065 3136 3141 3717
MIN063_8
18
E= -1916.40292171 ZPE= 75.44 Gcorr 0.051418000
H -4.488953 -0.589817 -0.358183
Co 0.466983 0.023021 0.030363
C 2.149050 -0.443872 0.429536
C 0.088636 -1.464748 -0.822304
O 3.221075 -0.759005 0.695948
O -0.147879 -2.359384 -1.511989
C 0.890943 1.586925 -0.693084
O 1.119504 2.517080 -1.336989
C -2.456098 -0.217894 0.257219
C -3.904329 0.187518 0.150452
H -1.017925 0.378197 1.996982
H -2.277284 -1.145243 0.809137
H -4.361960 0.325365 1.138391
H -4.017773 1.119744 -0.410680
C -1.423677 0.441470 -0.298274
O -0.083110 0.164021 2.171205
H -1.676879 1.316339 -0.901248
H 0.288361 0.935774 2.619138
FREQS= 44 63 71 82 93 102 115 154 200 220 234 262 276 314 318 397 407 466 494 509 517 537 572 577 618 673 769 945 1026 1062 1088 1265 1337 1420 1493 1499 1633 1647 2070 2093 2153 3025 3076 3098 3108 3123 3662 3860
MIN064_8
18
E= -1916.40272153 ZPE= 75.43 Gcorr 0.051300000
H -4.016134 1.093345 0.469491
Co 0.468278 0.012925 -0.026756
C 0.881542 1.592858 0.686246
C 2.156425 -0.429143 -0.418002
O 1.103714 2.538270 1.306481
O 3.235197 -0.729874 -0.675128
C 0.093332 -1.482087 0.804580
O -0.143756 -2.386445 1.483326
C -2.459072 -0.209783 -0.269458
C -3.906431 0.191308 -0.139440
H -2.284706 -1.109765 -0.867311
H -0.992983 0.519446 -1.979226
H -4.365452 0.383181 -1.117721
H -4.492293 -0.609963 0.329130
C -1.423730 0.415339 0.318184
O -0.050300 0.340594 -2.148771
H -0.027858 -0.454383 -2.697720
H -1.675319 1.257865 0.965857
FREQS= 45 60 69 82 93 103 113 156 201 218 235 255 275 316 320 394 406 467 491 497 521 535 574 579 631 673 765 945 1024 1061 1088 1266 1336 1420 1493 1499 1633 1650 2071 2094 2153 3025 3075 3088 3111 3124 3673 3862
MIN065_8
18
E= -1916.40269505 ZPE= 75.37 Gcorr 0.051340000
H 4.092150 -0.134331 -0.180304
Co -0.600343 -0.229337 0.149111
C -1.814502 -0.014665 -1.125425
C 0.200289 -1.820065 0.237930

O -2.645430 0.058842 -1.921715
O 0.643013 -2.879962 0.332191
C -0.811356 0.679160 1.657209
O -0.991116 1.194252 2.673864
C 2.147750 0.283995 -1.049326
C 3.111400 0.211350 0.156677
H 2.551756 0.971421 -1.801465
H 2.050212 -0.708910 -1.496091
H 3.235010 1.195149 0.617879
H 2.744005 -0.483272 0.915766
C 0.789300 0.755835 -0.612857
O 0.702042 2.058876 -0.824117
H -1.702499 -1.043222 0.762927
H -0.166729 2.365531 -0.511747
FREQS= 15 54 58 61 91 94 97 196 216 303 315 342 354 403 453 460 487 521 525 546 582
653 696 721 776 822 922 1013 1076 1109 1252 1320 1369 1373 1423 1498 1508 1522 1948 2081 2084 2142 3056
3061 3117 3139 3147 3720
MIN066_8
18
E= -1916.40773786 ZPE= 76.85 Gcorr 0.056384000
H 2.893452 1.826242 0.991748
Co -0.680699 0.023673 -0.163885
C -2.235758 -0.831374 -0.244665
C 0.207611 -1.325481 0.586816
O -3.229784 -1.374753 -0.443886
O 0.635007 -2.121617 1.298872
C -1.344928 1.597578 0.257076
O -1.717683 2.593380 0.701211
C 0.930541 0.934818 -0.793129
C 2.908963 0.749267 0.796964
H 0.934323 2.024249 -0.710558
H 0.592932 0.683557 -1.824353
H 2.317920 0.250652 1.570516
H 3.949349 0.413628 0.887212
C 2.354089 0.441981 -0.595057
O 2.380137 -0.958653 -0.879831
H 2.986538 0.963632 -1.336821
H 3.239826 -1.308737 -0.611812
FREQS= 37 56 81 93 97 132 147 162 249 286 319 329 356 392 423 437 484 493 506 522 577
589 599 730 841 974 981 1046 1121 1183 1222 1281 1378 1399 1423 1434 1507 1509 2081 2096 2160 2884 2941
3038 3105 3109 3132 3818
MIN067_8
18
E= -1916.39488937 ZPE= 73.31 Gcorr 0.043560000
H 3.763183 -1.818273 -0.197472
Co -0.740839 -0.060160 -0.240645
C -1.382609 -1.688863 -0.112997
C -0.977462 0.455193 1.442344
O -1.918341 -2.707029 -0.120301
O -1.260325 0.789940 2.503960
C -1.017984 1.488567 -1.037707
O -1.324955 2.432348 -1.615687
C 2.144975 -0.417843 -0.589655
C 2.677208 -1.828886 -0.334400
H 2.597345 0.050908 -1.469538
H 1.056233 -0.475717 -0.841356
H 2.230768 -2.267650 0.564289
H 2.452697 -2.489289 -1.175683
C 2.320871 0.523618 0.584537
O 2.481206 1.719258 0.481724
H -0.798016 -0.478649 -1.680660
H 2.289775 0.036144 1.587538
FREQS= 19 35 39 50 68 87 92 99 106 176 227 304 318 336 340 457 482 488 507 523 573
579 636 719 762 877 921 1039 1127 1142 1255 1329 1424 1434 1448 1511 1515 1826 1964 2102 2120 2180 2773
2873 3051 3095 3120 3135
MIN068_8
18
E= -1916.39859454 ZPE= 72.71 Gcorr 0.047355000
H 3.478604 0.881471 1.289955
Co -0.822358 0.000008 -0.400747
C -0.382091 -1.643917 -0.950817
C 0.895152 -0.000013 0.575942
O -0.114345 -2.716937 -1.272970
O 0.900204 -0.000014 1.780988
C -1.759745 0.000015 1.171906
O -2.329018 0.000017 2.166186
C 2.196322 -0.000031 -0.230207

C 3.449750 -0.000009 0.644598
H 2.173250 0.870749 -0.896698
H 2.173250 -0.870846 -0.896652
H 3.478607 -0.881461 1.289995
H 4.347101 -0.000022 0.018830
C -0.382051 1.643923 -0.950817
O -0.114279 2.716937 -1.272969
H -2.395337 0.000033 -1.062135
H -1.896333 0.000022 -1.696631
FREQS= 39 56 57 62 90 102 107 203 213 270 299 306 321 331 354 420 451 470 487 505 538
550 560 590 682 790 863 902 1011 1079 1098 1286 1350 1425 1461 1474 1505 1512 1802 2083 2108 2165 3053
3061 3086 3134 3143 3413
MIN069_8
18
E= -1916.40055019 ZPE= 73.24 Gcorr 0.049550000
H 3.244803 0.410264 -2.092265
Co -0.576502 -0.013965 0.364321
C -2.342044 -0.006104 -0.044873
C -0.303039 1.649798 -0.190410
O -3.449209 -0.010400 -0.342143
O -0.129695 2.715540 -0.592375
C -0.244859 -1.565296 -0.433691
O -0.050437 -2.556145 -0.987670
C 2.479337 0.418525 -0.071358
C 2.389144 0.006862 -1.543130
H 3.425935 0.090537 0.371185
H 2.449188 1.514209 0.006009
H 2.403370 -1.080962 -1.656072
H 1.480978 0.384828 -2.019542
C 1.376012 -0.098280 0.854485
O 1.648698 -0.541519 1.942250
H -0.858378 0.284183 2.008343
H -0.862512 -0.518849 1.959047
FREQS= 38 59 70 87 89 101 118 188 224 263 292 338 360 379 432 443 467 492 522 539 546
559 568 608 627 793 863 903 1038 1074 1124 1280 1334 1411 1428 1474 1513 1517 1800 2088 2109 2163 3035
3059 3094 3129 3138 3450
MIN070_8
18
E= -1916.39883960 ZPE= 73.31 Gcorr 0.047882000
H -2.367587 -1.383168 -1.634831
Co 0.488176 -0.025952 -0.233771
C 2.196662 -0.370294 -0.738690
C 0.813639 1.625038 0.348516
O 3.294315 -0.574069 -0.998011
O 1.050734 2.674622 0.754707
C 0.348661 -1.465606 0.820075
O 0.279241 -2.368434 1.526431
C -2.446586 0.619223 -0.741624
C -3.094807 -0.704248 -1.177138
H -2.009588 1.135387 -1.601113
H -3.201645 1.272095 -0.291296
H -3.883967 -0.516663 -1.910641
H -3.539791 -1.214092 -0.318068
C -1.385787 0.384097 0.348338
O -1.707971 0.425319 1.509510
H -0.133692 -0.591922 -1.640658
H 0.034259 0.210314 -1.789518
FREQS= 16 45 58 85 92 101 103 199 224 268 297 317 350 365 439 443 463 497 511 531 543
566 645 668 696 806 887 990 1017 1065 1123 1275 1315 1419 1493 1508 1518 1586 1791 2107 2120 2170 3050
3062 3086 3111 3124 3137
MIN071_8
18
E= -1916.40639604 ZPE= 76.81 Gcorr 0.055493000
H 3.547688 -1.402174 0.166426
Co -0.658488 0.106885 -0.315147
C -0.479737 -0.907894 -1.972736
C -1.655142 -0.982440 0.697562
O -0.200931 0.386009 -2.075941
O -2.387666 -1.658902 1.265917
C -0.733376 1.735529 0.469735
O -0.870349 2.752260 0.983183
C 2.178472 0.255969 -0.173884
C 3.496620 -0.324044 0.340059
H 2.129942 1.339676 -0.011554
H -1.417169 -1.273439 -2.407413
H 4.335121 0.147622 -0.180745
H 3.613015 -0.153548 1.412731

C 0.955364 -0.350241 0.536661
O 1.034054 -1.054927 1.502687
H 0.350956 -1.624158 -2.010668
H 2.045549 0.123324 -1.250940
FREQS= 42 52 70 75 89 114 125 162 197 216 293 333 391 409 417 433 479 508 517 545 598
683 780 796 871 995 1075 1085 1097 1203 1269 1280 1338 1423 1464 1505 1513 1562 1833 2111 2156 3013 3061
3062 3094 3117 3134 3147
MIN072_8
18
E= -1916.40682030 ZPE= 76.85 Gcorr 0.056005000
H 2.819661 1.836675 1.025643
Co -0.678250 0.023452 -0.167818
C -2.248775 -0.802195 -0.220896
C 0.195406 -1.360354 0.548869
O -3.256453 -1.323737 -0.410618
O 0.588489 -2.182073 1.249472
C -1.312771 1.610795 0.246489
O -1.667276 2.618020 0.678241
C 0.942059 0.915689 -0.810243
C 2.874526 0.759889 0.840180
H 0.941179 2.008214 -0.754776
H 0.627381 0.655029 -1.848831
H 2.270501 0.248289 1.595129
H 3.912670 0.436584 0.953562
C 2.369246 0.433398 -0.560459
O 2.518335 -0.978283 -0.736307
H 3.023383 0.949612 -1.286289
H 2.335067 -1.182347 -1.663292
FREQS= 31 50 80 92 95 134 147 160 225 316 329 344 365 390 422 438 479 490 504 522 579
589 599 730 847 964 978 1044 1093 1194 1235 1281 1349 1406 1434 1440 1498 1511 2086 2098 2162 2857 2947
3056 3092 3132 3137 3807
MIN073_8
18
E= -1916.40515855 ZPE= 76.36 Gcorr 0.054965000
H -2.615989 -1.305341 -1.640178
Co 0.486197 -0.054031 -0.110452
C 1.872459 -0.971415 -0.791234
C 1.362332 1.391100 0.448983
O 2.718464 -1.555242 -1.307022
O 1.926454 2.246583 0.974121
C -0.080862 -1.264582 1.024461
O -0.355715 -1.965270 1.902352
C -2.567254 0.271833 -0.138544
C -2.848318 -1.166158 -0.579221
H -3.268481 0.933199 -0.674165
H -2.793884 0.389738 0.929720
H -2.271121 -1.894600 -0.008091
H -3.907575 -1.398899 -0.435573
C -1.163180 0.813814 -0.385685
O -1.161567 2.196261 -0.447771
H -0.691841 0.375974 -1.372271
H -2.050584 2.532574 -0.263244
FREQS= 47 54 79 82 89 103 109 196 230 242 318 326 333 378 422 448 494 496 523 533 555
571 587 798 918 1018 1060 1099 1122 1189 1256 1312 1351 1388 1436 1485 1511 1520 2067 2094 2149 2276 2982
3052 3059 3122 3157 3775
MIN074_8
18
E= -1916.40679051 ZPE= 77.00 Gcorr 0.057078000
H 2.098177 2.073184 0.596179
Co -0.357827 0.013900 0.046541
C -0.557632 1.770120 0.004106
C -0.528439 -0.992061 -1.404011
O -0.726067 2.895637 -0.185414
O -0.660929 -1.528909 -2.415176
C -1.941252 -0.381006 0.769609
O -2.958164 -0.639250 1.238966
C 1.603018 0.122684 -0.249780
C 2.371605 1.433559 -0.249099
H 2.056983 -0.106808 1.854744
H 1.934676 -0.527015 -1.061274
H 3.453672 1.249632 -0.188394
H 2.189904 2.001681 -1.165772
C 1.522745 -0.632403 1.048548
O 1.838638 -2.010591 1.001662
H 2.711152 -2.091879 0.594053
H 0.448675 -0.634543 1.477308

FREQS=   45   57   79   91 100 106 162 235 238 261 318 331 340 400 416 449 471 501 511 531 548
564 580 852 934 999 1074 1098 1123 1151 1237 1333 1346 1410 1430 1502 1511 1603 2079 2096 2153 2435 3003
3019 3089 3113 3129 3815
MIN075_8
18
E= -1916.40651317 ZPE=   76.99 Gcorr   0.057102000
H 2.646833 0.146978 -1.161370
Co -0.406975 -0.000127 0.089552
C 0.023555 0.721970 -1.469586
C -1.185545 -1.588200 0.114676
O 0.210092 1.061430 -2.556920
O -1.667673 -2.629776 0.000914
C -1.859757 0.987490 0.419167
O -2.793246 1.621139 0.638107
C 1.377643 -0.855861 0.324995
C 2.497156 -0.831973 -0.697277
H 0.284418 0.599021 1.556490
H 1.267968 -1.858305 0.738583
H 3.447521 -1.121797 -0.229780
H 2.294048 -1.541699 -1.503892
C 1.377976 0.185059 1.404026
O 2.242631 1.283564 1.272925
H 2.182571 1.614365 0.366746
H 1.544345 -0.236897 2.399099
FREQS=   43   58   80   89   94 105 167 212 244 291 312 318 370 404 419 453 497 506 513 540 559
565 609 838 902 1007 1021 1095 1121 1186 1215 1296 1386 1429 1432 1500 1514 1622 2074 2093 2151 2232 3032
3093 3100 3119 3133 3815
MIN076_8
18
E= -1916.40423426 ZPE=   76.95 Gcorr   0.054974000
H 4.410441 -0.124181 -0.238467
Co -0.677348 -0.277480 -0.109219
C -0.424258 -1.947039 -1.079616
C -1.691363 1.287307 -0.113000
O -1.062040 -0.983830 -1.739805
O -2.405999 2.176594 -0.051358
C 0.999511 0.595198 -0.105992
O 1.038664 1.795481 -0.066790
C 3.544438 0.517415 -0.051774
C 2.252944 -0.264505 -0.300962
H 3.597158 0.872627 0.980939
H 3.605942 1.388079 -0.707942
H 2.179726 -1.150542 0.335533
H 2.212130 -0.629016 -1.335406
C -0.453596 -0.712019 1.576378
O -0.404181 -1.079309 2.667369
H -1.013141 -2.802453 -0.728721
H 0.598535 -2.192199 -1.382534
FREQS=   16   67   70   83   97 104 115 192 202 221 295 325 379 386 425 446 469 500 512 572 621
691 797 801 894 998 1074 1078 1103 1200 1265 1278 1336 1425 1470 1506 1514 1560 1814 2101 2169 3029 3055
3062 3109 3112 3134 3151
MIN077_8
18
E= -1916.39706951 ZPE=   72.57 Gcorr   0.047979000
H 4.431199 1.092582 -1.116448
Co -0.589757 0.096142 -0.171693
C -2.218306 0.584711 -0.817098
C -0.948151 -1.633977 0.270373
O -3.149806 0.964407 -1.362620
O -1.176029 -2.734630 0.467320
C -0.480210 0.986657 1.382476
O -0.374723 1.627740 2.326307
C 2.326596 0.731838 -0.754729
C 3.799078 0.343242 -0.630636
H 2.126698 1.711861 -0.302734
H 2.017218 0.832138 -1.802321
H 4.100209 0.266140 0.417293
H 3.990396 -0.625617 -1.098831
C 1.371666 -0.269415 -0.109614
O 1.778441 -1.271421 0.427385
H -0.203055 1.352185 -0.834429
H -0.266344 -0.372216 -1.538599
FREQS=   36   56   70   81   88   98 101 208 218 266 271 330 342 398 419 440 464 486 500 529 542
645 656 678 710 788 882 917 1015 1076 1099 1277 1345 1424 1460 1506 1513 1789 2020 2059 2143 2161 2199
3046 3060 3080 3132 3141
MIN078_8
18

```
E= -1916.40504516 ZPE=    76.89 Gcorr    0.056294000
H 2.592412 -1.586791 0.777743
Co -0.010299 -0.245179 0.191493
C -1.553337 -1.173946 0.586402
C 0.315167 0.946048 1.463213
O -2.490254 -1.774849 0.856366
O 0.564994 1.608861 2.373255
C -0.845169 1.028205 -0.901994
O -1.981112 0.882103 -1.269332
C 1.693254 -0.469925 -0.861776
C 2.866894 -0.836031 0.026503
H 0.762592 2.031903 -0.915215
H 1.890455 0.339773 -1.563563
H 3.678999 -1.271942 -0.570453
H 3.273917 0.031290 0.555182
C 0.731358 -1.433656 -1.242526
O -0.118388 2.097950 -1.310136
H 0.203942 -1.341133 -2.188421
H 0.824824 -2.459965 -0.885724
FREQS=   44   65   72   77   89  101  141  170  222  250  344  348  384  388  426  433  468  482  504  521  568
642  699  776  891  930  968  992 1074 1115 1189 1243 1261 1406 1432 1497 1504 1535 1823 2087 2150 3023 3077
3110 3115 3138 3205 3806
MIN079_8
18
E= -1916.39575830 ZPE=    73.71 Gcorr    0.047105000
H 3.858985 0.516013 0.738438
Co -0.850319 -0.108573 0.055767
C -0.357639 0.171809 1.753769
C -1.805837 1.207864 -0.682917
O -0.198701 0.310691 2.885155
O -2.521466 2.013429 -1.083811
C -1.025421 -1.805171 -0.452334
O -1.253326 -2.905771 -0.705330
C 2.410405 -0.589101 -0.445073
C 3.822193 -0.070505 -0.183092
H 2.362121 -1.202186 -1.357358
H 2.055725 -1.247668 0.357724
H 4.520150 -0.906098 -0.087606
H 4.162000 0.572738 -0.998498
C 1.395338 0.512470 -0.612971
O 1.615758 1.693471 -0.599991
H -2.135562 -0.402693 0.660061
H 0.362844 0.142600 -1.050931
FREQS=   29   46   60   61   69   84   92  143  164  200  272  312  336  345  419  428  450  454  513  517  521
666  691  714  768  858 1004 1053 1103 1227 1261 1360 1431 1455 1470 1508 1513 1849 2087 2091 2100 2159 2223
3030 3067 3072 3142 3145
MIN080_8
18
E= -1916.40239200 ZPE=    76.20 Gcorr    0.053917000
H 3.893074 -1.375270 -0.061121
Co -0.378806 -0.187318 -0.040846
C -1.265795 0.333185 1.479274
C -0.615678 1.385557 -0.823571
O -2.065468 1.192608 1.702195
O -0.697241 2.371781 -1.407126
C -1.712585 -1.277075 -0.639790
O -2.565250 -1.901864 -1.081254
C 2.571884 0.325910 -0.387334
C 3.938892 -0.282979 -0.031293
H 2.636834 1.420523 -0.384529
H 2.285497 0.015581 -1.399766
H 4.707192 0.048790 -0.736374
H 4.254743 0.015095 0.974239
C 1.500569 -0.110217 0.590916
O 0.983495 -1.319416 0.499533
H -0.898821 -0.368156 2.270666
H 1.601228 0.309875 1.603731
FREQS=   46   59   64   72   86  104  109  161  163  233  261  317  370  391  432  451  479  506  509  524  605
632  794  840  891  993 1007 1096 1152 1237 1282 1309 1347 1417 1475 1486 1511 1520 1846 2113 2161 2827 3006
3038 3047 3083 3118 3133
MIN081_8
18
E= -1916.40178639 ZPE=    75.96 Gcorr    0.053398000
H -2.586688 1.342527 0.472538
Co 0.446836 -0.252622 -0.144636
C 2.204807 -0.680028 0.063169
C -1.344777 -1.029805 -0.594853
```

O 3.334676 -0.869597 0.075356
O -0.404191 -1.896592 -0.296251
C 0.376890 1.258270 -1.045845
O 0.358332 2.181907 -1.730239
C -2.530716 -0.834895 0.326195
C -3.232726 0.515745 0.160934
H -2.215382 -0.994452 1.363788
H -3.239370 -1.645557 0.098010
H -3.516021 0.689143 -0.883058
H -4.145180 0.557462 0.762130
C 0.321336 0.606026 1.460966
O 0.522842 1.705837 1.878249
H -1.566705 -0.882630 -1.662527
H -0.057363 -0.230016 2.113959
FREQS= 36 54 65 73 75 101 115 177 185 235 281 335 361 399 434 447 472 487 508 510 554
624 788 811 896 976 1037 1105 1155 1212 1272 1317 1336 1430 1479 1489 1513 1518 1858 2109 2158 2750 3012
3017 3048 3075 3117 3128
MIN082_8
18
E= -1916.40153858 ZPE= 75.99 Gcorr 0.053244000
H -4.687883 0.173403 0.776113
Co 0.430417 -0.205304 -0.149649
C 0.660991 0.537809 1.501879
C 1.912759 -1.256237 -0.033104
O 1.265575 1.454443 1.968477
O 2.892198 -1.848910 -0.079384
C 0.894255 1.282545 -0.968335
O 1.194123 2.193746 -1.602051
C -2.545768 0.306296 0.389987
C -3.950700 -0.240674 0.081520
H -2.550770 1.401037 0.327777
H -2.263395 0.034844 1.413381
H -4.263945 0.021186 -0.934778
H -3.969803 -1.330698 0.170589
C -1.522809 -0.247626 -0.579010
O -0.963470 -1.416628 -0.357273
H 0.008464 -0.138828 2.122054
H -1.693690 0.028361 -1.630385
FREQS= 38 52 70 73 80 98 115 162 189 228 251 299 361 408 442 449 480 488 491 523 592
627 790 811 892 991 1009 1097 1152 1210 1283 1317 1344 1418 1481 1483 1511 1519 1860 2110 2158 2754 3015
3046 3048 3090 3120 3131
MIN083_8
18
E= -1916.39937818 ZPE= 75.71 Gcorr 0.051526000
H 4.066149 0.633844 -0.715838
Co -0.566609 -0.030116 0.126196
C -1.086882 1.466002 0.948110
C -0.161098 0.657554 -1.468526
O -1.396320 2.385464 1.568549
O 0.089927 1.106590 -2.496925
C -1.898461 -1.118440 -0.351907
O -2.735356 -1.876661 -0.575907
C 2.363087 -0.683852 -0.414227
C 3.169359 0.592523 -0.091503
H 2.987943 -1.564749 -0.224352
H 2.083573 -0.682650 -1.470455
H 2.579027 1.493298 -0.280720
H 3.480330 0.600284 0.956764
C 1.122947 -0.760703 0.445292
O 1.450706 -1.433131 1.545445
H 0.674423 -1.456119 2.127585
H -0.898377 -0.587371 1.466989
FREQS= 20 29 49 61 91 95 113 199 219 306 317 346 362 422 446 461 476 534 550 558 575
625 720 762 819 891 917 1013 1076 1097 1251 1320 1353 1370 1420 1501 1508 1523 1963 2093 2109 2151 3053
3057 3116 3135 3141 3747
MIN084_8
18
E= -1916.39981702 ZPE= 75.58 Gcorr 0.052041000
H -3.605811 -0.227837 1.467558
Co 0.380542 0.026825 0.012911
C 1.736028 -1.042538 0.489709
C -0.320705 -1.109546 -1.114932
O 2.588150 -1.743974 0.809015
O -0.724584 -1.767748 -1.973797
C 1.391183 1.426425 -0.428224
O 2.001330 2.272786 -0.920828
C -2.493060 0.823048 -0.065275

C -3.061175 -0.436107 0.537735
H -3.242443 1.593498 -0.269888
H -1.068015 0.807640 1.880544
H -3.789246 -0.885969 -0.149242
H -2.295328 -1.183611 0.755696
C -1.219864 1.092103 -0.411184
O -0.323806 0.221433 2.106799
H -1.065692 2.047560 -0.912956
H 0.268729 0.744160 2.663198
FREQS= 45 59 75 83 92 109 118 153 169 198 242 282 311 353 388 411 427 474 481 509 521 536 568 574 614 645 714 925 1011 1065 1072 1270 1362 1424 1494 1498 1634 1646 2068 2091 2151 3029 3075 3088 3131 3138 3680 3864
MIN085_8
18
E= -1916.39388070 ZPE= 74.41 Gcorr 0.046450000
H 5.389310 -0.721119 0.096506
Co -0.885874 0.015277 -0.208473
C -0.716015 1.719384 -0.576657
C -2.243401 -1.063524 -0.470208
O -0.753172 2.829676 -0.890542
O -3.207308 -1.646702 -0.720422
C -1.016331 0.151236 1.555910
O -1.172378 0.282708 2.688536
C 3.325151 -0.458826 -0.469525
C 4.587824 0.021919 0.185304
H -0.977441 0.010700 -1.707198
H 3.370145 -0.684134 -1.536083
H 4.951657 0.943126 -0.284555
H 4.434895 0.225246 1.249093
C 2.174089 -0.622390 0.177111
O 0.996208 -1.085824 -0.391106
H 1.143408 -1.286874 -1.326140
H 2.031932 -0.425073 1.233823
FREQS= 13 29 48 71 74 91 103 122 182 210 282 316 330 337 364 457 482 490 513 519 560 565 579 687 746 805 937 958 1067 1116 1125 1243 1340 1391 1436 1492 1510 1758 1942 2077 2097 2158 3036 3089 3121 3140 3228 3810
MIN086_8
18
E= -1916.39342484 ZPE= 74.46 Gcorr 0.046616000
H -3.342776 0.754829 -1.487746
Co 0.775348 0.023631 -0.216753
C 0.658439 -1.621303 -0.807386
C 1.957317 1.267123 -0.581351
O 0.711196 -2.666060 -1.295781
O 2.795762 1.978605 -0.931408
C 1.304249 -0.341381 1.437290
O 1.717535 -0.611016 2.476950
C -3.393547 0.045255 0.579634
C -4.034902 0.336474 -0.747617
H -4.013221 -0.433416 1.332479
H -1.557275 1.192324 -0.683969
H -4.450307 -0.577284 -1.186804
H -4.862235 1.047820 -0.640804
C -2.139749 0.309360 0.944124
O -1.176327 0.934623 0.166876
H -1.733733 0.068317 1.919023
H 0.528992 0.247003 -1.681105
FREQS= 24 25 48 60 72 89 100 105 131 193 307 318 335 353 418 460 482 497 518 526 562 580 658 688 751 762 921 954 1026 1057 1128 1249 1323 1422 1443 1486 1513 1758 1942 2077 2097 2158 3033 3093 3099 3192 3241 3814
MIN087_8
18
E= -1916.39476098 ZPE= 72.83 Gcorr 0.048166000
H 2.148662 -1.555282 -1.719693
Co -0.461032 -0.136193 -0.069629
C -1.920216 -1.011384 -0.709792
C -0.653137 -0.248017 1.710979
O -2.718953 -1.678547 -1.185890
O -0.732601 -0.403258 2.843495
C -0.921718 1.570768 -0.511504
O -1.199754 2.619039 -0.866157
C 2.542616 -0.649859 0.230204
C 2.978856 -1.100636 -1.171903
H 2.188726 -1.508928 0.807244
H 3.387204 -0.190390 0.755492
H 3.351285 -0.252331 -1.753482
H 3.780782 -1.840397 -1.095119

C 1.444027 0.411750 0.160194
O 1.723971 1.585100 0.218209
H 0.132498 -0.276707 -1.419131
H 0.054855 -1.513160 -0.021643
FREQS= 30 49 63 81 90 95 106 210 226 271 292 323 350 400 419 442 478 489 525 534 561 627 658 688 706 811 883 910 1018 1071 1130 1280 1324 1415 1486 1509 1519 1786 2028 2064 2143 2161 2199 3055 3057 3112 3130 3136
MIN088_8
18
E= -1916.39449630 ZPE= 72.80 Gcorr 0.048200000
H 3.494770 -0.686158 0.621841
Co -0.497999 -0.099988 -0.170985
C -1.995421 -1.029004 -0.618288
C -0.036037 -0.818056 1.408734
O -2.842101 -1.667969 -1.047799
O 0.294476 -1.339576 2.373742
C -1.245073 1.512057 0.226984
O -1.727053 2.532444 0.396685
C 2.410128 0.055473 -1.120935
C 3.076812 -1.069047 -0.314061
H 3.148405 0.826940 -1.365984
H 1.990173 -0.345510 -2.047767
H 2.369654 -1.868646 -0.075111
H 3.893315 -1.509725 -0.893009
C 1.299107 0.753921 -0.337461
O 1.498933 1.822027 0.189458
H -0.442443 0.339790 -1.584326
H 0.140965 -1.264485 -0.805581
FREQS= 34 48 63 81 90 94 103 204 212 274 292 324 354 397 421 442 475 491 529 538 563 612 670 679 721 807 879 909 1021 1072 1132 1279 1326 1415 1484 1511 1517 1785 2023 2063 2144 2161 2199 3053 3058 3113 3129 3134
MIN089_8
18
E= -1916.40073801 ZPE= 76.47 Gcorr 0.054595000
H -4.198654 -0.219266 -0.756409
Co 0.494318 -0.110523 0.042054
C 1.892174 -1.149879 0.589083
C 1.343841 0.600925 -1.451165
O 2.734982 -1.758005 1.072991
O 0.743569 0.725797 -2.480682
C 0.628400 1.445196 0.848466
O 0.647577 2.414959 1.470304
C -2.496519 0.173562 0.559251
C -3.832169 -0.516972 0.231683
H -2.636518 1.260740 0.586536
H -2.153230 -0.140955 1.552248
H -3.715500 -1.604428 0.234897
H -4.596788 -0.250808 0.967918
C -1.437147 -0.163252 -0.466796
O -0.812904 -1.319562 -0.406408
H 2.429977 0.825733 -1.366758
H -1.573161 0.270117 -1.466661
FREQS= 49 56 65 70 92 101 123 164 178 232 256 282 371 393 434 455 481 508 515 553 612 635 796 872 893 975 1006 1092 1152 1284 1292 1317 1348 1419 1475 1487 1511 1520 1822 2095 2148 2884 3040 3048 3052 3086 3120 3132
MIN090_8
18
E= -1916.40007611 ZPE= 76.79 Gcorr 0.054074000
H 4.601191 -0.079795 -1.107180
Co -0.854230 0.006490 -0.172672
C -2.436407 -0.808041 -0.409042
C -0.006134 -1.302429 0.655998
O -3.426348 -1.330203 -0.670428
O 0.527235 -2.061679 1.338961
C -1.442856 1.559016 0.421285
O -1.782459 2.510578 0.977633
C 2.939753 0.515044 0.140147
C 4.218487 -0.265810 -0.099779
H 2.552402 0.332440 1.153739
H 3.111953 1.598312 0.040772
H 4.983358 0.032732 0.623530
H 4.039593 -1.338906 0.009425
C 0.776117 0.805609 -0.762895
O 1.968602 0.099315 -0.819719
H 0.954461 1.875483 -0.607856
H 0.231237 0.640069 -1.736140

FREQS= 36 42 58 72 81 91 112 133 240 264 308 324 343 394 441 464 496 509 535 552 563
592 758 825 875 1043 1105 1145 1188 1236 1272 1305 1404 1429 1454 1499 1513 1534 2078 2096 2157 2739 2991
3028 3058 3066 3133 3139
MIN091_8
18
E= -1916.39590558 ZPE= 74.76 Gcorr 0.049928000
H 4.396533 0.553200 0.048544
Co -0.744872 0.149603 -0.017239
C -0.526764 1.718634 -0.816075
C -1.520786 -1.237488 -0.824728
O -0.470338 2.749477 -1.333135
O -2.096287 -2.091078 -1.341450
C -0.958412 0.201320 1.741898
O -1.141860 0.254314 2.879183
C 2.241168 0.443982 -0.096545
C 3.618427 -0.213746 0.058048
H 2.085776 1.199855 0.680528
H 2.190055 1.003112 -1.040208
H 3.714892 -0.754518 1.006555
H 3.854763 -0.899322 -0.765991
C 1.015123 -0.449449 -0.079966
O 1.281150 -1.741867 -0.104940
H -2.163868 0.638150 -0.037379
H 2.239540 -1.926039 -0.119638
FREQS= 13 49 59 63 84 90 94 233 241 317 319 326 351 404 427 458 486 507 527 538 559
632 671 695 704 801 901 1017 1074 1088 1281 1314 1340 1396 1431 1455 1518 1523 1951 2076 2088 2143 3032
3053 3089 3105 3134 3645
MIN092_8
18
E= -1916.40090295 ZPE= 76.45 Gcorr 0.055107000
H 3.425367 -0.652951 -0.931298
Co -0.374514 0.054083 -0.082421
C -1.567946 -1.367946 0.016839
C -0.207054 0.019446 1.666367
O -1.269768 -2.441700 -0.423502
O -0.024675 0.077736 2.802814
C -1.620502 1.321030 -0.502707
O -2.324807 2.210087 -0.671127
C 2.786803 1.180360 0.015587
C 2.670224 -0.328983 -0.198609
H 2.075182 1.533834 0.772009
H 3.788872 1.446184 0.363325
H 2.894778 -0.876223 0.724658
H 2.587700 1.725209 -0.910654
C 1.328471 -0.797058 -0.724891
O 0.668176 -0.065667 -1.592293
H 1.187626 -1.883772 -0.785506
H -2.579038 -1.157255 0.430186
FREQS= 51 64 71 74 79 109 126 164 177 237 275 298 373 394 402 451 480 495 526 587 626
640 785 867 875 958 1016 1107 1114 1287 1294 1320 1370 1417 1474 1490 1508 1516 1822 2093 2146 2883 3012
3042 3060 3073 3111 3140
MIN093_8
18
E= -1916.40065984 ZPE= 76.41 Gcorr 0.054987000
H 2.044889 0.162023 1.833225
Co -0.489253 0.077591 0.101613
C -1.079979 0.114198 -1.661118
C -0.036943 -1.608471 -0.097084
O -0.512222 0.785872 -2.474794
O 0.287400 -2.714521 -0.112574
C -2.145248 0.109242 0.870905
O -3.129520 0.030156 1.453209
C 3.356853 -0.399357 0.182005
C 2.424963 0.584138 0.894857
H 3.724170 0.017972 -0.761735
H 4.227115 -0.630979 0.802502
H 2.990875 1.484737 1.178354
H 2.848657 -1.341193 -0.045891
C 1.262894 1.049428 0.044165
O 0.253162 1.678834 0.601780
H 1.500534 1.318758 -0.994015
H -2.012207 -0.444065 -1.899319
FREQS= 48 63 68 82 84 102 126 165 179 238 272 318 378 389 411 452 481 494 517 534 597
629 784 871 901 957 1042 1105 1151 1276 1291 1318 1337 1430 1472 1486 1515 1519 1822 2091 2148 2884 3013
3047 3051 3067 3120 3129
MIN094_8
18

E= -1916.39577182 ZPE= 75.00 Gcorr 0.050491000
H -4.153671 -0.136263 -0.137716
Co 0.634006 -0.218605 0.161089
C -0.245605 -1.415853 1.127621
C 1.516536 1.160357 0.876152
O -0.745724 -2.223796 1.783352
O 2.147236 1.988125 1.368574
C 1.300650 -0.857241 -1.352730
O 1.768740 -1.296270 -2.310298
C -2.144729 0.111489 -0.932052
C -3.198014 0.284687 0.185027
H -2.014619 -0.948696 -1.154695
H -2.498488 0.601214 -1.854058
H -2.885638 -0.232775 1.094899
H -3.358284 1.339006 0.432261
C -0.817000 0.706275 -0.523994
O -0.778470 2.021595 -0.682035
H -1.601857 2.370458 -1.074310
H 1.789101 -0.946123 0.787321
FREQS= 11 57 64 66 83 95 97 208 231 296 316 328 351 409 441 457 488 515 526 549 588
623 682 706 731 815 919 1014 1071 1106 1250 1316 1331 1394 1425 1501 1511 1523 1950 2078 2091 2145 2995
3057 3123 3139 3149 3638
MIN095_8
18
E= -1916.39930221 ZPE= 76.57 Gcorr 0.054055000
H 2.792239 1.266314 -0.254704
Co -0.504299 -0.024820 0.123972
C -1.730021 0.087933 1.772587
C -1.721895 -0.497743 -1.110389
O -0.509125 0.435760 2.005299
O -2.451953 -0.740558 -1.965736
C 1.067649 -0.924565 -0.105380
O 0.513943 -1.854783 0.457804
C 2.483031 -0.870148 -0.596298
C 3.263478 0.313116 -0.004022
H 2.429923 -0.768829 -1.687699
H 2.967240 -1.825711 -0.367705
H 3.324730 0.238097 1.084731
H 4.280433 0.318650 -0.404763
C -0.030114 1.546547 -0.450457
O 0.248876 2.605448 -0.807771
H -2.077046 -0.911059 2.066663
H -2.508128 0.854910 1.663240
FREQS= 33 34 67 80 86 108 126 162 201 219 273 337 346 395 402 469 475 492 520 552 562
597 712 797 934 1024 1039 1076 1169 1218 1279 1334 1370 1430 1473 1512 1518 1580 1737 2093 2135 3007 3046
3066 3089 3099 3142 3150
MIN096_8
18
E= -1916.39632877 ZPE= 75.50 Gcorr 0.051532000
H -4.045842 0.215797 -0.882968
Co 0.569166 -0.153941 0.001839
C 1.326557 -0.716763 1.512664
C 1.327092 -0.753867 -1.494329
O 1.743563 -1.157744 2.491694
O 1.744550 -1.219137 -2.461861
C 1.121598 1.534540 -0.019155
O 1.443571 2.642250 -0.032996
C -2.363288 -0.854457 0.007507
C -3.818985 -0.381223 0.004204
H -2.158692 -1.491260 0.878143
H -2.159561 -1.505872 -0.852509
H -4.488245 -1.246444 0.011688
H -4.045028 0.230387 0.881607
C -1.273472 0.194628 -0.002128
O -1.846983 1.394980 -0.013895
H -1.172138 2.089922 -0.020407
H 0.107384 -1.556076 0.018683
FREQS= 21 41 57 67 77 91 95 228 232 314 314 340 365 427 435 447 479 495 539 551 570
678 679 740 791 853 913 1028 1072 1091 1276 1281 1332 1398 1429 1453 1509 1515 2034 2091 2102 2143 3045
3060 3070 3131 3138 3780
MIN097_8
18
E= -1916.39787287 ZPE= 76.76 Gcorr 0.053100000
H 3.763081 1.455843 -0.933330
Co -0.825138 0.042124 -0.151838
C -2.509758 -0.267838 -0.687457
C -0.518347 -1.500458 0.657455

O -3.558760 -0.464181 -1.114912
O -0.337543 -2.418703 1.328942
C -1.061090 1.631956 0.572185
O -1.215238 2.590876 1.195552
C 3.178487 -0.251017 0.286756
C 4.170655 0.498390 -0.593892
H 3.598454 -1.205537 0.615314
H 2.924884 0.328857 1.184377
H 5.090172 0.703528 -0.035801
H 4.426338 -0.097342 -1.474689
C 1.010001 0.388956 -0.467364
O 1.978721 -0.592190 -0.421911
H 1.423172 1.382825 -0.275973
H 0.495481 0.368118 -1.487737
FREQS= 14 49 57 72 85 90 107 156 234 243 319 337 377 404 433 467 497 514 526 558 567 603 786 809 870 1014 1101 1115 1199 1228 1258 1334 1403 1428 1452 1501 1508 1526 2077 2096 2155 2589 3032 3050 3093 3105 3124 3136
MIN098_8
18
E= -1916.40006957 ZPE= 75.92 Gcorr 0.055332000
H 1.706152 2.393732 0.914050
Co -0.663078 -0.024227 -0.076648
C -0.653640 1.681558 -0.465249
C 1.038634 -1.692848 -0.280441
O -0.619042 2.799930 -0.718184
O 1.827791 -2.602311 -0.258913
C -2.470115 -0.277447 0.304745
O -3.536414 -0.437120 0.672243
C 2.332062 0.316696 0.632038
C 2.580765 1.824910 0.582166
H 3.264785 -0.214438 0.397875
H 2.067543 0.014281 1.652455
H 3.415383 2.101426 1.233652
H 2.829295 2.155482 -0.432660
C 1.282771 -0.200811 -0.344637
O -0.307145 -1.807458 -0.208625
H -0.479421 0.568896 1.216113
H 1.514996 0.098064 -1.375882
FREQS= 29 60 72 82 94 103 160 216 276 316 335 351 391 406 433 470 494 545 605 655 671 721 769 799 896 960 1009 1077 1114 1140 1200 1276 1311 1387 1429 1492 1513 1518 1854 2125 2141 2198 3031 3042 3044 3070 3113 3118
MIN099_8
18
E= -1916.39894298 ZPE= 75.80 Gcorr 0.054365000
H -3.829497 -0.163950 0.523647
Co 0.215063 -0.008575 0.079081
C 0.942677 -0.976811 1.763802
C 1.908230 0.188882 -0.578751
O 0.812672 0.285623 1.945588
O 2.956519 0.314446 -1.019467
C -0.349887 1.638002 -0.412655
O -0.735798 2.669958 -0.729406
C -1.693072 -0.269223 0.824196
C -2.909183 0.022349 -0.045648
H -1.652081 0.383515 1.699043
H -1.741715 -1.301598 1.181722
H -2.949178 1.061659 -0.385542
H -2.949730 -0.616787 -0.934039
C -0.254866 -1.349114 -0.967878
O -0.617728 -2.217209 -1.630435
H 1.942247 -1.407225 1.603833
H 0.184529 -1.671149 2.147526
FREQS= 50 63 76 82 101 113 122 183 206 246 278 320 361 397 410 411 467 479 499 507 522 540 544 717 726 953 992 1053 1064 1212 1219 1269 1390 1431 1487 1510 1514 1589 2105 2129 2172 2996 3030 3065 3081 3091 3112 3137
MIN100_8
18
E= -1916.39643166 ZPE= 75.80 Gcorr 0.052125000
H 3.466449 -0.466154 -1.047560
Co -0.451732 -0.142020 0.064518
C -1.358311 -0.214645 1.598109
C -1.322155 1.156187 -0.783742
O -1.857036 -0.306478 2.631829
O -1.855447 2.014507 -1.339385
C -0.783802 -1.495985 -1.044844
O -0.914036 -2.419950 -1.720112
C 2.466511 0.165123 0.792633

C 3.170714 -0.891312 -0.084011
H 2.183039 -0.287450 1.746537
H 3.153809 0.997103 0.986750
H 4.072001 -1.249609 0.421241
H 2.516328 -1.746493 -0.270375
C 1.221425 0.689526 0.118659
O 1.546857 1.854485 -0.443789
H 0.789395 2.222064 -0.923370
H 0.286753 -1.228804 0.735625
FREQS= 26 37 51 62 79 89 102 206 222 308 330 351 360 426 442 463 479 535 549 558 587 619 714 741 816 862 929 1021 1075 1109 1246 1319 1347 1371 1416 1494 1508 1521 2041 2093 2106 2145 3051 3057 3113 3133 3143 3775
MIN101_8
18
E= -1916.39176279 ZPE= 72.23 Gcorr 0.047511000
H -4.499442 0.000006 -0.269516
Co 0.727332 -0.000051 -0.415443
C 0.623400 1.736746 -0.727682
C 1.717570 -0.000127 1.106440
O 0.580344 2.842381 -1.027301
O 2.397641 -0.000201 2.022737
C 0.623058 -1.736834 -0.727614
O 0.579770 -2.842471 -1.027198
C -2.334432 0.000081 -0.381030
C -3.641964 0.000112 0.409890
H -2.264768 -0.870819 -1.046573
H -2.264793 0.870897 -1.046685
H -3.710886 -0.880759 1.053262
H -3.710966 0.881108 1.053082
C -1.082264 0.000159 0.496117
O -1.156080 0.000335 1.701363
H 0.126203 -0.000046 -1.762171
H 2.041088 -0.000192 -1.177980
FREQS= 37 55 57 85 88 99 102 198 201 271 321 345 374 394 428 448 474 490 510 559 563 636 645 681 709 764 783 904 1008 1067 1096 1277 1345 1423 1457 1505 1512 1789 1843 2049 2135 2164 2200 3036 3060 3067 3133 3141
MIN102_8
18
E= -1916.39848548 ZPE= 75.96 Gcorr 0.054907000
H -3.778361 -0.462985 0.394522
Co 0.171392 -0.018256 0.008167
C 0.182450 -0.998203 1.827964
C 1.876676 0.599021 0.262062
O -0.189455 0.224962 1.942978
O 2.935228 1.004166 0.412957
C -0.434790 1.463913 -0.824833
O -0.847076 2.349608 -1.425608
C -1.757667 -0.702195 -0.323876
C -2.794579 0.003131 0.542313
H -1.750688 -1.774456 -0.106613
H -2.006335 -0.595327 -1.383813
H -2.547419 -0.050812 1.603934
H -2.895081 1.062745 0.286015
C 0.593456 -1.327338 -1.102155
O 0.817769 -2.181341 -1.838814
H 1.193450 -1.295798 2.140963
H -0.568157 -1.799583 1.823544
FREQS= 56 64 72 88 94 110 119 195 234 256 281 333 357 397 409 418 456 473 495 503 517 541 552 724 740 978 987 1044 1070 1220 1244 1278 1384 1422 1489 1501 1515 1589 2110 2130 2174 2997 3026 3059 3077 3094 3110 3150
MIN103_8
18
E= -1916.39101575 ZPE= 74.82 Gcorr 0.048722000
H -3.133741 0.882701 -0.018719
Co 0.092075 -0.000282 -0.071992
C 2.408542 0.004812 1.998658
C -0.502877 -1.580735 -0.577397
O 1.218445 0.003346 1.735652
O -1.000716 -2.541541 -0.981075
C 1.534752 -0.002499 -1.117409
O 2.465528 -0.003908 -1.796572
C -1.526936 0.002483 1.173019
C -2.939815 0.001866 0.601976
H -1.374412 0.883604 1.805660
H -1.375035 -0.876268 1.809100
H -3.134526 -0.881457 -0.014924
H -3.685543 0.003924 1.408483

C -0.502738 1.578027 -0.584129
O -1.000484 2.537148 -0.991918
H 3.180551 0.004266 1.211241
H 2.748931 0.006750 3.045920
FREQS= 42 46 66 70 83 99 104 118 126 166 226 257 271 296 331 394 404 446 505 510 521 543 563 572 732 953 986 1054 1175 1229 1261 1267 1429 1476 1513 1516 1532 1766 2063 2076 2135 2979 3025 3042 3073 3088 3088 3118
MIN104_8
18
E= -1916.39037954 ZPE= 72.39 Gcorr 0.048360000
H -4.079873 -0.000001 1.544863
Co 0.846075 0.000000 -0.227520
C 1.065196 0.000000 1.562491
C 0.879982 -1.731838 -0.585668
O 1.312713 -0.000001 2.680589
O 0.973666 -2.834417 -0.882978
C 0.879981 1.731838 -0.585667
O 0.973665 2.834418 -0.882977
C -2.026790 0.000000 0.848694
C -3.534018 -0.000001 0.596800
H -1.715823 0.873101 1.438456
H -1.715823 -0.873102 1.438456
H -3.834879 0.881030 0.024416
H -3.834879 -0.881031 0.024416
C -1.182509 0.000000 -0.428428
O -1.693962 0.000000 -1.518481
H 2.359177 0.000001 -0.249969
H 0.958389 0.000001 -1.696149
FREQS= 38 61 71 89 90 101 106 198 202 269 329 348 376 393 445 462 468 498 512 544 559 632 649 680 731 754 788 901 1008 1067 1098 1280 1339 1424 1462 1506 1511 1805 1864 2063 2139 2151 2191 3031 3061 3064 3134 3143
MIN105_8
18
E= -1916.39258278 ZPE= 75.05 Gcorr 0.050702000
H 2.894810 0.866830 -0.881584
Co -0.216440 0.068493 0.000003
C -0.475706 -3.030317 0.000134
C 0.210062 0.748461 1.569724
O -0.965127 -1.913008 0.000007
O 0.576735 1.292160 2.520643
C -1.896694 0.702069 -0.000009
O -2.957244 1.143432 -0.000013
C 1.660917 -0.706460 0.000035
C 2.875054 0.218306 -0.000022
H 1.703192 -1.358058 -0.883199
H 1.703210 -1.357965 0.883337
H 2.894822 0.866921 0.881474
H 3.810123 -0.357917 0.000002
C 0.210049 0.748325 -1.569785
O 0.576710 1.291939 -2.520756
H 0.613104 -3.188736 0.000300
H -1.126044 -3.918864 0.000087
FREQS= 47 53 76 81 96 106 107 146 157 188 236 257 273 305 334 401 410 438 501 512 517 542 562 568 750 955 987 1052 1169 1227 1267 1273 1432 1483 1513 1516 1528 1759 2062 2087 2147 2994 3004 3026 3052 3089 3090 3119
MIN106_8
18
E= -1916.39727871 ZPE= 76.07 Gcorr 0.055628000
H -3.152143 -0.126414 0.896712
Co 0.193597 0.019558 -0.063365
C -0.553294 -0.756698 1.332707
C 1.901800 -0.540961 0.297227
O -1.047340 -1.227812 2.258329
O 2.972898 -0.879010 0.511183
C 0.521542 1.776010 0.224047
O 0.658023 2.890075 0.457171
C -1.710481 0.666752 -0.554130
C -2.939538 -0.156313 -0.176063
H -1.576214 0.706954 -1.637591
H -1.856510 1.691050 -0.201814
H -3.823136 0.247497 -0.688570
H -2.854910 -1.209092 -0.466655
C 0.025226 -1.363500 -1.592876
O 0.531818 -0.254096 -1.995894
H -1.032224 -1.584380 -1.780674
H 0.673289 -2.238676 -1.442346

```
FREQS=   55   59   82   88  115  120  132  206  229  266  293  330  353  395  406  414  457  477  492  507  515
546  557  716  731  978  998 1039 1059 1218 1228 1295 1383 1425 1489 1509 1514 1587 2109 2132 2175 3004 3026
3068 3088 3093 3111 3139
MIN107_8
18
E= -1916.39040529 ZPE=    74.82 Gcorr    0.048760000
H -3.502975 -1.448831 -0.582134
Co 0.072886 0.179026 -0.021631
C 0.336500 1.190502 -1.446734
C -0.804166 0.743611 1.394052
O 0.409655 1.971562 -2.293475
O -1.392626 1.276453 2.233714
C 1.699160 0.406846 0.679402
O 2.751062 0.533384 1.131814
C -1.767911 -0.166840 -0.823740
C -2.600251 -1.166006 -0.022528
H 2.288923 -2.316045 0.290560
H -1.555236 -0.582273 -1.815102
H -2.048651 -2.089299 0.180428
H -2.929160 -0.757079 0.938144
C 1.355827 -2.647905 -0.193990
O 0.419682 -1.888934 -0.379555
H 1.292521 -3.700056 -0.512012
H -2.330482 0.758917 -0.974607
FREQS=   41   46   63   70   86   98  105  116  138  165  218  265  274  303  326  399  408  447  493  509  523
530  567  572  705  974  988 1040 1171 1238 1267 1279 1420 1473 1506 1516 1530 1761 2064 2076 2134 2981 3019
3048 3076 3087 3100 3124
MIN108_8
18
E= -1916.39573043 ZPE=    75.99 Gcorr    0.054775000
H 3.812152 0.551745 -0.242714
Co -0.188219 0.020579 -0.042585
C 0.275456 -1.370960 -1.529881
C -1.885447 -0.645044 -0.004342
O 0.088345 -0.196698 -1.999203
O -2.952836 -1.048363 0.090509
C -0.719429 1.731972 0.167318
O -1.015363 2.823925 0.354077
C 1.700874 0.876057 0.021601
C 2.880926 -0.026332 -0.315105
H 1.852402 1.342564 0.999532
H 1.635714 1.673073 -0.722976
H 2.979367 -0.879330 0.364904
H 2.826497 -0.401862 -1.340077
C 0.382586 -0.796969 1.416145
O 0.803938 -1.274088 2.374776
H -0.513444 -2.131742 -1.631739
H 1.286741 -1.760640 -1.372821
FREQS=   54   58   70   85   95  114  120  183  222  260  328  330  356  399  401  420  447  489  490  498  518
540  546  710  716  988  994 1043 1077 1224 1248 1290 1400 1419 1474 1505 1520 1599 2105 2125 2168 2997 3027
3061 3087 3102 3114 3135
MIN109_8
18
E= -1916.39319021 ZPE=    75.40 Gcorr    0.052243000
H -3.406090 -0.631111 -1.026674
Co 0.446030 -0.136555 0.076648
C 0.809578 -1.545931 -0.951217
C 1.220556 1.153294 -0.901019
O 0.957638 -2.510171 -1.564529
O 1.728428 1.969034 -1.528901
C 1.437698 -0.082925 1.556266
O 1.988182 -0.092513 2.568215
C -2.466647 0.144313 0.794236
C -3.129257 -0.979821 -0.026937
H -3.194079 0.960159 0.950827
H -2.194801 -0.240492 1.780737
H -2.447397 -1.825348 -0.138060
H -4.035652 -1.326539 0.477273
C -1.218313 0.671517 0.120533
O -1.436319 1.837536 -0.494202
H -2.350326 2.151603 -0.357635
H -0.239597 -1.195073 0.848201
FREQS=   37   40   51   66   87   92  105  205  219  310  327  344  367  430  440  460  481  528  549  555  577
600  667  737  809  867  931 1019 1069 1124 1246 1301 1332 1380 1419 1499 1507 1522 2030 2089 2119 2156 2968
3057 3107 3130 3151 3635
MIN110_8
18
```

E= -1916.38822748 ZPE= 72.20 Gcorr 0.047342000
H -3.991952 -0.881392 0.165267
Co 0.679856 0.000005 -0.046954
C 0.476658 -1.746707 -0.256016
C 0.475828 1.746552 -0.256478
O 0.366625 -2.874064 -0.429891
O 0.365265 2.873814 -0.430643
C 2.432909 0.000531 0.394504
O 3.520030 0.000891 0.749973
C -2.185726 0.000067 0.998287
C -3.691547 0.000240 0.737208
H -1.869908 0.868794 1.589057
H -1.870178 -0.868450 1.589507
H -4.239481 0.000582 1.683931
H -3.991666 0.881665 0.164798
C -1.352271 -0.000415 -0.277021
O -1.835593 -0.001008 -1.373971
H 0.862230 -0.000142 -1.544478
H 0.479129 0.000156 1.453039
FREQS= 34 42 67 93 93 98 109 206 211 259 259 335 372 388 441 464 477 527 536 540 552
600 667 681 686 783 796 903 1007 1074 1096 1270 1342 1425 1454 1507 1512 1828 1833 1993 2134 2150 2192
3052 3061 3082 3135 3142
MIN111_8
18
E= -1916.39305692 ZPE= 75.25 Gcorr 0.052215000
H 4.473232 1.296289 0.000090
Co -0.566902 0.158812 0.000017
C -1.365874 0.709371 1.493176
C -1.365847 0.709662 -1.493052
O -1.804401 1.150195 2.463725
O -1.804359 1.150657 -2.463531
C -1.049330 -1.567271 -0.000154
O -1.372698 -2.669118 -0.000253
C 2.350145 0.883226 0.000057
C 3.814582 0.424290 0.000064
H 2.151373 1.523919 0.867009
H 2.151404 1.523994 -0.866848
H 4.072184 -0.160380 0.891957
H 4.072212 -0.160338 -0.891850
C 1.257919 -0.176469 -0.000013
O 1.727187 -1.422852 -0.000112
H -0.131159 1.572486 0.000197
H 2.701714 -1.451791 -0.000122
FREQS= 32 45 61 72 91 92 93 241 250 317 321 339 359 431 438 443 480 502 531 552 560
606 668 724 792 860 907 1019 1067 1088 1279 1289 1324 1390 1427 1455 1522 1523 2026 2085 2116 2153 3031
3063 3084 3102 3129 3657
MIN112_8
18
E= -1916.39126020 ZPE= 75.37 Gcorr 0.050642000
H -4.055698 0.732712 0.642074
Co 0.563987 -0.033827 -0.106793
C 1.086793 1.350684 -1.096504
C 0.197178 0.799707 1.431556
O 1.390838 2.191335 -1.823137
O -0.022850 1.342127 2.422855
C 1.899933 -1.094037 0.404815
O 2.735316 -1.845963 0.656200
C -2.345949 -0.606144 0.490058
C -3.159248 0.621141 0.026114
H -2.063537 -0.481593 1.537344
H -2.979817 -1.507590 0.435061
H -2.564766 1.533943 0.112363
H -3.472028 0.521071 -1.017915
C -1.107939 -0.790542 -0.366803
O -1.363321 -1.557510 -1.426843
H 0.842601 -0.713991 -1.380709
H -2.278888 -1.895991 -1.412841
FREQS= 16 31 44 63 88 92 109 206 223 308 322 349 363 429 443 462 481 532 541 561 572
606 672 732 813 852 918 1012 1070 1114 1247 1313 1320 1389 1421 1505 1508 1526 2053 2092 2115 2148 2979
3055 3117 3134 3143 3634
MIN113_8
18
E= -1916.38778557 ZPE= 72.50 Gcorr 0.048350000
H 2.438230 0.376596 1.084035
Co -0.640966 0.411392 -0.230100
C -0.334778 0.933681 1.467475
C 0.465329 1.463495 -1.124038

O -0.215212 1.340679 2.531410
O 1.100903 2.175201 -1.758315
C -1.973685 -0.731581 -0.013343
O -2.889249 -1.416595 0.060484
C 1.524365 -1.583057 0.711000
C 2.737726 -0.655896 0.878124
H 1.858432 -2.606001 0.505092
H 0.923171 -1.587999 1.626755
H 3.358061 -0.992493 1.713359
H 3.354575 -0.657197 -0.025240
C 0.643395 -1.156229 -0.469117
O 0.720607 -1.724840 -1.527563
H -1.649611 1.538682 -0.221897
H -1.087278 0.202774 -1.618122
FREQS= 31 61 67 84 92 97 104 188 214 295 309 348 388 398 441 461 496 499 536 550 562
621 637 654 728 760 808 895 1011 1063 1123 1274 1316 1419 1487 1509 1517 1802 1866 2061 2139 2149 2191
3048 3054 3101 3126 3134
MIN114_8
18
E= -1916.39022516 ZPE= 76.32 Gcorr 0.054698000
H -3.833010 1.373735 0.899724
Co 0.589275 -0.063056 -0.171789
C 1.074275 -0.899006 1.281681
C 0.181644 -1.592452 -1.094116
O 1.294994 -1.422450 2.285604
O 1.077121 -1.427098 -1.898801
C 1.577376 1.480206 0.064009
O 2.284153 2.376867 0.132316
C -2.392545 -0.231896 0.522270
C -3.752521 0.457976 0.304462
H -2.289920 -0.521320 1.574869
H -2.344045 -1.142051 -0.083756
H -3.884475 0.725674 -0.747756
H -4.572613 -0.204782 0.596856
C -1.261394 0.697023 0.140984
O -0.885209 0.827618 -1.089031
H -0.557142 -2.412800 -1.164522
H -1.158713 1.573450 0.806458
FREQS= 49 57 72 80 90 102 146 163 182 226 235 260 374 393 424 469 482 496 524 544 573
641 796 879 891 961 1002 1092 1153 1252 1285 1331 1348 1417 1485 1510 1513 1523 1738 2097 2159 2960 2965
3048 3050 3099 3121 3133
MIN115_8
18
E= -1916.38829206 ZPE= 76.51 Gcorr 0.052871000
H 2.886207 0.132765 -1.483487
Co -0.230079 0.222010 0.046467
C -1.425996 1.409687 -0.254692
C -1.640394 -1.049764 0.168493
O -2.209895 2.220964 -0.499436
O -1.671532 -1.685022 1.211379
C 1.052139 1.402405 0.391885
O 1.833033 2.171160 0.745862
C 2.526745 -1.391284 0.005956
C 3.428006 -0.473964 -0.753775
H 0.638062 -2.255888 0.413358
H 3.018222 -2.060587 0.712867
H 3.960310 0.197734 -0.069427
H 4.194383 -1.053731 -1.282073
C 1.189775 -1.479841 -0.103896
O -2.551622 -1.270169 -0.810617
H 0.679822 -0.981266 -0.967259
H -3.146401 -1.972199 -0.479918
FREQS= 25 37 67 86 95 104 116 148 174 185 232 286 321 362 413 462 477 501 524 586 619
672 683 736 933 955 993 1075 1120 1135 1217 1313 1318 1412 1440 1484 1496 1677 1738 2081 2133 2779 3039
3094 3133 3156 3233 3652
MIN116_8
18
E= -1916.39092486 ZPE= 76.82 Gcorr 0.055634000
H 3.770302 -0.764064 -1.573503
Co -0.626356 0.293172 -0.031897
C 0.178079 -0.645010 1.416092
C 0.592319 1.662592 -0.052556
O 0.024745 -0.512665 2.587535
O 1.316144 2.535216 -0.183269
C -1.748807 -0.988553 -0.544277
O -2.458791 -1.773969 -0.985061
C 2.310850 -0.906829 0.035164

C 2.688720 -0.875212 -1.448940
H 2.861807 -1.714346 0.536756
H 2.628346 0.022228 0.521574
H 2.386610 -1.800675 -1.950594
H 2.198672 -0.043587 -1.964867
C 0.820933 -1.139074 0.269438
O -1.846425 1.576423 -0.429648
H 0.463587 -2.116197 -0.051699
H -2.735649 1.253469 -0.622449
FREQS= 30 46 74 91 96 123 144 181 229 268 287 320 366 395 426 447 462 482 494 533 575
621 651 789 856 906 946 1003 1078 1093 1134 1280 1328 1383 1427 1497 1508 1516 1977 2120 2186 3028 3046
3077 3118 3123 3145 3831
MIN117_8
18
E= -1916.39220867 ZPE= 78.20 Gcorr 0.057768000
H -4.354924 0.054762 -0.954326
Co 0.888743 -0.093777 -0.105104
C -0.813480 0.776583 -0.008518
C 2.310996 -1.152543 0.084457
O -1.320889 1.828015 0.314923
O 3.202094 -1.797365 0.427155
C 1.739466 1.383412 -0.052283
O 2.290780 2.397495 -0.068585
C -2.863932 -0.839271 0.369129
C -4.044233 0.101638 0.095516
H -2.597194 -0.816718 1.431899
H -3.139924 -1.876458 0.132468
H -4.904799 -0.175747 0.710675
H -3.768822 1.133374 0.323292
C -1.640338 -0.460148 -0.446161
O -0.535089 -1.435449 -0.260010
H -0.585295 -2.199762 -0.849077
H -1.871150 -0.387075 -1.517824
FREQS= 20 34 72 83 96 129 182 235 249 282 306 339 352 411 447 460 478 491 586 605 626
652 810 865 929 966 1030 1087 1148 1154 1262 1319 1346 1378 1426 1501 1511 1530 1798 2078 2135 3024 3040
3050 3082 3119 3153 3823
MIN118_8
18
E= -1916.38359230 ZPE= 72.85 Gcorr 0.049429000
H -1.900891 0.525335 1.982556
Co 0.464160 -0.067731 -0.253694
C 0.906216 1.643800 -0.029503
C 0.070889 -1.205676 1.059592
O 1.162775 2.745548 0.175334
O -0.137064 -1.871833 1.974051
C 2.154554 -0.645861 -0.408542
O 3.240422 -1.003076 -0.487256
C -1.498113 0.627036 -0.166071
C -1.924276 1.251049 1.162559
H -1.568586 1.349109 -0.984554
H 0.224173 -0.097480 -1.915151
H -1.282426 2.087732 1.448375
H -2.949545 1.637170 1.097111
C -2.322529 -0.562540 -0.528546
O -2.604839 -0.910456 -1.660052
H 0.013290 -0.841832 -1.677150
H -2.679128 -1.159614 0.344995
FREQS= 44 59 82 92 103 106 125 205 220 241 276 331 356 369 413 439 467 471 491 512 543
561 570 594 630 894 911 980 1024 1086 1138 1185 1327 1429 1435 1446 1506 1515 1791 2100 2117 2169 2860
3034 3089 3101 3131 3391
MIN119_8
18
E= -1916.38772728 ZPE= 75.73 Gcorr 0.053585000
H 2.991184 0.354368 0.304683
Co -0.222160 -0.123865 -0.010028
C -1.680569 -1.565125 0.403794
C -1.372272 1.093038 0.619336
O -1.606221 -1.269969 -0.846031
O -2.085354 1.896184 1.024908
C 0.292229 0.641168 -1.596373
O 0.549744 1.081002 -2.620740
C 1.089628 0.879798 1.272727
C 2.431826 1.234333 0.637812
H 1.227596 0.262011 2.163458
H 0.606701 1.797373 1.617221
H 2.314543 1.896381 -0.226596
H 3.064418 1.763753 1.362791

C 0.983611 -1.424342 0.233811
O 1.775646 -2.234180 0.420211
H -1.246438 -2.503230 0.776107
H -2.496905 -1.163820 1.019659
FREQS= 40 65 76 81 102 110 125 147 168 250 263 329 348 403 411 436 439 465 480 498 511 535 581 725 745 968 1001 1046 1075 1212 1241 1284 1373 1428 1492 1509 1511 1593 2107 2130 2170 3002 3027 3071 3073 3091 3105 3128
MIN120_8
18
E= -1916.38118111 ZPE= 73.86 Gcorr 0.047268000
H 4.326915 0.473367 -1.161529
Co -0.773702 0.102267 0.102935
C -1.004598 1.786448 -0.423489
C -1.827097 -1.155558 -0.600029
O -1.248676 2.882311 -0.680104
O -2.660731 -1.876440 -0.940512
C -0.139496 -0.096948 1.767306
O 0.166782 -0.167340 2.873962
C 2.227901 0.482001 -0.719775
C 3.652610 0.264000 -0.321741
H 1.924252 1.480807 -1.017984
H 0.329220 -0.258213 -1.292734
H 3.936388 0.948082 0.487141
H 3.821960 -0.761748 0.009205
C 1.283644 -0.473183 -0.749237
O 1.572933 -1.789465 -0.507218
H -2.002944 0.382982 0.816086
H 0.753896 -2.259565 -0.306651
FREQS= 32 48 58 64 79 90 109 118 173 210 225 266 310 346 353 439 440 455 495 512 521 531 662 702 709 809 932 1044 1060 1098 1183 1247 1276 1387 1429 1496 1499 1724 2080 2094 2105 2159 2784 3037 3085 3154 3203 3833
MIN121_8
18
E= -1916.38326756 ZPE= 72.92 Gcorr 0.049543000
H -1.638808 -0.827328 2.478284
Co 0.575698 -0.033255 0.339905
C 2.283576 -0.517007 0.087089
C -0.081787 -1.412003 -0.599360
O 3.373489 -0.831885 -0.074888
O -0.486951 -2.264238 -1.251225
C 0.844085 1.633350 -0.240576
O 1.027084 2.697206 -0.636478
C -1.395949 0.491044 0.752512
C -2.146944 -0.575662 1.543093
H -1.283425 1.407551 1.343326
H 0.588505 -0.613061 1.896713
H -3.149947 -0.211945 1.799133
H -2.282041 -1.492951 0.965910
C -2.054513 0.840112 -0.536270
O -2.851525 0.133249 -1.125512
H -1.757680 1.824429 -0.966101
H 0.791950 0.177531 1.971177
FREQS= 44 58 82 92 100 105 113 192 243 265 272 288 358 368 435 449 457 479 484 517 558 564 597 633 698 889 922 964 1001 1084 1113 1168 1370 1425 1438 1497 1499 1508 1790 2108 2125 2173 2881 3041 3062 3105 3142 3253
MIN122_8
18
E= -1916.38026663 ZPE= 73.90 Gcorr 0.046672000
H 3.966460 -0.714352 -0.173298
Co -0.796881 0.113558 0.095694
C -1.170767 1.665421 -0.686789
C -1.713744 -1.359203 -0.345281
O -1.530163 2.685411 -1.087785
O -2.432271 -2.241743 -0.519900
C -0.131929 0.199753 1.752333
O 0.202279 0.291228 2.851053
C 2.216638 0.498228 -0.638318
C 3.646629 0.332252 -0.220411
H 1.869392 1.507766 -0.830368
H 0.341412 -0.282051 -1.288165
H 4.315371 0.841166 -0.923361
H 3.823629 0.775992 0.767119
C 1.312187 -0.484555 -0.783661
O 1.500097 -1.825588 -0.635496
H -2.039873 0.411675 0.771487
H 2.365775 -1.992092 -0.237374

FREQS= 26 48 58 63 73 83 93 106 172 203 263 295 312 340 344 434 443 455 493 513 522
529 657 689 708 794 923 1046 1049 1106 1187 1234 1347 1393 1434 1486 1510 1713 2081 2089 2107 2160 2862
3034 3093 3105 3212 3808
MIN123_8
18
E= -1916.38201457 ZPE= 72.82 Gcorr 0.049267000
H 3.051566 -1.354598 -1.590487
Co -0.528280 -0.081288 -0.339074
C -2.307685 -0.088252 -0.116894
C -0.266185 -1.415028 0.812430
O -3.444309 -0.104916 0.029213
O -0.107026 -2.248089 1.589563
C -0.387516 1.686965 -0.013906
O -0.344831 2.806541 0.225413
C 1.511424 -0.101494 -0.709216
C 1.996559 -1.428533 -1.295368
H 1.661972 0.713117 -1.423870
H -0.665030 -0.836707 -1.808747
H 1.914978 -2.253727 -0.579925
H 1.434416 -1.714479 -2.188720
C 2.208485 0.262324 0.556301
O 2.457195 1.395206 0.924025
H -0.650872 -0.036799 -1.992804
H 2.497812 -0.607873 1.193757
FREQS= 40 55 84 94 102 112 113 212 214 243 273 332 356 371 417 447 454 465 483 511 558
563 588 620 646 911 927 978 1022 1082 1145 1179 1330 1429 1435 1503 1505 1513 1794 2105 2129 2174 2858
3030 3084 3096 3120 3239
MIN124_8
18
E= -1916.37948924 ZPE= 71.80 Gcorr 0.046814000
H 4.409542 0.278809 -0.270727
Co -0.578557 0.027201 -0.207312
C -2.238873 -0.537440 -0.505291
C -0.192872 -1.343865 0.895868
O -3.304218 -0.913080 -0.700909
O 0.033794 -2.093885 1.731946
C -1.097012 1.709003 0.273648
O -1.344660 2.746388 0.687817
C 2.335779 -1.197091 -0.917553
C 3.501177 0.793925 0.048620
H 3.303907 -1.619306 -1.156014
H 1.471269 -1.795363 -1.175865
H 3.465431 1.779890 -0.427127
H 3.547112 0.960163 1.130334
C 2.261020 0.003466 -0.302073
O 1.159290 0.667262 0.074830
H -0.315211 0.282667 -1.950750
H -0.029958 -0.442751 -1.861209
FREQS= 44 58 72 81 92 105 135 178 198 279 290 320 337 393 401 429 444 457 468 496 520
545 569 575 642 702 719 772 882 1004 1047 1071 1142 1316 1414 1444 1485 1504 1668 2127 2132 2184 3046
3106 3144 3186 3265 3739
MIN125_8
18
E= -1916.37866838 ZPE= 74.23 Gcorr 0.046079000
H 3.294116 -0.036419 -0.091453
Co 0.006614 0.192283 0.056047
C -1.607330 -1.080336 -1.670472
C -0.000441 -0.992370 1.366804
O -2.754470 -1.213671 -1.334718
O 0.122439 -1.701878 2.264773
C -1.311420 1.197871 0.717788
O -2.140270 1.854932 1.162356
C 1.441506 -0.983014 -0.769928
C 2.818910 -1.022384 -0.108523
H 1.547033 -0.656917 -1.812553
H 1.028245 -1.999529 -0.800505
H 2.766646 -1.375999 0.926151
H 3.492803 -1.698906 -0.649593
C 0.999281 1.539236 -0.517796
O 1.737415 2.383755 -0.777230
H -1.043750 -0.108777 -1.504557
H -1.027619 -1.854213 -2.209463
FREQS= 37 38 45 72 78 82 98 99 113 135 211 250 251 330 382 406 431 459 492 519 526
545 572 583 742 963 987 1042 1193 1225 1239 1269 1432 1477 1493 1513 1515 1838 2085 2109 2166 2603 2966
3015 3032 3060 3093 3117
MIN126_8
18

E= -1916.38514534 ZPE= 76.12 Gcorr 0.053675000
H -0.335038 -1.039174 -1.943123
Co 0.309948 -0.004246 0.039627
C 0.480510 -0.433024 2.009444
C 0.380080 1.687643 -0.366819
O 0.212791 -1.470381 1.288814
O 0.461410 2.825939 -0.533160
C -1.580029 0.202595 0.262775
O -2.240005 0.586892 1.193030
C -2.211024 -0.369509 -1.020170
C -1.332004 -1.437100 -1.689019
H -2.356653 0.482648 -1.697039
H -3.202011 -0.757397 -0.762722
H -1.197379 -2.305883 -1.040770
H -1.766644 -1.777560 -2.633071
C 2.059739 -0.420766 -0.437076
O 3.133024 -0.649252 -0.760673
H -0.324525 0.104287 2.522754
H 1.492245 -0.316886 2.425164
FREQS= 38 50 63 75 93 99 116 145 209 226 275 310 368 386 393 408 467 511 535 540 565
608 648 776 879 1014 1054 1081 1117 1223 1266 1314 1367 1425 1465 1499 1515 1577 1821 2096 2155 2977 3000
3042 3091 3106 3110 3144
MIN127_8
18
E= -1916.38954061 ZPE= 78.07 Gcorr 0.058379000
H -4.248810 -1.137112 0.021178
Co 0.889886 0.012599 -0.512913
C 1.963705 -1.319300 -0.119447
C 1.412132 1.650103 -0.183961
O 2.776299 -2.049026 0.261254
O 1.905513 2.642375 0.151172
C -0.207378 -0.167226 0.890951
O -0.169362 -0.098275 2.088946
C -2.681397 0.310557 0.467825
C -3.980879 -0.105706 -0.229412
H -2.452094 1.367058 0.266688
H -2.778275 0.226062 1.555188
H -4.806708 0.541116 0.079757
H -3.900967 -0.042256 -1.319812
C -1.476869 -0.531403 0.070099
O -1.114995 -0.388048 -1.336306
H -1.676817 -1.597932 0.219321
H -1.518781 0.424526 -1.670542
FREQS= 31 66 73 82 102 110 168 216 220 279 293 330 338 385 417 456 471 506 544 556 590
695 778 858 947 982 1056 1092 1112 1242 1261 1326 1353 1396 1432 1497 1512 1517 1840 2051 2112 3026 3048
3080 3097 3120 3129 3816
MIN128_8
18
E= -1916.38508323 ZPE= 76.41 Gcorr 0.054633000
H 1.555854 1.534117 -0.907486
Co -0.365947 0.013939 -0.079786
C -0.708900 1.688041 0.356316
C -2.082078 -0.493635 -0.066211
O -0.871236 2.740169 0.801526
O -3.183178 -0.822955 -0.083866
C 0.193951 -1.209989 1.059565
O 0.561226 -1.853086 1.947031
C 2.724080 0.404308 0.520292
C 2.606289 -0.391048 -0.760443
H 2.626376 -0.144987 1.451691
H 3.466471 1.196961 0.559950
H 3.270255 -0.130093 -1.582281
H 2.426610 -1.459190 -0.662167
C 1.513144 0.602523 -0.341381
O -0.176070 -0.464090 -2.237553
H 0.793751 -0.395807 -2.262766
H -0.383607 -1.378878 -2.468663
FREQS= 46 50 79 84 99 107 121 176 217 241 255 300 318 399 414 437 482 495 515 528 537
574 583 608 813 830 873 908 1039 1054 1092 1153 1204 1232 1294 1488 1520 1635 2061 2087 2148 3103 3115
3138 3194 3219 3710 3871
MIN129_8
18
E= -1916.37506227 ZPE= 71.84 Gcorr 0.045199000
H 4.678405 -0.836515 -1.039366
Co -0.901255 -0.018457 0.363855
C -2.578617 -0.300045 -0.154278
C -0.839812 1.674745 -0.259713

```
O -3.640407 -0.485394 -0.545490
O -0.664813 2.727240 -0.678049
C -0.287927 -1.604815 -0.248477
O 0.228749 -2.541148 -0.657300
C 3.089689 -0.212435 0.286389
C 4.306963 0.070343 -0.544136
H -1.339375 0.304064 2.073871
H 3.173047 -0.949226 1.083566
H 4.096617 0.812060 -1.322074
H 5.136790 0.456265 0.063121
C 1.918096 0.423660 0.131879
O 0.869465 0.262191 0.960891
H -1.211996 -0.468208 2.072816
H 1.806097 1.168075 -0.666429
FREQS=   38   43   54   85   91   93  119  143  212  248  280  314  346  352  367  417  433  448  473  486  491
508  566  571  648  712  830  937  968 1062 1129 1163 1216 1292 1376 1429 1491 1511 1712 2123 2129 2180 3016
3055 3061 3102 3165 3769
MIN130_8
18
E= -1916.38052122 ZPE=    75.26 Gcorr    0.050670000
H 3.700305 1.484456 -0.692517
Co -0.806860 0.024993 -0.197481
C 0.927499 -0.239127 -0.715508
C -0.450944 -0.149912 1.551170
O 1.957699 -0.476889 0.078027
O -0.214000 -0.262195 2.668817
C -2.032862 -1.250423 -0.428756
O -2.788040 -2.080766 -0.685074
C 3.292229 -0.631553 -0.474478
C 4.119910 0.608808 -0.188847
H 3.205603 -0.828493 -1.549395
H 3.710325 -1.515830 0.013798
H 4.156192 0.809544 0.885111
H 5.143789 0.462672 -0.547276
C -1.546995 1.647464 -0.233759
O -1.986686 2.703706 -0.363319
H -0.994515 0.156673 -1.657651
H 1.258709 -0.226236 -1.766609
FREQS=   23   29   65   72   82   90   96  181  213  267  311  342  349  414  441  444  473  479  528  551  558
695  772  821  863  872  952 1023 1118 1182 1271 1331 1406 1430 1466 1499 1506 1525 2026 2095 2121 2158 3000
3053 3056 3115 3133 3145
MIN131_8
18
E= -1916.37928273 ZPE=    74.04 Gcorr    0.050108000
H -3.978894 -1.449781 0.068852
Co 0.414627 -0.194972 -0.087972
C 2.013332 -1.047171 -0.402107
C 0.592341 1.371116 -0.966326
O 2.941268 -1.688809 -0.583224
O 0.722004 2.403964 -1.447242
C 0.882648 0.569018 1.567409
O 2.009112 0.633713 1.984844
C -2.619164 0.105699 0.747181
C -3.960312 -0.362502 0.177851
H -2.592850 1.195754 0.880502
H -2.432947 -0.309781 1.746158
H -4.778985 -0.067799 0.839887
H -4.140651 0.073251 -0.808174
C -1.428635 -0.259489 -0.094252
O -1.466723 -0.866763 -1.155501
H 0.036247 0.980615 2.164543
H 0.166599 -1.274884 0.913935
FREQS=   41   58   69   76   77   97  114  180  204  214  276  289  340  356  397  418  441  488  500  518  614
699  711  768  786  903  959 1025 1079 1114 1275 1321 1366 1427 1456 1509 1513 1727 1802 1967 2135 2176 2849
3041 3064 3072 3139 3143
MIN132_8
18
E= -1916.37313185 ZPE=    72.04 Gcorr    0.044499000
H 4.096912 -1.137311 -1.454650
Co -0.624318 -0.031609 -0.174620
C -2.284094 -0.191531 -0.809203
C -0.640016 -1.641122 0.676441
O -3.354795 -0.279607 -1.208209
O -0.589984 -2.594808 1.306124
C -0.905425 1.594833 0.548653
O -1.016861 2.568164 1.143505
C 3.182600 0.580467 -0.518208
```

C 3.736152 -0.812708 -0.470100
H 3.818462 1.370262 -0.912433
H 1.663828 1.995797 -0.141143
H 4.591589 -0.883463 0.215062
H 2.977481 -1.519245 -0.125307
C 1.971184 0.945673 -0.064253
O 1.116318 0.115469 0.562981
H 0.010263 0.239817 -1.791660
H 0.098210 -0.539837 -1.690319
FREQS= 15 45 54 89 90 93 112 135 202 241 251 338 350 393 421 428 441 447 462 521 526 551 563 575 724 743 781 936 949 1054 1069 1162 1245 1300 1403 1452 1497 1506 1702 2126 2134 2184 3022 3055 3064 3132 3173 3638
MIN133_8
18
E= -1916.37768999 ZPE= 74.30 Gcorr 0.050155000
H -2.437416 -0.211647 -1.717478
Co 0.490772 -0.308784 -0.009220
C 0.810496 0.789939 -1.499827
C 0.712550 1.082943 1.179095
O 1.105811 1.957729 -1.428002
O 0.829152 1.925809 1.940754
C 2.090229 -1.149122 0.008417
O 3.056144 -1.758617 -0.082352
C -3.907387 -0.334051 -0.096429
C -2.570494 0.125882 -0.681218
H -3.969850 -1.425023 -0.073462
H -4.032679 0.026935 0.927690
H -2.498251 1.220941 -0.728765
H -4.737140 0.046066 -0.698491
C -1.362430 -0.346781 0.073796
O -1.379527 -1.033590 1.086377
H 0.155844 -1.361079 -1.001966
H 0.738209 0.297475 -2.495830
FREQS= 40 51 59 77 79 98 131 148 198 207 272 297 312 360 408 422 457 483 502 521 634 699 739 778 894 901 958 1030 1079 1113 1275 1338 1368 1427 1455 1509 1514 1725 1783 1990 2142 2179 2866 3043 3063 3072 3138 3142
MIN134_8
18
E= -1916.37960156 ZPE= 76.58 Gcorr 0.052536000
H -3.897337 1.454340 1.272064
Co 0.598860 0.048837 0.048597
C 0.265052 1.482780 -0.958594
C 1.061995 -1.767558 -0.139700
O 0.132648 2.226615 -1.829709
O 0.391707 -2.499872 -0.883828
C 2.214590 0.485812 0.641369
O 3.156643 0.810216 1.221201
C -2.079578 0.262035 1.237392
C -3.262438 0.956106 0.534486
H -1.499765 0.990126 1.812861
H -2.456830 -0.496415 1.936594
H -2.909779 1.712175 -0.173744
H -3.867056 0.228908 -0.011999
C -1.175379 -0.447625 0.261266
O -1.768239 -1.399664 -0.378311
H 2.025158 -2.200679 0.199874
H -1.011139 -1.934712 -0.839905
FREQS= 6 36 66 72 91 98 143 200 226 259 287 320 363 383 423 460 482 528 553 588 639 689 808 872 927 1036 1055 1092 1146 1266 1338 1378 1411 1440 1484 1511 1520 1556 1641 2071 2118 2594 2913 3039 3058 3097 3130 3150
MIN135_8
18
E= -1916.37102668 ZPE= 73.65 Gcorr 0.044197000
H 4.491962 -1.623885 0.436666
Co -0.142077 0.337582 -0.081833
C -0.125826 1.773429 0.890366
C 0.325294 0.470445 -1.744067
O -0.014684 2.795808 1.415149
O 0.704004 0.709231 -2.808559
C -2.834959 -1.188885 0.259408
O -3.944883 -0.983364 -0.022580
C 2.643866 -0.513133 0.758072
C 3.419206 -1.750799 0.264358
H 2.828872 -0.393845 1.839984
H 3.018964 0.394527 0.275125
H 3.094391 -2.656674 0.786418
H 3.266273 -1.904614 -0.807990

C 1.171073 -0.637071 0.598191
O -1.722934 -1.425977 0.557926
H -1.428456 0.974837 -0.621127
H 0.800109 -1.594565 1.006956
FREQS= 20 34 41 49 68 76 86 97 127 153 230 286 301 344 416 498 505 534 542 595 623 633 710 725 790 851 937 1007 1042 1163 1249 1281 1366 1385 1421 1475 1510 1518 1836 2077 2124 2426 2983 2998 3049 3088 3125 3128
MIN136_8
18
E= -1916.37623826 ZPE= 74.36 Gcorr 0.050242000
H -2.919269 0.845907 0.718103
Co 0.435627 -0.290466 0.080809
C 0.254121 0.565187 -1.584149
C 0.818075 1.288618 0.949842
O 0.302114 1.760265 -1.747661
O 1.047004 2.254141 1.515160
C 2.055944 -1.022990 -0.230974
O 3.021191 -1.588264 -0.479217
C -3.077130 -0.346694 -1.082311
C -2.734839 -0.191047 0.401598
H -2.876172 -1.364034 -1.430112
H -4.137242 -0.136230 -1.246451
H -3.353543 -0.834598 1.038207
H -2.497591 0.346863 -1.696969
C -1.293035 -0.455879 0.731877
O -0.893496 -0.980306 1.763436
H -0.056752 -1.497598 -0.631410
H 0.125333 -0.107585 -2.462250
FREQS= 35 41 68 68 76 97 145 160 182 231 273 310 350 382 413 425 468 501 517 547 564 635 721 781 895 903 942 1046 1077 1166 1278 1339 1343 1431 1459 1514 1517 1717 1781 1985 2142 2179 2862 3029 3061 3083 3135 3142
MIN137_8
18
E= -1916.37158563 ZPE= 72.65 Gcorr 0.045637000
H 4.058227 0.874324 -1.106269
Co -0.688823 -0.000029 0.284779
C -2.466858 0.000211 0.046630
C -0.424021 1.634822 -0.372038
O -3.596695 0.000301 -0.149852
O -0.228080 2.677667 -0.815462
C -0.424224 -1.634283 -0.373373
O -0.228497 -2.676817 -0.817665
C 2.135109 0.000483 -0.647698
C 3.641029 0.001167 -0.589605
H 1.698889 0.000333 -1.641714
H 4.059813 -0.888606 -1.076086
H 4.044768 0.019853 0.430391
H 2.660334 -0.005957 1.737779
C 1.284662 -0.000680 0.393802
O 1.690677 -0.002204 1.706971
H -0.748408 0.404368 1.914449
H -0.748826 -0.405423 1.914169
FREQS= 14 38 45 74 93 97 111 196 220 277 302 327 335 369 379 449 460 470 501 520 542 564 570 615 616 691 796 909 946 1037 1043 1132 1288 1342 1432 1439 1485 1513 1685 2104 2117 2167 3021 3078 3080 3192 3389 3744
MIN138_8
18
E= -1916.37597582 ZPE= 75.12 Gcorr 0.050164000
H 3.020157 0.339334 1.156709
Co -0.547421 -0.008845 -0.105935
C -1.099065 -0.077772 1.661267
C -2.110025 -0.579565 -0.770310
O -0.469020 -0.809987 2.377427
O -3.120562 -0.742063 -1.297743
C -0.296077 1.715886 0.005525
O -0.244983 2.870511 0.013669
C 2.578429 0.058289 -0.953714
C 3.461349 -0.144033 0.280468
H 2.443517 1.117430 -1.194947
H 3.048611 -0.400687 -1.836964
H 3.575337 -1.206255 0.505792
H 4.451348 0.287269 0.111391
C 1.225072 -0.588017 -0.813261
O 0.986462 -1.627227 -0.187985
H 0.433778 -0.250322 -1.614224
H -1.965648 0.513446 2.029700

FREQS= 22 44 63 66 86 94 122 161 182 203 237 248 320 341 353 409 468 494 513 536 626
659 781 879 883 981 1015 1109 1135 1274 1292 1329 1368 1425 1475 1506 1516 1694 1806 2072 2123 2283 2890
3019 3065 3095 3137 3153
MIN139_8
18
E= -1916.37496469 ZPE= 73.86 Gcorr 0.050131000
H -3.303801 -0.700660 -1.123232
Co 0.382822 -0.166008 0.154499
C 1.894913 -1.186397 0.408464
C 0.180078 -0.128372 -1.623834
O 2.798854 -1.825092 0.696359
O 0.064340 -0.053106 -2.762955
C 1.128069 1.547209 0.281962
O 2.299487 1.771055 0.212077
C -2.169221 -0.807193 0.738148
C -3.484420 -0.733396 -0.044213
H -2.350187 -0.877791 1.817262
H -1.605294 -1.710975 0.461492
H -4.110554 -1.604688 0.167145
H -4.029869 0.171267 0.234283
C -1.327661 0.476663 0.541085
O -1.791214 1.583832 0.654478
H 0.337119 2.322145 0.426875
H 0.424115 0.058337 1.635358
FREQS= 37 52 71 78 84 102 122 188 217 239 259 322 343 372 378 422 437 482 519 554 621
668 696 730 782 875 939 988 1059 1099 1244 1267 1334 1419 1470 1509 1516 1811 1841 1965 2129 2169 2849
3004 3056 3077 3126 3147
MIN140_8
18
E= -1916.37903159 ZPE= 76.06 Gcorr 0.054325000
H 2.746292 0.228226 1.315309
Co -0.523649 -0.084649 0.067132
C -0.909316 -1.769009 0.593988
C 0.175287 1.564099 0.500331
O -0.755153 -1.450652 1.763087
O 0.577956 2.542765 0.940283
C -2.223637 0.394163 -0.321546
O -3.239205 0.699167 -0.761930
C 2.481310 0.019823 -0.841985
C 3.271785 -0.192841 0.452075
H 2.291039 1.082464 -1.024389
H 3.081274 -0.334932 -1.694402
H 3.430049 -1.259137 0.648494
H 4.255254 0.281971 0.394003
C 1.173132 -0.746198 -0.898750
O 0.211199 -0.379876 -1.680951
H -1.125691 -2.797778 0.255462
H 1.290573 -1.826754 -0.695628
FREQS= 38 51 74 83 88 114 139 182 193 235 265 317 360 391 409 421 465 482 492 507 556
636 789 881 888 951 1033 1097 1156 1226 1274 1329 1345 1429 1489 1506 1514 1519 1699 2111 2150 2946 2994
3009 3045 3088 3114 3125
MIN141_8
18
E= -1916.37898350 ZPE= 77.11 Gcorr 0.054448000
H -4.159852 -0.287169 1.081597
Co 0.413177 0.055170 -0.231551
C 0.442141 1.335840 0.954281
C -1.316665 0.293919 -0.847460
O 0.529208 2.060645 1.853360
O -0.907304 0.460455 -1.989753
C 0.245588 -1.570860 0.390755
O 0.221960 -2.583415 0.951614
C -2.763048 0.376097 -0.436036
C -3.104955 -0.417380 0.824909
H -3.377002 0.075281 -1.293507
H -2.954987 1.446783 -0.274108
H -2.921626 -1.486346 0.683090
H -2.504503 -0.083221 1.675530
C 2.146891 0.049738 -0.782727
O 3.322586 -0.125341 -0.206782
H 3.191411 -0.290792 0.741301
H 2.339461 0.232991 -1.851876
FREQS= 30 46 59 74 79 89 116 172 181 215 258 330 359 392 417 492 497 523 532 557 590
609 699 774 940 991 1046 1079 1150 1210 1276 1338 1351 1431 1455 1471 1511 1515 1717 2051 2098 3029 3036
3059 3080 3132 3136 3745
MIN142_8
18

```
E= -1916.37525736 ZPE=    74.81 Gcorr    0.050823000
H -3.851679 0.213055 -1.113458
Co 0.638564 0.208272 -0.001459
C 0.143260 1.901612 0.276345
C 0.579886 -0.920119 1.343565
O -0.122361 2.966335 0.622290
O 0.525664 -1.573038 2.291210
C 2.257518 -0.290325 -0.831317
O 3.264186 -0.904038 -1.051757
C -2.334033 -0.375250 0.330822
C -3.639867 -0.612220 -0.428518
H -2.377429 0.533976 0.942612
H -2.130690 -1.190395 1.037627
H -3.586855 -1.528609 -1.020983
H -4.474723 -0.699841 0.272423
C -1.115105 -0.267342 -0.573172
O -1.148669 -0.487344 -1.761630
H 1.406858 -0.159769 -1.603319
H 2.272785 0.574782 0.017232
FREQS=   34   43   62   80   90   96  101  164  209  256  284  304  321  369  379  419  448  503  523  528  560
664  692  787  925  953 1013 1079 1102 1280 1343 1353 1426 1461 1508 1513 1548 1791 1844 2067 2091 2138 2510
3047 3063 3081 3136 3145
MIN143_8
18
E= -1916.37266083 ZPE=    73.50 Gcorr    0.049036000
H 2.694268 -1.415808 0.719708
Co -0.109523 0.136345 -0.162666
C -1.212678 -1.269093 -0.998653
C 0.133135 -0.876319 1.313491
O -2.400657 -1.394740 -0.849819
O 0.276878 -1.564751 2.217737
C -1.646543 1.012805 0.278086
O -2.602654 1.601530 0.480645
C 1.552572 -0.740816 -1.030009
C 2.832898 -0.841713 -0.202396
H 1.752636 -0.198126 -1.956476
H 1.223141 -1.744661 -1.311155
H 3.612945 -1.352504 -0.782351
H 3.237724 0.136156 0.077946
C 0.985738 1.581077 0.014537
O 1.676219 2.493244 -0.024232
H -0.379937 0.591825 -1.547030
H -0.652672 -1.976102 -1.653649
FREQS=   23   56   72   89   99  113  113  147  199  253  270  312  352  403  409  414  455  484  487  507  520
537  629  668  715  795  903  986  994 1046 1233 1276 1365 1429 1485 1510 1513 1794 2010 2138 2151 2194 2846
3029 3066 3090 3106 3133
MIN144_8
18
E= -1916.37252224 ZPE=    73.54 Gcorr    0.049176000
H -4.015939 -0.882125 -1.033755
Co 0.942269 -0.000046 -0.560425
C 1.651030 -1.519730 -0.039136
C 1.650929 1.519767 -0.039406
O 2.256437 -2.393227 0.410298
O 2.256285 2.393389 0.409855
C -0.255639 0.000062 0.848430
O -0.292133 0.000178 2.053357
C -2.893995 0.000284 0.606068
C -4.051674 -0.000167 -0.388852
H -2.924262 0.866493 1.281899
H -2.924300 -0.865230 1.282776
H -5.007791 0.000275 0.140785
H -4.015707 0.880991 -1.034832
C -1.537137 -0.000033 -0.032324
O -1.329868 -0.000333 -1.245965
H 1.469906 0.401491 -2.127253
H 1.469972 -0.401794 -2.127185
FREQS=   29   44   67   68   89   98  131  189  232  285  300  323  327  372  379  483  492  499  539  552  570
571  624  710  790  791  901  997 1065 1075 1152 1261 1384 1430 1445 1507 1510 1512 1716 1818 2076 2129 3039
3063 3064 3139 3140 3465
MIN145_8
18
E= -1916.37671195 ZPE=    76.40 Gcorr    0.053506000
H -2.452617 -1.893179 -0.441563
Co 0.147509 0.118487 -0.012145
C 0.613827 0.851685 1.546356
C 0.237492 -1.653315 -0.045005
```

O 0.905083 1.323431 2.555516
O 0.273808 -2.808194 -0.023407
C -0.760036 1.269579 -1.038376
O -1.385743 2.019569 -1.649073
C -1.759842 -0.248530 0.827937
C -2.780345 -0.907274 -0.096534
H -2.137800 0.716324 1.177359
H -1.601707 -0.866324 1.716234
H -3.736479 -1.048206 0.426187
H -2.984993 -0.303018 -0.986095
C 1.726356 0.427205 -0.893571
O 2.789364 -0.336713 -1.051245
H 2.654660 -1.190551 -0.605084
H 1.951375 1.364949 -1.418313
FREQS= 40 52 64 88 91 92 104 132 146 247 255 319 347 420 432 453 479 485 486 510 522
574 643 714 718 972 983 1029 1049 1226 1246 1273 1343 1428 1487 1496 1510 1511 2064 2088 2134 3022 3062
3071 3089 3102 3125 3721
MIN146_8
18
E= -1916.38069430 ZPE= 76.41 Gcorr 0.057591000
H -1.774509 0.998589 -0.925460
Co -0.479408 0.112711 -0.105023
C 2.449764 -0.222753 -0.264735
C -0.420855 0.337276 1.637391
O 3.177000 -0.736325 0.561434
O -0.297096 0.484569 2.771046
C -1.526481 -1.378983 -0.107896
O -2.214210 -2.293567 -0.082945
C 0.132120 1.958335 -0.314513
C -1.219622 2.012975 -0.959370
H 0.966140 2.038693 -1.004901
H 0.257695 2.586642 0.563972
H -1.926664 2.681740 -0.466420
H -1.180297 2.199297 -2.033514
C 1.172187 -0.860235 -0.717527
O 0.491259 -0.383759 -1.712361
H 1.120825 -1.933551 -0.476914
H 2.702533 0.738333 -0.758626
FREQS= 59 65 78 91 105 137 151 225 255 316 355 365 393 410 434 442 477 512 524 538 550
620 816 908 936 983 1020 1055 1100 1137 1178 1304 1369 1392 1460 1480 1504 1599 1795 2115 2159 2454 2937
3017 3097 3138 3169 3220
MIN147_8
18
E= -1916.37386295 ZPE= 73.67 Gcorr 0.050801000
H 3.312399 -1.949740 -0.845850
Co -0.034162 0.115294 -0.028224
C -0.923026 -1.531548 -0.629826
C -0.432826 -0.202033 1.705319
O -2.093431 -1.634556 -0.891217
O -0.665669 -0.453023 2.798100
C -1.493228 1.078099 -0.537769
O -2.371952 1.684798 -0.940312
C 1.654589 -1.045814 0.221263
C 2.452968 -1.297556 -1.053144
H 1.298943 -1.994092 0.637565
H 2.299225 -0.593584 0.982136
H 1.850085 -1.788687 -1.824363
H 2.843527 -0.369908 -1.483568
C 1.156342 1.495459 0.012963
O 1.987058 2.278724 -0.068466
H 0.227436 0.009554 -1.482644
H -0.246191 -2.413670 -0.718904
FREQS= 56 64 75 81 97 102 114 159 233 263 304 328 345 406 409 422 451 464 497 508 519
540 632 668 735 817 904 985 990 1058 1246 1277 1354 1428 1486 1505 1511 1796 2019 2140 2152 2193 2839
3022 3046 3086 3090 3117
MIN148_8
18
E= -1916.37401993 ZPE= 73.42 Gcorr 0.051334000
H -2.649559 0.587244 -1.712408
Co 0.462341 -0.100389 -0.431880
C 0.983417 1.577163 -0.493100
C -0.174305 0.125636 1.338859
O 1.364792 2.661022 -0.543707
O 0.097673 0.631255 2.384217
C 2.008256 -1.042677 -0.242120
O 2.993866 -1.562793 0.025259
C -2.634864 0.091614 0.408907

C -3.202162 -0.041792 -1.006397
H -2.630392 1.133059 0.746278
H -3.274218 -0.455492 1.117367
H -3.138934 -1.076745 -1.350517
H -4.249922 0.269586 -1.030124
C -1.251784 -0.494902 0.569365
O -0.840915 -1.554468 -0.048254
H 0.347258 -0.514633 -2.102155
H -0.182127 0.077119 -2.000870
FREQS= 40 62 72 83 96 120 192 241 246 276 294 343 371 415 428 444 465 479 516 524 543 588 638 648 750 792 849 989 1055 1079 1169 1287 1374 1407 1412 1444 1487 1506 1519 1907 2112 2159 3025 3053 3093 3123 3146 3504
MIN149_8
18
E= -1916.37273816 ZPE= 75.05 Gcorr 0.050057000
H -3.963459 -1.067208 0.703687
Co 0.455435 0.280944 -0.120135
C 0.436242 -0.852826 1.204149
C 2.412459 -0.152054 -0.623467
O 0.354760 -1.518552 2.143525
O 3.356661 -0.649638 -0.054159
C -0.250897 1.884796 0.279986
O -0.671047 2.835787 0.775325
C -2.303091 -1.083292 -0.681463
C -3.023849 -0.530273 0.549721
H -2.068967 -2.147554 -0.536785
H -2.921374 -1.015143 -1.583687
H -2.419303 -0.646366 1.452677
H -3.258393 0.531199 0.429528
C -0.984905 -0.422586 -0.984254
O -0.488370 -0.281562 -2.090008
H 1.978488 0.890958 -0.176869
H 2.224475 -0.282234 -1.710385
FREQS= 22 44 66 76 84 89 125 139 183 220 236 242 312 358 369 397 459 495 509 540 562 573 752 780 942 1046 1076 1079 1158 1277 1338 1391 1433 1457 1513 1515 1607 1738 1778 1925 2079 2125 2917 3035 3063 3090 3136 3143
MIN150_8
18
E= -1916.37294355 ZPE= 73.86 Gcorr 0.050480000
H 1.883248 -1.636345 -1.954781
Co -0.022309 0.184365 -0.082796
C -0.979379 -1.328144 -0.881112
C -0.667780 -0.210575 1.580267
O -1.986556 -1.816126 -0.434328
O -1.039807 -0.486924 2.623603
C -1.297863 1.278526 -0.723259
O -2.069252 1.959926 -1.224603
C 1.411949 -1.295823 0.152384
C 2.393077 -1.428381 -1.007358
H 0.892042 -2.246377 0.308067
H 1.954027 -1.088293 1.081146
H 2.990119 -0.521936 -1.149203
H 3.091562 -2.257205 -0.828452
C 1.288019 1.387993 0.329348
O 2.204387 2.063342 0.448503
H 0.537546 0.348268 -1.436700
H -0.504505 -1.759279 -1.791628
FREQS= 52 64 71 79 93 94 112 121 227 271 297 303 371 403 413 423 442 461 499 509 521 539 629 724 810 848 908 980 998 1056 1243 1274 1358 1426 1488 1505 1511 1788 2052 2138 2157 2192 2859 3021 3048 3083 3089 3115
MIN151_8
18
E= -1916.37384994 ZPE= 73.31 Gcorr 0.051560000
H 3.628051 0.459160 1.720617
Co -0.627145 0.177435 -0.478880
C -1.881696 -0.009999 0.722751
C -0.105201 -1.657812 -0.266802
O -2.694481 -0.118980 1.529935
O -0.506405 -2.768222 -0.080370
C -0.302755 1.947030 -0.207157
O -0.009822 3.030881 0.024847
C 2.162571 -0.835098 0.785396
C 2.802951 0.536635 1.007501
H 2.930566 -1.562613 0.481256
H 1.724873 -1.233038 1.706808
H 3.191507 0.938868 0.069171
H 2.078948 1.252426 1.407799

C 1.126467 -0.865927 -0.311714
O 1.139529 -0.085930 -1.339819
H -1.408395 0.496484 -2.000622
H -1.457220 -0.292986 -1.911852
FREQS= 41 64 80 89 96 127 187 236 254 283 315 351 390 420 430 437 461 472 481 527 545
566 634 652 754 787 800 989 1050 1078 1164 1284 1293 1378 1419 1452 1484 1508 1518 1894 2110 2153 3019
3061 3090 3130 3147 3579
MIN152_8
18
E= -1916.37339999 ZPE= 73.30 Gcorr 0.051434000
H -1.598247 -0.065305 2.225054
Co 0.700323 0.159088 -0.477589
C 1.049639 1.462162 0.634415
C -1.092237 0.805805 -0.732251
O 1.281590 2.304710 1.382910
O -1.764551 1.788137 -0.836645
C 1.807692 -1.086603 0.254360
O 2.392514 -1.925614 0.772058
C -1.915795 -1.379281 0.513194
C -2.427519 -0.501794 1.659523
H -1.272653 -2.189204 0.877275
H -2.766110 -1.862306 0.009689
H -3.050100 0.316630 1.287438
H -3.029442 -1.095869 2.352001
C -1.166543 -0.648645 -0.571980
O -0.358616 -1.239175 -1.389582
H 1.131891 0.884594 -1.976256
H 1.737039 0.381749 -1.853806
FREQS= 46 48 76 91 96 144 164 236 265 288 347 361 387 417 429 438 462 476 485 517 539
564 632 653 715 788 810 965 1070 1081 1228 1275 1302 1335 1432 1454 1475 1513 1519 1894 2111 2155 3024
3056 3075 3128 3137 3575
MIN153_8
18
E= -1916.37331192 ZPE= 73.34 Gcorr 0.051385000
H -3.139539 0.807227 0.974232
Co 0.586649 -0.072088 -0.464060
C 0.669441 1.681175 -0.363855
C -0.470575 -0.151407 1.110359
O 0.770558 2.824929 -0.298309
O -0.580620 0.325377 2.198250
C 2.249458 -0.611613 0.043917
O 3.252626 -0.902854 0.515126
C -2.540093 -0.766703 -0.412807
C -3.168579 0.593929 -0.098089
H -3.140523 -1.564221 0.048667
H -2.533719 -0.971882 -1.489922
H -2.645102 1.402983 -0.617331
H -4.214322 0.611919 -0.416170
C -1.132704 -0.966513 0.094375
O -0.314327 -1.836264 -0.407841
H 0.250172 0.070108 -2.134618
H 0.915930 -0.372461 -2.136437
FREQS= 47 56 73 84 96 138 159 235 265 270 336 351 367 405 423 438 466 472 507 528 535
585 626 652 717 791 840 966 1072 1083 1230 1274 1335 1388 1432 1440 1480 1514 1519 1905 2112 2159 3024
3055 3074 3127 3136 3518
MIN154_8
18
E= -1916.37495439 ZPE= 76.36 Gcorr 0.053078000
H 2.178943 1.758699 0.881170
Co -0.019140 -0.232800 -0.000002
C -0.591682 -0.896522 1.510480
C -1.278257 1.176405 0.000005
O -1.000344 -1.486936 2.412646
O -0.653940 2.226327 0.000024
C -0.591664 -0.896506 -1.510497
O -1.000316 -1.486911 -2.412674
C 2.886382 -0.105563 0.000023
C 2.728314 1.415461 -0.000009
H 3.483369 -0.436826 -0.867735
H 3.483319 -0.436796 0.867826
H 3.706919 1.904389 0.000031
H 2.179031 1.758669 -0.881254
C 1.640027 -0.925486 0.000009
O -2.622440 1.131403 -0.000005
H -2.937449 2.056382 0.000006
H 1.880244 -1.996711 0.000013

FREQS= 25 57 58 74 86 95 95 132 160 269 273 323 341 393 423 487 502 507 551 592 669
672 701 716 898 900 1032 1033 1100 1153 1265 1279 1334 1366 1420 1440 1504 1514 1738 2082 2128 2977 2998
3057 3070 3130 3134 3662
MIN155_8
18
E= -1916.37191160 ZPE= 74.20 Gcorr 0.050131000
H 4.473691 -0.544876 -0.684594
Co -0.614932 0.067650 -0.308462
C -0.785015 -1.613325 -1.054815
C -1.357204 -0.359553 1.327594
O -1.954613 -1.594086 -1.393418
O -1.842297 -0.637413 2.323392
C -0.745306 1.872575 -0.383379
O -0.747626 3.004250 -0.560806
C 2.329420 -0.251223 -0.631489
C 3.684559 -0.400358 0.059247
H 2.319294 0.596009 -1.327875
H 2.088129 -1.127218 -1.244936
H 3.923297 0.487473 0.649764
H 3.689419 -1.257726 0.737438
C 1.169116 -0.031173 0.341584
O 1.352261 0.096300 1.528454
H -0.006510 0.237254 -1.650206
H -0.119373 -2.471539 -1.284539
FREQS= 27 61 67 77 88 105 115 187 203 218 234 296 327 375 403 425 447 483 500 520 627
687 754 788 850 894 918 1009 1077 1099 1275 1296 1339 1425 1458 1505 1512 1724 1786 2013 2142 2179 2909
3054 3060 3086 3133 3143
MIN156_8
18
E= -1916.37253906 ZPE= 73.75 Gcorr 0.050947000
H 3.620770 -1.180462 -0.830262
Co -0.122888 0.191962 -0.173885
C -1.896644 0.491067 0.116237
C 0.356211 -0.490899 1.432140
O -3.014641 0.703765 0.201309
O 0.647360 -0.938920 2.444435
C 0.413353 1.930363 -0.054133
O 0.787975 3.010348 -0.110019
C 1.739782 -0.123155 -1.020407
C 2.702423 -1.022178 -0.249127
H 2.223054 0.838129 -1.214204
H 1.522269 -0.555494 -2.000068
H 3.002437 -0.586237 0.709880
H 2.280114 -2.013358 -0.045902
C -0.568554 -1.603485 -0.846686
O -1.619972 -2.166156 -0.682799
H -0.464795 0.448307 -1.592237
H 0.248919 -2.096415 -1.423880
FREQS= 51 55 74 85 102 114 123 168 225 265 299 323 346 404 408 420 453 480 487 510 520
536 627 693 728 801 897 992 1002 1045 1249 1285 1354 1424 1487 1507 1513 1797 2019 2142 2154 2194 2840
3022 3066 3083 3096 3124
MIN157_8
18
E= -1916.37323646 ZPE= 74.03 Gcorr 0.051736000
H 3.579048 1.008882 -0.821683
Co -0.319935 0.093391 -0.197929
C 0.440078 -1.541552 -0.956708
C 0.433007 -0.245634 1.437205
O 1.037638 -2.378921 -0.327207
O 0.895719 -0.447865 2.460182
C -1.891313 -0.761728 -0.037647
O -2.901443 -1.302146 -0.041725
C 1.435674 0.811350 -1.054365
C 2.719756 0.634218 -0.249516
H 1.273027 1.871620 -1.265734
H 1.536853 0.328361 -2.030998
H 2.698597 1.190543 0.694147
H 2.918527 -0.416501 -0.015702
C -0.834639 1.826007 0.064837
O -1.119270 2.934421 0.056600
H -0.794831 0.372733 -1.565209
H 0.310499 -1.657070 -2.056381
FREQS= 61 72 76 83 100 103 124 136 259 266 292 299 367 400 409 421 447 474 495 504 520
535 632 721 774 858 914 991 1005 1052 1255 1278 1360 1423 1485 1510 1520 1783 2041 2136 2157 2194 2865
3025 3059 3085 3098 3121
MIN158_8
18

E= -1916.37500540 ZPE=   76.59 Gcorr   0.053609000
H -4.439877 -0.943583 -0.988801
Co 0.128656 0.163545 0.063110
C 0.279096 -0.539093 1.653471
C 1.471016 -0.882077 -0.732010
O 0.407335 -0.840743 2.758906
O 0.887757 -1.459660 -1.641027
C 0.910798 1.702335 -0.217766
O 1.407072 2.742714 -0.223975
C -2.538097 0.109845 -0.876409
C -3.744931 -0.593463 -0.220174
H -2.041495 -0.565783 -1.581560
H -2.906963 0.968568 -1.462488
H -3.420802 -1.459746 0.363494
H -4.285366 0.085284 0.446944
C -1.583123 0.663531 0.135485
O 2.775750 -1.054697 -0.475901
H -2.086085 1.326647 0.851050
H 3.114991 -1.694502 -1.132216
FREQS=   41   52   60   70   83   89   99 123 163 237 312 320 344 364 382 490 501 507 543 589 672
700 711 808 887 918 1002 1033 1108 1162 1240 1275 1345 1376 1418 1478 1508 1516 1721 2085 2129 2989 3051
3068 3085 3129 3132 3658
MIN159_8
18
E= -1916.37456999 ZPE=   76.33 Gcorr   0.053184000
H 4.493301 0.581560 0.002215
Co -0.556354 0.224477 -0.214698
C -2.129159 -0.626264 -0.315599
C 0.319155 -1.312292 0.553831
O -3.111284 -1.163317 -0.576830
O 0.752864 -1.288079 1.684843
C -1.164222 1.815951 0.219223
O -1.459306 2.828414 0.682092
C 2.352285 0.975530 -0.002051
C 3.596862 0.230558 -0.516800
H 2.239275 0.847731 1.078638
H 2.479586 2.057640 -0.182916
H 3.503371 -0.844062 -0.336273
H 3.740473 0.385394 -1.591131
C 1.092846 0.606568 -0.694930
O 0.344349 -2.451374 -0.193606
H 1.216641 0.507893 -1.788161
H 0.729331 -3.142487 0.380435
FREQS=   31   38   58   73   85   94 123 157 211 231 295 315 351 371 415 450 469 503 540 580 650
665 702 740 849 939 1005 1045 1093 1153 1246 1261 1299 1394 1420 1459 1508 1517 1772 2095 2139 2984 3001
3051 3099 3127 3132 3659
MIN160_8
18
E= -1916.37963086 ZPE=   76.08 Gcorr   0.058304000
H -1.403174 -1.760215 0.800955
Co 0.325902 -0.042071 -0.088585
C 1.530213 -1.332781 0.067200
C 1.423684 1.365543 -0.546867
O 2.255833 -2.219173 0.082211
O 2.025670 2.230365 -0.981949
C -0.142672 0.383452 1.674523
O -0.598444 0.714671 2.666585
C -2.178243 -0.882602 -1.035139
C -1.060892 -1.517428 -0.210450
H -3.101566 -1.478117 -0.970464
H -1.886754 -0.834422 -2.090384
H -0.678401 -2.435729 -0.661233
H 0.418499 -0.367013 -1.516620
C -2.371649 0.529540 -0.503279
O -1.115937 1.165283 -0.414719
H -2.862354 0.477598 0.489581
H -3.025219 1.130301 -1.152984
FREQS=   68   76   88   93 104 109 165 291 326 358 371 404 409 429 462 477 495 524 533 548 593
800 823 830 895 919 998 1021 1093 1166 1189 1263 1276 1358 1393 1483 1496 1525 2046 2146 2173 2200 2906
3009 3022 3060 3081 3126
MIN161_8
18
E= -1916.37723598 ZPE=   76.70 Gcorr   0.056147000
H -2.779082 -1.032356 0.882086
Co 0.258325 0.007723 0.000003
C 0.754452 1.789387 -0.000103
C 1.995292 -0.441205 0.000040

O 0.029718 2.891834 -0.000284
O 3.112931 -0.705247 0.000043
C -0.167261 -0.885707 -1.500500
O -0.463783 -1.385918 -2.493879
C -1.696379 0.655123 -0.000153
C -2.816604 -0.385998 0.000025
H -3.799662 0.103240 -0.000134
H -1.816546 1.284639 -0.892662
H -2.778997 -1.032753 -0.881743
H -1.816540 1.284953 0.892135
C -0.167307 -0.885359 1.500702
O -0.463842 -1.385342 2.494191
H -0.915117 2.650659 -0.000374
H 1.797810 2.133027 -0.000004
FREQS= 46 54 91 94 106 108 130 194 196 260 287 342 364 404 428 433 452 493 512 522 537
560 628 689 754 972 988 1013 1045 1204 1272 1299 1343 1431 1511 1512 1513 1523 2087 2102 2150 2994 3031
3051 3052 3095 3124 3657
MIN162_8
18
E= -1916.37476512 ZPE= 76.25 Gcorr 0.054148000
H -2.831555 1.018172 0.879592
Co 0.377977 0.315555 -0.260915
C 0.003797 1.977692 0.156740
C 0.323482 -1.503729 0.392084
O -0.341232 2.986987 0.592369
O -0.152199 -1.792316 1.468527
C 2.149090 0.397582 0.003753
O 3.296320 0.454970 -0.035449
C -2.535625 -0.328812 -0.798903
C -2.974676 -0.039565 0.637458
H -2.741574 -1.380835 -1.066590
H -3.135831 0.245362 -1.525074
H -4.034866 -0.274066 0.772819
H -2.390523 -0.633623 1.343341
C -1.104998 -0.100332 -1.114483
O 0.918931 -2.446449 -0.393377
H 0.873075 -3.283660 0.109229
H -0.885074 -0.253890 -2.185072
FREQS= 36 58 68 81 87 95 109 169 188 237 293 320 365 404 421 466 494 501 553 577 626
664 672 707 859 912 1018 1048 1087 1126 1270 1283 1298 1368 1422 1437 1512 1524 1771 2093 2138 2976 2999
3013 3056 3123 3153 3656
MIN163_8
18
E= -1916.37026157 ZPE= 73.62 Gcorr 0.050039000
H -2.579567 -0.245259 -1.665393
Co 0.222693 0.075502 -0.067939
C 1.915150 0.237632 -0.693541
C -0.083628 1.847214 0.175419
O 2.946548 0.354521 -1.174985
O -0.382280 2.950606 0.241484
C 0.516802 -0.577844 1.588513
O 0.662741 -1.027236 2.631775
C -1.808661 -0.035579 0.356383
C -2.729240 0.381756 -0.782434
H -2.017201 0.555214 1.254415
H -1.952392 -1.089816 0.588770
H -3.777409 0.268710 -0.472497
H -2.591220 1.426516 -1.079904
C 0.063642 -1.740548 -0.863744
O -0.862101 -2.501888 -0.765992
H 0.955197 -2.052890 -1.455984
H -0.213798 0.215154 -1.476894
FREQS= 45 62 74 82 86 93 110 152 241 252 312 337 352 390 415 423 442 463 498 502 521
536 602 638 730 794 908 982 1001 1061 1242 1284 1390 1421 1475 1505 1508 1805 2016 2137 2141 2181 2831
3023 3058 3087 3124 3161
MIN164_8
18
E= -1916.37459842 ZPE= 76.91 Gcorr 0.054438000
H -3.682697 1.472294 -0.690696
Co 0.439490 0.110521 -0.022750
C 1.413269 -0.956665 -1.001655
C 1.392629 1.638880 -0.450305
O 2.099556 -1.756581 -1.482368
O 0.500181 2.276739 -0.986276
C 0.689507 0.231150 1.701161
O 0.942164 0.140271 2.827669
C -2.450255 -0.306655 -0.593228

C -2.747627 1.153607 -0.221767
H -3.271339 -0.970223 -0.295615
H -2.356063 -0.389698 -1.686498
H -2.857732 1.266451 0.860967
H -1.950148 1.819972 -0.558095
C -1.159752 -0.841771 -0.031095
O -1.352238 -2.075094 0.412158
H 2.417728 2.048551 -0.350019
H -0.509921 -2.425361 0.746079
FREQS= 26 50 64 74 86 104 115 152 166 235 264 326 347 378 439 492 513 535 550 562 614 648 739 794 882 917 1034 1076 1112 1261 1288 1312 1364 1388 1431 1464 1514 1524 1710 2047 2098 2931 3020 3058 3074 3129 3149 3744
MIN165_8
18
E= -1916.37556125 ZPE= 76.62 Gcorr 0.055528000
H 2.791115 -0.128350 1.192088
Co -0.248218 -0.015993 0.006651
C 0.258651 -1.584717 0.839944
C -1.851178 -0.165376 0.801171
O 1.366958 -2.299843 0.765617
O -2.864387 -0.277748 1.330090
C -0.767051 -0.198393 -1.706842
O -1.037088 -0.376840 -2.810413
C 1.681312 0.179399 -0.693741
C 2.748627 0.558899 0.339634
H 3.747737 0.578087 -0.116831
H 1.688590 0.927890 -1.492204
H 2.564889 1.554318 0.753106
H 1.945958 -0.757936 -1.208003
C -0.075923 1.730209 0.386881
O 0.084758 2.846886 0.615399
H 2.004939 -1.836577 0.194547
H -0.409900 -2.165383 1.489908
FREQS= 29 61 83 91 98 110 155 178 211 266 302 330 362 392 434 438 459 475 501 518 540 557 630 693 736 953 981 1012 1036 1224 1260 1272 1335 1431 1505 1507 1521 1527 2092 2104 2151 2987 3024 3052 3075 3090 3121 3693
MIN166_8
18
E= -1916.37432164 ZPE= 76.66 Gcorr 0.055320000
H -1.982854 2.366042 0.151216
Co 0.091461 -0.127050 -0.024961
C 0.564886 1.576080 -0.537457
C -0.863129 -0.748112 1.364457
O 1.663401 2.304789 -0.373444
O -1.515794 -1.127247 2.233247
C 1.684750 -0.392935 0.713241
O 2.738316 -0.460271 1.178286
C -1.772915 0.390417 -0.776527
C -2.543020 1.432121 0.030338
H -2.375300 -0.517872 -0.874934
H -1.585837 0.756857 -1.791043
H -3.488095 1.684623 -0.469991
H -2.793400 1.074632 1.034004
C 0.142751 -1.377946 -1.318398
O 0.146672 -2.122848 -2.194350
H 2.313208 1.834385 0.173882
H -0.097881 2.198556 -1.153020
FREQS= 36 60 82 85 94 107 112 193 205 256 289 330 355 395 423 444 457 481 502 535 543 562 651 680 736 979 982 1013 1045 1231 1262 1275 1310 1429 1480 1503 1508 1513 2078 2094 2141 3021 3051 3064 3083 3096 3118 3738
MIN167_8
18
E= -1916.36965174 ZPE= 73.69 Gcorr 0.050805000
H 2.899155 1.135352 0.303520
Co -0.271615 0.104371 -0.154666
C -0.805865 -1.709406 -0.791008
C 0.178347 -0.558790 1.463438
O -0.085206 -2.627644 -1.079929
O 0.456605 -1.030703 2.469449
C -2.012658 0.531041 0.112197
O -3.118768 0.812625 0.192502
C 1.657943 -0.272392 -0.849939
C 2.830876 0.068787 0.063385
H 1.758349 0.253900 -1.801301
H 1.612873 -1.340817 -1.051498
H 2.804724 -0.482530 1.008714
H 3.770409 -0.202950 -0.436428

C 0.329541 1.814332 -0.197218
O 0.731689 2.874871 -0.354958
H -0.546622 0.196988 -1.608013
H -1.908957 -1.852590 -0.860649
FREQS= 57 69 83 86 92 103 110 157 204 266 274 337 353 393 413 419 448 481 486 506 520
535 600 647 734 778 908 991 996 1049 1230 1276 1392 1429 1499 1511 1512 1806 2011 2136 2139 2180 2835
3028 3082 3095 3112 3176
MIN168_8
18
E= -1916.36978315 ZPE= 73.86 Gcorr 0.051052000
H -2.852282 0.107872 -1.307689
Co 0.166946 -0.121461 -0.041589
C 0.242011 1.799518 0.388976
C 1.779441 0.024793 -0.814439
O 0.116406 2.720119 -0.373181
O 2.764498 0.168993 -1.380207
C -0.439534 -1.682996 -0.751058
O -0.938937 -2.557532 -1.295388
C -1.783095 0.246063 0.591281
C -2.716242 0.805442 -0.474979
H -1.737901 0.929404 1.447083
H -2.170498 -0.701522 0.980389
H -3.709116 1.003116 -0.048170
H -2.337858 1.745567 -0.886170
C 0.595534 -0.669206 1.641175
O 0.823956 -0.998825 2.712520
H 0.398562 2.005938 1.477751
H -0.294523 0.385346 -1.335978
FREQS= 66 73 77 81 84 92 110 138 244 266 291 331 358 407 414 428 444 455 489 499 524
536 612 726 791 827 908 982 995 1062 1245 1273 1375 1428 1489 1508 1517 1806 2088 2138 2153 2184 2787
3023 3039 3081 3093 3128
MIN169_8
18
E= -1916.36772446 ZPE= 72.91 Gcorr 0.049069000
H -3.123332 -0.000198 1.900552
Co 0.555890 -0.000021 0.386069
C 2.314235 -0.000007 0.064111
C 0.249558 1.622770 -0.276101
O 3.430823 0.000001 -0.202538
O 0.013724 2.657605 -0.719258
C 0.249555 -1.622734 -0.276286
O 0.013714 -2.657515 -0.719565
C -1.431370 -0.000047 0.552684
C -2.026458 -0.000191 1.938346
H 0.691391 0.407486 2.006654
H 0.691405 -0.407699 2.006610
H -1.719141 -0.880383 2.517860
H -1.719158 0.879891 2.518037
C -2.245834 0.000051 -0.505500
O -1.785376 0.000175 -1.803237
H -3.332065 0.000035 -0.396464
H -2.539333 0.000252 -2.403834
FREQS= 30 44 86 92 102 109 172 186 245 273 274 324 361 376 404 445 456 462 485 509 516
562 578 585 602 607 840 903 997 1062 1097 1162 1275 1363 1426 1434 1487 1510 1736 2103 2113 2163 3010
3065 3079 3122 3309 3856
MIN170_8
18
E= -1916.36421899 ZPE= 71.65 Gcorr 0.045763000
H 4.402575 0.225658 -0.500572
Co -0.620457 -0.008242 -0.225932
C -2.213354 -0.671983 -0.626706
C -0.214312 -0.992532 1.295690
O -3.192697 -1.133976 -0.995285
O 0.131754 -1.565987 2.216748
C -1.103876 1.681959 0.370796
O -1.346375 2.767733 0.614232
C 2.348697 -1.399404 -0.691409
C 3.488951 0.793227 -0.311940
H 3.315761 -1.847935 -0.881075
H 1.482530 -2.045222 -0.769999
H 3.420519 1.609425 -1.039365
H 3.554180 1.251570 0.680985
C 2.260595 -0.086302 -0.402036
O 1.160615 0.635640 -0.137422
H -0.646506 0.484378 -1.604225
H -0.203311 -1.032407 -1.178136

FREQS= 35 53 59 76 83 90 127 178 182 243 296 340 384 398 406 421 456 481 489 501 521
567 612 651 698 762 823 873 892 1004 1046 1070 1303 1411 1439 1485 1504 1682 2076 2098 2161 2191 2218
3044 3103 3143 3181 3262
MIN171_8
18
E= -1916.36615115 ZPE= 73.20 Gcorr 0.047927000
H -3.720497 0.696408 1.011776
Co 0.675202 -0.002709 -0.359501
C 1.522148 -0.055090 1.301343
C 0.564625 -1.751708 -0.667158
O 2.709164 -0.067096 1.089518
O 0.474358 -2.864698 -0.926807
C 0.642543 1.758235 -0.607410
O 0.597717 2.882594 -0.827713
C -2.209296 0.156986 -0.455532
C -3.576958 -0.026337 0.205369
H -2.059062 -0.552811 -1.275976
H -2.121907 1.150725 -0.913558
H -4.372356 0.110109 -0.532928
H -3.674454 -1.026602 0.635402
C -1.043305 0.019250 0.538596
O -1.221369 -0.057255 1.725128
H 0.788236 0.020241 -1.900196
H 1.052069 -0.081306 2.309742
FREQS= 34 42 59 76 81 107 110 181 205 210 255 278 344 359 396 450 460 462 505 550 553
627 655 684 786 872 900 996 1077 1094 1201 1283 1344 1424 1462 1505 1512 1780 1792 1820 2119 2171 2898
3050 3062 3091 3135 3147
MIN172_8
18
E= -1916.37217295 ZPE= 76.62 Gcorr 0.054193000
H 3.758938 -1.152159 0.397594
Co -0.542474 -0.166759 0.017013
C -1.637020 0.979942 -0.802764
C 0.531577 1.285521 0.721602
O -2.244567 1.647662 -1.515864
O 0.471970 1.576909 1.894083
C -1.716267 -1.445034 0.296132
O -2.459172 -2.246623 0.658970
C 1.931557 -1.446816 -0.774266
C 3.347980 -0.868550 -0.576406
H 1.556883 -1.209895 -1.774660
H 1.983599 -2.546979 -0.702795
H 4.019695 -1.242857 -1.354139
H 3.322697 0.222102 -0.636592
C 0.983898 -1.016016 0.295107
O 1.271873 2.017173 -0.161500
H 1.387026 -1.222190 1.296715
H 1.646787 2.759224 0.352584
FREQS= 31 59 63 85 86 97 104 140 203 233 273 315 370 378 415 429 469 506 554 587 660
686 702 804 916 929 1003 1043 1083 1123 1241 1281 1294 1377 1418 1476 1507 1524 1783 2093 2136 2987 3054
3057 3092 3129 3145 3659
MIN173_8
18
E= -1916.36891673 ZPE= 74.46 Gcorr 0.051208000
H 4.139843 -0.567349 -0.613821
Co -0.443599 0.043537 -0.342281
C -0.332816 -1.704472 -0.938974
C -1.643528 -0.322093 1.022009
O -1.335646 -1.735923 -1.630858
O -2.408643 -0.564809 1.833726
C -0.634284 1.816925 -0.623663
O -0.651644 2.927423 -0.906512
C 2.457342 0.445455 0.301883
C 3.117722 -0.801821 -0.303369
H 3.039346 0.805235 1.156945
H 2.402748 1.238613 -0.448996
H 3.164825 -1.614802 0.427334
H 2.573178 -1.153134 -1.183945
C 1.047568 0.152286 0.830322
O 0.856776 0.000676 2.014124
H 0.539655 0.146332 -1.445180
H 0.358804 -2.567018 -0.863844
FREQS= 37 60 72 73 90 99 128 184 200 223 233 312 324 376 407 429 472 488 511 572 614
636 760 805 858 898 911 1017 1072 1131 1281 1293 1325 1416 1484 1510 1520 1719 1780 2011 2140 2180 2934
3055 3061 3115 3130 3139
MIN174_8
18

E= -1916.37026632 ZPE= 74.11 Gcorr 0.052922000
H -3.902957 -0.655982 0.045856
Co 0.186825 0.128995 -0.098473
C 0.816425 1.697825 -0.530913
C -1.865485 -0.205631 0.403951
O 1.154787 2.746943 -0.850883
O -1.127261 -1.268011 0.375732
C 1.287781 -0.353167 1.289405
O 1.981710 -0.696647 2.129527
C -1.414092 0.878469 1.190123
C -3.086449 -0.144789 -0.477847
H -1.898734 1.842832 1.079726
H -0.977828 0.678546 2.161122
H -3.403379 0.882292 -0.672001
H -2.905423 -0.671722 -1.416441
C 1.209630 -0.961308 -1.347155
O 1.100676 -2.152585 -1.439474
H -0.452766 0.346573 -1.424812
H 1.930651 -0.411398 -1.999292
FREQS= 64 71 79 82 101 125 133 153 189 286 338 377 387 410 421 456 503 509 520 538 598
621 664 805 847 866 889 917 991 1043 1076 1346 1361 1413 1455 1489 1501 1535 1820 1970 2135 2174 2811
3054 3125 3163 3171 3262
MIN175_8
18
E= -1916.36682840 ZPE= 73.12 Gcorr 0.049550000
H 3.765514 -0.939540 0.117837
Co -0.099695 0.147535 -0.179011
C -0.242535 -1.530643 -0.759362
C -0.734024 -0.181086 1.676465
O -0.268209 -2.598331 -1.168352
O -1.334581 -1.159238 2.045676
C -1.755276 0.716754 -0.618526
O -2.796331 1.083224 -0.921756
C 1.703637 -0.535415 0.625412
C 2.854767 -0.567573 -0.370486
H 1.514217 -1.530074 1.035355
H 1.941371 0.109846 1.476405
H 2.646766 -1.226488 -1.218915
H 3.082076 0.423127 -0.775768
C 0.677360 1.729915 -0.006374
O 1.240698 2.726793 0.054260
H 0.445484 0.363578 -1.589553
H -0.459849 0.604806 2.426543
FREQS= 57 63 67 86 99 100 108 155 184 257 267 309 391 410 417 435 450 498 512 534 558
572 577 651 658 741 873 983 987 1060 1238 1269 1366 1431 1493 1507 1508 1785 1814 2134 2148 2189 2775
3028 3066 3096 3110 3131
MIN176_8
18
E= -1916.37100754 ZPE= 76.17 Gcorr 0.053963000
H -3.690489 1.263186 -0.238715
Co 0.127314 -0.130794 0.000429
C -0.812569 -1.332783 0.925383
C 0.342897 1.640017 0.071639
O -1.459165 -2.117469 1.469481
O 0.445103 2.786823 0.071197
C 0.572126 -0.765865 -1.607909
O 0.875474 -1.181848 -2.637749
C -1.776075 0.377534 -0.758873
C -2.729705 1.027665 0.240024
H -2.218713 -0.538369 -1.161038
H -1.609733 1.044448 -1.609658
H -2.944448 0.373931 1.091631
H -2.331031 1.964506 0.642634
C 1.665181 -0.505785 0.901923
O 2.705451 0.304656 1.050548
H 3.411326 -0.094061 1.586004
H 1.839553 -1.464201 1.416625
FREQS= 48 60 72 91 92 99 108 145 154 247 257 321 346 418 431 456 478 487 488 511 519
575 611 656 719 969 974 983 1050 1228 1262 1273 1298 1428 1488 1496 1510 1511 2079 2091 2138 3006 3020
3059 3087 3099 3122 3724
MIN177_8
18
E= -1916.36812147 ZPE= 75.81 Gcorr 0.051308000
H -4.917544 -0.190169 0.201564
Co 0.188811 0.060909 0.104866
C 2.062813 -0.311015 0.428119
C 0.044868 -1.330176 -0.942556

O 3.098370 -0.408480 -0.178272
O -0.097367 -2.129206 -1.759647
C 0.559582 1.661421 -0.500013
O 0.721310 2.659794 -1.050218
C -2.862006 0.517318 0.063223
C -3.931829 -0.459177 0.591290
H -2.864619 0.543652 -1.030881
H -3.118605 1.537154 0.400742
H -3.713512 -1.483942 0.277196
H -3.979805 -0.441324 1.684700
C -1.499951 0.249955 0.584100
O 2.007186 -0.494418 1.801927
H 2.915934 -0.696778 2.094905
H -1.496613 0.095382 1.685086
FREQS= 33 39 50 60 77 87 88 162 179 245 309 330 349 360 413 477 478 508 527 556 601 650 667 751 827 933 985 994 1034 1170 1239 1247 1258 1383 1422 1468 1509 1518 1822 2092 2136 2932 2983 3052 3090 3129 3133 3676
MIN178_8
18
E= -1916.36574585 ZPE= 73.00 Gcorr 0.048935000
H 1.658134 0.881004 2.318055
Co -0.685486 0.000010 0.302185
C -2.450961 -0.000004 0.026556
C -0.394113 -1.639808 -0.322001
O -3.571884 -0.000022 -0.219194
O -0.175366 -2.689973 -0.739399
C -0.394107 1.639809 -0.322042
O -0.175359 2.689965 -0.739465
C 1.318308 0.000009 0.363024
C 1.951779 0.000055 1.734041
H -0.763852 0.406517 1.930460
H -0.763841 -0.406467 1.930469
H 1.658163 -0.880872 2.318102
H 3.050693 0.000074 1.707759
C 2.041321 -0.000032 -0.769583
O 3.402730 -0.000045 -0.906884
H 1.580581 -0.000060 -1.752446
H 3.813927 -0.000028 -0.031827
FREQS= 34 45 75 94 98 114 124 225 226 282 283 340 340 366 367 455 461 479 500 510 516 560 565 588 591 688 900 910 951 1055 1086 1142 1276 1402 1422 1432 1483 1509 1691 2098 2112 2161 3007 3063 3077 3207 3340 3809
MIN179_8
18
E= -1916.37173341 ZPE= 75.85 Gcorr 0.054938000
H 3.801951 1.604458 1.032579
Co -0.746113 0.032836 -0.288740
C -0.749766 1.756910 -0.155265
C 0.946515 -1.374315 -0.252924
O -0.726624 2.905236 -0.143345
O -0.180390 -1.890786 -0.565855
C -2.339354 -0.393296 0.497670
O -3.337683 -0.714806 0.957400
C 2.277757 0.877291 -0.347220
C 2.898324 0.986820 1.061359
H 2.044689 1.883144 -0.712217
H 3.170132 0.000830 1.447494
H 3.029641 0.465127 -1.034637
H 2.192342 1.444151 1.761452
C 1.046092 0.033422 -0.389277
O 1.924851 -2.139598 0.251844
H -1.580319 0.156855 -1.507812
H 1.567986 -3.042483 0.322706
FREQS= 39 44 54 84 97 126 149 196 276 302 324 367 406 446 456 485 540 555 566 598 637 657 708 716 786 910 960 1066 1136 1148 1256 1297 1348 1416 1476 1501 1508 1521 1539 1932 2126 2161 3024 3048 3079 3120 3136 3735
MIN180_8
18
E= -1916.36884331 ZPE= 75.50 Gcorr 0.052108000
H 3.294887 2.152399 0.177216
Co -0.355175 -0.096542 0.169960
C -1.775562 1.620367 0.570113
C -1.474635 -0.665785 -1.052086
O -1.281537 2.717803 0.424237
O -2.197325 -1.195443 -1.779637
C 0.004392 -1.259785 1.457723
O 0.110312 -2.135127 2.200093
C 1.557342 1.065000 -0.535462

C 2.951712 1.117129 0.098417
H 0.840692 1.639823 0.075956
H 1.546821 1.533659 -1.524430
H 2.945475 0.681095 1.101367
H 3.661640 0.556348 -0.514400
C 1.130589 -0.422445 -0.771185
O 1.747784 -1.252652 -1.362585
H -1.261593 0.839654 1.274284
H -2.835103 1.400130 0.359106
FREQS= 39 59 70 84 89 101 112 149 170 204 251 267 293 335 375 393 455 487 520 531 571 667 715 796 829 985 1061 1091 1171 1283 1339 1396 1422 1459 1463 1508 1514 1779 1893 2078 2128 2249 2992 3005 3059 3093 3134 3146
MIN181_8
18
E= -1916.36637053 ZPE= 73.51 Gcorr 0.049681000
H -3.737686 -0.631882 -1.117046
Co 0.170281 -0.006185 -0.092427
C 1.911046 0.706381 0.632131
C -0.546302 -0.498538 1.494451
O 2.801628 1.159800 -0.036071
O -1.013800 -0.829389 2.485584
C 1.013534 -1.490941 -0.558858
O 1.533923 -2.437419 -0.942773
C -1.570138 -0.703153 -1.063717
C -2.913068 -0.228387 -0.513670
H -1.534347 -1.795985 -1.051611
H 0.671417 0.340799 -1.432507
H -3.086337 -0.556474 0.517118
H -3.011742 0.862441 -0.530172
C -0.393230 1.662780 -0.276340
O -0.756780 2.730199 -0.483559
H 2.027832 0.665544 1.744376
H -1.457538 -0.391821 -2.104061
FREQS= 48 71 76 78 85 96 107 138 168 246 268 316 342 434 447 462 484 489 492 510 531 547 591 698 734 771 858 978 989 1043 1226 1278 1365 1429 1486 1511 1514 1803 2057 2131 2147 2182 2784 3022 3068 3084 3100 3134
MIN182_8
18
E= -1916.36587977 ZPE= 73.30 Gcorr 0.049297000
H -3.691323 0.000752 -1.055664
Co 0.246461 -0.000044 -0.147977
C 0.403562 0.000041 1.857800
C 2.019310 -0.000617 -0.500878
O -0.523247 0.000306 2.626682
O 3.120446 -0.000987 -0.812447
C -0.022904 1.748662 -0.237987
O -0.240564 2.868137 -0.355910
C -1.842114 0.000575 0.049912
C -2.611686 0.000377 -1.260187
H 0.098439 -0.000077 -1.658206
H -2.081704 0.868539 0.665319
H -2.393772 0.881871 -1.870624
H -2.394284 -0.881630 -1.870066
C -0.023989 -1.748592 -0.237768
O -0.242356 -2.867944 -0.355548
H 1.443028 -0.000177 2.271165
H -2.082132 -0.866869 0.665881
FREQS= 41 52 61 90 97 104 136 157 160 263 267 324 382 394 417 424 453 493 528 551 551 560 587 632 734 744 862 980 987 1069 1238 1254 1372 1430 1484 1504 1507 1797 1851 2129 2142 2183 2788 3025 3091 3107 3117 3153
MIN183_8
18
E= -1916.36585311 ZPE= 75.13 Gcorr 0.049320000
H 4.691265 0.136076 -0.339096
Co -0.407243 0.003961 -0.032920
C -1.022323 1.340901 1.057257
C -1.886752 -0.931129 -0.635371
O -1.295549 2.307213 1.610437
O -2.745999 -1.438050 -1.198092
C 0.168660 -1.140833 1.180822
O 0.559942 -1.892827 1.955175
C 2.605130 -0.487597 -0.456182
C 3.707921 0.590585 -0.492368
H 2.804032 -1.210491 -1.267753
H 2.660304 -1.053438 0.479079
H 3.717122 1.113634 -1.453359
H 3.553405 1.334353 0.295188

C 1.249967 0.062410 -0.733789
O -0.879520 1.109363 -1.536765
H 1.234914 0.707900 -1.620979
H -1.712103 1.573407 -1.372483
FREQS= 16 31 61 74 93 105 117 132 194 256 260 317 326 346 396 414 427 437 442 513 536 541 582 672 797 857 941 981 1021 1036 1158 1244 1272 1386 1419 1474 1508 1517 2117 2123 2166 2976 3050 3074 3090 3125 3130 3807
MIN184_8
18
E= -1916.37008471 ZPE= 76.18 Gcorr 0.053639000
H 3.169381 0.454324 0.018558
Co -0.138973 0.098210 0.031958
C 0.214260 -0.695012 -1.525063
C -1.010389 -1.002762 1.137501
O 0.419120 -1.216263 -2.531812
O -1.530412 -1.742976 1.849332
C 0.768315 1.600706 0.365801
O 1.386826 2.545192 0.601775
C 1.585245 -0.825017 0.835703
C 2.891370 -0.603000 0.075002
H 1.373907 -1.896945 0.884660
H 1.685520 -0.472776 1.865779
H 3.718021 -1.127169 0.574167
H 2.843407 -0.982691 -0.950835
C -1.631516 1.010842 -0.478404
O -2.878385 0.563845 -0.431585
H -3.520650 1.222036 -0.745869
H -1.598225 2.038623 -0.874252
FREQS= 40 57 68 89 97 102 108 133 152 253 268 322 345 421 430 452 480 483 488 509 517 573 613 655 711 973 979 990 1044 1231 1262 1281 1301 1428 1487 1495 1513 1514 2077 2088 2137 3006 3022 3062 3087 3102 3126 3719
MIN185_8
18
E= -1916.37047678 ZPE= 76.24 Gcorr 0.054704000
H -3.538103 1.619562 0.394230
Co 0.090824 -0.106058 0.036449
C 0.171673 -1.310170 1.369647
C -0.833316 -0.830713 -1.325458
O 0.186909 -2.013928 2.279659
O -1.472797 -1.266574 -2.177646
C 1.712039 -0.386165 -0.682669
O 2.745809 -0.553426 -1.151531
C -1.776793 0.401287 0.770578
C -2.593104 1.354064 -0.099763
H -1.597965 0.847690 1.754812
H -2.351534 -0.514220 0.943617
H -2.063016 2.291253 -0.305538
H -2.847239 0.911784 -1.068156
C 0.525464 1.616703 0.427021
O 1.686880 2.234493 0.180784
H 1.695670 3.141672 0.525709
H -0.160249 2.291273 0.964943
FREQS= 34 63 84 95 96 110 113 190 199 260 285 331 339 398 422 442 453 480 497 533 540 552 579 670 733 947 979 983 1043 1228 1247 1276 1284 1427 1473 1498 1505 1512 2090 2107 2154 2999 3018 3045 3076 3091 3114 3736
MIN186_8
18
E= -1916.36149238 ZPE= 72.13 Gcorr 0.046101000
H 4.312949 -1.335805 -0.250582
Co -0.731067 -0.012413 -0.192793
C -2.318847 -0.590599 -0.739605
C -0.213325 -1.366050 0.954183
O -3.286206 -0.960148 -1.225034
O 0.236907 -2.211462 1.571783
C -1.207841 1.467207 0.816806
O -1.393996 2.464934 1.333549
C 3.159570 0.352927 -0.942685
C 3.821191 -0.438516 0.146388
H 3.761033 0.642441 -1.801645
H 1.483513 1.333247 -1.779228
H 4.599271 0.144971 0.657255
H 3.091912 -0.747558 0.898817
C 1.881786 0.767438 -0.931863
O 1.049291 0.600061 0.114889
H -0.826298 0.835165 -1.390090
H -0.266725 -0.838830 -1.309952

FREQS= 29 37 61 83 90 94 130 135 213 247 260 371 399 405 410 420 455 465 498 502 533
610 626 729 773 826 873 937 954 1055 1068 1164 1296 1401 1445 1497 1506 1703 2045 2073 2162 2187 2213
3021 3063 3088 3132 3173
MIN187_8
18
E= -1916.37151655 ZPE= 76.89 Gcorr 0.056243000
H -4.145945 0.844592 0.437456
Co 0.592558 -0.239155 -0.561964
C 0.568770 1.350289 0.369634
C -0.661493 -1.262772 0.298806
O 0.231398 1.622603 1.493489
O -0.820985 -1.847607 1.321378
C 2.238133 -0.687676 -0.104005
O 3.237367 -1.065485 0.320988
C -2.680901 -0.113682 -0.839069
C -3.124485 0.460000 0.510830
H -3.356579 -0.930384 -1.129708
H -2.766906 0.652141 -1.618945
H -3.107419 -0.305236 1.291893
H -2.467890 1.273689 0.830068
C -1.251804 -0.628055 -0.840593
O 1.005586 2.295819 -0.525886
H 0.958568 3.157470 -0.070517
H -0.869143 -0.986350 -1.820592
FREQS= 46 55 65 77 89 90 157 177 220 263 306 310 360 417 443 467 501 515 548 616 663
682 707 799 837 949 1031 1053 1108 1120 1273 1291 1303 1371 1436 1498 1511 1524 1817 1947 2130 2909 3021
3059 3067 3131 3141 3687
MIN188_8
18
E= -1916.36649825 ZPE= 75.32 Gcorr 0.051523000
H -2.943868 1.477739 -0.312148
Co 0.384995 -0.002533 -0.052888
C 1.713440 -1.295166 0.008932
C 1.331208 1.570357 0.101457
O 2.495143 -2.122480 -0.120938
O 1.787691 2.618711 0.088651
C -0.240172 -0.224596 1.591688
O -0.658352 -0.404970 2.645824
C -2.647706 0.439756 -0.080846
C -3.687229 -0.505776 -0.719718
H -2.675093 0.340789 1.008357
H -3.445027 -1.551888 -0.509683
H -4.684138 -0.301914 -0.318110
H -3.723650 -0.377389 -1.805966
C -1.284585 0.247312 -0.655111
O 0.973425 -0.138924 -1.874622
H -1.299129 0.340087 -1.748673
H 0.483034 -0.869050 -2.275514
FREQS= 31 42 70 75 102 115 128 141 195 257 298 327 346 363 386 404 418 438 448 504 516
543 586 680 808 858 929 943 1022 1036 1157 1248 1277 1379 1418 1485 1509 1518 2119 2134 2173 2982 3050
3065 3093 3127 3128 3821
MIN189_8
18
E= -1916.36668161 ZPE= 75.30 Gcorr 0.051831000
H -4.672327 -0.051100 -0.258921
Co 0.403821 -0.004066 -0.082093
C -0.090498 1.445732 0.811678
C 0.696836 -1.212691 1.273755
O -0.408214 2.403961 1.358960
O 0.735331 -2.049519 2.052296
C 2.117370 0.549607 -0.527202
O 3.149938 0.806371 -0.951713
C -2.581272 0.487355 -0.566825
C -3.702762 -0.549496 -0.351987
H -2.800683 1.045767 -1.494006
H -2.588652 1.224878 0.241780
H -3.757505 -1.251251 -1.189950
H -3.531143 -1.127685 0.560983
C -1.244731 -0.142371 -0.771644
O 0.994043 -1.320211 -1.348652
H -1.295885 -0.954821 -1.507907
H 0.804568 -0.969617 -2.229257
FREQS= 30 60 69 76 100 112 136 137 191 243 301 318 336 366 397 410 423 439 449 504 525
540 585 668 798 851 921 944 1030 1038 1155 1249 1276 1388 1420 1475 1508 1517 2119 2134 2173 2981 3049
3065 3090 3126 3128 3821
MIN190_8
18

E= -1916.36286673 ZPE=   72.50 Gcorr   0.048031000
H -2.309207 0.000017 2.175374
Co 0.495400 -0.000002 -0.619021
C -0.133488 -1.668157 -0.761283
C -0.133443 1.668170 -0.761282
O -0.531273 -2.746438 -0.800862
O -0.531199 2.746461 -0.800860
C 2.110742 -0.000028 0.248287
O 3.119350 -0.000045 0.791443
C -2.949051 0.000010 0.069789
C -1.912288 0.000011 1.154158
H -3.600865 -0.880579 0.141045
H -3.600851 0.880612 0.141030
H -0.302384 0.000016 2.934334
H -2.500372 -0.000002 -0.926105
C -0.574851 0.000007 1.028417
O 0.247394 0.000012 2.133769
H 0.634394 0.000003 -2.232985
H 1.423593 -0.000024 -1.975575
FREQS=   29   56   65   97   97 116 152 188 234 292 296 316 323 368 402 415 423 446 467 489 517
540 543 570 584 628 794 943 1039 1061 1116 1135 1248 1368 1428 1498 1503 1687 1709 2099 2119 2168 3022
3065 3074 3121 3132 3730
MIN191_8
18
E= -1916.36993683 ZPE=   77.40 Gcorr   0.055570000
H -2.792692 2.339700 1.610987
Co 0.716751 0.123296 0.149757
C -0.421324 -1.242697 -0.222333
C 2.088000 -0.868295 0.373850
O -0.611116 -2.429515 -0.318763
O 3.014770 -1.531112 0.552981
C 1.540056 1.652538 -0.342738
O 2.077846 2.484504 -0.928488
C -1.705178 0.644570 0.776956
C -2.735366 1.768749 0.680074
H -0.706501 1.107939 0.999682
H -1.918201 -0.005414 1.631523
H -2.492944 2.461630 -0.132459
H -3.719070 1.337630 0.476632
C -1.623899 -0.234244 -0.485152
O -2.806437 -0.949376 -0.718401
H -1.416077 0.400811 -1.362611
H -2.561041 -1.891004 -0.649767
FREQS=   21   39   59   74   97 118 158 200 226 268 307 324 355 384 392 400 475 478 557 567 599
722 757 831 899 1030 1068 1103 1166 1224 1304 1377 1397 1408 1423 1472 1510 1516 1822 2083 2138 2752 2973
3053 3091 3126 3141 3658
MIN192_8
18
E= -1916.36603869 ZPE=   75.34 Gcorr   0.051819000
H -4.586849 -0.668662 -0.362524
Co 0.374333 0.038431 -0.051044
C 0.506650 -0.309797 1.746618
C 1.980081 -0.593543 -0.724681
O 0.409896 -0.546332 2.861612
O 2.883776 -1.098487 -1.214951
C 0.769507 1.773062 -0.130699
O 1.005110 2.892826 -0.216878
C -2.460505 -0.443749 -0.775635
C -3.769686 0.012159 -0.105142
H -2.198452 -1.466723 -0.495655
H -2.610027 -0.441366 -1.871024
H -3.669937 0.014587 0.984776
H -4.055991 1.020978 -0.421086
C -1.313941 0.474738 -0.517551
O 0.027942 -1.821245 -0.370880
H -0.221686 -1.906241 -1.300621
H -1.650482 1.518478 -0.584348
FREQS=   31   67   70   76   86 103 107 155 215 250 294 319 340 382 402 412 425 433 454 497 519
549 584 664 787 865 919 944 1034 1044 1132 1249 1307 1401 1419 1478 1509 1516 2123 2134 2173 2967 3042
3046 3106 3122 3125 3824
MIN193_8
18
E= -1916.36346536 ZPE=   73.08 Gcorr   0.049361000
H -3.402495 1.730607 0.281058
Co 0.284459 0.065987 -0.254772
C 1.260858 1.547017 -0.194227
C -0.146813 -0.208601 1.662545

O 1.859133 2.523807 -0.229848
O -0.765617 -1.137391 2.119600
C 1.649285 -1.102080 -0.086403
O 2.518930 -1.839614 0.014012
C -1.302288 1.433251 -0.094222
C -2.717251 0.892131 0.100043
H -1.253452 2.038140 -1.002223
H -1.053245 2.083836 0.750371
H -3.087706 0.361167 -0.781434
H -2.794452 0.217132 0.956878
C -0.883146 -1.082376 -0.953999
O -1.606110 -1.802345 -1.470671
H 0.507785 0.343168 -1.740557
H 0.188616 0.612580 2.347589
FREQS= 48 59 74 86 99 105 110 130 211 256 293 310 390 399 417 434 446 485 508 543 548
566 575 634 652 708 874 985 1004 1045 1246 1290 1365 1427 1480 1513 1519 1782 1810 2137 2147 2190 2772
3033 3055 3101 3116 3139
MIN194_8
18
E= -1916.37203579 ZPE= 77.82 Gcorr 0.057954000
H 2.274874 3.050759 0.337659
Co -0.727686 0.123177 0.075983
C 0.594346 -1.201913 -0.149916
C -1.920392 -1.057615 0.404783
O 0.686696 -2.220162 -0.805623
O -2.715629 -1.838153 0.701024
C -1.815092 1.507370 -0.278700
O -2.538497 2.276406 -0.735538
C 1.516502 1.020022 0.075415
C 2.573929 2.019608 0.544471
H 0.585841 1.243604 0.673997
H 1.311538 1.178070 -0.993161
H 2.755883 1.920865 1.619064
H 3.510860 1.817451 0.019260
C 1.863809 -0.463541 0.310246
O 3.017793 -0.864450 -0.372752
H 1.963311 -0.629918 1.398980
H 2.763705 -1.679314 -0.842044
FREQS= 35 44 76 85 98 139 176 213 221 284 291 312 357 376 403 423 470 481 552 583 625
758 830 899 929 1029 1075 1113 1182 1233 1312 1360 1389 1408 1421 1506 1513 1542 1771 2086 2140 2691 2956
3031 3057 3131 3144 3689
MIN195_8
18
E= -1916.36752748 ZPE= 77.08 Gcorr 0.053464000
H -3.998109 0.132665 -1.224583
Co 0.477758 0.047156 0.167306
C 2.220032 0.372793 0.276968
C -1.225564 -0.369349 0.855247
O 3.346474 0.550783 0.441824
O -0.888572 -0.605674 2.002262
C 0.008733 1.675135 -0.313592
O -0.248066 2.689346 -0.809273
C -2.661579 -0.426871 0.382168
C -2.953493 0.282225 -0.937651
H -3.292720 -0.055353 1.198843
H -2.881405 -1.501179 0.292043
H -2.775437 1.357963 -0.863316
H -2.322500 -0.106119 -1.742419
C 0.542928 -1.683776 -0.399320
O 0.954238 -2.162926 -1.564581
H 1.227805 -1.418731 -2.126860
H 0.243971 -2.555618 0.204268
FREQS= 17 38 62 66 82 102 109 162 183 234 256 310 370 382 415 469 487 499 550 564 586
625 715 769 931 1027 1047 1079 1132 1217 1278 1316 1337 1432 1453 1480 1512 1517 1741 2061 2118 3023 3026
3060 3077 3130 3140 3733
MIN196_8
18
E= -1916.36427958 ZPE= 73.94 Gcorr 0.050391000
H 4.967387 0.337404 0.006099
Co -0.374721 0.032201 -0.159968
C -1.847087 -0.641226 -1.261022
C -0.823830 -1.091863 1.203510
O -3.009343 -0.326225 -1.263328
O -1.176751 -1.750927 2.072400
C -1.027700 1.530046 0.423341
O -1.445206 2.551077 0.743166
C 2.835694 0.600667 0.296297

C 4.033795 -0.138887 -0.305039
H 2.871178 0.623523 1.393695
H 2.812304 1.658283 -0.000473
H 4.055554 -1.181892 0.021292
H 3.990763 -0.130999 -1.397067
C 1.495771 0.050085 -0.068354
O 1.295280 -0.911389 -0.813178
H -0.411840 0.893656 -1.369849
H -1.479582 -1.422629 -1.979440
FREQS= 45 53 67 78 80 92 149 176 204 208 270 314 359 396 435 445 450 488 516 534 589 649 681 765 821 876 942 1025 1075 1109 1273 1342 1364 1427 1446 1508 1513 1676 1802 1986 2127 2159 2751 3043 3064 3070 3139 3143
MIN197_8
18
E= -1916.36409165 ZPE= 73.39 Gcorr 0.050290000
H 3.275159 -0.253908 -0.500301
Co -0.125021 0.050136 -0.295557
C 0.901241 -1.383096 -0.502565
C -0.127503 -0.024104 1.698186
O 1.544668 -2.307340 -0.714126
O -0.936952 -0.589350 2.388881
C -1.693121 -0.858691 -0.452881
O -2.677301 -1.419419 -0.590930
C 1.631554 1.171501 -0.199911
C 2.918360 0.471621 0.235853
H 2.813850 -0.045945 1.196144
H 1.420288 1.973807 0.514088
H 1.765393 1.624500 -1.183070
H 3.712491 1.218676 0.363857
C -0.859038 1.664367 -0.373813
O -1.294891 2.718191 -0.484510
H -0.045757 0.133625 -1.813668
H 0.720995 0.529337 2.179244
FREQS= 46 56 69 95 96 108 134 172 191 265 304 312 394 409 412 425 444 479 538 551 559 563 605 632 707 721 870 985 1010 1041 1246 1298 1353 1428 1479 1511 1516 1792 1830 2132 2158 2194 2772 3030 3058 3088 3118 3144
MIN198_8
18
E= -1916.36686627 ZPE= 76.43 Gcorr 0.053310000
H -2.563066 -0.814123 2.648971
Co 0.464650 0.089592 -0.136259
C -0.225229 -1.395295 -1.180487
C 1.180653 1.655018 0.175399
O -0.801744 -2.404363 -0.841600
O 1.660768 2.706254 0.177550
C 1.900635 -0.887408 0.218989
O 2.779019 -1.535279 0.585715
C -2.300803 0.260970 0.787023
C -1.760944 -0.584370 1.941902
H -3.108846 -0.268196 0.261618
H -2.747603 1.199779 1.145788
H -1.356189 -1.528533 1.568929
H -0.976266 -0.054182 2.490087
C -1.279595 0.621525 -0.250983
O -1.850461 1.321663 -1.224462
H 0.001508 -1.237037 -2.275310
H -1.184045 1.554479 -1.889764
FREQS= 27 44 57 79 87 93 95 151 208 236 288 313 379 398 418 464 497 510 529 550 572 597 699 776 868 935 1061 1068 1098 1277 1290 1342 1351 1390 1433 1441 1511 1524 1762 2074 2127 2677 3021 3055 3058 3124 3141 3766
MIN199_8
18
E= -1916.36380323 ZPE= 73.33 Gcorr 0.050302000
H -3.588178 -1.351846 0.068266
Co 0.169707 0.191807 -0.289751
C -0.899652 1.604460 -0.261580
C 0.018951 -0.433825 1.590524
O -1.612381 2.500330 -0.323157
O 0.558720 -1.411206 2.048699
C 1.703627 1.043082 0.130009
O 2.675394 1.601990 0.360871
C -1.650022 -0.792518 -0.686007
C -2.673411 -0.871362 0.439893
H -1.397025 -1.802902 -1.012982
H -2.062803 -0.281349 -1.557508
H -2.319419 -1.470697 1.283257
H -2.961791 0.112615 0.825743

C 0.904110 -1.311233 -0.904634
O 1.341937 -2.267504 -1.352736
H 0.260850 0.617110 -1.755103
H -0.644701 0.177763 2.252952
FREQS= 48 64 85 87 102 108 112 147 215 252 280 312 393 410 425 429 445 496 513 537 563 573 584 657 668 726 880 984 1004 1052 1249 1285 1368 1426 1487 1507 1515 1778 1810 2135 2149 2191 2784 3029 3081 3095 3117 3142
MIN200_8
18
E= -1916.36423947 ZPE= 75.22 Gcorr 0.050752000
H 3.771417 -0.919205 0.156880
Co -0.232001 0.044626 -0.128754
C -1.845365 -1.536156 -0.616126
C 1.348724 0.224307 0.671221
O -3.035689 -1.354440 -0.556462
O 2.087937 0.961949 1.246590
C -1.189182 0.502830 1.277234
O -1.791386 0.973305 2.139489
C 1.646871 -1.275231 0.309896
C 2.979593 -1.381276 -0.437615
H 0.843745 -1.740807 -0.291027
H 1.672575 -1.817806 1.259487
H 3.240011 -2.428218 -0.617047
H 2.933251 -0.867626 -1.401955
C 0.012518 1.321743 -1.327140
O 0.067897 2.263441 -1.989114
H -1.180592 -0.819971 -1.256365
H -1.365413 -2.502611 -0.362454
FREQS= 26 53 70 77 89 96 135 144 152 202 242 257 287 333 374 402 460 487 506 527 569 666 698 764 823 983 1060 1089 1167 1274 1311 1421 1424 1450 1461 1508 1515 1802 1892 2086 2135 2264 2932 2966 3059 3095 3134 3147
MIN201_8
18
E= -1916.36554787 ZPE= 75.55 Gcorr 0.052130000
H 3.925381 0.982621 -0.740520
Co -0.337571 0.042309 -0.005983
C -1.476330 -0.754829 -1.231395
C -1.182410 -0.251457 1.599822
O -2.059726 -1.357923 -2.011147
O -1.537062 -0.439884 2.670724
C -0.779628 1.730661 -0.361358
O -1.032508 2.820935 -0.614260
C 2.572211 -0.205329 0.522232
C 3.643064 -0.063436 -0.583220
H 2.310121 -1.252695 0.678955
H 2.998620 0.186535 1.460739
H 4.543125 -0.621013 -0.307856
H 3.278084 -0.459783 -1.535777
C 1.360547 0.609064 0.216054
O 0.225941 -1.791201 -0.019337
H 1.630505 1.673614 0.191376
H 0.830690 -1.895083 -0.766031
FREQS= 34 55 68 76 82 97 124 151 216 265 293 331 362 378 403 407 421 433 454 496 518 546 581 674 823 887 920 934 1022 1042 1140 1250 1310 1396 1419 1499 1510 1518 2123 2134 2173 2988 3044 3048 3114 3125 3137 3823
MIN202_8
18
E= -1916.36353390 ZPE= 74.02 Gcorr 0.050510000
H -2.914035 1.427792 0.836472
Co 0.365062 -0.061017 -0.243383
C 0.563532 1.613351 0.165772
C 0.643508 -0.842310 1.380424
O 0.693418 2.736996 0.365340
O 0.871799 -1.269170 2.419378
C 2.166596 -0.542857 -0.839792
O 3.214221 0.018772 -0.648189
C -2.900933 -0.163224 -0.652798
C -3.457159 0.506718 0.608144
H -3.438503 -1.085006 -0.905340
H -3.002557 0.517435 -1.511035
H -4.511755 0.756548 0.467129
H -3.380933 -0.156799 1.474255
C -1.435985 -0.452913 -0.572377
O -0.877527 -1.441463 -1.050499
H 2.141383 -1.483330 -1.453343
H 0.557086 0.597155 -1.561267

FREQS= 36 53 67 76 84 95 149 170 182 217 305 329 389 423 443 445 461 501 517 539 556
591 653 778 823 878 933 1036 1071 1160 1272 1339 1342 1431 1455 1511 1517 1676 1803 1986 2127 2159 2753
3027 3061 3083 3136 3141
MIN203_8
18
E= -1916.36420438 ZPE= 75.29 Gcorr 0.051189000
H 2.143549 -2.288508 0.391054
Co -0.254132 0.174729 -0.166935
C -1.704758 -1.468171 -0.921963
C -0.691151 -0.281853 1.478599
O -2.810391 -1.666399 -0.484254
O -1.001504 -0.398055 2.582070
C -0.651370 1.835082 -0.621452
O -0.973570 2.934905 -0.743112
C 1.819432 -0.583712 -0.928270
C 2.691426 -1.703300 -0.352864
H 2.345986 -0.020207 -1.704051
H -1.039617 -2.278506 -1.281485
H 3.026202 -2.379428 -1.144629
H 3.569692 -1.271259 0.133003
C 1.465253 0.466820 0.185578
O 2.251168 1.046756 0.869415
H -1.409283 -0.401098 -1.291164
H 0.926432 -1.005537 -1.426194
FREQS= 28 57 71 80 89 99 124 147 164 206 242 261 292 333 377 397 462 491 506 527 569
667 698 763 820 982 1062 1090 1170 1272 1312 1415 1425 1455 1466 1510 1515 1803 1892 2086 2135 2273 2935
2966 3059 3096 3134 3146
MIN204_8
18
E= -1916.36338996 ZPE= 73.99 Gcorr 0.050379000
H -3.339322 1.378091 -0.896015
Co 0.330863 -0.055012 -0.194765
C 0.415557 1.548294 0.464415
C 0.985060 -1.031382 1.199376
O 0.457508 2.635146 0.833390
O 1.447558 -1.578649 2.094052
C 2.038677 -0.181175 -1.143600
O 3.033217 0.487073 -1.024754
C -2.920626 -0.590127 -0.057440
C -3.466296 0.838229 0.046349
H -3.070053 -1.114603 0.897818
H -3.432602 -1.174779 -0.831181
H -4.533264 0.815855 0.282168
H -2.953110 1.399352 0.831923
C -1.445477 -0.639644 -0.301635
O -0.871432 -1.455088 -1.025203
H 2.010498 -1.010329 -1.900641
H 0.208370 0.818698 -1.390097
FREQS= 36 53 69 76 82 96 144 165 190 217 307 326 393 416 436 444 474 494 518 539 546
591 652 777 826 878 934 1037 1071 1158 1272 1339 1343 1430 1456 1510 1517 1675 1802 1979 2126 2159 2753
3027 3062 3081 3138 3141
MIN205_8
18
E= -1916.37034354 ZPE= 77.28 Gcorr 0.057377000
H 2.869955 1.275331 0.459642
Co -0.242965 -0.065476 -0.420623
C -1.436911 -0.020627 0.940011
C 1.268078 -1.076607 0.245989
O -1.400467 0.056788 2.140176
O 0.408355 -1.732956 -0.527129
C -0.001780 1.656755 -0.398325
O 0.299074 2.770311 -0.383884
C 2.661873 -0.777325 -0.255079
C 3.377093 0.310058 0.553040
H 2.616651 -0.514588 -1.318672
H 3.235439 -1.715116 -0.198177
H 3.412626 0.054027 1.617360
H 4.406673 0.437533 0.207521
C -2.714022 -0.347895 0.138632
O -2.611007 -0.401475 -1.074243
H 1.190917 -1.243322 1.330055
H -3.665838 -0.533524 0.674118
FREQS= 52 62 68 89 96 105 131 142 173 235 321 346 401 442 464 476 506 518 583 598 656
786 903 924 959 1011 1039 1111 1155 1266 1292 1330 1335 1430 1463 1487 1513 1519 1749 1836 2106 2970 3013
3031 3050 3070 3119 3128
MIN206_8
18

E= -1916.35959701 ZPE=   72.09 Gcorr   0.046984000
H -2.648043 -0.133519 1.959911
Co 0.465429 0.024191 -0.549325
C 0.265364 -1.720485 -0.771100
C 0.114322 1.751993 -0.707673
O 0.073246 -2.831470 -0.964791
O -0.172532 2.848844 -0.860487
C 1.996731 0.075614 0.430857
O 3.002720 0.111158 0.968304
C -3.100368 -0.043775 -0.188868
C -2.159122 -0.082792 0.979234
H -3.751251 -0.927840 -0.208676
H -3.763052 0.830608 -0.142205
H -0.740899 -0.149048 2.905865
H -2.570434 -0.004555 -1.143611
C -0.817449 -0.064347 0.977089
O -0.110442 -0.109178 2.167169
H -0.585266 -0.006343 -1.573952
H 1.351561 0.085455 -1.764364
FREQS=   13   57   71   91   94 105 142 201 236 305 341 356 381 416 425 445 467 472 493 508 553
555 572 645 670 738 773 801 944 1062 1112 1127 1249 1369 1430 1496 1507 1693 1918 2086 2153 2176 2207
3020 3057 3062 3125 3713
MIN207_8
18
E= -1916.36030201 ZPE=   72.58 Gcorr   0.048296000
H 4.188622 0.715886 -0.852948
Co -0.679771 -0.002443 0.195131
C -2.464024 0.006864 0.376313
C -0.415441 1.498039 -0.783689
O -3.581029 0.009956 0.624424
O -0.229927 2.505566 -1.292819
C -0.428574 -1.480676 -0.817781
O -0.249743 -2.477285 -1.350623
C 2.206855 0.007573 -0.390744
C 3.698973 0.019670 -0.163242
H 1.887607 0.012407 -1.428307
H 4.155457 -0.965948 -0.326015
H 3.981155 0.343899 0.846818
H 2.512025 -0.099343 2.008605
C 1.256237 -0.018679 0.564587
O 1.548726 -0.051276 1.900714
H -0.690696 0.875031 1.369982
H -0.708732 -0.908400 1.347104
FREQS=   51   51   61   78   92   94 105 199 229 286 316 338 363 396 403 420 467 476 514 528 546
587 640 669 700 776 784 871 952 1038 1060 1138 1306 1354 1429 1493 1513 1668 2078 2091 2153 2162 2192
3016 3066 3092 3186 3740
MIN208_8
18
E= -1916.36234618 ZPE=   75.42 Gcorr   0.050530000
H 4.645879 -0.172465 0.823425
Co -0.408734 0.018634 -0.237430
C -0.609563 -0.149846 1.570766
C 1.501633 0.134129 -0.605247
O -1.454527 -0.861481 2.046978
O 0.916155 -1.012863 -0.916695
C -1.029384 1.643478 -0.170836
O -1.323593 2.757514 -0.223012
C 2.517371 0.186138 0.512972
C 3.913551 -0.224406 0.012129
H 2.206436 -0.485399 1.320447
H 2.556059 1.204839 0.919549
H 4.256399 0.434336 -0.792813
H 3.898585 -1.248449 -0.371600
C -2.665700 -0.792882 -0.816159
O -1.909840 -1.657150 -0.815791
H 0.161305 0.356505 2.197040
H 1.718127 0.819685 -1.439041
FREQS=   46   56   65   79   88   95 103 128 147 159 186 198 235 265 333 448 480 504 531 565 614
644 797 888 899 993 1009 1102 1155 1277 1292 1310 1345 1419 1474 1486 1512 1520 1807 2100 2107 2862 3007
3040 3048 3091 3121 3133
MIN209_8
18
E= -1916.36131469 ZPE=   73.91 Gcorr   0.049584000
H -4.510946 0.107054 0.251035
Co 0.421828 0.051859 -0.208767
C 0.115179 1.737821 -0.435651
C -1.223185 -0.828044 -0.330393

O -0.086087 2.849069 -0.658839
O -0.462344 -1.784031 -0.434890
C 0.780706 -0.007583 1.576941
O 1.045475 0.034345 2.691622
C -2.713457 -0.869221 -0.462521
C -3.434867 0.135870 0.440075
H -3.056942 -1.897573 -0.298977
H 0.556464 0.228182 -1.678815
H -3.272083 -0.094090 1.496903
H -3.082214 1.154298 0.255321
C 2.338733 -0.152723 -0.623470
O 2.846251 -1.240802 -0.732548
H -2.905234 -0.629337 -1.518799
H 2.956594 0.765912 -0.782589
FREQS= 29 51 64 75 84 101 123 162 185 202 289 337 365 402 444 449 455 504 524 541 560
601 644 774 815 865 934 1037 1073 1155 1272 1338 1360 1430 1453 1509 1515 1703 1792 1979 2114 2152 2793
3028 3060 3084 3135 3140
MIN210_8
18
E= -1916.36120073 ZPE= 73.99 Gcorr 0.049938000
H -4.548368 0.102197 0.006052
Co 0.379653 0.066716 -0.154601
C 0.000337 1.753501 -0.136542
C -1.227939 -0.872562 0.026047
O -0.264475 2.872145 -0.197467
O -0.488992 -1.777995 -0.345569
C 1.160166 -0.139800 1.479436
O 1.688571 -0.189370 2.495661
C -2.696957 -0.985858 0.290527
C -3.494122 0.200853 -0.264628
H -2.795419 -1.018127 1.384878
H -3.057154 -1.944431 -0.100967
H -3.123907 1.147629 0.137773
H -3.427055 0.244562 -1.355105
C 2.144083 -0.015641 -1.030426
O 2.634319 -1.066951 -1.358908
H 0.147989 0.381662 -1.588752
H 2.684476 0.939582 -1.245883
FREQS= 31 53 70 73 84 101 119 163 186 212 295 322 373 401 429 446 485 502 516 541 558
600 651 784 816 865 932 1037 1070 1161 1273 1336 1360 1429 1460 1511 1517 1702 1791 1978 2114 2152 2793
3032 3062 3086 3139 3141
MIN211_8
18
E= -1916.36339842 ZPE= 75.21 Gcorr 0.052227000
H 3.060012 0.132534 1.031399
Co -0.358883 -0.057039 -0.110803
C 0.525537 1.262598 0.683534
C -1.886592 0.942334 -0.416658
O 0.969143 2.187495 1.200188
O -2.850739 1.474401 -0.735218
C -0.819927 -1.118303 1.316637
O -1.002409 -1.901093 2.130551
C 2.569695 -0.693313 -0.926414
C 3.310963 0.288375 -0.022291
H 2.874242 -0.540743 -1.976967
H 2.907210 -1.723842 -0.714664
H 4.391923 0.153184 -0.125963
H 3.081995 1.325887 -0.278976
C 1.080191 -0.748071 -0.935846
O -1.461524 -1.138442 -1.263323
H -1.288658 -0.852623 -2.170713
H 0.748139 -1.534961 -1.623787
FREQS= 22 67 71 82 104 126 136 185 202 249 294 305 373 388 395 410 433 443 467 492 523
545 583 669 686 876 919 936 1032 1049 1136 1278 1292 1372 1424 1435 1515 1518 2115 2130 2170 2969 2990
3054 3074 3121 3136 3817
MIN212_8
18
E= -1916.36021841 ZPE= 73.24 Gcorr 0.049570000
H -4.126137 0.101510 -0.673349
Co 0.517502 0.009031 -0.234400
C 0.170249 -1.736240 -0.461067
C 0.274737 1.758039 -0.439753
O -0.094067 -2.830227 -0.656790
O 0.064274 2.869142 -0.617690
C 2.316801 -0.059495 0.138833
O 3.403017 -0.100943 0.482932
C -2.316475 -0.005465 0.512724

C -3.813290 0.020619 0.371746
H 0.707227 0.028611 -1.711225
H -1.907127 -0.093450 1.518660
H -4.259044 -0.888341 0.794963
H -4.242731 0.865210 0.925335
C -1.458052 0.056113 -0.506051
O 0.406183 -0.109047 1.697291
H 0.376775 0.783655 2.066912
H -1.800573 0.126130 -1.537051
FREQS= 50 59 84 95 104 112 150 172 185 224 250 261 308 360 404 413 426 459 486 519 528 539 567 645 727 778 792 927 950 1019 1084 1091 1219 1315 1418 1493 1496 1671 1958 2148 2175 2203 3028 3081 3118 3158 3163 3819
MIN213_8
18
E= -1916.36011000 ZPE= 72.65 Gcorr 0.049502000
H -2.675728 0.000702 2.576268
Co 0.569187 0.000065 0.245153
C 2.356960 0.000019 0.197240
C 0.183563 1.465519 -0.747243
O 3.497232 0.000026 0.303142
O -0.054987 2.461159 -1.256253
C 0.183503 -1.465880 -0.746475
O -0.055070 -2.461808 -1.254908
C -1.313324 0.000263 0.893241
C -1.596477 0.000741 2.372107
H 0.748587 0.921836 1.376395
H 0.748450 -0.921292 1.376740
H -1.162039 -0.876745 2.865556
H -1.162222 0.878665 2.864940
C -2.338279 -0.000015 0.039257
O -2.175057 -0.000435 -1.336255
H -3.374619 0.000068 0.381264
H -3.043102 -0.000423 -1.754851
FREQS= 42 57 91 96 97 108 158 176 227 283 284 322 382 397 421 433 448 482 487 516 530 552 580 654 661 774 821 860 1002 1064 1103 1147 1269 1359 1427 1481 1509 1733 2050 2070 2150 2163 2194 3013 3080 3080 3127 3861
MIN214_8
18
E= -1916.35751443 ZPE= 72.22 Gcorr 0.047043000
H -3.618671 -0.978784 1.707354
Co 0.841189 0.123683 -0.283862
C 0.333421 1.687293 -0.946259
C 1.091700 0.561936 1.449555
O 0.011725 2.671260 -1.432546
O 1.304328 0.852755 2.533815
C 1.291416 -1.593640 -0.207970
O 1.604325 -2.692873 -0.231936
C -1.949122 0.041046 0.784676
C -3.409225 -0.320114 0.854109
H 0.901615 -0.162891 -1.735270
H -1.554763 0.640604 1.600087
H -4.031124 0.574395 0.985394
H -3.732248 -0.833036 -0.053540
C -1.113048 -0.323660 -0.200221
O -1.603019 -1.116132 -1.219747
H 2.293319 0.475338 -0.486060
H -0.979962 -1.092314 -1.953709
FREQS= 24 54 71 91 94 100 108 178 214 280 303 317 345 398 403 444 480 488 492 545 559 578 647 670 697 740 776 785 951 1047 1053 1135 1251 1350 1418 1501 1505 1700 1902 2018 2155 2168 2204 3025 3069 3145 3183 3857
MIN215_8
18
E= -1916.35529619 ZPE= 71.85 Gcorr 0.045100000
H 4.096331 0.881082 1.146852
Co -0.697727 0.000001 0.011038
C -2.485071 -0.000024 0.170522
C -0.408991 1.747116 -0.004329
O -3.625463 -0.000043 0.252327
O -0.185042 2.869636 -0.013377
C -0.408949 -1.747105 -0.004357
O -0.184986 -2.869622 -0.013442
C 2.157679 -0.000053 0.782241
C 3.659830 -0.000008 0.660551
H 1.758444 -0.000178 1.789553
H -0.880366 -0.000005 -1.483454
H 4.096341 -0.881934 1.145340
H 4.021406 0.000915 -0.375005

C 1.273879 0.000009 -0.231478
O 1.617781 0.000098 -1.556317
H -0.547573 0.000010 1.517006
H 2.585459 -0.000070 -1.630747
FREQS= 28 31 37 80 95 98 108 179 231 246 305 337 341 389 400 453 490 506 540 551 559
595 611 687 688 694 798 800 947 1043 1043 1132 1288 1338 1432 1486 1513 1679 1828 2002 2147 2160 2199
3023 3081 3084 3215 3740
MIN216_8
18
E= -1916.35992765 ZPE= 75.31 Gcorr 0.050029000
H 3.992850 -0.596514 1.025237
Co -0.385085 0.102238 -0.245788
C -0.193988 -0.444653 1.485554
C 1.364071 0.703556 -0.894103
O -0.668979 -1.487358 1.856869
O 0.900049 -0.417633 -1.416197
C -1.238546 1.516969 0.290926
O -1.732028 2.531477 0.532808
C 2.605648 0.697320 -0.018607
C 3.048954 -0.680175 0.478999
H 2.462771 1.397847 0.815470
H 3.412510 1.137496 -0.623320
H 3.195707 -1.364347 -0.360078
H 2.318861 -1.141386 1.149709
C -2.498476 -0.997089 -0.688120
O -1.651854 -1.721050 -0.966699
H 0.461349 0.171344 2.143780
H 1.249774 1.616085 -1.496658
FREQS= 42 50 60 79 83 93 103 125 144 155 180 202 229 285 318 454 459 493 555 592 631
651 771 859 891 972 1026 1103 1126 1282 1303 1309 1366 1424 1473 1487 1509 1516 1798 2096 2105 2860 3010
3027 3048 3063 3130 3143
MIN217_8
18
E= -1916.36107506 ZPE= 76.04 Gcorr 0.051893000
H -0.045167 2.660061 -1.292530
Co -0.611527 -0.169704 -0.148371
C 2.771125 -0.063981 0.625157
C -2.247205 -0.101942 0.346860
O 3.964294 -0.010820 0.838667
O -3.362721 -0.017142 0.632475
C -0.562854 -1.933129 -0.265274
O -0.468575 -3.072321 -0.109763
C -0.526406 1.677767 0.610878
C -0.729211 2.767431 -0.443867
H 0.499146 1.707831 1.011111
H -1.194728 1.859751 1.456757
H -1.747550 2.754825 -0.847385
H -0.568404 3.767914 -0.019259
C 2.216372 0.203025 -0.737893
O 1.003285 0.151345 -0.983990
H 2.928357 0.443566 -1.536223
H 2.018373 -0.315471 1.399270
FREQS= 26 36 57 63 92 108 120 151 180 200 252 318 367 384 415 461 472 489 530 586 593
598 731 831 926 986 1021 1023 1078 1208 1266 1343 1388 1425 1473 1511 1512 1642 1786 2075 2120 2957 2993
3019 3079 3092 3102 3112
MIN218_8
18
E= -1916.36164242 ZPE= 74.60 Gcorr 0.052496000
H 2.885939 2.479721 -0.535033
Co -0.040418 -0.026520 -0.220666
C 1.308714 -1.303511 -0.259971
C -0.578196 -0.252524 1.558223
O 2.174603 -2.049001 -0.264152
O -0.181615 -1.123758 2.272843
C -1.369585 -1.021554 -0.901199
O -2.244451 -1.640835 -1.309514
C 1.343132 1.286572 0.432314
C 2.132240 1.728609 -0.805884
H 2.012685 0.817605 1.159524
H 0.895025 2.153989 0.924680
H 2.670388 0.901493 -1.284001
H 1.497346 2.189674 -1.572125
C -1.105169 1.518513 -0.357352
O -1.877713 2.052718 0.399065
H -0.906106 1.988480 -1.368350
H -1.317428 0.535451 1.850573

FREQS= 57 75 80 85 92 109 153 170 198 224 238 305 321 343 382 412 439 466 490 519 526
564 644 763 877 941 968 981 1013 1225 1236 1262 1296 1427 1498 1507 1513 1796 1841 2127 2169 2673 2820
3020 3061 3079 3086 3118
MIN219_8
18
E= -1916.35645554 ZPE= 72.12 Gcorr 0.047591000
H -4.208613 0.965582 -0.095123
Co 0.729237 -0.029974 -0.461779
C 0.706289 1.726609 -0.686467
C 1.870924 -0.229642 0.917793
O 0.650696 2.849129 -0.904122
O 2.678691 -0.366348 1.717319
C 0.251256 -1.716034 -0.737910
O -0.065750 -2.787534 -0.985192
C -2.241174 0.098289 -0.254713
C -3.604648 0.180218 0.375686
H 0.158920 0.081455 -1.812801
H -2.197833 0.006755 -1.336093
H -4.157115 -0.760515 0.251225
H -3.532143 0.389543 1.444361
C -1.077530 0.124129 0.413853
O -1.113699 0.126232 1.796936
H 1.981145 -0.177897 -1.283805
H -0.363964 0.631143 2.131285
FREQS= 36 56 68 91 93 100 136 186 210 286 296 324 342 379 400 447 458 490 495 539 558
561 638 652 697 707 772 815 951 1039 1054 1134 1231 1333 1417 1499 1505 1694 1913 2067 2148 2156 2195
3027 3074 3146 3187 3845
MIN220_8
18
E= -1916.36124624 ZPE= 74.69 Gcorr 0.052479000
H -2.708587 0.808367 -0.898535
Co 0.076831 0.099259 -0.071920
C 0.745135 -0.394255 1.596262
C 0.106513 -1.586167 -0.868022
O 1.249650 -1.456657 1.831436
O 0.073135 -2.627253 -1.335747
C 1.658150 0.720859 -0.672096
O 2.677003 1.132392 -0.997598
C -1.738565 -0.361034 0.681984
C -2.739212 -0.196179 -0.464474
H -2.017867 0.285026 1.522659
H -1.750231 -1.387514 1.061347
H -3.763943 -0.360448 -0.106007
H -2.569146 -0.914063 -1.275212
C -0.418476 1.811241 0.500993
O -0.904879 2.496803 -0.368160
H -0.340109 2.169316 1.562027
H 0.634904 0.390242 2.388232
FREQS= 56 76 80 89 100 123 136 149 198 227 237 254 284 330 376 411 426 474 491 512 519
621 671 762 889 906 971 985 1023 1232 1260 1286 1322 1427 1494 1507 1518 1775 1820 2134 2174 2770 2799
3025 3040 3082 3092 3115
MIN221_8
18
E= -1916.36213336 ZPE= 76.12 Gcorr 0.054766000
H 2.994480 -0.213279 0.577978
Co -0.173364 0.300891 -0.319835
C -1.564055 -0.961683 -0.896552
C 0.394432 -0.854853 1.089725
O -1.372737 -2.363812 -0.873805
O 0.076388 -0.929951 2.230179
C -1.470791 1.370893 0.223955
O -2.294242 1.997181 0.729136
C 1.147031 -1.202287 -0.052291
C 2.664614 -1.066623 -0.020621
H 0.751247 -2.090172 -0.540997
H -1.853728 -2.715650 -0.114407
H 3.118609 -1.967630 0.409321
H 3.065938 -0.939509 -1.030383
C 1.122808 1.539052 -0.391905
O 1.946121 2.322470 -0.562546
H -1.408945 -0.629489 -1.945908
H -2.595247 -0.722421 -0.617643
FREQS= 43 48 82 90 92 144 165 175 185 248 276 305 309 353 432 438 446 483 498 543 553
566 617 685 842 908 1045 1056 1069 1115 1133 1260 1340 1374 1425 1462 1501 1515 1968 2092 2140 2894 3038
3070 3103 3127 3161 3834
MIN222_8
18

E= -1916.35862999 ZPE= 74.42 Gcorr 0.051623000
H -3.377743 1.850972 -0.049842
Co 0.235293 0.061519 -0.162339
C -0.220193 -0.227738 1.618935
C -0.863254 -1.207149 -0.973476
O -0.908549 -1.134058 1.998424
O -1.515061 -1.976812 -1.508891
C 1.726603 -0.942465 -0.071541
O 2.685290 -1.560019 0.044046
C -1.254598 1.420240 -0.186883
C -2.707209 0.984129 0.007216
H -1.106314 1.815825 -1.204779
H -1.046687 2.254030 0.494936
H -3.036195 0.272569 -0.756811
H -2.870362 0.512523 0.981135
C 1.245577 1.599320 0.193546
O 1.735503 2.078896 -0.801155
H 1.344454 2.085111 1.200261
H 0.200925 0.525855 2.332081
FREQS= 53 76 81 85 99 108 122 139 210 236 249 261 284 330 373 400 422 441 495 512 521 619 661 686 887 907 942 1009 1042 1223 1284 1292 1318 1428 1452 1512 1514 1780 1817 2133 2173 2776 2803 2993 3034 3054 3096 3121
MIN223_8
18
E= -1916.35785509 ZPE= 74.36 Gcorr 0.051025000
H -2.739041 -0.893494 1.308057
Co 0.141246 0.117398 -0.200220
C -0.005871 -0.501298 1.549266
C 0.967300 -1.387435 -0.939053
O 0.532647 -1.490558 1.961592
O 1.413153 -2.322837 -1.417817
C 1.633771 1.076081 0.123832
O 2.565296 1.694145 0.376179
C -1.623749 -0.785517 -0.573905
C -2.869262 -0.546609 0.275953
H -1.475411 -1.865440 -0.670683
H -1.780623 -0.392748 -1.592262
H -3.722941 -1.101275 -0.133415
H -3.167256 0.506007 0.316013
C -0.895027 1.650718 0.074077
O -1.028225 2.301952 -0.936174
H -1.381327 1.947852 1.039010
H -0.652990 0.132107 2.207945
FREQS= 51 59 78 81 89 105 121 148 221 237 247 266 292 331 369 400 431 439 483 508 523 613 659 683 890 907 930 1010 1039 1219 1282 1297 1316 1426 1453 1506 1516 1775 1819 2134 2175 2787 2805 2991 3029 3080 3083 3110
MIN224_8
18
E= -1916.35645875 ZPE= 72.91 Gcorr 0.049970000
H 2.527852 1.620385 0.796944
Co -0.565186 -0.069165 -0.339839
C -2.197689 -0.827760 -0.325854
C 0.162819 -1.229146 0.813536
O -3.238140 -1.309267 -0.294350
O 0.614989 -1.928681 1.603309
C -1.126915 1.536386 0.219448
O -1.479482 2.551071 0.625970
C 2.307498 -0.130080 -1.022236
C 3.322268 -0.684200 -0.348599
H 1.146504 1.629633 -1.152255
H 2.201931 -0.333193 -2.087999
H 3.451983 -0.520338 0.716784
H 4.043628 -1.327606 -0.840896
C 1.288397 0.793311 -0.450158
O 1.624001 1.270081 0.841558
H -0.237813 -0.682605 -1.817905
H -0.583269 0.064477 -1.967739
FREQS= 49 52 80 92 96 106 137 232 252 263 296 351 363 365 432 446 460 480 497 524 552 553 633 658 671 725 916 924 978 1018 1037 1127 1166 1289 1330 1380 1450 1594 1697 2114 2119 2169 2981 3046 3146 3168 3253 3738
MIN225_8
18
E= -1916.35614390 ZPE= 72.93 Gcorr 0.049903000
H 1.367059 0.878173 2.530722
Co -0.674589 0.000001 0.198621
C -2.458299 -0.000001 0.348799
C -0.391951 -1.489248 -0.783378

O -3.580929 -0.000002 0.570712
O -0.187709 -2.493607 -1.292953
C -0.391951 1.489247 -0.783382
O -0.187709 2.493605 -1.292960
C 1.295675 0.000001 0.556999
C 1.745752 0.000005 1.995861
H -0.716322 0.911413 1.352536
H -0.716319 -0.911409 1.352538
H 1.367059 -0.878158 2.530727
H 2.839132 0.000006 2.118360
C 2.164122 -0.000003 -0.468385
O 3.534265 -0.000004 -0.415624
H 1.853571 -0.000006 -1.509307
H 3.816296 -0.000001 0.509179
FREQS= 49 54 77 93 97 106 134 229 243 292 300 345 346 396 397 424 472 483 496 516 531
549 652 654 691 784 867 902 963 1055 1091 1146 1279 1416 1433 1476 1510 1688 2051 2068 2151 2157 2189
3002 3072 3092 3193 3808
MIN226_8
18
E= -1916.35900436 ZPE= 74.59 Gcorr 0.052787000
H -0.173886 3.112706 -1.192246
Co 0.001386 0.069165 -0.184319
C 1.829569 -0.300712 -0.238796
C -0.206668 -0.700725 1.532768
O 2.948927 -0.526540 -0.242339
O 0.663648 -1.265322 2.133829
C -0.564965 -1.360875 -1.094649
O -0.963108 -2.285447 -1.645645
C 0.395495 1.872000 0.527201
C 0.540599 2.329177 -0.922242
H 1.313599 1.955804 1.107119
H -0.414818 2.367915 1.060446
H 1.554016 2.658557 -1.166579
H 0.329289 1.511669 -1.664404
C -1.824916 0.669392 -0.094207
O -2.693416 0.412041 0.703250
H -2.058230 1.384744 -0.931707
H -1.250492 -0.586256 1.910775
FREQS= 56 66 84 88 106 121 146 173 193 207 269 285 320 361 401 410 441 479 490 523 542
592 647 824 893 914 943 993 1003 1150 1183 1265 1346 1432 1468 1502 1518 1791 1823 2127 2171 2733 2756
2856 3069 3111 3134 3173
MIN227_8
18
E= -1916.35589045 ZPE= 73.10 Gcorr 0.049763000
H 1.900929 0.713516 1.399823
Co -0.588025 -0.077613 -0.331879
C -2.248478 -0.785892 -0.325021
C 0.108672 -1.244903 0.811175
O -3.302599 -1.235971 -0.284120
O 0.562922 -1.931026 1.617450
C -1.097029 1.553893 0.205005
O -1.420171 2.580772 0.605222
C 2.302149 -0.268477 -0.965868
C 3.448902 -0.612327 -0.371801
H 1.194263 1.529148 -1.196218
H 2.032728 -0.739218 -1.909814
H 3.779532 -0.145149 0.550696
H 4.107382 -1.356926 -0.805993
C 1.328288 0.738936 -0.451462
O 1.745150 1.386443 0.722604
H -0.284138 -0.679174 -1.820321
H -0.591452 0.084221 -1.958846
FREQS= 46 51 75 86 94 98 117 233 243 269 304 358 360 387 432 447 465 481 503 523 546
548 629 656 659 712 909 921 986 1021 1036 1129 1137 1274 1332 1420 1456 1593 1700 2095 2116 2163 3066
3084 3158 3170 3251 3798
MIN228_8
18
E= -1916.35592527 ZPE= 73.38 Gcorr 0.049885000
H 2.390780 0.302053 -1.213055
Co -0.471706 0.087688 0.220470
C 0.273743 1.611209 -0.309417
C 0.072304 -1.201569 -1.013674
O 0.730803 2.638174 -0.525221
O 0.412075 -2.083844 -1.648147
C -2.243268 0.369299 -0.256203
O -3.372637 0.481366 -0.366229
C 2.461720 -0.294150 0.884414

C 3.108567 0.039193 -0.431147
H -1.246994 -1.219434 2.040412
H 3.170823 -0.601653 1.658217
H 3.802373 0.882543 -0.324585
H 3.700883 -0.808571 -0.797193
C 1.172503 -0.276311 1.233731
O -1.309271 -1.384853 1.090679
H -0.765633 0.950759 1.376287
H 0.922635 -0.566037 2.252356
FREQS= 54 64 70 83 100 107 138 169 188 213 278 343 363 385 407 415 427 450 464 492 516
528 580 642 686 786 820 926 936 970 1061 1068 1254 1339 1426 1494 1503 1687 2030 2154 2183 2208 3032
3081 3094 3123 3157 3833
MIN229_8
18
E= -1916.35661591 ZPE= 73.38 Gcorr 0.050715000
H -3.629912 1.832590 0.359601
Co 0.188635 0.148580 -0.164716
C 1.244463 1.632463 -0.415559
C 0.812110 -0.188380 1.664712
O 1.914471 2.516102 -0.699854
O 1.804489 -0.739972 2.065086
C 1.152341 -1.144667 -0.839216
O 1.764677 -1.979059 -1.327689
C -1.909922 0.530032 0.588123
C -3.166509 0.986168 -0.154593
H -1.192011 1.372385 0.639439
H -2.093971 0.256426 1.633426
H -2.926307 1.289160 -1.176359
H -3.882834 0.161834 -0.199494
C -1.352412 -0.780360 -0.078403
O -1.960203 -1.772371 -0.318231
H -0.325194 0.429014 -1.580466
H 0.089191 0.237797 2.426302
FREQS= 42 64 74 89 94 119 159 170 206 265 304 338 370 386 393 436 472 492 513 522 554
640 667 680 722 827 856 989 1067 1090 1280 1330 1367 1421 1456 1506 1515 1800 1817 1917 2139 2175 2640
2937 3062 3073 3140 3149
MIN230_8
18
E= -1916.35514162 ZPE= 73.00 Gcorr 0.049515000
H 1.288024 2.007840 1.061544
Co -0.586460 -0.067611 -0.332619
C -2.234813 -0.803919 -0.321895
C 0.128793 -1.229066 0.826355
O -3.286042 -1.259742 -0.268234
O 0.565532 -1.930272 1.623770
C -1.092772 1.549503 0.183214
O -1.350853 2.598176 0.590465
C 2.278550 -0.222360 -1.004559
C 3.340243 -0.731011 -0.372067
H 1.198439 1.592099 -1.163356
H 2.063636 -0.528427 -2.026875
H 3.587026 -0.437837 0.641994
H 3.991757 -1.447584 -0.861444
C 1.333348 0.776290 -0.439351
O 1.820662 1.251478 0.787031
H -0.241307 -0.710886 -1.799404
H -0.567623 0.036544 -1.966189
FREQS= 48 51 76 86 90 97 120 234 238 271 296 342 356 365 435 448 466 480 511 524 544
550 621 641 650 708 911 930 981 1019 1029 1126 1191 1247 1334 1410 1460 1584 1703 2083 2116 2160 3006
3095 3159 3175 3264 3836
MIN231_8
18
E= -1916.35611426 ZPE= 73.85 Gcorr 0.050652000
H 2.188521 -1.631101 0.109119
Co -0.527069 -0.025987 -0.454361
C -0.467473 -1.662915 -1.172255
C -1.977961 -0.114211 0.515647
O 0.548809 -1.295810 -1.747189
O -2.959617 -0.187184 1.100831
C -0.148535 1.759892 -0.452264
O 0.007808 2.891845 -0.519086
C 2.920333 0.402141 0.087864
C 2.161813 -0.750847 0.759895
H 3.976598 0.141290 -0.025559
H 2.525760 0.617952 -0.908837
H 2.865752 1.316482 0.687485
H 2.627918 -1.016614 1.714597

C 0.694875 -0.415664 1.089710
O 0.326881 -0.374633 2.241480
H -1.495488 0.332561 -1.595679
H -0.947544 -2.643040 -1.293227
FREQS= 40 47 70 83 94 105 129 184 199 216 299 303 340 384 434 473 500 519 535 547 584
620 698 725 795 859 889 1015 1062 1126 1209 1279 1318 1418 1480 1512 1516 1670 1748 1780 2141 2172 3051
3057 3073 3106 3124 3132
MIN232_8
18
E= -1916.35830085 ZPE= 74.55 Gcorr 0.053071000
H -0.233265 -2.777905 -1.402819
Co -0.050077 -0.015473 -0.060683
C 0.690278 0.564417 1.582521
C 1.645492 -0.461375 -0.724356
O 1.864527 0.688224 1.810786
O 2.689503 -0.730477 -1.098069
C -0.179034 1.604457 -0.816357
O -0.270813 2.664211 -1.245155
C -0.308659 -1.858116 0.585670
C -0.848777 -2.094134 -0.813562
H -1.045529 -1.997189 1.376353
H 0.597703 -2.414029 0.819850
H -1.893498 -2.410609 -0.822309
H -0.887322 -1.146205 -1.433774
C -1.816793 0.276300 0.669844
O -2.825075 0.071304 0.043116
H -1.877236 0.616784 1.741723
H -0.068965 0.811538 2.371430
FREQS= 62 67 84 87 102 133 158 183 199 214 248 301 329 331 383 422 435 480 501 518 550
621 658 828 894 902 916 996 1017 1144 1178 1315 1370 1439 1446 1510 1530 1795 1816 2133 2176 2664 2721
2765 3086 3110 3155 3177
MIN233_8
18
E= -1916.36054064 ZPE= 75.25 Gcorr 0.055580000
H 0.006381 -2.683777 0.327535
Co -0.064174 -0.090667 -0.028263
C -2.074916 -0.876045 0.096390
C 1.260085 -0.294599 1.182536
O -1.863182 -0.375235 1.238449
O 2.069164 -0.481985 1.965793
C -0.333212 1.698362 0.233633
O -0.513896 2.815995 0.384319
C 0.356690 -2.035180 -0.481970
C -1.849835 -0.117830 -1.094358
H 1.427672 -2.210474 -0.609569
H -0.145361 -2.324926 -1.409364
H -1.893384 -0.660326 -2.035626
H -2.250814 0.890242 -1.139336
C 1.091399 0.211887 -1.495783
O 2.252431 0.513830 -1.406017
H 0.609710 0.091995 -2.492464
H -2.278909 -1.955122 0.018883
FREQS= 67 80 88 96 127 140 159 217 225 234 258 321 352 381 402 429 448 476 496 516 537
571 627 796 837 865 911 955 1007 1062 1195 1275 1327 1387 1481 1486 1506 1588 1803 2155 2185 2870 3027
3040 3127 3137 3143 3227
MIN234_8
18
E= -1916.36022777 ZPE= 76.17 Gcorr 0.055409000
H -2.934691 -0.718968 0.088534
Co 0.272882 -0.095221 -0.307050
C 1.178120 1.552363 -0.695548
C -0.653565 0.203842 1.309022
O 0.640409 2.832308 -0.691072
O -0.481803 -0.127647 2.436240
C 1.825426 -0.807642 0.203804
O 2.768916 -1.244940 0.692677
C -1.412043 0.836334 0.284647
C -2.837506 0.367210 0.012402
H -1.302382 1.918817 0.339885
H -0.258443 2.788238 -1.043631
H -3.536562 0.813569 0.730356
H -3.160467 0.655436 -0.992831
C -0.546492 -1.621727 -0.803133
O -1.033099 -2.557962 -1.257770
H 1.119028 1.052725 -1.718571
H 2.226685 1.664804 -0.421162

FREQS=   39   57   88   89   98 133 159 174 208 258 277 310 340 430 443 464 471 490 509 527 554
563 638 713 842 938 1051 1064 1103 1125 1161 1220 1331 1350 1426 1473 1501 1514 1955 2100 2145 2602 3036
3100 3124 3139 3142 3810
MIN235_8
18
E= -1916.35510582 ZPE=   73.38 Gcorr   0.050630000
H -3.441438 -1.927962 0.584704
Co 0.282115 -0.109306 -0.294333
C 1.613443 -1.347233 -0.549228
C 1.133407 1.417188 -0.243384
O 2.488318 -2.032209 -0.826192
O 1.695297 2.414077 -0.256028
C 0.289716 -0.279401 1.653173
O 0.921360 0.299874 2.499203
C -1.875071 -0.870985 -0.482735
C -2.995127 -0.930432 0.557764
H -1.095744 -1.623834 -0.252789
H -2.213594 -1.126604 -1.488774
H -2.634046 -0.692879 1.561833
H -3.766704 -0.201880 0.296091
C -1.348578 0.606400 -0.597913
O -2.006479 1.578347 -0.784022
H -0.424582 -1.093954 1.981447
H 0.264279 -0.035568 -1.825261
FREQS=   36   63   73   89   95 112 166 196 214 259 310 329 367 390 395 441 453 488 501 514 584
624 649 681 728 822 854 982 1061 1087 1274 1324 1353 1424 1467 1507 1513 1799 1816 1913 2138 2175 2652
2942 3063 3122 3141 3146
MIN236_8
18
E= -1916.35306208 ZPE=   72.69 Gcorr   0.049077000
H -4.203150 -1.029266 -0.206627
Co 0.737954 -0.020639 0.362571
C 2.467343 0.269041 0.054245
C 0.724888 -1.676095 -0.370115
O 3.574132 0.441400 -0.192543
O 0.549480 -2.694222 -0.866633
C 0.241426 1.603084 -0.234828
O -0.098609 2.608028 -0.669564
C -2.908539 0.809186 -0.404252
C -3.466966 -0.412922 0.301221
H -3.263725 1.050229 -1.402599
H -2.694702 1.669388 0.222481
H 0.860142 0.315117 2.115540
H -3.595209 -0.341870 1.376679
C -2.048362 -0.419139 -0.200478
O -1.054159 -0.438237 0.758741
H 1.011711 -0.452608 2.080599
H -1.865291 -1.028426 -1.090267
FREQS=   35   53   77   90   94 101 127 175 248 301 346 350 390 418 431 444 457 481 489 507 561
567 617 704 769 806 850 941 1008 1049 1064 1130 1171 1199 1202 1238 1410 1464 1507 2119 2122 2177 3068
3135 3142 3220 3235 3758
MIN237_8
18
E= -1916.35647183 ZPE=   74.35 Gcorr   0.052626000
H -0.578886 -2.546518 -0.301517
Co -0.020071 -0.076284 -0.025808
C -2.500476 -0.495383 0.096274
C 1.332976 -0.462681 1.140433
O -1.855248 -0.726103 1.123839
O 2.132961 -0.794857 1.884784
C -0.275187 1.654473 0.418143
O -0.425604 2.772095 0.621672
C 0.231934 -1.948271 -0.731660
C -1.729388 -0.042519 -1.080992
H 1.179120 -2.418103 -0.453724
H 0.154324 -1.990658 -1.821780
H -1.770615 -0.790360 -1.881238
H -2.140984 0.891806 -1.483320
C 1.143669 0.386158 -1.422932
O 2.329861 0.560494 -1.328663
H 0.622222 0.493158 -2.402636
H -3.600204 -0.623348 0.112384
FREQS=   66   79   82   86   97 124 140 154 174 221 239 297 328 396 428 436 454 482 515 529 539
639 676 724 782 799 868 922 940 1162 1182 1279 1318 1388 1429 1473 1489 1706 1806 2133 2176 2856 2966
3036 3048 3095 3123 3129
MIN238_8
18

E= -1916.35326638 ZPE=   72.82 Gcorr   0.049817000
H -3.824558 0.240110 -1.607720
Co 0.848502 0.099255 -0.453586
C 2.341487 -0.752529 -0.378799
C 1.202593 1.554972 0.566391
O 3.320403 -1.351885 -0.393671
O 1.493779 2.498473 1.145213
C -0.349633 -0.907172 0.695666
O -0.443838 -1.756262 1.549217
C -3.012211 -0.466961 0.281293
C -3.973086 0.394972 -0.536048
H -3.160713 -0.350295 1.364847
H -3.168142 -1.542402 0.108931
H -5.008380 0.143981 -0.292049
H -3.820059 1.457694 -0.330736
C -1.565959 -0.217547 0.029758
O -1.118668 0.603095 -0.794571
H 1.511164 0.827896 -1.606100
H 0.688579 -1.038635 -1.389412
FREQS=   41   56   58   81   87 142 145 198 251 258 309 327 381 411 430 461 524 537 570 590 620
677 711 722 778 790 850 991 1058 1068 1165 1258 1391 1429 1439 1511 1513 1671 1815 1866 2012 2141 2173
3030 3053 3065 3141 3143
MIN239_8
18
E= -1916.35364782 ZPE=   73.55 Gcorr   0.050450000
H -3.766384 0.725426 -0.168964
Co 0.415799 0.044553 -0.197372
C 0.845714 1.940035 -0.666430
C 0.423873 0.480498 1.550780
O -0.026390 2.730369 -0.953038
O 0.529364 0.725959 2.666926
C 1.755904 -1.044989 -0.331448
O 2.686455 -1.707709 -0.462508
C -1.818950 -0.053944 -0.708968
C -3.037248 0.008742 0.218207
H -2.084269 -0.338066 -1.730346
H -1.367277 0.949690 -0.793873
H -3.508676 -0.974823 0.288322
H -2.753870 0.326306 1.225714
C -0.875089 -1.242562 -0.228458
O -1.217997 -2.359933 -0.027097
H 0.806984 0.200049 -1.627238
H 1.910250 2.272315 -0.660946
FREQS=   36   67   77   81   98 114 142 196 204 251 288 302 335 363 394 425 439 484 507 547 560
574 639 658 758 822 879 984 1062 1079 1282 1335 1354 1424 1470 1508 1513 1756 1916 1980 2118 2154 2837
2982 3059 3110 3136 3144
MIN240_8
18
E= -1916.35170066 ZPE=   73.31 Gcorr   0.049848000
H 2.453668 0.339692 -1.153300
Co -0.469491 0.080942 0.200084
C 0.294632 1.597100 -0.297317
C 0.070484 -1.154659 -1.049646
O 0.762934 2.621056 -0.504057
O 0.430981 -2.007117 -1.718528
C -2.247401 0.382322 -0.229796
O -3.378337 0.499554 -0.326984
C 2.454221 -0.353028 0.914662
C 3.146461 0.057640 -0.355461
H -1.438494 -2.085403 0.724797
H 3.133536 -0.710993 1.693740
H 3.814225 0.911035 -0.182326
H 3.773799 -0.757999 -0.735554
C 1.155763 -0.349621 1.223508
O -1.368819 -1.264091 1.231512
H -0.706093 0.952057 1.364204
H 0.826593 -0.707569 2.194929
FREQS=   55   65   77   98   99 109 141 163 187 197 215 334 376 389 404 419 433 450 472 499 520
534 572 644 694 760 828 932 974 990 1058 1064 1226 1326 1426 1493 1504 1688 2025 2153 2167 2195 3032
3081 3094 3122 3189 3806
MIN241_8
18
E= -1916.35113580 ZPE=   72.65 Gcorr   0.049973000
H 2.576087 1.678502 0.276579
Co -0.587023 -0.095909 -0.262834
C -2.190867 -0.901987 -0.344787
C 0.135933 -0.908399 1.187613

O -3.174148 -1.455966 -0.536756
O 0.620674 -1.486125 2.045779
C -1.067159 1.569123 0.272686
O -1.406520 2.633465 0.514908
C 2.244229 -0.422301 -1.097078
C 3.288256 -0.820273 -0.361234
H 1.107387 1.298530 -1.570319
H 2.074914 -0.868307 -2.076212
H 3.477952 -0.413672 0.627717
H 3.976978 -1.576801 -0.721863
C 1.263792 0.629930 -0.714825
O 1.667284 1.375023 0.432324
H -0.176364 -1.233230 -1.096547
H -0.890758 0.296800 -1.647126
FREQS= 46 64 80 88 96 106 146 242 253 259 305 362 381 404 416 452 469 499 517 524 540
613 627 644 731 790 873 917 919 1011 1041 1120 1167 1283 1325 1376 1453 1700 2056 2076 2150 2167 2196
3030 3156 3167 3249 3734
MIN242_8
18
E= -1916.35494373 ZPE= 75.77 Gcorr 0.054729000
H 4.096070 0.152391 0.712139
Co -0.662827 -0.127484 -0.104347
C -0.399996 0.837293 1.459827
C 0.981447 -1.015035 -0.241731
O 0.104453 1.912651 1.656857
O 0.526353 -1.723443 -1.133239
C -0.364303 1.239816 -1.151872
O -0.171189 2.135794 -1.842727
C 2.376958 -1.108009 0.307175
C 3.108316 0.243324 0.252505
H 2.923811 -1.890710 -0.231497
H -0.848848 0.255289 2.308843
H 3.248527 0.572160 -0.781567
H 2.548771 1.016095 0.784883
C -2.465016 -0.541997 -0.013928
O -1.987339 -1.410373 0.719385
H 2.275293 -1.423710 1.354313
H -3.549954 -0.488850 -0.203810
FREQS= 44 67 77 90 100 125 150 177 191 226 238 291 324 347 387 457 503 521 552 585 614
641 797 809 859 935 1035 1068 1176 1225 1274 1308 1331 1429 1472 1515 1523 1639 1714 1808 2139 2771 3010
3034 3060 3088 3131 3149
MIN243_8
18
E= -1916.34940033 ZPE= 73.64 Gcorr 0.049322000
H -3.135904 -2.274173 -0.155020
Co 0.031935 0.230193 -0.269245
C -1.400665 1.397817 -0.097078
C 0.188888 -0.519690 1.453096
O -2.283228 2.126485 -0.105743
O 1.047826 -1.225118 1.883025
C 1.346978 1.417165 0.016096
O 2.201687 2.148903 0.237187
C -1.275340 -1.273557 -0.654076
C -2.509025 -1.459022 0.227872
H -0.747084 -2.227952 -0.725173
H -1.590320 -1.006641 -1.675865
H -2.243459 -1.730517 1.256098
H -3.139369 -0.564284 0.277070
C 1.246274 -0.898217 -1.093930
O 2.037262 -1.757474 -0.855915
H 1.057110 -0.550802 -2.173806
H -0.674265 -0.190190 2.085996
FREQS= 41 68 69 85 88 92 112 138 216 248 254 262 314 339 364 403 422 459 484 497 512
533 585 694 851 870 938 1007 1040 1210 1225 1274 1292 1428 1466 1507 1516 1841 1890 2121 2159 2496 2797
2991 3028 3084 3092 3113
MIN244_8
18
E= -1916.35456139 ZPE= 75.75 Gcorr 0.054711000
H 4.128249 0.495243 0.803213
Co -0.591370 -0.251413 -0.070142
C -0.310562 0.684577 1.509601
C 1.150674 -0.395204 -0.751474
O -0.242778 1.865364 1.732810
O 0.730538 -1.189699 -1.587674
C -1.150684 1.153158 -0.947781
O -1.518613 2.072545 -1.527977
C 2.581805 0.054665 -0.649763

C 3.096534 0.133771 0.792419
H 2.605520 1.057303 -1.100648
H 3.201363 -0.599850 -1.274202
H 2.490487 0.819601 1.389500
H 3.080389 -0.848990 1.273263
C -1.991464 -1.361767 0.402830
O -1.036922 -1.992242 0.861348
H -0.230895 -0.059280 2.347027
H -3.023733 -1.738831 0.492624
FREQS= 43 68 73 84 100 131 154 189 203 221 245 287 316 360 403 464 495 515 546 560 617
638 786 812 861 937 1042 1070 1169 1225 1276 1309 1337 1429 1461 1516 1521 1640 1707 1811 2139 2770 3011
3027 3060 3083 3131 3146
MIN245_8
18
E= -1916.34852545 ZPE= 73.19 Gcorr 0.049613000
H 2.697045 -1.464505 0.824075
Co -0.261748 0.064393 -0.201044
C -1.483300 -1.330175 -0.961041
C -0.345072 -0.758523 1.405433
O -2.682693 -1.370168 -0.864930
O -0.468275 -1.221378 2.447774
C -1.214786 1.492665 0.054025
O -1.889877 2.413005 0.171215
C 1.848078 -0.453886 -0.913393
C 3.018388 -1.073676 -0.145647
H 2.136709 -0.086189 -1.899977
H 1.081200 -1.224532 -1.100019
H 3.457146 -1.896939 -0.716104
H 3.786978 -0.316795 0.030329
C 1.368193 0.865874 -0.134625
O 2.092437 1.747545 0.192334
H -0.705589 0.390190 -1.584109
H -0.948049 -2.145556 -1.525670
FREQS= 43 66 73 78 85 104 151 193 205 250 278 293 339 368 397 422 431 471 492 519 547
568 625 645 766 811 873 983 1061 1075 1277 1303 1368 1424 1455 1508 1513 1795 1913 1985 2128 2162 2694
2999 3058 3120 3136 3145
MIN246_8
18
E= -1916.34618535 ZPE= 72.30 Gcorr 0.049984000
H 3.022219 -0.162673 0.253178
Co -0.181031 0.238735 -0.487983
C -1.446188 -1.190422 -0.867245
C 0.499195 -0.811551 1.038522
O -1.655240 -2.178606 -0.205667
O 0.300906 -0.761405 2.205054
C -1.584456 1.080511 0.207464
O -2.469513 1.590687 0.733464
C 1.170422 -1.266336 -0.108151
C 2.676427 -1.083000 -0.224486
H 0.779865 -2.209970 -0.476398
H 0.143876 0.189151 -2.171908
H 3.195624 -1.921415 0.255371
H 2.987099 -1.051098 -1.273186
C 1.015927 1.594647 -0.211145
O 1.722584 2.480832 -0.041499
H -0.409488 0.763698 -2.126048
H -2.009222 -1.048689 -1.826038
FREQS= 55 78 86 93 103 146 172 184 201 256 289 316 342 400 409 435 442 479 507 537 550
587 605 625 633 785 864 906 920 1044 1070 1134 1338 1347 1376 1425 1499 1513 1789 1998 2114 2154 2780
3039 3104 3130 3189 3523
MIN247_8
18
E= -1916.34709021 ZPE= 74.82 Gcorr 0.050989000
H 2.252892 -1.005925 1.413390
Co -0.313316 -0.228422 -0.435093
C -0.118971 -2.038677 -0.186787
C -2.047679 -0.122713 0.107169
O 0.405784 -2.141750 -1.295127
O -2.651800 0.138257 1.101717
C -0.250393 1.449029 -1.089133
O -0.243728 2.544204 -1.430984
C 2.763388 0.357920 -0.192062
C 2.073130 0.042146 1.137652
H 3.843521 0.213249 -0.097015
H 2.422254 -0.293256 -1.003155
H 2.597102 1.397032 -0.492681
H 2.474348 0.657669 1.949196

C 0.534861 0.225833 1.189949
O -0.008014 0.590416 2.195008
H -2.595961 -0.412505 -0.852401
H -0.278579 -2.919113 0.464539
FREQS= 31 59 64 77 83 98 126 156 160 217 255 270 325 336 404 434 460 472 516 550 566 635 777 831 842 874 1023 1070 1113 1229 1273 1277 1324 1432 1469 1514 1518 1668 1821 1889 2136 2569 2961 3037 3050 3097 3122 3125

6. Transition States

In the following, the energies, Cartesian coordinates and vibrational frequencies of the optimized TS structures are listed. The structure of the data of each TS is the following:

1st line: TSX_Y (this is a label explained above)

2nd line: Number of atoms N

3rd line: Electronic energy (E), zero-point energy (ZPE), and Gibbs free-energy correction (Gcorr)

E and Gcorr are given in Hartrees, while ZPE is given in kcal/mol.

4th-$(4+N-1)^{th}$ line: The Cartesian coordinates given in Å.

Last line: Vibrational frequencies in cm^{-1}.

```
TS01_1
8
E= -1723.23713649 ZPE=    19.33 Gcorr   -0.020969000
H 0.000000 1.727371 0.288455
Co 0.000000 0.249060 -0.008657
C 1.728504 0.595856 -0.000730
C -1.728504 0.595856 -0.000730
O 2.830776 0.914654 -0.001305
O -2.830776 0.914654 -0.001305
C 0.000000 -1.506106 -0.070256
O 0.000000 -2.650013 0.049558
FREQS=   89   98  106  251  339  384  466  481  492  522  586  598  757 1934 2108 2117 2193
TS02_1
8
E= -1723.19896918 ZPE=    21.15 Gcorr   -0.018184000
H -0.374851 1.533417 1.763724
Co -0.003184 0.060968 -0.421352
C -0.059174 1.396308 0.714267
C 1.504594 -0.824661 -0.086356
O 0.350361 2.279528 -0.018005
O 2.303903 -1.473280 0.436449
C -1.553430 -0.617907 -0.009047
O -2.515653 -1.168996 0.319005
FREQS=   68  102  119  199  264  330  444  493  532  548  707  856 1247 1712 2058 2122 2991
TS03_1
8
E= -1723.19107388 ZPE=    20.28 Gcorr   -0.020287000
H 0.000000 1.452268 -1.529639
Co 0.000001 -0.394633 0.010425
C -0.000001 1.328489 -0.405471
C -1.775935 -0.589478 0.037729
O -0.000004 2.273080 0.338375
O -2.924106 -0.617442 0.032577
C 1.775937 -0.589474 0.037733
O 2.924107 -0.617439 0.032576
FREQS=   68   89  162  185  206  298  345  416  457  494  564  879 1254 1815 2086 2171 2698
TS04_1
8
E= -1723.18404316 ZPE=    18.24 Gcorr   -0.024527000
H -0.376658 -1.620236 -0.725552
Co 0.038003 -0.268636 -0.202272
C -1.654543 -0.402075 0.026799
C 1.494934 -1.112279 0.738650
O -2.782736 -0.555582 0.200081
O 2.250161 -0.932350 -0.128411
C 0.279746 1.478749 0.057566
O 0.361293 2.623811 0.084430
FREQS=   66   88   98  118  220  345  378  409  471  537  548  572  766 1927 1956 2107 2156
TS05_1
8
E= -1723.18299490 ZPE=    18.38 Gcorr   -0.024230000
H -0.198347 -1.664560 -0.582395
Co 0.096648 -0.265819 -0.135330
C 1.688005 -1.488870 0.389693
C -1.578066 -0.501932 0.020159
O 2.110335 -0.479319 -0.042379
O -2.707492 -0.706371 0.135290
C 0.152038 1.507251 0.078556
```

```
O 0.099282 2.653563 0.070321
FREQS=   45   78  104  143  316  340  397  411  463  553  567  584  751 1885 1957 2106 2158
TS06_1
8
E= -1723.18862140 ZPE=    21.13 Gcorr   -0.018036000
H -0.709379 2.140518 0.337930
Co -0.147496 -0.168552 -0.468151
C -1.820520 -0.249153 0.064394
C 0.177322 1.486903 0.153773
O -2.827967 -0.460353 0.589563
O 1.292683 1.936228 0.261213
C 1.420019 -0.885153 0.006237
O 2.289139 -1.439025 0.518688
FREQS=   60   98  123  267  291  382  465  482  517  536  658  804 1291 1775 2060 2136 2834
TS07_1
8
E= -1723.18209188 ZPE=    18.92 Gcorr   -0.021434000
H 0.040271 -1.382932 -0.851089
Co 0.020772 -0.019170 -0.329087
C -1.604209 -0.600650 -0.000364
C 1.643974 -0.541737 0.056113
O -2.559809 -1.147430 0.337650
O 2.594199 -1.070108 0.438116
C -0.595122 1.809479 0.646106
O 0.306989 1.954783 -0.085102
FREQS=   75  113  139  278  285  355  405  425  470  514  538  599  801 1929 2074 2083 2151
TS08_1
8
E= -1723.17917075 ZPE=    18.32 Gcorr   -0.023684000
H 0.000006 0.303582 1.929115
Co 0.000009 0.330990 0.381065
C -1.693537 0.535447 -0.205717
C 1.693567 0.535378 -0.205699
O -2.778317 0.758495 -0.515870
O 2.778332 0.758461 -0.515882
C -0.000027 -1.334450 -0.015926
O -0.000049 -2.474275 -0.174976
FREQS=   61   92   96  258  270  320  403  416  480  513  569  589  612 1792 2087 2101 2153
TS09_1
8
E= -1723.17937624 ZPE=    18.64 Gcorr   -0.022856000
H 0.000005 -1.651042 -0.000004
Co 0.000000 -0.181323 -0.000010
C 1.700415 -0.597530 0.000001
C -0.000006 2.856193 0.000017
O 2.752878 -1.067747 0.000013
O -0.000005 1.708002 -0.000008
C -1.700412 -0.597536 0.000001
O -2.752873 -1.067756 0.000013
FREQS=   92   95  107  246  263  291  349  420  422  507  542  557  789 2036 2073 2095 2158
TS10_1
8
E= -1723.17829198 ZPE=    18.16 Gcorr   -0.023709000
H 0.257443 0.686521 1.319646
Co 0.228221 -0.014345 0.047236
C -0.611533 1.472452 -0.053357
C 2.209976 0.255102 0.076247
O -1.188023 2.470479 -0.108878
O 1.988092 -0.885058 -0.189346
C -1.072411 -1.286661 -0.099677
O -1.997017 -1.953493 0.031438
FREQS=   74   87  136  164  291  352  360  404  451  483  540  583  670 1796 2062 2099 2150
TS11_1
8
E= -1723.17886666 ZPE=    18.57 Gcorr   -0.022368000
H 0.425648 -1.752704 -0.083579
Co -0.094323 -0.328280 0.008633
C 1.585086 -0.499217 0.004838
C -2.250534 -0.108765 0.013412
O 2.733220 -0.633823 -0.004802
O -1.872348 -1.217395 -0.020644
C -0.116199 1.422463 0.018896
O -0.009504 2.567391 -0.021101
FREQS=   74  111  133  181  289  342  406  443  480  525  568  622  742 1894 1933 2099 2148
TS12_1
8
E= -1723.17569107 ZPE=    18.45 Gcorr   -0.024785000
```

H -0.000124 -1.441413 -0.953645
Co 0.000003 -0.112574 -0.361660
C -1.608269 -0.613275 0.065725
C 1.608286 -0.613300 0.065667
O -2.548026 -1.143237 0.481001
O 2.548037 -1.143211 0.481020
C 0.000000 2.490271 0.723121
O -0.000019 1.898791 -0.263097
FREQS= 62 70 81 126 288 290 360 360 478 542 544 584 775 2059 2061 2087 2139
TS13_1
8
E= -1723.17538390 ZPE= 18.50 Gcorr -0.024354000
H 0.179690 -1.807507 -0.105771
Co -0.018935 -0.327459 0.148874
C 1.649465 -0.504079 -0.061408
C -2.519991 -0.772491 -0.686591
O 2.780434 -0.685948 -0.204821
O -1.978118 -0.656078 0.323429
C -0.081439 1.429592 0.104950
O -0.046899 2.558372 -0.125548
FREQS= 65 73 103 133 168 315 376 415 511 570 580 603 837 1901 2056 2090 2143
TS14_1
8
E= -1723.17863169 ZPE= 20.32 Gcorr -0.021073000
H 1.647940 -0.998061 1.502191
Co -0.047648 -0.316937 -0.092687
C 1.674241 -0.651915 0.417808
C -1.836144 -0.505659 -0.120634
O 2.652047 -0.866394 -0.256470
O -2.958924 -0.595447 0.116534
C 0.171404 1.354810 0.013423
O 0.254571 2.508334 0.032031
FREQS= 39 71 102 193 288 317 405 450 503 555 604 815 1262 1785 2075 2138 2612
TS001_2
14
E= -1801.87657174 ZPE= 56.30 Gcorr 0.030117000
H -3.072617 0.316925 1.835630
Co 0.054079 0.000810 0.045066
C 0.152921 1.639953 -0.597815
C 1.744213 -0.143684 0.587762
O 0.218414 2.629054 -1.185277
O 2.814745 -0.236521 0.996542
C -0.111465 -1.618020 -0.630496
O -0.206181 -2.592837 -1.237759
C -1.915660 0.161571 -0.002179
C -2.041750 0.101919 1.522671
H -2.303460 1.100212 -0.401025
H -2.459306 -0.653267 -0.482178
H -1.418197 0.850312 2.033356
H -1.791943 -0.884066 1.929721
FREQS= 51 77 87 97 106 152 194 314 402 405 469 498 504 533 548 578 581 792 944 958 984
1214 1245 1411 1499 1503 1510 2078 2097 2161 3007 3051 3089 3097 3146
TS002_2
14
E= -1801.87197501 ZPE= 56.35 Gcorr 0.030495000
H 1.733703 0.882105 1.540933
Co -0.239284 0.000002 0.237103
C -2.010045 -0.000367 0.099511
C 0.019399 1.677360 -0.250550
O -3.158617 -0.000591 0.151521
O 0.248934 2.693205 -0.740305
C 0.020084 -1.677282 -0.250499
O 0.249990 -2.693042 -0.740255
C 2.806029 0.000138 -0.112379
C 1.652518 0.000453 0.892183
H 2.783674 -0.881600 -0.761352
H 3.775698 0.000410 0.401818
H 2.783536 0.881383 -0.762015
H 1.733692 -0.880757 1.541549
FREQS= 41 74 92 95 121 218 253 301 380 406 454 482 490 528 530 577 589 746 951 981 1030
1231 1259 1430 1475 1515 1515 2081 2099 2165 3015 3029 3060 3090 3111
TS003_2
14
E= -1801.87134769 ZPE= 56.17 Gcorr 0.030554000
H -3.169225 -0.132097 0.057964
Co 0.164854 0.019141 0.114515
C 1.577406 -1.014660 0.446992

C -0.832852 -1.224874 -0.637770
O 2.442925 -1.668978 0.826953
O -1.399831 -1.956071 -1.323412
C 1.071533 1.468402 -0.343558
O 1.621287 2.347079 -0.843801
C -1.434579 1.173657 0.360666
C -2.699486 0.462672 0.847707
H -1.077133 1.858249 1.146282
H -1.666398 1.805613 -0.503239
H -3.444159 1.192629 1.193560
H -2.501315 -0.208626 1.691392
FREQS= 49 78 94 99 119 225 278 313 367 400 453 481 491 504 541 584 587 637 954 981 1033
1231 1275 1420 1455 1507 1513 2080 2097 2162 2992 3021 3073 3085 3113
TS004_2
14
E= -1801.87000891 ZPE= 56.27 Gcorr 0.029220000
H 2.212035 -1.117174 -1.491689
Co -0.203234 0.023993 0.155141
C 0.369307 1.702578 0.124352
C -0.387614 -1.735056 0.090003
O 0.780937 2.771991 0.063041
O -0.463746 -2.877974 0.015661
C -1.887775 0.398616 -0.161683
O -2.996327 0.643095 -0.355778
C 2.580026 -0.398624 -0.751449
C 1.744189 -0.412967 0.535490
H 2.586676 0.584588 -1.233619
H 3.624686 -0.662158 -0.540702
H 1.841866 -1.388890 1.021159
H 2.146329 0.311639 1.252382
FREQS= 34 44 92 99 142 184 230 248 360 410 428 461 474 521 535 588 607 748 966 985 1020
1236 1265 1428 1494 1512 1515 2097 2104 2182 3025 3047 3083 3087 3109
TS005_2
14
E= -1801.87211466 ZPE= 55.91 Gcorr 0.031377000
H 0.000506 1.101203 -1.434489
Co 0.000048 0.088213 -0.120745
C 1.519281 -0.687468 -0.692548
C -0.000503 -0.441232 1.530695
O 2.460538 -1.271852 -1.011289
O -0.000895 -0.779785 2.633369
C -1.519477 -0.686481 -0.693166
O -2.460988 -1.270267 -1.012253
C 0.001063 2.129280 -0.811115
C 0.000499 1.931557 0.647204
H 0.903281 2.594759 -1.209538
H -0.900350 2.595859 -1.210083
H -0.906310 2.214213 1.174290
H 0.907164 2.213508 1.174909
FREQS= 58 88 91 116 120 265 304 404 407 471 478 488 506 522 535 555 568 789 890 963 1096
1125 1170 1346 1480 1523 1753 2054 2080 2088 2144 3100 3143 3171 3222
TS006_2
14
E= -1801.86877001 ZPE= 55.84 Gcorr 0.029284000
H -2.992605 -0.443004 -0.322259
Co 0.191105 0.014894 0.175009
C -0.617429 -1.395763 -0.510999
C 0.763174 1.575544 -0.416802
O -1.119610 -2.225884 -1.131899
O 1.082241 2.542129 -0.955197
C 1.793233 -0.737602 0.384738
O 2.811518 -1.214877 0.622944
C -2.919363 0.321841 0.455114
C -1.495257 0.851872 0.669051
H -3.597024 1.130320 0.165186
H -3.322831 -0.123117 1.372030
H -1.095679 0.587233 1.683388
H -1.491049 1.940146 0.603006
FREQS= 44 77 87 95 122 145 293 307 321 405 442 492 506 515 527 576 584 654 944 1039 1049
1146 1245 1425 1456 1504 1521 2081 2099 2162 2796 3047 3104 3117 3132
TS007_2
14
E= -1801.86853580 ZPE= 54.52 Gcorr 0.029077000
H 0.567364 1.235473 2.220832
Co -0.002396 -0.000004 -0.130899
C -1.726161 -0.000294 -0.601259
C 0.985100 1.460277 -0.361314

O -2.803140 -0.000469 -1.009116
O 1.621263 2.390822 -0.597502
C 0.985722 -1.459844 -0.361416
O 1.622280 -2.390104 -0.597666
C -0.304774 0.691926 1.874575
C -0.304557 -0.692262 1.874488
H 0.567753 -1.235576 2.220677
H -1.234662 1.249131 1.884517
H -1.234268 -1.249763 1.884367
H 0.063291 0.000038 -1.592287
FREQS= 68 70 98 122 124 304 332 353 396 448 450 469 471 520 531 537 682 711 743 844 984 995 1015 1238 1292 1491 1589 2071 2090 2093 2153 3168 3174 3246 3268
TS008_2
14
E= -1801.86564971 ZPE= 54.37 Gcorr 0.028537000
H -1.513736 -1.165577 1.878934
Co 0.013090 -0.125132 -0.125647
C -1.549083 0.001222 -0.937943
C -0.035325 1.420732 0.751609
O -2.520237 0.057790 -1.556234
O -0.072843 2.443160 1.277201
C 1.648739 0.051399 -0.761086
O 2.686084 0.132565 -1.257294
C 0.244530 -1.996997 0.975682
C -0.434400 -1.116829 1.783855
H -0.289700 -2.765211 0.428570
H 1.308175 -2.164411 1.105112
H 0.066045 -1.179929 -1.166904
H 0.084972 -0.571595 2.564683
FREQS= 56 84 93 111 121 289 319 377 387 395 439 477 485 514 556 569 634 699 731 838 972 990 1023 1239 1313 1488 1604 2022 2087 2109 2154 3166 3175 3245 3269
TS009_2
14
E= -1801.86393206 ZPE= 54.20 Gcorr 0.026943000
H 0.000044 -1.479406 0.717906
Co -0.000011 -0.138134 0.062456
C -1.555993 0.026428 0.906197
C 1.555825 0.026601 0.906431
O -2.519804 0.134284 1.529978
O 2.519529 0.134568 1.530358
C -0.000044 1.418733 -0.771252
O -0.000055 2.434327 -1.312687
C 0.000340 -1.029461 -1.850412
C 0.000167 -2.048941 -0.893175
H -0.918398 -0.781177 -2.370607
H 0.919265 -0.781187 -2.370283
H 0.914416 -2.607116 -0.715475
H -0.914157 -2.607085 -0.715761
FREQS= 59 68 93 93 114 121 308 330 361 427 474 476 495 510 563 569 591 689 813 859 964 1034 1132 1253 1269 1487 1568 2047 2083 2109 2152 3152 3165 3232 3259
TS010_2
14
E= -1801.84322094 ZPE= 53.17 Gcorr 0.023129000
H -0.842473 0.266708 -1.583744
Co -0.258685 0.084433 -0.213991
C -0.276287 1.834150 -0.361092
C -1.510204 -1.143316 -0.244441
O -0.436736 2.969361 -0.477506
O -2.423669 -1.844021 -0.295052
C 0.218632 -0.042273 1.499310
O 0.457089 -0.094236 2.621781
C 1.703954 -1.102967 -1.086635
C 2.844571 -0.734422 -0.495719
H 1.070221 -0.362341 -1.597544
H 1.435295 -2.145782 -1.214556
H 3.535053 -1.464294 -0.081965
H 3.128935 0.310164 -0.406744
FREQS= 48 65 76 81 98 105 202 299 326 344 381 460 487 493 512 562 579 698 755 835 982 1005 1112 1230 1363 1464 1681 1960 2089 2111 2166 2997 3158 3220 3249
TS011_2
14
E= -1801.83598418 ZPE= 52.15 Gcorr 0.016751000
H 0.308070 -0.000893 -1.596692
Co -0.244452 -0.000010 -0.200189
C -0.524932 -1.657937 -0.709843
C -1.191250 0.001290 1.296658
O -0.796755 -2.676308 -1.170327

O -1.910030 0.002192 2.193372
C -0.521630 1.658212 -0.710703
O -0.791358 2.676893 -1.171727
C 2.543990 -0.000992 1.054908
C 3.422778 -0.002825 0.057680
H 2.841070 -0.000044 2.099799
H 1.458116 -0.000302 0.892393
H 3.112297 -0.003751 -0.982557
H 4.492061 -0.003456 0.249422
FREQS= 25 34 38 68 79 97 106 173 275 314 342 444 483 488 507 573 581 595 761 823 907 979 1038 1216 1377 1473 1711 1953 2098 2114 2178 3018 3160 3203 3249
TS012_2
14
E= -1801.84145600 ZPE= 53.14 Gcorr 0.023438000
H -0.455624 0.473684 -1.419677
Co 0.121996 0.005888 -0.126976
C -0.575978 -1.455143 -0.813488
C 1.245683 1.249304 -0.645174
O -0.954986 -2.378766 -1.388303
O 1.981180 2.003374 -1.111960
C 1.134341 -0.749438 1.126635
O 1.875338 -1.288091 1.822637
C -2.924068 0.926409 0.659526
C -1.680685 1.371795 0.854094
H -3.202892 -0.101677 0.871104
H -3.707093 1.574950 0.276828
H -1.406532 2.405916 0.679198
H -0.929780 0.738463 1.352356
FREQS= 57 67 75 92 99 106 200 307 336 352 369 456 487 488 515 565 567 659 744 833 978 1000 1109 1229 1363 1461 1684 1995 2090 2106 2164 2990 3160 3223 3251
TS013_2
14
E= -1801.83711270 ZPE= 52.56 Gcorr 0.019455000
H 0.173241 0.000008 -1.703177
Co 0.284057 -0.000004 -0.207771
C 0.736610 -1.647299 -0.620548
C 0.736715 1.647335 -0.620320
O 1.143634 -2.660495 -0.983291
O 1.143816 2.660560 -0.982893
C 0.776460 -0.000114 1.497617
O 1.215747 -0.000195 2.559618
C -2.505201 0.000343 -0.263274
C -3.665876 -0.000132 0.384712
H -2.435018 0.001514 -1.343684
H -1.572939 -0.000509 0.322802
H -3.722101 -0.001319 1.469285
H -4.610537 0.000647 -0.151988
FREQS= 42 44 51 75 94 98 124 179 321 334 341 452 485 485 509 570 575 629 747 877 885 976 1041 1262 1406 1483 1702 1963 2098 2114 2175 2980 3158 3233 3253
TS014_2
14
E= -1801.83765016 ZPE= 52.75 Gcorr 0.020445000
H -0.153871 -0.000282 -1.648679
Co 0.267390 0.000026 -0.208910
C 0.648059 -1.648515 -0.684997
C 0.647110 1.648801 -0.685034
O 0.991291 -2.663916 -1.102905
O 0.989799 2.664392 -1.102916
C 1.010914 0.000273 1.403574
O 1.581734 0.000395 2.400690
C -3.720824 -0.000447 0.149206
C -2.452827 -0.000885 0.548531
H -3.992008 -0.000422 -0.902401
H -4.538932 -0.000138 0.864226
H -2.172549 -0.000919 1.595203
H -1.659354 -0.001274 -0.214420
FREQS= 38 41 76 93 97 118 123 163 321 338 341 458 486 488 507 572 579 656 763 888 903 978 1044 1265 1408 1483 1702 1968 2098 2116 2177 2978 3158 3225 3249
TS015_2
14
E= -1801.84096936 ZPE= 53.24 Gcorr 0.023968000
H -0.628667 -0.248645 -1.396711
Co 0.120204 -0.024217 -0.127715
C 0.646690 -1.621030 -0.628827
C 0.116019 1.570327 -0.870486
O 1.016122 -2.610050 -1.090479
O 0.157292 2.554612 -1.466526

C 1.320341 0.277898 1.152335
O 2.176150 0.483391 1.891635
C -3.166155 -0.514702 0.498469
C -2.068735 0.040796 1.011113
H -4.130409 -0.016877 0.548418
H -3.140074 -1.480507 0.002726
H -1.125684 -0.534429 1.109450
H -2.106153 0.990969 1.531765
FREQS= 53 68 85 92 98 129 202 332 352 361 371 457 487 488 513 563 569 661 745 834 984
1015 1151 1246 1362 1455 1688 1994 2093 2111 2167 2888 3161 3219 3251
TS016_2
14
E= -1801.84096543 ZPE= 53.25 Gcorr 0.024012000
H -0.388163 0.542733 -1.415595
Co 0.150758 0.016357 -0.129454
C -0.583316 -1.401009 -0.869274
C 1.334212 1.232860 -0.579447
O -0.990107 -2.294316 -1.471386
O 2.109819 1.969576 -1.006741
C 1.096601 -0.802016 1.136825
O 1.781290 -1.376782 1.860289
C -3.002959 0.934160 0.710714
C -1.742333 1.366405 0.732871
H -3.819854 1.584294 0.410819
H -3.264845 -0.083723 0.984335
H -0.933943 0.753980 1.177812
H -1.484913 2.390847 0.490449
FREQS= 55 62 88 95 102 117 209 335 347 363 379 456 486 487 513 565 568 661 745 827 979
1011 1137 1239 1361 1456 1688 2003 2093 2110 2166 2914 3160 3223 3250
TS017_2
14
E= -1801.84594971 ZPE= 55.15 Gcorr 0.029269000
H 0.474072 0.058457 -2.203433
Co 0.145399 -0.185442 0.128091
C 1.183646 1.217996 0.477899
C -0.319397 0.447683 -1.513255
O 1.826401 2.116210 0.802584
O -1.242649 1.097517 -1.903939
C -1.498487 0.006371 0.798340
O -2.497102 0.110956 1.358763
C 0.252845 -2.203538 -0.072992
C 1.543315 -1.654400 -0.154938
H -0.070419 -2.687200 0.847079
H -0.260915 -2.559980 -0.961705
H 2.053865 -1.572989 -1.110570
H 2.212889 -1.713485 0.700597
FREQS= 65 70 90 108 122 160 274 375 396 411 455 474 498 507 512 589 661 811 825 935 957
973 1219 1238 1252 1477 1555 1845 2091 2139 2798 3131 3140 3202 3225
TS018_2
14
E= -1801.84736808 ZPE= 54.34 Gcorr 0.030702000
H -0.000600 0.255990 -1.490734
Co -0.000130 -0.021794 -0.008202
C 1.767924 -0.035284 -0.173900
C -1.768214 -0.037003 -0.173207
O 2.905405 -0.057339 -0.324827
O -2.905657 -0.059846 -0.324362
C -0.000761 1.739461 0.321783
O -0.001235 2.887056 0.229540
C 0.000291 -2.031585 -0.384753
C 0.002408 -1.829105 1.021660
H 0.907578 -2.345785 -0.891180
H -0.908450 -2.346875 -0.887895
H -0.907808 -1.986744 1.591108
H 0.914791 -1.986012 1.587855
FREQS= 99 101 123 132 166 338 355 377 426 441 461 514 520 547 558 603 611 635 753 835 947
956 1031 1208 1230 1488 1550 1838 2099 2117 2168 3152 3160 3224 3247
TS019_2
14
E= -1801.84319452 ZPE= 55.29 Gcorr 0.029337000
H 0.484834 -0.189633 2.288579
Co 0.128715 -0.038239 -0.199242
C -1.093098 1.233653 -0.524097
C -0.353185 -0.259212 1.559521
O -1.834465 2.073785 -0.781558
O -1.468175 -0.529882 1.906236
C 1.756102 0.620557 0.161868

O 2.810785 1.082785 0.203118
C 0.534834 -1.956582 -0.679280
C -0.757773 -1.612370 -1.115884
H 1.353571 -2.009962 -1.395264
H 0.668666 -2.591761 0.192612
H -1.634374 -1.951096 -0.573232
H -0.934453 -1.394867 -2.168286
FREQS= 62 81 87 105 122 130 267 375 391 401 448 479 489 510 517 625 677 811 839 932 962 976 1237 1244 1279 1473 1551 1817 2082 2135 2874 3124 3136 3202 3231
TS020_2
14
E= -1801.84209607 ZPE= 54.28 Gcorr 0.030198000
H 0.000000 0.124264 1.654466
Co 0.000000 -0.010767 0.151277
C -1.756528 -0.021465 -0.149764
C 1.756528 -0.021465 -0.149764
O -2.896729 -0.053613 -0.297762
O 2.896729 -0.053613 -0.297762
C 0.000000 1.748215 0.015174
O 0.000000 2.898388 0.032311
C 0.000000 -1.991210 0.737712
C 0.000000 -1.946429 -0.673023
H -0.911230 -2.237064 1.271495
H 0.911230 -2.237064 1.271495
H 0.909083 -2.147293 -1.229116
H -0.909083 -2.147293 -1.229115
FREQS= 101 102 112 136 158 241 315 381 419 449 483 508 509 528 572 592 620 625 776 842 953 979 1003 1229 1234 1492 1558 1858 2096 2106 2158 3166 3172 3239 3261
TS021_2
14
E= -1801.83797884 ZPE= 55.78 Gcorr 0.031920000
H 0.000056 1.419686 -1.113747
Co -0.000012 0.044195 -0.079235
C -1.770368 -0.052884 -0.273651
C 1.770346 -0.053009 -0.273507
O -2.914685 -0.106837 -0.382613
O 2.914657 -0.107039 -0.382519
C -0.000051 -1.672435 0.176703
O -0.000057 -2.802313 0.422242
C 0.000389 2.215673 -0.215204
C -0.000254 1.624080 1.161206
H -0.897889 2.781929 -0.468593
H 0.899410 2.780933 -0.468135
H 0.901861 1.792472 1.740200
H -0.902813 1.792664 1.739460
FREQS= 83 98 104 127 146 153 362 373 442 450 467 480 517 536 556 609 632 762 879 920 1047 1099 1186 1389 1464 1508 1665 2019 2075 2092 2152 3084 3155 3157 3231
TS022_2
14
E= -1801.83463216 ZPE= 55.11 Gcorr 0.029759000
H 1.854426 -1.850678 -0.458853
Co 0.033349 0.064789 -0.399348
C -0.655446 -1.293717 0.734568
C 1.436054 0.918235 0.264900
O -0.351472 -1.641980 1.821905
O 2.348017 1.376251 0.792508
C -1.179862 1.340548 -0.076185
O -1.953445 2.186457 -0.004215
C 1.353528 -1.199358 -1.183565
C -1.371052 -1.349480 -0.487453
H 2.141780 -0.649113 -1.711223
H 0.841420 -1.835794 -1.911714
H -1.166632 -2.223736 -1.100726
H -2.415542 -1.053187 -0.430268
FREQS= 62 75 90 104 127 280 291 369 406 435 447 467 477 528 544 564 593 760 764 827 878 983 1003 1273 1430 1468 1473 1992 2104 2153 3027 3101 3119 3126 3214
TS023_2
14
E= -1801.81807098 ZPE= 48.98 Gcorr 0.013606000
H 0.598218 -0.640379 2.444628
Co 0.179550 0.004351 0.229363
C 1.943227 0.014375 -0.003099
C -0.103094 -1.670657 -0.259264
O 3.079551 0.022322 -0.170649
O -0.339376 -2.702687 -0.707634
C -0.111038 1.691592 -0.215719
O -0.350963 2.733259 -0.638856

C -2.810949 -0.006430 -0.158414
C -1.723370 0.001385 0.612416
H -2.755146 -0.014567 -1.244534
H -3.814380 -0.004570 0.265210
H -1.863181 0.012899 1.700452
H 0.704288 -0.075611 2.923037
FREQS= 44 62 80 91 99 109 145 200 203 246 281 311 385 395 456 471 483 505 523 537 587
616 935 986 1015 1264 1434 1663 2096 2111 2172 3064 3130 3206 4356
TS024_2
14
E= -1801.83458814 ZPE= 55.72 Gcorr 0.030244000
H 0.000127 2.686306 -0.753429
Co -0.000003 0.076479 0.113959
C 0.000123 1.899789 0.025051
C -1.583085 -0.064769 -0.656587
O 0.000201 2.149042 1.217509
O -2.508428 -0.242345 -1.327481
C 1.583085 -0.064981 -0.656559
O 2.508418 -0.242678 -1.327435
C -0.000222 -1.406168 1.624296
C -0.000065 -2.121570 0.455976
H -0.922656 -1.240693 2.174053
H 0.922082 -1.240652 2.174262
H -0.918586 -2.517932 0.037074
H 0.918568 -2.517936 0.037324
FREQS= 51 95 98 111 127 183 238 304 313 411 445 480 514 514 526 585 665 846 889 968 1013
1017 1234 1285 1322 1484 1614 1722 2048 2102 2955 3148 3172 3231 3263
TS025_2
14
E= -1801.83044153 ZPE= 55.07 Gcorr 0.027655000
H -2.097912 -1.301576 -1.231647
Co 0.016021 -0.036462 0.011114
C -0.169672 -1.135734 1.696730
C 0.443698 1.605507 0.287649
O -0.603422 -1.892237 0.908727
O 0.705641 2.704452 0.520023
C 1.651307 -0.616086 -0.485189
O 2.673355 -0.896247 -0.928470
C -1.833328 0.548441 -0.066677
C -2.053184 -0.217594 -1.374565
H -2.043021 1.612241 -0.186361
H -2.449582 0.161862 0.748247
H -2.998640 0.077715 -1.848444
H -1.280939 -0.000725 -2.131808
FREQS= 53 81 90 103 124 155 197 205 259 354 404 425 446 483 551 553 579 777 911 954 979
1204 1240 1403 1492 1495 1500 1891 2091 2143 2985 3047 3090 3108 3147
TS026_2
14
E= -1801.82257217 ZPE= 50.57 Gcorr 0.020157000
H 0.969650 0.212467 3.013977
Co 0.172192 -0.041036 0.298781
C -0.141244 1.699476 0.284555
C 1.782776 -0.150279 -0.431495
O -0.302039 2.838436 0.289340
O 2.742608 -0.264894 -1.056302
C -1.476779 -0.443270 -0.567422
O -2.157651 0.017740 -1.425774
C -0.023610 -2.020486 0.489673
C -1.293898 -1.365006 0.493303
H 0.301568 -2.530927 -0.412701
H 0.297251 -2.507792 1.407145
H 0.411625 0.207894 2.514804
H -1.976102 -1.326551 1.339905
FREQS= 55 85 90 107 139 173 228 251 283 292 350 388 419 463 478 507 512 574 603 635 764
853 916 1017 1066 1206 1351 1521 1961 2094 2146 3138 3168 3215 4325
TS027_2
14
E= -1801.81543577 ZPE= 49.19 Gcorr 0.013370000
H -0.232034 0.577161 2.328335
Co 0.113645 0.007318 0.125213
C 0.733807 1.603079 -0.346839
C 1.710383 -0.750756 0.334033
O 1.080212 2.594758 -0.813402
O 2.736137 -1.256887 0.440390
C -0.703608 -1.428696 -0.484648
O -1.220594 -2.305103 -1.021545
C -2.678815 0.543649 0.781951

C -1.678010 0.770508 -0.075894
H -2.611112 -0.170684 1.600880
H -3.623734 1.081558 0.708675
H 0.191214 0.146859 2.770458
H -1.861340 1.478661 -0.884268
FREQS= 28 54 91 96 106 111 132 155 233 249 294 313 389 402 470 476 487 502 533 549 590
611 940 1009 1049 1294 1434 1643 2092 2109 2171 3115 3135 3194 4350
TS028_2
14
E= -1801.82638832 ZPE= 53.01 Gcorr 0.024353000
H 3.424597 1.117512 -0.379492
Co -0.140678 0.070431 -0.190016
C -1.520969 -1.017438 -0.552680
C 0.679007 -1.024349 0.984732
O -2.382879 -1.696747 -0.892695
O 1.223096 -1.710468 1.730549
C -0.973543 1.475197 0.537536
O -1.447634 2.422876 0.984241
C 1.375473 0.773957 -0.859625
C 2.793223 0.311223 -0.783155
H -0.339990 0.493627 -1.596028
H 1.338905 1.710721 -1.425688
H 3.163924 0.137196 -1.804979
H 2.951049 -0.597521 -0.200991
FREQS= 52 74 84 94 118 155 204 314 325 414 445 448 477 485 527 547 569 637 673 884 1000
1047 1128 1371 1384 1484 1492 1990 2104 2111 2157 3003 3048 3085 3151
TS029_2
14
E= -1801.82676504 ZPE= 53.59 Gcorr 0.025151000
H 2.998845 -0.872750 -0.235726
Co -0.179992 -0.000018 -0.236246
C -1.232294 1.434286 -0.034153
C -1.232154 -1.434390 -0.033919
O -1.878423 2.384482 -0.031022
O -1.878187 -2.384650 -0.030585
C 0.742113 0.000193 1.308480
O 1.358160 0.000354 2.276756
C 1.316163 -0.000142 -1.253492
C 2.768612 -0.000039 -0.861217
H 1.222096 -0.000350 -2.342741
H -0.803670 -0.000060 -1.569720
H 2.998789 0.872835 -0.235936
H 3.456683 -0.000126 -1.712610
FREQS= 36 74 81 94 115 134 306 312 345 434 443 464 468 521 535 557 634 653 760 869 1028
1055 1104 1352 1387 1489 1489 2041 2109 2128 2163 3022 3075 3093 3118
TS030_2
14
E= -1801.82728819 ZPE= 52.84 Gcorr 0.026752000
H -0.123797 0.885313 -2.075150
Co 0.076616 0.153061 -0.484283
C -1.691731 0.309455 -0.381511
C 0.010788 -1.240565 0.804898
O -2.829168 0.448371 -0.281055
O -0.500648 -1.510110 1.846009
C 0.745571 1.574417 0.367883
O 1.155289 2.404804 1.050931
C 0.733946 -1.738435 -0.328111
C 1.834706 -0.906693 -0.657142
H 0.342882 -2.540704 -0.947113
H 0.486776 0.402622 -2.160562
H 2.309672 -1.039215 -1.626475
H 2.512392 -0.574250 0.121772
FREQS= 55 80 102 109 222 280 318 358 395 425 439 479 494 507 538 581 618 645 752 800 872
920 1018 1056 1219 1260 1360 1534 1943 2098 2144 3144 3187 3226 3782
TS031_2
14
E= -1801.82145040 ZPE= 51.17 Gcorr 0.021210000
H -0.773467 -0.000072 2.090675
Co -0.194928 0.000004 0.395198
C 0.101508 1.663104 -0.142410
C 0.101535 -1.663092 -0.142419
O 0.330002 2.708876 -0.564616
O 0.330058 -2.708862 -0.564612
C -1.920744 -0.000017 -0.056201
O -3.005577 -0.000035 -0.434315
C 1.792259 0.000011 0.497883
C 2.674530 0.000010 -0.502179

H 2.181720 0.000015 1.518222
H 0.001561 0.000016 2.161884
H 2.375460 0.000010 -1.547746
H 3.747387 0.000007 -0.313081
FREQS= 49 74 94 104 119 142 265 294 366 370 415 449 473 479 486 496 527 540 579 608 730
944 1009 1028 1101 1288 1430 1657 2096 2110 2164 3105 3132 3208 3864
TS032_2
14
E= -1801.82107542 ZPE= 51.30 Gcorr 0.020838000
H -0.572770 0.000412 1.859325
Co 0.130649 -0.000005 0.246192
C 1.915290 -0.001290 0.269279
C -0.015964 -1.671862 -0.328450
O 3.062135 -0.002098 0.204140
O -0.157007 -2.719354 -0.782559
C -0.013498 1.672167 -0.328269
O -0.153109 2.719908 -0.782236
C -2.841888 0.001020 0.592514
C -1.800389 0.001244 -0.242860
H -2.745422 0.000009 1.677297
H -3.865029 0.001842 0.217350
H 0.191195 0.000328 2.022339
H -2.012954 0.002214 -1.311537
FREQS= 40 47 97 102 110 182 270 302 367 387 418 454 469 485 487 519 520 532 580 604 756
933 1018 1048 1136 1285 1437 1650 2096 2111 2166 3116 3144 3191 3822
TS033_2
14
E= -1801.82979963 ZPE= 55.70 Gcorr 0.030327000
H 2.545239 -1.171957 0.427812
Co 0.055982 0.017032 0.050908
C -0.477769 -1.594673 -0.334878
C 1.677420 -0.535739 0.685273
O -0.834885 -2.631453 -0.703061
O 1.594729 0.078069 1.739015
C -1.531196 0.719458 0.497573
O -2.505450 1.153708 0.930105
C 0.107413 1.206948 -1.683937
C 1.349652 1.281026 -1.075966
H -0.051293 0.563669 -2.544427
H -0.596181 2.026622 -1.586463
H 1.610427 2.126454 -0.443958
H 2.192009 0.730632 -1.484343
FREQS= 53 85 88 119 155 156 222 329 371 419 427 452 514 528 562 659 685 834 881 962 983
1007 1235 1265 1289 1483 1587 1693 2071 2122 2959 3143 3158 3221 3248
TS034_2
14
E= -1801.83193413 ZPE= 56.67 Gcorr 0.033012000
H -0.290693 -1.696488 0.899629
Co 0.263918 -0.129718 -0.012572
C -1.601778 0.393135 -0.081827
C 1.917524 -0.790469 -0.134193
O -2.237951 1.302568 -0.559762
O 3.023313 -1.100450 -0.192534
C 0.721521 1.506602 0.162252
O 1.002443 2.612059 0.331285
C -2.244148 -0.952482 0.332192
C -1.061648 -1.907439 0.116391
H -2.517726 -0.909695 1.393262
H -3.143153 -1.183526 -0.249043
H -0.623560 -1.735872 -0.892682
H -1.241918 -2.981526 0.187486
FREQS= 52 85 100 120 216 253 297 340 358 384 438 474 497 564 602 618 793 875 976 1023 1107
1237 1283 1384 1471 1503 1573 1798 2091 2146 2797 2883 3052 3111 3135
TS035_2
14
E= -1801.81595185 ZPE= 52.95 Gcorr 0.017343000
H -3.558204 -0.050522 -0.115000
Co 0.012647 -0.081302 -0.160196
C -0.196051 1.635713 0.197642
C -0.249360 -0.704151 1.471771
O -0.231505 2.770646 0.368443
O -0.322445 -1.174277 2.516800
C 1.774911 -0.423494 -0.720497
O 2.850587 -0.203052 -0.273347
C 0.751748 -0.866208 -1.574364
C -3.241733 -0.544672 -1.036232
H -2.148138 -0.596036 -1.085755

H 1.203983 -1.346481 -2.439650
H -3.642227 -1.559938 -1.062258
H -3.607071 0.018472 -1.897140
FREQS= 36 37 60 65 82 88 92 97 151 281 328 356 363 460 477 522 579 580 684 722 850 1057 1342 1347 1363 1567 1569 1956 2111 2160 3020 3118 3161 3168 3193
TS036_2
14
E= -1801.82556552 ZPE= 54.34 Gcorr 0.029457000
H 1.307039 -0.628548 -1.435908
Co -0.346119 -0.031051 0.042205
C 1.971804 -0.512778 -0.508403
C -0.060895 1.731853 0.114950
O 3.050204 0.035801 -0.621518
O 0.022961 2.859945 0.306971
C -2.049172 -0.332012 -0.365039
O -3.178274 -0.518758 -0.463123
C 0.193101 -1.886437 0.500973
C 1.302175 -1.016323 0.679630
H 0.130619 -2.503175 -0.394911
H -0.241514 -2.352867 1.379137
H 1.731185 -0.797218 1.651949
H -0.883319 0.198462 1.348894
FREQS= 66 78 85 106 188 326 370 391 419 428 445 481 487 518 605 660 710 812 904 944 975 1020 1159 1225 1350 1431 1526 1793 2114 2122 2167 2541 3129 3207 3226
TS037_2
14
E= -1801.82429195 ZPE= 55.10 Gcorr 0.028232000
H -2.487238 -0.886316 -1.303166
Co 0.099988 -0.018303 -0.071570
C -0.506215 -1.098267 1.489596
C 0.769998 1.534902 0.249528
O -0.860194 -1.850530 0.661791
O 1.190586 2.567595 0.537726
C 1.680993 -0.851088 -0.357571
O 2.660136 -1.298178 -0.757943
C -1.576392 0.931955 -0.451329
C -2.740294 -0.042795 -0.649970
H -1.352290 1.442743 -1.401899
H -1.866951 1.719627 0.250756
H -3.100953 -0.457672 0.295063
H -3.584994 0.476447 -1.122483
FREQS= 57 88 91 110 148 156 209 246 279 351 390 412 440 464 518 553 581 661 952 977 1016 1236 1273 1420 1459 1504 1510 1902 2095 2144 2991 3023 3074 3091 3123
TS038_2
14
E= -1801.81516576 ZPE= 50.34 Gcorr 0.019315000
H 0.029318 -0.199364 2.725791
Co 0.166352 -0.034658 0.090264
C -0.017580 1.686237 -0.155352
C 1.943681 -0.170678 0.120418
O -0.115221 2.802202 -0.429348
O 3.086452 -0.284155 0.054700
C -1.738208 -0.376222 0.000604
O -2.750781 0.047767 0.462262
C -0.017289 -2.029366 -0.183986
C -1.087269 -1.345568 -0.808034
H 0.668419 -2.585144 -0.815025
H -0.160582 -2.457037 0.807843
H -0.130254 0.304555 2.191290
H -1.162028 -1.240167 -1.889843
FREQS= 40 90 94 105 152 185 187 226 264 327 343 388 413 428 473 497 524 582 598 630 773 844 914 999 1051 1223 1372 1523 1959 2090 2139 3127 3154 3227 4270
TS039_2
14
E= -1801.81491365 ZPE= 50.41 Gcorr 0.019547000
H -0.128886 -0.154365 2.712722
Co 0.166785 -0.031422 0.086476
C -1.744203 -0.365658 0.001363
C 1.940964 -0.163558 0.116195
O -2.749988 0.059031 0.475951
O 3.084331 -0.273726 0.048906
C -0.017566 1.696539 -0.130901
O -0.115536 2.816195 -0.387936
C -0.025175 -2.018505 -0.215981
C -1.097608 -1.320816 -0.824212
H 0.232490 -0.542900 2.182807
H 0.654054 -2.563989 -0.862931

H -1.177208 -1.195116 -1.903573
H -0.172588 -2.475232 0.761974
FREQS= 44 89 95 99 170 179 203 220 263 302 343 391 411 426 475 491 526 584 602 628 776 853 914 1006 1054 1221 1371 1524 1963 2089 2138 3127 3154 3225 4307
TS040_2
14
E= -1801.82836880 ZPE= 56.40 Gcorr 0.033239000
H 2.188236 -0.111330 -1.357005
Co -0.106990 -0.083816 -0.056364
C 1.858573 -0.110379 -0.285837
C -1.820278 0.247003 0.100705
O 2.673853 -0.073729 0.602059
O -2.942146 0.492964 0.195657
C 0.257591 1.606884 -0.088786
O 0.445615 2.744459 -0.105979
C -0.847345 -2.012645 0.003801
C 0.539420 -2.055000 -0.131236
H -1.488019 -2.202050 -0.853262
H -1.300682 -2.215929 0.970303
H 1.160217 -2.277438 0.731089
H 0.982634 -2.294947 -1.095074
FREQS= 70 99 109 120 175 267 305 337 361 439 455 489 540 554 582 614 716 851 905 997 1006 1024 1247 1270 1357 1481 1566 1780 2087 2134 2775 3138 3145 3219 3241
TS041_2
14
E= -1801.82204880 ZPE= 53.20 Gcorr 0.027364000
H -0.332875 0.052274 -1.972809
Co -0.138970 -0.002759 -0.331588
C 1.707756 -0.602975 -0.120077
C -1.891634 -0.442355 -0.332942
O 2.798829 -0.407671 -0.561633
O -2.997437 -0.741138 -0.226986
C -0.005165 1.731531 0.084439
O 0.060511 2.811091 0.477822
C 0.870572 -1.479142 0.624586
C 0.036459 -0.769403 1.535606
H 0.459385 -0.060115 -1.863379
H 0.751251 -2.525568 0.359087
H -0.797752 -1.302803 1.983122
H 0.469031 -0.013484 2.183556
FREQS= 58 77 103 107 218 280 301 357 407 437 460 477 479 505 561 577 618 644 799 873 925 995 1039 1068 1210 1357 1535 1582 1941 2095 2137 3146 3186 3226 3437
TS042_2
14
E= -1801.81632166 ZPE= 51.20 Gcorr 0.021669000
H -0.467337 -0.683164 1.642735
Co -0.234211 0.000017 0.359306
C 0.187398 -1.504517 -0.546305
C 0.187191 1.504570 -0.546351
O 0.485877 -2.494530 -1.038260
O 0.485605 2.494552 -1.038411
C -2.019456 -0.000133 0.133063
O -3.161998 -0.000180 0.072571
C 1.652334 0.000302 0.962026
C 2.749136 -0.000114 0.203548
H 1.776348 0.000876 2.042146
H -0.467618 0.683015 1.642779
H 3.741751 0.000108 0.652574
H 2.725072 -0.000670 -0.884592
FREQS= 63 63 90 103 117 126 268 303 380 405 440 467 473 515 524 532 551 555 632 840 856 930 1025 1034 1295 1430 1657 2069 2081 2147 2161 2189 3123 3167 3201
TS043_2
14
E= -1801.81433542 ZPE= 51.02 Gcorr 0.020704000
H -0.153015 -0.511808 -1.619928
Co -0.128960 0.004892 -0.246570
C -1.801515 0.625360 -0.480195
C -0.531809 -1.577013 0.533560
O -2.838755 1.045215 -0.719969
O -0.746155 -2.619224 0.956766
C 0.502845 1.179414 0.972521
O 0.980056 1.932801 1.691223
C 2.718730 -0.043775 -1.154779
C 1.768419 -0.555841 -0.373029
H 2.544372 0.779567 -1.842627
H 3.732470 -0.442763 -1.133199
H 0.207252 0.782078 -1.444779

H 2.049656 -1.378368 0.285303
FREQS= 43 66 84 98 109 137 254 322 371 401 441 453 480 500 512 530 547 560 628 746 866
932 1023 1032 1308 1430 1661 2095 2098 2147 2167 2189 3117 3137 3210
TS044_2
14
E= -1801.81372913 ZPE= 51.35 Gcorr 0.022026000
H 0.645506 0.887949 -1.385491
Co 0.195133 0.044977 -0.263834
C 1.901208 -0.487458 -0.396270
C 0.176525 1.603398 0.653794
O 2.982922 -0.805511 -0.600952
O 0.133433 2.646761 1.123527
C -0.407396 -1.163069 0.930275
O -0.805238 -1.956516 1.652557
C -1.597141 0.305357 -1.081322
C -2.746567 -0.319350 -0.815249
H -0.235254 -0.764330 -1.405298
H -1.619266 1.110788 -1.810788
H -3.673274 0.004300 -1.286424
H -2.835005 -1.164213 -0.136902
FREQS= 57 71 85 97 121 140 276 326 410 417 433 459 481 502 524 525 560 604 672 736 873
923 1034 1049 1299 1434 1653 2045 2107 2145 2155 2189 3133 3172 3213
TS045_2
14
E= -1801.81355540 ZPE= 51.32 Gcorr 0.022046000
H -0.302007 -1.237273 -0.930335
Co 0.106847 -0.046863 -0.171885
C -0.517939 -0.977965 1.246362
C 1.715879 -0.710116 -0.597785
O -1.010515 -1.619896 2.056430
O 2.688369 -1.185240 -0.974660
C 0.611469 1.542879 0.519644
O 0.918877 2.573375 0.911727
C -1.680890 0.626949 -0.705749
C -2.658965 -0.021610 -1.341672
H -1.883815 1.646373 -0.379502
H -0.115124 0.530362 -1.498910
H -2.541902 -1.025005 -1.740932
H -3.633177 0.444104 -1.482192
FREQS= 69 73 86 93 104 145 285 329 404 413 435 464 474 495 510 541 563 605 671 735 856
926 1027 1048 1309 1426 1654 2068 2107 2145 2156 2189 3126 3146 3220
TS046_2
14
E= -1801.81784946 ZPE= 54.07 Gcorr 0.026481000
H 1.996804 -1.298636 1.340363
Co 0.054914 -0.000345 0.115902
C 1.191943 0.004070 -1.295633
C -1.079153 -1.323364 -0.127884
O 1.731257 0.007037 -2.316285
O -1.797005 -2.216657 -0.278353
C -1.078993 1.324036 -0.120689
O -1.796750 2.218223 -0.266169
C 1.066091 -0.802145 1.599174
C 1.064794 0.792479 1.604701
H 0.441190 -1.330399 2.315071
H 1.557165 -0.007685 2.469484
H 1.994863 1.292157 1.349687
H 0.439204 1.314597 2.324496
FREQS= 48 71 83 90 118 127 300 359 428 435 437 461 472 509 532 548 573 623 793 832 861
1063 1230 1264 1394 1485 1486 2066 2068 2129 2193 3135 3137 3231 3242
TS047_2
14
E= -1801.81935573 ZPE= 54.01 Gcorr 0.028211000
H -0.773750 1.412041 1.142296
Co -0.273550 0.250888 0.362485
C -1.942965 0.288275 -0.111636
C 0.723485 -0.928221 -1.002146
O -3.013911 0.450277 -0.511902
O 1.251296 -0.721137 -2.039037
C 1.102602 1.352430 0.251669
O 1.862095 2.208538 0.115214
C 0.791207 -1.722249 1.410123
C 0.193888 -1.682207 0.022044
H 0.422608 -0.884260 2.049786
H 1.881522 -1.668827 1.407599
H 0.477630 -2.638222 1.915106
H -0.627299 -2.344286 -0.236396

FREQS= 63 72 96 117 195 213 264 323 388 416 451 491 506 519 550 594 620 729 822 882 1042 1069 1117 1345 1427 1465 1489 1994 2044 2077 2139 2825 3095 3155 3185
TS048_2
14
E= -1801.80928793 ZPE= 50.43 Gcorr 0.019295000
H 0.180830 -0.087196 -1.846156
Co 0.139625 -0.036633 -0.284841
C 0.662999 -1.590250 0.483664
C 1.725426 0.721476 -0.487172
O 0.963897 -2.544923 1.049649
O 2.750624 1.211322 -0.656619
C -0.509182 1.209409 0.822192
O -0.941346 1.966141 1.574279
C -1.775819 -0.735258 -0.572746
C -2.893985 -0.013786 -0.698299
H -0.781163 -0.299768 -1.519090
H -1.855701 -1.814058 -0.465764
H -3.875368 -0.481248 -0.645787
H -2.880494 1.061495 -0.856794
FREQS= 40 68 78 97 105 140 246 281 305 407 423 444 453 491 504 534 564 583 619 798 967 993 1040 1072 1296 1424 1645 1836 1918 2096 2110 2161 3139 3173 3225
TS049_2
14
E= -1801.81013209 ZPE= 50.51 Gcorr 0.020150000
H 0.000013 -0.837446 -1.837817
Co 0.000003 -0.082708 -0.538674
C -1.729222 0.308792 -0.560176
C 1.729227 0.308800 -0.560166
O -2.836312 0.605334 -0.578757
O 2.836316 0.605345 -0.578740
C 0.000004 -1.620725 0.416850
O 0.000003 -2.629761 0.959091
C -0.000008 1.373692 0.851880
C -0.000018 1.189021 2.172646
H 0.000004 0.433800 -1.923999
H -0.000001 2.393623 0.469722
H -0.000021 2.033959 2.861730
H -0.000025 0.204359 2.635610
FREQS= 56 58 87 91 103 174 271 318 320 386 438 470 489 497 508 516 549 558 625 701 803 944 1007 1049 1271 1427 1651 1975 2059 2133 2148 2186 3116 3150 3198
TS050_2
14
E= -1801.81565658 ZPE= 53.81 Gcorr 0.026667000
H 0.384756 0.442510 -1.328407
Co 0.228948 0.037221 0.038666
C 1.704925 -1.006393 -0.179223
C -1.593306 0.177112 -0.492023
O 2.596106 -1.590459 -0.609458
O -2.374241 0.970898 -0.924504
C 0.727378 1.706676 0.414257
O 1.016565 2.812662 0.531538
C -1.284259 -1.181079 -0.223029
C -1.367643 -1.647650 1.228079
H -2.051206 -2.499939 1.303146
H -1.379086 -1.914910 -1.017840
H -0.402473 -1.992462 1.633103
H -1.763598 -0.876973 1.897040
FREQS= 51 70 82 92 138 217 284 317 398 427 438 449 480 515 564 596 621 656 828 876 1008 1062 1079 1299 1416 1492 1505 1939 2100 2122 2159 2989 3075 3112 3183
TS051_2
14
E= -1801.81529549 ZPE= 54.16 Gcorr 0.026898000
H 2.392490 -1.630221 0.841928
Co -0.280059 -0.155237 -0.222470
C -1.476492 -1.382410 0.186986
C 1.271697 0.840057 0.312542
O -2.163285 -2.196817 0.624346
O 1.518943 1.690020 1.112874
C -1.273535 1.298691 -0.170926
O -1.962525 2.200997 -0.365225
C 2.668528 -1.240533 -0.140505
C 1.707710 -0.146545 -0.592902
H 2.675358 -2.076884 -0.844859
H 3.682930 -0.832989 -0.067701
H 1.888689 0.258159 -1.591932
H 0.389597 -1.295842 -0.877877

FREQS= 40 63 89 94 177 211 241 283 340 393 419 456 516 549 574 625 655 682 869 906 1038
1065 1137 1323 1425 1505 1507 1945 2075 2122 2144 3049 3107 3120 3143
TS052_2
14
E= -1801.81507790 ZPE= 53.34 Gcorr 0.027175000
H -0.248432 0.089504 -1.627520
Co -0.128705 -0.015739 -0.142962
C 1.056009 1.291675 0.048780
C -1.236118 -1.401506 0.066767
O 1.811062 2.154460 0.122408
O -1.931123 -2.311962 0.145235
C -1.495016 1.145626 0.049229
O -2.370539 1.882737 0.137533
C 2.700228 -1.113830 0.087174
C 1.207884 -1.221428 0.054029
H 3.075708 -1.555451 1.022545
H 3.103256 -0.104175 0.002343
H 3.128606 -1.727803 -0.719042
H 0.942770 -2.282238 0.104375
FREQS= 96 98 108 121 158 169 236 310 338 422 445 446 469 532 561 584 597 627 662 815 987
1036 1126 1389 1403 1482 1502 1912 2109 2119 2166 3007 3048 3083 3152
TS053_2
14
E= -1801.80914091 ZPE= 53.09 Gcorr 0.021575000
H -1.605617 -1.184275 0.437940
Co 0.018092 -0.123187 -0.072363
C -0.236730 1.374525 0.813929
C 1.005771 -1.482297 0.472552
O -0.314466 2.369956 1.390941
O 1.659834 -2.265789 1.011894
C 1.292597 0.569057 -1.093658
O 2.375984 1.062990 -1.129219
C -2.051706 -0.711581 -0.452145
C -3.349921 -0.406602 -0.461047
H 0.655469 0.394638 -2.038354
H -1.458462 -0.708803 -1.373561
H -3.970095 -0.519596 0.422847
H -3.840663 -0.051787 -1.361803
FREQS= 33 60 64 85 123 132 153 180 231 321 337 402 442 497 510 531 552 726 853 869 975
1036 1151 1250 1384 1470 1686 1887 2074 2130 2490 2984 3093 3168 3256
TS054_2
14
E= -1801.81266980 ZPE= 54.64 Gcorr 0.026099000
H -2.269938 1.619512 -0.123125
Co -0.157878 -0.032178 -0.105981
C -1.456501 1.087854 -0.653158
C 0.540934 1.097738 1.042295
O -1.260369 1.188530 -1.848672
O 0.906587 1.721600 1.944686
C -1.272022 -1.280820 0.439442
O -1.981161 -1.976755 1.033310
C 1.793162 -0.809885 -0.977419
C 2.991093 -0.863083 -0.387968
H 1.665585 -0.413335 -1.981512
H 0.962616 -1.431652 -0.574310
H 3.127400 -1.309445 0.592446
H 3.876594 -0.454086 -0.865750
FREQS= 58 72 87 101 112 134 158 219 245 315 361 406 471 515 531 540 691 833 895 986 997
1145 1260 1282 1361 1458 1678 1734 2050 2107 2853 2956 3163 3195 3252
TS055_2
14
E= -1801.81115941 ZPE= 52.18 Gcorr 0.024683000
H 0.265470 0.362205 -2.042901
Co 0.184875 0.015564 -0.401315
C 1.320605 -1.322989 -0.207234
C 1.072878 1.531111 -0.012112
O 2.070950 -2.175590 -0.018895
O 1.707237 2.418071 0.354566
C -0.817551 -0.283360 0.974946
O -1.177802 -0.520242 2.068594
C -1.816749 0.516012 -0.220591
C -2.725691 -0.215070 -0.880909
H -2.044704 1.545895 0.044004
H 0.016731 -0.413980 -2.000844
H -3.655651 0.224997 -1.236504
H -2.577509 -1.271500 -1.086974

FREQS=   66   75   93 104 160 260 284 316 359 419 458 466 482 489 511 540 557 608 613 708 952
984 1018 1047 1286 1417 1553 1632 1974 2098 2144 3139 3171 3231 3286
TS056_2
14
E= -1801.80924280 ZPE=   52.09 Gcorr   0.023964000
H -0.467873 -0.025875 -2.067694
Co -0.240260 0.070012 -0.432978
C -1.895455 -0.524087 -0.236957
C 0.529247 -0.957396 0.722901
O -2.970253 -0.886772 -0.037924
O 0.772983 -1.621597 1.659262
C -0.116252 1.719199 0.278509
O -0.133661 2.715280 0.854739
C 2.766752 -0.023334 -0.463101
C 1.654477 -0.745534 -0.644109
H 3.695989 -0.277572 -0.970488
H 2.796955 0.836775 0.200386
H -0.220085 0.746987 -1.960661
H 1.696866 -1.639013 -1.263210
FREQS=   54   71   91   99 142 256 284 324 347 421 437 462 472 487 512 534 577 582 602 695 976
989 1015 1057 1288 1418 1597 1637 1985 2095 2140 3136 3167 3227 3259
TS057_2
14
E= -1801.80840786 ZPE=   51.86 Gcorr   0.023369000
H -0.226276 -0.157560 2.071131
Co -0.190728 0.125865 0.428669
C -1.614138 -0.962641 0.147094
C -0.824889 1.575343 -0.317220
O -2.462519 -1.695780 -0.106712
O -1.234346 2.531441 -0.814479
C 1.513593 0.409474 0.112352
O 2.529137 1.014955 0.020094
C 1.407303 -1.224113 0.085384
C 1.257715 -1.896680 -1.068218
H 1.865030 -1.713445 0.943009
H 0.011457 0.623574 1.980483
H 1.524899 -2.948213 -1.151352
H 0.878845 -1.415936 -1.964916
FREQS=   30   87   98 101 208 227 261 321 349 370 443 449 474 491 513 526 562 583 634 765 964
992 1031 1035 1293 1420 1475 1628 1919 2096 2140 3145 3164 3241 3244
TS001_3
10
E= -1836.59891884 ZPE=   24.40 Gcorr   -0.018809000
H -0.000008 0.000032 -1.689971
Co 0.000001 0.000002 -0.193619
C -1.686492 -0.000025 -0.719259
C 1.686496 0.000009 -0.719255
O -2.762172 -0.000036 -1.122408
O 2.762177 0.000019 -1.122402
C 0.000017 -1.496374 0.821029
O 0.000027 -2.437244 1.478441
C -0.000019 1.496384 0.821019
O -0.000037 2.437255 1.478428
FREQS=   65   85   95 105 116 293 314 349 414 433 475 479 501 503 529 566 616 640 1937 2118 2121
2127 2189
TS002_3
10
E= -1836.57021533 ZPE=   24.69 Gcorr   -0.017075000
H 0.000000 0.000000 -1.669110
Co 0.000000 0.000000 -0.147396
C -1.768763 -0.019047 0.065969
C 0.019047 -1.768762 0.065970
O -2.915227 -0.031358 0.127044
O 0.031358 -2.915227 0.127052
C 1.768763 0.019047 0.065969
O 2.915227 0.031358 0.127044
C -0.019047 1.768762 0.065970
O -0.031358 2.915227 0.127052
FREQS= 100 100 104 104 144 285 285 352 447 463 475 475 546 578 578 606 631 631 1816 2116 2116
2123 2194
TS003_3
10
E= -1836.56795313 ZPE=   26.29 Gcorr   -0.016098000
H -0.000020 -0.039647 -2.893300
Co -0.000009 0.104897 -0.111148
C -1.526308 0.437916 0.849844
C 0.000112 -1.633956 -0.178244

O -2.511952 0.577829 1.421524
O 0.000191 -2.784781 -0.233579
C 1.526247 0.438083 0.849846
O 2.511887 0.578110 1.421507
C -0.000043 0.371810 -1.875240
O -0.000101 1.569379 -1.607320
FREQS= 58 76 78 107 110 270 304 335 404 426 436 451 497 502 562 676 820 1187 1650 2100 2108 2163 3070
TS004_3
10
E= -1836.54870685 ZPE= 22.67 Gcorr -0.034133000
H 0.329154 0.227083 -1.765516
Co 0.517649 0.417592 -0.279789
C 0.573771 0.489742 1.473461
C 1.639693 -0.903286 -0.601061
O 0.508489 0.443066 2.620714
O 2.335420 -1.766198 -0.898752
C -2.794315 -2.348699 -0.260524
O -2.163130 -1.670820 0.401462
C -0.703310 1.640337 -0.629686
O -1.505869 2.397622 -0.945089
FREQS= 12 20 25 32 46 88 98 109 254 339 383 470 481 491 518 587 598 758 1931 2106 2117 2193 2202
TS005_3
10
E= -1836.55001108 ZPE= 22.87 Gcorr -0.031899000
H -0.004721 -0.041191 -1.871884
Co -0.060957 -0.617599 -0.479880
C -0.125879 -1.289055 1.149215
C 1.675719 -0.410614 -0.680797
O -0.165371 -1.699015 2.221509
O 2.779892 -0.139048 -0.842249
C -1.725212 -0.079364 -0.679911
O -2.756139 0.400646 -0.840422
C 0.214888 2.229783 1.186372
O 0.318300 3.188900 0.583582
FREQS= 9 30 37 38 69 90 97 137 280 335 416 473 483 502 516 588 589 758 1936 2102 2122 2187 2205
TS006_3
10
E= -1836.56177319 ZPE= 25.28 Gcorr -0.019480000
H -0.000082 -0.573074 -2.099439
Co -0.000037 -0.193891 0.159739
C 0.000165 1.433372 0.879436
C 1.611905 -1.047200 0.184660
O 0.000285 2.410367 1.486848
O 2.672635 -1.483342 0.260445
C 0.000069 0.406763 -1.537972
O 0.000255 1.472148 -2.067419
C -1.612185 -1.046822 0.184601
O -2.673006 -1.482743 0.260394
FREQS= 53 63 65 95 110 184 205 300 344 414 463 469 480 497 499 593 781 1137 1871 2093 2094 2159 2713
TS007_3
10
E= -1836.55251722 ZPE= 23.47 Gcorr -0.026804000
H 0.002916 -0.700871 1.501551
Co -0.000058 0.008410 0.176387
C -1.631914 0.304912 0.748692
C 1.630073 0.315864 0.747563
O -2.624726 0.585833 1.259487
O 2.621339 0.603456 1.257673
C -0.004100 1.080050 -1.238553
O -0.006992 1.857327 -2.084024
C 0.016023 -2.923792 -0.371289
O 0.002647 -2.070167 -1.130945
FREQS= 13 47 51 77 98 98 115 311 317 337 453 486 487 510 568 582 620 755 1950 2093 2115 2159 2176
TS008_3
10
E= -1836.56020163 ZPE= 25.74 Gcorr -0.018367000
H 0.543669 0.000191 2.215414
Co 0.165778 0.000125 -0.207734
C -0.689219 -1.525223 -0.625002
C 1.972322 0.001779 0.056943
O -1.229636 -2.489390 -0.941861
O 3.122874 0.002801 0.068317

C -0.350748 -0.000084 1.547707
O -1.465869 -0.000418 1.974878
C -0.692378 1.523586 -0.625562
O -1.234810 2.486520 -0.942722
FREQS= 54 56 68 98 108 210 290 297 327 413 467 476 496 497 501 594 802 1232 1835 2085 2094 2159 2845
TS009_3
10
E= -1836.55263292 ZPE= 23.52 Gcorr -0.025427000
H -0.027841 0.200807 -1.685099
Co 0.052847 -0.036732 -0.205509
C -1.318287 -1.029923 -0.664514
C 1.747392 0.067219 -0.654033
O -2.098837 -1.774013 -1.068554
O 2.825324 -0.004747 -1.050835
C 0.239717 -0.584516 1.472427
O 0.397038 -1.040739 2.514909
C -1.660241 2.445844 0.105997
O -0.554838 2.244398 0.313802
FREQS= 27 51 57 76 97 98 122 300 322 340 453 487 488 511 567 581 619 758 1958 2094 2114 2157 2174
TS010_3
10
E= -1836.56090690 ZPE= 26.36 Gcorr -0.016150000
H -2.193551 -0.001075 -1.389276
Co 0.105281 -0.000081 -0.080737
C -0.079311 -1.770588 -0.067780
C 1.870091 0.001295 0.097300
O -0.178412 -2.912472 -0.026041
O 3.015051 0.002224 0.190747
C -0.081495 1.770189 -0.067810
O -0.182062 2.911947 -0.026113
C -1.839947 -0.000924 -0.324929
O -2.637709 -0.001270 0.579969
FREQS= 39 86 93 97 174 196 256 342 398 410 451 482 516 541 565 588 891 1358 1781 2104 2117 2188 2765
TS011_3
10
E= -1836.55754242 ZPE= 26.25 Gcorr -0.016567000
H -0.835441 2.050454 -1.322375
Co 0.108106 0.009587 -0.075052
C 1.425632 -1.214160 -0.388809
C 1.064208 1.409009 0.442732
O 2.189843 -1.948302 -0.827115
O 1.637401 2.244721 0.989899
C -1.129634 -0.996724 0.732994
O -1.851574 -1.558430 1.426595
C -1.221084 1.108751 -0.854004
O -2.340438 0.743191 -1.120467
FREQS= 47 69 84 100 115 268 288 331 379 433 465 479 509 531 553 630 829 1330 1771 2087 2109 2167 2788
TS012_3
10
E= -1836.54375003 ZPE= 23.40 Gcorr -0.024785000
H -0.001129 -0.214922 -1.555717
Co -0.000188 -0.039299 -0.129971
C -1.549802 -0.890815 -0.369251
C 1.546748 -0.895243 -0.370666
O -2.515151 -1.435865 -0.678135
O 2.510246 -1.443068 -0.680426
C 0.000039 -0.449427 2.053162
O 0.002951 0.650596 2.362938
C 0.002126 1.718898 -0.515391
O 0.003398 2.775277 -0.969651
FREQS= 24 55 65 92 100 116 213 305 322 333 390 433 451 507 509 515 635 681 2095 2096 2114 2138 2178
TS013_3
10
E= -1836.54379906 ZPE= 23.50 Gcorr -0.024309000
H 0.543568 -0.000143 -1.491659
Co 0.086778 -0.000012 -0.128280
C -0.618868 1.545713 -0.670130
C -0.620709 -1.544783 -0.670438
O -0.983417 2.507943 -1.187767
O -0.986381 -2.506488 -1.188258
C -1.700842 0.000360 2.133410
O -0.563537 0.000314 1.997003

C 1.844271 -0.001127 0.216457
O 2.994626 -0.001835 0.241450
FREQS=   44   58   62   89   90   93 204 308 311 312 430 447 458 509 512 516 679 704 2088 2093 2120
2136 2176
TS001_4
10
E= -1724.43437200 ZPE=   29.75 Gcorr   -0.005856000
H 0.003110 0.229040 2.058590
Co 0.000169 -0.206576 0.417692
C 1.605913 -0.608482 -0.195787
C -0.000751 1.521608 -0.019859
O 2.601627 -0.947076 -0.662585
O -0.001289 2.620021 -0.353110
C -1.605331 -0.609553 -0.195784
O -2.600957 -0.948573 -0.662453
H 0.001007 -1.693207 0.507390
H -0.002711 -0.574699 2.050097
FREQS=   69   99 101 259 286 327 420 438 476 494 503 546 566 575 674 751 918 1406 2009 2104 2124
2175 3490
TS002_4
10
E= -1724.42890456 ZPE=   29.01 Gcorr   -0.007158000
H -0.653500 0.463564 1.705551
Co 0.000036 -0.028927 0.480071
C 1.461557 -0.780494 -0.226432
C -0.000836 1.630398 -0.195572
O 2.409959 -1.314466 -0.584424
O -0.001378 2.716916 -0.556019
C -1.460790 -0.781903 -0.226367
O -2.408712 -1.316704 -0.584392
H 0.000768 -1.240785 1.315984
H 0.653226 0.464286 1.705456
FREQS=   79   81   95 269 270 293 446 457 489 521 526 545 550 657 687 779 812 2054 2082 2086 2155
2162 2196
TS003_4
10
E= -1724.42111268 ZPE=   28.43 Gcorr   -0.007889000
H 0.000002 -0.688134 -1.279345
Co 0.000001 -0.213273 0.151509
C -0.000005 1.578123 -0.053311
C -1.696449 -0.646631 -0.059623
O -0.000012 2.721896 -0.099428
O -2.777966 -0.982883 -0.233751
C 1.696454 -0.646622 -0.059623
O 2.777972 -0.982869 -0.233751
H 0.000001 -0.001183 1.634198
H 0.000004 -1.310682 1.125187
FREQS=   82   89 107 228 312 344 384 456 483 501 527 553 557 623 654 671 806 1879 2036 2094 2141
2159 2200
TS004_4
10
E= -1724.41991702 ZPE=   29.15 Gcorr   -0.007369000
H -0.000023 0.808589 1.679479
Co -0.000008 -0.128829 0.507244
C -1.546221 -0.702109 -0.229491
C 1.546186 -0.702172 -0.229486
O -2.521305 -1.031215 -0.741227
O 2.521261 -1.031310 -0.741219
C 0.000045 1.501365 -0.092259
O 0.000074 2.594252 -0.452960
H -0.000030 -0.738303 1.960370
H -0.000011 -1.428217 1.455213
FREQS=   65   93   98 259 276 281 394 397 447 484 498 500 573 581 613 632 1152 1881 1919 2107 2114
2167 2861
TS005_4
10
E= -1724.39736659 ZPE=   29.62 Gcorr   -0.007894000
H 2.776651 0.629572 -0.821709
Co 0.207494 -0.053383 0.154129
C -1.257974 -1.067220 0.015185
C -0.513999 1.501780 0.007881
O -2.167066 -1.699114 -0.298462
O -0.957798 2.552555 -0.170374
C 1.881344 0.136986 -0.419044
O 1.821247 -1.091403 -0.242257
H 0.829871 -0.048699 2.530085
H 0.563847 -0.665110 2.194741

FREQS= 87 104 105 129 189 218 292 340 364 392 449 476 506 544 609 685 824 1211 1597 2086 2141 3068 4302
TS006_4
10
E= -1724.40050106 ZPE= 31.45 Gcorr -0.002321000
H -0.394132 -2.123276 -1.578611
Co 0.032885 -0.193505 0.356662
C -1.541495 0.408050 -0.034916
C 1.142711 1.193187 -0.019071
O -2.582407 0.814245 -0.322879
O 1.712477 2.106732 -0.423192
C 0.066381 -1.572725 -0.747254
O 1.083305 -1.929129 -0.154712
H 0.221747 -0.140586 2.048082
H -0.428109 -0.617352 1.914365
FREQS= 80 103 115 237 300 345 404 435 464 497 506 578 636 702 817 917 1201 1441 1645 2091 2138 3065 3279
TS007_4
10
E= -1724.39753510 ZPE= 30.70 Gcorr -0.004834000
H 2.574529 -0.888782 -0.864512
Co 0.124749 -0.161835 0.299094
C 0.475848 1.499884 -0.022452
C 1.575176 -0.920048 -0.407489
O 0.740646 2.587510 -0.306023
O 0.874222 -1.937515 -0.389650
C -1.649121 -0.148403 -0.001320
O -2.730545 -0.200764 -0.389945
H 0.469763 -0.800141 1.978438
H 0.101488 -0.123964 2.083038
FREQS= 77 91 109 139 274 315 338 408 431 465 495 532 581 679 692 840 1022 1215 1624 2087 2137 3053 3870
TS008_4
10
E= -1724.39859255 ZPE= 31.49 Gcorr -0.003709000
H -0.000015 1.538871 -1.844428
Co 0.000025 -0.000217 0.417155
C 1.568711 -0.638060 -0.081580
C -1.568607 -0.638322 -0.081499
O 2.491013 -1.179587 -0.516555
O -2.490801 -1.179948 -0.516577
C -0.000115 1.373580 -0.752109
O -0.000328 2.273660 0.068698
H 0.000092 -0.354967 2.062413
H 0.000240 -1.074235 1.725424
FREQS= 61 105 106 152 214 281 356 393 487 508 524 528 560 668 893 935 1275 1485 1722 2069 2126 2984 3601
TS009_4
10
E= -1724.39495018 ZPE= 30.50 Gcorr -0.004635000
H 2.570612 -1.194363 0.820464
Co 0.027791 -0.213266 0.259911
C 0.501140 1.466883 -0.148570
C 1.659720 -0.987493 0.236028
O 0.762160 2.584149 -0.207209
O 1.431947 -1.462135 -0.865443
C -1.747264 -0.233325 -0.096039
O -2.875930 -0.300972 -0.281248
H -0.104471 0.072252 1.679272
H -0.243501 -1.164421 1.365349
FREQS= 75 90 114 170 259 311 375 439 490 498 518 596 614 695 781 904 1247 1706 2023 2112 2132 2167 3021
TS010_4
10
E= -1724.39649259 ZPE= 33.15 Gcorr -0.000828000
H -1.918313 -2.821658 -0.459681
Co 0.092427 0.018637 -0.228587
C -1.071346 1.287950 0.018197
C 1.819671 -0.020838 0.040884
O -1.652691 2.254732 0.275383
O 2.929717 0.214371 0.253206
C -1.634817 -1.882821 0.032722
O -0.448589 -1.689986 0.358281
H -2.420714 -1.156544 0.265604
H 0.534971 0.936326 -1.279849
FREQS= 66 80 107 128 284 363 390 434 475 498 537 540 644 780 1066 1247 1489 1647 2045 2061 2127 3035 3144

TS011_4
10
E= -1724.38883669 ZPE= 30.35 Gcorr -0.006248000
H 0.000033 -1.058068 -1.477232
Co 0.000032 0.133162 0.299654
C -1.632578 0.815632 -0.118177
C 1.632795 0.815325 -0.118156
O -2.708940 1.165463 -0.319884
O 2.709209 1.164977 -0.319888
C -0.000165 -1.480580 -0.412329
O -0.000399 -2.631964 -0.120854
H 0.427779 -0.513765 1.681779
H -0.427956 -0.513607 1.681770
FREQS= 57 66 94 188 233 280 352 425 461 476 502 522 668 672 746 1059 1091 1783 1898 2098 2145
2558 2856
TS012_4
10
E= -1724.39013086 ZPE= 30.87 Gcorr -0.004473000
H -0.490558 2.075021 -0.762331
Co -0.136300 -0.032709 0.517073
C -1.759351 -0.173660 -0.205759
C 1.193477 -1.072901 -0.081960
O -2.785481 -0.460406 -0.647344
O 1.958340 -1.821491 -0.503095
C 0.361030 1.465201 -0.380352
O 1.496423 1.833464 -0.491269
H -0.373518 0.697324 1.951793
H 0.418978 0.386429 1.991662
FREQS= 62 94 102 206 252 265 346 457 459 474 517 528 618 724 811 1022 1261 1634 1818 2083 2136
2857 2870
TS013_4
10
E= -1724.38823536 ZPE= 30.10 Gcorr -0.006254000
H 0.486143 2.293471 0.634723
Co 0.110779 -0.144279 0.626215
C 1.594728 -0.483712 -0.318079
C -1.376670 -0.819877 -0.180629
O 2.588400 -0.667271 -0.861894
O -2.322335 -1.317641 -0.592174
C -0.116279 1.569853 0.042128
O -0.819059 1.909492 -0.864454
H -0.510403 0.292871 1.908522
H 0.846504 0.314972 1.836617
FREQS= 68 71 100 144 251 307 327 380 469 503 528 599 608 664 829 884 1258 1828 2009 2028 2137
2170 2895
TS014_4
10
E= -1724.38697789 ZPE= 31.26 Gcorr -0.003864000
H 0.000001 2.849157 0.764494
Co 0.000000 0.119938 0.307641
C -1.513403 -0.817602 -0.089841
C 1.513402 -0.817603 -0.089840
O -2.496554 -1.367787 -0.318778
O 2.496553 -1.367788 -0.318778
C 0.000001 1.887214 0.232540
O 0.000001 1.764283 -0.990179
H 0.429523 0.085402 1.816959
H -0.429524 0.085403 1.816959
FREQS= 60 88 96 198 259 332 419 425 441 454 472 554 587 687 832 1146 1186 1643 1859 2100 2143
2830 3055
TS015_4
10
E= -1724.38891975 ZPE= 32.20 Gcorr -0.001607000
H 1.969157 1.953178 0.432190
Co -0.000360 0.089041 -0.060873
C -1.702643 -0.472301 0.057299
C 1.268784 -1.093662 0.019992
O -2.743950 -0.947341 0.141764
O 2.034349 -1.954787 -0.015674
C 0.910534 1.673754 0.407765
O 0.049516 2.194899 -0.339557
H 0.538684 1.153500 1.389292
H -0.077499 -0.499692 -1.380515
FREQS= 66 97 114 179 304 354 370 427 463 490 540 666 681 731 1082 1187 1380 1615 2084 2100 2157
2338 3095
TS016_4
10

E= -1724.38422682 ZPE=   30.49 Gcorr   -0.004172000
H -2.227925 1.307854 -1.030509
Co -0.026279 0.126135 0.083519
C 1.783952 -0.031687 0.000950
C -0.843751 -1.384513 0.000966
O 2.904432 -0.209460 -0.150336
O -1.359689 -2.414239 -0.008855
C -1.317682 1.256560 -0.421200
O -0.885594 2.133997 0.307125
H -0.037014 0.578495 -1.381057
H -0.033829 -0.416533 1.488787
FREQS=   74 102 116 180 280 370 394 443 488 498 546 652 672 732 754 947 1227 1694 1821 1966 2117
2170 3087
TS017_4
10
E= -1724.38638348 ZPE=   31.93 Gcorr   -0.001651000
H 0.276315 2.085127 -0.654225
Co 0.162378 -0.161179 -0.155171
C -1.305147 -1.098367 0.030911
C 1.939916 -0.106659 0.047677
O -2.171444 -1.833532 0.199500
O 3.057664 -0.265283 0.251571
C -0.509491 1.624769 -0.003026
O -1.612617 2.096660 0.123446
H -0.043025 0.973123 0.960144
H 0.442019 -1.207648 -1.165802
FREQS=   74   93 112 242 326 343 390 459 475 506 565 590 733 800 1057 1332 1457 1784 1883 2019 2108
2169 2816
TS018_4
10
E= -1724.38483242 ZPE=   31.56 Gcorr   -0.002850000
H -0.000025 1.718816 -1.516864
Co -0.000002 -0.424813 -0.016085
C 1.778490 -0.424787 -0.016083
C -1.778495 -0.424762 -0.016023
O 2.920748 -0.517164 0.043664
O -2.920756 -0.517155 0.043642
C -0.000002 1.435974 -0.431161
O 0.000024 2.297822 0.417693
H -0.000016 -2.033284 -0.058286
H 0.000011 -1.842155 0.749053
FREQS=   75   88 128 191 238 345 366 369 448 521 531 577 621 702 902 1021 1359 1678 1775 2096 2175
2761 3105
TS019_4
10
E= -1724.38128777 ZPE=   29.77 Gcorr   -0.006274000
H -0.834607 2.043824 0.000049
Co -0.146571 -0.319011 -0.000001
C -1.930346 -0.271238 0.000003
C 1.598011 -0.750122 -0.000006
O -3.073721 -0.327758 0.000002
O 2.683095 -1.107887 0.000016
C 0.119109 1.469555 0.000020
O 1.187060 2.007172 -0.000034
H -0.150000 -0.345815 1.522517
H -0.150091 -0.346079 -1.522518
FREQS=   59   86 124 134 260 295 401 436 513 516 552 579 649 654 702 817 1267 1785 1837 1946 2138
2191 2880
TS020_4
10
E= -1724.38361482 ZPE=   31.59 Gcorr   -0.003455000
H 2.093006 -0.483270 1.521586
Co -0.082805 -0.402208 0.006552
C 0.135071 1.314888 0.040893
C 1.806117 -0.486759 0.436538
O 0.267803 2.454742 -0.051022
O 2.664258 -0.577094 -0.412254
C -1.849900 -0.363432 -0.106252
O -2.993421 -0.313529 0.015205
H 0.307782 -1.997015 0.134848
H -0.221907 -1.961237 -0.475834
FREQS=   61   88 104 185 233 302 351 429 446 498 545 559 585 627 869 1041 1363 1651 1773 2095 2147
2758 3383
TS021_4
10
E= -1724.38628370 ZPE=   33.54 Gcorr   0.001597000
H 2.021121 0.923932 1.435805

Co 0.245370 -0.000051 -0.285959
C -0.926518 1.240866 0.083388
C -0.926844 -1.240664 0.083407
O -1.630387 2.136303 0.228406
O -1.630951 -2.135912 0.228430
C 1.782587 -0.000165 0.906693
O 1.995594 -0.000199 -0.433870
H 2.020978 -0.924285 1.435830
H -0.116488 -0.000038 -1.775397
FREQS= 58 99 118 254 372 435 467 468 516 566 625 658 731 803 1046 1187 1189 1540 1814 2120 2150 3074 3169
TS022_4
10
E= -1724.37913933 ZPE= 30.76 Gcorr -0.003690000
H 0.000000 2.055903 1.570050
Co 0.000000 -0.208598 -0.209710
C -1.725995 -0.487833 0.089933
C 1.725995 -0.487833 0.089933
O -2.812195 -0.801853 0.286953
O 2.812195 -0.801853 0.286953
C 0.000000 1.476307 0.626635
O 0.000000 2.015347 -0.468643
H -0.000001 -1.643301 -0.162899
H 0.000000 -1.077421 -1.426080
FREQS= 81 106 118 182 227 331 400 460 493 523 530 595 663 728 820 852 1273 1706 1980 2116 2168 2223 2946
TS023_4
10
E= -1724.37608548 ZPE= 29.16 Gcorr -0.006642000
H 0.455389 0.724092 1.874403
Co -0.013256 0.051895 0.445081
C -1.502115 -0.672573 -0.168919
C 1.500188 -0.665495 -0.119324
O -2.391410 -1.300111 -0.543510
O 2.410911 -1.279580 -0.463112
C 0.599607 1.829565 -0.798810
O -0.430127 2.011506 -0.293672
H -0.376739 0.698321 1.913248
H -0.021806 -1.227092 1.119825
FREQS= 78 111 118 235 266 317 357 374 427 438 507 526 547 626 729 749 980 1577 1990 2103 2106 2156 3082
TS024_4
10
E= -1724.37781926 ZPE= 30.49 Gcorr -0.004883000
H 1.159816 0.283590 1.306033
Co 0.077183 0.181266 0.279098
C -1.593005 0.695241 -0.083655
C -0.098385 -1.516190 -0.138278
O -2.643794 1.097576 -0.302427
O -0.301612 -2.648787 -0.212892
C 1.914986 0.223043 -0.010802
O 2.049119 1.276369 -0.595342
H 2.686826 -0.558661 0.051086
H -0.101846 1.167064 1.388916
FREQS= 68 100 109 169 210 332 379 414 478 523 545 564 640 704 771 974 1303 1737 1964 2044 2107 2162 3032
TS001_5
16
E= -1915.22503422 ZPE= 61.65 Gcorr 0.032530000
H -3.502501 0.882136 -0.119923
Co 0.234304 -0.000016 -0.010206
C 0.855790 0.000353 -1.698180
C 1.843611 -0.000135 0.782395
O 1.193289 0.000609 -2.796628
O 2.867766 -0.000212 1.298654
C -0.304746 -1.570717 0.672651
O -0.697728 -2.576722 1.066497
C -2.875228 0.000074 0.044867
C -1.660438 0.000065 -0.903360
H -2.608717 0.000110 1.105422
H -3.502489 -0.882008 -0.119860
H -1.681057 0.876320 -1.553798
H -1.681108 -0.876137 -1.553866
C -0.304782 1.570426 0.673234
O -0.697774 2.576274 1.067471
FREQS= 49 52 90 90 92 99 100 133 257 316 355 364 417 433 475 478 487 496 510 542 553 562 724 1001 1012 1039 1224 1230 1429 1492 1506 1519 2101 2103 2119 2178 3048 3084 3107 3114 3140

TS002_5
16
E= -1915.22772156 ZPE= 62.82 Gcorr 0.035280000
H 4.508554 0.216751 -0.244196
Co -0.537752 0.001676 0.106367
C -2.215178 0.449319 0.623758
C -0.136518 1.428121 -0.851051
O -3.250548 0.731294 1.029604
O 0.068952 2.274366 -1.609084
C -0.873303 -1.549205 -0.671832
O -1.114584 -2.467855 -1.327258
C 2.546534 -0.695637 -0.072632
C 3.535085 0.455221 0.191728
H 2.423791 -0.872271 -1.143327
H 2.911392 -1.621167 0.390520
H 3.182145 1.387710 -0.258184
H 3.662655 0.616403 1.264701
C 1.205825 -0.392493 0.535533
O 0.978193 -0.280883 1.729932
FREQS= 38 51 75 84 86 115 204 215 255 300 302 359 408 461 475 511 521 540 546 576 683
806 942 1035 1077 1132 1267 1342 1421 1488 1511 1519 1741 2071 2098 2159 3053 3062 3124 3137 3151
TS003_5
16
E= -1915.22583367 ZPE= 62.50 Gcorr 0.033402000
H 4.448126 0.882526 0.265380
Co -0.559500 0.000006 0.097003
C -0.593235 -1.544858 -0.757580
C -2.242379 -0.000099 0.762487
O -0.663809 -2.463899 -1.452271
O -3.275828 -0.000166 1.260637
C -0.593402 1.544886 -0.757579
O -0.664099 2.463903 -1.452287
C 3.835845 0.000021 0.464562
C 2.560027 -0.000038 -0.401418
H 4.448104 -0.882534 0.265531
H 3.581990 0.000116 1.526849
H 2.508701 -0.866644 -1.072325
H 2.508737 0.866417 -1.072520
C 1.267663 0.000065 0.361426
O 1.129203 0.000173 1.573497
FREQS= 44 46 62 73 83 85 120 200 292 300 301 332 411 419 461 511 522 533 547 561 720
821 947 1054 1087 1093 1247 1352 1429 1456 1515 1520 1746 2072 2098 2159 3054 3071 3081 3143 3149
TS004_5
16
E= -1915.22426304 ZPE= 62.36 Gcorr 0.032500000
H 3.655375 -0.880336 -0.982416
Co -0.508694 -0.000103 0.142490
C -2.313438 0.000120 0.271879
C -0.286725 1.539381 -0.694667
O -3.448852 0.000178 0.436520
O -0.157660 2.456117 -1.383899
C -0.287323 -1.538335 -0.697015
O -0.158451 -2.454117 -1.387554
C 2.620798 -0.002381 0.732255
C 3.058576 0.002143 -0.740881
H 3.003873 -0.877239 1.272672
H 3.006698 0.867175 1.279125
H 2.192437 0.006096 -1.408194
H 3.658474 0.884268 -0.975956
C 1.134209 -0.001273 0.974769
O 0.597626 -0.001567 2.071122
FREQS= 33 41 49 75 84 86 116 169 286 299 338 394 426 433 473 509 521 536 551 560 564
800 973 1065 1071 1138 1244 1322 1438 1453 1514 1518 1734 2073 2098 2159 3046 3068 3083 3137 3143
TS005_5
16
E= -1915.22594478 ZPE= 62.16 Gcorr 0.035257000
H -2.952017 -0.884317 0.721863
Co 0.219286 -0.000017 -0.036250
C 0.364429 1.572148 -0.887547
C 1.914087 -0.000007 0.553076
O 0.405011 2.578883 -1.441780
O 3.000496 -0.000001 0.920091
C 0.364031 -1.572868 -0.886332
O 0.404369 -2.580025 -1.439816
C -2.926765 0.000034 0.077833
C -1.732542 -0.000112 -0.870872
H -3.855483 -0.000057 -0.508776

H -2.952005 0.884590 0.721578
H -1.787723 0.870911 -1.525511
H -1.787740 -0.871315 -1.525270
C -0.516057 0.000762 1.605332
O -0.983483 0.001257 2.654747
FREQS= 49 55 94 95 117 127 154 261 289 311 356 362 413 431 477 483 485 505 507 544 557 573 718 993 1013 1052 1269 1295 1425 1487 1510 1511 2100 2104 2120 2177 3026 3083 3098 3114 3144
TS006_5
16
E= -1915.22385287 ZPE= 62.13 Gcorr 0.034325000
H -3.418618 0.487847 -1.076661
Co 0.244862 -0.016082 -0.022439
C 0.734152 1.586443 -0.654041
C 0.282375 0.456217 1.661894
O 1.017309 2.597629 -1.116016
O 0.319173 0.713077 2.785479
C 1.692939 -1.093011 -0.419795
O 2.634103 -1.746541 -0.477255
C -2.964076 0.155880 -0.140760
C -1.060368 -1.549785 0.404995
H -2.766009 1.039838 0.471277
H -3.667054 -0.481586 0.401583
H -1.478095 -1.689109 1.397752
H -0.639915 -2.444103 -0.041590
C -1.689234 -0.595492 -0.465407
O -1.047624 -0.344186 -1.550687
FREQS= 22 61 71 80 95 120 175 278 324 353 377 381 420 443 445 464 491 522 540 575 610 824 849 981 992 1039 1082 1325 1406 1468 1489 1513 1523 2094 2114 2162 3065 3137 3154 3156 3243
TS007_5
16
E= -1915.22124632 ZPE= 62.07 Gcorr 0.032915000
H -4.732681 0.000075 0.302286
Co 0.519708 -0.000052 -0.114129
C 0.693111 0.000521 1.614449
C 1.409764 -1.533303 -0.565142
O 0.793411 0.000916 2.764124
O 1.952658 -2.524001 -0.777967
C 1.410128 1.532677 -0.566236
O 1.953380 2.523067 -0.779584
C -3.827834 -0.000254 -0.311383
C -2.584501 0.000723 0.581784
H -3.848403 -0.884206 -0.953835
H -3.848753 0.882623 -0.955301
H -2.558799 -0.868983 1.250548
H -2.559012 0.871714 1.248891
C -1.285246 0.000211 -0.159814
O -1.121576 -0.000388 -1.378205
FREQS= 42 53 65 77 77 97 172 197 258 285 297 356 366 410 436 451 458 525 535 568 691 767 953 1028 1081 1112 1278 1367 1428 1453 1508 1514 1695 2088 2097 2152 3052 3064 3081 3139 3144
TS008_5
16
E= -1915.22060290 ZPE= 62.20 Gcorr 0.033268000
H -4.323915 0.058600 -0.473845
Co 0.492373 -0.009824 0.111947
C 0.028792 0.286112 -1.536695
C 1.252329 1.597029 0.540357
O -0.277045 0.475998 -2.633540
O 1.685150 2.635477 0.776059
C 1.710490 -1.360681 -0.096673
O 2.444739 -2.221454 -0.299292
C -2.593732 -0.695334 0.583445
C -3.271212 0.321599 -0.341389
H -2.662813 -1.700932 0.146144
H -3.073680 -0.734737 1.567715
H -3.223971 1.329486 0.080121
H -2.797796 0.341175 -1.325598
C -1.129609 -0.446362 0.779881
O -0.502122 -0.545338 1.832941
FREQS= 28 55 74 77 92 96 157 198 265 292 339 366 408 434 446 452 464 516 553 561 583 781 939 1040 1077 1165 1277 1341 1432 1464 1512 1518 1687 2084 2098 2151 3038 3065 3092 3137 3150
TS009_5
16
E= -1915.21690945 ZPE= 61.74 Gcorr 0.033495000
H -2.876846 -0.887093 0.296131
Co 0.232803 0.000457 -0.004217
C -0.064614 1.729856 -0.233266
C 0.145133 -0.000221 1.794521

O -0.275315 2.849582 -0.388565
O 0.126108 -0.000569 2.943951
C 1.921502 0.005521 -0.625926
O 3.010380 0.008773 -0.995290
C -2.819372 -0.005857 -0.350383
C -1.559502 -0.004359 -1.204892
H -3.715154 -0.009018 -0.987203
H -2.880985 0.877257 0.293182
H -1.525300 0.864314 -1.864563
H -1.522092 -0.874411 -1.862550
C -0.054418 -1.730573 -0.234011
O -0.258383 -2.851484 -0.389770
FREQS= 36 60 88 96 99 116 155 230 239 261 310 361 408 437 458 474 494 503 527 566 577
591 735 973 974 1053 1217 1261 1427 1504 1505 1509 2102 2104 2112 2172 3020 3084 3098 3111 3139
TS010_5
16
E= -1915.21720928 ZPE= 62.27 Gcorr 0.034434000
H 3.132808 -1.100361 0.230576
Co -0.351396 -0.012323 -0.043096
C 1.331423 0.692714 0.059465
C -1.097327 1.560844 -0.353708
O 2.083404 1.417334 0.634194
O -1.643904 2.572487 -0.420389
C -0.283220 -0.631242 1.613990
O -0.346422 -0.929430 2.724593
C 2.829429 -1.197767 -0.814817
C 1.675822 -0.231007 -1.156162
H 3.704508 -0.975242 -1.429962
H 2.546695 -2.238005 -0.992331
H 0.798674 -0.797020 -1.529840
H 1.925668 0.456425 -1.968066
C -1.673548 -1.087264 -0.681330
O -2.507597 -1.766735 -1.082323
FREQS= 49 55 72 85 88 98 135 255 264 318 348 366 383 438 475 501 515 528 532 560 680
705 860 1001 1067 1087 1240 1314 1434 1460 1507 1514 1889 2091 2103 2163 2925 3066 3105 3140 3144
TS011_5
16
E= -1915.21671062 ZPE= 62.34 Gcorr 0.035204000
H -3.716463 -0.500576 0.373040
Co 0.276486 -0.018249 -0.019178
C -0.249711 0.835387 1.418668
C 0.895112 -1.615648 0.664044
O -0.658726 1.376050 2.349102
O 1.339111 -2.586341 1.081917
C 1.625041 1.101700 -0.424512
O 2.508483 1.767077 -0.731945
C -1.768685 -0.491347 -0.555868
C -3.030142 0.209888 -0.093522
H -1.203001 0.969723 -2.098402
H -1.736667 -1.568098 -0.423411
H -2.846747 1.005164 0.633785
H -3.552704 0.666143 -0.942696
C -0.969665 0.019598 -1.600626
O 0.133478 -0.611423 -1.883279
FREQS= 34 56 68 89 101 110 205 278 300 356 379 395 417 424 433 448 481 517 535 576 614
869 946 986 1059 1114 1242 1299 1358 1437 1500 1509 1536 2102 2115 2163 3048 3067 3111 3135 3198
TS012_5
16
E= -1915.21446272 ZPE= 62.27 Gcorr 0.033701000
H -3.757488 -0.000050 2.188950
Co 0.502083 -0.000001 0.019670
C 0.674095 -1.598703 -0.709964
C 0.674108 1.598714 -0.709929
O 0.893275 -2.555429 -1.312800
O 0.893295 2.555452 -1.312745
C 1.975774 -0.000016 1.069953
O 2.844566 -0.000026 1.820472
C -3.400165 -0.000015 1.155299
C -1.866142 -0.000014 1.130713
H -3.794521 -0.881840 0.645807
H -3.794522 0.881846 0.645868
H -1.480285 -0.881092 1.656754
H -1.480284 0.881042 1.656789
C -1.340444 0.000013 -0.352443
O -2.075198 0.000034 -1.298306
FREQS= 33 57 69 87 90 94 151 211 263 314 336 352 379 454 461 488 494 537 554 580 669
790 797 977 1059 1083 1279 1315 1419 1473 1504 1510 1844 2083 2101 2164 3046 3064 3084 3142 3154

TS013_5
16
E= -1915.21268762 ZPE= 61.67 Gcorr 0.032345000
H -2.355948 -1.826014 0.710845
Co 0.276142 0.028415 -0.019113
C 0.964708 -1.640967 -0.147727
C 1.268010 0.926524 -1.223225
O 1.361246 -2.714661 -0.239704
O 1.952167 1.543219 -1.910897
C 0.799809 0.321238 1.649094
O 1.180713 0.558165 2.708822
C -2.787254 -1.135413 -0.018759
C -1.716341 -0.517808 -0.906898
H -3.502356 -1.698197 -0.631220
H -3.347755 -0.371197 0.526836
H -2.131816 0.140362 -1.671414
H -1.156077 -1.280112 -1.446548
C -1.129143 1.052246 0.059444
O -1.914201 1.892405 0.261278
FREQS= 28 61 73 82 90 110 119 214 245 294 329 355 385 434 454 480 487 516 528 554 582
608 758 961 987 1061 1209 1264 1425 1485 1505 1514 2020 2102 2109 2166 3041 3102 3116 3130 3156
TS014_5
16
E= -1915.21110640 ZPE= 62.03 Gcorr 0.030952000
H -4.262772 0.014374 -1.598601
Co 0.652697 -0.001250 0.008966
C 2.388171 -0.012366 -0.344843
C 0.512619 1.760575 0.198282
O 3.494328 -0.019210 -0.658518
O 0.474803 2.887914 0.412459
C 0.490222 -1.761860 0.197699
O 0.438327 -2.888414 0.412573
C -3.685539 -0.011109 -0.669927
C -2.185679 0.029791 -0.969046
H -3.982865 0.840310 -0.052885
H -3.949850 -0.919864 -0.123252
H -1.914670 0.929032 -1.540329
H -1.876776 -0.814655 -1.600764
C -1.309418 0.011023 0.279985
O -1.769727 0.005738 1.398593
FREQS= 12 39 60 88 97 103 137 192 254 257 268 362 370 428 454 460 486 540 583 585 688
780 893 996 1066 1096 1273 1346 1421 1458 1506 1512 1776 2096 2109 2179 3026 3057 3062 3135 3143
TS015_5
16
E= -1915.21134884 ZPE= 62.06 Gcorr 0.033308000
H 1.834606 0.000755 3.247780
Co -0.345233 -0.000002 -0.057336
C -2.018679 0.000148 0.614413
C -0.358715 1.635216 -0.724899
O -3.053353 0.000230 1.111513
O -0.445771 2.626186 -1.304349
C -0.358836 -1.635433 -0.724362
O -0.445942 -2.626575 -1.303510
C 2.221362 0.000248 1.107029
C 1.271615 0.000389 2.309881
H 2.876518 0.879315 1.125694
H 2.876775 -0.878614 1.126088
H 0.634011 0.891522 2.324989
H 0.634350 -0.890976 2.325473
C 1.571141 -0.000128 -0.301014
O 2.257280 -0.000415 -1.289685
FREQS= 35 50 67 89 95 99 139 185 260 309 314 357 407 459 470 479 499 541 551 563 582
764 863 1032 1049 1098 1277 1317 1425 1455 1511 1512 1808 2085 2104 2167 3037 3049 3087 3108 3114
TS016_5
16
E= -1915.21116204 ZPE= 62.29 Gcorr 0.034134000
H 2.283349 -2.003228 -0.698266
Co -0.379657 0.031857 0.041573
C -0.802653 1.746102 -0.028627
C -1.909860 -0.645934 -0.630656
O -1.166307 2.831166 0.098791
O -2.827761 -1.105127 -1.145397
C -0.134116 -1.135658 1.345317
O -0.084846 -1.789685 2.291319
C 1.908549 -1.388211 -1.521784
C 2.054404 0.108879 -1.205136
H 2.481419 -1.643874 -2.417894

H 0.868197 -1.675289 -1.707150
H 1.590362 0.711803 -1.994670
H 3.109778 0.395395 -1.151758
C 1.459291 0.539564 0.171887
O 2.136906 1.064473 1.012946
FREQS= 39 59 68 89 93 96 147 226 238 314 322 358 391 457 477 488 505 547 551 559 629
770 843 999 1065 1116 1274 1310 1427 1483 1504 1515 1821 2085 2102 2165 3046 3048 3097 3119 3129
TS017_5
16
E= -1915.20949427 ZPE= 62.10 Gcorr 0.033211000
H -1.138339 -0.084995 2.270832
Co 0.479123 -0.000068 -0.053658
C 2.187080 -0.202183 0.371584
C 0.179388 -1.715988 -0.420003
O 3.291007 -0.332910 0.665260
O 0.047818 -2.810160 -0.741652
C 0.517927 1.773707 -0.124122
O 0.603835 2.910843 -0.261368
C -2.088125 -0.439816 1.854265
C -2.460914 0.318959 0.579777
H -1.994252 -1.514406 1.667640
H -2.853314 -0.303376 2.623581
H -2.564455 1.391539 0.799959
H -3.421441 -0.013753 0.169740
C -1.430113 0.231634 -0.545424
O -1.742158 0.323347 -1.709670
FREQS= 37 41 78 89 96 103 115 232 251 264 310 365 387 433 460 483 486 542 558 585 601
773 895 1030 1060 1115 1272 1325 1425 1461 1508 1514 1777 2094 2109 2178 3026 3041 3085 3110 3125
TS018_5
16
E= -1915.20759637 ZPE= 61.89 Gcorr 0.031443000
H 1.666054 -1.022022 2.804390
Co -0.352227 0.000958 0.004122
C -2.057853 -0.001990 0.586145
C -0.353812 1.654049 -0.619440
O -3.141978 -0.001814 0.963327
O -0.429034 2.662612 -1.169815
C -0.360595 -1.628403 -0.681969
O -0.429543 -2.618312 -1.264851
C 2.296889 0.079551 1.007756
C 1.383518 -0.132522 2.236042
H 2.777527 1.064431 1.038047
H 3.108688 -0.654192 0.965724
H 1.398125 0.720317 2.918009
H 0.328027 -0.291182 1.951969
C 1.569133 0.012087 -0.346801
O 2.171558 -0.009967 -1.388639
FREQS= 26 43 51 74 91 98 101 146 296 310 320 359 409 453 464 485 507 537 556 563 577
807 914 1035 1038 1097 1233 1301 1439 1462 1468 1517 1809 2087 2105 2167 2949 3056 3096 3102 3145
TS019_5
16
E= -1915.20292067 ZPE= 61.61 Gcorr 0.030289000
H 3.308624 0.584098 0.369191
Co -0.563213 0.022886 0.281481
C 0.846488 0.011509 -0.890378
C -1.648995 0.800983 -0.879660
O 1.021040 0.548313 -1.944127
O -2.452474 1.352787 -1.492836
C 0.165798 1.144627 1.491298
O 0.497796 1.991072 2.199298
C 3.281469 -0.292249 -0.285180
C 1.901057 -0.958092 -0.265462
H 3.533138 0.028444 -1.298398
H 4.040374 -0.998627 0.063584
H 1.635948 -1.279079 0.742331
H 1.881335 -1.843264 -0.911464
C -1.148001 -1.697693 0.584986
O -1.513805 -2.787674 0.600310
FREQS= 31 37 59 68 78 89 112 176 234 260 299 317 364 412 442 452 486 493 550 556 661
767 829 979 1058 1097 1268 1313 1419 1480 1505 1516 1840 2076 2087 2147 3058 3067 3128 3132 3155
TS020_5
16
E= -1915.18778287 ZPE= 59.59 Gcorr 0.015347000
H -2.887337 0.940156 -0.786202
Co -0.564669 -0.036564 -0.194882
C 0.176483 -0.681888 -1.681285
C 0.165555 1.566413 -0.249901

O 0.603547 -1.097098 -2.664284
O 0.763105 2.546879 -0.147393
C -0.366661 -1.473405 0.803429
O -0.095558 -2.318896 1.538382
C -1.850364 0.650223 1.139216
C -3.042348 0.467843 0.195708
H -1.720098 1.694735 1.426412
H -1.967347 0.065527 2.052528
H -3.293259 -0.585368 0.030191
H -3.942003 0.944242 0.608492
C 3.665725 0.467573 1.286321
O 3.299626 -0.137463 0.394478
FREQS= 8 12 21 29 41 51 78 82 96 105 151 196 316 403 406 468 500 506 534 547 577
581 793 941 955 984 1210 1243 1409 1497 1503 1510 2075 2096 2160 2205 3004 3048 3091 3098 3147
TS021_5
16
E= -1915.20424552 ZPE= 62.21 Gcorr 0.034461000
H -4.078906 0.576350 0.043003
Co 0.588552 -0.031758 0.012607
C 2.074322 -0.557331 0.915726
C 0.273640 -1.499564 -0.911018
O 2.865500 -0.882281 1.681564
O 0.206758 -2.351926 -1.685265
C 1.081814 1.624517 -0.458384
O 1.449678 2.593368 -0.952553
C -2.300452 -0.648430 0.259760
C -3.659560 -0.148401 0.744837
H -1.873299 -1.383448 0.953865
H -2.381900 -1.174627 -0.701655
H -4.358180 -0.984124 0.846498
H -3.573129 0.347195 1.715470
C -1.276466 0.479370 0.033063
O -1.620095 1.637733 0.118569
FREQS= 40 66 84 90 94 106 192 202 257 294 315 338 378 424 467 482 483 526 560 572 680
781 892 1000 1057 1099 1276 1331 1422 1459 1504 1511 1760 2077 2103 2162 3033 3060 3066 3132 3144
TS022_5
16
E= -1915.18785490 ZPE= 59.75 Gcorr 0.018312000
H -0.153550 3.106974 -1.656425
Co -0.557968 -0.070316 0.089091
C -2.150621 -0.508958 -0.524106
C 0.198838 -1.674704 -0.059611
O -3.246554 -0.789631 -0.743819
O 0.721308 -2.689444 -0.198442
C 0.148109 0.369427 1.642948
O 0.447978 0.620809 2.726744
C -0.090476 2.067303 -1.306658
C -1.063858 1.829436 -0.148092
H -0.307293 1.438649 -2.179404
H 0.955949 1.903394 -1.022039
H -2.095901 2.014565 -0.450511
H -0.845929 2.486830 0.695339
C 4.345018 0.118907 -0.809736
O 3.225995 0.075723 -0.604592
FREQS= 12 19 28 37 51 62 78 94 104 118 163 176 314 402 406 470 497 503 534 547 579
583 789 952 959 986 1219 1249 1412 1503 1505 1510 2077 2096 2160 2203 3015 3064 3086 3092 3141
TS023_5
16
E= -1915.19847172 ZPE= 61.64 Gcorr 0.030074000
H -0.708073 -0.522334 1.730605
Co 0.483648 -0.000574 -0.075211
C 0.508031 1.651710 -0.698884
C 1.985739 0.134960 0.866935
O 0.575079 2.655506 -1.256967
O 2.943164 0.221837 1.495284
C 0.747900 -1.664326 -0.612847
O 0.955113 -2.679290 -1.112914
C -1.707721 -0.054020 1.719206
C -1.355771 -0.146884 -0.862803
H -2.351281 -0.565015 2.439859
H -1.603839 0.988566 2.042529
H -1.644612 0.627504 -1.578350
H -1.549775 -1.094644 -1.376371
C -2.317336 -0.073687 0.323240
O -3.519101 -0.011191 0.170014
FREQS= 8 40 49 80 96 103 143 152 296 313 391 408 458 474 481 487 515 527 578 589 653
746 778 958 977 1153 1178 1188 1382 1451 1453 1478 1815 2090 2108 2169 2956 3068 3081 3114 3137

TS024_5
16
E= -1915.18530446 ZPE=   59.42 Gcorr   0.018997000
H -2.599201 2.711702 -1.393029
Co -0.278947 -0.170475 0.150151
C -1.630754 -1.086457 -0.512688
C 0.903433 -1.502648 0.156671
O -2.576053 -1.698732 -0.753935
O 1.725754 -2.302368 0.075623
C 0.169598 0.528217 1.707615
O 0.314012 0.912722 2.783422
C -2.482940 1.690871 -1.005165
C -1.133475 1.524564 -0.318815
H -3.307040 1.521956 -0.303793
H -2.618622 1.003702 -1.845833
H -1.006719 2.316701 0.422369
H -0.292421 1.619649 -1.046994
C 3.974117 0.824368 -1.234770
O 2.855750 1.032830 -1.185595
FREQS=   11   25   27   41   48   74   85   94 103 141 224 290 315 338 404 438 492 505 513 530 579
586 651 881 1020 1054 1192 1285 1425 1445 1507 1514 2081 2097 2161 2202 2855 3027 3086 3100 3122
TS025_5
16
E= -1915.20111330 ZPE=   62.38 Gcorr   0.034861000
H 3.609706 -1.205025 1.238947
Co -0.456459 -0.063496 -0.040792
C -1.460853 1.417482 -0.169141
C 1.168004 0.998596 0.026740
O -2.139302 2.295873 -0.461367
O 1.135834 2.179281 0.300768
C 0.217348 -1.180733 -1.223014
O 0.488199 -1.825185 -2.140522
C 2.518888 0.260745 0.058514
C 2.628961 -0.720917 1.236487
H 3.295986 1.028429 0.132084
H 2.646161 -0.273767 -0.889619
H 2.509607 -0.198992 2.190769
H 1.870271 -1.508790 1.181116
C -1.653212 -1.176408 0.749369
O -2.249998 -1.864974 1.447917
FREQS=   43   67   71   86   98 111 165 217 272 300 324 327 380 441 465 481 526 542 554 578 619
804 893 1011 1063 1129 1271 1320 1419 1486 1509 1517 1753 2077 2103 2162 3047 3053 3103 3122 3129
TS026_5
16
E= -1915.18511246 ZPE=   59.42 Gcorr   0.019130000
H -2.413814 -3.006912 -0.774459
Co -0.218755 0.230172 0.134527
C 0.312488 -0.174865 1.768259
C 0.860514 1.626718 -0.099961
O 0.508798 -0.371467 2.885100
O 1.615278 2.465819 -0.319188
C -1.634461 0.929611 -0.653130
O -2.618296 1.432311 -0.977289
C -2.342336 -1.928685 -0.579153
C -0.974681 -1.572452 -0.009757
H -2.559392 -1.410806 -1.518302
H -3.141615 -1.680242 0.127200
H -0.168051 -1.756176 -0.757932
H -0.766248 -2.216539 0.847541
C 3.033969 -1.258930 -0.554674
O 2.922038 -1.260705 -1.686847
FREQS=   13   22   33   37   48   70   83   95 103 141 219 290 315 337 404 438 490 502 511 529 579
586 649 878 1020 1053 1193 1287 1424 1444 1508 1513 2084 2099 2163 2206 2865 3027 3087 3099 3122
TS027_5
16
E= -1915.18349745 ZPE=   59.29 Gcorr   0.017690000
H -0.740926 -0.902384 -2.502863
Co 0.505834 -0.000014 -0.074624
C -0.020017 -1.516122 0.735973
C 2.188428 0.000355 0.347690
O -0.313022 -2.453796 1.339850
O 3.310584 0.000599 0.614886
C -0.020648 1.515980 0.735780
O -0.314041 2.453597 1.339557
C -0.312535 -0.000332 -2.064621
C 1.155514 0.000006 -1.963124
H -0.741329 0.901461 -2.503005

H -0.869329 -0.000364 -1.000580
H 1.663717 0.906622 -2.279242
H 1.664125 -0.906426 -2.279114
C -4.299049 0.000018 0.443155
O -3.531511 -0.000145 -0.397975
FREQS= 10 11 30 31 46 58 88 91 117 121 264 305 405 409 472 480 490 509 525 535 558
569 790 889 965 1096 1122 1169 1346 1479 1523 1755 2071 2078 2088 2143 2202 3100 3143 3172 3221
TS028_5
16
E= -1915.18521593 ZPE= 59.46 Gcorr 0.019592000
H -0.335060 3.000558 -0.152615
Co 0.376747 -0.211411 -0.048993
C -0.029759 0.710056 -1.496572
C 1.812279 -0.916856 0.699787
O -0.112228 1.273125 -2.497033
O 2.836997 -1.318630 1.037303
C -0.371005 -1.743462 -0.562992
O -0.929238 -2.714980 -0.820571
C 0.589840 2.853234 0.413414
C 0.731740 1.435437 0.953780
H 1.424898 3.110823 -0.246694
H 0.594065 3.584268 1.232856
H -0.150500 1.148553 1.572971
H 1.603677 1.387949 1.609949
C -3.126260 0.366511 0.510826
O -3.164314 -0.083711 1.554910
FREQS= 14 24 32 39 47 75 87 95 105 144 226 288 314 336 404 437 490 501 511 531 579
586 646 881 1019 1053 1193 1288 1425 1447 1508 1515 2084 2099 2163 2206 2867 3027 3087 3100 3121
TS029_5
16
E= -1915.18775848 ZPE= 60.30 Gcorr 0.022588000
H 0.770572 2.365483 -0.890238
Co -0.438254 -0.144627 0.000434
C -1.048262 -0.247683 -1.648495
C 0.390237 -1.718714 0.002002
O -1.608401 -0.354820 -2.649813
O 0.919999 -2.739266 0.003043
C -1.042541 -0.241355 1.651902
O -1.599203 -0.344670 2.655554
C 0.154585 2.557603 -0.004694
C -1.139136 1.734885 -0.002018
H 0.772354 2.368519 0.880245
H -0.074484 3.631912 -0.006334
H -1.746670 1.980133 -0.876176
H -1.745032 1.983013 0.872470
C 2.809105 0.353946 -0.003313
O 3.926631 0.556726 -0.004285
FREQS= 10 27 51 56 63 78 96 107 140 156 192 238 312 405 407 465 495 504 535 542 582
588 775 970 985 1005 1242 1262 1418 1505 1509 1511 2076 2096 2160 2223 3020 3070 3085 3095 3126
TS030_5
16
E= -1915.20010502 ZPE= 62.26 Gcorr 0.035311000
H -4.519224 -0.459604 -0.100704
Co 0.684360 -0.003306 -0.154481
C 1.590833 -1.540291 0.092848
C -0.334029 1.504974 -0.288789
O 2.110937 -2.538012 0.290652
O -0.819615 2.522757 -0.480012
C 2.063805 0.950351 0.314927
O 2.993734 1.571080 0.591709
C -2.468119 -0.323359 0.548764
C -3.939903 -0.124802 0.765901
H -2.601427 -1.251971 -1.351206
H -1.798141 -0.074286 1.370819
H -4.301246 -0.677115 1.643124
H -4.186685 0.931432 0.936587
C -1.940720 -0.868757 -0.567667
O -0.647831 -1.051810 -0.842789
FREQS= 40 67 90 98 110 133 171 230 273 299 320 345 400 417 428 487 488 543 594 625 628
803 938 961 1060 1127 1241 1296 1355 1431 1492 1510 1675 2108 2133 2194 3019 3064 3103 3108 3149
TS031_5
16
E= -1915.19836028 ZPE= 62.11 Gcorr 0.033602000
H -2.542812 -1.017248 2.211768
Co 0.442196 0.013552 -0.102626
C 1.516344 -0.017600 1.298056
C 1.116856 -1.491086 -0.826529

O 2.102250 -0.064953 2.285603
O 1.602068 -2.352693 -1.405118
C 0.584670 1.793765 -0.345489
O 0.837001 2.873088 -0.643464
C -2.791512 0.187766 0.433918
C -2.731105 -1.135529 1.137586
H -2.203342 1.538517 -1.083347
H -3.580138 0.876171 0.731080
H -1.948844 -1.772633 0.717634
H -3.686450 -1.667600 1.045575
C -2.015077 0.571206 -0.603278
O -1.048665 -0.177220 -1.164195
FREQS= 31 48 86 88 92 104 148 170 266 303 379 395 406 446 452 470 493 554 581 613 692 803 926 953 1051 1064 1161 1292 1403 1444 1496 1498 1663 2105 2113 2176 3030 3073 3075 3129 3173
TS032_5
16
E= -1915.19747076 ZPE= 61.73 Gcorr 0.033613000
H 2.176695 0.000445 3.061528
Co -0.196607 -0.000011 -0.227443
C -1.457769 1.249344 -0.066652
C 0.917011 -1.255296 -0.829650
O -2.247600 2.062490 0.125557
O 1.662921 -2.071542 -1.144546
C -1.457420 -1.249711 -0.066587
O -2.247015 -2.063086 0.125629
C 1.936571 0.000024 1.988499
C 0.426043 0.000380 1.768687
H 2.421026 0.881970 1.554494
H 2.420524 -0.882599 1.555308
H -0.021506 0.878185 2.242431
H -0.022044 -0.876853 2.242975
C 0.916760 1.255463 -0.829716
O 1.662511 2.071879 -1.144548
FREQS= 52 74 87 99 103 106 111 128 246 275 348 435 439 443 458 461 469 470 537 617 622 624 691 973 986 1045 1216 1276 1426 1489 1511 1513 2100 2100 2108 2177 3011 3062 3077 3092 3122
TS033_5
16
E= -1915.18418189 ZPE= 59.85 Gcorr 0.020743000
H -3.229083 -0.263118 0.476856
Co 0.008815 -0.168991 -0.158530
C -0.111810 1.074782 -1.399706
C 1.463965 -0.963659 -0.797188
O -0.335321 1.785390 -2.277807
O 2.452263 -1.437365 -1.144186
C -0.791187 -1.637245 0.416904
O -1.418405 -2.579347 0.626622
C -2.857012 0.765092 0.424137
C -1.389375 0.870625 0.839353
H -3.014649 1.114970 -0.601573
H -3.494660 1.376271 1.075793
H -1.270880 0.531283 1.877325
H -1.086446 1.924261 0.813728
C 2.191379 1.813192 1.654170
O 1.904210 0.774116 2.021893
FREQS= 18 31 39 43 46 57 77 95 98 127 226 258 307 381 407 451 488 494 526 531 578 589 744 947 988 1037 1231 1267 1430 1476 1515 1516 2079 2099 2164 2195 3015 3029 3064 3090 3113
TS034_5
16
E= -1915.19693850 ZPE= 61.91 Gcorr 0.033569000
H -0.530871 0.349456 2.306696
Co 0.477815 -0.009357 0.081912
C 0.801158 -1.551356 -0.712503
C -0.074848 1.603526 -0.439996
O 0.976928 -2.477181 -1.372162
O -0.327955 2.584090 -0.984757
C 2.183711 0.455957 0.279322
O 3.276251 0.730121 0.502822
C -1.344725 -0.682283 0.526099
C -0.985017 -0.611829 2.015464
H -2.551467 1.147504 0.681127
H -1.544607 -1.700909 0.193297
H -1.880414 -0.707368 2.644045
H -0.309850 -1.418775 2.312735
C -2.474187 0.187502 0.111815
O -3.265267 -0.065327 -0.774746
FREQS= 43 50 75 90 96 110 125 197 253 306 317 390 405 432 475 494 499 528 576 578 599 899 944 1009 1061 1121 1193 1327 1408 1431 1488 1499 1802 2092 2106 2167 2833 2991 3049 3116 3135

TS035_5
16
E= -1915.18375780 ZPE=   59.70 Gcorr   0.020479000
H -3.597065 -0.057438 1.113729
Co 0.192118 0.305684 -0.003138
C 1.036845 1.054935 1.356295
C 1.654970 -0.253388 -0.856392
O 1.558579 1.730194 2.130122
O 2.567131 -0.715207 -1.381685
C -0.657916 0.890642 -1.428624
O -1.126395 1.471774 -2.306870
C -2.788462 0.043093 0.376726
C -1.487958 0.471448 1.059767
H -2.694694 -0.926515 -0.125409
H -3.121304 0.769108 -0.371629
H -1.262116 -0.223680 1.883562
H -1.619412 1.454087 1.524160
C -0.329193 -3.669889 0.467248
O -0.182104 -2.548022 0.334709
FREQS=   17   22   35   42   54   71   88   94 104 120 239 278 317 374 401 447 486 499 505 540 583
588 646 959 983 1036 1233 1278 1421 1457 1508 1515 2075 2095 2159 2195 2997 3022 3075 3088 3114
TS036_5
16
E= -1915.18411447 ZPE=   59.92 Gcorr   0.021305000
H -3.750336 1.301100 -0.486539
Co -0.178422 -0.031010 0.224552
C 0.839269 -0.643909 1.522637
C -0.791056 -1.608711 -0.332601
O 1.395972 -1.019084 2.458476
O -1.155393 -2.606598 -0.764834
C 0.387088 1.644340 0.282571
O 0.767802 2.727367 0.250176
C -2.892264 0.863227 0.041156
C -1.688598 0.729984 -0.899337
H -2.681760 1.511462 0.898911
H -3.221091 -0.102647 0.439722
H -1.442581 1.703163 -1.333521
H -1.936311 0.085189 -1.747659
C 1.890527 -0.443104 -1.733367
O 2.914080 0.034319 -1.583838
FREQS=   14   32   36   42   60   67   97   99 124 147 194 225 282 388 404 434 456 482 519 534 584
602 757 969 982 1016 1238 1262 1425 1502 1510 1512 2097 2102 2168 2189 3021 3062 3082 3091 3111
TS037_5
16
E= -1915.18376581 ZPE=   59.73 Gcorr   0.020988000
H -2.740866 0.783660 0.228865
Co 0.208508 -0.281357 -0.008199
C 1.062060 -0.886483 -1.432191
C 1.658447 0.286638 0.862203
O 1.597892 -1.476373 -2.264016
O 2.558847 0.746576 1.408792
C -0.578096 -1.016808 1.383088
O -0.993698 -1.687310 2.223633
C -2.788529 -0.161900 -0.323276
C -1.481350 -0.475992 -1.054524
H -3.060259 -0.944902 0.391423
H -3.618663 -0.071071 -1.037254
H -1.315745 0.273769 -1.843492
H -1.567507 -1.438695 -1.568610
C -0.613808 3.608875 -0.302036
O -0.272920 2.525597 -0.212051
FREQS=   20   25   36   43   54   71   91   95 104 121 235 279 317 375 401 447 486 500 505 540 582
588 648 961 983 1036 1235 1279 1421 1459 1508 1514 2075 2095 2159 2195 3001 3022 3075 3088 3115
TS038_5
16
E= -1915.17507685 ZPE=   57.56 Gcorr   0.013410000
H -2.669677 -0.719451 -1.809538
Co -0.615150 0.010274 -0.141700
C -0.476410 -0.024065 1.618623
C 0.051475 -1.602454 -0.475305
O -0.380768 -0.046579 2.765121
O 0.556589 -2.607968 -0.727788
C 0.309176 1.498203 -0.437380
O 0.976464 2.410686 -0.666583
C -2.442400 0.174329 -1.236379
C -2.699079 0.184819 0.138484
H -0.786256 0.042978 -1.624453

H -2.516046 1.102741 -1.794639
H -3.080755 -0.708919 0.620160
H -2.925812 1.121960 0.635224
C 3.587217 -0.027246 -0.706456
O 3.673681 -0.048418 0.427954
FREQS= 8 15 24 37 49 62 66 93 93 115 120 309 331 361 428 475 477 494 511 563 569
590 689 814 859 963 1036 1132 1253 1268 1487 1568 2047 2080 2108 2152 2207 3152 3165 3231 3258
TS039_5
16
E= -1915.18271890 ZPE= 59.86 Gcorr 0.021124000
H -2.729025 -1.233360 0.208545
Co 0.075542 0.303558 -0.489167
C 0.112299 1.844703 0.385806
C 1.812736 0.183781 -0.745345
O 0.100166 2.802050 1.018470
O 2.924362 0.174649 -1.045990
C -0.303780 -1.359106 -0.986547
O -0.558160 -2.454259 -1.218542
C -2.814846 -0.151378 0.352737
C -1.918792 0.644715 -0.602071
H -3.870537 0.112898 0.208395
H -2.572562 0.054909 1.400611
H -2.138235 1.708957 -0.474627
H -2.166709 0.409294 -1.645950
C 0.425182 -0.891541 2.307747
O 0.978713 -1.881915 2.400631
FREQS= 13 30 40 48 57 60 99 100 113 139 210 252 278 375 407 444 461 467 521 528 583
604 731 938 1001 1033 1223 1285 1428 1481 1514 1515 2096 2101 2174 2195 3023 3028 3079 3089 3113
TS040_5
16
E= -1915.19400342 ZPE= 61.95 Gcorr 0.033496000
H 3.838870 -0.344430 1.902573
Co -0.686141 -0.003649 -0.004761
C -1.893742 1.007088 0.781271
C 0.134527 1.365926 -0.853053
O -2.627376 1.656991 1.382049
O 0.562539 2.158515 -1.560569
C -1.780712 -1.419898 -0.194595
O -2.449540 -2.306633 -0.471058
C 2.912075 -0.317053 -0.043606
C 4.076284 -0.076561 0.867959
H 1.606485 -1.104550 1.414250
H 3.035489 -0.088257 -1.101237
H 4.952360 -0.663878 0.562790
H 4.388724 0.975738 0.855661
C 1.734523 -0.836478 0.356891
O 0.715147 -1.135653 -0.474758
FREQS= 37 58 75 88 96 122 127 212 272 296 306 371 403 436 438 472 481 535 587 592 636
839 932 977 1059 1129 1195 1285 1371 1428 1489 1505 1666 2112 2120 2183 3021 3041 3067 3109 3164
TS041_5
16
E= -1915.19398336 ZPE= 61.75 Gcorr 0.035094000
H 0.618631 1.945556 2.232844
Co -0.419119 0.059571 0.149715
C -0.943006 1.337705 -0.948469
C 0.257971 -1.504690 -0.403551
O -1.231199 2.088095 -1.775154
O 0.503207 -2.507943 -0.907926
C -2.047779 -0.577681 0.491663
O -3.101224 -0.966226 0.737242
C 1.301829 0.952337 0.390505
C 0.817406 0.952453 1.828219
H 3.452891 0.707117 0.803647
H 1.372383 1.964297 -0.019544
H -0.178785 0.402901 1.936885
H 1.471644 0.397830 2.506708
C 2.635816 0.292709 0.163987
O 2.884971 -0.581816 -0.633784
FREQS= 44 58 88 92 113 137 167 259 291 322 346 386 418 461 494 503 511 525 568 571 648
877 930 973 1065 1119 1136 1330 1402 1413 1469 1554 1822 2087 2104 2161 2580 2851 3080 3085 3153
TS042_5
16
E= -1915.19314738 ZPE= 61.70 Gcorr 0.034432000
H 2.769512 2.366381 -0.400117
Co -0.462586 -0.031814 -0.085268
C -2.023943 -0.782912 -0.493891
C 0.123740 -1.411211 0.868923

O -2.975534 -1.280253 -0.899853
O 0.409275 -2.201132 1.652369
C -1.133219 1.582859 0.205172
O -1.583463 2.564778 0.600943
C 1.361001 0.701568 -0.405211
C 1.831476 2.069317 0.086795
H 3.194585 -0.140584 0.486241
H 1.034643 0.731721 -1.467491
H 1.100951 2.856529 -0.109371
H 2.019857 2.059527 1.165875
C 2.429402 -0.330569 -0.303141
O 2.504665 -1.332005 -0.994055
FREQS= 48 69 86 97 105 117 182 196 252 286 321 387 400 427 472 490 497 520 579 583 596
852 909 985 1088 1126 1151 1277 1423 1432 1506 1515 1778 2092 2110 2169 2871 2904 3032 3093 3139
TS043_5
16
E= -1915.18981109 ZPE= 60.10 Gcorr 0.031705000
H 2.647469 0.879042 -1.100147
Co -0.246446 0.139185 -0.096790
C -0.877872 0.120525 1.587555
C -0.055768 1.919428 -0.388072
O -1.281202 0.120068 2.661392
O 0.196304 3.006457 -0.654256
C -1.674260 -0.510164 -0.940257
O -2.541183 -0.935021 -1.560218
C 2.891807 0.112956 -0.361500
C 1.682962 -0.340938 0.455721
H 3.350107 -0.730223 -0.890540
H 3.644105 0.532222 0.311730
H 0.269909 0.025161 -1.485828
H 1.784307 -0.266116 1.535617
C 0.923718 -1.449308 0.043593
O 0.827911 -2.605638 -0.214387
FREQS= 55 61 67 87 89 106 177 256 293 317 373 401 426 441 464 474 514 525 532 561 630
723 816 880 884 1046 1076 1137 1331 1425 1496 1513 1972 2002 2121 2130 2174 3049 3118 3136 3165
TS044_5
16
E= -1915.19296229 ZPE= 62.09 Gcorr 0.035218000
H 2.097819 1.271888 -1.392357
Co -0.553063 -0.045460 0.132720
C 0.136413 -1.498181 -0.613515
C -2.174947 -0.740774 0.311189
O 0.516385 -2.347525 -1.285620
O -3.190237 -1.199489 0.590296
C -1.133370 1.608734 -0.175852
O -1.517156 2.617762 -0.567895
C 1.917544 1.714686 -0.409311
C 1.288893 0.702534 0.546047
H 2.892404 2.043447 -0.031668
H 1.289095 2.598128 -0.545357
H 0.990892 1.170790 1.500295
H 1.907675 -1.038362 1.751745
C 2.212479 -0.420262 0.872155
O 3.225101 -0.698111 0.256920
FREQS= 43 76 89 101 107 118 172 212 278 280 301 373 393 445 472 478 498 527 584 590 699
888 907 965 1090 1114 1140 1337 1423 1434 1500 1509 1788 2098 2110 2173 2842 2980 3050 3114 3138
TS045_5
16
E= -1915.19130713 ZPE= 62.07 Gcorr 0.035189000
H 0.494340 2.970532 -0.274357
Co -0.437609 -0.076902 -0.032925
C 0.332935 -1.604399 0.478087
C -1.278490 1.332487 0.638553
O 0.668504 -2.567644 1.000862
O -1.825391 2.119936 1.273452
C -1.911856 -0.868984 -0.628741
O -2.805567 -1.322758 -1.190209
C 1.143300 2.252580 -0.781435
C 1.298263 0.953078 0.024833
H 0.721871 2.077596 -1.779402
H 2.113631 2.743944 -0.933750
H 2.632800 0.304688 -1.606900
H 1.545034 1.158621 1.072680
C 2.393310 0.100932 -0.534532
O 3.017827 -0.751182 0.069662
FREQS= 47 77 81 97 106 127 177 195 239 312 334 386 391 455 469 477 492 514 579 586 590
903 950 985 1065 1130 1164 1306 1425 1438 1499 1514 1786 2095 2111 2173 2847 3022 3068 3077 3128

TS046_5
16
E= -1915.18497969 ZPE= 59.72 Gcorr 0.030363000
H 3.695884 0.535076 -0.193070
Co -0.349788 0.014530 -0.115365
C -1.741264 -1.089101 0.107979
C -0.002021 0.223404 1.617291
O -2.617848 -1.823020 0.198573
O 0.250564 0.400674 2.724668
C -1.032502 1.590192 -0.734448
O -1.380019 2.610159 -1.128706
C 1.601562 0.375536 -0.648611
C 2.734415 0.606601 0.330411
H -0.170544 -0.699343 -1.464057
H 1.599500 1.031063 -1.516781
H 2.674983 1.598504 0.787984
H 2.737630 -0.136635 1.131742
C 1.182384 -0.960471 -0.910130
O 1.553728 -2.087555 -1.070274
FREQS= 31 67 72 91 96 110 195 211 282 290 317 408 423 441 447 459 472 521 534 563 590
705 894 898 923 1039 1094 1165 1374 1423 1501 1510 1880 1910 2113 2121 2169 3039 3105 3133 3157
TS047_5
16
E= -1915.18999788 ZPE= 62.46 Gcorr 0.037194000
H -2.743072 -1.123823 -1.352703
Co 0.315833 -0.023651 0.139428
C 1.884903 -0.432892 -0.458118
C 0.754856 1.714652 0.045720
O 2.914497 -0.713189 -0.900361
O 1.052560 2.819477 0.052953
C 0.032831 -1.778715 0.364166
O -0.089357 -2.910648 0.504116
C -1.737386 0.627177 -0.573645
C -2.548502 -0.081894 -1.626800
H -2.282159 -0.722134 1.015726
H -1.554566 1.684538 -0.742658
H -3.516609 0.413756 -1.779142
H -2.036460 -0.087143 -2.593607
C -1.703509 0.183847 0.759027
O -0.939423 0.689404 1.671508
FREQS= 65 83 89 106 108 133 185 223 301 335 340 375 397 432 443 479 511 544 581 599 615
862 954 1022 1050 1134 1240 1330 1364 1429 1491 1509 1568 2091 2124 2178 2980 3027 3092 3118 3185
TS048_5
16
E= -1915.18580462 ZPE= 62.15 Gcorr 0.034038000
H 4.750985 -0.000003 0.906760
Co -0.571231 0.000000 -0.113269
C -2.177328 -0.000010 0.535962
C -0.705118 1.769583 -0.355538
O -3.209504 -0.000017 1.057541
O -0.776908 2.899204 -0.552259
C -0.705099 -1.769584 -0.355542
O -0.776875 -2.899207 -0.552263
C 3.887579 -0.000003 0.235882
C 2.586123 0.000012 1.042529
H 3.951919 0.882227 -0.406383
H 3.951907 -0.882243 -0.406370
H 2.515959 0.868122 1.712790
H 2.515950 -0.868083 1.712810
C 1.325242 0.000009 0.231590
O 1.321803 0.000013 -1.011849
FREQS= 47 64 75 82 96 103 158 182 218 282 306 378 396 445 447 472 507 547 598 627 679
748 936 1015 1055 1107 1267 1358 1423 1444 1507 1512 1617 2076 2103 2159 3037 3062 3063 3137 3140
TS049_5
16
E= -1915.18516905 ZPE= 62.19 Gcorr 0.033429000
H 3.230334 -1.329666 1.019327
Co -0.510626 -0.010445 -0.184154
C -1.894361 -0.421452 0.774261
C -1.064949 1.692669 -0.193980
O -2.759880 -0.692236 1.491826
O -1.417113 2.784938 -0.245857
C -0.317405 -1.744778 -0.589247
O -0.191396 -2.846006 -0.890973
C 3.165373 -0.304286 1.395017
C 2.750795 0.666571 0.283225
H 2.442855 -0.289851 2.215623

H 4.145531 -0.028987 1.793101
H 3.461188 0.669819 -0.553729
H 2.712819 1.690286 0.684939
C 1.371299 0.402403 -0.242214
O 1.084597 0.481259 -1.448680
FREQS= 26 60 72 85 98 103 155 188 202 301 362 382 413 444 465 502 509 529 553 598 629
773 926 1026 1060 1162 1267 1332 1425 1455 1510 1517 1617 2076 2104 2159 3018 3058 3070 3132 3138
TS050_5
16
E= -1915.18567015 ZPE= 62.50 Gcorr 0.034218000
H 3.491050 -1.255419 0.460040
Co -0.321738 -0.053239 0.009666
C -0.241168 -1.394536 -1.178996
C -1.189112 -0.745185 1.439277
O -0.037102 -2.253600 -1.917891
O -1.700440 -1.145750 2.388947
C -1.426152 1.071794 -0.799460
O -2.101474 1.858212 -1.305884
C 2.856580 -0.582056 -0.122438
C 1.636551 -0.168088 0.695211
H 3.460373 0.290311 -0.399517
H 2.586342 -1.102790 -1.045767
H 0.795379 2.864785 -0.006966
H 1.611814 -0.545748 1.714245
C 0.978853 1.047821 0.441288
O 1.470097 2.267114 0.355790
FREQS= 29 63 68 78 81 87 140 253 294 313 401 410 436 460 465 485 505 525 543 570 652
706 846 925 1050 1068 1140 1310 1337 1419 1499 1507 1569 2083 2092 2142 3042 3106 3127 3162 3734
TS051_5
16
E= -1915.17740901 ZPE= 59.37 Gcorr 0.026151000
H 5.615424 0.000114 -0.744328
Co -0.768411 0.000011 -0.171861
C -1.386167 1.559960 -0.681156
C -1.386188 -1.559851 -0.681375
O -1.880932 2.506135 -1.120024
O -1.880965 -2.505957 -1.120379
C -1.375702 -0.000115 1.510572
O -1.844022 -0.000192 2.560541
C 3.513155 0.000090 -0.431864
C 4.793751 -0.000027 -0.036092
H 2.729398 -0.000354 1.612054
H -0.485726 0.000104 -1.639384
H 5.057282 -0.000268 1.019060
H 3.228256 0.000333 -1.480611
C 2.428101 -0.000099 0.546124
O 1.241015 0.000015 0.244385
FREQS= 24 39 45 74 81 102 110 174 211 300 317 340 378 445 479 494 520 548 563 587 610
717 731 937 997 1011 1035 1188 1306 1398 1469 1689 1753 1960 2078 2100 2152 2960 3159 3199 3255
TS052_5
16
E= -1915.17962637 ZPE= 62.53 Gcorr 0.029149000
H 5.030176 -0.885813 -0.044145
Co -0.738745 0.000016 -0.047110
C 0.835218 0.000019 -0.541285
C -2.017543 0.000203 -1.306977
O 2.069386 0.000022 -0.819257
O -2.760285 0.000328 -2.185252
C -1.086762 1.451042 0.935612
O -1.234170 2.396417 1.577136
C 4.462722 0.000217 -0.337040
C 3.069652 -0.000343 0.285589
H 4.380320 0.000770 -1.426377
H 5.029868 0.886139 -0.043224
H 2.861665 -0.890246 0.882204
H 2.861347 0.888884 0.883100
C -1.086893 -1.451215 0.935258
O -1.234385 -2.396730 1.576555
FREQS= 17 24 26 64 70 83 84 149 283 304 313 338 444 477 483 506 525 527 586 627 700
825 855 1035 1082 1165 1265 1369 1438 1504 1505 1520 1584 2091 2098 2152 3074 3095 3142 3152 3162
TS053_5
16
E= -1915.18109349 ZPE= 61.72 Gcorr 0.030624000
H -4.039294 -0.882029 1.388666
Co 0.665160 0.000002 0.100492
C 0.485896 1.736730 -0.220816
C 2.406832 0.000009 0.399620

O 0.343271 2.829362 -0.539561
O 3.539519 0.000014 0.595789
C 0.485910 -1.736732 -0.220801
O 0.343294 -2.829368 -0.539536
C -3.627214 0.000007 0.882934
C -2.118571 0.000010 0.915807
H -4.039300 0.000098 -0.133454
H -4.039290 0.881958 1.388814
H -1.665383 0.000024 1.905678
H -2.647132 -0.000047 -1.500162
C -1.285237 -0.000006 -0.137399
O -1.677912 -0.000029 -1.451553
FREQS= 16 49 55 73 94 97 152 158 261 286 312 347 388 390 451 467 487 493 571 593 621
695 759 942 1034 1042 1127 1290 1336 1428 1486 1513 1685 2105 2113 2185 3021 3078 3082 3152 3739
TS054_5
16
E= -1915.18667834 ZPE= 63.06 Gcorr 0.036241000
H 1.769115 1.735657 1.356916
Co -0.520776 0.111324 -0.010963
C -1.278981 -1.153918 0.904248
C -1.917763 1.245682 -0.150170
O -1.746108 -1.976788 1.561242
O -2.830618 1.892771 -0.394168
C 0.667032 -1.080487 -0.959772
O 0.618324 -2.151778 -1.482086
C 2.647425 0.053059 0.270228
C 2.704171 1.171056 1.333262
H 3.495581 0.134670 -0.423727
H 2.736408 -0.940950 0.720475
H 2.883182 0.758925 2.329309
H 3.502128 1.885619 1.119062
C 1.410868 0.076322 -0.600713
O 0.743156 1.179550 -0.883557
FREQS= 33 43 69 83 95 119 204 263 274 304 393 425 430 469 483 518 534 588 607 638 771
826 1001 1068 1076 1173 1264 1354 1379 1437 1476 1508 1522 1944 2106 2161 3034 3068 3089 3141 3143
TS055_5
16
E= -1915.18596818 ZPE= 63.10 Gcorr 0.036258000
H -4.350711 -0.146071 0.426706
Co 0.641354 -0.100058 0.086002
C 0.963804 1.571973 0.427243
C 2.328247 -0.737853 0.138733
O 1.150771 2.673174 0.708545
O 3.395675 -1.126467 -0.006253
C -0.878542 0.266207 -1.046356
O -1.179890 1.044398 -1.899878
C -2.333396 -0.930797 0.770398
C -3.362235 0.219494 0.713890
H -1.898491 -1.039114 1.770542
H -2.816981 -1.894166 0.562436
H -3.460131 0.709636 1.685275
H -3.084128 0.987067 -0.013109
C -1.205216 -0.855592 -0.234728
O -0.214318 -1.730652 -0.223538
FREQS= 32 39 65 85 97 143 175 265 295 314 404 413 442 468 486 495 531 588 624 644 721
838 1006 1067 1086 1241 1249 1301 1379 1449 1484 1515 1526 1938 2107 2162 3043 3070 3075 3139 3143
TS056_5
16
E= -1915.17993683 ZPE= 59.27 Gcorr 0.030382000
H 2.609451 1.587330 1.060540
Co -0.305482 0.057904 0.311769
C 0.951220 -0.024269 -1.392956
C 0.519020 -1.186292 1.255825
O 1.009828 -0.799446 -2.284068
O 1.041463 -1.953539 1.935833
C -1.544195 -0.998521 -0.420674
O -2.356928 -1.675643 -0.870816
C 2.672294 1.017151 0.129567
C 1.338491 1.034308 -0.599402
H 3.453650 1.463935 -0.496734
H 2.981622 -0.000063 0.381359
H -0.084449 0.673783 1.662777
H 0.992234 2.001490 -0.954070
C -1.292131 1.518158 0.363752
O -1.890950 2.496990 0.458013
FREQS= 42 78 88 104 105 129 175 186 241 262 355 372 418 437 452 468 501 524 527 575 592
625 649 845 882 1041 1070 1138 1356 1423 1497 1510 1919 2033 2101 2111 2162 3041 3111 3138 3175

TS057_5
16
E= -1915.18103388 ZPE=   61.84 Gcorr   0.032043000
H 2.025216 -0.747640 2.184365
Co -0.271053 -0.126900 0.136164
C -0.821991 -1.623181 -0.536166
C -1.828424 0.284542 0.974597
O -1.162754 -2.602111 -1.033030
O -2.816201 0.489884 1.517213
C 0.042505 1.582480 -0.899949
O -0.114325 1.931044 -2.017203
C 2.536825 -0.613158 0.070875
C 2.674934 -0.174433 1.512545
H 1.477743 -1.002586 -1.679586
H 3.461740 -0.935304 -0.413713
H 3.699273 -0.306503 1.869980
H 2.417424 0.883947 1.630672
C 1.406224 -0.640284 -0.654613
O 0.365354 1.761008 0.274036
FREQS=   25   33   54   85   86 116 142 205 228 311 341 400 407 456 475 485 527 554 573 627 643
679 947 974 1048 1064 1208 1238 1353 1421 1500 1508 1633 2111 2125 2163 3039 3096 3103 3129 3148
TS058_5
16
E= -1915.17817869 ZPE=   59.56 Gcorr   0.029637000
H 3.425952 -1.399489 -0.863319
Co -0.308338 -0.023750 -0.128976
C -1.355975 1.316847 -0.695479
C -1.245225 -1.559164 -0.140584
O -2.034595 2.164704 -1.071022
O -1.811614 -2.556523 -0.213666
C -0.199814 0.131750 1.654689
O -0.198141 0.231687 2.799312
C 2.395774 -1.497721 -0.499215
C 1.557656 -0.338781 -1.009792
H 2.001328 -2.455372 -0.851043
H 2.416136 -1.525048 0.592652
H -0.078668 -0.466183 -1.521049
H 1.648780 -0.148106 -2.077463
C 1.428454 0.854607 -0.247410
O 1.972646 1.808908 0.214040
FREQS=   38   62   73   80   94 105 177 178 252 296 312 361 410 440 460 473 479 530 533 540 587
642 750 867 1013 1037 1078 1175 1344 1422 1493 1507 1944 2076 2108 2128 2166 3038 3109 3134 3149
TS059_5
16
E= -1915.18208777 ZPE=   60.91 Gcorr   0.033816000
H 1.005684 -2.415381 -0.807125
Co 0.147838 -0.024715 0.002181
C 0.148729 1.669343 0.601158
C -0.378293 -0.742282 1.549315
O 0.166110 2.752914 0.976778
O -0.736746 -1.246495 2.516179
C 1.952209 -0.178745 -0.273859
O 3.050321 -0.349272 -0.556713
C 0.012929 -1.983020 -0.670518
C -0.483288 0.584160 -1.851964
H -0.507270 -2.007713 -1.630928
H -0.538807 -2.623338 0.019082
H -0.423347 1.648174 -2.060717
H -0.166199 -0.076249 -2.650995
C -1.536862 0.171441 -1.020059
O -2.686464 -0.030095 -0.802823
FREQS=   60   65   89   93   99 133 168 245 296 356 393 404 427 430 444 465 494 501 520 539 554
577 762 864 868 879 990 1024 1306 1436 1479 1490 1990 2117 2121 2169 3059 3146 3152 3157 3245
TS060_5
16
E= -1915.18345101 ZPE=   62.27 Gcorr   0.035402000
H 2.959773 -0.625340 -1.285586
Co -0.345289 0.017246 0.023771
C -1.196983 -0.263539 1.565910
C -0.782069 1.512782 -0.835810
O -1.772303 -0.342656 2.561510
O -1.093034 2.503047 -1.335100
C -1.178487 -1.173698 -1.025004
O -1.610120 -1.997850 -1.702146
C 2.746198 -1.341151 -0.485557
C 1.633083 -0.851165 0.418969
H 2.476124 -2.290625 -0.958108

H 3.675514 -1.516407 0.074904
H 2.998536 1.345298 0.517387
H 1.319067 -1.528538 1.203996
C 1.304389 0.496162 0.497256
O 2.067584 1.571662 0.349611
FREQS= 56 64 80 89 92 123 154 240 291 315 376 381 410 434 467 489 516 535 553 559 578
708 754 926 1041 1054 1130 1299 1383 1416 1494 1509 1560 2086 2094 2146 3017 3088 3120 3214 3713
TS061_5
16
E= -1915.16480990 ZPE= 58.59 Gcorr 0.016805000
H 2.681362 -0.332930 -3.046509
Co 0.046975 -0.131600 0.192931
C -2.012570 0.617334 -0.601208
C -0.290670 -1.818619 -0.356696
O -3.052277 0.180567 -0.290289
O -0.467203 -2.889121 -0.730841
C 0.674827 -0.402610 1.797512
O 1.006217 -0.526519 2.892745
C 2.864364 -0.140756 -1.987348
C -0.893701 1.221589 -1.009334
H 3.552250 -0.889697 -1.590694
H 3.290201 0.855847 -1.858477
H 1.915379 -0.197896 -1.441709
H -0.727525 1.488734 -2.047499
C 0.140468 1.653289 0.115779
O 0.653724 2.716047 0.256325
FREQS= 16 26 30 33 41 64 80 94 114 134 144 238 286 336 368 383 441 480 492 527 562
597 602 689 782 1042 1217 1346 1348 1351 1564 1566 1896 2100 2132 2182 3019 3116 3167 3171 3209
TS062_5
16
E= -1915.18165448 ZPE= 62.24 Gcorr 0.033970000
H 2.527685 -1.246694 -1.007985
Co -0.322180 -0.043524 0.012871
C -1.219826 -0.650847 1.462237
C -1.394529 1.103121 -0.822991
O -1.739825 -0.995879 2.429053
O -2.058976 1.879445 -1.351646
C -0.304966 -1.417299 -1.141399
O -0.133152 -2.303094 -1.857066
C 2.822295 -0.692874 -0.111949
C 1.621656 -0.199431 0.693860
H 3.442154 -1.357345 0.496088
H 3.461089 0.136967 -0.445737
H 2.371920 2.335793 0.519824
H 1.601047 -0.525049 1.731543
C 1.004737 1.033814 0.387858
O 1.446798 2.266100 0.223791
FREQS= 29 66 69 80 87 88 141 256 294 314 403 404 437 459 467 485 500 526 540 566 577
703 850 917 1046 1065 1126 1303 1354 1423 1494 1511 1540 2090 2098 2148 3019 3094 3126 3151 3691
TS063_5
16
E= -1915.17717824 ZPE= 59.13 Gcorr 0.029828000
H 2.844046 -0.208877 -0.934653
Co -0.313858 -0.002817 -0.334739
C 0.743953 -0.174701 1.376459
C 0.642088 1.286736 -1.077779
O 0.747308 0.366862 2.435386
O 1.254177 2.084301 -1.634359
C -1.634529 0.987816 0.376090
O -2.469035 1.615427 0.851810
C 2.770036 -0.814909 -0.029423
C 1.334866 -1.039106 0.446721
H 3.227609 -1.782479 -0.250152
H 3.370414 -0.331028 0.749447
H -0.470743 -0.230164 -1.824873
H 1.041479 -2.076660 0.577646
C -1.185033 -1.525085 -0.532466
O -1.728315 -2.518995 -0.732472
FREQS= 44 62 85 90 101 113 171 230 271 279 350 361 402 432 462 469 498 527 542 560 599
606 629 856 882 1049 1073 1139 1330 1429 1499 1513 1853 1972 2110 2122 2168 3049 3120 3139 3177
TS064_5
16
E= -1915.16978637 ZPE= 59.12 Gcorr 0.023589000
H -1.988357 2.593960 0.148419
Co 0.197361 -0.131506 -0.064344
C 1.192236 1.233450 0.712381
C 0.970325 -1.292985 1.014405

O 2.304825 1.641285 0.870931
O 1.561950 -2.104942 1.577350
C 0.765686 0.360738 -1.660056
O 1.232449 0.543170 -2.696795
C -2.494709 1.637683 0.246254
C -2.509680 0.961159 1.398911
H -3.017298 1.276050 -0.634735
H 0.292836 1.786078 1.143658
H -3.043562 0.020757 1.501895
H -2.015121 1.335758 2.290918
C -1.197462 -1.167856 -0.597616
O -2.088676 -1.811396 -0.926304
FREQS= 14 60 72 81 86 92 96 103 109 192 212 263 320 376 385 431 483 506 507 534 554
558 818 830 973 992 1062 1224 1241 1382 1483 1690 1856 2088 2106 2164 2610 3151 3164 3230 3254
TS065_5
16
E= -1915.18155909 ZPE= 62.89 Gcorr 0.035631000
H -3.627086 1.540126 1.011789
Co 0.789823 0.124816 0.118044
C 2.098579 -0.976072 0.143437
C 1.779978 1.615999 -0.034854
O 2.953463 -1.748858 0.176527
O 2.400990 2.526372 -0.359885
C -0.519044 -1.222954 -0.148340
O -0.758268 -2.388159 -0.395573
C -3.081717 -0.303997 -0.005247
C -3.875139 0.997656 0.095834
H -3.331365 -0.883706 -0.905808
H -3.308586 -0.994143 0.821444
H -3.657999 1.656999 -0.748596
H -4.947732 0.788426 0.100204
C -1.601665 -0.146869 -0.014197
O -1.003486 0.953106 0.118181
FREQS= 30 48 70 81 97 150 180 242 258 317 352 355 408 451 461 550 574 601 616 715 770
794 991 1046 1065 1163 1257 1392 1427 1439 1511 1512 1619 1780 2093 2145 3030 3053 3065 3141 3142
TS066_5
16
E= -1915.17567304 ZPE= 59.58 Gcorr 0.029786000
H -2.338880 -2.659886 -0.302661
Co 0.289584 -0.101815 0.049246
C -1.455546 0.642401 -0.447061
C 0.292335 0.312637 1.795353
O -2.007991 1.696110 -0.399219
O 0.312482 0.559325 2.917608
C 1.051000 1.284440 -0.793415
O 1.592053 2.165301 -1.295199
C -2.583621 -1.598311 -0.209360
C -1.505242 -0.719453 -0.836661
H -3.537467 -1.416154 -0.717720
H -2.718055 -1.373120 0.851876
H -1.322737 -0.903088 -1.893118
H -0.403127 -1.370073 0.331739
C 1.589978 -1.283192 -0.326916
O 2.374465 -2.090712 -0.559613
FREQS= 35 62 72 85 95 99 196 228 257 293 319 347 401 440 460 470 481 526 537 554 560
651 777 884 899 1036 1064 1136 1335 1427 1504 1511 1952 2096 2107 2153 2171 3044 3115 3135 3162
TS067_5
16
E= -1915.16466983 ZPE= 58.54 Gcorr 0.018935000
H -0.142634 0.368970 -2.248383
Co -0.691236 -0.102631 -0.004462
C 0.061003 0.023718 1.591482
C -1.799742 1.261992 0.166109
O 0.639550 0.145970 2.579014
O -2.649734 2.027385 0.286480
C -0.599284 -1.858595 -0.146935
O -0.709841 -3.001392 -0.220206
C -1.107006 -0.067390 -1.934968
C 3.509622 -0.208761 0.135024
H -1.908962 0.595778 -2.262956
H -1.238197 -1.027493 -2.436450
H 4.004548 -1.050958 -0.330213
H 3.537701 -0.075679 1.208609
C 2.880224 0.671550 -0.610444
O 2.312775 1.456203 -1.271755
FREQS= 12 23 41 43 63 80 97 109 119 158 179 307 353 410 436 459 486 490 529 531 533
587 596 607 705 880 991 1175 1254 1422 1437 1502 2088 2097 2168 2225 2987 3118 3153 3201 3297

TS068_5
16
E= -1915.17265900 ZPE= 60.99 Gcorr 0.027130000
H 2.493548 -0.890332 -1.234752
Co -0.507225 0.081703 0.071040
C 0.960480 -0.169433 1.068681
C -0.344949 -1.364770 -0.904983
O 0.391673 -0.209812 2.149395
O -0.097682 -2.170887 -1.700783
C -0.069534 1.651528 -0.587750
O 0.324780 2.552173 -1.199673
C 2.941769 -0.147927 -0.569618
C 2.449461 -0.317128 0.865581
H 4.027588 -0.271133 -0.610101
H 2.700505 0.844388 -0.959135
H 2.922562 0.407174 1.541263
H 2.707238 -1.309341 1.259567
C -3.368088 -0.544738 0.399828
O -2.690172 0.374536 0.307893
FREQS= 28 36 49 61 81 84 92 117 167 196 240 284 298 357 406 453 484 535 551 561 573
766 951 1064 1081 1146 1281 1337 1439 1453 1515 1519 1728 2038 2099 2127 3039 3065 3078 3136 3146
TS069_5
16
E= -1915.17557953 ZPE= 61.49 Gcorr 0.030453000
H 2.867811 1.865771 1.661796
Co -0.464516 0.027635 0.013367
C -2.116447 0.568066 0.376583
C 0.016416 1.442734 -0.916280
O -3.157981 0.954553 0.672054
O 0.302712 2.281829 -1.650703
C -0.806953 -1.726856 0.100372
O -1.049400 -2.843744 0.014033
C 2.354543 0.007764 0.688532
C 2.128968 1.058619 1.737415
H 2.059238 -1.051698 -1.881004
H 3.351767 -0.432141 0.631659
H 1.131298 1.506410 1.661118
H 2.218013 0.642515 2.749017
C 1.423498 -0.462898 -0.152797
O 1.768876 -1.467834 -1.058688
FREQS= 15 41 67 88 96 100 136 182 261 272 292 342 382 414 445 467 483 491 532 571 588
597 832 931 1060 1089 1135 1220 1284 1423 1486 1501 1681 2091 2110 2176 3021 3076 3090 3133 3811
TS070_5
16
E= -1915.17051217 ZPE= 59.07 Gcorr 0.025760000
H 4.627543 0.033987 -1.271977
Co -0.891296 -0.033568 0.200140
C -0.512954 0.246096 -1.510749
C -1.290773 -1.735193 0.104572
O -0.367768 0.411237 -2.640563
O -1.703885 -2.812436 0.077416
C -1.888371 1.381036 0.483741
O -2.668961 2.205578 0.686360
C 4.335850 0.512517 -0.331602
C 3.397958 -0.381316 0.452162
H 5.245649 0.711197 0.244100
H 3.871311 1.471568 -0.570447
H 3.720504 -1.370248 0.767486
H -1.475419 -0.307022 1.551536
C 2.179052 -0.048487 0.791207
O 1.084466 0.260486 1.119228
FREQS= 31 45 52 82 87 104 108 143 177 218 322 339 351 456 485 494 499 517 562 571 584
636 669 742 904 1056 1081 1156 1413 1428 1495 1519 1966 2081 2099 2156 2199 3050 3112 3142 3190
TS071_5
16
E= -1915.16224904 ZPE= 58.50 Gcorr 0.018111000
H 1.438266 -0.537623 -2.575562
Co 0.806107 -0.006018 -0.048252
C 0.357848 -0.255726 1.647167
C 1.520014 -1.604869 -0.310436
O -0.001963 -0.425198 2.725376
O 2.140827 -2.556176 -0.483739
C 1.118235 1.727540 0.066325
O 1.486020 2.815568 0.120155
C 0.902299 0.228577 -2.013205
C -2.765307 0.983236 -0.292488
H 1.233769 1.201590 -2.379957

H -0.174925 0.127586 -2.228834
H -3.185522 1.966478 -0.456467
H -1.703489 0.864548 -0.116363
C -3.546070 -0.074856 -0.308065
O -4.236774 -1.019630 -0.321267
FREQS= 13 18 41 54 63 75 82 100 112 146 165 309 354 410 446 458 483 487 528 530 541
585 590 604 707 882 994 1176 1264 1421 1436 1502 2094 2109 2174 2238 2991 3116 3152 3187 3285
TS072_5
16
E= -1915.16217239 ZPE= 58.54 Gcorr 0.018422000
H 0.920171 -2.744810 0.207492
Co 0.840189 -0.103151 -0.083105
C 1.103780 1.622130 0.211120
C 2.215461 -0.689341 0.867297
O 1.194430 2.739777 0.464793
O 3.183508 -1.109488 1.321847
C 0.135897 0.061198 -1.694536
O -0.177780 0.097723 -2.799480
C 0.212830 -1.981884 -0.122238
C -2.665565 0.892177 0.804017
H -0.240710 -2.331451 -1.051238
H -0.579151 -1.901830 0.641982
H -1.653695 0.604882 0.547966
H -2.881073 1.889497 1.163082
C -3.639452 0.018621 0.670793
O -4.503700 -0.761588 0.552320
FREQS= 14 16 37 51 66 81 97 109 125 142 164 309 356 410 446 459 482 485 527 531 543
584 594 604 703 879 996 1176 1265 1420 1436 1501 2096 2107 2174 2238 2988 3115 3151 3189 3288
TS073_5
16
E= -1915.17073924 ZPE= 58.72 Gcorr 0.027750000
H 1.548358 -2.192396 -1.937872
Co -0.347196 -0.084485 -0.119657
C 1.835528 0.579953 -0.061783
C -0.726029 1.654496 -0.184022
O 2.338048 1.225719 0.772512
O -1.060729 2.758041 -0.190557
C -0.102711 -0.757995 1.533399
O -0.090076 -1.126574 2.624818
C 2.142112 -1.574230 -1.261842
C 1.502818 -0.192494 -1.134045
H 2.184805 -2.083742 -0.296734
H 3.160112 -1.495551 -1.659417
H -0.692469 -1.202541 -1.117682
H 1.384537 0.376028 -2.054099
C -1.926530 -0.660786 -0.501940
O -3.007515 -1.033983 -0.712031
FREQS= 41 54 85 94 105 108 136 174 213 247 298 335 342 398 437 477 491 499 512 561 594
610 702 841 893 1050 1072 1133 1351 1424 1501 1509 1881 2057 2083 2110 2158 3050 3127 3151 3169
TS074_5
16
E= -1915.17399369 ZPE= 61.11 Gcorr 0.031105000
H 2.998500 -1.278028 0.276633
Co -0.422843 -0.102497 -0.086007
C 1.104616 0.818382 0.091079
C -1.426710 1.336091 -0.129621
O 1.598915 1.684604 0.749067
O -2.102761 2.259695 0.008318
C -0.292577 -0.985541 1.468380
O -0.361290 -1.377639 2.548390
C 3.165359 -0.530968 -0.504683
C 1.837387 0.021384 -1.033811
H 3.761250 0.280204 -0.079951
H 3.732767 -1.001241 -1.312679
H 1.229211 -0.793630 -1.451920
H 1.995510 0.737750 -1.846563
C -1.349390 -0.946353 -1.797097
O -1.701436 -1.748611 -1.034378
FREQS= 41 65 71 86 95 119 124 169 187 238 254 296 345 367 395 439 474 511 518 571 672
742 829 979 1060 1093 1273 1309 1420 1456 1507 1515 1872 1976 2085 2136 3023 3058 3091 3133 3148
TS075_5
16
E= -1915.16153257 ZPE= 58.35 Gcorr 0.018897000
H 3.164612 -0.263897 -1.647054
Co -0.732412 0.233129 -0.230649
C -1.172810 0.176603 1.476300
C 0.127123 1.760756 -0.264807

O -1.515529 0.203975 2.573378
O 0.592386 2.806764 -0.386882
C -2.087211 -0.658055 -0.913363
O -3.021773 -1.082940 -1.431122
C 3.463386 0.137753 -0.675975
C 2.750433 -0.567389 0.458469
H 4.548036 0.019389 -0.582919
H 3.238399 1.209713 -0.673088
H -0.476721 0.401865 -1.705012
H 2.968749 -0.307000 1.490216
C 1.844009 -1.505846 0.286378
O 1.042722 -2.354985 0.137548
FREQS= 10 25 27 50 59 70 86 97 159 207 217 308 338 445 482 485 489 506 551 565 583
589 640 759 904 1059 1086 1158 1416 1430 1496 1522 1942 2089 2112 2178 2198 3044 3104 3136 3195
TS076_5
16
E= -1915.17569262 ZPE= 61.78 Gcorr 0.033161000
H 3.494753 -1.618666 0.128567
Co -0.113624 -0.363596 0.154616
C -1.872692 -0.869191 0.250167
C 0.264245 0.628124 1.584249
O -2.938981 -1.281403 0.313942
O 0.566674 1.175089 2.548907
C -0.470649 1.199835 -0.839601
O -1.516618 1.460639 -1.385327
C 1.543820 -0.994775 -0.425418
C 3.004443 -0.756896 -0.339311
H 0.327905 -2.552373 -1.549778
H 0.348844 2.725976 -1.564105
H 3.429077 -0.645860 -1.343340
H 3.248193 0.136100 0.239328
C 0.586394 -1.717711 -0.916763
O 0.624641 2.000125 -0.973178
FREQS= 31 55 74 76 91 100 110 247 276 350 361 412 424 453 458 496 510 522 568 624 678
704 750 871 985 1013 1084 1092 1313 1407 1482 1497 1780 1791 2112 2160 3042 3103 3139 3288 3688
TS077_5
16
E= -1915.16216492 ZPE= 58.57 Gcorr 0.019659000
H 0.457819 -2.645457 -0.683535
Co 0.881750 -0.116962 0.023327
C 0.690640 1.441534 0.844801
C 0.112097 -1.107589 1.265471
O 0.510905 2.470753 1.323136
O -0.230040 -1.813449 2.105953
C 2.400307 0.266224 -0.800982
O 3.462027 0.399538 -1.220311
C 0.785151 -1.712956 -1.146522
C -2.553755 0.764803 -1.050934
H 1.666319 -1.935287 -1.750606
H -0.015190 -1.363547 -1.820951
H -2.514932 1.558476 -1.784696
H -1.643979 0.349819 -0.635696
C -3.717033 0.297973 -0.652549
O -4.750610 -0.120086 -0.297537
FREQS= 15 34 39 59 70 81 91 104 114 146 166 309 355 410 448 458 483 487 528 529 542
583 588 599 708 881 999 1177 1265 1423 1436 1501 2094 2108 2174 2239 2990 3116 3151 3187 3287
TS078_5
16
E= -1915.16722421 ZPE= 58.89 Gcorr 0.025018000
H -4.891044 -0.907666 -0.426465
Co 0.655183 -0.018358 -0.132352
C 0.360919 1.585534 -0.781096
C 1.716313 0.453275 1.223897
O 0.241844 2.589778 -1.334760
O 2.449545 0.773041 2.046684
C 1.583034 -1.383757 -0.742921
O 2.214822 -2.186414 -1.274356
C -4.308193 -0.275113 0.251440
C -2.897458 -0.092245 -0.272254
H -4.816346 0.691516 0.333279
H -4.314580 -0.743637 1.238219
H -0.074963 -0.360160 -1.399139
H -2.728107 0.358391 -1.243034
C -1.841260 -0.467934 0.408876
O -0.974340 -0.859072 1.130308
FREQS= 22 45 62 71 82 96 104 141 153 261 323 338 363 444 479 485 488 510 556 576 589
600 675 754 905 1051 1075 1143 1395 1425 1496 1517 1952 2088 2114 2138 2170 3045 3106 3138 3223

TS079_5
16
E= -1915.16686025 ZPE= 58.79 Gcorr 0.024791000
H -4.108679 -0.630771 -1.116149
Co 0.526002 -0.010397 -0.109180
C -0.084261 1.552701 -0.618619
C 1.785408 0.579091 1.016377
O -0.433895 2.542021 -1.097315
O 2.625126 0.976349 1.689267
C 1.478705 -1.198975 -0.996956
O 2.080388 -1.879355 -1.704207
C -3.472232 0.055688 -0.547323
C -2.896426 -0.634283 0.663238
H -2.686534 0.417960 -1.210742
H -4.088438 0.910298 -0.248341
H -0.419144 -0.431560 -1.195506
H -3.574394 -1.036822 1.415924
C -1.636738 -0.843611 0.964289
O -0.568069 -1.140521 1.414334
FREQS= 16 52 56 76 86 97 108 136 151 289 320 338 370 442 462 476 487 510 551 567 573
585 678 752 887 1062 1065 1163 1408 1428 1494 1518 1956 2087 2114 2125 2170 3048 3102 3150 3164
TS080_5
16
E= -1915.16942195 ZPE= 60.73 Gcorr 0.027500000
H 3.701324 -0.616601 -0.253022
Co -0.314405 0.044045 -0.038679
C 1.310196 0.701624 -0.003457
C -1.154407 1.487050 -0.599135
O 2.046018 1.515596 0.463994
O -1.696073 2.491934 -0.774967
C -0.435191 -0.385340 1.665346
O -0.542132 -0.436883 2.815037
C 2.858938 -1.265731 -0.504637
C 1.696812 -0.422648 -1.036038
H 3.187870 -1.989507 -1.256155
H 2.568199 -1.812551 0.396692
H 0.836995 -1.072778 -1.293619
H 1.968147 0.097425 -1.959271
C -2.312623 -2.191287 -0.435127
O -1.752304 -1.487799 -1.143064
FREQS= 24 47 65 79 82 86 97 130 147 200 248 271 314 359 380 454 491 514 538 568 662
698 820 985 1060 1094 1273 1318 1420 1454 1506 1514 1893 2063 2121 2140 2941 3056 3094 3131 3144
TS081_5
16
E= -1915.17087974 ZPE= 59.83 Gcorr 0.029070000
H -1.671296 -0.883740 1.602930
Co 0.314422 0.000012 0.087492
C 1.650975 0.001070 -1.069256
C 0.627999 1.636080 0.664895
O 2.567234 0.001691 -1.768115
O 0.863109 2.656481 1.151016
C 0.629589 -1.636197 0.663607
O 0.865690 -2.656708 1.149019
C -1.024861 -0.001446 1.641820
C -1.195296 0.000026 -1.660128
H -1.671407 0.880847 1.604815
H -0.523503 -0.002442 2.611976
H -0.970237 -0.923163 -2.186644
H -0.970476 0.923412 -2.186396
C -2.378903 -0.000188 -1.026913
O -3.363471 -0.000375 -0.418562
FREQS= 41 59 74 92 100 110 141 152 180 213 335 403 403 415 455 483 483 504 522 529 547
580 586 772 815 822 1041 1127 1282 1410 1470 1485 2067 2081 2140 2231 3040 3116 3141 3157 3242
TS082_5
16
E= -1915.16156640 ZPE= 58.39 Gcorr 0.019780000
H -4.641506 0.076215 -0.069602
Co 0.802489 0.235033 -0.225950
C -0.164632 1.696801 -0.286460
C 0.995556 0.070660 1.520972
O -0.694409 2.712280 -0.403227
O 1.176070 0.033607 2.655421
C 2.301683 -0.514210 -0.755834
O 3.329380 -0.840783 -1.155747
C -3.578076 0.231880 0.139917
C -2.726707 -0.684634 -0.712799
H -3.343773 1.282783 -0.061622

H -3.418380 0.050717 1.205585
H 0.751680 0.502441 -1.706597
H -2.804701 -0.656563 -1.795773
C -1.848304 -1.534476 -0.225702
O -1.071995 -2.304805 0.209565
FREQS= 14 23 35 53 60 71 87 97 163 209 216 308 338 442 482 486 489 506 553 564 583
589 641 763 904 1058 1086 1158 1416 1429 1497 1522 1942 2089 2113 2178 2199 3044 3104 3134 3198
TS083_5
16
E= -1915.17410016 ZPE= 62.06 Gcorr 0.032800000
H 4.585835 -0.540672 0.714233
Co -0.742494 -0.092335 -0.321383
C 0.112470 1.357124 0.193389
C -2.338335 0.518005 -0.122938
O 0.312630 2.398201 0.734395
O -3.391498 0.940150 0.082203
C -0.980205 -1.804284 0.208599
O -1.141840 -2.784794 0.788234
C 3.743841 -0.501389 0.018507
C 2.559468 0.216819 0.664100
H 4.070684 0.017870 -0.886157
H 3.480237 -1.524060 -0.262800
H 2.811117 1.236839 0.981801
H 2.224129 -0.289000 1.581777
C 1.354301 0.320800 -0.222967
O 1.241468 -0.184853 -1.340289
FREQS= 30 42 64 79 92 118 171 199 219 254 293 337 394 433 442 480 526 576 590 667 699
784 952 1015 1063 1108 1263 1370 1429 1451 1508 1512 1682 1891 2087 2140 3031 3065 3073 3141 3142
TS084_5
16
E= -1915.16501220 ZPE= 60.03 Gcorr 0.024288000
H 3.543431 0.226111 -0.307479
Co -0.209666 -0.078335 0.029828
C -1.436227 -1.283818 -0.288041
C -0.039814 -0.027157 1.821668
O -2.203038 -2.125940 -0.469308
O 0.206245 -0.326969 2.908588
C 1.067132 -0.751910 -0.875619
O 1.808156 -1.532396 -1.357670
C 1.541244 0.956897 -0.688401
C 2.878540 1.021455 0.040016
H 0.851313 1.715042 -0.297626
H 1.647095 1.150690 -1.757474
H 3.371550 1.981237 -0.149638
H 2.751948 0.911216 1.120409
C -2.546139 2.461230 -0.735582
O -1.722958 1.719126 -0.463834
FREQS= 29 38 46 66 74 81 103 106 131 136 209 262 305 357 372 406 420 487 509 545 608
615 791 973 1027 1066 1238 1286 1425 1459 1504 1517 1960 2070 2128 2169 3047 3049 3115 3122 3141
TS085_5
16
E= -1915.17310094 ZPE= 60.12 Gcorr 0.032903000
H -0.207412 -2.300262 -1.261324
Co 0.249809 0.010138 -0.080776
C 1.849950 -0.686512 -0.568926
C 0.686012 1.768770 -0.078847
O 2.784288 -1.243490 -0.932043
O 0.919399 2.886997 -0.129780
C 0.024175 -0.214953 1.685628
O -0.095186 -0.400759 2.812864
C -2.006857 -1.256324 -0.527345
C -0.588935 -1.826062 -0.357009
H -2.543135 -1.599592 -1.423707
H -2.658993 -1.429929 0.337302
H -0.344732 0.228149 -1.457928
H -0.481510 -2.519032 0.477236
C -1.681847 0.212934 -0.600502
O -2.384007 1.177229 -0.727117
FREQS= 66 68 84 90 98 102 139 276 343 387 412 426 450 468 486 498 527 536 553 571 619
761 868 949 985 1010 1090 1172 1227 1284 1439 1499 1847 1912 2118 2135 2180 3025 3086 3105 3166
TS086_5
16
E= -1915.17318549 ZPE= 62.22 Gcorr 0.033058000
H 2.060223 0.058586 2.037825
Co -0.696279 0.023300 -0.387373
C -2.182721 -0.632252 0.180214
C -0.933883 1.771094 0.008333

O -3.147465 -1.074896 0.630504
O -1.038089 2.801513 0.509402
C 0.332891 -1.319534 0.096689
O 0.697401 -2.279015 0.700206
C 2.743169 -0.028154 -0.034576
C 2.630810 0.668214 1.332114
H 3.335378 0.556301 -0.745794
H 3.224946 -1.005798 0.094896
H 3.628409 0.824546 1.750114
H 2.144647 1.644050 1.243847
C 1.401180 -0.286033 -0.655916
O 1.045308 0.084048 -1.775483
FREQS= 29 37 58 83 94 118 156 208 221 280 321 345 398 438 472 487 525 568 580 608 699
800 933 1032 1059 1166 1276 1331 1424 1468 1511 1515 1679 1888 2086 2138 3048 3060 3107 3137 3142
TS087_5
16
E= -1915.15962984 ZPE= 58.44 Gcorr 0.019984000
H 2.260095 -3.086531 0.532532
Co -0.773977 0.133432 -0.259593
C -1.913467 -1.198618 -0.344070
C -0.776791 0.425741 1.479453
O -2.759195 -1.965493 -0.483937
O -0.874637 0.661661 2.600171
C -0.365525 1.754859 -0.821273
O -0.254273 2.812936 -1.252964
C 2.691699 -1.204527 -0.466585
C 1.749624 -2.317597 -0.058560
H 3.474237 -1.385675 -1.197000
H 1.321985 -2.801004 -0.942516
H 0.918378 -1.931152 0.539132
H -0.945786 -0.022954 -1.747270
C 2.644751 0.008816 0.039884
O 2.598944 1.092473 0.493110
FREQS= 12 30 39 52 55 69 87 98 166 205 231 302 342 442 481 488 502 506 545 568 583
586 644 766 907 1055 1087 1157 1418 1427 1490 1522 1948 2099 2117 2184 2214 3036 3100 3115 3202
TS088_5
16
E= -1915.17356375 ZPE= 62.19 Gcorr 0.034076000
H -4.230636 0.012439 -0.893790
Co 0.446230 -0.009845 0.000001
C 1.415558 -0.488761 1.447278
C 1.415250 -0.488149 -1.447684
O 1.951422 -0.863179 2.394740
O 1.950921 -0.862121 -2.395431
C 0.568549 1.760526 0.000355
O 0.595157 2.910856 0.000607
C -0.881811 -1.542158 -0.000073
C -3.675772 -0.278938 -0.000030
H -0.966321 -2.128157 0.913876
H -0.966461 -2.128014 -0.914102
H -3.477382 -1.351964 0.000892
H -4.231660 0.013821 0.892631
C -1.296133 -0.183643 0.000080
O -2.428699 0.461249 0.000197
FREQS= 33 65 77 78 84 90 161 241 276 315 346 411 426 453 473 479 486 534 547 552 751
791 920 961 992 1115 1162 1197 1419 1469 1497 1506 1575 2091 2099 2150 3066 3108 3161 3169 3180
TS089_5
16
E= -1915.14753208 ZPE= 55.91 Gcorr 0.008130000
H 1.691586 -2.491214 -1.280687
Co -0.902408 0.322478 0.201506
C 2.888350 0.960481 0.349783
C -0.607091 -0.208425 -1.456075
O 4.014463 0.897523 0.491627
O -0.442629 -0.528842 -2.547857
C -0.453850 2.017204 0.047250
O -0.322537 3.156793 -0.025354
C 2.004956 -2.707154 -0.262791
C 1.381183 -2.180612 0.787734
H -1.253310 0.849655 1.566914
H 2.851029 -3.379023 -0.148119
H 1.680709 -2.388801 1.810830
H 0.527009 -1.514834 0.659984
C -2.200532 -0.766473 0.665179
O -3.150561 -1.334576 0.977074
FREQS= 10 29 35 40 43 54 64 66 81 96 98 120 155 222 312 341 448 483 488 504 569
581 588 764 836 971 983 1067 1241 1380 1473 1706 1944 2099 2113 2181 2216 3099 3155 3211 3243

TS090_5
16
E= -1915.16526157 ZPE=   59.26 Gcorr   0.026017000
H -0.702683 0.499816 -1.677065
Co -0.698291 0.075589 -0.234731
C -1.389797 1.681057 -0.110172
C -0.930880 -1.482909 -1.029269
O -1.965018 2.678921 -0.124884
O -1.201286 -2.440145 -1.604034
C -0.896300 -0.457059 1.449398
O -1.109106 -0.808673 2.521324
C 2.125336 0.654395 -0.359386
C 2.794757 1.811971 -0.385544
H 3.724175 -0.449762 0.647287
H 1.145136 0.551308 -0.847439
H 3.780879 1.899898 0.065012
H 2.387226 2.704093 -0.849325
C 2.677028 -0.546701 0.288113
O 2.055191 -1.581451 0.442645
FREQS=   21   40   46   65   91   97 105 164 220 310 320 341 356 459 482 487 506 575 584 585 622
668 770 925 1003 1029 1069 1178 1314 1394 1463 1693 1788 1956 2095 2121 2179 2924 2997 3157 3251
TS091_5
16
E= -1915.16152635 ZPE=   58.55 Gcorr   0.022762000
H 0.651475 0.662328 -1.098226
Co -1.000404 -0.077931 -0.191491
C -2.056524 1.324332 -0.216886
C -0.909667 -1.757229 -0.702467
O -2.855899 2.147595 -0.299879
O -0.998853 -2.837989 -1.085283
C -0.866946 -0.305505 1.561396
O -0.882132 -0.488817 2.695564
C 2.507180 1.041479 0.022279
C 1.678556 1.063867 -1.237638
H 2.128869 0.463304 -2.031480
H 2.262158 1.691240 0.856758
H -1.293618 0.046079 -1.659159
H 1.557083 2.085129 -1.612969
C 3.565281 0.272117 0.181671
O 4.511591 -0.405576 0.322749
FREQS=   18   28   44   62   87   97 100 143 192 236 307 311 335 454 485 487 508 514 548 576 580
618 646 755 904 1042 1085 1161 1412 1435 1473 1496 1954 2097 2116 2177 2228 2839 3073 3136 3201
TS092_5
16
E= -1915.16218293 ZPE=   58.72 Gcorr   0.023682000
H 0.049170 0.465762 -1.536290
Co -0.607053 0.151671 -0.223709
C -1.543923 -0.178180 1.251441
C -1.125845 -1.222170 -1.202790
O -2.200321 -0.373056 2.172173
O -1.540842 -1.997287 -1.943435
C -0.720186 1.897577 -0.262454
O -0.895547 3.022992 -0.439613
C 3.557614 0.831683 -0.239896
C 3.085227 -0.397061 0.000472
H 3.001680 1.717299 0.059301
H 4.505905 0.997564 -0.740128
H 3.625038 -1.295253 -0.288134
H 1.274038 0.338616 0.996517
C 1.805735 -0.600672 0.693821
O 1.334568 -1.690922 0.974040
FREQS=   22   33   51   59   70   94   98 130 153 278 317 323 344 453 480 485 509 569 573 583 593
620 761 929 984 1004 1031 1179 1302 1381 1462 1694 1767 1960 2090 2120 2177 2793 3159 3196 3253
TS093_5
16
E= -1915.16034250 ZPE=   58.85 Gcorr   0.022855000
H -0.685222 -0.413855 1.033820
Co 1.018527 0.032564 0.205833
C 2.072615 -1.348423 0.475673
C 1.040377 0.060381 -1.572190
O 2.864579 -2.148195 0.714447
O 1.149339 0.112525 -2.714252
C 1.016856 1.762675 0.498515
O 1.153651 2.877990 0.747975
C -1.579092 -0.904449 0.599110
C -2.644121 0.150188 0.377285
H -1.307190 -1.434810 -0.318269

H -1.866166 -1.655734 1.341990
H 1.205880 0.086805 1.692365
H -2.418173 1.202956 0.509232
C -3.883802 -0.126715 0.028529
O -4.988364 -0.370635 -0.280442
FREQS= 12 22 55 70 87 97 101 106 228 289 319 336 349 459 477 486 490 508 542 571 579
649 665 755 912 1056 1086 1167 1391 1412 1506 1532 1965 2096 2117 2176 2227 2896 3070 3102 3209
TS094_5
16
E= -1915.15882211 ZPE= 60.02 Gcorr 0.021518000
H 0.401989 2.704321 0.637965
Co 0.167299 -0.018446 -0.013326
C -0.205798 -1.164137 -1.293410
C 1.890470 -0.141106 0.074673
O -0.436017 -2.062853 -1.982986
O 2.877500 -0.773371 0.257289
C -0.275159 -0.503324 1.614486
O -0.564893 -1.004468 2.615323
C 0.581461 2.285900 -0.352096
C 1.867254 1.430861 -0.401722
H -0.340271 1.761648 -0.698789
H 0.676366 3.114142 -1.060250
H 2.604305 1.870689 0.272920
H 2.290007 1.412418 -1.408159
C -4.051827 0.394286 -0.106037
O -3.000073 0.818184 -0.214532
FREQS= 20 24 43 47 54 68 72 93 102 118 272 279 322 339 354 447 478 521 543 559 594
714 791 982 1027 1068 1246 1273 1429 1466 1478 1546 1917 2061 2121 2197 2838 3083 3090 3143 3163
TS095_5
16
E= -1915.16831216 ZPE= 59.88 Gcorr 0.031132000
H 1.718615 -1.601945 -0.671837
Co -0.443870 -0.038212 0.010148
C -1.116793 -1.460504 0.827923
C -0.349879 1.497751 0.977416
O -1.544996 -2.354390 1.415357
O -0.443947 2.394499 1.690446
C -1.885384 0.292020 -0.978049
O -2.784910 0.518211 -1.651048
C 2.802953 0.252690 -0.257798
C 3.938366 -0.346165 0.100247
H 0.939260 -0.467505 0.601612
H 2.717745 1.334555 -0.329051
H 4.017206 -1.429328 0.158306
H 4.832961 0.220894 0.335701
C 1.569494 -0.508739 -0.634019
O 0.774623 0.018274 -1.527636
FREQS= 49 66 69 82 99 124 178 199 280 320 336 383 413 450 465 470 504 539 561 589 646
701 903 948 971 1024 1160 1261 1301 1342 1419 1478 1716 1894 2094 2117 2169 2980 3155 3187 3246
TS096_5
16
E= -1915.16614970 ZPE= 60.18 Gcorr 0.029076000
H 1.518623 2.437510 -0.630522
Co 0.137400 0.188944 -0.084441
C 1.797258 -0.410163 -0.192472
C -0.424638 -0.832987 -1.420207
O 2.893606 -0.758760 -0.234510
O -0.779484 -1.482352 -2.302387
C -1.141109 1.388345 -0.319856
O -1.941791 2.204352 -0.454135
C 1.205749 1.961142 0.299263
C -0.128163 -0.152957 1.994279
H 2.089789 1.747771 0.903343
H 0.585646 2.659518 0.865074
H 0.802223 -0.437641 2.473869
H -0.698444 0.644688 2.458975
C -0.838493 -1.147398 1.378916
O -1.526237 -2.086893 1.212235
FREQS= 10 79 92 98 99 110 127 160 168 291 378 398 424 426 439 458 501 512 520 569 580
599 662 791 835 851 982 1072 1267 1422 1485 1486 2075 2092 2103 2158 3061 3144 3157 3166 3249
TS097_5
16
E= -1915.16658336 ZPE= 61.16 Gcorr 0.029752000
H 3.757760 0.008188 -2.189145
Co -0.472602 -0.000028 -0.001293
C 1.313105 -0.001509 0.336503
C -0.722548 1.561994 0.759445

O 2.041576 -0.005169 1.289972
O -0.947521 2.458249 1.453613
C -0.723025 -1.567155 0.748623
O -0.948239 -2.468128 1.436580
C 3.390620 0.004324 -1.158637
C 1.857763 0.003874 -1.143429
H 3.781248 0.884031 -0.642534
H 3.781678 -0.878967 -0.649019
H 1.476827 -0.877654 -1.673006
H 1.476356 0.888916 -1.666775
C -2.830943 0.008025 -2.180641
O -2.048748 0.004915 -1.344638
FREQS= 37 50 68 71 82 96 135 181 194 214 216 278 316 372 384 450 472 527 537 579 674 793 814 980 1059 1087 1281 1311 1419 1476 1504 1511 1842 2057 2112 2131 3039 3063 3079 3139 3153
TS098_5
16
E= -1915.16927196 ZPE= 60.86 Gcorr 0.032738000
H 0.189678 2.382609 -1.233076
Co -0.272678 0.085510 -0.001468
C -1.358734 -0.365806 -1.379864
C 0.036498 -1.621384 0.450393
O -2.515581 -0.603510 -1.527787
O 0.201693 -2.679669 0.862545
C -1.601583 0.746780 0.966733
O -2.382629 1.170200 1.695114
C 1.559337 0.778119 -0.689900
C 0.653427 1.828930 -0.422163
H 2.532369 0.928486 1.264780
H 1.743811 0.445147 -1.709245
H -0.613487 -0.354748 -2.224024
H 0.785552 2.412857 0.488978
C 2.639224 0.449500 0.261215
O 3.590939 -0.264515 0.011847
FREQS= 47 63 70 79 100 126 171 191 255 326 370 388 420 436 468 480 495 507 592 599 758 800 890 975 992 1021 1157 1188 1229 1360 1445 1528 1796 1871 2108 2152 2754 2851 3126 3170 3216
TS099_5
16
E= -1915.16848907 ZPE= 60.60 Gcorr 0.032323000
H 0.877263 -2.458683 -1.236905
Co -0.000013 -0.167540 -0.049490
C 0.000134 1.774107 -0.486443
C -0.000002 -0.072701 1.742975
O 0.000228 2.807326 0.102226
O 0.000001 -0.001292 2.888319
C 1.742427 -0.434351 -0.240404
O 2.859744 -0.648062 -0.401049
C -0.000176 -2.219544 -0.635198
C 0.000062 1.065112 -1.705670
H -0.877486 -2.458462 -1.237182
H -0.000390 -2.837346 0.263614
H 0.918050 1.062227 -2.285104
H -0.917944 1.062366 -2.285076
C -1.742488 -0.434111 -0.240393
O -2.859834 -0.647673 -0.401037
FREQS= 54 63 64 94 108 125 146 160 276 366 367 419 423 438 469 471 492 526 554 554 575 601 753 791 844 864 962 1021 1255 1432 1481 1490 1970 2110 2121 2166 3071 3147 3160 3173 3237
TS100_5
16
E= -1915.16272229 ZPE= 59.24 Gcorr 0.026674000
H -0.482243 2.601090 0.097657
Co 0.343650 0.073894 0.041121
C 0.384052 -1.674640 0.429076
C 2.051322 0.188743 -0.435603
O 0.434430 -2.786820 0.706301
O 3.179386 0.345690 -0.597336
C -0.507226 0.574147 1.491022
O -0.996762 0.962795 2.460597
C 0.290462 2.038867 -0.430445
C -3.086690 -0.048828 -0.636666
H 0.051872 2.024607 -1.498964
H 1.232686 2.571311 -0.289308
H -3.953139 -0.202637 -1.271001
H -3.214309 0.217288 0.402245
C -1.898159 -0.184410 -1.177435
O -0.906553 -0.342923 -1.818384
FREQS= 43 51 57 79 96 97 120 161 187 195 245 336 381 402 411 447 488 512 522 526 532 570 574 650 801 879 963 1147 1283 1421 1465 1493 2082 2108 2132 2162 3049 3140 3148 3188 3295

TS101_5
16
E= -1915.17012272 ZPE= 62.14 Gcorr 0.034103000
H 1.526038 1.560990 1.347576
Co -0.390109 -0.058292 -0.016444
C -0.119603 -1.495426 -0.998513
C -1.567595 -0.754486 1.144289
O -0.125528 -2.351159 -1.774212
O -2.210235 -1.163523 2.005364
C -1.275472 1.416293 -0.482403
O -1.933877 2.232073 -0.958946
C 2.983221 -0.417446 1.019973
C 2.317175 -0.250505 -0.134378
H 2.934129 0.287648 1.846340
H 3.642179 -1.265497 1.167806
H 2.453436 -0.986087 -0.923852
H 1.445990 1.219716 -1.455867
C 1.340711 0.816061 -0.449438
O 1.327210 1.891380 0.460893
FREQS= 35 67 70 87 96 101 144 215 297 325 336 364 387 464 481 495 509 529 559 567 624
699 892 901 1013 1029 1083 1140 1245 1323 1414 1467 1678 2067 2090 2148 3140 3155 3177 3246 3808
TS102_5
16
E= -1915.16616383 ZPE= 61.11 Gcorr 0.030180000
H 1.525866 1.450475 1.836794
Co -0.371555 0.076939 0.027114
C 1.262944 -0.788084 -0.115389
C -1.038324 -1.274732 0.925707
O 1.523043 -1.905954 -0.472522
O -1.551144 -2.219784 1.340419
C -0.481718 0.267125 -1.747043
O -0.720306 0.184315 -2.870258
C 2.048552 1.551514 0.881547
C 2.406478 0.192448 0.276422
H 1.434347 2.155417 0.204610
H 2.960694 2.124726 1.071589
H 3.039343 -0.383471 0.960148
H 2.981461 0.322800 -0.648021
C -1.255177 1.496134 1.324470
O -1.697374 1.889706 0.323427
FREQS= 33 40 61 90 97 106 120 156 191 264 281 322 341 376 398 447 463 512 526 563 574
764 864 1037 1058 1102 1282 1319 1429 1458 1517 1518 1816 1966 2081 2136 3046 3054 3099 3115 3129
TS001_6
16
E= -1803.08280358 ZPE= 67.51 Gcorr 0.042232000
H -2.448802 -0.064734 -1.959432
Co 0.407816 0.011372 -0.212158
C 1.103267 -1.589919 -0.396332
C 0.205817 0.029416 1.549408
O 1.695919 -2.562930 -0.565118
O 0.158849 0.041978 2.697552
C 0.965000 1.656791 -0.458986
O 1.469246 2.670754 -0.668830
C -3.503138 0.040885 -0.041633
C -2.279912 -0.278990 -0.900937
H -3.787087 1.094160 -0.126706
H -4.362044 -0.561896 -0.353209
H -3.316040 -0.171252 1.015565
H -1.436799 0.379169 -0.591083
H 0.795945 -0.000577 -1.659775
H -1.994535 -1.329429 -0.815021
FREQS= 20 50 64 78 92 96 136 176 304 327 344 353 452 487 489 511 568 581 622 760 817
861 1011 1205 1241 1378 1431 1501 1512 1515 1517 1972 2092 2112 2172 2843 3051 3093 3117 3123 3146
TS002_6
16
E= -1803.08197531 ZPE= 67.61 Gcorr 0.042497000
H -3.988354 -1.487710 -0.196253
Co 0.265354 -0.000062 -0.173925
C 1.286490 -1.332515 -0.689155
C -0.040415 1.635176 -0.736416
O 2.014232 -2.099429 -1.146460
O -0.134289 2.672914 -1.226950
C 1.098818 0.380125 1.349355
O 1.731844 0.662674 2.266108
C -3.463446 -0.589292 0.142355
C -2.082893 -0.938176 0.699594
H -4.085222 -0.105262 0.901676

H -3.386354 0.091750 -0.710414
H -1.574601 -0.030260 1.059511
H -2.134690 -1.621880 1.551586
H -1.474201 -1.435078 -0.068490
H -0.206763 -0.231058 -1.577631
FREQS= 30 44 60 82 93 101 109 162 275 329 344 347 461 486 493 511 566 578 653 757 834 837 1011 1218 1226 1399 1433 1507 1516 1517 1541 1963 2090 2109 2170 2993 3040 3053 3105 3125 3130
TS003_6
16
E= -1803.08190790 ZPE= 67.64 Gcorr 0.042854000
H -3.853381 -1.263031 0.777422
Co 0.338128 0.029851 -0.220017
C 0.462275 0.190329 1.545558
C 0.306444 1.738768 -0.623159
O 0.661664 0.341278 2.667524
O 0.439670 2.835774 -0.947943
C 1.448450 -1.310899 -0.451721
O 2.270190 -2.084174 -0.681356
C -3.415016 -0.488543 0.140666
C -2.130522 -0.982200 -0.525672
H -3.228784 0.390006 0.766032
H -4.162674 -0.209565 -0.608086
H -2.296304 -1.859184 -1.157735
H -1.704092 -0.208824 -1.178804
H 0.505255 0.005037 -1.709158
H -1.391458 -1.288171 0.230969
FREQS= 31 40 73 87 93 105 109 166 280 328 342 351 461 486 491 510 566 577 653 752 828 835 1010 1218 1220 1402 1434 1512 1517 1518 1541 1963 2091 2110 2171 2994 3043 3052 3104 3125 3128
TS004_6
16
E= -1803.06299378 ZPE= 67.59 Gcorr 0.043748000
H -1.500063 0.711757 0.260979
Co 0.197143 0.055589 -0.148803
C -0.137568 -1.296293 0.959490
C 0.891629 1.520998 0.600243
O -0.216004 -2.152077 1.727276
O 1.454947 2.356060 1.159357
C 1.540951 -0.569258 -0.988247
O 2.556543 -1.011425 -1.372501
C -3.408832 -0.018171 -0.448966
C -2.142191 0.814382 -0.646859
H -3.178386 -1.085672 -0.392554
H -3.930128 0.262711 0.470739
H -4.099208 0.131682 -1.284473
H -1.632831 0.538129 -1.574203
H 0.537076 -0.196077 -1.723427
H -2.347136 1.886166 -0.706403
FREQS= 24 59 66 93 109 131 150 202 263 296 353 362 411 480 500 504 546 565 597 819 871 1014 1097 1192 1244 1368 1431 1500 1513 1516 1606 1815 2014 2085 2141 2816 3056 3086 3118 3129 3140
TS005_6
16
E= -1803.05035683 ZPE= 63.75 Gcorr 0.035461000
H 2.722483 -0.852988 -0.949296
Co -0.236371 0.005478 0.238840
C -0.021790 -1.652947 -0.317014
C 0.111307 1.672376 -0.234061
O 0.191103 -2.661515 -0.830269
O 0.397582 2.682399 -0.706560
C -1.993375 0.086084 -0.018549
O -3.135214 0.134925 -0.139208
C 2.792914 -0.027692 -0.232886
C 1.690025 -0.094495 0.823483
H 2.758757 0.903902 -0.807026
H 3.785371 -0.086715 0.232239
H 1.795133 -1.021840 1.398804
H 1.808724 0.731635 1.536198
H -0.717544 -0.658634 2.390030
H -0.873177 -0.309691 3.032815
FREQS= 41 50 81 90 97 106 128 168 195 209 236 255 313 382 408 452 491 502 525 531 577 587 743 953 986 1038 1234 1264 1430 1476 1514 1515 2080 2101 2164 3023 3030 3069 3090 3113 4348
TS006_6
16
E= -1803.05048413 ZPE= 64.26 Gcorr 0.037790000
H -2.251283 0.512459 1.972001
Co 0.142840 0.009817 0.099476
C 0.846687 1.535809 -0.429185
C -0.663326 -1.402385 -0.591862

O 1.267999 2.485649 -0.925881
O -1.142669 -2.243078 -1.216023
C 1.686971 -0.808138 0.439749
O 2.682042 -1.337868 0.662436
C -2.622471 0.388125 0.949834
C -1.643781 0.925722 -0.099930
H -3.576534 0.929405 0.892798
H -2.845327 -0.675180 0.811116
H -2.056405 0.781692 -1.103482
H -1.534899 2.007566 0.027464
H 0.036010 0.361868 2.765741
H 0.288308 0.744716 2.172607
FREQS= 38 58 85 94 105 121 146 213 243 265 280 298 326 395 408 462 487 512 523 537 581 587 726 963 993 1024 1240 1280 1424 1484 1507 1514 2082 2101 2164 3024 3054 3087 3092 3117 4308
TS007_6
16
E= -1803.05625327 ZPE= 66.31 Gcorr 0.044387000
H 2.714823 0.245969 1.769146
Co -0.172069 0.021115 0.265174
C 0.516963 -1.386966 -0.555575
C -0.747417 1.576503 -0.357935
O 0.960087 -2.230532 -1.204040
O -1.069577 2.558587 -0.866587
C -1.804513 -0.689811 0.396262
O -2.857955 -1.147456 0.425425
C 2.841314 0.170498 0.682462
C 1.700375 0.840097 -0.086120
H 2.944070 -0.892063 0.440901
H 3.797369 0.653323 0.439833
H 1.658975 1.900051 0.182633
H 1.898034 0.798917 -1.160262
H -0.195866 -0.011474 2.020787
H 0.527676 0.228466 1.834333
FREQS= 49 74 92 104 109 126 251 256 289 373 379 435 440 475 506 508 524 539 575 584 716 790 978 993 1041 1227 1244 1282 1428 1485 1507 1510 2084 2102 2158 3023 3061 3078 3104 3124 3765
TS008_6
16
E= -1803.05655526 ZPE= 66.28 Gcorr 0.044705000
H 2.020801 -0.878755 1.324297
Co -0.266399 -0.000011 0.413800
C 0.038096 -1.634892 -0.192138
C 0.038231 1.634872 -0.192058
O 0.297809 -2.653663 -0.663592
O 0.298008 2.653620 -0.663528
C -1.988047 0.000095 -0.046433
O -3.078992 0.000160 -0.407656
C 2.662361 0.000005 -0.541213
C 1.791335 -0.000069 0.712349
H 2.485933 0.881759 -1.164567
H 3.726173 0.000336 -0.268884
H 2.486434 -0.882017 -1.164329
H 2.020800 0.878565 1.324377
H -0.858045 -0.000525 2.071242
H -0.075765 -0.000070 2.140434
FREQS= 46 90 101 104 110 137 251 252 283 364 380 432 433 477 496 502 523 542 569 586 735 779 976 987 1050 1213 1242 1273 1432 1486 1511 1514 2083 2102 2158 3029 3046 3090 3096 3126 3754
TS009_6
16
E= -1803.05667426 ZPE= 66.83 Gcorr 0.045885000
H -1.869413 0.874117 1.497072
Co 0.282922 -0.000002 0.398903
C -0.064743 1.577014 -0.340437
C -0.064724 -1.577022 -0.340436
O -0.354210 2.563108 -0.858661
O -0.354175 -2.563120 -0.858662
C 2.041196 0.000015 0.044562
O 3.156622 0.000022 -0.224601
C -2.750755 -0.000002 -0.291687
C -1.725711 -0.000006 0.856529
H -3.399391 0.881217 -0.248720
H -2.289140 0.000000 -1.283469
H -3.399391 -0.881222 -0.248726
H -1.869415 -0.874133 1.497067
H 0.495195 0.411091 1.990298
H 0.495196 -0.411097 1.990298
FREQS= 49 82 91 94 100 142 258 274 366 373 433 454 469 500 513 530 553 561 603 633 735 936 1002 1012 1041 1224 1226 1429 1492 1508 1513 1520 2097 2110 2164 3045 3066 3097 3110 3123 3221

```
TS010_6
16
E= -1803.05602522 ZPE=    66.73 Gcorr    0.045341000
H 2.496807 -0.892389 1.221992
Co -0.176825 0.018632 0.265999
C 0.522699 -1.271567 -0.732958
C -0.756666 1.549023 -0.424885
O 0.994669 -2.037754 -1.450576
O -1.076285 2.528149 -0.938044
C -1.797471 -0.727140 0.457375
O -2.831795 -1.213540 0.557829
C 2.825505 0.057863 0.789237
C 1.693567 0.860726 0.115282
H 3.620830 -0.184990 0.076525
H 3.282971 0.628957 1.604522
H 1.612043 1.846231 0.580828
H 1.924743 1.035891 -0.937313
H 0.251349 -0.367059 1.821939
H -0.032999 0.402011 1.871565
FREQS=   50   74   88   92  101  121  259  274  364  370  443  450  471  482  501  528  557  562  589  636  727
932  998 1016 1035 1227 1237 1425 1491 1503 1514 1517 2098 2111 2164 3040 3072 3100 3101 3131 3230
TS011_6
16
E= -1803.05449269 ZPE=    66.24 Gcorr    0.044496000
H 1.991471 -0.732916 1.501823
Co -0.267397 0.005826 0.389808
C 0.062345 -1.610080 -0.253341
C 0.012636 1.627034 -0.264207
O 0.323829 -2.614426 -0.753914
O 0.269056 2.627194 -0.775862
C -2.010315 -0.028858 0.007611
O -3.122841 -0.046491 -0.277647
C 2.713703 -0.037255 -0.419301
C 1.778004 0.069757 0.781254
H 2.546493 0.770077 -1.139237
H 3.761328 0.025697 -0.096376
H 2.595255 -0.983487 -0.955298
H 1.961275 1.012368 1.307542
H -0.002290 -0.156895 2.009118
H -0.732395 0.054049 2.194889
FREQS=   49   87   97   99  113  136  245  260  280  369  378  408  429  468  480  499  521  550  570  584  673
737  982  991 1052 1249 1279 1348 1430 1488 1511 1514 2083 2103 2159 3017 3030 3079 3095 3124 3772
TS012_6
16
E= -1803.05212516 ZPE=    65.99 Gcorr    0.043986000
H -2.880642 -1.058646 -0.355677
Co 0.191352 -0.003622 -0.248519
C -0.480805 -1.081026 1.027609
C 0.670477 1.562707 0.497996
O -0.971083 -1.775086 1.797311
O 0.937982 2.597596 0.911889
C 1.810570 -0.743725 -0.481681
O 2.817651 -1.230762 -0.726763
C -2.754279 -0.169185 -0.981220
C -1.702689 0.788065 -0.429679
H -3.731096 0.331159 -1.034846
H -2.504046 -0.511848 -1.989883
H -1.615862 1.658791 -1.082973
H -2.014472 1.154991 0.553440
H -0.235953 -0.812018 -1.397873
H 0.279517 0.460366 -1.639806
FREQS=   64   67   82   95   97  124  249  265  286  379  402  443  453  488  502  514  528  550  620  724  796
856  988  994 1059 1250 1282 1428 1490 1509 1513 2081 2088 2140 2158 2183 3023 3060 3091 3108 3131
TS013_6
16
E= -1803.05201568 ZPE=    65.97 Gcorr    0.044270000
H 2.746258 -0.882078 -0.603695
Co -0.291406 -0.000008 0.353308
C 0.125382 -1.493128 -0.561731
C 0.125351 1.493135 -0.561708
O 0.404337 -2.485701 -1.063655
O 0.404265 2.485710 -1.063650
C -2.073931 -0.000005 0.129594
O -3.218195 -0.000019 0.084726
C 2.767896 0.000042 0.044906
C 1.642517 -0.000004 1.075525
H 2.746248 0.882211 -0.603626
```

H 3.741963 0.000028 0.552494
H 1.727988 -0.877522 1.719715
H 1.727963 0.877480 1.719765
H -0.534495 -0.688968 1.628559
H -0.534505 0.688913 1.628577
FREQS= 62 72 91 97 114 115 253 259 269 382 403 440 469 493 499 515 524 551 616 720 795
846 989 996 1051 1241 1278 1431 1487 1510 1511 2079 2079 2138 2154 2182 3026 3077 3092 3105 3139
TS014_6
16
E= -1803.05036978 ZPE= 69.32 Gcorr 0.047291000
H 2.787334 -0.888189 -1.236327
Co -0.096629 0.000002 -0.034638
C -0.628583 1.555046 -0.648244
C -0.630123 -1.554654 -0.647889
O -1.121880 2.451404 -1.188506
O -1.124311 -2.450630 -1.187968
C -1.282623 0.000713 1.334976
O -0.678245 0.000461 2.387448
C 2.329305 -0.001373 -0.790922
C 2.509047 -0.000608 0.731502
H 2.788595 0.884291 -1.237329
H 3.578303 -0.001155 0.964166
H 2.082310 -0.889884 1.205223
H 2.083430 0.889769 1.204154
H 1.293881 -0.000811 -1.212781
H -2.391527 0.001297 1.283799
FREQS= 49 57 84 90 102 114 154 175 224 272 280 315 404 472 517 538 540 677 817 892 900
991 1198 1224 1308 1427 1429 1470 1509 1514 1593 1751 2049 2114 2806 2914 3052 3086 3117 3120 3147
TS015_6
16
E= -1803.04509503 ZPE= 65.32 Gcorr 0.042727000
H 0.581505 2.901061 0.263584
Co 0.028932 -0.270451 -0.417220
C -1.719138 -0.015479 -0.502323
C 1.686029 0.341897 -0.501029
O -2.847321 0.186113 -0.566418
O 2.748101 0.772605 -0.564633
C 0.190133 -1.807461 0.521328
O 0.295582 -2.813249 1.060765
C -0.267997 2.527930 0.844714
C -0.112537 1.056076 1.208422
H -1.171427 2.713925 0.254921
H -0.338373 3.145393 1.751152
H 0.783090 0.901415 1.816390
H -0.957490 0.716918 1.813956
H -0.020526 0.208432 -1.810968
H 0.112220 -1.066513 -1.688471
FREQS= 60 66 83 88 103 132 240 258 302 327 395 438 459 490 497 515 541 572 625 666 727
792 970 980 1055 1227 1266 1429 1492 1508 1511 1965 2074 2120 2142 2178 3020 3064 3088 3101 3123
TS016_6
16
E= -1803.04874650 ZPE= 66.21 Gcorr 0.046398000
H -0.057602 1.230680 -1.127489
Co -0.218745 -0.032762 -0.380266
C -1.874061 0.581236 -0.365241
C 1.386652 -0.228938 -1.081184
O -2.927953 1.025562 -0.432569
O 2.397580 -0.309570 -1.614018
C -0.362399 -1.519224 0.623317
O -0.453248 -2.457700 1.271913
C 1.842182 1.320287 1.453897
C 0.343371 1.098415 1.261589
H 2.295984 1.842207 0.604669
H 2.031085 1.933129 2.346284
H -0.070991 0.625178 2.156867
H -0.157667 2.064764 1.162206
H 2.387937 0.379928 1.591006
H -0.668144 -0.768315 -1.623239
FREQS= 63 85 94 103 105 251 281 349 389 420 438 455 474 498 506 548 557 611 654 695 715
759 986 990 1049 1248 1283 1423 1488 1510 1514 1874 2042 2140 2155 2193 3018 3059 3079 3095 3119
TS017_6
16
E= -1803.04395852 ZPE= 65.37 Gcorr 0.042885000
H 2.220623 0.098465 1.686203
Co -0.193152 0.070019 -0.528229
C -1.844137 0.582858 -0.157867
C 1.502593 0.416269 -0.901466

O -2.909434 0.942148 0.073374
O 2.588594 0.682051 -1.160650
C -0.187052 -1.660449 -0.004435
O -0.207661 -2.776743 0.254777
C 1.248128 0.237347 2.169626
C 0.262735 1.013089 1.299623
H 1.429361 0.773548 3.111285
H 0.869680 -0.755666 2.434483
H -0.669371 1.157086 1.851108
H 0.641069 2.011589 1.069207
H -0.413059 0.886228 -1.739161
H -0.528798 -0.376101 -1.923827
FREQS= 52 73 83 93 104 127 248 265 299 327 399 437 449 489 501 510 544 577 621 679 720 790 967 999 1047 1229 1281 1427 1491 1509 1511 1960 2061 2120 2140 2178 3022 3073 3089 3104 3131
TS018_6
16
E= -1803.04453101 ZPE= 65.86 Gcorr 0.043707000
H 1.268935 2.951546 1.349452
Co -0.030055 -0.216651 -0.361528
C -1.714426 0.262954 -0.579607
C 1.718140 -0.129346 -0.589296
O -2.784882 0.618497 -0.781638
O 2.839574 -0.023132 -0.799386
C -0.199097 -1.718315 0.612628
O -0.310372 -2.708752 1.175462
C 0.333463 2.574553 0.921017
C 0.124342 1.085452 1.252817
H 0.376293 2.779474 -0.152455
H -0.476543 3.191683 1.325635
H 0.944732 0.711501 1.870059
H -0.781927 0.949564 1.847835
H 0.087876 0.845061 -1.379427
H -0.136966 -1.123949 -1.560704
FREQS= 41 57 88 89 102 120 263 350 369 415 428 448 486 497 504 550 562 628 645 692 704 763 996 1003 1034 1211 1227 1422 1491 1503 1521 1892 2052 2138 2155 2192 3039 3072 3092 3107 3123
TS019_6
16
E= -1803.04400924 ZPE= 66.13 Gcorr 0.043486000
H 2.834886 -1.315346 -1.232135
Co -0.236226 0.022769 -0.220472
C -0.743409 1.639824 0.371453
C -1.669637 -1.005337 -0.468743
O -1.073437 2.705111 0.643030
O -2.585022 -1.647587 -0.728233
C 0.455818 -0.736982 1.250454
O 0.903658 -1.236702 2.180392
C 2.884175 -0.344784 -0.727453
C 1.663471 0.525828 -1.020602
H 3.794454 0.161564 -1.072369
H 3.008136 -0.538639 0.342483
H 1.787077 1.497346 -0.538542
H 1.578656 0.715760 -2.090463
H 0.588934 -0.745042 -1.146210
H -0.718149 0.571722 -1.502192
FREQS= 37 69 75 94 103 117 244 263 308 389 414 439 460 477 494 518 540 558 643 741 755 823 993 1019 1064 1245 1283 1431 1495 1509 1514 2045 2113 2134 2174 2199 3035 3085 3102 3113 3151
TS020_6
16
E= -1803.04166376 ZPE= 65.31 Gcorr 0.041896000
H -0.837051 -0.396623 -1.311258
Co 0.182563 -0.028694 -0.229597
C -0.423820 1.246038 0.879947
C 1.721244 0.736450 -0.617602
O -0.801648 2.032433 1.632330
O 2.715445 1.239004 -0.901523
C 0.853899 -1.531067 0.497843
O 1.289164 -2.464112 1.013464
C -1.825396 -0.913152 -0.339720
C -2.999032 -0.026460 -0.750507
H -1.857970 -1.127972 0.726031
H -1.841251 -1.876077 -0.858381
H -3.946213 -0.538962 -0.544717
H -3.003896 0.916301 -0.196742
H -2.981097 0.218327 -1.817939
H -0.046775 0.050296 -1.771785
FREQS= 55 69 72 89 93 128 227 233 288 300 376 417 437 458 489 504 536 563 579 659 742 968 994 1042 1066 1228 1274 1432 1487 1506 1512 1884 2087 2101 2108 2161 3040 3086 3103 3127 3166

TS021_6
16
E= -1803.04038746 ZPE= 65.48 Gcorr 0.042179000
H -2.593280 0.205155 1.428723
Co 0.253239 -0.290839 -0.093342
C 1.957183 -0.351004 0.365118
C -1.117019 -1.334969 -0.470996
O 3.065861 -0.458483 0.646161
O -1.958096 -2.076239 -0.721867
C 0.100139 1.131913 -1.213306
O -0.001103 2.036524 -1.910201
C -1.982121 1.113861 1.427124
C -0.496453 0.800742 1.563119
H -2.321451 1.731721 2.269168
H -2.208441 1.658381 0.505411
H 0.114435 1.704328 1.519009
H -0.308000 0.373148 2.552858
H 0.023148 -0.805805 1.245943
H 0.832470 -1.191941 -1.139990
FREQS= 37 70 74 92 101 109 247 265 326 329 395 437 465 487 503 508 513 562 625 648 734
778 971 1020 1069 1236 1292 1429 1498 1509 1511 1934 2117 2121 2170 2208 3027 3063 3091 3107 3127
TS022_6
16
E= -1803.04104208 ZPE= 66.01 Gcorr 0.044656000
H -3.449869 0.881847 -0.308828
Co 0.245602 0.000009 0.073176
C -0.034857 -1.737481 -0.077354
C -0.034850 1.737498 -0.077359
O -0.259481 -2.853353 -0.209771
O -0.259480 2.853369 -0.209778
C 2.035129 -0.000021 -0.047580
O 3.179565 -0.000020 -0.040409
C -2.804178 -0.000035 -0.382262
C -1.734602 0.000016 0.720621
H -2.378962 -0.000048 -1.389031
H -3.449823 -0.881947 -0.308791
H -1.834901 0.866553 1.375159
H -1.834878 -0.866481 1.375214
H 0.528380 -0.000012 1.554489
H -0.055877 0.000014 -1.410695
FREQS= 44 58 95 99 108 161 267 286 381 401 448 464 507 518 539 550 567 606 638 683 753
761 1006 1009 1038 1218 1228 1423 1496 1503 1522 1821 1990 2136 2149 2193 3045 3099 3101 3124 3143
TS023_6
16
E= -1803.03823407 ZPE= 66.55 Gcorr 0.044225000
H 2.544202 0.814224 0.196628
Co 0.003462 -0.016677 0.188410
C 0.076073 1.247010 -1.110323
C -1.554964 -0.790207 -0.242398
O 0.165794 2.091409 -1.881832
O -2.566130 -1.287447 -0.454162
C 1.073500 -1.388105 -0.222867
O 1.805500 -2.248267 -0.429153
C -0.953954 1.040552 1.724180
C 1.720633 0.825862 0.914644
H 1.510933 1.862844 1.185184
H -1.010335 2.038511 1.287004
H -1.964694 0.693749 1.941307
H 2.034544 0.291178 1.813101
H -0.217445 -0.429610 1.571201
H -0.399727 1.123141 2.660264
FREQS= 61 76 94 100 121 130 143 163 256 364 420 441 446 463 477 496 519 524 542 557 724
833 858 921 966 1275 1311 1476 1480 1495 1501 2105 2123 2164 2181 3055 3069 3145 3153 3154 3167
TS024_6
16
E= -1803.03317373 ZPE= 67.31 Gcorr 0.046243000
H -3.950955 0.139873 -0.358751
Co 0.643961 -0.212567 0.379279
C -1.275710 0.132467 0.366865
C 2.263051 -0.785270 -0.055962
O -1.907214 0.772746 1.176283
O 3.219326 -1.227777 -0.521707
C 0.792095 1.349730 -0.334553
O 0.975714 2.441863 -0.657453
C -3.446944 -0.792044 -0.623041
C -1.951776 -0.545112 -0.836835
H -3.905562 -1.198391 -1.529055

H -3.616281 -1.501516 0.192454
H -1.781867 0.126268 -1.690522
H -1.421110 -1.474684 -1.083672
H -0.052643 -1.153245 1.525246
H 0.754574 -1.252286 1.627949
FREQS= 49 55 92 103 115 196 232 257 279 335 363 385 452 492 503 576 586 596 687 780 880 992 1041 1074 1097 1271 1340 1418 1464 1506 1514 1677 1783 2084 2136 3025 3056 3060 3127 3149 3256
TS025_6
16
E= -1803.03114720 ZPE= 67.15 Gcorr 0.046217000
H 2.391089 -2.569619 -1.241216
Co -0.359147 -0.004565 0.333615
C -1.879480 -0.973503 0.141324
C 1.431653 0.495909 0.269042
O -2.812920 -1.614897 -0.052706
O 2.169298 1.223060 0.877273
C -0.837857 1.483053 -0.445597
O -1.228509 2.495628 -0.836921
C 1.830264 -1.964739 -0.522375
C 1.972079 -0.468335 -0.852759
H 2.220967 -2.184824 0.475490
H 0.788895 -2.302914 -0.557958
H 3.026631 -0.193563 -0.952332
H 1.480285 -0.236983 -1.803564
H -0.302535 0.067991 1.894058
H -0.031284 -0.721478 1.738929
FREQS= 44 60 83 94 142 197 224 233 330 345 400 454 462 496 529 544 562 630 659 760 837 990 1049 1088 1114 1275 1302 1428 1484 1504 1516 1725 1831 2087 2140 2904 3043 3058 3100 3118 3128
TS026_6
16
E= -1803.02774856 ZPE= 66.16 Gcorr 0.045480000
H 0.116803 -1.211255 1.371403
Co -0.339132 -0.062440 -0.233873
C 1.529792 0.085073 -0.560799
C -2.080945 -0.596955 -0.152316
O 2.203440 0.660999 -1.371399
O -3.178290 -0.895779 -0.296670
C -0.374783 1.506206 0.606757
O -0.440126 2.588410 0.989091
C 1.056024 -1.751541 1.119036
C 2.120205 -0.794641 0.557736
H 0.826788 -2.562369 0.425922
H 1.360706 -2.199283 2.069358
H 2.462755 -0.102798 1.337297
H 3.001187 -1.325519 0.182164
H -0.227241 -0.844924 -1.491361
H -0.566378 0.414148 -1.570885
FREQS= 67 74 89 96 109 193 267 299 322 347 357 437 461 488 515 547 577 610 726 762 794 896 1017 1040 1110 1257 1309 1428 1461 1488 1546 1841 2025 2116 2154 2181 2876 3050 3091 3106 3150
TS027_6
16
E= -1803.02232231 ZPE= 66.23 Gcorr 0.044763000
H -2.246289 -0.709710 1.261189
Co 0.216503 0.152236 -0.381292
C 1.900785 -0.532317 -0.354083
C -1.526499 0.102732 -0.595726
O 2.965513 -0.928454 -0.507452
O -2.559353 0.428341 -1.066313
C 0.328558 1.564997 0.702699
O 0.428974 2.567322 1.252720
C -1.550192 -1.178822 0.561092
C -0.494063 -1.970975 1.366326
H -2.118790 -1.882229 -0.050890
H -1.028456 -2.681228 2.008996
H 0.112920 -1.339389 2.017834
H 0.171289 -2.554498 0.726497
H 0.137399 -0.466493 -1.717857
H 0.493761 1.071797 -1.464380
FREQS= 36 72 77 98 102 203 294 320 346 359 419 439 472 482 496 534 572 602 629 692 745 780 983 1052 1075 1297 1302 1429 1462 1516 1520 1950 2049 2111 2130 2166 3040 3077 3112 3133 3155
TS028_6
16
E= -1803.01430841 ZPE= 63.25 Gcorr 0.037836000
H 0.505840 -2.402425 -1.170202
Co -0.028093 0.080737 -0.376032
C 0.332932 -1.431745 0.712508
C -1.176522 1.200090 0.369932

O 0.025568 -1.733916 1.814525
O -1.956461 1.830840 0.930714
C 1.460232 0.999635 0.010795
O 2.415663 1.601906 0.216963
C -1.650600 -0.874694 -1.052269
C 0.965464 -1.646110 -0.538964
H -2.615807 -0.411695 -0.835306
H -1.509050 -0.871837 -2.140494
H -1.716068 -1.916506 -0.723195
H 2.052428 -1.638177 -0.525333
H 0.457969 0.661584 -2.558154
H 0.116014 1.325458 -2.604092
FREQS= 59 68 84 94 109 135 149 265 271 279 297 360 389 426 441 455 464 492 537 539 569 600 756 770 872 882 991 1007 1285 1430 1459 1498 1987 2106 2154 3029 3111 3127 3132 3218 4350
TS029_6
16
E= -1803.01669201 ZPE= 65.07 Gcorr 0.044033000
H -0.708526 -1.656050 -1.421194
Co 0.326704 -0.258914 0.524550
C -1.536210 -0.685696 0.261288
C 1.987319 -0.489117 -0.141457
O -2.507911 -0.811530 0.944037
O 2.954652 -0.745800 -0.705769
C 0.192700 1.526147 0.409596
O 0.138601 2.670555 0.332835
C -1.315552 0.378237 -2.044901
C -0.936471 -0.672620 -1.018884
H -2.143290 0.008903 -2.663296
H -1.642771 1.311088 -1.578036
H -0.478742 0.612068 -2.710509
H 0.382166 -1.747341 0.605612
H 0.192490 -0.030931 2.168624
H 0.544205 -0.754569 2.073280
FREQS= 53 79 84 109 173 184 257 292 298 371 427 447 455 486 514 535 555 587 632 737 858 874 886 987 1039 1068 1143 1351 1422 1496 1510 1561 1948 1990 2116 2149 3035 3099 3127 3173 3406
TS030_6
16
E= -1803.02327518 ZPE= 67.80 Gcorr 0.050621000
H -0.276772 -2.512164 1.409442
Co 0.110451 -0.033234 -0.084520
C 1.763968 0.734479 -0.101645
C -1.559401 -0.838932 -0.667243
O 2.767178 1.214243 -0.381810
O -2.657535 -0.519522 -0.273949
C -0.806614 1.465922 0.324620
O -1.377679 2.440434 0.506243
C 0.502312 -1.783569 1.179358
C 0.943414 -1.789234 -0.255433
H 1.315628 -1.847920 1.903864
H -0.012628 -0.785817 1.495056
H 2.018990 -1.835336 -0.408810
H 0.422208 -2.489215 -0.905573
H -1.471293 -1.700385 -1.376029
H 0.103897 0.254925 -1.517729
FREQS= 72 93 118 124 161 259 325 361 374 399 418 451 474 491 535 541 598 659 715 856 883 893 1001 1047 1136 1172 1373 1381 1471 1519 1646 1776 2058 2126 2171 2374 2801 3095 3124 3162 3196
TS031_6
16
E= -1803.01606062 ZPE= 66.47 Gcorr 0.043731000
H 3.023673 2.128292 0.858859
Co -0.100081 -0.137564 -0.060415
C -0.992612 1.321183 -0.460771
C 1.126953 -1.456260 -0.329114
O -1.530417 2.270101 -0.815552
O 1.875555 -2.245185 -0.687774
C -1.509624 -1.097646 0.560078
O -2.696883 -1.098510 0.474351
C 2.362824 1.512841 0.233777
C 1.171913 0.988481 1.048078
H 2.973913 0.704130 -0.181635
H 2.046320 2.139868 -0.606940
H 1.531634 0.350097 1.863727
H 0.650112 1.829286 1.514083
H -0.720645 -0.780665 -1.247706
H -0.945593 -1.679636 1.350328
FREQS= 39 51 69 103 112 137 184 236 287 355 403 435 472 485 509 519 567 591 711 748 808 932 982 1010 1175 1214 1264 1426 1486 1512 1515 1873 1978 2119 2168 2682 3021 3042 3081 3089 3110

TS032_6
16
E= -1803.02005825 ZPE=   67.72 Gcorr   0.047923000
H -0.218952 -2.594352 0.787077
Co -0.096232 -0.017682 0.087290
C -1.849983 -0.475253 -0.001796
C 1.682287 0.597443 0.577414
O -2.971890 -0.671378 0.142415
O 2.661345 0.382925 -0.101047
C -0.397148 1.691793 -0.193795
O -0.588663 2.819279 -0.336660
C 0.765851 -1.748565 -0.989055
C 0.525887 -1.854702 0.505251
H 0.154964 -2.444601 -1.563996
H 0.511858 -0.723205 -1.447537
H 1.822495 -1.831624 -1.239815
H 1.438273 -1.972095 1.083736
H -0.076660 -0.600825 1.448488
H 1.798591 1.133210 1.549427
FREQS=   34   70   91 112 135 197 247 285 339 368 407 445 454 504 515 557 576 619 760 883 918
1011 1016 1069 1194 1207 1370 1412 1458 1512 1594 1779 2035 2097 2142 2484 2833 3109 3139 3186 3212
TS033_6
16
E= -1803.01548183 ZPE=   65.03 Gcorr   0.043668000
H 0.177338 -2.522113 -1.027577
Co -0.026199 0.084588 -0.475244
C 0.225862 -1.368868 0.762143
C -0.993496 1.348993 0.307202
O -0.033937 -1.547848 1.906113
O -1.654365 2.081981 0.895542
C 1.565360 0.823318 -0.120366
O 2.582732 1.292582 0.131573
C -1.852383 -0.788928 -0.803910
C 0.752195 -1.778223 -0.483688
H -1.765527 -1.428578 -1.687581
H -2.160971 -1.410057 0.040514
H -2.652902 -0.066641 -0.984403
H 1.832125 -1.889777 -0.533477
H 0.326687 0.644895 -2.192933
H -0.390042 0.356929 -2.217061
FREQS=   68   84   93 102 124 162 266 295 343 398 425 431 443 459 471 487 531 537 567 587 648
761 818 858 878 1000 1014 1099 1287 1429 1482 1493 1981 2109 2152 3044 3129 3134 3140 3224 3937
TS034_6
16
E= -1803.01324071 ZPE=   65.68 Gcorr   0.041725000
H 3.925747 0.492477 0.298238
Co -0.697273 -0.187506 -0.007723
C -2.418127 -0.702579 -0.178874
C 1.220884 -0.365577 0.305804
O -3.490863 -1.091954 -0.267833
O 1.551379 -0.562200 1.452075
C -0.630432 1.562051 0.001235
O -0.619808 2.711931 0.006504
C 3.685497 -0.325772 -0.385606
C 2.224403 -0.257887 -0.832776
H 3.888166 -1.262618 0.138852
H 4.353432 -0.260045 -1.249292
H 2.011656 0.675922 -1.372529
H 1.979045 -1.064883 -1.536516
H -0.302023 -1.631347 -0.378819
H -1.048673 0.189526 1.323922
FREQS=   26   50   68   81   98 200 208 277 292 307 360 375 452 457 511 542 659 667 697 704 785
914 1007 1074 1100 1275 1350 1423 1460 1507 1513 1775 1866 2117 2144 2173 3027 3061 3063 3134 3143
TS035_6
16
E= -1803.01573156 ZPE=   67.19 Gcorr   0.046481000
H -2.277401 0.510758 1.371217
Co -0.155401 -0.159065 0.066936
C -0.411115 1.377321 -0.838022
C 1.173683 -1.131591 -0.666615
O -0.468866 2.382081 -1.396598
O 1.990041 -1.788392 -1.136073
C 0.979794 0.742823 1.257836
O 2.177979 0.820874 1.185559
C -2.270363 -1.042374 -0.204475
C -1.832729 -0.452635 1.124706
H -2.575121 -2.088328 -0.128644

H -3.053654 -0.456007 -0.688024
H -1.436947 -1.071053 -0.976874
H -1.994754 -1.132421 1.960970
H 0.433222 1.231202 2.099257
H -0.328378 -0.977166 1.291132
FREQS= 60 69 89 100 128 139 179 266 308 340 399 419 444 473 497 509 562 625 721 879 912
985 1022 1075 1160 1204 1326 1424 1460 1527 1566 1805 2070 2123 2149 2632 2840 3089 3108 3149 3167
TS036_6
16
E= -1803.01704585 ZPE= 67.40 Gcorr 0.047808000
H -1.754657 -2.031737 1.444428
Co -0.098564 -0.032111 0.033619
C -1.729609 0.717398 -0.226219
C 1.714658 -0.710213 -0.083601
O -2.669847 1.267350 -0.588772
O 2.584668 -0.460849 -0.875911
C 0.697333 1.497383 0.387520
O 1.228050 2.501627 0.573324
C -0.677924 -1.863258 -0.451224
C -0.743262 -1.886668 1.065025
H 0.059660 -2.546819 -0.865882
H -1.640557 -1.985013 -0.940443
H -0.157991 -0.538200 -1.350631
H -0.058744 -2.620744 1.495759
H -0.407884 -0.911753 1.579265
H 1.911259 -1.491609 0.711652
FREQS= 53 70 93 112 128 186 237 321 340 368 401 435 446 498 510 553 564 619 748 874 900
1003 1011 1069 1193 1203 1370 1423 1459 1518 1592 1812 2060 2104 2146 2475 2659 3091 3134 3166 3206
TS037_6
16
E= -1803.01538940 ZPE= 66.33 Gcorr 0.047284000
H -1.381930 -2.250562 0.232816
Co 0.134140 -0.052596 -0.282963
C 1.935280 -0.323195 -0.228198
C -1.695716 0.184405 -0.920984
O 3.063730 -0.437537 -0.390592
O -2.657889 -0.342668 -0.412955
C 0.065722 1.609703 0.319601
O 0.012856 2.696059 0.688183
C -0.430881 -1.890035 0.611689
C -0.453483 -0.847460 1.553285
H -0.058515 -1.377723 -0.942460
H 0.376666 -2.616088 0.639367
H -1.413041 -0.424404 1.826852
H 0.342065 -0.763624 2.285941
H 0.476604 0.292880 -1.679137
H -1.838725 0.832271 -1.812831
FREQS= 72 85 91 111 168 206 247 334 371 394 419 437 500 511 534 545 621 634 674 834 848
864 912 990 1073 1165 1255 1261 1373 1468 1546 1782 2017 2058 2131 2160 2881 3151 3167 3241 3267
TS038_6
16
E= -1803.01292763 ZPE= 66.52 Gcorr 0.046041000
H 0.973029 2.096267 -0.454050
Co 0.064248 0.006518 -0.250547
C -1.598655 0.528527 -0.842530
C 1.808758 -0.511063 -0.569133
O -2.728885 0.164982 -0.968862
O 2.772825 -1.059513 -0.857083
C -0.589488 -1.248555 0.724982
O -1.004037 -2.091202 1.387701
C 0.691756 2.312847 0.600297
C 0.398472 1.053723 1.407157
H -0.173618 2.979746 0.545692
H 1.548727 2.872590 0.992642
H 1.248418 0.721454 2.001925
H -0.473286 1.156223 2.053326
H -1.281515 1.584173 -1.131615
H -0.160732 -0.513437 -1.661839
FREQS= 54 85 97 106 110 145 262 276 349 388 408 430 463 496 515 521 562 595 655 780 853
936 967 1004 1167 1204 1230 1422 1482 1498 1507 1858 1894 2118 2155 2607 2877 3054 3109 3120 3170
TS039_6
16
E= -1803.01109589 ZPE= 65.69 Gcorr 0.044494000
H 0.448373 0.732282 -1.662152
Co 0.140606 0.041308 -0.388585
C 0.107087 1.558366 0.548877
C -0.369500 -1.324035 0.699070

O 0.063163 2.570105 1.092789
O -0.670261 -2.203980 1.366806
C -1.709846 0.341284 -0.856612
O -2.664306 -0.143844 -0.304241
C 2.078432 -0.984186 -0.687352
C 2.294656 0.050654 0.188064
H 2.321303 -0.879438 -1.737755
H 1.958208 -2.001653 -0.332601
H 2.332072 -0.122911 1.258388
H 2.717502 0.986859 -0.158260
H 0.048936 -0.504306 -1.762948
H -1.856501 1.043119 -1.707996
FREQS= 60 75 93 109 115 164 210 247 284 333 406 447 486 491 518 527 603 632 658 793 844 921 961 977 1011 1034 1242 1319 1364 1490 1606 1794 2032 2061 2138 2162 2867 3170 3181 3252 3276
TS040_6
16
E= -1803.01149594 ZPE= 66.96 Gcorr 0.045354000
H -0.642690 -1.076173 1.737920
Co 0.182808 0.056388 -0.077824
C 1.605112 -0.921041 -0.480163
C -1.139563 1.095299 -0.636900
O 2.439280 -1.611046 -0.867469
O -2.002167 1.673396 -1.130525
C 1.257897 1.562178 0.349753
O 1.129396 1.484399 1.559062
C -2.404467 -1.525031 0.541609
C -0.895966 -1.425841 0.721806
H -2.907683 -0.568968 0.719036
H -2.674999 -1.859295 -0.465617
H -2.830270 -2.250884 1.247354
H -0.431805 -2.405504 0.587169
H 1.869653 2.359428 -0.121468
H -0.388181 -0.808445 -1.068327
FREQS= 51 75 97 100 111 123 195 224 268 280 344 448 458 490 521 538 543 625 685 781 844 913 998 1054 1205 1275 1293 1426 1463 1507 1510 1721 2097 2147 2211 2910 2978 3026 3085 3101 3121
TS041_6
16
E= -1802.99958663 ZPE= 62.98 Gcorr 0.035221000
H 2.837302 -1.345963 -0.751974
Co -0.070044 0.055290 -0.501139
C 1.344328 -0.411413 0.481866
C -1.503276 -0.822382 0.295728
O 1.527073 -0.760306 1.613429
O -2.380991 -0.560197 1.053711
C -0.112258 1.741934 0.016425
O -0.246537 2.791195 0.472071
C -0.826161 -1.733530 -0.577366
C 2.435185 -0.339619 -0.610241
H 2.062715 0.028387 -1.575683
H -0.337520 -2.580110 -0.099530
H -1.364975 -1.976181 -1.496528
H 3.230901 0.328322 -0.269111
H -1.736442 0.684079 -2.821261
H -2.024056 0.993156 -2.207310
FREQS= 49 72 80 88 96 119 140 151 161 188 209 306 348 419 439 462 481 504 577 593 623 671 828 876 953 962 981 1073 1361 1401 1458 1474 1861 1951 2119 3025 3092 3109 3150 3188 4418
TS042_6
16
E= -1803.00427519 ZPE= 64.50 Gcorr 0.042547000
H 0.338405 -2.253053 1.834915
Co -0.308749 0.064546 -0.541176
C 1.600800 -0.061935 -0.469580
C -1.999010 -0.406543 -0.174635
O 2.560186 0.604853 -0.722444
O -3.037051 -0.805224 0.120007
C -0.231381 1.463655 0.560224
O -0.230889 2.355100 1.287808
C 1.177894 -1.620305 1.532610
C 1.123851 -1.303926 0.048675
H 2.107489 -2.156762 1.760494
H 1.146392 -0.711640 2.138298
H -0.302914 -1.366114 -0.926375
H 1.352367 -2.148022 -0.598711
H -0.287364 0.229995 -2.172078
H -0.389038 0.999353 -1.891539
FREQS= 42 72 90 98 178 199 249 294 314 360 380 423 460 477 485 528 533 548 588 662 745 867 995 1014 1041 1088 1182 1346 1423 1495 1509 1539 1931 2073 2112 2146 3037 3108 3134 3150 3206

TS043_6
16
E= -1802.99921472 ZPE= 63.87 Gcorr 0.042767000
H -2.664210 -2.275888 -0.335712
Co 0.364084 0.407083 -0.366343
C 2.024890 -0.146559 -0.102760
C -0.813796 1.612765 0.178760
O 3.139034 -0.384289 0.054918
O -1.482176 2.486226 0.515107
C -0.349002 -0.927594 0.811511
O -0.474880 -1.228314 1.957011
C -2.221579 -1.405565 -0.831984
C -0.729656 -1.349855 -0.528161
H -2.736129 -0.512036 -0.469461
H -2.407422 -1.486881 -1.906863
H -0.160943 -2.219543 -0.857409
H -0.217414 -0.386877 -1.563906
H 0.298268 0.545853 -1.924444
H 1.136611 1.655995 -0.431435
FREQS= 67 75 101 103 175 187 278 299 324 341 412 470 486 505 533 583 594 613 678 743 796
838 862 1037 1063 1096 1137 1324 1427 1501 1508 1794 1925 2019 2080 2105 2155 3050 3121 3130 3144
TS001_7
12
E= -1837.78171667 ZPE= 41.28 Gcorr 0.005865000
H 3.537052 0.000076 -0.844626
Co -0.209437 -0.000009 -0.238357
C 2.716280 0.000030 -0.114914
C -1.970281 -0.000022 -0.163461
O 1.507141 0.000039 -0.819638
O -3.118907 -0.000012 -0.192296
C 0.014430 -1.700954 0.296072
O 0.292081 -2.703200 0.777886
C 0.014380 1.700945 0.296077
O 0.292021 2.703192 0.777895
H 2.845117 -0.890501 0.523390
H 2.845079 0.890513 0.523463
FREQS= 42 79 79 80 107 185 212 268 390 412 444 445 458 510 543 585 618 1134 1180 1192 1489
1495 1520 2110 2115 2184 2952 2993 3054
TS002_7
12
E= -1837.77685307 ZPE= 40.95 Gcorr 0.004485000
H 2.147874 -0.885695 1.630496
Co -0.134841 -0.000238 0.022712
C 2.368230 0.004255 1.013348
C 0.010393 -1.757931 -0.351119
O 1.711593 0.003453 -0.233183
O 0.180996 -2.818803 -0.741479
C -1.854605 -0.003586 0.352782
O -2.971152 -0.005749 0.629497
C 0.003504 1.758035 -0.351090
O 0.169993 2.819620 -0.741285
H 2.144253 0.892783 1.631228
H 3.452010 0.006526 0.833134
FREQS= 29 72 86 92 105 155 168 253 385 401 431 435 469 479 561 597 610 1066 1181 1181 1485
1499 1519 2115 2130 2201 2931 2962 3044
TS003_7
12
E= -1837.77549221 ZPE= 40.62 Gcorr 0.004558000
H 1.227793 2.738822 1.536726
Co -0.038899 -0.044930 0.075062
C 1.219641 1.673832 1.262480
C 1.417996 -0.906173 -0.525595
O 0.954177 1.527791 -0.106861
O 2.317625 -1.361665 -1.069463
C -0.966232 -1.490888 0.467020
O -1.566262 -2.428406 0.752153
C -1.436682 0.949277 -0.482784
O -2.229329 1.595294 -0.998007
H 2.179231 1.228503 1.572544
H 0.425214 1.225379 1.914758
FREQS= 51 62 83 93 97 126 204 277 398 408 444 451 468 501 555 586 611 1041 1151 1167 1448
1486 1498 2116 2121 2189 2770 2979 3034
TS004_7
12
E= -1837.77549214 ZPE= 40.62 Gcorr 0.004560000
H -1.231246 2.736874 1.537232
Co 0.038994 -0.044833 0.075073

C -1.222022 1.672021 1.262496
C 1.435687 0.950908 -0.482703
O -0.955753 1.526841 -0.106779
O 2.227631 1.597847 -0.997854
C 0.967910 -1.489773 0.467034
O 1.568988 -2.426620 0.752162
C -1.416905 -0.907682 -0.525741
O -2.315955 -1.364178 -1.069727
H -0.427522 1.223987 1.914986
H -2.181363 1.225680 1.571874
FREQS= 51 63 83 93 97 126 204 277 398 408 444 451 468 501 555 586 611 1041 1151 1167 1448 1486 1498 2116 2121 2189 2769 2979 3034
TS005_7
12
E= -1837.77214310 ZPE= 42.54 Gcorr 0.010881000
H 1.566752 -1.731373 -1.366347
Co -0.283148 -0.106274 0.107296
C 1.184471 -1.452699 -0.376560
C -0.163335 1.606678 0.011499
O 0.069329 -1.946575 0.070696
O -0.001015 2.745647 -0.098287
C -2.038585 -0.211092 -0.022209
O -3.179095 -0.285296 -0.132175
C 1.725726 -0.384746 0.368379
O 2.813904 0.318344 -0.116968
H 1.686760 -0.461346 1.458792
H 2.516828 1.056313 -0.662218
FREQS= 45 74 94 96 233 310 358 398 441 449 468 489 518 574 636 646 813 987 1128 1225 1271 1323 1377 1537 2085 2153 3083 3107 3840
TS006_7
12
E= -1837.76252931 ZPE= 38.52 Gcorr 0.001700000
H 0.933992 -2.674958 -0.570049
Co 0.000333 -0.124310 0.030238
C 0.003629 -2.128947 -0.790219
C -0.003442 1.443917 -0.822773
O 0.002891 -1.212207 -1.672455
O -0.006089 2.441077 -1.385677
C -1.573137 0.069604 0.843371
O -2.522966 0.219024 1.475907
C 1.573692 0.075343 0.842568
O 2.523147 0.228594 1.474721
H -0.925538 -2.677352 -0.570951
H 0.002238 -1.462719 0.746921
FREQS= 60 64 89 91 115 117 295 304 341 409 452 455 467 506 547 554 596 662 936 1222 1234 1426 1602 1970 2096 2124 2177 2983 3054
TS007_7
12
E= -1837.76252931 ZPE= 38.52 Gcorr 0.001700000
H 0.002270 -1.462717 0.746924
Co 0.000338 -0.124311 0.030235
C 0.003681 -2.128953 -0.790202
C -1.573133 0.069568 0.843375
O 0.002929 -1.212221 -1.672447
O -2.522964 0.218965 1.475912
C -0.003490 1.443915 -0.822776
O -0.006175 2.441077 -1.385677
C 1.573696 0.075387 0.842559
O 2.523149 0.228669 1.474708
H -0.925477 -2.677376 -0.570938
H 0.934052 -2.674945 -0.570025
FREQS= 60 64 89 91 115 117 295 304 341 409 452 455 467 506 547 554 596 662 936 1222 1234 1426 1602 1970 2096 2124 2177 2983 3054
TS008_7
12
E= -1837.76252931 ZPE= 38.52 Gcorr 0.001700000
H 0.933992 -2.674958 -0.570049
Co 0.000333 -0.124310 0.030238
C 0.003629 -2.128947 -0.790219
C -0.003442 1.443917 -0.822773
O 0.002891 -1.212207 -1.672455
O -0.006089 2.441077 -1.385677
C -1.573137 0.069604 0.843371
O -2.522966 0.219024 1.475907
C 1.573692 0.075343 0.842568
O 2.523147 0.228594 1.474721
H -0.925538 -2.677352 -0.570951

H 0.002238 -1.462719 0.746921
FREQS=   60   64   89   91 115 117 295 304 341 409 452 455 467 506 547 554 596 662 936 1222 1234
1426 1602 1970 2096 2124 2177 2983 3054
TS009_7
12
E= -1837.76252931 ZPE=   38.52 Gcorr   0.001700000
H 0.002270 -1.462717 0.746924
Co 0.000338 -0.124311 0.030235
C 0.003681 -2.128953 -0.790202
C -1.573133 0.069568 0.843375
O 0.002929 -1.212221 -1.672447
O -2.522964 0.218965 1.475912
C -0.003490 1.443915 -0.822776
O -0.006175 2.441077 -1.385677
C 1.573696 0.075387 0.842559
O 2.523149 0.228669 1.474708
H -0.925477 -2.677376 -0.570938
H 0.934052 -2.674945 -0.570025
FREQS=   60   64   89   91 115 117 295 304 341 409 452 455 467 506 547 554 596 662 936 1222 1234
1426 1602 1970 2096 2124 2177 2983 3054
TS010_7
12
E= -1837.76364413 ZPE=   38.64 Gcorr   0.003206000
H 0.013508 1.226583 1.084850
Co -0.004167 0.113443 0.088734
C 0.251879 2.047723 -0.948027
C -0.025223 -1.472368 -0.752641
O -0.183893 1.150266 -1.704689
O -0.041897 -2.499186 -1.259199
C 1.597715 -0.074438 0.804988
O 2.588634 -0.188872 1.380511
C -1.625301 0.028350 0.804795
O -2.612302 -0.028923 1.392209
H -0.427289 2.791445 -0.506049
H 1.327532 2.277125 -0.899981
FREQS=   57   85   87 111 118 253 316 344 357 394 405 455 466 495 548 563 604 695 747 1112 1240
1478 1649 1984 2098 2128 2177 2990 3071
TS011_7
12
E= -1837.76680620 ZPE=   41.37 Gcorr   0.007376000
H -1.705340 1.924692 0.108759
Co 0.148892 0.019853 -0.126841
C -1.397845 1.123328 -0.572277
C 1.553099 -1.059781 -0.443996
O -2.495142 0.364782 -1.040941
O 2.394598 -1.747974 -0.814870
C -0.879851 -1.118239 0.742732
O -1.501326 -1.756705 1.474865
C 1.040174 1.454820 0.383960
O 1.579768 2.312943 0.933453
H -0.953936 1.578309 -1.474008
H -3.077442 0.175831 -0.292605
FREQS=   56   77   90 100 109 250 311 338 393 401 450 490 493 511 548 567 598 697 1053 1136 1252
1383 1453 2071 2094 2157 2974 3074 3811
TS012_7
12
E= -1837.76235637 ZPE=   38.51 Gcorr   0.003415000
H 0.932549 0.283189 2.368860
Co 0.000034 -0.056293 -0.140712
C -0.000521 -0.139429 1.973577
C 0.000209 1.738012 -0.045431
O -0.000115 -1.287537 1.415504
O 0.000311 2.885802 -0.090241
C 1.662455 -0.572946 -0.573741
O 2.705056 -0.909626 -0.913217
C -1.662397 -0.572726 -0.573977
O -2.704989 -0.909256 -0.913636
H -0.933999 0.282794 2.368320
H -0.000049 0.001394 -1.607810
FREQS=   71   91 110 111 124 172 311 349 399 407 448 451 477 485 515 538 643 676 723 1049 1227
1404 1599 2036 2118 2122 2176 3011 3096
TS013_7
12
E= -1837.76176912 ZPE=   38.65 Gcorr   0.003765000
H 0.088885 -0.549407 2.579628
Co -0.013328 -0.136227 -0.146012
C -0.019067 1.449227 0.667008

C 0.513876 -1.131827 1.747802
O -0.024707 2.517668 1.091508
O -0.216865 -1.834035 1.011137
C 1.579220 -0.027366 -0.888501
O 2.568334 0.042351 -1.472774
C -1.706592 -0.079545 -0.695265
O -2.766606 -0.069501 -1.134091
H 1.607985 -1.253873 1.744186
H -0.022863 -1.033391 -1.333987
FREQS= 69 85 93 113 122 261 317 375 383 394 431 463 474 509 533 561 625 644 705 1087 1237 1474 1645 1992 2107 2119 2172 2985 3064
TS014_7
12
E= -1837.76492495 ZPE= 41.30 Gcorr 0.007155000
H -2.298707 -0.084384 -1.776586
Co 0.146835 -0.019456 -0.097472
C -1.357914 -1.209697 -0.488550
C 1.476512 1.137602 -0.431331
O -2.508802 -0.514554 -0.938077
O 2.262788 1.867586 -0.844299
C -0.960519 1.086917 0.735072
O -1.590067 1.728901 1.452218
C 1.132325 -1.409731 0.362030
O 1.739221 -2.245972 0.872468
H -1.686614 -1.874990 0.311869
H -0.946751 -1.833554 -1.305349
FREQS= 52 79 91 100 116 246 294 334 360 395 457 482 486 508 547 570 600 695 1036 1144 1251 1377 1453 2081 2096 2161 2928 3126 3824
TS015_7
12
E= -1837.76492494 ZPE= 41.30 Gcorr 0.007156000
H 2.299839 0.083166 -1.775375
Co -0.146894 0.019460 -0.097650
C 1.358162 1.209312 -0.488682
C -1.477041 -1.137179 -0.431191
O 2.509464 0.514199 -0.937190
O -2.263683 -1.866944 -0.843837
C 0.960173 -1.087298 0.734704
O 1.589621 -1.729524 1.451720
C -1.131892 1.410058 0.361986
O -1.738370 2.246550 0.872503
H 1.686329 1.875146 0.311507
H 0.947284 1.832660 -1.306033
FREQS= 51 79 91 100 116 246 294 334 360 395 457 482 486 508 547 570 600 695 1036 1144 1251 1377 1453 2081 2096 2161 2928 3126 3824
TS016_7
12
E= -1837.76301565 ZPE= 40.78 Gcorr 0.005735000
H -2.875594 -0.114912 0.121045
Co 0.156945 0.013246 -0.171035
C -1.436780 0.925673 -0.693552
C 0.827123 1.527592 0.448709
O -2.737043 0.403075 -0.679026
O 1.216714 2.456667 1.005324
C -0.726953 -1.304051 0.590557
O -1.316037 -2.053183 1.240033
C 1.711021 -0.848684 -0.413867
O 2.686443 -1.398484 -0.667987
H -0.927767 0.674529 -1.678611
H -1.481227 2.014965 -0.602330
FREQS= 50 80 91 93 103 164 308 320 333 403 444 497 508 520 544 571 585 717 1087 1149 1240 1320 1466 2080 2104 2164 2646 3080 3859
TS017_7
12
E= -1837.76347685 ZPE= 40.93 Gcorr 0.006209000
H 0.985044 1.851575 -1.267863
Co -0.152717 0.022899 -0.115634
C 1.353374 1.224125 -0.433477
C -1.141621 1.409981 0.343218
O 2.465915 0.441079 -0.861975
O -1.737177 2.255962 0.849249
C -1.497375 -1.121113 -0.399486
O -2.311370 -1.844370 -0.766247
C 0.969521 -1.134724 0.632945
O 1.609391 -1.811307 1.304908
H 1.620802 1.893753 0.395923
H 3.200043 1.035861 -1.072617

FREQS=   51   81   94 102 125 159 259 319 355 398 456 481 486 509 545 585 602 705 1034 1167 1230
1327 1478 2087 2105 2170 2924 3026 3770
TS018_7
12
E= -1837.74819156 ZPE=   39.91 Gcorr   -0.008810000
H 2.809631 -1.834561 1.244040
Co -0.392935 -0.164326 0.312846
C 1.885026 -2.101041 0.716343
C -1.350162 -0.814014 -0.926798
O 1.064910 -0.971394 0.631269
O -1.956140 -1.312431 -1.773178
C -1.524789 1.104293 0.650798
O -2.250568 1.957711 0.926711
C 2.826921 1.694673 -0.261506
O 2.294392 1.869123 -1.252177
H 2.158634 -2.455857 -0.286486
H 1.398250 -2.920312 1.261593
FREQS=   8   14   34   39   46   70   78 103 124 237 289 438 473 480 561 605 710 1172 1182 1196 1480
1499 1506 2084 2141 2204 3001 3067 3075
TS019_7
12
E= -1837.76090289 ZPE=   41.14 Gcorr   0.005185000
H 0.938926 -0.001841 -1.979729
Co -0.029984 0.000064 -0.037181
C 1.923733 -0.003957 -0.213875
C -1.744452 0.003587 -0.522547
O 1.853400 -0.003769 -1.640077
O -2.822663 0.005806 -0.919415
C 0.036370 -1.630028 0.641148
O 0.067464 -2.609413 1.245559
C 0.043141 1.629846 0.641163
O 0.078367 2.609069 1.245609
H 2.450835 -0.897380 0.127390
H 2.454505 0.887257 0.127482
FREQS=   49   50   77   93 102 148 182 309 399 404 478 494 497 530 572 573 584 870 1006 1129 1204
1307 1533 2085 2102 2166 3072 3129 3631
TS020_7
12
E= -1837.75434793 ZPE=   37.53 Gcorr   -0.001189000
H 2.676416 1.301037 0.859557
Co -0.119638 -0.048124 -0.199290
C 1.777258 1.866405 1.149854
C -0.641066 -1.329464 0.941703
O 0.654545 1.509081 0.820412
O -1.019492 -2.232073 1.550709
C -1.661081 0.735810 -0.578503
O -2.667574 1.183829 -0.913636
C 1.358553 -0.723668 -0.869872
O 2.280324 -1.187406 -1.387690
H -0.342009 -0.260473 -1.647242
H 1.911420 2.776850 1.751055
FREQS=   55   78   86   91   97 116 203 273 311 316 385 406 466 476 501 517 567 607 663 1138 1262
1525 1734 1973 2079 2090 2146 2994 3095
TS021_7
12
E= -1837.74633295 ZPE=   37.14 Gcorr   -0.008413000
H -3.797487 -0.054333 -1.112875
Co 0.319970 0.002726 -0.233614
C -2.862467 -0.035610 -0.516280
C 0.732006 -1.664264 -0.604250
O -2.866209 -0.025627 0.692963
O 1.129209 -2.690229 -0.938736
C 0.690439 1.678761 -0.607900
O 1.061960 2.713595 -0.944498
C 0.632784 0.008476 1.514855
O 0.896970 0.012818 2.631234
H 0.140785 -0.001390 -1.727957
H -1.914499 -0.026520 -1.097833
FREQS=   13   26   35   63   80   96   97 178 289 297 333 446 477 486 502 577 587 621 758 1208 1276
1545 1825 1941 2097 2123 2185 2858 2959
TS022_7
12
E= -1837.74633237 ZPE=   37.14 Gcorr   -0.008375000
H -3.796158 0.114800 -1.113479
Co 0.319819 -0.005931 -0.233557
C -2.861667 0.076196 -0.517016
C 0.631808 -0.018006 1.515038

O -2.865793 0.056263 0.692106
O 0.895443 -0.027184 2.631517
C 0.666568 -1.686711 -0.609514
O 1.023551 -2.726350 -0.947016
C 0.756295 1.655181 -0.602254
O 1.168745 2.675519 -0.935540
H -1.913855 0.056792 -1.098562
H 0.141307 0.002612 -1.727961
FREQS= 13 26 35 62 80 96 97 178 289 297 333 446 477 486 502 577 587 621 759 1208 1276
1545 1825 1941 2097 2123 2185 2858 2959
TS023_7
12
E= -1837.76573225 ZPE= 42.59 Gcorr 0.011139000
H 1.721150 -0.848294 -1.939674
Co -0.173385 -0.084618 -0.112782
C 1.516936 -0.831746 -0.861786
C -0.610680 1.582482 -0.170977
O 0.727581 -1.721099 -0.325260
O -0.883732 2.700409 -0.256548
C -1.784963 -0.733848 0.214658
O -2.822525 -1.168658 0.439024
C 1.855987 0.289082 -0.074399
O 2.152407 0.179318 1.278622
H 2.353326 1.123022 -0.565052
H 1.353390 0.254386 1.818149
FREQS= 52 74 95 115 205 261 345 378 430 449 462 490 520 595 625 789 931 970 1017 1159 1279
1301 1451 1516 2096 2158 3072 3171 3787
TS024_7
12
E= -1837.75421126 ZPE= 38.04 Gcorr -0.000080000
H -0.476616 2.453803 0.931915
Co 0.069508 -0.072212 -0.127138
C -1.001287 1.659446 1.481476
C 1.320359 1.082463 -0.638089
O -0.465539 0.584906 1.758460
O 2.162040 1.732354 -1.083414
C 0.770742 -1.633833 0.430448
O 1.243308 -2.661758 0.630437
C -1.577550 -0.267042 -0.807090
O -2.541255 -0.512902 -1.390094
H 0.359000 -0.573379 -1.458991
H -2.021111 1.882293 1.836226
FREQS= 53 66 82 87 102 110 290 306 335 350 401 444 446 459 494 511 526 677 739 1143 1258
1518 1709 2080 2085 2098 2161 2991 3086
TS025_7
12
E= -1837.74823046 ZPE= 39.73 Gcorr -0.005790000
H -0.696732 -0.677819 -1.352196
Co 0.503265 -0.188994 -0.263985
C -0.606285 -1.820840 -1.017278
C 1.795834 0.084876 0.850581
O 0.340403 -1.966945 -0.041400
O 2.667298 0.248248 1.581906
C 0.341835 1.472089 -0.733134
O 0.192772 2.570074 -1.056567
C -3.878297 0.437022 1.097276
O -2.800968 0.284724 0.761844
H -1.636299 -2.034109 -0.700287
H -0.369680 -2.312927 -1.970847
FREQS= 6 21 31 42 75 88 102 132 330 409 413 471 492 506 560 615 679 1110 1170 1253 1324
1533 1738 2093 2158 2171 2203 3006 3063
TS026_7
12
E= -1837.72936996 ZPE= 30.55 Gcorr -0.024572000
H 2.503111 -2.027617 0.118074
Co 0.434763 -0.419488 -0.300295
C -1.508362 2.066997 0.205211
C -1.010553 -1.363356 -0.654081
O -2.627214 1.872872 0.132810
O -1.977909 -1.898955 -0.962721
C 1.646530 0.833125 -0.598025
O 2.363884 1.684650 -0.872991
C 0.432536 -0.469927 1.458897
O 0.367218 -0.435702 2.606148
H 0.349049 -0.286486 -1.800904
H 3.040509 -2.543662 0.092815

FREQS= 14 33 43 51 55 75 93 97 100 146 171 279 285 316 339 412 470 481 504 522 584
592 758 1935 2108 2120 2188 2203 4398
TS027_7
12
E= -1837.74511468 ZPE= 34.25 Gcorr -0.008738000
H -1.719645 -0.000003 1.845213
Co 0.115051 0.000000 0.266415
C -1.733154 -0.000004 0.713289
C 1.929401 0.000005 0.174903
O -2.778495 -0.000006 0.126073
O 3.071680 0.000008 0.079949
C -0.130549 1.609340 -0.419438
O -0.286968 2.582202 -1.013462
C -0.130539 -1.609345 -0.419433
O -0.286949 -2.582210 -1.013454
H 0.624092 -0.374273 2.616425
H 0.624089 0.374341 2.616398
FREQS= 45 53 82 86 94 105 178 266 291 323 327 360 373 377 436 483 510 512 551 554 563
851 1307 1830 2094 2114 2173 2676 4342
TS028_7
12
E= -1837.74511468 ZPE= 34.25 Gcorr -0.008738000
H 0.624085 -0.374297 2.616417
Co 0.115050 0.000000 0.266415
C -1.733155 -0.000001 0.713289
C -0.130547 1.609341 -0.419437
O -2.778495 -0.000005 0.126072
O -0.286965 2.582203 -1.013460
C 1.929404 0.000003 0.174904
O 3.071682 0.000007 0.079949
C -0.130540 -1.609344 -0.419435
O -0.286952 -2.582209 -1.013456
H -1.719645 0.000004 1.845213
H 0.624091 0.374318 2.616409
FREQS= 45 53 82 86 94 105 178 266 291 323 327 360 373 377 436 483 510 512 551 554 563
851 1307 1830 2094 2114 2173 2676 4342
TS029_7
12
E= -1837.74511468 ZPE= 34.25 Gcorr -0.008738000
H -1.719645 -0.000003 1.845213
Co 0.115051 0.000000 0.266415
C -1.733154 -0.000004 0.713289
C 1.929401 0.000005 0.174903
O -2.778495 -0.000006 0.126073
O 3.071680 0.000008 0.079949
C -0.130549 1.609340 -0.419438
O -0.286968 2.582202 -1.013462
C -0.130539 -1.609345 -0.419433
O -0.286949 -2.582210 -1.013454
H 0.624092 -0.374273 2.616425
H 0.624089 0.374341 2.616398
FREQS= 45 53 82 86 94 105 178 266 291 323 327 360 373 377 436 483 510 512 551 554 563
851 1307 1830 2094 2114 2173 2676 4342
TS030_7
12
E= -1837.74511468 ZPE= 34.25 Gcorr -0.008738000
H 0.624085 -0.374297 2.616417
Co 0.115050 0.000000 0.266415
C -1.733155 -0.000001 0.713289
C -0.130547 1.609341 -0.419437
O -2.778495 -0.000005 0.126072
O -0.286965 2.582203 -1.013460
C 1.929404 0.000003 0.174904
O 3.071682 0.000007 0.079949
C -0.130540 -1.609344 -0.419435
O -0.286952 -2.582209 -1.013456
H -1.719645 0.000004 1.845213
H 0.624091 0.374318 2.616409
FREQS= 45 53 82 86 94 105 178 266 291 323 327 360 373 377 436 483 510 512 551 554 563
851 1307 1830 2094 2114 2173 2676 4342
TS031_7
12
E= -1837.72948776 ZPE= 30.97 Gcorr -0.023970000
H -0.425038 -0.521366 -1.683669
Co -0.433585 -0.458930 -0.177049
C 1.421635 2.139243 -0.009133
C -0.403681 -0.339894 1.581533

O 2.519368 2.011288 -0.279950
O -0.344105 -0.214587 2.722518
C -1.689532 0.720750 -0.569481
O -2.436756 1.521276 -0.910872
C 1.036770 -1.381476 -0.475751
O 2.013242 -1.915095 -0.757937
H -1.808544 -2.276821 -1.421645
H -2.264758 -2.865476 -1.467432
FREQS= 7 32 45 48 59 87 90 95 96 126 273 278 336 389 393 414 470 481 504 572 584
591 755 1939 2108 2120 2188 2203 4383
TS032_7
12
E= -1837.75990574 ZPE= 41.43 Gcorr 0.006713000
H -1.765896 0.865889 1.559293
Co 0.215680 0.006450 0.223569
C -1.673159 -0.022938 0.914627
C -0.069572 1.655627 -0.321627
O -2.773385 -0.138790 0.041906
O -0.302067 2.642620 -0.871845
C 2.000377 0.030067 0.166884
O 3.142164 0.051476 0.295327
C -0.016176 -1.662513 -0.329459
O -0.182901 -2.659158 -0.878527
H -2.868544 0.696371 -0.433551
H -1.708231 -0.907057 1.560453
FREQS= 40 70 85 92 115 219 298 354 359 398 452 475 486 533 545 563 574 806 1056 1124 1268
1386 1492 2072 2098 2162 2968 3072 3821
TS033_7
12
E= -1837.74694965 ZPE= 35.05 Gcorr -0.006062000
H 2.470778 -0.000465 -0.900708
Co -0.023601 -0.000031 0.143355
C 1.898399 -0.000191 0.052937
C -1.809687 0.000157 0.471568
O 2.474841 0.000013 1.117842
O -2.941200 0.000291 0.653775
C 0.030867 -1.620629 -0.556964
O 0.035941 -2.600351 -1.162593
C 0.031150 1.620523 -0.557121
O 0.036361 2.600171 -1.162867
H -0.154314 0.000634 2.597050
H 0.568839 0.000507 2.401302
FREQS= 55 68 87 88 110 119 178 279 318 328 345 390 396 449 458 491 504 529 552 554 617
910 1354 1767 2084 2110 2169 2880 4334
TS034_7
12
E= -1837.74694965 ZPE= 35.05 Gcorr -0.006062000
H -2.470820 0.001547 -0.900630
Co 0.023598 0.000059 0.143331
C -1.898393 0.000735 0.052985
C 1.809685 -0.000645 0.471561
O -2.474782 0.000266 1.117921
O 2.941189 -0.001095 0.653816
C -0.030420 1.620703 -0.556896
O -0.035254 2.600456 -1.162478
C -0.031619 -1.620445 -0.557243
O -0.037092 -2.600060 -1.163037
H -0.568752 -0.001025 2.401360
H 0.154398 -0.000732 2.597119
FREQS= 55 68 87 88 110 119 178 279 318 328 345 390 396 449 458 491 504 529 552 554 617
910 1354 1767 2084 2110 2169 2880 4334
TS035_7
12
E= -1837.74694965 ZPE= 35.05 Gcorr -0.006062000
H 2.470778 -0.000465 -0.900708
Co -0.023601 -0.000031 0.143355
C 1.898399 -0.000191 0.052937
C -1.809687 0.000157 0.471568
O 2.474841 0.000013 1.117842
O -2.941200 0.000291 0.653775
C 0.030867 -1.620629 -0.556964
O 0.035941 -2.600351 -1.162593
C 0.031150 1.620523 -0.557121
O 0.036361 2.600171 -1.162867
H -0.154314 0.000634 2.597050
H 0.568839 0.000507 2.401302

FREQS=   55   68   87   88 110 119 178 279 318 328 345 390 396 449 458 491 504 529 552 554 617
910 1354 1767 2084 2110 2169 2880 4334
TS036_7
12
E= -1837.74694965 ZPE=   35.05 Gcorr   -0.006062000
H -2.470820 0.001547 -0.900630
Co 0.023598 0.000059 0.143331
C -1.898393 0.000735 0.052985
C 1.809685 -0.000645 0.471561
O -2.474782 0.000266 1.117921
O 2.941189 -0.001095 0.653816
C -0.030420 1.620703 -0.556896
O -0.035254 2.600456 -1.162478
C -0.031619 -1.620445 -0.557243
O -0.037092 -2.600060 -1.163037
H -0.568752 -0.001025 2.401360
H 0.154398 -0.000732 2.597119
FREQS=   55   68   87   88 110 119 178 279 318 328 345 390 396 449 458 491 504 529 552 554 617
910 1354 1767 2084 2110 2169 2880 4334
TS037_7
12
E= -1837.75965805 ZPE=   41.21 Gcorr   0.006801000
H -0.958621 2.370310 -0.206992
Co 0.169436 -0.029515 -0.064468
C -1.268014 1.326721 -0.350341
C 1.243666 1.365051 -0.014314
O -2.355907 1.024351 0.525791
O 1.917032 2.293964 0.042327
C -1.154256 -1.249548 -0.080505
O -1.957812 -2.063103 -0.108771
C 1.452598 -1.221219 0.094650
O 2.306307 -1.990739 0.162032
H -1.552527 1.225654 -1.413393
H -2.984545 1.759111 0.493053
FREQS=   43   75 100 106 150 210 254 282 350 406 429 449 484 516 532 592 604 773 1033 1193 1231
1362 1501 2101 2113 2191 2944 3028 3775
TS038_7
12
E= -1837.75103295 ZPE=   36.54 Gcorr   -0.001702000
H -2.254052 -0.000001 -1.124484
Co 0.051982 0.000000 0.247838
C -1.892384 0.000000 -0.071713
C 1.858775 0.000000 0.403251
O -2.682108 0.000001 0.842193
O 3.005249 -0.000001 0.418998
C -0.013472 1.627434 -0.454706
O -0.049758 2.636494 -1.008181
C -0.013474 -1.627434 -0.454706
O -0.049760 -2.636494 -1.008181
H 0.091058 0.000002 2.050549
H -0.666161 0.000000 1.890946
FREQS=   48   80   90   94 106 181 278 286 377 380 420 446 462 483 523 529 546 562 607 720 902
1120 1358 1779 2089 2112 2166 2868 3940
TS039_7
12
E= -1837.75103295 ZPE=   36.54 Gcorr   -0.001702000
H -2.254052 0.000008 -1.124484
Co 0.051982 0.000000 0.247838
C -1.892384 0.000006 -0.071713
C 1.858775 -0.000005 0.403251
O -2.682108 0.000008 0.842192
O 3.005249 -0.000008 0.418997
C -0.013468 1.627433 -0.454706
O -0.049750 2.636494 -1.008181
C -0.013478 -1.627434 -0.454706
O -0.049769 -2.636494 -1.008181
H 0.091058 -0.000003 2.050548
H -0.666161 0.000004 1.890945
FREQS=   48   80   90   94 106 181 278 286 377 380 420 446 462 483 523 529 546 562 607 720 902
1120 1358 1779 2089 2112 2166 2868 3940
TS040_7
12
E= -1837.75103295 ZPE=   36.54 Gcorr   -0.001702000
H -2.254052 -0.000001 -1.124484
Co 0.051982 0.000000 0.247838
C -1.892384 0.000000 -0.071713
C 1.858775 0.000000 0.403251

O -2.682108 0.000001 0.842193
O 3.005249 -0.000001 0.418998
C -0.013472 1.627434 -0.454706
O -0.049758 2.636494 -1.008181
C -0.013474 -1.627434 -0.454706
O -0.049760 -2.636494 -1.008181
H 0.091058 0.000002 2.050549
H -0.666161 0.000000 1.890946
FREQS= 48 80 90 94 106 181 278 286 377 380 420 446 462 483 523 529 546 562 607 720 902
1120 1358 1779 2089 2112 2166 2868 3940
TS041_7
12
E= -1837.75103295 ZPE= 36.54 Gcorr -0.001702000
H -2.254052 0.000008 -1.124484
Co 0.051982 0.000000 0.247838
C -1.892384 0.000006 -0.071713
C 1.858775 -0.000005 0.403251
O -2.682108 0.000008 0.842192
O 3.005249 -0.000008 0.418997
C -0.013468 1.627433 -0.454706
O -0.049750 2.636494 -1.008181
C -0.013478 -1.627434 -0.454706
O -0.049769 -2.636494 -1.008181
H 0.091058 -0.000003 2.050548
H -0.666161 0.000004 1.890945
FREQS= 48 80 90 94 106 181 278 286 377 380 420 446 462 483 523 529 546 562 607 720 902
1120 1358 1779 2089 2112 2166 2868 3940
TS042_7
12
E= -1837.75244887 ZPE= 37.82 Gcorr -0.000259000
H -0.808366 0.130808 1.635285
Co -0.097354 0.032087 0.228144
C -1.681017 -0.402862 0.697208
C 0.240041 -1.278947 -0.913005
O -2.793498 -0.694278 0.904954
O 0.401979 -2.127110 -1.678507
C -0.291349 1.617888 -0.606553
O -0.543165 2.534633 -1.257091
C 2.840022 0.037777 0.661976
O 1.690445 0.189480 1.054026
H 3.066440 -0.257886 -0.373477
H 3.678224 0.195778 1.353500
FREQS= 56 63 82 86 117 139 223 287 330 335 385 426 470 495 498 541 563 614 898 1142 1263
1525 1735 1821 2033 2080 2131 3005 3111
TS043_7
12
E= -1837.75858294 ZPE= 41.46 Gcorr 0.006241000
H 1.912356 0.732848 1.427891
Co -0.188500 -0.050133 0.117465
C 1.495971 1.059653 0.461954
C -0.967346 1.536452 -0.006844
O 2.446530 0.995674 -0.590026
O -1.447162 2.572385 -0.124785
C 0.921273 -1.431131 0.086924
O 1.672795 -2.295101 -0.018643
C -1.660528 -0.990969 -0.090729
O -2.630408 -1.604869 -0.174018
H 2.827854 0.108774 -0.614386
H 1.279028 2.123233 0.566878
FREQS= 35 54 86 99 140 184 247 331 370 410 414 461 484 525 533 583 602 792 1035 1151 1278
1403 1492 2086 2106 2176 2981 3122 3820
TS044_7
12
E= -1837.75839078 ZPE= 41.17 Gcorr 0.006220000
H 1.837755 -0.886760 1.491101
Co -0.207958 0.000000 0.228875
C 1.690142 0.000001 0.853529
C -1.978157 0.000000 0.093793
O 2.592710 0.000000 -0.246480
O -3.125739 0.000002 0.162434
C 0.042461 1.690317 -0.239108
O 0.245435 2.717210 -0.712905
C 0.042461 -1.690318 -0.239108
O 0.245434 -2.717211 -0.712904
H 3.495199 0.000000 0.102389
H 1.837755 0.886762 1.491101

```
FREQS=   37   71   93   95  123  207  255  294  380  404  460  470  482  523  543  576  591  828 1038 1179 1187
1355 1513 2093 2104 2173 2954 2991 3775
TS045_7
12
E= -1837.75839078 ZPE=    41.17 Gcorr    0.006220000
H -1.837755 -0.886760 1.491101
Co 0.207958 0.000000 0.228875
C -1.690142 0.000001 0.853529
C -0.042461 -1.690318 -0.239108
O -2.592710 0.000000 -0.246480
O -0.245434 -2.717211 -0.712904
C -0.042461 1.690317 -0.239108
O -0.245435 2.717210 -0.712905
C 1.978157 0.000000 0.093793
O 3.125739 0.000001 0.162434
H -1.837755 0.886762 1.491100
H -3.495199 0.000000 0.102389
FREQS=   37   71   93   95  123  207  255  294  380  404  460  470  482  523  543  576  591  828 1038 1179 1187
1355 1513 2093 2104 2173 2954 2991 3775
TS046_7
12
E= -1837.75839078 ZPE=    41.17 Gcorr    0.006220000
H 1.837755 -0.886760 1.491101
Co -0.207958 0.000000 0.228875
C 1.690142 0.000001 0.853529
C -1.978157 0.000000 0.093793
O 2.592710 0.000000 -0.246480
O -3.125739 0.000002 0.162434
C 0.042461 1.690317 -0.239108
O 0.245435 2.717210 -0.712905
C 0.042461 -1.690318 -0.239108
O 0.245434 -2.717211 -0.712904
H 3.495199 0.000000 0.102389
H 1.837755 0.886762 1.491101
FREQS=   37   71   93   95  123  207  255  294  380  404  460  470  482  523  543  576  591  828 1038 1179 1187
1355 1513 2093 2104 2173 2954 2991 3775
TS047_7
12
E= -1837.75839078 ZPE=    41.17 Gcorr    0.006220000
H -1.837755 -0.886760 1.491101
Co 0.207958 0.000000 0.228875
C -1.690142 0.000001 0.853529
C -0.042461 -1.690318 -0.239108
O -2.592710 0.000000 -0.246480
O -0.245434 -2.717211 -0.712904
C -0.042461 1.690317 -0.239108
O -0.245435 2.717210 -0.712905
C 1.978157 0.000000 0.093793
O 3.125739 0.000001 0.162434
H -1.837755 0.886762 1.491100
H -3.495199 0.000000 0.102389
FREQS=   37   71   93   95  123  207  255  294  380  404  460  470  482  523  543  576  591  828 1038 1179 1187
1355 1513 2093 2104 2173 2954 2991 3775
TS048_7
12
E= -1837.75086728 ZPE=    36.78 Gcorr   -0.001283000
H -0.619786 0.400093 -1.998958
Co -0.204098 -0.000118 -0.391874
C 0.766733 1.512967 -0.511770
C -1.967098 -0.000551 0.027474
O 1.405860 2.465485 -0.562165
O -3.071797 -0.000791 0.344715
C 0.226627 -0.000002 1.498340
O 1.338567 0.000455 1.953043
C 0.767796 -1.512510 -0.511742
O 1.407640 -2.464549 -0.562141
H -0.619790 -0.400712 -1.998847
H -0.656274 -0.000419 2.176973
FREQS=   31   62   96  114  124  228  310  317  347  357  410  469  488  491  506  522  564  569  624  847  898
1360 1443 1804 2101 2112 2169 2857 3505
TS049_7
12
E= -1837.75714280 ZPE=    39.56 Gcorr    0.005058000
H 2.770215 0.029536 0.268880
Co 0.155756 -0.126637 -0.145296
C 0.379127 1.089731 1.084870
C 2.137464 -0.208104 -0.594476
```

O 0.517258 1.833113 1.952035
O 1.538064 -1.352824 -0.652311
C -0.937584 0.845268 -1.131876
O -1.813578 1.619845 -1.300516
C -1.044586 -1.333099 0.482189
O -1.767334 -2.045103 1.012714
H 2.379663 0.327779 -1.523360
H -0.357089 0.258862 -1.962147
FREQS= 59 79 90 132 138 166 283 379 393 419 446 467 496 512 539 596 670 752 1073 1119 1217
1364 1585 1946 2116 2167 2374 3004 3093
TS050_7
12
E= -1837.74884701 ZPE= 39.85 Gcorr -0.002267000
H -1.341518 -2.437106 -0.245425
Co 0.498105 -0.295409 0.211607
C -0.642635 -2.047660 0.507724
C 0.454623 1.365261 0.691682
O -0.995794 -0.844792 1.055299
O 0.405775 2.465269 1.019439
C 1.949332 -0.151440 -0.726455
O 2.910740 -0.088533 -1.361422
C -3.377746 0.296755 -0.674515
O -2.621787 0.768634 -1.383461
H -0.363313 -2.824074 1.232757
H 0.363066 -1.944884 -0.130176
FREQS= 17 24 54 74 83 100 102 133 328 405 413 471 489 505 558 613 677 1109 1170 1254 1325
1535 1741 2095 2160 2165 2200 3008 3065
TS051_7
12
E= -1837.75567314 ZPE= 39.70 Gcorr 0.005127000
H -0.222490 0.002503 2.425424
Co -0.124919 0.000119 -0.176144
C 0.457620 0.001665 1.562735
C 1.424015 0.002846 -1.044750
O 1.675375 0.002029 2.058266
O 2.376903 0.004515 -1.695971
C -1.115317 -1.476302 -0.108843
O -1.770652 -2.421847 -0.131490
C -1.122213 1.471905 -0.108407
O -1.781975 2.414370 -0.131088
H 2.325612 0.001345 1.334220
H -0.592155 -0.000272 -1.605913
FREQS= 56 68 91 108 123 313 326 331 408 453 455 477 512 516 547 633 671 705 757 1052 1246
1362 1493 1949 2081 2102 2154 3068 3716
TS052_7
12
E= -1837.75550685 ZPE= 39.09 Gcorr 0.004969000
H 2.769066 0.069785 -0.092800
Co 0.075872 -0.021171 -0.200815
C 0.464862 1.387243 0.816203
C 2.052601 -0.077763 -0.911244
O 0.651159 2.242101 1.562387
O 1.426374 -1.185938 -1.029446
C -1.464869 0.422712 -0.850887
O -2.585736 0.725991 -1.009417
C -0.445306 -1.328193 0.898709
O -0.751346 -2.151491 1.634830
H 2.156892 0.594093 -1.774378
H -0.541840 0.438426 -1.694326
FREQS= 76 84 97 121 143 231 306 344 392 421 459 484 487 520 545 579 596 700 1055 1148 1223
1404 1599 1933 2014 2116 2164 3007 3091
TS053_7
12
E= -1837.75063650 ZPE= 36.98 Gcorr 0.000272000
H 1.815631 1.851799 0.608590
Co -0.099807 0.034852 0.321130
C 1.733973 0.744123 0.495318
C -0.546915 1.554999 -0.497463
O 2.728768 0.070352 0.566419
O -0.793287 2.514487 -1.081212
C 0.597601 -1.310566 -0.641380
O 1.042025 -2.129316 -1.309559
C -1.798099 -0.634919 0.370352
O -2.861387 -1.058561 0.397055
H 0.163100 -0.437760 1.858254
H -0.132248 0.347444 1.920058

FREQS= 54 82 90 109 116 246 305 352 358 424 449 466 471 484 527 545 613 622 664 873 993
1373 1609 1800 2111 2131 2178 2824 2997
TS054_7
12
E= -1837.74654389 ZPE= 37.39 Gcorr -0.003406000
H 0.045336 -1.336048 -1.723587
Co -0.092500 0.237557 -0.206713
C 0.192940 -2.124627 -0.949634
C 0.016515 -0.145294 1.531968
O 1.268129 -2.282660 -0.404984
O 0.051473 -0.278845 2.670549
C 1.372759 1.219967 -0.365497
O 2.227396 1.974103 -0.503537
C -1.827813 0.408949 -0.346700
O -2.940623 0.681709 -0.459317
H -0.681589 -2.770186 -0.740077
H -0.243651 0.783767 -1.597601
FREQS= 33 54 73 80 97 100 119 258 321 340 347 452 476 485 506 564 577 645 762 1126 1255
1526 1778 1952 2102 2126 2180 2850 2972
TS055_7
12
E= -1837.74905629 ZPE= 40.25 Gcorr -0.000778000
H -2.935448 0.236662 -1.284195
Co 0.057819 0.080922 -0.148121
C -2.770199 -0.531999 -0.517080
C -0.436847 1.583796 0.463412
O -1.408086 -0.811140 -0.322169
O -0.835905 2.590884 0.860933
C 1.716218 0.597829 -0.357583
O 2.790908 0.949018 -0.585164
C 0.726603 -2.465863 1.225532
O 1.013282 -2.212687 0.149252
H -3.246390 -0.202207 0.417224
H -3.275507 -1.450519 -0.842258
FREQS= 30 48 64 71 81 102 107 130 165 260 311 446 474 496 563 600 700 1150 1173 1191 1483
1501 1508 2084 2139 2164 2993 3055 3070
TS056_7
12
E= -1837.74685521 ZPE= 36.19 Gcorr -0.002656000
H -0.681122 0.000007 2.098611
Co -0.135917 -0.000002 0.407504
C -1.908877 -0.000061 0.058678
C 1.846625 0.000092 0.498962
O -3.006803 -0.000101 -0.274029
O 2.621149 0.000033 -0.419457
C 0.107549 1.639209 -0.233074
O 0.261701 2.660679 -0.738730
C 0.107658 -1.639209 -0.233049
O 0.261904 -2.660661 -0.738710
H 0.093184 0.000037 2.167242
H 2.236352 0.000221 1.549830
FREQS= 47 65 91 101 109 143 269 308 352 369 413 447 464 476 510 511 549 558 595 747 877
1109 1367 1803 2098 2114 2169 2769 3883
TS057_7
12
E= -1837.74685521 ZPE= 36.19 Gcorr -0.002656000
H -2.236332 -0.000125 1.549827
Co 0.135915 0.000005 0.407495
C -1.846622 -0.000057 0.498953
C -0.107573 -1.639209 -0.233067
O -2.621153 -0.000033 -0.419459
O -0.261733 -2.660682 -0.738712
C -0.107635 1.639219 -0.233041
O -0.261867 2.660673 -0.738703
C 1.908876 0.000035 0.058677
O 3.006802 0.000060 -0.274033
H -0.093179 -0.000046 2.167276
H 0.681124 -0.000029 2.098643
FREQS= 47 65 91 101 109 143 269 308 352 369 413 447 464 476 510 511 549 558 595 747 877
1109 1367 1803 2098 2114 2169 2769 3883
TS058_7
12
E= -1837.74685521 ZPE= 36.19 Gcorr -0.002656000
H -0.681122 0.000007 2.098611
Co -0.135917 -0.000002 0.407504
C -1.908877 -0.000061 0.058678
C 1.846625 0.000092 0.498962

O -3.006803 -0.000101 -0.274029
O 2.621149 0.000033 -0.419457
C 0.107549 1.639209 -0.233074
O 0.261701 2.660679 -0.738730
C 0.107658 -1.639209 -0.233049
O 0.261904 -2.660661 -0.738710
H 0.093184 0.000037 2.167242
H 2.236352 0.000221 1.549830
FREQS= 47 65 91 101 109 143 269 308 352 369 413 447 464 476 510 511 549 558 595 747 877 1109 1367 1803 2098 2114 2169 2769 3883
TS059_7
12
E= -1837.74685521 ZPE= 36.19 Gcorr -0.002656000
H -2.236332 -0.000125 1.549827
Co 0.135915 0.000005 0.407495
C -1.846622 -0.000057 0.498953
C -0.107573 -1.639209 -0.233067
O -2.621153 -0.000033 -0.419459
O -0.261733 -2.660682 -0.738712
C -0.107635 1.639219 -0.233041
O -0.261867 2.660673 -0.738703
C 1.908876 0.000035 0.058677
O 3.006802 0.000060 -0.274033
H -0.093179 -0.000046 2.167276
H 0.681124 -0.000029 2.098643
FREQS= 47 65 91 101 109 143 269 308 352 369 413 447 464 476 510 511 549 558 595 747 877 1109 1367 1803 2098 2114 2169 2769 3883
TS060_7
12
E= -1837.74705830 ZPE= 36.07 Gcorr -0.002316000
H -1.806245 0.967395 1.820965
Co 0.167747 0.030961 0.356047
C -1.691816 0.317239 0.926055
C -0.376990 -1.396553 -0.632376
O -2.663098 -0.148284 0.388933
O -0.717915 -2.318923 -1.213537
C 0.017435 1.533232 -0.622818
O -0.078462 2.515586 -1.205291
C 1.966714 -0.259971 0.221547
O 3.097325 -0.425446 0.263261
H 0.500152 0.661107 1.650412
H 0.182057 -0.611598 1.693987
FREQS= 55 71 81 97 115 154 255 300 368 402 420 461 479 496 516 527 580 637 764 895 910 1368 1798 2035 2066 2145 2161 2197 2878
TS061_7
12
E= -1837.74705830 ZPE= 36.07 Gcorr -0.002316000
H -1.806250 0.967234 1.821063
Co 0.167754 0.030957 0.356050
C -1.691814 0.317185 0.926076
C 1.966726 -0.259922 0.221524
O -2.663094 -0.148262 0.388885
O 3.097343 -0.425367 0.263225
C 0.017386 1.533225 -0.622812
O -0.078545 2.515578 -1.205282
C -0.376963 -1.396574 -0.632356
O -0.717878 -2.318956 -1.213504
H 0.500158 0.661117 1.650409
H 0.182114 -0.611605 1.693988
FREQS= 55 71 81 97 115 154 255 300 368 402 420 461 479 496 516 527 580 637 764 895 910 1368 1798 2035 2066 2145 2161 2197 2878
TS062_7
12
E= -1837.74705830 ZPE= 36.07 Gcorr -0.002316000
H -1.806245 0.967395 1.820965
Co 0.167747 0.030961 0.356047
C -1.691816 0.317239 0.926055
C -0.376990 -1.396553 -0.632376
O -2.663098 -0.148284 0.388933
O -0.717915 -2.318923 -1.213537
C 0.017435 1.533232 -0.622818
O -0.078462 2.515586 -1.205291
C 1.966714 -0.259971 0.221547
O 3.097325 -0.425446 0.263261
H 0.500152 0.661107 1.650412
H 0.182057 -0.611598 1.693987

FREQS=   55   71   81   97 115 154 255 300 368 402 420 461 479 496 516 527 580 637 764 895 910
1368 1798 2035 2066 2145 2161 2197 2878
TS063_7
12
E= -1837.74705830 ZPE=   36.07 Gcorr   -0.002316000
H -1.806250 0.967234 1.821063
Co 0.167754 0.030957 0.356050
C -1.691814 0.317185 0.926076
C 1.966726 -0.259922 0.221524
O -2.663094 -0.148262 0.388885
O 3.097343 -0.425367 0.263225
C 0.017386 1.533225 -0.622812
O -0.078545 2.515578 -1.205282
C -0.376963 -1.396574 -0.632356
O -0.717878 -2.318956 -1.213504
H 0.500158 0.661117 1.650409
H 0.182114 -0.611605 1.693988
FREQS=   55   71   81   97 115 154 255 300 368 402 420 461 479 496 516 527 580 637 764 895 910
1368 1798 2035 2066 2145 2161 2197 2878
TS064_7
12
E= -1837.74976291 ZPE=   37.97 Gcorr   0.000578000
H 0.587422 -1.221784 0.968604
Co -0.185447 -0.052830 -0.156608
C 1.567096 -1.652327 0.560105
C 0.845096 1.057518 -1.059883
O 2.632049 -1.200002 0.893678
O 1.424784 1.767031 -1.753526
C -1.735215 -0.849296 -0.410769
O -2.742282 -1.314078 -0.716152
C -0.643780 1.050838 1.167676
O -0.977103 1.798883 1.970924
H 1.451551 -2.575804 -0.033883
H 0.069330 -0.831065 -1.408470
FREQS=   62   67   86   95 116 122 247 326 349 353 447 473 483 501 562 577 603 690 758 1196 1263
1476 1821 1977 2105 2126 2178 2505 2993
TS065_7
12
E= -1837.75543586 ZPE=   40.73 Gcorr   0.007359000
H 1.391467 -1.270039 -2.037234
Co -0.112938 -0.168973 -0.184415
C 1.141723 -1.438540 -0.984129
C 0.604799 1.393434 -0.606129
O -0.060659 -1.917725 -0.696912
O 1.046487 2.381514 -0.991758
C -1.883986 0.151562 0.215934
O -2.989216 0.435047 0.314866
C 0.547407 -0.083001 1.549516
O 1.687233 0.064482 1.874855
H 1.981759 -1.704682 -0.335129
H -0.254338 -0.310287 2.292010
FREQS=   69   79   98 116 130 176 300 384 397 416 460 480 495 530 579 629 789 850 1072 1205 1274
1279 1563 1844 2112 2159 2843 3035 3127
TS066_7
12
E= -1837.75496522 ZPE=   40.74 Gcorr   0.006979000
H -1.352189 -2.355699 -0.049115
Co -0.098721 -0.053577 -0.191139
C -1.226078 -1.547623 -0.779140
C 0.261109 -0.533842 1.562862
O -0.063455 -1.436964 -1.394295
O 1.143806 -1.332817 1.709912
C 1.469137 0.861461 -0.506863
O 2.398868 1.505979 -0.673348
C -1.279367 1.157344 0.288788
O -2.085324 1.963781 0.448340
H -2.133115 -1.257534 -1.321269
H -0.349196 -0.164059 2.412360
FREQS=   70   80   93 109 127 156 245 372 395 404 454 483 500 539 589 663 799 854 1086 1207 1291
1297 1562 1811 2103 2168 2914 3022 3108
TS067_7
12
E= -1837.74749927 ZPE=   38.00 Gcorr   -0.000178000
H -0.471803 -0.000040 1.774629
Co -0.029720 -0.000026 0.170423
C 3.095644 -0.000019 0.723627
C -0.219952 -1.478631 -0.801720

O 1.896794 -0.000098 0.511425
O -0.443769 -2.355689 -1.515628
C -1.510005 0.000255 1.030669
O -2.624246 0.000466 1.404243
C -0.219655 1.478405 -0.802003
O -0.443250 2.355402 -1.516056
H 3.823515 0.000097 -0.103558
H 3.490305 -0.000063 1.752214
FREQS= 50 66 83 86 121 163 190 283 293 327 375 400 481 489 501 553 578 581 1139 1142 1257
1546 1775 1835 2000 2083 2133 2979 3071
TS068_7
12
E= -1837.74419584 ZPE= 35.96 Gcorr -0.003033000
H 0.146936 0.465157 -1.664444
Co 0.098136 0.003392 -0.259039
C -1.755774 0.620255 -0.585085
C 0.514601 1.559692 0.566054
O -2.625716 -0.033252 -1.090533
O 0.756313 2.575232 1.037075
C -0.569671 -1.160605 0.944683
O -1.018531 -1.926474 1.667829
C 1.793369 -0.639669 -0.438086
O 2.821156 -1.076854 -0.683749
H -1.962889 1.668211 -0.264933
H -0.194647 -0.812218 -1.446936
FREQS= 51 66 80 92 103 134 255 325 359 389 428 450 478 497 509 535 579 636 744 872 909
1378 1817 2052 2090 2147 2157 2191 2832
TS069_7
12
E= -1837.75495522 ZPE= 40.80 Gcorr 0.008069000
H -1.739577 0.946761 1.743900
Co 0.119766 -0.037585 0.004242
C -1.618170 0.320255 0.871733
C 1.031336 1.413172 -0.001986
O -1.396783 -1.012407 0.976228
O 1.626469 2.403013 0.047212
C 1.502808 -1.130416 -0.090226
O 2.403285 -1.844307 -0.093521
C -1.775033 0.612919 -0.505347
O -1.714823 -0.495594 -1.226281
H -1.982763 1.575162 -0.959115
H -1.702152 -1.208353 -0.173456
FREQS= 32 91 93 110 141 311 384 392 448 460 486 572 611 631 730 824 881 898 1083 1162 1239
1310 1418 1459 2032 2087 2147 3234 3272
TS070_7
12
E= -1837.74988044 ZPE= 39.47 Gcorr 0.003593000
H 0.386438 0.382769 -1.625287
Co 0.118995 0.058386 -0.195415
C 0.908367 1.510093 0.518993
C -1.398586 0.751001 -0.920592
O 1.385319 2.452095 0.973553
O -2.648381 0.315945 -0.900255
C -0.687186 -0.960744 1.022094
O -1.253636 -1.615240 1.785599
C 1.483090 -1.045686 -0.524812
O 2.352266 -1.733889 -0.829743
H -1.419196 1.689930 -1.483356
H -2.698744 -0.528398 -0.422483
FREQS= 44 72 81 87 110 199 315 332 410 442 458 483 501 530 555 573 672 696 758 1050 1251
1330 1500 1969 2087 2108 2154 3103 3742
TS071_7
12
E= -1837.74542352 ZPE= 36.62 Gcorr -0.000455000
H -1.637791 0.882373 -2.007911
Co 0.176285 0.061920 -0.266133
C -1.583408 0.126964 -1.200968
C -0.400365 -1.170258 0.942240
O -2.568161 -0.462778 -0.827097
O -0.743689 -1.957273 1.693990
C 1.920451 -0.410370 -0.349618
O 3.025977 -0.650193 -0.529185
C 0.000981 1.588462 0.636951
O -0.096779 2.616489 1.136097
H -0.332490 -0.720620 -1.451686
H 0.645869 0.987658 -1.316873

FREQS=   69   75   90   96 113 189 288 327 395 416 427 457 485 513 538 541 611 697 845 868 1024
1375 1766 1949 2034 2135 2157 2199 2939
TS072_7
12
E= -1837.74542352 ZPE=   36.62 Gcorr   -0.000455000
H -0.332489 -0.720621 -1.451685
Co 0.176284 0.061921 -0.266133
C -1.583408 0.126964 -1.200968
C -0.400365 -1.170258 0.942240
O -2.568160 -0.462778 -0.827097
O -0.743688 -1.957275 1.693988
C 1.920450 -0.410371 -0.349617
O 3.025976 -0.650194 -0.529186
C 0.000981 1.588463 0.636951
O -0.096779 2.616490 1.136097
H 0.645871 0.987659 -1.316872
H -1.637790 0.882372 -2.007912
FREQS=   69   75   90   96 113 189 288 327 395 416 427 457 485 513 538 541 611 697 845 868 1024
1375 1766 1949 2034 2135 2157 2199 2939
TS073_7
12
E= -1837.74542352 ZPE=   36.62 Gcorr   -0.000455000
H -1.637791 0.882373 -2.007911
Co 0.176285 0.061920 -0.266133
C -1.583408 0.126964 -1.200968
C -0.400365 -1.170258 0.942240
O -2.568161 -0.462778 -0.827097
O -0.743689 -1.957273 1.693990
C 1.920451 -0.410370 -0.349618
O 3.025977 -0.650193 -0.529185
C 0.000981 1.588462 0.636951
O -0.096779 2.616489 1.136097
H -0.332490 -0.720620 -1.451686
H 0.645869 0.987658 -1.316873
FREQS=   69   75   90   96 113 189 288 327 395 416 427 457 485 513 538 541 611 697 845 868 1024
1375 1766 1949 2034 2135 2157 2199 2939
TS074_7
12
E= -1837.74542352 ZPE=   36.62 Gcorr   -0.000455000
H -0.332489 -0.720621 -1.451685
Co 0.176284 0.061921 -0.266133
C -1.583408 0.126964 -1.200968
C -0.400365 -1.170258 0.942240
O -2.568160 -0.462778 -0.827097
O -0.743688 -1.957275 1.693988
C 1.920450 -0.410371 -0.349617
O 3.025976 -0.650194 -0.529186
C 0.000981 1.588463 0.636951
O -0.096779 2.616490 1.136097
H 0.645871 0.987659 -1.316872
H -1.637790 0.882372 -2.007912
FREQS=   69   75   90   96 113 189 288 327 395 416 427 457 485 513 538 541 611 697 845 868 1024
1375 1766 1949 2034 2135 2157 2199 2939
TS075_7
12
E= -1837.74905174 ZPE=   39.18 Gcorr   0.003523000
H -1.551222 1.995623 -0.597400
Co 0.048594 0.009675 -0.161340
C -1.460769 0.903399 -0.619098
C 0.786415 1.439998 0.646853
O -2.625997 0.442845 -1.032929
O 1.242401 2.358696 1.169499
C -0.585039 -1.247953 0.943448
O -1.009688 -2.011586 1.694798
C 1.586033 -0.740977 -0.678388
O 2.536888 -1.230433 -1.099170
H -2.585009 -0.528232 -1.082769
H -0.284483 -0.331585 -1.578108
FREQS=   57   83   90 101 106 167 310 317 397 429 457 479 493 516 541 560 632 711 755 1028 1242
1343 1494 1948 2089 2106 2153 3088 3716
TS076_7
12
E= -1837.74906758 ZPE=   39.37 Gcorr   0.004734000
H 0.125440 -0.000005 2.405815
Co 0.137554 0.000001 -0.183599
C -0.523241 -0.000004 1.513920
C -1.411825 0.000006 -1.075779

O -1.800030 -0.000007 1.856180
O -2.356924 0.000010 -1.731710
C 1.132423 1.467159 -0.085119
O 1.795161 2.409127 -0.079405
C 1.132419 -1.467161 -0.085128
O 1.795154 -2.409131 -0.079419
H -1.927523 -0.000009 2.820148
H 0.642583 0.000003 -1.601313
FREQS= 62 69 98 107 125 303 324 330 408 448 452 485 512 513 548 616 642 702 713 1006 1264
1312 1495 1943 2095 2098 2155 3001 3714
TS077_7
12
E= -1837.74877402 ZPE= 39.36 Gcorr 0.004906000
H -0.749666 0.000345 -1.567942
Co -0.189353 -0.000111 -0.171066
C 0.349457 -0.001630 1.570178
C 0.633441 1.465223 -0.743802
O 1.603611 -0.001180 1.990048
O 1.148746 2.409806 -1.151997
C 0.639344 -1.460631 -0.747568
O 1.158352 -2.402093 -1.158277
C -1.949821 -0.003316 0.147888
O -3.097177 -0.005254 0.251164
H -0.352453 -0.003162 2.419142
H 1.671867 -0.002299 2.959904
FREQS= 70 74 94 105 126 311 315 337 411 443 454 488 512 513 539 616 646 698 714 982 1264
1307 1496 1939 2092 2102 2155 3012 3714
TS078_7
12
E= -1837.74811379 ZPE= 39.49 Gcorr 0.004855000
H 2.453406 -0.000205 0.631425
Co -0.022661 -0.000004 -0.156535
C 1.799690 0.000068 -0.251990
C -0.107982 1.463152 0.870389
O 2.585823 0.000410 -1.303624
O -0.163352 2.393141 1.546198
C -1.666470 0.000103 -0.831664
O -2.688473 0.000219 -1.359828
C -0.107868 -1.463489 0.869963
O -0.163126 -2.393700 1.545473
H 0.063633 0.000053 -1.671620
H 2.023603 0.000690 -2.099295
FREQS= 68 81 89 98 117 280 301 352 411 416 463 491 493 520 534 569 642 768 809 1047 1240
1375 1496 1843 2097 2109 2153 3063 3700
TS079_7
12
E= -1837.74811379 ZPE= 39.49 Gcorr 0.004855000
H -2.453413 0.000207 -0.631421
Co 0.022660 0.000002 0.156528
C -1.799690 0.000040 0.251989
C 0.107884 -1.463244 -0.870286
O -2.585814 -0.000111 1.303631
O 0.163169 -2.393296 -1.546013
C 0.107972 1.463408 -0.870067
O 0.163309 2.393564 -1.545647
C 1.666466 -0.000120 0.831664
O 2.688466 -0.000213 1.359836
H -0.063642 -0.000043 1.671610
H -2.023588 -0.000271 2.099297
FREQS= 68 81 89 98 117 280 301 352 411 416 463 491 493 520 534 569 642 768 809 1047 1240
1375 1496 1843 2097 2109 2153 3063 3700
TS080_7
12
E= -1837.74811379 ZPE= 39.49 Gcorr 0.004855000
H 2.453406 -0.000205 0.631425
Co -0.022661 -0.000004 -0.156535
C 1.799690 0.000068 -0.251990
C -0.107982 1.463152 0.870389
O 2.585823 0.000410 -1.303624
O -0.163352 2.393141 1.546198
C -1.666470 0.000103 -0.831664
O -2.688473 0.000219 -1.359828
C -0.107868 -1.463489 0.869963
O -0.163126 -2.393700 1.545473
H 0.063633 0.000053 -1.671620
H 2.023603 0.000690 -2.099295

FREQS= 68 81 89 98 117 280 301 352 411 416 463 491 493 520 534 569 642 768 809 1047 1240 1375 1496 1843 2097 2109 2153 3063 3700
TS081_7
12
E= -1837.74811379 ZPE= 39.49 Gcorr 0.004855000
H -2.453413 0.000207 -0.631421
Co 0.022660 0.000002 0.156528
C -1.799690 0.000040 0.251989
C 0.107884 -1.463244 -0.870286
O -2.585814 -0.000111 1.303631
O 0.163169 -2.393296 -1.546013
C 0.107972 1.463408 -0.870067
O 0.163309 2.393564 -1.545647
C 1.666466 -0.000120 0.831664
O 2.688466 -0.000213 1.359836
H -0.063642 -0.000043 1.671610
H -2.023588 -0.000271 2.099297
FREQS= 68 81 89 98 117 280 301 352 411 416 463 491 493 520 534 569 642 768 809 1047 1240 1375 1496 1843 2097 2109 2153 3063 3700
TS082_7
12
E= -1837.74219711 ZPE= 36.45 Gcorr -0.000952000
H -1.882120 1.621714 -0.689692
Co 0.083989 -0.058715 -0.171161
C -1.675349 0.552653 -0.910413
C -0.574527 -0.921157 1.244826
O -2.520736 -0.150409 -1.397389
O -1.025191 -1.536910 2.100338
C 1.735060 -0.682930 -0.551955
O 2.716904 -1.164738 -0.892195
C 0.536552 1.555506 0.510455
O 0.829312 2.579723 0.928435
H -0.258510 0.452583 -1.544824
H -0.259806 -1.334769 -0.815146
FREQS= 69 75 90 94 105 175 306 335 394 403 442 457 473 496 535 543 604 709 785 846 1011 1389 1791 1955 2063 2138 2149 2189 2880
TS083_7
12
E= -1837.74219711 ZPE= 36.45 Gcorr -0.000952000
H 1.882119 1.621714 -0.689694
Co -0.083989 -0.058714 -0.171161
C 1.675349 0.552653 -0.910413
C 0.574527 -0.921157 1.244826
O 2.520736 -0.150410 -1.397388
O 1.025192 -1.536909 2.100338
C -1.735060 -0.682930 -0.551954
O -2.716904 -1.164738 -0.892195
C -0.536552 1.555506 0.510455
O -0.829311 2.579723 0.928434
H 0.258510 0.452583 -1.544824
H 0.259806 -1.334769 -0.815146
FREQS= 69 75 90 94 105 175 306 335 394 403 442 457 473 496 535 543 604 709 785 846 1011 1389 1791 1955 2063 2138 2149 2189 2880
TS084_7
12
E= -1837.74219711 ZPE= 36.45 Gcorr -0.000952000
H -1.882120 1.621714 -0.689692
Co 0.083989 -0.058715 -0.171161
C -1.675349 0.552653 -0.910413
C -0.574527 -0.921157 1.244826
O -2.520736 -0.150409 -1.397389
O -1.025191 -1.536910 2.100338
C 1.735060 -0.682930 -0.551955
O 2.716904 -1.164738 -0.892195
C 0.536552 1.555506 0.510455
O 0.829312 2.579723 0.928435
H -0.258510 0.452583 -1.544824
H -0.259806 -1.334769 -0.815146
FREQS= 69 75 90 94 105 175 306 335 394 403 442 457 473 496 535 543 604 709 785 846 1011 1389 1791 1955 2063 2138 2149 2189 2880
TS085_7
12
E= -1837.74219711 ZPE= 36.45 Gcorr -0.000952000
H 1.882119 1.621714 -0.689694
Co -0.083989 -0.058714 -0.171161
C 1.675349 0.552653 -0.910413
C 0.574527 -0.921157 1.244826

O 2.520736 -0.150410 -1.397388
O 1.025192 -1.536909 2.100338
C -1.735060 -0.682930 -0.551954
O -2.716904 -1.164738 -0.892195
C -0.536552 1.555506 0.510455
O -0.829311 2.579723 0.928434
H 0.258510 0.452583 -1.544824
H 0.259806 -1.334769 -0.815146
FREQS= 69 75 90 94 105 175 306 335 394 403 442 457 473 496 535 543 604 709 785 846 1011
1389 1791 1955 2063 2138 2149 2189 2880
TS086_7
12
E= -1837.74517837 ZPE= 41.06 Gcorr 0.003624000
H -0.271319 2.469607 -0.583044
Co 0.556332 0.158023 0.326973
C 0.272001 2.008659 0.242433
C 1.748613 -0.107956 -0.861559
O -0.615282 1.400867 1.221538
O 2.531733 -0.239138 -1.703063
C 0.183719 -1.500296 0.760879
O -0.135939 -2.589455 0.975195
C -2.701549 0.254517 -0.593249
O -3.180174 -0.402535 -1.385165
H -1.503370 1.272973 0.839131
H 0.934312 2.703330 0.756592
FREQS= 28 54 84 90 109 139 155 175 379 399 434 476 527 556 616 655 727 755 940 1073 1165
1320 1493 2065 2127 2227 3099 3189 3668
TS087_7
12
E= -1837.73898733 ZPE= 40.37 Gcorr -0.002336000
H 0.867909 -2.526294 1.160852
Co 0.493906 -0.134781 0.164038
C 0.294611 -1.982646 0.409338
C 1.978133 -0.016743 -0.662377
O -0.834677 -1.256823 0.994317
O 3.006647 0.005561 -1.190943
C 0.175571 1.567931 0.445805
O 0.014848 2.679149 0.714444
C -3.306585 0.295635 -0.277971
O -3.209730 -0.072796 -1.350066
H -0.785077 -1.234391 1.961978
H -0.085391 -2.626022 -0.382646
FREQS= 16 26 44 59 78 91 97 117 371 393 425 476 525 552 601 639 657 726 917 1014 1168
1271 1492 2069 2134 2208 3100 3190 3782
TS088_7
12
E= -1837.74016285 ZPE= 37.17 Gcorr -0.000842000
H -0.298535 -2.360091 -1.037530
Co -0.172997 0.307159 -0.092746
C 0.537573 -1.638526 -1.015837
C -1.887307 0.291421 -0.518745
O 1.697351 -1.967257 -0.985793
O -3.007206 0.369981 -0.767385
C -0.136976 -0.442403 1.542615
O -0.152103 -0.821246 2.626676
C 1.326977 1.251100 -0.133070
O 2.248471 1.934771 -0.144909
H -0.614840 1.721325 -0.114351
H 0.250580 -0.554084 -1.422474
FREQS= 62 75 87 97 100 162 171 300 335 354 423 461 474 498 521 553 592 683 806 1149 1429
1495 1799 1968 2024 2112 2121 2174 2977
TS089_7
12
E= -1837.74016285 ZPE= 37.17 Gcorr -0.000841000
H -0.298554 -2.360050 -1.037603
Co -0.172996 0.307160 -0.092737
C 0.537560 -1.638493 -1.015883
C -0.136965 -0.442450 1.542603
O 1.697336 -1.967235 -0.985851
O -0.152084 -0.821328 2.626652
C 1.326977 1.251105 -0.133043
O 2.248469 1.934778 -0.144865
C -1.887306 0.291440 -0.518731
O -3.007207 0.370012 -0.767362
H 0.250577 -0.554036 -1.422489
H -0.614845 1.721324 -0.114278

FREQS=   62   75   87   97 100 162 171 300 335 354 423 461 474 498 521 553 592 683 806 1149 1429
1495 1799 1968 2024 2112 2121 2174 2977
TS090_7
12
E= -1837.74388819 ZPE=   39.00 Gcorr   0.003140000
H 1.483268 1.684256 1.397879
Co -0.115350 0.063916 0.197640
C 1.425972 0.730523 0.856148
C 0.661471 -1.074257 -0.952725
O 2.628009 0.168497 0.752360
O 1.165651 -1.797107 -1.691993
C -1.548221 -0.945501 0.542234
O -2.457715 -1.573411 0.859936
C -0.848281 1.519173 -0.549280
O -1.282169 2.467184 -1.037760
H -0.261575 0.380747 1.652791
H 3.316903 0.708343 1.174459
FREQS=   51   74   83   94 115 205 313 326 410 446 448 484 491 531 552 569 592 682 699 1014 1263
1294 1496 1928 2097 2107 2155 3045 3722
TS091_7
12
E= -1837.74071776 ZPE=   40.60 Gcorr   0.000296000
H 1.864370 -0.780209 -0.898188
Co -0.461180 -0.172481 -0.361259
C 0.239060 -1.908595 -0.466942
C -0.478674 1.568226 -0.579829
O 1.009219 -0.992114 -1.303607
O -0.428626 2.719209 -0.658185
C -1.743697 -0.337354 0.746742
O -2.574301 -0.495933 1.536142
C 2.391660 0.654527 1.663091
O 2.932975 0.091005 0.831530
H -0.216757 -2.661892 -1.107728
H 0.840024 -2.339073 0.334487
FREQS=   25   38   54   71   90   93 115 132 374 396 429 475 525 554 612 658 683 730 927 1036 1168
1283 1492 2067 2131 2177 3100 3189 3778
TS092_7
12
E= -1837.73750682 ZPE=   35.64 Gcorr   -0.002823000
H -0.000003 -0.951560 -1.686484
Co -0.000001 -0.098552 -0.479522
C 0.000004 -1.008039 1.323720
C 1.736801 -0.471801 -0.477805
O 0.000004 -0.405000 2.362732
O 2.854861 -0.720424 -0.537934
C 0.000000 1.643050 0.046408
O -0.000002 2.755491 0.308870
C -1.736802 -0.471802 -0.477801
O -2.854862 -0.720426 -0.537929
H -0.000007 0.308953 -1.935766
H 0.000005 -2.122054 1.316295
FREQS=   61   68   88   89   99 187 278 323 323 410 433 470 486 500 539 545 567 630 692 807 877
1343 1804 1926 2064 2125 2153 2192 2850
TS093_7
12
E= -1837.73999029 ZPE=   37.90 Gcorr   0.000496000
H 2.633346 -0.130336 -1.801373
Co -0.042225 0.015689 -0.290628
C 2.809448 0.167666 -0.760298
C 0.122834 -1.539105 0.638287
O 1.883096 0.324615 0.026213
O 0.241504 -2.540099 1.196636
C -0.366871 1.600064 0.552077
O -0.568847 2.614341 1.058644
C -1.727361 -0.254692 -0.567943
O -2.855752 -0.447180 -0.694034
H 0.039328 -0.076630 -1.810710
H 3.839097 0.326346 -0.413358
FREQS=   38   84   93   98 106 234 250 317 327 354 402 417 455 459 476 520 541 599 756 1179 1263
1517 1741 1808 2078 2095 2144 3025 3140
TS094_7
12
E= -1837.73726916 ZPE=   35.78 Gcorr   -0.002191000
H -0.451652 -0.811634 2.312041
Co -0.150992 0.251723 -0.119013
C 0.401501 -0.477614 1.683690
C -0.131466 -1.420107 -0.822110

O 1.540332 -0.673770 2.018331
O -0.129456 -2.463318 -1.294905
C -1.866349 0.621739 0.113045
O -2.970994 0.914911 0.215628
C 1.387947 0.963360 -0.656175
O 2.341539 1.471172 -1.034737
H 0.084146 0.976351 1.161876
H -0.556885 0.922543 -1.405807
FREQS= 65 76 93 97 106 195 276 309 344 404 431 467 479 496 506 554 579 615 675 870 986
1364 1788 1895 2004 2133 2145 2190 2889
TS095_7
12
E= -1837.73801817 ZPE= 37.00 Gcorr -0.001285000
H -0.233230 -0.033644 -1.574725
Co -0.152527 0.033870 -0.101919
C 1.210245 -1.374056 0.914598
C -1.290825 -1.258214 -0.485459
O 2.403906 -1.186626 0.973497
O -2.024331 -2.076363 -0.824467
C -1.238672 1.301549 0.616650
O -1.946339 2.110250 1.018418
C 1.207662 1.013698 -0.696166
O 2.040110 1.642919 -1.171118
H 0.766576 -2.384576 0.939411
H 0.467645 -0.515575 1.318756
FREQS= 57 80 83 95 106 147 169 293 326 384 422 439 479 491 513 558 581 676 805 1121 1426
1525 1777 1925 2011 2113 2127 2176 2976
TS096_7
12
E= -1837.73600238 ZPE= 35.43 Gcorr -0.003288000
H 0.000001 -0.434282 -1.722463
Co 0.000000 0.147901 -0.364444
C 1.734618 -0.232828 -0.448182
C 0.000000 1.746524 0.483734
O 2.850285 -0.466752 -0.569524
O -0.000001 2.796283 0.942432
C -1.734617 -0.232829 -0.448182
O -2.850284 -0.466752 -0.569524
C 0.000000 -1.194775 1.151330
O -0.000001 -2.387735 1.029156
H 0.000001 -0.731170 2.167982
H 0.000000 0.855216 -1.698054
FREQS= 58 83 86 90 103 157 265 303 321 391 438 472 491 496 534 553 559 622 688 791 861
1344 1812 1932 2057 2129 2145 2187 2811
TS097_7
12
E= -1837.73600238 ZPE= 35.43 Gcorr -0.003288000
H 0.000004 -0.731170 2.167982
Co 0.000000 0.147901 -0.364444
C 0.000002 -1.194776 1.151329
C -0.000002 1.746524 0.483734
O 0.000001 -2.387736 1.029156
O -0.000003 2.796283 0.942432
C 1.734618 -0.232827 -0.448182
O 2.850285 -0.466749 -0.569524
C -1.734617 -0.232830 -0.448182
O -2.850284 -0.466754 -0.569523
H 0.000001 -0.434282 -1.722463
H 0.000000 0.855216 -1.698054
FREQS= 58 83 86 90 103 157 265 303 321 391 438 472 491 496 534 553 559 622 688 791 861
1344 1812 1932 2057 2129 2145 2187 2811
TS098_7
12
E= -1837.73600238 ZPE= 35.43 Gcorr -0.003288000
H 0.000001 -0.434282 -1.722463
Co 0.000000 0.147901 -0.364444
C 1.734618 -0.232828 -0.448182
C 0.000000 1.746524 0.483734
O 2.850285 -0.466752 -0.569524
O -0.000001 2.796283 0.942432
C -1.734617 -0.232829 -0.448182
O -2.850284 -0.466752 -0.569524
C 0.000000 -1.194775 1.151330
O -0.000001 -2.387735 1.029156
H 0.000001 -0.731170 2.167982
H 0.000000 0.855216 -1.698054

```
FREQS=   58   83   86   90  103  157  265  303  321  391  438  472  491  496  534  553  559  622  688  791  861
1344 1812 1932 2057 2129 2145 2187 2811
TS099_7
12
E= -1837.73600238 ZPE=    35.43 Gcorr   -0.003288000
H 0.000004 -0.731170 2.167982
Co 0.000000 0.147901 -0.364444
C 0.000002 -1.194776 1.151329
C -0.000002 1.746524 0.483734
O 0.000001 -2.387736 1.029156
O -0.000003 2.796283 0.942432
C 1.734618 -0.232827 -0.448182
O 2.850285 -0.466749 -0.569524
C -1.734617 -0.232830 -0.448182
O -2.850284 -0.466754 -0.569523
H 0.000001 -0.434282 -1.722463
H 0.000000 0.855216 -1.698054
FREQS=   58   83   86   90  103  157  265  303  321  391  438  472  491  496  534  553  559  622  688  791  861
1344 1812 1932 2057 2129 2145 2187 2811
TS100_7
12
E= -1837.74146828 ZPE=    38.81 Gcorr    0.002686000
H 1.483472 -1.986439 -0.646761
Co -0.063091 -0.005482 -0.139206
C 1.431591 -0.887358 -0.626523
C -0.796192 -1.433748 0.672548
O 2.591662 -0.352808 -0.995695
O -1.248804 -2.362008 1.183798
C 0.592289 1.287983 0.918075
O 1.062565 2.080319 1.607068
C -1.598511 0.728736 -0.681173
O -2.548709 1.207657 -1.117168
H 0.343507 0.410456 -1.505970
H 3.247712 -1.034953 -1.210279
FREQS=   54   82   89  100  105  164  310  324  393  429  454  474  492  512  532  543  566  667  722  973 1263
1301 1496 1988 2092 2105 2153 3015 3746
TS101_7
12
E= -1837.74434067 ZPE=    40.42 Gcorr    0.006055000
H 2.501984 0.910804 -0.673894
Co 0.041358 -0.041967 0.001182
C 1.029559 -0.774762 1.598646
C -1.753991 -0.533166 -0.293542
O 0.384549 -1.676762 0.939356
O -2.818895 -0.848927 -0.555226
C -0.224884 1.656284 0.200898
O -0.408319 2.770231 0.443977
C 1.630169 0.250776 -0.834139
O 1.535224 -0.528619 -1.756144
H 2.118561 -0.687586 1.476239
H 0.637193 -0.412285 2.558853
FREQS=   58   75   85  114  135  195  248  273  389  395  407  436  527  539  571  672  723  874 1074 1209 1263
1369 1586 1752 2096 2174 2976 2992 3069
TS102_7
12
E= -1837.73225373 ZPE=    37.60 Gcorr   -0.005123000
H 0.906945 -2.243605 1.339807
Co 0.395043 -0.036542 0.038480
C 0.059421 -1.832040 0.782766
C 0.099654 1.723835 0.304570
O -0.631132 -0.847673 1.324365
O 0.057262 2.860138 0.444326
C 1.980570 -0.341963 -0.545807
O 3.058041 -0.514611 -0.917612
C -2.546769 -0.305762 -1.012569
O -3.618279 -0.247397 -0.637825
H 0.399771 0.295687 -1.376255
H -0.457275 -2.533544 0.117678
FREQS=   19   37   53   74   77  107  134  178  274  380  407  430  439  511  539  592  660  721  794 1079 1208
1298 1564 2084 2109 2166 2214 3031 3121
TS103_7
12
E= -1837.74697590 ZPE=    41.84 Gcorr    0.009813000
H -1.153605 0.124678 -1.327405
Co 0.243750 -0.072119 -0.058536
C -1.744748 -0.699010 -0.782072
C 1.704352 -0.940677 0.201948
```

O -0.884236 -1.498316 -0.043899
O 2.672458 -1.549879 0.336201
C 0.992924 1.497609 -0.173977
O 1.436149 2.556891 -0.265747
C -2.569981 0.119037 0.204113
O -1.945894 0.892786 0.906890
H -3.660241 -0.019483 0.312043
H -2.290505 -1.231600 -1.571803
FREQS= 57 74 94 102 196 210 334 351 448 473 480 550 572 606 674 887 957 1065 1098 1248 1334
1377 1495 1782 2094 2151 2499 3006 3054
TS104_7
12
E= -1837.74024493 ZPE= 38.62 Gcorr 0.003110000
H -1.738154 1.741571 1.024238
Co -0.016824 -0.025476 0.141227
C -1.619750 0.660114 1.115740
C 1.676403 -0.576005 0.274633
O -0.705784 0.261699 1.996550
O 2.747745 -0.975086 0.379719
C -0.873492 -1.204358 -0.869019
O -1.386723 -1.976215 -1.551631
C 0.250844 1.477433 -0.823520
O 0.395165 2.395347 -1.498618
H -0.288213 -0.904504 1.359169
H -2.526657 0.061724 1.008321
FREQS= 66 78 86 114 128 131 309 370 404 438 471 472 503 512 531 543 578 790 883 1082 1199
1264 1544 1890 2104 2117 2169 3072 3170
TS105_7
12
E= -1837.73451171 ZPE= 35.67 Gcorr -0.001905000
H -0.540424 -2.315667 -1.005456
Co 0.124297 0.014408 0.048192
C -1.063162 -1.378104 -0.729159
C 1.345798 -1.222218 0.404052
O -2.261014 -1.291939 -0.833791
O 2.116112 -2.013913 0.711836
C 1.225026 1.419250 -0.381502
O 1.882350 2.263986 -0.785151
C -1.329574 0.924474 0.561036
O -2.235314 1.491894 0.964973
H 0.251753 0.028968 1.545665
H -0.152951 -0.162956 -1.430901
FREQS= 79 85 89 106 120 174 308 318 344 412 416 468 474 481 509 555 584 603 671 764 986
1385 1771 1857 2003 2136 2152 2198 2906
TS106_7
12
E= -1837.73451171 ZPE= 35.67 Gcorr -0.001905000
H 0.540424 -2.315667 -1.005456
Co -0.124297 0.014408 0.048192
C 1.063162 -1.378104 -0.729159
C 1.329574 0.924474 0.561036
O 2.261014 -1.291939 -0.833791
O 2.235314 1.491894 0.964973
C -1.225026 1.419250 -0.381502
O -1.882350 2.263986 -0.785151
C -1.345798 -1.222218 0.404052
O -2.116112 -2.013913 0.711836
H 0.152951 -0.162956 -1.430901
H -0.251753 0.028968 1.545665
FREQS= 79 85 89 106 120 174 308 318 344 412 416 468 474 481 509 555 584 603 671 764 986
1385 1771 1857 2003 2136 2152 2198 2906
TS107_7
12
E= -1837.73451171 ZPE= 35.67 Gcorr -0.001905000
H -0.540424 -2.315667 -1.005456
Co 0.124297 0.014408 0.048192
C -1.063162 -1.378104 -0.729159
C 1.345798 -1.222218 0.404052
O -2.261014 -1.291939 -0.833791
O 2.116112 -2.013913 0.711836
C 1.225026 1.419250 -0.381502
O 1.882350 2.263986 -0.785151
C -1.329574 0.924474 0.561036
O -2.235314 1.491894 0.964973
H 0.251753 0.028968 1.545665
H -0.152951 -0.162956 -1.430901

FREQS=   79   85   89 106 120 174 308 318 344 412 416 468 474 481 509 555 584 603 671 764 986
1385 1771 1857 2003 2136 2152 2198 2906
TS108_7
12
E= -1837.73451171 ZPE=   35.67 Gcorr   -0.001905000
H 0.540424 -2.315667 -1.005456
Co -0.124297 0.014408 0.048192
C 1.063162 -1.378104 -0.729159
C 1.329574 0.924474 0.561036
O 2.261014 -1.291939 -0.833791
O 2.235314 1.491894 0.964973
C -1.225026 1.419250 -0.381502
O -1.882350 2.263986 -0.785151
C -1.345798 -1.222218 0.404052
O -2.116112 -2.013913 0.711836
H 0.152951 -0.162956 -1.430901
H -0.251753 0.028968 1.545665
FREQS=   79   85   89 106 120 174 308 318 344 412 416 468 474 481 509 555 584 603 671 764 986
1385 1771 1857 2003 2136 2152 2198 2906
TS109_7
12
E= -1837.73389563 ZPE=   37.41 Gcorr   -0.002452000
H -0.167725 -0.010386 -1.934245
Co -0.167049 -0.059355 0.142748
C 0.764788 0.610938 -1.998856
C -1.623315 0.946564 -0.075275
O 1.839035 0.135988 -1.689659
O -2.605157 1.545218 -0.004332
C -0.327190 -1.835462 0.110182
O -0.481987 -2.969166 0.227402
C 1.151139 0.637863 1.155668
O 1.875398 1.043795 1.949837
H -0.770900 -0.237385 1.432448
H 0.638094 1.644271 -2.368707
FREQS=   46   57   64   88   95 119 164 294 310 333 412 431 453 460 501 509 520 666 689 1150 1256
1518 1776 2089 2103 2129 2176 2767 2998
TS110_7
12
E= -1837.73873627 ZPE=   40.02 Gcorr   0.002401000
H 2.107694 1.001216 1.520854
Co -0.066997 0.028006 -0.024324
C 2.401196 0.092155 0.963388
C -0.119636 -1.688294 -0.299426
O 1.725849 -0.061730 -0.264908
O -0.091796 -2.816310 -0.500639
C -1.787850 0.170464 0.363281
O -2.879458 0.221028 0.720116
C -0.039997 1.806707 -1.255983
O 0.148838 2.224775 -0.190322
H 3.475811 0.185301 0.756130
H 2.275689 -0.770976 1.638226
FREQS=   37   83   85 101 125 134 153 177 224 250 351 437 452 495 515 593 639 1062 1173 1184 1478
1501 1513 1995 2112 2164 2932 2984 3049
TS111_7
12
E= -1837.73856543 ZPE=   39.07 Gcorr   0.002610000
H -0.118964 -2.159925 1.373104
Co -0.104877 -0.029738 -0.195759
C 0.526800 -1.439047 0.849475
C -0.565357 1.340360 0.848647
O 1.757334 -1.814781 1.138535
O -0.855404 2.211640 1.543198
C 1.336035 0.697876 -0.895015
O 2.304597 1.090529 -1.387450
C -1.750402 -0.545057 -0.574184
O -2.794752 -0.934193 -0.866651
H -0.016982 -0.566817 -1.583933
H 2.390971 -1.220705 0.701736
FREQS=   49   76   82   92 105 121 315 347 358 445 472 474 504 533 573 590 626 687 690 1038 1241
1345 1501 1971 2079 2101 2147 3039 3728
TS112_7
12
E= -1837.73856543 ZPE=   39.07 Gcorr   0.002610000
H 0.016988 -0.566777 -1.583947
Co 0.104874 -0.029734 -0.195756
C -0.526791 -1.439036 0.849493
C 1.750392 -0.545071 -0.574188

O -1.757324 -1.814789 1.138537
O 2.794736 -0.934217 -0.866664
C -1.336047 0.697866 -0.895006
O -2.304605 1.090527 -1.387441
C 0.565368 1.340376 0.848631
O 0.855425 2.211655 1.543180
H 0.118978 -2.159888 1.373151
H -2.390963 -1.220730 0.701717
FREQS= 49 76 82 92 105 121 315 347 358 445 472 474 504 533 573 590 626 687 690 1038 1241 1345 1501 1971 2079 2101 2147 3039 3728
TS113_7
12
E= -1837.73856543 ZPE= 39.07 Gcorr 0.002610000
H -0.118964 -2.159925 1.373104
Co -0.104877 -0.029738 -0.195759
C 0.526800 -1.439047 0.849475
C -0.565357 1.340360 0.848647
O 1.757334 -1.814781 1.138535
O -0.855404 2.211640 1.543198
C 1.336035 0.697876 -0.895015
O 2.304597 1.090529 -1.387450
C -1.750402 -0.545057 -0.574184
O -2.794752 -0.934193 -0.866651
H -0.016982 -0.566817 -1.583933
H 2.390971 -1.220705 0.701736
FREQS= 49 76 82 92 105 121 315 347 358 445 472 474 504 533 573 590 626 687 690 1038 1241 1345 1501 1971 2079 2101 2147 3039 3728
TS114_7
12
E= -1837.73856543 ZPE= 39.07 Gcorr 0.002610000
H 0.016988 -0.566777 -1.583947
Co 0.104874 -0.029734 -0.195756
C -0.526791 -1.439036 0.849493
C 1.750392 -0.545071 -0.574188
O -1.757324 -1.814789 1.138537
O 2.794736 -0.934217 -0.866664
C -1.336047 0.697866 -0.895006
O -2.304605 1.090527 -1.387441
C 0.565368 1.340376 0.848631
O 0.855425 2.211655 1.543180
H 0.118978 -2.159888 1.373151
H -2.390963 -1.220730 0.701717
FREQS= 49 76 82 92 105 121 315 347 358 445 472 474 504 533 573 590 626 687 690 1038 1241 1345 1501 1971 2079 2101 2147 3039 3728
TS115_7
12
E= -1837.74716974 ZPE= 42.46 Gcorr 0.011378000
H 2.553087 -1.161621 1.169394
Co -0.238438 -0.196642 -0.026004
C 2.184121 -1.013505 0.148498
C -1.930009 -0.750860 -0.009786
O 0.941110 -1.771914 -0.075827
O -3.067452 -0.932067 -0.036362
C -0.712120 1.435184 0.042882
O -0.976925 2.555902 0.118452
C 1.582903 0.400089 -0.043614
O 2.265457 1.392820 -0.147118
H 0.892663 -2.602686 0.413407
H 2.945197 -1.309757 -0.581721
FREQS= 54 87 107 131 256 291 329 356 400 452 453 488 501 587 604 639 868 948 957 1138 1214 1332 1480 1809 2081 2138 3047 3119 3838
TS116_7
12
E= -1837.73866427 ZPE= 39.96 Gcorr 0.002952000
H 3.337031 0.237530 -0.878638
Co -0.032044 -0.007553 -0.004887
C 2.257302 0.345483 -1.052641
C -0.677048 1.579980 0.299722
O 1.628869 0.696868 0.156943
O -1.043243 2.644667 0.515230
C -1.602498 -0.729563 -0.407311
O -2.602699 -1.154401 -0.780730
C 0.478492 -1.504817 1.422441
O 0.861095 -2.017374 0.451108
H 2.114585 1.096850 -1.844614
H 1.923904 -0.635030 -1.458471

FREQS= 59 67 92 99 119 144 157 195 218 277 363 444 450 502 512 592 643 1047 1154 1184 1465 1494 1503 1974 2113 2165 2875 2996 3045
TS117_7
12
E= -1837.74279612 ZPE= 41.69 Gcorr 0.007550000
H -1.728516 -0.404579 1.557894
Co 0.462640 -0.146618 0.134705
C -1.394626 -0.536571 0.528659
C 0.533363 1.558842 0.134710
O -0.574354 -1.698792 0.338839
O 0.530313 2.712449 0.143501
C 2.146344 -0.462065 -0.235910
O 3.245022 -0.692280 -0.495142
C -2.398742 -0.314031 -0.522110
O -3.316220 0.478806 -0.430789
H -2.237604 -0.916930 -1.448238
H -0.921294 -2.598334 0.369932
FREQS= 37 59 82 96 140 219 299 392 402 428 461 471 517 575 606 660 905 993 1023 1088 1132 1306 1422 1795 2086 2146 2858 3146 3817
TS118_7
12
E= -1837.74276905 ZPE= 42.69 Gcorr 0.008761000
H -2.003148 -1.879824 0.000067
Co 0.655022 0.180847 -0.000106
C -2.156961 -0.805301 0.000014
C 2.122891 1.108892 0.000044
O -1.042153 -0.029093 -0.000240
O 3.066849 1.771247 0.000185
C 1.488863 -1.294154 0.000019
O 1.994900 -2.330102 0.000113
C -3.383143 -0.265540 0.000158
O -3.649364 1.072493 0.000087
H -4.276040 -0.878019 0.000350
H -2.798152 1.535236 -0.000124
FREQS= 35 36 99 124 140 215 258 400 430 461 463 519 521 596 685 699 852 857 1071 1172 1261 1369 1412 1728 2089 2141 3197 3246 3786
TS119_7
12
E= -1837.73787920 ZPE= 38.48 Gcorr 0.004331000
H -0.925320 -2.307065 1.162873
Co 0.000008 -0.017099 0.136395
C 0.000011 -2.006074 0.659086
C -1.776003 -0.020803 -0.173251
O 0.000016 -1.840058 -0.631740
O -2.902115 -0.081812 -0.370946
C -0.000029 1.737363 0.067933
O -0.000058 2.885909 0.131020
C 1.776017 -0.020760 -0.173251
O 2.902131 -0.081743 -0.370942
H 0.000010 -0.120919 1.649361
H 0.925340 -2.307070 1.162873
FREQS= 90 100 101 139 160 161 325 342 407 413 431 454 481 543 575 595 597 618 784 1081 1215 1322 1577 1831 2115 2131 2181 3033 3114
TS120_7
12
E= -1837.73816056 ZPE= 40.71 Gcorr 0.006212000
H -2.974175 2.118960 0.185963
Co 0.161278 0.011312 -0.141359
C -2.183453 1.394243 0.414694
C 1.398040 1.276081 0.041147
O -1.264155 1.245583 -0.394585
O 2.250607 2.046031 0.108437
C -1.190808 -1.371390 -0.340886
O -2.236168 -1.305444 0.286702
C 1.278869 -1.264644 0.079010
O 2.012796 -2.136915 0.268503
H -1.057343 -2.243278 -1.026409
H -2.243544 0.819128 1.340903
FREQS= 45 57 93 107 203 219 268 291 346 405 418 448 471 503 561 611 613 860 1104 1267 1382 1521 1698 1722 2078 2136 2816 3053 3180
TS121_7
12
E= -1837.73423293 ZPE= 39.29 Gcorr 0.002721000
H 2.486825 2.370842 1.415698
Co -0.197435 0.034750 -0.094379
C 2.110014 1.519202 0.835634
C -1.748831 0.976128 -0.199849

O 0.900888 1.446060 0.608523
O -2.728464 1.462080 -0.558395
C 1.213835 -0.742672 -0.934406
O 2.364914 -1.069906 -0.788932
C -0.813853 -1.494658 0.444747
O -1.198945 -2.455187 0.959651
H 0.762010 -0.671211 -1.992455
H 2.807777 0.749765 0.481456
FREQS= 54 57 85 100 115 180 245 284 353 373 394 426 440 471 526 536 559 810 1144 1208 1279
1509 1690 1808 2072 2123 2480 3024 3141
TS122_7
12
E= -1837.73381889 ZPE= 38.46 Gcorr 0.002603000
H 2.355091 -0.714591 -0.156232
Co -0.286261 0.059298 0.150532
C 0.616959 -0.947334 -1.059408
C -0.546673 1.815847 -0.155822
O 1.844539 -1.183698 -1.169649
O -0.572120 2.876632 -0.602501
C -1.836569 -0.907793 0.114305
O -2.746858 -1.576332 0.331954
C 1.225910 0.132661 1.122876
O 2.420684 -0.197848 0.906801
H 1.024296 0.466347 2.168316
H 0.021947 -1.263113 -1.941006
FREQS= 41 46 85 87 224 248 328 359 398 426 444 470 497 512 598 613 707 847 886 1308 1328
1342 1624 1652 1809 2082 2129 2879 2935
TS123_7
12
E= -1837.73817033 ZPE= 39.99 Gcorr 0.006982000
H -0.796881 -1.562067 1.743687
Co -0.054140 -0.056740 -0.364872
C 0.070177 -1.505241 1.069980
C -1.905475 -0.037450 -0.283083
O -0.035038 -1.963028 -0.132268
O -2.985539 -0.013516 0.095336
C 0.071158 1.620907 0.111631
O 0.146261 2.702847 0.497034
C 1.789707 -0.076003 -0.446321
O 2.775873 -0.151775 0.220102
H 1.841988 -0.089391 -1.599848
H 1.050806 -1.426067 1.552826
FREQS= 80 95 100 121 162 265 290 352 376 424 438 452 491 514 550 576 669 843 1082 1208 1222
1367 1585 1873 2108 2156 2463 3006 3106
TS124_7
12
E= -1837.72966996 ZPE= 39.46 Gcorr -0.001292000
H 1.396045 -1.216654 -2.090436
Co -0.414979 0.016921 -0.273578
C 3.140165 0.439595 0.921138
C -1.290624 -1.348345 0.280965
O 2.485106 -0.574416 1.007830
O -1.861805 -2.298075 0.604901
C -1.423637 1.318510 0.391340
O -2.150490 2.162403 0.682836
C 0.792779 -0.552983 -1.457383
O 0.974812 0.680497 -1.432945
H 3.967987 0.660159 1.624102
H 2.947322 1.195684 0.135608
FREQS= 30 36 61 85 90 115 133 162 179 371 377 433 475 506 556 625 685 816 1210 1215 1274
1541 1578 1826 2084 2143 2925 3000 3074
TS125_7
12
E= -1837.73198330 ZPE= 39.46 Gcorr 0.001098000
H 2.423252 0.860721 2.309581
Co -0.097935 -0.110237 -0.153064
C 0.253611 -1.378482 -1.342685
C -0.456362 1.651982 -0.185974
O 1.118335 -0.569858 -1.703171
O -0.826779 2.709975 -0.459605
C -1.545719 -0.667431 0.594737
O -2.538235 -1.058764 1.036960
C 2.248123 0.379614 1.331301
O 1.546484 -0.617914 1.230682
H 0.074254 -2.333029 -1.857036
H 2.750388 0.827108 0.456980

FREQS= 34 59 86 87 112 136 171 179 233 316 376 425 480 499 528 589 691 829 1154 1212 1261
1528 1615 1743 2072 2121 2962 3047 3057
TS126_7
12
E= -1837.73590968 ZPE= 38.61 Gcorr 0.005157000
H -0.922953 -2.287836 -0.986924
Co 0.000000 -0.012851 0.168473
C 0.000000 -2.008627 -0.462015
C -1.767602 -0.057940 -0.207754
O 0.000000 -1.854315 0.820840
O -2.903400 -0.087562 -0.359655
C 1.767602 -0.057939 -0.207754
O 2.903400 -0.087562 -0.359655
C 0.000000 1.727442 0.022737
O -0.000001 2.877174 0.018914
H 0.922953 -2.287836 -0.986925
H 0.000000 0.523156 1.590250
FREQS= 95 97 115 121 159 296 303 377 397 425 449 501 517 543 548 557 586 647 729 1068 1208
1348 1589 1830 2119 2121 2174 3012 3082
TS127_7
12
E= -1837.73204214 ZPE= 38.70 Gcorr 0.001834000
H 0.017100 -0.611780 -1.563350
Co 0.113268 -0.033406 -0.194592
C -0.598283 -1.447596 0.768883
C 1.757312 -0.548108 -0.565723
O -1.881255 -1.673028 0.997340
O 2.802619 -0.944666 -0.851211
C -1.301245 0.762496 -0.903121
O -2.235573 1.209019 -1.407280
C 0.570983 1.297510 0.897768
O 0.861934 2.142391 1.624621
H 0.010659 -2.252792 1.218549
H -2.040399 -2.489017 1.504171
FREQS= 43 76 86 96 103 116 311 343 353 444 469 477 501 542 574 594 601 642 704 973 1260
1312 1492 1984 2086 2101 2149 2965 3678
TS128_7
12
E= -1837.72576966 ZPE= 39.14 Gcorr -0.004279000
H -1.735976 -0.578707 -0.743878
Co 0.629731 -0.222398 0.149465
C -2.774904 -0.406435 -1.104786
C 1.569641 0.896598 -0.873999
O -3.477505 0.450852 -0.628848
O 2.280666 1.596579 -1.444220
C -0.167139 0.916322 1.162486
O -0.683602 1.640459 1.895474
C 0.382117 -1.859432 0.820826
O 1.097146 -2.056058 -0.180361
H 0.058903 -2.683377 1.471435
H -3.117587 -1.070147 -1.926626
FREQS= 19 26 43 71 88 92 113 124 199 371 375 433 468 502 551 619 680 817 1208 1216 1280
1545 1585 1840 2097 2152 2854 2939 3067
TS129_7
12
E= -1837.73390912 ZPE= 39.18 Gcorr 0.004017000
H 0.392589 0.658475 -1.163489
Co -0.369571 -0.048589 -0.132308
C 1.432322 -0.848415 -0.390938
C -0.802537 1.611428 -0.013947
O 0.545316 -1.620533 0.190491
O -1.025680 2.733749 0.114552
C -2.017965 -0.727379 0.155250
O -3.071879 -1.172354 0.127937
C 2.476318 -0.173021 0.456963
O 3.431112 0.406347 -0.011892
H 1.727479 -1.043352 -1.428314
H 2.298570 -0.256588 1.551442
FREQS= 53 63 93 104 136 201 306 365 388 414 452 499 521 531 586 611 704 977 1054 1092 1281
1355 1447 1810 2074 2120 2171 2921 3080
TS130_7
12
E= -1837.73591212 ZPE= 40.35 Gcorr 0.006218000
H -2.774939 -0.163621 -0.147666
Co -0.051390 -0.025822 -0.009049
C 0.161239 -1.884611 0.868533
C -0.261472 1.699558 0.276698

O -0.460343 -1.031869 1.599205
O -0.352288 2.808171 0.574096
C 1.754372 -0.026580 -0.368119
O 2.831424 -0.043530 -0.761098
C -1.760606 -0.254703 -0.575065
O -1.518205 -0.678673 -1.686304
H 1.191034 -2.167797 1.122154
H -0.394478 -2.606147 0.250357
FREQS= 57 83 91 118 135 184 254 342 366 406 420 465 491 525 532 647 702 885 1039 1216 1240
1385 1588 1740 2102 2146 2991 2994 3077
TS131_7
12
E= -1837.73597413 ZPE= 40.40 Gcorr 0.006701000
H 0.131125 0.101451 2.638652
Co 0.102186 -0.017683 -0.067663
C 0.310385 -0.730255 1.940232
C -1.432384 -0.908571 -0.345621
O 1.390647 -0.815565 1.296910
O -2.377207 -1.454851 -0.708590
C -0.560724 1.569494 0.147578
O -0.983706 2.636215 0.273768
C 1.544699 0.501606 -1.052095
O 1.471230 -0.601208 -1.570087
H -0.343619 -1.606962 2.056812
H 2.273898 1.272578 -1.345112
FREQS= 65 89 95 126 158 180 244 294 369 389 437 470 496 523 567 633 660 840 1087 1219 1231
1456 1632 1699 2087 2133 2987 3033 3064
TS132_7
12
E= -1837.73289688 ZPE= 39.14 Gcorr 0.004177000
H -1.840850 0.471814 1.472236
Co 0.371669 0.036288 0.117425
C -1.543895 -0.170185 0.631621
C 1.315403 1.465474 -0.016912
O -0.779595 -1.212070 0.847364
O 1.911137 2.451106 -0.031722
C 1.711158 -1.130433 -0.181575
O 2.489307 -1.877808 -0.563715
C -2.541515 -0.229249 -0.496333
O -3.503323 0.502353 -0.558032
H -0.447824 1.046403 -0.565618
H -2.333484 -1.020278 -1.249062
FREQS= 54 76 96 102 134 211 304 372 388 406 459 482 520 546 590 624 692 969 1036 1090 1283
1356 1450 1820 2054 2117 2169 2925 3055
TS133_7
12
E= -1837.73401232 ZPE= 40.30 Gcorr 0.005903000
H 2.044708 -0.772716 1.074081
Co -0.126001 -0.035261 -0.101863
C 1.485373 0.624714 -0.833766
C -0.545285 1.584831 0.325772
O 2.623137 0.572181 -0.421672
O -0.817855 2.629064 0.736528
C -1.830588 -0.668329 -0.137991
O -2.904502 -0.963839 -0.418157
C 1.332021 -1.607267 1.031079
O 0.607023 -1.801543 0.030596
H 1.303926 0.996393 -1.881216
H 1.341839 -2.362224 1.828521
FREQS= 44 87 89 104 174 225 273 292 375 414 432 437 469 515 561 569 604 874 1105 1273 1315
1480 1633 1769 2083 2135 2734 3004 3121
TS134_7
12
E= -1837.73150472 ZPE= 39.10 Gcorr 0.003662000
H -0.263255 0.670807 1.369377
Co 0.306952 -0.040069 0.215505
C -1.494546 -0.699619 0.727560
C 0.892251 1.572976 0.072000
O -0.774679 -1.535744 0.030347
O 1.214011 2.670422 -0.058979
C 1.819910 -0.865644 -0.305171
O 2.812250 -1.421034 -0.434415
C -2.534359 0.156935 0.044603
O -2.660582 0.235184 -1.156991
H -1.671748 -0.899294 1.792884
H -3.180241 0.731826 0.745462

FREQS= 56 63 94 102 108 178 279 339 381 424 452 500 514 567 603 698 802 891 960 1073 1303
1406 1459 1812 2042 2120 2170 2904 3049
TS135_7
12
E= -1837.72131091 ZPE= 37.71 Gcorr -0.006333000
H 0.012093 0.478155 2.702492
Co -0.344161 0.191590 0.113894
C 0.643113 0.342126 1.816384
C -0.682211 -1.522017 0.124863
O 0.786886 1.339653 0.972628
O -0.846656 -2.660336 0.114910
C -1.760292 0.704361 -0.660516
O -2.827967 1.001372 -1.031777
C 3.398025 0.457023 -1.118720
O 2.741932 -0.465770 -0.995165
H 1.458996 -0.381712 1.921914
H -0.604103 1.122327 -1.156367
FREQS= 18 24 45 46 69 100 118 149 367 388 433 451 518 528 589 607 649 765 976 1076 1206
1314 1567 1864 2041 2138 2199 3025 3112
TS136_7
12
E= -1837.73109683 ZPE= 40.03 Gcorr 0.003756000
H -3.699663 0.838556 -0.540697
Co 0.073222 0.033119 -0.018632
C -2.681017 0.646571 -0.900702
C -0.410729 -1.813905 0.180342
O -1.794811 0.377560 -0.097555
O -0.572656 -2.200773 1.316543
C 1.618473 -0.516907 -0.491513
O 2.632733 -0.851095 -0.939059
C 0.596983 1.691915 0.369730
O 0.997424 2.676581 0.814230
H -0.635017 -2.503153 -0.676362
H -2.486095 0.706162 -1.980292
FREQS= 51 68 83 98 124 152 204 230 344 374 390 441 461 499 568 591 603 880 1127 1257 1351
1527 1718 1774 2066 2124 2759 3017 3124
TS137_7
12
E= -1837.72111033 ZPE= 37.82 Gcorr -0.006175000
H -0.987201 1.994072 1.221541
Co 0.334540 -0.025830 0.199716
C 1.193419 1.198067 -0.699968
C -0.460849 1.112544 1.603848
O 1.703106 2.020274 -1.322558
O -1.006348 -0.071948 1.436451
C 1.404247 -1.310462 -0.077573
O 2.267161 -2.094525 -0.156889
C -3.526430 -0.024274 -0.551533
O -2.965655 -0.719013 -1.258016
H 0.184285 1.285601 2.473084
H 0.121914 -1.515828 -0.327499
FREQS= 12 21 53 64 76 100 120 147 368 387 436 451 519 529 594 609 650 767 994 1079 1205
1313 1569 1874 2042 2137 2202 3026 3114
TS138_7
12
E= -1837.73702717 ZPE= 41.67 Gcorr 0.009938000
H 2.269444 -0.959130 0.579644
Co -0.136801 0.005992 0.322588
C 1.549986 -0.387393 1.164625
C -1.546614 1.019318 -0.051661
O 1.403802 0.935562 0.794758
O -2.518593 1.628918 -0.135317
C 1.105530 0.856726 -0.960701
O 1.755012 0.106535 -1.629042
C -0.736874 -1.605094 -0.062546
O -1.053188 -2.658436 -0.396578
H 0.903741 1.918975 -1.142752
H 1.592017 -0.523618 2.244363
FREQS= 67 78 87 113 200 251 384 418 431 446 460 509 544 564 645 653 795 917 1077 1155 1185
1255 1498 1792 2098 2150 3073 3102 3202
TS139_7
12
E= -1837.72788717 ZPE= 37.14 Gcorr 0.001295000
H -1.061318 -1.053519 1.771816
Co -0.012051 -0.117686 -0.356369
C -0.054181 -0.720002 1.431911
C -0.296106 1.603171 0.074311

O 0.912583 -0.733432 2.137828
O -0.487528 2.679565 0.416760
C 1.794241 -0.181220 -0.708521
O 2.895543 -0.341121 -0.966271
C -1.714377 -0.546043 -0.743821
O -2.762634 -0.957855 -0.360090
H 0.050895 -1.608332 -0.436801
H -1.505365 -0.273324 -1.862153
FREQS= 74 79 87 98 179 230 287 360 384 392 418 455 477 490 530 627 661 729 823 926 1137
1287 1824 1904 1985 2144 2181 2340 2872
TS140_7
12
E= -1837.72788717 ZPE= 37.14 Gcorr 0.001295000
H -1.061006 -1.053443 1.771991
Co -0.012059 -0.117658 -0.356387
C -0.053913 -0.719980 1.431906
C -0.296430 1.603118 0.074319
O 0.912968 -0.733459 2.137663
O -0.488044 2.679445 0.416874
C 1.794250 -0.180975 -0.708567
O 2.895561 -0.340786 -0.966332
C -1.714345 -0.546244 -0.743769
O -2.762551 -0.958119 -0.359968
H 0.051120 -1.608315 -0.436631
H -1.505370 -0.273652 -1.862139
FREQS= 74 79 87 98 179 230 287 360 384 392 418 455 477 490 530 627 661 729 823 926 1137
1287 1824 1904 1985 2144 2181 2340 2872
TS141_7
12
E= -1837.72788717 ZPE= 37.14 Gcorr 0.001295000
H -1.061318 -1.053519 1.771816
Co -0.012051 -0.117686 -0.356369
C -0.054181 -0.720002 1.431911
C -0.296106 1.603171 0.074311
O 0.912583 -0.733432 2.137828
O -0.487528 2.679565 0.416760
C 1.794241 -0.181220 -0.708521
O 2.895543 -0.341121 -0.966271
C -1.714377 -0.546043 -0.743821
O -2.762634 -0.957855 -0.360090
H 0.050895 -1.608332 -0.436801
H -1.505365 -0.273324 -1.862153
FREQS= 74 79 87 98 179 230 287 360 384 392 418 455 477 490 530 627 661 729 823 926 1137
1287 1824 1904 1985 2144 2181 2340 2872
TS142_7
12
E= -1837.72788717 ZPE= 37.14 Gcorr 0.001295000
H -1.061006 -1.053443 1.771991
Co -0.012059 -0.117658 -0.356387
C -0.053913 -0.719980 1.431906
C -0.296430 1.603118 0.074319
O 0.912968 -0.733459 2.137663
O -0.488044 2.679445 0.416874
C 1.794250 -0.180975 -0.708567
O 2.895561 -0.340786 -0.966332
C -1.714345 -0.546244 -0.743769
O -2.762551 -0.958119 -0.359968
H 0.051120 -1.608315 -0.436631
H -1.505370 -0.273652 -1.862139
FREQS= 74 79 87 98 179 230 287 360 384 392 418 455 477 490 530 627 661 729 823 926 1137
1287 1824 1904 1985 2144 2181 2340 2872
TS143_7
12
E= -1837.72777187 ZPE= 37.81 Gcorr 0.001426000
H -3.030052 2.314639 0.169164
Co 0.102810 -0.010436 -0.159015
C -2.530183 1.344484 0.072363
C -1.056920 -1.336124 0.072972
O -1.299638 1.299318 0.082638
O -1.878922 -2.141439 0.153823
C 1.261064 1.347285 0.089158
O 1.981986 2.240053 0.156756
C 1.414719 -1.130680 0.065505
O 2.290831 -1.878539 0.112846
H 0.114155 0.020672 -1.693795
H -3.146095 0.441529 -0.030446

FREQS=   83   88   95 103 118 170 243 269 354 371 418 441 474 498 543 573 588 604 630 1087 1260
1514 1681 1756 2077 2095 2147 3028 3138
TS144_7
12
E= -1837.72626220 ZPE=   37.92 Gcorr   -0.000021000
H 0.280558 0.049914 -1.628138
Co -0.072082 -0.004438 -0.113619
C 2.754687 0.434558 -0.388586
C -0.428093 1.723340 0.170973
O 1.840285 0.364175 0.419597
O -0.600202 2.856050 0.294682
C -1.776317 -0.328652 -0.086368
O -2.907471 -0.543478 -0.162958
C 0.233439 -1.738097 0.182771
O 0.494918 -2.853222 0.313264
H 2.568645 0.304715 -1.463870
H 3.774485 0.630096 -0.029696
FREQS=   22   84   93 104 132 155 259 261 345 372 445 445 477 499 517 568 609 621 651 1209 1259
1505 1685 1765 2078 2084 2146 3012 3127
TS145_7
12
E= -1837.73133736 ZPE=   40.43 Gcorr   0.005279000
H -2.800845 -0.084189 0.982790
Co 0.157632 0.006401 0.058908
C -2.485630 0.934199 0.737617
C 1.281329 1.395903 -0.180815
O -1.358774 1.191653 0.325782
O 2.021865 2.164281 -0.610754
C 1.427725 -1.113858 0.286421
O 2.244207 -1.888338 0.550391
C -0.991036 -1.458928 -0.390435
O -2.017854 -1.198607 -1.007472
H -0.759150 -2.538182 -0.218655
H -3.205948 1.753748 0.865039
FREQS=   40   77   94 107 157 193 246 279 344 364 376 424 462 474 561 601 644 849 1156 1270 1401
1526 1699 1731 2074 2128 2810 3037 3157
TS146_7
12
E= -1837.72772968 ZPE=   38.39 Gcorr   0.001871000
H -1.556720 0.441958 -1.034174
Co -0.120956 0.074171 -0.035241
C -2.198270 0.010090 -0.166863
C 0.814482 1.472703 -0.662870
O -2.470344 0.716724 0.793212
O 1.489427 2.251639 -1.170092
C 0.789822 -0.256929 1.420451
O 1.735939 -0.603653 2.038240
C 0.190515 -1.453423 -0.840847
O 0.521308 -2.401868 -1.404619
H -2.736342 -0.918073 -0.416827
H -0.231062 0.136114 1.849356
FREQS=   63   70   85 100 119 159 265 300 348 361 416 457 470 500 521 555 633 746 1138 1168 1259
1449 1729 1948 2099 2149 2348 2379 3019
TS147_7
12
E= -1837.71917436 ZPE=   35.02 Gcorr   -0.006000000
H 1.108065 -1.530607 -1.824757
Co -0.164522 -0.065451 0.133282
C 1.094669 -1.378613 -0.738236
C 1.744217 -0.274008 -0.082282
O 0.196582 -1.929312 0.027722
O 2.779878 0.234838 0.217588
C -1.962246 -0.264286 0.047084
O -3.079140 -0.409292 -0.160946
C -0.100728 1.656643 -0.096899
O -0.052668 2.788185 -0.303051
H 0.122705 -0.007922 2.750837
H -0.201386 -0.608051 2.446779
FREQS=   46   79   86   97 116 127 178 225 262 271 337 385 408 423 443 509 528 586 606 662 850
918 1284 1413 1934 2111 2163 3069 4384
TS148_7
12
E= -1837.73085394 ZPE=   39.43 Gcorr   0.005727000
H -0.715722 2.427995 0.133340
Co 0.108410 0.001485 -0.150450
C -1.055041 1.387870 0.125655
C 1.405825 1.214681 0.002308

O -2.378536 1.455672 0.237679
O 2.231458 2.012767 0.035418
C -1.234392 -1.124480 -0.041788
O -2.170248 -1.799892 -0.029162
C 1.297589 -1.317895 0.120970
O 2.058069 -2.166590 0.267699
H 0.163904 -0.019803 -1.655498
H -2.785082 0.574987 0.248357
FREQS= 91 97 99 122 142 207 307 367 369 432 454 473 537 562 572 607 630 677 692 971 1267
1314 1519 1858 2082 2113 2158 3102 3757
TS149_7
12
E= -1837.71969618 ZPE= 37.24 Gcorr -0.005183000
H -0.045104 -0.744740 1.528893
Co 0.103633 -0.025724 0.226332
C -0.440111 1.189461 1.779875
C 0.732787 0.982615 -1.115442
O -1.121328 1.375358 0.705016
O 1.268948 1.699841 -1.833861
C 1.405564 -1.155208 0.251780
O 2.279256 -1.902851 0.346632
C -2.909364 -1.262693 -0.953366
O -1.800050 -1.126810 -0.727615
H -0.914798 0.710481 2.643517
H 0.413938 1.839466 2.018157
FREQS= 31 41 57 76 95 98 119 135 202 378 386 424 463 486 544 565 593 680 884 1125 1209
1385 1593 1951 2099 2151 2180 3009 3094
TS150_7
12
E= -1837.72246853 ZPE= 37.35 Gcorr -0.002343000
H -0.905778 -0.057290 2.799697
Co 0.016843 0.094211 -0.236558
C -0.503113 -1.003730 2.413848
C -1.633183 0.577703 -0.488512
O -0.100137 -1.111590 1.261180
O -2.658571 1.088456 -0.645119
C 1.320523 1.202101 0.229274
O 2.002962 2.106063 0.450775
C 0.211289 -1.573707 -1.596335
O 1.306191 -1.671999 -1.238968
H 0.138711 0.882512 -1.470404
H -0.464355 -1.870556 3.085177
FREQS= 56 66 79 95 100 115 147 214 246 324 347 368 449 476 478 521 547 700 764 1137 1260
1521 1725 2017 2028 2070 2124 3020 3129
TS151_7
12
E= -1837.71311075 ZPE= 34.16 Gcorr -0.011621000
H 2.196764 0.000018 -1.494786
Co 0.312402 -0.000001 -0.535308
C 2.074148 0.000013 -0.353480
C 0.055378 -1.499901 0.346456
O 2.976152 0.000018 0.419212
O -0.169719 -2.388757 1.045105
C 0.055357 1.499893 0.346456
O -0.169750 2.388747 1.045105
C -2.618927 -0.000017 -0.470040
O -3.589802 0.000004 0.119291
H -0.201201 0.404114 -2.148983
H -0.201194 -0.404124 -2.148983
FREQS= 24 34 65 71 95 101 104 178 186 256 349 385 433 450 510 514 526 574 689 759 841
1126 1354 1900 2085 2136 2218 2543 3389
TS152_7
12
E= -1837.72353839 ZPE= 36.04 Gcorr -0.001117000
H 2.568928 -0.000169 0.692495
Co -0.011364 0.000001 -0.149434
C 1.863485 -0.000067 -0.154568
C -0.027824 -1.562657 0.698188
O 2.389137 0.000008 -1.302819
O -0.019343 -2.529857 1.322495
C -0.027790 1.562656 0.698220
O -0.019271 2.529851 1.322530
C -1.770205 -0.000030 -0.576388
O -2.846536 0.000054 -0.967176
H 1.257791 0.000099 -1.824806
H 0.222200 0.000170 -1.825934

FREQS=   58   85   97 106 117 158 320 348 401 427 441 494 496 503 514 568 576 653 939 1097 1217
1349 1487 1612 1738 2094 2119 2169 3023
TS153_7
12
E= -1837.70926066 ZPE=   34.24 Gcorr   -0.015133000
H -0.391643 -2.234884 -1.489886
Co 0.427263 -0.079890 0.105144
C -0.344188 -1.266421 -0.972195
C 1.941326 -0.870485 0.372279
O -1.093147 -0.319404 -1.243386
O 2.950597 -1.421364 0.477069
C 0.999614 1.623695 -0.003996
O 1.368246 2.676275 -0.285630
C -4.017375 -0.002708 -0.019748
O -3.424101 0.035685 0.951749
H -0.845084 -0.235098 1.412879
H -0.188396 -0.047009 1.781660
FREQS=   14   25   42   57   73   82   96 117 144 278 327 343 409 432 464 496 531 580 684 695 839
1032 1215 1614 2088 2137 2199 3056 3885
TS154_7
12
E= -1837.72515418 ZPE=   37.92 Gcorr   0.000790000
H 0.287705 -1.361864 1.981010
Co 0.153529 0.004376 -0.192435
C -0.402865 -0.854175 1.285397
C -1.402826 -0.025739 -1.054291
O -1.673195 -0.945223 1.762890
O -2.378456 -0.025027 -1.662497
C 0.491715 1.681901 0.313369
O 0.701319 2.754447 0.665179
C 1.695965 -0.878023 -0.335416
O 2.703275 -1.407852 -0.499504
H -2.152613 -1.712426 1.420048
H 0.604133 0.401586 -1.588223
FREQS=   53   74   81   97 109 155 288 330 333 431 448 467 492 500 514 552 654 659 734 819 1013
1238 1420 1881 2106 2116 2165 2984 3816
TS155_7
12
E= -1837.71749612 ZPE=   36.87 Gcorr   -0.006467000
H 0.870632 -0.855072 2.123452
Co -0.035919 -0.432521 -0.320746
C -0.077833 -1.074417 1.611452
C 1.732408 -0.338914 -0.539510
O -0.146082 -2.016714 0.749289
O 2.846815 -0.188502 -0.760395
C -1.774746 -0.090383 -0.533927
O -2.857182 0.216462 -0.751493
C 0.147932 2.115889 0.924795
O 0.267032 3.117496 0.397585
H 0.031853 0.553954 -1.447166
H -0.983902 -0.723788 2.127115
FREQS=   17   49   66   68   82   91 128 142 266 376 406 429 438 452 453 570 610 624 751 1075 1218
1399 1604 1948 2109 2174 2194 2988 3065
TS156_7
12
E= -1837.71501702 ZPE=   36.94 Gcorr   -0.008526000
H -1.520866 2.919734 0.533941
Co 0.671875 -0.128911 -0.336468
C -0.838834 2.125921 0.857270
C 2.338907 0.335643 -0.202797
O -0.376973 1.344347 0.025251
O 3.469718 0.429790 0.024080
C -0.089629 -1.527665 0.344807
O -0.364917 -2.510958 0.890379
C -3.273558 -0.442086 -0.870806
O -3.496188 0.329782 -0.063356
H 1.289683 -1.180914 -1.151792
H -0.583879 2.047212 1.920825
FREQS=   21   37   48   65   70   73   78 179 185 280 352 410 431 465 487 553 560 628 774 1079 1252
1521 1693 2042 2053 2120 2197 3037 3147
TS157_7
12
E= -1837.72422873 ZPE=   36.97 Gcorr   0.001072000
H 2.424878 -0.784661 -1.062356
Co -0.154181 -0.080737 -0.458343
C 1.588893 -0.990225 -0.386314
C -1.707550 -0.552765 0.367247

O 0.641760 -1.849376 -0.652201
O -2.560708 -0.842843 1.074881
C 1.288054 -0.183472 0.775732
O 1.693366 0.312720 1.777680
C -0.147369 1.680208 -0.349243
O -0.169234 2.826490 -0.271637
H -0.848279 0.137232 -2.189674
H -0.387338 -0.471076 -2.247019
FREQS= 54 79 98 102 223 268 313 355 371 392 402 446 470 495 519 564 613 636 645 817 955
1023 1260 1385 1935 2115 2163 3094 4067
TS158_7
12
E= -1837.73009006 ZPE= 41.48 Gcorr 0.006997000
H -1.100813 0.620610 -0.000317
Co 0.483185 -0.027602 0.000099
C -1.560859 -0.455575 0.000166
C 1.252818 1.531183 0.000022
O -0.659849 -1.436787 0.000701
O 1.727851 2.580929 0.000235
C 1.963551 -0.924489 -0.000205
O 2.929680 -1.543905 -0.000628
C -2.898288 -0.455940 0.000042
O -3.727334 0.636400 -0.000544
H -3.208708 1.452526 -0.001100
H -3.442592 -1.392071 0.000484
FREQS= 30 74 76 100 172 192 315 351 382 447 458 497 545 591 595 629 694 816 1099 1184 1275
1374 1612 1842 2105 2167 2345 3243 3806
TS159_7
12
E= -1837.71644692 ZPE= 37.06 Gcorr -0.006602000
H -0.237658 1.002576 3.489836
Co 0.104546 -0.050977 -0.213763
C 0.289205 0.569875 2.631377
C 1.378722 0.972974 -0.797716
O -0.264494 0.536053 1.533807
O 2.348492 1.428785 -1.237374
C 0.175910 -1.781434 -0.107923
O 0.457258 -2.904570 -0.149599
C -3.011802 0.286195 -0.919242
O -2.159650 0.977676 -0.607770
H 0.066990 -0.391742 -1.646918
H 1.302874 0.176335 2.777194
FREQS= 30 41 60 71 75 90 97 147 206 296 348 391 466 467 494 558 559 664 770 1101 1255
1516 1700 2022 2047 2112 2176 3030 3139
TS160_7
12
E= -1837.73181665 ZPE= 41.38 Gcorr 0.008780000
H 1.558285 -0.217757 -1.712478
Co -0.215394 -0.152879 0.067224
C 1.443541 -1.393015 0.359703
C -1.936909 -0.361959 -0.166441
O 0.177449 -1.881371 0.372246
O -3.051337 -0.531297 -0.379241
C -0.323833 1.595596 0.149653
O -0.416694 2.724821 0.350313
C 1.763178 -0.017253 -0.637663
O 2.608111 0.760044 -0.262795
H 2.114450 -1.968175 -0.305491
H 1.926806 -1.204107 1.327228
FREQS= 39 91 99 107 189 214 344 385 428 446 472 488 541 561 635 720 792 1008 1158 1215 1253
1340 1498 1758 2108 2161 2892 2942 3063
TS161_7
12
E= -1837.72123837 ZPE= 37.40 Gcorr -0.001770000
H 2.767296 -0.649452 -0.620530
Co -0.217974 0.065122 0.146623
C 2.054076 -0.921708 -1.410947
C 0.053626 1.810757 -0.096126
O 0.852691 -0.715793 -1.293636
O -0.043621 2.951002 -0.237513
C -1.732033 -0.742355 -0.172002
O -2.812535 -1.092274 -0.377376
C 0.552960 -1.102738 1.692316
O 1.526774 -0.467388 1.621858
H -1.076475 0.615168 1.201834
H 2.436241 -1.392123 -2.326248

FREQS=   52   68   86   94 114 132 148 226 293 330 354 359 433 467 483 507 538 682 772 1158 1268
1523 1738 1952 2033 2078 2131 3019 3125
TS162_7
12
E= -1837.72232600 ZPE=   37.88 Gcorr   -0.000631000
H -0.540164 -0.103791 -2.775710
Co 0.050388 0.109626 -0.204770
C -0.897233 -0.586456 -1.857462
C -0.387536 -1.249100 0.946317
O -1.680736 0.048955 -1.061122
O -0.592999 -2.168664 1.594349
C 1.774904 -0.021740 -0.135105
O 2.922835 -0.096463 -0.196211
C -0.349222 2.237460 0.571894
O -0.817612 1.658163 1.447390
H -0.906117 -1.686492 -1.878122
H 0.588440 1.013477 -1.246497
FREQS=   60   69   79   87 104 120 139 168 369 385 418 421 442 522 549 561 660 735 882 1091 1214
1385 1596 1999 2079 2116 2169 2997 3082
TS163_7
12
E= -1837.72128391 ZPE=   37.80 Gcorr   -0.001479000
H 0.188968 0.586822 2.714145
Co -0.004254 -0.074622 0.184489
C 0.164937 1.151720 1.773614
C -1.329152 -1.185346 0.170403
O 1.202842 1.174959 1.010957
O -2.201827 -1.931316 0.274338
C -0.657005 1.140389 -0.998784
O -1.166418 1.893172 -1.694816
C 2.451952 -1.447870 -0.590675
O 1.711613 -0.812165 -1.188655
H 0.323869 -1.108285 1.216584
H -0.552050 1.985689 1.746112
FREQS=   50   57   76   85 110 119 126 158 340 392 419 426 453 525 556 571 665 729 885 1087 1214
1376 1593 1932 2109 2145 2163 2996 3080
TS164_7
12
E= -1837.72549514 ZPE=   38.08 Gcorr   0.002843000
H -1.339855 -1.220450 0.543518
Co -0.049338 -0.029383 -0.276872
C -1.858996 0.052955 -1.004600
C 1.565392 -0.832086 -0.369781
O -1.581093 -1.244139 -0.801373
O 2.622594 -1.257593 -0.504728
C 0.335758 1.659625 -0.104656
O 0.589472 2.777155 0.006629
C -0.596084 -0.563962 1.406858
O -0.322864 -0.371616 2.527806
H -1.821128 0.368392 -2.053026
H -2.648172 0.515761 -0.408552
FREQS=   70   79   85 112 154 243 259 354 363 387 426 474 497 523 541 570 606 747 1055 1122 1213
1242 1514 1548 1991 2103 2158 3054 3148
TS165_7
12
E= -1837.72805464 ZPE=   39.55 Gcorr   0.005518000
H -2.325935 -1.642409 1.078310
Co 0.199786 -0.062126 -0.000539
C -1.816120 -1.452350 0.122742
C 0.560124 1.665718 0.087362
O -0.426867 -1.741785 0.164429
O 0.729144 2.798897 0.141874
C 1.964534 -0.632772 0.024111
O 3.016149 -1.045395 -0.140220
C -1.743480 0.042933 -0.123870
O -2.569850 0.906718 -0.140653
H -2.353451 -1.998478 -0.671018
H -0.493805 0.229635 -1.258271
FREQS=   46   82   88 104 155 256 330 391 432 439 452 504 527 583 588 697 787 957 993 1039 1167
1314 1490 1864 2046 2135 2179 2979 3040
TS166_7
12
E= -1837.72792586 ZPE=   39.15 Gcorr   0.005543000
H -0.755335 -2.427945 0.092554
Co 0.109071 -0.014630 -0.148898
C -1.061623 -1.374199 0.098142
C 1.298233 1.309597 0.105735

O -2.393042 -1.265024 0.207844
O 2.058825 2.161331 0.236073
C 1.392553 -1.233426 0.017653
O 2.206894 -2.044296 0.065173
C -1.238877 1.134023 -0.016494
O -2.106941 1.884294 0.004675
H -2.818866 -2.135040 0.239169
H 0.161681 0.051604 -1.651814
FREQS= 98 99 104 132 153 219 313 342 374 431 448 469 534 550 570 588 610 630 699 914 1243
1292 1508 1860 2105 2115 2167 3048 3765
TS167_7
12
E= -1837.72792587 ZPE= 39.15 Gcorr 0.005544000
H -2.819030 -2.134904 0.238743
Co 0.109088 -0.014642 -0.148864
C -1.061737 -1.374131 0.097991
C 1.298303 1.309480 0.105569
O -2.393179 -1.264908 0.207370
O 2.058912 2.161238 0.235657
C -1.238759 1.134104 -0.016245
O -2.106773 1.884425 0.005112
C 1.392469 -1.233508 0.017830
O 2.206751 -2.044433 0.065463
H -0.755500 -2.427893 0.092637
H 0.161821 0.051891 -1.651748
FREQS= 98 99 104 132 153 219 313 342 374 431 448 469 534 550 570 588 610 630 699 914 1243
1292 1508 1860 2105 2115 2167 3048 3765
TS168_7
12
E= -1837.72457520 ZPE= 39.57 Gcorr 0.002311000
H 0.770665 -2.004870 1.507629
Co 0.100125 0.015065 -0.115596
C -0.107832 -2.287663 0.909583
C 1.895792 -0.051634 -0.101333
O -0.252205 -1.907243 -0.259197
O 3.013522 0.028217 -0.366700
C -0.049361 1.676329 0.288745
O -0.144482 2.780289 0.613703
C -1.720363 0.134333 -0.683341
O -2.738042 0.046436 -0.039453
H -1.777355 0.164511 -1.812626
H -0.836451 -2.976176 1.357322
FREQS= 33 67 89 94 108 145 211 238 345 365 402 434 454 465 543 566 585 873 1114 1249 1334
1498 1680 1789 2078 2126 2673 3009 3114
TS169_7
12
E= -1837.71224952 ZPE= 35.21 Gcorr -0.009672000
H -2.441769 0.000024 1.332155
Co -0.424864 -0.000001 -0.353209
C 2.561044 -0.000014 -0.682345
C -0.020078 -1.514251 0.436097
O 3.630492 0.000000 -0.300400
O 0.372289 -2.398006 1.068052
C -0.020033 1.514230 0.436107
O 0.372358 2.397968 1.068073
C -2.125169 0.000025 0.270467
O -2.904353 0.000044 -0.651742
H -0.113891 -0.402386 -2.014665
H -0.113880 0.402393 -2.014664
FREQS= 24 34 66 77 98 112 116 160 170 259 294 367 441 468 511 539 544 677 716 859 888
1279 1364 1773 2066 2120 2221 2945 3442
TS170_7
12
E= -1837.71773831 ZPE= 38.39 Gcorr -0.003234000
H 1.105794 1.657296 1.825525
Co 0.053680 0.060499 0.084184
C 0.185736 1.068874 1.729069
C 1.130715 0.658000 -1.150679
O -0.747221 1.476486 0.883690
O 2.221606 1.041307 -1.426201
C 0.861569 -1.467941 0.282741
O 1.403481 -2.450066 0.548446
C -3.409049 -0.383076 -0.657731
O -2.284916 -0.542840 -0.768104
H 0.216511 0.707855 -1.862510
H -0.129107 0.547118 2.640974

FREQS=   27   41   54   73   91 100 122 129 264 319 419 481 482 502 543 632 744 755 1090 1157 1200
1287 1566 1913 2117 2179 2444 3019 3103
TS171_7
12
E= -1837.72672402 ZPE=   39.98 Gcorr   0.006130000
H 0.797521 1.254543 0.759893
Co -0.098840 0.043951 0.076384
C 1.032820 1.443865 -0.474855
C 1.162919 -1.125786 0.547948
O 2.376802 1.303866 -0.716223
O 1.985513 -1.860613 0.867989
C -1.115534 -1.266630 -0.483242
O -1.763253 -2.108183 -0.934574
C -1.542886 1.042357 0.395356
O -2.456936 1.709190 0.602698
H 2.819850 2.164941 -0.690494
H 0.690402 2.476900 -0.602143
FREQS=   75   95   99 107 108 233 306 374 423 435 453 470 487 547 567 613 644 667 1103 1135 1256
1397 1452 1751 2087 2102 2160 3068 3751
TS172_7
12
E= -1837.72677711 ZPE=   40.82 Gcorr   0.006401000
H -2.142793 0.962187 -1.133963
Co -0.009211 0.104428 0.027849
C -0.387748 0.351702 -1.810988
C -1.058121 -1.271945 0.479864
O -1.242948 1.311405 -1.200711
O -1.468168 -2.329414 0.700490
C 1.589761 -0.507136 -0.248940
O 2.622712 -0.995473 -0.421927
C -0.304766 1.186084 1.915986
O 0.579115 1.664363 1.315830
H -0.919126 -0.419439 -2.369812
H 0.350163 0.878427 -2.413138
FREQS=   79   80   95 111 131 149 225 263 318 354 390 474 487 522 560 583 614 801 1012 1070 1200
1327 1515 1919 2063 2112 3095 3189 3816
TS173_7
12
E= -1837.72010173 ZPE=   37.88 Gcorr   0.000086000
H 2.192981 -0.885938 -1.353667
Co -0.086814 0.066791 -0.167972
C 1.360064 -0.160281 -1.330612
C 0.588501 1.020867 1.187561
O 1.355847 0.906748 -1.930848
O 1.056775 1.658244 2.019940
C -1.905977 0.263694 -0.253331
O -3.045185 0.349221 -0.341744
C 0.005728 -1.495280 0.849313
O 0.676989 -2.459011 0.603562
H 0.177975 -0.856114 -1.375303
H -0.672280 -1.476914 1.739353
FREQS=   46   65   76   91 110 175 194 261 330 394 415 437 471 493 513 598 721 876 1028 1140 1279
1301 1680 1824 1906 2117 2159 2811 2985
TS174_7
12
E= -1837.71875555 ZPE=   36.24 Gcorr   -0.000922000
H -0.000066 -1.856449 -1.148782
Co 0.000069 0.004113 -0.429981
C -1.732887 0.395508 -0.556934
C 0.000023 -1.723976 0.029579
O -2.829707 0.705020 -0.668454
O 0.000137 -2.601853 0.822766
C 0.000020 0.502905 1.447394
O -0.000553 1.655926 1.767845
C 1.732955 0.395646 -0.556795
O 2.829724 0.705396 -0.668210
H 0.000198 0.935905 -1.644462
H 0.000524 -0.326922 2.191698
FREQS=   52   80   89   97 151 198 291 337 382 426 434 448 487 490 523 563 616 642 746 913 1065
1305 1824 1844 1942 2137 2180 2229 2858
TS175_7
12
E= -1837.72783480 ZPE=   41.01 Gcorr   0.008414000
H 1.871257 -2.090865 -0.352382
Co 0.104026 -0.106805 -0.062758
C 0.971329 -1.930443 0.253165
C 1.765123 0.433797 0.073524

O -0.184666 -1.983574 -0.312595
O 2.838051 0.852958 0.076851
C -0.461461 1.530856 -0.059188
O -0.784115 2.636354 -0.020450
C -1.854287 -0.339918 -0.336397
O -2.635861 -0.307840 0.569276
H 1.064994 -2.140182 1.327324
H -2.136467 -0.634155 -1.371764
FREQS= 63 89 110 120 165 240 265 334 358 438 451 482 544 559 574 603 761 894 1099 1217 1308
1380 1584 1825 2095 2138 2879 3011 3103
TS176_7
12
E= -1837.71929031 ZPE= 37.79 Gcorr -0.000103000
H 0.810637 0.370758 -2.350504
Co -0.106494 -0.260007 -0.102675
C -1.769700 -0.637928 -0.852073
C -0.427868 1.129972 1.007803
O -2.237057 -1.260787 0.098422
O -0.679454 1.981454 1.730423
C 1.369368 -1.153961 0.490152
O 2.302354 -1.729293 0.822385
C 0.768240 0.829133 -1.330206
O 1.284516 1.893464 -1.120981
H -2.376098 -0.294272 -1.708664
H -0.562316 -1.138314 -1.304654
FREQS= 47 66 80 92 113 137 196 265 348 386 391 428 470 499 510 629 703 866 1023 1152 1296
1317 1657 1811 1862 2132 2171 2806 2981
TS177_7
12
E= -1837.71955933 ZPE= 37.10 Gcorr 0.000816000
H 0.189056 0.602141 -2.067736
Co 0.181233 0.044654 -0.447563
C 1.059360 -1.468765 -0.212825
C -0.860630 -0.111538 0.966020
O 1.763813 -2.348900 0.020458
O -1.073161 -0.249855 2.126048
C 1.098282 1.481869 0.042854
O 1.778221 2.320579 0.437099
C -1.964760 -0.321536 -0.206039
O -2.322471 0.555729 -0.971116
H -2.457759 -1.312398 -0.148293
H 0.210694 -0.195996 -2.139733
FREQS= 60 73 93 100 186 209 290 325 336 437 466 480 504 511 528 555 564 602 731 884 925
1343 1458 1700 1908 2101 2149 2944 3491
TS178_7
12
E= -1837.71989038 ZPE= 38.13 Gcorr 0.001207000
H 1.503611 -0.887643 -1.654273
Co 0.025369 -0.184923 -0.129042
C 1.905729 -0.753995 -0.617522
C 0.457548 1.194628 0.873959
O 3.068820 -0.523795 -0.391979
O 0.634749 2.049252 1.621720
C -1.163564 0.737667 -1.110949
O -2.113773 1.453850 -1.110782
C -1.188713 -1.348823 0.476032
O -1.947342 -2.003957 1.037751
H 1.258684 -1.273790 0.232303
H -0.652881 0.374691 -2.076698
FREQS= 61 81 83 95 107 185 226 256 342 381 414 435 477 495 516 539 674 748 1060 1171 1335
1574 1788 1896 2107 2151 2158 2502 2815
TS179_7
12
E= -1837.71989038 ZPE= 38.13 Gcorr 0.001207000
H -1.258684 -1.273790 0.232303
Co -0.025369 -0.184923 -0.129042
C 1.188713 -1.348823 0.476032
C -1.905729 -0.753995 -0.617522
O 1.947342 -2.003957 1.037751
O -3.068820 -0.523795 -0.391979
C 1.163564 0.737667 -1.110949
O 2.113773 1.453850 -1.110782
C -0.457548 1.194628 0.873959
O -0.634749 2.049252 1.621720
H -1.503611 -0.887643 -1.654273
H 0.652881 0.374691 -2.076698

FREQS= 61 81 83 95 107 185 226 256 342 381 414 435 477 495 516 539 674 748 1060 1171 1335
1574 1788 1896 2107 2151 2158 2502 2815
TS180_7
12
E= -1837.71989038 ZPE= 38.13 Gcorr 0.001207000
H 1.503611 -0.887643 -1.654273
Co 0.025369 -0.184923 -0.129042
C 1.905729 -0.753995 -0.617522
C 0.457548 1.194628 0.873959
O 3.068820 -0.523795 -0.391979
O 0.634749 2.049252 1.621720
C -1.163564 0.737667 -1.110949
O -2.113773 1.453850 -1.110782
C -1.188713 -1.348823 0.476032
O -1.947342 -2.003957 1.037751
H 1.258684 -1.273790 0.232303
H -0.652881 0.374691 -2.076698
FREQS= 61 81 83 95 107 185 226 256 342 381 414 435 477 495 516 539 674 748 1060 1171 1335
1574 1788 1896 2107 2151 2158 2502 2815
TS181_7
12
E= -1837.71989038 ZPE= 38.13 Gcorr 0.001207000
H -1.258684 -1.273790 0.232303
Co -0.025369 -0.184923 -0.129042
C 1.188713 -1.348823 0.476032
C -1.905729 -0.753995 -0.617522
O 1.947342 -2.003957 1.037751
O -3.068820 -0.523795 -0.391979
C 1.163564 0.737667 -1.110949
O 2.113773 1.453850 -1.110782
C -0.457548 1.194628 0.873959
O -0.634749 2.049252 1.621720
H -1.503611 -0.887643 -1.654273
H 0.652881 0.374691 -2.076698
FREQS= 61 81 83 95 107 185 226 256 342 381 414 435 477 495 516 539 674 748 1060 1171 1335
1574 1788 1896 2107 2151 2158 2502 2815
TS182_7
12
E= -1837.72841716 ZPE= 41.09 Gcorr 0.009883000
H -0.213891 -2.577989 0.197093
Co 0.191417 -0.021009 -0.401458
C -0.656816 -1.571247 0.166401
C -1.508151 0.781879 -0.648012
O -1.769165 -1.586545 0.812539
O -2.524942 0.636961 0.042393
C 0.756820 1.559747 0.187334
O 1.048962 2.479745 0.817125
C 1.795378 -0.778749 -0.168760
O 2.815079 -1.231610 0.109105
H -2.219118 -0.650679 0.703132
H -1.618100 1.457727 -1.531931
FREQS= 79 90 98 170 261 297 312 380 405 433 456 492 511 555 571 655 870 902 1237 1366 1376
1433 1616 1672 2082 2132 2428 2826 3033
TS183_7
12
E= -1837.72078338 ZPE= 39.63 Gcorr 0.002544000
H 3.314841 -0.598888 0.054466
Co -0.135417 0.018076 0.145022
C 1.553114 -0.479517 0.833893
C -1.837913 0.593412 0.310008
O 2.730303 0.102153 0.369739
O -2.925035 0.870021 0.561225
C -0.453012 -1.587033 -0.371687
O -0.646366 -2.671947 -0.717324
C 0.495810 1.193520 -1.350247
O 0.710306 2.085959 -0.636703
H 1.717232 -1.484094 1.233899
H 1.122540 0.183155 1.648751
FREQS= 52 83 86 91 129 147 154 201 282 355 370 395 448 460 538 559 577 751 1100 1124 1242
1351 1456 1968 2083 2138 2663 3088 3830
TS184_7
12
E= -1837.71964073 ZPE= 40.38 Gcorr 0.001772000
H -2.111207 1.078901 -1.152246
Co -0.033672 -0.031177 -0.035782
C -1.023630 1.025911 -1.232787
C 0.949721 1.283983 0.560610

O -0.584216 0.009908 -2.146813
O 1.414082 2.212130 1.078664
C -1.394334 -0.625694 0.914580
O -2.270431 -0.736878 1.666515
C 2.442451 -1.612300 -0.145535
O 1.323485 -1.736035 0.070005
H -0.596834 1.977410 -1.547455
H -1.291413 -0.638934 -2.262347
FREQS= 36 60 69 82 98 110 133 194 243 297 403 476 499 520 539 567 634 839 987 1067 1235
1326 1515 2033 2092 2121 3081 3169 3823
TS185_7
12
E= -1837.72003561 ZPE= 38.17 Gcorr 0.002396000
H -0.320237 -0.854866 2.165450
Co 0.034802 -0.040288 -0.206646
C 1.347665 -1.219323 -0.750435
C 1.061772 0.977707 0.919491
O 1.904916 -0.520736 -1.578266
O 1.731140 1.613507 1.594936
C -1.378053 1.012034 -0.634312
O -2.318471 1.567637 -0.975075
C -0.780452 -1.047119 1.162825
O -1.707470 -1.800984 1.069691
H 1.638540 -2.270899 -0.561969
H -0.644452 -1.001637 -1.099734
FREQS= 68 75 84 114 129 194 227 332 376 398 429 458 488 506 604 618 721 845 881 902 1283
1336 1719 1831 2030 2144 2175 2787 2949
TS186_7
12
E= -1837.70821191 ZPE= 35.37 Gcorr -0.009383000
H -0.416615 2.796975 -0.960427
Co -0.539879 0.121387 -0.486018
C 0.093528 1.843211 -0.707864
C -0.644860 -1.696129 -0.411401
O 1.304772 1.776663 -0.614410
O -0.667168 -2.840180 -0.344072
C -1.333740 0.515422 1.022798
O -1.965117 0.766967 1.954902
C 2.220233 -0.736673 0.502295
O 3.236464 -0.457268 0.926766
H -1.520687 0.296555 -1.670052
H -0.768546 0.104587 -2.067497
FREQS= 11 58 72 75 81 97 140 161 227 229 377 409 465 479 529 545 590 649 693 898 1072
1320 1742 1763 2096 2153 2217 2687 2905
TS187_7
12
E= -1837.71980096 ZPE= 38.62 Gcorr 0.002222000
H 0.858513 0.726226 -1.519998
Co 0.177228 0.154534 -0.118614
C 1.893442 0.459442 -0.918232
C 0.451542 -1.260730 0.901988
O 2.221359 1.210023 0.006104
O 0.590849 -2.160802 1.604489
C -1.131510 1.249458 0.534573
O -1.914763 1.908099 1.048604
C -0.925499 -0.874106 -1.207068
O -2.102029 -1.079828 -1.098565
H 2.619324 -0.080897 -1.547810
H -0.354182 -1.282054 -2.082247
FREQS= 55 67 85 89 143 168 244 265 350 368 397 432 486 498 516 600 755 882 1116 1287 1308
1429 1684 1824 1912 2111 2160 2777 3008
TS188_7
12
E= -1837.71449325 ZPE= 36.50 Gcorr -0.003043000
H 0.905996 -0.057126 2.157987
Co 0.491579 -0.146605 0.406893
C -1.119507 -0.773733 0.023007
C 0.232064 1.545842 0.119976
O -0.554394 -1.878386 -0.015242
O 0.057879 2.659959 -0.115113
C 2.153516 -0.297034 -0.281617
O 3.104023 -0.455821 -0.908273
C -2.565530 -0.570607 -0.384366
O -3.076161 0.522461 -0.398425
H -3.107676 -1.496086 -0.664640
H 0.475023 -0.700948 2.154977

FREQS= 33 55 76 89 111 169 252 286 310 371 400 436 470 480 515 540 571 630 679 975 1006
1159 1373 1628 1813 2099 2143 2958 3908
TS189_7
12
E= -1837.71994566 ZPE= 40.49 Gcorr 0.002663000
H -1.602380 -2.067043 -0.160622
Co -0.033942 -0.024965 -0.094446
C -1.641970 -0.984681 -0.276109
C -0.718566 1.304948 0.829396
O -1.427270 -0.666907 -1.657914
O -1.148226 2.001942 1.651548
C 0.839663 -1.334774 0.679283
O 1.300306 -2.136956 1.377344
C 2.370587 1.404840 -0.653766
O 1.519746 0.910276 -1.243654
H -2.576313 -0.573173 0.110536
H -1.959618 0.105438 -1.891289
FREQS= 52 60 70 80 101 109 140 216 254 298 410 477 503 525 530 563 635 847 992 1072 1238
1329 1516 2034 2091 2109 3082 3169 3824
TS190_7
12
E= -1837.70833721 ZPE= 35.22 Gcorr -0.008926000
H -2.064343 -0.372080 1.723144
Co -0.035234 -0.037205 0.433990
C -1.846071 -0.007990 0.685265
C -0.110578 -1.572975 -0.454846
O -2.726395 0.318640 -0.056874
O -0.223092 -2.521059 -1.098514
C -0.032140 1.482088 -0.498901
O 0.003409 2.417197 -1.167803
C 2.793578 -0.330357 -0.343514
O 2.657552 0.297386 0.600034
H 0.264943 -0.491757 1.997293
H 0.230200 0.346469 2.019064
FREQS= 38 50 55 63 76 84 104 147 263 330 363 403 442 480 501 510 527 620 834 867 976
1286 1582 1837 2094 2142 2169 2791 3002
TS191_7
12
E= -1837.70475143 ZPE= 36.38 Gcorr -0.012126000
H 0.584404 2.333416 -0.327283
Co -0.818915 0.191946 0.023750
C 0.044149 1.715258 0.401336
C 3.129686 0.197737 -0.199623
O 3.328524 1.260282 0.246437
O 2.941431 -0.864631 -0.649779
C -0.315205 -1.493889 0.274381
O -0.017434 -2.587009 0.468207
C -2.540659 0.325094 -0.199363
O -3.680098 0.444071 -0.315007
H 0.032108 2.269460 1.350450
H -0.993020 -0.272323 -1.323661
FREQS= 10 20 28 41 61 80 92 127 243 332 391 426 482 511 578 631 640 646 693 737 954
1371 1397 2095 2120 2156 2430 3048 3111
TS192_7
12
E= -1837.71643399 ZPE= 39.20 Gcorr -0.000330000
H 0.494878 1.128658 -2.495336
Co -0.051270 0.041015 -0.173566
C 0.820491 -1.451141 -0.374100
C 0.465654 1.403966 -1.435012
O 1.421384 -2.396165 -0.650254
O -0.723769 1.553490 -0.860826
C 0.843777 0.449543 1.345895
O 1.924597 0.933985 1.522206
C -2.744584 -0.819214 0.110293
O -2.228214 -0.345140 1.012240
H 1.299769 2.021917 -1.085935
H 0.125623 0.273734 2.198156
FREQS= 37 56 69 84 93 104 113 168 188 361 425 482 513 544 557 646 736 851 1089 1197 1270
1279 1561 1846 2110 2162 2726 3030 3121
TS193_7
12
E= -1837.72233807 ZPE= 39.01 Gcorr 0.005590000
H 0.383955 -0.060107 1.062217
Co -0.261302 -0.092137 -0.369386
C 1.644770 -0.461676 -0.762333
C -0.601790 1.618129 -0.218368

O 0.798310 -1.509519 -0.661731
O -0.793699 2.746401 -0.089087
C -1.775705 -0.801334 0.209404
O -2.710387 -1.243689 0.708042
C 2.288739 0.010210 0.454857
O 1.688769 0.038025 1.555382
H 2.155373 -0.293392 -1.711278
H 3.315791 0.399496 0.420275
FREQS= 64 77 88 108 214 248 367 404 431 452 482 526 528 571 648 795 821 928 988 1008 1207
1353 1388 1470 1659 2101 2158 3068 3134
TS194_7
12
E= -1837.72081817 ZPE= 40.10 Gcorr 0.004345000
H -2.099482 -1.388747 -0.489869
Co 0.160893 -0.121397 0.036872
C -1.572437 -0.913631 0.346793
C -0.619215 1.265484 -0.619404
O -2.426675 -0.152895 1.169081
O -1.164321 2.157963 -1.106960
C 1.758993 0.643509 0.450367
O 2.654540 1.208711 0.893182
C 0.733691 -1.459501 -1.316488
O 0.952389 -2.113570 -0.364815
H -1.152469 -1.703782 0.998580
H -3.025834 0.353417 0.604248
FREQS= 54 85 87 106 137 166 188 264 331 370 389 412 427 468 525 539 606 694 1074 1122 1244
1377 1448 1889 2087 2143 2941 3062 3815
TS195_7
12
E= -1837.71643866 ZPE= 37.26 Gcorr 0.000110000
H -0.000550 -1.094406 2.177524
Co 0.000066 -0.017735 -0.329626
C -0.000776 -1.433007 1.109645
C 0.000605 1.416563 0.688453
O -0.001496 -2.624700 0.890710
O 0.000946 2.300551 1.424341
C 1.755025 0.193531 -0.624406
O 2.895471 0.253172 -0.759892
C -1.754733 0.194814 -0.624590
O -2.895125 0.255295 -0.760116
H -0.000280 -1.374729 -1.178780
H -0.000042 -0.757975 -1.753805
FREQS= 55 83 98 99 107 128 282 310 392 406 443 467 473 547 587 599 614 686 732 816 1074
1377 1756 1821 2099 2105 2161 2765 2984
TS196_7
12
E= -1837.71788134 ZPE= 39.56 Gcorr 0.001694000
H -2.070755 -1.799419 -0.966398
Co -0.191035 -0.198600 -0.129824
C -1.154403 1.260700 -0.169153
C -1.284349 -1.774795 -0.202858
O -1.897919 2.133542 -0.284261
O -0.024441 -1.930601 -0.589343
C 0.181448 0.014808 1.632711
O 1.301712 0.306938 1.966051
C 2.228268 0.029979 -0.867495
O 1.821142 1.038032 -1.221628
H -1.583674 -2.173141 0.776190
H -0.617361 -0.232682 2.369689
FREQS= 52 67 73 85 118 127 138 165 183 306 432 480 507 565 616 660 759 891 1103 1196 1274
1297 1563 1798 2109 2147 2865 3007 3092
TS197_7
12
E= -1837.71942529 ZPE= 39.76 Gcorr 0.003375000
H 2.099537 -1.489244 1.041064
Co 0.239343 -0.153229 -0.148081
C 1.778911 -1.292362 0.008670
C -0.311787 0.040033 1.577999
O 0.671209 -1.876782 -0.430191
O -1.479821 0.099495 1.859752
C 0.729261 1.519440 -0.231736
O 1.204356 2.563861 -0.347228
C -2.217649 0.536741 -1.119308
O -1.835825 -0.543191 -1.096021
H 0.487934 -0.012689 2.354025
H 2.598490 -1.131061 -0.701153

FREQS=   69   77   85 114 122 130 158 180 184 319 442 486 505 566 615 659 769 894 1103 1196 1276
1300 1563 1809 2108 2131 2854 3005 3092
TS198_7
12
E= -1837.71931121 ZPE=   39.26 Gcorr   0.003476000
H -0.587446 1.234721 0.941242
Co 0.102719 0.157248 -0.136977
C -1.306033 0.347317 -1.274268
C 0.259717 -1.554387 -0.328151
O -0.824214 1.314795 -1.836303
O 0.356866 -2.701830 -0.397192
C 1.806445 0.559316 0.370653
O 2.907304 0.834483 0.533846
C -1.005740 0.327864 1.612366
O -2.199606 0.103941 1.639907
H -2.291739 -0.083163 -1.521546
H -0.343386 0.110956 2.473029
FREQS=   57   73   87   96 123 185 212 273 354 389 432 440 477 530 563 664 761 871 1145 1244 1412
1429 1721 1751 1981 2098 2153 2939 3005
TS199_7
12
E= -1837.71457151 ZPE=   36.21 Gcorr   -0.000502000
H 0.000000 0.297592 -2.177725
Co 0.000000 -0.500739 -0.118755
C 0.000000 0.960299 -1.233239
C 0.000000 0.642873 1.399554
O 0.000000 2.144164 -1.276470
O 0.000000 1.824012 1.550517
C -1.758198 -0.792576 -0.034939
O -2.875888 -1.033107 0.031595
C 1.758198 -0.792576 -0.034939
O 2.875888 -1.033107 0.031595
H 0.000000 -0.061701 2.279574
H 0.000000 -2.039751 -0.171965
FREQS=   67   82   88 112 181 228 235 324 371 406 421 435 466 494 510 545 579 640 804 870 1127
1249 1806 1855 1930 2132 2179 2470 2722
TS200_7
12
E= -1837.71857404 ZPE=   39.27 Gcorr   0.003625000
H -0.242424 -0.124920 1.659201
Co 0.226274 -0.182576 0.041216
C -0.607174 1.008771 1.539206
C -1.268951 -1.078608 -0.527521
O -1.799835 1.241271 1.526280
O -2.113838 -1.732985 -0.935541
C 0.372631 0.951852 -1.254915
O 0.452072 1.746775 -2.087246
C 1.981246 -0.194188 0.511226
O 1.992553 -1.410713 0.630162
H 2.885349 0.426902 0.650015
H 0.133541 1.745831 1.900731
FREQS=   63   76   84 106 136 169 192 261 372 385 411 444 472 524 566 660 762 870 1161 1261 1396
1423 1701 1749 2028 2101 2165 2955 2976
TS201_7
12
E= -1837.70035952 ZPE=   32.81 Gcorr   -0.014247000
H 0.823134 0.375234 2.572659
Co 0.025837 0.000030 0.275314
C 3.327462 0.000023 0.194395
C -0.120812 1.555418 -0.531480
O 2.189485 0.000112 0.299730
O -0.275272 2.461026 -1.230229
C -0.120545 -1.555452 -0.531352
O -0.274848 -2.461126 -1.230050
C -1.682992 -0.000140 0.813913
O -2.800506 -0.000221 0.387042
H 0.823430 -0.373285 2.573210
H -1.473693 -0.000174 1.935859
FREQS=   26   60   68   70   87 102 139 157 196 220 298 321 323 365 371 404 442 502 523 540 560
798 1229 1860 2070 2129 2150 2605 4340
TS202_7
12
E= -1837.71397088 ZPE=   35.94 Gcorr   -0.000471000
H -2.005098 0.656468 1.919883
Co 0.090925 0.062707 0.408700
C -1.684551 0.104973 1.017699
C 0.082439 1.582710 -0.512509

O -2.593684 -0.542138 0.455805
O 0.065110 2.602186 -1.040972
C 1.843746 -0.306234 0.465399
O 2.950106 -0.575720 0.603765
C -0.582101 -1.139488 -0.938571
O 0.009272 -1.695176 -1.780311
H -1.846229 -1.055889 -0.461623
H -0.007292 -1.058642 1.408439
FREQS= 66 83 90 100 183 248 340 356 366 423 432 465 467 513 526 558 595 700 728 924 1241
1302 1577 1651 1943 1982 2121 2163 2998
TS203_7
12
E= -1837.71871147 ZPE= 40.11 Gcorr 0.004351000
H 2.209785 -1.117574 0.615088
Co -0.148838 -0.124188 -0.004663
C 1.613410 -0.889239 -0.270891
C -1.737758 0.633966 -0.451517
O 2.434116 -0.134458 -1.137893
O -2.618208 1.181746 -0.945707
C 0.623804 1.291275 0.625524
O 1.118826 2.208047 1.113740
C -0.735098 -1.478791 1.307133
O -0.948419 -2.119783 0.345242
H 2.021778 -0.109901 -2.010760
H 1.310389 -1.847126 -0.742967
FREQS= 53 87 88 112 138 167 182 254 327 357 394 409 424 469 523 541 599 712 1053 1131 1242
1371 1453 1887 2097 2145 2904 3120 3822
TS204_7
12
E= -1837.70436825 ZPE= 34.38 Gcorr -0.009992000
H -2.015606 0.945387 -1.655345
Co -0.022083 0.072790 -0.404734
C -1.819561 0.266180 -0.797138
C 0.028057 1.542280 0.619643
O -2.717134 -0.271871 -0.208502
O 0.042274 2.527028 1.209409
C -0.287087 -1.428577 0.596986
O -0.394595 -2.402835 1.188650
C 3.444857 -0.381562 -0.385837
O 2.326209 -0.243432 -0.212939
H 0.301535 0.775777 -1.673537
H 0.058675 -0.547541 -1.758166
FREQS= 43 49 68 71 90 107 109 137 170 251 335 378 415 477 498 526 595 646 719 898 917
1313 1814 2009 2032 2137 2170 2181 2895
TS205_7
12
E= -1837.71707283 ZPE= 39.58 Gcorr 0.002901000
H 0.599822 -2.605024 -0.582509
Co -0.089928 -0.206129 0.152189
C 0.111576 -2.117549 0.270995
C 1.262366 0.611145 0.913262
O -1.138385 -1.691791 0.123088
O 2.151034 1.014280 1.524771
C 0.541565 0.074076 -1.529958
O 1.438900 0.737595 -1.970840
C -2.670932 1.715982 0.184233
O -1.692324 1.128071 0.123741
H -0.124314 -0.521338 -2.212229
H 0.411300 -2.515320 1.248369
FREQS= 55 61 91 109 128 147 183 195 203 332 438 474 501 524 606 649 780 829 1106 1199 1266
1276 1568 1831 2115 2147 2772 3011 3088
TS206_7
12
E= -1837.71573387 ZPE= 37.48 Gcorr 0.001577000
H 0.203161 -1.324806 2.462787
Co 0.119429 -0.130978 -0.093713
C 0.279049 -1.200370 1.371501
C -0.512255 1.345827 0.596159
O 0.454401 -2.125086 0.590316
O -0.917708 2.318450 1.049765
C 1.862727 0.350862 -0.492441
O 2.858223 0.784154 -0.854571
C -1.598674 -0.633872 -0.789950
O -2.627850 -0.047390 -0.981749
H -1.519906 -1.746300 -0.898157
H -0.229443 -0.008204 -1.576073

```
FREQS=   76   84   98  106  148  213  219  294  349  385  411  455  477  511  517  606  656  753  842  977 1215
1310 1679 1815 1898 2129 2167 2799 3033
TS207_7
12
E= -1837.71480362 ZPE=    38.24 Gcorr    0.000700000
H -0.803933 0.931569 1.608589
Co 0.043532 0.172973 -0.280028
C -1.967318 0.750287 -0.746813
C -0.144541 -1.570720 -0.376383
O -2.794531 0.932745 0.126300
O -0.160858 -2.715856 -0.495110
C 1.715425 0.724921 -0.580611
O 2.815504 0.940019 -0.836116
C 0.087941 0.239573 1.506701
O 0.726970 -0.191413 2.409485
H -0.972107 1.424381 -0.745249
H -2.245048 0.385467 -1.756430
FREQS=   64   70   82   85   99  175  180  235  325  357  367  452  484  496  518  557  737  809 1139 1177 1433
1472 1752 1889 1942 2096 2145 2690 2924
TS208_7
12
E= -1837.70932005 ZPE=    35.66 Gcorr   -0.004550000
H -0.594457 -0.163809 2.067471
Co -0.128507 -0.124906 -0.359101
C 0.259777 0.227198 1.449172
C -0.602980 1.590793 -0.501881
O 1.191354 0.766292 1.980828
O -0.703517 2.727349 -0.652113
C 1.512511 -0.740176 -0.565397
O 2.626706 -0.975620 -0.728521
C -1.350488 -1.918325 0.205715
O -2.303824 -1.300799 0.381998
H -0.924044 -0.275151 -1.879720
H -0.410478 -0.883305 -1.875197
FREQS=   52   66   81   96  113  136  157  216  330  349  367  408  435  491  499  522  535  557  603  835  885
1337 1429 1822 2062 2099 2146 2751 3567
TS209_7
12
E= -1837.71475062 ZPE=    37.31 Gcorr    0.001194000
H 0.367767 0.876849 -1.608114
Co 0.141084 0.248305 -0.215587
C 0.081550 -1.374303 -0.852517
C 1.866056 0.664138 -0.464735
O 0.035590 -2.424721 -1.311599
O 1.764285 1.581732 0.335544
C 0.072265 -0.401578 1.606886
O -0.466153 -1.323912 2.151565
C -1.567746 0.895246 -0.265072
O -2.628921 1.295031 -0.415502
H 2.776673 0.358513 -0.994278
H 0.695119 0.334351 2.195810
FREQS=   71   76   96  102  143  190  276  288  344  416  439  468  482  520  527  585  717  728  853  898 1211
1298 1677 1770 1833 2134 2173 2700 3084
TS210_7
12
E= -1837.71517321 ZPE=    37.54 Gcorr    0.001976000
H 0.619508 -2.251335 1.342971
Co 0.048830 0.044584 0.002023
C 0.509153 -1.761613 0.355817
C -0.585628 1.728257 -0.246275
O 0.664059 -2.359616 -0.685549
O -0.985059 2.799478 -0.189286
C -1.667131 -0.629121 -0.128909
O -2.699520 -0.808071 0.434725
C 1.828491 0.454322 0.031929
O 2.918521 0.740404 0.232087
H -0.120520 0.246227 1.425728
H -1.510705 -0.927300 -1.234507
FREQS=   77   84   95  118  133  198  285  351  396  404  434  454  456  511  533  624  648  721  862  909 1196
1319 1760 1892 2089 2136 2172 2463 2943
TS211_7
12
E= -1837.71508915 ZPE=    39.39 Gcorr    0.001913000
H 0.850974 -1.352719 -2.271394
Co -0.101504 -0.038661 -0.223822
C 0.551179 -1.543185 -1.234122
C 1.002752 1.313374 -0.374380
```

O -0.744478 -1.560613 -0.950842
O 1.779642 2.129615 -0.611892
C 0.488438 -0.387216 1.465463
O 1.405302 -1.075990 1.813398
C -2.471734 0.287991 0.518114
O -2.008743 1.317586 0.312422
H 1.224316 -2.185980 -0.657392
H -0.212279 0.071968 2.216840
FREQS= 61 67 77 100 113 122 141 179 190 357 432 468 497 545 567 633 750 859 1090 1202 1279
1301 1563 1839 2100 2124 2749 3027 3121
TS212_7
12
E= -1837.71169307 ZPE= 36.56 Gcorr -0.000849000
H 1.268762 -1.201074 -1.821558
Co -0.161225 0.075559 0.398758
C 1.121763 -1.258519 -0.724122
C -0.412439 1.701457 -0.179084
O 0.795586 -2.285103 -0.151295
O -0.626268 2.784106 -0.509005
C -1.808434 -0.614652 0.017134
O -2.815701 -1.047317 -0.316824
C 1.588029 0.092137 0.050584
O 2.685696 0.544454 -0.010641
H 0.177050 0.149305 2.006705
H -0.340765 -0.479975 1.963431
FREQS= 40 79 97 103 189 223 248 300 311 350 407 439 492 509 522 539 567 606 720 915 980
1343 1504 1681 1897 2107 2157 2937 3315
TS213_7
12
E= -1837.71169307 ZPE= 36.56 Gcorr -0.000849000
H 1.268763 -1.201074 -1.821558
Co -0.161225 0.075559 0.398758
C 1.121763 -1.258519 -0.724122
C -0.412439 1.701457 -0.179084
O 0.795587 -2.285103 -0.151295
O -0.626269 2.784106 -0.509005
C -1.808434 -0.614652 0.017134
O -2.815701 -1.047318 -0.316824
C 1.588029 0.092137 0.050584
O 2.685696 0.544454 -0.010641
H -0.340765 -0.479975 1.963431
H 0.177050 0.149305 2.006705
FREQS= 40 79 97 103 189 223 248 300 311 350 407 439 492 509 522 539 567 606 720 915 980
1343 1504 1681 1897 2107 2157 2937 3315
TS214_7
12
E= -1837.71169307 ZPE= 36.56 Gcorr -0.000849000
H 1.268762 -1.201074 -1.821558
Co -0.161225 0.075559 0.398758
C 1.121763 -1.258519 -0.724122
C -0.412439 1.701457 -0.179084
O 0.795586 -2.285103 -0.151295
O -0.626268 2.784106 -0.509005
C -1.808434 -0.614652 0.017134
O -2.815701 -1.047317 -0.316824
C 1.588029 0.092137 0.050584
O 2.685696 0.544454 -0.010641
H 0.177050 0.149305 2.006705
H -0.340765 -0.479975 1.963431
FREQS= 40 79 97 103 189 223 248 300 311 350 407 439 492 509 522 539 567 606 720 915 980
1343 1504 1681 1897 2107 2157 2937 3315
TS215_7
12
E= -1837.71169307 ZPE= 36.56 Gcorr -0.000849000
H 1.268763 -1.201074 -1.821558
Co -0.161225 0.075559 0.398758
C 1.121763 -1.258519 -0.724122
C -0.412439 1.701457 -0.179084
O 0.795587 -2.285103 -0.151295
O -0.626269 2.784106 -0.509005
C -1.808434 -0.614652 0.017134
O -2.815701 -1.047318 -0.316824
C 1.588029 0.092137 0.050584
O 2.685696 0.544454 -0.010641
H -0.340765 -0.479975 1.963431
H 0.177050 0.149305 2.006705

FREQS= 40 79 97 103 189 223 248 300 311 350 407 439 492 509 522 539 567 606 720 915 980
1343 1504 1681 1897 2107 2157 2937 3315
TS216_7
12
E= -1837.69544855 ZPE= 34.94 Gcorr -0.017070000
H 0.608244 -2.312213 0.474797
Co 0.335617 0.177958 -0.433704
C 1.143539 -1.562862 -0.165921
C -0.591187 1.677861 -0.597096
O 2.183499 -1.867307 -0.706121
O -1.308832 2.577850 -0.576490
C 1.163245 0.631408 1.016318
O 1.773356 0.989875 1.924959
C -3.299028 -1.419558 1.111054
O -2.626203 -1.395356 0.192404
H -0.257807 -0.794846 -1.630133
H -0.086058 -0.099398 -1.998790
FREQS= 8 16 32 35 51 58 88 105 183 230 296 349 429 446 498 550 559 586 632 868 1027
1363 1631 1771 2093 2145 2199 2763 3431
TS217_7
12
E= -1837.69576548 ZPE= 33.91 Gcorr -0.016476000
H 0.094932 -0.290882 -1.327807
Co 0.579763 -0.060070 0.108792
C 0.131263 -1.728744 -0.349445
C 2.289040 -0.106009 -0.071887
O -0.803347 -1.733309 0.435877
O 3.438192 -0.099963 -0.148105
C -0.091960 1.630951 0.043712
O -0.434710 2.712463 -0.103897
C -3.713364 -0.702483 -0.245701
O -3.330785 0.336497 0.018882
H 1.147545 0.194722 1.480229
H 0.459264 -2.569750 -0.971937
FREQS= 10 15 26 50 73 76 107 117 182 279 369 395 442 489 498 547 654 675 733 754 947
1228 1686 1821 1968 2118 2172 2204 3088
TS218_7
12
E= -1837.71168323 ZPE= 39.72 Gcorr -0.000462000
H -1.625664 1.917181 -0.111078
Co 0.135038 0.026412 -0.204216
C -1.442881 0.973799 -0.638844
C 0.892801 1.473420 0.425469
O -2.625154 0.227019 -0.787545
O 1.273346 2.340351 1.093042
C -0.695066 -1.239769 0.681748
O -1.214724 -1.894052 1.483291
C 2.608772 -1.413144 -0.286541
O 1.814444 -1.027808 -1.020739
H -1.086604 1.197178 -1.665428
H -3.098816 0.242605 0.054946
FREQS= 23 49 60 85 92 106 127 219 274 321 348 422 469 501 527 550 586 718 1074 1121 1262
1375 1450 2039 2090 2107 2918 3059 3815
TS219_7
12
E= -1837.69856190 ZPE= 33.68 Gcorr -0.013333000
H -2.010241 0.000056 1.771647
Co -0.065374 -0.000018 0.415892
C -1.868159 0.000079 0.645562
C -0.091342 -1.551379 -0.416850
O -2.816227 0.000156 -0.085668
O -0.118315 -2.463832 -1.124203
C -0.091186 1.551354 -0.416839
O -0.118075 2.463803 -1.124200
C 3.460811 0.000025 -0.022443
O 2.367088 -0.000116 0.304257
H 0.035679 -0.000058 2.352918
H 0.763148 -0.000080 2.148284
FREQS= 41 48 50 51 75 98 112 130 150 241 333 356 377 380 439 488 518 525 572 623 785
822 1274 1841 2065 2124 2169 2639 4230
TS220_7
12
E= -1837.71771802 ZPE= 41.04 Gcorr 0.006005000
H -1.663794 -0.489163 1.524208
Co 0.462782 -0.177980 0.149822
C -1.333150 -0.619446 0.491284
C 0.417000 1.528211 0.168082

O -0.738331 -1.907388 0.295443
O 0.367939 2.678840 0.165449
C 2.165689 -0.372720 -0.222754
O 3.277292 -0.494512 -0.500141
C -2.356264 -0.307876 -0.536672
O -3.172117 0.582136 -0.413431
H -2.305391 -0.946165 -1.447128
H 0.236160 -2.000825 0.099535
FREQS= 30 56 78 101 143 211 293 325 356 429 449 495 517 576 609 635 885 985 1091 1110 1268
1320 1412 1803 2093 2151 2903 3119 3267
TS221_7
12
E= -1837.71294621 ZPE= 37.62 Gcorr 0.001438000
H 0.441796 -0.476478 2.644633
Co -0.041789 -0.045164 -0.100818
C 1.659362 0.429348 -0.888507
C -0.399407 1.612372 0.287528
O 2.545939 -0.385644 -0.851189
O -0.647116 2.705541 0.546671
C -1.720979 -0.748851 -0.415875
O -2.769580 -1.048000 -0.764320
C 0.367907 -0.682506 1.565503
O 0.774060 -1.693580 1.026950
H 1.826462 1.456776 -1.267150
H 0.192315 -0.049580 -1.612175
FREQS= 74 89 90 102 138 196 226 236 323 403 425 457 481 517 535 626 649 757 838 982 1208
1356 1710 1791 1879 2113 2160 2920 3029
TS222_7
12
E= -1837.70195599 ZPE= 34.80 Gcorr -0.009322000
H -1.976267 1.051383 -1.641644
Co -0.024050 0.080195 -0.396287
C -1.804412 0.336086 -0.812296
C 0.112671 1.518918 0.659567
O -2.705866 -0.209068 -0.240759
O 0.158472 2.508537 1.236260
C -0.317497 -1.401226 0.635867
O -0.434167 -2.383106 1.209012
C 2.839956 0.249230 -0.402345
O 2.655223 -0.873039 -0.323624
H 0.342315 0.940618 -1.544333
H -0.090308 -0.721906 -1.646155
FREQS= 34 50 61 67 77 79 113 163 262 326 358 402 427 493 514 573 631 653 715 851 905
1290 1816 2012 2032 2145 2178 2183 2935
TS223_7
12
E= -1837.71812070 ZPE= 39.70 Gcorr 0.006992000
H -0.106200 -2.561152 -0.577376
Co -0.204248 -0.041720 0.015873
C 0.395818 -1.872114 0.107240
C -0.345209 1.749815 -0.148935
O 1.582063 -1.348480 -0.544035
O -0.398523 2.891902 -0.118625
C -1.957793 -0.465939 -0.047668
O -3.044733 -0.804035 0.082361
C 1.666119 -0.086902 0.110471
O 2.674899 0.521420 0.268882
H -0.078731 -0.003374 1.455853
H 0.636356 -2.344641 1.057634
FREQS= 60 83 93 112 265 323 352 414 429 468 485 523 545 568 574 666 675 811 828 1008 1182
1233 1513 1898 2094 2136 2179 3081 3175
TS224_7
12
E= -1837.72249441 ZPE= 42.74 Gcorr 0.011442000
H 3.147974 1.299754 0.046320
Co -0.495650 -0.092824 -0.065411
C 1.327267 -0.352853 -0.018801
C -0.765541 1.598740 -0.012759
O 0.580090 -1.663826 -0.074760
O -0.876525 2.745180 0.052307
C -2.172333 -0.608622 -0.000462
O -3.255518 -0.990476 0.095441
C 2.649883 -0.541516 0.015016
O 3.592047 0.439005 0.054078
H 0.579396 -2.077340 0.803693
H 3.098767 -1.529727 0.001588

```
FREQS=   55   68   95  190  196  285  399  416  436  448  482  498  513  551  597  664  704  808  875 1086 1167
1301 1380 1706 2084 2142 3211 3744 3792
TS225_7
12
E= -1837.71298185 ZPE=    37.59 Gcorr    0.002089000
H -0.577584 -2.471996 -0.633356
Co 0.132015 0.016666 0.325291
C -0.774201 -1.402012 -0.749813
C 0.001748 1.801136 -0.044239
O -0.051900 -0.621748 -1.503776
O -0.106903 2.873431 -0.432341
C -1.676882 -0.655371 0.094870
O -2.771057 -0.601299 0.564414
C 1.905337 -0.483456 0.309880
O 2.982342 -0.848157 0.173751
H -0.473283 -0.006991 1.828182
H 0.330596 0.049411 1.941760
FREQS=   61   70   94   96  216  265  311  351  390  416  424  438  451  483  559  576  621  643  818  945 1057
1265 1386 1684 1923 2127 2167 3099 3360
TS226_7
12
E= -1837.70908181 ZPE=    36.41 Gcorr   -0.001802000
H 2.100513 -0.324978 -1.856093
Co -0.028829 -0.258748 -0.263231
C 1.821388 -0.581965 -0.809747
C 0.044070 -0.199570 1.533319
O 2.650821 -0.998670 -0.035697
O -0.338047 0.387019 2.482741
C 0.110541 1.490426 -0.427149
O 0.169517 2.617665 -0.638723
C -1.799351 -0.602228 -0.408716
O -2.891288 -0.829148 -0.669861
H 0.695315 -1.129782 1.495402
H 0.194650 -1.613948 -0.966011
FREQS=   51   78   84   97  105  198  219  269  303  395  441  448  468  506  521  555  606  628  764  930 1078
1357 1763 1848 1932 2121 2165 2679 2862
TS227_7
12
E= -1837.71838069 ZPE=    40.24 Gcorr    0.007673000
H 1.870013 1.191911 -0.927314
Co -0.190825 -0.000361 0.025756
C 2.507576 0.612225 -0.021441
C -1.389679 -1.255635 0.001551
O 1.175417 1.295328 0.082982
O -2.166141 -2.104600 -0.012483
C -1.395013 1.247468 0.000796
O -2.172867 2.094907 -0.036875
C 2.364740 -0.764845 -0.022801
O 1.186550 -1.279753 -0.009716
H 3.223884 -1.438585 -0.073098
H 3.348957 1.174078 0.365108
FREQS=   44   70  103  114  243  353  371  442  452  478  482  502  538  578  611  670  791  878  941 1060 1112
1318 1380 1595 2100 2154 2401 3131 3238
TS228_7
12
E= -1837.71065965 ZPE=    37.79 Gcorr    0.000052000
H 2.616545 0.040824 -0.679833
Co -0.026772 -0.060004 -0.235818
C 1.727699 0.085697 -1.324551
C 0.658194 0.660224 1.308891
O 0.997643 1.139023 -1.334529
O 1.065600 1.174164 2.244855
C -1.731394 0.479152 -0.149166
O -2.809597 0.857501 -0.248899
C 0.435286 -2.104607 0.030267
O -0.442695 -2.163580 0.778056
H 1.710555 -0.605414 -2.175194
H -0.630557 -0.594949 -1.486392
FREQS=   64   75   83   87  119  137  154  272  351  356  394  417  423  467  519  536  688  752  885 1042 1219
1394 1594 1971 2022 2135 2174 3008 3100
TS229_7
12
E= -1837.70106896 ZPE=    34.01 Gcorr   -0.009474000
H 1.186454 0.997856 -1.545718
Co 0.308757 -0.406418 -0.154362
C -1.534384 1.618823 -0.845857
C 0.412200 0.376437 1.444944
```

O -2.534803 2.036205 -0.505270
O 0.589210 0.716672 2.526284
C -1.241398 -1.364750 -0.086268
O -2.066908 -2.158093 -0.039886
C 1.817089 0.308172 -0.871014
O 3.007040 0.312162 -0.906708
H 0.723809 -1.430264 -1.136734
H 1.075945 -1.481978 0.404034
FREQS= 35 46 77 82 98 111 124 222 277 312 396 414 436 472 509 514 534 616 634 774 824
1193 1896 2031 2129 2151 2173 2212 2500
TS230_7
12
E= -1837.71775293 ZPE= 40.81 Gcorr 0.007270000
H -1.548365 -1.811129 1.505146
Co -0.292158 -0.209323 -0.126515
C -1.432188 -1.696383 0.421212
C -1.314918 1.204075 -0.243469
O -0.280181 -2.019656 -0.136615
O -2.044323 2.088715 -0.330290
C 0.957844 0.471542 0.845078
O 1.448501 0.903673 1.830603
C 1.889150 -0.116999 -0.442304
O 1.953636 0.406294 -1.526770
H 2.565435 -0.925589 -0.101746
H -2.349201 -1.817178 -0.166016
FREQS= 52 92 94 114 122 202 225 338 414 427 464 494 544 574 628 698 738 920 1080 1200 1293
1333 1567 1764 1940 2133 2956 3026 3115
TS231_7
12
E= -1837.70254381 ZPE= 35.04 Gcorr -0.007595000
H -1.966022 1.152561 -1.565651
Co -0.003782 0.074353 -0.405115
C -1.791900 0.408259 -0.763191
C 0.263494 1.521206 0.621551
O -2.695299 -0.110777 -0.169635
O 0.383892 2.520248 1.169740
C -0.339870 -1.366466 0.668613
O -0.489951 -2.328490 1.267018
C 2.348233 -0.771422 -0.974081
O 2.686225 -0.344340 0.030369
H 0.340060 0.929045 -1.564551
H -0.230593 -0.731722 -1.628992
FREQS= 46 60 70 75 79 88 117 174 265 330 378 411 436 494 517 585 640 656 740 865 912
1298 1813 2017 2031 2142 2150 2182 2938
TS232_7
12
E= -1837.70177745 ZPE= 35.12 Gcorr -0.008185000
H -1.054592 -1.957297 -0.479002
Co -0.092495 0.113380 0.327701
C -1.473978 -1.148876 0.202120
C -0.988945 1.132688 -0.773350
O -2.560117 -1.293685 0.693938
O -1.614951 1.879595 -1.389654
C 1.411311 1.129322 0.466534
O 2.317133 1.825245 0.576423
C 2.250661 -2.338799 -0.988219
O 1.463883 -1.628429 -0.566627
H -0.517059 0.324110 1.808418
H 0.027134 -0.335900 1.867518
FREQS= 29 54 71 76 91 94 146 176 250 287 374 389 433 486 526 539 572 593 697 851 1074
1311 1746 1831 2096 2148 2177 2624 2824
TS233_7
12
E= -1837.70713468 ZPE= 36.98 Gcorr -0.002731000
H 3.316616 -0.180509 -0.918665
Co -0.080795 -0.190535 0.065270
C 2.363246 -0.734166 -0.931197
C -0.498221 1.523556 -0.289699
O 1.367945 -0.265525 -1.476714
O -0.855874 2.581822 -0.547393
C -1.645763 -0.865535 -0.141637
O -2.673908 -1.371408 -0.272966
C 0.248807 0.041023 2.032886
O 1.371938 0.173156 1.708422
H 2.349842 -1.732202 -0.464523
H 0.025786 -1.676456 0.307993

FREQS= 51 65 76 92 120 129 165 194 251 311 383 385 392 446 465 538 556 697 785 1162 1257
1521 1728 1877 1913 2104 2156 2978 3070
TS234_7
12
E= -1837.71213287 ZPE= 37.90 Gcorr 0.002310000
H 1.495661 -2.065602 -0.717646
Co -0.133256 -0.113470 -0.048028
C -0.886963 -1.776915 0.060703
C 1.540754 -0.956459 -0.561972
O -0.694306 -1.753264 1.265089
O 2.608780 -0.405078 -0.683487
C -1.751041 0.738169 -0.234410
O -2.671358 1.282833 -0.646407
C 0.809000 1.371696 0.469425
O 1.400973 2.302509 0.761150
H 0.001207 0.093042 -1.485733
H -1.322166 -2.638697 -0.473116
FREQS= 53 87 109 116 149 209 239 321 365 406 419 460 488 513 561 583 676 719 869 897 1242
1395 1710 1778 2059 2134 2185 2762 3007
TS235_7
12
E= -1837.71656094 ZPE= 40.90 Gcorr 0.006751000
H 0.653086 -2.401570 -0.811746
Co -0.164253 0.002956 -0.159502
C 0.541458 -1.660249 -0.006482
C -1.871869 -0.432420 -0.203337
O 1.024743 -2.215464 1.086564
O -2.969353 -0.724456 -0.399318
C -0.357754 1.692650 0.336919
O -0.466605 2.706801 0.873683
C 1.577343 0.429915 -0.838689
O 2.667835 0.635640 -0.370591
H 0.974719 -1.583159 1.824241
H 1.418997 0.505368 -1.959124
FREQS= 50 75 94 96 143 228 276 304 362 417 448 477 517 532 571 633 719 858 1039 1221 1308
1341 1485 1797 2082 2130 2638 3044 3727
TS236_7
12
E= -1837.70118158 ZPE= 35.23 Gcorr -0.008558000
H -0.046718 -1.141399 1.551491
Co 0.522019 -0.363186 0.223738
C -1.956446 0.885916 1.284151
C 0.138294 0.586751 -1.168869
O -2.707358 1.569440 0.772197
O -0.147406 1.331070 -2.000877
C 2.172258 0.268398 0.447677
O 3.301403 0.491516 0.450085
C -0.756289 -1.630107 -0.397761
O -1.957694 -1.708169 -0.423647
H 0.581976 -0.701331 1.829209
H -0.128232 -2.487863 -0.794896
FREQS= 30 46 64 90 96 98 102 181 252 308 354 383 401 451 480 535 556 582 591 841 1054
1299 1669 1804 2091 2140 2205 2636 3304
TS237_7
12
E= -1837.71282555 ZPE= 40.05 Gcorr 0.003131000
H -0.521857 2.464448 -0.725813
Co -0.291034 -0.124182 -0.231337
C 0.312885 1.776132 -0.545156
C -1.666664 0.483237 0.566904
O 0.642838 0.925701 -1.463106
O -2.661280 0.870113 1.014134
C -0.542024 -1.787821 0.319995
O -0.035052 -2.118041 -0.747104
C 2.137552 -0.345916 0.711181
O 2.908111 0.383633 1.124378
H 1.063184 2.128568 0.174527
H -0.970831 -2.485157 1.053432
FREQS= 24 71 107 121 135 150 167 202 213 328 412 441 521 535 637 708 761 845 1111 1216 1230
1368 1589 1677 2094 2184 3016 3057 3096
TS238_7
12
E= -1837.70924346 ZPE= 37.27 Gcorr -0.000278000
H -0.301486 -1.172683 -1.207998
Co 0.062922 -0.102913 -0.180892
C 0.569547 1.408682 0.950709
C -1.571748 0.644092 -0.470038

O 0.985610 2.446340 0.491375
O -2.565689 1.099426 -0.799942
C 1.773884 -0.583897 -0.324959
O 2.856994 -0.879533 -0.553494
C -0.565567 -1.717811 0.435322
O -1.688441 -1.834064 0.851160
H 0.453502 1.289944 2.060070
H 0.204610 -2.502369 0.573008
FREQS= 42 87 92 94 127 159 203 285 322 362 425 463 487 515 539 581 672 773 867 931 1290
1343 1762 1787 1849 2130 2186 2765 2935
TS239_7
12
E= -1837.70964181 ZPE= 39.62 Gcorr 0.000127000
H -1.380342 2.052435 -0.260722
Co 0.180770 -0.015166 -0.198410
C -1.319701 1.046828 -0.689510
C 0.997495 1.411582 0.410639
O -2.578475 0.441267 -0.771983
O 1.396199 2.291021 1.048357
C -0.806501 -1.195068 0.649423
O -1.427797 -1.798025 1.415514
C 1.993740 -1.232132 0.046472
O 2.022338 -1.380994 -1.091558
H -0.921019 1.104390 -1.724940
H -3.067736 0.639260 0.037944
FREQS= 39 49 56 97 99 115 128 194 268 316 328 437 455 502 529 544 570 690 1083 1112 1250
1373 1452 2047 2073 2116 2901 3075 3815
TS240_7
12
E= -1837.71299946 ZPE= 40.33 Gcorr 0.003634000
H 3.067757 -1.544213 0.709117
Co -0.169849 0.001624 -0.004077
C 1.151563 -1.424438 0.314707
C -1.400076 1.268057 0.185318
O 2.451245 -0.836094 0.479810
O -2.197488 2.019248 0.543843
C -1.309726 -1.246464 -0.218557
O -2.048827 -2.091182 -0.490362
C 2.187086 1.878586 -0.662014
O 1.259515 1.254443 -0.419308
H 1.181275 -2.192965 -0.473915
H 0.848261 -1.932438 1.246295
FREQS= 32 78 83 102 120 175 202 250 299 326 349 430 464 495 528 594 622 710 1014 1180 1245
1342 1481 2076 2124 2144 2956 2998 3791
TS241_7
12
E= -1837.71642112 ZPE= 41.22 Gcorr 0.007252000
H 0.574647 -2.357284 -0.905059
Co -0.139944 0.049206 -0.095180
C 0.932393 -1.349190 -0.653047
C -1.427215 -0.998528 0.513387
O 2.228966 -1.367566 -0.882433
O -2.182290 -1.679941 1.060761
C 1.077943 0.912789 0.835603
O 1.830615 1.351832 1.598759
C -1.249189 1.519390 -0.339568
O -1.063592 1.542444 -1.547052
H -1.916862 2.243708 0.165537
H 2.607518 -0.495914 -0.669143
FREQS= 53 88 94 104 119 174 244 298 348 459 472 496 522 528 628 633 720 877 1038 1246 1277
1345 1500 1703 2050 2105 2949 3059 3700
TS242_7
12
E= -1837.71525912 ZPE= 39.21 Gcorr 0.006108000
H 2.088741 0.535573 -1.711948
Co -0.081147 -0.026475 0.035785
C 1.740949 -0.116487 -0.899562
C -0.744773 1.581369 -0.113818
O 1.102567 -1.202093 -1.124365
O -1.245254 2.614599 -0.204128
C -1.551929 -1.020736 0.068861
O -2.509132 -1.649009 0.084679
C 1.852764 0.326796 0.466501
O 1.402807 -0.484932 1.415721
H 2.268816 1.304444 0.717224
H 0.043448 0.020636 1.521365

FREQS= 55 88 100 113 199 298 355 378 413 462 484 499 532 542 570 728 831 903 938 1040 1238
1331 1442 1512 1892 2113 2168 3066 3138
TS243_7
12
E= -1837.70704635 ZPE= 37.48 Gcorr -0.001976000
H 3.475621 -0.331638 -0.448504
Co -0.441570 -0.247073 0.305365
C 2.387222 -0.479701 -0.622534
C -0.453591 1.387698 -0.297361
O 1.943532 -0.964077 -1.640320
O -0.496198 2.490236 -0.619287
C -2.165768 -0.566983 0.013886
O -3.249479 -0.800802 -0.288135
C 1.469206 -0.046001 0.537286
O 1.992585 0.327825 1.568688
H -0.494817 -1.447847 1.405875
H -0.004338 -1.745066 0.842536
FREQS= 23 40 85 98 114 138 259 270 336 363 405 471 479 513 570 578 592 672 952 1029 1067
1341 1631 1750 1804 2107 2156 2922 3450
TS244_7
12
E= -1837.71448984 ZPE= 40.73 Gcorr 0.005563000
H 0.124622 2.308423 -0.864415
Co 0.128323 -0.110487 -0.051097
C -0.508358 1.441237 -0.634240
C 1.643851 0.548810 0.719445
O -1.759550 1.825663 -0.829257
O 2.607749 0.997674 1.160759
C -1.262335 -0.659597 0.969217
O -2.153619 -1.037878 1.597912
C 0.459576 -1.016477 -1.571046
O 0.844232 -1.935548 -0.847170
H 0.458501 -1.087126 -2.670753
H -2.354734 1.078743 -0.643416
FREQS= 46 66 76 98 122 165 298 317 352 398 431 445 465 499 602 675 740 814 1008 1217 1231
1327 1481 1636 2069 2118 3012 3070 3718
TS245_7
12
E= -1837.69611092 ZPE= 34.02 Gcorr -0.012756000
H 0.247078 -0.146445 1.405342
Co 0.197474 -0.173571 -0.105112
C 1.825995 -0.773814 0.477619
C -0.581468 -1.762761 -0.221414
O 2.821970 -0.102746 0.457888
O -1.137260 -2.757799 -0.337214
C 0.700102 1.546445 -0.295908
O 0.946178 2.652055 -0.443592
C -3.418536 1.261755 0.613412
O -2.446867 0.855862 0.178989
H 1.852437 -1.849500 0.751638
H -0.060059 -0.126377 -1.609773
FREQS= 19 27 50 59 72 87 132 158 249 281 340 401 443 502 515 566 631 672 675 731 893
1298 1801 1811 1954 2134 2193 2196 2906
TS246_7
12
E= -1837.70762148 ZPE= 39.09 Gcorr -0.001065000
H -3.424529 -0.745248 -0.727554
Co 0.097109 0.009268 -0.128343
C -1.501372 -0.797276 -0.547367
C 1.646951 0.773430 -0.555554
O -2.670855 -0.179719 -0.937638
O 2.542206 1.363212 -0.982710
C -0.530460 1.244127 0.863931
O -1.006962 2.088707 1.496126
C 1.137112 -2.332809 1.387654
O 0.976041 -2.042826 0.291693
H -1.672017 -1.819972 -0.179472
H -0.762236 -0.844831 -1.459479
FREQS= 34 41 74 86 95 135 139 162 228 316 329 422 441 492 549 575 633 745 1125 1169 1308
1321 1457 2067 2117 2132 2313 3015 3823
TS247_7
12
E= -1837.70085787 ZPE= 34.92 Gcorr -0.007811000
H -0.070404 -0.343276 -1.958805
Co -0.042599 0.074030 -0.400642
C 0.942134 -1.599021 1.560624
C -0.553310 1.269495 0.770614

O 1.068761 -2.255012 0.632678
O -0.894858 2.124111 1.462262
C 1.707116 0.560807 -0.555126
O 2.778466 0.944538 -0.695364
C -1.726115 -0.723014 -0.313245
O -2.841376 -0.491651 -0.687607
H -1.493597 -1.721650 0.183339
H -0.392736 0.440633 -1.880157
FREQS= 45 65 76 86 95 97 116 153 243 274 375 387 425 479 512 532 557 560 689 830 1072
1293 1720 1842 2106 2142 2156 2610 2885
TS248_7
12
E= -1837.70085787 ZPE= 34.92 Gcorr -0.007811000
H 1.493599 -1.721650 0.183334
Co 0.042598 0.074030 -0.400642
C 1.726116 -0.723012 -0.313247
C -1.707116 0.560805 -0.555127
O 2.841377 -0.491647 -0.687607
O -2.778467 0.944535 -0.695365
C 0.553308 1.269495 0.770615
O 0.894854 2.124110 1.462264
C -0.942130 -1.599019 1.560624
O -1.068758 -2.255014 0.632680
H 0.070404 -0.343275 -1.958806
H 0.392736 0.440634 -1.880157
FREQS= 45 65 76 86 95 97 116 153 243 274 375 387 425 479 512 532 557 560 689 830 1072
1293 1720 1842 2106 2142 2156 2610 2885
TS249_7
12
E= -1837.70085787 ZPE= 34.92 Gcorr -0.007811000
H -0.070404 -0.343276 -1.958805
Co -0.042599 0.074030 -0.400642
C 0.942134 -1.599021 1.560624
C -0.553310 1.269495 0.770614
O 1.068761 -2.255012 0.632678
O -0.894858 2.124111 1.462262
C 1.707116 0.560807 -0.555126
O 2.778466 0.944538 -0.695364
C -1.726115 -0.723014 -0.313245
O -2.841376 -0.491651 -0.687607
H -1.493597 -1.721650 0.183339
H -0.392736 0.440633 -1.880157
FREQS= 45 65 76 86 95 97 116 153 243 274 375 387 425 479 512 532 557 560 689 830 1072
1293 1720 1842 2106 2142 2156 2610 2885
TS250_7
12
E= -1837.70085787 ZPE= 34.92 Gcorr -0.007811000
H 1.493599 -1.721650 0.183334
Co 0.042598 0.074030 -0.400642
C 1.726116 -0.723012 -0.313247
C -1.707116 0.560805 -0.555127
O 2.841377 -0.491647 -0.687607
O -2.778467 0.944535 -0.695365
C 0.553308 1.269495 0.770615
O 0.894854 2.124110 1.462264
C -0.942130 -1.599019 1.560624
O -1.068758 -2.255014 0.632680
H 0.070404 -0.343275 -1.958806
H 0.392736 0.440634 -1.880157
FREQS= 45 65 76 86 95 97 116 153 243 274 375 387 425 479 512 532 557 560 689 830 1072
1293 1720 1842 2106 2142 2156 2610 2885
TS251_7
12
E= -1837.71259744 ZPE= 38.57 Gcorr 0.004059000
H -0.947572 -1.693880 1.558664
Co 0.121631 -0.000434 -0.142990
C -1.250319 -1.598699 0.507230
C 0.426205 1.711906 0.087967
O -0.338581 -1.818947 -0.458118
O 0.619352 2.842039 0.121564
C 1.866205 -0.531603 0.140029
O 2.935689 -0.934022 0.160567
C -1.681505 0.082614 0.057496
O -2.737537 0.585722 0.019854
H -2.239202 -2.054666 0.350032
H -0.092153 0.376625 -1.555249

FREQS=   45   84 102 117 179 291 330 363 392 405 451 470 501 511 579 593 634 721 866 1126 1173
1211 1533 1945 2002 2137 2176 2996 3050
TS252_7
12
E= -1837.71259744 ZPE=   38.57 Gcorr   0.004059000
H 0.947572 -1.693880 1.558664
Co -0.121630 -0.000434 -0.142990
C 1.250319 -1.598699 0.507230
C -1.866204 -0.531603 0.140029
O 0.338580 -1.818947 -0.458117
O -2.935688 -0.934023 0.160567
C -0.426205 1.711906 0.087967
O -0.619353 2.842039 0.121564
C 1.681505 0.082614 0.057496
O 2.737537 0.585722 0.019854
H 0.092154 0.376624 -1.555249
H 2.239202 -2.054666 0.350032
FREQS=   45   84 102 117 179 291 330 363 392 405 451 470 501 511 579 593 634 721 866 1126 1173
1211 1533 1945 2002 2137 2176 2996 3050
TS253_7
12
E= -1837.71259744 ZPE=   38.57 Gcorr   0.004059000
H -0.947572 -1.693880 1.558664
Co 0.121631 -0.000434 -0.142990
C -1.250319 -1.598699 0.507230
C 0.426205 1.711906 0.087967
O -0.338581 -1.818947 -0.458118
O 0.619352 2.842039 0.121564
C 1.866205 -0.531603 0.140029
O 2.935689 -0.934022 0.160567
C -1.681505 0.082614 0.057496
O -2.737537 0.585722 0.019854
H -2.239202 -2.054666 0.350032
H -0.092153 0.376625 -1.555249
FREQS=   45   84 102 117 179 291 330 363 392 405 451 470 501 511 579 593 634 721 866 1126 1173
1211 1533 1945 2002 2137 2176 2996 3050
TS254_7
12
E= -1837.71259744 ZPE=   38.57 Gcorr   0.004059000
H 0.947572 -1.693880 1.558664
Co -0.121630 -0.000434 -0.142990
C 1.250319 -1.598699 0.507230
C -1.866204 -0.531603 0.140029
O 0.338580 -1.818947 -0.458117
O -2.935688 -0.934023 0.160567
C -0.426205 1.711906 0.087967
O -0.619353 2.842039 0.121564
C 1.681505 0.082614 0.057496
O 2.737537 0.585722 0.019854
H 0.092154 0.376624 -1.555249
H 2.239202 -2.054666 0.350032
FREQS=   45   84 102 117 179 291 330 363 392 405 451 470 501 511 579 593 634 721 866 1126 1173
1211 1533 1945 2002 2137 2176 2996 3050
TS255_7
12
E= -1837.70871214 ZPE=   37.64 Gcorr   0.000282000
H 1.377338 -2.034273 -1.030358
Co -0.144708 -0.105004 -0.021118
C 1.481576 -0.951474 -0.792796
C 0.807163 1.367034 0.529760
O 2.583637 -0.440167 -0.814450
O 1.385841 2.263723 0.936148
C -1.710861 0.724976 -0.380842
O -2.616815 1.250597 -0.854417
C -0.890831 -1.745283 0.241021
O -0.690181 -1.654109 1.440269
H -1.317732 -2.643812 -0.239152
H 0.425377 -0.218680 -1.403560
FREQS=   51   79   94 110 119 124 202 254 351 383 407 441 479 502 516 572 658 881 896 1049 1246
1405 1707 1731 1944 2113 2176 2850 2990
TS256_7
12
E= -1837.70871206 ZPE=   37.64 Gcorr   0.000287000
H 1.378775 -2.033498 -1.030547
Co -0.144716 -0.104968 -0.021066
C 1.482540 -0.950619 -0.793227
C 0.805643 1.368043 0.529646

O 2.584401 -0.438844 -0.814152
O 1.383310 2.265387 0.936060
C -1.711406 0.723432 -0.381305
O -2.617912 1.248299 -0.854686
C -0.889117 -1.745917 0.241375
O -0.688469 -1.654644 1.440594
H -1.314974 -2.644939 -0.238829
H 0.426942 -0.218628 -1.403314
FREQS= 51 79 94 110 119 124 202 253 351 383 407 440 479 502 516 572 658 882 896 1050 1246
1405 1707 1731 1943 2113 2176 2850 2990
TS257_7
12
E= -1837.70152190 ZPE= 34.84 Gcorr -0.006780000
H 1.731251 1.331832 -1.719865
Co -0.172683 0.080100 -0.381785
C 1.604208 0.562761 -0.924627
C -0.222183 1.496584 0.700753
O 2.575534 0.028095 -0.442005
O -0.305236 2.496626 1.261214
C -1.887531 -0.530448 -0.209081
O -2.992189 -0.827732 -0.265085
C 1.230311 -2.361969 1.273585
O 0.640750 -1.539565 0.745959
H -0.174553 -0.440646 -1.786829
H -0.593942 0.685157 -1.629537
FREQS= 53 66 80 84 101 145 149 176 228 303 317 380 430 450 503 515 576 643 774 872 923
1374 1785 1986 2117 2141 2167 2172 2863
TS258_7
12
E= -1837.71352874 ZPE= 39.64 Gcorr 0.005257000
H -2.385761 0.800106 -1.719962
Co 0.247466 0.027106 -0.309957
C -1.679648 0.358816 0.026665
C 1.793023 -0.834155 -0.360906
O -2.545906 0.122162 -1.051118
O 2.738143 -1.483496 -0.303021
C 0.747084 1.612301 0.300308
O 1.014304 2.648660 0.719211
C -1.154913 -0.827119 0.636853
O -1.231292 -1.692333 1.448490
H -0.350060 -1.298628 -0.751939
H -1.981023 1.147670 0.714709
FREQS= 50 70 88 98 197 246 324 366 378 420 446 474 508 562 580 652 654 838 916 1019 1119
1264 1377 1914 1948 2111 2158 3134 3816
TS259_7
12
E= -1837.70639291 ZPE= 36.47 Gcorr -0.001741000
H -0.151715 1.123540 -1.254642
Co -0.074406 -0.014701 0.002616
C 1.422467 0.849223 0.980616
C 1.177412 -1.147658 -0.570227
O 2.084039 1.754191 0.548987
O 1.952209 -1.868389 -1.015874
C -1.288437 -1.256247 0.402725
O -2.020621 -2.055534 0.785021
C -1.118936 1.310691 -0.540685
O -2.007335 2.064871 -0.728211
H -0.669128 1.133614 0.828179
H 1.608420 0.442619 2.001871
FREQS= 27 83 90 96 108 209 258 316 346 415 468 478 523 533 539 556 613 703 778 874 1231
1330 1803 1933 2002 2086 2116 2155 2843
TS260_7
12
E= -1837.70693140 ZPE= 36.63 Gcorr -0.001114000
H 0.601111 -1.123892 -0.491697
Co -0.423432 -0.077603 -0.020602
C 1.309950 -0.270489 0.440687
C -1.962999 -1.042180 -0.115454
O 0.989856 -0.415108 1.627698
O -2.903711 -1.670217 -0.294347
C -0.850837 1.645670 -0.014238
O -1.108428 2.754736 -0.161587
C 2.733945 -0.118122 -0.049464
O 3.082107 -0.499803 -1.139909
H 3.413306 0.381535 0.669389
H -0.440709 0.191512 -1.445464

FREQS= 51 65 75 98 136 176 273 302 369 417 451 467 486 509 542 632 713 736 876 1014 1142 1375 1642 1819 1909 2087 2126 2170 2965
TS261_7
12
E= -1837.69566734 ZPE= 33.95 Gcorr -0.012362000
H -1.965512 0.000001 1.706158
Co 0.084241 0.000000 0.390926
C -1.758823 0.000001 0.595502
C -0.048045 -1.618897 -0.318008
O -2.666037 0.000000 -0.185988
O -0.177156 -2.602016 -0.905184
C -0.048043 1.618898 -0.318008
O -0.177153 2.602016 -0.905184
C 2.279515 0.000000 -0.673337
O 2.539265 -0.000002 0.448679
H 0.123745 0.000000 2.277705
H 0.868279 0.000000 2.125644
FREQS= 14 37 70 78 104 118 125 149 161 252 352 369 395 403 455 488 496 510 574 633 838 858 1303 1835 2063 2079 2136 2691 4162
TS262_7
12
E= -1837.69694562 ZPE= 35.23 Gcorr -0.011036000
H 0.874762 1.880022 -1.793328
Co -0.376314 -0.000408 -0.439489
C 1.124173 1.053332 -1.077663
C -0.487365 1.195861 0.801612
O 2.282018 0.856289 -0.779520
O -0.545399 1.935952 1.682271
C -1.966709 -0.713589 -0.119772
O -3.077584 -1.002913 -0.029438
C 2.579116 -1.470602 1.111481
O 1.571698 -1.824629 0.709387
H -0.171538 -1.298950 -1.434123
H 0.116091 -0.677653 -1.861897
FREQS= 27 32 39 63 86 95 111 123 197 250 314 353 434 441 498 546 562 585 621 872 1029 1364 1641 1768 2092 2145 2178 2768 3411
TS263_7
12
E= -1837.70535598 ZPE= 36.14 Gcorr -0.002623000
H -2.147374 0.931202 0.749416
Co 0.059767 0.084548 -0.254582
C 0.383874 2.110925 0.406665
C -0.297670 -1.427276 0.608771
O 0.269688 1.716382 1.485951
O -0.517913 -2.454445 1.076331
C -1.846047 0.377442 -0.181211
O -2.677164 0.075810 -0.996606
C 1.852762 -0.204112 -0.442711
O 2.944694 -0.481045 -0.650735
H -0.020631 0.800253 -1.715495
H -0.157654 -0.009763 -1.828821
FREQS= 54 79 87 93 139 148 232 234 317 328 380 391 431 474 506 525 594 603 702 896 1033 1352 1674 1815 2023 2113 2157 2753 3146
TS264_7
12
E= -1837.70535597 ZPE= 36.14 Gcorr -0.002623000
H 2.147387 -0.930958 0.749664
Co -0.059721 -0.084514 -0.254553
C 1.846076 -0.377402 -0.181090
C -1.852742 0.204009 -0.442727
O 2.677207 -0.075974 -0.996546
O -2.944707 0.480786 -0.650781
C 0.297595 1.427390 0.608722
O 0.517700 2.454641 1.076165
C -0.383764 -2.110901 0.406637
O -0.269752 -1.716400 1.485957
H 0.020726 -0.800086 -1.715556
H 0.157791 0.009931 -1.828780
FREQS= 54 79 87 93 139 148 232 234 317 328 380 391 431 474 506 525 594 603 702 896 1033 1352 1674 1815 2023 2113 2157 2753 3146
TS265_7
12
E= -1837.70926563 ZPE= 37.74 Gcorr 0.001334000
H -0.630491 -1.808142 1.436940
Co 0.047218 -0.139756 -0.390139
C 0.212775 -1.143370 1.155317
C -0.668538 1.346304 0.371409

```
O 1.195867 -1.037087 1.834945
O -1.101864 2.230394 0.951347
C 1.834955 0.360963 -0.634164
O 2.926412 0.521539 -0.928806
C -1.671663 -0.865329 -0.803710
O -2.742330 -0.387125 -0.497474
H -1.670193 -1.761357 -1.477312
H 0.555959 -1.470255 -0.839085
FREQS=   67   80   84  116  125  172  277  283  329  333  383  408  479  520  593  633  674  857  889  901 1277
1360 1756 1806 1971 2150 2183 2782 2912
TS266_7
12
E= -1837.70891749 ZPE=    36.49 Gcorr    0.001002000
H 1.674027 0.045377 1.895369
Co -0.174019 0.074736 -0.236185
C 1.355996 0.697705 1.063377
C -1.933604 0.521274 -0.228720
O 0.384787 1.495540 1.181158
O -3.024453 0.856260 -0.288160
C 1.735959 0.317073 -0.311481
O 2.689047 0.167859 -1.004880
C -0.251232 -1.642546 0.291341
O -0.330267 -2.777353 0.443510
H -0.016867 0.952756 -1.419961
H -0.154258 -0.315496 -1.638534
FREQS=   77   78   90  118  234  269  312  321  364  394  437  444  490  516  574  593  661  719  756  891  923
1334 1529 1930 2034 2118 2143 2180 2998
TS267_7
12
E= -1837.70891749 ZPE=    36.49 Gcorr    0.001002000
H -1.674004 -0.045371 1.895364
Co 0.174027 -0.074738 -0.236187
C -1.355988 -0.697712 1.063377
C 0.251197 1.642545 0.291335
O -0.384780 -1.495547 1.181159
O 0.330212 2.777352 0.443512
C 1.933618 -0.521244 -0.228709
O 3.024470 -0.856221 -0.288155
C -1.735954 -0.317086 -0.311484
O -2.689043 -0.167881 -1.004882
H 0.016915 -0.952786 -1.419951
H 0.154270 0.315462 -1.638546
FREQS=   77   78   90  118  234  269  312  321  364  394  437  444  490  516  574  593  661  719  756  891  923
1334 1529 1930 2034 2118 2143 2180 2998
TS268_7
12
E= -1837.70891749 ZPE=    36.49 Gcorr    0.001002000
H 1.674027 0.045377 1.895369
Co -0.174019 0.074736 -0.236185
C 1.355996 0.697705 1.063377
C -1.933604 0.521274 -0.228720
O 0.384787 1.495540 1.181158
O -3.024453 0.856260 -0.288160
C 1.735959 0.317073 -0.311481
O 2.689047 0.167859 -1.004880
C -0.251232 -1.642546 0.291341
O -0.330267 -2.777353 0.443510
H -0.016867 0.952756 -1.419961
H -0.154258 -0.315496 -1.638534
FREQS=   77   78   90  118  234  269  312  321  364  394  437  444  490  516  574  593  661  719  756  891  923
1334 1529 1930 2034 2118 2143 2180 2998
TS269_7
12
E= -1837.70891749 ZPE=    36.49 Gcorr    0.001002000
H -1.674004 -0.045371 1.895364
Co 0.174027 -0.074738 -0.236187
C -1.355988 -0.697712 1.063377
C 0.251197 1.642545 0.291335
O -0.384780 -1.495547 1.181159
O 0.330212 2.777352 0.443512
C 1.933618 -0.521244 -0.228709
O 3.024470 -0.856221 -0.288155
C -1.735954 -0.317086 -0.311484
O -2.689043 -0.167881 -1.004882
H 0.016915 -0.952786 -1.419951
H 0.154270 0.315462 -1.638546
```

FREQS=   77   78   90 118 234 269 312 321 364 394 437 444 490 516 574 593 661 719 756 891 923
1334 1529 1930 2034 2118 2143 2180 2998
TS270_7
12
E= -1837.71368722 ZPE=   40.09 Gcorr   0.005883000
H -2.612409 -1.386213 1.218126
Co -0.376370 -0.175198 0.370754
C 1.340240 0.731364 0.119611
C -1.240964 1.130321 -0.441958
O 1.537033 1.924863 0.029202
O -1.856003 1.845811 -1.094531
C 0.147966 -1.548236 -0.583759
O 0.344286 -2.406636 -1.321833
C -1.649907 -0.886533 1.349387
O 2.398348 -0.125772 0.304249
H -1.391436 -0.792337 2.420077
H 3.192517 0.441281 0.355059
FREQS=   66   73   92 102 126 233 314 324 426 450 472 474 492 505 568 598 663 715 773 959 1070
1288 1408 1766 2114 2156 2999 3160 3662
TS271_7
12
E= -1837.69810238 ZPE=   33.63 Gcorr   -0.009649000
H -0.928651 0.114875 2.537150
Co -0.181369 0.006410 0.244009
C -2.378080 -0.713550 0.288847
C 0.141206 -1.526045 -0.547281
O -2.363419 0.439811 0.170984
O 0.420523 -2.421820 -1.216347
C 0.148182 1.552642 -0.563687
O 0.420815 2.433904 -1.251172
C 1.508695 -0.043265 0.947176
O 2.625233 -0.031366 0.515490
H -0.832924 0.824057 2.315043
H 1.313309 -0.094909 2.057600
FREQS=   60   65   76   94 106 132 157 170 201 272 330 373 391 407 427 442 458 498 524 529 577
837 1276 1844 2002 2088 2141 2724 4319
TS272_7
12
E= -1837.71544298 ZPE=   41.34 Gcorr   0.007706000
H -1.821321 1.338033 1.101532
Co 0.114474 -0.001040 0.108518
C -1.193896 1.242846 0.202941
C 1.491619 1.061279 0.273091
O -1.630476 2.121970 -0.678363
O 2.376914 1.801129 0.291033
C 1.054552 -1.470372 -0.212232
O 1.668561 -2.401962 -0.494717
C -1.492670 -1.089940 -0.222110
O -2.106893 -1.590683 0.692158
H -1.088248 2.070223 -1.483100
H -1.803723 -1.286688 -1.279451
FREQS=   59   71   92   96 148 218 268 323 373 434 450 500 533 554 580 627 732 875 1033 1213 1330
1371 1475 1764 2086 2134 2783 3053 3741
TS273_7
12
E= -1837.71612191 ZPE=   41.67 Gcorr   0.008470000
H -1.236473 -0.013781 1.028600
Co 0.481567 -0.074959 -0.006173
C -1.401018 -0.203677 -0.061730
C 0.956337 1.578302 -0.064591
O -0.754132 -1.562903 -0.182440
O 1.238751 2.689785 -0.162905
C 2.079562 -0.761185 0.147762
O 3.116887 -1.260491 0.187153
C -2.876503 -0.304555 -0.105271
O -3.631537 0.544656 0.320298
H -0.821481 -1.850509 -1.106883
H -3.254375 -1.253491 -0.548920
FREQS=   58   60   90   97 162 198 370 394 426 437 468 505 564 587 650 730 820 888 1036 1124 1176
1359 1424 1791 2096 2151 2829 2888 3768
TS274_7
12
E= -1837.70422737 ZPE=   37.17 Gcorr   -0.003257000
H -2.023112 -1.336363 -1.012058
Co 0.111251 -0.018461 -0.237105
C -1.014951 -1.292085 -1.455631
C 0.299045 -0.682740 1.421552

O -0.033754 -1.866880 -0.885584
O 0.470655 -1.116807 2.468433
C 1.564358 0.916387 -0.335208
O 2.565394 1.466666 -0.501068
C -2.550695 1.924220 0.473336
O -1.739180 1.144526 0.280389
H -0.968884 -1.048256 -2.523156
H 0.096763 0.668331 -1.584607
FREQS= 45 53 80 91 95 104 144 153 322 336 395 419 448 468 526 537 632 655 798 1052 1219
1422 1604 1918 2099 2153 2159 2981 3089
TS275_7
12
E= -1837.71256672 ZPE= 39.38 Gcorr 0.005139000
H 0.401986 -2.633134 0.833101
Co -0.251354 -0.070255 -0.099706
C 0.530055 -1.859931 0.075517
C 1.662581 -0.028392 0.028167
O 1.887425 -1.392910 0.031399
O 2.549670 0.777022 -0.018592
C -2.009470 -0.480149 -0.096714
O -3.095089 -0.798661 0.087233
C -0.383679 1.716452 -0.038753
O -0.406191 2.849394 0.124983
H -0.109508 -0.777160 1.153647
H 0.210652 -2.259473 -0.904164
FREQS= 46 80 90 97 156 274 325 360 419 443 473 521 534 574 600 681 714 841 972 1058 1161
1259 1487 1846 2124 2147 2174 2954 3140
TS276_7
12
E= -1837.71062817 ZPE= 39.33 Gcorr 0.003331000
H 0.330960 -0.665110 2.451503
Co 0.251351 0.068616 0.447435
C -1.321618 -0.653747 -0.397960
C 1.527358 -0.466607 -0.553903
O -1.532937 -1.141880 -1.479314
O 2.371295 -0.841496 -1.237730
C -0.079387 1.789794 -0.083367
O -0.191178 2.885543 -0.397168
C 1.079515 -0.829114 1.646286
O -2.289675 -0.605313 0.581599
H -3.079289 -1.054608 0.221472
H 1.866611 -1.549688 1.840859
FREQS= 56 70 76 88 132 155 276 326 361 408 439 481 490 509 558 608 651 660 772 840 1026
1259 1344 1814 2126 2162 2929 3236 3659
TS277_7
12
E= -1837.70745542 ZPE= 37.24 Gcorr 0.000209000
H 0.023921 -1.542667 -0.859969
Co -0.107271 -0.119154 -0.335137
C 0.219373 -1.268201 1.056236
C 1.625871 0.122102 -0.807927
O 1.302995 -1.387805 1.547546
O 2.686608 0.197279 -1.227872
C -1.874662 -0.357860 -0.431846
O -2.983997 -0.574443 -0.624403
C -0.305601 1.749265 0.229608
O -0.248713 2.074356 1.392256
H -0.696641 -1.720833 1.487518
H -0.475986 2.533725 -0.555502
FREQS= 55 80 90 109 126 155 202 301 364 409 442 454 504 523 537 567 652 715 863 869 1261
1361 1768 1821 1847 2124 2177 2745 2927
TS278_7
12
E= -1837.69763620 ZPE= 33.64 Gcorr -0.009384000
H 2.498446 -0.445875 0.780593
Co -0.023415 -0.098345 -0.054629
C 1.873848 -0.213443 -0.115284
C -1.820378 0.040446 -0.429659
O 2.370118 -0.155301 -1.216529
O -2.899256 0.241118 -0.755738
C 0.210767 1.556328 0.388139
O 0.338843 2.645539 0.741964
C -0.173799 -1.263526 1.584683
O -0.110030 -2.094852 0.756851
H -0.205356 -1.653754 -1.944228
H 0.199080 -1.055925 -2.141033

FREQS=   47   79   86 102 131 145 151 188 237 247 276 336 362 382 396 411 432 466 532 554 618
890 1340 1779 1892 2103 2159 2837 4353
TS279_7
12
E= -1837.71505356 ZPE=   41.58 Gcorr   0.008411000
H 3.086370 0.041051 0.642537
Co -0.239261 -0.176305 0.182233
C 2.253716 -0.240619 -0.009576
C 1.211171 0.873974 -0.211693
O 1.412747 -1.241014 0.660286
O 1.469815 1.990721 -0.585743
C -1.395010 1.102934 0.215311
O -2.169847 1.953208 0.292041
C -1.044564 -1.407485 -1.045356
O -1.591096 -2.015349 -0.199534
H 1.621089 -2.140644 0.376417
H 2.627772 -0.613528 -0.967778
FREQS=   62   71   92 119 137 183 312 320 371 403 426 469 498 538 588 621 895 966 974 1140 1235
1338 1480 1830 1887 2112 3060 3131 3828
TS280_7
12
E= -1837.70750817 ZPE=   37.51 Gcorr   0.000972000
H -0.401216 -0.517986 -1.465138
Co 0.239674 -0.090588 -0.179221
C -0.522551 -1.536876 0.416498
C 2.002946 -0.558465 -0.362900
O -1.032310 -2.503152 0.769647
O 2.038110 0.478019 -1.017106
C 0.132411 1.010923 1.271759
O -0.012055 1.637121 2.221348
C -1.164813 0.902029 -1.170575
O -2.360788 0.839744 -1.040303
H 2.876831 -1.221196 -0.253886
H -0.698420 1.665524 -1.839388
FREQS=   66   75   86 110 143 154 246 288 347 405 426 445 475 508 519 588 627 813 851 992 1203
1354 1663 1790 1943 2124 2160 2822 3020
TS281_7
12
E= -1837.70669418 ZPE=   37.19 Gcorr   0.000453000
H 0.000008 2.934693 -0.282697
Co -0.000001 0.062431 -0.094615
C 0.000005 1.963342 0.259111
C -0.000002 -1.580637 0.827001
O 0.000009 1.916967 1.475941
O -0.000004 -2.761875 0.654371
C -1.718595 0.088994 -0.533065
O -2.792866 0.043078 -0.933682
C 1.718593 0.088987 -0.533068
O 2.792863 0.043066 -0.933686
H -0.000006 -0.768011 -1.268807
H 0.000001 -1.146325 1.882676
FREQS=   67   81   98 103 113 167 212 346 365 413 443 455 485 524 534 589 638 742 793 823 1239
1297 1722 1867 2120 2144 2170 2585 2881
TS282_7
12
E= -1837.70669418 ZPE=   37.19 Gcorr   0.000453000
H 0.000003 2.934696 -0.282691
Co 0.000000 0.062432 -0.094615
C 0.000001 1.963342 0.259112
C 1.718593 0.088991 -0.533068
O 0.000003 1.916961 1.475942
O 2.792863 0.043070 -0.933686
C 0.000000 -1.580635 0.827004
O -0.000001 -2.761873 0.654370
C -1.718594 0.088993 -0.533067
O -2.792864 0.043073 -0.933684
H -0.000002 -0.768018 -1.268801
H 0.000000 -1.146328 1.882680
FREQS=   67   81   98 103 113 167 212 346 365 413 443 455 485 524 534 589 638 742 793 823 1239
1297 1722 1867 2120 2144 2170 2585 2881
TS283_7
12
E= -1837.70669418 ZPE=   37.19 Gcorr   0.000453000
H 0.000008 2.934693 -0.282697
Co -0.000001 0.062431 -0.094615
C 0.000005 1.963342 0.259111
C -0.000002 -1.580637 0.827001

O 0.000009 1.916967 1.475941
O -0.000004 -2.761875 0.654371
C -1.718595 0.088994 -0.533065
O -2.792866 0.043078 -0.933682
C 1.718593 0.088987 -0.533068
O 2.792863 0.043066 -0.933686
H -0.000006 -0.768011 -1.268807
H 0.000001 -1.146325 1.882676
FREQS= 67 81 98 103 113 167 212 346 365 413 443 455 485 524 534 589 638 742 793 823 1239
1297 1722 1867 2120 2144 2170 2585 2881
TS284_7
12
E= -1837.70669418 ZPE= 37.19 Gcorr 0.000453000
H 0.000003 2.934696 -0.282691
Co 0.000000 0.062432 -0.094615
C 0.000001 1.963342 0.259112
C 1.718593 0.088991 -0.533068
O 0.000003 1.916961 1.475942
O 2.792863 0.043070 -0.933686
C 0.000000 -1.580635 0.827004
O -0.000001 -2.761873 0.654370
C -1.718594 0.088993 -0.533067
O -2.792864 0.043073 -0.933684
H -0.000002 -0.768018 -1.268801
H 0.000000 -1.146328 1.882680
FREQS= 67 81 98 103 113 167 212 346 365 413 443 455 485 524 534 589 638 742 793 823 1239
1297 1722 1867 2120 2144 2170 2585 2881
TS285_7
12
E= -1837.70576775 ZPE= 37.38 Gcorr -0.000443000
H 0.744071 2.453760 0.414064
Co -0.147965 0.024051 -0.219349
C 0.361925 1.475941 0.746507
C 0.102679 -1.806546 0.263224
O 0.318292 1.085555 1.884045
O 0.286454 -2.887562 0.578357
C -1.941766 0.282138 -0.278868
O -3.030648 0.531241 -0.539753
C 1.609415 0.102992 -0.980137
O 2.536272 0.806186 -0.638656
H -0.166777 0.866055 -1.408621
H 1.741287 -0.579695 -1.859330
FREQS= 61 65 76 86 119 180 220 277 299 353 399 416 443 474 576 630 670 722 856 917 1221
1360 1746 1782 2078 2133 2175 2773 3036
TS286_7
12
E= -1837.70768623 ZPE= 36.80 Gcorr 0.001517000
H 2.495904 -0.390451 1.650119
Co -0.229131 -0.085250 -0.189479
C 1.689288 -0.732668 0.970588
C -0.291534 1.639873 0.321992
O 0.642433 -1.234143 1.410578
O -0.378608 2.778219 0.427191
C 1.626088 -0.293158 -0.478522
O 2.506410 -0.133779 -1.275851
C -1.970267 -0.606343 -0.158528
O -3.039717 -0.987119 -0.294783
H -0.059799 -1.230739 -1.107238
H -0.415173 0.491306 -1.497207
FREQS= 67 78 91 110 235 289 333 345 366 411 439 481 502 562 606 631 652 669 763 876 960
1338 1646 1856 2034 2128 2142 2180 2955
TS287_7
12
E= -1837.70442724 ZPE= 37.60 Gcorr -0.001680000
H 2.135748 1.289244 -0.868992
Co -0.058107 -0.094328 -0.211839
C 1.365451 0.731114 -1.421762
C 0.010364 1.174190 1.103571
O 0.185192 1.241207 -1.551476
O -0.035362 2.021130 1.871647
C -1.740835 -0.615058 -0.069341
O -2.843534 -0.934667 -0.128421
C 2.161115 -1.878600 0.621616
O 1.084961 -1.595736 0.891978
H 1.739039 0.033316 -2.181686
H -0.212532 -1.101042 -1.303994

FREQS= 44 69 74 81 107 126 140 181 343 382 398 429 432 481 532 553 704 736 874 1026 1212 1358 1576 1956 2120 2130 2166 2992 3076
TS288_7
12
E= -1837.70419186 ZPE= 35.31 Gcorr -0.001855000
H 0.000003 0.666894 2.550307
Co 0.000002 -0.073715 -0.161106
C 0.000009 0.920780 1.473056
C 1.675089 0.310260 -0.605529
O 0.000026 2.142826 1.183489
O 2.744289 0.485057 -0.987652
C -0.000038 -1.796460 0.383337
O -0.000067 -2.883993 0.743736
C -1.675061 0.310326 -0.605551
O -2.744260 0.485146 -0.987665
H 0.000022 1.628864 -0.116482
H 0.000013 -0.607177 -1.571109
FREQS= 68 80 101 107 119 192 352 359 416 417 460 488 494 527 527 557 614 677 686 859 921 1316 1437 1618 1917 2117 2131 2179 2962
TS289_7
12
E= -1837.70419182 ZPE= 35.31 Gcorr -0.001853000
H 0.000001 -0.666857 2.550254
Co 0.000000 0.073821 -0.161105
C 0.000001 -0.920759 1.473009
C -0.000001 1.796159 0.383365
O 0.000001 -2.142796 1.183439
O -0.000001 2.883686 0.743812
C 1.674988 -0.310265 -0.605570
O 2.744204 -0.485083 -0.987639
C -1.674988 -0.310266 -0.605570
O -2.744204 -0.485085 -0.987639
H 0.000001 -1.628793 -0.116566
H -0.000001 0.607478 -1.571029
FREQS= 68 80 101 107 119 192 352 359 417 417 460 488 494 527 527 557 614 677 686 859 921 1316 1437 1618 1917 2117 2131 2179 2962
TS290_7
12
E= -1837.69529584 ZPE= 33.05 Gcorr -0.010714000
H 1.710322 0.233941 1.838538
Co -0.088449 -0.066827 0.166365
C 0.340771 -1.220980 -1.443204
C 0.031763 1.619532 -0.202159
O 0.274514 -2.037417 -0.603867
O 0.095018 2.719751 -0.538198
C -1.924590 -0.190687 0.128023
O -3.064766 -0.155215 0.230862
C 1.713887 0.045893 0.722671
O 2.753470 -0.157693 0.155025
H -0.490495 -1.118955 2.366206
H -0.268582 -1.788615 2.120845
FREQS= 54 72 88 97 137 146 165 190 199 251 273 287 300 353 381 400 421 451 537 548 556 870 1308 1816 1908 2101 2156 2676 4378
TS291_7
12
E= -1837.71437499 ZPE= 41.33 Gcorr 0.008430000
H 0.916597 1.990248 -0.368465
Co -0.606394 -0.118089 -0.503615
C 1.073945 0.912220 -0.500181
C -0.615359 -1.662769 0.486350
O 0.721437 0.335333 -1.632246
O 0.458173 -2.217333 0.477610
C -1.851062 0.644091 0.413600
O -2.616774 1.305621 0.966091
C 2.197479 0.339049 0.304852
O 2.628917 0.896515 1.293440
H -1.501607 -2.132209 0.965906
H 2.593598 -0.626259 -0.066726
FREQS= 55 77 95 120 144 211 244 342 387 456 481 536 582 621 671 849 911 1026 1094 1269 1315 1357 1453 1772 1796 2110 2894 2973 3067
TS292_7
12
E= -1837.71026224 ZPE= 39.12 Gcorr 0.004375000
H -0.181432 -0.962665 0.758546
Co 0.388945 -0.094101 -0.318700
C -1.458129 -0.446603 -0.816300
C 0.541841 1.650827 -0.121208

O -0.713192 -1.543836 -0.452579
O 0.582042 2.779624 0.090120
C 1.963755 -0.663228 0.243655
O 2.962071 -1.043128 0.662771
C -2.440113 0.064083 0.166615
O -2.464477 -0.259084 1.340288
H -3.158409 0.806588 -0.245217
H -1.737352 -0.402270 -1.869806
FREQS= 55 69 90 107 119 226 269 394 420 448 468 508 526 576 655 749 814 932 938 1035 1175
1386 1413 1781 1889 2113 2165 2912 3135
TS293_7
12
E= -1837.70976466 ZPE= 40.14 Gcorr 0.004018000
H 2.609644 -0.801285 -0.633928
Co 0.054198 -0.003625 -0.149370
C 1.744156 -0.617981 -1.286774
C 0.585028 -0.900952 1.284709
O 1.380875 0.563925 -1.557968
O 0.787065 -1.504183 2.245889
C -0.177341 1.642423 0.505668
O -0.417486 2.678497 0.941653
C -1.385491 -0.610738 -0.655628
O -2.603669 -1.037955 -0.622985
H -3.257736 -0.316359 -0.567252
H 1.412347 -1.463267 -1.906406
FREQS= 49 72 79 88 112 124 258 291 345 390 439 473 512 517 545 565 647 683 1054 1063 1230
1440 1494 1617 2094 2138 2997 3076 3687
TS294_7
12
E= -1837.70500962 ZPE= 36.98 Gcorr -0.000720000
H 0.000030 -1.288564 -1.078660
Co -0.000064 -0.005799 -0.306842
C 0.000318 -1.127441 1.124052
C 1.744712 -0.072956 -0.663754
O 0.001003 -2.277406 1.395348
O 2.832408 -0.215392 -0.996727
C -1.744902 -0.073585 -0.663524
O -2.832569 -0.216491 -0.996387
C -0.000321 1.875672 0.316725
O -0.000394 2.132501 1.503104
H -0.000317 -0.269031 1.861039
H -0.000422 2.718339 -0.421344
FREQS= 36 68 89 98 153 178 229 301 360 429 444 458 481 510 519 558 663 773 808 856 1117
1358 1749 1917 1928 2123 2172 2718 2776
TS295_7
12
E= -1837.70500962 ZPE= 36.98 Gcorr -0.000720000
H -0.000302 -0.269030 1.861040
Co -0.000061 -0.005800 -0.306842
C 0.000304 -1.127440 1.124052
C -0.000306 1.875671 0.316725
O 0.000957 -2.277405 1.395349
O -0.000374 2.132501 1.503103
C 1.744716 -0.072971 -0.663749
O 2.832412 -0.215418 -0.996720
C -1.744898 -0.073570 -0.663529
O -2.832565 -0.216465 -0.996395
H -0.000401 2.718338 -0.421345
H 0.000028 -1.288565 -1.078658
FREQS= 36 68 89 98 153 178 229 301 360 429 444 458 481 510 519 558 663 773 808 856 1117
1358 1749 1917 1928 2123 2172 2718 2776
TS296_7
12
E= -1837.70500962 ZPE= 36.98 Gcorr -0.000720000
H 0.000030 -1.288564 -1.078660
Co -0.000064 -0.005799 -0.306842
C 0.000318 -1.127441 1.124052
C 1.744712 -0.072956 -0.663754
O 0.001003 -2.277406 1.395348
O 2.832408 -0.215392 -0.996727
C -1.744902 -0.073585 -0.663524
O -2.832569 -0.216491 -0.996387
C -0.000321 1.875672 0.316725
O -0.000394 2.132501 1.503104
H -0.000317 -0.269031 1.861039
H -0.000422 2.718339 -0.421344

FREQS=   36   68   89   98 153 178 229 301 360 429 444 458 481 510 519 558 663 773 808 856 1117
1358 1749 1917 1928 2123 2172 2718 2776
TS297_7
12
E= -1837.70500962 ZPE=   36.98 Gcorr   -0.000720000
H -0.000302 -0.269030 1.861040
Co -0.000061 -0.005800 -0.306842
C 0.000304 -1.127440 1.124052
C -0.000306 1.875671 0.316725
O 0.000957 -2.277405 1.395349
O -0.000374 2.132501 1.503103
C 1.744716 -0.072971 -0.663749
O 2.832412 -0.215418 -0.996720
C -1.744898 -0.073570 -0.663529
O -2.832565 -0.216465 -0.996395
H -0.000401 2.718338 -0.421345
H 0.000028 -1.288565 -1.078658
FREQS=   36   68   89   98 153 178 229 301 360 429 444 458 481 510 519 558 663 773 808 856 1117
1358 1749 1917 1928 2123 2172 2718 2776
TS298_7
12
E= -1837.70969785 ZPE=   39.05 Gcorr   0.004039000
H -1.715556 -0.064943 1.540087
Co 0.429286 -0.110861 0.040099
C -1.403219 -0.474753 0.578377
C 0.545667 1.647899 0.043444
O -0.548466 -1.559509 0.610136
O 0.617090 2.786866 0.172020
C 2.093760 -0.594971 -0.293169
O 3.181250 -0.914165 -0.475205
C -2.427640 -0.483955 -0.489020
O -3.378370 0.272572 -0.509650
H -2.251198 -1.230464 -1.295995
H 0.552605 -0.962787 1.257037
FREQS=   56   65   91 105 136 195 306 409 415 444 451 492 502 548 579 683 766 953 1021 1124 1163
1331 1425 1795 1943 2115 2165 2900 3134
TS299_7
12
E= -1837.70984638 ZPE=   39.04 Gcorr   0.004237000
H -1.671012 -0.858479 1.514966
Co 0.474748 -0.128205 0.213745
C -1.327515 -0.783665 0.482206
C 0.328588 1.626828 0.131942
O -0.402561 -1.741717 0.106743
O 0.185314 2.755350 -0.023806
C 2.188710 -0.376234 -0.142445
O 3.289389 -0.547802 -0.418313
C -2.357121 -0.398118 -0.509795
O -3.350237 0.244546 -0.235415
H -2.143105 -0.736826 -1.550118
H 0.224724 -1.039042 -0.941084
FREQS=   56   66   90 104 141 226 298 407 422 434 467 487 504 548 584 678 765 959 1024 1110 1154
1328 1423 1801 1933 2117 2166 2882 3137
TS300_7
12
E= -1837.70316662 ZPE=   36.16 Gcorr   -0.002273000
H -1.120097 -0.270357 -0.928483
Co -0.043513 0.090165 0.116938
C -1.768624 -0.310584 0.348332
C -0.170083 1.841672 -0.283771
O -2.923624 -0.547755 0.372953
O -0.212188 2.919171 -0.671504
C 1.706548 0.149736 0.363483
O 2.839751 0.206776 0.553371
C 0.367350 -1.847245 -0.055981
O 0.564286 -2.401082 -1.099972
H 0.402385 -2.412246 0.907499
H -1.064377 -0.170216 1.392497
FREQS=   28   67   89   98 109 193 300 326 380 431 456 462 495 521 568 589 653 679 773 895 1157
1350 1822 1841 1927 1989 2115 2162 2819
TS301_7
12
E= -1837.71071542 ZPE=   40.14 Gcorr   0.005312000
H -1.083391 -0.920023 1.615489
Co 0.057169 0.152644 -0.228635
C -1.523385 -0.273496 -0.929793
C 1.803209 -0.182662 -0.461903

O -2.673505 -0.676898 -0.389701
O 2.877620 -0.383608 -0.822115
C -0.013011 1.850570 0.341235
O -0.162451 2.840899 0.909482
C -0.184227 -1.215646 1.007344
O 0.458307 -2.187908 1.290031
H -3.358613 -0.847589 -1.054917
H -1.596850 -0.166252 -2.030306
FREQS= 59 77 87 89 152 201 279 322 361 375 436 469 490 507 545 567 652 825 912 1208 1262
1284 1457 1819 2082 2131 2746 2942 3745
TS302_7
12
E= -1837.70954516 ZPE= 40.17 Gcorr 0.004247000
H -2.660903 -0.612171 -0.669067
Co -0.065097 -0.003298 -0.148813
C -1.788705 -0.432688 -1.314029
C 0.394424 1.593189 0.512073
O -1.358477 0.742704 -1.503550
O 0.766413 2.589232 0.944942
C -0.697488 -0.830800 1.287123
O -0.956117 -1.410599 2.249997
C 1.313538 -0.725486 -0.670306
O 2.545007 -1.119627 -0.638531
H -1.515458 -1.247179 -2.000444
H 2.628758 -2.090587 -0.624571
FREQS= 53 73 80 91 115 124 260 291 346 391 439 470 512 515 547 567 649 685 1049 1066 1229
1439 1487 1617 2092 2145 2995 3072 3703
TS303_7
12
E= -1837.69984121 ZPE= 34.96 Gcorr -0.005268000
H 1.974152 0.703591 -1.787804
Co -0.117197 -0.014506 -0.339380
C 0.428108 -2.067737 0.660777
C 0.004979 1.599115 0.444879
O 0.427363 -1.247046 1.471608
O 0.058755 2.684988 0.808692
C 1.725682 0.113696 -0.878017
O 2.598726 -0.447878 -0.260925
C -1.918503 -0.329008 -0.177061
O -3.049643 -0.461221 -0.284070
H -0.116663 -0.861903 -1.556149
H -0.416369 0.422847 -1.678702
FREQS= 65 79 79 100 122 155 179 182 239 304 341 380 424 448 485 509 582 639 746 864 924
1373 1789 2039 2055 2136 2159 2181 2876
TS001_8
18
E= -1916.41983500 ZPE= 74.23 Gcorr 0.051021000
H 4.912782 0.934404 -0.000048
Co -0.891968 0.028988 0.000032
C -1.213725 -1.719597 0.002921
C -1.162721 0.933700 1.516541
O -1.605306 -2.806913 0.004711
O -1.499464 1.502118 2.461534
C -1.162933 0.928619 -1.519441
O -1.499831 1.493909 -2.466258
C 3.391999 -0.604023 -0.000102
C 3.822546 0.860815 0.000006
H 3.791025 -1.149751 -0.869186
H 3.791179 -1.149927 0.868796
H 3.442415 1.382073 -0.882014
H 3.442516 1.381899 0.882173
C 1.914487 -0.828630 -0.000011
O 1.099232 0.088396 -0.000026
H -2.350565 0.046922 0.000164
H 1.558826 -1.873670 0.000072
FREQS= 43 55 63 65 81 131 139 165 206 240 302 340 358 372 445 446 457 512 518 528 687
700 725 727 880 922 1015 1120 1145 1281 1382 1416 1434 1454 1511 1514 1759 2064 2070 2079 2149 3004 3022
3040 3069 3145 3146
TS002_8
18
E= -1916.41544964 ZPE= 74.02 Gcorr 0.048768000
H -4.677060 0.884955 -0.985309
Co 0.822375 -0.000003 -0.150755
C 1.368306 1.565178 -0.722722
C 1.648044 0.000015 1.430434
O 1.789388 2.521833 -1.214214
O 2.239380 0.000029 2.416265

C 1.368436 -1.565132 -0.722744
O 1.789602 -2.521744 -1.214246
C -3.511037 -0.000040 0.632602
C -4.691178 -0.000034 -0.341228
H -3.539631 0.873079 1.298194
H -3.539618 -0.873165 1.298183
H -4.677077 -0.885030 -0.985300
H -5.639122 -0.000022 0.202162
C -2.167750 -0.000015 -0.045366
O -1.115898 -0.000072 0.582644
H 0.302335 -0.000007 -1.556067
H -2.142642 0.000065 -1.150930
FREQS= 29 40 52 73 83 88 129 134 193 217 318 320 336 391 452 481 501 517 554 574 575 665 738 746 905 918 1050 1134 1151 1283 1339 1419 1437 1454 1516 1517 1757 1947 2073 2099 2154 2987 3041 3055 3072 3124 3138
TS003_8
18
E= -1916.42181188 ZPE= 77.33 Gcorr 0.055382000
H 2.524952 -0.134990 1.893507
Co -0.686386 0.022317 0.018743
C -2.170047 -0.264279 0.924213
C -1.191165 1.681893 -0.516414
O -3.059238 -0.443599 1.631787
O -1.522865 2.656490 -1.016723
C -0.604318 -1.651842 -0.571701
O -0.613747 -2.666395 -1.109768
C 3.390509 0.229169 -0.087834
C 3.442025 0.271202 1.455192
H 3.497958 1.236489 -0.504586
H 4.225303 -0.361558 -0.485510
H 4.281837 -0.308212 1.850291
H 3.545183 1.292810 1.830754
C 2.096668 -0.369139 -0.645638
O 1.011276 0.470109 -0.311802
H 1.969087 -1.389185 -0.244529
H 2.182668 -0.452771 -1.740847
FREQS= 23 40 55 79 94 100 104 183 218 278 300 380 407 427 435 465 480 519 583 590 671 828 875 965 1037 1082 1123 1159 1260 1295 1371 1406 1438 1492 1508 1518 1524 2102 2115 2181 2956 3000 3043 3054 3086 3118 3126
TS004_8
18
E= -1916.41453710 ZPE= 74.12 Gcorr 0.048313000
H -3.175584 -0.000196 1.522080
Co 0.797087 0.000009 -0.152841
C 1.470209 1.566697 -0.560698
C 1.179823 -0.000109 1.588669
O 2.005427 2.523626 -0.923936
O 1.492434 -0.000195 2.695057
C 1.470250 -1.566603 -0.560905
O 2.005493 -2.523468 -0.924273
C -3.595087 0.000050 -0.613049
C -4.042073 -0.000108 0.857310
H -3.985152 -0.864953 -1.170068
H -3.985138 0.865184 -1.169877
H -4.643147 0.881887 1.089147
H -4.643175 -0.882136 1.088946
C -2.114352 0.000069 -0.847924
O -1.275931 0.000007 0.044861
H 0.663491 0.000074 -1.645560
H -1.784662 0.000147 -1.902043
FREQS= 26 37 42 71 88 89 130 134 159 181 316 333 336 392 455 481 504 520 556 574 681 705 739 744 864 968 1049 1100 1128 1261 1365 1415 1431 1461 1517 1523 1765 1947 2073 2098 2154 2999 3026 3044 3074 3144 3151
TS005_8
18
E= -1916.41148746 ZPE= 73.80 Gcorr 0.046349000
H -4.431871 -0.016727 -1.518015
Co 0.699799 -0.000997 -0.150324
C 1.163945 1.565831 -0.781315
C 1.682016 0.007647 1.340550
O 1.534931 2.517409 -1.319930
O 2.394647 0.012673 2.243125
C 1.158277 -1.578133 -0.759300
O 1.525824 -2.538501 -1.284515
C -3.598451 0.005845 0.480215
C -3.449507 -0.009977 -1.038812
H -4.172907 0.878740 0.827132

H -4.172137 -0.860178 0.845223
H -2.902443 0.868523 -1.389826
H -2.899416 -0.893851 -1.371079
C -2.303863 0.014175 1.241535
O -1.198617 0.008610 0.729378
H 0.106705 -0.009565 -1.523373
H -2.391286 0.026102 2.346995
FREQS= 8 34 45 75 82 101 104 168 168 237 240 320 340 373 453 483 497 521 553 566 666 669 707 735 860 902 1009 1116 1142 1281 1376 1427 1437 1459 1511 1512 1801 1958 2076 2099 2154 2946 3016 3039 3067 3142 3144
TS006_8
18
E= -1916.42072630 ZPE= 77.34 Gcorr 0.055986000
H -4.975508 -0.292905 1.444562
Co 0.763064 -0.014619 0.057723
C 2.245026 0.518779 0.848041
C 1.414175 -1.666395 -0.318290
O 3.140970 0.863903 1.481788
O 1.824066 -2.654074 -0.726866
C 0.486026 1.563487 -0.710489
O 0.368787 2.506245 -1.355951
C -3.298818 -0.674252 0.099560
C -4.127912 0.242323 1.004220
H -2.925964 -1.529344 0.673175
H -3.934876 -1.085278 -0.692985
H -3.519828 0.637653 1.825817
H -4.529939 1.097105 0.448603
C -2.064781 0.023224 -0.521044
O -0.890498 -0.662745 -0.129824
H -2.043104 1.078804 -0.203633
H -2.142392 0.019052 -1.619215
FREQS= 24 42 61 79 91 99 130 193 253 297 319 369 382 411 438 462 482 520 585 590 676 776 890 956 1040 1066 1137 1162 1272 1295 1355 1414 1426 1498 1514 1518 1523 2102 2114 2180 2962 3002 3037 3052 3088 3111 3112
TS007_8
18
E= -1916.40985600 ZPE= 73.84 Gcorr 0.046166000
H -4.210488 1.455399 0.208397
Co 0.913438 0.022023 -0.200186
C 1.460377 1.669839 -0.438022
C 0.843595 0.013838 1.582685
O 1.952964 2.691378 -0.653972
O 0.889967 0.019724 2.732172
C 1.834881 -1.449815 -0.435182
O 2.554829 -2.326564 -0.649162
C -3.359978 -0.559198 0.220406
C -4.533592 0.421760 0.054679
H -2.895822 -0.480266 1.207613
H -3.731213 -1.588815 0.106375
H -4.976081 0.349434 -0.944223
H -5.317321 0.201270 0.783558
C -2.303641 -0.358968 -0.829358
O -1.116423 -0.218663 -0.597172
H 1.225870 0.058691 -1.662225
H -2.658327 -0.342081 -1.880632
FREQS= 11 35 45 57 76 92 101 154 170 214 320 322 341 373 453 482 493 506 521 553 566 707 736 757 877 921 1016 1128 1160 1276 1332 1424 1437 1474 1514 1519 1800 1962 2077 2098 2153 2942 3024 3055 3110 3130 3139
TS008_8
18
E= -1916.41762314 ZPE= 77.03 Gcorr 0.054857000
H -4.264416 -0.002357 -1.740069
Co 0.856412 0.000046 0.275895
C 2.559555 -0.000691 -0.152157
C 0.573211 1.718845 -0.175463
O 3.688172 -0.001082 -0.371547
O 0.266564 2.754233 -0.559621
C 0.572116 -1.718721 -0.174666
O 0.265262 -2.754175 -0.558499
C -2.478341 -0.000792 -0.489542
C -3.997947 -0.000378 -0.678804
H -2.043908 -0.883175 -0.975617
H -2.043035 0.879730 -0.978225
H -4.456107 -0.883464 -0.219901
H -4.455237 0.884980 -0.223433
C -2.071056 0.001203 0.989780
O -0.690237 0.001325 1.218438

H -2.521968 -0.883790 1.472724
H -2.521753 0.887625 1.470312
FREQS= 28 32 74 81 85 104 116 122 223 266 272 372 408 433 436 448 488 535 543 587 618 765 896 904 1038 1121 1155 1181 1278 1322 1340 1411 1426 1501 1511 1517 1538 2109 2112 2182 2947 2970 3038 3042 3078 3111 3117
TS009_8
18
E= -1916.41069663 ZPE= 73.99 Gcorr 0.049155000
H -4.599443 0.790830 -0.550862
Co 0.615623 0.030505 -0.132946
C 1.095731 -1.458669 -0.920923
C 0.868664 1.676948 -0.694036
O 1.454715 -2.346125 -1.566859
O 1.090525 2.690082 -1.196700
C 1.775429 0.008360 1.227930
O 2.552910 0.001765 2.072371
C -3.128941 -0.687533 0.031374
C -3.607606 0.756326 -0.092957
H -3.829111 -1.302853 0.618153
H -3.076880 -1.188217 -0.946516
H -3.662390 1.237630 0.886556
H -2.922957 1.341388 -0.712107
C -1.783777 -0.848784 0.673901
O -1.124635 0.084623 1.135015
H -0.229059 0.060285 -1.372276
H -1.407103 -1.885355 0.764232
FREQS= 20 46 60 74 81 100 103 165 214 250 317 320 338 379 450 478 496 517 552 576 661 676 719 751 862 898 1008 1117 1132 1279 1378 1416 1431 1455 1509 1515 1724 1952 2079 2109 2163 2970 3018 3047 3067 3141 3144
TS010_8
18
E= -1916.41636236 ZPE= 76.69 Gcorr 0.054896000
H 2.932303 -1.173754 -1.454423
Co -0.626486 -0.041805 0.013566
C -2.016181 -0.627919 -0.898108
C -0.211155 -1.622494 0.761399
O -2.894329 -1.007239 -1.534912
O 0.049845 -2.541887 1.392397
C -1.333649 1.570893 0.402093
O -1.792119 2.517682 0.856605
C 3.047768 0.784879 -0.504139
C 3.363437 -0.709559 -0.558529
H 3.512976 1.233731 0.381623
H 3.464240 1.298689 -1.380548
H 4.443182 -0.886328 -0.583594
H 2.955501 -1.219736 0.317803
C 1.545979 1.087606 -0.435022
O 0.981989 0.549621 0.736625
H 1.089048 0.652411 -1.361731
H 1.377596 2.177881 -0.497293
FREQS= 21 47 77 79 86 100 123 159 232 281 298 391 411 422 446 453 480 502 577 589 654 781 871 922 989 1071 1121 1164 1272 1308 1369 1383 1425 1491 1497 1510 1523 2114 2118 2186 2756 2952 3037 3038 3079 3106 3135
TS011_8
18
E= -1916.41595874 ZPE= 76.68 Gcorr 0.054707000
H -5.084823 -0.337511 0.839831
Co 0.734556 -0.011593 -0.007482
C 2.146878 -0.131177 1.036537
C 0.749452 -1.725475 -0.561915
O 3.048666 -0.207931 1.744966
O 0.764046 -2.745641 -1.081175
C 1.015638 1.691420 -0.514323
O 1.209565 2.699394 -1.022626
C -3.113335 -0.222845 -0.077020
C -4.105769 0.108357 1.039782
H -2.993662 -1.307742 -0.178262
H -3.485331 0.141962 -1.041496
H -3.761853 -0.271319 2.008884
H -4.249460 1.190050 1.139802
C -1.729531 0.396604 0.143614
O -0.880296 0.093552 -0.937270
H -1.343521 -0.007122 1.113545
H -1.830233 1.488408 0.288494
FREQS= 22 59 72 78 88 97 127 137 228 265 277 363 403 415 445 449 477 502 576 590 657 766 895 904 1038 1052 1125 1184 1273 1318 1334 1405 1426 1499 1510 1513 1525 2115 2121 2189 2778 2940 3039 3050 3085 3109 3117

TS012_8
18
E= -1916.40906710 ZPE= 74.02 Gcorr 0.048169000
H -5.215007 0.310741 -0.429094
Co 0.722563 0.012058 -0.137146
C 1.561065 1.458642 -0.681297
C 1.664259 -0.330931 1.344441
O 2.152509 2.321646 -1.164185
O 2.303493 -0.556392 2.270817
C 0.757318 -1.583558 -0.859427
O 0.861581 -2.566061 -1.457456
C -3.050415 0.488521 -0.348928
C -4.391651 -0.016844 0.210420
H -2.922756 0.117727 -1.376063
H -3.002060 1.580476 -0.380997
H -4.418889 -1.110452 0.255914
H -4.572287 0.369465 1.217717
C -1.883214 -0.017455 0.445512
O -1.009262 0.708447 0.923232
H 0.067296 0.263744 -1.461779
H -1.856226 -1.108628 0.633657
FREQS= 16 37 53 71 78 99 102 136 214 256 318 339 376 385 450 478 494 514 518 552 575
719 745 751 864 923 1017 1119 1163 1272 1331 1421 1431 1472 1514 1519 1722 1954 2080 2110 2162 2960 3034
3055 3112 3131 3138
TS013_8
18
E= -1916.41015288 ZPE= 73.98 Gcorr 0.049434000
H -2.727681 -0.534099 1.412657
Co 0.737632 0.091698 -0.158699
C 0.566361 1.809469 0.133789
C 0.628970 -0.477177 1.527719
O 0.602652 2.955460 0.275689
O 0.615557 -0.814165 2.625913
C 2.158643 -0.818090 -0.667061
O 3.158401 -1.269540 -1.018197
C -3.070937 0.159077 -0.618620
C -3.430675 -0.698552 0.591647
H -3.795553 0.030069 -1.438230
H -3.108265 1.233709 -0.388395
H -4.434238 -0.453293 0.948290
H -3.403032 -1.761808 0.341376
C -1.720065 -0.122288 -1.208905
O -0.980029 -1.030547 -0.827462
H 1.008360 0.663840 -1.517392
H -1.422081 0.501432 -2.072386
FREQS= 20 49 64 71 82 95 112 164 223 249 317 323 343 372 451 480 497 513 555 573 659
679 696 749 862 896 1007 1116 1132 1279 1377 1414 1432 1457 1510 1514 1724 1957 2079 2107 2160 2978 3018
3048 3066 3139 3144
TS014_8
18
E= -1916.40873032 ZPE= 73.94 Gcorr 0.048193000
H -3.103308 1.216301 -0.176432
Co 0.708991 0.026679 -0.179652
C 0.010014 1.579754 -0.586233
C 1.223378 0.402449 1.484859
O -0.340816 2.617101 -0.955685
O 1.606631 0.691500 2.528298
C 2.179229 -0.764182 -0.741722
O 3.153178 -1.159923 -1.212901
C -2.994489 -0.876238 0.446453
C -3.696213 0.300435 -0.250153
H -2.818919 -0.687063 1.509381
H -3.631902 -1.771675 0.375284
H -3.863444 0.091779 -1.311800
H -4.669353 0.488308 0.210184
C -1.689831 -1.241381 -0.199924
O -0.669444 -1.528277 0.425129
H 0.443429 -0.132230 -1.648189
H -1.688180 -1.293987 -1.306233
FREQS= 11 51 53 66 79 97 105 160 226 263 321 322 344 371 453 481 493 511 517 557 574
685 740 745 878 914 1022 1123 1164 1273 1331 1421 1434 1467 1514 1518 1732 1947 2075 2107 2161 2964 3015
3057 3109 3131 3140
TS015_8
18
E= -1916.40717652 ZPE= 73.74 Gcorr 0.047335000
H 4.405623 -0.028129 -1.077363
Co -0.633059 0.000776 -0.146638

C -1.668378 -1.211393 -0.890670
C -1.747129 0.573596 1.122576
O -2.341807 -1.910365 -1.511605
O -2.514789 0.973882 1.877495
C -0.062752 1.551989 -0.722987
O 0.240341 2.532662 -1.254198
C 2.841624 -0.959563 0.108095
C 3.618504 0.272324 -0.381525
H 2.343539 -1.488134 -0.708722
H 3.543539 -1.664459 0.581903
H 2.959278 0.974926 -0.898310
H 4.093153 0.801672 0.451427
C 1.826853 -0.628479 1.164232
O 0.686852 -1.079912 1.213461
H 0.150717 -0.378349 -1.367053
H 2.179643 0.040547 1.977793
FREQS= 16 45 55 64 79 86 98 106 216 238 311 326 344 360 455 482 490 504 514 557 575
681 731 751 873 920 1021 1126 1162 1274 1333 1423 1433 1462 1513 1521 1746 1960 2074 2106 2160 2931 3010
3056 3115 3132 3143
TS016_8
18
E= -1916.41738475 ZPE= 77.50 Gcorr 0.057676000
H 1.367471 -0.770326 1.632207
Co -0.520794 0.029493 -0.066236
C -1.694802 -0.989148 0.766437
C -1.636197 1.465874 -0.124020
O -2.369316 -1.647078 1.426556
O -2.344851 2.338841 -0.336542
C 0.042194 -1.302667 -1.098550
O 0.288403 -2.086938 -1.901648
C 2.988140 0.121853 0.461079
C 2.227159 -0.102438 1.769434
H 3.859801 0.751862 0.672368
H 3.388576 -0.838006 0.112590
H 2.881562 -0.566838 2.514040
H 1.850089 0.836754 2.182282
C 2.161539 0.785194 -0.690173
O 0.845123 1.157809 -0.345291
H 2.159897 0.113909 -1.561842
H 2.670962 1.703244 -1.013110
FREQS= 40 44 61 86 97 102 120 252 262 289 312 377 406 429 452 479 512 540 578 594 649
753 840 923 1019 1073 1128 1175 1292 1314 1359 1394 1425 1500 1506 1514 1523 2098 2114 2179 2993 3029 3032
3041 3077 3099 3131
TS017_8
18
E= -1916.41160882 ZPE= 74.57 Gcorr 0.053304000
H 3.742870 0.916486 0.517477
Co -0.555609 -0.002465 -0.125598
C -0.167065 1.662768 -0.524927
C -1.985533 -0.738917 -0.902358
O 0.075487 2.727521 -0.890692
O -2.869488 -1.147994 -1.509319
C -1.210423 0.229322 1.551575
O -1.657462 0.390879 2.592905
C 2.575473 -0.580409 -0.561493
C 3.867508 -0.088690 0.100073
H 2.310122 0.079659 -1.392288
H 2.705181 -1.580325 -0.991146
H 4.180360 -0.752334 0.912925
H 4.682227 -0.045449 -0.628011
C 1.428932 -0.683412 0.437029
O 0.556350 -1.601681 0.369236
H -0.111356 -0.115061 -1.535450
H 1.599588 -0.190193 1.411210
FREQS= 48 57 67 79 88 111 159 205 259 316 330 369 403 424 462 472 494 508 523 536 552
740 750 806 870 931 1033 1103 1128 1276 1325 1387 1428 1481 1513 1516 1564 2004 2106 2134 2176 2968 3047
3064 3099 3120 3128
TS018_8
18
E= -1916.41152283 ZPE= 74.73 Gcorr 0.053457000
H -3.464811 0.507103 1.054591
Co 0.569027 0.075501 -0.174422
C 0.075989 1.733351 0.122135
C 0.314803 -0.622660 1.480000
O -0.188172 2.847096 0.246495
O 0.193464 -1.047482 2.536220
C 2.331601 -0.205638 -0.354250

O 3.461192 -0.283147 -0.536745
C -2.429282 -0.906125 -0.262299
C -3.283998 0.354654 -0.012451
H -2.135585 -1.396932 0.669975
H -3.016996 -1.654402 -0.814414
H -2.789882 1.256580 -0.387570
H -4.258299 0.291545 -0.503598
C -1.196527 -0.673296 -1.110348
O -0.224825 -1.495255 -1.123370
H 0.840377 0.726173 -1.479814
H -1.347332 -0.019996 -1.987301
FREQS= 44 58 69 84 84 112 120 185 270 326 349 370 406 426 458 476 496 512 514 530 551
744 795 822 925 954 1040 1092 1149 1250 1327 1375 1440 1483 1512 1521 1557 1998 2109 2135 2176 2987 3022
3059 3100 3123 3136
TS019_8
18
E= -1916.41031478 ZPE= 74.23 Gcorr 0.052737000
H 2.969861 0.323592 -1.414280
Co -0.510303 0.013941 -0.091186
C -0.800899 1.635628 -0.709010
C -0.501004 -1.526842 -0.954092
O -0.982451 2.643022 -1.239908
O -0.506770 -2.449738 -1.643338
C -2.073242 -0.178130 0.764958
O -3.090528 -0.303825 1.277925
C 2.748624 -0.390262 0.632191
C 3.534030 0.311817 -0.477016
H 2.480884 -1.417529 0.366567
H 3.371682 -0.459072 1.538282
H 3.762722 1.348855 -0.208495
H 4.482698 -0.199295 -0.660306
C 1.506972 0.335954 1.085167
O 0.582166 -0.231276 1.697352
H 1.551431 1.441764 1.067734
H 0.652673 0.190822 -1.016917
FREQS= 46 74 75 82 99 113 124 190 240 316 332 337 364 395 435 466 479 504 523 550 570
677 730 750 895 913 1039 1106 1157 1278 1337 1406 1431 1465 1512 1517 1658 1980 2087 2118 2169 2966 3012
3052 3100 3123 3134
TS020_8
18
E= -1916.40770989 ZPE= 74.21 Gcorr 0.051324000
H 3.778677 1.371630 -0.320060
Co -0.485469 0.034418 -0.012923
C -0.202409 -1.422628 -1.013288
C -2.024884 -0.468426 0.735941
O -0.082866 -2.284006 -1.767554
O -2.997902 -0.797904 1.243050
C -1.092384 1.540273 -0.734585
O -1.476038 2.465905 -1.302957
C 2.694837 -0.234428 0.683526
C 3.547356 0.310314 -0.462573
H 2.441315 -1.288947 0.536692
H 3.253461 -0.176241 1.628133
H 4.496289 -0.228293 -0.529620
H 3.036955 0.204672 -1.426015
C 1.412500 0.541029 0.944542
O 0.518231 0.047375 1.720564
H 0.872013 0.499336 -0.533437
H 1.527462 1.640787 0.927039
FREQS= 35 54 73 79 86 99 122 165 223 282 293 327 392 413 452 464 489 510 528 545 564
598 750 846 895 1033 1065 1140 1207 1286 1337 1366 1430 1478 1510 1514 1521 1971 2090 2119 2172 2962 3038
3046 3097 3117 3129
TS021_8
18
E= -1916.40740753 ZPE= 74.35 Gcorr 0.051588000
H 4.218596 1.277524 0.334167
Co -0.540509 0.029224 -0.059321
C -0.521354 -1.586709 -0.825524
C -1.726931 -0.321853 1.227274
O -0.606063 -2.572690 -1.413549
O -2.466635 -0.548442 2.072227
C -1.419641 1.393895 -0.782755
O -2.011278 2.210432 -1.339541
C 2.704267 -0.119690 -0.405677
C 3.964904 0.214437 0.407229
H 2.867992 0.099777 -1.466875
H 2.463125 -1.182790 -0.310524

H 3.816967 -0.028766 1.462712
H 4.819491 -0.358950 0.036957
C 1.532685 0.696376 0.118308
O 0.970605 0.360487 1.220557
H 0.540608 0.373981 -1.076486
H 1.570338 1.773134 -0.128976
FREQS= 44 54 65 77 89 94 122 178 230 245 290 315 396 425 453 460 502 505 539 549 580
598 801 827 891 1013 1064 1144 1191 1288 1340 1373 1419 1489 1511 1518 1528 1976 2091 2119 2172 2967 3050
3057 3107 3125 3140
TS022_8
18
E= -1916.41175286 ZPE= 76.54 Gcorr 0.057036000
H 3.326359 1.240057 -1.015012
Co -0.382584 0.117655 -0.270334
C 0.278337 1.141026 0.957190
C -1.882759 1.106271 -0.583359
O 0.699172 1.752863 1.835741
O -2.731176 1.821047 -0.868160
C -1.280634 -1.507599 0.396839
O -1.566091 -1.949709 1.459441
C 2.697336 -0.485833 0.164951
C 3.697810 0.245511 -0.740762
H 3.119356 -1.454974 0.455389
H 2.601353 0.077099 1.099527
H 4.662988 0.378630 -0.241450
H 3.873280 -0.309998 -1.668467
C 1.335655 -0.718836 -0.505816
O -1.249269 -1.596849 -0.836494
H 1.156250 -1.784022 -0.678798
H 1.294639 -0.225522 -1.510653
FREQS= 35 61 76 88 91 129 207 242 252 261 307 351 374 425 445 494 528 551 561 588 634
657 794 914 1004 1077 1095 1146 1202 1229 1309 1362 1421 1435 1509 1513 1526 2079 2111 2161 2815 3036 3051
3075 3096 3108 3115
TS023_8
18
E= -1916.39741934 ZPE= 70.78 Gcorr 0.042962000
H -4.447008 -0.000293 -1.048368
Co 0.643186 0.000012 0.234889
C 2.452676 -0.000003 0.174303
C 0.464713 -1.630320 -0.416859
O 3.593781 -0.000011 0.055062
O 0.357414 -2.629139 -0.983081
C 0.464710 1.630280 -0.417023
O 0.357389 2.629025 -0.983370
C -2.313433 -0.000223 -0.662971
C -3.767506 -0.000035 -0.191367
H -2.093650 -0.871253 -1.294603
H -2.093593 0.870399 -1.295145
H -3.982014 -0.881222 0.418711
H -3.981972 0.881555 0.418145
C -1.297497 0.000068 0.464603
O -1.620550 0.000330 1.634627
H 0.478129 0.000105 2.470436
H 1.227829 0.000135 2.498791
FREQS= 39 49 54 81 88 98 100 136 197 200 276 285 313 322 363 370 411 451 463 492 506
536 544 562 575 694 784 922 1014 1076 1102 1278 1358 1425 1457 1507 1513 1771 2074 2101 2161 3041 3061
3071 3135 3141 4314
TS024_8
18
E= -1916.39631388 ZPE= 73.29 Gcorr 0.041951000
H -4.009794 1.489933 0.128012
Co 0.912257 0.097394 -0.264379
C 0.977184 1.847990 -0.359753
C 1.713976 -0.149413 1.305231
O 1.126594 2.971854 -0.557783
O 2.281456 -0.295042 2.291276
C 1.442512 -1.352255 -1.099643
O 1.877293 -2.194964 -1.751637
C -3.517982 -0.639456 0.094166
C -4.056793 0.642252 -0.562077
H -4.064144 -0.896032 1.006615
H -3.622625 -1.478495 -0.609314
H -3.483074 0.902668 -1.457806
H -5.099581 0.509356 -0.861474
C -2.054200 -0.529476 0.436281
O -1.572580 -0.755569 1.528032
H 0.311047 0.324979 -1.623951

H -1.393064 -0.210144 -0.408167
FREQS= 10 23 24 48 64 84 91 96 97 215 293 303 327 336 450 479 487 505 505 574 587 633 760 765 879 928 1016 1139 1160 1277 1332 1426 1442 1476 1514 1519 1809 1951 2092 2122 2181 2802 3027 3052 3108 3127 3135
TS025_8
18
E= -1916.40670062 ZPE= 74.42 Gcorr 0.052353000
H 2.695414 1.153187 0.674897
Co -0.462429 -0.040056 0.044383
C -1.576251 -1.227843 0.761578
C -0.019540 1.436839 0.946456
O -2.298777 -1.927672 1.322749
O 0.161222 2.345198 1.630876
C -1.636973 0.702178 -1.074462
O -2.365322 1.178882 -1.819416
C 2.762498 -0.915954 -0.009638
C 3.182713 0.549003 -0.095303
H 3.370617 -1.516598 -0.702525
H 2.939702 -1.334391 0.987691
H 4.263186 0.643745 0.044054
H 2.920772 0.965194 -1.070768
C 1.328749 -1.212607 -0.436694
O 0.801404 -0.574508 -1.417200
H 0.624632 -0.725991 0.876757
H 1.035864 -2.268517 -0.296151
FREQS= 38 60 78 86 86 99 120 195 235 288 292 308 379 419 451 461 498 517 544 560 593 634 775 838 865 1022 1062 1113 1207 1295 1364 1371 1423 1480 1512 1519 1527 1951 2089 2117 2170 2971 3024 3064 3081 3132 3148
TS026_8
18
E= -1916.40994456 ZPE= 76.60 Gcorr 0.055843000
H 4.640329 -0.490187 0.030608
Co -0.576485 0.023972 -0.089267
C -2.191402 -0.389749 -0.752864
C -0.256895 -1.511206 0.722177
O -3.199531 -0.647551 -1.239465
O -0.113620 -2.417169 1.423418
C -1.043411 1.505688 0.748327
O -1.338926 2.371701 1.452183
C 2.476724 -0.322382 0.277823
C 3.819860 0.152869 -0.302038
H 2.288347 -1.357222 -0.029349
H 2.534743 -0.324140 1.373001
H 4.049385 1.177778 0.009398
H 3.819400 0.127624 -1.398862
C 1.310172 0.555126 -0.131134
O 0.951791 0.448188 -1.504925
H 1.488261 1.601586 0.141819
H 1.656635 0.633882 -2.139836
FREQS= 45 61 74 85 91 96 123 188 230 253 286 307 391 409 449 469 514 524 531 560 566 636 777 878 969 1030 1051 1117 1145 1199 1298 1350 1411 1422 1483 1513 1517 2067 2091 2154 3032 3047 3051 3085 3105 3120 3784
TS027_8
18
E= -1916.40843620 ZPE= 74.91 Gcorr 0.054706000
H -2.377330 -0.393019 1.432663
Co 0.553782 0.059568 -0.187036
C 0.176042 1.771731 -0.080805
C 0.212491 -0.451216 1.515973
O -0.001793 2.909488 -0.066458
O 0.087230 -0.774797 2.607646
C 2.276928 -0.409359 -0.254580
O 3.402681 -0.599428 -0.372900
C -2.635177 -0.588833 -0.732321
C -2.904620 0.098709 0.612854
H -2.947323 -1.638801 -0.666260
H -3.259274 -0.115354 -1.499478
H -2.608317 1.151226 0.592266
H -3.974176 0.057001 0.841064
C -1.214194 -0.616960 -1.285619
O -0.383065 -1.528181 -0.984887
H 0.980533 0.539974 -1.526060
H -1.075440 -0.090457 -2.244435
FREQS= 51 62 70 84 106 113 140 217 277 327 352 393 401 427 461 474 490 511 532 536 560 743 777 812 870 924 1049 1105 1123 1300 1357 1398 1424 1487 1514 1519 1565 1992 2105 2130 2172 2993 3040 3058 3086 3124 3151
TS028_8

18
E= -1916.39970779 ZPE= 72.02 Gcorr 0.046494000
H -3.982085 0.881192 0.144960
Co 0.677603 -0.000002 0.328086
C 2.460600 -0.000063 0.033314
C 0.458254 -1.644366 -0.289869
O 3.571835 -0.000102 -0.253773
O 0.316104 -2.676890 -0.782577
C 0.458362 1.644370 -0.289880
O 0.316287 2.676904 -0.782590
C -2.208529 0.000098 -0.754879
C -3.703971 -0.000052 -0.438725
H -1.925719 -0.870255 -1.360942
H -1.925865 0.870637 -1.360748
H -4.288293 0.000032 -1.363404
H -3.981960 -0.881499 0.144715
C -1.319884 0.000053 0.481877
O -1.780290 0.000050 1.601978
H 0.388995 0.000019 2.127933
H 1.159176 0.000004 2.093823
FREQS= 30 42 66 91 92 100 108 195 203 257 264 310 360 380 394 451 467 480 490 513 539
568 570 673 688 783 910 1014 1069 1076 1099 1282 1358 1424 1459 1506 1512 1779 2078 2102 2158 3046 3061
3076 3134 3141 3982
TS029_8
18
E= -1916.39816308 ZPE= 73.18 Gcorr 0.045062000
H 4.356850 0.464583 -0.637005
Co -0.742129 -0.049900 0.219188
C -0.683975 1.499898 1.063271
C -1.674162 0.441931 -1.213715
O -0.747505 2.430261 1.735556
O -2.318241 0.761649 -2.107756
C -1.428899 -1.639802 0.481693
O -1.952515 -2.612305 0.809982
C 2.824426 -0.970072 -0.077147
C 3.958654 -0.007311 0.264762
H 3.172675 -1.798556 -0.713094
H 2.413810 -1.455661 0.818526
H 3.607157 0.790451 0.924378
H 4.772711 -0.535931 0.767507
C 1.674630 -0.324508 -0.805418
O 1.635594 0.837718 -1.163334
H 0.820212 -1.007262 -1.054914
H -0.068618 -0.449722 1.500262
FREQS= 20 31 54 60 71 85 94 99 144 229 259 283 320 344 453 481 484 508 569 584 606
661 683 763 858 889 1006 1112 1131 1280 1373 1400 1430 1460 1508 1513 1791 1955 2092 2121 2177 2783 3021
3060 3064 3139 3142
TS030_8
18
E= -1916.39861454 ZPE= 72.04 Gcorr 0.046470000
H -3.990561 -0.878825 0.179864
Co 0.678911 0.000045 0.315294
C 2.467851 0.000986 0.057691
C 0.462589 -1.639259 -0.316083
O 3.589025 0.001599 -0.186435
O 0.322519 -2.666427 -0.820533
C 0.460880 1.639187 -0.315984
O 0.319617 2.666198 -0.820414
C -2.223753 -0.001633 -0.736938
C -3.716456 0.000852 -0.408040
H -1.947134 -0.873683 -1.343470
H -1.944773 0.867299 -1.346779
H -3.988570 0.883918 0.175720
H -4.308500 -0.000589 -1.327799
C -1.324287 -0.000833 0.491586
O -1.775511 -0.000733 1.616710
H 0.343575 -0.000200 2.027509
H 1.099240 -0.000013 2.173999
FREQS= 30 44 65 90 92 100 107 194 201 254 260 312 359 383 393 450 468 474 485 534 540
570 572 636 685 783 910 1014 1076 1098 1116 1281 1357 1423 1458 1505 1512 1772 2078 2103 2159 3046 3061
3076 3134 3141 3989
TS031_8
18
E= -1916.39487309 ZPE= 70.76 Gcorr 0.043104000
H -3.511280 -0.202448 -1.835574
Co 0.549977 -0.007548 0.274058
C 2.333536 0.016584 -0.039937

C 0.284324 -1.641279 -0.335997
O 3.451813 0.044498 -0.294164
O 0.090125 -2.644422 -0.871439
C 0.246372 1.593799 -0.405109
O 0.062854 2.574559 -0.983160
C -2.551466 -0.328264 0.099381
C -2.578661 0.119108 -1.363713
H -3.408942 0.064413 0.657029
H -2.612008 -1.425122 0.150565
H -2.517258 1.207704 -1.450579
H -1.751022 -0.312164 -1.933389
C -1.301577 0.045528 0.881944
O -1.350419 0.364648 2.050075
H 1.346984 -0.650123 2.299260
H 0.973990 -0.025596 2.483206
FREQS= 29 53 76 82 92 100 104 127 175 202 225 305 327 338 365 401 421 452 470 488 504 538 551 555 561 609 788 916 1042 1074 1132 1278 1335 1428 1466 1513 1516 1776 2073 2101 2160 3028 3059 3090 3131 3135 4310
TS032_8
18
E= -1916.39812836 ZPE= 73.32 Gcorr 0.046405000
H 2.342712 1.331029 1.279812
Co -0.634896 0.159717 0.190268
C -1.057828 1.846475 -0.053641
C -1.593567 -0.577793 -1.115064
O -1.388777 2.949322 -0.073855
O -2.281148 -1.032791 -1.913047
C -0.878859 -1.063697 1.431731
O -1.103592 -1.756659 2.322299
C 3.063353 -0.096698 -0.202551
C 2.957962 1.313216 0.375990
H 3.724811 -0.103810 -1.080739
H 3.508368 -0.801571 0.509296
H 3.949507 1.692725 0.636416
H 2.516123 2.009251 -0.344717
C 1.760350 -0.705544 -0.676399
O 1.614485 -1.859106 -0.993775
H -0.036475 0.814971 1.396210
H 0.900940 0.043182 -0.826877
FREQS= 11 44 48 72 76 93 107 114 202 283 322 327 349 366 450 478 488 507 535 566 581 628 710 759 899 951 1046 1118 1172 1284 1318 1361 1433 1455 1512 1513 1835 1973 2095 2120 2174 2476 3035 3055 3086 3125 3136
TS033_8
18
E= -1916.39759653 ZPE= 73.36 Gcorr 0.047320000
H -2.308347 -2.545724 -0.900227
Co 0.725987 -0.235483 -0.088993
C 1.404481 0.102050 -1.671071
C 0.821934 1.403241 0.613969
O 1.984150 0.220765 -2.659847
O 1.027157 2.437627 1.063226
C 1.248196 -1.167583 1.300882
O 1.723651 -1.821787 2.121453
C -2.019922 -0.445003 -0.436729
C -2.873685 -1.714667 -0.472425
H -1.135064 -0.601177 0.218005
H -1.678737 -0.198470 -1.450818
H -3.197298 -2.007318 0.531002
H -3.768019 -1.566867 -1.086695
C -2.735256 0.782078 0.085209
O -2.182635 1.704183 0.642665
H 0.826264 -1.613057 -0.673267
H -3.833530 0.803654 -0.094178
FREQS= 28 48 55 75 78 94 96 106 164 222 304 325 328 344 455 487 490 512 527 560 583 615 746 760 887 917 1033 1125 1146 1246 1305 1414 1432 1482 1511 1517 1824 1963 2088 2120 2176 2851 2905 3055 3057 3124 3142
TS034_8
18
E= -1916.39437316 ZPE= 71.65 Gcorr 0.045605000
H 4.453035 -0.963293 -1.148679
Co -0.594301 -0.078268 -0.233591
C -2.298556 -0.516821 -0.704600
C -0.388063 -1.157855 1.186909
O -3.325729 -0.835649 -1.095627
O -0.240625 -1.877981 2.067966
C -0.933387 1.624838 0.320376
O -1.147727 2.707010 0.618788

C 2.338261 -0.624751 -0.826747
C 3.807787 -0.284901 -0.582540
H 2.082066 -0.561109 -1.892033
H 2.108066 -1.657555 -0.536678
H 4.029178 0.740304 -0.889094
H 4.062048 -0.372581 0.476941
C 1.367817 0.293561 -0.080768
O 1.769722 1.217877 0.582907
H -0.295517 0.232145 -1.652900
H -0.201027 -0.999149 -1.318666
FREQS= 20 52 60 80 85 97 100 206 219 252 267 333 346 389 425 454 466 488 504 526 532 602 680 775 792 889 912 1013 1075 1100 1278 1344 1424 1460 1505 1512 1793 2051 2072 2137 2156 2191 3045 3060 3078 3132 3142
TS035_8
18
E= -1916.40613073 ZPE= 76.64 Gcorr 0.057371000
H 4.018573 -0.624242 -0.698871
Co -0.651753 0.039068 -0.116342
C -0.779162 -1.478746 0.767993
C -0.446334 1.687343 0.489696
O -0.854472 -2.366630 1.500970
O -0.393821 2.674189 1.086331
C -2.360888 0.175148 -0.665933
O -3.399950 0.223527 -1.154381
C 2.393921 0.449441 0.298066
C 3.723319 0.403597 -0.471293
H 2.515386 -0.043022 1.274352
H 2.129146 1.487094 0.521516
H 3.642550 0.940299 -1.422660
H 4.525155 0.868877 0.110199
C 1.242845 -0.217157 -0.439125
O 1.543221 -1.544878 -0.815077
H 1.011147 0.298365 -1.404021
H 1.953344 -1.989618 -0.058447
FREQS= 47 71 81 88 99 113 198 221 258 285 303 329 384 396 434 480 499 508 532 563 568 612 781 899 961 990 1085 1118 1136 1237 1329 1350 1390 1422 1491 1509 1519 2066 2089 2151 2825 3020 3049 3098 3118 3132 3782
TS036_8
18
E= -1916.38925960 ZPE= 70.10 Gcorr 0.040668000
H 4.288623 -0.021424 1.618766
Co -0.588614 -0.004368 0.141157
C -2.333120 -0.072028 0.557672
C -0.606171 1.660957 -0.455941
O -3.465231 -0.121930 0.741398
O -0.676939 2.686081 -0.973752
C -0.492259 -1.672769 -0.440576
O -0.497441 -2.702558 -0.954070
C 2.211263 0.044172 1.001986
C 3.706815 -0.041067 0.692517
H 1.889322 -0.788433 1.640197
H 1.975693 0.957457 1.563678
H 3.942768 -0.962513 0.154355
H 4.021599 0.795064 0.064067
C 1.340109 0.045160 -0.270022
O 1.835139 0.072615 -1.369367
H -0.265179 0.203450 2.810252
H -0.484300 0.674127 2.269951
FREQS= 32 41 67 81 91 96 106 119 175 200 225 266 282 307 316 327 368 390 461 465 480 513 542 566 578 682 791 870 991 1066 1095 1280 1337 1421 1466 1504 1511 1790 2088 2107 2169 3039 3061 3074 3135 3147 4318
TS037_8
18
E= -1916.40182587 ZPE= 75.15 Gcorr 0.053317000
H -4.313971 0.303087 -0.161968
Co 0.697468 0.158623 0.099185
C 0.803724 1.448380 -1.119905
C 0.214877 0.722187 1.710817
O 0.942364 2.301077 -1.882100
O -0.036748 1.112577 2.765430
C 1.761666 -1.256158 -0.044291
O 2.540010 -2.107246 -0.112871
C -2.208255 0.214133 -0.675384
C -3.441836 -0.309191 0.082120
H -2.386043 0.146115 -1.759742
H -2.031595 1.266032 -0.446644
H -3.661037 -1.345373 -0.182805

H -3.287026 -0.256096 1.164142
C -0.942722 -0.561281 -0.410370
O -1.185787 -1.847771 -0.598167
H 2.019591 0.742542 0.510374
H -0.374994 -2.356643 -0.437621
FREQS= 19 54 63 83 88 89 211 241 292 316 331 352 406 440 458 479 514 527 544 574 662
695 721 737 800 921 1037 1059 1088 1256 1307 1369 1400 1424 1481 1512 1520 1946 2071 2091 2144 3022 3060
3129 3133 3154 3753
TS038_8
18
E= -1916.40182183 ZPE= 75.20 Gcorr 0.053661000
H -4.335547 0.477579 0.217559
Co 0.696380 0.142261 -0.101307
C 0.439676 1.898982 0.082927
C 1.699705 -0.682048 1.107389
O 0.360476 3.044912 0.173180
O 2.423514 -1.151022 1.873606
C 0.711670 -0.494518 -1.755030
O 0.780772 -0.858560 -2.847025
C -2.233671 0.321638 0.731817
C -3.473260 -0.133753 -0.061233
H -2.044784 1.383006 0.570781
H -2.419313 0.185940 1.808424
H -3.312402 -0.017245 -1.137354
H -3.710709 -1.180274 0.139328
C -0.988087 -0.465501 0.418617
O -1.253937 -1.754601 0.567314
H 2.046100 0.655441 -0.513791
H -0.448425 -2.260114 0.366813
FREQS= 18 59 62 91 92 96 211 247 292 315 336 341 401 447 458 487 516 522 544 574 661
697 720 762 798 921 1037 1056 1088 1254 1304 1363 1398 1424 1482 1511 1520 1946 2082 2086 2143 3022 3061
3131 3139 3157 3733
TS039_8
18
E= -1916.40010806 ZPE= 73.32 Gcorr 0.051983000
H -3.580763 0.041574 -1.883895
Co 0.600136 -0.012951 0.338358
C 2.355735 0.231505 -0.037674
C 0.545842 -1.634813 -0.386430
O 3.456158 0.395202 -0.314082
O 0.529092 -2.672229 -0.887077
C 0.108089 1.577895 -0.279316
O -0.162921 2.612999 -0.705752
C -2.383520 -0.691877 -0.236354
C -2.784101 0.408898 -1.230786
H -3.259043 -1.014491 0.337387
H -1.990969 -1.553992 -0.783563
H -3.156650 1.295398 -0.709490
H -1.946864 0.709846 -1.865442
C -1.352036 -0.224632 0.792406
O -1.688101 0.035620 1.920941
H 0.809428 0.347387 1.981449
H 0.907304 -0.450626 1.944581
FREQS= 38 65 86 89 108 116 206 232 266 292 317 358 373 437 444 463 502 518 543 548 566
568 615 659 803 867 902 1022 1072 1130 1283 1329 1415 1422 1487 1515 1517 1802 2088 2109 2163 3058 3060
3113 3131 3142 3447
TS040_8
18
E= -1916.40178778 ZPE= 75.25 Gcorr 0.054369000
H -4.161462 0.201354 -0.194891
Co 0.645324 0.193554 0.137321
C 0.534315 1.745396 -0.728853
C 0.084887 0.229644 1.816985
O 0.527190 2.772535 -1.250856
O -0.237282 0.290377 2.922271
C 1.938499 -0.945482 -0.294657
O 2.856836 -1.600140 -0.542769
C -2.153827 -0.019220 -0.993665
C -3.218937 -0.268690 0.097672
H -2.020683 1.053047 -1.142502
H -2.494183 -0.463567 -1.937757
H -3.394841 -1.338442 0.235063
H -2.909841 0.158343 1.055747
C -0.840505 -0.648388 -0.611363
O -0.927652 -1.950792 -0.830773
H 1.842078 0.887312 0.722318
H -0.104153 -2.379388 -0.545358

FREQS=   32   55   62   86   91   93 209 237 290 315 335 349 402 447 461 479 519 525 543 589 654
693 721 744 814 921 1015 1082 1090 1253 1315 1364 1380 1422 1495 1510 1522 1946 2074 2087 2142 3049 3061
3132 3138 3151 3744
TS041_8
18
E= -1916.39442057 ZPE=   72.08 Gcorr   0.047036000
H -4.019219 -0.881053 -0.184597
Co 0.623344 -0.000011 0.305505
C 2.416977 -0.000120 0.403256
C 0.544033 -1.654231 -0.338169
O 3.564561 -0.000187 0.359577
O 0.525723 -2.696889 -0.825432
C 0.544232 1.654269 -0.338062
O 0.526042 2.696961 -0.825254
C -2.366530 -0.000054 0.917303
C -3.814848 -0.000023 0.428452
H -2.158037 0.875629 1.547054
H -2.158008 -0.875869 1.546861
H -4.503830 -0.000146 1.278175
H -4.019259 0.881152 -0.184376
C -1.336651 0.000090 -0.222518
O -1.685519 0.000245 -1.375309
H -0.057026 -0.000027 1.921618
H 0.715366 -0.000002 2.056416
FREQS=   31   40   76   94   96 101 114 206 208 256 264 329 357 368 406 459 466 474 508 529 532
564 567 678 780 791 890 1003 1068 1098 1171 1283 1339 1423 1465 1506 1512 1797 2091 2107 2163 3028 3062
3064 3136 3146 3774
TS042_8
18
E= -1916.40440835 ZPE=   76.61 Gcorr   0.057227000
H -4.505050 0.742206 0.349640
Co 0.662518 0.039944 -0.114493
C 0.486978 1.734019 0.371687
C 0.631392 -1.456242 0.819470
O 0.437564 2.757342 0.901626
O 0.631126 -2.307237 1.596245
C 2.410198 0.062016 -0.542812
O 3.470459 0.028056 -0.986429
C -2.355863 0.387904 0.355869
C -3.731572 0.361601 -0.324953
H -2.112138 1.408466 0.668026
H -2.388809 -0.216853 1.270414
H -3.738487 0.985689 -1.226138
H -4.000624 -0.655868 -0.617681
C -1.236386 -0.137101 -0.524557
O -1.568970 -1.457015 -0.909698
H -1.015309 -1.719234 -1.655867
H -1.117488 0.494763 -1.439248
FREQS=   47   70   82   89   99 121 193 226 239 282 292 333 352 392 433 476 492 498 533 560 566
608 779 901 956 991 1065 1117 1165 1240 1325 1345 1396 1419 1486 1509 1522 2071 2089 2152 2807 3044 3052
3095 3111 3142 3826
TS043_8
18
E= -1916.40342223 ZPE=   76.91 Gcorr   0.056318000
H -3.394346 1.818138 0.319697
Co 0.594348 0.047429 -0.051571
C 2.017800 -0.280432 0.964076
C 0.549578 -1.508665 -0.872150
O 2.901702 -0.495567 1.666457
O 0.609630 -2.427881 -1.563092
C 0.955121 1.744318 -0.401815
O 1.243741 2.787408 -0.792070
C -1.239397 0.376561 -0.763840
C -3.437321 0.733872 0.453900
H -1.592494 -0.306549 -1.538963
H -1.393652 1.395281 -1.126767
H -3.980985 0.296190 -0.388687
H -3.985444 0.512081 1.374019
C -2.031762 0.134336 0.530154
O -2.218412 -1.258112 0.803791
H -1.520076 0.614469 1.389640
H -1.357789 -1.656910 0.980846
FREQS=   34   44   69   94   98 117 142 163 239 300 309 379 404 433 464 480 491 495 535 577 583
626 815 874 954 1002 1030 1115 1172 1198 1277 1368 1395 1418 1488 1497 1510 2084 2102 2165 2884 3053 3082
3133 3135 3140 3828
TS044_8
18

E= -1916.40178677 ZPE=   76.11 Gcorr   0.054767000
H -4.484033 0.907442 0.291469
Co 0.661680 0.040493 -0.131903
C 0.467906 1.723687 0.378230
C 0.697111 -1.517293 0.706164
O 0.402101 2.739689 0.919587
O 0.733781 -2.413798 1.425394
C 2.412207 0.125411 -0.517872
O 3.483775 0.142108 -0.932567
C -2.346945 0.482961 0.322207
C -3.743951 0.373218 -0.311848
H -2.111170 1.541709 0.470436
H -2.367295 0.025472 1.318971
H -3.761428 0.803724 -1.319627
H -4.090891 -0.665194 -0.379371
C -1.235952 -0.167179 -0.496240
O -1.497524 -1.539260 -0.763703
H -2.424380 -1.638336 -1.026587
H -1.105492 0.377136 -1.467446
FREQS=   39   50   73   85   94 105 127 200 233 272 297 320 340 393 437 476 489 504 529 563 576
596 773 891 940 989 1073 1118 1172 1270 1289 1344 1375 1421 1486 1512 1524 2077 2100 2161 2774 3033 3050
3086 3107 3118 3765
TS045_8
18
E= -1916.39986575 ZPE=   75.08 Gcorr   0.053748000
H -3.967867 -1.270709 -0.295106
Co 0.483053 -0.125342 -0.164772
C 1.730605 0.679570 -1.114964
C 0.304072 1.181772 0.972171
O 2.700743 1.341886 -1.251578
O 0.185369 1.989418 1.784923
C 1.510158 -1.414448 0.581200
O 2.127072 -2.156792 1.199466
C -2.569284 0.287206 0.317332
C -3.955407 -0.185873 -0.157279
H -2.583833 1.369810 0.485334
H -2.314697 -0.198446 1.264989
H -4.721514 0.076209 0.578411
H -4.231426 0.281036 -1.108788
C -1.519007 -0.058681 -0.717012
O -0.982826 -1.232207 -0.749305
H 1.124991 0.152972 -1.965186
H -1.597772 0.497661 -1.667560
FREQS=   30   71   77   84   95 124 137 185 231 245 274 373 414 432 447 469 495 510 534 545 593
669 798 886 962 1005 1091 1121 1156 1291 1335 1359 1418 1486 1511 1515 1525 1939 2105 2157 2385 2978 3049
3056 3102 3122 3135
TS046_8
18
E= -1916.40229386 ZPE=   76.49 Gcorr   0.056518000
H -2.839425 -1.573102 -1.061694
Co 0.532176 -0.051857 -0.121055
C 2.192063 -0.535992 -0.617523
C 0.961733 1.349913 0.855772
O 3.218627 -0.781181 -1.072879
O 1.209169 2.159523 1.639568
C 0.001492 -1.636957 0.458699
O -0.161200 -2.609030 1.061615
C -2.596076 0.369265 -0.100241
C -3.032707 -1.096558 -0.094444
H -3.276712 0.936306 -0.752295
H -2.718654 0.793601 0.907119
H -2.531020 -1.681001 0.677515
H -4.108641 -1.160898 0.093495
C -1.188895 0.684914 -0.588141
O -1.073472 2.052144 -0.916318
H -0.927182 0.136753 -1.534601
H -1.537777 2.569322 -0.241683
FREQS=   40   52   76   87   95 117 188 229 249 287 325 337 386 407 436 478 491 510 521 550 564
579 780 902 939 1053 1099 1111 1159 1220 1309 1368 1379 1432 1495 1512 1524 2061 2087 2147 2762 3006 3039
3056 3117 3161 3784
TS047_8
18
E= -1916.39966466 ZPE=   76.49 Gcorr   0.053962000
H -2.190860 0.894676 1.255990
Co 0.664226 0.035919 -0.087336
C 0.315010 -1.494851 0.739083
C 2.417544 -0.213827 -0.365545

O 0.147113 -2.367024 1.472035
O 3.500242 -0.390936 -0.706829
C 0.730011 1.742392 0.354130
O 0.763177 2.797552 0.814102
C -3.753122 0.278075 -0.114986
C -2.352859 0.121038 0.494389
H -3.932831 -0.490113 -0.871819
H -4.526826 0.189234 0.654496
H -2.284149 -0.844934 1.007641
H -3.868179 1.256711 -0.594666
C -1.245958 0.230933 -0.542078
O -1.398568 -0.780979 -1.544346
H -1.331796 1.205987 -1.050104
H -0.558917 -1.232847 -1.713110
FREQS= 22 41 75 85 89 97 126 169 215 232 282 315 384 393 449 473 489 492 539 565 573
648 784 895 961 1032 1077 1110 1183 1251 1305 1354 1397 1419 1487 1509 1521 2079 2097 2160 2964 3040 3044
3082 3111 3133 3752
TS048_8
18
E= -1916.40138108 ZPE= 76.30 Gcorr 0.055852000
H 2.957260 1.746338 0.987464
Co -0.671808 0.023841 -0.177835
C -2.258492 -0.784849 -0.316002
C 0.110912 -1.295608 0.695181
O -3.262413 -1.297087 -0.537142
O 0.486859 -2.043819 1.486861
C -1.290797 1.617265 0.273119
O -1.640496 2.611550 0.734162
C 0.969108 0.887195 -0.784468
C 2.966741 0.671643 0.776091
H 0.982437 1.975261 -0.692975
H 0.592184 0.647899 -1.810872
H 2.386496 0.159619 1.549584
H 3.999143 0.316603 0.833688
C 2.395415 0.380328 -0.613955
O 2.561499 -1.002494 -0.926206
H 3.009288 0.927110 -1.349037
H 1.691078 -1.417594 -0.957508
FREQS= 37 51 81 91 100 120 143 171 240 312 324 358 387 423 429 479 497 509 519 568 580
601 717 839 966 977 1024 1160 1175 1201 1285 1370 1392 1419 1427 1499 1510 2081 2103 2163 2828 2975 3048
3111 3123 3135 3822
TS049_8
18
E= -1916.39168764 ZPE= 72.16 Gcorr 0.046391000
H 3.912216 -0.494257 -1.732315
Co -0.509996 -0.036566 -0.269349
C -2.242916 -0.448545 -0.499573
C -0.148309 -1.554065 0.584694
O -3.363025 -0.698822 -0.540543
O 0.039124 -2.505439 1.204227
C -0.852783 1.650976 0.164306
O -1.114131 2.716327 0.514039
C 2.458462 0.677092 -0.620625
C 3.090258 -0.662809 -1.031047
H 3.209226 1.313023 -0.139256
H 2.072638 1.204715 -1.500036
H 3.489545 -1.183379 -0.156117
H 2.367092 -1.325236 -1.517889
C 1.334122 0.478133 0.408384
O 1.561885 0.607482 1.584223
H 0.314113 -0.062412 -1.811515
H -0.418782 -0.266239 -2.002838
FREQS= 21 41 54 91 99 105 108 190 217 261 285 310 354 372 411 466 474 500 508 529 544
556 571 652 784 808 889 1005 1064 1123 1177 1273 1315 1420 1491 1509 1517 1793 2091 2107 2162 3049 3052
3100 3124 3136 3768
TS050_8
18
E= -1916.39809950 ZPE= 74.77 Gcorr 0.052990000
H 3.503104 1.325207 -0.639495
Co -0.399916 0.141906 0.063310
C -1.123898 -1.414397 -0.345309
C -0.064983 -0.121771 1.793561
O -1.592852 -2.438140 -0.598798
O 0.085422 -0.252198 2.928830
C -1.743717 1.181638 -0.444064
O -2.621504 1.878516 -0.719830
C 1.411107 0.913386 -0.842709

C 2.600296 1.367490 -0.019427
H 0.285965 1.445974 0.268909
H 1.090260 1.600904 -1.620457
H 2.474614 2.393758 0.334462
H 2.759502 0.716080 0.841112
C 1.248430 -0.445686 -1.155571
O 2.139287 -1.342701 -0.604966
H 0.833764 -0.721939 -2.122249
H 1.804296 -2.239219 -0.732407
FREQS= 35 55 68 83 93 98 189 218 240 271 309 327 348 405 434 482 485 506 533 563 574 588 636 784 905 924 1025 1042 1097 1159 1258 1268 1398 1435 1495 1503 1557 2060 2080 2117 2140 3053 3122 3155 3162 3186 3833
TS051_8
18
E= -1916.39788287 ZPE= 74.64 Gcorr 0.052934000
H -1.970875 2.467713 0.921195
Co 0.322724 0.084870 0.130145
C 1.066405 1.692257 0.074212
C 0.963287 -1.127595 1.272922
O 1.575897 2.727515 0.090864
O 1.431537 -1.853880 2.034623
C 0.973116 -0.520201 -1.395838
O 1.409948 -0.927092 -2.383469
C -1.787355 0.347320 0.477804
C -2.312121 1.736695 0.183575
H -0.355860 0.636972 1.332776
H -2.143526 -0.099086 1.405492
H -1.995226 2.083444 -0.803791
H -3.408441 1.725204 0.205143
C -1.581126 -0.581113 -0.551108
O -1.868921 -1.905506 -0.291186
H -1.454441 -2.458029 -0.966466
H -1.706097 -0.260183 -1.584313
FREQS= 32 58 70 85 90 104 216 223 230 304 314 338 368 373 430 475 481 502 515 534 561 575 585 767 905 940 1039 1097 1141 1167 1219 1306 1354 1435 1497 1507 1572 2055 2082 2110 2142 3043 3112 3132 3147 3155 3825
TS052_8
18
E= -1916.39169742 ZPE= 71.95 Gcorr 0.046925000
H 3.875185 -1.457716 -1.045395
Co -0.496430 -0.085837 -0.228076
C -2.057380 -0.962929 -0.559522
C 0.006630 -0.945925 1.268559
O -2.991593 -1.550687 -0.861999
O 0.337114 -1.522330 2.203609
C -1.215386 1.532391 0.196781
O -1.671809 2.557605 0.414140
C 2.446836 0.173273 -1.055483
C 3.036735 -1.104160 -0.438571
H 3.218265 0.947878 -1.122376
H 2.068848 -0.036235 -2.060702
H 3.408651 -0.915360 0.572940
H 2.297942 -1.909147 -0.388825
C 1.317585 0.767742 -0.207138
O 1.525387 1.736079 0.482887
H -0.344066 0.145373 -1.684856
H 0.075876 -0.984895 -1.249592
FREQS= 34 43 64 81 89 95 105 204 225 255 291 333 355 393 430 459 478 495 521 526 563 573 663 789 809 893 907 1019 1072 1130 1279 1324 1419 1487 1513 1518 1788 2052 2080 2138 2157 2190 3054 3058 3111 3129 3134
TS053_8
18
E= -1916.39339526 ZPE= 72.39 Gcorr 0.048771000
H 3.935987 0.590333 -0.099658
Co -0.588434 -0.098228 -0.171524
C -2.211406 -0.602575 -0.817003
C -0.477384 -0.976152 1.390163
O -3.139299 -0.992501 -1.361367
O -0.369689 -1.609082 2.339086
C -0.960819 1.633070 0.256842
O -1.198102 2.733225 0.444661
C 2.329650 -0.725049 -0.743655
C 3.819317 -0.354803 -0.634196
H 2.001684 -0.831395 -1.783915
H 2.110566 -1.696650 -0.284938
H 4.382870 -1.120356 -0.094503
H 4.275142 -0.242227 -1.621350

C 1.370033 0.282894 -0.104993
O 1.760264 1.293609 0.427324
H -0.266744 0.362768 -1.540890
H -0.193515 -1.356634 -0.824165
FREQS= 36 57 71 81 88 99 104 226 266 273 338 354 398 419 437 449 483 496 529 540 645 657 692 714 829 882 905 1043 1081 1083 1248 1329 1421 1463 1513 1524 1791 2023 2060 2144 2162 2200 3054 3066 3087 3133 3147
TS054_8
18
E= -1916.40133770 ZPE= 76.36 Gcorr 0.056790000
H -3.814311 -0.303126 0.803967
Co 0.627884 0.026176 -0.123775
C 0.441782 1.561168 0.721490
C 0.898592 -1.491855 0.749177
O 0.350798 2.480249 1.416954
O 1.075304 -2.393586 1.445284
C 2.282259 0.265124 -0.781646
O 3.307013 0.410128 -1.283799
C -2.323114 0.777792 -0.395801
C -3.737968 0.238479 -0.147093
H -2.323072 1.354237 -1.328844
H -2.079056 1.492972 0.392570
H -4.443044 1.071667 -0.087578
H -4.086404 -0.414756 -0.955282
C -1.198430 -0.275033 -0.487154
O -1.646682 -1.575866 -0.548231
H -2.564146 -1.626761 -0.246687
H -0.513038 -0.102414 -1.431720
FREQS= 42 57 78 88 95 102 222 249 276 306 318 345 368 405 449 497 514 518 546 562 572 615 781 877 1023 1057 1079 1107 1204 1294 1312 1340 1376 1423 1488 1522 1526 2066 2090 2146 2251 3034 3055 3096 3121 3128 3797
TS055_8
18
E= -1916.39243656 ZPE= 72.12 Gcorr 0.048023000
H -4.494925 1.172240 -0.696662
Co 0.593712 0.137636 -0.114562
C 2.074127 0.660261 -0.998110
C 0.517281 0.878006 1.502822
O 2.941858 1.087831 -1.613872
O 0.434836 1.437489 2.502357
C 1.079405 -1.570284 0.277541
O 1.407465 -2.640326 0.504289
C -2.371909 0.783153 -0.530242
C -3.828993 0.322242 -0.521513
H -2.100166 1.258665 -1.480445
H -2.193385 1.547170 0.237140
H -4.011202 -0.423911 -1.299460
H -4.092525 -0.131239 0.436953
C -1.381064 -0.350773 -0.284669
O -1.739444 -1.472244 -0.012047
H -0.233250 -0.278324 -1.301680
H 0.204422 1.501586 -0.523460
FREQS= 48 59 69 73 87 93 100 213 217 271 306 331 372 412 437 457 490 499 533 541 578 689 703 798 824 865 932 1018 1081 1100 1277 1351 1426 1462 1507 1513 1782 1959 2042 2125 2150 2192 3046 3061 3081 3133 3143
TS056_8
18
E= -1916.40005302 ZPE= 76.59 Gcorr 0.055917000
H -4.095767 -0.340260 0.924728
Co 0.549163 0.006813 -0.091393
C 0.799142 1.581116 0.665797
C -0.094305 -1.493712 0.588115
O 0.924458 2.529418 1.308514
O -0.467717 -2.387091 1.213213
C 2.264074 -0.483021 -0.199092
O 3.359675 -0.790874 -0.359939
C -2.510298 -0.147917 -0.551352
C -3.117746 0.138824 0.824415
H -2.372841 -1.225367 -0.697446
H -3.210425 0.180089 -1.333785
H -2.479065 -0.233154 1.631567
H -3.258018 1.214501 0.978914
C -1.200086 0.581766 -0.781017
O -0.706569 0.334494 -2.101302
H 0.217436 0.009782 -2.134844
H -1.352189 1.660537 -0.666599

FREQS= 38 52 78 90 101 104 130 184 217 238 312 348 396 401 457 482 505 506 518 555 567
589 817 894 1005 1055 1078 1095 1180 1250 1300 1338 1409 1429 1502 1513 1521 2077 2096 2158 3017 3047 3051
3077 3116 3126 3559
TS057_8
18
E= -1916.39389476 ZPE= 72.66 Gcorr 0.050255000
H -4.248384 -1.199340 -0.796785
Co 0.568547 -0.099681 -0.185448
C 2.179813 -0.819219 -0.626144
C 1.129560 1.542825 0.375110
O 3.111134 -1.325627 -1.057080
O 1.499447 2.586357 0.650859
C 0.100198 -0.994432 1.296663
O -0.235364 -1.634546 2.186206
C -2.347388 -0.209219 -1.160805
C -3.470523 -0.683805 -0.226370
H -1.890686 -1.047251 -1.687937
H -2.754026 0.484913 -1.907539
H -3.092339 -1.381389 0.528555
H -3.922285 0.166317 0.290271
C -1.294254 0.581094 -0.378787
O -1.602291 1.625211 0.145818
H 0.102694 -1.274722 -0.935206
H 0.486414 0.428243 -1.568694
FREQS= 29 60 81 90 93 102 217 241 269 290 303 344 397 418 441 476 486 527 532 558 625
658 686 691 801 885 918 1021 1069 1113 1268 1329 1416 1489 1510 1520 1783 2014 2071 2141 2161 2199 3049
3053 3120 3133 3150
TS058_8
18
E= -1916.39464805 ZPE= 74.79 Gcorr 0.051622000
H -3.760001 0.652115 1.143121
Co 0.485698 -0.087321 0.066887
C 0.626544 -1.548114 -0.979920
C 1.274360 1.277385 -0.756377
O 0.683901 -2.489705 -1.638850
O 1.784419 2.158548 -1.300316
C 1.688332 -0.276513 1.366800
O 2.396353 -0.428842 2.262542
C -2.487039 -0.365426 -0.313906
C -3.415478 -0.359460 0.916483
H -2.183733 -1.385362 -0.553358
H -3.027455 0.042242 -1.179113
H -4.289032 -0.989081 0.726878
H -2.897107 -0.754238 1.795592
C -1.259421 0.486284 -0.107158
O -1.652245 1.756020 -0.038870
H -0.881129 2.318018 0.132603
H -0.338610 -0.779130 1.096739
FREQS= 25 48 63 71 88 93 102 217 248 294 309 347 389 430 462 482 499 527 557 573 576
698 711 756 811 926 1032 1071 1086 1252 1310 1349 1382 1420 1492 1510 1522 1976 2087 2094 2144 3042 3058
3129 3134 3151 3773
TS059_8
18
E= -1916.39431784 ZPE= 74.77 Gcorr 0.051390000
H -4.018293 1.253413 -0.358081
Co 0.477993 -0.000826 -0.114632
C 0.121040 1.191480 1.170984
C 1.473020 -1.361906 0.498339
O -0.094836 1.949110 2.013260
O 2.113433 -2.199917 0.964619
C 1.691179 0.988318 -0.959409
O 2.434869 1.604555 -1.585416
C -2.510404 -0.149744 0.325207
C -3.070235 0.843152 -0.716343
H -2.343227 0.373588 1.270036
H -3.237477 -0.952387 0.494002
H -3.251942 0.343847 -1.672176
H -2.375283 1.668993 -0.885563
C -1.212137 -0.751363 -0.145488
O -1.469941 -1.942240 -0.668233
H 0.141082 -0.209449 -1.559024
H -0.643637 -2.307409 -1.027702
FREQS= 20 28 65 80 96 107 137 204 216 301 321 358 398 436 458 495 497 521 553 569 573
679 713 767 820 924 1020 1078 1109 1252 1324 1365 1374 1421 1500 1507 1522 1933 2079 2095 2142 3057 3059
3119 3137 3147 3728
TS060_8
18

E= -1916.39743790 ZPE= 75.86 Gcorr 0.054840000
H -4.296421 0.108238 -1.142320
Co 0.384939 0.155353 0.205120
C 0.468615 -0.300326 -1.558687
C 1.726264 1.373108 0.080796
O 1.112089 -1.024705 -2.254614
O 2.647336 2.054305 0.117451
C 1.074626 -1.379048 0.717415
O 1.543559 -2.333574 1.155173
C -2.602170 -0.600653 0.017599
C -3.504931 0.521811 -0.510675
H -3.190660 -1.318764 0.601185
H -2.194617 -1.172394 -0.824467
H -3.973900 1.070804 0.310948
H -2.935802 1.243633 -1.102418
C -1.485791 -0.077733 0.923025
O -1.059292 1.157410 0.811267
H -0.366376 0.344010 -1.959173
H -1.444798 -0.520511 1.926948
FREQS= 29 59 71 75 92 110 179 187 232 244 300 358 407 429 449 468 491 510 545 610 620
779 812 869 970 1017 1088 1131 1209 1282 1324 1364 1421 1468 1501 1508 1518 1862 2107 2156 2732 3034 3049
3057 3077 3131 3134
TS061_8
18
E= -1916.39130802 ZPE= 72.54 Gcorr 0.049582000
H 2.306438 -2.141337 0.092259
Co -0.426432 -0.122996 -0.088320
C -1.837235 -1.180698 -0.524063
C -0.299729 -0.311642 1.691318
O -2.604244 -1.943216 -0.899018
O -0.161024 -0.517573 2.809837
C -1.183794 1.516306 -0.364399
O -1.664178 2.520789 -0.613491
C 2.686172 -0.066211 -0.472465
C 2.657319 -1.560290 -0.765966
H 3.356651 0.148624 0.369678
H 3.106295 0.479869 -1.327163
H 2.014834 -1.795426 -1.618687
H 3.668620 -1.905780 -1.004627
C 1.390941 0.696441 -0.143878
O 1.476921 1.881882 0.082555
H -0.060625 -0.149894 -1.524927
H 0.259605 -1.413662 -0.164242
FREQS= 18 59 81 88 92 104 210 258 275 292 345 380 412 434 443 468 489 509 525 545 551
651 678 702 765 866 909 1061 1074 1104 1294 1338 1427 1455 1510 1512 1766 2020 2113 2142 2165 2201 3038
3051 3077 3115 3133
TS062_8
18
E= -1916.39732578 ZPE= 76.62 Gcorr 0.055676000
H 4.153324 0.897705 -1.449264
Co -0.843574 0.030004 -0.151890
C -2.475392 -0.448756 -0.726658
C -0.441025 -1.433064 0.756178
O -3.488673 -0.755053 -1.175356
O -0.206880 -2.295841 1.483008
C -1.237584 1.621826 0.495620
O -1.494001 2.589288 1.070458
C 3.197134 0.084040 0.319766
C 4.352698 0.159138 -0.666863
H 3.411791 -0.633800 1.119131
H 3.012356 1.059154 0.787905
H 5.274731 0.445150 -0.149886
H 4.512469 -0.811970 -1.143503
C 0.959646 0.512200 -0.444638
O 2.007215 -0.372328 -0.339104
H 1.286042 1.547529 -0.309549
H 0.451629 0.385269 -1.466273
FREQS= 25 51 65 81 89 94 158 225 250 320 338 390 405 426 461 498 515 527 557 570 611
789 809 870 1020 1110 1117 1202 1225 1268 1331 1402 1427 1450 1500 1508 1528 2076 2095 2154 2541 3036 3051
3083 3086 3125 3137
TS063_8
18
E= -1916.39414327 ZPE= 74.88 Gcorr 0.052814000
H -4.055637 1.029365 0.481808
Co 0.562370 -0.044582 -0.167894
C 2.204295 0.450091 -0.639976
C 0.134157 1.488115 0.649266

O 3.250286 0.732704 -1.028127
O -0.146331 2.471964 1.181304
C 0.886277 -1.367012 1.002017
O 1.071041 -2.171888 1.806691
C -2.360612 0.487747 -0.762756
C -3.186152 0.369029 0.537935
H -2.987040 0.203843 -1.616669
H -2.033212 1.519229 -0.901642
H -3.540476 -0.654810 0.683197
H -2.596004 0.659884 1.410972
C -1.161954 -0.425442 -0.716965
O -1.548940 -1.628026 -1.122319
H -0.784928 -2.227261 -1.084144
H 0.708808 -0.579728 -1.557907
FREQS= 25 60 67 80 96 107 129 202 225 303 308 358 405 440 456 495 497 522 550 569 592
669 736 763 820 923 1011 1081 1100 1252 1320 1360 1375 1421 1502 1509 1522 1939 2080 2095 2143 3058 3061
3130 3138 3149 3737
TS064_8
18
E= -1916.39029534 ZPE= 72.61 Gcorr 0.049125000
H -2.759928 -2.396247 -0.857807
Co 0.440371 -0.134020 -0.059322
C 1.870751 -1.132789 -0.567948
C 1.024480 1.510849 -0.582211
O 2.649032 -1.875244 -0.958958
O 1.383416 2.515900 -0.985886
C 0.541093 -0.133279 1.732263
O 0.558839 -0.215374 2.875157
C -2.590145 -0.466718 0.188243
C -2.838585 -1.328406 -1.073979
H -3.474839 0.128792 0.434024
H -2.362320 -1.105793 1.047039
H -3.836451 -1.138516 -1.476308
H -2.117352 -1.106182 -1.865449
C -1.440006 0.531848 0.035311
O -1.652751 1.718638 -0.017412
H -0.159945 -1.472130 0.053301
H -0.093009 -0.331784 -1.426401
FREQS= 32 59 70 81 91 95 106 209 277 292 319 351 402 420 443 479 489 525 535 547 624
660 689 693 850 888 933 1021 1079 1119 1250 1318 1433 1487 1511 1518 1788 2028 2069 2142 2161 2199 3058
3065 3110 3130 3144
TS065_8
18
E= -1916.38695783 ZPE= 71.20 Gcorr 0.045909000
H 3.646481 0.882106 1.151480
Co -0.769424 -0.000287 -0.474395
C -0.460449 -1.735190 -0.675360
C 1.038677 0.000675 0.507618
O -0.297835 -2.855785 -0.866212
O 1.048795 0.001082 1.710865
C -1.892673 -0.000724 0.953794
O -2.655516 -0.001001 1.805776
C 2.315189 0.000764 -0.325709
C 3.597223 0.000586 0.507224
H 2.264893 0.871598 -0.992720
H 2.264845 -0.869845 -0.992998
H 3.645963 -0.880745 1.151784
H 4.475030 0.000223 -0.145440
C -0.462194 1.734900 -0.675506
O -0.300786 2.855661 -0.866412
H -1.935682 -0.000915 -1.434131
H -0.758982 -0.000385 -1.953823
FREQS= 48 53 57 85 85 97 100 194 196 258 292 322 330 374 434 453 474 507 510 555 556
622 679 687 777 797 894 1008 1071 1094 1279 1348 1424 1456 1505 1512 1810 1923 2049 2115 2145 2185 3040
3061 3071 3134 3141
TS066_8
18
E= -1916.38848920 ZPE= 72.06 Gcorr 0.047984000
H -4.056450 -0.663840 0.132867
Co 0.647722 -0.078529 -0.154898
C 2.258370 0.216732 -0.890436
C 0.491807 1.473904 0.752356
O 3.253469 0.307611 -1.452932
O 0.443870 2.468302 1.319933
C 0.748195 -1.285691 1.154150
O 0.817379 -2.120394 1.938534
C -2.328157 0.649539 0.011049

C -3.789157 0.310034 -0.283365
H -2.056399 1.635058 -0.386874
H -2.150235 0.705022 1.093121
H -4.448410 1.066704 0.151852
H -3.973245 0.269703 -1.360052
C -1.345395 -0.373331 -0.563360
O -1.722812 -1.391400 -1.082662
H 0.818393 -1.289813 -0.971614
H -0.171362 0.337386 -1.342399
FREQS= 41 63 68 77 93 95 101 205 216 268 309 339 384 403 447 461 490 509 529 546 567 683 698 781 806 847 914 1013 1078 1099 1282 1347 1425 1464 1506 1513 1804 1967 2061 2130 2140 2180 3039 3062 3077 3135 3146
TS067_8
18
E= -1916.39112853 ZPE= 74.63 Gcorr 0.051193000
H -4.321646 0.291663 -0.659022
Co 0.734629 -0.145996 0.050058
C 0.754334 -1.410274 -1.190764
C 1.658874 1.368845 -0.170100
O 0.837234 -2.246740 -1.981483
O 2.336000 2.291521 -0.296398
C 0.499384 -0.748088 1.700209
O 0.403060 -1.159779 2.773430
C -2.212871 -0.329499 -0.443411
C -3.553851 0.213739 0.114192
H -2.277710 -0.458345 -1.535386
H -2.002264 -1.324147 -0.050625
H -3.459929 1.199724 0.585706
H -3.936180 -0.450611 0.891965
C -0.971375 0.519036 -0.249104
O -1.186954 1.818607 -0.359219
H 2.130028 -0.657338 0.264322
H -2.128977 2.029529 -0.505303
FREQS= 20 51 58 63 82 94 95 241 312 320 332 351 402 424 455 488 510 522 537 561 630 696 700 724 841 904 1048 1054 1081 1227 1313 1338 1400 1444 1475 1508 1530 1949 2080 2088 2144 3019 3047 3115 3138 3152 3641
TS068_8
18
E= -1916.38976869 ZPE= 72.61 Gcorr 0.050608000
H 3.984405 0.881090 0.596592
Co -0.669432 0.000000 0.213287
C -0.466282 1.480437 -0.789259
C -0.466284 -1.480438 -0.789256
O -0.362312 2.481439 -1.337914
O -0.362315 -2.481442 -1.337909
C -2.469877 0.000001 0.405771
O -3.572349 0.000002 0.711785
C 2.303428 -0.000004 -0.463008
C 3.762797 0.000000 -0.010553
H 2.083289 0.872527 -1.091443
H 2.083291 -0.872541 -1.091435
H 3.984408 -0.881084 0.596600
H 4.429461 -0.000003 -0.878102
C 1.302502 0.000000 0.701848
O 1.685171 0.000003 1.839806
H -0.696724 0.887277 1.384816
H -0.696724 -0.887273 1.384819
FREQS= 47 51 88 93 100 103 205 205 266 270 350 361 365 428 446 469 497 518 534 539 605 643 676 777 796 847 902 1009 1071 1097 1284 1345 1423 1458 1504 1512 1823 2066 2083 2145 2147 2184 3042 3061 3076 3134 3144
TS069_8
18
E= -1916.39393391 ZPE= 75.06 Gcorr 0.054975000
H 4.153725 0.445086 -0.849935
Co -0.594331 -0.006089 0.185492
C -2.306710 0.316476 0.512435
C -0.160359 1.496983 -0.676179
O -3.399141 0.517077 0.815987
O 0.124013 2.454155 -1.252598
C -0.776468 -1.390743 -0.926904
O -0.886468 -2.272579 -1.660905
C 2.321236 -0.587068 -0.308403
C 3.319408 0.580787 -0.156776
H 2.830319 -1.531510 -0.075076
H 1.958765 -0.639607 -1.336383
H 2.842001 1.537561 -0.385543
H 3.717221 0.624928 0.859704

C 1.153341 -0.440645 0.633335
O 1.572547 -0.646721 1.868320
H 0.808463 -0.523044 2.457928
H -0.853850 -0.019205 1.689545
FREQS= 59 61 76 84 93 104 196 225 270 293 297 353 386 431 468 494 499 537 537 558 590 714 782 812 821 918 1021 1075 1089 1259 1316 1376 1393 1426 1494 1512 1522 1804 2082 2099 2143 3046 3063 3128 3138 3152 3708
TS070_8
18
E= -1916.39607930 ZPE= 76.02 Gcorr 0.057586000
H 3.860947 0.163809 -0.573805
Co -0.204643 0.018709 -0.087057
C -0.489455 -1.360318 -1.608411
C -1.945408 -0.183578 0.444511
O -0.727929 -0.159579 -1.991720
O -3.034858 -0.286752 0.776583
C -0.133635 1.774309 0.300870
O -0.020831 2.879169 0.588864
C 1.722000 0.351628 -0.788782
C 2.953554 -0.117454 -0.022554
H 1.814979 1.422685 -0.983142
H 1.649989 -0.128154 -1.766013
H 3.029973 0.340979 0.969271
H 2.981450 -1.203443 0.111270
C 0.497006 -1.018465 1.163861
O 0.961262 -1.669090 1.990198
H -1.324847 -2.045778 -1.405933
H 0.467340 -1.841953 -1.849476
FREQS= 54 70 84 101 126 132 201 255 270 296 332 354 398 409 411 469 481 497 503 522 545 554 714 722 977 1004 1050 1066 1222 1234 1286 1389 1425 1475 1512 1513 1593 2110 2129 2173 2999 3026 3076 3077 3090 3109 3142
TS071_8
18
E= -1916.39389024 ZPE= 75.53 Gcorr 0.055656000
H 1.659584 2.422816 1.090441
Co -0.675548 -0.056268 -0.116158
C -0.967352 1.631648 -0.495554
C 1.153780 -1.596306 -0.287083
O -1.105626 2.742796 -0.747594
O 2.014727 -2.428387 -0.434854
C -2.405073 -0.465444 0.353006
O -3.419367 -0.716072 0.813430
C 2.429463 0.434724 0.613193
C 2.554410 1.957868 0.660559
H 3.361677 -0.004832 0.235756
H 2.296442 0.034632 1.625388
H 3.408434 2.260033 1.274029
H 2.699743 2.377863 -0.341138
C 1.316899 -0.081715 -0.299349
O -0.149718 -1.811312 -0.080308
H 0.133557 0.546321 0.896477
H 1.467463 0.301563 -1.318691
FREQS= 27 62 77 90 106 119 164 230 291 317 350 392 416 429 447 473 543 573 594 667 741 781 816 894 958 1011 1068 1108 1145 1226 1277 1306 1384 1430 1497 1514 1516 1847 2129 2181 2228 3029 3037 3042 3076 3107 3120
TS072_8
18
E= -1916.38629765 ZPE= 72.19 Gcorr 0.048152000
H -2.306513 1.872650 -0.454961
Co 0.562804 -0.030815 -0.150359
C 1.968422 0.827063 -0.862927
C 0.099694 1.208907 1.074709
O 2.850089 1.291455 -1.430498
O -0.156721 2.023910 1.838878
C 1.089987 -1.403524 0.859732
O 1.444834 -2.327223 1.440565
C -2.499655 -0.201895 0.231186
C -3.047695 1.067992 -0.436929
H -2.213304 0.007578 1.266554
H -3.264979 -0.984386 0.240421
H -3.353556 0.864347 -1.467189
H -3.920989 1.431868 0.111461
C -1.310810 -0.786087 -0.536960
O -1.445085 -1.746574 -1.249238
H 0.960810 -0.956173 -1.221685
H -0.441771 0.388840 -1.185413

FREQS= 44 46 68 75 92 97 113 195 223 291 302 343 384 418 450 462 490 511 537 550 556
662 708 786 812 853 906 1018 1071 1127 1275 1323 1421 1493 1510 1517 1802 1956 2063 2129 2139 2179 3055
3063 3111 3131 3136
TS073_8
18
E= -1916.39517621 ZPE= 77.29 Gcorr 0.057510000
H -2.178230 1.821894 -0.005814
Co 0.536741 -0.010144 -0.019741
C 0.491415 1.728714 0.274180
C 0.842414 -1.445410 0.954708
O 0.558494 2.814528 0.652868
O 1.082821 -2.261000 1.731739
C 2.084097 -0.029669 -0.909005
O 3.037946 -0.061006 -1.548912
C -1.406921 -0.184590 0.460734
C -2.251786 1.050041 0.771314
H -1.176313 -0.301209 -1.717216
H -1.588974 -0.920096 1.247835
H -3.308819 0.770623 0.865896
H -1.955170 1.512943 1.716424
C -1.736597 -0.813495 -0.902832
O -3.125938 -0.826093 -1.234110
H -3.411005 0.088485 -1.357491
H -1.435820 -1.863713 -0.923889
FREQS= 28 49 73 93 100 110 176 269 282 306 313 346 383 394 429 473 496 500 538 572 581
608 840 890 1022 1037 1087 1102 1159 1254 1364 1392 1417 1425 1495 1509 1527 2079 2098 2160 2847 3021 3073
3092 3106 3120 3825
TS074_8
18
E= -1916.38293730 ZPE= 73.69 Gcorr 0.046024000
H -4.585311 -0.122402 0.048945
Co 0.676580 0.167939 0.220670
C -0.101674 1.657608 0.719321
C 2.170456 -0.700455 0.543020
O -0.485681 2.659425 1.140101
O 3.196508 -1.118446 0.857557
C 0.955264 0.558783 -1.479906
O 1.191602 0.871306 -2.561239
C -2.551890 -0.781741 0.338671
C -3.561659 0.209474 -0.161827
H 0.581400 -0.026139 1.711231
H -2.571793 -1.022561 1.402443
H -3.435782 1.185335 0.323215
H -3.474408 0.359342 -1.242247
C -1.628452 -1.360684 -0.436352
O -0.670021 -2.261110 -0.030402
H -0.763123 -2.414554 0.921507
H -1.570165 -1.200680 -1.508903
FREQS= 11 37 41 68 84 95 98 116 201 264 281 296 324 341 437 460 487 491 508 539 573
588 613 762 797 935 968 1065 1115 1126 1259 1337 1395 1436 1492 1510 1731 1939 2084 2105 2169 3031 3083
3116 3141 3209 3792
TS075_8
18
E= -1916.39230881 ZPE= 75.67 Gcorr 0.055593000
H -2.722008 -1.148546 0.254205
Co 0.195351 0.009329 -0.054914
C -0.522553 -0.529758 1.460778
C 1.880488 -0.630541 0.273172
O -1.021863 -0.830615 2.452532
O 2.931910 -1.030395 0.479218
C 0.647870 1.766646 0.022680
O 0.866010 2.886726 0.125105
C -1.709624 0.711917 -0.449334
C -2.936544 -0.202838 -0.251849
H -1.568726 0.949155 -1.505270
H -1.864036 1.650170 0.087547
H -3.706917 0.297740 0.344201
H -3.393339 -0.460808 -1.213107
C -0.126047 -1.487063 -1.445966
O 0.382999 -0.432408 -1.973343
H -1.199592 -1.686149 -1.537877
H 0.506149 -2.370098 -1.271997
FREQS= 59 62 81 88 112 119 122 204 233 272 332 353 394 407 415 448 482 492 505 511 542
556 706 722 1002 1013 1040 1059 1209 1218 1256 1384 1431 1488 1509 1517 1589 2109 2133 2175 3002 3044 3079
3099 3103 3110 3146
TS076_8
18

E= -1916.38843650 ZPE= 74.33 Gcorr 0.051783000
H -4.186360 1.139802 -0.986503
Co 0.495469 0.073155 -0.066823
C 0.616334 1.402065 1.128842
C 1.166435 -1.494804 0.493488
O 0.674576 2.233300 1.925657
O 1.601992 -2.493314 0.866084
C 1.820959 0.458463 -1.191722
O 2.610219 0.736570 -1.982299
C -2.449965 0.534219 0.174116
C -3.345833 0.447040 -1.077857
H -2.097594 1.557602 0.303153
H -3.034535 0.273751 1.071062
H -2.775892 0.711806 -1.972663
H -3.759267 -0.556206 -1.229068
C -1.261662 -0.402479 0.104603
O -1.627485 -1.683029 0.163034
H -0.282160 0.663743 -1.202945
H -2.593868 -1.780930 0.252557
FREQS= 43 54 57 76 92 94 109 208 256 294 313 351 386 439 454 487 493 516 555 567 577
610 690 724 808 923 1037 1069 1095 1253 1317 1326 1400 1425 1494 1511 1519 1923 2085 2099 2146 3006 3049
3116 3140 3148 3656
TS077_8
18
E= -1916.38810097 ZPE= 74.41 Gcorr 0.051715000
H 3.760651 -0.534647 0.480127
Co -0.545284 0.072109 -0.137355
C -1.002804 -1.337387 0.874694
C -0.193657 1.548630 0.815813
O -1.297470 -2.237126 1.529502
O 0.064495 2.477733 1.447976
C -2.175861 0.381071 -0.778720
O -3.190918 0.599250 -1.276521
C 2.320779 0.308294 -0.965063
C 3.362841 0.436302 0.166330
H 1.975016 1.298122 -1.261883
H 2.785201 -0.154945 -1.850819
H 4.204814 1.046888 -0.169781
H 2.921097 0.916737 1.043404
C 1.136720 -0.533384 -0.537263
O 1.454282 -1.822842 -0.423422
H -0.235727 0.516360 -1.528882
H 2.380403 -1.992724 -0.678612
FREQS= 36 49 58 79 93 94 110 204 255 299 312 352 383 440 455 491 493 520 549 567 605
612 688 740 811 921 1030 1072 1099 1251 1315 1322 1397 1426 1498 1512 1521 1947 2085 2099 2145 3000 3052
3120 3141 3151 3653
TS078_8
18
E= -1916.38839696 ZPE= 75.06 Gcorr 0.052071000
H -2.382776 -1.388534 -0.785112
Co 0.420484 -0.145204 0.068387
C 0.737478 -1.538960 -0.994791
C 1.326448 1.127057 -0.814677
O 0.850040 -2.490081 -1.635904
O 1.919779 1.929050 -1.382697
C 1.338393 -0.201539 1.593830
O 1.835837 -0.278708 2.630284
C -2.510087 0.281739 0.637636
C -3.054188 -1.021762 -0.006743
H -3.272141 1.077297 0.571658
H -2.330761 0.118051 1.705716
H -4.033113 -0.847061 -0.460663
H -3.164453 -1.817136 0.732905
C -1.195620 0.761541 0.057059
O -1.288985 1.985685 -0.465685
H -0.363907 -1.180882 0.772370
H -2.193830 2.342748 -0.385192
FREQS= 35 38 44 62 88 91 101 204 310 327 342 368 430 441 462 480 528 548 551 577 584
664 736 842 862 952 1028 1071 1120 1222 1288 1339 1376 1435 1481 1512 1520 2033 2087 2118 2155 2965 3071
3085 3143 3154 3633
TS079_8
18
E= -1916.38224267 ZPE= 73.72 Gcorr 0.045943000
H -3.191688 1.277651 -0.792621
Co 0.616266 -0.064367 -0.244092
C 0.670307 -1.775493 -0.618041
C 1.162599 1.431527 -0.978350

O 0.797752 -2.852116 -1.009062
O 1.597578 2.304654 -1.592490
C 1.646585 -0.051443 1.197237
O 2.393798 -0.063606 2.070551
C -2.724619 -0.520018 0.355349
C -3.442373 0.211748 -0.742405
H -2.957777 -1.573469 0.480838
H -1.808322 1.810790 0.585503
H -3.205760 -0.216413 -1.723305
H -4.528863 0.143781 -0.613612
C -1.816144 -0.015927 1.200135
O -1.348847 1.277642 1.250113
H -1.373613 -0.606208 1.995424
H -0.073531 -0.113173 -1.578199
FREQS= 22 38 49 56 68 82 95 97 99 275 291 320 339 377 456 483 488 493 511 570 584
617 650 751 757 918 945 1035 1054 1124 1247 1338 1424 1444 1486 1511 1730 1948 2082 2107 2169 3032 3093
3094 3190 3223 3799
TS080_8
18
E= -1916.38626524 ZPE= 74.29 Gcorr 0.050155000
H -4.006485 -1.289169 0.589851
Co 0.490774 -0.004176 0.075531
C 0.275888 -1.129081 -1.292901
C 1.499842 1.436636 -0.322008
O 0.130807 -1.850750 -2.182729
O 2.121990 2.363237 -0.601782
C 1.603244 -1.037235 0.999239
O 2.278953 -1.685853 1.669919
C -2.475861 -0.056716 -0.340276
C -3.080857 -0.784811 0.879709
H -2.256450 -0.784254 -1.123405
H -3.215964 0.651994 -0.748667
H -2.381932 -1.530354 1.265317
H -3.311689 -0.085436 1.689349
C -1.212953 0.683379 0.040976
O -1.458669 1.926761 0.444578
H -2.407383 2.142404 0.385911
H 0.088550 0.307364 1.473976
FREQS= 20 29 61 79 94 102 112 207 220 297 318 360 397 431 454 488 492 518 551 565 569
609 682 718 813 925 1018 1075 1115 1249 1316 1323 1391 1421 1503 1507 1524 1982 2080 2096 2142 2980 3057
3123 3139 3149 3668
TS081_8
18
E= -1916.38555773 ZPE= 74.40 Gcorr 0.049548000
H 3.765364 -0.761379 -0.552681
Co -0.145067 0.099587 0.037071
C 0.139883 -1.087851 1.306076
C 0.544305 1.702358 -0.256733
O 0.448144 -1.757217 2.195529
O 1.080237 2.720395 -0.320116
C -1.709274 0.696603 0.665785
O -2.706222 1.086560 1.083750
C 1.605563 -0.602349 -0.736462
C 2.917267 -0.324082 -0.009188
H -1.083666 -2.532822 -2.263098
H 1.632523 -0.183017 -1.747939
H 3.115310 0.748182 0.088652
H 2.925531 -0.750029 0.999534
C -1.460882 -1.673577 -1.681788
O -1.059480 -0.537695 -1.894300
H -2.233178 -1.887704 -0.923025
H 1.472316 -1.685034 -0.847395
FREQS= 43 60 67 75 87 96 104 127 137 183 249 261 273 332 389 401 442 496 504 524 533
565 574 735 956 987 1051 1160 1231 1261 1265 1430 1477 1512 1515 1531 1747 2068 2093 2149 2961 3026 3034
3045 3083 3089 3116
TS082_8
18
E= -1916.39312884 ZPE= 76.03 Gcorr 0.057345000
H -3.866143 -0.322477 -0.152998
Co 0.165729 -0.011923 0.007171
C -0.258335 1.162413 -1.659828
C 1.949594 0.128996 -0.349127
O -0.301088 -0.089825 -1.922191
O 3.076747 0.203872 -0.530458
C 0.270693 -1.745400 0.490516
O 0.301323 -2.837606 0.839474
C -1.850046 -0.247077 0.586333

C -2.884694 0.030348 -0.497296
H -2.047473 0.375004 1.462345
H -1.958649 -1.277239 0.931419
H -2.994416 1.098787 -0.707418
H -2.652514 -0.478937 -1.434505
C 0.146379 1.214592 1.279322
O 0.094164 1.995945 2.122563
H 0.616391 1.756015 -1.963576
H -1.182576 1.728450 -1.503515
FREQS= 51 72 84 107 126 133 195 204 256 297 336 358 399 406 415 440 478 493 505 523 536 560 699 731 988 1013 1043 1065 1220 1271 1291 1394 1414 1493 1499 1520 1593 2105 2128 2170 3004 3029 3077 3090 3107 3120 3149
TS083_8
18
E= -1916.37784258 ZPE= 73.69 Gcorr 0.043534000
H 2.938165 -1.426401 0.437268
Co 0.101825 0.214478 0.163384
C 0.403413 -0.877392 1.525118
C 0.820596 1.513390 -0.787749
O 0.749624 -1.489147 2.435311
O 1.425123 2.351093 -1.296393
C -1.126815 1.205451 0.977778
O -1.958203 1.827253 1.470061
C 1.266545 -1.024913 -0.920252
C 2.758746 -1.104678 -0.593612
H -1.446438 -0.233092 -1.322795
H 1.146108 -0.745464 -1.974295
H 3.266253 -1.822000 -1.251223
H 3.257326 -0.138314 -0.723255
C -2.254896 -0.907282 -1.706184
O -2.906894 -1.608880 -0.976270
H -2.404355 -0.878774 -2.805312
H 0.810941 -2.016890 -0.804011
FREQS= 22 29 45 55 66 80 91 96 102 133 219 233 257 313 381 408 452 485 493 524 529 577 588 745 957 987 1035 1194 1235 1258 1268 1430 1478 1515 1515 1535 1846 2083 2104 2166 2759 2942 3018 3030 3062 3091 3113
TS084_8
18
E= -1916.37744497 ZPE= 73.65 Gcorr 0.043528000
H 2.588740 -2.904467 0.641407
Co 0.217674 0.259117 -0.063325
C 1.559312 1.067955 0.748776
C -0.188897 -0.699689 -1.494900
O 2.535309 1.549959 1.123662
O -0.284665 -1.293412 -2.474828
C -0.634642 1.733945 -0.561715
O -1.224373 2.678922 -0.844551
C 0.969791 -1.448454 0.700204
C 2.297484 -1.969123 0.146782
H -1.593227 -0.326729 0.962661
H 0.198923 -2.217793 0.569524
H 2.239847 -2.174851 -0.926960
H 3.113407 -1.254445 0.296394
C -2.533338 -0.630861 1.495291
O -3.502882 -1.010756 0.893001
H -2.497694 -0.558176 2.601302
H 1.067446 -1.280027 1.780531
FREQS= 18 37 44 56 58 74 93 97 122 130 224 246 253 312 381 407 452 485 493 523 529 576 588 744 957 985 1033 1190 1235 1255 1265 1429 1477 1513 1515 1520 1850 2085 2104 2166 2731 2947 3018 3031 3064 3092 3113
TS085_8
18
E= -1916.38540664 ZPE= 74.50 Gcorr 0.052877000
H -3.976032 0.631454 0.105462
Co 0.500841 0.098493 -0.146920
C 0.402412 1.700512 -0.901409
C 0.329978 0.584199 1.550170
O 0.334771 2.710938 -1.458055
O 0.205590 0.885594 2.655811
C 2.073175 -0.756501 -0.093023
O 3.077698 -1.319203 -0.140547
C -2.390511 -0.695144 -0.537271
C -2.931866 0.426676 0.356482
H -3.042199 -1.583676 -0.463453
H -2.425366 -0.367573 -1.586922
H -2.883155 0.148872 1.413085
H -2.357009 1.343905 0.217984

C -0.948517 -1.070357 -0.293706
O -0.743138 -2.371499 -0.325258
H 0.521163 -0.343350 -1.568549
H -1.567517 -2.871900 -0.493833
FREQS= 39 53 61 82 90 96 184 211 280 309 330 348 388 413 478 482 502 549 554 562 582 646 692 721 798 924 1033 1079 1118 1271 1319 1352 1394 1428 1455 1508 1518 2007 2075 2099 2139 2962 3045 3065 3136 3156 3587
TS086_8
18
E= -1916.38110792 ZPE= 73.66 Gcorr 0.048594000
H -3.762331 0.930691 0.612524
Co 0.758156 0.111935 0.112342
C 1.087799 1.721103 -0.567679
C 0.042916 0.083760 1.754820
O 1.403117 2.774508 -0.912691
O -0.319916 0.115014 2.846287
C 1.786657 -1.250083 -0.403907
O 2.605207 -2.036608 -0.613147
C -2.165289 0.468231 -0.739663
C -3.572000 0.297669 -0.262944
H -0.308263 -0.336925 -1.379316
H -1.838052 1.460708 -1.032240
H -3.781895 -0.739166 0.003834
H -4.283377 0.610337 -1.037082
C -1.262524 -0.523461 -0.842076
O -1.585676 -1.826426 -0.581436
H 1.961264 0.421204 0.854798
H -0.784775 -2.364305 -0.599166
FREQS= 32 52 61 74 80 93 100 162 213 216 264 310 339 347 435 440 457 482 513 520 530 662 693 704 791 930 1027 1059 1061 1151 1262 1283 1394 1432 1497 1499 1721 2074 2091 2106 2157 2884 3037 3086 3155 3208 3844
TS087_8
18
E= -1916.37381463 ZPE= 70.03 Gcorr 0.042036000
H -1.333786 -0.705640 2.476365
Co 0.540273 -0.016108 0.192413
C 2.218177 -0.582928 0.022909
C -0.166036 -1.510496 -0.482930
O 3.303986 -0.952412 -0.019706
O -0.579091 -2.391533 -1.090311
C 0.920606 1.652313 -0.258233
O 1.130899 2.693229 -0.700500
C -1.336331 0.590351 0.709104
C -1.988931 -0.422197 1.647293
H -1.180389 1.551528 1.214452
H 1.065424 -0.132523 2.789639
H -2.899626 0.004935 2.086868
H -2.284549 -1.328260 1.113863
C -2.100830 0.839301 -0.541544
O -2.914806 0.075433 -1.029629
H -1.863193 1.801915 -1.051356
H 1.100892 0.447137 2.316598
FREQS= 39 48 83 91 98 102 114 147 182 222 245 255 269 274 285 321 388 400 457 474 495 502 526 583 586 692 881 961 1006 1075 1110 1160 1366 1417 1432 1497 1510 1786 2095 2115 2173 2871 3035 3050 3099 3142 4327
TS088_8
18
E= -1916.38020084 ZPE= 73.76 Gcorr 0.048704000
H 3.897883 -0.659514 -0.082821
Co -0.775651 0.116908 0.110689
C -1.210279 1.617545 -0.735373
C -1.695767 -1.381423 -0.226622
O -1.607820 2.608485 -1.172423
O -2.420203 -2.271198 -0.325035
C -0.043902 0.287431 1.730328
O 0.334366 0.433618 2.809280
C 2.144088 0.478810 -0.698121
C 3.551165 0.373479 -0.193576
H 1.775817 1.471059 -0.934651
H 0.316784 -0.372306 -1.388770
H 4.244550 0.874845 -0.877914
H 3.655552 0.862890 0.782498
C 1.283598 -0.539141 -0.878503
O 1.507607 -1.869377 -0.693998
H -1.988334 0.434970 0.828818
H 2.355316 -2.000909 -0.247147

FREQS= 29 54 61 70 75 92 92 167 209 268 298 311 337 340 434 441 455 480 512 523 528
656 684 703 783 922 1037 1050 1071 1141 1241 1349 1403 1435 1486 1509 1708 2079 2085 2107 2158 2951 3035
3094 3106 3215 3808
TS089_8
18
E= -1916.37892288 ZPE= 71.43 Gcorr 0.047568000
H -2.986775 -0.613501 -1.093775
Co 0.590817 -0.085520 -0.095490
C -0.236986 -1.562898 -0.604216
C 1.193115 1.548445 -0.536305
O -0.712616 -2.518688 -1.029743
O 1.587479 2.559790 -0.893728
C 2.144815 -0.822576 0.528292
O 3.063445 -1.267801 1.047023
C -2.328289 -0.127746 0.935304
C -3.382590 -0.157196 -0.181146
H -2.757806 0.326996 1.836247
H -1.996823 -1.135947 1.193876
H -4.252340 -0.740039 0.134622
H -3.711793 0.857062 -0.419473
C -1.143923 0.755748 0.539304
O -1.277356 1.956207 0.510687
H 0.785794 -0.191562 -1.581885
H 0.203227 0.167310 1.347302
FREQS= 36 67 77 88 99 110 127 206 231 289 303 320 344 409 420 458 468 497 511 551 576
595 640 710 769 811 911 1017 1076 1118 1272 1329 1418 1497 1510 1520 1776 1856 2001 2119 2145 2190 3055
3057 3116 3128 3146
TS090_8
18
E= -1916.38499624 ZPE= 74.61 Gcorr 0.053643000
H 2.077544 -2.159048 -0.410083
Co -0.452741 -0.085344 -0.092810
C -1.722714 -1.192714 -0.604185
C -0.471735 -0.347718 1.673603
O -2.505993 -1.951915 -0.982379
O -0.478925 -0.498018 2.816142
C -1.204061 1.486843 -0.490495
O -1.685626 2.505829 -0.729603
C 2.424070 -0.087939 -0.986346
C 2.896065 -1.436809 -0.408100
H 3.277456 0.610406 -1.032859
H 2.068837 -0.239599 -2.009533
H 3.715389 -1.831941 -1.014851
H 3.253509 -1.324622 0.619942
C 1.293604 0.511556 -0.184742
O 1.663722 1.566061 0.530900
H -0.008738 -1.114081 -1.114948
H 2.603228 1.788188 0.389332
FREQS= 44 62 65 85 92 100 206 221 241 302 332 356 400 436 467 491 497 524 556 563 592
645 698 744 810 932 1021 1074 1126 1252 1311 1337 1382 1420 1495 1507 1521 1889 2087 2091 2139 2975 3059
3105 3133 3155 3651
TS091_8
18
E= -1916.38022780 ZPE= 72.07 Gcorr 0.048922000
H -1.834011 0.263964 2.094577
Co 0.459561 -0.059234 -0.265607
C 0.897001 1.642316 -0.019246
C 0.020642 -1.375048 0.848193
O 1.149756 2.732050 0.251458
O -0.213810 -2.140411 1.675640
C 2.161461 -0.589100 -0.355269
O 3.263776 -0.907863 -0.360365
C -1.489253 0.669992 -0.027214
C -1.858619 1.102550 1.391064
H -1.555652 1.510619 -0.724844
H -0.268346 -0.218564 -1.916051
H -1.177194 1.867079 1.771683
H -2.870671 1.526405 1.418684
C -2.350085 -0.424216 -0.558304
O -2.520371 -0.670666 -1.739926
H 0.424451 -0.563537 -1.980884
H -2.848399 -1.050487 0.218412
FREQS= 46 69 93 94 105 107 141 192 206 237 305 331 369 384 418 437 475 484 497 514 553
574 581 594 729 912 978 1014 1085 1117 1133 1185 1326 1425 1434 1506 1515 1781 2088 2108 2162 2876 3034
3082 3100 3131 3886
TS092_8
18

E= -1916.38274956 ZPE= 74.06 Gcorr 0.052035000
H -3.805120 0.127087 -1.530714
Co 0.551873 -0.009009 -0.097070
C 1.060478 1.393427 0.907956
C 0.288199 -1.390710 0.976987
O 1.392006 2.238918 1.612919
O 0.185191 -2.291226 1.688264
C 2.083095 -0.291587 -0.974876
O 3.046537 -0.526712 -1.554605
C -2.535272 -0.241201 0.204924
C -3.851891 0.225060 -0.442280
H -2.606009 -0.161160 1.295480
H -2.355404 -1.294224 -0.035969
H -4.691304 -0.376073 -0.079979
H -4.063109 1.273230 -0.205233
C -1.369743 0.586560 -0.281141
O -0.859976 0.345386 -1.486494
H -0.128768 -0.800644 -1.217327
H -1.410100 1.654819 -0.035462
FREQS= 54 61 74 79 85 94 128 187 231 261 310 367 397 444 462 472 498 511 527 529 550
617 794 844 907 993 1007 1095 1160 1260 1297 1347 1417 1466 1485 1511 1519 1873 2097 2110 2162 3048 3055
3063 3099 3122 3132
TS093_8
18
E= -1916.37888413 ZPE= 72.53 Gcorr 0.048317000
H -2.526522 1.167916 -1.161677
Co 0.502681 0.151737 -0.280193
C 0.366318 1.945975 -0.112241
C 0.606248 -0.222445 1.423639
O 0.292824 3.077599 0.088200
O 0.725585 -0.418116 2.553424
C 2.086792 -0.595870 -0.728459
O 3.099226 -1.087417 -0.962819
C -2.481815 -0.385747 0.382675
C -3.145400 0.342841 -0.796374
H -2.283269 0.322475 1.193187
H -3.138453 -1.179873 0.755596
H -4.104943 0.762130 -0.482424
H -3.333282 -0.344125 -1.627136
C -1.167399 -1.050201 -0.023991
O -1.082061 -2.229198 -0.238087
H 0.274532 0.370736 -1.872262
H -0.333503 -0.146434 -1.637315
FREQS= 38 49 57 92 93 100 177 191 211 234 265 291 350 362 437 466 476 511 522 534 545
576 604 615 804 885 1012 1044 1071 1124 1279 1320 1420 1482 1513 1518 1746 1818 2089 2096 2151 3052 3055
3091 3109 3131 3134
TS094_8
18
E= -1916.38283598 ZPE= 74.05 Gcorr 0.052423000
H 4.239331 -0.228012 -0.525457
Co -0.529459 -0.041899 0.108755
C -0.739635 1.607474 -0.573878
C 0.116987 -1.088004 -1.162704
O -0.858814 2.631027 -1.083822
O 0.467243 -1.769385 -2.023845
C -2.250125 -0.467489 0.336183
O -3.339766 -0.797901 0.488261
C 2.501371 -0.329528 0.774663
C 3.265058 0.255803 -0.415393
H 2.308686 -1.398841 0.634996
H 3.118311 -0.244256 1.682659
H 2.713672 0.117612 -1.349864
H 3.441273 1.329165 -0.284783
C 1.202047 0.377653 1.077219
O 0.337661 -0.175392 1.920334
H -0.215818 -1.155633 1.112756
H 1.265137 1.468990 1.169357
FREQS= 45 63 78 86 87 110 128 182 232 300 348 363 390 440 462 474 488 511 515 529 551
572 793 849 910 980 1044 1106 1163 1265 1288 1338 1431 1465 1485 1514 1517 1877 2094 2110 2161 3010 3051
3060 3085 3120 3133
TS095_8
18
E= -1916.37967152 ZPE= 73.75 Gcorr 0.049389000
H 4.148950 1.462814 0.065639
Co -0.823823 0.018031 0.098944
C -2.543558 0.243306 0.153919
C -0.626455 1.761736 -0.224191

```
O -3.680932 0.395236 0.281842
O -0.426773 2.887331 -0.376186
C -1.080409 -1.732181 -0.134049
O -1.176358 -2.877733 -0.226027
C 3.419753 -0.576502 -0.244910
C 4.387553 0.444288 0.383803
H 3.438355 -0.543818 -1.337417
H 3.712013 -1.589688 0.069771
H 4.351014 0.409994 1.476933
H 5.411752 0.223784 0.074206
C 2.019348 -0.374508 0.228435
O 1.070594 -0.243039 -0.542929
H -0.396882 -0.002000 1.593967
H 1.828380 -0.339126 1.313793
FREQS=   22   59   76   85   92   95  104  186  192  227  265  273  367  413  446  473  498  519  569  574  607
623  669  777  897  949 1019 1145 1176 1276 1332 1406 1430 1473 1514 1519 1681 1751 2073 2080 2142 3024 3031
3060 3118 3138 3144
TS096_8
18
E= -1916.37819456 ZPE=    72.04 Gcorr    0.048429000
H -1.846892 -0.316549 2.513968
Co 0.575860 -0.025354 0.367239
C 2.234207 -0.572275 0.008007
C -0.135359 -1.518615 -0.315781
O 3.294809 -0.918250 -0.261794
O -0.569933 -2.440618 -0.842929
C 0.929678 1.628508 -0.176230
O 1.131856 2.682786 -0.590890
C -1.403169 0.643002 0.597327
C -2.257390 -0.243043 1.501731
H -1.297637 1.646429 1.025916
H 1.046600 -0.211958 2.060847
H -3.267687 0.175801 1.586266
H -2.367216 -1.253584 1.102532
C -1.946363 0.779187 -0.783000
O -2.741169 0.012774 -1.296115
H -1.562394 1.654501 -1.354982
H 0.292880 -0.004212 2.091506
FREQS=   42   59   87   97  103  110  119  186  236  268  271  289  367  377  427  446  462  480  488  515  532
576  583  691  768  884  960 1003 1080 1109 1161 1191 1368 1424 1436 1496 1508 1789 2098 2117 2168 2885 3039
3061 3098 3141 3799
TS097_8
18
E= -1916.38180139 ZPE=    74.19 Gcorr    0.052923000
H 2.713578 -0.123396 -1.369649
Co -0.534855 0.044962 0.124540
C -0.725065 1.591400 -0.713132
C 0.081529 -1.161566 -1.068381
O -0.887894 2.578455 -1.284679
O 0.429112 -1.882635 -1.893780
C -2.171947 -0.625541 0.370051
O -3.238270 -1.013856 0.549376
C 2.456537 -0.362879 0.786092
C 3.270324 0.047816 -0.444072
H 2.165392 -1.417728 0.738031
H 3.081488 -0.257562 1.685668
H 3.538958 1.109052 -0.405568
H 4.199042 -0.526572 -0.499974
C 1.232003 0.488613 1.020038
O 0.352849 0.048129 1.917805
H -0.767208 0.723546 1.470756
H 1.403180 1.570870 1.004780
FREQS=   48   67   77   85   93  116  130  181  233  306  315  374  408  437  467  471  496  503  516  526  543
575  797  851  922  989 1047 1109 1171 1266 1292 1339 1432 1465 1489 1514 1518 1891 2097 2108 2161 3016 3052
3066 3087 3122 3134
TS098_8
18
E= -1916.37989060 ZPE=    74.07 Gcorr    0.051029000
H 4.461400 0.612624 0.406789
Co -0.665124 0.101516 -0.244454
C -0.484632 1.778781 -0.730961
C -1.485500 -1.420856 -0.596098
O -0.345813 2.856605 -1.124274
O -1.998659 -2.407360 -0.898777
C -1.431583 0.405970 1.331619
O -1.895266 0.601084 2.369982
C 2.325300 0.447356 0.105148
```

C 3.705433 -0.174731 0.357997
H 2.084535 1.176927 0.888083
H 2.334499 1.034584 -0.821699
H 3.753165 -0.714237 1.311831
H 4.013658 -0.853164 -0.447081
C 1.118437 -0.468994 0.006775
O 1.408468 -1.751949 0.135750
H -0.885742 0.121504 -1.724109
H 2.362260 -1.911370 0.278130
FREQS= 29 48 72 75 94 96 107 223 242 312 338 346 352 459 468 478 495 508 532 579 629
640 663 679 795 889 1015 1065 1084 1284 1311 1333 1391 1427 1452 1520 1522 1944 2076 2089 2140 3033 3055
3080 3101 3132 3612
TS099_8
18
E= -1916.38343526 ZPE= 75.60 Gcorr 0.055347000
H 3.825074 0.731884 0.602542
Co -0.316468 0.034447 0.094731
C -1.080603 1.347211 1.536680
C -1.773971 -0.836139 -0.423163
O -0.921550 0.147884 1.964251
O -2.713765 -1.400433 -0.759511
C 0.844999 -1.239042 -0.167496
O 1.410189 -2.191779 -0.543343
C 3.047602 0.274263 -0.019808
C 1.795950 0.040157 0.815383
H 2.853527 0.950183 -0.858176
H 3.445484 -0.661020 -0.421262
H 1.429931 0.982268 1.218793
H 1.957786 -0.624188 1.665209
C -0.176411 1.284910 -1.181474
O -0.085768 2.022828 -2.059127
H -2.081449 1.710573 1.268484
H -0.343958 2.124065 1.787771
FREQS= 48 68 77 88 100 131 164 176 224 270 311 351 381 395 407 444 480 497 509 538 578
580 699 764 977 993 1036 1064 1218 1227 1270 1386 1427 1463 1506 1518 1590 2006 2109 2155 2996 3047 3077
3107 3121 3135 3173
TS100_8
18
E= -1916.37743205 ZPE= 74.10 Gcorr 0.049828000
H -4.034594 -0.674289 1.012548
Co 0.536603 -0.117189 -0.012182
C 0.313278 1.403629 -0.850031
C 1.365634 0.537024 1.385382
O 0.290956 2.379724 -1.465428
O 2.206243 1.173720 1.924581
C 1.826590 -1.185156 -0.626648
O 2.708442 -1.723161 -1.134134
C -2.735984 -0.115674 -0.637752
C -3.740182 0.222756 0.463451
H -3.186629 -0.794663 -1.379420
H -2.426438 0.769559 -1.202595
H -3.310252 0.927311 1.181262
H -4.635516 0.678426 0.033270
C -1.509284 -0.830242 -0.126817
O -1.435921 -1.433974 0.933123
H -0.694491 -1.023492 -0.934830
H 0.521578 -0.083241 1.908043
FREQS= 25 39 66 82 86 100 120 160 198 251 280 322 340 376 414 460 485 512 538 559 649
662 766 864 1009 1011 1107 1139 1172 1274 1369 1374 1429 1467 1508 1515 1757 1934 2084 2136 2352 2387 3012
3064 3096 3136 3150
TS101_8
18
E= -1916.37007488 ZPE= 70.86 Gcorr 0.042796000
H 3.013090 -1.295752 0.363202
Co -0.659279 -0.022871 -0.161946
C -2.334272 -0.413333 -0.592022
C -0.320219 -1.673903 0.501587
O -3.432789 -0.665176 -0.809488
O -0.057783 -2.644721 1.047942
C -1.060958 1.626239 0.437368
O -1.242820 2.618676 0.980637
C 3.129326 0.628461 -0.580032
C 3.730707 -0.691845 -0.196483
H 3.741847 1.325463 -1.147853
H 0.327821 -0.069858 -1.877822
H 4.055554 -1.263355 -1.075806
H 4.618883 -0.558388 0.435049

C 1.898536 1.042125 -0.230585
O 1.060398 0.338744 0.557555
H -0.383865 0.157858 -2.031055
H 1.552451 2.034912 -0.545347
FREQS= 20 37 57 92 95 96 117 136 188 220 238 320 357 383 402 410 433 445 447 465 522 570 580 594 725 780 830 934 952 1057 1066 1162 1295 1403 1450 1499 1507 1701 2121 2126 2184 3023 3049 3065 3136 3173 4103
TS102_8
18
E= -1916.38216318 ZPE= 75.40 Gcorr 0.055000000
H 2.523488 -0.811254 -1.506392
Co -0.220230 -0.004223 0.071635
C -0.297242 -0.172735 2.263392
C 0.096030 1.717172 -0.246754
O -1.421236 0.129414 1.800826
O 0.372788 2.805935 -0.490613
C -1.678827 -0.058023 -0.969598
O -2.590283 -0.098675 -1.664212
C 1.872450 0.062996 0.389326
C 2.693279 0.081339 -0.896762
H 2.151819 -0.798092 1.003146
H 2.092092 0.949629 0.990882
H 2.465071 0.951683 -1.519649
H 3.766287 0.118941 -0.664284
C 0.200299 -1.688395 -0.266231
O 0.551790 -2.753241 -0.528006
H -0.055468 -1.215985 2.522086
H 0.382514 0.597504 2.655863
FREQS= 52 84 89 99 106 114 143 169 215 251 262 355 370 378 384 437 453 484 513 546 555 587 624 739 980 983 1052 1080 1232 1238 1272 1434 1475 1496 1511 1511 1647 2089 2117 2164 2982 3026 3059 3065 3094 3101 3126
TS103_8
18
E= -1916.36875888 ZPE= 73.84 Gcorr 0.041715000
H 3.437005 -0.739515 1.070314
Co -0.363405 -0.340181 -0.039558
C -0.531078 0.323468 1.624710
C -1.450856 0.699515 -1.016811
O -0.523568 0.726760 2.699166
O -2.037086 1.399205 -1.719078
C -1.188318 -1.926708 0.024633
O -1.617744 -2.993360 0.027276
C 2.574844 -0.715931 -0.937620
C 3.587082 -0.222568 0.119391
H 2.747001 -0.213769 -1.902229
H 2.705374 -1.793711 -1.121142
H 4.607163 -0.416085 -0.223204
H 3.479281 0.851017 0.293212
C 1.183106 -0.492719 -0.546809
O 0.135516 3.297144 0.035151
H 0.662742 2.588337 -0.355976
H -0.532252 3.480289 -0.638013
FREQS= 13 26 39 51 62 79 87 89 140 177 180 198 225 288 313 337 431 469 472 485 489 523 553 586 669 778 957 1036 1042 1245 1249 1356 1425 1439 1508 1515 1664 2094 2112 2159 3004 3041 3062 3140 3146 3797 3905
TS104_8
18
E= -1916.38068305 ZPE= 74.05 Gcorr 0.053781000
H 4.143915 0.174046 1.541990
Co -0.493680 0.022316 -0.031528
C -1.992869 0.740279 0.525768
C 0.091201 1.660501 -0.478311
O -2.935806 1.203857 0.997034
O 0.486877 2.692362 -0.783123
C -1.318721 -1.574658 -0.098421
O -1.796581 -2.614275 -0.159283
C 2.678753 -0.153236 -0.027427
C 3.148755 -0.263435 1.423180
H 2.602711 0.890429 -0.346066
H 3.424786 -0.618521 -0.691072
H 2.462347 0.255566 2.098430
H 3.205941 -1.309454 1.743642
C 1.390392 -0.885867 -0.329127
O 0.740430 -0.670012 -1.429883
H 0.211180 -0.135432 1.306990
H 1.334055 -1.896119 0.105400

FREQS=   55   74   90 101 106 123 162 204 238 292 328 364 405 420 436 461 477 504 540 582 599
619 621 780 899 971 1040 1099 1165 1278 1337 1342 1427 1481 1499 1512 1519 1825 2105 2122 2174 3007 3012
3050 3100 3122 3131
TS105_8
18
E= -1916.38191475 ZPE=   76.04 Gcorr   0.055451000
H -4.107770 0.458135 -1.088641
Co 0.669383 -0.133076 -0.026050
C 0.712291 0.722979 1.470641
C 1.441573 -1.653249 0.611089
O 0.754330 1.280115 2.483016
O 1.898126 -1.828315 -0.507398
C 1.098127 1.231194 -1.111234
O 1.571331 2.039739 -1.780447
C -2.466886 0.318852 0.360138
C -3.889361 -0.020508 -0.128249
H -2.378154 1.400912 0.509957
H -2.262201 -0.179576 1.312092
H -4.009657 -1.100508 -0.251175
H -4.631536 0.327604 0.595707
C -1.452582 -0.133070 -0.660729
O -0.919354 -1.271617 -0.631369
H 1.653154 -2.313532 1.467574
H -1.431602 0.443469 -1.602533
FREQS=   44   61   78   88 106 127 148 168 198 218 233 345 365 431 465 472 488 521 530 576 650
798 842 878 938 1000 1091 1158 1230 1283 1332 1394 1420 1486 1512 1519 1617 1701 2077 2124 2983 3017 3051
3057 3110 3127 3136
TS106_8
18
E= -1916.38024704 ZPE=   74.12 Gcorr   0.053811000
H -4.690750 -0.301747 0.586790
Co 0.577159 -0.005347 0.047176
C 2.228536 -0.547129 0.280742
C 0.105313 -1.698331 -0.326027
O 3.292178 -0.899119 0.546438
O -0.219420 -2.767115 -0.583711
C 1.188796 1.675755 -0.145461
O 1.525384 2.763329 -0.274933
C -2.518805 -0.198270 0.703078
C -3.877466 0.311458 0.187583
H -2.497991 -0.178069 1.797408
H -2.369021 -1.234048 0.386309
H -4.057667 1.347238 0.494696
H -3.919288 0.268950 -0.904360
C -1.410719 0.680025 0.165038
O -0.986024 0.545589 -1.053791
H 0.145693 0.068600 1.503408
H -1.385138 1.690929 0.600244
FREQS=   68   87   90   92 101 110 152 204 228 241 324 365 406 435 443 461 483 499 565 585 599
618 624 797 889 987 1002 1093 1163 1286 1332 1349 1418 1488 1502 1511 1521 1825 2106 2122 2174 3011 3049
3070 3112 3126 3137
TS107_8
18
E= -1916.38032486 ZPE=   76.00 Gcorr   0.053945000
H 3.392034 0.285656 -1.752564
Co -0.488396 0.281799 0.054061
C -2.103682 -0.097268 -0.370840
C -0.301469 -1.618974 0.295244
O -3.189096 -0.337839 -0.677772
O -0.215420 -2.085530 1.413424
C -0.777729 2.039468 0.117540
O -1.042285 3.153527 0.216030
C 2.984215 0.277984 0.367274
C 3.924493 0.230015 -0.799067
H 1.052127 0.429836 1.200574
H 3.433123 0.218488 1.357764
H 4.643841 1.056406 -0.755407
H 4.508146 -0.697981 -0.784126
C 1.661176 0.394883 0.281019
O -0.213432 -2.406580 -0.810186
H 1.186908 0.470542 -0.720709
H -0.069633 -3.316792 -0.484158
FREQS=   25   58   67   96 109 116 123 180 196 219 237 319 368 422 468 477 488 512 576 613 617
668 729 771 943 989 1036 1070 1111 1238 1303 1358 1426 1489 1493 1563 1748 1769 2093 2147 2854 2998 3038
3094 3127 3166 3656
TS108_8
18

E= -1916.37388626 ZPE=   71.75 Gcorr   0.047558000
H 5.014051 -0.880031 -0.413648
Co -0.910223 -0.000001 0.349141
C -2.618604 0.000002 -0.135387
C -0.587960 1.678070 -0.243197
O -3.706287 0.000004 -0.497279
O -0.243806 2.696722 -0.636326
C -0.587961 -1.678070 -0.243201
O -0.243809 -2.696721 -0.636332
C 3.212717 -0.000004 0.407840
C 4.370611 0.000004 -0.548281
H -1.244974 0.390538 2.077387
H 3.429062 -0.000016 1.474589
H 4.029469 0.000017 -1.589438
H 5.014056 0.880033 -0.413629
C 1.930821 0.000002 0.017466
O 0.890417 -0.000005 0.879823
H -1.244979 -0.390548 2.077383
H 1.689481 0.000013 -1.049972
FREQS=   41   50   82   92   93 113 149 200 249 292 294 347 348 360 418 429 444 466 476 477 504
570 574 671 722 797 942 949 1063 1129 1139 1217 1295 1368 1431 1492 1515 1729 2131 2131 2186 3012 3055
3085 3097 3166 3796
TS109_8
18
E= -1916.36789788 ZPE=   73.76 Gcorr   0.041637000
H 3.681136 -0.333889 -0.796572
Co -0.112186 0.322205 -0.015431
C -1.078844 0.239412 1.506583
C 0.679561 1.917578 0.044525
O -1.607374 0.121854 2.518062
O 1.287930 2.893777 0.075197
C -1.365296 0.519032 -1.299220
O -2.093252 0.570686 -2.186349
C 2.136354 -1.787221 -0.270888
C 3.504359 -1.107768 -0.045579
H 2.108184 -2.274167 -1.258085
H 1.976741 -2.581793 0.474781
H 3.547285 -0.643404 0.942663
H 4.305791 -1.848441 -0.115863
C 1.010620 -0.854652 -0.185037
O -2.312598 -2.260640 0.021779
H -2.689495 -3.024371 -0.431810
H -1.418796 -2.157176 -0.330272
FREQS=   27   29   36   45   52   78   80   88   99 128 178 201 283 304 316 338 428 468 470 479 490
525 548 601 656 780 956 1039 1044 1248 1252 1355 1423 1441 1508 1515 1626 2103 2115 2163 3002 3038 3061
3139 3146 3780 3908
TS110_8
18
E= -1916.36825190 ZPE=   73.92 Gcorr   0.042099000
H -3.602098 -0.428112 0.887334
Co 0.136888 0.329422 -0.000163
C -0.658912 1.924676 0.000765
C 1.245193 0.404913 1.423414
O -1.267702 2.900416 0.001212
O 1.870085 0.388832 2.385798
C 1.249411 0.409212 -1.420317
O 1.877180 0.395909 -2.380867
C -2.130212 -1.774844 -0.004288
C -3.497136 -1.056292 -0.000567
H -2.039773 -2.429726 0.876396
H -2.041828 -2.424630 -0.888938
H -3.604327 -0.423155 -0.884670
H -4.305266 -1.792836 -0.001613
C -0.996025 -0.849986 -0.002847
O 2.185612 -2.472046 -0.000829
H 1.322749 -2.032085 -0.001972
H 1.979237 -3.414797 -0.001631
FREQS=   19   32   32   45   50   77   80   91 107 178 203 240 280 306 334 338 428 469 470 479 488
525 547 600 656 780 956 1038 1042 1247 1251 1354 1423 1441 1508 1515 1657 2108 2118 2165 3002 3039 3062
3140 3147 3751 3886
TS111_8
18
E= -1916.37928013 ZPE=   74.82 Gcorr   0.053959000
H -4.078174 0.860077 -0.036512
Co 0.572259 -0.062900 -0.246612
C 0.218010 -1.583033 0.605689
C 2.304274 -0.475646 -0.418482

O -0.040249 -2.603277 1.079972
O 3.422235 -0.737952 -0.440059
C 1.058710 1.601717 0.232748
O 1.375240 2.697863 0.424497
C -2.357123 -0.175537 -0.899136
C -3.760770 -0.154845 -0.285482
H -2.066707 -1.176963 -1.229922
H -2.326655 0.428970 -1.819040
H -4.479533 -0.573566 -0.995246
H -3.802843 -0.754013 0.629020
C -1.229520 0.366476 -0.067820
O -1.692792 1.177554 0.862625
H -0.953667 1.513784 1.398880
H 0.339635 -0.348274 -1.710038
FREQS= 22 66 91 93 96 106 215 230 266 307 351 362 386 421 446 475 510 518 539 568 597 687 728 769 793 920 1026 1078 1091 1264 1302 1359 1415 1431 1453 1510 1517 1867 2052 2092 2145 3027 3061 3104 3131 3146 3707
TS112_8
18
E= -1916.38492626 ZPE= 76.60 Gcorr 0.060131000
H -3.298775 -1.361555 1.261166
Co 0.160269 0.120087 -0.134338
C -0.590006 1.537032 0.579102
C 1.322523 -1.403940 0.461388
O -1.091335 2.471365 1.022814
O 1.874405 -1.901808 1.390778
C 1.654257 1.007888 -0.316006
O 2.599801 1.623069 -0.545451
C -1.770300 -0.991375 -0.260817
C -3.132219 -0.727821 0.385058
H -1.031198 -0.939584 0.651818
H -1.640370 -2.038275 -0.545844
H -3.230156 0.316681 0.696639
H -3.922209 -0.931432 -0.343768
C -1.383043 -0.054422 -1.376200
O 1.031799 -1.534041 -0.753429
H -1.049742 -0.523536 -2.298477
H -2.079429 0.762500 -1.547261
FREQS= 85 90 97 106 109 151 185 232 254 284 358 400 420 453 479 504 522 542 553 622 669 763 809 908 964 988 1070 1118 1180 1192 1325 1372 1428 1489 1505 1510 1596 2015 2101 2140 2227 3052 3106 3128 3130 3136 3215
TS113_8
18
E= -1916.38384710 ZPE= 77.61 Gcorr 0.059114000
H 4.145684 1.133546 -0.096491
Co -0.769206 -0.001557 -0.349279
C -1.976947 1.276238 -0.220534
C -1.410476 -1.626337 -0.202330
O -2.872083 1.973118 -0.001363
O -1.972136 -2.614968 0.010834
C 0.116089 0.161159 1.109974
O 0.328290 0.201954 2.273517
C 2.588512 -0.316520 0.381171
C 3.885942 0.095353 -0.326535
H 2.361844 -1.376317 0.193728
H 2.697296 -0.217171 1.464922
H 4.714969 -0.538448 0.000695
H 3.808158 0.007582 -1.414869
C 1.388845 0.516663 -0.040829
O 1.080058 0.354374 -1.444848
H 1.558514 1.585320 0.102065
H 1.417282 -0.507617 -1.730145
FREQS= 37 73 80 92 104 117 186 218 257 284 318 330 371 455 464 501 509 536 561 570 634 728 789 869 965 1049 1091 1107 1224 1248 1331 1360 1405 1430 1499 1511 1518 1933 2061 2119 3025 3051 3106 3120 3128 3129 3796
TS114_8
18
E= -1916.37573929 ZPE= 73.86 Gcorr 0.051154000
H 2.410977 0.342606 -1.732916
Co -0.417991 0.196390 0.091827
C -0.873008 -0.553299 -1.573036
C -0.588778 -1.379121 0.955052
O -1.996773 -0.621663 -1.996773
O -0.712430 -2.417097 1.426183
C -2.024246 1.034272 0.405641
O -2.957129 1.668061 0.588749
C 3.979688 0.353928 -0.182972

C 2.613535 -0.088395 -0.744464
H 4.639044 -0.502105 -0.021995
H 4.485136 1.039830 -0.866743
H 2.561858 -1.173687 -0.900624
H 3.852022 0.866433 0.773315
C 1.426048 0.269875 0.106406
O 1.457584 0.866158 1.173764
H -0.172256 1.289779 -0.895554
H -0.020493 -0.952618 -2.169965
FREQS= 48 59 74 76 77 98 115 183 222 289 292 331 353 396 417 431 485 497 516 614 710 721 769 823 903 955 1054 1083 1094 1244 1321 1351 1428 1460 1515 1521 1729 1802 1967 2136 2176 2849 3048 3070 3076 3142 3147
TS115_8
18
E= -1916.37533934 ZPE= 74.95 Gcorr 0.050877000
H 4.743882 1.063438 -0.802313
Co -0.835756 0.007785 -0.198287
C 0.884099 -0.218379 -0.782324
C -0.389968 -0.003752 1.538127
O 1.962057 -0.325391 -0.026895
O -0.094941 -0.010094 2.647649
C -2.001510 -1.340302 -0.263918
O -2.723559 -2.224590 -0.413525
C 3.272192 -0.540755 -0.622990
C 4.278515 0.449699 -0.027112
H 3.172992 -0.434905 -1.708690
H 3.537720 -1.577915 -0.400296
H 3.763329 1.118590 0.666697
H 5.070513 -0.060125 0.526231
C -1.657685 1.585627 -0.328298
O -2.155999 2.605593 -0.520959
H -1.099277 0.010149 -1.652493
H 1.161929 -0.286412 -1.846451
FREQS= 21 32 64 74 82 92 96 181 223 311 320 349 412 443 458 474 479 533 551 559 696 770 854 864 892 952 1038 1096 1171 1256 1322 1397 1436 1469 1503 1508 1528 2024 2094 2120 2157 3002 3054 3069 3110 3145 3148
TS116_8
18
E= -1916.36685762 ZPE= 70.49 Gcorr 0.042679000
H 3.567722 1.285226 1.634113
Co -0.590033 0.051447 -0.329899
C -1.811573 -0.504549 0.766692
C 0.365399 -1.599811 -0.278008
O -2.591070 -0.871302 1.531505
O 0.237133 -2.789957 -0.229511
C -0.959026 1.821107 -0.247156
O -1.082864 2.945999 -0.062791
C 2.480176 -0.305361 0.637898
C 2.683257 1.167708 1.002306
H 3.392630 -0.710498 0.174164
H 2.299607 -0.922492 1.524607
H 2.816327 1.774643 0.103853
H 1.821364 1.559037 1.551478
C 1.381162 -0.555044 -0.360836
O 1.051278 0.292918 -1.285932
H -1.962216 -0.294825 -2.647137
H -1.756723 -0.845723 -2.185344
FREQS= 34 52 71 87 95 98 120 142 174 187 217 234 266 278 329 392 420 435 453 475 534 543 589 633 664 755 786 985 1045 1076 1166 1282 1381 1415 1443 1474 1508 1517 1887 2103 2154 3020 3059 3088 3129 3147 4361
TS117_8
18
E= -1916.37724425 ZPE= 75.53 Gcorr 0.053338000
H -3.157290 -0.446876 1.725631
Co 0.285714 0.005136 -0.029424
C 0.383146 -1.293988 -1.235394
C 1.020428 1.613272 -0.378549
O 0.467109 -2.071313 -2.086164
O 1.509814 2.596617 -0.727038
C 1.645533 -0.595003 0.931700
O 2.465365 -0.989354 1.630373
C -2.068563 0.628296 0.215540
C -3.104897 -0.361482 0.640056
H -1.248690 1.587239 -1.507484
H -2.007667 1.536585 0.804396
H -4.078289 -0.005648 0.273746
H -2.931533 -1.345852 0.195195

C -1.519524 0.636464 -1.062510
O -0.655693 -0.051007 1.746456
H -0.888481 -0.978142 1.893331
H -1.831829 -0.130868 -1.764442
FREQS= 30 49 53 90 93 106 137 199 255 264 310 375 410 413 435 451 472 480 496 517 544 556 572 806 830 924 962 972 1016 1066 1208 1283 1399 1447 1481 1510 1566 2069 2098 2153 3031 3104 3155 3161 3219 3245 3818
TS118_8
18
E= -1916.37255015 ZPE= 73.54 Gcorr 0.049007000
H -3.600920 1.359974 0.170798
Co 0.662993 -0.176763 -0.185285
C 1.402870 -0.715557 1.338547
C 1.414814 1.328244 -0.789399
O 1.884416 -1.067640 2.321898
O 1.962454 2.252992 -1.200162
C -0.012462 -1.550905 -1.084303
O -0.378081 -2.462488 -1.686867
C -2.171618 -0.187471 0.763272
C -3.415772 0.297299 -0.006898
H -2.321315 0.007957 1.836496
H -2.037369 -1.263728 0.641104
H -3.301702 0.134133 -1.083551
H -4.299292 -0.255771 0.321773
C -0.908760 0.542776 0.386830
O -1.055608 1.887154 0.624024
H 1.903709 -0.731163 -0.855362
H -1.403819 2.354735 -0.148025
FREQS= 26 39 58 69 84 95 104 209 254 298 310 325 356 418 439 453 490 503 523 547 569 654 690 699 798 940 966 1046 1080 1099 1221 1268 1357 1413 1478 1515 1523 1896 2092 2103 2153 3018 3054 3121 3131 3142 3819
TS119_8
18
E= -1916.37745691 ZPE= 74.16 Gcorr 0.054281000
H 3.962294 -0.172315 -1.632057
Co -0.581469 -0.039619 0.312929
C -1.260351 1.542417 -0.223040
C 0.364559 -1.572743 0.245575
O -1.729947 2.559641 -0.470211
O 0.905903 -2.584118 0.297754
C -1.932597 -0.862301 -0.426117
O -2.862643 -1.409043 -0.824655
C 2.650796 0.334580 0.025742
C 2.931523 0.153343 -1.468125
H 2.748555 -0.604724 0.575903
H 3.401731 1.018895 0.453490
H 2.264556 -0.594464 -1.907178
H 2.788185 1.091370 -2.015370
C 1.325905 0.991122 0.340987
O 0.733906 0.835344 1.474232
H -1.485844 -0.332829 1.501566
H 1.163432 1.930677 -0.212517
FREQS= 36 81 98 104 120 134 180 228 251 300 311 379 410 430 456 477 502 519 547 559 579 597 653 787 888 939 1031 1093 1163 1271 1334 1353 1430 1486 1511 1513 1525 1809 2106 2115 2165 2989 3002 3051 3109 3126 3132
TS120_8
18
E= -1916.37620429 ZPE= 74.52 Gcorr 0.053956000
H 2.828775 -0.270743 1.997844
Co -0.493968 -0.045539 0.199672
C -2.198978 -0.430410 0.592712
C -1.021119 1.619129 -0.260840
O -3.313566 -0.671228 0.738995
O -1.367127 2.682627 -0.532173
C -0.329684 -1.616036 -0.616328
O -0.205763 -2.643791 -1.128671
C 2.435404 -0.587398 -0.119472
C 3.370150 -0.213646 1.050313
H 2.988878 -0.536489 -1.071012
H 2.099940 -1.615848 0.005741
H 4.214494 -0.906800 1.088093
H 3.777480 0.798945 0.956127
C 1.248248 0.347781 -0.201943
O 1.608209 1.577564 -0.566665
H 0.063329 -0.158206 1.603682
H 2.566085 1.640788 -0.730149

FREQS= 33 53 96 99 108 114 216 257 267 300 317 360 388 403 447 466 506 509 554 586 589 606 627 704 809 926 1035 1072 1100 1256 1316 1329 1404 1426 1494 1511 1521 1839 2071 2098 2146 3002 3050 3119 3146 3159 3688
TS121_8
18
E= -1916.37804367 ZPE= 75.84 Gcorr 0.055848000
H -2.922737 0.623599 -1.155808
Co 0.579300 -0.055523 -0.056951
C 1.325894 -1.637967 -0.540733
C -0.275912 1.504071 -0.484328
O 0.977549 -1.616289 -1.706151
O -0.756818 2.451011 -0.921019
C 2.121954 0.639337 0.428669
O 3.080017 1.115799 0.856221
C -2.523995 -0.126810 0.858978
C -3.399561 0.004908 -0.390003
H -2.292057 0.848369 1.299187
H -3.077288 -0.683227 1.631462
H -3.603922 -0.975852 -0.833568
H -4.361502 0.462811 -0.144099
C -1.244176 -0.905422 0.639478
O -0.291215 -0.872021 1.490294
H 1.863229 -2.481918 -0.071053
H -1.348302 -1.791353 -0.015578
FREQS= 46 71 85 91 99 116 164 173 223 238 291 354 371 406 416 469 502 510 521 529 646 765 877 893 907 1037 1095 1153 1245 1277 1335 1368 1432 1481 1513 1517 1546 1727 2093 2135 2950 2983 3011 3051 3090 3120 3132
TS122_8
18
E= -1916.36769135 ZPE= 71.11 Gcorr 0.045857000
H -3.342899 0.843836 0.509166
Co 0.591430 -0.113279 -0.233622
C 0.795317 1.617089 -0.406024
C -0.701302 0.078847 1.155691
O 0.926594 2.751731 -0.543979
O -0.915010 0.778788 2.098031
C 2.317267 -0.628587 -0.044846
O 3.366176 -0.931664 0.304306
C -2.474808 -0.885064 -0.501680
C -3.187061 0.471111 -0.507328
H -3.115533 -1.635779 -0.017375
H -2.292514 -1.247916 -1.519852
H -2.609271 1.222267 -1.055668
H -4.165505 0.384372 -0.986967
C -1.159529 -0.917833 0.240643
O -0.218061 -1.811443 -0.010618
H 0.398094 -0.920958 -2.303716
H 0.342127 -0.299967 -2.718440
FREQS= 42 45 80 84 94 144 148 164 209 242 271 304 326 344 389 402 415 455 480 489 497 525 567 617 634 724 794 972 1070 1085 1253 1274 1318 1382 1432 1487 1513 1518 1928 2107 2158 3027 3054 3077 3125 3135 4339
TS123_8
18
E= -1916.37019675 ZPE= 73.44 Gcorr 0.048897000
H -2.832775 -1.120739 -1.533598
Co 0.477714 -0.243094 0.000189
C 1.000357 0.826500 -1.435223
C 0.499031 1.160132 1.143642
O 1.397658 1.961965 -1.401364
O 0.484960 2.018013 1.902907
C 2.045604 -1.158456 0.107462
O 3.015708 -1.766304 0.173400
C -3.753414 0.081302 0.029290
C -2.579989 -0.262461 -0.893215
H -3.982586 -0.755190 0.693042
H -3.521392 0.951301 0.650500
H -2.332021 0.563490 -1.567151
H -4.643680 0.313987 -0.560685
C -1.339388 -0.653638 -0.133321
O -1.328723 -1.279721 0.928353
H -0.317818 -1.104581 -1.012851
H 0.941977 0.283387 -2.412524
FREQS= 32 53 59 68 71 98 137 162 212 216 281 292 340 373 399 437 470 495 512 617 640 677 791 878 959 1022 1060 1109 1132 1271 1317 1367 1426 1472 1510 1517 1681 1800 1864 2124 2162 2801 3027 3063 3097 3134 3150
TS124_8
18

E= -1916.37028284 ZPE=   73.33 Gcorr   0.049083000
H -3.121661 -0.241090 1.055417
Co 0.414737 -0.250323 -0.017101
C 0.421602 1.228987 -1.155081
C 0.685146 0.783785 1.443769
O 0.678766 2.372015 -0.878678
O 0.840731 1.396195 2.399391
C 1.999066 -0.915922 -0.610993
O 2.984037 -1.376978 -0.974508
C -3.024073 0.539120 -0.978591
C -2.761195 -0.544819 0.064359
H -2.690716 0.224314 -1.972820
H -4.093717 0.755157 -1.041645
H -3.295030 -1.473476 -0.179018
H -2.506801 1.468964 -0.725450
C -1.311146 -0.906592 0.247784
O -0.893684 -1.780970 1.010442
H -0.602197 -0.915074 -0.984239
H 0.177019 0.950474 -2.211184
FREQS=   33   54   62   68   72   99 148 165 197 221 273 319 357 398 424 457 467 494 512 543 613
634 787 882 945 1025 1081 1123 1143 1278 1317 1344 1432 1454 1512 1515 1674 1798 1852 2124 2162 2805 3039
3054 3079 3125 3136
TS125_8
18
E= -1916.37100953 ZPE=   73.90 Gcorr   0.050131000
H -2.563357 1.701252 0.590428
Co 0.378684 0.147354 -0.025915
C 0.688629 1.153591 1.419514
C 1.383288 -1.317795 0.311371
O 0.748029 1.834013 2.344909
O 2.037002 -2.234900 0.524256
C 1.301597 0.841346 -1.393999
O 1.780146 1.319079 -2.325063
C -2.620806 -0.330585 -0.187737
C -2.947113 1.155355 -0.275876
H -3.105874 -0.780037 0.692778
H -3.064663 -0.871595 -1.040393
H -2.523805 1.610534 -1.176017
H -4.032320 1.296466 -0.307850
C -1.180909 -0.776627 -0.108288
O -1.193459 -2.165182 0.027130
H -1.161138 -2.582589 -0.845475
H -0.495178 1.271615 -0.373543
FREQS=   26   34   68   86   90   99 104 214 252 311 336 339 413 429 438 458 482 493 523 536 541
572 675 766 855 957 988 1056 1099 1122 1176 1284 1363 1426 1435 1508 1511 2092 2099 2143 2167 2990 3027
3052 3119 3132 3807
TS126_8
18
E= -1916.36610443 ZPE=   72.11 Gcorr   0.046162000
H 2.477874 -1.136625 -0.291152
Co -0.416579 -0.034402 -0.074394
C -0.165999 0.658545 1.518531
C -2.044399 0.688792 -0.391359
O 0.027291 1.132793 2.547486
O -3.116002 1.042945 -0.618956
C -0.359272 -1.750915 0.487378
O -0.402011 -2.866661 0.762583
C 2.506859 1.018388 -0.442409
C 3.173148 -0.299758 -0.197810
H 3.188405 1.875115 -0.433615
H 0.648541 0.289160 -1.525286
H 4.004267 -0.446322 -0.898918
H 3.612161 -0.323915 0.808299
C 1.193650 1.224770 -0.692326
O -0.093311 -0.631851 -1.933354
H 0.904022 2.262706 -0.861607
H -0.739313 -0.247999 -2.543181
FREQS=   28   49   53   77   89 105 117 140 175 253 267 377 382 410 420 437 443 462 485 504 545
555 572 640 735 855 928 1033 1061 1078 1242 1341 1394 1413 1472 1486 1532 1654 2088 2104 2153 3030 3079
3087 3131 3144 3813
TS127_8
18
E= -1916.36957407 ZPE=   72.52 Gcorr   0.049812000
H -2.642730 0.520470 -1.709699
Co 0.469362 -0.114605 -0.415636
C 0.872557 1.596372 -0.510978
C -0.148638 0.118628 1.343324

O 1.171787 2.705461 -0.578647
O 0.113716 0.654204 2.375902
C 2.066455 -0.987816 -0.284166
O 3.066102 -1.454210 0.028628
C -2.614148 0.053990 0.418578
C -3.187077 -0.102879 -0.992323
H -2.620915 1.100021 0.741272
H -3.243295 -0.492393 1.136480
H -3.119543 -1.142469 -1.322069
H -4.237205 0.200362 -1.015491
C -1.221133 -0.509534 0.572187
O -0.803292 -1.584962 -0.026497
H -0.190339 -0.566784 -2.037589
H 0.386657 -0.101396 -2.245573
FREQS= 37 61 73 82 99 116 190 240 244 264 294 353 376 391 402 440 451 487 519 528 544 628 639 702 749 793 989 1055 1079 1119 1172 1289 1374 1406 1430 1486 1506 1521 1909 2105 2154 3025 3053 3093 3123 3143 3992
TS128_8
18
E= -1916.37002345 ZPE= 73.00 Gcorr 0.050278000
H 3.245755 0.333211 -0.057199
Co -0.112253 0.107949 -0.124827
C -1.201471 -1.208623 -1.156668
C 0.162304 -0.896959 1.335333
O -2.405522 -1.278655 -1.134345
O 0.344831 -1.588599 2.232977
C -1.634493 0.944707 0.430526
O -2.576502 1.494650 0.767248
C 1.569039 -0.794553 -0.916284
C 2.887027 -0.696187 -0.153371
H 1.686106 -0.380174 -1.921452
H 1.293783 -1.847813 -1.026961
H 2.819210 -1.115725 0.855618
H 3.668001 -1.258079 -0.682381
C 0.906163 1.610863 -0.136347
O 1.558394 2.547804 -0.240883
H -0.592788 0.179621 -1.548566
H -0.590241 -1.982736 -1.667830
FREQS= 59 63 77 94 99 110 124 165 206 254 272 325 365 405 414 435 463 487 496 526 533 538 629 721 880 977 996 1005 1051 1234 1277 1368 1431 1485 1510 1513 1773 1952 2121 2140 2187 2890 3031 3055 3093 3105 3127
TS129_8
18
E= -1916.36906851 ZPE= 72.46 Gcorr 0.049932000
H -3.134120 0.774338 0.951678
Co 0.590834 -0.078162 -0.443221
C 0.566170 1.681503 -0.377551
C -0.450571 -0.163182 1.124837
O 0.587420 2.830854 -0.328306
O -0.577609 0.336333 2.200442
C 2.301069 -0.535632 0.001270
O 3.296520 -0.788927 0.510401
C -2.506221 -0.816677 -0.402751
C -3.153045 0.541741 -0.116904
H -3.097138 -1.613092 0.072496
H -2.497107 -1.041928 -1.476253
H -2.634497 1.347448 -0.646299
H -4.196628 0.542059 -0.442593
C -1.094655 -0.982924 0.103450
O -0.258850 -1.854786 -0.386452
H 0.863056 0.082872 -2.259792
H 0.387596 -0.518068 -2.195038
FREQS= 42 54 74 82 99 138 159 236 260 271 343 356 371 388 398 441 465 479 512 525 541 623 634 676 730 794 967 1072 1083 1089 1233 1274 1333 1419 1432 1482 1513 1519 1907 2106 2155 3023 3055 3069 3126 3135 4007
TS130_8
18
E= -1916.37202893 ZPE= 75.18 Gcorr 0.053196000
H -4.248534 -0.692841 0.524019
Co 0.463200 0.244618 -0.121387
C 0.739230 -0.750090 1.287973
C 2.380704 -0.013090 -0.849075
O 0.838881 -1.316535 2.288338
O 3.452173 -0.339674 -0.394001
C -0.356048 1.805375 0.187267
O -0.815005 2.768609 0.623020
C -2.254380 -1.286071 -0.083022

C -3.360333 -0.227501 0.089221
H -1.999883 -1.722182 0.887381
H -2.589673 -2.084769 -0.753673
H -3.041107 0.580064 0.753210
H -3.638731 0.207551 -0.874436
C -1.012571 -0.676220 -0.676784
O -0.693945 -0.703439 -1.853495
H 1.887505 1.008777 -0.430513
H 2.047570 -0.287377 -1.872923
FREQS= 34 62 74 81 91 118 137 188 212 225 247 298 332 365 398 460 503 511 535 577 645 744 810 935 1022 1072 1082 1163 1280 1327 1387 1422 1487 1512 1518 1608 1746 1780 1965 2077 2124 2915 3060 3063 3116 3141 3144
TS131_8
18
E= -1916.36793501 ZPE= 74.89 Gcorr 0.049130000
H 3.929332 0.218853 1.186526
Co -0.504106 0.120800 0.104048
C -0.363442 1.841885 -0.262027
C -0.641534 -1.040877 -1.202924
O -0.307349 2.905730 -0.707677
O -0.693516 -1.651890 -2.182584
C -2.335371 -0.664700 1.226429
O -3.291891 -0.369812 0.538349
C 2.491838 -0.073911 -0.421089
C 3.776461 -0.438299 0.326804
H 2.525228 0.949162 -0.815233
H 2.333039 -0.716112 -1.296229
H 3.737905 -1.466537 0.695089
H 4.639030 -0.342317 -0.338220
C 1.247507 -0.176717 0.424456
O 1.195203 -0.521534 1.590282
H -2.228143 -1.636355 1.740351
H -1.597872 0.147476 1.561562
FREQS= 32 36 49 65 75 77 109 136 186 204 215 266 315 322 367 435 468 498 534 558 649 704 782 955 1021 1086 1109 1213 1252 1277 1359 1426 1459 1469 1508 1514 1752 1770 2062 2115 2481 2988 3054 3064 3086 3139 3144
TS132_8
18
E= -1916.36916975 ZPE= 74.49 Gcorr 0.050661000
H -2.890269 1.406474 1.499537
Co 0.464887 0.017991 -0.060754
C 1.343209 -0.691049 1.420926
C 0.121770 -1.592976 -0.689049
O 2.278842 -0.153420 1.959674
O -0.023725 -2.627898 -1.177832
C 1.917631 0.940723 -0.672308
O 2.812280 1.433783 -1.192992
C -3.770108 0.121591 -0.022611
C -2.636639 0.496704 0.933120
H -3.533355 -0.796361 -0.568110
H -4.696209 -0.041416 0.534384
H -2.455568 -0.278673 1.686189
H -3.939298 0.912476 -0.756518
C -1.338384 0.784408 0.231962
O -1.233372 1.164466 -0.941145
H -0.438193 1.033814 0.978518
H 0.903874 -1.613935 1.872482
FREQS= 33 36 57 73 90 93 147 189 204 223 270 305 322 340 396 450 491 504 520 631 653 764 857 870 991 1015 1108 1130 1270 1326 1367 1375 1429 1465 1509 1515 1696 1788 2086 2103 2145 2821 3018 3065 3082 3137 3150
TS133_8
18
E= -1916.37162484 ZPE= 73.92 Gcorr 0.053381000
H 3.227187 -2.036105 -1.031303
Co -0.053730 0.167481 -0.125806
C -1.290255 -1.063424 -1.029521
C -0.504200 -0.520771 1.507108
O -2.158501 -1.689992 -0.476833
O -0.736740 -0.997430 2.517801
C -1.298298 1.439391 -0.386359
O -2.066543 2.246844 -0.650390
C 1.231471 -1.430158 -0.457880
C 2.675348 -1.106396 -0.838388
H 0.791042 -2.036050 -1.254795
H 1.217748 -2.054803 0.440477
H 2.735294 -0.496723 -1.745587
H 3.215178 -0.576925 -0.046820

C 1.332789 1.244512 0.378267
O 2.225497 1.936296 0.560203
H 0.403671 0.506845 -1.485558
H -1.130225 -1.172910 -2.125274
FREQS= 67 68 83 98 102 115 127 258 263 294 297 369 402 410 416 441 465 503 509 523 535 627 716 766 853 919 990 1017 1050 1264 1297 1358 1424 1478 1512 1514 1787 2041 2138 2157 2193 2870 3027 3055 3090 3097 3123
TS134_8
18
E= -1916.36418315 ZPE= 71.18 Gcorr 0.046044000
H 4.403813 0.226144 -0.486490
Co -0.615925 -0.007805 -0.232632
C -2.218660 -0.663861 -0.617730
C -0.218071 -1.010587 1.278936
O -3.208533 -1.119673 -0.965140
O 0.127298 -1.586292 2.199415
C -1.105238 1.683768 0.362330
O -1.344304 2.769029 0.612926
C 2.345923 -1.389106 -0.718262
C 3.491540 0.790737 -0.283012
H 3.311965 -1.834762 -0.919559
H 1.478867 -2.031973 -0.808622
H 3.424813 1.625749 -0.988933
H 3.557972 1.222938 0.721469
C 2.261566 -0.083604 -0.395188
O 1.162635 0.633483 -0.112039
H -0.595874 0.430627 -1.629854
H -0.190723 -0.964463 -1.250679
FREQS= 41 55 62 76 83 95 142 177 206 252 301 381 387 397 414 450 478 488 501 521 549 605 651 699 763 837 870 890 1004 1045 1070 1303 1411 1440 1484 1504 1682 2082 2099 2159 2188 2215 3045 3103 3144 3182 3263
TS135_8
18
E= -1916.36903991 ZPE= 74.36 Gcorr 0.051106000
H -3.237343 -1.054142 0.199794
Co 0.410418 -0.141848 0.026994
C 0.696344 1.700680 -0.059078
C 0.204286 -0.079200 1.779143
O 1.435354 2.217322 -0.859187
O 0.148121 -0.084359 2.930814
C 2.060774 -0.593301 -0.623833
O 3.108432 -0.958587 -0.910734
C -3.085090 1.040761 -0.423851
C -2.783021 -0.448428 -0.592054
H -2.615386 1.636112 -1.213885
H -4.162233 1.219695 -0.471049
H -3.201263 -0.820922 -1.538911
H -2.724025 1.410860 0.540165
C -1.316930 -0.782399 -0.615656
O -0.844551 -1.916341 -0.459069
H -0.656979 0.004904 -1.264228
H 0.078924 2.340437 0.616663
FREQS= 37 47 61 68 90 95 148 170 219 234 292 316 330 390 400 450 485 493 515 536 632 755 870 893 997 1028 1101 1156 1273 1329 1343 1375 1433 1459 1513 1515 1694 1791 1979 2091 2149 2830 3028 3053 3092 3125 3136
TS136_8
18
E= -1916.36989994 ZPE= 75.07 Gcorr 0.052572000
H -3.489991 -1.548120 -1.665540
Co 0.528727 0.050509 0.168199
C 1.473367 -1.473795 0.189176
C 0.028050 1.705557 0.603789
O 2.014241 -2.488298 0.227606
O -0.406384 2.725931 0.919614
C 1.632180 0.700317 -0.994293
O 2.285942 1.107252 -1.855563
C -2.419010 -0.140259 -0.423161
C -3.693813 -0.697188 -1.008469
H -2.572044 0.716412 0.243474
H -1.259076 -0.815025 1.549549
H -4.391071 -1.028675 -0.227735
H -4.223504 0.065787 -1.594070
C -1.170312 -0.572501 -0.691149
O -0.374059 -0.651740 1.937303
H -0.477766 -0.030350 2.670914
H -1.122879 -1.401731 -1.405010

FREQS=   49   69   74   88   99 102 148 170 195 212 251 309 310 346 387 435 442 456 506 544 586
616 621 636 727 740 937 1036 1060 1075 1249 1347 1416 1493 1497 1589 1634 2073 2082 2144 3020 3064 3070
3075 3117 3568 3848
TS137_8
18
E= -1916.37394171 ZPE=   76.60 Gcorr   0.056655000
H 3.588976 1.807315 0.000956
Co -0.417560 0.092167 0.000008
C -1.040012 -0.497120 1.520245
C -1.463078 1.628092 0.000512
O -1.514758 -1.040119 2.426349
O -0.593157 2.481571 0.001266
C -1.038984 -0.495539 -1.521265
O -1.513109 -1.037592 -2.428258
C 2.573495 -0.099017 0.000536
C 2.562986 1.428632 0.000620
H 3.118488 -0.487511 -0.872606
H 3.117922 -0.487597 0.873999
H 2.055097 1.828440 -0.880453
H 2.054528 1.828368 0.881391
C 1.228804 -0.774979 0.000081
O 1.419040 -2.089052 -0.000306
H -2.539718 1.895184 0.000306
H 0.555442 -2.531579 -0.000592
FREQS=   39   61   74   87 103 112 142 161 228 262 329 358 408 422 494 514 524 543 562 564 642
705 771 882 925 1055 1062 1095 1275 1290 1294 1352 1385 1437 1442 1507 1514 1724 2047 2098 2921 3016 3050
3064 3135 3145 3759
TS138_8
18
E= -1916.36779360 ZPE=   73.14 Gcorr   0.050612000
H 2.882628 1.205025 0.331594
Co -0.263850 0.088628 -0.140687
C -0.826260 -1.678915 -0.914814
C 0.145972 -0.549926 1.486845
O -0.109253 -2.597295 -1.220218
O 0.408393 -1.006979 2.505950
C -1.990441 0.554227 0.157724
O -3.078276 0.870878 0.322655
C 1.696162 -0.325618 -0.709697
C 2.837211 0.119303 0.196656
H 1.810653 0.112109 -1.704387
H 1.669729 -1.408587 -0.821262
H 2.788938 -0.336152 1.190525
H 3.793980 -0.185888 -0.247910
C 0.318647 1.786519 -0.350539
O 0.724123 2.838001 -0.562314
H -0.540927 -0.240489 -1.579349
H -1.928692 -1.809361 -0.936286
FREQS=   55   71   82   89   98 109 112 191 211 255 276 342 367 417 421 427 453 488 497 511 535
540 610 732 845 984 988 1015 1054 1227 1269 1393 1430 1498 1508 1513 1785 1963 2123 2129 2174 2885 3030
3081 3093 3115 3169
TS139_8
18
E= -1916.37007305 ZPE=   74.35 Gcorr   0.052954000
H 3.067176 -0.962540 -0.701409
Co -0.387472 -0.216038 0.130858
C -0.435554 1.727186 0.402218
C -0.296784 -0.304069 -1.620046
O -1.472218 2.359956 0.421455
O -0.247152 -0.223183 -2.768864
C -2.180885 -0.518586 0.401648
O -3.259994 -0.831006 0.613646
C 3.161538 1.027014 0.153698
C 2.808073 -0.458711 0.241549
H 2.912779 1.551497 1.080973
H 4.234020 1.148789 -0.019441
H 3.370348 -0.969784 1.031896
H 2.632904 1.514273 -0.670454
C 1.347774 -0.759566 0.447523
O 0.924909 -1.773491 0.988082
H -0.279523 0.787562 1.336601
H 0.534709 2.225409 0.214584
FREQS=   38   62   80   85 104 113 171 191 218 250 295 331 385 391 429 435 495 526 546 557 572
724 777 939 1014 1050 1081 1159 1216 1278 1342 1373 1430 1454 1514 1517 1708 1722 1784 2102 2167 2944 3028
3057 3084 3132 3135
TS140_8
18

E= -1916.36795500 ZPE= 73.31 Gcorr 0.050948000
H 3.155213 -1.843015 -0.035071
Co -0.126297 0.138841 -0.159718
C -1.230012 -1.259635 -1.001147
C 0.152318 -0.885929 1.301331
O -2.417245 -1.390235 -0.851352
O 0.342750 -1.584856 2.188464
C -1.658053 1.010887 0.304038
O -2.610870 1.597444 0.527604
C 1.539300 -0.742425 -1.021072
C 2.851810 -0.805826 -0.212135
H 1.701036 -0.225291 -1.967565
H 1.208226 -1.752792 -1.269283
H 3.674249 -0.315227 -0.743030
H 2.797481 -0.331618 0.773504
C 0.985832 1.574536 0.001645
O 1.698766 2.468322 -0.049952
H -0.412640 0.604249 -1.538352
H -0.667904 -1.960048 -1.661899
FREQS= 54 73 81 90 96 109 112 146 199 272 312 352 403 411 413 455 482 493 511 520 536 630 667 722 800 904 1005 1014 1042 1220 1235 1362 1434 1494 1511 1517 1795 2006 2140 2152 2194 2843 3042 3080 3099 3106 3144
TS141_8
18
E= -1916.37634923 ZPE= 76.85 Gcorr 0.059700000
H 4.313074 0.679397 0.052286
Co -0.564242 0.151298 -0.285995
C 0.108161 1.765701 -0.066921
C -1.295444 -1.603980 -0.524447
O 0.512524 2.833430 0.095187
O -1.055764 -2.528544 0.265982
C -2.204805 0.680120 0.117471
O -3.220699 0.972099 0.576448
C 2.460395 -0.223251 -0.638374
C 3.348259 0.380587 0.470359
H 2.314119 0.501031 -1.443999
H 2.955608 -1.107981 -1.059746
H 2.882445 1.267072 0.909917
H 3.523579 -0.348578 1.264586
C 1.121835 -0.665221 -0.110487
O 1.229989 -1.670832 0.693469
H -1.998472 -1.844398 -1.352944
H 0.285366 -2.084534 0.817457
FREQS= 57 66 82 98 112 204 220 231 270 285 319 366 392 442 457 502 525 569 578 596 678 814 896 931 1034 1065 1096 1151 1263 1337 1382 1414 1452 1493 1513 1522 1580 1652 2077 2125 2532 2877 3044 3062 3111 3136 3150
TS142_8
18
E= -1916.36724879 ZPE= 73.58 Gcorr 0.051103000
H 2.697763 -0.489481 0.751484
Co -0.149725 0.234366 -0.187629
C -1.187370 -1.190954 -1.051520
C 0.030380 -0.772453 1.326459
O -1.825517 -2.029798 -0.467272
O 0.173170 -1.428445 2.249757
C -1.701594 1.097554 0.105659
O -2.689491 1.668485 0.199390
C 1.424129 -0.810198 -1.053817
C 2.726819 -0.956632 -0.238488
H 1.620820 -0.305341 -2.000040
H 1.042967 -1.801104 -1.310716
H 2.972353 -2.010641 -0.071583
H 3.576330 -0.507126 -0.763761
C 1.030574 1.595926 0.117480
O 1.785595 2.455665 0.130307
H -0.230167 0.886988 -1.508778
H -1.125184 -1.207897 -2.162716
FREQS= 57 68 80 86 92 99 109 125 219 274 296 375 402 416 418 452 475 500 506 520 533 627 714 786 837 912 1002 1014 1049 1224 1253 1357 1435 1491 1511 1518 1786 2032 2139 2157 2192 2867 3042 3079 3098 3105 3141
TS143_8
18
E= -1916.36921331 ZPE= 73.83 Gcorr 0.053102000
H 3.059645 0.430476 -0.023564
Co -0.295182 0.045867 -0.160038
C 1.007101 -1.269050 -0.794593
C 0.310277 -0.125793 1.558155

O 1.606376 -2.032364 -0.078635
O 0.732702 -0.183439 2.617075
C -1.516212 -1.266740 -0.300291
O -2.294307 -2.087431 -0.480604
C 1.189001 1.486144 -0.481746
C 2.617325 1.055878 -0.804655
H 1.203118 2.099483 0.425078
H 0.816079 2.123134 -1.286293
H 2.691257 0.506712 -1.748813
H 3.250922 1.947038 -0.908967
C -1.446033 1.461787 0.019445
O -2.126287 2.379732 -0.036197
H -0.547409 0.274485 -1.594771
H 1.179685 -1.285062 -1.892645
FREQS= 67 72 83 92 104 108 127 232 279 293 305 377 393 403 422 435 476 488 503 522 529
623 705 763 856 916 993 997 1044 1254 1293 1356 1428 1484 1510 1518 1782 2036 2139 2156 2192 2880 3027
3054 3090 3109 3131
TS144_8
18
E= -1916.36828690 ZPE= 74.31 Gcorr 0.052422000
H -4.328919 0.353848 -0.625958
Co 0.536218 -0.045025 -0.328205
C 0.324322 1.608680 -1.130663
C 1.407732 0.586651 1.183608
O 1.435788 1.738124 -1.614214
O 1.975369 0.995578 2.085620
C 0.974711 -1.796134 -0.386969
O 1.161975 -2.915586 -0.544946
C -2.426622 -0.588756 -0.169038
C -3.376179 0.619816 -0.159296
H -2.883385 -1.420493 0.380970
H -2.218145 -0.915752 -1.187478
H -3.570916 0.946727 0.865091
H -2.956915 1.463722 -0.716073
C -1.123329 -0.249758 0.570054
O -1.141024 -0.103767 1.770468
H -0.182810 -0.365771 -1.580585
H -0.477482 2.355617 -1.296040
FREQS= 23 69 72 89 98 120 185 207 221 246 294 317 370 404 428 468 486 509 562 613 662
749 804 856 896 919 1014 1068 1116 1274 1297 1324 1417 1490 1510 1518 1717 1777 2018 2142 2180 2931 3054
3057 3124 3135 3153
TS145_8
18
E= -1916.36066566 ZPE= 71.32 Gcorr 0.045045000
H -4.296074 1.356174 -0.236927
Co 0.727568 0.003480 -0.224008
C 2.350422 0.583177 -0.679168
C 0.193678 1.402938 0.862347
O 3.357534 0.955925 -1.072263
O -0.266150 2.254365 1.466457
C 1.206442 -1.476936 0.789429
O 1.376908 -2.463081 1.335312
C -3.175015 -0.368269 -0.891719
C -3.805317 0.468371 0.181860
H -3.797497 -0.681641 -1.727050
H -1.529925 -1.399595 -1.726748
H -4.578560 -0.088323 0.728632
H -3.057542 0.795860 0.907669
C -1.902118 -0.797567 -0.891812
O -1.045822 -0.604685 0.130691
H 0.734788 -0.698887 -1.517935
H 0.292148 0.611990 -1.486628
FREQS= 30 35 61 84 91 98 129 141 216 238 256 376 389 404 409 418 458 465 497 529 540
593 728 775 856 889 937 955 1055 1068 1164 1295 1402 1446 1497 1506 1703 2055 2086 2157 2180 2206 3021
3064 3082 3134 3173
TS146_8
18
E= -1916.36709300 ZPE= 73.68 Gcorr 0.051911000
H 3.615683 0.133679 -1.054449
Co -0.125466 0.197941 -0.105246
C -0.021375 -1.657342 -0.717464
C -0.533122 -0.331836 1.598269
O -0.397783 -2.611454 -0.084701
O -0.741863 -0.664005 2.669898
C -1.753206 0.300988 -0.857184
O -2.741969 0.367040 -1.431857
C 1.894097 -0.200508 0.248122

C 2.788001 -0.575737 -0.949474
H 1.920975 -0.990396 1.001883
H 2.290071 0.694040 0.733658
H 3.227947 -1.570740 -0.825516
H 2.262865 -0.582412 -1.909709
C 0.234643 1.968285 0.177529
O 0.585454 3.056354 0.230508
H 0.345692 0.562927 -1.452799
H 0.439400 -1.798091 -1.720989
FREQS= 66 69 78 89 98 100 116 152 251 276 306 362 399 408 422 446 461 495 504 523 536
629 724 757 863 916 1005 1023 1041 1241 1245 1355 1430 1491 1507 1517 1790 2047 2136 2156 2193 2869 3043
3075 3101 3103 3130
TS147_8
18
E= -1916.36361818 ZPE= 73.04 Gcorr 0.048595000
H 2.997407 -0.462715 -1.641795
Co -0.055461 0.134435 -0.092483
C -1.404345 -1.022485 -0.933390
C -0.554061 -0.490140 1.552706
O -2.331046 -1.555237 -0.379283
O -0.853287 -0.907164 2.575130
C -1.148424 1.463918 -0.578416
O -1.842102 2.312804 -0.919996
C 1.250263 -1.424640 -0.730855
C 2.751713 -1.166639 -0.839942
H 0.885537 -1.901934 -1.645380
H 1.053401 -2.133845 0.075601
H 3.173957 -0.770858 0.088411
H 3.273471 -2.107101 -1.055813
C 1.319724 1.185446 0.477947
O 2.200445 1.856512 0.770316
H 0.672587 0.005390 -1.356039
H -1.240230 -1.186773 -2.025570
FREQS= 36 53 66 80 93 98 111 124 237 259 286 312 359 404 417 438 442 460 499 518 525
532 624 750 865 908 991 1026 1071 1250 1290 1366 1430 1491 1509 1514 1796 2114 2127 2164 2186 2828 3037
3065 3099 3109 3129
TS148_8
18
E= -1916.36561535 ZPE= 73.37 Gcorr 0.050612000
H 2.507491 -1.350031 0.400162
Co -0.167891 0.172219 -0.158316
C -0.084197 -1.677609 -0.807113
C 0.451525 -0.253072 1.484245
O -0.940045 -2.507889 -0.633227
O 0.874519 -0.535383 2.510253
C -1.962399 -0.032608 0.084461
O -3.095792 -0.145378 0.152684
C 1.787045 0.371750 -0.838852
C 2.842646 -0.659788 -0.380194
H 2.130790 1.373023 -0.570498
H 1.699917 0.360354 -1.925765
H 3.187434 -1.281657 -1.211910
H 3.726105 -0.151042 0.020521
C -0.172076 2.001432 -0.138299
O -0.116011 3.137119 -0.265726
H -0.510897 0.244215 -1.599109
H 0.835575 -1.933158 -1.376226
FREQS= 49 63 73 85 102 108 114 135 210 280 309 342 402 406 420 443 482 486 504 524 536
625 684 717 799 900 1000 1018 1045 1230 1259 1352 1436 1494 1511 1517 1789 2009 2142 2153 2194 2891 3046
3080 3105 3107 3143
TS149_8
18
E= -1916.36861687 ZPE= 73.85 Gcorr 0.053702000
H 3.459206 -1.142168 0.690256
Co -0.200862 0.129638 0.100240
C -0.869641 1.680031 0.531742
C 1.903928 -0.178586 -0.400922
O -1.232919 2.720044 0.855122
O 1.161686 -1.231691 -0.344635
C -1.280345 -0.370556 -1.297681
O -1.967333 -0.718545 -2.142019
C 1.403725 0.923515 -1.136275
C 3.157136 -0.123614 0.440034
H 1.889281 1.886988 -1.017187
H 0.968297 0.741882 -2.111532
H 3.964836 0.390111 -0.087058
H 2.965091 0.412717 1.374442

C -1.211682 -0.994294 1.328430
O -1.075726 -2.183871 1.411445
H 0.421353 0.371061 1.432032
H -1.949197 -0.467266 1.981300
FREQS= 64 70 79 82 101 129 141 187 282 337 368 386 407 420 455 501 510 515 539 610 624 673 783 825 868 888 920 986 1044 1088 1343 1361 1406 1458 1490 1508 1534 1818 1965 2133 2172 2809 3063 3133 3163 3167 3255
TS150_8
18
E= -1916.37148031 ZPE= 75.12 Gcorr 0.056686000
H -1.162902 -2.036837 0.340864
Co 0.310333 -0.034482 -0.072824
C 1.652012 -1.200836 -0.011167
C 1.270241 1.404448 -0.689791
O 2.483892 -1.989510 -0.010273
O 1.756729 2.324260 -1.161547
C 0.002259 0.208849 1.750585
O -0.325656 0.484389 2.810676
C -2.305183 -0.804490 -1.046956
C -1.036076 -1.541681 -0.625873
H -3.183735 -1.457855 -0.952247
H -2.231709 -0.491699 -2.095018
H -0.719771 -2.306725 -1.338025
H 0.033106 -0.541326 -1.412753
C -2.414985 0.429796 -0.158957
O -1.193329 1.126647 -0.169393
H -2.693141 0.108848 0.865544
H -3.203543 1.113788 -0.504895
FREQS= 61 74 88 95 96 106 183 294 330 352 379 405 411 428 453 466 484 527 532 550 593 818 848 905 927 997 1025 1096 1177 1189 1256 1277 1356 1397 1489 1501 1520 2124 2136 2175 2190 2902 3022 3033 3070 3084 3126
TS151_8
18
E= -1916.36835152 ZPE= 74.56 Gcorr 0.053949000
H -4.209238 -0.819951 0.838465
Co 0.523253 -0.050667 0.094588
C 0.174023 -1.770160 -0.136373
C 0.958916 1.675931 0.088248
O -0.013936 -2.903420 -0.229401
O 1.263890 2.779645 0.173121
C 2.270558 -0.447511 0.041545
O 3.395715 -0.689840 0.030471
C -2.442485 -0.474112 -0.387472
C -3.305654 -0.204700 0.864888
H -2.193654 -1.533438 -0.429870
H -3.027451 -0.253424 -1.294799
H -2.745031 -0.446170 1.771237
H -3.617431 0.842810 0.934876
C -1.196954 0.389718 -0.402871
O -1.513138 1.669960 -0.669865
H -2.469780 1.781865 -0.808388
H 0.324079 -0.069442 1.592206
FREQS= 26 52 100 105 118 140 203 210 249 307 326 351 368 427 455 465 540 550 562 579 591 620 643 681 808 920 1025 1070 1102 1241 1296 1325 1392 1423 1503 1510 1525 1832 2089 2104 2157 3002 3052 3119 3144 3159 3693
TS152_8
18
E= -1916.36701565 ZPE= 73.59 Gcorr 0.053196000
H 2.624135 -0.871403 1.317462
Co -0.020565 -0.196382 0.167504
C -1.371819 -1.323007 0.411404
C -0.218402 1.029941 1.459409
O -2.210126 -2.088201 0.576816
O -0.311866 1.656614 2.417582
C -0.872025 0.980600 -1.148126
O -2.037236 0.965223 -1.421116
C 1.889842 -0.270501 -0.648796
C 2.963670 -0.323925 0.431008
H 1.023892 0.923104 -0.824491
H 2.252512 0.205317 -1.566959
H 3.860514 -0.834928 0.060723
H 3.259469 0.677962 0.756119
C 1.049646 -1.429351 -0.904220
O 0.173919 1.614239 -1.523008
H 0.721473 -1.617335 -1.924814
H 1.250267 -2.345945 -0.346920

FREQS=   67   70   76   81 103 105 198 213 215 278 338 396 412 427 453 483 500 517 542 550 614
751 775 834 902 957 1010 1073 1157 1193 1228 1258 1368 1431 1489 1503 1513 1607 1917 2108 2157 3034 3064
3095 3106 3117 3187
TS153_8
18
E= -1916.36068518 ZPE=   71.91 Gcorr   0.046896000
H -1.889073 3.123030 -0.440010
Co 0.475269 -0.190588 -0.290222
C 2.111942 0.518424 -0.482677
C 0.382674 0.138705 1.444257
O 3.177359 0.934980 -0.602323
O 0.382356 0.338244 2.575704
C 0.216782 -1.973252 -0.163397
O 0.100541 -3.111333 -0.072602
C -1.295434 1.049764 -0.688201
C -1.036637 2.473111 -0.203987
H -1.633724 1.053067 -1.730586
H -0.150149 0.205098 -1.654074
H -0.877026 2.512582 0.878220
H -0.153555 2.907854 -0.679502
C -2.282181 0.250903 0.084662
O -3.022256 -0.597236 -0.378028
H 0.490637 -0.410601 -1.839152
H -2.306259 0.491682 1.175136
FREQS=   29   41   55   94   97 119 126 211 228 238 255 293 318 402 436 455 457 488 537 548 553
574 599 716 893 981 1030 1071 1124 1188 1208 1325 1421 1428 1501 1512 1783 2026 2103 2115 2164 2382 2854
3031 3062 3100 3126
TS154_8
18
E= -1916.36106079 ZPE=   72.86 Gcorr   0.047342000
H 2.549658 1.098765 -1.950432
Co -0.142206 -0.151240 -0.031608
C -1.148845 -1.373165 -0.892056
C 1.201593 -1.311646 0.339196
O -1.772066 -2.124016 -1.495455
O 2.067376 -2.028413 0.560329
C -0.720134 0.132909 1.657447
O -1.067401 0.350678 2.729539
C 1.349853 1.368637 -0.137873
C 2.676891 1.165000 -0.864987
H 1.535259 1.502199 0.930273
H 0.818094 2.246032 -0.500094
H 3.336436 2.018248 -0.661863
H 3.209069 0.265729 -0.539882
C -1.503305 1.210790 -0.563916
O -1.317842 2.382629 -0.761401
H -2.537205 0.806857 -0.674480
H 0.511400 0.343496 -1.241087
FREQS=   20   55   59   73   91   95 109 148 202 269 286 337 356 401 425 447 450 460 493 505 528
536 604 712 771 898 991 1016 1061 1230 1272 1385 1428 1503 1507 1514 1807 2107 2124 2166 2188 2830 3034
3080 3099 3115 3178
TS155_8
18
E= -1916.37253443 ZPE=   76.53 Gcorr   0.058841000
H 2.819194 1.708821 1.081592
Co -0.090482 -0.094732 -0.028441
C 0.226626 -1.511993 0.977228
C -1.212948 1.431253 0.494411
O 0.506307 -2.442262 1.589719
O -1.949288 1.926248 1.292641
C -1.458234 -0.871199 -0.740073
O -2.306180 -1.400052 -1.307840
C 2.319099 0.752472 -0.849913
C 2.325586 0.814967 0.686826
H 1.920083 1.695562 -1.235958
H 3.354699 0.677161 -1.210819
H 1.298276 0.877755 1.124363
H 2.795252 -0.068816 1.128493
C 1.442252 -0.410896 -1.318490
O -0.583413 1.680673 -0.581540
H 1.112333 -0.303916 -2.354606
H 1.949483 -1.373285 -1.208919
FREQS=   64   84   95 101 104 134 162 177 233 296 344 364 411 426 464 515 534 542 556 636 695
734 804 885 966 1015 1041 1144 1162 1219 1290 1362 1434 1463 1481 1510 1530 1970 2111 2145 2809 3018 3072
3073 3092 3130 3134
TS156_8
18

E= -1916.37028134 ZPE= 75.79 Gcorr 0.057125000
H 3.858127 1.761648 1.047937
Co -0.782679 -0.025838 -0.218289
C -0.963245 1.694040 -0.289896
C 1.112399 -1.268991 -0.228851
O -1.047244 2.833766 -0.402109
O -0.005433 -1.878543 -0.332698
C -2.372679 -0.538540 0.509759
O -3.369206 -0.895290 0.947911
C 2.172529 1.098598 -0.166954
C 3.025855 1.053631 1.117196
H 1.808250 2.120188 -0.315867
H 3.442609 0.055237 1.278311
H 2.819526 0.871241 -1.027129
H 2.423919 1.315854 1.992605
C 1.018474 0.147391 -0.129248
O 2.228120 -2.000889 -0.235084
H -1.577542 -0.084625 -1.473061
H 1.947569 -2.931038 -0.305191
FREQS= 42 48 79 97 104 161 189 273 300 314 367 386 440 456 485 548 552 584 615 657 661 725 737 790 908 964 1066 1145 1150 1250 1296 1352 1417 1477 1500 1510 1521 1538 1915 2125 2159 3012 3047 3080 3120 3131 3726
TS157_8
18
E= -1916.36249830 ZPE= 73.16 Gcorr 0.049368000
H 3.477186 1.229080 -1.000997
Co -0.254989 0.078955 -0.157166
C 0.530385 -1.603330 -0.780316
C 0.413450 -0.226044 1.519324
O 1.013522 -2.467924 -0.093922
O 0.797439 -0.435057 2.576402
C -1.825803 -0.765021 -0.279994
O -2.835556 -1.300857 -0.390392
C 1.356547 0.910064 -1.299709
C 2.627697 0.813548 -0.441860
H 1.159463 1.956654 -1.543142
H 1.544812 0.395060 -2.248725
H 2.535912 1.379715 0.489190
H 2.874934 -0.219684 -0.185518
C -0.893168 1.756421 0.164743
O -1.267878 2.833863 0.269562
H -0.158204 0.486316 -1.561152
H 0.535718 -1.712950 -1.892507
FREQS= 45 64 67 75 91 94 112 127 248 260 302 316 358 403 420 427 463 478 488 521 528 550 627 716 829 906 968 1070 1084 1297 1319 1368 1426 1485 1509 1516 1791 2114 2127 2163 2190 2816 3024 3047 3101 3110 3140
TS158_8
18
E= -1916.36963850 ZPE= 76.41 Gcorr 0.056667000
H -2.936160 0.246839 0.867635
Co 0.261569 -0.005120 -0.086346
C 0.392366 1.712888 -0.754985
C 2.045334 0.020392 0.124755
O -0.522551 2.611170 -1.068771
O 3.188449 0.049492 0.237883
C 0.281390 -1.740502 -0.535643
O 0.252078 -2.847693 -0.848980
C -1.661902 -0.178303 -0.870505
C -2.789070 -0.523786 0.103171
H -3.742962 -0.641779 -0.428641
H -1.641770 -0.929098 -1.665467
H -2.595697 -1.465681 0.627544
H -1.920355 0.738029 -1.417800
C -0.306900 0.149281 1.614601
O -0.660409 0.325726 2.694297
H -1.402669 2.195218 -1.021130
H 1.349407 2.245344 -0.834587
FREQS= 30 66 85 94 103 121 161 238 265 302 321 350 383 422 438 448 493 501 516 536 557 631 708 716 965 990 1010 1048 1220 1261 1271 1338 1428 1497 1511 1513 1526 2095 2102 2149 3018 3025 3057 3077 3089 3110 3666
TS159_8
18
E= -1916.35516274 ZPE= 73.92 Gcorr 0.042313000
H 0.185096 -3.603516 0.801295
Co 0.364875 0.282894 -0.198690
C -0.936049 0.785583 -1.610839
C 0.591459 1.914973 0.390489

O -0.938442 -0.481875 -1.269052
O 0.668861 2.964585 0.858924
C 1.972619 -0.221170 0.028207
O 3.097270 -0.497461 0.077896
C 0.472596 -1.469189 0.905881
C 0.539287 -2.787854 0.157920
H -0.549053 -1.207068 1.202685
H 1.101815 -1.464248 1.797512
H -0.084143 -2.769483 -0.738411
H 1.565372 -3.029633 -0.137145
C -3.679272 -0.761494 0.384937
O -3.278529 0.159600 0.922169
H -0.477291 1.082973 -2.561549
H -1.750540 1.428939 -1.258835
FREQS= 15 17 33 36 64 77 98 110 120 153 167 214 267 371 379 419 431 463 513 572 587 639 718 776 909 985 1055 1073 1194 1208 1266 1316 1421 1470 1504 1517 1567 2070 2124 2198 3020 3035 3056 3099 3105 3128 3150
TS160_8
18
E= -1916.36438295 ZPE= 74.79 Gcorr 0.051784000
H 4.788471 0.035185 -0.343027
Co -0.420303 0.008180 -0.020382
C -1.405299 1.469906 0.487023
C -1.617279 -1.346372 -0.412902
O -1.911861 2.485692 0.647674
O -2.280952 -2.168626 -0.855045
C 0.147120 -0.544813 1.554260
O 0.557166 -0.910821 2.562565
C 2.648820 -0.061253 0.075073
C 3.836778 0.195816 -0.859439
H 2.702803 -1.080873 0.487487
H 2.719244 0.584143 0.965291
H 3.806028 -0.474192 -1.724409
H 3.825758 1.224682 -1.232788
C 1.299103 0.135611 -0.539192
O -0.807692 0.488618 -1.843746
H 1.322420 0.457538 -1.587794
H -1.725289 0.780372 -1.934975
FREQS= 20 59 70 95 100 102 172 178 242 263 304 322 327 391 413 417 437 441 512 540 541 582 604 733 856 948 983 1074 1081 1101 1259 1282 1374 1425 1435 1512 1514 2116 2122 2166 3002 3022 3048 3081 3121 3123 3808
TS161_8
18
E= -1916.35933161 ZPE= 71.68 Gcorr 0.046854000
H -4.147990 0.747217 0.959918
Co 0.684055 -0.002264 -0.235276
C 2.480497 0.004470 -0.286493
C 0.413434 1.531923 0.688615
O 3.617743 0.006419 -0.413143
O 0.222393 2.548726 1.179733
C 0.421157 -1.515728 0.721874
O 0.233539 -2.521984 1.235901
C -2.187003 0.010875 0.454805
C -3.685781 0.020671 0.282343
H -1.830229 0.018446 1.479960
H -4.136236 -0.955863 0.504790
H -4.006901 0.300788 -0.729155
H -2.571380 -0.094887 -1.935849
C -1.269182 -0.016169 -0.531345
O -1.605140 -0.050356 -1.858573
H 0.712082 0.676515 -1.536858
H 0.724172 -0.709788 -1.520503
FREQS= 48 49 60 75 92 97 107 200 234 268 314 340 355 397 402 448 472 478 517 522 553 575 644 697 784 832 858 952 1039 1057 1137 1303 1352 1429 1490 1513 1671 2086 2093 2147 2163 2187 3017 3068 3090 3188 3742
TS162_8
18
E= -1916.36555506 ZPE= 75.35 Gcorr 0.053123000
H 2.393362 -0.959917 2.892435
Co -0.422519 -0.048391 -0.269678
C -0.663103 1.761848 -0.564524
C -0.365761 -1.707586 -0.891733
O -0.778614 2.853216 -0.085740
O -0.360845 -2.715648 -1.451228
C -1.911781 -0.166610 0.667524
O -2.767544 -0.291234 1.431162
C 2.253256 0.068990 0.995109

C 1.703184 -0.894480 2.046610
H 2.380189 1.070921 1.435426
H 3.252977 -0.232714 0.655116
H 0.734730 -0.558376 2.425694
H 1.577198 -1.900664 1.636791
C 1.384162 0.294782 -0.210214
O 2.134713 0.793708 -1.187570
H -0.731625 1.620069 -1.717135
H 1.579772 1.005235 -1.956628
FREQS= 24 52 65 84 87 102 134 192 233 244 318 374 381 416 426 461 516 520 552 567 588 696 779 807 932 1045 1087 1097 1202 1271 1287 1357 1386 1432 1445 1510 1514 1845 2077 2124 2354 3012 3058 3060 3130 3137 3728
TS163_8
18
E= -1916.36440512 ZPE= 75.03 Gcorr 0.052398000
H -4.696872 -0.241201 -0.188503
Co 0.387048 -0.004162 -0.068460
C -0.218084 1.172569 1.106385
C 1.187287 -1.221278 1.090323
O -0.639480 1.935289 1.853759
O 1.623320 -2.102013 1.673715
C 1.816401 0.957494 -0.690240
O 2.656585 1.499549 -1.252050
C -2.638758 0.415367 -0.487649
C -3.704503 -0.686618 -0.305921
H -2.895957 0.994453 -1.391943
H -2.677474 1.116776 0.351363
H -3.728664 -1.358402 -1.169175
H -3.495837 -1.288297 0.583694
C -1.281009 -0.150032 -0.729400
O 0.861244 -1.356266 -1.351038
H -1.274489 -0.887815 -1.540044
H 1.337643 -0.960634 -2.093051
FREQS= 27 63 69 84 100 120 136 170 199 260 315 328 361 376 424 431 436 444 514 530 540 581 672 808 865 941 966 1022 1032 1155 1241 1260 1381 1421 1483 1509 1518 2115 2131 2174 2981 3051 3081 3096 3127 3131 3818
TS164_8
18
E= -1916.36018521 ZPE= 72.30 Gcorr 0.048186000
H 0.957767 0.683898 2.368282
Co -0.726935 -0.059682 -0.549045
C -1.447473 -1.276516 0.461653
C 0.797586 -0.961968 -0.753574
O -1.975812 -2.073235 1.106546
O 1.478294 -1.934264 -0.860796
C -1.547668 1.399967 0.160496
O -1.989940 2.313562 0.696032
C 2.386269 0.584396 0.719162
C 1.819717 0.087850 2.054544
H 2.730857 1.621334 0.784302
H 3.247771 -0.035406 0.434897
H 1.510105 -0.958845 1.993045
H 2.582474 0.165137 2.834263
C 1.432478 0.523437 -0.456689
O 1.273738 1.436366 -1.254358
H -1.087316 -0.370317 -2.134037
H -1.250105 0.423190 -2.069493
FREQS= 31 49 60 83 96 118 165 190 232 253 276 317 335 378 417 442 486 496 521 536 555 571 635 702 805 926 956 1046 1073 1150 1282 1339 1429 1468 1477 1511 1515 1697 1881 2097 2148 3040 3058 3102 3130 3139 3358
TS165_8
18
E= -1916.36688639 ZPE= 76.17 Gcorr 0.055563000
H -3.210423 1.507543 -1.313565
Co 0.290663 -0.067100 -0.013929
C -0.346988 -0.170482 1.646957
C 1.982284 -0.615054 -0.307266
O -0.713291 -0.295709 2.733897
O 3.058103 -0.995173 -0.470636
C 0.748204 1.663578 0.008437
O 0.998607 2.785618 -0.020985
C -1.199552 0.665311 -1.335627
C -2.456078 1.134269 -0.606524
H -1.454488 -0.094671 -2.077027
H -0.764976 1.490694 -1.903703
H -2.945658 0.334612 -0.031111
H -2.244050 1.937531 0.106146

C -0.340339 -1.667596 -0.595737
O -1.553566 -2.178439 -0.503020
H 0.273988 -2.422796 -1.100298
H -2.146294 -1.531729 -0.079847
FREQS= 49 55 72 90 93 113 120 192 237 264 303 328 405 419 446 449 476 496 511 549 560
671 713 744 965 973 1008 1039 1212 1248 1270 1340 1429 1491 1496 1502 1512 2075 2093 2139 2999 3043 3083
3084 3100 3127 3702
TS166_8
18
E= -1916.36053329 ZPE= 74.33 Gcorr 0.049546000
H 2.629551 1.662321 -0.054420
Co -0.326576 0.136721 -0.138658
C -0.865225 -1.924623 -1.168237
C 0.680636 -0.375034 1.163568
O 0.050512 -2.700653 -1.321610
O 1.075115 -0.596170 2.250310
C -1.852720 0.180862 0.707545
O -2.833411 0.236892 1.308668
C 1.802851 -0.348143 -0.218355
C 2.924906 0.624543 0.122964
H 1.491309 -0.234908 -1.261033
H 2.093467 -1.394942 -0.120895
H 3.216903 0.528298 1.172098
H 3.804179 0.412378 -0.494597
C -0.320063 1.784604 -0.895825
O -0.287210 2.905469 -1.153675
H -0.798983 -0.844347 -1.565465
H -1.881240 -2.237834 -0.871427
FREQS= 28 53 62 81 91 102 111 132 150 210 255 269 346 358 369 421 440 462 494 546 588
603 710 784 974 1018 1069 1203 1239 1265 1279 1426 1454 1470 1504 1517 1790 1969 2090 2138 2412 2990 3051
3076 3119 3131 3146
TS167_8
18
E= -1916.35958007 ZPE= 72.87 Gcorr 0.048599000
H -2.593128 -1.608862 -1.535829
Co 0.229349 0.010857 -0.008527
C -1.061998 1.379089 0.589073
C 0.942394 1.346221 -0.962336
O -2.001847 1.789341 -0.041137
O 1.375530 2.194179 -1.603008
C 1.189369 -1.345332 -0.752588
O 1.689103 -2.230054 -1.280880
C -1.420242 -1.326591 0.290299
C -2.728423 -1.278534 -0.500336
H -1.627674 -1.134423 1.344838
H -0.972545 -2.318486 0.213876
H -3.464771 -1.944691 -0.032973
H -3.151395 -0.271669 -0.522809
C 0.982822 -0.056669 1.647421
O 1.421307 -0.098666 2.706356
H -0.879702 1.782560 1.615555
H -0.799478 -0.345056 -0.972272
FREQS= 56 60 63 82 86 91 119 130 171 257 311 329 353 404 422 427 445 458 494 508 527
537 611 712 826 899 992 1005 1044 1240 1284 1378 1427 1478 1506 1522 1798 2111 2127 2170 2226 2801 3033
3077 3097 3132 3150
TS168_8
18
E= -1916.34897907 ZPE= 70.57 Gcorr 0.038258000
H -3.411120 -0.197075 -0.473636
Co 0.544970 0.106779 -0.048489
C 1.725065 1.251183 -0.785347
C 0.689204 0.040316 1.756010
O 2.421658 2.035032 -1.251394
O 0.730355 0.032938 2.901882
C 1.281498 -1.486712 -0.425379
O 1.702113 -2.509204 -0.733290
C -2.165589 1.521896 -0.062844
C -3.508718 0.883135 -0.345664
H -2.144037 2.588021 0.176901
H -1.550018 -1.656784 -0.212382
H -4.196747 1.071662 0.485413
H -3.953764 1.315740 -1.249018
C -1.008292 0.913266 -0.054702
O -2.236363 -2.327781 -0.339404
H -2.171917 -2.547866 -1.277756
H -0.307696 -0.043113 -1.255113

FREQS= 20 46 56 65 83 89 91 108 132 140 173 196 239 279 325 344 393 418 438 462 475
480 504 545 557 618 685 728 965 1056 1064 1225 1423 1507 1514 1654 1783 2010 2121 2128 2172 3040 3102
3119 3147 3766 3883
TS169_8
18
E= -1916.35573039 ZPE= 73.80 Gcorr 0.045035000
H 1.208256 -3.061820 -1.188915
Co -0.535393 0.246334 0.180380
C 0.611335 1.340200 1.379231
C -1.466303 1.524826 -0.614680
O 0.950372 0.070781 1.263626
O -2.031385 2.336231 -1.203497
C -1.784190 -0.909263 0.328453
O -2.652386 -1.652856 0.494324
C 0.010565 -1.278056 -1.034587
C 0.650776 -2.507302 -0.421919
H 0.753090 -0.582383 -1.449060
H -0.710232 -1.530929 -1.814740
H 1.345941 -2.239829 0.376445
H -0.098303 -3.191311 -0.010576
C 3.202758 0.652994 -0.670027
O 4.142431 0.151224 -0.272262
H 0.120866 1.677446 2.299600
H 1.214099 2.094374 0.860637
FREQS= 25 32 51 58 74 91 96 113 128 148 157 225 265 381 388 411 431 486 500 553 577
616 682 768 894 978 1051 1052 1195 1204 1260 1296 1419 1467 1500 1516 1556 2084 2131 2213 3022 3023 3030
3097 3108 3121 3151
TS170_8
18
E= -1916.36307298 ZPE= 73.72 Gcorr 0.052525000
H -3.070390 1.672227 -0.224206
Co 0.344691 -0.075606 -0.217611
C 0.435234 1.600255 0.225399
C 0.828709 -0.868216 1.351624
O 0.491178 2.725917 0.446244
O 1.183245 -1.298571 2.353102
C 2.123527 -0.382138 -0.976577
O 3.130934 0.266340 -0.857197
C -2.926910 -0.494814 -0.348571
C -3.420107 0.788399 0.316603
H -3.288515 -1.387004 0.182157
H -3.297246 -0.588799 -1.378801
H -4.512864 0.813161 0.334374
H -3.062283 0.859978 1.347625
C -1.435055 -0.619761 -0.413849
O -0.831032 -1.542709 -0.964076
H 2.127144 -1.307712 -1.612731
H 0.370507 0.629341 -1.525290
FREQS= 52 59 74 79 94 143 168 182 216 308 366 386 401 438 445 458 486 518 535 539 591
640 738 821 877 942 1040 1075 1128 1272 1336 1342 1433 1439 1511 1514 1673 1802 1975 2126 2159 2754 3029
3060 3068 3135 3137
TS171_8
18
E= -1916.36229680 ZPE= 73.08 Gcorr 0.051965000
H -3.326173 -0.089039 -0.276882
Co 0.097489 0.102569 -0.284179
C -1.129733 1.383226 -0.292883
C 0.241642 -0.317520 1.657546
O -1.908237 2.219560 -0.387444
O 1.239492 -0.680530 2.229164
C 1.511415 1.228310 -0.164598
O 2.408705 1.933374 -0.100972
C -1.479850 -1.274721 -0.250734
C -2.813744 -0.828184 0.345197
H -2.710888 -0.406942 1.352007
H -1.116217 -2.136521 0.315818
H -1.623556 -1.592303 -1.285144
H -3.481239 -1.694745 0.435934
C 1.013195 -1.321894 -0.836085
O 1.555827 -2.240456 -1.248866
H -0.078180 0.332916 -1.783490
H -0.719800 -0.253623 2.228886
FREQS= 57 74 89 100 104 119 132 194 265 301 305 391 411 418 423 440 481 525 545 558 563
584 641 665 711 865 983 1006 1042 1248 1293 1362 1427 1481 1510 1516 1784 1813 2134 2154 2191 2781 3030
3066 3086 3115 3139
TS172_8
18

E= -1916.36229181 ZPE= 73.23 Gcorr 0.052110000
H -3.603282 1.521519 0.096724
Co 0.216634 0.112118 -0.210446
C 1.009034 1.697133 -0.131853
C 0.170671 -0.412408 1.708277
O 1.480988 2.741463 -0.152409
O -0.135300 -1.497571 2.135400
C 1.735607 -0.840004 -0.430307
O 2.701984 -1.434744 -0.578563
C -1.476724 1.244657 0.294550
C -2.845344 0.786127 -0.203574
H -1.298696 2.253689 -0.083845
H -1.480929 1.310025 1.386521
H -2.886615 0.709788 -1.294744
H -3.148227 -0.177355 0.216692
C -0.882355 -1.175831 -0.766698
O -1.573286 -1.985543 -1.182649
H 0.103317 0.556868 -1.666881
H 0.404903 0.411401 2.430981
FREQS= 53 58 86 101 111 117 139 228 267 301 313 393 405 418 433 447 482 517 546 550 570
589 641 664 717 876 994 1019 1048 1267 1305 1365 1422 1480 1514 1515 1784 1816 2137 2150 2192 2773 3030
3061 3094 3109 3132
TS173_8
18
E= -1916.35646962 ZPE= 70.76 Gcorr 0.046476000
H -4.394334 -0.000025 1.175427
Co 0.764647 0.000003 -0.139286
C 1.150134 0.000005 1.624201
C 0.945043 -1.676902 -0.685690
O 1.367109 0.000006 2.750391
O 1.180019 -2.749767 -1.025523
C 0.945027 1.676908 -0.685694
O 1.179993 2.749774 -1.025529
C -2.248424 -0.000008 0.869252
C -3.684841 -0.000003 0.343207
H -2.052002 0.868927 1.513538
H -2.052005 -0.868950 1.513530
H -3.876963 0.881472 -0.273736
H -3.876954 -0.881454 -0.273774
C -1.173986 -0.000006 -0.182317
O -1.494679 -0.000013 -1.401136
H 2.249004 0.000009 -0.400867
H -0.099465 -0.000008 -1.576769
FREQS= 48 55 72 84 88 101 123 201 203 277 350 370 384 423 460 465 498 507 538 541 564
678 680 693 775 845 945 1027 1064 1113 1271 1362 1423 1438 1450 1508 1512 1619 1912 2110 2123 2171 3030
3056 3063 3136 3142
TS174_8
18
E= -1916.36150526 ZPE= 74.73 Gcorr 0.051585000
H -4.727475 -0.716399 0.000116
Co 0.365205 0.043680 0.000009
C 1.414842 -0.398016 1.467066
C 1.414668 -0.398319 -1.467096
O 1.895354 -0.795007 2.425629
O 1.895047 -0.795554 -2.425625
C 0.627290 1.805250 -0.000187
O 0.753631 2.945201 -0.000334
C -2.549848 -0.582025 0.000212
C -3.946544 0.050525 -0.000028
H -2.438340 -1.245466 0.872581
H -2.438220 -1.245866 -0.871832
H -4.093478 0.678137 0.884936
H -4.093373 0.677694 -0.885324
C -1.405570 0.391193 0.000106
O 0.108280 -1.840799 0.000151
H -0.826082 -2.068801 0.000167
H -1.791101 1.418966 0.000114
FREQS= 36 51 66 87 96 101 140 190 212 261 300 304 332 382 385 408 415 438 499 521 548
592 642 732 864 923 957 1072 1074 1083 1273 1306 1387 1426 1459 1514 1514 2128 2140 2179 2993 3017 3045
3051 3120 3120 3894
TS175_8
18
E= -1916.35810634 ZPE= 71.83 Gcorr 0.048429000
H -2.869765 -0.017234 2.349066
Co 0.569788 -0.001864 0.313741
C 2.356315 -0.001201 0.167018
C 0.212113 1.513486 -0.609647

O 3.501579 -0.001422 0.153399
O -0.025715 2.521419 -1.098187
C 0.211537 -1.506116 -0.627235
O -0.027277 -2.507830 -1.127961
C -1.369392 -0.005234 0.788541
C -1.777292 -0.016486 2.239153
H 0.739206 0.652807 1.623534
H 0.739813 -0.670855 1.616179
H -1.389465 -0.899384 2.761769
H -1.389231 0.858017 2.775510
C -2.322456 0.001897 -0.146149
O -2.050493 0.011754 -1.501318
H -3.382380 0.000036 0.113617
H -2.882166 0.017513 -1.988236
FREQS= 39 61 85 97 101 109 167 179 237 274 288 321 380 400 420 438 447 486 496 520 520 554 585 618 824 855 856 1002 1063 1101 1153 1270 1359 1428 1484 1510 1733 2070 2080 2141 2160 2185 3014 3076 3081 3125 3860
TS176_8
18
E= -1916.35896485 ZPE= 74.62 Gcorr 0.049446000
H 2.797416 -1.851950 0.053317
Co -0.325765 0.066640 -0.075519
C -1.671656 -1.535104 -1.195058
C -0.457381 -0.757822 1.474076
O -2.716576 -1.163629 -0.700685
O -0.606738 -1.121927 2.558445
C -1.001666 1.676790 -0.324415
O -1.498180 2.716676 -0.297963
C 1.942335 -0.193566 -1.074354
C 3.121806 -1.069334 -0.638674
H 2.258222 0.568680 -1.794200
H -1.376859 -2.596976 -1.269294
H 3.587682 -1.547772 -1.504895
H 3.868812 -0.454856 -0.131089
C 1.367947 0.586188 0.149782
O 2.030206 1.248694 0.894726
H -0.998423 -0.792267 -1.717831
H 1.180790 -0.805558 -1.581347
FREQS= 35 53 62 67 83 88 103 112 160 182 236 252 324 367 376 439 450 485 521 539 572 675 746 832 978 1058 1091 1156 1248 1274 1303 1421 1458 1495 1507 1516 1772 1860 2077 2131 2663 2989 3012 3058 3089 3131 3149
TS177_8
18
E= -1916.36905197 ZPE= 77.59 Gcorr 0.059566000
H 2.561406 2.828356 -0.969266
Co -0.738523 0.126368 -0.132958
C 0.566430 -1.199487 0.120185
C -1.978308 -1.038242 -0.121263
O 0.759788 -2.385464 -0.051632
O -2.816960 -1.828742 -0.127953
C -1.810629 1.548539 0.090014
O -2.523214 2.369923 0.466836
C 1.624722 0.925003 -0.456433
C 2.682034 2.010688 -0.253437
H 0.628886 1.418535 -0.279669
H 1.636520 0.574063 -1.494852
H 2.634138 2.425869 0.758109
H 3.671533 1.568314 -0.393388
C 1.762975 -0.283643 0.489135
O 2.996212 -0.931134 0.346915
H 1.627463 0.059547 1.530014
H 2.770221 -1.860416 0.156386
FREQS= 41 59 80 100 111 148 204 224 279 293 312 338 392 400 419 471 476 572 585 612 731 801 885 915 1029 1072 1113 1175 1231 1317 1386 1402 1419 1427 1450 1508 1514 1776 2087 2143 2739 2966 3056 3072 3130 3145 3667
TS178_8
18
E= -1916.36637678 ZPE= 76.93 Gcorr 0.057022000
H 3.773067 0.298690 -1.142364
Co -0.534426 -0.000017 -0.232341
C 0.542536 0.999615 0.775439
C -1.475664 -1.559212 -0.224113
O 0.697034 1.934536 1.508332
O -2.146549 -2.439100 0.090444
C -1.792634 1.147962 -0.367085
O -2.646178 1.921110 -0.458511
C 1.818096 -0.594260 -0.844507

C 2.771566 0.347120 -1.582667
H 0.858823 -0.618055 -1.415589
H 2.183895 -1.625639 -0.870180
H 2.852557 0.074178 -2.638276
H 2.430800 1.385863 -1.530254
C 1.637341 -0.198667 0.633025
O 1.295385 -1.358619 1.387948
H 1.155579 -1.089243 2.306807
H 2.569804 0.255899 0.996794
FREQS= 36 58 73 85 108 111 161 181 248 267 319 346 356 392 433 455 474 484 579 583 626
739 850 908 1035 1041 1102 1140 1227 1274 1306 1358 1401 1434 1504 1511 1531 1852 2076 2134 2806 3013 3050
3090 3122 3132 3806
TS179_8
18
E= -1916.35731048 ZPE= 72.86 Gcorr 0.048178000
H 4.026999 0.000052 -1.163337
Co -0.526751 -0.000014 -0.184649
C -0.244576 1.773065 -0.196528
C -0.244548 -1.773077 -0.196438
O -0.009672 2.889501 -0.154097
O -0.009634 -2.889513 -0.154017
C -2.356011 -0.000037 -0.106315
O -3.495500 -0.000059 -0.114394
C 2.432322 -0.000037 0.296796
C 3.890337 -0.000003 -0.078351
H -0.711644 -0.000118 -1.664625
H 2.240494 -0.000101 1.370596
H 4.400654 0.880108 0.332466
H 4.400676 -0.880142 0.332379
C 1.425342 0.000008 -0.579159
O -0.446004 0.000191 1.741802
H 0.475810 0.000040 2.025615
H 1.658581 0.000063 -1.641958
FREQS= 30 45 72 89 94 104 124 187 192 233 253 304 363 400 408 441 451 474 521 537 546
568 661 710 760 829 896 959 1015 1063 1090 1250 1330 1420 1492 1497 1677 1946 2163 2181 2211 3030 3083
3118 3135 3164 3853
TS180_8
18
E= -1916.35537661 ZPE= 70.95 Gcorr 0.046487000
H -2.681793 -0.000788 1.859061
Co 0.493206 0.000164 -0.584829
C 0.100024 -1.730133 -0.599621
C 0.099166 1.730275 -0.599183
O -0.188510 -2.839056 -0.625508
O -0.189905 2.839065 -0.624775
C 2.090230 0.000471 0.290097
O 3.130375 0.000678 0.765214
C -3.029313 -0.000133 -0.318460
C -2.152824 -0.000477 0.899009
H -3.685226 -0.880254 -0.340600
H -3.685145 0.880060 -0.340170
H -0.757715 -0.000990 2.865776
H -2.439969 0.000062 -1.238322
C -0.811565 -0.000424 0.939690
O -0.120047 -0.000768 2.132308
H -0.083862 0.000149 -1.944035
H 1.187536 0.000502 -1.918430
FREQS= 33 58 82 89 91 106 166 183 241 293 317 319 352 390 426 432 455 478 503 510 542
557 567 628 694 799 806 941 1063 1120 1128 1250 1363 1429 1500 1506 1690 1972 2068 2133 2157 2190 3022
3065 3069 3131 3717
TS181_8
18
E= -1916.36367213 ZPE= 75.95 Gcorr 0.054789000
H -0.833978 3.745975 -0.379288
Co 0.150474 -0.198933 -0.042510
C 1.098551 -1.013896 -1.332370
C 1.472996 0.519109 0.921958
O 1.681439 -1.570206 -2.156418
O 2.303219 1.034920 1.529740
C -0.419032 -1.213390 1.309846
O -0.785531 -1.896615 2.162785
C -0.037908 1.768924 -0.818066
C -0.827588 2.736158 0.055743
H -0.437179 1.746265 -1.836870
H 0.998163 2.104242 -0.920198
H -1.870224 2.424873 0.180686
H -0.398100 2.817887 1.059173

C -1.461371 -0.204513 -0.851381
O -2.639924 -0.394932 -0.265332
H -3.372490 -0.357605 -0.902524
H -1.576500 -0.050120 -1.933790
FREQS= 40 54 63 83 96 110 116 222 233 297 325 329 382 437 441 459 467 488 530 551 561 603 685 714 936 976 1001 1052 1216 1253 1264 1293 1426 1483 1499 1504 1512 2080 2092 2140 3014 3031 3056 3083 3092 3115 3723
TS182_8
18
E= -1916.36052630 ZPE= 75.16 Gcorr 0.052391000
H -3.745415 -1.139122 -1.465680
Co 0.358641 -0.009276 -0.052571
C 0.286883 -1.162231 1.318533
C 2.158027 -0.007767 -0.419171
O 0.119587 -1.802168 2.257075
O 3.301391 0.223302 -0.523600
C 0.106089 1.598919 0.566702
O -0.084357 2.637694 1.026282
C -2.622348 0.484787 -0.511768
C -3.714653 -0.606087 -0.510249
H -2.849356 1.199882 -1.320741
H -2.649676 1.049787 0.425551
H -3.533753 -1.339127 0.281279
H -4.697051 -0.157228 -0.337883
C -1.269976 -0.071094 -0.780356
O 1.400112 -1.004605 -1.520856
H -1.244877 -0.687414 -1.693618
H 1.478806 -0.529268 -2.362831
FREQS= 25 45 73 79 89 110 130 153 230 312 316 332 373 398 425 468 493 519 546 563 614 631 673 792 837 936 947 1008 1035 1165 1247 1273 1380 1419 1478 1508 1517 1995 2103 2144 2988 3028 3051 3085 3128 3130 3783
TS183_8
18
E= -1916.35529631 ZPE= 70.99 Gcorr 0.047201000
H -2.508806 -0.371170 1.299093
Co 0.639227 -0.258529 -0.055654
C 0.249168 -0.810383 1.618630
C 0.137938 -1.576186 -1.131105
O -0.006006 -1.155420 2.682544
O -0.097618 -2.493454 -1.782587
C 1.841808 1.031480 0.129504
O 2.699226 1.781339 0.284214
C -2.071233 1.424703 0.125857
C -3.001205 0.288448 0.579864
H -2.585890 2.106480 -0.561539
H -1.752825 2.005249 1.002913
H -3.892008 0.703517 1.058702
H -3.328308 -0.317316 -0.270507
C -0.818844 0.930185 -0.544479
O -0.554772 1.333144 -1.708285
H 1.877506 -1.110794 0.037908
H 0.578790 0.509980 -1.540634
FREQS= 44 65 70 85 93 103 121 180 217 307 354 385 404 420 458 486 503 527 532 560 565 600 681 692 801 846 935 1036 1061 1169 1270 1333 1426 1446 1472 1514 1518 1617 1918 2111 2121 2170 3030 3059 3085 3132 3141
TS184_8
18
E= -1916.35304907 ZPE= 70.69 Gcorr 0.045139000
H 4.653698 0.583640 -0.129825
Co -0.592952 0.024794 -0.127076
C -2.176539 -0.296506 -0.875697
C -0.721462 -0.944299 1.367978
O -3.135158 -0.551291 -1.455431
O -0.822897 -1.564144 2.330868
C -0.677212 1.773430 0.373472
O -0.622964 2.891790 0.624192
C 2.490499 0.551455 -0.022061
C 3.831954 -0.097270 -0.368666
H 2.436655 0.827130 1.040650
H 2.346143 1.497472 -0.562266
H 3.977989 -1.023773 0.192208
H 3.885678 -0.343447 -1.432086
C 1.278679 -0.288207 -0.311356
O 1.423120 -1.449391 -0.793650
H -0.255989 0.376609 -1.540042
H 0.073194 -1.394397 -0.767435

FREQS= 39 51 59 73 85 90 113 198 205 282 302 359 378 418 443 455 478 499 515 526 557
654 696 748 771 861 948 1027 1066 1113 1273 1366 1422 1446 1481 1507 1512 1624 1968 2116 2118 2168 3034
3061 3063 3137 3143
TS185_8
18
E= -1916.36209916 ZPE= 75.59 Gcorr 0.054342000
H -2.885146 0.875836 1.231065
Co 0.263727 -0.030714 -0.007051
C 0.132986 1.710680 0.211223
C 1.560579 -0.135071 1.212076
O 0.052684 2.851040 0.343375
O 2.420590 -0.309338 1.954557
C 0.889070 -0.159473 -1.741655
O 1.441917 -0.211029 -2.744969
C -1.963752 0.006246 -0.539807
C -3.142421 0.205549 0.404245
H -2.182343 -0.673610 -1.364077
H -1.738990 0.967557 -1.001956
H -3.984455 0.660394 -0.132363
H -3.508901 -0.730137 0.840641
C -1.097582 -1.615600 0.581651
O 0.098833 -1.939772 0.209219
H -1.928664 -2.163168 0.131327
H -1.277607 -1.308785 1.621889
FREQS= 37 63 69 83 98 113 158 189 232 283 289 350 351 366 407 432 452 465 510 536 575
593 740 828 938 976 1041 1164 1208 1230 1261 1364 1428 1501 1508 1513 1567 2099 2108 2160 3009 3032 3085
3102 3106 3112 3147
TS186_8
18
E= -1916.35641532 ZPE= 72.98 Gcorr 0.048906000
H -3.711076 -0.751149 1.371842
Co 0.521027 0.051894 -0.297789
C 1.684777 0.470428 1.223287
C 1.293390 -1.606738 -0.553390
O 2.810438 0.904996 1.186805
O 1.736578 -2.609884 -0.873177
C 0.181667 1.752704 -0.550495
O -0.013777 2.849502 -0.833190
C -2.442393 0.391820 0.022167
C -3.726706 -0.374413 0.346923
H -2.447116 0.787532 -0.996680
H -2.326115 1.257102 0.687907
H -4.591156 0.285285 0.231191
H -3.854992 -1.227982 -0.323935
C -1.187426 -0.456136 0.192071
O -1.149663 -1.489456 0.811355
H -0.234914 -0.297831 -1.566604
H 1.209165 0.238642 2.218848
FREQS= 38 53 62 86 91 93 117 181 216 222 270 313 361 388 409 438 462 495 551 558 629
696 729 791 858 929 1015 1084 1102 1282 1341 1347 1426 1469 1506 1515 1766 1799 1849 2116 2175 2701 3050
3064 3116 3138 3151
TS187_8
18
E= -1916.36352833 ZPE= 76.24 Gcorr 0.056192000
H 2.197050 -0.623190 2.144734
Co -0.473452 -0.020107 -0.176111
C -0.852382 -1.724043 -1.207278
C -0.889530 1.688129 -0.037455
O 0.130197 -1.844384 -0.362926
O -1.155973 2.804314 0.037149
C 1.389572 0.423634 -0.282716
O 1.161594 0.529447 -1.487368
C 2.745225 0.485859 0.351373
C 2.963677 -0.645385 1.365742
H 2.795287 1.457049 0.864290
H 3.505316 0.485867 -0.439946
H 2.916586 -1.621289 0.876922
H 3.943022 -0.540130 1.839939
C -1.889052 -0.420245 0.743352
O -2.751904 -0.704170 1.455358
H -0.645694 -1.519024 -2.266550
H -1.804737 -2.225752 -1.000202
FREQS= 32 58 73 102 107 132 152 196 205 229 320 355 402 443 467 492 517 532 539 556 625
703 788 927 1031 1057 1062 1165 1204 1269 1323 1329 1426 1462 1507 1517 1568 1676 2086 2133 3013 3024 3063
3077 3097 3139 3147
TS188_8
18

E= -1916.36275324 ZPE=   76.06 Gcorr   0.055590000
H 3.103408 -0.173107 -0.117660
Co -0.136284 0.119535 -0.007569
C -1.885449 0.491910 -0.027609
C 0.305539 1.739427 0.606006
O -3.011949 0.727846 -0.073726
O 0.621178 2.759263 1.036990
C 0.603183 0.170451 -1.643455
O 0.996502 0.194624 -2.726077
C 1.449060 -0.543353 1.251454
C 2.683364 -0.989971 0.477582
H 1.118780 -1.324460 1.942932
H 1.688037 0.308670 1.891948
H 3.471522 -1.342748 1.157745
H 2.472376 -1.814622 -0.215379
C -0.416912 -1.654078 0.157272
O -1.545802 -2.251393 0.529603
H 0.369313 -2.391650 -0.062178
H -1.455911 -3.218572 0.545132
FREQS=   38   58   73   87   97 116 131 234 265 298 324 331 374 426 443 453 472 494 535 547 558
603 686 712 928 979 1023 1049 1213 1255 1265 1293 1425 1483 1500 1505 1515 2084 2092 2141 3012 3026 3058
3068 3099 3116 3726
TS189_8
18
E= -1916.35398666 ZPE=   72.42 Gcorr   0.047034000
H -3.745694 0.606032 1.250049
Co 0.114197 0.049791 0.052783
C 1.849745 -0.771939 -0.566317
C -0.532093 0.620023 -1.551660
O 2.671702 -1.297814 0.132285
O -1.005095 1.008853 -2.521551
C 1.189465 1.360663 0.589568
O 1.845102 2.216838 0.984898
C -1.605348 0.867956 1.072230
C -2.892585 0.111281 0.767505
H -1.622214 1.878469 0.660518
H -0.206549 -0.018325 1.476567
H -3.102137 0.064586 -0.305463
H -2.864697 -0.916918 1.142020
C -0.386900 -1.649663 0.161040
O -0.734212 -2.737792 0.283440
H 2.012122 -0.704996 -1.670275
H -1.507846 0.996198 2.154681
FREQS=   40   58   72   75   81   94 105 124 161 243 263 299 322 418 432 441 460 480 504 516 523
559 592 717 754 859 973 1017 1066 1236 1289 1348 1430 1495 1510 1510 1807 2109 2110 2154 2177 2804 3030
3065 3095 3111 3131
TS190_8
18
E= -1916.35538297 ZPE=   73.50 Gcorr   0.048712000
H -4.441078 -1.534400 0.685849
Co 0.210361 0.350525 -0.088462
C -0.969106 1.421219 -0.678470
C 1.342246 1.615213 0.582015
O -1.746409 2.132670 -1.146175
O 2.039091 2.411041 1.029415
C 2.174505 -1.682401 -0.145328
O 2.729774 -2.235795 -1.000378
C -2.530856 -1.054639 -0.253887
C -3.413216 -1.306570 0.985188
H -2.569188 -1.951147 -0.894017
H -2.956435 -0.238419 -0.844783
H -3.037994 -2.149800 1.574733
H -3.433299 -0.425959 1.634249
C -1.102536 -0.804061 0.106856
O 1.650600 -1.173931 0.784340
H 1.040402 0.274217 -1.363758
H -0.672810 -1.643107 0.680324
FREQS=   27   31   58   84 100 115 138 151 175 229 259 335 365 418 428 486 519 558 583 608 617
645 681 775 800 943 1016 1053 1173 1257 1287 1351 1379 1418 1494 1509 1518 1806 2099 2138 2413 2992 3004
3045 3092 3119 3125
TS191_8
18
E= -1916.36199842 ZPE=   75.47 Gcorr   0.055403000
H 4.068811 -0.080755 -0.597065
Co -0.169973 -0.203641 -0.100142
C 0.017379 1.644896 0.408509
C -1.496389 -0.046553 -1.356292

O -0.582220 2.665727 0.575792
O -2.405791 0.100465 -2.034451
C -1.015341 -0.697579 1.332912
O -1.566907 -1.102508 2.254813
C 1.967129 0.488273 -0.554496
C 3.102785 -0.515330 -0.328777
H 2.266369 1.338392 -1.182830
H 1.201524 0.010903 -1.265144
H 3.148195 -0.827912 0.717693
H 2.940178 -1.415053 -0.925977
C 1.242731 0.956340 0.665983
O 0.280746 -2.022912 -0.429237
H 1.683383 0.867362 1.654435
H 0.564435 -2.501096 0.360338
FREQS= 37 65 79 89 117 139 142 221 242 283 312 364 386 416 420 450 485 514 549 555 569 627 759 836 899 945 994 1060 1094 1141 1196 1251 1377 1437 1497 1510 1603 1926 2128 2167 2503 3032 3066 3137 3149 3191 3828
TS192_8
18
E= -1916.35682774 ZPE= 72.65 Gcorr 0.050360000
H -0.759848 3.154720 0.258194
Co 0.381413 0.082243 -0.508165
C 2.051483 0.028644 0.013325
C -0.927617 -0.243681 1.196548
O 3.167773 -0.005698 0.278633
O -0.493079 -0.377597 2.308438
C 0.085656 -1.607862 -0.244680
O -0.000404 -2.756803 -0.262009
C -2.205783 0.034043 0.642601
C 0.255380 2.762185 0.319977
H 0.381886 2.223116 1.259025
H -3.085824 0.045922 1.276659
H 0.984281 3.573405 0.269735
H 0.433521 2.136619 -0.572805
C -2.257279 0.332141 -0.706605
O -1.218217 0.383083 -1.475371
H 1.300906 0.311179 -1.705598
H -3.212700 0.557775 -1.189268
FREQS= 58 72 86 101 108 112 131 172 205 216 310 328 349 388 425 437 470 483 508 535 555 587 595 685 746 809 1013 1031 1117 1302 1333 1353 1393 1441 1554 1575 1585 1820 1846 2100 2152 2943 3099 3118 3175 3186 3213
TS193_8
18
E= -1916.35464050 ZPE= 71.95 Gcorr 0.048284000
H -1.477563 -0.878385 2.480721
Co 0.679436 0.000027 0.245853
C 2.472801 0.000073 0.257004
C 0.390672 1.529858 -0.669067
O 3.614298 0.000150 0.340519
O 0.179758 2.545380 -1.156908
C 0.390770 -1.530130 -0.668554
O 0.179940 -2.545847 -1.156023
C -1.310883 0.000125 0.510286
C -1.827631 0.000651 1.927418
H 0.730596 -0.679345 1.551920
H 0.730668 0.679740 1.551741
H -1.477518 0.880070 2.480084
H -2.925252 0.000711 1.997201
C -2.132240 -0.000265 -0.553448
O -3.502303 -0.000284 -0.563832
H -1.771979 -0.000631 -1.577968
H -3.828189 0.000039 0.346406
FREQS= 40 56 74 94 96 109 141 231 242 269 299 342 344 381 397 447 476 485 498 521 526 552 609 688 833 845 907 959 1054 1088 1146 1278 1414 1431 1479 1509 1688 2064 2070 2142 2154 2182 3004 3069 3088 3197 3809
TS194_8
18
E= -1916.35794387 ZPE= 75.21 Gcorr 0.051681000
H 4.278042 0.398393 -0.994905
Co -0.393183 -0.066323 0.249410
C -0.370975 0.278554 -1.541926
C 1.432005 -0.511793 0.810906
O -1.030465 1.168540 -2.011556
O 0.988493 0.692791 1.135574
C -1.241812 -1.560016 -0.025961
O -1.703631 -2.613494 -0.112435
C 2.564993 -0.674703 -0.198814

C 3.472768 0.560261 -0.272390
H 2.175565 -0.898632 -1.200092
H 3.145134 -1.558411 0.092061
H 3.924132 0.775421 0.700506
H 2.909241 1.445506 -0.576360
C -2.551041 1.053935 0.710400
O -1.683127 1.791805 0.850112
H 0.357418 -0.302757 -2.154919
H 1.420614 -1.263361 1.612783
FREQS= 40 54 62 77 89 107 126 148 153 181 200 251 257 324 445 480 488 551 602 607 638 777 877 884 969 1017 1090 1135 1274 1293 1314 1360 1420 1461 1499 1509 1519 1806 2102 2108 2854 3020 3038 3058 3074 3129 3141
TS195_8
18
E= -1916.35960383 ZPE= 74.99 Gcorr 0.053418000
H 3.639817 -1.157954 -1.401596
Co -0.433206 -0.000503 -0.101848
C 0.176517 -0.775523 1.363944
C -0.461212 1.740861 0.211958
O 0.614554 -1.205714 2.337623
O -0.398437 2.859182 0.477551
C -2.216514 -0.427307 0.007253
O -3.330736 -0.708106 0.027692
C 2.541978 0.458634 -0.405150
C 3.574646 -0.691657 -0.414152
H 2.517490 0.942137 0.576965
H 2.868517 1.220242 -1.133254
H 4.563854 -0.309067 -0.147827
H 3.301324 -1.463569 0.310464
C 1.198228 0.000926 -0.833002
O -1.104252 -0.974458 -1.705751
H -1.488771 -0.334845 -2.322260
H 1.163443 -0.486208 -1.814619
FREQS= 39 71 77 86 104 116 138 164 235 266 313 328 371 399 411 434 482 498 511 512 546 569 673 808 838 885 936 1005 1038 1160 1236 1259 1364 1419 1481 1509 1518 2097 2114 2146 2992 3055 3080 3097 3134 3136 3804
TS196_8
18
E= -1916.35201738 ZPE= 70.85 Gcorr 0.045950000
H -2.485713 1.204404 0.843549
Co 0.539445 0.023585 -0.145123
C 2.287664 -0.021544 -0.474024
C 0.191715 1.782546 0.173187
O 3.389691 -0.114605 -0.785456
O -0.097928 2.887364 0.284707
C 0.465559 -0.792625 1.442317
O 0.434049 -1.307939 2.469232
C -2.518532 -0.163556 -0.864559
C -3.086772 0.377641 0.456387
H -3.154590 -0.956393 -1.275572
H -2.472189 0.648153 -1.603627
H -4.104837 0.744950 0.302498
H -3.124307 -0.404975 1.220081
C -1.123081 -0.706080 -0.738690
O -0.897989 -1.893897 -1.112007
H 0.363405 -1.534280 -0.764583
H 0.491323 0.235661 -1.623554
FREQS= 42 54 66 82 85 91 111 173 206 297 321 360 394 423 453 468 498 513 525 552 557 567 663 730 798 867 938 1038 1062 1173 1270 1335 1427 1467 1484 1512 1518 1621 1962 2116 2118 2168 3033 3060 3088 3132 3142
TS197_8
18
E= -1916.35758943 ZPE= 74.11 Gcorr 0.051867000
H 0.676689 -3.192641 1.009960
Co 0.045337 0.043822 0.062079
C -0.720802 0.497757 -1.585019
C -1.542791 -0.630081 0.789097
O -1.899389 0.494929 -1.813606
O -2.513306 -1.064623 1.203896
C -0.032878 1.696574 0.768894
O -0.079458 2.775091 1.154520
C 0.452963 -1.765221 -0.642240
C 1.112548 -2.253271 0.654878
H 1.138165 -1.790925 -1.492693
H -0.433668 -2.342620 -0.908657
H 2.191504 -2.376378 0.542036
H 1.003873 -1.543764 1.502183

C 1.784128 0.448895 -0.603012
O 2.734655 0.369070 0.135106
H 1.919079 0.710135 -1.690087
H 0.021246 0.789337 -2.373773
FREQS= 36 72 80 95 98 118 150 168 182 211 255 291 327 378 415 431 475 497 514 542 621 664 810 895 907 946 990 993 1182 1187 1301 1346 1425 1480 1488 1511 1792 1819 2133 2175 2726 2779 2898 3072 3084 3131 3151
TS198_8
18
E= -1916.35059098 ZPE= 72.22 Gcorr 0.045126000
H 1.226260 -0.284568 0.488957
Co -0.474485 0.010689 0.083185
C -0.977521 1.643507 0.624819
C -1.212195 0.170864 -1.510868
O -1.314483 2.639151 1.095634
O -1.667609 0.273790 -2.559403
C -1.382386 -1.506103 0.525248
O -1.977306 -2.402679 0.924373
C 1.617153 -0.298959 -0.894016
C 3.943960 0.300232 -0.115838
H 1.309472 -1.116485 -1.548118
H 1.647835 0.639842 -1.449290
H 4.896038 0.010903 0.317381
H 3.828042 1.346104 -0.391638
C 2.970771 -0.595105 -0.324332
O 0.712851 -0.272913 1.602599
H 3.138866 -1.627918 -0.019313
H 0.978259 0.558111 2.020318
FREQS= 17 43 52 79 84 91 106 130 219 225 278 389 395 408 424 431 448 453 482 503 554 562 592 686 730 888 907 928 1018 1046 1118 1225 1332 1408 1448 1474 1677 1710 2090 2114 2161 3084 3141 3149 3160 3238 3822
TS199_8
18
E= -1916.35886172 ZPE= 74.31 Gcorr 0.053525000
H 0.003566 3.159418 -1.129811
Co 0.000992 0.030285 -0.193908
C 1.800123 -0.453118 -0.248957
C -0.293380 -0.635842 1.543090
O 2.908820 -0.727583 -0.251371
O 0.512027 -1.257512 2.174016
C -0.668526 -1.395238 -1.045492
O -1.130523 -2.313827 -1.554667
C 0.549919 1.806146 0.507620
C 0.750825 2.395477 -0.895660
H 1.470204 1.786944 1.092282
H -0.209827 2.338446 1.080463
H 1.748548 2.824781 -1.031483
H 0.653194 1.645202 -1.712059
C -1.746709 0.798055 -0.172318
O -2.643514 0.708735 0.630553
H -1.896567 1.447813 -1.081609
H -1.323902 -0.391702 1.899780
FREQS= 64 70 83 88 108 138 153 180 200 214 294 322 357 399 407 442 474 488 522 540 581 645 814 891 934 946 989 992 1173 1188 1247 1328 1422 1486 1492 1513 1791 1830 2127 2170 2721 2838 2870 3066 3100 3124 3158
TS200_8
18
E= -1916.35726582 ZPE= 73.56 Gcorr 0.051957000
H 3.842957 0.309275 -0.017819
Co -0.109282 0.055279 -0.204848
C 0.310985 -1.702389 -0.599943
C -0.593556 -0.125155 1.619216
O 0.636152 -2.773618 -0.827747
O -0.813810 -1.181738 2.144606
C -1.841983 0.033853 -0.690397
O -2.962534 0.017685 -0.929556
C 1.770766 0.183284 0.613475
C 2.844676 0.266225 -0.472461
H 1.935522 -0.700943 1.235720
H 1.832946 1.054203 1.272874
H 2.837046 -0.601994 -1.141382
H 2.740782 1.159481 -1.097840
C 0.038454 1.827590 -0.332695
O -0.004067 2.939273 0.076588
H 0.392819 1.537816 -1.435104
H -0.653442 0.836340 2.180136

FREQS=   69   73   80   83   90 110 148 188 221 252 260 349 378 386 418 465 477 489 500 506 531
625 741 791 904 981 987 1036 1115 1238 1271 1302 1428 1489 1507 1511 1810 1932 2132 2154 2180 2859 3026
3060 3086 3095 3120
TS201_8
18
E= -1916.36155238 ZPE=   76.26 Gcorr   0.056437000
H 3.061050 1.240689 0.066638
Co -0.244528 -0.052311 -0.105980
C -1.658778 0.184353 0.845674
C 1.362370 -1.039693 0.477468
O -2.290366 0.453997 1.808575
O 0.375254 -1.755845 -0.024588
C 0.194672 1.624178 -0.305298
O 0.537858 2.713729 -0.450541
C 2.655129 -0.876171 -0.291505
C 3.535956 0.267164 0.220856
H 2.435111 -0.761637 -1.358907
H 3.199726 -1.828021 -0.193918
H 3.740415 0.160742 1.291724
H 4.497375 0.278161 -0.299692
C -2.310282 -0.757258 -0.417033
O -2.604709 -0.325271 -1.503036
H 1.471285 -1.033536 1.572412
H -2.621395 -1.752325 -0.041043
FREQS=   52   60   80   85   95 106 143 176 224 238 307 344 417 432 443 494 508 544 576 627 692
782 899 917 951 1040 1106 1155 1272 1306 1333 1337 1431 1471 1486 1515 1519 1766 1939 2120 2948 3011 3020
3051 3077 3120 3131
TS202_8
18
E= -1916.35525189 ZPE=   72.81 Gcorr   0.050270000
H 3.651685 -1.694242 0.816638
Co -0.194839 -0.079877 -0.142053
C -1.026339 -1.588768 -0.743746
C -1.075688 -0.093183 1.588317
O -1.572206 -2.448157 -1.269431
O -2.221380 0.044180 1.923640
C -1.165353 1.310875 -0.566699
O -1.820710 2.189710 -0.903401
C 1.929228 -0.376173 0.748207
C 3.220130 -0.942506 0.150214
H 1.213316 -1.202947 0.893128
H 2.077849 0.076661 1.734242
H 3.030146 -1.409481 -0.819949
H 3.943302 -0.135997 0.006306
C 1.407817 0.787477 -0.163410
O 2.021199 1.759109 -0.479043
H 0.645456 -0.120881 -1.428359
H -0.295101 -0.301497 2.381994
FREQS=   50   64   76   85   96 122 160 161 210 267 284 337 373 384 421 450 465 492 520 551 614
680 714 748 834 849 987 1068 1096 1285 1328 1351 1420 1461 1505 1514 1809 1845 1894 2127 2164 2653 2980
3060 3087 3138 3147
TS203_8
18
E= -1916.35252932 ZPE=   72.50 Gcorr   0.047562000
H 4.819299 0.826705 0.044251
Co -0.447893 0.011584 -0.162916
C -1.915129 -0.697128 -1.076127
C -0.517415 -0.929643 1.383049
O -3.096143 -0.699150 -1.232909
O -0.605890 -1.446149 2.402566
C -1.260364 1.451970 0.283463
O -1.758109 2.450157 0.568805
C 2.656640 0.719718 0.173227
C 4.006936 0.185248 -0.308296
H 2.608030 0.782612 1.269149
H 2.471257 1.744189 -0.176793
H 4.182896 -0.827793 0.062679
H 4.046158 0.148320 -1.399949
C 1.467722 -0.098938 -0.263270
O 1.547071 -1.109750 -0.948559
H -0.129708 1.047010 -1.181123
H -1.230588 -1.382029 -1.691001
FREQS=   31   50   60   78   85   97 117 179 207 231 272 333 369 387 407 444 460 498 523 578 621
691 718 772 789 943 1017 1068 1105 1196 1271 1357 1425 1454 1509 1513 1716 1882 1998 2118 2156 2540 3037
3063 3067 3137 3141
TS204_8
18

E= -1916.35649762 ZPE=   74.94 Gcorr   0.051630000
H 4.670719 0.349490 0.276874
Co -0.442132 -0.033019 0.048321
C 0.000239 1.269252 -1.062531
C -0.897917 -1.533048 -0.916962
O 0.323189 2.093828 -1.801136
O -1.073052 -2.427603 -1.613233
C -2.060662 0.654739 0.501808
O -3.045325 1.075524 0.911698
C 2.513839 0.493640 0.544689
C 3.789864 -0.291846 0.181677
H 2.604225 0.838889 1.589913
H 2.419305 1.397370 -0.064834
H 3.922786 -1.156168 0.839452
H 3.748067 -0.653286 -0.850086
C 1.284420 -0.345128 0.512739
O -0.492279 -0.746602 1.865267
H 1.444830 -1.326204 0.964750
H -0.351328 -0.005412 2.469969
FREQS=   29   46   60   73   93 110 123 187 220 276 298 345 370 408 424 437 453 467 498 522 548
561 654 775 833 913 942 1034 1036 1144 1244 1291 1388 1421 1463 1509 1517 2087 2119 2158 2984 3052 3091
3128 3129 3136 3822
TS205_8
18
E= -1916.35150253 ZPE=   71.14 Gcorr   0.046951000
H -4.800840 -0.670025 0.137276
Co 0.701962 -0.003150 0.013731
C 1.080959 1.675028 -0.453543
C 2.382856 -0.392869 0.242745
O 1.271068 2.735867 -0.844351
O 3.491660 -0.631705 0.450313
C 0.277998 -1.724743 -0.280335
O 0.033248 -2.807279 -0.569318
C -2.693166 -0.194955 0.009345
C -4.132969 0.044618 -0.361049
H -2.318106 -1.206580 -0.133513
H -1.490395 0.185382 -1.052201
H -4.453375 1.053217 -0.080516
H -4.297931 -0.065451 -1.438570
C -2.016201 0.634243 0.902662
O -0.772091 0.555458 1.189733
H -0.635356 0.196610 -1.250937
H -2.544914 1.525237 1.277788
FREQS=   31   57   86   92   95 109 147 187 218 285 315 344 388 396 425 454 457 486 510 577 596
604 619 821 894 939 984 1049 1131 1154 1197 1255 1371 1400 1432 1495 1512 1583 2098 2113 2168 2281 3016
3029 3084 3112 3164
TS206_8
18
E= -1916.35624370 ZPE=   73.61 Gcorr   0.051794000
H -4.136628 -0.048271 -1.016288
Co 0.444715 -0.126292 -0.232065
C 2.069686 -0.862181 -0.794943
C 0.789212 1.662391 -0.325418
O 2.937438 -1.483414 -1.203789
O 1.039546 2.776622 -0.288604
C 0.694091 -0.283355 1.590153
O 1.777649 -0.516260 2.050345
C -2.414962 -1.049269 -0.143836
C -3.896668 -0.678937 -0.156334
H -2.160986 -1.698700 0.704540
H -2.138168 -1.635197 -1.030804
H -4.516402 -1.579431 -0.204541
H -4.167871 -0.119283 0.742696
C -1.469198 0.159675 -0.075220
O -1.934777 1.283655 -0.027510
H -0.195862 -0.126453 2.235379
H 0.116792 -1.557560 0.024822
FREQS=   55   74   80   85   96 128 144 198 205 260 296 315 320 353 394 413 475 500 514 625 664
686 782 887 898 917 1006 1061 1100 1268 1283 1336 1421 1454 1504 1510 1737 1804 1987 2145 2179 2909 3035
3058 3064 3130 3138
TS207_8
18
E= -1916.35431868 ZPE=   74.60 Gcorr   0.050329000
H 2.560462 -1.257182 -0.855145
Co -0.451393 0.044311 -0.027655
C -1.862197 1.239917 -0.151481
C -1.111354 -1.648290 0.117139

O -2.693218 1.973732 -0.451194
O -1.480663 -2.733585 0.138368
C 0.015933 0.292288 1.651912
O 0.379392 0.472323 2.728677
C 2.534596 -0.363058 -0.206141
C 3.806732 0.466788 -0.450672
H 2.499053 -0.746728 0.819980
H 3.817626 1.365195 0.174039
H 4.699257 -0.117864 -0.209727
H 3.877646 0.780120 -1.497032
C 1.268416 0.319296 -0.585060
O -0.440772 -0.279145 -1.925418
H 1.394600 0.935717 -1.478464
H -0.691693 0.536103 -2.384620
FREQS= 20 50 58 77 86 97 128 183 209 281 287 340 370 395 423 429 433 455 494 526 535 583 663 734 825 924 938 1020 1045 1132 1244 1287 1388 1422 1451 1509 1517 2100 2110 2158 2982 3050 3068 3115 3125 3128 3790
TS208_8
18
E= -1916.35536593 ZPE= 74.61 Gcorr 0.051451000
H -3.042884 -0.255927 1.003750
Co 0.370373 0.033055 -0.035169
C -0.394497 -0.335146 1.511248
C 0.922715 -1.646037 -0.397708
O -0.870686 -0.546120 2.537925
O 1.247261 -2.696532 -0.733256
C 1.864746 1.020973 0.537916
O 2.771401 1.696430 0.715855
C -2.485356 0.956195 -0.721944
C -3.179639 -0.242688 -0.079869
H -2.751606 1.025900 -1.790678
H -2.873859 1.905213 -0.306724
H -4.255010 -0.215764 -0.280003
H -2.784608 -1.181428 -0.480302
C -1.004818 1.074852 -0.642696
O 0.807561 0.438802 -1.869674
H 0.381454 -0.219849 -2.434197
H -0.656760 1.939855 -1.210773
FREQS= 27 58 66 74 91 110 127 174 232 279 307 353 364 398 410 434 442 450 504 519 547 572 633 686 852 919 938 1011 1055 1119 1271 1297 1370 1417 1436 1510 1519 2096 2112 2160 2966 2995 3058 3125 3131 3145 3827
TS209_8
18
E= -1916.35432960 ZPE= 73.13 Gcorr 0.050419000
H 4.147537 -0.181929 -0.381291
Co -0.555063 0.041545 -0.405306
C -0.845550 -1.812735 -0.906620
C -1.702870 -0.108380 0.915119
O 0.033278 -2.640798 -0.817020
O -2.547177 -0.164059 1.693163
C -0.469724 1.811732 -0.755147
O -0.589277 2.909395 -1.065513
C 2.013937 -0.431401 -0.166697
C 3.337050 0.236969 0.221574
H 1.776133 -0.259177 -1.218974
H 2.031620 -1.518921 -0.039406
H 3.307408 1.316511 0.049019
H 3.555044 0.066206 1.278051
C 0.884996 0.048713 0.776340
O 1.004904 0.301263 1.932513
H -0.491583 -0.150202 -1.944618
H -1.860309 -2.109982 -1.252070
FREQS= 38 65 75 93 100 106 134 202 223 233 272 331 342 407 440 468 472 523 538 552 575 630 680 777 823 927 978 1068 1095 1261 1331 1367 1420 1466 1504 1515 1749 1815 1882 2110 2153 2866 3061 3083 3119 3135 3153
TS210_8
18
E= -1916.35307879 ZPE= 72.85 Gcorr 0.049854000
H 3.288100 -1.110079 -1.867162
Co -0.244391 0.125821 -0.098722
C -0.117451 -0.620984 1.793692
C -1.513815 1.365629 -0.353564
O 0.692954 -1.445597 2.125971
O -2.311505 2.164685 -0.567198
C -1.316599 -1.269980 -0.350225
O -2.000898 -2.168058 -0.558621
C 1.316887 -1.020441 -1.001750

C 2.513007 -0.356603 -1.679381
H 0.208315 0.140604 -1.511209
H 0.889318 -1.785660 -1.651309
H 2.251500 0.097021 -2.640493
H 2.962628 0.422543 -1.057551
C 0.973160 1.258764 0.525865
O 1.757505 2.002722 0.914036
H -0.850510 -0.229029 2.538132
H 1.613613 -1.500892 -0.066247
FREQS= 56 71 86 93 97 109 116 144 180 255 277 309 374 410 433 447 466 484 529 531 545 560 593 690 786 853 991 1030 1067 1241 1289 1340 1430 1504 1507 1514 1793 2060 2108 2115 2166 2829 3038 3078 3103 3120 3147
TS211_8
18
E= -1916.35405497 ZPE= 74.72 Gcorr 0.051238000
H -3.472101 -0.204137 1.628163
Co 0.616622 -0.040941 -0.354704
C 2.351494 -0.679246 0.055816
C 1.325718 1.609467 -0.196159
O 2.853468 -0.878014 1.133222
O 1.807441 2.636169 -0.038349
C 0.136857 -1.728550 -0.205312
O -0.156673 -2.828882 -0.035003
C -2.707800 -0.225468 -0.405392
C -3.817400 0.017853 0.614913
H -2.385916 -1.271766 -0.413400
H -3.057866 -0.026645 -1.430760
H -4.144411 1.059995 0.598228
H -4.677471 -0.620932 0.397988
C -1.507949 0.673094 -0.205708
O -1.480446 1.624605 0.543985
H -0.731029 0.603195 -1.098957
H 2.944158 -0.868226 -0.884050
FREQS= 33 38 80 84 89 111 127 178 202 221 244 292 309 334 404 427 445 513 532 545 669 766 855 897 1009 1049 1104 1200 1274 1333 1366 1430 1460 1496 1508 1514 1794 1799 2083 2101 2172 2711 3017 3066 3098 3139 3148
TS212_8
18
E= -1916.35472993 ZPE= 73.01 Gcorr 0.052077000
H -3.390880 -1.855613 0.997560
Co 0.262254 -0.083470 -0.275178
C 1.587066 -1.236610 -0.774776
C 0.965096 1.510621 -0.117791
O 2.459233 -1.840418 -1.207933
O 1.458354 2.543326 -0.061503
C 0.544044 -0.378876 1.625082
O 1.367550 0.026698 2.402001
C -1.907192 -0.940183 -0.294502
C -2.957094 -0.869141 0.814468
H -1.098286 -1.644127 -0.028709
H -2.307637 -1.334662 -1.232257
H -2.529527 -0.507167 1.753878
H -3.752240 -0.180748 0.517446
C -1.417793 0.504179 -0.656656
O -2.111299 1.432447 -0.929754
H -0.241068 -1.104162 1.998218
H -0.036680 0.043800 -1.773779
FREQS= 64 72 83 92 114 138 174 202 242 260 319 333 372 381 425 447 470 503 515 584 598 662 705 725 825 845 981 1067 1089 1273 1324 1346 1424 1476 1508 1512 1805 1843 1900 2131 2168 2655 2972 3060 3105 3135 3146
TS213_8
18
E= -1916.34964673 ZPE= 71.70 Gcorr 0.047157000
H -3.884270 0.381927 -1.444042
Co 0.840293 0.186865 -0.506628
C 2.170379 -0.901718 -0.355221
C 1.322383 1.576752 0.567141
O 3.046835 -1.640940 -0.285343
O 1.717586 2.447559 1.198197
C -0.305165 -0.918249 0.583541
O -0.365977 -1.795370 1.414808
C -2.972919 -0.513819 0.315560
C -3.987311 0.409982 -0.356309
H -3.071620 -0.524160 1.410538
H -3.115372 -1.566113 0.027624
H -5.004987 0.106283 -0.098694
H -3.847283 1.446227 -0.037568

C -1.545670 -0.195241 0.025990
O -1.161752 0.712441 -0.736354
H 1.375099 0.925674 -1.713888
H 0.876802 -0.350939 -1.879681
FREQS= 32 44 61 80 81 117 147 183 254 266 304 317 337 376 450 485 496 554 561 580 606 682 717 778 792 855 993 1055 1072 1169 1258 1391 1429 1440 1510 1513 1670 1797 1918 2056 2132 2165 3032 3056 3065 3140 3142
TS214_8
18
E= -1916.35619179 ZPE= 74.19 Gcorr 0.053706000
H -0.485284 -2.583353 0.049924
Co -0.023155 -0.062943 -0.028935
C -2.445841 -0.596493 0.067882
C 1.311897 -0.455513 1.150030
O -1.907776 -0.513297 1.179679
O 2.100554 -0.781840 1.908842
C -0.194256 1.697227 0.357422
O -0.322862 2.819690 0.542809
C 0.142569 -1.985527 -0.621446
C -1.750565 0.022321 -1.071192
H 1.158167 -2.386817 -0.567855
H -0.213239 -2.122975 -1.645910
H -1.870199 -0.534759 -2.003913
H -2.115531 1.044343 -1.227520
C 1.145336 0.266876 -1.456271
O 2.337122 0.403961 -1.370238
H 0.628583 0.331181 -2.441250
H -3.388461 -1.169610 -0.029517
FREQS= 65 82 83 95 110 122 144 171 214 231 281 329 394 420 432 449 481 510 528 540 621 641 756 786 838 858 918 968 1139 1182 1274 1317 1394 1451 1473 1488 1692 1805 2138 2177 2864 2962 3033 3061 3118 3125 3133
TS215_8
18
E= -1916.35264355 ZPE= 72.60 Gcorr 0.050231000
H -1.069818 1.074630 -1.562747
Co 0.561334 -0.062191 0.312489
C 2.188784 -0.842859 0.309966
C -0.200670 -1.313920 -0.711657
O 3.223132 -1.334787 0.261687
O -0.667164 -2.099935 -1.408182
C 1.170915 1.537248 -0.198646
O 1.568010 2.557737 -0.550529
C -2.314810 -0.045914 0.994861
C -3.385451 -0.556363 0.379066
H -1.138763 1.703694 1.026675
H -2.153986 -0.251461 2.053240
H -3.580681 -0.344040 -0.666236
H -4.098474 -1.177385 0.911281
C -1.298616 0.841788 0.363708
O -1.730527 1.305707 -0.903309
H 0.217293 -0.608462 1.829877
H 0.559895 0.132548 1.949579
FREQS= 47 72 81 95 97 103 129 238 252 273 297 358 368 431 449 460 478 506 526 545 553 606 627 640 713 921 935 955 1008 1032 1128 1141 1289 1327 1380 1445 1539 1701 2103 2116 2165 3007 3144 3172 3188 3262 3879
TS216_8
18
E= -1916.35747100 ZPE= 75.69 Gcorr 0.055073000
H -3.010909 -0.372382 0.401352
Co 0.241239 -0.233822 -0.312961
C 1.384905 1.301718 -0.793648
C -0.603374 0.668566 1.153082
O 0.894517 2.635147 -0.659935
O -0.359624 0.660717 2.310170
C 1.717694 -1.076079 0.185582
O 2.641461 -1.561113 0.669677
C -1.336385 0.977868 -0.011927
C -2.807436 0.583709 -0.088297
H -1.075312 1.966230 -0.386388
H 0.886522 3.051607 -1.529858
H -3.431668 1.343915 0.396513
H -3.132550 0.498404 -1.129413
C -0.787156 -1.693327 -0.447844
O -1.428284 -2.613487 -0.700651
H 1.459689 1.005577 -1.861737
H 2.396736 1.275000 -0.376287

FREQS= 48 59 79 90 93 135 163 173 192 269 306 310 340 422 439 447 480 493 543 553 569
610 684 843 919 1021 1051 1070 1116 1131 1295 1328 1340 1425 1460 1501 1515 1986 2093 2142 2892 3039 3065
3104 3127 3150 3835
TS217_8
18
E= -1916.35206128 ZPE= 71.77 Gcorr 0.049862000
H -4.228319 0.019577 0.686477
Co 0.757463 0.253506 -0.202020
C 0.274516 1.907134 -0.599796
C 0.859508 0.316640 1.606452
O 0.008537 2.967750 -0.938180
O 0.887437 0.360952 2.748858
C 1.659036 -1.201627 -0.646766
O 2.277295 -2.090193 -1.020373
C -2.233199 0.129739 -0.127159
C -3.603185 -0.490372 -0.057094
H 0.414258 0.100656 -1.623100
H -2.196272 1.206451 -0.268034
H -4.127090 -0.410199 -1.018819
H -3.542703 -1.547949 0.204990
C -1.076888 -0.544088 -0.045493
O -1.138336 -1.930199 0.018656
H 2.128878 0.865848 -0.402818
H -0.458436 -2.250073 0.623294
FREQS= 56 77 91 95 99 135 173 223 291 298 327 337 345 430 436 453 483 498 519 532 552
603 654 673 699 754 795 955 1035 1055 1133 1227 1347 1417 1499 1507 1700 1874 2069 2146 2160 2196 3026
3071 3147 3181 3830
TS218_8
18
E= -1916.35540079 ZPE= 75.58 Gcorr 0.053368000
H -1.466283 -1.636911 2.019192
Co -0.399306 0.094997 0.155623
C 2.236692 -0.637441 -0.652780
C -1.283367 0.378317 -1.294825
O 3.163102 -0.163763 -1.290879
O -1.813072 0.538068 -2.306719
C -0.334589 1.831853 0.630909
O -0.471763 2.930216 0.939961
C -0.874680 -1.794232 -0.097780
C -1.887928 -1.914972 1.046361
H -0.000311 -2.426002 0.089305
H -1.321721 -2.111795 -1.042442
H -2.781363 -1.301855 0.880064
H -2.237834 -2.952166 1.145356
C 2.025651 -0.223813 0.767464
O 0.977953 -0.407798 1.422595
H 2.894032 0.257046 1.245323
H 1.554298 -1.405296 -1.054363
FREQS= 40 47 56 87 95 108 129 157 193 222 252 337 376 411 452 457 488 543 567 578 585
765 840 942 964 986 995 1083 1215 1257 1351 1387 1414 1486 1501 1510 1613 1736 2090 2132 3015 3016 3043
3058 3073 3094 3124
TS219_8
18
E= -1916.35472041 ZPE= 74.23 Gcorr 0.052756000
H 3.126378 1.577481 -0.866274
Co -0.255899 0.052566 0.163236
C 0.253751 -0.202785 -1.606569
C 0.845067 -1.208282 0.988729
O 1.000103 -1.069932 -1.968732
O 1.523578 -1.944099 1.537715
C -1.740165 -0.953874 0.026801
O -2.687915 -1.581285 -0.122804
C 1.252182 1.393102 0.247846
C 2.704733 0.976041 -0.054992
H 1.133893 1.671962 1.306581
H 1.010109 2.296286 -0.321260
H 3.349381 1.118399 0.819126
H 2.808996 -0.067317 -0.367562
C -1.254785 1.596449 -0.186088
O -1.754770 2.065043 0.809279
H -1.338945 2.096870 -1.186656
H -0.193211 0.525313 -2.329350
FREQS= 55 81 83 95 112 117 134 148 215 246 254 277 332 372 394 423 446 496 515 520 620
656 691 887 909 980 1011 1051 1184 1264 1283 1316 1430 1464 1505 1525 1778 1815 2132 2174 2779 2810 3000
3046 3071 3110 3115
TS220_8
18

E= -1916.34973298 ZPE=   71.72 Gcorr   0.048685000
H 2.566422 1.658480 0.459943
Co -0.578566 -0.090143 -0.306706
C -2.202819 -0.867132 -0.341797
C 0.136617 -1.014598 1.080839
O -3.216767 -1.387065 -0.454701
O 0.607691 -1.629772 1.922314
C -1.076738 1.556906 0.271527
O -1.415121 2.606980 0.575943
C 2.271862 -0.330452 -1.079267
C 3.297834 -0.788966 -0.353363
H 1.134707 1.419316 -1.438652
H 2.128378 -0.695282 -2.095675
H 3.461247 -0.465012 0.670103
H 3.996356 -1.513678 -0.757786
C 1.280850 0.686688 -0.633761
O 1.656779 1.339713 0.573507
H -0.222101 -1.006940 -1.404807
H -0.750033 0.143443 -1.753659
FREQS=   50   63   77   90   98 108 142 241 249 256 300 358 371 397 437 465 469 496 510 519 543
598 637 731 843 849 918 921 1013 1038 1121 1169 1285 1326 1380 1451 1699 2064 2076 2142 2162 2189 3017
3153 3166 3249 3737
TS221_8
18
E= -1916.35414625 ZPE=   75.81 Gcorr   0.053367000
H 4.623845 0.235527 0.248235
Co -0.353666 0.207309 -0.381895
C -0.924182 -1.364974 -0.863223
C -0.295762 -0.280103 1.389088
O -1.244910 -2.355610 -1.356764
O 0.096188 0.624004 2.096987
C -1.855538 1.259472 -0.379209
O -2.826553 1.872611 -0.380949
C 2.574049 0.857245 -0.164961
C 3.750861 -0.126323 -0.304106
H 2.335941 1.035971 0.888209
H 2.877720 1.833445 -0.583315
H 3.486698 -1.110522 0.095671
H 4.039648 -0.255714 -1.352529
C 1.350521 0.473205 -0.934365
O -0.601220 -1.508607 1.853061
H 1.641942 0.187686 -1.959056
H -0.444535 -1.494062 2.815920
FREQS=   35   43   68   73   84 106 125 147 210 243 312 338 352 408 435 446 482 533 581 613 677
689 780 866 926 995 1034 1109 1140 1237 1269 1336 1382 1418 1476 1508 1517 1761 2090 2133 2978 3005 3043
3091 3119 3120 3692
TS222_8
18
E= -1916.35683655 ZPE=   76.43 Gcorr   0.056475000
H 2.607782 -0.601814 -1.546520
Co -0.103717 -0.037625 0.217060
C 1.015017 -1.365894 0.626921
C 0.998249 1.164694 0.977346
O 1.733945 -2.237002 0.843340
O 1.706334 1.942510 1.443198
C -1.364159 1.171519 0.040206
O -2.233095 1.909895 -0.146492
C 0.556607 0.143982 -1.746930
C 2.065938 0.268801 -1.932361
H 0.056143 1.014452 -2.179322
H 0.182098 -0.733413 -2.279418
H 2.321239 0.356443 -2.998584
H 2.475585 1.153945 -1.433346
C -1.389710 -1.342300 0.115958
O -2.701948 -1.315942 -0.127756
H -3.041192 -0.408137 -0.163019
H -1.134830 -2.406101 0.154424
FREQS=   47   69   88   94 101 104 129 142 246 263 300 365 421 446 456 463 470 517 535 566 618
671 678 693 955 965 986 1043 1209 1263 1273 1302 1424 1488 1509 1511 1518 2069 2098 2142 3010 3064 3078
3093 3095 3125 3760
TS223_8
18
E= -1916.35084353 ZPE=   73.09 Gcorr   0.050781000
H -2.346466 0.312851 1.282167
Co 0.483832 0.085241 -0.238678
C -0.276778 1.602055 0.271530
C -0.003606 -1.240720 0.992726

O -0.744161 2.626550 0.478802
O -0.280439 -2.163648 1.600678
C 2.209389 0.426552 0.245379
O 3.326893 0.612087 0.394511
C -2.480434 -0.276981 -0.812084
C -3.090436 0.083203 0.514086
H 2.056647 -1.479208 -1.263054
H -3.207647 -0.604932 -1.560431
H -3.750449 0.955232 0.422798
H -3.709986 -0.740255 0.889645
C -1.203884 -0.266824 -1.200102
O 1.092254 -1.456497 -1.196956
H 0.747507 0.933356 -1.411073
H -0.934929 -0.590209 -2.201241
FREQS= 58 65 83 98 105 107 149 161 192 231 342 357 394 407 418 435 452 466 499 522 532
571 644 693 771 804 933 969 994 1060 1064 1228 1327 1425 1493 1504 1689 2028 2148 2171 2201 3031 3082
3095 3121 3195 3810
TS224_8
18
E= -1916.35202823 ZPE= 75.51 Gcorr 0.052080000
H 2.896545 -1.164205 0.841403
Co -0.291108 -0.257714 -0.340435
C -1.371473 -1.634068 0.221437
C -0.144850 0.707749 1.223386
O -2.089877 -2.461992 0.562089
O 0.439192 0.098159 2.094312
C -1.402198 0.906331 -0.988873
O -2.107667 1.599343 -1.580793
C 2.714281 -0.142529 -1.068221
C 3.213630 -0.228070 0.373505
H 3.061308 0.792906 -1.542345
H 3.186092 -0.931985 -1.679812
H 4.307093 -0.180989 0.405487
H 2.820054 0.588981 0.982594
C 1.243743 -0.257697 -1.306325
O -0.577424 1.977124 1.372002
H -0.323859 2.257753 2.271456
H 1.080081 -0.215546 -2.397376
FREQS= 14 57 60 72 88 107 118 171 189 267 315 332 359 399 427 457 492 527 557 598 678
689 693 864 915 1028 1029 1116 1138 1262 1275 1334 1367 1423 1437 1507 1512 1763 2090 2135 2964 2988 2993
3056 3125 3143 3690
TS225_8
18
E= -1916.35397347 ZPE= 74.57 Gcorr 0.054071000
H -3.462397 1.793275 -0.096887
Co 0.182870 0.093359 -0.102353
C 0.113710 -0.502127 1.662595
C -0.928342 -1.200786 -0.856129
O -0.385131 -1.537750 2.004230
O -1.592830 -1.996021 -1.334614
C 1.740830 -0.753516 -0.433269
O 2.749937 -1.269439 -0.601720
C -1.413296 1.268930 0.396881
C -2.721969 1.027254 -0.362702
H -1.097851 2.302881 0.214768
H -1.609302 1.206042 1.473134
H -2.589267 1.080802 -1.449484
H -3.167706 0.055352 -0.127576
C 1.130545 1.658031 0.287136
O 1.417129 2.280428 -0.709712
H 1.369963 2.038756 1.314494
H 0.577382 0.197734 2.402536
FREQS= 55 81 83 94 111 126 173 206 227 247 265 295 329 375 408 419 465 486 493 521 615
667 682 893 907 989 1007 1039 1264 1284 1301 1319 1421 1460 1510 1515 1773 1819 2135 2175 2784 2811 3023
3038 3076 3083 3112
TS226_8
18
E= -1916.34891065 ZPE= 72.50 Gcorr 0.049082000
H 4.351337 0.103018 0.614549
Co -0.399759 0.033325 -0.010284
C -2.020409 -0.422590 -0.698471
C -0.415558 -1.087707 1.378938
O -2.988517 -0.741869 -1.220346
O -0.408387 -1.705032 2.350279
C -0.848669 1.731053 0.302449
O -1.115226 2.812172 0.602303
C 2.406233 -0.320558 -0.271191

C 3.843186 0.174787 -0.353615
H 1.766897 -0.506942 -1.289226
H 2.316410 -1.376446 0.012173
H 4.417989 -0.418501 -1.071468
H 3.886241 1.219216 -0.678344
C 1.378058 0.503662 0.262397
O 0.305265 -0.911035 -1.632054
H 1.680323 1.494921 0.618203
H -0.027835 -0.520832 -2.452731
FREQS= 50 62 71 90 96 97 145 195 228 282 293 310 369 401 407 430 447 478 495 510 519 552 554 678 745 901 930 963 1074 1086 1217 1265 1317 1427 1465 1508 1517 1947 2082 2112 2161 3043 3070 3089 3109 3121 3807
TS227_8
18
E= -1916.35622076 ZPE= 76.49 Gcorr 0.056454000
H -0.664292 -2.392193 -1.689513
Co 0.064764 0.191984 0.203799
C 1.128417 1.638095 0.083045
C -1.364530 1.156809 -0.126188
O 1.814308 2.554579 -0.029381
O -2.352356 1.700552 -0.380246
C 1.424532 -0.762593 0.856702
O 2.294686 -1.416181 1.229056
C 0.322041 -0.440189 -1.762955
C 0.295883 -1.951480 -1.978035
H 1.272849 -0.028896 -2.112676
H -0.462626 0.032898 -2.359429
H 0.458559 -2.197376 -3.037997
H 1.076277 -2.468839 -1.409185
C -1.021765 -1.084699 0.946856
O -2.345519 -1.216495 1.044878
H -2.809159 -0.430140 0.716994
H -0.616667 -2.014344 1.358236
FREQS= 44 67 87 90 100 106 126 143 238 282 305 365 424 445 457 462 470 522 533 567 615 675 683 694 960 976 986 1048 1219 1264 1275 1303 1426 1487 1509 1513 1520 2067 2099 2142 3006 3060 3076 3094 3097 3120 3756
TS228_8
18
E= -1916.34995810 ZPE= 72.39 Gcorr 0.050290000
H -3.776163 -1.289244 -0.846094
Co 0.791185 -0.000008 0.348524
C 2.529172 -0.000060 -0.008743
C 0.494183 -1.675784 -0.257041
O 3.638477 -0.000073 -0.301167
O 0.159262 -2.691580 -0.668105
C 0.494291 1.675796 -0.257039
O 0.159398 2.691605 -0.668091
C -3.297133 0.761178 -0.026238
C -3.297083 -0.761206 -0.026070
H -3.776244 1.289001 -0.846381
H -3.384167 1.247352 0.940776
H 0.979059 0.390857 2.094199
H -3.384085 -1.247171 0.941052
C -2.023030 0.000004 -0.278845
O -1.050040 0.000159 0.717722
H 0.979049 -0.390950 2.094170
H -1.658628 -0.000089 -1.306891
FREQS= 43 52 88 88 94 99 190 255 277 349 352 394 418 422 437 448 472 475 505 566 570 623 698 768 788 844 931 1005 1057 1057 1112 1144 1187 1198 1234 1396 1464 1508 2124 2124 2180 3101 3132 3137 3217 3231 3784
TS229_8
18
E= -1916.35053190 ZPE= 72.87 Gcorr 0.051110000
H 3.387168 -0.876792 0.820304
Co -0.244530 0.149182 -0.231828
C -1.200704 -1.364850 -0.991255
C 0.728070 -0.651507 1.006535
O -2.367293 -1.644127 -0.933780
O 1.136562 -1.056423 2.028708
C -1.702944 0.593389 0.669507
O -2.645297 0.883269 1.249415
C 1.672907 -0.997312 -0.510950
C 3.088074 -0.541729 -0.175935
H 1.398075 -0.668891 -1.514123
H 1.549624 -2.082596 -0.486400
H 3.792037 -0.959628 -0.903585
H 3.181894 0.547627 -0.212926

C 0.501702 1.818689 -0.387254
O 0.871544 2.884146 -0.585464
H -0.710477 0.387083 -1.668472
H -0.482780 -2.049725 -1.510355
FREQS= 55 68 79 97 107 130 160 208 219 277 302 332 347 380 404 423 485 495 534 550 575
582 635 689 796 923 979 1008 1063 1217 1278 1354 1425 1473 1504 1516 1806 1862 1994 2138 2175 2789 3051
3091 3119 3132 3151
TS230_8
18
E= -1916.33649883 ZPE= 71.13 Gcorr 0.037459000
H 1.736623 3.533593 -0.820418
Co 0.160075 -0.090962 -0.212406
C 1.922658 -0.626802 -0.271492
C -0.025900 -0.307155 1.605623
O 2.503646 -1.130402 0.648570
O -0.198473 -0.372319 2.733562
C -0.527965 -1.606115 -0.881446
O -0.940142 -2.520707 -1.437459
C 0.688770 1.795732 -0.024334
C 1.607468 2.465507 -1.040361
H 1.037053 2.026838 0.988431
H -0.330449 2.221108 -0.107054
H 2.611474 2.022698 -1.029561
H 1.222287 2.376047 -2.060583
C -3.360487 0.577924 -0.588940
O -3.247401 1.103853 0.413780
H 0.218239 0.268704 -1.649940
H 2.414453 -0.430958 -1.247837
FREQS= 19 21 32 38 67 74 80 89 101 124 193 245 286 318 379 396 405 444 482 524 540
625 659 797 833 888 912 1006 1056 1211 1276 1291 1423 1448 1506 1512 1811 2014 2134 2173 2205 2912 2941
3021 3065 3072 3113
TS231_8
18
E= -1916.35095126 ZPE= 73.71 Gcorr 0.051973000
H -3.524150 -1.669401 -0.518395
Co 0.361488 -0.015628 -0.113714
C 1.366087 1.383366 0.287448
C 0.187427 -0.768943 1.525104
O 2.060246 2.280151 0.467488
O 0.103108 -1.247188 2.562852
C 1.422677 -1.254845 -0.901525
O 2.166801 -1.897955 -1.492928
C -1.661290 -0.627331 -0.824927
C -2.972348 -0.839888 -0.061239
H -1.170723 -1.598713 -0.890615
H -1.818604 -0.254797 -1.834322
H -2.796873 -1.106692 0.987304
H -3.621584 0.039207 -0.081735
C -1.305055 1.115700 -0.042641
O -1.565754 2.027041 -0.790516
H 0.552585 0.585429 -1.446678
H -1.721028 1.082183 0.986236
FREQS= 60 67 75 79 90 100 126 235 254 317 336 354 363 400 411 421 461 487 502 523 535
603 746 760 838 916 981 1032 1062 1205 1277 1364 1423 1479 1504 1521 1749 2051 2115 2133 2172 2881 3038
3092 3103 3128 3185
TS232_8
18
E= -1916.34923476 ZPE= 73.29 Gcorr 0.050624000
H -3.210407 -2.142353 -0.059860
Co 0.068062 0.230930 -0.271315
C -1.325125 1.443399 -0.187252
C 0.141412 -0.508026 1.474244
O -2.197806 2.182209 -0.237704
O 0.914286 -1.307062 1.909713
C 1.410920 1.364785 0.094144
O 2.272008 2.063936 0.383901
C -1.322113 -1.234008 -0.611441
C -2.535976 -1.358898 0.308240
H -0.820930 -2.203600 -0.666361
H -1.657224 -0.985028 -1.628763
H -2.256742 -1.645574 1.327881
H -3.122277 -0.435708 0.374317
C 1.199587 -0.916598 -1.100245
O 1.957968 -1.826454 -1.042340
H 0.916596 -0.392313 -2.109658
H -0.670544 -0.075475 2.113239

FREQS= 55 65 73 86 90 96 131 198 223 254 265 337 349 373 410 425 464 486 494 507 530
592 702 817 876 952 1004 1040 1139 1229 1289 1297 1429 1471 1507 1516 1841 1924 2124 2160 2309 2790 3013
3031 3086 3098 3118
TS233_8
18
E= -1916.34666005 ZPE= 72.39 Gcorr 0.048077000
H -3.974769 -0.169944 -0.914416
Co 0.383307 0.013064 0.043675
C 0.765932 -1.534957 0.755102
C 0.653613 -0.136736 -1.765039
O 1.012685 -2.576638 1.186579
O 0.868115 -0.401741 -2.860680
C 1.904881 0.838177 0.584837
O 2.807227 1.439789 0.957827
C -2.403374 0.313798 0.505974
C -3.866053 0.105126 0.139170
H -1.765169 1.303612 0.107150
H -2.253420 0.616298 1.549640
H -4.327153 -0.685206 0.742638
H -4.442584 1.021480 0.300606
C -1.394454 -0.553362 0.018360
O -0.375467 1.857311 0.113924
H -1.723916 -1.411021 -0.581472
H -0.326020 2.229999 1.005000
FREQS= 30 59 66 85 95 99 139 201 245 276 298 310 335 413 422 433 450 462 495 516 535
547 575 691 788 862 912 968 1071 1082 1213 1263 1390 1426 1465 1508 1516 1806 2089 2116 2158 3042 3064
3075 3107 3122 3818
TS234_8
18
E= -1916.34979219 ZPE= 72.26 Gcorr 0.051219000
H -3.355767 0.344134 -1.767133
Co 0.696396 0.087444 -0.456281
C 1.861450 -1.181265 -0.541289
C 1.632578 1.441139 0.310624
O 2.614509 -2.039008 -0.660902
O 2.255060 2.317614 0.699736
C -0.342411 -0.588106 1.031854
O -0.311900 -1.209888 2.060652
C -2.850137 -0.513014 0.164583
C -3.780680 0.266068 -0.763367
H -3.262073 -0.626436 1.178292
H -2.699890 -1.543187 -0.187434
H -3.946748 1.281199 -0.394006
H -4.748897 -0.235943 -0.834989
C -1.492095 0.095934 0.331237
O -1.091746 1.105919 -0.303396
H 0.154043 -0.819924 -1.495556
H 1.157025 0.717538 -1.750142
FREQS= 54 68 76 82 104 162 195 236 273 286 332 382 402 418 452 509 522 552 562 609 625
652 693 770 799 847 990 1021 1086 1147 1270 1384 1429 1446 1509 1512 1598 1836 1892 2007 2143 2179 3031
3059 3066 3141 3144
TS235_8
18
E= -1916.34555706 ZPE= 70.92 Gcorr 0.047066000
H -4.089134 0.107499 -1.643720
Co 0.638775 -0.054712 0.100903
C 2.177811 -0.599200 -0.668161
C 1.079327 1.667471 0.291674
O 3.102113 -0.958945 -1.243662
O 1.396294 2.750113 0.480741
C 0.118223 -1.611879 0.759804
O -0.204380 -2.586446 1.272286
C -2.228939 -0.465824 -0.666654
C -3.712957 -0.204298 -0.659699
H -1.902594 -1.396905 -1.117158
H 0.796855 0.050640 1.605459
H -4.260477 -1.112879 -0.385910
H -4.009915 0.563139 0.065769
C -1.281423 0.357485 -0.175595
O -1.576941 1.604869 0.320990
H 0.058782 0.092317 -1.263600
H -2.489393 1.834150 0.083731
FREQS= 54 67 83 92 96 113 115 199 235 303 328 357 366 367 403 447 469 486 502 530 542
594 605 627 692 750 798 952 1038 1052 1131 1292 1346 1427 1495 1511 1668 1858 2037 2131 2141 2188 3017
3070 3094 3202 3743
TS236_8
18

E= -1916.35260734 ZPE= 74.60 Gcorr 0.054165000
H -3.216092 -0.025721 0.742634
Co 0.090420 0.091722 -0.135876
C 0.486998 -0.329578 1.636134
C 0.990059 -1.311072 -0.977626
O 1.277640 -1.168517 1.966494
O 1.466074 -2.197934 -1.516378
C 1.415303 1.309284 -0.236982
O 2.248878 2.094570 -0.269421
C -1.492027 -1.214699 0.059949
C -2.924697 -0.691797 -0.076824
H -1.388192 -1.742912 1.013658
H -1.338199 -1.982400 -0.706997
H -3.626233 -1.536236 -0.065890
H -3.084204 -0.144378 -1.010407
C -1.118899 1.441339 0.290286
O -1.540247 1.989072 -0.703433
H -1.460331 1.731404 1.317751
H -0.087283 0.265365 2.390171
FREQS= 56 81 83 96 108 125 160 210 237 246 291 296 330 370 405 430 446 484 502 525 616
659 690 890 904 989 1005 1033 1257 1281 1301 1312 1426 1471 1508 1513 1768 1819 2136 2174 2787 2809 3027
3040 3080 3088 3121
TS237_8
18
E= -1916.33587604 ZPE= 71.12 Gcorr 0.037473000
H 1.376813 2.619858 0.457675
Co 0.087038 -0.281504 -0.334389
C 1.752117 -1.064551 -0.416110
C 0.257627 0.377822 1.378836
O 2.556290 -1.021345 0.473355
O 0.265524 0.799414 2.441234
C -0.818570 -1.803750 -0.060988
O -1.402645 -2.790469 -0.025314
C 0.754062 1.349768 -1.219974
C 1.750993 2.262648 -0.507970
H -0.224524 1.858571 -1.319228
H 1.093673 1.162299 -2.241089
H 2.701237 1.752343 -0.317291
H 1.977482 3.149526 -1.114564
C -3.395800 0.677081 -0.444338
O -3.029570 1.576335 0.149060
H -0.152625 -0.657404 -1.749011
H 1.958548 -1.590167 -1.371415
FREQS= 19 23 30 36 68 76 85 91 100 127 178 225 275 319 371 395 409 435 492 519 542
629 659 793 825 886 919 1012 1044 1211 1273 1288 1424 1448 1509 1513 1806 2006 2131 2171 2204 2920 2942
3025 3082 3100 3116
TS238_8
18
E= -1916.35400405 ZPE= 75.99 Gcorr 0.055628000
H 4.464584 -0.880788 0.576903
Co -0.651655 -0.159237 -0.120598
C -0.976365 -1.864723 0.100239
C 0.074557 1.579064 0.207856
O -1.127302 -2.965959 0.411610
O -0.038700 2.474570 0.991886
C -2.327885 0.402349 -0.134914
O -3.398639 0.792857 -0.292151
C 2.297883 -0.784890 0.392425
C 3.712575 -0.453230 -0.092494
H 2.175510 -1.881645 0.443177
H 2.133325 -0.433088 1.416498
H 3.889444 -0.846679 -1.098736
H 3.873924 0.628878 -0.124674
C 1.170343 -0.378900 -0.495975
O 1.311225 1.575646 -0.614569
H 1.967767 2.197594 -0.235125
H 1.410822 -0.499800 -1.558939
FREQS= 36 54 71 84 94 112 177 216 263 278 326 341 391 438 467 482 519 538 577 598 635
654 746 855 919 963 1036 1057 1125 1139 1245 1279 1393 1429 1455 1510 1514 1853 2081 2136 2990 3050 3078
3091 3122 3128 3612
TS239_8
18
E= -1916.34952659 ZPE= 73.32 Gcorr 0.051261000
H -3.126509 1.002085 0.594583
Co 0.406531 0.045595 -0.196781
C 0.794632 1.947829 -0.672019
C 0.347493 0.498063 1.548023

O -0.091716 2.709199 -0.992540
O 0.407099 0.762049 2.663119
C 1.810141 -0.967398 -0.274632
O 2.776995 -1.581757 -0.372586
C -1.800144 -0.126496 -0.730432
C -2.997624 -0.022225 0.237890
H -2.081476 -0.445366 -1.736061
H -1.350575 0.871168 -0.876728
H -3.917200 -0.323378 -0.269250
H -2.878063 -0.676137 1.105663
C -0.824335 -1.298226 -0.261759
O -1.125465 -2.431540 -0.087801
H 0.812490 0.175448 -1.623990
H 1.848719 2.312211 -0.645086
FREQS= 42 67 79 81 95 114 141 200 253 290 306 343 370 399 412 431 481 507 543 557 577
638 649 768 858 874 1004 1067 1077 1245 1324 1356 1434 1470 1509 1513 1756 1919 1985 2120 2155 2832 2979
3066 3119 3139 3148
TS240_8
18
E= -1916.35399668 ZPE= 76.07 Gcorr 0.055766000
H -4.035803 -0.664432 -0.494508
Co 0.601364 -0.159577 -0.063082
C 2.159347 0.325776 0.617146
C 0.019766 1.642559 -0.342723
O 3.092264 0.639666 1.213287
O 0.419246 2.631247 -0.886349
C 1.001654 -1.829296 -0.408278
O 1.263716 -2.885201 -0.792984
C -2.195809 -0.916431 0.650995
C -3.656389 -0.477743 0.515836
H -1.829039 -0.764992 1.671100
H -2.115844 -2.003180 0.466221
H -4.292617 -1.019903 1.221462
H -3.752610 0.590900 0.726402
C -1.239180 -0.354752 -0.347740
O -1.423736 1.595625 -0.045519
H -1.669282 -0.316007 -1.355111
H -1.889878 2.294804 -0.551247
FREQS= 34 54 82 86 95 119 154 215 258 279 320 356 378 438 463 484 529 542 584 600 638
661 757 859 916 971 1042 1072 1121 1139 1252 1278 1390 1422 1452 1508 1520 1847 2082 2135 2984 3050 3082
3095 3124 3134 3610
TS241_8
18
E= -1916.34469333 ZPE= 71.98 Gcorr 0.046696000
H 4.312051 0.682645 -0.905013
Co -0.754738 0.086402 0.014365
C -0.737895 1.825890 -0.320564
C -1.382296 -1.003576 -1.242116
O -0.807299 2.962922 -0.512281
O -1.930790 -1.639552 -2.035736
C -0.924245 -0.331459 1.735581
O -1.141746 -0.539317 2.849392
C 2.277103 0.516054 -0.230246
C 3.718763 0.132768 -0.169555
H 2.023722 1.562725 -0.376072
H 1.199218 0.077607 -1.219177
H 4.106144 0.397480 0.824033
H 3.850014 -0.941976 -0.301724
C 1.197465 -0.353117 -0.077550
O 1.561501 -1.659807 0.086410
H -2.203936 0.346391 0.154095
H 0.744009 -2.171051 0.160411
FREQS= 39 57 62 71 82 90 105 155 207 279 305 313 369 385 458 459 470 499 523 540 553
563 689 695 704 803 933 1017 1081 1157 1205 1280 1365 1413 1479 1496 1579 2012 2066 2073 2134 2263 3039
3110 3166 3203 3807
TS242_8
18
E= -1916.35377940 ZPE= 76.00 Gcorr 0.055807000
H 1.746754 -1.067190 1.761391
Co -0.565385 -0.123651 -0.191044
C -0.907980 -1.830037 -0.010872
C 0.260035 1.580624 0.087055
O -1.047301 -2.944243 0.256766
O 0.305813 2.442492 0.914622
C -2.199963 0.476003 0.117183
O -3.269850 0.896375 0.167486
C 2.413071 -0.874380 -0.300289

C 2.587939 -0.647091 1.202626
H 3.290596 -0.524633 -0.860544
H 2.356739 -1.959091 -0.505215
H 3.508910 -1.115193 1.562082
H 2.638558 0.420406 1.436093
C 1.149016 -0.368848 -0.910017
O 1.343869 1.582775 -0.927261
H 2.077698 2.166195 -0.638721
H 1.173198 -0.418734 -2.003906
FREQS= 35 53 76 88 95 109 161 202 264 297 332 369 427 447 455 476 516 535 573 600 623 654 747 858 898 981 1031 1055 1124 1130 1263 1281 1383 1432 1448 1508 1514 1851 2081 2136 2981 3045 3056 3099 3130 3133 3609
TS243_8
18
E= -1916.33614401 ZPE= 71.07 Gcorr 0.038287000
H -1.444932 -3.728678 -0.724162
Co -0.176525 0.024000 -0.165574
C -1.961384 0.509267 -0.306698
C -0.084748 0.240159 1.632740
O -2.425563 1.531098 0.113218
O -0.047640 0.320889 2.773871
C 0.470342 1.596165 -0.791590
O 0.915187 2.516854 -1.307611
C -0.499887 -1.910624 0.021919
C -1.409994 -2.653540 -0.945562
H -0.751279 -2.201560 1.048373
H 0.549418 -2.221623 -0.140261
H -2.448289 -2.298985 -0.879598
H -1.090049 -2.532403 -1.984751
C 3.894346 -0.815817 -0.234101
O 2.853915 -0.479493 0.085628
H -0.285361 -0.317479 -1.610665
H -2.582582 -0.255714 -0.819531
FREQS= 20 29 40 52 62 66 77 92 97 122 187 257 294 333 378 397 411 434 477 528 543 614 671 755 829 895 910 1009 1056 1210 1278 1294 1424 1455 1502 1513 1816 1990 2134 2170 2196 2904 2954 3012 3058 3058 3113
TS244_8
18
E= -1916.35400920 ZPE= 76.11 Gcorr 0.056285000
H 2.549258 -0.718875 -1.593807
Co -0.105451 -0.047004 0.218019
C 0.990087 1.180222 0.951078
C -1.387729 1.170402 0.046932
O 1.697282 1.970398 1.397894
O -2.193319 1.972306 -0.117296
C 1.009226 -1.364146 0.653004
O 1.719643 -2.239461 0.886626
C 0.555231 0.178125 -1.737200
C 2.067672 0.203827 -1.936477
H 0.112709 1.099287 -2.126026
H 0.114332 -0.646271 -2.302741
H 2.320373 0.319221 -3.000872
H 2.544144 1.035119 -1.404938
C -1.376345 -1.338146 0.116786
O -2.679559 -1.152526 -0.163450
H -3.147014 -1.997400 -0.242760
H -1.147864 -2.409415 0.189687
FREQS= 47 73 88 97 104 112 142 150 247 268 320 340 422 441 457 463 464 489 533 564 596 616 680 695 891 962 986 1043 1202 1237 1272 1288 1424 1487 1504 1510 1512 2092 2102 2153 3007 3039 3062 3076 3093 3124 3768
TS245_8
18
E= -1916.33482192 ZPE= 70.82 Gcorr 0.037344000
H -0.703218 -2.995371 0.552530
Co -0.225103 0.139235 -0.286115
C -2.030980 0.526289 -0.238413
C -0.106273 -0.514673 1.392830
O -2.501676 1.584136 0.068306
O -0.080748 -0.899778 2.471946
C 0.326284 1.869283 -0.053949
O 0.760288 2.927984 -0.060280
C -0.370098 -1.627885 -1.134327
C -1.105951 -2.769808 -0.440860
H 0.714831 -1.848142 -1.177144
H -0.712351 -1.558970 -2.169278
H -2.173308 -2.539692 -0.313884
H -1.061379 -3.695464 -1.029760

C 3.394112 0.015031 -0.734449
O 3.380828 -0.655270 0.184987
H -0.434969 0.578346 -1.690105
H -2.663922 -0.346052 -0.511925
FREQS= 21 25 35 36 64 73 83 91 115 147 158 262 277 310 332 403 407 418 482 523 539 620 660 686 823 890 898 1019 1042 1204 1270 1288 1429 1455 1502 1517 1820 1999 2132 2169 2205 2896 2939 3012 3062 3089 3114
TS246_8
18
E= -1916.34047387 ZPE= 69.55 Gcorr 0.043190000
H 3.018383 -0.170278 0.305418
Co -0.177061 0.226936 -0.369191
C -1.376388 -1.137362 -0.956626
C 0.478546 -0.848107 1.065783
O -1.767481 -2.154952 -0.441134
O 0.262103 -0.848199 2.228423
C -1.605747 1.096338 0.227259
O -2.494617 1.603691 0.750451
C 1.166194 -1.259427 -0.105355
C 2.679765 -1.091512 -0.176084
H 0.791250 -2.211245 -0.474828
H 0.439732 0.349616 -2.522073
H 3.182424 -1.930894 0.319186
H 3.017674 -1.066044 -1.216429
C 1.017686 1.598195 -0.186259
O 1.729628 2.495176 -0.133762
H 0.042364 0.873871 -2.881342
H -1.708594 -0.886780 -2.005907
FREQS= 55 71 79 88 96 105 136 170 178 222 268 282 308 325 377 390 405 417 439 458 480 510 538 554 573 622 840 885 930 1048 1067 1122 1335 1341 1426 1499 1514 1796 1984 2107 2152 2703 3040 3105 3128 3165 4316
TS247_8
18
E= -1916.35380608 ZPE= 76.19 Gcorr 0.056662000
H -1.623119 -0.133144 -3.366499
Co 0.239334 -0.005579 0.211843
C 1.480105 -1.281008 0.183853
C -1.004282 1.209454 0.578541
O 2.249904 -2.133575 0.104714
O -1.813216 2.001945 0.771519
C 1.513616 1.266668 0.210540
O 2.322167 2.084506 0.173415
C -0.082624 0.026981 -1.840435
C -1.527783 -0.200913 -2.272521
H 0.564628 -0.735616 -2.281508
H 0.270591 0.992273 -2.213680
H -1.896809 -1.188520 -1.976330
H -2.214573 0.538421 -1.846266
C -0.883007 -1.292700 0.822810
O -2.176512 -1.124715 1.149720
H -2.595057 -1.967293 1.381704
H -0.602562 -2.351657 0.891139
FREQS= 55 71 89 97 103 112 142 159 249 274 321 338 423 440 455 460 466 492 533 565 596 616 683 701 895 971 986 1046 1211 1237 1274 1287 1425 1490 1503 1508 1513 2092 2101 2152 3006 3041 3061 3078 3095 3121 3766
TS248_8
18
E= -1916.34826303 ZPE= 72.99 Gcorr 0.051131000
H 3.471729 -1.987084 -0.665764
Co -0.177637 0.025336 -0.064431
C -1.232514 -1.525557 -0.747777
C -0.901826 -0.214670 1.577640
O -2.374193 -1.521440 -1.128720
O -1.403172 -0.324555 2.602816
C -1.084783 1.402743 -0.635392
O -1.680253 2.278149 -1.074172
C 1.862724 -0.877503 0.259717
C 2.973580 -1.019230 -0.778948
H 1.134642 -1.686520 0.138395
H 2.228823 -0.958938 1.285523
H 2.573751 -0.959040 -1.794542
H 3.719089 -0.229864 -0.653968
C 1.411523 0.781012 0.251880
O 2.226301 1.623866 0.370675
H -0.003923 -0.100142 -1.533957
H -0.649617 -2.491402 -0.763568

FREQS=   56   71   77   90   94 134 154 191 218 278 318 341 361 382 412 452 467 490 515 558 568
606 619 779 801 870 980 1031 1066 1247 1276 1363 1425 1464 1507 1516 1800 1957 2004 2135 2168 2683 3057
3072 3120 3130 3148
TS249_8
18
E= -1916.34645866 ZPE=   73.11 Gcorr   0.049390000
H -1.614722 0.053184 -1.867986
Co 0.393484 0.074802 0.011804
C 0.251578 -1.667183 -0.174477
C 1.768611 0.289440 -1.165016
O 0.099091 -2.797658 -0.308169
O 2.677835 0.514877 -1.829159
C 0.757783 0.033833 1.767701
O 1.064215 0.103282 2.876874
C -2.715944 -0.562903 -0.062971
C -2.933038 0.881115 -0.304752
H -2.529726 -0.890175 0.955685
H -3.288470 -1.273240 -0.652872
H -3.626510 1.194951 -1.078879
H -2.868505 1.567464 0.535755
C -1.613949 0.203604 -0.791196
O 0.060816 2.008883 -0.028496
H -0.894643 1.272082 -0.373544
H -0.107385 2.353557 0.858995
FREQS=   26   50   67   82   87   99 132 195 220 265 330 389 409 419 440 453 482 495 534 555 573
602 786 802 847 866 881 995 1068 1095 1160 1207 1226 1262 1430 1488 1524 1563 2084 2110 2158 3133 3136
3157 3210 3225 3825
TS250_8
18
E= -1916.34813826 ZPE=   72.97 Gcorr   0.051078000
H 2.679036 -1.422009 0.829152
Co -0.237963 0.098010 -0.180273
C -1.383355 -1.317446 -1.001093
C -0.283748 -0.798290 1.391341
O -2.582235 -1.408054 -0.949245
O -0.364667 -1.331218 2.403357
C -1.366739 1.393569 0.125902
O -2.129861 2.237389 0.264264
C 1.818173 -0.449748 -0.925305
C 3.001796 -0.988013 -0.121916
H 2.114031 -0.021839 -1.883766
H 1.129441 -1.267456 -1.161351
H 3.515642 -1.769657 -0.690000
H 3.716286 -0.189383 0.092343
C 1.314806 0.981515 -0.115356
O 2.087229 1.839930 0.118062
H -0.623642 0.484231 -1.561766
H -0.795114 -2.094064 -1.570175
FREQS=   56   73   76   86   97 140 146 192 214 273 321 336 355 386 415 442 471 489 518 549 568
585 633 783 796 872 982 1034 1061 1251 1279 1366 1426 1461 1507 1515 1800 1958 2001 2134 2167 2683 3055
3078 3122 3137 3151
TS251_8
18
E= -1916.33501118 ZPE=   70.93 Gcorr   0.038442000
H -1.914326 -3.360062 -1.020307
Co -0.164515 0.153259 -0.274205
C -1.819364 0.976765 -0.303040
C -0.283238 -0.478916 1.412687
O -2.022794 2.123072 -0.020983
O -0.411376 -0.835416 2.494464
C 0.791591 1.695410 -0.031390
O 1.475855 2.612422 -0.026126
C -0.719442 -1.536226 -1.114734
C -1.744535 -2.442640 -0.441346
H 0.274920 -2.023900 -1.117808
H -0.998320 -1.398443 -2.161758
H -1.448284 -2.744449 0.569127
H -2.722761 -1.948233 -0.358621
C 3.201427 -1.234588 0.397598
O 3.151291 -0.829540 -0.664851
H -0.213045 0.608412 -1.687552
H -2.638721 0.285538 -0.598231
FREQS=   25   32   37   40   66   74   84   94 115 151 161 262 278 316 334 404 408 421 482 523 540
621 669 688 829 894 898 1019 1041 1205 1274 1289 1427 1457 1503 1516 1819 2005 2131 2168 2203 2897 2943
3012 3062 3089 3114
TS252_8
18

E= -1916.34766757 ZPE= 74.40 Gcorr 0.051493000
H -4.366856 0.323390 -0.381777
Co 0.509273 -0.124231 0.056135
C 0.501305 0.865157 -1.409140
C -1.174058 -0.944303 0.082473
O 0.300724 1.913422 -1.933636
O -0.546585 -1.862918 0.616248
C 0.417571 1.145673 1.210929
O 0.342393 2.006587 1.972049
C -2.638639 -0.977938 -0.233228
C -3.320381 0.385167 -0.072236
H -3.118139 -1.760144 0.367581
H 0.879657 -0.079756 -1.953006
H -3.297553 0.717740 0.969691
H -2.825803 1.145762 -0.682377
C 2.364117 -0.541768 0.280842
O 2.606264 -1.449036 -0.488462
H -2.696631 -1.298157 -1.284353
H 3.153101 -0.150943 0.961188
FREQS= 32 46 62 78 105 123 136 153 178 218 294 327 356 385 447 483 524 539 548 595 616 745 774 821 939 1036 1070 1142 1168 1270 1317 1336 1431 1456 1512 1517 1673 1753 1906 2120 2503 2873 3023 3060 3080 3133 3141
TS253_8
18
E= -1916.34376459 ZPE= 73.58 Gcorr 0.047975000
H 4.789560 -0.084612 0.374058
Co -0.551205 -0.012904 -0.067007
C -0.650799 -0.062810 1.681240
C -0.625436 1.772005 -0.370369
O -0.699224 -0.101908 2.833559
O -0.763587 2.906817 -0.514978
C -2.061022 -0.926796 -0.417168
O -3.040214 -1.477586 -0.668005
C 2.854191 0.073478 -0.542328
C 3.769372 -0.447823 0.541974
H 2.734537 1.161577 -0.567266
H 3.071112 -0.285303 -1.545760
H 3.459716 -0.111559 1.536417
H 3.808561 -1.541490 0.544377
C 1.108187 -0.886617 -0.597047
O 0.449444 -0.587472 -1.649834
H 1.577605 0.030617 0.027413
H 1.503145 -1.868263 -0.343795
FREQS= 27 49 53 71 81 89 102 146 186 220 270 311 371 374 430 452 466 478 498 539 553 568 675 775 950 995 1040 1056 1135 1231 1251 1341 1423 1451 1485 1503 1508 2080 2086 2141 2292 3046 3087 3112 3125 3160 3188
TS254_8
18
E= -1916.35020113 ZPE= 75.88 Gcorr 0.054418000
H 4.690902 -0.726896 -0.165593
Co -0.365411 0.205145 -0.313729
C -1.562307 -1.160279 0.263328
C 0.752307 0.453459 0.954830
O -2.080409 -1.457386 1.309896
O 1.313912 0.787294 1.920565
C -1.642695 1.405482 -0.392407
O -2.425212 2.245915 -0.351013
C 2.872227 0.374614 -0.632490
C 3.608997 -0.887875 -0.145086
H 3.132836 1.225518 0.004684
H 3.210000 0.621904 -1.648421
H 3.320796 -1.131797 0.881124
H 3.381053 -1.749835 -0.779931
C 1.384861 0.231436 -0.701710
O -1.701361 -1.951537 -0.863999
H -2.249816 -2.716686 -0.603844
H 1.044551 -0.556424 -1.419726
FREQS= 35 44 62 74 86 96 168 194 230 272 328 364 368 432 481 498 527 562 584 606 658 674 702 800 939 1007 1029 1065 1168 1245 1253 1293 1362 1421 1487 1510 1517 1818 2032 2136 2842 3032 3052 3095 3127 3136 3667
TS255_8
18
E= -1916.34846812 ZPE= 74.95 Gcorr 0.052879000
H -2.009135 2.355566 0.167652
Co 0.087851 -0.095429 -0.043841
C 0.555290 1.566751 -0.532390
C -0.840063 -0.689175 1.390232

O 1.753210 2.212552 -0.331290
O -1.472063 -0.997193 2.299083
C 1.726762 -0.355339 0.662515
O 2.769442 -0.500445 1.115765
C -1.767737 0.411196 -0.813433
C -2.563662 1.425211 0.003380
H -2.359810 -0.501308 -0.938934
H -1.569599 0.800708 -1.816529
H -3.494895 1.691002 -0.515486
H -2.842463 1.036316 0.987859
C 0.062185 -1.474497 -1.206413
O 0.028599 -2.267283 -2.037942
H 2.373432 2.046195 -1.055395
H -0.139657 2.262178 -1.033731
FREQS= 28 67 82 96 104 112 119 197 203 263 289 333 339 408 421 434 452 477 491 534 538 544 721 739 805 982 984 1025 1046 1197 1235 1277 1414 1430 1490 1506 1510 2101 2116 2160 2982 3022 3051 3085 3095 3119 3803
TS256_8
18
E= -1916.34750541 ZPE= 74.48 Gcorr 0.052009000
H -4.366944 -0.439672 0.518864
Co 0.494892 0.142057 -0.053735
C 0.306883 -0.968110 1.309284
C -1.247651 0.691701 -0.468917
O 0.173245 -2.079232 1.711691
O -0.674946 1.739344 -0.780554
C 0.906909 -1.013960 -1.257138
O 1.167683 -1.801910 -2.055477
C -2.723960 0.457559 -0.575882
C -3.308376 -0.215933 0.673447
H -2.851807 -0.207830 -1.441868
H -3.224841 1.404593 -0.808984
H -2.788430 -1.151400 0.896897
H -3.227843 0.437027 1.547479
C 2.247507 0.860269 0.223070
O 2.134950 1.719539 1.073080
H 0.369195 -0.029905 1.978148
H 3.233264 0.660572 -0.252786
FREQS= 37 50 71 82 102 110 135 157 191 220 277 343 355 377 446 499 511 539 559 595 612 740 789 822 938 1039 1065 1140 1175 1272 1317 1336 1430 1462 1512 1520 1673 1754 1905 2120 2504 2874 3028 3060 3085 3134 3142
TS257_8
18
E= -1916.34458022 ZPE= 73.75 Gcorr 0.049201000
H 4.356023 0.192971 -0.351920
Co -0.493853 -0.041106 -0.037101
C -1.093611 0.684401 1.441331
C -0.003898 1.440164 -0.951988
O -1.471284 1.153386 2.425483
O 0.219906 2.406545 -1.538887
C -2.029460 -0.816993 -0.569399
O -3.012498 -1.278442 -0.951096
C 2.800623 -0.735002 0.794457
C 3.264980 0.234070 -0.262541
H 3.137145 -1.756883 0.636817
H 2.967757 -0.437897 1.833984
H 2.835833 -0.042192 -1.230670
H 2.982039 1.264748 -0.033130
C 0.907740 -1.314819 0.434741
O 0.721992 -1.349311 -0.831986
H 1.435507 -0.346096 0.897394
H 0.876557 -2.173148 1.101528
FREQS= 33 53 63 80 83 84 112 146 180 224 273 332 377 388 433 457 470 485 506 538 562 573 682 775 928 984 1007 1070 1145 1192 1252 1338 1414 1451 1487 1496 1513 2079 2086 2141 2336 3051 3093 3119 3138 3166 3190
TS258_8
18
E= -1916.35214286 ZPE= 75.76 Gcorr 0.056800000
H 2.833337 -0.254750 2.243785
Co -0.558543 -0.066023 -0.193772
C -1.667509 1.081658 0.555124
C 1.232453 -1.081575 0.131056
O -2.401209 1.814239 1.051072
O 0.605728 -1.419378 -0.949777
C 0.387572 1.263041 -0.776754
O 0.954445 2.129386 -1.288529
C 2.627061 -0.503297 0.086284

C 3.016253 0.310020 1.323190
H 2.764914 0.068092 -0.835899
H 3.291295 -1.379020 0.004777
H 4.078140 0.569603 1.298529
H 2.442178 1.239583 1.381179
C -1.977283 -1.329525 -0.456372
O -1.700707 -1.697294 0.684428
H 0.987046 -1.614217 1.066638
H -2.693585 -1.904220 -1.079886
FREQS= 55 67 92 105 128 137 155 189 219 236 300 342 380 446 463 492 503 510 542 562 638 744 776 894 904 1035 1094 1160 1261 1276 1331 1342 1430 1484 1500 1513 1517 1652 2081 2135 2908 2984 3003 3050 3103 3122 3130
TS259_8
18
E= -1916.33471741 ZPE= 71.23 Gcorr 0.039650000
H -1.618612 -2.549647 0.680279
Co -0.052654 0.152756 -0.312992
C -1.697005 0.973521 -0.570947
C -0.348886 -0.310853 1.420720
O -2.150003 1.822434 0.145175
O -0.517537 -0.597078 2.515984
C 0.862789 1.706310 -0.125997
O 1.485186 2.667409 -0.149735
C -0.676722 -1.574640 -1.051342
C -1.823246 -2.324722 -0.372373
H 0.252932 -2.164553 -0.972411
H -0.893860 -1.490446 -2.119493
H -2.758479 -1.751194 -0.404080
H -2.027457 -3.280725 -0.872916
C 3.589424 -1.366236 -0.212819
O 2.570337 -0.957911 0.092492
H 0.175461 0.415993 -1.759237
H -2.250322 0.597057 -1.456146
FREQS= 25 34 46 59 62 74 80 93 99 131 167 261 284 331 374 395 420 426 485 521 548 624 687 741 808 890 927 1014 1040 1218 1278 1299 1429 1459 1507 1518 1809 1992 2134 2169 2192 2918 2979 3018 3071 3083 3106
TS260_8
18
E= -1916.34883313 ZPE= 74.95 Gcorr 0.053858000
H 2.227956 -2.154742 0.421690
Co -0.324070 -0.029747 -0.063192
C 0.074587 -0.078744 1.685066
C -1.533304 1.261405 -0.167408
O 0.145345 -0.199943 2.832532
O -2.303890 2.116611 -0.245260
C -1.374135 -1.395523 -0.471196
O -2.034677 -2.311654 -0.716500
C 1.298773 -0.729243 -0.861523
C 2.501343 -1.221131 -0.075729
H 1.869107 0.148577 -1.700103
H 1.058089 -1.340858 -1.734374
H 3.370835 -1.421314 -0.713111
H 2.793789 -0.503971 0.693091
C 1.119848 0.881952 -1.050165
O 1.999721 1.734348 -0.393846
H 1.744043 1.743223 0.539052
H 0.711427 1.305061 -1.969742
FREQS= 36 65 74 86 91 103 175 202 256 279 299 376 394 425 441 474 485 508 533 540 559 589 617 698 920 985 1071 1103 1116 1236 1254 1318 1382 1421 1483 1497 1505 2060 2071 2128 2210 3045 3109 3124 3131 3158 3795
TS261_8
18
E= -1916.35228580 ZPE= 76.91 Gcorr 0.057328000
H 3.856900 -0.865664 2.035964
Co -0.413896 0.175780 -0.281713
C -1.745652 0.488713 0.748192
C 1.362187 -0.495001 -0.348123
O -2.616327 0.573698 1.514730
O 1.203493 -0.345644 -1.565120
C 0.050393 1.926789 -0.350956
O 0.301084 3.044657 -0.446338
C 2.607044 -1.058483 0.272039
C 2.988610 -0.374325 1.588793
H 3.420392 -1.025639 -0.464126
H 2.376684 -2.118030 0.458737
H 3.242411 0.677430 1.427180
H 2.162300 -0.415659 2.303844

C -1.140858 -1.429896 -0.643619
O -2.065330 -2.223042 -0.114606
H -2.459624 -1.812890 0.671883
H -0.737563 -1.929741 -1.534503
FREQS= 34 61 82 91 99 114 187 205 220 237 309 356 387 445 451 473 522 543 574 580 654 681 775 922 943 1031 1063 1160 1231 1270 1333 1339 1427 1457 1462 1510 1517 1651 2039 2124 3019 3057 3073 3073 3131 3137 3750
TS262_8
18
E= -1916.34521349 ZPE= 72.97 Gcorr 0.050340000
H 3.863261 -0.699966 -0.884691
Co -0.340873 0.057422 -0.027095
C -1.655101 -1.128520 -0.444942
C 0.161602 -0.713539 1.494606
O -2.425998 -1.897034 -0.800908
O 0.436556 -1.092757 2.547040
C -1.437458 1.463876 0.109056
O -2.111371 2.377938 0.307830
C 2.347526 0.825342 -0.602567
C 3.112568 -0.425885 -0.139105
H 3.019450 1.651537 -0.850596
H 1.723228 0.329055 -1.525003
H 3.628971 -0.223796 0.805356
H 2.440520 -1.273587 -0.009579
C 1.106853 1.225582 -0.032661
O 0.563784 -0.702822 -1.652228
H 0.978700 2.300194 0.128071
H 0.029737 -0.597576 -2.452197
FREQS= 51 61 77 90 93 108 163 184 211 283 294 360 368 395 420 439 462 474 503 509 544 552 554 684 850 893 946 976 1067 1128 1218 1245 1359 1402 1434 1505 1514 1914 2081 2109 2160 3056 3086 3110 3126 3175 3812
TS263_8
18
E= -1916.34206922 ZPE= 72.44 Gcorr 0.047581000
H 2.753038 1.078650 0.215486
Co -0.290272 0.007468 0.039784
C -0.149586 0.159692 1.799182
C -0.114226 1.776665 -0.270744
O -0.141252 0.233817 2.949677
O 0.056745 2.886689 -0.499588
C -1.861728 -0.138401 -0.774306
O -2.917884 -0.247181 -1.223167
C 1.643926 -0.162393 -1.208441
C 2.907259 0.194934 -0.411713
H 1.348153 0.661711 -1.864217
H 1.915519 -0.908453 -1.982855
H 3.201780 -0.620034 0.259664
H 3.767673 0.406191 -1.060300
C -0.240833 -1.847087 0.241490
O 0.693815 -2.467004 -0.339913
H -0.933936 -2.483204 0.814264
H 1.144827 -1.487518 -0.805093
FREQS= 27 49 55 89 97 104 116 157 199 255 311 329 347 396 428 443 487 498 502 527 567 570 617 715 940 952 966 1017 1168 1238 1303 1361 1424 1433 1510 1517 1569 1788 2081 2108 2157 2930 3023 3034 3074 3087 3106
TS264_8
18
E= -1916.34908606 ZPE= 74.32 Gcorr 0.054694000
H 0.958714 2.907037 -0.936332
Co 0.199021 0.131446 -0.096102
C -0.400036 -0.645746 1.541764
C -0.967560 -0.793763 -1.167012
O -1.129163 -1.601651 1.592097
O -1.692921 -1.344877 -1.853923
C 1.572827 -1.065522 -0.178292
O 2.404967 -1.847635 -0.087337
C 1.443511 1.453359 0.631330
C 1.427082 1.929824 -0.801935
H 2.416029 1.108886 0.980427
H 1.002077 2.145846 1.348788
H 2.403931 1.908967 -1.290181
H 0.798094 1.248772 -1.476383
C -1.261005 1.394079 0.302606
O -2.448298 1.201129 0.214255
H -0.903454 2.420035 0.601875
H -0.014574 -0.177716 2.475064

FREQS= 69 78 82 100 107 148 169 193 277 284 311 323 329 404 410 423 477 495 525 541 589
643 841 862 891 904 996 1031 1137 1179 1321 1372 1415 1456 1514 1557 1773 1811 2134 2183 2545 2710 2868
3085 3102 3150 3173
TS265_8
18
E= -1916.32470256 ZPE= 69.63 Gcorr 0.030640000
H -1.395124 3.479610 -0.986038
Co 0.546535 0.048066 -0.364721
C 2.315749 -0.185707 -0.205272
C 0.317988 0.416475 1.343847
O 3.185271 -0.550452 0.508474
O 0.045231 0.677544 2.427759
C 0.169666 -1.682070 -0.537667
O -0.204997 -2.757311 -0.679931
C 0.191117 2.010426 -0.744997
C -1.286450 2.400765 -0.810321
H 0.670882 2.227097 -1.704179
H 0.696937 2.618406 0.012872
H -1.808187 1.878250 -1.617405
H -1.817640 2.176037 0.121038
C -3.968249 -0.781706 -0.016385
O -3.013702 -0.744553 0.602847
H -0.054310 0.004508 -1.773415
H 2.417649 0.247400 -1.253819
FREQS= 12 18 27 33 34 45 59 83 89 108 129 211 243 255 378 399 438 455 475 513 525
559 610 700 753 769 963 987 1039 1071 1230 1271 1424 1488 1509 1514 1878 1922 2119 2159 2203 2643 3023
3046 3084 3095 3120
TS266_8
18
E= -1916.34610467 ZPE= 74.02 Gcorr 0.052329000
H 2.283955 1.325884 -0.992848
Co -0.419270 0.161499 0.443429
C -0.132007 1.973169 0.431723
C -2.079709 0.092468 -0.127731
O 0.536709 1.870897 1.456596
O -2.903997 -0.008254 -0.971146
C -0.215899 -1.594473 0.827671
O -0.105794 -2.721031 1.007595
C 2.821521 -0.421236 0.163950
C 2.139313 0.237862 -1.038631
H 3.900277 -0.244915 0.124092
H 2.458782 -0.016870 1.113179
H 2.664693 -1.504537 0.166765
H 2.575761 -0.106969 -1.981730
C 0.619927 0.009361 -1.167382
O 0.119996 -0.239960 -2.232153
H -2.332841 0.242053 1.025413
H -0.324536 2.948759 -0.052178
FREQS= 53 58 71 87 98 117 140 184 225 243 251 331 348 371 432 455 468 522 547 579 627
755 784 836 886 1025 1071 1119 1122 1264 1277 1326 1427 1470 1515 1519 1676 1813 1946 2138 2184 2973 3035
3053 3097 3125 3132

7. Structures of the main intermediates

Structures of the main intermediates (**I-XVII**) constructed with the visualization software Avogadro

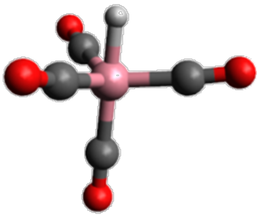

I

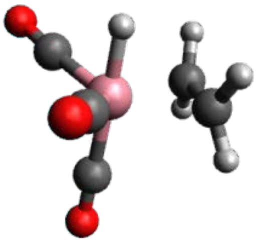

II

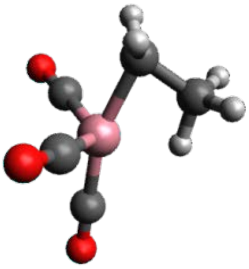

III

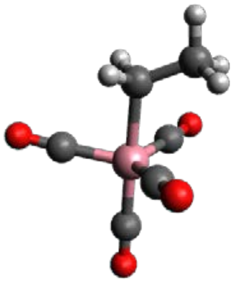

IV

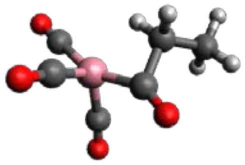

V

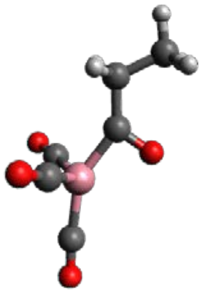

VI

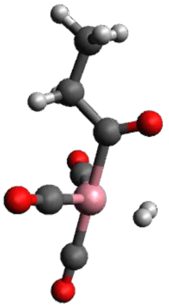

VII

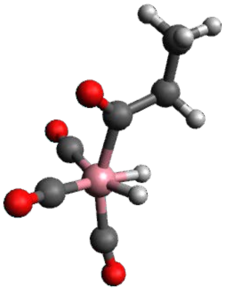

VIII

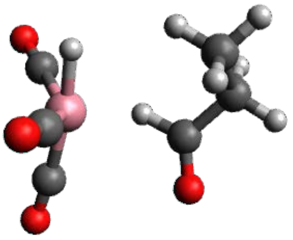

IX

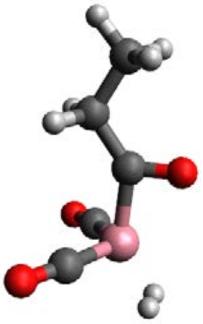

X

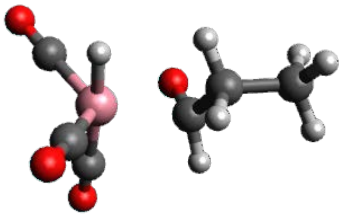

XI

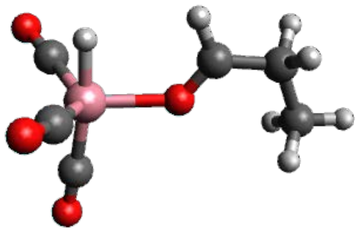

XII

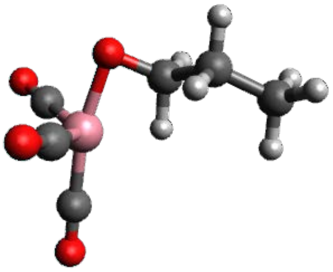

XIII

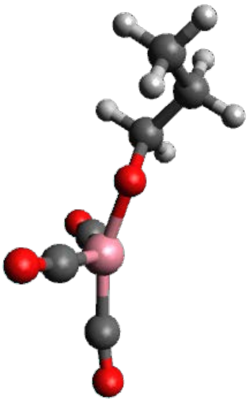

XIV

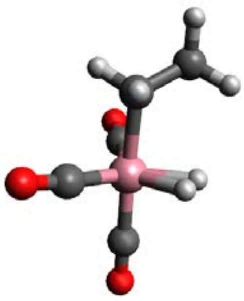

XV

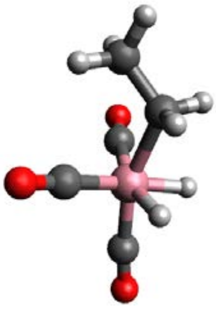

XVI

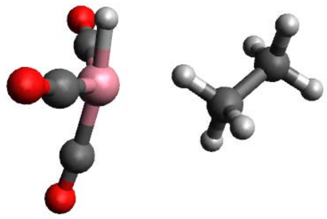

XVII

References

1. Martinez-Nunez, E., An automated method to find transition states using chemical dynamics simulations. *J. Comput. Chem.* **2015,** *36*, 222-234.
2. Hase, W. L.; Buckowski, D. G.; Swamy, K. N., Dynamics of ethyl radical decomposition. 3. Effect of chemical activation vs. microcanonical sampling. *J. Phys. Chem.* **1983,** *87* (15), 2754.
3. Press, W. H.; Flannery, B. P.; Teukolsky, S. A.; Vetterling, W. T., *Numerical Recipes: The Art of Scientific Computing*. Cambridge University: Cambridge, 1988.
4. Martinez-Nunez, E., Automated transition state search using classical trajectories initialized at multiple minima. *Phys. Chem. Chem. Phys.* **2015,** *17*, 14912-13921.
5. (a) Maia, J. D. C.; Carvalho, G. A. U.; Mangueira Jr., C. P.; Santana, S. R.; Cabral, L. A. F.; Rocha, G. B., GPU Linear Algebra Libraries and GPGPU Programming for Accelerating MOPAC Semiempirical Quantum Chemistry Calculations. *J. Chem. Theor. Comput.* **2012,** *8*, 3072-3081; (b) Stewart, J. J. P. *MOPAC2016, Stewart Computational Chemistry, Colorado Springs, CO, USA, HTTP://OpenMOPAC.net*.
6. Martinez-Nunez, E.; Shalashilin, D. V., Acceleration of classical mechanics by phase space constraints. *J. Chem. Theor. Comput.* **2006,** *2* (4), 912-919.
7. (a) Kim, Y.; Choi, S.; Kim, W. Y., Efficient Basin-Hopping Sampling of Reaction Intermediates through Molecular Fragmentation and Graph Theory. *J. Chem. Theory Comput., Article ASAP*; (b) Moussa, J. E., Comment on "Fast and Accurate Modeling of Molecular Atomization Energies with Machine Learning". *Phys. Rev. Lett.* **2012,** *109*, 059801; (c) Sadeghi, A.; S.A., G.; B., S.; Mohr, S.; Lill, M. A.; Goedecker, S. J., Metrics for Measuring Distances in Configuration Spaces. *J. Chem. Phys.* **2013,** *139*, 184118.
8. Cordero, B.; Gomez, V.; Platero-Prats, A. E.; Reves, M.; Echevarria, J.; Cremades, E.; Barragan, F.; Alvarez, S., Covalent Radii Revisited. *Dalton Trans.* **2008**, 2832-2838.
9. Pietrucci, F.; Andreoni, W., *Phys. Rev. Lett.* **2011,** *107*, 085504.
10. Bollobas, B., *Modern Graph Theory*. Springer-Verlag: Berlin, 1998.
11. Rush, L. E.; Pringle, P. G.; Harvey, J. N., Computational kinetics of cobalt-catalyzed alkene hydroformylation. *Angew. Chem. Int. Ed.* **2014,** *53*, 8672-8676.
12. Haberson, S., Automated prediction of catalytic mechanism and rate law using graph-based reaction path sampling. *J. Chem. Theor. Comput.* **2016,** *12*, 1786-1798.
13. Sanft, K. R.; Wu, S.; Roh, M.; Fu, J.; Lim, R. K.; Petzold, L. R., StochKit2: software for discrete stochastic simulation of biochemical systems with events. *Bioinformatics* **2011,** *27*, 2457-2458.
14. Ding, A.; Cassidy, R. A.; Futrell, J. H.; Cordis, L., Ion-molecule reactions within methane clusters initiated by photoionization. *J. Phys. Chem.* **1987,** *91* (10), 2562.
